# Are you also what your mother eats? Distinct proteomic portrait as a result of maternal high-fat diet in the cerebral cortex of the adult mouse

**DOI:** 10.1038/ijo.2015.35

**Published:** 2015-04-21

**Authors:** A Manousopoulou, J Woo, C H Woelk, H E Johnston, A Singhania, C Hawkes, S D Garbis, R O Carare

**Affiliations:** 1 Centre for Proteomic Research, Institute for Life Sciences, University of Southampton, Southampton, UK; 2 Clinical and Experimental Sciences, University of Southampton, Southampton, UK; 3 Cancer Sciences, University of Southampton, Southampton, UK

## Abstract

Epidemiological studies suggest an association between maternal obesity and adverse neurodevelopmental outcomes in offspring. Our aim was to compare the global proteomic portrait in the cerebral cortex between mice born to mothers on a high-fat or control diet who themselves were fed a high-fat or control diet. Male mice born to dams fed a control (C) or high-fat (H) diet 4 weeks before conception and during gestation, and lactation were assigned to either C or H diet at weaning. Mice were killed at 19 weeks and their cerebral cortices were analysed using a two-dimensional liquid chromatography-mass spectrometry methodology. In total, 6 695 proteins were identified (*q*<0.01), 10% of which were modulated in at least one of the groups relative to controls. *In silico* analysis revealed that mice clustered based on the diet of the mother and not their own diet and that maternal high-fat diet was significantly associated with response to hypoxia/oxidative stress and apoptosis in the cerebral cortex of the adult offspring. Maternal high-fat diet resulted in distinct endophenotypic changes of the adult offspring cerebral cortex independent of its current diet. The identified proteins could represent novel therapeutic targets for the prevention of neuropathological features resulting from maternal obesity.

## Introduction

Females of reproductive age have not been exempted from the obesity epidemic.^1^ Since the ‘Barker' theory arose 22 years ago,^2^ accumulating evidence corroborates that fetal adaptations to nutritionally compromised intrauterine environments (for example, malnutrition, obesity) may result in later-life adverse health consequences, a process defined as developmental programming.^3, 4^ Epidemiological studies have found an association between maternal obesity and neuropathological features in the offspring such as cognitive problems in childhood, eating disorders in adolescence and psychotic episodes in adulthood.^5^

High-fat diet-induced obesity in rodents has been extensively used as an *in vivo* model to study the effects of obesity on various organ systems.^6^ To our best knowledge, global tissue proteomics has not been previously applied to assess the effects of maternal obesity on brain regions of the adult offspring. Our aim was to examine and compare the endophenotypic portrait of male adult mouse cerebral cortices whose mothers during pregnancy/lactation and themselves after weaning were exposed to a high-fat or control diet.

## Materials and methods

**Figure 1:**
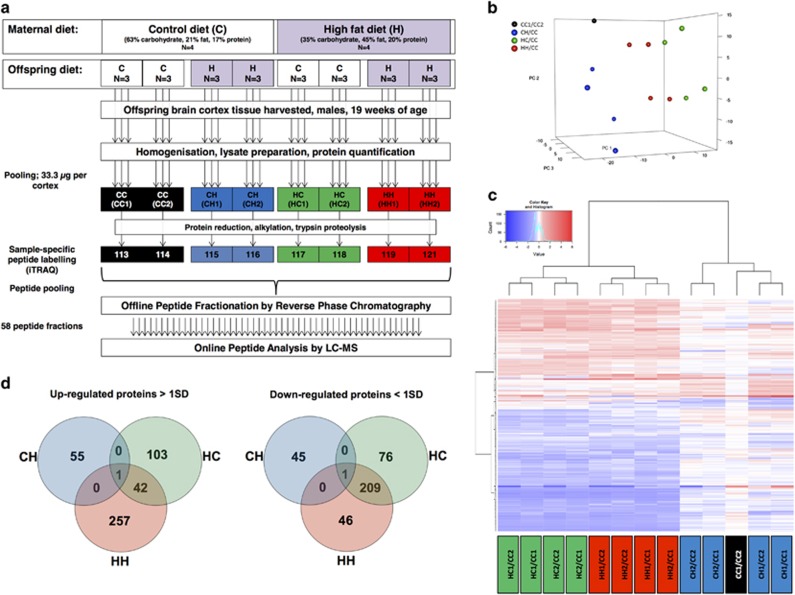
(**a**) Proteomics workflow and labelling scheme, (**b**) Principal component analysis of the iTRAQ ratios of all analysed proteins in the cerebral cortex showing clustering of mice based on the maternal diet and not their current diet, that is, blue dots (CH/CC) clustering separately from red (HH/CC) and green dots (HC/CC) along principal component 1. The sample division along principal component 2 results from dividing each sample by a different control, that is, CC1 (dots in the top) or CC2 (dots in the bottom). (**c**) Venn diagrams of commonly up- and downregulated proteins in CH, HC and HH mice compared with controls (CC). (**d**) Hierarchical clustering analysis of modulated proteins: the HC and HH groups have a similar proteomic portrait, which was different from that of CH mice.

Proven C57b1/6 dams were fed a control (C) (21% kcal fat, 17% kcal protein, 63% kcal carbohydrate, *n*=4) or high-fat (H) chow diet (45% kcal fat, 20% kcal protein, 35% kcal carbohydrate; Special Diet Services, United Kingdom, *n*=4) 4 weeks before conception and during gestation and lactation. At weaning, 4-week-old male offspring (*n*=24) were assigned to C or H, generating four groups (CC, CH, HC, HH, *n*=6 for each) (Figure 1a).

Nineteen week-old mice were anaesthetised, perfused intracardially with phosphate-buffered saline, brains removed, dissected for frontoparietal cortices and snap frozen. Experimental procedures were approved by the Institutional Animal Care and Use Committee at the University of Southampton and the Home Office.^7^

Specimens were dissolved in 0.5 m triethylammonium bicarbonate, 0.05% sodium dodecyl sulfate and homogenised using the FastPrep system (Savant Bio, Illkirch, France) followed by pulsed probe sonication (Misonix, Farmingdale, NY, USA). Lysates were centrifuged (16 000 *g*, 10 min, 4 °C) and supernatants were measured for protein content using the bicinchoninic acid assay (Thermo Pierce, Rockford, IL, USA) per manufacturer's instructions. Three individual protein extracts were pooled (33.3 μg from each lysate giving 100 μg final protein content) to form two biological replicates for each of the four conditions and subjected to reduction, alkylation, trypsin proteolysis and Isobaric tags for relative and absolute quantitation (iTRAQ) labelling per supplier's specifications (ABSciex, San Hose, CA, USA). Only biological replicates were included in the study design as the technical reproducibility of the iTRAQ proteomics method used has been reported by the authors.^8, 9^ Labelled peptides were pooled and fractionated with high-PH reverse phase chromatography using the Waters, XBridge C_8_ column (150 × 3 mm, 3.5 μm particle) with the UltiMate HPLC (LC Packings, Amsterdam, NL, USA) (Supplementary Methods 1).^9^ Each resulting fraction was liquid chromatography-mass spectrometry analysed with low-pH reverse phase capillary chromatography (PepMap C_18_, 75 μm ID × 50 cm length, 100 Å pore, 3.5 μm particle) and nanospray ionisation FT-MS (Ultimate 3000 UHPLC - LTQ-Velos Pro Orbitrap Elite, Thermo Scientific, Bremen, DE, USA) (Figure 1a) (Supplementary Methods 2).^8, 9^

The unprocessed raw files were submitted to Proteome Discoverer 1.4 for target decoy searching with SequestHT for tryptic peptides, allowing two missed cleavages, 10 ppm tolerance, minimum peptide length 6 and 2 maximum variable (1 equal) modifications: oxidation (M), deamidation (N, Q), phosphorylation (S, T, Y), iTRAQ 8plex (Y). Methythio (C) and iTRAQ (K and N-teminus) were set as fixed modifications. Fragment ion mass tolerances were 0.02 Da and 0.5 Da for the higher energy collisional induced dissociation and collision induced dissociation spectra, respectively. False discovery rate (FDR) was estimated with the Percolator at ⩽0.01 and validation set at *q*-value <0.01. Reporter ions extracted within 20 ppm and rejected if any channels were absent. Quantification ratios were median-normalised and log_2_ transformed. A protein was considered modulated in one group relative to controls when its log_2_ ratio was above or below±1 s.d. across all biological replicates. Proteomics data were deposited to the ProteomeXchange Consortium via the PRIDE partner repository (data set identifier PXD001540).

Principal component analysis using reporter ion ratios of the study proteome and heatmap construction of reproducibly modulated proteins were generated using BioConductor-R (version 2.15.1) and g-plots in R (version 3.0.2). MetaCore (GeneGo, St Joseph, MI, USA) and BiNGO were applied to identify prebuilt processed networks and gene ontology terms overrepresented in the modulated proteome. FDR corrected *P*-values <0.05 were considered significant.

## Results

The proteomic analysis resulted in the identification of 18 543 peptides surrogate to 6695 unique proteins (Supplementary Table 1). The average coefficient variation for the iTRAQ ratios of all proteins profiled across biological replicates was determined to be 16%, 12% and 13% for the CH, HC and HH groups, respectively. Analogous coefficient variation values between biological replicates were reported by the authors using similar proteomics methodologies.^8, 9^ Principal component analysis of the analysed proteome showed clustering of mice perinatally exposed to high-fat diet irrespective of their current diet (Figure 1b). A total of 662 proteins (Supplementary Table 2) were found modulated in at least one of the three groups. Their hierarchical clustering revealed that the cerebral cortex of mice whose mothers were on high-fat diet, regardless their own diet, shared a very similar endophenotypic portrait, which was distinct from that of mice whose mothers were on control diet (Figure 1c).

**Figure 2:**
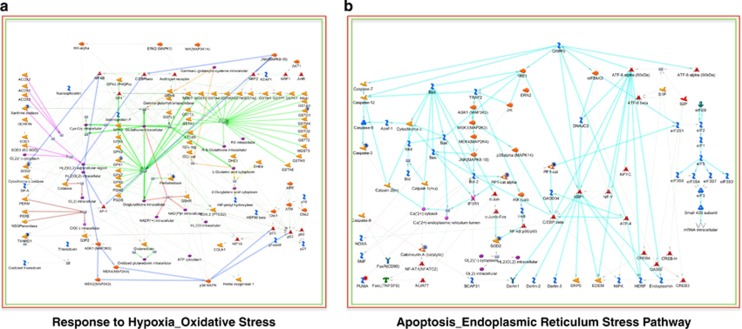
Process Network Analysis using MetaCore: significant enrichment for (**a**) response to hypoxia/oxidative stress (FDR corrected *P*-value=3.53E-02) and (**b**) apoptosis/endoplasmic reticulum stress pathway (FDR corrected *P*-value=1.45E-02) in the cerebral cortex of the adult offspring as a result of maternal high-fat diet. Analysed proteins are denoted with a circle (red=upregulation, blue=downregulation).

Of the modulated proteins, 251 were common in the HC and HH groups (Figure 1d and Supplementary Table 3). MetaCore analysis showed that response to hypoxia/oxidative stress (FDR corrected *P-*value=1.45E-02) and the apoptosis/endoplasmic reticulum stress pathway (FDR corrected *P*-value=3.53E-02) were significantly overrepresented processes only in the cerebral cortices of mice perinatally exposed to high-fat diet (Figure 2). Induction of apoptosis by oxidative stress was cross-referenced with BiNGO (Supplementary Figure 1). By contrast these functions were not significantly enriched in CH mice.

## Discussion

Our study constitutes the most comprehensive proteomic profiling of the mouse cerebral cortex to date. The results provide novel evidence of an association between maternal high-fat diet with endophenotypic alterations in the cerebral cortex of the adult offspring. Epigenetic DNA methylation patterns may be a possible mechanism by which this ‘nutritional imprinting' was established,^10^ but deciphering this was beyond the scope of this study.

*In silico* interpretation of proteins commonly modulated in the cerebral cortex of mice perinatally exposed to high-fat diet revealed a significant overrepresentation of response to hypoxia/oxidative stress and apoptosis/endoplasmic reticulum stress (Figure 2), both suggestive of a progression to a neurodegenerative phenotype.

The analysed enzymes associated with response to hypoxia/oxidative stress were peroxiredoxin-1, peroxiredoxin-2, peroxiredoxin-4, superoxide dismutase (Mn) mitochondrial, glutathione peroxidase 1, glutathione S-transferase omega 1, thioredoxin reductase 1 cytoplasmic and xanthine dehydrogenase/oxidase. The downregulation of these reactive oxygen species (ROS) scavenging proteins could suggest increased oxidative stress in the cerebral cortex of adult mice as a result of maternal obesity.

It has been previously reported that maternal high-fat diet leads to increased oxidative stress in brain regions of the adult offspring by measuring levels of 3-nitrotyrosine and protein carboxylation, providing thus an indirect cue to oxidative stress.^11^ The oxidative metabolism of fatty acids typically generates ROS, that cause the covalent modification of intracellular nucleophiles such as mitochondrial DNA and proteins, including those involved in redox processing.^12^ The accumulation of oxidative damage products in the cytoplasm of neurons precedes the deposition of Aβ in cerebral amyloid angiopathy and Alzheimer's disease.^13^ The ROS-mediated covalent modification of Aβ, among other proteins, may also have a role to its reduced clearance.^14^

Global cerebral ischaemia/reperfusion (I/R) is a useful model on the effects of increased oxidative stress in brain regions. I/R leads to increased free radical production and oxidative stress, which in turn can cause neuronal apoptosis.^15^ Through the I/R model, it has been found that by reducing oxidative stress neuronal damage in the brain could be prevented.^16^ As neuronal apoptosis is an irreversible process, ameliorating oxidative stress could reduce the risk of neurodegenerative disease. A recent study showed the neuroprotective effects of β-myrcene, a natural product derived from thyme and parsley, in mice following I/R.^17^ In this study, β-myrcene treatment concomitantly with the induction of I/R reduced oxidative stress and prevented neurodegeneration via the induction of ROS-scavenging enzymes such as glutathione peroxidase and superoxide dismutase.

Another study showed that pre-treatment of Swiss albino mice with S-allyl cysteine, a phytochemical in garlic, prevented the cognitive and behavioural impairment of streptozotocin-induced experimental dementia. This effect was attributed to the induction of ROS-scavenging proteins, including glutathione peroxidase.^18^ Similar trends have been observed for fruit-derived polyphenols. Despite their low systemic bioavailability and slow reactivity in the direct sequestration of ROS species, polyphenols trigger cellular and molecular mechanisms, in part through the induction of ROS-scavenging enzymes, that result in reduced neuronal oxidative damage and cognitive decline.^19^ Denny Joseph *et al.*^20^ highlighted the efficiency of combining fish oil and quercetin, a compound found in red onions, in lowering oxidative stress in rat brain and thus protecting against neurodegeneration.

Study limitations include the non-validated mass spectrometry analysis results using alternative approaches (for example, immunohistochemistry), the lack of functional assays and protein oxidation status assessment. These constitute objectives for prospective studies. In conclusion, our study demonstrated that maternal obesity resulted in distinct proteomic portraits, suggesting a neurogenerative phenotype in the adult offspring cerebral cortex.

## Supplementary Material

Supplementary Method 1

Supplementary Method 2
