# Supplementary material for: Are you also what your mother eats? Distinct proteomic portrait as a result of maternal high-fat diet in the cerebral cortex of the adult mouse
Source: Int J Obes (Lond). 2015 Apr 21;39(8):1325–8. doi: 10.1038/ijo.2015.35 (PMC5399160; doi:10.1038/ijo.2015.35)
Supplement: Supplementary Method 1 [file ijo201535x1.docx]

**Supplementary Methods 1**

High-pH Reverse Phase (RP) Peptide Fractionation

High-PH RP C_8_ fractionation of the iTRAQ labelled peptides was performed on the Ultimate (LC Packings, USA) HPLC system using the Waters, XBridge C8 column (150 × 3 mm, 3.5 μm particle). The composition of mobile phase (A) was 2% acetonitrile, 0.1% ammonium hydroxide, whereas the composition of mobile phase (B) was 100% acetonitrile, 0.1% ammonium hydroxide. The dried-up peptide pellet was dissolved in 100 μL of 95% mobile phase (A) and 5% mobile phase (B) with extensive vortex mixing. The sample was centrifuged at 13K rpm for 10 min, and the supernatant was injected in a 100 μL sample loop. The gradient used was the following: for 10 min isocratic 5% (B), for 60 min gradient up to 30% (B), for 20 min gradient up to 85% (B), for 10 min isocratic 85% (B), for 10 min down at 2% (B) at a flow rate 0.2 mL/min. Signal response was monitored at 215 and the column temperature was set at 30 °C. Fraction collection was performed on a peak-dependent manner and a total of 58 fractions were collected. The peptide fractions were dried-up using a speedvac concentrator for 4−5 h at 30°C and stored at −20 °C until the LC−MS analysis.
