# Supplementary material for: Are you also what your mother eats? Distinct proteomic portrait as a result of maternal high-fat diet in the cerebral cortex of the adult mouse
Source: Int J Obes (Lond). 2015 Apr 21;39(8):1325–8. doi: 10.1038/ijo.2015.35 (PMC5399160; doi:10.1038/ijo.2015.35)
Supplement: Supplementary Method 2 [file ijo201535x2.docx]

**Supplementary Methods 2**

LC-FT-Orbitrap MS Analysis

The LC−MS experiments were performed on the Dionex Ultimate 3000 UHPLC system coupled with the high resolution nano-ESI LTQ-Velos Pro Orbitrap-Elite mass spectrometer (Thermo Scientific). Individual peptide fractions were reconstituted in 30 μL of loading solution (2% acetonitrile, 0.1% formic acid), and a 10 μL volume was loaded on the Acclaim PepMap 100, 100 μm × 2 cm C18, 5 μm particle trapping column with the ulPickUp Injection mode using the loading pump at 5 μL/min flow rate for 5 min. Two separate analyses for HCD and CID fragmentation for each one of the collected fractions were performed. For the analytical separation the Acclaim PepMap RSLC, 75 μm × 25 cm, nanoViper, C18, 2 μm particle column retrofitted to a PicoTip emitter (FS360-20-10-D-20-C7) was used for multistep gradient elution. Mobile phase (A) was composed of 2% acetonitrile, 0.1% formic acid, and mobile phase (B) was composed of 100% acetonitrile, 0.1% formic acid. The gradient elution method at flow rate 300 nL/min was as follows: for 10 min isocratic gradient 5% (B), for 90 min gradient up to 40% (B), for 10 min gradient up to 85% (B), for 10 min isocratic 85% (B), for 5 min down to 2% (B), for 8 min isocratic equilibration 3% (B) at 35 °C. Separated peptides were transferred to the gaseous phase with positive ion electrospray ionization applying a voltage of 2.5 kV. Top 10 multiply charged precursor isotopic clusters with m/z value larger than 350 or smaller than 1900 and intensity threshold 500 counts were selected with FT mass resolution of 120000 and isolated for HCD fragmentation within a mass window of 1.2 Da. Tandem mass spectra were acquired with FT resolution of 15 000 with in m/z range of 100−1900. For the CID experiments, top 20 precursors were selected with FT mass resolution of 240 000 within a mass window of 2 Da. Normalized collision energy was set to 35, and already targeted precursors were dynamically excluded for further isolation and activation for 30 s with 5 ppm mass tolerance for both types of analysis.
