# Supplementary material for: Are you also what your mother eats? Distinct proteomic portrait as a result of maternal high-fat diet in the cerebral cortex of the adult mouse
Source: Int J Obes (Lond). 2015 Apr 21;39(8):1325–8. doi: 10.1038/ijo.2015.35 (PMC5399160; doi:10.1038/ijo.2015.35)
Supplement: Supplementary Table 1 [file ijo201535x3.pdf]

Accession	Description	ΣCoverage	Σ# Proteins	Σ# Unique Peptides	Σ# Peptides	Σ# PSMs	114/113	115/113	115/114	116/113	116/114	117/113	117/114	118/113	118/114	119/113	119/114	121/113	121/114	MW [kDa]	calc. pI
Q8R0J1	Pleckstrin homology domain-containing family G member 6 OS=Mus musculus GN=Plekhg6 PE=1 SV=2 - [PKHG6_MOUSE]	1.78	1	1	1	1	0.09	0.13	1.48	0.09	1.00	0.10	1.16	0.16	1.79	0.13	1.48	0.11	1.26	89.09	7.53
Q9DCD6	Gamma-aminobutyric acid receptor-associated protein OS=Mus musculus GN=Gabarap PE=1 SV=2 - [GBRAP_MOUSE]	34.19	2	1	4	21	0.82	0.41	0.50	0.69	0.84	0.32	0.39	0.28	0.34	0.28	0.34	0.27	0.32	13.91	8.79
A2AQ34	Ubiquitin carboxyl-terminal hydrolase 20 (Fragment) OS=Mus musculus GN=Usp20 PE=2 SV=1 - [A2AQ34_MOUSE]	21.21	5	1	1	1	0.74	0.81	1.09	0.80	1.09	0.58	0.78	0.35	0.47	0.60	0.81	0.57	0.78	6.98	5.19
Q3UGR5	Haloacid dehalogenase-like hydrolase domain-containing protein 2 OS=Mus musculus GN=Hdhd2 PE=1 SV=2 - [HDHD2_MOUSE]	75.68	4	13	13	120	1.16	0.85	0.67	0.81	0.70	0.36	0.31	0.35	0.27	0.35	0.30	0.39	0.34	28.71	6.05
Q11136	Xaa-Pro dipeptidase OS=Mus musculus GN=Pepd PE=2 SV=3 - [PEPD_MOUSE]	26.57	2	12	12	28	1.02	0.88	0.87	0.80	0.84	0.38	0.34	0.35	0.34	0.36	0.34	0.33	0.30	54.99	5.78
P61965	WD repeat-containing protein 5 OS=Mus musculus GN=Wdr5 PE=1 SV=1 - [WDR5_MOUSE]	19.46	2	4	4	9	0.99	0.65	0.71	0.92	0.91	0.38	0.37	0.37	0.35	0.40	0.38	0.45	0.44	36.57	8.27
E9Q1B3	Uncharacterized protein OS=Mus musculus GN=Gm7275 PE=4 SV=1 - [E9Q1B3_MOUSE]	4.86	1	1	1	2	1.07	2.09	1.96	0.89	0.84	0.40	0.37	0.37	0.35	0.39	0.37	0.41	0.39	29.07	7.11
P05202	Aspartate aminotransferase, mitochondrial OS=Mus musculus GN=Got2 PE=1 SV=1 - [AATM_MOUSE]	80.23	1	38	39	843	1.01	0.84	0.87	0.83	0.83	0.36	0.37	0.37	0.38	0.37	0.38	0.42	0.43	47.38	9.00
Q3TTY0-3	Isoform 3 of Phospholipase B1, membrane-associated OS=Mus musculus GN=Plb1 - [PLB1_MOUSE]	1.08	2	1	1	1	1.42	0.86	0.60	0.97	0.68	0.52	0.36	0.38	0.27	0.39	0.28	0.77	0.54	102.64	6.96
P61963	DDB1- and CUL4-associated factor 7 OS=Mus musculus GN=Dcaf7 PE=2 SV=1 - [DCAF7_MOUSE]	19.88	1	5	5	10	1.03	0.83	0.80	0.88	0.86	0.34	0.34	0.38	0.37	0.43	0.42	0.49	0.47	38.90	5.52

Q9DBB8	Trans-1,2-dihydrobenzene-1,2-diol dehydrogenase OS=Mus musculus GN=Dhdh PE=2 SV=1 - [DHDH_MOUSE]	33.03	1	9	9	16	1.24	0.92	0.75	0.87	0.72	0.36	0.31	0.39	0.32	0.39	0.35	0.52	0.44	36.28	6.44
P29974	cGMP-gated cation channel alpha-1 OS=Mus musculus GN=Cnga1 PE=2 SV=2 - [CNGA1_MOUSE]	2.34	1	1	1	1	5.68	10.29	1.81	0.38	0.07	0.31	0.05	0.39	0.07	0.41	0.07	0.26	0.05	79.41	7.58
P05201	Aspartate aminotransferase, cytoplasmic OS=Mus musculus GN=Got1 PE=1 SV=3 - [AATC_MOUSE]	75.79	2	35	35	491	1.00	0.91	0.89	0.83	0.83	0.38	0.39	0.40	0.39	0.41	0.40	0.44	0.44	46.22	7.14
Q9CQ65	S-methyl-5'-thioadenosine phosphorylase OS=Mus musculus GN=Mtap PE=2 SV=1 - [MTAP_MOUSE]	38.52	1	8	8	22	1.17	1.00	0.84	0.88	0.74	0.41	0.35	0.40	0.32	0.43	0.37	0.46	0.41	31.04	7.14
O55208	Factor in the germline alpha OS=Mus musculus GN=Figla PE=2 SV=1 - [FIGLA_MOUSE]	8.76	1	1	1	1	1.19	0.86	0.72	1.07	0.90	0.34	0.28	0.40	0.34	0.43	0.36	0.62	0.52	21.52	7.91
Q8VEB4	Group XV phospholipase A2 OS=Mus musculus GN=Pla2g15 PE=1 SV=1 - [PAG15_MOUSE]	11.65	1	4	4	8	1.03	1.02	0.95	0.90	0.81	0.41	0.41	0.40	0.36	0.47	0.44	0.48	0.48	47.28	6.47
Q8VDK1-	Isoform 2 of Nitrilase homolog 1 OS=Mus musculus GN=Nit1 - [NIT1_MOUSE]	47.24	5	10	10	40	1.06	0.88	0.77	0.87	0.84	0.40	0.35	0.40	0.37	0.42	0.39	0.43	0.42	31.87	7.05
O70494-3	Isoform 3 of Transcription factor Sp3 OS=Mus musculus GN=Sp3 - [SP3_MOUSE]	2.23	3	1	1	3	1.05	3.61	3.44	0.88	0.84	1.77	1.68	0.40	0.38	1.13	1.08	1.06	1.01	76.68	6.33
Q99KJ6	Beta-glucuronidase OS=Mus musculus GN=Gusb PE=2 SV=1 - [Q99KJ6_MOUSE]	4.40	2	1	1	2	0.99	1.06	1.07	0.91	0.92	0.37	0.38	0.41	0.41	0.45	0.46	0.42	0.42	28.49	6.00
D3Z0K8	Thioredoxin reductase 2, mitochondrial OS=Mus musculus GN=Txnrd2 PE=2 SV=1 - [D3Z0K8_MOUSE]	31.57	7	10	11	36	0.97	0.83	0.83	0.81	0.82	0.41	0.41	0.41	0.36	0.36	0.38	0.43	0.43	53.04	9.04
F6W7I2	Deoxyhypusine synthase (Fragment) OS=Mus musculus GN=Dhps PE=2 SV=1 - [F6W7I2_MOUSE]	8.05	2	1	1	2	1.04	1.18	1.13	0.65	0.62	0.41	0.39	0.41	0.39	0.51	0.49	0.35	0.34	16.26	7.12
Q8R086	Sulfite oxidase, mitochondrial OS=Mus musculus GN=Suox PE=1 SV=2 - [SUOX_MOUSE]	3.66	1	1	1	2	0.99	0.93	0.93	1.14	1.15	0.69	0.69	0.41	0.41	0.56	0.56	0.91	0.91	60.72	6.54

P63005	Platelet-activating factor acetylhydrolase IB subunit alpha OS=Mus musculus GN=Pafah1b1 PE=1 SV=2 - [LIS1_MOUSE]	56.10	3	21	21	128	0.99	0.77	0.79	0.84	0.84	0.38	0.39	0.41	0.42	0.42	0.41	0.45	0.44	46.64	7.37
H3BLA9	Sentrin-specific protease 6 (Fragment) OS=Mus musculus GN=Senp6 PE=2 SV=1 - [H3BLA9_MOUSE]	3.43	1	1	1	1	1.17	1.05	0.89	0.90	0.77	0.42	0.36	0.42	0.36	0.51	0.44	0.57	0.49	23.50	5.85
D3YZU6	Hydroxyacylglutathione hydrolase-like protein OS=Mus musculus GN=Haghl PE=4 SV=1 - [D3YZU6_MOUSE]	45.53	4	7	7	21	0.98	0.90	0.89	0.81	0.86	0.56	0.49	0.42	0.41	0.44	0.42	0.46	0.52	26.09	5.91
G5E8Q8	MCG115189 OS=Mus musculus GN=Gpr116 PE=4 SV=1 - [G5E8Q8_MOUSE]	0.89	1	1	1	1	0.78	1.02	1.30	0.86	1.10	0.52	0.67	0.43	0.55	0.65	0.83	0.79	1.02	149.29	7.02
P23492	Purine nucleoside phosphorylase OS=Mus musculus GN=Pnp PE=1 SV=2 - [PNPH_MOUSE]	58.82	3	13	13	30	1.09	0.95	0.91	0.87	0.80	0.42	0.39	0.43	0.44	0.44	0.42	0.54	0.49	32.26	6.16
P20108	Thioredoxin-dependent peroxide reductase, mitochondrial OS=Mus musculus GN=Prdx3 PE=1 SV=1 - [PRDX3_MOUSE]	40.47	1	9	9	105	1.04	0.77	0.72	0.84	0.82	0.45	0.42	0.43	0.41	0.43	0.41	0.47	0.43	28.11	7.58
Q3UR97	Protein Snx21 OS=Mus musculus GN=Snx21 PE=2 SV=1 - [Q3UR97_MOUSE]	24.24	4	3	3	3	1.00	0.83	0.82	0.99	0.99	0.54	0.54	0.43	0.43	0.46	0.46	0.47	0.47	40.28	5.36
Q61532	Mitogen-activated protein kinase 6 OS=Mus musculus GN=Mapk6 PE=1 SV=3 - [MK06_MOUSE]	1.39	1	1	1	1	1.41	0.81	0.57	1.00	0.71	0.42	0.30	0.43	0.30	0.46	0.33	0.55	0.39	82.15	5.20
Q8C7K6	Prenylcysteine oxidase-like OS=Mus musculus GN=Pcyox1l PE=2 SV=1 - [PCYXL_MOUSE]	11.92	1	4	4	7	1.08	1.06	0.92	0.87	0.81	0.47	0.43	0.43	0.37	0.56	0.49	0.58	0.54	54.84	7.65
Q5M8N0	CB1 cannabinoid receptor-interacting protein 1 OS=Mus musculus GN=Cnrip1 PE=1 SV=1 - [CNRP1_MOUSE]	62.20	2	6	6	40	0.95	0.73	0.79	0.76	0.85	0.46	0.49	0.43	0.47	0.48	0.50	0.43	0.47	18.60	7.96
Q9CR39	WD repeat domain phosphoinositide-interacting protein 3 OS=Mus musculus GN=Wdr45b PE=2 SV=2 - [WIPI3_MOUSE]	15.12	3	4	4	10	1.13	0.85	0.76	0.89	0.79	0.47	0.39	0.44	0.38	0.45	0.39	0.53	0.46	38.00	7.56

P35505	Fumarylacetoacetase OS=Mus musculus GN=Fah PE=1 SV=2 - [FAAA_MOUSE]	27.92	2	8	8	22	1.13	0.87	0.79	0.79	0.73	0.40	0.34	0.44	0.39	0.43	0.40	0.46	0.40	46.15	7.18
Q9CQT1	Methylthioribose-1-phosphate isomerase OS=Mus musculus GN=Mri1 PE=2 SV=1 - [MTNA_MOUSE]	17.07	3	5	5	15	0.96	0.91	0.97	0.73	0.76	0.42	0.42	0.44	0.46	0.40	0.44	0.41	0.44	39.39	5.91
O70472	Transmembrane protein 131 OS=Mus musculus GN=Tmem131 PE=1 SV=2 - [TM131_MOUSE]	1.17	1	1	1	1	5.61	4.46	0.79	0.32	0.06	0.32	0.06	0.44	0.08	0.52	0.09	0.88	0.16	204.52	8.54
Q640M6	Glycerophosphodiester phosphodiesterase domain-containing protein 5 OS=Mus musculus GN=Gdpd5 PE=2 SV=1 - [GDPD5_MOUSE]	2.14	1	1	1	2	0.80	1.30	1.62	1.37	1.72	0.55	0.68	0.44	0.56	0.89	1.12	1.04	1.31	68.85	8.00
Q61646	Haptoglobin OS=Mus musculus GN=Hp PE=1 SV=1 - [HPT_MOUSE]	3.75	1	1	1	1	1.73	1.81	1.04	1.36	0.79	1.06	0.61	0.45	0.26	18.79	10.90	8.46	4.91	38.73	6.29
Q6T264	Mastermind-like protein 1 OS=Mus musculus GN=Maml1 PE=1 SV=2 - [MAML1_MOUSE]	2.25	1	1	1	1	4.69	6.01	1.28	0.39	0.08	0.29	0.06	0.45	0.10	0.30	0.06	0.39	0.08	107.66	7.85
O89106	Bis(5'-adenosyl)-triphosphatase OS=Mus musculus GN=Fhit PE=2 SV=3 - [FHIT_MOUSE]	56.00	5	7	7	18	1.04	0.93	0.84	0.78	0.73	0.53	0.45	0.45	0.42	0.38	0.36	0.46	0.45	17.22	6.73
Q571E4	N-acetylgalactosamine-6-sulfatase OS=Mus musculus GN=Galns PE=2 SV=2 - [GALNS_MOUSE]	6.35	1	2	2	2	0.99	1.12	1.13	0.71	0.72	0.45	0.46	0.45	0.45	0.42	0.42	0.47	0.48	57.64	6.52
Q8CFD4	Sorting nexin-8 OS=Mus musculus GN=Snx8 PE=2 SV=1 - [SNX8_MOUSE]	1.74	1	1	1	2	1.03	0.81	0.79	0.96	0.94	0.46	0.45	0.45	0.44	0.48	0.47	0.66	0.64	52.03	8.03
Q6NT99	Dual specificity protein phosphatase 23 OS=Mus musculus GN=Dusp23 PE=2 SV=1 - [DUS23_MOUSE]	19.33	1	3	3	5	1.11	1.05	0.97	0.94	0.81	0.45	0.40	0.45	0.43	0.46	0.44	0.68	0.61	16.63	8.21
Q5RKZ7-[illegible]	Isoform 3 of Molybdenum cofactor biosynthesis protein 1 OS=Mus musculus GN=Mocs1 - [MOCS1_MOUSE]	3.21	2	1	1	1	1.05	0.66	0.63	0.79	0.75	0.54	0.51	0.46	0.43	0.49	0.46	0.62	0.59	26.61	8.75
P58058	NAD kinase OS=Mus musculus GN=Nadk PE=1 SV=2 - [NADK_MOUSE]	4.78	4	2	2	4	0.87	1.19	1.37	0.95	1.09	0.46	0.52	0.46	0.53	0.44	0.51	0.49	0.57	48.57	6.52

O70250	Phosphoglycerate mutase 2 OS=Mus musculus GN=Pgam2 PE=1 SV=3 - [PGAM2_MOUSE]	36.36	1	3	9	85	1.18	0.77	0.65	0.81	0.66	0.51	0.43	0.47	0.39	0.55	0.46	0.45	0.39	28.81	8.50
Q8QZR5	Alanine aminotransferase 1 OS=Mus musculus GN=Gpt PE=2 SV=3 - [ALAT1_MOUSE]	35.28	1	12	12	24	1.09	0.90	0.86	0.80	0.75	0.44	0.40	0.47	0.46	0.39	0.42	0.48	0.43	55.11	6.64
Q91X72	Hemopexin OS=Mus musculus GN=Hpx PE=1 SV=2 - [HEMO_MOUSE]	17.61	1	5	5	8	0.92	0.84	0.82	0.67	0.80	0.67	0.72	0.47	0.60	1.00	1.08	0.69	0.73	51.29	7.80
Q99KN2	Probable cytosolic iron-sulfur protein assembly protein CIAO1 OS=Mus musculus GN=Ciao1 PE=2 SV=1 - [CIAO1_MOUSE]	27.14	3	6	6	10	0.94	0.98	1.04	0.87	0.89	0.47	0.53	0.47	0.54	0.48	0.56	0.53	0.58	37.61	4.88
Q8R3P0	Aspartoacylase OS=Mus musculus GN=Aspa PE=1 SV=2 - [ACY2_MOUSE]	47.76	3	9	9	35	0.73	0.84	1.14	0.79	1.07	0.45	0.62	0.47	0.67	0.46	0.62	0.50	0.66	35.32	6.58
Q61233	Plastin-2 OS=Mus musculus GN=Lcp1 PE=1 SV=4 - [PLSL_MOUSE]	36.68	5	14	18	46	1.07	0.97	0.94	0.97	0.88	0.47	0.46	0.48	0.47	0.50	0.51	0.50	0.50	70.10	5.33
P51174	Long-chain specific acyl-CoA dehydrogenase, mitochondrial OS=Mus musculus GN=Acadl PE=2 SV=2 - [ACADL_MOUSE]	42.56	1	17	17	58	0.98	0.83	0.89	0.90	0.92	0.48	0.50	0.48	0.51	0.50	0.49	0.54	0.55	47.88	8.31
Q9Z1Z2	Serine-threonine kinase receptor-associated protein OS=Mus musculus GN=Strap PE=1 SV=2 - [STRAP_MOUSE]	60.57	1	15	15	66	0.97	0.78	0.87	0.89	0.92	0.47	0.50	0.48	0.51	0.48	0.48	0.51	0.51	38.42	5.12
Q3UWE6	MCG14935, isoform CRA_a OS=Mus musculus GN=Wdr20a PE=2 SV=1 - [Q3UWE6_MOUSE]	16.17	2	8	8	19	0.99	1.01	0.95	0.89	0.89	0.47	0.48	0.48	0.45	0.45	0.44	0.53	0.50	62.85	8.00
Q8VCN5	Cystathionine gamma-lyase OS=Mus musculus GN=Cth PE=1 SV=1 - [CGL_MOUSE]	10.05	1	3	3	7	0.90	1.04	1.14	0.93	1.02	0.45	0.47	0.48	0.49	0.52	0.54	0.55	0.56	43.54	7.65
Q9Z2W0	Aspartyl aminopeptidase OS=Mus musculus GN=Dnpep PE=2 SV=2 - [DNPEP_MOUSE]	49.68	1	16	16	77	1.09	0.83	0.75	0.86	0.78	0.47	0.42	0.48	0.44	0.50	0.44	0.53	0.49	52.17	7.25
G3UYJ3	Solute carrier family 25 member 51 (Fragment) OS=Mus musculus GN=Slc25a51 PE=2 SV=1 - [G3UYJ3_MOUSE]	4.59	4	1	1	3	0.88	0.96	1.09	1.01	1.15	0.59	0.67	0.48	0.55	0.60	0.68	0.73	0.83	22.16	9.44

Q60571	Corticotropin-releasing factor-binding protein OS=Mus musculus GN=Crhbp PE=2 SV=1 - [CRHBP_MOUSE]	3.42	1	1	1	2	1.34	1.11	0.82	0.95	0.71	0.49	0.36	0.48	0.36	0.43	0.32	0.66	0.49	36.02	6.60
P54729	NEDD8 ultimate buster 1 OS=Mus musculus GN=Nub1 PE=1 SV=2 - [NUB1_MOUSE]	2.44	1	1	1	1	0.55	0.81	1.48	0.39	0.72	0.50	0.91	0.49	0.88	0.58	1.05	0.62	1.14	70.26	5.88
Q8CFI5	Probable proline--tRNA ligase, mitochondrial OS=Mus musculus GN=Pars2 PE=2 SV=2 - [SYPM_MOUSE]	19.37	2	7	7	12	1.07	0.81	0.82	0.88	0.81	0.53	0.51	0.49	0.50	0.54	0.51	0.59	0.58	53.48	7.84
Q64288	Olfactory marker protein OS=Mus musculus GN=Omp PE=1 SV=3 - [OMP_MOUSE]	56.44	1	6	6	16	0.73	0.34	0.44	0.50	0.66	1.32	1.88	0.49	0.61	0.70	0.93	1.55	2.20	18.85	5.14
P50428	Arylsulfatase A OS=Mus musculus GN=Arsa PE=2 SV=2 - [ARSA_MOUSE]	18.38	2	6	6	19	1.03	0.79	0.77	0.88	0.83	0.46	0.44	0.49	0.50	0.49	0.46	0.58	0.55	53.71	5.87
Q9CPU0	Lactoylglutathione lyase OS=Mus musculus GN=Glo1 PE=1 SV=3 - [LGUL_MOUSE]	47.28	1	9	9	45	0.98	0.71	0.75	0.85	0.81	0.47	0.50	0.49	0.50	0.50	0.48	0.51	0.51	20.80	5.47
Q9R1P3	Proteasome subunit beta type-2 OS=Mus musculus GN=Psmb2 PE=1 SV=1 - [PSB2_MOUSE]	50.75	1	7	7	16	0.94	1.29	1.26	1.02	1.11	0.57	0.63	0.50	0.52	0.57	0.58	0.70	0.77	22.89	7.02
Q9QXP7	Complement C1q tumor necrosis factor-related protein 1 OS=Mus musculus GN=C1qtnf1 PE=2 SV=1 - [C1QT1_MOUSE]	2.85	1	1	1	1	1.25	1.16	0.93	0.92	0.73	0.53	0.42	0.50	0.40	0.50	0.40	0.52	0.42	31.99	5.87
Q80TL4-2	Isoform 2 of Protein KIAA1045 OS=Mus musculus GN=Kiaa1045 - [K1045_MOUSE]	38.84	3	1	13	86	0.60	0.80	1.33	0.49	0.81	0.58	0.96	0.50	0.83	0.48	0.80	0.61	1.03	41.30	5.45
Q3V1L4	Cytosolic purine 5'-nucleotidase OS=Mus musculus GN=Nt5c2 PE=1 SV=2 - [5NTC_MOUSE]	6.07	3	3	3	7	1.01	0.87	0.85	0.85	0.88	0.51	0.52	0.50	0.44	0.51	0.53	0.58	0.55	64.77	6.21
Q8VEB6	Zinc phosphodiesterase ELAC protein 1 OS=Mus musculus GN=Elac1 PE=2 SV=1 - [RNZ1_MOUSE]	20.44	2	5	5	10	1.14	1.04	0.84	0.93	0.80	0.53	0.49	0.50	0.40	0.55	0.47	0.65	0.56	39.71	6.60
P40142	Transketolase OS=Mus musculus GN=Tkt PE=1 SV=1 - [TKT_MOUSE]	66.13	2	37	37	334	1.03	0.80	0.77	0.83	0.81	0.47	0.46	0.50	0.48	0.50	0.48	0.54	0.49	67.59	7.50
Q3U6C5	Voltage-gated potassium channel subunit beta-1 OS=Mus musculus GN=Kcnab1 PE=2 SV=1 - [Q3U6C5_MOUSE]	31.19	5	3	6	8	1.02	0.98	0.95	0.85	0.84	0.57	0.58	0.51	0.52	0.78	0.81	0.59	0.61	33.18	8.79

P01872	Ig mu chain C region secreted form OS=Mus musculus GN=Igh-6 PE=1 SV=2 - [IGHM_MOUSE]	6.39	2	2	2	5	0.74	0.93	1.26	0.46	0.66	0.75	1.07	0.51	0.66	1.20	1.53	0.68	0.97	49.94	7.01
Q8BM88	Cathepsin O OS=Mus musculus GN=Ctso PE=2 SV=1 - [CATO_MOUSE]	3.21	1	1	1	2	1.02	1.35	1.33	0.79	0.78	0.47	0.46	0.51	0.50	0.45	0.45	0.46	0.46	34.70	7.80
P47857	6-phosphofructokinase, muscle type OS=Mus musculus GN=Pfkm PE=1 SV=3 - [K6PF_MOUSE]	55.90	2	34	39	367	1.03	0.91	0.88	0.95	0.92	0.53	0.51	0.51	0.49	0.53	0.52	0.56	0.54	85.21	8.00
E9PYT3	Atlastin-3 OS=Mus musculus GN=Atl3 PE=2 SV=1 - [E9PYT3_MOUSE]	3.92	2	1	1	2	0.59	0.41	0.69	0.56	0.96	0.53	0.90	0.51	0.87	0.51	0.87	0.49	0.84	60.19	5.81
Q9JMH6-	Isoform 2 of Thioredoxin reductase 1, cytoplasmic OS=Mus musculus GN=Txnrd1 - [TRXR1_MOUSE]	43.49	2	13	14	29	1.13	0.95	0.80	0.92	0.82	0.45	0.41	0.51	0.48	0.50	0.48	0.57	0.54	54.51	6.33
P24547	Inosine-5'-monophosphate dehydrogenase 2 OS=Mus musculus GN=Impdh2 PE=1 SV=2 - [IMDH2_MOUSE]	24.51	1	12	13	32	1.10	0.99	0.89	1.04	0.93	0.57	0.51	0.51	0.48	0.59	0.52	0.62	0.54	55.78	7.28
Q9DCF9-	Isoform 2 of Translocon-associated protein subunit gamma OS=Mus musculus GN=Ssr3 - [SSRG_MOUSE]	7.82	2	1	1	1	0.82	1.07	1.31	1.05	1.28	0.47	0.57	0.51	0.63	0.57	0.70	0.61	0.74	20.69	9.35
Q923A2	Protein Spindly OS=Mus musculus GN=Spdl1 PE=1 SV=2 - [SPDLY_MOUSE]	1.81	1	1	1	1	0.90	1.11	1.23	0.26	0.29	0.75	0.84	0.51	0.57	0.79	0.88	0.52	0.58	70.19	6.00
Q8C605	6-phosphofructokinase OS=Mus musculus GN=Pfkp PE=2 SV=1 - [Q8C605_MOUSE]	55.23	5	36	40	275	1.01	0.92	0.93	0.95	0.94	0.53	0.53	0.52	0.53	0.53	0.53	0.57	0.56	85.49	6.89
Q8BTY1	Kynurenine--oxoglutarate transaminase 1 OS=Mus musculus GN=Ccbl1 PE=2 SV=1 - [KAT1_MOUSE]	19.34	3	8	8	19	1.12	0.85	0.76	0.79	0.70	0.52	0.43	0.52	0.46	0.56	0.48	0.62	0.52	47.53	6.95
Q924B0	Inositol (Myo)-1(Or 4)-monophosphatase 1 OS=Mus musculus GN=Impa1 PE=2 SV=1 - [Q924B0_MOUSE]	53.79	4	14	14	83	1.21	0.89	0.75	1.00	0.81	0.49	0.40	0.52	0.41	0.57	0.50	0.64	0.49	30.41	5.19
Q9CPY7-	Isoform 2 of Cytosol aminopeptidase OS=Mus musculus GN=Lap3 - [AMPL_MOUSE]	73.98	2	29	29	188	1.11	0.87	0.82	0.85	0.78	0.54	0.51	0.52	0.51	0.57	0.54	0.57	0.54	52.72	7.03

Q8BWT1	3-ketoacyl-CoA thiolase, mitochondrial OS=Mus musculus GN=Acaa2 PE=1 SV=3 - [THIM_MOUSE]	60.20	1	17	17	42	1.11	0.91	0.83	0.89	0.82	0.59	0.48	0.53	0.50	0.59	0.51	0.63	0.58	41.80	8.09
Q9CX80	Cytoglobin OS=Mus musculus GN=Cygb PE=2 SV=1 - [CYGB_MOUSE]	16.32	1	3	3	11	1.22	0.82	0.79	0.95	0.73	0.55	0.41	0.53	0.43	0.59	0.49	0.59	0.50	21.45	6.80
Q8CDI6	Coiled-coil domain-containing protein 158 OS=Mus musculus GN=Ccdc158 PE=2 SV=1 - [CD158_MOUSE]	2.34	2	2	3	3	2.62	2.58	0.98	0.76	0.29	0.55	0.21	0.53	0.20	0.54	0.21	0.69	0.26	126.75	6.40
Q8CBC8	Branched-chain-amino-acid aminotransferase OS=Mus musculus GN=Bcat1 PE=2 SV=1 - [Q8CBC8_MOUSE]	28.70	7	10	10	38	1.03	0.94	0.88	0.85	0.84	0.52	0.51	0.53	0.52	0.52	0.55	0.58	0.54	49.87	6.77
O88958	Glucosamine-6-phosphate isomerase 1 OS=Mus musculus GN=Gnpda1 PE=2 SV=3 - [GNPI1_MOUSE]	47.06	2	8	10	24	1.11	0.80	0.87	0.88	0.83	0.64	0.63	0.53	0.67	0.60	0.70	0.55	0.70	32.53	6.60
Q8BHA3	Probable D-tyrosyl-tRNA(Tyr) deacylase 2 OS=Mus musculus GN=Dtd2 PE=2 SV=1 - [DTD2_MOUSE]	32.14	2	4	4	8	1.13	0.78	0.65	0.75	0.67	0.57	0.45	0.53	0.43	0.41	0.38	0.60	0.56	18.22	7.91
E0CX20	Protein BUD31 homolog OS=Mus musculus GN=Bud31 PE=4 SV=1 - [E0CX20_MOUSE]	20.83	2	2	2	19	0.85	0.64	0.77	0.83	1.02	0.51	0.60	0.54	0.64	0.46	0.56	0.48	0.60	16.99	8.82
Q9WV54	Acid ceramidase OS=Mus musculus GN=Asah1 PE=1 SV=1 - [ASAH1_MOUSE]	35.79	3	12	12	25	1.00	0.87	0.91	0.88	0.89	0.46	0.50	0.54	0.49	0.53	0.56	0.57	0.57	44.64	8.46
Q8BG93	Probable 8-oxo-dGTP diphosphatase NUDT15 OS=Mus musculus GN=Nudt15 PE=1 SV=1 - [NUD15_MOUSE]	6.47	1	1	1	2	0.58	0.48	0.83	0.49	0.85	0.62	1.08	0.54	0.93	0.45	0.77	0.47	0.83	19.56	5.27
Q8R0N6-2	Isoform 2 of Hydroxyacid-oxoacid transhydrogenase, mitochondrial OS=Mus musculus GN=Adhfe1 - [HOT_MOUSE]	16.71	2	5	5	10	1.02	0.79	0.84	0.88	0.83	0.55	0.55	0.54	0.52	0.44	0.44	0.52	0.52	44.99	7.12
Q8BTR5	Dual specificity phosphatase 28 OS=Mus musculus GN=Dusp28 PE=1 SV=1 - [DUS28_MOUSE]	6.75	1	1	1	2	0.98	1.08	1.10	1.01	1.03	0.56	0.57	0.54	0.55	0.53	0.54	0.52	0.53	17.49	7.75
Q9JI46	Diphosphoinositol polyphosphate phosphohydrolase 1 OS=Mus musculus GN=Nudt3 PE=1 SV=1 - [NUDT3_MOUSE]	65.48	5	7	8	46	0.86	0.85	0.92	0.84	0.96	0.55	0.60	0.54	0.64	0.54	0.61	0.55	0.61	19.02	6.34

Q8CC86	Nicotinate phosphoribosyltransferase OS=Mus musculus GN=Naprt1 PE=2 SV=1 - [PNCB_MOUSE]	7.81	1	2	2	4	0.88	0.93	1.06	0.71	0.80	0.53	0.60	0.54	0.61	0.64	0.73	0.54	0.62	58.23	6.46
Q99KW9	T-cell immunomodulatory protein OS=Mus musculus GN=Itfg1 PE=2 SV=2 - [TIP_MOUSE]	2.30	1	1	1	1	1.07	0.69	0.65	0.78	0.73	0.96	0.89	0.54	0.50	0.96	0.90	1.32	1.23	67.42	5.73
O08807	Peroxiredoxin-4 OS=Mus musculus GN=Prdx4 PE=1 SV=1 - [PRDX4_MOUSE]	27.37	2	4	6	41	1.05	0.73	0.72	0.90	0.86	0.53	0.50	0.54	0.54	0.52	0.52	0.54	0.54	31.03	7.15
Q9R111	Guanine deaminase OS=Mus musculus GN=Gda PE=1 SV=1 - [GUAD_MOUSE]	57.05	2	21	22	201	1.02	0.97	0.91	0.89	0.85	0.56	0.53	0.55	0.53	0.54	0.52	0.62	0.55	50.98	5.53
Q99K85	Phosphoserine aminotransferase OS=Mus musculus GN=Psat1 PE=1 SV=1 - [SERC_MOUSE]	50.00	3	16	16	89	0.97	0.82	0.85	0.94	0.95	0.57	0.57	0.55	0.56	0.59	0.60	0.61	0.62	40.45	8.03
A3KMP2-	Isoform 2 of Tetratricopeptide repeat protein 38 OS=Mus musculus GN=Ttc38 - [TTC38_MOUSE]	10.39	2	2	2	6	0.99	1.14	1.15	0.81	0.82	0.60	0.60	0.55	0.55	0.62	0.63	0.71	0.72	31.57	6.29
P26043	Radixin OS=Mus musculus GN=Rdx PE=1 SV=3 - [RADI_MOUSE]	46.14	2	17	29	65	0.82	0.70	0.92	0.85	1.00	0.53	0.67	0.55	0.62	0.49	0.60	0.53	0.70	68.50	6.20
P42125	Enoyl-CoA delta isomerase 1, mitochondrial OS=Mus musculus GN=Eci1 PE=2 SV=2 - [ECI1_MOUSE]	31.83	1	8	8	34	1.12	0.90	0.75	0.96	0.84	0.56	0.43	0.55	0.44	0.56	0.47	0.58	0.45	32.23	8.98
Q8JZR2	Adapter molecule crk OS=Mus musculus GN=Crk PE=2 SV=1 - [Q8JZR2_MOUSE]	80.88	5	1	14	140	0.91	0.82	0.92	0.76	0.94	0.50	0.53	0.55	0.58	0.50	0.53	0.55	0.61	22.88	5.48
Q9CWQ0	Diphthine synthase OS=Mus musculus GN=Dph5 PE=2 SV=2 - [DPH5_MOUSE]	25.62	2	5	5	11	1.30	1.12	0.81	0.94	0.65	0.53	0.35	0.55	0.36	0.57	0.38	0.67	0.45	31.20	5.29
Q8CAY6	Acetyl-CoA acetyltransferase, cytosolic OS=Mus musculus GN=Acat2 PE=1 SV=2 - [THIC_MOUSE]	49.12	2	4	10	44	1.02	0.72	0.67	0.82	0.82	0.70	0.50	0.55	0.47	0.59	0.46	0.59	0.47	41.27	7.50
P01878	Ig alpha chain C region OS=Mus musculus PE=1 SV=1 - [IGHA_MOUSE]	6.69	1	2	2	3	1.21	0.68	0.48	0.68	0.56	0.50	0.36	0.55	0.43	0.72	0.51	0.49	0.38	36.85	5.06
Q9D0M5	Dynein light chain 2, cytoplasmic OS=Mus musculus GN=Dynll2 PE=1 SV=1 - [DYL2_MOUSE]	74.16	2	5	9	130	0.92	0.81	0.85	0.86	0.92	0.59	0.64	0.56	0.67	0.54	0.60	0.46	0.62	10.34	7.37

Q80X81	Acetyl-Coenzyme A acetyltransferase 3 OS=Mus musculus GN=Acat3 PE=2 SV=1 - [Q80X81_MOUSE]	37.28	2	2	9	29	1.19	1.02	0.85	0.81	0.69	0.51	0.58	0.56	0.49	0.49	0.50	0.56	0.46	41.44	7.94
Q69ZF7	Metal transporter CNNM4 OS=Mus musculus GN=Cnnm4 PE=1 SV=2 - [CNNM4_MOUSE]	1.69	1	1	1	1	0.92	0.89	0.97	0.72	0.78	0.84	0.90	0.56	0.60	0.72	0.78	0.72	0.79	86.57	6.07
P50247	Adenosylhomocysteinase OS=Mus musculus GN=Ahcy PE=1 SV=3 - [SAHH_MOUSE]	44.68	2	16	18	59	0.93	0.86	1.02	0.98	1.06	0.60	0.70	0.56	0.60	0.61	0.66	0.60	0.66	47.66	6.54
P63084	Protein S100-A5 OS=Mus musculus GN=S100a5 PE=2 SV=1 - [S10A5_MOUSE]	19.35	1	2	2	3	0.74	0.69	0.93	0.67	0.82	2.11	2.77	0.56	0.75	0.88	1.18	1.90	2.44	10.81	5.26
Q64010	Adapter molecule crk OS=Mus musculus GN=Crk PE=1 SV=1 - [CRK_MOUSE]	60.20	5	3	16	139	0.83	0.80	0.90	0.85	1.02	0.54	0.58	0.56	0.67	0.47	0.56	0.68	0.77	33.79	5.55
O88325	Alpha-N-acetylglucosaminidase OS=Mus musculus GN=Naglu PE=2 SV=1 - [O88325_MOUSE]	1.49	1	1	1	2	0.93	0.81	0.87	0.84	0.90	0.60	0.64	0.56	0.60	0.58	0.63	0.53	0.57	82.54	6.61
Q8BHS3	Pre-mRNA-splicing factor RBM22 OS=Mus musculus GN=Rbm22 PE=2 SV=1 - [RBM22_MOUSE]	11.43	1	4	4	12	0.85	0.78	0.81	0.84	0.99	0.56	0.62	0.56	0.62	0.60	0.70	0.51	0.59	46.87	8.54
O09159	Lysosomal alpha-mannosidase OS=Mus musculus GN=Man2b1 PE=2 SV=4 - [MA2B1_MOUSE]	3.26	1	3	3	4	0.94	0.98	1.05	0.81	0.86	0.67	0.71	0.56	0.61	0.61	0.66	0.65	0.69	114.58	8.13
O35459	Delta(3,5)-Delta(2,4)-dienoyl-CoA isomerase, mitochondrial OS=Mus musculus GN=Ech1 PE=2 SV=1 - [ECH1_MOUSE]	51.07	2	12	12	29	1.10	0.94	0.82	0.80	0.74	0.41	0.41	0.56	0.53	0.68	0.61	0.70	0.67	36.10	7.71
Q8CJG0	Protein argonaute-2 OS=Mus musculus GN=Ago2 PE=1 SV=3 - [AGO2_MOUSE]	17.67	2	8	12	24	0.90	1.03	1.09	0.94	1.07	0.60	0.65	0.57	0.62	0.56	0.69	0.62	0.74	97.24	9.19
Q9D614	Syntaxin 17, isoform CRA_b OS=Mus musculus GN=Stx17 PE=2 SV=1 - [Q9D614_MOUSE]	23.55	2	1	4	5	0.75	0.64	0.84	0.44	0.59	0.53	0.70	0.57	0.75	0.55	0.73	0.35	0.46	31.49	6.47
Q99KR3	Beta-lactamase-like protein 2 OS=Mus musculus GN=Lactb2 PE=1 SV=1 - [LACB2_MOUSE]	29.51	1	7	7	22	1.07	0.97	0.83	0.87	0.78	0.53	0.49	0.57	0.50	0.58	0.50	0.73	0.56	32.73	6.33

O55234	Proteasome subunit beta type-5 OS=Mus musculus GN=Psmb5 PE=1 SV=3 - [PSB5_MOUSE]	33.71	1	9	9	28	0.88	1.05	1.20	1.05	1.18	0.62	0.73	0.57	0.68	0.60	0.70	0.71	0.81	28.51	7.02
Q9CQQ7	ATP synthase subunit b, mitochondrial OS=Mus musculus GN=Atp5f1 PE=1 SV=1 - [AT5F1_MOUSE]	16.41	1	5	5	21	0.96	1.04	1.07	1.13	1.20	0.61	0.63	0.57	0.61	0.68	0.72	0.73	0.73	28.93	9.06
Q9Z2Y8	Proline synthase co-transcribed bacterial homolog protein OS=Mus musculus GN=Prosc PE=1 SV=1 - [PROSC_MOUSE]	35.04	1	4	8	35	0.90	0.86	0.95	0.85	0.94	0.57	0.63	0.57	0.67	0.54	0.60	0.63	0.67	30.03	8.27
P21956-2	Isoform 2 of Lactadherin OS=Mus musculus GN=Mfge8 - [MFGM_MOUSE]	2.35	2	1	1	4	1.16	0.88	0.76	1.06	0.92	0.55	0.47	0.57	0.49	0.57	0.49	0.64	0.56	47.14	7.05
Q00519	Xanthine dehydrogenase/oxidase OS=Mus musculus GN=Xdh PE=1 SV=5 - [XDH_MOUSE]	5.62	1	6	6	10	0.93	0.97	0.88	0.83	0.90	0.70	0.55	0.57	0.64	0.55	0.65	0.55	0.60	146.47	7.56
Q91VR7	Microtubule-associated proteins 1A/1B light chain 3A OS=Mus musculus GN=Map1lc3a PE=1 SV=1 - [MLP3A_MOUSE]	19.83	2	2	3	18	0.87	0.52	0.56	0.81	0.94	0.54	0.63	0.57	0.66	0.50	0.58	0.54	0.65	14.26	8.68
P62141	Serine/threonine-protein phosphatase PP1-beta catalytic subunit OS=Mus musculus GN=Ppp1cb PE=1 SV=3 - [PP1B_MOUSE]	71.87	1	7	20	127	0.98	0.91	0.87	0.89	0.91	0.52	0.54	0.57	0.60	0.56	0.54	0.59	0.64	37.16	6.19
P97470	Serine/threonine-protein phosphatase 4 catalytic subunit OS=Mus musculus GN=Ppp4c PE=1 SV=2 - [PP4C_MOUSE]	28.01	1	4	5	13	1.09	1.00	0.94	1.03	0.93	0.62	0.53	0.57	0.55	0.60	0.55	0.62	0.58	35.06	5.06
Q9D240	Pleckstrin homology domain-containing family J member 1 OS=Mus musculus GN=Plekhj1 PE=2 SV=1 - [PKHJ1_MOUSE]	9.15	1	1	1	1	1.06	0.80	0.75	0.80	0.75	0.53	0.50	0.57	0.54	0.55	0.52	0.55	0.52	19.02	7.03
Q8BWR2	PITH domain-containing protein 1 OS=Mus musculus GN=Pithd1 PE=2 SV=1 - [PITH1_MOUSE]	69.19	4	14	14	57	1.13	0.87	0.74	0.85	0.72	0.49	0.41	0.57	0.43	0.55	0.46	0.67	0.53	24.18	5.74
Q9DBJ1	Phosphoglycerate mutase 1 OS=Mus musculus GN=Pgam1 PE=1 SV=3 - [PGAM1_MOUSE]	86.22	1	16	21	479	0.93	0.83	0.90	0.86	0.94	0.58	0.63	0.57	0.62	0.58	0.63	0.56	0.62	28.81	7.18

P60487	Pyridoxal phosphate phosphatase OS=Mus musculus GN=Pdxp PE=1 SV=1 - [PLPP_MOUSE]	77.05	1	18	18	131	1.09	0.98	0.86	0.91	0.84	0.54	0.49	0.57	0.52	0.59	0.55	0.63	0.61	31.49	5.74
P03921	NADH-ubiquinone oxidoreductase chain 5 OS=Mus musculus GN=Mtnd5 PE=3 SV=2 - [NU5M_MOUSE]	2.14	2	1	1	2	1.13	1.28	1.13	1.29	1.14	0.49	0.43	0.57	0.51	0.74	0.66	0.90	0.80	68.39	9.03
F6XMH5	E3 ubiquitin-protein ligase TRIM37 (Fragment) OS=Mus musculus GN=Trim37 PE=4 SV=1 - [F6XMH5_MOUSE]	5.90	2	1	1	1	1.63	0.88	0.54	0.81	0.50	1.10	0.67	0.58	0.35	0.92	0.56	0.88	0.54	36.60	5.01
P83877	Thioredoxin-like protein 4A OS=Mus musculus GN=Txnl4a PE=2 SV=1 - [TXN4A_MOUSE]	33.10	2	4	4	8	1.14	0.73	0.63	0.86	0.75	0.76	0.61	0.58	0.45	0.61	0.51	0.75	0.68	16.78	5.85
P63087-2	Isoform Gamma-2 of Serine/threonine-protein phosphatase PP1-gamma catalytic subunit OS=Mus musculus GN=Ppp1cc - [PP1G_MOUSE]	74.48	2	6	21	118	0.97	0.94	0.98	0.90	0.96	0.49	0.56	0.58	0.63	0.54	0.61	0.64	0.68	38.48	6.13
Q99LL5	Periodic tryptophan protein 1 homolog OS=Mus musculus GN=Pwp1 PE=1 SV=1 - [PWP1_MOUSE]	4.59	1	1	1	2	1.10	0.96	0.87	0.91	0.83	0.52	0.47	0.58	0.53	0.44	0.40	0.53	0.48	55.55	4.82
Q80TB8	Synaptic vesicle membrane protein VAT-1 homolog-like OS=Mus musculus GN=Vat1l PE=2 SV=2 - [VAT1L_MOUSE]	47.24	2	17	17	43	1.35	1.12	0.87	0.99	0.77	0.71	0.53	0.58	0.41	0.81	0.61	0.91	0.68	45.79	5.06
Q8BFQ8	Parkinson disease 7 domain-containing protein 1 OS=Mus musculus GN=Pddc1 PE=1 SV=1 - [PDDC1_MOUSE]	50.45	1	7	7	14	1.12	0.98	1.02	1.08	1.00	0.62	0.58	0.59	0.59	0.63	0.58	0.64	0.71	23.26	7.05
Q9DAR7	m7GpppX diphosphatase OS=Mus musculus GN=Dcps PE=1 SV=1 - [DCPS_MOUSE]	31.07	3	7	7	13	0.89	0.90	1.03	0.81	1.07	0.62	0.70	0.59	0.69	0.55	0.70	0.53	0.62	38.96	6.48
Q68EF9	Scube1 protein OS=Mus musculus GN=Scube1 PE=2 SV=1 - [Q68EF9_MOUSE]	1.32	5	1	1	1	0.59	0.94	1.59	0.93	1.57	1.07	1.81	0.59	1.00	0.74	1.26	0.87	1.49	99.40	6.83
Q61474-2	Isoform 2 of RNA-binding protein Musashi homolog 1 OS=Mus musculus GN=Msi1 - [MSI1H_MOUSE]	4.98	3	1	1	1	1.16	0.88	0.75	0.86	0.74	0.51	0.43	0.59	0.51	0.69	0.59	0.71	0.62	34.01	9.66

Q922B6	E3 ubiquitin-protein ligase TRAF7 OS=Mus musculus GN=Traf7 PE=1 SV=1 - [TRAF7_MOUSE]	2.53	3	1	1	2	1.00	1.20	1.20	1.07	1.07	0.87	0.87	0.59	0.59	1.12	1.13	1.08	1.09	66.44	6.99
Q9CSP9-4	Isoform 4 of Tetratricopeptide repeat protein 14 OS=Mus musculus GN=Ttc14 - [TTC14_MOUSE]	1.55	4	1	1	1	0.85	0.95	1.11	0.68	0.80	0.46	0.53	0.59	0.69	0.43	0.50	0.59	0.69	80.94	9.13
Q9JHE3	Neutral ceramidase OS=Mus musculus GN=Asah2 PE=1 SV=1 - [ASAH2_MOUSE]	3.31	1	2	2	3	0.88	1.04	1.18	0.94	1.07	0.77	0.87	0.60	0.67	0.70	0.80	0.62	0.71	83.46	6.86
F6QKK2	ADP-ribosylation factor-like protein 8A (Fragment) OS=Mus musculus GN=Arl8a PE=4 SV=1 - [F6QKK2_MOUSE]	47.88	2	3	7	23	1.15	0.93	0.80	1.12	0.98	0.68	0.72	0.60	0.63	0.64	0.66	0.69	0.78	18.85	8.16
Q91V61-2	Isoform 2 of Sideroflexin-3 OS=Mus musculus GN=Sfxn3 - [SFXN3_MOUSE]	46.53	3	8	10	38	0.92	1.36	1.32	1.30	1.43	0.60	0.67	0.60	0.63	0.77	0.84	0.76	0.84	31.64	9.45
Q6WVG3	BTB/POZ domain-containing protein KCTD12 OS=Mus musculus GN=Kctd12 PE=1 SV=1 - [KCD12_MOUSE]	37.92	1	7	8	26	1.09	0.98	0.98	0.90	0.84	0.57	0.56	0.60	0.51	0.60	0.58	0.64	0.61	35.87	5.81
Q99PI8	Reticulon-4 receptor OS=Mus musculus GN=Rtn4r PE=2 SV=1 - [RTN4R_MOUSE]	19.03	1	5	5	7	0.88	1.03	1.10	0.94	1.07	0.67	0.74	0.60	0.64	0.67	0.72	0.72	0.81	50.96	8.69
Q60994	Adiponectin OS=Mus musculus GN=Adipoq PE=1 SV=2 - [ADIPO_MOUSE]	8.91	2	2	2	4	1.15	1.18	1.03	0.97	0.84	0.63	0.54	0.60	0.52	0.73	0.64	0.69	0.61	26.79	5.57
Q9WUI0	Homeobox protein MIXL1 OS=Mus musculus GN=Mixl1 PE=1 SV=1 - [MIXL1_MOUSE]	8.23	1	1	1	1	1.76	2.38	1.35	0.88	0.50	0.54	0.30	0.60	0.34	0.95	0.54	0.86	0.49	24.83	8.81
Q9JME7	Trafficking protein particle complex subunit 2-like protein OS=Mus musculus GN=Trappc2l PE=2 SV=1 - [TPC2L_MOUSE]	10.07	1	1	1	2	1.01	0.96	0.94	1.22	1.20	0.90	0.88	0.60	0.59	1.02	1.01	1.12	1.10	16.01	6.77
Q99LI2	Chloride channel CLIC-like protein 1 OS=Mus musculus GN=Clcc1 PE=1 SV=1 - [CLCC1_MOUSE]	6.49	2	3	3	5	1.01	1.36	1.35	1.02	1.02	0.84	0.83	0.60	0.73	0.88	0.87	1.04	0.94	60.58	5.68
Q8BIY1	G patch domain-containing protein 3 OS=Mus musculus GN=Gpatch3 PE=2 SV=1 - [GPTC3_MOUSE]	2.29	1	1	1	1	0.51	5.25	10.18	0.50	0.96	0.57	1.10	0.60	1.16	0.58	1.14	0.53	1.03	59.14	5.05

Q5SWT3	Solute carrier family 25 member 35 OS=Mus musculus GN=Slc25a35 PE=2 SV=2 - [S2535_MOUSE]	2.67	1	1	1	2	0.99	1.36	1.36	1.13	1.14	0.61	0.61	0.60	0.60	0.71	0.72	0.89	0.90	32.61	9.52
Q9DB50	AP-1 complex subunit sigma-2 OS=Mus musculus GN=Ap1s2 PE=2 SV=1 - [AP1S2_MOUSE]	13.13	4	1	2	3	0.98	1.21	1.24	1.14	1.17	0.71	0.72	0.60	0.61	0.74	0.76	0.72	0.74	18.92	5.48
Q9CR62	Mitochondrial 2-oxoglutarate/malate carrier protein OS=Mus musculus GN=Slc25a11 PE=1 SV=3 - [M2OM_MOUSE]	39.17	3	10	10	23	1.12	1.32	1.08	1.29	1.15	0.60	0.56	0.60	0.51	0.77	0.66	0.90	0.76	34.13	9.94
Q8CJG1	Protein argonaute-1 OS=Mus musculus GN=Ago1 PE=1 SV=2 - [AGO1_MOUSE]	12.02	4	4	8	18	1.03	1.10	1.05	0.90	0.92	0.51	0.47	0.60	0.55	0.55	0.52	0.75	0.74	97.15	9.16
P70677	Caspase-3 OS=Mus musculus GN=Casp3 PE=1 SV=1 - [CASP3_MOUSE]	23.47	1	5	5	16	1.21	0.86	0.72	0.98	0.85	0.66	0.53	0.60	0.50	0.67	0.59	0.72	0.64	31.45	6.92
Q91YN0	Uncharacterized protein C12orf4 homolog OS=Mus musculus GN=D6Wsu163e PE=1 SV=1 - [CL004_MOUSE]	4.35	1	1	1	2	0.89	0.72	0.81	0.74	0.83	0.75	0.84	0.60	0.67	0.66	0.74	0.74	0.83	63.60	6.18
P49722	Proteasome subunit alpha type-2 OS=Mus musculus GN=Psma2 PE=1 SV=3 - [PSA2_MOUSE]	46.58	1	9	9	34	0.88	0.98	1.13	1.04	1.21	0.69	0.77	0.60	0.77	0.69	0.76	0.68	0.81	25.91	7.43
P00405	Cytochrome c oxidase subunit 2 OS=Mus musculus GN=Mtco2 PE=1 SV=1 - [COX2_MOUSE]	13.22	1	2	2	7	0.94	0.89	1.17	1.12	1.22	0.59	0.69	0.61	0.67	0.73	0.79	0.73	0.80	25.96	4.73
P85094	Isochorismatase domain-containing protein 2A, mitochondrial OS=Mus musculus GN=Isoc2a PE=2 SV=1 - [ISC2A_MOUSE]	38.35	1	4	4	16	1.02	0.98	0.98	1.02	1.01	0.68	0.72	0.61	0.61	0.74	0.73	0.83	0.82	22.40	8.02
O70435	Proteasome subunit alpha type-3 OS=Mus musculus GN=Psma3 PE=1 SV=3 - [PSA3_MOUSE]	24.31	5	6	6	17	0.86	0.92	1.01	1.00	1.13	0.61	0.75	0.61	0.73	0.60	0.67	0.75	0.83	28.39	5.44
O09131	Glutathione S-transferase omega-1 OS=Mus musculus GN=Gsto1 PE=2 SV=2 - [GSTO1_MOUSE]	23.75	3	6	6	13	0.92	0.78	0.81	0.89	0.93	0.66	0.70	0.61	0.67	0.64	0.71	0.66	0.72	27.48	7.36
Q9CZP5	Mitochondrial chaperone BCS1 OS=Mus musculus GN=Bcs1l PE=1 SV=1 - [BCS1_MOUSE]	8.13	1	3	3	4	0.89	0.98	1.17	1.11	1.25	0.62	0.69	0.61	0.68	0.86	0.97	0.80	1.04	47.38	7.93

Q61327	Sodium-dependent dopamine transporter OS=Mus musculus GN=Slc6a3 PE=1 SV=2 - [SC6A3_MOUSE]	1.29	1	1	1	1	0.88	0.83	0.95	1.37	1.56	1.00	1.14	0.61	0.69	1.26	1.44	1.89	2.17	68.76	7.21
P62881-2	Isoform 2 of Guanine nucleotide-binding protein subunit beta-5 OS=Mus musculus GN=Gnb5 - [GBB5_MOUSE]	36.83	2	12	12	42	0.91	0.89	0.96	0.96	1.02	0.70	0.74	0.61	0.67	0.63	0.70	0.63	0.71	38.71	6.06
Q99PT1	Rho GDP-dissociation inhibitor 1 OS=Mus musculus GN=Arhgdia PE=1 SV=3 - [GDIR1_MOUSE]	67.16	1	15	15	114	1.06	0.90	0.84	1.00	0.96	0.63	0.59	0.61	0.59	0.60	0.58	0.61	0.57	23.39	5.20
Q8C0J6	Ankyrin repeat domain-containing protein SOWAHC OS=Mus musculus GN=Sowahc PE=2 SV=2 - [SWAHC_MOUSE]	3.32	1	1	1	2	0.80	1.03	1.28	0.71	0.89	1.05	1.30	0.61	0.76	1.07	1.33	0.70	0.88	54.90	6.00
O88653	Ragulator complex protein LAMTOR3 OS=Mus musculus GN=Lamtor3 PE=1 SV=1 - [LTOR3_MOUSE]	8.87	1	1	1	2	1.18	0.54	0.46	0.81	0.69	0.64	0.54	0.61	0.52	0.50	0.43	0.52	0.44	13.54	7.34
Q9CQM9	Glutaredoxin-3 OS=Mus musculus GN=Glrx3 PE=1 SV=1 - [GLRX3_MOUSE]	45.40	1	13	13	89	1.02	0.77	0.76	0.87	0.85	0.56	0.55	0.61	0.60	0.57	0.60	0.60	0.63	37.75	5.59
Q9QUP5	Hyaluronan and proteoglycan link protein 1 OS=Mus musculus GN=Hapln1 PE=2 SV=1 - [HPLN1_MOUSE]	57.02	1	16	17	80	0.94	0.85	0.93	0.90	0.97	0.62	0.70	0.61	0.67	0.67	0.73	0.72	0.73	40.45	7.80
Q8R3W2	MCG6979, isoform CRA_a OS=Mus musculus GN=0610009B22Rik PE=2 SV=1 - [Q8R3W2_MOUSE]	18.57	2	2	2	4	0.78	0.98	1.25	0.80	1.03	0.62	0.79	0.61	0.79	0.56	0.72	0.69	0.89	16.43	6.61
Q80V42	Carboxypeptidase M OS=Mus musculus GN=Cpm PE=2 SV=2 - [CBPM_MOUSE]	23.25	2	6	6	12	0.80	0.90	1.07	0.91	1.06	0.71	0.87	0.61	0.86	0.72	0.86	0.66	0.83	50.52	7.78
Q8CA71	Protein shisa-4 OS=Mus musculus GN=Shisa4 PE=2 SV=1 - [SHSA4_MOUSE]	4.57	1	1	1	2	1.05	1.16	1.10	1.21	1.16	0.85	0.81	0.62	0.59	0.87	0.84	1.03	0.98	21.47	7.42
Q9R1E6	Ectonucleotide pyrophosphatase/phosphodiesterase family member 2 OS=Mus musculus GN=Enpp2 PE=1 SV=3 - [ENPP2_MOUSE]	2.44	4	2	2	3	0.95	0.85	0.89	1.08	1.13	0.85	0.88	0.62	0.64	0.66	0.70	0.55	0.58	98.82	7.27

Q8CCH2	NHL repeat-containing protein 3 OS=Mus musculus GN=Nhlrc3 PE=2 SV=1 - [NHLC3_MOUSE]	9.80	1	2	2	3	1.05	0.94	0.89	0.88	0.83	0.53	0.51	0.62	0.58	0.89	0.85	0.80	0.76	38.17	6.23
P07724	Serum albumin OS=Mus musculus GN=Alb PE=1 SV=3 - [ALBU_MOUSE]	74.51	1	41	41	409	0.94	0.81	0.86	0.58	0.60	0.53	0.56	0.62	0.65	0.67	0.71	0.52	0.56	68.65	6.07
Q91YA2	Serine/threonine-protein kinase H1 OS=Mus musculus GN=Pskh1 PE=2 SV=3 - [KPSH1_MOUSE]	4.01	1	1	1	3	1.00	0.57	0.57	0.89	0.89	0.56	0.56	0.62	0.62	0.55	0.55	0.49	0.49	48.07	9.83
Q8R238	Serine dehydratase-like OS=Mus musculus GN=Sdsl PE=2 SV=1 - [SDSL_MOUSE]	7.90	2	2	2	4	0.74	0.75	1.01	0.67	0.91	0.56	0.76	0.62	0.84	0.60	0.82	0.59	0.81	34.71	6.43
Q9ER35	Fructosamine-3-kinase OS=Mus musculus GN=Fn3k PE=2 SV=1 - [FN3K_MOUSE]	18.77	3	5	5	9	0.98	1.08	1.05	0.95	0.94	0.58	0.69	0.62	0.66	0.56	0.64	0.61	0.68	35.01	8.40
Q9D404	3-oxoacyl-[acyl-carrier-protein] synthase, mitochondrial OS=Mus musculus GN=Oxsm PE=2 SV=1 - [OXSM_MOUSE]	19.39	2	6	6	14	1.00	0.91	0.90	0.84	0.85	0.66	0.63	0.62	0.62	0.71	0.64	0.77	0.92	48.60	7.06
Q8BR65	Sin3 histone deacetylase corepressor complex component SDS3 OS=Mus musculus GN=Suds3 PE=1 SV=1 - [SDS3_MOUSE]	5.49	1	1	1	2	0.89	0.77	0.86	0.85	0.96	0.73	0.82	0.62	0.70	0.69	0.78	0.71	0.80	38.08	5.66
P51881	ADP/ATP translocase 2 OS=Mus musculus GN=Slc25a5 PE=1 SV=3 - [ADT2_MOUSE]	54.36	2	9	17	150	1.09	1.13	1.04	1.34	1.26	0.69	0.60	0.62	0.55	0.79	0.68	0.84	0.79	32.91	9.73
D3YXJ2	Protein Gm9920 OS=Mus musculus GN=Gm9920 PE=2 SV=1 - [D3YXJ2_MOUSE]	34.13	1	1	1	1	0.90	0.79	0.88	0.99	1.10	0.42	0.47	0.62	0.69	0.38	0.42	0.45	0.50	13.44	7.56
Q60692	Proteasome subunit beta type-6 OS=Mus musculus GN=Psmb6 PE=1 SV=3 - [PSB6_MOUSE]	38.24	1	6	6	21	0.81	1.22	1.43	0.94	1.15	0.66	0.82	0.62	0.82	0.62	0.84	0.73	0.90	25.36	5.11
Q8BGU2	Cerebellin-2 OS=Mus musculus GN=Cbln2 PE=1 SV=1 - [CBLN2_MOUSE]	14.73	1	2	3	7	1.05	0.88	0.83	0.85	0.83	0.64	0.60	0.62	0.59	0.54	0.51	0.56	0.57	24.04	8.18
Q8BUR4	Dedicator of cytokinesis protein 1 OS=Mus musculus GN=Dock1 PE=1 SV=3 - [DOCK1_MOUSE]	1.13	1	1	1	4	0.98	0.65	0.67	0.81	0.83	0.47	0.48	0.62	0.64	0.64	0.65	0.51	0.52	214.95	7.62
P03899	NADH-ubiquinone oxidoreductase chain 3 OS=Mus musculus GN=Mtnd3 PE=3 SV=3 - [NU3M_MOUSE]	13.04	1	1	1	4	1.11	1.37	1.24	1.48	1.34	0.60	0.54	0.62	0.56	0.77	0.69	1.11	1.01	13.21	4.64

Q8R1G2	Carboxymethylenebutenolidase homolog OS=Mus musculus GN=Cmbl PE=2 SV=1 - [CMBL_MOUSE]	15.51	3	3	3	6	1.28	0.87	0.68	0.89	0.72	0.60	0.48	0.62	0.51	0.66	0.54	0.72	0.57	27.88	7.18
O88968	Transcobalamin-2 OS=Mus musculus GN=Tcn2 PE=2 SV=1 - [TCO2_MOUSE]	5.58	1	1	1	2	1.00	0.88	0.88	0.91	0.91	0.62	0.62	0.63	0.62	0.58	0.58	0.57	0.57	47.56	6.33
P27545	Ceramide synthase 1 OS=Mus musculus GN=Cers1 PE=1 SV=1 - [CERS1_MOUSE]	4.57	1	1	1	1	1.15	2.59	2.24	1.37	1.19	0.85	0.73	0.63	0.54	0.98	0.85	1.13	0.98	40.07	8.38
Q8BGF3	WD repeat-containing protein 92 OS=Mus musculus GN=Wdr92 PE=2 SV=1 - [WDR92_MOUSE]	5.60	1	2	2	2	0.94	1.00	1.06	0.79	0.83	0.53	0.56	0.63	0.66	0.58	0.62	0.70	0.75	39.77	8.15
Q61599	Rho GDP-dissociation inhibitor 2 OS=Mus musculus GN=Arhgdib PE=1 SV=3 - [GDIR2_MOUSE]	38.50	2	4	4	28	0.85	0.74	0.85	0.97	1.13	0.67	0.73	0.63	0.70	0.69	0.77	0.75	0.86	22.84	5.11
P35290	Ras-related protein Rab-24 OS=Mus musculus GN=Rab24 PE=1 SV=2 - [RAB24_MOUSE]	19.21	1	4	4	11	0.93	0.85	1.00	1.00	1.00	0.66	0.69	0.63	0.62	0.65	0.71	0.84	0.84	23.13	6.23
P61971	Nuclear transport factor 2 OS=Mus musculus GN=Nutf2 PE=2 SV=1 - [NTF2_MOUSE]	44.88	1	3	3	12	0.95	0.92	0.97	1.00	0.98	0.62	0.70	0.63	0.67	0.72	0.74	0.67	0.72	14.47	5.38
G5E8Q5	DCN1-like protein 2 OS=Mus musculus GN=Dcun1d2 PE=4 SV=1 - [G5E8Q5_MOUSE]	17.77	5	1	3	5	1.79	1.20	0.67	1.12	0.62	0.90	0.50	0.63	0.35	0.54	0.30	0.99	0.56	22.75	7.97
Q8CII5	Nucleolysin TIA-1 OS=Mus musculus GN=Tia1 PE=2 SV=1 - [Q8CII5_MOUSE]	14.39	7	1	4	5	0.82	0.97	1.17	0.89	1.08	0.67	0.81	0.63	0.76	0.72	0.88	0.71	0.87	31.56	8.29
Q8BGT1	Fibronectin leucine rich transmembrane protein 3 OS=Mus musculus GN=Flrt3 PE=2 SV=1 - [Q8BGT1_MOUSE]	7.70	1	3	4	8	1.09	0.78	0.74	1.01	0.94	0.74	0.67	0.63	0.60	0.61	0.57	0.68	0.65	72.83	8.06
P00015	Cytochrome c, testis-specific OS=Mus musculus GN=Cyct PE=1 SV=3 - [CYC2_MOUSE]	25.71	1	1	3	21	0.82	0.49	0.59	0.58	0.70	0.56	0.68	0.63	0.76	0.52	0.63	0.53	0.64	11.71	9.51
Q3U595	UPF0545 protein C22orf39 homolog OS=Mus musculus PE=2 SV=1 - [CV039_MOUSE]	7.62	1	1	1	1	0.70	0.92	1.30	0.88	1.25	0.68	0.97	0.63	0.89	0.75	1.06	0.84	1.20	12.50	8.12

Q9QWW1	Isoform 2 of Homer protein homolog 2 OS=Mus musculus GN=Homer2 - [HOME2_MOUSE]	39.36	4	14	15	33	1.13	0.86	0.78	0.93	0.84	0.61	0.53	0.63	0.57	0.53	0.44	0.60	0.53	39.44	6.38
O08966	Solute carrier family 22 member 1 OS=Mus musculus GN=Slc22a1 PE=2 SV=2 - [S22A1_MOUSE]	2.88	1	1	1	1	1.29	1.05	0.81	0.73	0.57	0.52	0.40	0.63	0.49	0.53	0.41	0.68	0.53	61.48	6.67
P48962	ADP/ATP translocase 1 OS=Mus musculus GN=Slc25a4 PE=1 SV=4 - [ADT1_MOUSE]	67.45	2	12	20	170	1.01	1.13	1.17	1.34	1.31	0.67	0.66	0.63	0.63	0.78	0.79	0.79	0.85	32.88	9.72
H3BK32	Sentrin-specific protease 6 (Fragment) OS=Mus musculus GN=Senp6 PE=2 SV=1 - [H3BK32_MOUSE]	1.31	4	1	1	1	5.24	7.13	1.36	0.76	0.15	0.59	0.11	0.63	0.12	0.57	0.11	0.68	0.13	103.64	6.37
Q8VEM8	Phosphate carrier protein, mitochondrial OS=Mus musculus GN=Slc25a3 PE=1 SV=1 - [MPCP_MOUSE]	27.17	2	10	10	60	0.99	1.25	1.10	1.28	1.23	0.67	0.65	0.63	0.64	0.78	0.80	0.88	0.82	39.61	9.26
Q8K0S5	Reticulon-4 receptor-like 1 OS=Mus musculus GN=Rtn4rl1 PE=2 SV=1 - [R4RL1_MOUSE]	6.29	1	2	2	4	0.96	0.92	0.96	0.94	0.98	0.68	0.71	0.63	0.66	0.69	0.73	0.76	0.79	49.80	8.65
Q9R1P1	Proteasome subunit beta type-3 OS=Mus musculus GN=Psmb3 PE=1 SV=1 - [PSB3_MOUSE]	26.83	3	4	4	14	0.96	1.15	1.12	1.18	1.23	0.65	0.66	0.63	0.71	0.72	0.71	0.73	0.78	22.95	6.55
Q9Z2Z6	Mitochondrial carnitine/acylcarnitine carrier protein OS=Mus musculus GN=Slc25a20 PE=1 SV=1 - [MCAT_MOUSE]	4.32	1	1	1	2	0.61	0.95	1.55	1.12	1.84	0.68	1.11	0.63	1.03	0.95	1.56	0.72	1.18	33.00	9.11
Q9QUM9	Proteasome subunit alpha type-6 OS=Mus musculus GN=Psma6 PE=1 SV=1 - [PSA6_MOUSE]	45.12	3	12	12	92	0.78	1.11	1.40	0.97	1.24	0.64	0.78	0.63	0.78	0.64	0.82	0.64	0.84	27.35	6.76
E0CY96	tRNA-splicing endonuclease subunit Sen15 OS=Mus musculus GN=Tsen15 PE=2 SV=1 - [E0CY96_MOUSE]	29.69	3	2	2	3	0.83	0.93	1.12	0.79	0.96	0.62	0.74	0.63	0.77	0.73	0.88	0.66	0.80	14.01	4.41
Q8BG73	SH3 domain-binding glutamic acid-rich-like protein 2 OS=Mus musculus GN=Sh3bgrl2 PE=1 SV=1 - [SH3L2_MOUSE]	46.73	1	5	5	55	0.89	0.69	0.83	0.80	0.95	0.62	0.71	0.64	0.71	0.55	0.63	0.59	0.69	12.25	5.55
P63300	Selenoprotein W OS=Mus musculus GN=Sepw1 PE=1 SV=3 - [SELW_MOUSE]	14.77	1	1	1	2	1.00	0.53	0.53	0.90	0.91	0.67	0.67	0.64	0.64	0.60	0.60	0.63	0.64	9.68	8.72

E9QLX9	Myb-related protein A OS=Mus musculus GN=Mybl1 PE=2 SV=1 - [E9QLX9_MOUSE]	2.89	3	1	1	1	0.80	0.63	0.79	0.83	1.04	0.75	0.93	0.64	0.80	0.70	0.87	0.75	0.94	78.68	6.55
G3UZW8	Proteasome subunit beta type (Fragment) OS=Mus musculus GN=Psmb8 PE=2 SV=2 - [G3UZW8_MOUSE]	10.89	2	2	2	3	1.03	1.26	1.23	1.01	0.98	0.71	0.69	0.64	0.62	0.68	0.66	0.62	0.60	26.98	7.75
P60521	Gamma-aminobutyric acid receptor-associated protein-like 2 OS=Mus musculus GN=Gabarapl2 PE=1 SV=1 - [GBRL2_MOUSE]	60.68	1	7	8	33	0.90	0.74	0.81	0.78	0.87	0.57	0.67	0.64	0.63	0.58	0.59	0.61	0.69	13.66	8.10
P97434-3	Isoform 3 of Myosin phosphatase Rho-interacting protein OS=Mus musculus GN=Mprip - [MPRIP_MOUSE]	30.97	8	1	23	49	0.68	1.22	1.78	0.55	0.81	0.61	0.88	0.64	0.93	0.55	0.81	0.53	0.78	114.02	6.37
P70195	Proteasome subunit beta type-7 OS=Mus musculus GN=Psmb7 PE=1 SV=1 - [PSB7_MOUSE]	27.08	1	6	6	15	0.82	0.98	1.23	0.99	1.18	0.79	0.96	0.64	0.87	0.64	0.81	0.66	0.83	29.87	7.99
P09671	Superoxide dismutase [Mn], mitochondrial OS=Mus musculus GN=Sod2 PE=1 SV=3 - [SODM_MOUSE]	77.93	1	14	14	227	0.99	0.80	0.81	0.92	0.93	0.63	0.65	0.64	0.64	0.63	0.64	0.63	0.63	24.59	8.62
Q9D9V3-2	Isoform 2 of Ethylmalonyl-CoA decarboxylase OS=Mus musculus GN=Echdc1 - [ECHD1_MOUSE]	22.74	3	5	5	11	1.01	1.12	0.86	1.00	0.95	0.70	0.65	0.64	0.61	0.68	0.68	0.50	0.53	32.71	7.02
D6RIN1	Protein unc-45 homolog A OS=Mus musculus GN=Unc45a PE=2 SV=1 - [D6RIN1_MOUSE]	3.52	2	1	1	2	1.06	1.04	0.98	0.63	0.60	0.81	0.76	0.64	0.60	1.10	1.04	0.64	0.61	68.47	7.99
O88485	Cytoplasmic dynein 1 intermediate chain 1 OS=Mus musculus GN=Dync1i1 PE=1 SV=2 - [DC1I1_MOUSE]	47.61	5	18	19	196	0.98	1.02	1.05	0.91	0.97	0.65	0.67	0.64	0.71	0.65	0.68	0.63	0.65	70.68	5.12
Q9JMA2	Queuine tRNA-ribosyltransferase OS=Mus musculus GN=Qtrt1 PE=1 SV=2 - [TGT_MOUSE]	3.47	1	1	1	2	1.08	1.59	1.48	1.07	0.99	0.72	0.66	0.64	0.60	0.78	0.72	0.91	0.85	44.06	7.68
Q61171	Peroxiredoxin-2 OS=Mus musculus GN=Prdx2 PE=1 SV=3 - [PRDX2_MOUSE]	73.23	2	12	12	164	1.04	0.80	0.77	0.97	0.93	0.65	0.63	0.64	0.62	0.63	0.62	0.63	0.60	21.77	5.41

Q9D8Z6	Autophagy-related protein 101 OS=Mus musculus GN=Atg101 PE=2 SV=1 - [ATGA1_MOUSE]	7.34	1	1	1	3	1.03	1.32	1.29	0.91	0.89	0.60	0.58	0.64	0.63	0.70	0.69	0.64	0.62	24.99	6.15
P01756	Ig heavy chain V region MOPC 104E OS=Mus musculus PE=1 SV=1 - [HVM12_MOUSE]	16.24	3	1	1	2	1.12	0.71	0.63	0.98	0.88	1.25	1.11	0.65	0.57	0.79	0.70	0.50	0.45	12.98	7.11
E9Q1V6	Asc-type amino acid transporter 1 OS=Mus musculus GN=Slc7a10 PE=2 SV=1 - [E9Q1V6_MOUSE]	46.75	4	1	1	2	0.81	1.17	1.45	0.77	0.95	0.67	0.83	0.65	0.80	0.69	0.85	0.66	0.82	7.72	7.24
Q71RI9-2	Isoform 2 of Kynurenine--oxoglutarate transaminase 3 OS=Mus musculus GN=Ccbl2 - [KAT3_MOUSE]	2.86	2	1	1	2	0.97	1.04	1.06	0.90	0.93	0.60	0.61	0.65	0.66	0.64	0.65	0.70	0.72	47.29	7.20
Q64378	Peptidyl-prolyl cis-trans isomerase FKBP5 OS=Mus musculus GN=Fkbp5 PE=1 SV=1 - [FKBP5_MOUSE]	4.82	2	2	2	2	0.91	0.77	0.85	0.73	0.80	0.69	0.75	0.65	0.71	0.63	0.70	0.66	0.73	50.93	7.80
G3XA11	Cornichon homolog 3 (Drosophila), isoform CRA_a OS=Mus musculus GN=Cnih3 PE=4 SV=1 - [G3XA11_MOUSE]	10.71	3	1	1	2	0.90	0.98	1.08	0.86	0.95	0.72	0.79	0.65	0.72	0.94	1.04	0.86	0.95	13.22	7.31
Q6P2B2	Casein kinase I isoform gamma-1 OS=Mus musculus GN=Csnk1g1 PE=2 SV=1 - [Q6P2B2_MOUSE]	4.65	2	1	3	3	0.84	0.84	1.01	0.97	1.16	0.54	0.64	0.65	0.77	0.70	0.83	0.70	0.84	49.57	8.97
Q8K215	LYR motif-containing protein 4 OS=Mus musculus GN=Lyrm4 PE=2 SV=1 - [LYRM4_MOUSE]	10.99	1	1	1	1	0.99	0.65	0.66	0.93	0.94	0.71	0.71	0.65	0.66	0.64	0.65	0.73	0.74	10.85	10.13
Q8C0L8	Conserved oligomeric Golgi complex subunit 5 OS=Mus musculus GN=Cog5 PE=2 SV=3 - [COG5_MOUSE]	1.93	1	1	1	1	0.98	0.77	0.78	0.80	0.82	0.56	0.57	0.65	0.66	0.56	0.57	0.67	0.68	91.33	6.27
J3QMM7	ATP-dependent (S)-NAD(P)H-hydrate dehydratase OS=Mus musculus GN=Carkd PE=3 SV=1 - [J3QMM7_MOUSE]	51.68	7	11	11	45	0.96	1.07	1.12	0.96	0.96	0.73	0.80	0.65	0.71	0.71	0.81	0.75	0.82	35.15	8.13
Q9D0I6	WD repeat, SAM and U-box domain-containing protein 1 OS=Mus musculus GN=Wdsub1 PE=2 SV=1 - [WSDU1_MOUSE]	7.38	1	2	2	4	0.90	0.80	0.88	0.88	0.97	0.56	0.61	0.65	0.72	0.68	0.75	0.71	0.78	51.66	6.34

D3Z1E2	Pleckstrin homology domain-containing family B member 1 (Fragment) OS=Mus musculus GN=Plekhb1 PE=2 SV=1 - [D3Z1E2_MOUSE]	27.05	8	3	3	9	1.04	1.21	1.15	0.87	0.82	0.61	0.58	0.66	0.59	0.66	0.61	0.71	0.68	14.35	6.32
P59235	Nucleoporin Nup43 OS=Mus musculus GN=Nup43 PE=2 SV=2 - [NUP43_MOUSE]	4.21	1	1	1	2	0.80	0.88	1.10	0.88	1.09	0.56	0.69	0.66	0.81	0.65	0.80	0.71	0.89	41.96	5.39
Q99LH2	Phosphatidylserine synthase 1 OS=Mus musculus GN=Ptdss1 PE=2 SV=1 - [PTSS1_MOUSE]	2.96	1	1	1	2	1.14	1.13	0.99	1.32	1.16	0.82	0.71	0.66	0.57	0.89	0.78	0.89	0.78	55.57	8.28
P11352	Glutathione peroxidase 1 OS=Mus musculus GN=Gpx1 PE=1 SV=2 - [GPX1_MOUSE]	51.74	1	8	8	26	0.93	0.87	0.90	0.94	1.04	0.64	0.69	0.66	0.69	0.64	0.70	0.64	0.76	22.32	7.21
D3YXP6	Phosphomevalonate kinase OS=Mus musculus GN=Pmvk PE=2 SV=1 - [D3YXP6_MOUSE]	5.06	2	1	1	1	0.51	0.58	1.12	0.73	1.41	0.63	1.22	0.66	1.27	0.66	1.28	0.64	1.24	17.93	9.86
O09061	Proteasome subunit beta type-1 OS=Mus musculus GN=Psmb1 PE=1 SV=1 - [PSB1_MOUSE]	51.67	1	10	10	52	0.83	1.18	1.38	0.98	1.21	0.68	0.83	0.66	0.77	0.65	0.79	0.65	0.78	26.36	7.81
P01863	Ig gamma-2A chain C region, A allele OS=Mus musculus GN=Ighg PE=1 SV=1 - [GCAA_MOUSE]	4.85	4	1	1	2	1.17	1.12	0.95	0.87	0.75	0.53	0.45	0.66	0.56	0.93	0.80	0.91	0.78	36.37	7.40
Q9CRB8	Mitochondrial fission process protein 1 OS=Mus musculus GN=Mtfp1 PE=1 SV=1 - [MTFP1_MOUSE]	27.11	1	3	3	5	0.96	1.11	1.16	1.19	1.28	0.71	0.74	0.66	0.67	0.75	0.71	1.07	1.12	18.30	8.68
Q8VC57	BTB/POZ domain-containing protein KCTD5 OS=Mus musculus GN=Kctd5 PE=2 SV=1 - [KCTD5_MOUSE]	5.13	1	1	1	2	1.08	0.97	0.90	0.95	0.88	0.62	0.57	0.66	0.61	0.69	0.64	0.79	0.74	26.15	5.95
Q07235	Glia-derived nexin OS=Mus musculus GN=Serpine2 PE=2 SV=2 - [GDN_MOUSE]	8.06	1	2	2	3	1.04	1.08	1.03	1.00	0.96	0.91	0.87	0.66	0.63	1.12	1.08	1.01	0.97	44.18	9.85
Q9DD18	D-tyrosyl-tRNA(Tyr) deacylase 1 OS=Mus musculus GN=Dtd1 PE=1 SV=2 - [DTD1_MOUSE]	34.93	2	6	6	49	1.11	1.06	0.96	0.90	0.88	0.65	0.60	0.66	0.64	0.69	0.61	0.66	0.63	23.37	7.87
Q9D8B6	Protein FAM210B OS=Mus musculus GN=Fam210b PE=2 SV=3 - [F210B_MOUSE]	6.32	1	1	1	1	1.15	0.81	0.70	1.28	1.11	0.73	0.63	0.66	0.57	0.84	0.73	0.86	0.74	20.33	10.51

Q924M7	Mannose-6-phosphate isomerase OS=Mus musculus GN=Mpi PE=2 SV=1 - [MPI_MOUSE]	34.99	2	8	8	23	1.11	0.88	0.80	0.96	0.89	0.70	0.61	0.66	0.63	0.69	0.63	0.74	0.66	46.54	5.95
O08553	Dihydropyrimidinase-related protein 2 OS=Mus musculus GN=Dpysl2 PE=1 SV=2 - [DPYL2_MOUSE]	79.02	1	38	43	1771	0.92	0.92	0.98	0.90	0.98	0.67	0.73	0.67	0.74	0.64	0.72	0.69	0.75	62.24	6.38
Q922V4	Pleiotropic regulator 1 OS=Mus musculus GN=Plrg1 PE=2 SV=1 - [PLRG1_MOUSE]	37.43	3	12	12	22	1.11	0.77	0.78	0.89	0.77	0.72	0.58	0.67	0.65	0.68	0.57	0.70	0.59	56.90	9.17
P17183	Gamma-enolase OS=Mus musculus GN=Eno2 PE=1 SV=2 - [ENOG_MOUSE]	79.95	4	23	30	1568	1.01	0.92	0.91	0.88	0.88	0.65	0.64	0.67	0.67	0.64	0.64	0.63	0.62	47.27	5.11
Q9R0D8	WD repeat-containing protein 54 OS=Mus musculus GN=Wdr54 PE=2 SV=1 - [WDR54_MOUSE]	42.22	2	7	7	26	1.09	0.97	0.95	0.82	0.78	0.61	0.55	0.67	0.58	0.65	0.60	0.78	0.72	35.59	6.21
Q9CRB0	Sorting nexin-24 OS=Mus musculus GN=Snx24 PE=2 SV=1 - [SNX24_MOUSE]	5.33	1	1	1	1	0.77	0.98	1.27	1.06	1.38	0.97	1.25	0.67	0.86	1.07	1.39	0.94	1.22	19.64	7.01
A2AWP7	Rho guanine nucleotide exchange factor 10-like protein (Fragment) OS=Mus musculus GN=Arhgef10l PE=2 SV=1 - [A2AWP7_MOUSE]	17.79	5	1	1	2	0.97	0.96	0.99	0.76	0.78	0.78	0.81	0.67	0.69	1.06	1.10	0.89	0.92	17.14	4.27
D3YTT9	ZZ-type zinc finger-containing protein 3 OS=Mus musculus GN=Zzz3 PE=2 SV=1 - [D3YTT9_MOUSE]	1.76	2	1	1	2	1.00	0.80	1.08	0.67	0.91	1.18	1.18	0.67	0.90	0.91	1.24	0.93	0.93	102.14	5.86
Q6GQT6	Sterol regulatory element-binding protein cleavage-activating protein OS=Mus musculus GN=Scap PE=1 SV=1 - [SCAP_MOUSE]	1.88	1	2	2	2	0.86	1.40	1.63	1.03	1.19	0.96	1.11	0.67	0.77	0.56	0.65	0.59	0.69	139.52	7.08
Q9CQ26	STAM-binding protein OS=Mus musculus GN=Stambp PE=2 SV=1 - [STABP_MOUSE]	6.84	1	2	2	3	0.66	0.64	0.97	0.93	1.40	0.66	1.00	0.67	1.01	0.69	1.05	0.87	1.32	48.48	6.64
Q9CQJ4	E3 ubiquitin-protein ligase RING2 OS=Mus musculus GN=Rnf2 PE=1 SV=1 - [RING2_MOUSE]	3.87	1	1	1	2	0.93	0.59	0.63	0.91	0.97	0.66	0.70	0.67	0.72	0.63	0.68	0.66	0.70	37.60	6.84
Q9R1P0	Proteasome subunit alpha type-4 OS=Mus musculus GN=Psma4 PE=1 SV=1 - [PSA4_MOUSE]	22.61	3	5	5	23	0.85	1.05	1.21	0.94	1.04	0.67	0.82	0.67	0.78	0.65	0.76	0.68	0.85	29.45	7.72

Q9CRC9	Glucosamine-6-phosphate isomerase 2 OS=Mus musculus GN=Gnpda2 PE=2 SV=1 - [GNPI2_MOUSE]	56.16	5	7	9	19	0.96	0.95	1.09	0.88	0.92	0.72	0.68	0.67	0.67	0.60	0.69	0.68	0.87	31.06	6.90
Q64521	Glycerol-3-phosphate dehydrogenase, mitochondrial OS=Mus musculus GN=Gpd2 PE=1 SV=2 - [GPDM_MOUSE]	71.25	2	43	43	148	1.07	1.01	0.94	1.02	0.94	0.70	0.67	0.67	0.65	0.69	0.67	0.70	0.70	80.90	6.61
P11404	Fatty acid-binding protein, heart OS=Mus musculus GN=Fabp3 PE=1 SV=5 - [FABPH_MOUSE]	66.92	2	12	12	126	0.96	0.90	1.01	0.88	0.94	0.62	0.69	0.67	0.74	0.57	0.61	0.61	0.68	14.81	6.57
Q9CQ10	Charged multivesicular body protein 3 OS=Mus musculus GN=Chmp3 PE=1 SV=3 - [CHMP3_MOUSE]	25.00	1	3	3	10	1.11	0.72	0.70	0.85	0.85	0.88	0.80	0.67	0.72	0.91	0.73	0.99	0.80	25.20	5.06
Q9Z2L6	Multiple inositol polyphosphate phosphatase 1 OS=Mus musculus GN=Minpp1 PE=1 SV=3 - [MINP1_MOUSE]	17.67	1	6	6	9	0.98	1.12	1.18	0.95	1.01	0.72	0.73	0.67	0.72	0.77	0.75	0.84	0.85	54.50	7.49
Q8BJD1	Inter-alpha-trypsin inhibitor heavy chain H5 OS=Mus musculus GN=Itih5 PE=2 SV=1 - [ITIH5_MOUSE]	1.47	1	1	1	2	0.75	0.73	0.97	0.75	0.99	0.69	0.91	0.68	0.90	0.82	1.09	0.61	0.81	106.68	8.16
P51855	Glutathione synthetase OS=Mus musculus GN=Gss PE=2 SV=1 - [GSHB_MOUSE]	27.22	5	9	9	13	0.78	1.04	1.09	0.90	1.05	0.70	0.93	0.68	1.04	0.68	1.14	0.71	0.99	52.21	5.80
D3YTP0	Metalloreductase STEAP3 (Fragment) OS=Mus musculus GN=Steap3 PE=2 SV=1 - [D3YTP0_MOUSE]	2.86	4	1	1	1	0.95	1.04	1.09	0.89	0.93	0.59	0.62	0.68	0.71	0.64	0.67	0.62	0.65	46.94	9.16
P63325	40S ribosomal protein S10 OS=Mus musculus GN=Rps10 PE=1 SV=1 - [RS10_MOUSE]	33.33	3	6	6	21	0.78	0.71	0.88	0.77	0.97	0.72	0.88	0.68	0.87	0.68	0.84	0.65	0.83	18.90	10.15
Q9D6M3	Mitochondrial glutamate carrier 1 OS=Mus musculus GN=Slc25a22 PE=1 SV=1 - [GHC1_MOUSE]	30.34	5	6	8	53	1.03	1.38	1.26	1.25	1.10	0.68	0.63	0.68	0.63	0.88	0.80	1.02	0.96	34.65	9.09
P01837	Ig kappa chain C region OS=Mus musculus PE=1 SV=1 - [IGKC_MOUSE]	26.42	1	2	2	8	1.19	0.72	0.60	0.77	0.72	0.79	0.64	0.68	0.65	1.25	1.18	0.79	0.73	11.77	5.41
Q7M6Z0	Reticulon-4 receptor-like 2 OS=Mus musculus GN=Rtn4rl2 PE=2 SV=1 - [R4RL2_MOUSE]	13.81	2	4	4	14	0.97	0.92	0.97	0.96	0.97	0.83	0.83	0.68	0.75	0.87	0.87	0.86	0.87	46.05	7.65

G3XA35	MCG116562, isoform CRA_a OS=Mus musculus GN=Vcan PE=4 SV=1 - [G3XA35_MOUSE]	11.53	7	1	22	293	0.82	0.58	0.70	0.71	0.87	0.73	0.89	0.68	0.83	0.58	0.71	0.73	0.89	262.55	4.65
P12382	6-phosphofructokinase, liver type OS=Mus musculus GN=Pfkl PE=1 SV=4 - [K6PL_MOUSE]	32.56	1	16	21	110	1.01	1.05	1.06	1.03	1.01	0.64	0.62	0.68	0.66	0.65	0.65	0.75	0.72	85.31	7.17
O88374	Branched-chain-amino-acid aminotransferase OS=Mus musculus GN=Bcat2 PE=2 SV=1 - [O88374_MOUSE]	10.20	4	3	3	3	0.96	0.80	1.07	1.28	1.01	0.67	0.54	0.68	0.71	0.55	0.58	0.61	0.82	39.75	7.68
Q3UAW9	Transcription factor IIIB 50 kDa subunit OS=Mus musculus GN=Brf2 PE=2 SV=1 - [BRF2_MOUSE]	5.00	1	1	1	1	1.15	0.78	0.68	0.93	0.81	0.82	0.71	0.68	0.59	0.84	0.74	0.70	0.61	47.02	7.27
Q6IEE6	Transmembrane protein 132E OS=Mus musculus GN=Tmem132e PE=2 SV=1 - [T132E_MOUSE]	3.05	1	2	2	3	1.32	1.15	0.87	1.00	0.75	0.83	0.62	0.68	0.51	1.04	0.79	0.91	0.69	106.92	6.04
Q8K183	Pyridoxal kinase OS=Mus musculus GN=Pdxk PE=1 SV=1 - [PDXK_MOUSE]	49.68	2	14	14	79	0.90	0.92	1.11	1.06	1.16	0.73	0.80	0.68	0.80	0.77	0.87	0.81	0.89	34.99	6.29
P62137	Serine/threonine-protein phosphatase PP1-alpha catalytic subunit OS=Mus musculus GN=Ppp1ca PE=1 SV=1 - [PP1A_MOUSE]	75.45	1	5	20	135	1.03	1.09	1.07	0.92	0.98	0.55	0.61	0.68	0.69	0.59	0.67	0.65	0.74	37.52	6.33
G3X9A7	MCG49978 OS=Mus musculus GN=Plcxd3 PE=4 SV=1 - [G3X9A7_MOUSE]	8.41	2	2	2	6	1.37	0.74	0.54	1.07	0.95	0.87	0.70	0.68	0.50	1.09	0.94	1.04	0.94	36.29	6.46
P07310	Creatine kinase M-type OS=Mus musculus GN=Ckm PE=1 SV=1 - [KCRM_MOUSE]	37.01	1	9	10	25	0.65	0.46	0.75	0.74	1.15	5.86	9.18	0.68	1.04	0.63	1.02	0.73	1.16	43.02	7.06
Q9Z2U1	Proteasome subunit alpha type-5 OS=Mus musculus GN=Psma5 PE=1 SV=1 - [PSA5_MOUSE]	43.98	2	10	10	54	0.90	0.97	1.13	0.96	1.09	0.70	0.82	0.68	0.80	0.68	0.81	0.70	0.84	26.39	4.79
K4DI63	Cellular repressor of E1A-stimulated genes 1, isoform CRA_a OS=Mus musculus GN=Creg1 PE=4 SV=1 - [K4DI63_MOUSE]	11.70	2	1	1	1	0.99	0.73	0.73	0.78	0.79	0.55	0.55	0.68	0.69	0.58	0.58	0.60	0.60	11.01	8.03
Q921I1	Serotransferrin OS=Mus musculus GN=Tf PE=1 SV=1 - [TRFE_MOUSE]	60.55	8	2	42	264	0.93	0.88	0.95	0.69	0.76	0.69	0.74	0.68	0.73	1.01	1.09	0.67	0.76	76.67	7.18

P21460	Cystatin-C OS=Mus musculus GN=Cst3 PE=2 SV=2 - [CYTC_MOUSE]	54.29	2	7	7	103	0.90	0.85	0.96	0.85	0.97	0.67	0.74	0.68	0.76	0.70	0.81	0.67	0.70	15.52	9.00
Q9D023	Mitochondrial pyruvate carrier 2 OS=Mus musculus GN=Mpc2 PE=1 SV=1 - [MPC2_MOUSE]	26.77	4	4	4	8	0.80	0.99	1.11	1.11	1.38	0.65	0.80	0.68	0.80	0.66	0.81	0.69	0.94	14.28	10.61
O88967	ATP-dependent zinc metalloprotease YME1L1 OS=Mus musculus GN=Yme1l1 PE=2 SV=1 - [YMEL1_MOUSE]	1.54	1	1	1	2	1.10	1.16	1.05	1.29	1.17	0.69	0.62	0.68	0.62	0.87	0.79	0.77	0.70	79.98	8.97
Q8VHK5	Membrane protein MLC1 OS=Mus musculus GN=Mlc1 PE=2 SV=1 - [MLC1_MOUSE]	4.97	2	2	2	4	0.92	1.11	1.21	0.93	1.01	0.61	0.66	0.68	0.74	0.76	0.83	0.72	0.78	41.57	7.90
Q99JR1	Sideroflexin-1 OS=Mus musculus GN=Sfxn1 PE=1 SV=3 - [SFXN1_MOUSE]	31.06	1	5	7	22	1.04	1.48	1.43	1.17	1.10	0.65	0.63	0.68	0.71	0.90	0.84	0.94	0.93	35.63	9.23
Q8BM13	Noelin-2 OS=Mus musculus GN=Olfm2 PE=1 SV=2 - [NOE2_MOUSE]	16.52	1	7	7	10	0.97	0.88	0.85	1.00	1.07	0.69	0.64	0.69	0.65	0.62	0.65	0.67	0.69	50.69	8.51
P61329-2	Isoform 2 of Fibroblast growth factor 12 OS=Mus musculus GN=Fgf12 - [FGF12_MOUSE]	22.65	2	3	3	4	1.01	0.77	0.89	0.88	0.84	0.69	0.74	0.69	0.66	0.70	0.65	0.71	0.66	20.41	8.87
Q8C4U8	EGF-like repeat and discoidin I-like domain-containing protein 3 OS=Mus musculus GN=Edil3 PE=2 SV=1 - [Q8C4U8_MOUSE]	14.04	3	6	6	11	1.03	0.98	1.00	0.95	0.93	0.70	0.73	0.69	0.73	0.66	0.64	0.80	0.79	52.66	7.80
Q9Z2U0	Proteasome subunit alpha type-7 OS=Mus musculus GN=Psma7 PE=1 SV=1 - [PSA7_MOUSE]	52.42	2	11	11	47	0.90	0.93	1.07	0.98	1.09	0.67	0.72	0.69	0.75	0.65	0.76	0.70	0.77	27.84	8.46
O08749	Dihydrolipoyl dehydrogenase, mitochondrial OS=Mus musculus GN=Dld PE=1 SV=2 - [DLDH_MOUSE]	66.21	1	21	21	221	1.07	0.90	0.85	0.82	0.78	0.70	0.67	0.69	0.66	0.68	0.64	0.82	0.78	54.24	7.90
Q9CQW2	ADP-ribosylation factor-like protein 8B OS=Mus musculus GN=Arl8b PE=2 SV=1 - [ARL8B_MOUSE]	38.71	1	2	6	19	1.13	0.92	0.81	1.05	0.93	0.68	0.59	0.69	0.61	0.72	0.64	0.75	0.67	21.53	8.43
P21614	Vitamin D-binding protein OS=Mus musculus GN=Gc PE=1 SV=2 - [VTDB_MOUSE]	4.20	1	1	1	2	0.86	1.09	1.27	0.66	0.77	0.54	0.62	0.69	0.80	0.76	0.88	0.73	0.85	53.56	5.50

Q3UPV6	Voltage-gated potassium channel subunit beta-2 OS=Mus musculus GN=Kcnab2 PE=2 SV=1 - [Q3UPV6_MOUSE]	49.01	5	12	14	69	0.96	1.08	1.05	1.00	1.07	0.70	0.75	0.69	0.71	0.69	0.73	0.71	0.75	39.28	8.63
E9Q4V9	Nuclear pore complex protein Nup107 OS=Mus musculus GN=Nup107 PE=2 SV=1 - [E9Q4V9_MOUSE]	2.49	2	2	2	4	0.72	1.08	1.49	0.78	1.07	0.72	0.99	0.69	0.95	0.67	0.92	0.69	0.96	106.44	5.43
O70370	Cathepsin S OS=Mus musculus GN=Ctss PE=2 SV=2 - [CATS_MOUSE]	14.71	2	4	4	8	1.06	0.88	0.87	0.90	0.87	0.74	0.68	0.69	0.54	0.67	0.62	0.63	0.63	38.45	6.96
Q921W2	Nucleolysin TIAR OS=Mus musculus GN=Tial1 PE=2 SV=1 - [Q921W2_MOUSE]	19.73	4	4	7	13	0.89	0.74	0.92	0.91	1.08	0.67	0.79	0.69	0.87	0.67	0.79	0.73	0.86	41.50	7.74
Q93092	Transaldolase OS=Mus musculus GN=Taldo1 PE=1 SV=2 - [TALDO_MOUSE]	38.28	1	15	15	46	0.94	0.87	1.00	0.92	0.99	0.71	0.79	0.69	0.73	0.72	0.78	0.70	0.77	37.36	7.03
Q99KC8	von Willebrand factor A domain-containing protein 5A OS=Mus musculus GN=Vwa5a PE=1 SV=2 - [VMA5A_MOUSE]	13.87	4	9	9	19	1.01	0.92	0.94	0.85	0.92	0.67	0.65	0.69	0.70	0.70	0.69	0.70	0.68	87.09	6.58
Q8CIF4	Biotinidase OS=Mus musculus GN=Btd PE=1 SV=2 - [BTD_MOUSE]	4.23	1	1	2	3	0.90	1.12	1.24	0.99	1.10	0.72	0.80	0.69	0.76	0.73	0.81	0.71	0.79	58.12	5.80
G3UYE9	Killer cell lectin-like receptor subfamily B member 1C (Fragment) OS=Mus musculus GN=Klrb1c PE=2 SV=1 - [G3UYE9_MOUSE]	23.85	1	1	1	1	0.87	0.94	1.08	0.65	0.75	0.92	1.05	0.69	0.79	0.86	0.99	0.81	0.93	11.68	8.44
P42227-2	Isoform Stat3B of Signal transducer and activator of transcription 3 OS=Mus musculus GN=Stat3 - [STAT3_MOUSE]	1.94	4	1	1	2	0.92	0.94	1.03	0.98	1.07	0.78	0.84	0.69	0.75	0.75	0.82	0.72	0.79	83.07	7.12
Q8BGA9	Mitochondrial inner membrane protein OXA1L OS=Mus musculus GN=Oxa1l PE=2 SV=1 - [OXA1L_MOUSE]	2.08	1	1	1	2	0.85	1.38	1.61	0.90	1.06	0.70	0.81	0.69	0.81	0.84	0.99	0.90	1.06	48.19	9.61
Q9CZP7-2	Isoform 2 of Hsp90 co-chaperone Cdc37-like 1 OS=Mus musculus GN=Cdc37l1 - [CD37L_MOUSE]	2.93	4	1	1	3	0.78	0.61	0.78	0.79	1.01	0.66	0.84	0.69	0.88	0.64	0.82	0.61	0.78	35.38	5.92
P41539-2	Isoform Gamma of Protachykinin-1 OS=Mus musculus GN=Tac1 - [TKN1_MOUSE]	11.30	2	1	1	4	0.62	0.47	0.76	0.57	0.92	0.76	1.22	0.69	1.11	0.75	1.21	0.74	1.19	13.39	9.35

P25785	Metalloproteinase inhibitor 2 OS=Mus musculus GN=Timp2 PE=1 SV=2 - [TIMP2_MOUSE]	16.82	3	5	5	6	1.02	0.78	0.98	0.88	0.77	0.70	0.66	0.69	0.67	0.74	0.83	0.81	0.75	24.31	7.49
P61922	4-aminobutyrate aminotransferase, mitochondrial OS=Mus musculus GN=Abat PE=1 SV=1 - [GABT_MOUSE]	70.60	5	26	26	136	0.99	1.05	1.05	1.05	1.08	0.76	0.75	0.69	0.73	0.84	0.84	0.86	0.88	56.42	8.09
Q99JW2	Aminoacylase-1 OS=Mus musculus GN=Acy1 PE=1 SV=1 - [ACY1_MOUSE]	31.37	1	10	10	23	1.02	1.03	0.95	1.03	1.07	0.67	0.68	0.69	0.78	0.80	0.75	0.76	0.76	45.75	6.32
Q8CIH9	Amidophosphoribosyltransferase OS=Mus musculus GN=Ppat PE=2 SV=1 - [Q8CIH9_MOUSE]	11.99	1	5	5	11	0.91	1.14	1.23	0.93	1.04	0.72	1.00	0.69	0.75	0.66	0.68	0.77	0.91	57.37	6.96
P35700	Peroxiredoxin-1 OS=Mus musculus GN=Prdx1 PE=1 SV=1 - [PRDX1_MOUSE]	88.44	4	14	16	162	1.02	0.83	0.83	0.91	0.90	0.64	0.64	0.69	0.67	0.64	0.62	0.62	0.63	22.16	8.12
Q8BW96-	Isoform 2 of Calcium/calmodulin-dependent protein kinase type 1D OS=Mus musculus GN=Camk1d - [KCC1D_MOUSE]	41.71	2	9	12	40	0.97	0.77	0.79	0.93	0.90	0.70	0.67	0.69	0.69	0.64	0.63	0.63	0.60	41.08	6.74
P40237	CD82 antigen OS=Mus musculus GN=Cd82 PE=1 SV=1 - [CD82_MOUSE]	16.17	3	4	4	22	0.98	0.81	0.81	1.05	1.02	0.70	0.76	0.69	0.70	0.73	0.77	0.70	0.76	29.61	5.02
Q8R164	Valacyclovir hydrolase OS=Mus musculus GN=Bphl PE=2 SV=1 - [BPHL_MOUSE]	39.52	1	9	9	18	0.76	1.06	1.20	1.08	1.38	0.66	0.88	0.69	0.86	0.72	0.90	0.76	0.92	32.83	8.94
Q8BH82	N-acyl-phosphatidylethanolamine-hydrolyzing phospholipase D OS=Mus musculus GN=Napepld PE=1 SV=1 - [NAPEP_MOUSE]	6.57	1	2	2	2	1.05	0.73	0.70	1.02	0.97	0.85	0.80	0.69	0.66	0.83	0.79	1.00	0.95	45.79	5.94
P22315	Ferrochelatase, mitochondrial OS=Mus musculus GN=Fech PE=1 SV=2 - [HEMH_MOUSE]	33.57	2	10	10	32	1.00	0.87	0.88	0.90	0.96	0.74	0.74	0.69	0.71	0.72	0.70	0.67	0.73	47.10	8.91
Q32MT6	Prestin OS=Mus musculus GN=Slc26a5 PE=2 SV=1 - [Q32MT6_MOUSE]	0.85	3	1	1	1	1.07	1.00	0.92	1.32	1.23	0.58	0.54	0.69	0.64	1.08	1.01	0.51	0.47	77.23	5.80
Q9EPB4	Apoptosis-associated speck-like protein containing a CARD OS=Mus musculus GN=Pycard PE=1 SV=1 - [ASC_MOUSE]	45.08	1	5	5	9	1.03	0.94	0.92	0.89	0.90	0.71	0.65	0.70	0.73	0.70	0.64	0.78	0.76	21.45	5.43

P61982	14-3-3 protein gamma OS=Mus musculus GN=Ywhag PE=1 SV=2 - [1433G_MOUSE]	85.02	3	17	25	631	0.95	0.92	0.96	0.94	0.99	0.69	0.75	0.70	0.77	0.68	0.74	0.70	0.75	28.28	4.89
Q8BGN3	Ectonucleotide pyrophosphatase/phosphodiesterase family member 6 OS=Mus musculus GN=Enpp6 PE=2 SV=1 - [ENPP6_MOUSE]	35.00	3	13	13	54	0.95	0.94	1.08	0.87	0.96	0.67	0.73	0.70	0.76	0.69	0.75	0.84	0.91	50.59	7.31
P57716	Nicastrin OS=Mus musculus GN=Ncstn PE=1 SV=3 - [NICA_MOUSE]	11.44	1	6	6	10	0.93	0.97	1.04	0.88	0.98	0.71	0.83	0.70	0.76	0.71	0.80	0.83	0.92	78.44	6.09
O35425	Bcl-2-related ovarian killer protein OS=Mus musculus GN=Bok PE=1 SV=1 - [BOK_MOUSE]	4.69	1	1	1	1	0.73	1.09	1.49	1.18	1.62	0.80	1.09	0.70	0.96	0.70	0.96	1.09	1.50	23.44	9.11
Q3TCH7	Cullin-4A OS=Mus musculus GN=Cul4a PE=1 SV=1 - [CUL4A_MOUSE]	7.64	3	3	7	10	0.83	0.94	1.18	1.17	1.28	0.91	1.00	0.70	0.87	0.81	1.02	0.98	1.17	87.70	8.35
A2AI91	Phosphorylase b kinase regulatory subunit alpha, skeletal muscle isoform OS=Mus musculus GN=Phka1 PE=2 SV=1 - [A2AI91_MOUSE]	1.52	6	2	2	3	0.93	1.05	1.12	0.92	0.99	0.78	0.83	0.70	0.74	0.85	0.91	0.88	0.95	132.23	5.54
Q3TCR7	Dynamin-2 OS=Mus musculus GN=Dnm2 PE=2 SV=1 - [Q3TCR7_MOUSE]	21.52	15	2	22	79	1.03	1.25	1.21	1.19	1.24	0.69	0.67	0.70	0.73	0.78	0.82	0.88	0.92	97.93	7.44
Q8CGK7	Guanine nucleotide-binding protein G(olf) subunit alpha OS=Mus musculus GN=Gnal PE=1 SV=1 - [GNAL_MOUSE]	20.73	6	4	6	37	1.11	1.54	1.18	1.53	1.56	1.13	0.92	0.70	0.56	1.30	1.20	2.07	1.78	44.28	6.65
Q8R4V2	Dual specificity protein phosphatase 15 OS=Mus musculus GN=Dusp15 PE=2 SV=3 - [DUS15_MOUSE]	5.53	1	1	1	1	0.96	1.01	1.05	0.89	0.92	0.77	0.79	0.70	0.72	0.61	0.64	0.67	0.70	26.17	9.26
Q920A5	Retinoid-inducible serine carboxypeptidase OS=Mus musculus GN=Scpep1 PE=2 SV=2 - [RISC_MOUSE]	3.10	1	1	1	3	0.99	0.89	0.75	0.98	0.83	0.87	0.73	0.70	0.59	0.84	0.85	1.23	1.24	50.93	5.66
P62835	Ras-related protein Rap 1A OS=Mus musculus GN=Rap1a PE=2 SV=1 - [RAP1A_MOUSE]	57.07	1	3	9	50	0.94	1.03	1.00	0.94	0.95	0.87	0.87	0.70	0.74	0.77	0.82	0.77	0.80	20.97	6.67
Q9ER73	Elongator complex protein 4 OS=Mus musculus GN=Elp4 PE=2 SV=2 - [ELP4_MOUSE]	7.82	2	2	2	3	0.96	1.05	1.10	0.94	0.98	0.74	0.77	0.70	0.73	0.76	0.80	0.88	0.92	46.30	8.78

E9Q555	E3 ubiquitin-protein ligase RNF213 OS=Mus musculus GN=Rnf213 PE=2 SV=1 - [RN213_MOUSE]	0.78	2	1	4	4	1.07	1.05	0.98	1.08	1.00	0.71	0.66	0.70	0.65	0.87	0.81	0.70	0.66	584.13	6.77
Q9D1X8	Tetraspanin 2, isoform CRA_b OS=Mus musculus GN=Tspan2 PE=2 SV=1 - [Q9D1X8_MOUSE]	12.44	2	2	2	13	1.14	1.49	1.14	1.08	0.91	0.71	0.67	0.70	0.71	0.81	0.79	0.75	0.68	23.43	8.00
F6UP77	Putative N-acetylglucosamine-6-phosphate deacetylase (Fragment) OS=Mus musculus GN=Amdhd2 PE=2 SV=1 - [F6UP77_MOUSE]	6.73	2	1	1	2	1.05	1.02	0.97	0.95	0.90	0.80	0.76	0.70	0.67	0.73	0.69	0.88	0.84	22.20	7.02
P63330	Serine/threonine-protein phosphatase 2A catalytic subunit alpha isoform OS=Mus musculus GN=Ppp2ca PE=1 SV=1 - [PP2AA_MOUSE]	61.17	1	1	14	170	0.84	0.77	0.93	0.94	1.12	0.74	0.87	0.70	0.79	0.70	0.92	0.79	0.99	35.59	5.54
Q62361	Pro-thyrotropin-releasing hormone OS=Mus musculus GN=Trh PE=2 SV=2 - [TRH_MOUSE]	13.28	1	2	2	2	1.89	1.44	0.76	0.94	0.50	1.16	0.61	0.70	0.37	2.34	1.24	1.76	0.94	29.18	5.88
Q4FJZ2	Importin subunit alpha OS=Mus musculus GN=Kpna6 PE=2 SV=1 - [Q4FJZ2_MOUSE]	14.63	3	2	5	8	1.01	0.84	0.84	0.90	0.83	0.61	0.66	0.70	0.70	0.69	0.68	0.78	0.79	59.57	4.98
Q9D2G2	Dihydrolipoyllysine-residue succinyltransferase component of 2-oxoglutarate dehydrogenase complex, mitochondrial OS=Mus musculus GN=Dlst PE=1 SV=1 - [ODO2_MOUSE]	42.07	2	15	15	526	1.01	0.84	0.85	0.87	0.86	0.65	0.64	0.70	0.69	0.70	0.69	0.61	0.62	48.96	8.95
Q4FCQ7	Arginyl-tRNA--protein transferase 1 OS=Mus musculus GN=Ate1 PE=2 SV=1 - [Q4FCQ7_MOUSE]	11.59	4	4	4	6	1.12	0.82	0.84	1.09	1.03	0.83	0.77	0.70	0.71	0.78	0.80	0.90	0.92	58.28	8.16
Q3V360	Receptor-type tyrosine-protein phosphatase U OS=Mus musculus GN=Ptpru PE=2 SV=1 - [Q3V360_MOUSE]	1.87	3	1	1	2	0.73	0.98	1.34	0.72	0.98	0.65	0.88	0.70	0.96	0.67	0.92	0.64	0.87	100.88	8.56
Q9R1P4	Proteasome subunit alpha type-1 OS=Mus musculus GN=Psma1 PE=1 SV=1 - [PSA1_MOUSE]	40.68	1	9	9	48	0.85	1.11	1.43	0.99	1.20	0.73	0.83	0.70	0.90	0.70	0.91	0.77	0.92	29.53	6.46

P62254	Ubiquitin-conjugating enzyme E2 G1 OS=Mus musculus GN=Ube2g1 PE=2 SV=3 - [UB2G1_MOUSE]	14.71	1	2	2	2	1.09	0.92	0.84	0.80	0.74	0.80	0.73	0.70	0.65	0.65	0.60	0.92	0.84	19.50	5.30
P53395	Lipoamide acyltransferase component of branched-chain alpha-keto acid dehydrogenase complex, mitochondrial OS=Mus musculus GN=Dbt PE=2 SV=2 - [ODB2_MOUSE]	20.75	1	10	10	19	0.97	0.80	0.74	0.93	0.92	0.69	0.70	0.70	0.75	0.67	0.73	0.75	0.77	53.21	8.60
Q9D1H7-2	Isoform 2 of Golgi to ER traffic protein 4 homolog OS=Mus musculus GN=Get4 - [GET4_MOUSE]	4.01	2	1	1	1	0.77	0.97	1.25	0.94	1.21	0.93	1.20	0.71	0.91	1.17	1.51	0.88	1.14	30.54	4.98
Q5DTY9	BTB/POZ domain-containing protein KCTD16 OS=Mus musculus GN=Kctd16 PE=1 SV=2 - [KCD16_MOUSE]	29.04	1	11	12	32	0.95	0.95	1.01	0.99	1.04	0.75	0.77	0.71	0.74	0.70	0.74	0.69	0.75	48.94	8.19
P16460	Argininosuccinate synthase OS=Mus musculus GN=Ass1 PE=1 SV=1 - [ASSY_MOUSE]	26.94	2	9	9	16	1.20	0.91	0.68	1.10	0.92	0.84	0.65	0.71	0.63	0.97	0.85	1.06	0.86	46.56	8.22
Q07113	Cation-independent mannose-6-phosphate receptor OS=Mus musculus GN=Igf2r PE=1 SV=1 - [MPRI_MOUSE]	0.52	1	1	1	1	0.87	0.63	0.72	0.81	0.94	0.67	0.77	0.71	0.81	0.65	0.76	0.63	0.73	273.64	5.71
E9Q800	Mitochondrial inner membrane protein OS=Mus musculus GN=Immt PE=2 SV=1 - [E9Q800_MOUSE]	59.06	2	1	35	117	0.88	0.61	0.69	1.15	1.31	0.85	0.96	0.71	0.80	0.79	0.89	1.01	1.15	75.55	7.80
Q925I1	ATPase family AAA domain-containing protein 3 OS=Mus musculus GN=Atad3 PE=1 SV=1 - [ATAD3_MOUSE]	15.06	5	7	7	11	1.00	1.28	1.22	1.21	1.18	0.69	0.67	0.71	0.72	0.76	0.77	0.88	0.90	66.70	9.29
E9PZ69	Transmembrane 9 superfamily member 2 OS=Mus musculus GN=Tm9sf2 PE=2 SV=1 - [E9PZ69_MOUSE]	6.84	2	3	3	5	1.09	1.07	1.16	1.18	1.23	0.75	0.63	0.71	0.67	0.79	0.93	0.81	0.81	56.28	7.58
Q8K1L0-2	Isoform 2 of Cyclic AMP-responsive element-binding protein 5 OS=Mus musculus GN=Creb5 - [CREB5_MOUSE]	12.86	1	1	1	1	0.58	0.79	1.35	0.58	1.00	0.61	1.05	0.71	1.22	0.66	1.14	0.86	1.49	26.53	7.59

Q9D7X1	BTB/POZ domain-containing protein KCTD4 OS=Mus musculus GN=Kctd4 PE=2 SV=1 - [KCTD4_MOUSE]	17.76	1	4	4	7	1.12	1.24	1.06	0.94	0.77	0.91	0.82	0.71	0.63	0.93	0.84	0.92	0.72	29.96	7.06
P99026	Proteasome subunit beta type-4 OS=Mus musculus GN=Psmb4 PE=1 SV=1 - [PSB4_MOUSE]	42.80	1	8	8	29	0.80	1.10	1.36	1.01	1.19	0.71	0.88	0.71	0.86	0.64	0.79	0.72	0.88	29.10	5.64
D3YWD1	Protein Col6a3 (Fragment) OS=Mus musculus GN=Col6a3 PE=4 SV=2 - [D3YWD1_MOUSE]	0.76	3	1	1	1	1.07	0.72	0.67	0.76	0.71	0.99	0.92	0.71	0.66	1.10	1.03	1.08	1.01	185.62	5.53
Q3URE9	Leucine-rich repeat and immunoglobulin-like domain-containing nogo receptor-interacting protein 2 OS=Mus musculus GN=Lingo2 PE=2 SV=1 - [LIGO2_MOUSE]	2.15	1	1	1	1	0.89	0.77	0.87	0.86	0.97	0.87	0.97	0.71	0.80	1.27	1.43	1.16	1.31	68.03	8.13
Q8JZW4	Copine-5 OS=Mus musculus GN=Cpne5 PE=2 SV=1 - [CPNE5_MOUSE]	29.51	2	3	14	36	0.99	0.90	0.89	1.07	1.15	0.87	0.88	0.71	0.80	0.87	0.95	0.88	0.89	65.55	5.77
Q8C0M9	Isoaspartyl peptidase/L-asparaginase OS=Mus musculus GN=Asrgl1 PE=1 SV=1 - [ASGL1_MOUSE]	58.90	1	17	17	100	1.05	0.81	0.76	0.90	0.85	0.69	0.64	0.71	0.66	0.69	0.66	0.76	0.72	33.93	7.65
P51910	Apolipoprotein D OS=Mus musculus GN=Apod PE=2 SV=1 - [APOD_MOUSE]	23.28	1	4	4	12	0.90	0.95	1.04	0.92	1.04	0.65	0.68	0.71	0.70	0.56	0.63	0.56	0.65	21.52	4.91
E0CZ78	Serine/threonine-protein phosphatase OS=Mus musculus GN=Ppp3cb PE=2 SV=1 - [E0CZ78_MOUSE]	38.55	6	8	17	180	0.95	1.08	1.15	0.97	1.04	0.73	0.78	0.72	0.75	0.71	0.73	0.76	0.81	59.04	5.91
Q3TC72	Fumarylacetoacetate hydrolase domain-containing protein 2A OS=Mus musculus GN=Fahd2 PE=1 SV=1 - [FAHD2_MOUSE]	37.38	1	8	8	20	0.99	0.93	0.94	1.06	1.05	0.85	0.83	0.72	0.72	0.81	0.82	0.83	0.85	34.67	8.16
P01027	Complement C3 OS=Mus musculus GN=C3 PE=1 SV=3 - [CO3_MOUSE]	3.37	1	3	3	5	0.69	0.95	1.27	0.49	0.65	0.60	0.73	0.72	0.96	1.03	1.28	0.43	0.63	186.37	6.73
Q3UMR5	Calcium uniporter protein, mitochondrial OS=Mus musculus GN=Mcu PE=1 SV=2 - [MCU_MOUSE]	23.14	2	7	7	23	0.97	1.29	1.21	1.20	1.26	0.74	0.83	0.72	0.75	0.89	0.87	0.89	0.94	39.66	8.56
Q8K0C9	GDP-mannose 4,6 dehydratase OS=Mus musculus GN=Gmds PE=2 SV=1 - [GMDS_MOUSE]	9.68	1	3	3	4	0.87	0.84	0.97	0.95	1.09	0.84	0.94	0.72	0.76	0.85	0.86	0.85	0.90	41.96	7.03

P63056-2	Isoform 2 of Noelin-3 OS=Mus musculus GN=Olfm3 - [NOE3_MOUSE]	8.73	3	3	3	6	0.91	1.09	1.20	0.93	1.12	0.81	0.93	0.72	0.94	0.63	0.80	0.64	0.63	52.81	8.15
E9Q735	Protein Ube4a OS=Mus musculus GN=Ube4a PE=2 SV=1 - [E9Q735_MOUSE]	1.17	2	1	1	1	0.82	0.98	1.20	0.68	0.83	0.90	1.09	0.72	0.87	0.88	1.08	0.68	0.83	118.12	5.33
Q9DCU2	Plasmolipin OS=Mus musculus GN=Pllp PE=2 SV=1 - [PLLP_MOUSE]	21.43	1	2	2	4	1.10	1.30	1.09	0.87	0.86	0.81	0.80	0.72	0.69	0.74	0.73	0.95	0.99	19.79	9.41
Q3UEZ8	Sodium/bile acid cotransporter 4 OS=Mus musculus GN=Slc10a4 PE=2 SV=1 - [NTCP4_MOUSE]	3.20	1	1	1	23	1.09	0.89	0.72	1.30	1.22	0.92	0.90	0.72	0.61	1.15	1.09	1.31	1.17	46.61	5.01
Q3UYH7	Beta-adrenergic receptor kinase 2 OS=Mus musculus GN=Adrbk2 PE=2 SV=2 - [ARBK2_MOUSE]	10.32	3	2	6	12	1.10	0.93	0.80	1.10	0.99	0.77	0.69	0.72	0.65	1.04	0.94	0.97	0.88	79.61	7.69
Q9D958	Signal peptidase complex subunit 1 OS=Mus musculus GN=Spcs1 PE=2 SV=3 - [SPCS1_MOUSE]	8.07	1	1	1	1	0.87	0.61	0.70	1.07	1.23	0.76	0.87	0.72	0.83	0.82	0.95	0.76	0.88	18.17	10.01
Q8BVA2	Transmembrane protein 222 OS=Mus musculus GN=Tmem222 PE=2 SV=1 - [TM222_MOUSE]	12.50	1	1	1	2	0.85	0.92	1.08	1.14	1.33	0.88	1.02	0.72	0.84	0.83	0.97	0.81	0.95	23.16	6.61
Q8BH24	Transmembrane 9 superfamily member 4 OS=Mus musculus GN=Tm9sf4 PE=2 SV=1 - [TM9S4_MOUSE]	1.24	1	1	1	1	1.04	1.19	1.14	1.29	1.24	0.84	0.81	0.72	0.69	0.95	0.91	0.88	0.84	74.64	7.23
P39038	Cadherin-4 OS=Mus musculus GN=Cdh4 PE=2 SV=1 - [CADH4_MOUSE]	2.41	1	1	2	5	0.85	0.82	0.95	0.77	0.90	0.78	0.90	0.72	0.84	0.75	0.88	0.75	0.88	99.97	4.81
Q99ME2	WD repeat-containing protein 6 OS=Mus musculus GN=Wdr6 PE=2 SV=1 - [WDR6_MOUSE]	2.13	1	2	2	5	1.13	1.24	1.05	1.02	0.92	0.68	0.55	0.72	0.61	0.82	0.75	0.97	0.74	121.82	6.98
Q9DB42	Zinc finger protein 593 OS=Mus musculus GN=Znf593 PE=2 SV=2 - [ZN593_MOUSE]	23.88	1	2	2	3	1.00	0.85	0.85	0.79	0.79	0.73	0.73	0.72	0.72	0.82	0.83	0.77	0.77	15.14	9.55
Q9CQE6	Histone chaperone ASF1A OS=Mus musculus GN=Asf1a PE=2 SV=1 - [ASF1A_MOUSE]	5.39	1	1	1	3	0.94	0.66	0.70	0.83	0.88	0.75	0.79	0.72	0.76	0.72	0.76	0.70	0.75	22.93	4.41
Q8VDH1-	Isoform 2 of F-box only protein 21 OS=Mus musculus GN=Fbxo21 - [FBX21_MOUSE]	3.71	2	1	1	2	0.67	1.01	1.50	0.79	1.17	0.69	1.03	0.72	1.08	0.89	1.34	0.80	1.20	71.34	5.99

Q8VEG4	Exonuclease 3'-5' domain-containing protein 2 OS=Mus musculus GN=Exd2 PE=2 SV=1 - [EXD2_MOUSE]	2.02	2	1	1	1	0.75	0.38	0.50	0.87	1.16	0.77	1.02	0.72	0.96	0.69	0.93	0.64	0.86	56.75	8.16
Q8CC88	von Willebrand factor A domain-containing protein 8 OS=Mus musculus GN=Vwa8 PE=2 SV=2 - [VWA8_MOUSE]	3.25	2	5	5	9	1.04	1.23	1.20	1.15	1.09	0.77	0.72	0.72	0.65	0.82	0.83	0.96	0.83	213.29	6.60
Q8BH15-6	Isoform 6 of CCR4-NOT transcription complex subunit 10 OS=Mus musculus GN=Cnot10 - [CNO10_MOUSE]	30.00	3	1	2	3	0.74	0.72	0.96	0.86	1.16	0.75	1.00	0.72	0.97	0.64	0.87	0.73	0.99	11.36	9.77
Q99K70-2	Isoform 2 of Ras-related GTP-binding protein C OS=Mus musculus GN=Rragc - [RRAGC_MOUSE]	7.69	2	1	1	2	1.14	0.79	0.69	1.10	0.96	0.83	0.72	0.72	0.63	0.74	0.65	0.98	0.86	19.64	5.38
Q8BH59	Calcium-binding mitochondrial carrier protein Aralar1 OS=Mus musculus GN=Slc25a12 PE=1 SV=1 - [CMC1_MOUSE]	55.54	2	28	28	97	1.07	1.16	1.15	1.26	1.24	0.74	0.71	0.72	0.67	0.82	0.79	0.95	0.89	74.52	8.25
Q6P5U8-2	Isoform 2 of Coiled-coil domain-containing protein 148 OS=Mus musculus GN=Ccdc148 - [CC148_MOUSE]	6.02	2	1	1	2	0.78	0.80	1.02	0.63	0.81	0.62	0.79	0.72	0.93	0.70	0.90	0.47	0.60	15.97	8.90
Q8C4X2	Casein kinase I isoform gamma-3 OS=Mus musculus GN=Csnk1g3 PE=1 SV=2 - [KC1G3_MOUSE]	10.38	3	2	4	8	0.89	0.81	0.96	0.94	1.06	0.85	1.09	0.72	0.81	0.86	1.10	0.90	0.97	48.91	9.11
Q52KF0	Thioredoxin-like 4B OS=Mus musculus GN=Txnl4b PE=2 SV=1 - [Q52KF0_MOUSE]	6.71	2	1	1	1	0.93	0.86	0.93	0.70	0.76	0.71	0.76	0.72	0.78	0.68	0.73	0.77	0.84	17.01	6.33
F7ANA1	PHD and RING finger domain-containing protein 1 (Fragment) OS=Mus musculus GN=Phrf1 PE=4 SV=1 - [F7ANA1_MOUSE]	1.68	3	1	1	3	0.73	0.77	1.06	0.98	1.34	1.06	1.37	0.72	0.99	1.05	1.42	0.91	1.18	85.95	9.66
Q6NVG1	Lysophospholipid acyltransferase LPCAT4 OS=Mus musculus GN=Lpcat4 PE=1 SV=1 - [LPCT4_MOUSE]	8.78	1	3	3	5	0.95	1.19	1.25	1.31	1.38	0.94	0.98	0.73	0.77	0.86	0.90	1.05	1.09	57.11	8.75
D3YX28	Serine protease HTRA2, mitochondrial OS=Mus musculus GN=Htra2 PE=2 SV=1 - [D3YX28_MOUSE]	21.33	5	5	5	9	0.95	0.79	0.86	0.90	0.90	0.65	0.67	0.73	0.74	0.76	0.77	0.65	0.69	38.92	10.13

P11679	Keratin, type II cytoskeletal 8 OS=Mus musculus GN=Krt8 PE=1 SV=4 - [K2C8_MOUSE]	6.94	3	1	4	6	1.29	1.02	0.79	2.38	1.84	0.80	0.62	0.73	0.56	1.61	1.25	1.00	0.77	54.53	5.82
A2A8U2-:	Isoform Samp1 of Transmembrane protein 201 OS=Mus musculus GN=Tmem201 - [TM201_MOUSE]	5.61	3	2	2	3	0.92	1.06	1.15	1.13	1.23	0.71	0.76	0.73	0.79	0.84	0.91	0.93	1.01	43.52	8.56
Q9DC50	Peroxisomal carnitine O-octanoyltransferase OS=Mus musculus GN=Crot PE=1 SV=1 - [OCTC_MOUSE]	4.08	1	2	2	5	1.23	1.26	1.02	0.70	0.57	0.76	0.67	0.73	0.59	0.78	0.64	0.63	0.64	70.22	6.73
Q8VCV1	Alpha/beta hydrolase domain-containing protein 17C OS=Mus musculus GN=Abhd17c PE=2 SV=2 - [AB17C_MOUSE]	7.50	1	1	1	1	0.43	0.89	2.07	0.84	1.95	0.78	1.80	0.73	1.69	1.23	2.87	0.80	1.88	35.08	5.64
P42669	Transcriptional activator protein Pur-alpha OS=Mus musculus GN=Pura PE=1 SV=1 - [PURA_MOUSE]	59.50	1	16	18	128	1.04	0.98	0.90	0.92	0.89	0.68	0.64	0.73	0.69	0.65	0.64	0.67	0.65	34.86	6.44
E9PWY6	2-amino-3-ketobutyrate coenzyme A ligase, mitochondrial OS=Mus musculus GN=Gcat PE=2 SV=1 - [E9PWY6_MOUSE]	10.99	2	3	3	8	0.82	1.29	1.46	0.99	1.27	0.80	0.84	0.73	0.78	0.75	1.00	0.77	1.03	41.31	7.91
Q58A65-2	Isoform 2 of C-Jun-amino-terminal kinase-interacting protein 4 OS=Mus musculus GN=Spag9 - [JIP4_MOUSE]	27.70	10	26	28	98	0.96	0.88	0.93	0.92	0.97	0.74	0.77	0.73	0.79	0.71	0.76	0.72	0.76	144.61	5.15
F7BX26	Serine/threonine-protein phosphatase (Fragment) OS=Mus musculus GN=Ppp5c PE=3 SV=1 - [F7BX26_MOUSE]	42.44	3	15	15	123	0.99	0.98	0.98	0.93	0.93	0.69	0.71	0.73	0.72	0.71	0.72	0.71	0.72	54.28	6.20
Q60759	Glutaryl-CoA dehydrogenase, mitochondrial OS=Mus musculus GN=Gcdh PE=2 SV=2 - [GCDH_MOUSE]	13.47	2	4	4	9	1.00	0.99	0.92	1.06	1.06	0.88	0.79	0.73	0.74	0.92	0.95	0.92	0.89	48.57	8.73
Q8VDP6	CDP-diacylglycerol--inositol 3-phosphatidyltransferase OS=Mus musculus GN=Cdipt PE=1 SV=1 - [CDIPT_MOUSE]	5.16	1	1	1	2	0.98	1.04	1.06	1.19	1.21	0.75	0.76	0.73	0.74	0.71	0.72	1.03	1.05	23.58	8.27
Q811C2	Cysteine protease ATG4C OS=Mus musculus GN=Atg4c PE=2 SV=2 - [ATG4C_MOUSE]	4.37	1	1	1	2	0.85	1.09	1.27	1.00	1.18	1.02	1.19	0.73	0.85	1.06	1.25	1.11	1.30	52.02	6.43

O54864-2	Isoform 2 of Histone-lysine N-methyltransferase SUV39H1 OS=Mus musculus GN=Suv39h1 - [SUV91_MOUSE]	3.53	1	1	1	1	0.79	1.28	1.62	0.92	1.16	0.78	0.98	0.73	0.92	0.77	0.98	0.66	0.84	51.86	8.37
Q62465	Synaptic vesicle membrane protein VAT-1 homolog OS=Mus musculus GN=Vat1 PE=1 SV=3 - [VAT1_MOUSE]	41.38	1	12	12	29	1.15	0.95	0.84	1.01	0.84	0.80	0.67	0.73	0.66	0.91	0.82	0.89	0.83	43.07	6.37
Q64674	Spermidine synthase OS=Mus musculus GN=Srm PE=2 SV=1 - [SPEE_MOUSE]	10.26	1	3	3	5	1.16	1.02	0.79	1.20	1.03	0.93	0.80	0.73	0.63	0.83	0.82	0.98	0.83	33.97	5.50
Q3TL44	NLR family member X1 OS=Mus musculus GN=Nlrx1 PE=2 SV=1 - [NLRX1_MOUSE]	1.03	1	1	1	2	1.26	1.59	1.26	1.25	0.99	0.85	0.67	0.73	0.58	0.85	0.68	1.14	0.91	107.76	7.37
F6W3U3	Protein 2310061I04Rik (Fragment) OS=Mus musculus GN=2310061I04Rik PE=4 SV=1 - [F6W3U3_MOUSE]	11.01	4	1	1	4	0.97	1.00	1.03	1.09	1.12	0.77	0.79	0.73	0.75	0.80	0.82	0.96	0.98	13.20	10.26
Q8BIP0	Aspartate--tRNA ligase, mitochondrial OS=Mus musculus GN=Dars2 PE=2 SV=1 - [SYDM_MOUSE]	3.83	1	2	2	2	1.02	1.09	1.07	0.99	0.97	0.86	0.84	0.73	0.71	0.91	0.89	0.91	0.89	74.06	6.98
Q9CQV6	Microtubule-associated proteins 1A/1B light chain 3B OS=Mus musculus GN=Map1lc3b PE=1 SV=3 - [MLP3B_MOUSE]	20.00	3	3	4	35	0.80	0.68	0.98	0.84	1.20	0.67	0.85	0.73	0.94	0.67	0.92	0.61	0.78	14.61	8.43
Q99PH1	Leucine-rich repeat-containing protein 4 OS=Mus musculus GN=Lrrc4 PE=1 SV=2 - [LRRC4_MOUSE]	2.15	1	1	1	2	1.05	1.24	1.18	1.04	0.99	0.75	0.71	0.73	0.70	0.64	0.61	0.68	0.65	72.57	7.01
Q9CQR6	Serine/threonine-protein phosphatase 6 catalytic subunit OS=Mus musculus GN=Ppp6c PE=2 SV=1 - [PPP6_MOUSE]	8.20	1	2	2	4	1.15	1.04	0.90	0.97	0.84	0.81	0.70	0.73	0.63	0.73	0.63	0.92	0.80	35.14	5.69
Q3TYS2	Uncharacterized protein C17orf62 homolog OS=Mus musculus PE=2 SV=2 - [CQ062_MOUSE]	5.35	1	1	1	2	1.06	1.17	1.11	0.91	0.86	0.85	0.80	0.73	0.69	0.83	0.78	1.05	1.00	20.91	8.59
Q3TE80	Protein 2410002F23Rik OS=Mus musculus GN=2410002F23Rik PE=2 SV=1 - [Q3TE80_MOUSE]	10.92	4	3	3	5	1.06	1.23	1.19	1.04	0.98	0.94	0.89	0.73	0.69	0.79	0.75	0.95	0.96	32.35	6.32

Q8BFS6	Calcineurin-like phosphoesterase domain-containing protein 1 OS=Mus musculus GN=Cpped1 PE=2 SV=1 - [CPPED_MOUSE]	35.26	6	7	7	22	1.00	0.80	0.81	0.94	0.94	0.81	0.80	0.73	0.71	0.66	0.64	0.72	0.72	35.23	5.34
Q80UK0	SEC14 domain and spectrin repeat-containing protein 1 OS=Mus musculus GN=Sestd1 PE=2 SV=1 - [SESD1_MOUSE]	1.44	1	1	1	1	0.82	0.91	1.11	0.81	0.99	0.69	0.83	0.74	0.89	1.02	1.25	1.07	1.31	79.33	5.10
D3Z3D2	Nuclear cap-binding protein subunit 2 OS=Mus musculus GN=Ncbp2 PE=2 SV=1 - [D3Z3D2_MOUSE]	10.68	2	1	1	1	0.56	0.78	1.39	0.71	1.27	0.69	1.22	0.74	1.31	0.71	1.27	0.58	1.05	11.94	8.34
Q9Z2Y3	Homer protein homolog 1 OS=Mus musculus GN=Homer1 PE=1 SV=2 - [HOME1_MOUSE]	61.20	9	17	25	240	0.87	0.89	1.00	0.84	0.97	0.69	0.76	0.74	0.85	0.61	0.69	0.61	0.71	41.39	5.53
Q99PP2	Zinc finger protein 318 (Fragment) OS=Mus musculus GN=Znf318 PE=1 SV=2 - [ZN318_MOUSE]	1.11	4	2	2	3	0.79	0.72	0.91	0.67	0.85	0.95	1.19	0.74	0.93	0.91	1.15	0.81	1.03	228.08	5.96
Q62413	Ephrin type-A receptor 6 OS=Mus musculus GN=Epha6 PE=2 SV=2 - [EPHA6_MOUSE]	3.38	5	1	4	7	1.03	1.04	1.01	1.07	1.04	0.85	0.82	0.74	0.71	0.92	0.89	0.88	0.86	116.11	6.95
Q8C0J2-4	Isoform 4 of Autophagy-related protein 16-1 OS=Mus musculus GN=Atg16l1 - [A16L1_MOUSE]	15.60	6	4	4	5	0.83	0.97	1.30	0.97	1.09	0.88	0.99	0.74	1.01	0.96	1.03	0.87	1.21	52.99	5.94
P26049	Gamma-aminobutyric acid receptor subunit alpha-3 OS=Mus musculus GN=Gabra3 PE=1 SV=1 - [GBRA3_MOUSE]	6.30	3	1	3	8	0.99	0.79	0.80	1.13	1.14	0.74	0.74	0.74	0.74	0.88	0.89	0.83	0.83	55.36	8.94
E9PV21	Tetratricopeptide repeat protein 39C OS=Mus musculus GN=Ttc39c PE=2 SV=1 - [E9PV21_MOUSE]	2.30	3	1	1	2	1.17	1.02	0.87	1.11	0.95	0.92	0.78	0.74	0.63	1.09	0.93	1.17	1.00	58.88	7.50
F6SZ47	Serine/threonine-protein kinase B-raf (Fragment) OS=Mus musculus GN=Braf PE=2 SV=1 - [F6SZ47_MOUSE]	12.53	4	1	6	14	1.08	0.90	0.83	0.58	0.53	1.35	1.24	0.74	0.68	0.99	0.92	0.91	0.85	83.15	7.68

E9PX48	Dedicator of cytokinesis protein 7 OS=Mus musculus GN=Dock7 PE=2 SV=1 - [E9PX48_MOUSE]	0.81	4	1	1	2	1.05	0.99	0.94	1.06	1.00	0.99	0.94	0.74	0.70	0.98	0.93	0.99	0.94	237.95	6.79
Q9D2F8	Protein 4930547C10Rik OS=Mus musculus GN=4930547C10Rik PE=2 SV=1 - [Q9D2F8_MOUSE]	3.22	1	1	1	1	1.68	2.08	1.24	0.69	0.41	0.71	0.42	0.74	0.44	0.70	0.42	0.74	0.44	77.84	9.61
Q8BRQ9	Sideroflexin-5 OS=Mus musculus GN=Sfxn5 PE=2 SV=1 - [Q8BRQ9_MOUSE]	23.84	3	5	5	11	1.01	1.25	1.17	1.24	1.26	0.61	0.75	0.74	0.69	0.82	0.79	0.72	0.76	32.76	9.36
P42208	Septin-2 OS=Mus musculus GN=Sept2 PE=1 SV=2 - [SEPT2_MOUSE]	70.91	10	16	17	59	0.94	0.97	0.97	0.97	1.03	0.75	0.78	0.74	0.79	0.74	0.76	0.77	0.83	41.50	6.55
Q4VBE7	BTB/POZ domain-containing protein KCTD2 OS=Mus musculus GN=Kctd2 PE=2 SV=1 - [Q4VBE7_MOUSE]	6.84	3	1	1	2	1.15	1.31	1.13	1.13	0.99	0.62	0.53	0.74	0.64	0.62	0.54	0.67	0.58	28.56	5.29
O09164	Extracellular superoxide dismutase [Cu-Zn] OS=Mus musculus GN=Sod3 PE=1 SV=1 - [SODE_MOUSE]	11.16	1	2	2	5	1.02	0.95	1.02	0.90	0.88	0.79	0.76	0.74	0.72	0.83	0.81	0.98	0.96	27.37	6.84
Q8CGQ8-	Isoform 2 of Sodium/potassium/calcium exchanger 4 OS=Mus musculus GN=Slc24a4 - [NCKX4_MOUSE]	2.89	3	1	1	3	0.87	0.91	1.03	1.00	1.14	0.76	0.86	0.74	0.85	0.89	1.02	0.91	1.05	61.08	7.93
Q9CXJ4	ATP-binding cassette sub-family B member 8, mitochondrial OS=Mus musculus GN=Abcb8 PE=2 SV=1 - [ABCB8_MOUSE]	7.53	3	4	4	7	1.03	1.30	1.28	1.17	1.16	0.72	0.72	0.74	0.72	0.87	0.91	0.95	0.95	77.95	9.07
Q8BW22	Calcium-responsive transactivator OS=Mus musculus GN=Ss18l1 PE=1 SV=1 - [CREST_MOUSE]	13.93	1	3	3	4	0.81	0.60	0.82	0.68	0.84	0.94	1.04	0.74	0.96	0.65	0.85	0.63	0.82	43.70	6.58
Q3UEG7	Inter-alpha-trypsin inhibitor heavy chain H2 (Fragment) OS=Mus musculus GN=Itih2 PE=2 SV=1 - [Q3UEG7_MOUSE]	3.32	3	1	1	2	0.82	0.93	1.13	0.58	0.71	0.80	0.97	0.74	0.90	0.91	1.11	0.75	0.92	50.71	6.21
Q9JJA4	Ribosome biogenesis protein WDR12 OS=Mus musculus GN=Wdr12 PE=2 SV=1 - [WDR12_MOUSE]	13.95	2	3	3	5	0.99	0.79	0.85	0.99	0.87	0.72	0.68	0.74	0.71	0.66	0.67	0.83	0.80	47.32	5.60

Q99KE1	NAD-dependent malic enzyme, mitochondrial OS=Mus musculus GN=Me2 PE=2 SV=1 - [MAOM_MOUSE]	23.94	1	8	9	19	1.00	1.16	1.15	1.23	1.30	0.91	0.91	0.74	0.78	0.97	0.97	0.99	1.06	65.76	7.61
P17563	Selenium-binding protein 1 OS=Mus musculus GN=Selenbp1 PE=1 SV=2 - [SBP1_MOUSE]	42.58	6	15	15	37	1.12	1.02	1.03	1.05	0.93	0.76	0.71	0.74	0.71	0.76	0.68	0.80	0.71	52.48	6.29
Q6ZWZ2	Ubiquitin-conjugating enzyme E2 R2 OS=Mus musculus GN=Ube2r2 PE=2 SV=1 - [UB2R2_MOUSE]	18.49	1	2	2	7	0.94	1.09	1.15	0.89	0.95	0.86	0.91	0.74	0.75	0.82	0.87	0.79	0.84	27.15	4.42
Q8R349	Cell division cycle protein 16 homolog OS=Mus musculus GN=Cdc16 PE=2 SV=1 - [CDC16_MOUSE]	2.74	1	1	1	1	0.85	1.08	1.27	0.57	0.67	0.52	0.61	0.75	0.87	0.71	0.84	0.59	0.69	71.41	5.76
Q9Z0S9	Prenylated Rab acceptor protein 1 OS=Mus musculus GN=Rabac1 PE=1 SV=1 - [PRAF1_MOUSE]	7.57	1	1	1	3	0.81	1.39	1.72	0.98	1.22	0.80	0.99	0.75	0.92	0.77	0.96	0.89	1.10	20.61	7.90
Q9JHW2	Omega-amidase NIT2 OS=Mus musculus GN=Nit2 PE=1 SV=1 - [NIT2_MOUSE]	42.75	1	8	8	26	0.97	0.87	0.93	0.97	1.01	0.76	0.75	0.75	0.72	0.77	0.78	0.83	0.88	30.48	6.90
Q80Y50-5	Isoform 5 of Calmodulin-binding transcription activator 2 OS=Mus musculus GN=Camta2 - [CMTA2_MOUSE]	1.90	7	1	1	2	1.23	1.21	0.99	1.49	1.21	0.91	0.74	0.75	0.61	0.79	0.65	1.10	0.90	107.79	6.42
Q922P8	Transmembrane protein 132A OS=Mus musculus GN=Tmem132a PE=2 SV=2 - [T132A_MOUSE]	16.40	2	11	11	28	1.04	1.08	1.05	0.99	0.93	0.78	0.73	0.75	0.78	0.77	0.73	0.70	0.72	110.17	5.63
D3YWZ5	Transporter (Fragment) OS=Mus musculus GN=Slc6a13 PE=2 SV=1 - [D3YWZ5_MOUSE]	4.69	2	1	1	2	1.06	1.05	0.99	0.99	0.93	0.52	0.49	0.75	0.70	0.82	0.78	0.68	0.64	21.51	5.10
P61514	60S ribosomal protein L37a OS=Mus musculus GN=Rpl37a PE=2 SV=2 - [RL37A_MOUSE]	32.61	1	2	2	2	0.76	0.95	1.25	0.98	1.30	0.82	1.08	0.75	0.98	0.84	1.12	0.92	1.21	10.27	10.43
P23818	Glutamate receptor 1 OS=Mus musculus GN=Gria1 PE=1 SV=1 - [GRIA1_MOUSE]	20.73	2	1	15	34	1.00	0.70	0.70	0.98	0.98	0.79	0.79	0.75	0.75	0.84	0.84	0.79	0.79	101.50	7.69
Q9CQS4	Solute carrier family 25 member 46 OS=Mus musculus GN=Slc25a46 PE=1 SV=1 - [S2546_MOUSE]	11.00	1	3	3	7	0.88	1.14	1.38	1.04	1.17	0.70	0.84	0.75	0.86	0.77	0.87	0.77	0.87	46.19	7.64

O55125	Protein NipSnap homolog 1 OS=Mus musculus GN=Nipsnap1 PE=1 SV=1 - [NIPS1_MOUSE]	60.92	2	13	13	33	0.88	0.92	1.14	0.95	1.08	0.71	0.82	0.75	0.89	0.74	0.87	0.79	0.87	33.34	9.44
Q61884	Meiosis-specific nuclear structural protein 1 OS=Mus musculus GN=Mns1 PE=1 SV=1 - [MNS1_MOUSE]	1.83	1	1	1	1	1.27	0.78	0.61	0.95	0.75	0.58	0.46	0.75	0.59	0.74	0.58	0.73	0.57	60.20	6.65
Q99ML4	Protein FAM69B OS=Mus musculus GN=Fam69b PE=1 SV=1 - [FA69B_MOUSE]	3.25	1	1	1	2	1.07	1.32	1.23	1.12	1.05	0.78	0.72	0.75	0.70	0.91	0.85	1.02	0.95	48.76	8.15
Q810C1	SLIT and NTRK-like protein 1 OS=Mus musculus GN=Slitrk1 PE=2 SV=1 - [SLIK1_MOUSE]	4.45	1	3	3	4	0.98	0.73	0.67	0.91	0.93	0.75	0.70	0.75	0.62	0.75	0.62	0.75	0.67	77.77	6.49
Q3TYX3	SET and MYND domain-containing protein 5 OS=Mus musculus GN=Smyd5 PE=2 SV=2 - [SMYD5_MOUSE]	4.81	1	2	2	3	1.05	1.17	1.11	1.06	1.00	0.81	0.76	0.75	0.71	0.91	0.86	1.04	0.99	47.06	5.21
P61021	Ras-related protein Rab-5B OS=Mus musculus GN=Rab5b PE=1 SV=1 - [RAB5B_MOUSE]	25.12	2	2	4	30	1.15	1.08	0.89	1.25	1.06	0.77	0.69	0.75	0.70	0.81	0.65	0.99	0.85	23.69	8.13
Q9CQI3	Glia maturation factor beta OS=Mus musculus GN=Gmfb PE=1 SV=3 - [GMFB_MOUSE]	65.49	5	9	9	83	0.97	0.81	0.84	0.82	0.90	0.71	0.75	0.75	0.78	0.64	0.70	0.69	0.72	16.71	5.16
Q60631	Growth factor receptor-bound protein 2 OS=Mus musculus GN=Grb2 PE=1 SV=1 - [GRB2_MOUSE]	65.90	4	13	13	55	0.89	0.69	0.81	0.78	0.93	0.67	0.75	0.75	0.87	0.66	0.82	0.69	0.82	25.22	6.32
Q8CHK3	Lysophospholipid acyltransferase 7 OS=Mus musculus GN=Mboat7 PE=2 SV=1 - [MBOA7_MOUSE]	2.54	1	1	1	2	0.95	0.63	0.67	1.29	1.36	0.79	0.83	0.75	0.79	0.97	1.02	0.98	1.04	53.40	8.69
Q9CXW3	Calcyclin-binding protein OS=Mus musculus GN=Cacybp PE=1 SV=1 - [CYBP_MOUSE]	50.22	1	11	12	41	1.22	0.67	0.61	0.92	0.78	0.70	0.58	0.75	0.60	0.71	0.58	0.74	0.63	26.49	7.87
Q9Z255	Ubiquitin-conjugating enzyme E2 A OS=Mus musculus GN=Ube2a PE=2 SV=1 - [UBE2A_MOUSE]	17.76	1	2	2	5	1.02	0.92	0.90	0.84	0.82	0.78	0.78	0.75	0.68	0.67	0.66	0.68	0.68	17.30	5.15
G3X9E8	MCG15724, isoform CRA_a OS=Mus musculus GN=Ppp2r3a PE=4 SV=1 - [G3X9E8_MOUSE]	2.64	2	1	1	2	1.04	1.17	1.12	1.00	0.96	0.82	0.78	0.75	0.72	0.80	0.77	0.95	0.91	61.07	4.96

A2ALK8	Tyrosine-protein phosphatase non-receptor type 3 OS=Mus musculus GN=Ptpn3 PE=2 SV=1 - [PTN3_MOUSE]	1.20	1	1	1	2	0.82	0.44	0.54	1.01	1.23	0.80	0.97	0.75	0.92	0.75	0.92	0.59	0.73	103.83	7.20
P03911	NADH-ubiquinone oxidoreductase chain 4 OS=Mus musculus GN=Mtnd4 PE=1 SV=1 - [NU4M_MOUSE]	3.05	1	1	1	2	0.96	1.17	1.22	1.17	1.22	0.67	0.70	0.75	0.78	0.73	0.76	0.89	0.94	51.85	9.38
Q8R5H1	Ubiquitin carboxyl-terminal hydrolase 15 OS=Mus musculus GN=Usp15 PE=2 SV=1 - [UBP15_MOUSE]	26.91	5	19	20	32	0.90	0.83	0.96	0.95	1.06	0.74	0.84	0.75	0.92	0.75	0.85	0.71	0.79	112.25	5.17
B1AS67	Pleckstrin homology domain-containing family G member 5 OS=Mus musculus GN=Plekhg5 PE=2 SV=1 - [B1AS67_MOUSE]	3.49	3	2	2	4	0.85	1.81	2.12	0.95	1.12	0.92	1.07	0.75	0.88	0.82	0.97	0.84	0.99	117.77	6.83
P34884	Macrophage migration inhibitory factor OS=Mus musculus GN=Mif PE=1 SV=2 - [MIF_MOUSE]	36.52	1	3	3	21	0.84	1.02	1.22	0.93	1.10	0.67	0.79	0.75	0.82	0.73	0.82	0.87	0.98	12.50	7.34
D3Z2B3	Protein Acad12 OS=Mus musculus GN=Acad12 PE=2 SV=1 - [D3Z2B3_MOUSE]	2.68	3	1	1	1	0.87	0.99	1.14	0.99	1.13	0.72	0.82	0.75	0.86	0.76	0.88	0.72	0.83	41.32	8.31
E9QA19	CCR4-NOT transcription complex subunit 4 OS=Mus musculus GN=Cnot4 PE=2 SV=1 - [E9QA19_MOUSE]	11.53	4	6	6	10	1.05	0.89	0.86	1.02	0.97	0.66	0.66	0.75	0.80	0.69	0.74	0.71	0.74	70.74	7.14
P32211	Muscarinic acetylcholine receptor M4 OS=Mus musculus GN=Chrm4 PE=2 SV=1 - [ACM4_MOUSE]	5.43	1	1	1	1	1.04	0.80	0.77	0.95	0.91	0.69	0.66	0.75	0.72	0.71	0.69	0.76	0.73	52.94	9.88
Q8R5L3-2	Isoform 2 of Vam6/Vps39-like protein OS=Mus musculus GN=Vps39 - [VPS39_MOUSE]	2.51	2	1	2	2	1.40	2.49	1.78	1.29	0.92	1.16	0.83	0.75	0.54	1.05	0.75	1.10	0.79	100.59	7.12
Q922E6	FAST kinase domain-containing protein 2 OS=Mus musculus GN=Fastkd2 PE=2 SV=2 - [FAKD2_MOUSE]	2.61	1	1	1	2	0.99	0.98	0.99	1.03	1.04	0.85	0.86	0.75	0.76	0.89	0.90	1.00	1.01	78.90	8.90
Q921Y0-2	Isoform 2 of MOB kinase activator 1A OS=Mus musculus GN=Mob1a - [MOB1A_MOUSE]	7.86	4	1	1	2	0.70	0.71	1.01	0.82	1.18	0.60	0.86	0.75	1.07	0.74	1.05	0.55	0.78	16.33	8.94

O09126	Semaphorin-4D OS=Mus musculus GN=Sema4d PE=1 SV=2 - [SEM4D_MOUSE]	5.11	1	3	3	5	0.97	0.78	0.80	1.04	1.07	0.76	0.78	0.75	0.78	0.97	0.97	0.86	0.89	95.58	7.81
Q9EPK7-2	Isoform 2 of Exportin-7 OS=Mus musculus GN=Xpo7 - [XPO7_MOUSE]	5.58	3	5	5	6	0.99	1.07	1.03	1.02	0.92	0.80	0.83	0.75	0.86	0.88	0.78	1.08	1.15	120.31	6.38
P0C6F1	Dynein heavy chain 2, axonemal OS=Mus musculus GN=Dnah2 PE=2 SV=1 - [DYH2_MOUSE]	0.40	2	1	1	1	0.61	0.93	1.52	1.28	2.08	0.62	1.01	0.75	1.23	0.87	1.41	0.58	0.94	511.24	6.43
Q9R112	Sulfide:quinone oxidoreductase, mitochondrial OS=Mus musculus GN=Sqrdl PE=2 SV=3 - [SQRD_MOUSE]	2.22	1	1	1	1	1.01	0.74	0.74	0.98	0.97	0.78	0.77	0.75	0.75	0.85	0.85	0.85	0.85	50.25	9.09
Q8BGN5	NIPA-like protein 3 OS=Mus musculus GN=Nipal3 PE=2 SV=1 - [NPAL3_MOUSE]	2.20	1	1	1	2	1.00	0.96	0.96	1.02	1.02	0.75	0.74	0.75	0.75	0.82	0.82	0.83	0.83	44.78	7.75
Q80V26	Inositol monophosphatase 3 OS=Mus musculus GN=Impad1 PE=1 SV=1 - [IMPA3_MOUSE]	6.18	1	2	2	3	0.96	0.78	0.81	0.94	0.98	0.72	0.75	0.76	0.78	0.72	0.75	0.69	0.72	38.59	6.47
P13020-2	Isoform 2 of Gelsolin OS=Mus musculus GN=Gsn - [GELS_MOUSE]	28.18	3	14	14	37	0.90	0.91	1.04	0.89	1.02	0.75	0.83	0.76	0.90	0.76	0.84	0.77	0.87	80.71	5.76
P97765	WW domain-binding protein 2 OS=Mus musculus GN=Wbp2 PE=1 SV=1 - [WBP2_MOUSE]	38.70	2	8	8	69	0.92	0.82	0.89	0.91	1.00	0.84	0.85	0.76	0.85	0.77	0.78	0.77	0.83	28.01	6.33
Q8BW75	Amine oxidase [flavin-containing] B OS=Mus musculus GN=Maob PE=1 SV=4 - [AOFB_MOUSE]	37.88	3	14	15	38	0.97	0.98	1.01	1.06	1.08	0.78	0.79	0.76	0.74	0.85	0.86	0.89	0.94	58.52	8.29
A7E1Z5	Electrogenic Na-bicarbonate cotransporter splice variant NBCe1-E OS=Mus musculus GN=Slc4a4 PE=2 SV=1 - [A7E1Z5_MOUSE]	19.72	7	1	12	38	0.85	1.10	1.29	1.25	1.46	1.00	1.16	0.76	0.88	0.96	1.12	0.78	0.92	120.39	6.84
A2ASZ8-4	Isoform 4 of Calcium-binding mitochondrial carrier protein SCaMC-2 OS=Mus musculus GN=Slc25a25 - [SCMC2_MOUSE]	11.04	6	5	5	8	1.10	1.12	1.04	1.16	1.13	0.77	0.66	0.76	0.75	0.89	0.73	0.94	0.80	54.56	8.63
Q8BU30	Isoleucine--tRNA ligase, cytoplasmic OS=Mus musculus GN=Iars PE=2 SV=2 - [SYIC_MOUSE]	10.22	2	11	11	19	0.91	0.93	1.09	0.99	1.15	0.84	1.02	0.76	0.95	0.86	1.05	0.86	0.99	144.18	6.55

Q69Z99	Zinc finger protein 512 OS=Mus musculus GN=Znf512 PE=2 SV=2 - [ZN512_MOUSE]	5.69	1	2	2	6	0.81	1.07	1.15	1.08	1.14	0.75	0.84	0.76	0.88	0.89	0.94	0.84	0.89	63.87	9.51
Q9ERI6	Retinol dehydrogenase 14 OS=Mus musculus GN=Rdh14 PE=1 SV=1 - [RDH14_MOUSE]	29.64	1	7	7	13	0.95	1.08	1.13	1.11	1.16	0.88	0.96	0.76	0.88	0.85	0.98	0.99	0.99	36.34	8.18
Q8JZK9	Hydroxymethylglutaryl-CoA synthase, cytoplasmic OS=Mus musculus GN=Hmgcs1 PE=1 SV=1 - [HMCS1_MOUSE]	19.04	2	7	7	19	0.93	0.88	0.95	0.94	1.01	0.86	0.93	0.76	0.82	0.89	0.93	0.85	0.95	57.53	5.99
Q64310	Surfeit locus protein 4 OS=Mus musculus GN=Surf4 PE=2 SV=1 - [SURF4_MOUSE]	3.72	1	1	1	4	1.02	1.31	1.29	1.04	1.02	0.72	0.71	0.76	0.74	0.77	0.76	0.93	0.92	30.36	7.78
Q69ZK0	Phosphatidylinositol 3,4,5-trisphosphate-dependent Rac exchanger 1 protein OS=Mus musculus GN=Prex1 PE=1 SV=2 - [PREX1_MOUSE]	3.33	3	5	5	6	0.90	1.06	1.17	0.95	1.06	0.81	0.87	0.76	0.80	0.83	0.95	0.81	0.97	184.82	6.29
H3BJ97	Tubulointerstitial nephritis antigen-like OS=Mus musculus GN=Tinagl1 PE=2 SV=1 - [H3BJ97_MOUSE]	19.31	3	5	5	7	0.94	1.14	1.21	1.01	1.07	0.72	0.79	0.76	0.80	0.90	0.90	0.87	0.87	49.24	7.15
Q61024	Asparagine synthetase [glutamine-hydrolyzing] OS=Mus musculus GN=Asns PE=2 SV=3 - [ASNS_MOUSE]	14.44	2	5	5	9	0.86	0.79	0.91	1.09	1.25	0.84	0.95	0.76	0.88	0.92	1.02	0.97	1.09	64.24	6.58
Q3UN70	Myelin regulatory factor-like protein OS=Mus musculus GN=Myrfl PE=2 SV=1 - [MRFL_MOUSE]	1.77	1	1	1	1	1.42	1.27	0.89	1.31	0.92	1.23	0.86	0.76	0.53	1.19	0.84	0.99	0.70	101.62	7.27
P63328	Serine/threonine-protein phosphatase 2B catalytic subunit alpha isoform OS=Mus musculus GN=Ppp3ca PE=1 SV=1 - [PP2BA_MOUSE]	51.44	2	1	24	364	0.97	0.99	0.94	0.90	0.93	0.78	0.74	0.76	0.70	0.76	0.71	0.81	0.81	58.61	5.86
E9QAD4	Coiled-coil domain-containing protein 93 OS=Mus musculus GN=Ccdc93 PE=2 SV=1 - [E9QAD4_MOUSE]	3.66	2	2	2	3	0.90	0.84	0.93	1.02	1.13	0.90	0.99	0.76	0.84	0.78	0.86	1.04	1.15	72.43	8.29
P23953	Carboxylesterase 1C OS=Mus musculus GN=Ces1c PE=1 SV=4 - [EST1C_MOUSE]	9.39	2	3	3	5	0.81	1.11	1.37	0.71	0.87	0.68	0.84	0.76	0.94	1.07	1.32	0.69	0.86	61.02	5.06

Q8VI63-2	Isoform 2 of MOB kinase activator 2 OS=Mus musculus GN=Mob2 - [MOB2_MOUSE]	7.33	2	1	1	2	0.97	0.89	0.91	1.09	1.11	0.90	0.92	0.76	0.78	0.89	0.91	0.87	0.90	16.98	6.62
J3QMC5	Midasin OS=Mus musculus GN=Mdn1 PE=3 SV=1 - [J3QMC5_MOUSE]	0.25	2	1	1	1	0.85	0.59	0.70	0.93	1.09	0.79	0.93	0.76	0.89	0.81	0.96	0.67	0.79	629.19	5.73
P63328-2	Isoform 2 of Serine/threonine-protein phosphatase 2B catalytic subunit alpha isoform OS=Mus musculus GN=Ppp3ca - [PP2BA_MOUSE]	50.49	2	1	24	331	1.02	1.17	1.16	0.93	0.94	0.80	0.81	0.76	0.74	0.77	0.76	0.82	0.80	57.58	6.30
Q64133	Amine oxidase [flavin-containing] A OS=Mus musculus GN=Maoa PE=1 SV=3 - [AOFA_MOUSE]	25.29	1	10	11	43	1.06	1.05	0.95	1.00	0.95	0.79	0.71	0.76	0.71	0.85	0.80	0.92	0.86	59.56	7.81
Q9QWR8	Alpha-N-acetylgalactosaminidase OS=Mus musculus GN=Naga PE=2 SV=2 - [NAGAB_MOUSE]	2.65	1	1	1	1	0.87	0.88	1.02	0.94	1.08	0.80	0.92	0.76	0.87	0.97	1.12	0.99	1.14	47.20	6.44
P54731	FAS-associated factor 1 OS=Mus musculus GN=Faf1 PE=1 SV=2 - [FAF1_MOUSE]	9.40	1	3	3	6	1.13	1.19	0.96	1.16	1.03	1.05	0.94	0.76	0.67	1.01	0.96	0.95	0.85	73.82	4.86
Q80TJ1-4	Isoform 4 of Calcium-dependent secretion activator 1 OS=Mus musculus GN=Cadps - [CAPS1_MOUSE]	30.35	4	2	26	86	1.07	0.84	0.79	1.07	0.96	0.88	0.86	0.76	0.77	0.88	0.81	0.95	0.89	152.57	5.74
P03930	ATP synthase protein 8 OS=Mus musculus GN=Mtatp8 PE=1 SV=1 - [ATP8_MOUSE]	23.88	1	2	2	2	0.81	0.91	1.12	0.99	1.22	0.74	0.91	0.76	0.94	0.78	0.97	0.83	1.04	7.76	9.88
A2ADR8	Nuclear inhibitor of protein phosphatase 1 OS=Mus musculus GN=Ppp1r8 PE=2 SV=1 - [A2ADR8_MOUSE]	34.57	2	7	7	15	0.89	0.86	0.96	0.87	1.01	0.77	0.87	0.76	0.87	0.72	0.87	0.75	0.88	38.38	7.15
Q8BH10	Protein orai-2 OS=Mus musculus GN=Orai2 PE=2 SV=1 - [ORAI2_MOUSE]	2.80	1	1	1	1	1.00	0.93	0.92	1.07	1.07	0.86	0.85	0.76	0.76	0.80	0.80	0.77	0.77	28.18	7.94
Q9D0M1	Phosphoribosyl pyrophosphate synthase-associated protein 1 OS=Mus musculus GN=Prpsap1 PE=1 SV=1 - [KPRA_MOUSE]	22.19	4	2	5	10	0.91	0.80	0.88	0.92	1.01	0.85	0.93	0.76	0.83	0.78	0.86	0.87	0.95	39.41	7.20
Q3URS9-	Isoform 2 of Coiled-coil domain-containing protein 51 OS=Mus musculus GN=Ccdc51 - [CCD51_MOUSE]	5.87	2	2	2	6	1.24	1.31	0.99	1.37	1.11	0.81	0.65	0.76	0.62	1.03	0.80	0.90	0.81	41.74	7.56

E9Q1V2	Copine-5 (Fragment) OS=Mus musculus GN=Cpne5 PE=2 SV=1 - [E9Q1V2_MOUSE]	50.47	2	2	8	18	0.84	1.20	1.43	1.10	1.32	0.79	0.86	0.76	0.92	0.86	0.97	0.86	0.93	23.69	5.11
D3YWG2	Chitinase domain-containing protein 1 (Fragment) OS=Mus musculus GN=Chid1 PE=2 SV=1 - [D3YWG2_MOUSE]	17.49	4	4	4	7	1.04	1.15	1.05	0.97	0.94	0.92	0.91	0.76	0.80	0.88	0.81	0.82	0.83	29.99	6.96
Q91XB7	Protein YIF1A OS=Mus musculus GN=Yif1a PE=2 SV=1 - [YIF1A_MOUSE]	10.24	1	1	1	1	2.04	0.79	0.38	0.83	0.40	0.89	0.43	0.76	0.37	0.71	0.35	0.71	0.35	32.11	9.09
Q3TH73	Protein tweety homolog 2 OS=Mus musculus GN=Ttyh2 PE=2 SV=1 - [TTYH2_MOUSE]	4.89	2	1	2	8	1.31	1.27	0.96	1.33	1.01	0.96	0.73	0.76	0.58	1.01	0.77	1.22	0.93	58.97	6.07
G5E8T9	Hydroxyacyl glutathione hydrolase OS=Mus musculus GN=Hagh PE=3 SV=1 - [G5E8T9_MOUSE]	36.89	8	9	9	46	0.93	0.81	0.82	0.82	0.89	0.65	0.69	0.76	0.83	0.65	0.69	0.68	0.72	34.08	7.94
P70451-4	Isoform 4 of Tyrosine-protein kinase Fer OS=Mus musculus GN=Fer - [FER_MOUSE]	2.65	4	1	1	2	0.89	0.78	0.87	0.90	1.01	0.76	0.85	0.76	0.85	0.73	0.82	0.81	0.90	51.57	8.32
Q61838	Alpha-2-macroglobulin OS=Mus musculus GN=A2m PE=1 SV=3 - [A2M_MOUSE]	3.48	3	4	4	10	0.82	1.00	1.27	0.52	0.66	0.68	0.85	0.76	0.96	0.96	1.08	0.58	0.77	165.75	6.68
Q5SUC9	Protein SCO1 homolog, mitochondrial OS=Mus musculus GN=Sco1 PE=2 SV=1 - [SCO1_MOUSE]	13.03	2	3	3	5	0.90	0.80	0.88	0.94	1.05	0.82	0.93	0.76	1.14	0.76	0.98	0.65	0.73	31.60	8.47
P70207	Plexin-A2 OS=Mus musculus GN=Plxna2 PE=1 SV=2 - [PLXA2_MOUSE]	10.67	1	9	17	30	1.03	1.07	1.03	1.00	1.01	0.78	0.79	0.76	0.80	0.83	0.83	0.93	1.04	211.40	6.54
P24529	Tyrosine 3-monooxygenase OS=Mus musculus GN=Th PE=1 SV=3 - [TY3H_MOUSE]	14.46	4	5	5	10	1.00	1.16	1.13	1.44	1.51	0.99	0.99	0.76	0.75	1.40	1.19	1.81	1.71	55.96	6.15
Q8R1B4	Eukaryotic translation initiation factor 3 subunit C OS=Mus musculus GN=Eif3c PE=1 SV=1 - [EIF3C_MOUSE]	19.21	1	14	14	38	1.05	0.92	0.83	1.07	0.98	0.79	0.77	0.76	0.75	0.83	0.81	0.83	0.85	105.46	5.78
Q91YS8	Calcium/calmodulin-dependent protein kinase type 1 OS=Mus musculus GN=Camk1 PE=1 SV=1 - [KCC1A_MOUSE]	31.02	2	6	9	29	1.01	0.73	0.74	0.89	0.99	0.78	0.74	0.76	0.75	0.73	0.72	0.68	0.75	41.60	5.35

Q91V09	WD repeat-containing protein 13 OS=Mus musculus GN=Wdr13 PE=1 SV=1 - [WDR13_MOUSE]	26.60	2	9	9	27	0.99	1.09	1.05	0.92	0.91	0.69	0.66	0.76	0.73	0.71	0.72	0.69	0.70	53.63	9.14
Q6KAU4	Multivesicular body subunit 12B OS=Mus musculus GN=Mvb12b PE=2 SV=2 - [MB12B_MOUSE]	3.47	1	1	1	4	0.86	0.94	1.09	0.91	1.06	0.86	1.00	0.76	0.89	0.84	0.99	0.95	1.11	35.39	7.93
Q80ZJ6-2	Isoform 2 of Protein zer-1 homolog OS=Mus musculus GN=Zer1 - [ZER1_MOUSE]	4.57	3	3	3	3	0.78	0.83	1.07	0.89	1.12	0.92	0.97	0.76	0.81	0.82	1.04	0.89	1.02	87.68	5.80
O88507	Ciliary neurotrophic factor receptor subunit alpha OS=Mus musculus GN=Cntfr PE=1 SV=2 - [CNTFR_MOUSE]	19.09	1	5	5	21	1.13	0.98	0.81	1.00	0.84	0.75	0.72	0.77	0.71	0.75	0.61	0.93	0.83	40.78	6.83
O08599	Syntaxin-binding protein 1 OS=Mus musculus GN=Stxbp1 PE=1 SV=2 - [STXB1_MOUSE]	76.26	1	3	45	493	0.80	0.77	0.79	1.04	1.31	0.94	1.20	0.77	0.87	0.75	0.95	1.24	1.17	67.53	6.96
H7BXB9	Protein 1700037H04Rik (Fragment) OS=Mus musculus GN=1700037H04Rik PE=2 SV=1 - [H7BXB9_MOUSE]	9.93	3	1	1	4	1.00	0.78	0.78	0.89	0.89	0.80	0.79	0.77	0.77	0.83	0.83	1.16	1.17	15.76	8.32
Q3UN10	Wolframin OS=Mus musculus GN=Wfs1 PE=2 SV=1 - [Q3UN10_MOUSE]	5.53	3	4	4	14	1.13	0.93	0.80	1.14	0.94	0.86	0.85	0.77	0.81	1.02	0.96	1.14	1.07	91.76	7.99
B1AY64	MCG1034295, isoform CRA_a OS=Mus musculus GN=Cypt3 PE=4 SV=1 - [B1AY64_MOUSE]	3.45	1	1	1	1	1.08	0.81	0.75	0.94	0.87	0.93	0.85	0.77	0.71	1.05	0.97	0.67	0.62	19.51	10.56
Q8BFQ4	WD repeat-containing protein 82 OS=Mus musculus GN=Wdr82 PE=1 SV=1 - [WDR82_MOUSE]	6.39	1	1	1	1	1.06	0.95	0.90	0.94	0.89	0.60	0.56	0.77	0.72	0.73	0.69	0.50	0.47	35.06	7.69
P53994	Ras-related protein Rab-2A OS=Mus musculus GN=Rab2a PE=1 SV=1 - [RAB2A_MOUSE]	71.23	3	7	14	95	1.01	0.94	0.93	0.92	0.99	0.82	0.80	0.77	0.77	0.80	0.81	0.79	0.82	23.53	6.54
H7BX88	Carnitine O-acetyltransferase OS=Mus musculus GN=Crat PE=2 SV=1 - [H7BX88_MOUSE]	7.60	4	4	4	8	0.96	0.85	0.86	0.86	0.90	0.83	0.79	0.77	0.78	0.79	0.81	0.88	0.89	68.60	8.07
Q62056	MCG130054 OS=Mus musculus GN=Psg17 PE=2 SV=1 - [Q62056_MOUSE]	3.58	1	1	1	1	0.95	0.86	0.90	1.01	1.06	0.79	0.83	0.77	0.80	0.85	0.89	0.82	0.86	52.88	7.44

P14148	60S ribosomal protein L7 OS=Mus musculus GN=Rpl7 PE=2 SV=2 - [RL7_MOUSE]	40.37	2	11	11	21	1.02	0.83	0.76	1.00	1.02	0.97	0.93	0.77	0.77	0.89	0.86	0.98	0.96	31.40	10.89
Q9JLJ2	4-trimethylaminobutyraldehyde dehydrogenase OS=Mus musculus GN=Aldh9a1 PE=1 SV=1 - [AL9A1_MOUSE]	27.33	2	11	11	20	1.03	0.88	0.84	1.08	1.02	0.89	0.85	0.77	0.70	0.89	0.87	0.89	0.89	53.48	6.98
Q9CWR0-	Isoform 2 of Rho guanine nucleotide exchange factor 25 OS=Mus musculus GN=Arhgef25 - [ARHGP_MOUSE]	3.45	3	1	1	1	1.08	3.47	3.20	1.10	1.01	1.09	1.00	0.77	0.71	0.79	0.73	0.89	0.82	67.41	5.19
Q8BGA3	Leucine-rich repeat transmembrane neuronal protein 2 OS=Mus musculus GN=Lrrtm2 PE=2 SV=1 - [LRRT2_MOUSE]	5.24	1	3	3	5	1.06	0.84	0.78	1.04	1.11	0.78	0.73	0.77	0.72	0.80	0.76	0.81	0.85	58.78	8.00
Q3TJ22	Angio-associated migratory protein OS=Mus musculus GN=Aamp PE=2 SV=1 - [Q3TJ22_MOUSE]	4.14	2	2	2	3	0.99	1.00	1.02	0.93	0.94	0.78	0.79	0.77	0.78	0.77	0.78	0.79	0.80	46.87	4.41
P26048	Gamma-aminobutyric acid receptor subunit alpha-2 OS=Mus musculus GN=Gabra2 PE=1 SV=1 - [GBRA2_MOUSE]	10.86	2	2	5	10	1.25	1.16	0.93	0.96	0.77	0.89	0.71	0.77	0.61	0.89	0.71	0.92	0.74	51.11	9.06
P29341	Polyadenylate-binding protein 1 OS=Mus musculus GN=Pabpc1 PE=1 SV=2 - [PABP1_MOUSE]	39.31	4	15	23	62	0.94	0.79	0.84	0.87	0.97	0.77	0.82	0.77	0.83	0.75	0.87	0.82	0.85	70.63	9.50
P56387	Dynein light chain Tctex-type 3 OS=Mus musculus GN=Dynlt3 PE=1 SV=1 - [DYLT3_MOUSE]	8.62	1	1	1	4	1.14	1.24	1.09	1.10	0.97	0.64	0.56	0.77	0.67	0.70	0.61	0.89	0.79	12.95	5.12
Q9QYG0-	Isoform 2 of Protein NDRG2 OS=Mus musculus GN=Ndrg2 - [NDRG2_MOUSE]	67.79	1	1	14	119	0.79	0.87	1.10	1.04	1.32	0.75	0.94	0.77	0.97	0.94	1.19	0.99	1.26	39.26	5.63
Q9D1C8	Vacuolar protein sorting-associated protein 28 homolog OS=Mus musculus GN=Vps28 PE=2 SV=1 - [VPS28_MOUSE]	50.68	1	8	8	15	0.95	1.07	1.14	1.03	1.04	0.76	0.83	0.77	0.81	0.75	0.83	0.87	0.86	25.44	5.54

B1B0C7	Basement membrane-specific heparan sulfate proteoglycan core protein OS=Mus musculus GN=Hspg2 PE=2 SV=1 - [B1B0C7_MOUSE]	10.51	3	31	31	57	0.86	1.04	1.17	0.89	1.02	0.73	0.77	0.77	0.87	0.80	0.87	0.75	0.83	468.73	6.48
Q9DBX3	Sushi domain-containing protein 2 OS=Mus musculus GN=Susd2 PE=1 SV=1 - [SUSD2_MOUSE]	6.34	3	4	4	6	1.10	1.10	1.01	1.02	0.91	0.81	0.74	0.77	0.70	0.95	0.87	0.90	0.82	90.58	6.62
Q61503	5'-nucleotidase OS=Mus musculus GN=Nt5e PE=1 SV=2 - [5NTD_MOUSE]	11.46	1	5	5	9	1.05	0.98	0.80	1.11	1.02	0.87	0.86	0.77	0.73	0.91	0.86	1.04	0.99	63.82	6.64
D3YVB6	Ketohexokinase (Fragment) OS=Mus musculus GN=Khk PE=2 SV=1 - [D3YVB6_MOUSE]	3.28	1	1	1	1	0.96	1.18	1.23	0.97	1.02	0.80	0.83	0.77	0.80	0.87	0.91	1.10	1.16	27.19	6.23
Q9D1R9	60S ribosomal protein L34 OS=Mus musculus GN=Rpl34 PE=3 SV=2 - [RL34_MOUSE]	15.38	2	2	2	6	0.96	0.82	0.86	1.11	1.10	0.87	0.90	0.77	0.77	0.89	0.90	0.92	0.91	13.28	11.47
Q99KK7	Dipeptidyl peptidase 3 OS=Mus musculus GN=Dpp3 PE=2 SV=2 - [DPP3_MOUSE]	24.12	1	11	11	18	0.98	0.88	0.93	1.01	1.03	0.86	0.83	0.77	0.77	0.86	0.77	0.91	0.94	82.85	5.38
P56376	Acylphosphatase-1 OS=Mus musculus GN=Acyp1 PE=2 SV=2 - [ACYP1_MOUSE]	66.67	2	9	9	18	0.97	0.76	0.75	0.88	0.95	0.72	0.75	0.77	0.78	0.63	0.74	0.63	0.74	11.23	9.04
Q6P6M7	O-phosphoseryl-tRNA(Sec) selenium transferase OS=Mus musculus GN=Sepsecs PE=1 SV=2 - [SPCS_MOUSE]	7.74	4	3	3	7	1.07	0.88	0.72	0.96	0.90	0.88	0.89	0.77	0.67	0.83	0.68	0.92	0.75	55.29	8.06
Q8R033	LYR motif-containing protein 2 OS=Mus musculus GN=Lyrm2 PE=2 SV=1 - [LYRM2_MOUSE]	10.23	1	1	1	2	1.00	1.28	1.29	0.87	0.87	0.74	0.74	0.77	0.77	0.72	0.73	0.73	0.73	10.37	10.40
Q8R016	Bleomycin hydrolase OS=Mus musculus GN=Blmh PE=2 SV=1 - [BLMH_MOUSE]	23.30	5	8	8	17	1.00	0.93	0.95	1.02	0.98	0.90	0.87	0.77	0.78	0.91	0.78	1.03	0.91	52.48	6.48
Q5SYL3	UPF0378 protein KIAA0100 OS=Mus musculus GN=Kiaa0100 PE=2 SV=1 - [K0100_MOUSE]	0.54	1	1	1	1	0.90	1.18	1.31	0.83	0.93	0.75	0.84	0.77	0.86	0.82	0.91	0.94	1.05	254.31	7.34
Q9QYB5-	Isoform 1 of Gamma-adducin OS=Mus musculus GN=Add3 - [ADDG_MOUSE]	36.94	1	1	19	166	0.86	0.55	0.64	0.86	1.00	0.78	0.90	0.77	0.89	0.82	0.96	0.68	0.79	75.36	6.34

Q9D0R2	Threonine--tRNA ligase, cytoplasmic OS=Mus musculus GN=Tars PE=1 SV=2 - [SYTC_MOUSE]	20.50	1	12	12	28	1.00	0.92	0.91	1.01	1.01	0.82	0.80	0.77	0.75	0.82	0.82	0.86	0.82	83.30	7.36
Q9ET30	Transmembrane 9 superfamily member 3 OS=Mus musculus GN=Tm9sf3 PE=1 SV=1 - [TM9S3_MOUSE]	3.58	1	2	2	3	1.04	1.01	0.96	1.27	1.21	0.91	0.86	0.77	0.74	0.89	0.85	0.89	0.85	67.50	7.21
P61027	Ras-related protein Rab 10 OS=Mus musculus GN=Rab10 PE=1 SV=1 - [RAB10_MOUSE]	40.00	11	5	8	72	1.02	1.03	1.07	1.01	0.98	0.85	0.84	0.77	0.75	0.80	0.83	0.89	0.88	22.53	8.38
Q9D4H1	Exocyst complex component 2 OS=Mus musculus GN=Exoc2 PE=1 SV=1 - [EXOC2_MOUSE]	7.90	1	6	6	12	0.98	1.20	1.25	1.13	1.15	0.84	0.88	0.77	0.82	0.90	0.90	1.00	0.99	103.89	7.18
P10493	Nidogen-1 OS=Mus musculus GN=Nid1 PE=1 SV=2 - [NID1_MOUSE]	10.12	1	11	11	19	0.93	0.96	0.98	0.89	0.99	0.74	0.86	0.77	0.84	0.81	0.84	0.72	0.76	136.45	5.44
Q3TFD2-3	Isoform 3 of Lysophosphatidylcholine acyltransferase 1 OS=Mus musculus GN=Lpcat1 - [PCAT1_MOUSE]	3.63	3	1	1	1	1.23	1.26	1.02	1.40	1.13	0.95	0.77	0.77	0.63	1.02	0.83	1.26	1.02	36.96	4.65
P29758	Ornithine aminotransferase, mitochondrial OS=Mus musculus GN=Oat PE=1 SV=1 - [OAT_MOUSE]	49.89	1	15	15	40	1.10	0.97	0.92	1.05	0.97	0.73	0.67	0.77	0.73	0.79	0.76	0.77	0.75	48.32	6.62
Q6P5F9	Exportin-1 OS=Mus musculus GN=Xpo1 PE=1 SV=1 - [XPO1_MOUSE]	11.02	3	8	8	16	0.96	0.89	0.94	1.08	1.09	0.90	0.89	0.77	0.78	0.91	0.93	1.03	1.02	123.01	6.07
D3Z041	Long-chain-fatty-acid--CoA ligase 1 OS=Mus musculus GN=Acsl1 PE=2 SV=1 - [D3Z041_MOUSE]	11.73	4	6	7	13	0.93	0.84	0.98	1.07	1.09	0.83	0.84	0.78	0.79	0.87	0.92	0.88	0.92	77.98	7.47
Q8VE62	Polyadenylate-binding protein-interacting protein 1 OS=Mus musculus GN=Paip1 PE=2 SV=1 - [PAIP1_MOUSE]	20.50	5	1	8	19	0.97	0.75	0.78	0.96	0.99	0.91	0.93	0.78	0.80	0.80	0.83	0.84	0.87	45.67	4.55
Q62351	Transferrin receptor protein 1 OS=Mus musculus GN=Tfrc PE=1 SV=1 - [TFR1_MOUSE]	18.09	2	11	11	20	1.00	1.16	1.06	0.98	0.95	0.78	0.90	0.78	0.76	0.85	0.93	0.87	0.86	85.68	6.57
Q80TQ2	Ubiquitin carboxyl-terminal hydrolase CYLD OS=Mus musculus GN=Cyld PE=1 SV=2 - [CYLD_MOUSE]	7.35	3	6	6	10	0.95	0.89	0.89	1.07	1.21	0.89	0.95	0.78	0.83	0.93	0.99	1.04	1.11	106.52	5.63

O70152	Dolichol-phosphate mannosyltransferase OS=Mus musculus GN=Dpm1 PE=2 SV=1 - [DPM1_MOUSE]	14.23	3	3	3	6	0.85	1.07	1.44	1.03	1.18	0.83	1.05	0.78	0.96	0.85	1.10	0.96	1.14	29.16	9.51
P00397	Cytochrome c oxidase subunit 1 OS=Mus musculus GN=Mtco1 PE=3 SV=1 - [COX1_MOUSE]	6.23	2	1	1	2	1.28	0.94	0.73	1.51	1.18	1.02	0.80	0.78	0.61	0.88	0.69	1.28	1.00	56.82	6.70
Q9D7X3	Dual specificity protein phosphatase 3 OS=Mus musculus GN=Dusp3 PE=1 SV=1 - [DUS3_MOUSE]	42.16	5	6	6	36	0.85	0.96	1.16	1.00	1.20	0.75	0.89	0.78	0.89	0.71	0.83	0.72	0.87	20.46	6.54
Q9Z2X2	26S proteasome non-ATPase regulatory subunit 10 OS=Mus musculus GN=Psmd10 PE=1 SV=3 - [PSD10_MOUSE]	7.79	1	2	2	3	1.06	1.11	1.04	0.99	0.93	0.72	0.67	0.78	0.73	0.76	0.72	0.92	0.87	25.07	6.06
Q922S4	cGMP-dependent 3',5'-cyclic phosphodiesterase OS=Mus musculus GN=Pde2a PE=1 SV=3 - [PDE2A_MOUSE]	31.11	3	18	18	45	1.02	1.04	1.01	1.22	1.12	0.86	0.81	0.78	0.77	1.00	0.99	1.08	1.03	103.18	5.38
O35683	NADH dehydrogenase [ubiquinone] 1 alpha subcomplex subunit 1 OS=Mus musculus GN=Ndufa1 PE=2 SV=1 - [NDUA1_MOUSE]	14.29	1	1	1	2	0.95	1.16	1.23	0.96	1.01	0.65	0.68	0.78	0.82	0.79	0.84	0.89	0.94	8.13	9.48
Q64516-1	Isoform 1 of Glycerol kinase OS=Mus musculus GN=Gk - [GLPK_MOUSE]	9.54	5	5	5	11	1.05	1.07	1.08	1.18	1.13	0.83	0.81	0.78	0.78	0.80	0.83	0.89	0.89	57.42	5.64
Q8R4E6	Purine-rich element-binding protein gamma OS=Mus musculus GN=Purg PE=1 SV=1 - [PURG_MOUSE]	18.00	2	4	7	10	0.87	0.97	0.94	0.97	1.11	0.82	0.79	0.78	0.90	0.84	0.88	0.83	0.90	39.91	9.51
Q5NCF2	Trafficking protein particle complex subunit 1 OS=Mus musculus GN=Trappc1 PE=1 SV=1 - [TPPC1_MOUSE]	12.41	3	2	2	2	1.21	1.44	1.19	0.98	0.81	0.82	0.67	0.78	0.64	0.86	0.72	0.88	0.73	16.87	9.16
Q8C7D2-2	Isoform 2 of Protein cereblon OS=Mus musculus GN=Crbn - [CRBN_MOUSE]	9.05	3	2	2	4	1.01	0.90	0.89	1.07	1.06	0.97	0.96	0.78	0.77	0.95	0.95	0.99	0.98	49.45	5.45
Q91VT4	Carbonyl reductase family member 4 OS=Mus musculus GN=Cbr4 PE=2 SV=2 - [CBR4_MOUSE]	9.75	2	2	2	4	0.98	1.06	1.05	1.10	1.11	0.72	0.68	0.78	0.81	0.79	0.80	0.88	0.90	25.40	9.69
E9Q171	Neurofascin OS=Mus musculus GN=Nfasc PE=2 SV=1 - [E9Q171_MOUSE]	42.52	2	4	38	174	0.98	1.20	1.22	0.96	0.95	0.78	0.75	0.78	0.82	0.82	0.83	0.77	0.80	130.16	6.84

A2AQ17	Complex I intermediate-associated protein 30, mitochondrial OS=Mus musculus GN=Ndufaf1 PE=4 SV=1 - [A2AQ17_MOUSE]	6.71	2	2	2	4	0.88	0.86	0.98	0.99	1.13	0.90	1.02	0.78	0.88	0.81	0.93	0.85	0.97	37.76	8.60
Q8BW41-	Isoform 2 of Glycosyltransferase-like domain-containing protein 2 OS=Mus musculus GN=Gtdc2 - [GTDC2_MOUSE]	1.72	2	1	1	2	1.01	1.31	1.29	1.09	1.07	0.84	0.82	0.78	0.77	0.92	0.91	1.00	0.99	66.62	8.47
Q99KI3	ER membrane protein complex subunit 3 OS=Mus musculus GN=Emc3 PE=2 SV=3 - [EMC3_MOUSE]	15.71	1	3	3	9	1.08	1.02	0.98	1.25	1.19	0.85	0.87	0.78	0.78	0.88	0.90	1.14	1.09	29.96	6.81
P58281	Dynamin-like 120 kDa protein, mitochondrial OS=Mus musculus GN=Opa1 PE=1 SV=1 - [OPA1_MOUSE]	40.00	5	33	33	91	0.97	1.09	1.12	1.23	1.27	0.79	0.78	0.78	0.78	0.84	0.89	0.92	0.91	111.27	7.55
Q7TNB5	Glutamate receptor 1 OS=Mus musculus GN=Gria1 PE=2 SV=1 - [Q7TNB5_MOUSE]	20.73	1	1	15	34	0.97	0.88	0.90	1.14	1.17	0.76	0.77	0.78	0.80	0.79	0.81	0.80	0.82	101.53	7.53
Q8BIJ6	Isoleucine--tRNA ligase, mitochondrial OS=Mus musculus GN=Iars2 PE=2 SV=1 - [SYIM_MOUSE]	24.51	3	18	18	42	1.00	0.89	0.82	1.10	1.09	0.86	0.84	0.78	0.76	0.83	0.79	0.88	0.83	112.73	6.81
Q8VCL2	Protein SCO2 homolog, mitochondrial OS=Mus musculus GN=Sco2 PE=2 SV=1 - [SCO2_MOUSE]	15.69	1	2	2	3	1.04	0.96	0.92	1.08	1.03	0.83	0.79	0.78	0.75	0.72	0.70	0.84	0.81	28.93	8.29
Q9JLI6	Selenocysteine lyase OS=Mus musculus GN=Scly PE=1 SV=1 - [SCLY_MOUSE]	10.42	7	4	4	7	0.93	1.04	1.07	0.93	1.01	0.76	0.81	0.78	0.84	0.72	0.78	0.78	0.84	47.14	6.80
O35435	Dihydroorotate dehydrogenase (quinone), mitochondrial OS=Mus musculus GN=Dhodh PE=2 SV=2 - [PYRD_MOUSE]	18.23	4	6	6	9	0.99	1.25	1.19	0.90	0.96	0.82	0.89	0.78	0.79	0.78	0.85	0.85	0.88	42.67	9.55
Q5Y5T1-2	Isoform 2 of Probable palmitoyltransferase ZDHHC20 OS=Mus musculus GN=Zdhhc20 - [ZDH20_MOUSE]	2.99	2	1	1	2	0.96	0.83	0.87	1.00	1.04	0.88	0.92	0.78	0.81	0.88	0.92	0.85	0.89	42.53	8.00
A2AMZ4	Uncharacterized protein C17orf89 homolog OS=Mus musculus PE=4 SV=1 - [CQ089_MOUSE]	24.32	1	1	1	1	0.69	1.08	1.55	0.78	1.12	0.87	1.25	0.78	1.12	0.69	1.00	0.88	1.27	7.78	9.55

H7BX36	Serine/threonine-protein kinase DCLK1 OS=Mus musculus GN=Dclk1 PE=2 SV=1 - [H7BX36_MOUSE]	35.55	1	3	10	52	0.93	1.00	1.07	0.77	0.82	0.80	0.85	0.78	0.83	0.67	0.72	0.83	0.89	46.57	5.85
Q9Z2I0	LETM1 and EF-hand domain-containing protein 1, mitochondrial OS=Mus musculus GN=Letm1 PE=2 SV=1 - [LETM1_MOUSE]	35.64	1	20	20	48	1.05	1.02	1.00	1.12	1.09	0.85	0.77	0.78	0.74	0.80	0.78	0.93	0.91	82.94	6.52
E9QAS4	Chromodomain-helicase-DNA-binding protein 4 OS=Mus musculus GN=Chd4 PE=2 SV=1 - [E9QAS4_MOUSE]	1.52	7	2	2	3	0.77	0.85	1.09	0.87	1.12	0.72	0.93	0.78	1.00	0.60	0.78	0.70	0.91	216.23	5.92
E9Q150	Segment polarity protein dishevelled homolog DVL-3 OS=Mus musculus GN=Dvl3 PE=2 SV=1 - [E9Q150_MOUSE]	2.72	3	1	2	2	0.66	1.09	1.65	0.83	1.26	0.71	1.06	0.78	1.18	0.75	1.14	0.97	1.47	76.28	6.70
Q80YA8	Protein crumbs homolog 2 OS=Mus musculus GN=Crb2 PE=2 SV=3 - [CRUM2_MOUSE]	1.25	1	1	1	1	0.63	0.75	1.20	0.58	0.92	1.11	1.75	0.78	1.24	0.84	1.33	0.86	1.37	134.67	5.22
Q921M3	Splicing factor 3B subunit 3 OS=Mus musculus GN=Sf3b3 PE=2 SV=1 - [SF3B3_MOUSE]	23.01	2	25	25	59	1.04	1.02	0.99	0.96	0.92	0.79	0.76	0.78	0.77	0.76	0.72	0.80	0.77	135.46	5.26
O35129	Prohibitin-2 OS=Mus musculus GN=Phb2 PE=1 SV=1 - [PHB2_MOUSE]	47.83	3	13	13	38	1.05	1.20	1.15	1.17	1.12	0.78	0.76	0.78	0.70	0.84	0.80	0.88	0.85	33.28	9.83
Q9DBS1	Transmembrane protein 43 OS=Mus musculus GN=Tmem43 PE=1 SV=1 - [TMM43_MOUSE]	10.00	1	4	4	10	0.95	1.20	1.26	1.02	1.08	0.80	0.77	0.78	0.84	0.94	0.98	1.04	1.03	44.76	7.36
Q7M6W1	RTN1-C OS=Mus musculus GN=Rtn1 PE=2 SV=1 - [Q7M6W1_MOUSE]	27.40	2	2	5	26	1.03	0.98	0.87	1.06	0.96	0.89	0.79	0.78	0.77	0.94	0.95	1.01	0.91	23.54	8.91
Q9D662	Protein transport protein Sec23B OS=Mus musculus GN=Sec23b PE=2 SV=1 - [SC23B_MOUSE]	5.22	5	1	4	7	0.85	0.83	0.97	1.00	1.18	0.86	1.00	0.78	0.92	0.87	1.03	0.82	0.97	86.38	6.96
Q3UH45	Protein Tecpr2 OS=Mus musculus GN=Tecpr2 PE=2 SV=1 - [Q3UH45_MOUSE]	1.41	1	2	2	3	0.75	0.98	1.30	0.84	1.12	0.82	1.10	0.78	1.04	0.73	0.99	1.06	1.11	154.84	5.38

H3BKC5	U6 snRNA-associated Sm-like protein LSm5 (Fragment) OS=Mus musculus GN=Lsm5 PE=2 SV=1 - [H3BKC5_MOUSE]	38.10	1	1	1	1	1.04	0.63	0.61	0.81	0.77	0.80	0.77	0.78	0.75	0.72	0.69	0.73	0.70	4.61	5.10
Q9WVQ5	Methylthioribulose-1-phosphate dehydratase OS=Mus musculus GN=Apip PE=1 SV=1 - [MTNB_MOUSE]	53.11	1	8	8	21	1.11	0.97	0.74	0.89	0.87	0.77	0.74	0.78	0.70	0.77	0.66	0.75	0.59	26.93	6.90
Q8K0B2-3	Isoform 3 of Probable lysosomal cobalamin transporter OS=Mus musculus GN=Lmbrd1 - [LMBD1_MOUSE]	3.85	3	1	1	2	0.99	1.30	1.31	1.06	1.07	0.95	0.96	0.78	0.79	0.96	0.97	0.80	0.81	53.41	7.80
E9Q330	Platelet-activating factor acetylhydrolase (Fragment) OS=Mus musculus GN=Pla2g7 PE=2 SV=1 - [E9Q330_MOUSE]	10.40	4	3	3	6	0.99	0.86	0.90	0.93	0.93	0.79	0.78	0.78	0.77	0.87	0.88	0.77	0.78	33.17	7.23
P22599	Alpha-1-antitrypsin 1-2 OS=Mus musculus GN=Serpina1b PE=1 SV=2 - [A1AT2_MOUSE]	23.00	4	3	8	18	0.83	0.98	1.17	0.73	0.90	0.71	0.86	0.78	0.90	1.08	1.20	0.62	0.77	45.95	5.54
I7HPY0	SH3 domain-binding glutamic acid-rich-like protein 3 OS=Mus musculus GN=Sh3bgrl3 PE=4 SV=1 - [I7HPY0_MOUSE]	94.59	1	1	2	23	0.88	0.70	0.79	0.42	0.48	0.86	0.97	0.78	0.89	0.65	0.74	0.80	0.90	4.21	4.32
Q9D387	Lysosome-associated membrane glycoprotein 5 OS=Mus musculus GN=Lamp5 PE=1 SV=2 - [LAMP5_MOUSE]	7.14	1	2	2	3	1.46	0.94	0.64	1.07	0.73	0.90	0.61	0.78	0.53	0.82	0.56	1.07	0.73	31.70	6.14
Q9WTS5	Teneurin-2 OS=Mus musculus GN=Tenm2 PE=2 SV=1 - [TEN2_MOUSE]	8.72	1	15	16	38	1.04	1.20	1.09	1.05	1.03	0.77	0.76	0.78	0.79	0.82	0.75	0.81	0.81	306.27	6.68
Q61239	Protein farnesyltransferase/geranylgeranyltransferase type-1 subunit alpha OS=Mus musculus GN=Fnta PE=1 SV=1 - [FNTA_MOUSE]	7.96	1	3	3	6	0.95	0.93	0.92	0.99	1.03	0.89	0.96	0.78	0.82	0.84	0.87	0.86	0.93	43.99	4.93
Q8R307	Vacuolar protein sorting-associated protein 18 homolog OS=Mus musculus GN=Vps18 PE=1 SV=2 - [VPS18_MOUSE]	8.94	1	6	6	18	1.07	1.30	1.11	1.21	1.14	0.88	0.87	0.78	0.76	0.98	0.92	1.05	1.09	110.15	6.09

Q5M8N4-	Isoform 2 of Epimerase family protein SDR39U1 OS=Mus musculus GN=Sdr39u1 - [D39U1_MOUSE]	4.44	2	1	1	2	0.94	1.19	1.27	0.84	0.89	0.71	0.75	0.78	0.83	0.88	0.94	1.04	1.11	31.44	9.31
Q9CQC6	Basic leucine zipper and W2 domain-containing protein 1 OS=Mus musculus GN=Bzw1 PE=1 SV=1 - [BZW1_MOUSE]	7.40	1	4	4	7	0.96	0.94	0.82	0.98	0.95	0.83	0.83	0.78	0.81	0.82	0.85	0.84	0.79	48.01	5.92
Q66GT5	Phosphatidylglycerophosphatase and protein-tyrosine phosphatase 1 OS=Mus musculus GN=Ptpmt1 PE=1 SV=1 - [PTPM1_MOUSE]	4.15	2	1	1	1	0.96	1.20	1.25	1.07	1.11	0.77	0.80	0.78	0.82	0.83	0.87	0.98	1.03	21.93	9.72
Q9D5T0	ATPase family AAA domain-containing protein 1 OS=Mus musculus GN=Atad1 PE=1 SV=1 - [ATAD1_MOUSE]	12.47	1	3	3	4	0.79	1.44	1.18	0.94	1.19	0.83	0.96	0.78	0.94	0.61	0.78	0.72	1.02	40.72	6.90
P35285	Ras-related protein Rab 22A OS=Mus musculus GN=Rab22a PE=1 SV=2 - [RB22A_MOUSE]	31.44	3	3	5	6	1.17	0.94	0.80	1.07	0.88	0.90	0.80	0.79	0.76	0.86	0.84	0.83	0.82	21.79	8.15
Q6ZQH8	Nucleoporin NUP188 homolog OS=Mus musculus GN=Nup188 PE=1 SV=2 - [NU188_MOUSE]	0.68	1	1	1	2	0.99	1.27	1.28	1.32	1.33	0.67	0.67	0.79	0.79	0.96	0.97	0.90	0.91	196.57	7.01
Q9CZN7	Serine hydroxymethyltransferase OS=Mus musculus GN=Shmt2 PE=2 SV=1 - [Q9CZN7_MOUSE]	9.52	1	5	5	6	1.04	1.10	1.02	1.16	1.15	0.89	0.83	0.79	0.78	0.81	0.84	0.88	0.85	55.72	8.47
P70362	Ubiquitin fusion degradation protein 1 homolog OS=Mus musculus GN=Ufd1l PE=1 SV=2 - [UFD1_MOUSE]	18.89	2	5	5	9	1.10	0.76	0.78	1.02	1.09	0.82	0.82	0.79	0.87	0.74	0.72	0.72	0.71	34.46	6.70
Q9DCC4	Pyrroline-5-carboxylate reductase 3 OS=Mus musculus GN=Pycrl PE=2 SV=2 - [P5CR3_MOUSE]	26.64	1	5	5	7	1.31	0.93	0.76	0.97	0.85	0.70	0.70	0.79	0.60	0.77	0.77	0.76	0.58	28.70	7.27
Q791V5	Mitochondrial carrier homolog 2 OS=Mus musculus GN=Mtch2 PE=1 SV=1 - [MTCH2_MOUSE]	29.37	3	7	7	15	1.01	1.17	1.12	1.14	1.13	0.78	0.69	0.79	0.76	0.84	0.81	0.99	0.94	33.48	8.25
P06151	L-lactate dehydrogenase A chain OS=Mus musculus GN=Ldha PE=1 SV=3 - [LDHA_MOUSE]	59.94	7	18	20	157	0.99	0.93	0.95	1.08	1.08	0.84	0.85	0.79	0.79	0.85	0.87	0.95	0.93	36.48	7.74
P21570	Angiogenin OS=Mus musculus GN=Ang PE=1 SV=1 - [ANGI_MOUSE]	7.59	1	1	1	1	0.90	0.97	1.08	0.94	1.05	0.85	0.94	0.79	0.87	0.88	0.98	0.82	0.92	16.22	9.41

Q6P8I6	Cytochrome c oxidase assembly protein COX11, mitochondrial OS=Mus musculus GN=Cox11 PE=2 SV=1 - [COX11_MOUSE]	2.55	1	1	1	1	1.19	1.10	0.92	0.89	0.75	1.18	0.98	0.79	0.66	1.17	0.98	0.96	0.81	30.83	8.78
Q8BHE8	Uncharacterized protein C2orf47 homolog, mitochondrial OS=Mus musculus PE=2 SV=1 - [CB047_MOUSE]	7.22	5	1	2	9	0.99	0.91	0.92	1.08	1.09	0.79	0.80	0.79	0.79	0.85	0.86	0.92	0.94	32.96	9.14
P36552	Coproporphyrinogen-III oxidase, mitochondrial OS=Mus musculus GN=Cpox PE=1 SV=2 - [HEM6_MOUSE]	16.93	1	5	5	11	0.92	1.02	1.13	0.94	1.06	0.86	0.91	0.79	0.94	0.84	0.90	0.91	0.98	49.68	8.53
D3YU06	Abhydrolase domain-containing protein 3 OS=Mus musculus GN=Abhd3 PE=2 SV=1 - [D3YU06_MOUSE]	7.21	4	2	2	4	0.88	0.96	1.09	1.04	1.18	0.76	0.86	0.79	0.89	0.89	1.01	0.90	1.03	45.21	7.30
D3Z151	Translation initiation factor eIF-2B subunit alpha (Fragment) OS=Mus musculus GN=Eif2b1 PE=2 SV=1 - [D3Z151_MOUSE]	5.38	2	1	1	2	1.27	0.83	0.65	1.17	0.92	0.95	0.75	0.79	0.62	0.83	0.65	0.92	0.73	20.62	9.19
F6ZEW4	Exportin-2 (Fragment) OS=Mus musculus GN=Cse1l PE=2 SV=1 - [F6ZEW4_MOUSE]	5.62	4	4	4	5	0.96	1.15	1.26	1.11	1.09	0.86	0.89	0.79	0.83	0.96	0.99	1.13	1.07	74.36	6.18
Q9CZW5	Mitochondrial import receptor subunit TOM70 OS=Mus musculus GN=Tomm70a PE=1 SV=2 - [TOM70_MOUSE]	35.02	1	17	17	51	0.98	0.90	0.90	1.10	1.13	0.85	0.92	0.79	0.84	0.87	0.96	0.96	1.00	67.55	7.53
Q9DB73	NADH-cytochrome b5 reductase 1 OS=Mus musculus GN=Cyb5r1 PE=2 SV=1 - [NB5R1_MOUSE]	31.15	3	8	8	18	0.90	0.98	1.08	1.03	1.14	0.81	0.81	0.79	0.79	0.86	0.96	0.95	0.97	34.11	8.87
Q3UE31	Uncharacterized protein KIAA0930 homolog OS=Mus musculus PE=1 SV=2 - [K0930_MOUSE]	5.20	2	2	2	4	0.90	1.80	2.01	1.01	1.13	0.69	0.76	0.79	0.88	0.76	0.85	0.92	1.02	45.93	8.13
Q8R1V9	Protein Qars OS=Mus musculus GN=Qars PE=2 SV=1 - [Q8R1V9_MOUSE]	5.61	3	3	3	5	0.98	0.87	1.00	1.03	1.09	0.84	0.86	0.79	0.80	0.97	0.99	0.97	1.03	68.78	7.39
Q9D486-3	Isoform 3 of C-Maf-inducing protein OS=Mus musculus GN=Cmip - [CMIP_MOUSE]	5.77	2	2	2	2	0.88	0.97	1.10	0.97	1.10	0.81	0.92	0.79	0.89	0.89	1.01	0.90	1.03	54.14	7.12
P28474	Alcohol dehydrogenase class-3 OS=Mus musculus GN=Adh5 PE=1 SV=3 - [ADHX_MOUSE]	31.55	2	8	8	20	0.97	1.01	0.98	1.10	1.16	0.85	0.80	0.79	0.81	0.81	0.80	0.85	0.83	39.52	7.25

Q91W43	Glycine dehydrogenase [decarboxylating], mitochondrial OS=Mus musculus GN=Gldc PE=1 SV=1 - [GCSP_MOUSE]	10.93	1	9	9	17	0.97	1.12	1.14	1.11	1.13	0.80	0.81	0.79	0.77	0.82	0.89	0.83	0.89	113.20	7.56
P31648	Sodium- and chloride-dependent GABA transporter 1 OS=Mus musculus GN=Slc6a1 PE=1 SV=2 - [SC6A1_MOUSE]	14.02	1	5	5	44	1.05	0.96	0.96	1.09	1.01	0.87	0.83	0.79	0.78	0.93	0.86	0.88	0.82	66.96	7.96
Q6PE15	Mycophenolic acid acyl-glucuronide esterase, mitochondrial OS=Mus musculus GN=Abhd10 PE=2 SV=1 - [ABHDA_MOUSE]	17.51	3	5	5	7	0.97	1.20	1.22	1.01	1.03	0.73	0.74	0.79	0.78	0.75	0.82	0.76	0.82	33.02	8.79
Q925H0	Acid-sensing ion channel 2 OS=Mus musculus GN=Asic2 PE=1 SV=1 - [ASIC2_MOUSE]	2.54	2	1	1	2	1.00	1.04	1.04	1.20	1.20	0.71	0.70	0.79	0.79	0.77	0.77	0.81	0.81	57.70	5.19
Q9D0I9	Arginine--tRNA ligase, cytoplasmic OS=Mus musculus GN=Rars PE=2 SV=2 - [SYRC_MOUSE]	12.42	1	8	8	11	1.08	0.96	0.84	1.16	1.10	0.90	0.83	0.79	0.71	0.96	0.93	1.06	0.97	75.63	7.55
Q8BP00-2	Isoform 2 of IQ calmodulin-binding motif-containing protein 1 OS=Mus musculus GN=Iqcb1 - [IQCB1_MOUSE]	5.53	2	3	3	4	1.10	1.24	1.21	1.05	0.98	0.84	0.82	0.79	0.74	0.85	0.82	1.06	0.86	69.03	9.51
Q9JI75	Ribosyldihydronicotinamide dehydrogenase [quinone] OS=Mus musculus GN=Nqo2 PE=2 SV=3 - [NQO2_MOUSE]	24.68	1	4	4	7	1.00	0.91	0.91	1.05	1.06	0.83	0.81	0.79	0.79	0.85	0.81	0.85	0.84	26.23	7.01
P06684	Complement C5 OS=Mus musculus GN=C5 PE=1 SV=2 - [CO5_MOUSE]	0.95	1	1	1	1	0.95	1.18	1.24	0.94	0.99	1.00	1.05	0.79	0.83	0.84	0.89	0.74	0.78	188.76	6.81
P60202	Myelin proteolipid protein OS=Mus musculus GN=Plp1 PE=1 SV=2 - [MYPR_MOUSE]	28.52	2	9	9	159	1.08	1.07	1.01	1.18	1.05	0.81	0.73	0.79	0.72	0.84	0.77	0.94	0.83	30.06	8.35
Q9JHU9	Inositol-3-phosphate synthase 1 OS=Mus musculus GN=Isyna1 PE=2 SV=1 - [INO1_MOUSE]	15.62	1	7	7	17	1.22	1.02	0.86	0.89	0.76	0.75	0.59	0.79	0.66	0.81	0.65	0.93	0.78	60.89	6.42
P61620	Protein transport protein Sec61 subunit alpha isoform 1 OS=Mus musculus GN=Sec61a1 PE=2 SV=2 - [S61A1_MOUSE]	5.67	2	1	2	3	1.06	0.86	0.82	0.93	0.88	0.83	0.79	0.79	0.75	0.87	0.82	0.99	0.94	52.23	8.06

Q8JZN5	Acyl-CoA dehydrogenase family member 9, mitochondrial OS=Mus musculus GN=Acad9 PE=2 SV=2 - [ACAD9_MOUSE]	27.68	3	14	14	30	1.04	1.05	1.14	1.10	1.06	0.82	0.83	0.79	0.83	0.85	0.86	0.98	0.92	68.68	7.46
Q920M7	Synaptotagmin-17 OS=Mus musculus GN=Syt17 PE=2 SV=1 - [SYT17_MOUSE]	9.79	1	4	4	5	1.06	1.09	0.85	0.91	0.91	0.93	0.87	0.79	0.75	1.01	0.96	1.11	1.05	53.26	7.15
Q3UHC9	Potassium voltage-gated channel subfamily H member 1 OS=Mus musculus GN=Kcnh1 PE=2 SV=1 - [Q3UHC9_MOUSE]	1.66	2	1	2	2	0.93	1.03	1.10	0.96	1.04	0.90	0.97	0.79	0.85	0.82	0.89	0.86	0.93	108.42	7.69
Q8BTZ7	Mannose-1-phosphate guanyltransferase beta OS=Mus musculus GN=Gmppb PE=2 SV=1 - [GMPPB_MOUSE]	15.56	1	4	4	7	0.92	1.21	1.13	1.01	1.00	0.93	0.94	0.79	0.91	0.87	0.93	1.02	0.94	39.89	6.74
A2AT37	Protein Upf2 OS=Mus musculus GN=Upf2 PE=2 SV=1 - [A2AT37_MOUSE]	4.33	2	5	5	6	0.93	1.01	1.02	0.86	1.08	0.84	1.01	0.79	0.91	0.78	1.03	0.64	0.84	147.46	5.64
Q9DC69	NADH dehydrogenase [ubiquinone] 1 alpha subcomplex subunit 9, mitochondrial OS=Mus musculus GN=Ndufa9 PE=1 SV=2 - [NDUA9_MOUSE]	32.63	1	12	12	42	1.03	1.03	1.08	1.17	1.14	0.79	0.77	0.79	0.76	0.85	0.81	0.94	0.86	42.50	9.74
Q9ESM3	Hyaluronan and proteoglycan link protein 2 OS=Mus musculus GN=Hapln2 PE=1 SV=1 - [HPLN2_MOUSE]	13.78	1	4	4	10	0.98	1.25	1.25	1.02	1.02	0.80	0.87	0.79	0.79	0.88	0.84	1.02	0.99	37.90	9.09
Q3UY51	Leucine-rich repeat-containing protein 55 OS=Mus musculus GN=Lrrc55 PE=2 SV=1 - [LRC55_MOUSE]	4.18	1	1	1	2	0.98	1.08	1.11	1.04	1.07	0.79	0.81	0.79	0.81	0.83	0.86	1.15	1.18	34.44	6.39
Q3TYK3	Nuclear factor 1 OS=Mus musculus GN=Nfix PE=2 SV=1 - [Q3TYK3_MOUSE]	3.58	6	1	1	1	0.87	0.51	0.59	1.02	1.18	0.89	1.02	0.79	0.91	0.84	0.97	0.61	0.70	43.57	7.99
Q3TPJ8	Cytoplasmic dynein 1 intermediate chain 2 OS=Mus musculus GN=Dync1i2 PE=2 SV=1 - [Q3TPJ8_MOUSE]	31.33	5	13	13	71	0.96	0.83	0.90	0.92	0.95	0.80	0.90	0.79	0.85	0.81	0.85	0.71	0.79	70.57	5.24

Q9ERZ4	Muscarinic acetylcholine receptor M2 OS=Mus musculus GN=Chrm2 PE=1 SV=2 - [ACM2_MOUSE]	1.72	1	1	1	1	0.68	0.83	1.22	1.02	1.51	0.91	1.33	0.79	1.16	0.77	1.13	0.90	1.33	51.47	8.92
D3YVU9	3-hydroxymethyl-3-methylglutaryl-CoA lyase, cytoplasmic OS=Mus musculus GN=Hmgcll1 PE=2 SV=1 - [D3YVU9_MOUSE]	4.85	3	1	1	2	1.05	1.21	1.15	1.04	0.99	0.74	0.70	0.79	0.75	0.90	0.85	0.92	0.88	22.47	6.73
D3YWW2	Gamma-glutamyltranspeptidase 1 (Fragment) OS=Mus musculus GN=Ggt1 PE=2 SV=1 - [D3YWW2_MOUSE]	5.29	8	1	1	2	1.00	1.25	1.25	0.93	0.93	0.76	0.76	0.79	0.79	0.81	0.81	0.85	0.85	20.36	9.14
E9Q4K2	Lactation elevated protein 1 OS=Mus musculus GN=Lace1 PE=2 SV=1 - [E9Q4K2_MOUSE]	4.30	2	1	1	2	1.00	0.89	0.89	1.17	1.17	0.83	0.83	0.79	0.79	0.82	0.82	0.85	0.85	31.19	9.42
Q8R3H7	Heparan sulfate 2-O-sulfotransferase 1 OS=Mus musculus GN=Hs2st1 PE=1 SV=2 - [HS2ST_MOUSE]	2.53	1	1	1	1	0.82	0.97	1.19	0.97	1.18	0.74	0.90	0.79	0.97	0.66	0.80	0.84	1.03	41.79	8.57
A6PWX1	Calcium channel flower homolog OS=Mus musculus GN=Cacfd1 PE=2 SV=1 - [A6PWX1_MOUSE]	6.42	2	1	1	1	1.09	0.99	0.91	1.04	0.96	0.80	0.73	0.79	0.73	0.91	0.84	0.91	0.84	12.04	5.06
Q8BUV8	Protein GPR107 OS=Mus musculus GN=Gpr107 PE=2 SV=2 - [GP107_MOUSE]	1.63	1	1	1	1	1.02	0.52	0.51	1.19	1.17	0.90	0.88	0.79	0.78	0.84	0.82	0.83	0.82	62.02	7.59
Q91WH7	ATPase, H+/K+ exchanging, gastric, alpha polypeptide OS=Mus musculus GN=Atp4a PE=2 SV=1 - [Q91WH7_MOUSE]	9.95	3	4	9	95	0.90	0.88	0.98	0.96	1.02	0.87	0.97	0.79	0.88	0.89	0.99	0.89	1.00	112.96	5.81
A2AVP4	RING finger and CCCH-type zinc finger domain-containing protein 2 OS=Mus musculus GN=Rc3h2 PE=2 SV=1 - [A2AVP4_MOUSE]	1.33	2	1	1	2	0.92	0.74	0.81	0.87	0.95	0.88	0.95	0.79	0.86	0.70	0.77	1.12	1.22	124.63	7.20
Q8C0D5	Elongation factor Tu GTP-binding domain-containing protein 1 OS=Mus musculus GN=Eftud1 PE=2 SV=1 - [ETUD1_MOUSE]	1.95	1	2	2	3	0.97	1.23	1.28	1.18	1.22	0.98	1.01	0.79	0.82	1.11	1.15	0.96	1.00	125.70	6.16

Q9D1P4	Cysteine and histidine-rich domain-containing protein 1 OS=Mus musculus GN=Chordc1 PE=1 SV=1 - [CHRD1_MOUSE]	44.71	1	11	11	29	0.96	0.86	0.77	0.94	0.94	0.82	0.83	0.79	0.85	0.81	0.86	0.78	0.82	37.33	7.90
Q99MN9	Propionyl-CoA carboxylase beta chain, mitochondrial OS=Mus musculus GN=Pccb PE=1 SV=2 - [PCCB_MOUSE]	39.74	3	15	15	29	0.93	0.94	1.03	1.05	1.11	0.89	0.92	0.79	0.85	0.94	1.00	0.96	0.99	58.37	7.66
Q3UUI3	Acyl-coenzyme A thioesterase THEM4 OS=Mus musculus GN=Them4 PE=1 SV=1 - [THEM4_MOUSE]	19.13	2	4	4	10	0.95	1.00	0.85	1.12	1.13	0.93	0.91	0.79	0.84	0.92	0.97	0.87	1.01	26.01	9.64
E9Q305	Protein Sbf2 OS=Mus musculus GN=Sbf2 PE=2 SV=1 - [E9Q305_MOUSE]	1.19	1	1	1	2	0.77	1.06	1.36	1.03	1.33	0.94	1.21	0.79	1.02	0.92	1.18	0.91	1.18	207.50	7.18
F8VPK0	Protein Ttc37 OS=Mus musculus GN=Ttc37 PE=2 SV=1 - [F8VPK0_MOUSE]	0.64	1	1	1	1	1.27	1.09	0.86	1.14	0.90	1.12	0.88	0.79	0.63	1.09	0.86	1.52	1.20	173.83	7.12
Q6NY15-	Isoform 2 of Testis-specific gene 10 protein OS=Mus musculus GN=Tsga10 - [TSG10_MOUSE]	5.36	4	3	3	3	1.08	1.13	1.05	1.07	1.02	0.96	0.78	0.79	0.75	1.38	1.12	1.07	1.02	80.30	5.80
O08967	Cytohesin-3 OS=Mus musculus GN=Cyth3 PE=1 SV=1 - [CYH3_MOUSE]	20.05	3	4	7	19	0.77	0.94	1.09	0.85	1.12	0.67	0.96	0.79	0.99	0.67	0.88	0.66	0.88	46.25	5.54
Q8R127	Saccharopine dehydrogenase-like oxidoreductase OS=Mus musculus GN=Sccpdh PE=2 SV=1 - [SCPDL_MOUSE]	22.38	2	7	7	14	1.00	1.07	1.05	1.14	1.14	0.83	0.85	0.79	0.77	0.99	0.98	1.04	1.02	47.10	8.60
Q8BIF2-4	Isoform 4 of RNA binding protein fox-1 homolog 3 OS=Mus musculus GN=Rbfox3 - [RFOX3_MOUSE]	29.07	7	4	8	15	0.90	0.81	0.85	0.92	1.02	0.78	0.85	0.80	0.89	0.66	0.77	0.95	1.04	34.05	7.27
E9Q264	Protein Myh15 OS=Mus musculus GN=Myh15 PE=4 SV=1 - [E9Q264_MOUSE]	2.60	1	1	5	9	0.98	0.77	0.78	0.73	0.74	0.99	1.01	0.80	0.81	0.95	0.97	1.03	1.05	221.71	6.13
P58802	TBC1 domain family member 10A OS=Mus musculus GN=Tbc1d10a PE=1 SV=1 - [TB10A_MOUSE]	1.40	2	1	1	1	0.79	0.98	1.24	0.93	1.18	0.69	0.87	0.80	1.01	0.79	1.00	0.80	1.01	56.17	7.85
Q7TPM6-	Isoform 2 of Fibronectin type III and SPRY domain-containing protein 1 OS=Mus musculus GN=Fsd1 - [FSD1_MOUSE]	11.90	2	4	4	9	1.03	0.94	0.79	1.06	0.91	0.93	0.83	0.80	0.75	0.87	0.79	0.98	0.92	53.65	6.95

Q8CGB6-	Isoform 2 of Tensin-like C1 domain-containing phosphatase OS=Mus musculus GN=Tenc1 - [TENC1_MOUSE]	1.74	4	1	2	4	1.01	1.11	1.10	1.03	1.01	0.62	0.61	0.80	0.78	0.79	0.78	0.88	0.87	149.41	8.34
Q9JL56	Glycerophosphodiester phosphodiesterase 1 OS=Mus musculus GN=Gde1 PE=2 SV=1 - [GDE1_MOUSE]	16.92	2	5	5	11	0.91	0.98	0.96	1.08	1.20	0.85	0.92	0.80	0.86	0.88	0.96	0.95	1.02	37.61	6.90
Q99KF1	Transmembrane emp24 domain-containing protein 9 OS=Mus musculus GN=Tmed9 PE=2 SV=2 - [TMED9_MOUSE]	11.91	2	2	2	8	0.96	0.99	1.09	0.89	0.97	0.71	0.77	0.80	0.82	0.83	0.86	0.88	0.90	27.11	8.41
Q5U458	DnaJ homolog subfamily C member 11 OS=Mus musculus GN=Dnajc11 PE=2 SV=2 - [DJC11_MOUSE]	15.38	3	7	7	14	1.12	1.02	0.93	1.03	1.12	0.85	0.85	0.80	0.75	0.94	0.84	0.95	0.85	63.19	8.32
Q80X95	Ras-related GTP-binding protein A OS=Mus musculus GN=Rraga PE=2 SV=1 - [RRAGA_MOUSE]	19.81	3	5	5	8	1.04	1.11	1.12	1.02	1.04	0.88	0.90	0.80	0.75	0.93	0.85	1.02	0.95	36.54	7.72
P61804	Dolichyl-diphosphooligosaccharide--protein glycosyltransferase subunit DAD1 OS=Mus musculus GN=Dad1 PE=2 SV=3 - [DAD1_MOUSE]	8.85	1	1	1	4	1.00	1.05	1.04	1.13	1.12	0.81	0.80	0.80	0.79	0.86	0.86	0.85	0.85	12.49	7.08
Q921E2	Ras-related protein Rab-31 OS=Mus musculus GN=Rab31 PE=1 SV=1 - [RAB31_MOUSE]	51.55	3	6	8	13	1.05	0.92	0.92	1.08	1.08	0.95	0.91	0.80	0.85	0.88	0.89	0.88	0.88	21.32	7.40
Q9D0L4	Uncharacterized aarF domain-containing protein kinase 1 OS=Mus musculus GN=Adck1 PE=2 SV=1 - [ADCK1_MOUSE]	5.33	2	2	2	4	0.98	1.22	1.24	1.17	1.19	0.76	0.77	0.80	0.81	0.83	0.86	1.06	1.09	59.70	8.05
P62743	AP-2 complex subunit sigma OS=Mus musculus GN=Ap2s1 PE=1 SV=1 - [AP2S1_MOUSE]	21.83	1	3	3	40	1.05	1.11	1.09	1.06	1.03	0.87	0.80	0.80	0.78	0.86	0.87	0.96	0.93	17.01	6.18
Q920P3	Deleted in bladder cancer protein 1 homolog OS=Mus musculus GN=Dbc1 PE=2 SV=1 - [DBC1_MOUSE]	5.13	2	4	4	7	0.99	1.08	0.94	1.12	1.09	0.89	0.84	0.80	0.80	0.87	0.87	0.92	0.92	88.58	8.97
Q8QZY6	Tetraspanin-14 OS=Mus musculus GN=Tspan14 PE=1 SV=1 - [TSN14_MOUSE]	6.30	1	1	1	1	1.09	0.92	0.85	0.93	0.86	0.63	0.58	0.80	0.73	0.80	0.74	0.55	0.51	30.65	6.51

Q6NXK8	Acid-sensing ion channel 1 OS=Mus musculus GN=Asic1 PE=1 SV=1 - [ASIC1_MOUSE]	3.99	2	2	2	4	0.78	0.90	0.84	0.84	1.07	0.94	1.18	0.80	0.92	0.84	1.07	0.84	0.77	59.63	5.58
P34914	Bifunctional epoxide hydrolase 2 OS=Mus musculus GN=Ephx2 PE=1 SV=2 - [HYES_MOUSE]	25.45	2	10	10	20	0.98	0.94	0.96	1.06	1.08	0.81	0.86	0.80	0.84	0.85	0.93	0.91	0.97	62.47	6.19
Q9QZE5	Coatomer subunit gamma-1 OS=Mus musculus GN=Copg1 PE=2 SV=1 - [COPG1_MOUSE]	7.21	2	2	4	8	0.88	1.09	1.24	0.90	1.03	0.77	0.87	0.80	0.91	0.74	0.84	0.89	1.02	97.45	5.35
P61924	Coatomer subunit zeta-1 OS=Mus musculus GN=Copz1 PE=2 SV=1 - [COPZ1_MOUSE]	22.60	1	3	3	7	0.93	0.79	0.82	0.97	1.01	0.89	0.92	0.80	0.84	0.87	0.93	0.82	0.87	20.19	4.81
E0CYK4	Repressor of RNA polymerase III transcription MAF1 homolog (Fragment) OS=Mus musculus GN=Maf1 PE=2 SV=1 - [E0CYK4_MOUSE]	3.47	3	1	1	1	0.91	0.89	0.97	0.83	0.91	0.92	1.00	0.80	0.87	0.78	0.86	0.76	0.84	22.16	5.39
Q9ET43	Claudin-12 OS=Mus musculus GN=Cldn12 PE=1 SV=2 - [CLD12_MOUSE]	6.15	1	1	1	1	0.62	0.72	1.16	0.91	1.47	0.65	1.05	0.80	1.28	0.66	1.07	0.58	0.94	26.98	8.47
Q3UUQ7	GPI inositol-deacylase OS=Mus musculus GN=Pgap1 PE=1 SV=3 - [PGAP1_MOUSE]	1.30	1	1	1	2	1.30	1.21	0.93	1.07	0.82	0.79	0.60	0.80	0.61	0.91	0.70	1.16	0.89	104.51	8.91
Q8R4G0-3	Isoform 1C of Netrin-G1 OS=Mus musculus GN=Ntng1 - [NTNG1_MOUSE]	10.27	14	4	4	4	0.99	0.83	0.79	1.00	1.06	0.90	0.91	0.80	0.81	1.01	1.02	0.99	1.00	49.31	6.28
Q3TJZ6	Protein FAM98A OS=Mus musculus GN=Fam98a PE=2 SV=1 - [FA98A_MOUSE]	5.63	1	1	2	7	0.76	1.20	1.57	0.98	1.29	0.92	1.20	0.80	1.05	0.72	0.95	0.81	1.06	55.02	8.95
P70665-3	Isoform 3 of Sialate O-acetylesterase OS=Mus musculus GN=Siae - [SIAE_MOUSE]	3.85	3	1	1	2	1.00	0.86	0.87	1.09	1.09	0.76	0.76	0.80	0.80	0.84	0.84	0.76	0.76	43.86	5.86
Q6PGE7	Sodium-dependent proline transporter OS=Mus musculus GN=Slc6a7 PE=2 SV=1 - [SC6A7_MOUSE]	7.22	1	3	3	5	1.22	1.08	0.98	1.15	1.01	0.88	0.68	0.80	0.65	1.01	0.83	1.19	0.98	71.02	6.61
P15864	Histone H1.2 OS=Mus musculus GN=Hist1h1c PE=1 SV=2 - [H12_MOUSE]	30.66	3	1	11	74	0.97	0.51	0.52	0.80	0.82	0.78	0.79	0.80	0.82	0.89	0.91	0.78	0.80	21.25	11.00
B2RWC4	Gm88 protein OS=Mus musculus GN=Lrrc73 PE=2 SV=1 - [B2RWC4_MOUSE]	7.91	1	2	2	4	0.94	1.04	1.10	1.00	1.06	0.85	0.90	0.80	0.85	0.83	0.88	0.96	1.02	33.43	4.84

Q9R1M5	NACHT, LRR and PYD domains-containing protein 5 OS=Mus musculus GN=Nlrp5 PE=1 SV=2 - [NALP5_MOUSE]	2.67	8	2	2	2	1.34	1.20	0.89	1.21	0.90	0.83	0.61	0.80	0.59	0.75	0.56	0.96	0.71	131.23	5.71
Q6RKD8	Fibronectin leucine rich transmembrane protein 1 OS=Mus musculus GN=Flrt1 PE=2 SV=1 - [Q6RKD8_MOUSE]	3.12	1	1	2	2	1.02	1.27	1.24	1.32	1.29	0.89	0.87	0.80	0.78	1.03	1.01	0.81	0.79	74.10	6.60
Q80YE7-2	Isoform 2 of Death-associated protein kinase 1 OS=Mus musculus GN=Dapk1 - [DAPK1_MOUSE]	1.12	2	1	1	2	0.77	0.96	1.25	1.07	1.38	0.95	1.23	0.80	1.04	0.95	1.24	0.97	1.26	159.88	6.92
Q5DTT2-2	Isoform 2 of PH and SEC7 domain-containing protein 1 OS=Mus musculus GN=Psd - [PSD1_MOUSE]	13.49	1	1	5	8	0.93	0.72	0.77	0.98	1.05	0.85	0.91	0.80	0.86	0.81	0.87	0.66	0.72	43.69	10.11
P28028	Serine/threonine-protein kinase B-raf OS=Mus musculus GN=Braf PE=1 SV=3 - [BRAF_MOUSE]	12.06	4	2	7	13	1.24	1.10	0.88	1.12	0.90	0.95	0.83	0.80	0.77	0.87	0.78	1.02	0.92	88.72	7.65
Q80Y73	UPF0722 protein C11orf88 homolog OS=Mus musculus PE=2 SV=1 - [CK088_MOUSE]	4.17	1	1	1	1	0.94	1.07	1.14	0.94	1.00	0.79	0.83	0.80	0.85	0.81	0.87	0.82	0.87	19.06	9.00
Q9CPP6	NADH dehydrogenase [ubiquinone] 1 alpha subcomplex subunit 5 OS=Mus musculus GN=Ndufa5 PE=1 SV=3 - [NDUA5_MOUSE]	70.69	2	9	9	63	0.90	0.76	0.77	0.85	0.91	0.77	0.84	0.80	0.86	0.79	0.83	0.76	0.84	13.35	8.10
Q8R151	NFX1-type zinc finger-containing protein 1 OS=Mus musculus GN=Znfx1 PE=2 SV=3 - [ZNFX1_MOUSE]	1.15	4	2	2	3	0.91	1.03	1.13	0.97	1.07	0.90	0.99	0.80	0.88	0.82	0.91	0.83	0.92	218.69	7.47
O08739	AMP deaminase 3 OS=Mus musculus GN=Ampd3 PE=2 SV=2 - [AMPD3_MOUSE]	6.92	6	5	5	8	1.04	1.13	1.13	0.98	0.92	0.86	0.89	0.80	0.75	0.86	0.78	1.18	1.23	88.60	7.33
Q9CXY6	Interleukin enhancer-binding factor 2 OS=Mus musculus GN=Ilf2 PE=1 SV=1 - [ILF2_MOUSE]	17.69	1	5	5	14	1.07	0.88	0.98	1.05	1.04	0.80	0.83	0.80	0.88	0.79	0.94	0.87	0.88	43.04	5.26
Q810U4-2	Isoform 2 of Neuronal cell adhesion molecule OS=Mus musculus GN=Nrcam - [NRCAM_MOUSE]	40.47	2	1	36	373	0.89	0.70	0.79	0.80	0.90	0.95	1.05	0.80	0.90	1.05	1.17	0.66	0.74	131.19	6.06
P22892	AP-1 complex subunit gamma-1 OS=Mus musculus GN=Ap1g1 PE=1 SV=3 - [AP1G1_MOUSE]	2.07	2	2	2	3	0.94	0.93	0.98	1.09	1.16	0.84	0.89	0.80	0.85	0.87	0.93	0.98	1.04	91.29	6.80

Q8BU14	Translocation protein SEC62 OS=Mus musculus GN=Sec62 PE=1 SV=1 - [SEC62_MOUSE]	11.06	1	3	3	9	0.97	1.03	0.99	1.19	1.19	0.90	0.90	0.80	0.78	0.90	0.88	0.87	0.82	45.55	7.31
A2ARS0	Ankyrin repeat domain-containing protein 63 OS=Mus musculus GN=Ankrd63 PE=4 SV=1 - [ANR63_MOUSE]	6.92	1	2	2	5	0.99	1.31	1.38	1.45	1.58	1.05	1.04	0.80	0.80	1.37	1.39	1.65	1.69	41.04	10.83
Q6P5E4	UDP-glucose:glycoprotein glucosyltransferase 1 OS=Mus musculus GN=Uggt1 PE=1 SV=4 - [UGGG1_MOUSE]	11.61	6	14	14	25	0.90	0.91	1.06	1.05	1.15	0.87	0.91	0.80	0.89	0.89	0.96	0.90	0.95	176.32	5.62
Q3TMH2	Secernin-3 OS=Mus musculus GN=Scrn3 PE=1 SV=1 - [SCRN3_MOUSE]	19.62	2	7	7	17	0.98	0.78	0.76	1.02	0.96	0.86	0.79	0.80	0.69	0.83	0.85	0.91	0.82	47.63	5.66
D3YXG2	N-acetyl-D-glucosamine kinase OS=Mus musculus GN=Nagk PE=2 SV=1 - [D3YXG2_MOUSE]	12.76	3	3	3	5	0.98	1.02	1.11	1.02	1.04	0.66	0.78	0.80	0.77	0.95	0.97	1.11	0.94	36.18	5.59
Q99KP3	Lambda-crystallin homolog OS=Mus musculus GN=Cryl1 PE=2 SV=3 - [CRYL1_MOUSE]	32.60	1	6	6	9	1.10	1.16	1.00	0.99	0.90	0.97	0.83	0.80	0.75	0.97	0.75	0.87	0.79	35.19	5.86
H3BKN2	Alkyldihydroxyacetone phosphate synthase, peroxisomal OS=Mus musculus GN=Agps PE=2 SV=1 - [H3BKN2_MOUSE]	1.94	3	1	1	2	0.92	1.02	1.11	1.08	1.18	0.81	0.87	0.80	0.87	0.92	1.00	1.13	1.24	63.80	6.95
O88543	COP9 signalosome complex subunit 3 OS=Mus musculus GN=Cops3 PE=1 SV=3 - [CSN3_MOUSE]	14.66	1	5	5	19	0.90	0.93	0.98	0.97	1.04	0.84	0.92	0.80	0.88	0.79	0.88	0.87	1.00	47.80	6.65
Q99J39-2	Isoform Cytoplasmic+peroxisomal of Malonyl-CoA decarboxylase, mitochondrial OS=Mus musculus GN=Mlycd - [DCMC_MOUSE]	7.49	2	2	2	3	1.01	1.83	1.82	1.08	1.07	0.95	0.94	0.80	0.80	0.92	0.92	1.05	1.04	50.73	7.88
Q60823	RAC-beta serine/threonine-protein kinase OS=Mus musculus GN=Akt2 PE=1 SV=1 - [AKT2_MOUSE]	7.90	7	1	3	6	1.02	1.05	1.02	1.06	1.03	0.78	0.76	0.80	0.78	0.81	0.79	0.82	0.80	55.71	6.37
Q6PB66	Leucine-rich PPR motif-containing protein, mitochondrial OS=Mus musculus GN=Lrpprc PE=1 SV=2 - [LPPRC_MOUSE]	20.04	2	20	20	42	1.01	1.13	1.20	1.28	1.25	0.87	0.89	0.80	0.80	0.92	0.92	0.97	1.00	156.52	6.83

O70340	Neuronal pentraxin-2 OS=Mus musculus GN=Nptx2 PE=2 SV=1 - [NPTX2_MOUSE]	29.37	1	9	9	22	0.98	0.94	0.98	0.85	0.87	0.77	0.79	0.80	0.73	0.74	0.83	0.76	0.79	47.11	5.81
D3YWV5	Semaphorin-4A OS=Mus musculus GN=Sema4a PE=2 SV=1 - [D3YWV5_MOUSE]	6.53	4	3	3	10	1.10	0.96	0.84	1.10	1.01	0.78	0.64	0.80	0.73	0.85	0.83	0.90	0.81	68.88	7.33
Q8VD37	SH3-containing GRB2-like protein 3-interacting protein 1 OS=Mus musculus GN=Sgip1 PE=1 SV=1 - [SGIP1_MOUSE]	52.85	5	1	29	154	0.76	1.11	1.45	0.96	1.26	0.85	1.11	0.80	1.05	0.72	0.95	0.77	1.01	86.01	7.87
F6QP00	WD repeat domain phosphoinositide-interacting protein 4 (Fragment) OS=Mus musculus GN=Wdr45 PE=2 SV=1 - [F6QP00_MOUSE]	12.82	7	1	1	3	0.92	1.08	1.17	0.95	1.03	0.81	0.87	0.80	0.87	0.78	0.85	0.89	0.97	8.54	6.10
Q3UX43	Ankyrin repeat domain-containing protein 13C OS=Mus musculus GN=Ankrd13c PE=2 SV=2 - [AN13C_MOUSE]	2.40	1	1	1	2	1.00	1.47	1.46	0.94	0.93	0.84	0.84	0.81	0.80	0.86	0.86	0.90	0.90	60.11	6.90
Q9CQF4	Uncharacterized protein C6orf203 homolog OS=Mus musculus PE=1 SV=1 - [CF203_MOUSE]	19.17	2	5	5	50	1.03	0.83	0.77	0.85	0.81	0.86	0.78	0.81	0.79	0.74	0.65	0.70	0.66	27.83	9.36
L7N479	Protein Tcrg-C3 OS=Mus musculus GN=Tcrg-C3 PE=4 SV=1 - [L7N479_MOUSE]	8.72	2	1	1	1	0.97	0.88	0.90	0.94	0.97	1.07	1.10	0.81	0.83	0.91	0.94	0.82	0.85	19.77	8.84
Q9CPQ8	ATP synthase subunit g, mitochondrial OS=Mus musculus GN=Atp5l PE=1 SV=1 - [ATP5L_MOUSE]	48.54	3	4	4	33	0.96	0.99	1.06	1.17	1.17	0.86	0.91	0.81	0.85	0.93	0.96	0.92	0.98	11.42	9.74
O08582	GTP-binding protein 1 OS=Mus musculus GN=Gtpbp1 PE=1 SV=2 - [GTPB1_MOUSE]	5.24	1	3	3	6	0.93	0.64	0.68	1.10	1.16	0.89	0.95	0.81	0.87	0.82	0.88	0.94	1.00	72.25	8.29
P98064	Mannan-binding lectin serine protease 1 OS=Mus musculus GN=Masp1 PE=1 SV=2 - [MASP1_MOUSE]	1.99	2	1	1	1	1.39	0.81	0.58	0.85	0.61	1.12	0.80	0.81	0.58	1.31	0.94	1.04	0.75	79.92	5.55
Q8R0G9	Nuclear pore complex protein Nup133 OS=Mus musculus GN=Nup133 PE=1 SV=2 - [NU133_MOUSE]	2.86	1	2	2	5	0.70	0.89	1.26	1.14	1.55	0.88	1.33	0.81	1.06	0.93	1.04	1.09	1.21	128.54	5.20

O09161	Calsequestrin-2 OS=Mus musculus GN=Casq2 PE=2 SV=3 - [CASQ2_MOUSE]	5.06	3	2	2	3	0.91	0.79	0.86	0.79	0.86	0.69	0.76	0.81	0.88	0.63	0.69	0.74	0.82	48.15	4.27
P21836	Acetylcholinesterase OS=Mus musculus GN=Ache PE=1 SV=1 - [ACES_MOUSE]	17.92	1	7	7	19	1.12	1.29	1.15	1.18	1.06	0.82	0.72	0.81	0.70	0.98	0.88	1.21	1.06	68.13	6.33
Q8K2I1	Protein farnesyltransferase subunit beta OS=Mus musculus GN=Fntb PE=1 SV=1 - [FNTB_MOUSE]	2.06	2	1	1	1	0.89	1.01	1.13	1.01	1.13	0.76	0.85	0.81	0.90	1.00	1.13	0.85	0.95	48.79	5.80
P17047	Lysosome-associated membrane glycoprotein 2 OS=Mus musculus GN=Lamp2 PE=2 SV=2 - [LAMP2_MOUSE]	2.17	3	1	1	3	0.81	0.85	1.06	0.88	1.09	0.83	1.02	0.81	1.00	0.71	0.88	1.22	1.52	45.65	7.39
Q3TAA7	Serine/threonine-protein kinase 11-interacting protein OS=Mus musculus GN=Stk11ip PE=1 SV=1 - [S11IP_MOUSE]	1.31	1	1	1	2	0.94	1.07	1.13	1.08	1.14	0.78	0.83	0.81	0.85	0.99	1.06	1.08	1.15	117.93	4.96
P61226	Ras-related protein Rap 2b OS=Mus musculus GN=Rap2b PE=1 SV=1 - [RAP2B_MOUSE]	67.76	2	5	10	43	1.00	0.91	0.86	1.05	1.03	0.89	0.85	0.81	0.79	0.82	0.79	0.86	0.85	20.49	4.81
Q8CE96-3	Isoform 3 of tRNA (adenine(58)-N(1))-methyltransferase non-catalytic subunit TRM6 OS=Mus musculus GN=Trmt6 - [TRM6_MOUSE]	3.50	2	1	1	2	1.19	1.18	0.99	1.06	0.89	0.94	0.78	0.81	0.67	0.84	0.70	1.15	0.97	34.78	5.63
O54828	Regulator of G-protein signaling 9 OS=Mus musculus GN=Rgs9 PE=1 SV=3 - [RGS9_MOUSE]	8.30	4	5	6	11	0.92	1.34	1.48	1.31	1.55	1.00	1.20	0.81	0.80	1.27	1.58	1.70	1.78	76.93	9.33
Q9QZ88-2	Isoform 2 of Vacuolar protein sorting-associated protein 29 OS=Mus musculus GN=Vps29 - [VPS29_MOUSE]	43.55	2	1	6	23	0.87	0.77	0.89	0.79	0.90	0.71	0.81	0.81	0.93	0.88	1.01	0.92	1.06	20.90	7.05
P62965	Cellular retinoic acid-binding protein 1 OS=Mus musculus GN=Crabp1 PE=1 SV=2 - [RABP1_MOUSE]	9.49	1	1	1	9	1.15	1.12	0.88	0.86	0.73	1.06	0.88	0.81	0.73	1.47	1.33	2.19	1.85	15.58	5.38
Q9R0N8-2	Isoform 2 of Synaptotagmin-6 OS=Mus musculus GN=Syt6 - [SYT6_MOUSE]	7.51	4	2	2	3	1.58	0.98	0.70	1.44	0.91	1.21	0.76	0.81	0.58	1.31	0.83	1.37	0.99	48.31	8.43

F6RMJ1	SUN domain-containing protein 1 (Fragment) OS=Mus musculus GN=Sun1 PE=4 SV=1 - [F6RMJ1_MOUSE]	10.85	6	2	2	8	1.08	1.10	0.99	1.07	0.94	0.90	0.80	0.81	0.76	0.83	0.76	0.98	0.94	28.99	5.07
Q64444	Carbonic anhydrase 4 OS=Mus musculus GN=Ca4 PE=1 SV=1 - [CAH4_MOUSE]	29.18	2	9	9	28	0.98	0.88	0.92	0.97	0.98	0.75	0.79	0.81	0.82	0.72	0.74	0.72	0.77	34.33	8.21
D3YZN4	Paraplegin OS=Mus musculus GN=Spg7 PE=2 SV=1 - [D3YZN4_MOUSE]	4.88	6	3	3	6	1.10	0.87	0.79	1.12	1.08	0.98	0.81	0.81	0.84	0.72	0.65	0.92	0.80	74.96	7.40
Q9CXT7-2	Isoform 2 of Transmembrane protein 192 OS=Mus musculus GN=Tmem192 - [TM192_MOUSE]	6.79	2	1	1	2	1.10	1.35	1.23	1.12	1.01	0.76	0.69	0.81	0.73	0.84	0.77	0.91	0.83	25.38	9.14
Q8BRF7-3	Isoform 3 of Sec1 family domain-containing protein 1 OS=Mus musculus GN=Scfd1 - [SCFD1_MOUSE]	16.75	3	6	6	10	0.92	1.12	1.31	1.11	1.20	0.95	1.02	0.81	0.91	0.91	1.02	1.01	1.09	67.36	5.74
Q8BP71-6	Isoform 6 of RNA binding protein fox-1 homolog 2 OS=Mus musculus GN=Rbfox2 - [RFOX2_MOUSE]	17.90	9	1	6	11	1.19	1.23	1.03	1.01	0.84	0.64	0.53	0.81	0.68	0.82	0.69	0.83	0.69	37.88	7.20
Q9CZT8	Ras-related protein Rab 3B OS=Mus musculus GN=Rab3b PE=1 SV=1 - [RAB3B_MOUSE]	53.88	11	5	10	106	1.17	0.93	0.85	1.10	0.92	1.04	0.92	0.81	0.68	1.00	0.87	1.01	0.85	24.74	5.11
Q7TRP3	Olfactory receptor 670 OS=Mus musculus GN=Olfr670 PE=3 SV=1 - [Q7TRP3_MOUSE]	8.33	1	1	1	2	0.98	1.15	1.18	1.15	1.17	0.76	0.78	0.81	0.82	0.71	0.72	0.86	0.88	35.24	9.23
Q8VC30	Bifunctional ATP-dependent dihydroxyacetone kinase/FAD-AMP lyase (cyclizing) OS=Mus musculus GN=Dak PE=2 SV=1 - [DHAK_MOUSE]	2.42	1	1	1	2	0.96	1.06	1.10	1.09	1.14	0.85	0.88	0.81	0.84	0.90	0.93	1.04	1.08	59.65	6.92
O54950	5'-AMP-activated protein kinase subunit gamma-1 OS=Mus musculus GN=Prkag1 PE=1 SV=2 - [AAKG1_MOUSE]	23.64	6	5	7	12	1.01	1.02	1.01	1.00	0.97	0.85	0.80	0.81	0.73	0.86	0.85	0.85	0.83	37.50	7.14
Q5SQX6	Cytoplasmic FMR1-interacting protein 2 OS=Mus musculus GN=Cyfip2 PE=1 SV=2 - [CYFP2_MOUSE]	20.91	4	13	22	49	0.96	1.09	1.11	1.18	1.19	0.93	0.93	0.81	0.88	0.93	1.00	1.07	1.08	145.56	7.05
Q8CGA0	Protein phosphatase 1F OS=Mus musculus GN=Ppm1f PE=2 SV=1 - [PPM1F_MOUSE]	15.71	1	4	4	8	1.08	1.02	0.96	1.02	1.13	0.95	0.92	0.81	0.91	0.86	0.95	1.02	1.04	49.58	5.30

B9EHT4	CAP-Gly domain-containing linker protein 3 OS=Mus musculus GN=Clip3 PE=1 SV=1 - [CLIP3_MOUSE]	1.46	1	1	1	2	1.20	1.14	0.95	1.09	0.91	0.97	0.81	0.81	0.67	0.84	0.70	0.88	0.74	59.55	7.94
Q99P58	Ras-related protein Rab 27B OS=Mus musculus GN=Rab27b PE=1 SV=3 - [RB27B_MOUSE]	22.94	2	4	4	8	1.32	1.30	0.82	1.20	0.94	0.92	0.63	0.81	0.57	1.01	0.69	0.91	0.78	24.54	5.54
Q9EQ06	Estradiol 17-beta-dehydrogenase 11 OS=Mus musculus GN=Hsd17b11 PE=2 SV=1 - [DHB11_MOUSE]	25.17	5	6	6	12	1.03	0.95	0.86	1.04	1.02	0.87	0.74	0.81	0.75	0.94	0.91	1.03	0.98	32.86	8.66
Q8BH86-2	Isoform 2 of UPF0317 protein C14orf159 homolog, mitochondrial OS=Mus musculus - [CN159_MOUSE]	10.70	3	5	5	9	0.97	0.99	1.01	0.99	1.05	0.73	0.80	0.81	0.89	0.84	0.93	0.89	0.98	61.21	7.44
Q9CYW4	Haloacid dehalogenase-like hydrolase domain-containing protein 3 OS=Mus musculus GN=Hdhd3 PE=2 SV=1 - [HDHD3_MOUSE]	4.78	1	1	1	2	0.99	1.07	1.08	1.15	1.16	0.90	0.90	0.81	0.82	0.98	0.99	0.79	0.80	28.01	6.80
Q8VE47	Ubiquitin-like modifier-activating enzyme 5 OS=Mus musculus GN=Uba5 PE=2 SV=2 - [UBA5_MOUSE]	34.99	3	7	7	13	0.92	0.96	1.03	1.03	1.18	0.94	1.02	0.81	0.92	0.97	1.01	1.03	1.08	44.76	4.96
Q8BLY2	Probable threonine--tRNA ligase 2, cytoplasmic OS=Mus musculus GN=Tarsl2 PE=2 SV=1 - [SYTC2_MOUSE]	11.77	1	8	8	12	0.93	0.87	0.92	1.00	1.05	0.78	0.84	0.81	0.94	0.81	0.95	0.79	0.85	91.26	7.53
Q9WUL7	ADP-ribosylation factor-like protein 3 OS=Mus musculus GN=Arl3 PE=1 SV=1 - [ARL3_MOUSE]	64.29	1	9	9	29	0.93	0.87	0.90	0.92	0.97	0.77	0.82	0.81	0.86	0.74	0.77	0.73	0.87	20.47	7.24
D6RIL4	Methylosome protein 50 OS=Mus musculus GN=Wdr77 PE=2 SV=1 - [D6RIL4_MOUSE]	21.67	2	1	1	2	1.03	0.85	0.82	0.97	0.94	0.89	0.86	0.81	0.78	0.90	0.87	0.82	0.80	6.75	9.31
Q6PA06-2	Isoform 2 of Atlastin-2 OS=Mus musculus GN=Atl2 - [ATLA2_MOUSE]	16.49	3	4	5	14	0.94	1.07	1.05	1.04	1.13	0.97	0.99	0.81	0.90	0.91	1.01	0.97	1.03	44.33	6.21
Q9DCN2-	Isoform 2 of NADH-cytochrome b5 reductase 3 OS=Mus musculus GN=Cyb5r3 - [NB5R3_MOUSE]	48.92	4	11	11	31	1.08	0.99	0.91	1.15	0.99	0.86	0.79	0.81	0.75	0.97	0.86	1.08	0.97	31.53	8.38

Q80TI0-2	Isoform 2 of GRAM domain-containing protein 1B OS=Mus musculus GN=Gramd1b - [GRM1B_MOUSE]	3.30	6	2	2	6	1.16	0.89	0.95	1.18	1.26	0.84	1.01	0.81	0.87	1.00	0.86	1.31	1.13	80.98	5.78
Q9WUT3	Ribosomal protein S6 kinase alpha-2 OS=Mus musculus GN=Rps6ka2 PE=2 SV=1 - [KS6A2_MOUSE]	1.36	1	1	1	2	0.95	0.99	1.04	1.04	1.10	0.99	1.04	0.81	0.86	1.13	1.19	1.30	1.38	83.10	8.50
Q6NS52	Diacylglycerol kinase beta OS=Mus musculus GN=Dgkb PE=2 SV=2 - [DGKB_MOUSE]	14.96	1	1	10	20	0.76	0.72	0.94	0.96	1.26	0.92	1.20	0.81	1.06	0.99	1.30	1.07	1.41	90.21	7.93
Q6RI63-2	Isoform 2 of Constitutive coactivator of peroxisome proliferator-activated receptor gamma OS=Mus musculus GN=Fam120b - [F120B_MOUSE]	1.82	2	1	1	1	1.16	0.78	0.67	1.17	1.01	0.81	0.70	0.81	0.70	0.89	0.77	1.03	0.89	55.79	5.43
Q8JZQ2	AFG3-like protein 2 OS=Mus musculus GN=Afg3l2 PE=1 SV=1 - [AFG32_MOUSE]	22.19	3	15	15	24	1.00	1.03	1.05	1.13	1.12	0.89	0.89	0.81	0.86	0.85	0.86	0.88	0.88	89.46	8.60
Q91YR1	Twinfilin-1 OS=Mus musculus GN=Twf1 PE=1 SV=2 - [TWF1_MOUSE]	29.14	2	7	7	23	0.93	0.86	0.90	1.01	1.01	0.86	0.85	0.81	0.85	0.90	0.93	0.89	0.87	40.05	6.67
Q9CX56	26S proteasome non-ATPase regulatory subunit 8 OS=Mus musculus GN=Psmd8 PE=1 SV=2 - [PSMD8_MOUSE]	18.70	1	4	4	8	0.94	0.81	0.96	1.14	1.12	0.93	0.99	0.81	0.96	0.85	1.02	0.93	1.12	39.90	9.58
P32261	Antithrombin-III OS=Mus musculus GN=Serpinc1 PE=1 SV=1 - [ANT3_MOUSE]	5.38	1	2	2	5	1.05	1.00	0.92	0.58	0.55	0.83	0.73	0.81	0.71	0.83	0.79	0.88	0.76	51.97	6.46
Q3TVF4	Geranylgeranyl transferase type-2 subunit beta OS=Mus musculus GN=Rabggtb PE=2 SV=1 - [Q3TVF4_MOUSE]	7.25	2	2	2	4	0.97	0.89	0.92	1.11	1.14	0.85	0.87	0.81	0.84	0.88	0.91	0.88	0.91	36.86	5.08
Q3V038	Tetratricopeptide repeat protein 9A OS=Mus musculus GN=Ttc9 PE=2 SV=1 - [TTC9A_MOUSE]	41.10	1	6	7	16	0.88	0.96	1.04	0.86	1.00	0.76	0.86	0.81	0.94	0.70	0.78	0.73	0.81	24.34	8.85
Q8BH58	TIP41-like protein OS=Mus musculus GN=Tiprl PE=2 SV=1 - [TIPRL_MOUSE]	26.57	1	4	4	10	0.96	0.90	1.03	0.93	0.98	0.95	1.01	0.82	0.82	1.03	0.99	1.06	1.11	31.23	5.63

Q8R3V2	Aminoacyl tRNA synthase complex-interacting multifunctional protein 2 OS=Mus musculus GN=Aimp2 PE=2 SV=1 - [Q8R3V2_MOUSE]	16.07	2	2	2	3	0.94	0.88	0.93	0.90	0.95	0.79	0.84	0.82	0.84	1.06	1.12	0.89	0.95	31.08	7.46
Q60625	Intercellular adhesion molecule 5 OS=Mus musculus GN=Icam5 PE=1 SV=2 - [ICAM5_MOUSE]	24.97	1	17	17	115	0.99	1.07	1.07	0.98	1.00	0.81	0.87	0.82	0.84	0.81	0.82	0.87	0.92	96.88	6.32
E0CZE0	NEDD8-activating enzyme E1 regulatory subunit OS=Mus musculus GN=Nae1 PE=2 SV=1 - [E0CZE0_MOUSE]	22.75	2	8	8	49	1.00	1.08	1.09	0.95	1.00	0.99	1.01	0.82	0.88	0.94	0.92	0.94	1.06	57.58	5.85
P16125	L-lactate dehydrogenase B chain OS=Mus musculus GN=Ldhb PE=1 SV=2 - [LDHB_MOUSE]	66.77	2	17	19	224	0.99	0.96	0.99	1.06	1.07	0.82	0.85	0.82	0.81	0.86	0.89	0.87	0.88	36.55	6.05
Q3TXY2	Protein Tex264 OS=Mus musculus GN=Tex264 PE=2 SV=1 - [Q3TXY2_MOUSE]	8.12	2	2	2	3	1.06	1.11	1.05	1.26	1.19	1.01	0.95	0.82	0.77	1.23	1.17	1.14	1.08	28.99	5.07
A2AUF7	Rab9 effector protein with kelch motifs OS=Mus musculus GN=Rabepk PE=2 SV=1 - [A2AUF7_MOUSE]	8.72	3	2	2	8	0.99	1.07	1.11	0.89	0.89	0.72	0.71	0.82	0.82	0.74	0.74	1.01	1.02	34.56	5.54
Q9QXK7	Cleavage and polyadenylation specificity factor subunit 3 OS=Mus musculus GN=Cpsf3 PE=1 SV=2 - [CPSF3_MOUSE]	5.12	1	3	3	6	0.94	1.16	1.20	0.83	0.88	0.90	0.94	0.82	0.89	0.82	0.94	0.81	0.87	77.46	5.60
P47708	Rabphilin-3A OS=Mus musculus GN=Rph3a PE=1 SV=2 - [RP3A_MOUSE]	60.79	2	35	36	341	1.03	0.96	0.91	0.97	0.93	0.78	0.78	0.82	0.79	0.75	0.74	0.71	0.69	75.44	8.27
O08914	Fatty-acid amide hydrolase 1 OS=Mus musculus GN=Faah PE=2 SV=1 - [FAAH1_MOUSE]	6.56	1	3	3	3	0.95	0.85	0.89	1.12	1.21	0.86	1.32	0.82	1.12	1.04	1.11	0.97	1.03	63.18	7.87
Q8BHF7	CDP-diacylglycerol--glycerol-3-phosphate 3-phosphatidyltransferase, mitochondrial OS=Mus musculus GN=Pgs1 PE=2 SV=1 - [PGPS1_MOUSE]	2.53	1	1	1	2	0.91	1.19	1.30	1.05	1.16	0.66	0.72	0.82	0.89	0.79	0.87	0.93	1.03	62.45	8.90
Q8BGE6	Cysteine protease ATG4B OS=Mus musculus GN=Atg4b PE=1 SV=2 - [ATG4B_MOUSE]	8.14	2	2	2	4	0.90	0.86	0.95	1.03	1.15	0.85	0.94	0.82	0.91	0.85	0.95	0.91	1.01	44.35	5.07

Q9DCL2	MIP18 family protein FAM96A OS=Mus musculus GN=Fam96a PE=2 SV=1 - [FA96A_MOUSE]	5.63	1	1	1	2	0.89	0.98	1.10	0.84	0.95	1.02	1.14	0.82	0.92	0.83	0.94	0.91	1.03	18.41	4.82
P59325	Eukaryotic translation initiation factor 5 OS=Mus musculus GN=Eif5 PE=1 SV=1 - [IF5_MOUSE]	35.20	1	15	15	38	0.85	0.73	0.81	0.83	0.92	0.80	0.89	0.82	0.92	0.71	0.83	0.72	0.81	48.94	5.52
O08688	Calpain-5 OS=Mus musculus GN=Capn5 PE=2 SV=1 - [CAN5_MOUSE]	19.84	1	9	9	23	0.91	0.98	1.10	1.12	1.18	0.84	0.88	0.82	0.88	0.91	0.96	0.86	0.92	72.91	7.36
Q9CT10	Ran-binding protein 3 OS=Mus musculus GN=Ranbp3 PE=1 SV=2 - [RANB3_MOUSE]	31.98	1	13	13	41	1.00	0.90	0.88	0.89	0.89	0.72	0.77	0.82	0.80	0.71	0.69	0.68	0.71	52.54	5.12
D3YVU0	Ubiquitin carboxyl-terminal hydrolase 46 OS=Mus musculus GN=Usp46 PE=2 SV=1 - [D3YVU0_MOUSE]	12.39	2	2	3	5	0.98	0.72	0.74	0.94	0.96	0.79	0.80	0.82	0.83	0.81	0.83	0.87	0.89	39.47	7.36
Q99M80-4	Isoform 4 of Receptor-type tyrosine-protein phosphatase T OS=Mus musculus GN=Ptprt - [PTPRT_MOUSE]	8.78	6	9	9	20	0.99	1.05	1.02	0.91	0.93	0.83	0.84	0.82	0.85	0.85	0.86	0.86	0.89	161.07	6.84
P08249	Malate dehydrogenase, mitochondrial OS=Mus musculus GN=Mdh2 PE=1 SV=3 - [MDHM_MOUSE]	69.53	1	26	26	537	0.85	0.93	1.07	0.96	1.13	0.87	1.02	0.82	0.95	0.82	0.97	0.85	0.99	35.59	8.68
Q8BTZ4-2	Isoform 2 of Anaphase-promoting complex subunit 5 OS=Mus musculus GN=Anapc5 - [APC5_MOUSE]	3.44	2	2	2	3	0.78	1.13	1.46	0.96	1.24	0.84	1.08	0.82	1.05	1.10	1.42	0.96	1.25	81.64	6.71
Q8K2Q7	BRO1 domain-containing protein BROX OS=Mus musculus GN=Brox PE=2 SV=1 - [BROX_MOUSE]	3.89	1	2	2	8	1.25	1.07	0.85	1.27	1.01	1.02	0.81	0.82	0.65	1.01	0.80	1.17	0.93	46.17	7.69
Q91ZP9	N-terminal EF-hand calcium-binding protein 2 OS=Mus musculus GN=Necab2 PE=1 SV=1 - [NECA2_MOUSE]	39.07	3	13	13	24	1.15	1.20	0.95	1.03	0.91	0.85	0.71	0.82	0.68	0.91	0.79	1.13	1.00	43.41	5.30
Q3THS6	S-adenosylmethionine synthase isoform type-2 OS=Mus musculus GN=Mat2a PE=2 SV=2 - [METK2_MOUSE]	26.84	1	9	9	31	1.01	0.99	0.91	0.99	0.96	0.91	0.88	0.82	0.77	0.87	0.82	0.88	0.84	43.66	6.48
E9Q1F5	Protein Myo5c OS=Mus musculus GN=Myo5c PE=2 SV=1 - [E9Q1F5_MOUSE]	5.05	2	1	6	21	0.90	1.00	1.11	1.09	1.21	0.82	0.91	0.82	0.91	0.97	1.07	1.09	1.21	202.57	7.59

E9Q2V1	Carbonic anhydrase-related protein 10 OS=Mus musculus GN=Car10 PE=2 SV=1 - [E9Q2V1_MOUSE]	22.70	4	4	4	11	0.83	1.06	1.18	0.95	1.18	0.71	0.87	0.82	1.05	0.73	0.98	0.78	0.89	34.93	8.09
Q9CZS1	Aldehyde dehydrogenase X, mitochondrial OS=Mus musculus GN=Aldh1b1 PE=2 SV=1 - [AL1B1_MOUSE]	43.35	4	14	16	32	0.98	0.89	0.83	1.04	0.96	0.89	0.91	0.82	0.84	0.95	0.95	1.01	1.01	57.52	7.02
Q91WU5	Arsenite methyltransferase OS=Mus musculus GN=As3mt PE=2 SV=2 - [AS3MT_MOUSE]	10.90	1	3	3	7	1.02	1.00	0.98	0.89	0.86	0.87	0.86	0.82	0.77	0.92	0.88	0.94	0.89	41.77	5.86
D6RG44	Transmembrane protein 126A OS=Mus musculus GN=Tmem126a PE=4 SV=1 - [D6RG44_MOUSE]	35.96	1	1	2	3	0.87	0.79	0.90	0.96	1.10	1.06	1.20	0.82	0.93	0.67	0.76	0.86	0.98	9.78	9.60
E9PWK1	Epoxide hydrolase 1 OS=Mus musculus GN=Ephx1 PE=2 SV=1 - [E9PWK1_MOUSE]	9.30	3	4	4	17	0.93	1.17	1.28	1.11	1.18	0.81	0.86	0.82	0.86	0.92	0.98	0.96	1.04	50.94	8.15
B9EJ80	PDZ domain containing 8 OS=Mus musculus GN=Pdzd8 PE=2 SV=1 - [B9EJ80_MOUSE]	5.49	1	5	5	12	0.91	0.98	1.15	1.09	1.17	0.97	1.12	0.82	0.98	0.95	1.07	0.83	1.05	127.66	6.04
H3BJD6	Protein Ppp1r9a OS=Mus musculus GN=Ppp1r9a PE=2 SV=1 - [H3BJD6_MOUSE]	40.63	7	1	40	216	1.05	0.99	0.94	1.01	0.96	1.01	0.95	0.82	0.78	1.24	1.18	1.09	1.04	144.31	5.55
P70662-2	Isoform 2 of LIM domain-binding protein 1 OS=Mus musculus GN=Ldb1 - [LDB1_MOUSE]	3.13	4	1	1	1	0.96	1.07	1.12	1.01	1.06	0.84	0.87	0.82	0.85	0.88	0.92	0.98	1.03	36.64	8.63
Q9QXG4	Acetyl-coenzyme A synthetase, cytoplasmic OS=Mus musculus GN=Acss2 PE=1 SV=2 - [ACSA_MOUSE]	4.42	4	2	2	3	1.01	1.19	1.18	1.11	1.10	0.85	0.84	0.82	0.81	0.82	0.81	0.80	0.79	78.81	6.64
P51150	Ras-related protein Rab 7a OS=Mus musculus GN=Rab7a PE=1 SV=2 - [RAB7A_MOUSE]	72.46	1	16	16	74	0.98	0.99	0.97	1.03	1.08	0.81	0.80	0.82	0.82	0.83	0.83	0.84	0.83	23.47	6.70
Q8C7R4	Ubiquitin-like modifier-activating enzyme 6 OS=Mus musculus GN=Uba6 PE=1 SV=1 - [UBA6_MOUSE]	15.00	2	12	12	20	1.05	1.01	0.92	1.06	1.06	0.86	0.87	0.82	0.85	0.94	0.93	0.91	0.88	117.89	6.11

Q3TCN2-2	Isoform 2 of Putative phospholipase B-like 2 OS=Mus musculus GN=Plbd2 - [PLBL2_MOUSE]	5.99	2	2	2	3	1.14	0.77	0.67	0.88	0.77	0.86	0.74	0.82	0.71	0.76	0.66	0.87	0.76	49.90	5.91
Q61542	StAR-related lipid transfer protein 3 OS=Mus musculus GN=Stard3 PE=1 SV=1 - [STAR3_MOUSE]	4.71	1	2	2	4	1.13	1.16	1.03	1.08	0.95	0.91	0.80	0.82	0.72	0.90	0.80	0.96	0.85	50.44	7.49
P21981	Protein-glutamine gamma-glutamyltransferase 2 OS=Mus musculus GN=Tgm2 PE=1 SV=4 - [TGM2_MOUSE]	2.33	1	1	1	2	0.89	0.84	0.94	0.82	0.91	0.78	0.87	0.82	0.92	0.88	0.99	0.74	0.83	77.01	5.10
Q505D1	Serine/threonine-protein phosphatase 6 regulatory ankyrin repeat subunit A OS=Mus musculus GN=Ankrd28 PE=1 SV=1 - [ANR28_MOUSE]	4.08	3	3	3	6	1.03	1.21	1.13	1.24	1.11	0.97	0.94	0.82	0.80	0.90	0.87	1.09	1.10	112.83	6.27
P62259	14-3-3 protein epsilon OS=Mus musculus GN=Ywhae PE=1 SV=1 - [1433E_MOUSE]	86.27	4	22	25	720	0.96	1.01	1.06	1.00	1.06	0.84	0.89	0.82	0.87	0.83	0.88	0.87	0.91	29.16	4.74
P27048	Small nuclear ribonucleoprotein-associated protein B OS=Mus musculus GN=Snrpb PE=1 SV=1 - [RSMB_MOUSE]	14.72	2	3	3	8	0.86	0.86	1.05	0.91	1.15	0.90	1.08	0.82	1.02	0.95	1.18	0.88	1.05	23.64	10.90
Q922Q4	Pyrroline-5-carboxylate reductase 2 OS=Mus musculus GN=Pycr2 PE=2 SV=1 - [P5CR2_MOUSE]	7.81	1	2	2	5	0.79	0.82	0.90	0.92	1.17	0.80	1.01	0.82	1.01	0.90	1.06	0.70	0.85	33.64	7.77
Q5RKR3	Immunoglobulin superfamily containing leucine-rich repeat protein 2 OS=Mus musculus GN=Islr2 PE=1 SV=1 - [ISLR2_MOUSE]	6.17	2	4	4	7	0.96	1.10	1.11	0.95	1.04	0.88	0.82	0.82	0.76	0.90	0.85	0.93	0.93	79.71	5.49
Q80Y17	Lethal(2) giant larvae protein homolog 1 OS=Mus musculus GN=Llgl1 PE=1 SV=1 - [L2GL1_MOUSE]	13.71	1	9	9	15	0.91	1.01	1.09	0.97	1.13	0.86	0.96	0.82	0.92	0.97	0.93	1.01	1.24	112.55	6.48
Q8CGK3	Lon protease homolog, mitochondrial OS=Mus musculus GN=Lonp1 PE=1 SV=2 - [LONM_MOUSE]	21.92	1	14	14	29	1.08	0.99	0.94	1.15	1.10	0.92	0.85	0.82	0.87	0.86	0.83	0.90	0.86	105.78	6.57
D6RCG2	Dolichyl-phosphate beta-glucosyltransferase OS=Mus musculus GN=Alg5 PE=4 SV=1 - [D6RCG2_MOUSE]	14.29	2	1	1	2	0.87	0.75	0.86	1.06	1.21	0.88	1.01	0.82	0.94	0.92	1.05	0.88	1.01	9.10	5.29

G5E870	E3 ubiquitin-protein ligase TRIP12 OS=Mus musculus GN=Trip12 PE=1 SV=1 - [TRIPC_MOUSE]	0.59	1	1	1	5	1.08	1.16	1.07	1.15	1.06	0.96	0.88	0.82	0.76	0.96	0.89	1.21	1.12	223.99	8.35
Q8CDN6	Thioredoxin-like protein 1 OS=Mus musculus GN=Txnl1 PE=1 SV=3 - [TXNL1_MOUSE]	58.48	1	14	14	42	0.94	0.83	0.88	0.87	0.90	0.77	0.77	0.82	0.84	0.75	0.79	0.84	0.87	32.22	4.96
O08586	Phosphatidylinositol 3,4,5-trisphosphate 3-phosphatase and dual-specificity protein phosphatase PTEN OS=Mus musculus GN=Pten PE=1 SV=1 - [PTEN_MOUSE]	14.14	1	4	4	9	0.99	0.87	0.83	1.04	1.18	0.71	0.95	0.82	0.81	1.00	1.10	0.80	0.98	47.12	6.37
Q9JJ59-2	Isoform 2 of ATP-binding cassette sub-family B member 9 OS=Mus musculus GN=Abcb9 - [ABCB9_MOUSE]	5.35	3	2	3	5	0.88	1.02	1.15	1.14	1.29	0.87	0.98	0.82	0.93	0.85	0.97	0.82	0.94	70.17	7.94
Q9DBH5	Vesicular integral-membrane protein VIP36 OS=Mus musculus GN=Lman2 PE=2 SV=2 - [LMAN2_MOUSE]	20.95	1	6	6	11	1.05	1.01	1.00	0.97	0.99	0.95	0.87	0.82	0.86	0.94	0.89	0.94	0.89	40.40	6.95
Q5DQR4-	Isoform 2 of Syntaxin-binding protein 5-like OS=Mus musculus GN=Stxbp5l - [STB5L_MOUSE]	12.94	4	2	11	18	0.71	0.90	1.25	0.85	1.19	0.75	1.04	0.82	1.15	0.82	1.15	0.69	0.97	125.72	6.65
F8WHY8	Metastasis-associated protein MTA1 OS=Mus musculus GN=Mta1 PE=2 SV=1 - [F8WHY8_MOUSE]	3.87	3	3	3	4	1.07	0.83	0.89	1.13	1.11	0.95	1.03	0.82	0.98	0.90	0.96	1.15	1.07	79.14	9.42
Q99KQ4	Nicotinamide phosphoribosyltransferase OS=Mus musculus GN=Nampt PE=1 SV=1 - [NAMPT_MOUSE]	23.42	1	10	10	20	1.00	0.99	1.05	1.04	1.09	0.94	0.81	0.82	0.87	0.85	0.87	0.93	0.90	55.41	7.15
O55100-2	Isoform 1B of Synaptogyrin-1 OS=Mus musculus GN=Syngr1 - [SNG1_MOUSE]	12.57	2	2	2	23	1.13	1.11	1.05	1.19	1.06	0.86	0.75	0.82	0.72	0.90	0.81	0.99	0.86	21.28	5.26
B2RWJ3	EG381582 protein OS=Mus musculus GN=Tmem240 PE=2 SV=1 - [B2RWJ3_MOUSE]	4.62	1	1	1	1	0.84	1.28	1.53	1.32	1.58	0.83	0.99	0.82	0.98	0.92	1.10	0.86	1.03	19.99	7.80
Q8R3R8	Gamma-aminobutyric acid receptor-associated protein-like 1 OS=Mus musculus GN=Gabarapl1 PE=1 SV=2 - [GBRL1_MOUSE]	35.04	1	2	5	27	0.61	0.82	1.23	0.89	1.29	0.71	1.11	0.82	1.35	0.79	1.17	0.64	0.94	14.04	8.73

Q921G8	Gamma-tubulin complex component 2 OS=Mus musculus GN=Tubgcp2 PE=2 SV=2 - [GCP2_MOUSE]	3.65	1	2	2	3	0.81	1.31	1.61	1.18	1.45	0.86	1.06	0.82	1.01	0.85	1.05	0.88	1.09	103.16	6.77
P61294	Ras-related protein Rab 6B OS=Mus musculus GN=Rab6b PE=1 SV=1 - [RAB6B_MOUSE]	52.40	10	6	10	79	1.08	1.05	0.99	0.97	0.97	0.79	0.78	0.82	0.75	0.85	0.79	0.94	0.96	23.45	5.53
P51660	Peroxisomal multifunctional enzyme type 2 OS=Mus musculus GN=Hsd17b4 PE=1 SV=3 - [DHB4_MOUSE]	19.05	1	10	10	24	0.94	1.09	1.14	0.99	1.02	0.84	0.83	0.82	0.89	0.88	0.89	0.96	0.98	79.43	8.57
Q6ZQ29-2	Isoform 2 of Serine/threonine-protein kinase TAO2 OS=Mus musculus GN=Taok2 - [TAOK2_MOUSE]	10.33	2	5	6	11	0.72	0.97	1.43	0.82	1.20	0.92	1.38	0.82	1.31	0.96	1.33	1.01	1.41	119.88	7.28
A6H5Y3	Methionine synthase OS=Mus musculus GN=Mtr PE=2 SV=1 - [METH_MOUSE]	0.80	1	1	1	2	0.91	1.25	1.37	1.16	1.28	0.77	0.84	0.82	0.90	1.03	1.13	1.18	1.30	138.98	5.57
Q91VK4	Integral membrane protein 2C OS=Mus musculus GN=Itm2c PE=1 SV=2 - [ITM2C_MOUSE]	27.88	1	5	5	8	1.21	1.00	0.77	1.11	1.00	0.97	0.80	0.82	0.68	1.00	0.79	0.93	0.80	30.46	8.59
Q8C031	Leucine-rich repeat-containing protein 4C OS=Mus musculus GN=Lrrc4c PE=1 SV=2 - [LRC4C_MOUSE]	5.16	1	3	3	7	1.00	1.27	1.24	1.11	1.20	0.91	0.91	0.82	0.89	0.91	0.91	0.88	0.93	71.95	7.15
Q9D4C9	Clavesin-1 OS=Mus musculus GN=Clvs1 PE=2 SV=1 - [CLVS1_MOUSE]	14.12	3	2	4	7	0.88	0.84	0.96	0.90	1.02	0.92	1.04	0.82	0.93	0.90	1.03	0.82	0.93	40.59	6.73
A2AGS6	Tumor protein p53-inducible protein 11 (Fragment) OS=Mus musculus GN=Trp53i11 PE=2 SV=1 - [A2AGS6_MOUSE]	29.89	2	3	3	12	1.04	1.00	1.00	0.90	0.96	0.81	0.80	0.82	0.80	0.79	0.78	0.86	0.83	9.59	9.60
Q3TBW2	39S ribosomal protein L10, mitochondrial OS=Mus musculus GN=Mrpl10 PE=2 SV=2 - [RM10_MOUSE]	8.78	1	1	1	1	0.85	0.97	1.14	1.36	1.61	1.03	1.21	0.82	0.97	1.26	1.48	0.85	1.00	29.38	9.69
Q9D6D0	Protein Slc25a27 OS=Mus musculus GN=Slc25a27 PE=2 SV=1 - [Q9D6D0_MOUSE]	9.63	1	3	3	5	1.04	1.37	1.31	1.18	1.04	0.82	0.74	0.82	0.79	0.85	0.82	0.96	0.93	35.77	9.39
Q8BG51	Mitochondrial Rho GTPase 1 OS=Mus musculus GN=Rhot1 PE=2 SV=1 - [MIRO1_MOUSE]	14.42	8	6	6	9	1.06	1.10	1.12	1.16	1.17	0.82	0.83	0.82	0.81	0.97	0.91	0.94	0.93	72.20	6.49

P98086	Complement C1q subcomponent subunit A OS=Mus musculus GN=C1qa PE=1 SV=2 - [C1QA_MOUSE]	21.63	1	3	3	3	1.07	0.74	0.68	1.00	0.88	0.86	0.80	0.82	0.87	0.82	0.77	0.84	0.79	25.96	9.11
Q62095	ATP-dependent RNA helicase DDX3Y OS=Mus musculus GN=Ddx3y PE=1 SV=2 - [DDX3Y_MOUSE]	36.02	3	5	20	44	1.12	0.96	0.96	1.05	1.05	1.01	0.92	0.82	0.81	0.97	0.98	0.97	0.99	73.38	7.53
P97494	Glutamate--cysteine ligase catalytic subunit OS=Mus musculus GN=Gclc PE=2 SV=4 - [GSH1_MOUSE]	16.01	1	8	8	21	1.01	0.95	0.96	1.04	1.04	0.94	0.91	0.82	0.77	0.92	0.90	0.93	0.91	72.53	5.90
Q6ZWN5	40S ribosomal protein S9 OS=Mus musculus GN=Rps9 PE=2 SV=3 - [RS9_MOUSE]	31.44	6	8	8	13	1.11	1.00	0.91	1.14	1.03	0.91	0.83	0.82	0.78	0.96	0.87	0.99	0.95	22.58	10.65
Q9DBG7	Signal recognition particle receptor subunit alpha OS=Mus musculus GN=Srpr PE=1 SV=1 - [SRPR_MOUSE]	2.20	1	1	1	1	0.85	0.93	1.10	1.07	1.26	0.92	1.09	0.82	0.97	0.95	1.12	0.92	1.09	69.58	8.95
Q62443	Neuronal pentraxin-1 OS=Mus musculus GN=Nptx1 PE=2 SV=1 - [NPTX1_MOUSE]	32.41	1	12	13	21	0.92	0.88	1.04	0.94	1.02	0.92	1.06	0.82	1.08	0.75	1.05	0.75	0.91	47.09	6.44
A2AFS3-3	Isoform 3 of UPF0577 protein KIAA1324 OS=Mus musculus GN=Kiaa1324 - [K1324_MOUSE]	1.64	3	1	1	2	0.95	1.07	1.12	1.19	1.25	0.92	0.96	0.83	0.86	0.95	1.00	0.98	1.03	86.66	7.40
E0CX81	Glucose 1,6-bisphosphate synthase OS=Mus musculus GN=Pgm2l1 PE=2 SV=1 - [E0CX81_MOUSE]	43.08	1	1	10	26	0.96	1.27	1.32	1.04	1.07	0.98	1.01	0.83	0.85	1.30	1.35	0.94	0.98	28.86	7.15
P97950	Ras-related protein Rab-33A OS=Mus musculus GN=Rab33a PE=2 SV=1 - [RB33A_MOUSE]	16.03	1	3	3	4	0.85	1.01	1.04	0.94	1.11	0.93	1.08	0.83	0.97	0.78	0.92	0.94	0.85	26.52	7.88
Q5ICG5	Long chain acyl-CoA synthetase 6 isoform 3 OS=Mus musculus GN=Acsl6 PE=2 SV=1 - [Q5ICG5_MOUSE]	40.32	11	25	25	60	0.93	0.99	1.07	1.09	1.21	0.81	0.90	0.83	0.91	0.85	0.92	0.84	0.94	77.92	7.24
Q80XI4	Phosphatidylinositol 5-phosphate 4-kinase type-2 beta OS=Mus musculus GN=Pip4k2b PE=1 SV=1 - [PI42B_MOUSE]	27.64	1	6	9	58	1.03	1.07	1.09	1.04	1.03	0.85	0.80	0.83	0.83	0.83	0.86	0.85	0.83	47.29	7.33

O08532-5	Isoform 2E of Voltage-dependent calcium channel subunit alpha-2/delta-1 OS=Mus musculus GN=Cacna2d1 - [CA2D1_MOUSE]	42.07	3	2	34	153	0.86	0.83	0.88	0.90	1.00	0.98	1.03	0.83	1.08	0.94	1.14	0.93	1.03	123.18	5.27
Q9R0N5	Synaptotagmin-5 OS=Mus musculus GN=Syt5 PE=1 SV=1 - [SYT5_MOUSE]	13.47	1	3	6	35	1.15	1.06	0.92	1.05	0.99	0.83	0.73	0.83	0.77	1.01	0.88	0.91	0.86	43.10	9.60
Q9D328	Transmembrane protein 35 OS=Mus musculus GN=Tmem35 PE=2 SV=1 - [TMM35_MOUSE]	18.56	2	4	4	7	1.24	1.12	0.99	1.20	1.00	0.94	0.80	0.83	0.62	1.03	0.88	1.16	0.99	18.49	9.99
G3UX05	Protein Gstt3 OS=Mus musculus GN=Gstt3 PE=2 SV=1 - [G3UX05_MOUSE]	14.00	4	2	2	3	0.96	1.34	1.39	0.97	1.01	0.83	0.85	0.83	0.85	0.97	1.00	1.01	1.05	22.67	7.37
Q922Q1	MOSC domain-containing protein 2, mitochondrial OS=Mus musculus GN=Marc2 PE=1 SV=1 - [MOSC2_MOUSE]	25.15	6	7	7	12	0.94	0.70	0.84	0.82	0.91	0.76	0.82	0.83	0.80	0.67	0.70	0.68	0.71	38.17	8.68
P35283	Ras-related protein Rab 12 OS=Mus musculus GN=Rab12 PE=1 SV=3 - [RAB12_MOUSE]	44.44	11	9	10	73	1.09	0.95	1.21	1.07	1.00	0.91	0.87	0.83	0.94	0.88	1.03	0.91	0.96	27.31	8.41
B1AT36	26S proteasome non-ATPase regulatory subunit 12 OS=Mus musculus GN=Psmd12 PE=2 SV=1 - [B1AT36_MOUSE]	31.19	3	11	11	26	1.00	1.05	0.95	1.11	1.04	0.92	0.94	0.83	0.81	0.97	0.94	1.01	0.99	50.54	7.36
Q8C1A5	Thimet oligopeptidase OS=Mus musculus GN=Thop1 PE=1 SV=1 - [THOP1_MOUSE]	31.00	1	16	16	43	0.94	1.00	1.07	1.00	1.10	0.82	0.87	0.83	0.84	0.86	0.90	0.92	0.99	77.98	6.06
P11276	Fibronectin OS=Mus musculus GN=Fn1 PE=1 SV=4 - [FINC_MOUSE]	9.25	1	16	16	39	1.01	1.00	1.00	0.88	0.86	0.75	0.77	0.83	0.82	0.81	0.81	0.73	0.70	272.37	5.59
P62331	ADP-ribosylation factor 6 OS=Mus musculus GN=Arf6 PE=1 SV=2 - [ARF6_MOUSE]	32.57	4	4	4	12	0.99	0.83	0.79	0.99	0.96	0.88	0.89	0.83	0.79	0.87	0.86	0.87	0.82	20.07	8.95
A2A701	Eukaryotic translation initiation factor 3 subunit M (Fragment) OS=Mus musculus GN=Eif3m PE=4 SV=1 - [A2A701_MOUSE]	12.59	3	1	1	2	1.04	1.11	1.06	0.98	0.93	0.91	0.87	0.83	0.79	0.90	0.87	1.04	1.00	16.51	9.19
Q921C1	Gap junction gamma-3 protein OS=Mus musculus GN=Gjc3 PE=2 SV=2 - [CXG3_MOUSE]	4.46	1	1	1	2	1.16	1.03	0.89	1.06	0.91	0.78	0.67	0.83	0.71	0.79	0.68	0.88	0.76	30.27	8.37

P49446-3	Isoform 3 of Receptor-type tyrosine-protein phosphatase epsilon OS=Mus musculus GN=Ptpre - [PTPRE_MOUSE]	8.46	3	3	4	9	1.10	1.34	1.34	0.98	1.03	0.91	0.87	0.83	0.79	0.94	0.92	1.09	1.13	71.42	6.83
Q7TNF9-2	Isoform 2 of Protein FAM117A OS=Mus musculus GN=Fam117a - [F117A_MOUSE]	2.20	2	1	1	2	0.94	0.91	0.96	0.90	0.95	1.16	1.22	0.83	0.87	1.17	1.24	1.30	1.38	43.73	8.32
Q5SVE1	Disintegrin and metalloproteinase domain-containing protein 23 OS=Mus musculus GN=Adam23 PE=2 SV=1 - [Q5SVE1_MOUSE]	28.84	7	12	12	23	1.00	0.94	1.05	1.07	1.02	0.95	0.94	0.83	0.81	0.91	0.92	0.96	1.02	71.63	7.40
F6V8A7	Protein RMD5 homolog A (Fragment) OS=Mus musculus GN=Rmnd5a PE=4 SV=1 - [F6V8A7_MOUSE]	4.13	2	1	1	1	0.86	1.09	1.26	1.06	1.23	0.82	0.95	0.83	0.96	0.80	0.94	1.04	1.21	24.58	7.61
B1AY13	Ubiquitin carboxyl-terminal hydrolase 24 OS=Mus musculus GN=Usp24 PE=1 SV=1 - [UBP24_MOUSE]	0.88	3	1	2	3	0.86	1.37	1.58	1.03	1.19	0.90	1.03	0.83	0.95	1.11	1.29	1.24	1.44	293.81	6.19
P47199	Quinone oxidoreductase OS=Mus musculus GN=Cryz PE=2 SV=1 - [QOR_MOUSE]	43.20	5	8	9	23	1.05	0.83	0.77	1.08	0.97	0.96	0.89	0.83	0.78	0.97	0.92	1.08	1.07	35.25	8.07
P14824	Annexin A6 OS=Mus musculus GN=Anxa6 PE=1 SV=3 - [ANXA6_MOUSE]	50.97	2	29	30	69	0.95	0.95	1.02	0.99	1.05	0.90	0.90	0.83	0.86	0.88	0.94	0.95	0.96	75.84	5.50
Q8VBZ3	Cleft lip and palate transmembrane protein 1 homolog OS=Mus musculus GN=Clptm1 PE=1 SV=1 - [CLPT1_MOUSE]	6.17	1	3	3	4	1.00	0.93	1.02	1.02	1.02	0.83	0.90	0.83	0.82	0.93	1.04	1.10	1.15	75.24	6.30
P55258	Ras-related protein Rab 8A OS=Mus musculus GN=Rab8a PE=1 SV=2 - [RAB8A_MOUSE]	29.47	11	1	7	61	0.88	1.04	1.18	0.86	0.97	0.60	0.68	0.83	0.93	1.09	1.24	0.88	1.00	23.65	9.07
Q8VBT0	Thioredoxin-related transmembrane protein 1 OS=Mus musculus GN=Tmx1 PE=1 SV=1 - [TMX1_MOUSE]	14.03	2	4	4	6	1.14	0.87	0.69	1.15	1.00	0.98	0.83	0.83	0.75	1.00	0.85	1.12	0.89	31.38	5.29
Q61423	Potassium voltage-gated channel subfamily A member 4 OS=Mus musculus GN=Kcna4 PE=1 SV=2 - [KCNA4_MOUSE]	11.16	1	5	6	21	1.05	0.96	0.91	1.06	0.98	0.88	0.83	0.83	0.77	0.86	0.80	1.00	0.94	73.42	5.29

Q9CZX0	Elongator complex protein 3 OS=Mus musculus GN=Elp3 PE=2 SV=1 - [ELP3_MOUSE]	8.78	2	4	4	6	1.02	1.18	1.17	1.16	1.13	0.92	0.90	0.83	0.79	1.01	1.03	1.08	1.01	62.35	8.90
E9Q8I9	Protein furry homolog OS=Mus musculus GN=Fry PE=1 SV=1 - [FRY_MOUSE]	2.19	2	3	4	8	0.78	1.11	1.43	1.21	1.45	0.88	1.11	0.83	1.19	0.97	1.17	1.05	1.43	338.88	6.01
P52480-2	Isoform M1 of Pyruvate kinase PKM OS=Mus musculus GN=Pkm - [KPYM_MOUSE]	79.66	4	4	46	878	0.86	1.09	1.29	1.03	1.20	0.86	0.98	0.83	0.94	0.84	0.95	0.89	1.06	57.95	7.14
Q80UW8	DNA-directed RNA polymerases I, II, and III subunit RPABC1 OS=Mus musculus GN=Polr2e PE=2 SV=1 - [RPAB1_MOUSE]	16.67	1	3	3	6	0.96	0.81	0.85	0.87	0.89	0.75	0.74	0.83	0.89	0.67	0.72	0.68	0.77	24.55	5.95
P61750	ADP-ribosylation factor 4 OS=Mus musculus GN=Arf4 PE=2 SV=2 - [ARF4_MOUSE]	60.56	5	3	8	42	1.08	0.87	0.82	1.09	1.01	0.88	0.81	0.83	0.77	0.90	0.87	0.96	0.92	20.38	7.14
Q8R0Y6	Cytosolic 10-formyltetrahydrofolate dehydrogenase OS=Mus musculus GN=Aldh1l1 PE=2 SV=1 - [AL1L1_MOUSE]	44.12	1	28	31	85	0.92	0.90	0.96	1.05	1.09	0.92	0.94	0.83	0.91	0.92	0.99	0.92	0.98	98.65	5.91
D3Z1B2	MCG50540 OS=Mus musculus GN=Gm4953 PE=4 SV=1 - [D3Z1B2_MOUSE]	18.63	1	1	3	6	0.88	1.06	1.21	0.93	1.06	1.00	1.14	0.83	0.94	0.89	1.02	0.78	0.89	18.51	5.44
A2ABY3	Ethanolamine-phosphate cytidylyltransferase OS=Mus musculus GN=Pcyt2 PE=4 SV=1 - [A2ABY3_MOUSE]	21.50	2	7	7	22	1.06	0.93	0.99	0.94	0.95	0.78	0.80	0.83	0.89	0.81	0.87	0.95	0.88	43.42	6.73
B2M1R6	Heterogeneous nuclear ribonucleoprotein K OS=Mus musculus GN=Hnrnpk PE=2 SV=1 - [B2M1R6_MOUSE]	56.82	8	1	22	304	0.98	0.75	0.84	0.65	0.95	0.87	1.03	0.83	0.89	0.82	0.92	0.69	0.79	48.53	5.54
P46638	Ras-related protein Rab 11B OS=Mus musculus GN=Rab11b PE=1 SV=3 - [RB11B_MOUSE]	63.76	12	14	14	71	1.02	0.93	0.94	1.04	1.03	0.88	0.82	0.83	0.81	0.84	0.80	0.90	0.84	24.47	5.94
Q9JI91	Alpha-actinin-2 OS=Mus musculus GN=Actn2 PE=1 SV=2 - [ACTN2_MOUSE]	45.97	5	20	36	150	0.97	1.02	1.03	1.07	1.17	1.03	1.10	0.83	0.90	0.97	0.98	1.13	1.14	103.77	5.45
Q8C7H1	Methylmalonic aciduria type A homolog, mitochondrial OS=Mus musculus GN=Mmaa PE=2 SV=1 - [MMAA_MOUSE]	3.13	1	1	1	2	0.98	1.00	1.02	1.00	1.02	0.69	0.70	0.83	0.84	0.93	0.95	0.94	0.97	45.90	9.32

P98200	Probable phospholipid-transporting ATPase IB OS=Mus musculus GN=Atp8a2 PE=1 SV=1 - [AT8A2_MOUSE]	3.75	5	2	4	6	1.01	1.04	1.02	1.02	1.01	0.81	0.80	0.83	0.82	0.88	0.87	0.99	0.99	129.33	7.33
Q99KK2	N-acylneuraminate cytidylyltransferase OS=Mus musculus GN=Cmas PE=1 SV=2 - [NEUA_MOUSE]	18.98	2	7	7	13	0.84	0.82	1.00	1.02	1.10	0.89	0.91	0.83	0.94	0.97	1.00	0.93	0.98	48.03	8.10
Q32P14	Predicted gene, OTTMUSG00000010173 OS=Mus musculus GN=Gm13051 PE=2 SV=1 - [Q32P14_MOUSE]	20.06	168	1	2	5	0.63	0.93	1.46	0.95	1.49	1.00	1.57	0.83	1.30	0.66	1.05	0.99	1.56	74.16	8.88
Q9CQN1	Heat shock protein 75 kDa, mitochondrial OS=Mus musculus GN=Trap1 PE=1 SV=1 - [TRAP1_MOUSE]	22.38	1	11	12	57	0.98	1.27	1.17	1.21	1.24	0.79	0.81	0.83	0.84	0.92	0.92	0.97	0.96	80.16	6.68
Q8R2T7	Oxysterol-binding protein OS=Mus musculus GN=Osbpl10 PE=2 SV=1 - [Q8R2T7_MOUSE]	4.74	1	2	2	3	1.18	1.44	1.23	1.15	0.98	1.07	0.90	0.83	0.70	1.31	1.12	1.07	0.91	65.76	7.39
Q8C8R3-4	Isoform 4 of Ankyrin-2 OS=Mus musculus GN=Ank2 - [ANK2_MOUSE]	58.83	6	5	160	1011	0.96	0.87	1.19	0.96	0.97	0.98	1.05	0.83	0.93	0.88	0.94	0.97	0.92	432.60	5.16
P32921-2	Isoform 2 of Tryptophan--tRNA ligase, cytoplasmic OS=Mus musculus GN=Wars - [SYWC_MOUSE]	27.79	2	10	10	23	1.03	0.88	0.95	1.05	1.03	0.97	0.93	0.83	0.76	0.94	0.94	0.93	0.87	53.61	7.08
E9Q1N6	Protein Rasgef1a OS=Mus musculus GN=Rasgef1a PE=2 SV=1 - [E9Q1N6_MOUSE]	1.64	1	1	1	1	1.90	1.77	0.93	0.88	0.46	1.07	0.56	0.83	0.43	1.16	0.61	0.96	0.51	55.79	8.02
A2AS98	Nck-associated protein 1 OS=Mus musculus GN=Nckap1 PE=2 SV=1 - [A2AS98_MOUSE]	25.31	4	24	24	57	0.98	0.88	0.90	1.09	1.15	0.84	0.85	0.83	0.81	0.85	0.89	0.90	0.96	129.43	6.68
E0CY49	Argininosuccinate lyase (Fragment) OS=Mus musculus GN=Asl PE=2 SV=1 - [E0CY49_MOUSE]	22.90	4	5	6	12	0.93	0.87	0.93	1.01	1.08	1.01	0.90	0.83	0.89	1.05	1.12	1.02	1.03	29.46	6.96
E9QP46	Nesprin-2 OS=Mus musculus GN=Syne2 PE=2 SV=1 - [E9QP46_MOUSE]	0.54	6	2	4	5	1.23	1.35	1.10	1.16	0.94	0.63	0.51	0.83	0.67	0.57	0.47	0.52	0.43	781.66	5.31
Q8C0Z1	Protein ITFG3 OS=Mus musculus GN=Itfg3 PE=1 SV=1 - [ITFG3_MOUSE]	1.98	1	1	1	2	0.91	0.59	0.65	1.01	1.11	0.81	0.88	0.83	0.91	0.75	0.82	0.74	0.82	60.54	5.68

G3XA00	Glutamate receptor, metabotropic 4 OS=Mus musculus GN=Grm4 PE=3 SV=1 - [G3XA00_MOUSE]	4.50	4	2	3	4	2.33	22.43	4.27	1.74	0.74	2.23	0.95	0.83	0.75	1.17	0.50	1.48	0.63	101.71	8.85
P27612	Phospholipase A-2-activating protein OS=Mus musculus GN=Plaa PE=2 SV=4 - [PLAP_MOUSE]	18.89	2	12	12	26	0.95	1.02	1.06	1.06	1.11	0.89	0.91	0.83	0.84	0.87	0.88	0.90	0.93	87.17	6.14
Q61830	Macrophage mannose receptor 1 OS=Mus musculus GN=Mrc1 PE=1 SV=2 - [MRC1_MOUSE]	0.82	1	1	1	2	0.85	1.30	1.51	0.79	0.92	0.82	0.96	0.83	0.97	0.83	0.97	0.85	1.00	164.87	6.83
Q9CPU4	Microsomal glutathione S-transferase 3 OS=Mus musculus GN=Mgst3 PE=1 SV=1 - [MGST3_MOUSE]	32.03	1	3	3	22	0.94	1.00	1.18	1.13	1.23	0.92	1.03	0.83	0.88	0.88	0.99	0.86	0.93	16.95	9.50
D3YVK0	Acyl-CoA:lysophosphatidylglycerol acyltransferase 1 (Fragment) OS=Mus musculus GN=Lpgat1 PE=2 SV=1 - [D3YVK0_MOUSE]	5.43	3	1	1	1	0.83	0.97	1.16	1.07	1.29	0.69	0.82	0.83	0.99	0.82	0.99	0.82	0.99	25.73	9.39
D3Z580	Protein Celf5 OS=Mus musculus GN=Celf5 PE=2 SV=1 - [D3Z580_MOUSE]	4.31	5	1	2	3	0.72	1.16	1.60	0.81	1.12	0.80	1.10	0.83	1.14	1.02	1.41	0.91	1.26	42.52	7.90
Q04859-3	Isoform 3 of Serine/threonine-protein kinase MAK OS=Mus musculus GN=Mak - [MAK_MOUSE]	3.09	51	1	2	5	0.99	1.55	1.57	1.19	1.21	1.14	1.15	0.83	0.84	1.03	1.04	1.02	1.04	62.09	9.58
Q9JIZ9	Phospholipid scramblase 3 OS=Mus musculus GN=Plscr3 PE=1 SV=1 - [PLS3_MOUSE]	15.88	2	3	3	6	1.01	1.12	1.11	1.04	1.02	0.88	0.88	0.83	0.83	0.95	0.94	1.16	1.15	31.78	6.38
Q8C163	Nuclease EXOG, mitochondrial OS=Mus musculus GN=Exog PE=2 SV=1 - [EXOG_MOUSE]	16.58	2	5	5	9	0.98	0.96	0.99	1.19	1.18	0.97	0.93	0.83	0.89	0.96	0.98	0.98	1.03	41.36	8.12
B1AU22	Fibroblast growth factor 13 (Fragment) OS=Mus musculus GN=Fgf13 PE=2 SV=1 - [B1AU22_MOUSE]	37.21	3	1	1	2	1.11	1.51	1.36	0.93	0.83	0.74	0.66	0.83	0.74	0.64	0.57	0.56	0.50	4.79	6.65
Q9Z2D0	Myotubularin-related protein 9 OS=Mus musculus GN=Mtmr9 PE=2 SV=2 - [MTMR9_MOUSE]	12.11	1	5	5	11	1.05	1.18	1.13	0.95	1.01	0.97	0.96	0.83	0.81	0.91	0.91	0.99	1.05	62.87	6.62

Q9DBC3	Cap-specific mRNA (nucleoside-2'-O-)-methyltransferase 1 OS=Mus musculus GN=Ftsjd2 PE=1 SV=1 - [MTR1_MOUSE]	4.42	3	3	4	5	1.01	1.34	1.08	1.00	1.05	0.90	0.98	0.83	1.01	0.92	0.89	1.10	1.10	95.62	7.27
Q99PU5	Long-chain-fatty-acid--CoA ligase ACSBG1 OS=Mus musculus GN=Acsbg1 PE=1 SV=1 - [ACBG1_MOUSE]	38.00	3	18	18	47	0.98	0.96	0.98	1.04	1.07	0.89	0.91	0.83	0.86	0.92	0.95	0.96	1.04	80.37	5.94
Q8BMJ2	Leucine--tRNA ligase, cytoplasmic OS=Mus musculus GN=Lars PE=2 SV=2 - [SYLC_MOUSE]	22.67	1	22	22	35	0.99	1.07	1.06	1.09	1.14	0.90	0.93	0.83	0.86	0.97	0.98	0.97	1.01	134.11	7.05
Q64735-2	Isoform 2 of Complement component receptor 1-like protein OS=Mus musculus GN=Cr1l - [CR1L_MOUSE]	2.27	2	1	1	1	1.09	0.93	0.85	1.34	1.23	0.85	0.78	0.83	0.76	0.87	0.80	0.92	0.84	49.04	5.73
Q91XE8	Transmembrane protein 205 OS=Mus musculus GN=Tmem205 PE=1 SV=1 - [TM205_MOUSE]	8.99	1	1	1	2	0.84	1.04	1.24	1.07	1.28	0.85	1.01	0.83	0.99	0.86	1.03	0.98	1.17	21.17	9.42
Q9CYN2	Signal peptidase complex subunit 2 OS=Mus musculus GN=Spcs2 PE=2 SV=1 - [SPCS2_MOUSE]	28.76	1	3	3	4	1.10	1.24	1.13	1.01	0.92	1.00	0.91	0.83	0.75	1.05	0.96	1.05	0.96	24.96	8.57
Q9ET01	Glycogen phosphorylase, liver form OS=Mus musculus GN=Pygl PE=1 SV=4 - [PYGL_MOUSE]	8.47	2	2	8	29	0.95	0.98	1.03	1.00	1.06	0.95	1.00	0.83	0.87	0.99	1.05	1.05	1.10	97.40	7.09
Q91WA3	Histone deacetylase 11 OS=Mus musculus GN=Hdac11 PE=2 SV=1 - [HDA11_MOUSE]	8.93	3	3	3	4	1.07	1.24	1.16	1.09	0.97	0.83	0.79	0.83	0.74	0.90	0.80	1.13	1.06	39.13	7.14
Q3UYV9	Nuclear cap-binding protein subunit 1 OS=Mus musculus GN=Ncbp1 PE=1 SV=2 - [NCBP1_MOUSE]	3.92	1	2	2	4	0.76	0.98	1.29	0.83	1.09	0.70	0.92	0.83	1.10	0.81	1.08	0.80	1.06	91.87	6.64
Q9D2R0	Acetoacetyl-CoA synthetase OS=Mus musculus GN=Aacs PE=1 SV=1 - [AACS_MOUSE]	3.87	1	3	3	5	1.12	1.28	1.12	1.07	1.01	1.10	0.98	0.83	0.74	1.06	1.01	1.07	0.95	75.15	6.71
Q80ZW2	Protein THEM6 OS=Mus musculus GN=Them6 PE=2 SV=1 - [THEM6_MOUSE]	25.12	1	4	4	13	0.90	1.07	1.11	1.03	1.08	0.81	0.89	0.83	0.89	0.89	0.89	0.86	0.98	23.79	9.11
Q8C0C7	Phenylalanine--tRNA ligase alpha subunit OS=Mus musculus GN=Farsa PE=2 SV=1 - [SYFA_MOUSE]	13.78	2	5	6	11	0.96	0.93	1.02	1.01	1.07	0.91	0.95	0.83	0.92	0.89	0.96	0.92	0.99	57.56	8.28

Q62348	Translin OS=Mus musculus GN=Tsn PE=1 SV=1 - [TSN_MOUSE]	16.67	1	3	3	5	1.01	0.96	0.97	1.11	1.08	0.89	0.88	0.83	0.86	0.94	0.90	0.97	0.91	26.18	6.44
D3YYG8	Uncharacterized protein (Fragment) OS=Mus musculus GN=Fam213a PE=2 SV=1 - [D3YYG8_MOUSE]	37.14	8	6	6	21	1.00	0.84	0.83	1.06	1.05	0.92	0.85	0.83	0.81	0.99	0.95	0.96	0.94	19.71	8.68
Q60809	CCR4-NOT transcription complex subunit 7 OS=Mus musculus GN=Cnot7 PE=1 SV=1 - [CNOT7_MOUSE]	4.91	1	1	1	2	0.86	0.87	1.01	0.93	1.08	0.93	1.08	0.83	0.97	0.89	1.04	0.87	1.01	32.70	4.84
Q91ZW3	SWI/SNF-related matrix-associated actin-dependent regulator of chromatin subfamily A member 5 OS=Mus musculus GN=Smarca5 PE=1 SV=1 - [SMCA5_MOUSE]	7.61	5	6	6	9	0.98	1.06	1.01	1.03	1.05	0.97	0.95	0.83	0.82	0.89	0.98	1.08	1.11	121.55	8.15
Q9DBE0	Cysteine sulfinic acid decarboxylase OS=Mus musculus GN=Csad PE=2 SV=1 - [CSAD_MOUSE]	5.27	1	2	2	4	1.05	1.00	0.95	1.07	1.02	0.96	0.91	0.83	0.79	0.90	0.86	1.05	1.01	55.11	6.61
Q91VF2	Histamine N-methyltransferase OS=Mus musculus GN=Hnmt PE=1 SV=1 - [HNMT_MOUSE]	3.39	1	1	1	1	0.99	0.99	1.00	1.08	1.08	1.02	1.02	0.83	0.83	1.02	1.03	0.89	0.90	33.64	5.06
Q8BXC6	COMM domain-containing protein 2 OS=Mus musculus GN=Commd2 PE=1 SV=1 - [COMD2_MOUSE]	5.03	1	1	1	2	0.92	0.67	0.73	0.96	1.04	0.94	1.02	0.83	0.90	0.95	1.04	0.80	0.87	22.83	6.62
E9PVI4	Netrin receptor UNC5C OS=Mus musculus GN=Unc5c PE=2 SV=1 - [E9PVI4_MOUSE]	0.80	3	1	1	1	0.99	1.11	1.12	0.99	1.00	0.92	0.92	0.83	0.84	0.87	0.88	1.06	1.07	96.57	6.49
P55088-2	Isoform 1 of Aquaporin-4 OS=Mus musculus GN=Aqp4 - [AQP4_MOUSE]	15.28	3	5	5	64	1.27	1.12	0.83	1.05	0.83	0.88	0.72	0.83	0.62	0.87	0.68	0.96	0.77	32.07	6.86
Q78IK4	Apolipoprotein O-like OS=Mus musculus GN=Apool PE=2 SV=1 - [APOOL_MOUSE]	13.21	2	3	3	6	1.01	0.86	0.83	1.06	1.05	0.84	0.84	0.83	0.85	0.94	0.87	0.96	0.89	29.24	9.31
Q8R0Y8	Mitochondrial coenzyme A transporter SLC25A42 OS=Mus musculus GN=Slc25a42 PE=2 SV=1 - [S2542_MOUSE]	7.23	1	2	2	3	1.32	1.44	1.09	1.21	0.91	0.93	0.70	0.83	0.63	0.91	0.69	1.10	0.83	35.22	10.05

Q9Z1S3	RAS guanyl-releasing protein 1 OS=Mus musculus GN=Rasgrp1 PE=1 SV=1 - [GRP1_MOUSE]	4.40	2	2	2	3	0.91	0.59	0.65	0.98	1.08	0.88	0.96	0.83	0.91	0.90	0.99	0.83	0.91	90.25	8.10
Q04690-2	Isoform 1 of Neurofibromin OS=Mus musculus GN=Nf1 - [NF1_MOUSE]	4.15	4	7	10	15	1.09	1.03	1.05	1.08	1.07	0.90	0.83	0.83	0.78	0.94	0.91	1.00	0.94	317.02	7.27
Q8BFZ9	Erlin-2 OS=Mus musculus GN=Erlin2 PE=1 SV=1 - [ERLN2_MOUSE]	26.76	2	7	9	20	1.11	0.98	0.85	1.13	1.01	0.87	0.84	0.83	0.75	0.93	0.84	0.94	0.84	37.85	5.50
Q9WV31	Activity-regulated cytoskeleton-associated protein OS=Mus musculus GN=Arc PE=1 SV=1 - [ARC_MOUSE]	2.78	1	1	1	1	0.87	1.34	1.54	1.06	1.22	0.69	0.79	0.83	0.96	0.78	0.90	0.93	1.07	45.29	5.39
Q8BGD5	Carnitine O-palmitoyltransferase 1, brain isoform OS=Mus musculus GN=Cpt1c PE=1 SV=1 - [CPT1C_MOUSE]	11.15	1	5	5	6	1.08	1.24	1.21	1.24	1.11	0.83	0.75	0.83	0.80	0.97	0.98	0.93	0.97	89.97	7.49
Q99JI6	Ras-related protein Rap 1b OS=Mus musculus GN=Rap1b PE=2 SV=2 - [RAP1B_MOUSE]	67.93	1	5	11	57	1.00	0.81	0.78	1.01	0.99	0.92	0.94	0.83	0.81	0.83	0.82	0.80	0.82	20.81	5.78
Q99JT9	1,2-dihydroxy-3-keto-5-methylthiopentene dioxygenase OS=Mus musculus GN=Adi1 PE=1 SV=1 - [MTND_MOUSE]	7.26	1	1	1	2	0.75	0.78	1.03	0.82	1.09	0.79	1.05	0.83	1.10	0.65	0.86	0.84	1.12	21.51	5.50
Q9EPR5	VPS10 domain-containing receptor SorCS2 OS=Mus musculus GN=Sorcs2 PE=1 SV=2 - [SORC2_MOUSE]	2.16	1	2	2	3	1.07	1.04	0.97	0.97	0.91	0.91	0.85	0.83	0.78	0.96	0.90	1.26	1.19	128.82	7.34
Q9D517	1-acyl-sn-glycerol-3-phosphate acyltransferase gamma OS=Mus musculus GN=Agpat3 PE=1 SV=2 - [PLCC_MOUSE]	15.43	1	5	5	9	1.07	1.03	0.99	1.16	1.12	0.81	0.79	0.83	0.79	0.98	0.91	0.99	0.92	43.27	8.51
P39053-3	Isoform 3 of Dynamin-1 OS=Mus musculus GN=Dnm1 - [DYN1_MOUSE]	69.92	11	2	55	491	1.02	1.13	1.11	1.15	1.15	0.81	0.82	0.83	0.88	0.83	0.85	1.10	1.10	95.87	6.76
Q9Z1Q2	Abhydrolase domain-containing protein 16A OS=Mus musculus GN=Abhd16a PE=1 SV=3 - [ABHGA_MOUSE]	13.62	4	6	6	11	0.98	1.28	1.20	1.11	1.10	0.81	0.84	0.83	0.81	0.85	0.91	0.89	0.88	63.05	8.25

A2RTH5	Leucine carboxyl methyltransferase 1 OS=Mus musculus GN=Lcmt1 PE=2 SV=1 - [A2RTH5_MOUSE]	6.33	1	2	2	4	0.90	0.95	0.89	0.96	1.04	0.85	0.79	0.83	0.78	0.82	0.77	0.93	0.94	38.17	5.62
Q9CXI3	DBH-like monooxygenase protein 1 OS=Mus musculus GN=Moxd1 PE=1 SV=1 - [MOXD1_MOUSE]	1.63	1	1	1	1	1.10	1.24	1.13	1.05	0.95	0.86	0.78	0.83	0.76	0.82	0.75	1.09	0.99	69.63	6.67
O08547	Vesicle-trafficking protein SEC22b OS=Mus musculus GN=Sec22b PE=1 SV=3 - [SC22B_MOUSE]	48.37	2	9	9	25	0.94	1.16	1.20	1.09	1.16	0.88	0.92	0.83	0.93	0.88	0.97	0.89	0.95	24.72	8.51
Q8CI71	Coiled-coil domain-containing protein 132 OS=Mus musculus GN=Ccdc132 PE=1 SV=2 - [CC132_MOUSE]	17.63	5	12	12	24	0.89	1.01	1.15	1.07	1.22	0.83	0.95	0.83	0.92	0.94	1.03	1.06	1.12	111.10	6.07
P17809	Solute carrier family 2, facilitated glucose transporter member 1 OS=Mus musculus GN=Slc2a1 PE=1 SV=4 - [GTR1_MOUSE]	6.91	1	4	4	8	1.15	1.30	1.15	1.06	0.93	0.96	0.78	0.83	0.75	0.90	0.79	0.91	0.76	53.95	8.87
Q61735	Leukocyte surface antigen CD47 OS=Mus musculus GN=Cd47 PE=1 SV=2 - [CD47_MOUSE]	12.21	2	3	3	29	1.00	0.95	0.97	1.08	1.07	0.88	0.85	0.83	0.83	0.91	0.89	0.91	0.93	33.08	8.63
O88322	Nidogen-2 OS=Mus musculus GN=Nid2 PE=1 SV=2 - [NID2_MOUSE]	14.54	1	13	13	30	0.85	1.06	1.25	0.91	1.05	0.81	0.94	0.83	0.97	0.76	0.93	0.79	0.94	153.82	5.38
E9PXL0	Protein FAM222B OS=Mus musculus GN=Fam222b PE=2 SV=1 - [E9PXL0_MOUSE]	2.30	2	1	1	3	1.23	1.12	0.91	0.96	0.78	1.14	0.92	0.84	0.68	1.09	0.89	0.97	0.79	45.49	8.07
Q3V4B5	COMM domain-containing protein 6 OS=Mus musculus GN=Commd6 PE=2 SV=1 - [COMD6_MOUSE]	14.94	1	1	1	2	0.90	0.60	0.67	0.81	0.90	0.94	1.03	0.84	0.92	0.78	0.87	0.99	1.09	9.79	5.38
Q61885	Myelin-oligodendrocyte glycoprotein OS=Mus musculus GN=Mog PE=1 SV=1 - [MOG_MOUSE]	24.80	3	6	6	26	0.92	1.19	1.28	1.10	1.21	0.85	0.91	0.84	0.81	0.79	0.88	0.82	0.90	28.25	7.96
Q7M729	Sodium channel subunit beta-4 OS=Mus musculus GN=Scn4b PE=2 SV=1 - [SCN4B_MOUSE]	3.51	1	1	1	3	0.82	0.87	1.06	1.14	1.38	0.94	1.13	0.84	1.01	1.02	1.24	0.92	1.12	25.18	8.82

Q8BMF3	NADP-dependent malic enzyme, mitochondrial OS=Mus musculus GN=Me3 PE=1 SV=2 - [MAON_MOUSE]	33.77	2	12	13	29	0.94	0.96	1.00	1.04	1.09	0.93	0.93	0.84	0.90	0.94	0.90	0.94	0.99	67.06	7.83
Q921Q3-2	Isoform 2 of Chitobiosyldiphosphodolichol beta-mannosyltransferase OS=Mus musculus GN=Alg1 - [ALG1_MOUSE]	2.85	2	1	1	3	0.93	0.84	0.90	1.13	1.22	0.88	0.94	0.84	0.90	1.00	1.08	1.01	1.10	51.41	8.21
G3X8P9	Aldehyde oxidase OS=Mus musculus GN=Aox1 PE=4 SV=1 - [G3X8P9_MOUSE]	3.68	2	2	2	2	1.06	0.45	0.64	0.78	0.73	0.80	1.12	0.84	1.18	1.07	1.52	0.65	0.93	146.55	6.93
Q8BG32	26S proteasome non-ATPase regulatory subunit 11 OS=Mus musculus GN=Psmd11 PE=1 SV=3 - [PSD11_MOUSE]	39.10	11	13	13	31	0.94	1.02	1.04	0.99	1.11	0.95	1.00	0.84	0.92	0.89	0.95	1.04	1.12	47.41	6.48
E9Q401	Ryanodine receptor 2 OS=Mus musculus GN=Ryr2 PE=1 SV=1 - [RYR2_MOUSE]	7.59	2	26	28	51	0.91	1.00	1.04	1.12	1.27	0.86	0.93	0.84	0.92	0.90	0.98	0.95	1.03	564.46	6.09
D3Z4I0	Protein Pigg OS=Mus musculus GN=Pigg PE=2 SV=1 - [D3Z4I0_MOUSE]	1.44	2	1	1	2	0.99	1.55	1.56	1.20	1.20	0.86	0.86	0.84	0.84	1.21	1.22	1.17	1.17	107.62	7.83
P32507-2	Isoform Alpha of Poliovirus receptor-related protein 2 OS=Mus musculus GN=Pvrl2 - [PVRL2_MOUSE]	3.64	2	1	1	2	0.98	1.14	1.16	1.35	1.37	0.97	0.99	0.84	0.85	0.99	1.01	1.07	1.09	50.75	5.48
P28650	Adenylosuccinate synthetase isozyme 1 OS=Mus musculus GN=Adssl1 PE=1 SV=2 - [PURA1_MOUSE]	28.88	3	9	9	15	0.92	0.95	0.95	0.93	1.03	0.90	0.95	0.84	0.88	0.86	0.91	0.82	0.91	50.22	8.43
O54990-6	Isoform 6 of Prominin-1 OS=Mus musculus GN=Prom1 - [PROM1_MOUSE]	8.87	10	5	5	12	0.88	0.97	1.03	1.09	1.22	0.82	0.94	0.84	0.91	0.84	0.88	0.73	0.84	92.14	6.55
Q8CEB6	1,5-anhydro-D-fructose reductase OS=Mus musculus GN=Akr1e1 PE=2 SV=1 - [Q8CEB6_MOUSE]	29.80	2	5	5	10	1.04	0.85	0.81	0.99	0.95	0.90	0.88	0.84	0.78	0.83	0.82	0.95	1.04	28.25	7.83
P05064	Fructose-bisphosphate aldolase A OS=Mus musculus GN=Aldoa PE=1 SV=2 - [ALDOA_MOUSE]	88.19	6	10	30	898	0.87	0.90	1.02	0.96	1.09	0.85	0.95	0.84	0.94	0.82	0.93	0.85	0.96	39.33	8.09
Q8BHZ0	Protein FAM49A OS=Mus musculus GN=Fam49a PE=2 SV=1 - [FA49A_MOUSE]	32.20	1	5	7	20	0.98	0.96	0.92	1.10	1.12	0.85	0.91	0.84	0.83	0.93	0.95	1.15	1.13	37.32	6.01
Q8C8N2	Protein SCAI OS=Mus musculus GN=Scai PE=1 SV=2 - [SCAI_MOUSE]	36.96	3	18	18	53	0.92	1.04	1.11	1.09	1.10	0.86	0.90	0.84	0.89	0.83	0.89	0.85	0.88	70.23	8.60

Q9CZU6	Citrate synthase, mitochondrial OS=Mus musculus GN=Cs PE=1 SV=1 - [CISY_MOUSE]	48.06	1	10	17	114	0.97	0.99	0.91	1.09	1.09	0.88	0.92	0.84	0.86	0.95	0.97	1.02	1.03	51.70	8.57
Q8CGY8-	Isoform 2 of UDP-N-acetylglucosamine--peptide N-acetylglucosaminyltransferase 110 kDa subunit OS=Mus musculus GN=Ogt - [OGT1_MOUSE]	18.73	2	14	14	29	1.00	0.87	0.88	1.10	1.15	0.92	0.94	0.84	0.85	0.94	0.94	0.97	0.95	115.66	6.65
P12657	Muscarinic acetylcholine receptor M1 OS=Mus musculus GN=Chrm1 PE=2 SV=2 - [ACM1_MOUSE]	5.65	1	2	2	7	0.91	1.05	1.12	1.11	1.25	0.87	0.98	0.84	0.95	0.97	1.12	0.99	1.15	51.35	9.32
Q8CAA7	Glucose 1,6-bisphosphate synthase OS=Mus musculus GN=Pgm2l1 PE=1 SV=1 - [PGM2L_MOUSE]	32.85	1	7	16	56	1.04	1.03	1.09	1.10	1.00	0.90	0.84	0.84	0.80	0.83	0.85	0.96	0.89	70.23	6.49
Q8R1K4	5-phosphohydroxy-L-lysine phospho-lyase OS=Mus musculus GN=Phykpl PE=2 SV=1 - [AT2L2_MOUSE]	5.14	3	2	2	3	0.99	0.90	0.91	0.93	0.93	0.80	0.80	0.84	0.84	0.76	0.76	0.96	0.96	51.93	6.46
G3UWD7	MCG123152 OS=Mus musculus GN=Gm10269 PE=3 SV=1 - [G3UWD7_MOUSE]	7.32	2	1	1	1	1.10	0.68	0.62	1.03	0.94	0.90	0.82	0.84	0.76	0.85	0.78	0.98	0.89	14.58	11.06
P52623	Uridine-cytidine kinase 1 OS=Mus musculus GN=Uck1 PE=2 SV=2 - [UCK1_MOUSE]	25.27	3	5	5	11	1.08	1.06	1.02	0.98	0.95	0.96	0.83	0.84	0.77	1.02	0.89	0.95	0.91	31.05	7.88
Q8CC21-2	Isoform 2 of Tetratricopeptide repeat protein 19, mitochondrial OS=Mus musculus GN=Ttc19 - [TTC19_MOUSE]	13.64	3	3	3	7	1.06	1.08	1.05	0.97	0.91	0.86	0.84	0.84	0.83	0.92	0.94	1.00	0.88	39.75	6.21
Q923X4-2	Isoform 2 of Glutaredoxin-2, mitochondrial OS=Mus musculus GN=Glrx2 - [GLRX2_MOUSE]	40.65	3	4	4	7	0.99	0.63	0.60	0.88	0.88	0.93	0.94	0.84	0.83	0.74	0.80	0.86	0.80	14.03	8.94
Q6PDS3-2	Isoform 2 of Sterile alpha and TIR motif-containing protein 1 OS=Mus musculus GN=Sarm1 - [SARM1_MOUSE]	5.42	4	3	3	7	0.96	1.19	1.34	1.09	1.18	0.90	0.93	0.84	0.89	0.96	0.98	0.99	1.12	78.96	6.44
P63046	Sulfotransferase 4A1 OS=Mus musculus GN=Sult4a1 PE=2 SV=1 - [ST4A1_MOUSE]	18.31	2	4	4	6	0.98	0.89	0.91	1.07	1.07	0.87	0.84	0.84	0.94	0.80	0.83	0.95	0.96	33.03	5.53

Q61626	Glutamate receptor ionotropic, kainate 5 OS=Mus musculus GN=Grik5 PE=2 SV=2 - [GRIK5_MOUSE]	1.02	1	1	1	2	1.00	1.34	1.33	1.13	1.12	0.91	0.90	0.84	0.83	0.91	0.91	1.02	1.02	109.21	8.21
Q3U1J4	DNA damage-binding protein 1 OS=Mus musculus GN=Ddb1 PE=1 SV=2 - [DDB1_MOUSE]	29.39	1	30	30	67	0.99	0.98	1.05	1.01	1.02	0.85	0.85	0.84	0.80	0.81	0.82	0.88	0.86	126.77	5.26
Q8BHH2	Ras-related protein Rab 9B OS=Mus musculus GN=Rab9b PE=2 SV=1 - [RAB9B_MOUSE]	16.42	1	2	2	3	1.09	1.07	0.98	1.10	1.01	1.03	0.93	0.84	0.76	1.05	0.97	1.34	1.23	22.69	4.93
Q8BH57-2	Isoform 2 of WD repeat containing protein 48 OS=Mus musculus GN=Wdr48 - [WDR48_MOUSE]	23.56	3	14	14	34	0.97	1.05	1.06	1.07	1.11	0.88	0.91	0.84	0.88	0.91	0.93	0.93	0.98	74.35	7.17
Q9R1Z8	Vinexin OS=Mus musculus GN=Sorbs3 PE=1 SV=1 - [VINEX_MOUSE]	10.64	1	6	6	12	0.94	0.93	0.96	1.01	1.04	0.77	0.73	0.84	0.84	0.86	0.78	0.83	0.75	82.30	9.20
Q8BFX3	BTB/POZ domain-containing protein KCTD3 OS=Mus musculus GN=Kctd3 PE=2 SV=1 - [KCTD3_MOUSE]	8.47	3	5	5	8	1.06	1.00	0.88	0.95	0.90	0.79	0.74	0.84	0.66	0.82	0.67	0.79	0.74	88.81	7.15
Q78YY6	DnaJ homolog subfamily C member 15 OS=Mus musculus GN=Dnajc15 PE=2 SV=1 - [DJC15_MOUSE]	11.41	1	1	2	4	0.93	0.80	0.86	0.97	1.05	0.99	1.06	0.84	0.90	0.93	1.00	0.91	0.98	15.94	9.99
Q8BKC5-	Isoform 2 of Importin-5 OS=Mus musculus GN=Ipo5 - [IPO5_MOUSE]	13.89	2	9	10	18	1.01	0.99	0.89	1.03	0.99	0.90	0.89	0.84	0.82	0.89	0.86	1.11	1.07	116.84	4.92
Q9QZD8	Mitochondrial dicarboxylate carrier OS=Mus musculus GN=Slc25a10 PE=2 SV=2 - [DIC_MOUSE]	15.33	1	4	4	7	1.17	1.37	1.18	1.28	1.08	0.83	0.74	0.84	0.77	0.94	0.81	1.11	1.00	31.69	9.32
Q3UR70	Transforming growth factor-beta receptor-associated protein 1 OS=Mus musculus GN=Tgfbrap1 PE=2 SV=1 - [TGFA1_MOUSE]	4.19	1	3	3	5	0.90	0.93	1.04	1.14	1.20	0.82	0.92	0.84	0.93	0.88	0.98	1.05	1.14	97.24	6.70
Q5S003	Sperm-associated antigen 17 OS=Mus musculus GN=Spag17 PE=1 SV=1 - [SPG17_MOUSE]	0.55	2	1	2	2	0.88	0.78	0.89	0.65	0.74	0.69	0.78	0.84	0.95	0.76	0.86	0.54	0.61	246.23	6.01

Q8K2B3	Succinate dehydrogenase [ubiquinone] flavoprotein subunit, mitochondrial OS=Mus musculus GN=Sdha PE=1 SV=1 - [DHSA_MOUSE]	56.78	1	25	25	255	0.98	0.96	0.99	0.97	0.97	0.87	0.87	0.84	0.85	0.86	0.86	0.89	0.91	72.54	7.37
P25911-2	Isoform 2 of Tyrosine-protein kinase Lyn OS=Mus musculus GN=Lyn - [LYN_MOUSE]	7.74	50	1	4	11	0.93	1.02	1.10	1.11	1.20	0.82	0.87	0.84	0.90	0.93	1.00	1.07	1.15	56.25	6.57
Q9D0Q7	39S ribosomal protein L45, mitochondrial OS=Mus musculus GN=Mrpl45 PE=2 SV=1 - [RM45_MOUSE]	11.76	1	2	2	3	1.02	0.76	0.75	1.04	1.02	0.87	0.85	0.84	0.82	0.86	0.84	0.89	0.88	35.39	9.23
Q8K004	Protein Spata2 OS=Mus musculus GN=Spata2 PE=2 SV=1 - [Q8K004_MOUSE]	2.33	1	1	1	3	0.81	0.71	0.88	0.85	1.05	0.96	1.18	0.84	1.03	0.85	1.05	0.78	0.96	57.77	8.63
Q9QZX7-	Isoform 2 of Serine racemase OS=Mus musculus GN=Srr - [SRR_MOUSE]	36.31	4	8	8	30	1.08	0.93	0.82	1.01	0.93	0.83	0.71	0.84	0.77	0.80	0.75	0.90	0.78	34.01	6.01
Q11011	Puromycin-sensitive aminopeptidase OS=Mus musculus GN=Npepps PE=1 SV=2 - [PSA_MOUSE]	39.46	7	30	30	87	0.99	0.87	0.91	1.10	1.12	0.88	0.88	0.84	0.87	0.94	0.98	1.00	1.01	103.26	5.90
Q06335	Amyloid-like protein 2 OS=Mus musculus GN=Aplp2 PE=1 SV=4 - [APLP2_MOUSE]	21.78	2	1	14	38	0.92	1.55	1.09	1.15	1.15	1.08	0.96	0.84	0.81	1.10	0.82	0.92	0.78	80.42	4.70
Q6P9K9	Neurexin-3 OS=Mus musculus GN=Nrxn3 PE=1 SV=2 - [NRX3A_MOUSE]	29.73	3	22	35	143	0.99	1.05	1.04	1.02	1.01	0.86	0.83	0.84	0.86	0.87	0.84	0.85	0.87	173.32	6.14
E9Q0K9	ELMO domain-containing protein 1 OS=Mus musculus GN=Elmod1 PE=2 SV=1 - [E9Q0K9_MOUSE]	4.91	2	1	1	2	0.86	0.97	1.14	1.06	1.24	0.84	0.98	0.84	0.98	0.88	1.03	0.88	1.03	37.88	8.28
Q9D0M3-	Isoform 2 of Cytochrome c1, heme protein, mitochondrial OS=Mus musculus GN=Cyc1 - [CY1_MOUSE]	41.35	2	7	7	90	0.93	1.03	1.07	1.02	1.10	0.86	0.96	0.84	0.92	0.81	0.94	0.91	0.97	29.48	7.05
Q9CRY7	Glycerophosphodiester phosphodiesterase domain-containing protein 1 OS=Mus musculus GN=Gdpd1 PE=2 SV=1 - [GDPD1_MOUSE]	12.74	1	3	3	7	1.03	1.01	0.88	1.30	1.18	0.91	0.84	0.84	0.87	0.95	0.88	0.99	1.09	35.84	8.31
P26039	Talin-1 OS=Mus musculus GN=Tln1 PE=1 SV=2 - [TLN1_MOUSE]	11.37	3	13	19	35	0.97	0.90	0.92	1.07	1.10	0.88	0.94	0.84	0.88	0.96	0.98	0.98	1.05	269.65	6.18

Q9D0K1	Peroxisomal membrane protein PEX13 OS=Mus musculus GN=Pex13 PE=1 SV=1 - [PEX13_MOUSE]	9.14	1	1	1	5	0.91	1.06	1.17	0.91	1.00	1.15	1.26	0.84	0.92	1.05	1.16	1.05	1.16	44.58	7.53
Q8VIJ6	Splicing factor, proline- and glutamine-rich OS=Mus musculus GN=Sfpq PE=1 SV=1 - [SFPQ_MOUSE]	43.20	1	27	28	215	0.97	0.85	0.90	0.90	0.92	0.79	0.82	0.84	0.86	0.72	0.77	0.73	0.78	75.39	9.44
Q01065	Calcium/calmodulin-dependent 3',5'-cyclic nucleotide phosphodiesterase 1B OS=Mus musculus GN=Pde1b PE=1 SV=2 - [PDE1B_MOUSE]	29.35	1	12	12	27	1.02	1.06	1.07	1.31	1.36	0.98	1.02	0.84	0.83	1.18	1.17	1.43	1.38	61.19	5.72
D3Z5T2	Armadillo repeat-containing protein 10 OS=Mus musculus GN=Armc10 PE=2 SV=1 - [D3Z5T2_MOUSE]	23.11	3	4	4	6	1.02	1.19	1.01	1.13	1.12	0.88	0.80	0.84	0.82	1.01	0.92	0.91	0.92	25.79	8.85
P58242	Acid sphingomyelinase-like phosphodiesterase 3b OS=Mus musculus GN=Smpdl3b PE=1 SV=1 - [ASM3B_MOUSE]	14.04	1	3	3	7	1.24	1.20	1.01	0.97	0.76	0.90	0.73	0.84	0.62	0.89	0.70	1.17	0.99	51.57	6.43
Q8BR90-2	Isoform 2 of UPF0600 protein C5orf51 homolog OS=Mus musculus - [CE051_MOUSE]	6.19	2	1	1	2	0.88	0.87	0.99	1.15	1.31	1.00	1.14	0.84	0.95	1.10	1.25	1.11	1.26	26.11	5.34
Q9CQE8	UPF0568 protein C14orf166 homolog OS=Mus musculus PE=2 SV=1 - [CN166_MOUSE]	60.25	2	12	12	36	0.88	0.79	0.87	0.85	0.95	0.81	0.92	0.84	0.92	0.75	0.84	0.80	0.87	28.13	6.89
P01668	Ig kappa chain V-III region PC 7210 OS=Mus musculus PE=1 SV=1 - [KV3AG_MOUSE]	16.36	17	1	1	1	0.94	1.09	1.15	1.07	1.14	0.77	0.82	0.84	0.89	1.09	1.15	1.12	1.18	11.94	4.75
Q8BZW8	NHL repeat-containing protein 2 OS=Mus musculus GN=Nhlrc2 PE=2 SV=1 - [NHLC2_MOUSE]	9.24	1	4	4	11	0.82	1.18	1.16	0.89	1.05	0.80	0.91	0.84	1.00	0.79	0.97	0.84	1.02	78.38	5.54
P63085	Mitogen-activated protein kinase 1 OS=Mus musculus GN=Mapk1 PE=1 SV=3 - [MK01_MOUSE]	50.84	17	11	16	77	1.07	0.92	0.84	1.00	0.91	0.95	0.85	0.84	0.79	0.93	0.85	1.01	0.97	41.25	6.98
Q96DY5-2	Isoform 2 of RING finger protein 112 OS=Mus musculus GN=Rnf112 - [RN112_MOUSE]	2.06	3	1	1	1	1.09	1.31	1.20	1.07	0.98	0.91	0.84	0.84	0.77	0.93	0.86	0.80	0.74	68.73	8.51
Q8K4Z0-2	Isoform 2 of Leucine-rich repeat LGI family member 2 OS=Mus musculus GN=Lgi2 - [LGI2_MOUSE]	12.18	2	6	6	10	0.98	1.10	1.09	1.02	1.06	0.94	0.95	0.84	0.85	0.85	0.86	0.92	1.02	61.97	6.54

D3Z217	Thiopurine S-methyltransferase (Fragment) OS=Mus musculus GN=Tpmt PE=2 SV=1 - [D3Z217_MOUSE]	16.48	6	3	3	5	1.15	0.99	0.81	1.05	0.86	1.00	0.94	0.84	0.73	1.01	0.90	1.07	0.93	20.83	6.64
A2AAN0	Exocyst complex component 7 OS=Mus musculus GN=Exoc7 PE=2 SV=1 - [A2AAN0_MOUSE]	11.40	4	7	7	13	0.88	0.99	1.11	1.08	1.12	0.91	0.92	0.84	0.90	0.94	1.01	1.03	1.09	78.46	7.11
Q3UPH1	Protein PRRC1 OS=Mus musculus GN=Prrc1 PE=2 SV=1 - [PRRC1_MOUSE]	3.39	1	1	1	4	0.84	0.84	1.00	0.94	1.12	0.88	1.04	0.84	1.00	0.85	1.01	0.80	0.96	46.27	5.95
P17427	AP-2 complex subunit alpha-2 OS=Mus musculus GN=Ap2a2 PE=1 SV=2 - [AP2A2_MOUSE]	46.38	2	26	37	119	1.05	1.09	1.07	1.15	1.11	0.86	0.83	0.84	0.79	0.95	0.90	1.10	1.01	103.95	6.93
G3UYK8	Coronin OS=Mus musculus GN=Coro1a PE=2 SV=1 - [G3UYK8_MOUSE]	33.16	4	1	14	40	0.74	0.75	0.99	0.96	1.29	0.78	1.05	0.84	1.14	0.80	1.00	0.69	0.93	43.23	6.74
Q8BH69	Selenide, water dikinase 1 OS=Mus musculus GN=Sephs1 PE=2 SV=1 - [SPS1_MOUSE]	18.62	1	5	5	8	1.03	0.90	0.88	1.02	0.89	0.93	0.77	0.84	0.72	0.79	0.62	0.87	0.84	42.88	5.97
Q8CDM8-	Isoform 2 of Protein FAM160B1 OS=Mus musculus GN=Fam160b1 - [F16B1_MOUSE]	2.29	2	1	1	2	0.77	0.89	1.17	0.94	1.23	0.78	1.02	0.84	1.10	0.81	1.06	1.08	1.41	58.50	5.10
Q9CZW4	Long-chain-fatty-acid--CoA ligase 3 OS=Mus musculus GN=Acsl3 PE=2 SV=2 - [ACSL3_MOUSE]	14.86	3	7	7	37	1.00	1.15	1.21	1.06	1.09	0.90	0.89	0.84	0.90	0.93	0.94	1.01	1.08	80.44	8.54
F8VQE9	Arf-GAP with GTPase, ANK repeat and PH domain-containing protein 3 OS=Mus musculus GN=Agap3 PE=2 SV=1 - [F8VQE9_MOUSE]	10.00	3	5	7	13	0.99	0.94	0.95	0.99	1.00	0.83	0.83	0.84	0.82	0.99	0.99	1.01	1.02	97.63	7.77
B2RSI6	Leucine rich repeat containing 8 family, member B OS=Mus musculus GN=Lrrc8b PE=2 SV=1 - [B2RSI6_MOUSE]	3.49	2	2	2	3	1.00	1.07	1.06	1.02	1.01	0.84	0.84	0.84	0.84	0.94	0.94	0.88	0.88	92.13	6.70
Q8K2C9	Very-long-chain (3R)-3-hydroxyacyl-[acyl-carrier protein] dehydratase 3 OS=Mus musculus GN=ptplad1 PE=1 SV=2 - [HACD3_MOUSE]	11.60	1	4	4	29	0.98	1.20	1.12	1.21	1.11	0.91	0.91	0.84	0.89	0.93	0.93	1.08	1.05	43.10	9.13

Q61823	Programmed cell death protein 4 OS=Mus musculus GN=Pdcd4 PE=1 SV=1 - [PDCD4_MOUSE]	16.20	1	5	5	12	1.11	0.98	0.87	0.94	0.83	0.91	0.84	0.84	0.77	0.92	0.80	1.14	0.94	51.67	5.16
D3YTU5	Protein Ankrd29 OS=Mus musculus GN=Ankrd29 PE=2 SV=1 - [D3YTU5_MOUSE]	22.10	2	4	4	6	0.83	1.06	1.21	0.87	1.05	0.91	1.09	0.84	0.98	0.80	0.91	0.85	1.01	29.90	8.18
Q6PE80	Axl protein OS=Mus musculus GN=Axl PE=2 SV=1 - [Q6PE80_MOUSE]	1.25	2	1	1	1	0.88	0.91	1.03	0.91	1.02	0.82	0.92	0.84	0.95	0.79	0.89	0.81	0.92	97.16	5.64
P50544	Very long-chain specific acyl-CoA dehydrogenase, mitochondrial OS=Mus musculus GN=Acadvl PE=1 SV=3 - [ACADV_MOUSE]	20.73	2	11	11	21	0.94	0.97	1.01	1.11	1.19	0.84	0.92	0.84	0.87	0.97	0.93	0.78	0.82	70.83	8.75
Q61672	Equilibrative nucleoside transporter 2 OS=Mus musculus GN=Slc29a2 PE=1 SV=2 - [S29A2_MOUSE]	2.63	1	1	1	2	1.10	1.09	0.99	1.23	1.12	0.82	0.74	0.84	0.76	1.03	0.94	1.06	0.96	50.22	6.51
Q9WV96	Mitochondrial import inner membrane translocase subunit Tim10 B OS=Mus musculus GN=Timm10b PE=2 SV=1 - [T10B_MOUSE]	27.00	4	2	2	5	1.02	1.04	0.97	1.01	0.94	0.80	0.78	0.84	0.82	0.95	0.89	1.06	1.02	11.31	7.08
D3YW48	Calpain small subunit 1 (Fragment) OS=Mus musculus GN=Capns1 PE=2 SV=1 - [D3YW48_MOUSE]	29.46	3	3	3	6	1.04	0.98	0.97	0.93	0.92	0.88	0.85	0.84	0.84	0.95	0.81	0.93	0.85	25.29	5.60
P51863	V-type proton ATPase subunit d 1 OS=Mus musculus GN=Atp6v0d1 PE=1 SV=2 - [VA0D1_MOUSE]	44.16	1	14	14	61	0.99	0.94	0.90	1.08	1.15	0.88	0.87	0.84	0.86	0.89	0.90	0.90	0.97	40.28	5.00
Q8BMS1	Trifunctional enzyme subunit alpha, mitochondrial OS=Mus musculus GN=Hadha PE=1 SV=1 - [ECHA_MOUSE]	33.68	1	21	21	48	1.01	1.00	0.98	1.18	1.16	0.87	0.85	0.84	0.80	0.96	0.93	0.93	0.91	82.62	9.14
Q69ZQ1	Uncharacterized family 31 glucosidase KIAA1161 OS=Mus musculus GN=Kiaa1161 PE=1 SV=2 - [K1161_MOUSE]	1.54	1	1	1	2	0.92	1.05	1.13	0.98	1.06	0.84	0.91	0.84	0.91	1.04	1.13	0.90	0.98	81.31	8.29
Q8C4G3	Probable phospholipid-transporting ATPase IIA OS=Mus musculus GN=Atp9a PE=2 SV=1 - [Q8C4G3_MOUSE]	3.40	4	2	3	5	1.02	1.26	1.23	1.15	1.13	0.93	0.91	0.84	0.82	1.01	0.99	1.09	1.07	116.35	7.27

O35343	Importin subunit alpha-3 OS=Mus musculus GN=Kpna4 PE=2 SV=1 - [IMA3_MOUSE]	19.00	2	4	6	15	1.02	1.22	1.25	0.96	1.06	0.69	0.78	0.84	0.90	1.00	1.02	1.17	1.18	57.89	4.94
Q80UG2	Plexin-A4 OS=Mus musculus GN=Plxna4 PE=1 SV=3 - [PLXA4_MOUSE]	18.38	1	18	26	52	0.97	1.08	1.15	1.07	1.09	0.85	0.87	0.84	0.87	0.90	0.95	1.00	1.02	212.42	6.83
Q9DBS2	Tumor protein p63-regulated gene 1-like protein OS=Mus musculus GN=Tprg1l PE=1 SV=1 - [TPRGL_MOUSE]	43.61	2	6	6	24	1.15	0.99	0.90	1.08	0.98	0.98	0.85	0.84	0.75	0.99	0.85	1.22	1.09	29.80	7.37
Q99J47-2	Isoform 2 of Dehydrogenase/reductase SDR family member 7B OS=Mus musculus GN=Dhrs7b - [DRS7B_MOUSE]	4.78	2	1	1	6	0.95	0.88	0.92	1.09	1.14	0.89	1.01	0.84	0.97	1.08	1.25	1.02	1.18	33.96	9.50
Q61191	Host cell factor 1 OS=Mus musculus GN=Hcfc1 PE=1 SV=2 - [HCFC1_MOUSE]	8.95	3	11	11	20	0.96	0.89	0.94	0.99	1.01	0.94	0.95	0.84	0.92	0.83	0.91	0.83	0.85	210.31	7.18
P68134	Actin, alpha skeletal muscle OS=Mus musculus GN=Acta1 PE=1 SV=1 - [ACTS_MOUSE]	70.03	4	2	26	1018	1.29	1.52	1.18	1.29	1.00	10.75	8.31	0.84	0.65	1.30	1.01	1.21	0.94	42.02	5.39
Q9JIK5	Nucleolar RNA helicase 2 OS=Mus musculus GN=Ddx21 PE=1 SV=3 - [DDX21_MOUSE]	2.70	1	2	2	3	0.87	1.01	1.16	0.99	1.14	0.91	1.03	0.84	0.96	0.97	1.11	0.96	1.11	93.49	9.11
G3UYQ3	Phosphatidylserine synthase 2 OS=Mus musculus GN=Ptdss2 PE=4 SV=1 - [G3UYQ3_MOUSE]	16.25	3	1	1	3	1.24	1.11	0.89	1.07	0.86	0.97	0.77	0.84	0.68	1.07	0.86	0.95	0.76	8.85	6.58
Q80TE0-2	Isoform 2 of RNA polymerase II-associated protein 1 OS=Mus musculus GN=Rpap1 - [RPAP1_MOUSE]	1.32	2	1	1	1	0.98	1.21	1.22	0.68	0.69	1.17	1.18	0.84	0.86	0.97	0.99	0.91	0.92	141.51	6.60
P56564	Excitatory amino acid transporter 1 OS=Mus musculus GN=Slc1a3 PE=1 SV=2 - [EAA1_MOUSE]	30.20	3	9	11	185	1.09	1.02	0.88	1.16	1.05	0.85	0.76	0.84	0.76	0.98	0.89	0.97	0.87	59.58	8.40
O35604	Niemann-Pick C1 protein OS=Mus musculus GN=Npc1 PE=1 SV=2 - [NPC1_MOUSE]	0.94	1	1	1	2	1.13	1.25	1.11	1.10	0.98	0.86	0.76	0.84	0.75	0.92	0.81	1.05	0.93	142.79	5.71
E9QAT6	DNA fragmentation factor subunit beta OS=Mus musculus GN=Cad PE=2 SV=1 - [E9QAT6_MOUSE]	1.34	5	1	1	1	0.94	0.94	0.99	0.99	1.05	0.89	0.94	0.85	0.89	0.83	0.88	1.02	1.09	105.62	6.28
P24549	Retinal dehydrogenase 1 OS=Mus musculus GN=Aldh1a1 PE=1 SV=5 - [AL1A1_MOUSE]	31.94	3	10	11	20	1.02	0.92	0.88	1.28	1.22	0.85	0.95	0.85	0.97	1.05	1.14	1.14	1.12	54.43	7.80

B1AU25	Apoptosis-inducing factor 1, mitochondrial OS=Mus musculus GN=Aifm1 PE=2 SV=1 - [B1AU25_MOUSE]	33.39	2	14	14	32	1.00	1.06	1.11	1.11	1.22	0.84	0.88	0.85	0.91	0.92	0.98	0.96	1.01	66.07	9.11
Q811S7	Upstream-binding protein 1 OS=Mus musculus GN=Ubp1 PE=1 SV=1 - [UBIP1_MOUSE]	7.22	2	3	3	7	1.00	0.92	0.91	0.99	1.08	0.99	0.94	0.85	0.92	0.96	1.00	1.00	1.00	60.17	6.27
Q61563	Fibroblast growth factor receptor OS=Mus musculus GN=Fgfr3 PE=2 SV=2 - [Q61563_MOUSE]	2.43	55	1	2	6	0.93	1.18	1.27	0.95	1.02	0.96	1.03	0.85	0.91	0.99	1.07	0.87	0.94	85.82	6.92
Q99L43	Phosphatidate cytidylyltransferase 2 OS=Mus musculus GN=Cds2 PE=1 SV=1 - [CDS2_MOUSE]	18.47	4	5	5	16	1.01	0.99	0.93	1.14	1.02	0.83	0.84	0.85	0.81	0.91	0.86	0.88	0.85	51.28	7.05
Q7TMQ7	WD repeat-containing protein 91 OS=Mus musculus GN=Wdr91 PE=1 SV=1 - [WDR91_MOUSE]	4.81	1	3	3	5	0.96	0.82	0.77	0.90	0.94	0.86	0.90	0.85	0.88	0.87	0.91	0.88	0.85	83.37	6.64
Q4KUS2	Protein unc-13 homolog A OS=Mus musculus GN=Unc13a PE=1 SV=3 - [UN13A_MOUSE]	10.98	9	14	14	27	0.98	1.17	1.11	1.02	1.09	0.91	0.94	0.85	0.88	0.94	0.94	1.01	1.04	193.66	5.25
Q80XN0	D-beta-hydroxybutyrate dehydrogenase, mitochondrial OS=Mus musculus GN=Bdh1 PE=1 SV=2 - [BDH_MOUSE]	37.90	2	12	12	26	0.89	0.99	1.12	1.06	1.17	0.93	1.04	0.85	0.99	0.98	1.11	0.99	1.14	38.27	9.01
Q8K0P3	TLD domain-containing protein 1 OS=Mus musculus GN=Tldc1 PE=2 SV=1 - [TLDC1_MOUSE]	3.52	1	1	1	1	1.24	1.02	0.82	1.30	1.05	1.02	0.82	0.85	0.68	1.01	0.81	1.18	0.95	50.81	6.52
P10833	Ras-related protein R-Ras OS=Mus musculus GN=Rras PE=2 SV=1 - [RRAS_MOUSE]	23.85	1	2	4	8	1.14	1.25	1.09	0.93	0.81	0.93	0.81	0.85	0.74	0.96	0.84	1.05	0.92	23.75	6.79
Q3UHB1	5'-nucleotidase domain-containing protein 3 OS=Mus musculus GN=Nt5dc3 PE=2 SV=1 - [NT5D3_MOUSE]	27.66	1	12	12	34	1.07	1.03	0.98	1.05	1.01	0.94	0.88	0.85	0.79	0.92	0.85	1.00	0.90	63.13	8.56
Q8JZQ9	Eukaryotic translation initiation factor 3 subunit B OS=Mus musculus GN=Eif3b PE=1 SV=1 - [EIF3B_MOUSE]	40.97	1	22	22	66	1.04	0.97	0.96	0.99	0.96	0.92	0.86	0.85	0.85	0.88	0.83	0.87	0.83	91.31	5.02

P55772	Ectonucleoside triphosphate diphosphohydrolase 1 OS=Mus musculus GN=Entpd1 PE=2 SV=1 - [ENTP1_MOUSE]	5.88	5	2	2	6	0.99	0.80	0.95	1.19	1.23	1.21	0.91	0.85	1.01	1.11	1.33	1.04	1.11	57.17	5.86
O35405	Phospholipase D3 OS=Mus musculus GN=Pld3 PE=2 SV=1 - [PLD3_MOUSE]	20.70	1	7	7	11	0.89	1.05	1.08	1.04	1.03	0.99	0.99	0.85	0.79	0.76	0.85	0.85	0.93	54.35	6.52
Q3U1V6-2	Isoform 2 of Ubiquitin-conjugating enzyme E2 variant 3 OS=Mus musculus GN=Uevld - [UEVLD_MOUSE]	4.40	2	1	1	2	0.86	1.07	1.24	1.00	1.16	0.83	0.96	0.85	0.98	0.99	1.15	1.01	1.18	28.06	6.80
P19096	Fatty acid synthase OS=Mus musculus GN=Fasn PE=1 SV=2 - [FAS_MOUSE]	21.88	1	42	42	126	1.00	1.04	0.93	1.09	1.07	0.90	0.89	0.85	0.86	0.92	0.92	0.99	1.01	272.26	6.58
Q9JM14	5'(3')-deoxyribonucleotidase, cytosolic type OS=Mus musculus GN=Nt5c PE=1 SV=1 - [NT5C_MOUSE]	43.00	1	1	6	21	0.96	1.05	1.08	0.76	0.79	0.85	0.88	0.85	0.87	0.81	0.84	1.07	1.11	23.06	5.49
Q01341	Adenylate cyclase type 6 OS=Mus musculus GN=Adcy6 PE=1 SV=1 - [ADCY6_MOUSE]	3.69	2	2	3	5	0.92	1.07	1.16	1.09	1.18	0.79	0.86	0.85	0.92	0.98	1.07	0.83	0.90	130.23	7.87
P63318	Protein kinase C gamma type OS=Mus musculus GN=Prkcg PE=1 SV=1 - [KPCG_MOUSE]	50.65	2	22	25	90	1.05	1.06	1.10	1.06	1.01	0.90	0.87	0.85	0.87	0.96	0.93	1.15	1.08	78.31	7.46
Q9DBL7	Bifunctional coenzyme A synthase OS=Mus musculus GN=Coasy PE=1 SV=2 - [COASY_MOUSE]	4.09	1	1	1	2	1.00	0.77	0.77	0.87	0.87	0.71	0.71	0.85	0.84	0.99	0.99	0.97	0.98	61.98	7.11
P63011	Ras-related protein Rab 3A OS=Mus musculus GN=Rab3a PE=1 SV=1 - [RAB3A_MOUSE]	62.73	14	5	14	212	0.98	0.86	0.87	1.05	1.06	0.91	0.94	0.85	0.88	0.85	0.87	0.89	0.92	24.95	5.03
Q3KNM2	E3 ubiquitin-protein ligase MARCH5 OS=Mus musculus GN=March5 PE=2 SV=1 - [MARH5_MOUSE]	7.55	2	2	2	7	1.07	1.16	1.05	1.08	0.99	0.74	0.75	0.85	0.77	0.87	0.85	1.01	0.95	31.21	8.70
Q7TMB8	Cytoplasmic FMR1-interacting protein 1 OS=Mus musculus GN=Cyfip1 PE=1 SV=1 - [CYFP1_MOUSE]	13.89	4	5	14	31	0.94	1.15	1.09	1.18	1.26	0.85	0.89	0.85	0.81	0.95	1.01	1.03	1.09	145.15	6.90
Q91VC3	Eukaryotic initiation factor 4A-III OS=Mus musculus GN=Eif4a3 PE=2 SV=3 - [IF4A3_MOUSE]	27.74	3	8	11	42	0.92	0.85	0.92	0.95	1.07	0.82	0.91	0.85	0.91	0.85	0.99	0.82	0.91	46.81	6.73

Q6TEK5	Vitamin K epoxide reductase complex subunit 1-like protein 1 OS=Mus musculus GN=Vkorc1l1 PE=2 SV=1 - [VKORL_MOUSE]	10.80	2	2	2	3	1.33	1.10	0.82	1.22	0.92	0.92	0.69	0.85	0.64	1.02	0.77	1.12	0.84	19.77	8.95
O89114	DnaJ homolog subfamily B member 5 OS=Mus musculus GN=Dnajb5 PE=2 SV=1 - [DNJB5_MOUSE]	12.93	2	2	2	5	1.07	1.25	1.04	0.96	0.86	1.78	1.53	0.85	0.79	1.01	0.93	0.86	0.76	39.10	9.04
Q3TYH2	Ras-related protein Rab 15 OS=Mus musculus GN=Rab15 PE=2 SV=1 - [Q3TYH2_MOUSE]	25.94	15	3	5	53	1.12	2.15	1.84	1.21	1.11	1.02	0.87	0.85	0.70	0.84	0.75	1.12	0.93	24.32	5.71
A2AMW0	Capping protein (Actin filament) muscle Z-line, beta, isoform CRA_a OS=Mus musculus GN=Capzb PE=4 SV=1 - [A2AMW0_MOUSE]	61.92	6	3	17	127	0.97	0.88	0.88	0.99	1.02	1.03	1.00	0.85	0.87	0.93	0.94	0.96	0.99	29.28	6.92
Q8R2Y0-	Isoform 2 of Monoacylglycerol lipase ABHD6 OS=Mus musculus GN=Abhd6 - [ABHD6_MOUSE]	14.88	2	3	3	7	0.93	0.88	0.93	1.10	1.27	0.84	0.87	0.85	0.90	0.89	0.98	0.89	1.00	32.77	8.34
Q9R0X4	Acyl-coenzyme A thioesterase 9, mitochondrial OS=Mus musculus GN=Acot9 PE=1 SV=1 - [ACOT9_MOUSE]	20.05	2	7	7	15	0.96	1.14	1.07	1.01	1.04	0.92	0.91	0.85	0.87	0.91	0.97	1.02	1.00	50.53	8.59
Q9Z0J0	Epididymal secretory protein E1 OS=Mus musculus GN=Npc2 PE=2 SV=1 - [NPC2_MOUSE]	28.19	1	4	4	10	0.93	0.65	0.77	0.90	1.02	0.83	0.92	0.85	1.03	0.77	0.88	0.70	0.78	16.43	7.68
Q9DBG3	AP-2 complex subunit beta OS=Mus musculus GN=Ap2b1 PE=1 SV=1 - [AP2B1_MOUSE]	51.87	6	22	44	197	0.95	0.95	1.02	1.10	1.10	0.92	0.94	0.85	0.90	0.91	0.96	0.96	0.99	104.52	5.38
Q68FD5	Clathrin heavy chain 1 OS=Mus musculus GN=Cltc PE=1 SV=3 - [CLH1_MOUSE]	67.76	3	95	95	830	0.96	1.08	1.12	1.16	1.21	0.85	0.87	0.85	0.88	0.92	0.98	1.00	1.02	191.43	5.69
O54829	Regulator of G-protein signaling 7 OS=Mus musculus GN=Rgs7 PE=1 SV=2 - [RGS7_MOUSE]	35.18	1	10	13	38	0.85	0.87	1.06	1.02	1.13	0.91	1.06	0.85	1.00	0.85	0.99	0.85	0.97	54.76	8.13
Q9QYB5	Gamma-adducin OS=Mus musculus GN=Add3 PE=1 SV=2 - [ADDG_MOUSE]	34.70	1	1	19	172	1.13	0.93	0.83	0.95	0.82	0.89	0.81	0.85	0.77	0.78	0.71	0.84	0.81	78.73	5.95
Q9D5V5	Cullin-5 OS=Mus musculus GN=Cul5 PE=1 SV=3 - [CUL5_MOUSE]	19.49	4	14	14	28	1.02	0.99	0.93	1.11	1.07	0.97	0.87	0.85	0.86	0.91	0.91	1.08	1.05	90.92	7.81

Q3UNZ8	Quinone oxidoreductase-like protein 2 OS=Mus musculus PE=2 SV=1 - [QORL2_MOUSE]	4.00	1	1	1	4	1.25	1.06	0.84	1.12	0.90	0.96	0.76	0.85	0.67	0.97	0.78	1.07	0.85	37.78	8.90
Q9EQQ9	Bifunctional protein NCOAT OS=Mus musculus GN=Mgea5 PE=1 SV=2 - [NCOAT_MOUSE]	12.99	3	9	9	15	0.98	1.09	1.03	1.06	1.11	0.94	0.96	0.85	0.88	0.94	0.95	0.83	0.93	103.10	4.92
G3UVV4	Hexokinase 1, isoform CRA_f OS=Mus musculus GN=Hk1 PE=3 SV=1 - [G3UVV4_MOUSE]	47.98	15	42	42	338	0.95	0.96	1.02	1.06	1.12	0.89	0.95	0.85	0.89	0.92	0.98	0.97	1.04	101.81	6.67
P47743	Metabotropic glutamate receptor 8 OS=Mus musculus GN=Grm8 PE=1 SV=2 - [GRM8_MOUSE]	0.99	2	1	1	2	0.83	1.25	1.51	1.03	1.25	0.82	0.99	0.85	1.02	0.93	1.13	0.94	1.14	101.76	8.15
P70404	Isocitrate dehydrogenase [NAD] subunit gamma 1, mitochondrial OS=Mus musculus GN=Idh3g PE=1 SV=1 - [IDHG1_MOUSE]	34.61	1	11	11	42	0.97	0.97	0.96	1.16	1.13	0.85	0.94	0.85	0.89	0.91	1.01	1.05	1.06	42.76	9.01
Q7TPV4	Myb-binding protein 1A OS=Mus musculus GN=Mybbp1a PE=1 SV=2 - [MBB1A_MOUSE]	3.20	1	3	3	5	0.87	0.81	0.97	1.04	1.14	0.83	0.93	0.85	0.83	0.83	0.91	0.96	1.01	151.94	8.95
Q921E6-3	Isoform 3 of Polycomb protein EED OS=Mus musculus GN=Eed - [EED_MOUSE]	2.34	3	1	1	1	1.13	1.31	1.15	1.06	0.93	0.92	0.81	0.85	0.75	1.07	0.94	0.98	0.86	48.73	7.31
Q9D1E8	1-acyl-sn-glycerol-3-phosphate acyltransferase epsilon OS=Mus musculus GN=Agpat5 PE=2 SV=2 - [PLCE_MOUSE]	6.85	2	2	2	4	1.00	1.30	1.30	1.16	1.16	0.74	0.74	0.85	0.85	0.94	0.94	1.01	1.01	42.18	9.38
Q9DD03	Ras-related protein Rab 13 OS=Mus musculus GN=Rab13 PE=1 SV=1 - [RAB13_MOUSE]	14.85	1	1	3	14	1.15	1.22	1.06	1.16	1.00	0.93	0.80	0.85	0.73	0.94	0.82	1.14	0.99	22.76	9.50
Q9D273	Cob(I)yrinic acid a,c-diamide adenosyltransferase, mitochondrial OS=Mus musculus GN=Mmab PE=2 SV=1 - [MMAB_MOUSE]	31.65	3	6	6	19	0.92	0.85	0.87	0.91	0.96	0.87	0.93	0.85	0.90	0.96	0.94	0.87	0.91	26.26	9.20
A2AKD7	Alpha-1-syntrophin OS=Mus musculus GN=Snta1 PE=2 SV=1 - [A2AKD7_MOUSE]	23.05	2	7	7	11	0.99	0.96	1.04	0.91	0.91	1.00	0.94	0.85	0.88	0.94	0.96	0.96	1.11	53.25	6.80

Q9DB34	Charged multivesicular body protein 2a OS=Mus musculus GN=Chmp2a PE=1 SV=1 - [CHM2A_MOUSE]	22.52	1	5	5	14	0.99	0.90	1.01	0.90	0.92	0.82	0.81	0.85	0.83	0.80	0.79	0.85	0.84	25.12	5.97
F6RL36	Troponin T, fast skeletal muscle (Fragment) OS=Mus musculus GN=Tnnt3 PE=2 SV=1 - [F6RL36_MOUSE]	35.05	33	5	5	16	0.72	0.84	0.94	0.68	0.85	16.91	20.90	0.85	1.20	1.02	1.58	0.82	1.23	25.66	8.12
G3UWE1	MCG11048, isoform CRA_c OS=Mus musculus GN=Tecr PE=4 SV=1 - [G3UWE1_MOUSE]	16.38	3	5	5	16	1.11	0.97	0.84	1.11	1.00	0.82	0.73	0.85	0.76	0.90	0.82	1.01	1.00	34.22	9.47
Q8K3A9	7SK snRNA methylphosphate capping enzyme OS=Mus musculus GN=Mepce PE=1 SV=2 - [MEPCE_MOUSE]	12.01	1	3	3	4	0.90	1.33	1.47	0.98	1.09	0.92	1.02	0.85	0.94	0.92	1.02	1.05	1.16	72.01	9.25
O54833	Casein kinase II subunit alpha' OS=Mus musculus GN=Csnk2a2 PE=2 SV=1 - [CSK22_MOUSE]	36.86	1	10	10	21	1.04	0.92	1.03	0.99	1.04	0.89	0.97	0.85	1.06	0.86	0.93	0.88	0.87	41.19	8.56
O09114-2	Isoform 2 of Prostaglandin-H2 D-isomerase OS=Mus musculus GN=Ptgds - [PTGDS_MOUSE]	20.63	2	2	2	10	1.23	0.74	0.60	1.01	0.90	0.74	0.67	0.85	0.72	0.71	0.66	0.85	0.72	13.93	6.77
Q6PAR5-	Isoform 5 of GTPase-activating protein and VPS9 domain-containing protein 1 OS=Mus musculus GN=Gapvd1 - [GAPD1_MOUSE]	4.53	12	5	5	12	0.89	1.11	1.26	1.03	1.17	0.89	0.95	0.85	0.98	0.93	1.07	1.00	1.16	159.88	5.20
E9PZJ3	Protein 4933411K20Rik (Fragment) OS=Mus musculus GN=4933411K20Rik PE=2 SV=1 - [E9PZJ3_MOUSE]	6.34	4	1	1	2	1.14	0.74	0.64	0.91	0.80	1.17	1.01	0.85	0.74	0.83	0.73	0.96	0.84	22.91	6.27
E9Q357	Protein Gm17673 OS=Mus musculus GN=Gm17673 PE=4 SV=2 - [E9Q357_MOUSE]	2.20	1	2	2	3	1.81	0.95	0.52	0.99	0.55	1.04	0.57	0.85	0.47	1.12	0.62	1.13	0.63	109.61	9.16
Q9QY93	dCTP pyrophosphatase 1 OS=Mus musculus GN=Dctpp1 PE=1 SV=1 - [DCTP1_MOUSE]	11.18	1	1	1	2	1.12	1.67	1.49	1.23	1.11	0.92	0.82	0.85	0.76	0.68	0.61	0.73	0.66	18.78	5.03
Q7TNC9	Inpp5a protein OS=Mus musculus GN=Inpp5a PE=2 SV=1 - [Q7TNC9_MOUSE]	9.71	3	4	4	7	1.19	0.97	0.84	1.00	0.91	0.85	0.82	0.85	0.78	0.91	0.82	0.91	0.83	47.59	6.92

Q9CR57	60S ribosomal protein L14 OS=Mus musculus GN=Rpl14 PE=2 SV=3 - [RL14_MOUSE]	11.52	1	2	2	5	0.98	1.21	1.00	1.01	1.03	0.85	0.81	0.85	0.91	0.93	0.97	1.14	1.16	23.55	11.02
Q8BGR2	Leucine-rich repeat-containing protein 8D OS=Mus musculus GN=Lrrc8d PE=2 SV=1 - [LRC8D_MOUSE]	1.86	1	1	1	1	1.02	1.27	1.25	1.06	1.04	0.72	0.70	0.85	0.83	0.85	0.83	0.92	0.90	98.05	7.44
E9PVZ0	GTP-binding protein REM 2 OS=Mus musculus GN=Rem2 PE=2 SV=1 - [E9PVZ0_MOUSE]	10.00	3	1	1	2	0.93	0.78	0.84	1.36	1.47	1.16	1.24	0.85	0.92	1.46	1.58	1.64	1.78	18.95	6.10
Q9JLV5	Cullin-3 OS=Mus musculus GN=Cul3 PE=1 SV=1 - [CUL3_MOUSE]	27.08	10	17	18	33	0.99	0.89	0.88	1.09	1.10	0.87	0.88	0.85	0.81	0.90	0.88	0.95	0.95	88.89	8.46
E9Q1L5	Sentrin-specific protease 7 OS=Mus musculus GN=Senp7 PE=2 SV=1 - [E9Q1L5_MOUSE]	0.79	2	1	1	1	0.89	0.94	1.05	0.89	0.99	0.89	0.99	0.85	0.95	0.85	0.95	0.80	0.90	113.29	6.47
Q9WVK8	Cholesterol 24-hydroxylase OS=Mus musculus GN=Cyp46a1 PE=2 SV=1 - [CP46A_MOUSE]	22.60	1	10	10	23	0.96	1.02	1.02	1.09	1.06	0.87	0.84	0.85	0.87	0.92	0.97	0.89	0.91	56.78	8.82
Q80X68	Citrate synthase OS=Mus musculus GN=Csl PE=1 SV=1 - [Q80X68_MOUSE]	25.54	1	1	10	76	1.21	1.10	0.91	1.01	0.83	0.91	0.75	0.85	0.70	0.94	0.78	1.28	1.06	52.29	8.79
Q923L3-2	Isoform 2 of CUB and sushi domain-containing protein 1 OS=Mus musculus GN=Csmd1 - [CSMD1_MOUSE]	0.68	2	1	2	4	0.95	0.97	1.02	1.01	1.06	0.81	0.85	0.85	0.90	0.86	0.91	1.14	1.21	386.00	6.09
Q5SW88	Ras-related protein Rab 1A OS=Mus musculus GN=Rab1 PE=2 SV=1 - [Q5SW88_MOUSE]	72.28	13	7	13	183	1.03	0.97	1.00	1.00	0.96	0.90	0.90	0.85	0.88	0.85	0.95	0.94	0.99	22.36	6.21
Q9WUP7-	Isoform 2 of Ubiquitin carboxyl-terminal hydrolase isozyme L5 OS=Mus musculus GN=Uchl5 - [UCHL5_MOUSE]	40.85	2	8	8	16	0.97	0.99	0.97	0.97	1.00	0.94	0.90	0.85	0.89	0.96	0.96	0.88	0.92	37.46	5.40
O35295	Transcriptional activator protein Pur-beta OS=Mus musculus GN=Purb PE=1 SV=3 - [PURB_MOUSE]	49.38	1	9	13	49	0.96	1.02	1.05	0.89	0.97	0.88	0.89	0.85	0.90	0.78	0.81	0.76	0.85	33.88	5.43
P62823	Ras-related protein Rab 3C OS=Mus musculus GN=Rab3c PE=1 SV=1 - [RAB3C_MOUSE]	66.08	11	7	15	107	1.09	1.00	0.89	1.10	0.98	0.95	0.84	0.85	0.74	1.00	0.88	1.06	0.95	25.86	5.24

Q9QZN4	F-box only protein 6 OS=Mus musculus GN=Fbxo6 PE=1 SV=1 - [FBX6_MOUSE]	3.73	1	1	1	2	0.93	1.38	1.49	1.14	1.23	1.00	1.07	0.85	0.91	1.06	1.14	0.93	1.00	34.47	8.18
Q8VHE0	Translocation protein SEC63 homolog OS=Mus musculus GN=Sec63 PE=1 SV=4 - [SEC63_MOUSE]	7.24	1	4	4	5	1.00	1.13	1.13	1.02	1.02	0.97	0.98	0.85	0.85	0.94	0.92	0.96	0.97	87.81	5.38
Q6NVE8	WD repeat-containing protein 44 OS=Mus musculus GN=Wdr44 PE=1 SV=1 - [WDR44_MOUSE]	38.58	2	25	25	66	0.91	0.86	0.91	0.88	0.96	0.83	0.88	0.85	0.92	0.78	0.81	0.76	0.82	101.49	5.24
B1AWJ4	Transmembrane protein 8B OS=Mus musculus GN=Tmem8b PE=2 SV=1 - [B1AWJ4_MOUSE]	1.12	1	1	1	2	1.13	1.00	0.89	1.07	0.95	0.75	0.67	0.85	0.75	0.89	0.79	0.91	0.81	98.03	8.91
Q80XK6	Autophagy-related protein 2 homolog B OS=Mus musculus GN=Atg2b PE=1 SV=3 - [ATG2B_MOUSE]	2.65	3	3	3	5	0.96	1.08	1.11	0.88	0.91	0.96	0.99	0.85	0.88	0.97	1.01	1.03	1.07	231.25	5.88
Q9WUA2	Phenylalanine--tRNA ligase beta subunit OS=Mus musculus GN=Farsb PE=2 SV=2 - [SYFB_MOUSE]	21.22	1	11	11	20	0.99	0.84	0.82	1.05	1.02	0.95	0.97	0.85	0.84	0.87	0.86	1.02	1.02	65.66	7.12
Q60764	Probable E3 ubiquitin-protein ligase makorin-3 OS=Mus musculus GN=Mkrn3 PE=2 SV=2 - [MKRN3_MOUSE]	1.29	1	1	1	2	0.79	1.22	1.53	1.18	1.48	0.90	1.13	0.85	1.07	1.01	1.27	0.78	0.99	59.40	6.64
Q9WUB3	Glycogen phosphorylase, muscle form OS=Mus musculus GN=Pygm PE=1 SV=3 - [PYGM_MOUSE]	46.91	2	23	35	102	1.03	1.00	1.02	1.08	1.03	0.97	0.93	0.85	0.85	0.92	0.89	0.94	0.93	97.22	7.11
P97819-2	Isoform Short of 85/88 kDa calcium-independent phospholipase A2 OS=Mus musculus GN=Pla2g6 - [PLPL9_MOUSE]	3.59	5	2	2	4	1.01	0.96	0.96	1.11	1.11	0.97	0.95	0.85	0.87	0.95	0.98	0.93	1.01	83.65	7.18
Q3URE1	Acyl-CoA synthetase family member 3, mitochondrial OS=Mus musculus GN=Acsf3 PE=2 SV=2 - [ACSF3_MOUSE]	13.38	2	5	5	8	0.84	0.86	1.04	1.13	1.33	0.86	0.96	0.85	1.03	0.96	1.08	0.90	1.09	65.04	8.02
P62754	40S ribosomal protein S6 OS=Mus musculus GN=Rps6 PE=1 SV=1 - [RS6_MOUSE]	18.88	1	4	4	17	0.98	1.07	1.08	1.03	1.03	0.93	0.95	0.85	0.85	0.93	0.97	1.04	1.07	28.66	10.84

Q8VEE4	Replication protein A 70 kDa DNA-binding subunit OS=Mus musculus GN=Rpa1 PE=2 SV=1 - [RFA1_MOUSE]	1.93	2	1	1	1	0.90	1.00	1.11	1.00	1.11	0.93	1.03	0.85	0.94	0.93	1.04	1.05	1.17	68.99	7.91
E9QAZ2	Ribosomal protein L15 OS=Mus musculus GN=Gm10020 PE=3 SV=1 - [E9QAZ2_MOUSE]	23.53	4	4	4	11	0.99	0.93	0.91	1.00	1.05	0.88	0.98	0.85	0.96	0.94	1.02	0.97	0.95	24.06	11.58
Q9DBF1-	Isoform 2 of Alpha-aminoadipic semialdehyde dehydrogenase OS=Mus musculus GN=Aldh7a1 - [AL7A1_MOUSE]	54.99	6	18	18	53	1.07	1.00	0.97	1.16	1.06	0.97	0.88	0.85	0.82	0.98	0.88	1.00	0.94	55.61	6.40
Q9CZD3	Glycine--tRNA ligase OS=Mus musculus GN=Gars PE=1 SV=1 - [SYG_MOUSE]	32.10	1	18	18	54	0.97	0.91	0.95	1.09	1.14	0.91	0.96	0.85	0.90	0.93	0.96	0.90	0.93	81.83	6.65
Q9QZB7	Actin-related protein 10 OS=Mus musculus GN=Actr10 PE=1 SV=2 - [ARP10_MOUSE]	30.46	1	7	7	18	1.03	0.88	0.86	1.06	1.06	0.94	0.92	0.85	0.90	0.87	0.93	1.02	0.98	46.18	7.61
Q9QX11-	Isoform 2 of Cytohesin-1 OS=Mus musculus GN=Cyth1 - [CYH1_MOUSE]	20.65	4	3	7	18	0.92	0.92	1.08	0.88	1.10	0.69	0.98	0.85	0.90	0.90	1.00	0.68	0.96	46.19	5.63
Q9WVT6	Carbonic anhydrase 14 OS=Mus musculus GN=Ca14 PE=1 SV=1 - [CAH14_MOUSE]	16.91	2	4	4	8	1.10	1.01	0.96	0.95	0.86	0.88	0.76	0.85	0.77	0.75	0.74	0.94	0.84	37.48	6.35
P60843	Eukaryotic initiation factor 4A-I OS=Mus musculus GN=Eif4a1 PE=2 SV=1 - [IF4A1_MOUSE]	38.92	1	5	14	63	1.11	0.92	0.83	0.99	0.91	0.92	0.92	0.85	0.92	0.78	0.77	1.09	0.93	46.12	5.48
Q8CAK1	Putative transferase CAF17 homolog, mitochondrial OS=Mus musculus GN=Iba57 PE=2 SV=1 - [CAF17_MOUSE]	10.06	2	3	3	6	1.01	1.03	1.18	0.94	0.89	0.85	0.82	0.85	0.80	0.89	0.95	0.89	0.93	38.38	9.01
O55126	Protein NipSnap homolog 2 OS=Mus musculus GN=Gbas PE=2 SV=1 - [NIPS2_MOUSE]	42.35	2	9	9	51	0.97	1.01	1.00	1.00	1.05	0.83	0.87	0.85	0.87	0.88	0.90	0.99	1.02	32.91	9.26
F6YAR3	Transducin beta-like protein 3 (Fragment) OS=Mus musculus GN=Tbl3 PE=4 SV=1 - [F6YAR3_MOUSE]	4.35	2	1	1	5	1.02	1.31	1.28	1.06	1.03	0.98	0.95	0.85	0.83	0.94	0.92	1.03	1.01	33.45	6.74
Q9DCD0	6-phosphogluconate dehydrogenase, decarboxylating OS=Mus musculus GN=Pgd PE=2 SV=3 - [6PGD_MOUSE]	25.88	1	9	9	23	1.11	0.98	0.88	1.22	1.10	0.98	0.86	0.85	0.78	1.03	0.93	1.07	0.99	53.21	7.23

P11881-8	Isoform 8 of Inositol 1,4,5-trisphosphate receptor type 1 OS=Mus musculus GN=Itpr1 - [ITPR1_MOUSE]	17.04	14	34	34	68	0.91	1.03	1.16	1.20	1.32	0.87	0.94	0.85	0.97	0.94	1.05	0.98	1.11	306.67	6.29
D3YU17	Nicalin OS=Mus musculus GN=Ncln PE=2 SV=1 - [D3YU17_MOUSE]	16.01	3	9	9	17	0.98	1.20	1.25	1.12	1.14	0.89	0.83	0.85	0.90	0.95	0.93	0.94	0.92	62.74	6.49
Q8BG89-2	Isoform 2 of Protein ZNF365 OS=Mus musculus GN=Znf365 - [ZN365_MOUSE]	5.42	2	2	2	3	1.13	1.21	1.07	1.11	0.99	0.88	0.77	0.85	0.75	0.85	0.75	0.92	0.82	38.09	6.90
Q8BU31	Ras-related protein Rap 2c OS=Mus musculus GN=Rap2c PE=1 SV=1 - [RAP2C_MOUSE]	53.55	2	1	7	33	1.19	1.02	0.85	1.15	0.96	0.94	0.79	0.85	0.71	1.01	0.85	1.11	0.93	20.73	4.94
Q4VBD2	Transmembrane anterior posterior transformation protein 1 OS=Mus musculus GN=Tapt1 PE=2 SV=2 - [TAPT1_MOUSE]	4.61	1	2	2	4	1.02	0.92	0.91	1.11	1.09	0.90	0.88	0.85	0.84	0.87	0.86	0.95	0.94	63.85	8.22
P56389	Cytidine deaminase OS=Mus musculus GN=Cda PE=1 SV=2 - [CDD_MOUSE]	20.55	1	2	2	3	1.39	0.92	0.66	1.00	0.72	1.01	0.73	0.85	0.61	0.96	0.69	1.14	0.83	16.12	5.58
P0C192	Leucine-rich repeat-containing protein 4B OS=Mus musculus GN=Lrrc4b PE=1 SV=1 - [LRC4B_MOUSE]	11.00	3	6	6	11	1.02	1.01	0.99	0.97	0.96	0.90	0.89	0.85	0.83	0.91	0.87	1.01	0.96	76.11	7.24
P52189	Inward rectifier potassium channel 4 OS=Mus musculus GN=Kcnj4 PE=1 SV=1 - [IRK4_MOUSE]	2.02	2	1	1	1	1.10	0.98	0.89	1.20	1.10	0.96	0.87	0.85	0.78	0.92	0.84	0.93	0.85	49.87	6.21
Q8VE38-2	Isoform 2 of Oxidoreductase NAD-binding domain-containing protein 1 OS=Mus musculus GN=Oxnad1 - [OXND1_MOUSE]	9.20	2	2	2	3	1.00	1.02	1.02	1.04	1.05	0.81	0.80	0.85	0.85	0.87	0.87	0.94	0.94	28.15	6.79
G3X9V4	Glutamate receptor ionotropic, NMDA 2B OS=Mus musculus GN=Grin2b PE=4 SV=1 - [G3X9V4_MOUSE]	12.75	2	16	16	44	1.02	1.24	1.11	1.12	1.15	0.86	0.82	0.85	0.90	0.85	0.80	0.93	0.92	165.89	6.87
P62874	Guanine nucleotide-binding protein G(I)/G(S)/G(T) subunit beta-1 OS=Mus musculus GN=Gnb1 PE=1 SV=3 - [GBB1_MOUSE]	52.35	4	9	16	189	1.01	0.96	0.97	1.04	1.08	0.88	0.88	0.85	0.89	0.89	0.91	0.93	0.98	37.35	6.00

Q60972	Histone-binding protein RBBP4 OS=Mus musculus GN=Rbbp4 PE=1 SV=5 - [RBBP4_MOUSE]	18.82	1	3	6	19	0.99	0.82	1.00	0.85	0.95	0.75	0.89	0.86	0.91	0.72	0.82	0.71	0.77	47.63	4.89
Q3UVK0	Endoplasmic reticulum metallopeptidase 1 OS=Mus musculus GN=Ermp1 PE=1 SV=2 - [ERMP1_MOUSE]	2.34	4	2	2	3	1.02	1.02	1.00	1.07	1.04	0.89	0.87	0.86	0.83	0.93	0.91	0.91	0.90	100.08	7.49
Q8K339	DNA/RNA-binding protein KIN17 OS=Mus musculus GN=Kin PE=2 SV=1 - [KIN17_MOUSE]	6.39	1	2	2	2	0.98	1.16	1.18	0.74	0.80	0.66	0.91	0.86	0.87	1.09	1.07	0.98	1.00	44.69	9.04
Q9CQ06	39S ribosomal protein L24, mitochondrial OS=Mus musculus GN=Mrpl24 PE=2 SV=1 - [RM24_MOUSE]	11.11	1	2	2	4	0.82	1.08	1.32	0.98	1.20	0.86	1.05	0.86	1.04	0.87	1.06	0.86	1.05	24.93	9.50
Q03059	Choline O-acetyltransferase OS=Mus musculus GN=Chat PE=2 SV=2 - [CLAT_MOUSE]	4.52	2	2	2	4	1.03	0.97	0.94	1.14	1.10	0.95	0.92	0.86	0.82	1.02	0.99	1.09	1.05	71.81	7.74
Q8HW98	IgLON family member 5 OS=Mus musculus GN=Iglon5 PE=2 SV=2 - [IGLO5_MOUSE]	13.69	1	3	3	7	0.91	1.02	0.99	1.02	1.09	0.94	0.91	0.86	0.84	0.90	0.95	0.88	0.91	36.74	7.69
E0CXQ1	High affinity cAMP-specific and IBMX-insensitive 3',5'-cyclic phosphodiesterase 8B (Fragment) OS=Mus musculus GN=Pde8b PE=2 SV=2 - [E0CXQ1_MOUSE]	9.42	9	2	2	4	1.02	1.31	1.28	1.17	1.15	0.69	0.67	0.86	0.84	1.07	1.05	1.04	1.02	37.17	6.43
Q5SYD0	Unconventional myosin-Id OS=Mus musculus GN=Myo1d PE=1 SV=1 - [MYO1D_MOUSE]	11.73	3	10	10	21	0.99	0.96	1.01	1.15	1.12	0.90	0.88	0.86	0.93	0.97	1.01	1.05	0.98	116.01	9.41
Q9ESJ4	NCK-interacting protein with SH3 domain OS=Mus musculus GN=Nckipsd PE=2 SV=2 - [SPN90_MOUSE]	15.97	1	10	10	20	0.96	0.99	1.06	1.00	1.06	0.88	0.92	0.86	0.93	0.91	0.91	0.93	0.97	78.52	6.05
Q8BZN6	Dedicator of cytokinesis protein 10 OS=Mus musculus GN=Dock10 PE=1 SV=3 - [DOC10_MOUSE]	1.21	4	2	2	6	1.51	1.26	1.02	1.18	0.86	0.90	0.64	0.86	0.58	1.04	0.81	1.64	1.10	245.60	7.05
Q8VCT3	Aminopeptidase B OS=Mus musculus GN=Rnpep PE=2 SV=2 - [AMPB_MOUSE]	26.77	2	12	12	30	1.08	1.06	0.94	1.08	1.08	0.96	0.85	0.86	0.76	0.97	0.88	1.06	0.97	72.37	5.35
Q9EQK5	Major vault protein OS=Mus musculus GN=Mvp PE=1 SV=4 - [MVP_MOUSE]	3.48	2	3	3	4	0.87	1.18	1.30	1.12	1.24	0.91	1.02	0.86	0.98	0.89	1.03	1.00	1.15	95.87	5.59

B1AS29	Glutamate receptor ionotropic, kainate 3 OS=Mus musculus GN=Grik3 PE=2 SV=1 - [GRIK3_MOUSE]	7.83	5	5	6	9	0.98	0.93	0.88	1.00	1.05	0.89	0.87	0.86	0.87	0.89	0.83	0.98	0.94	103.98	7.52
E9Q2C7	Borealin (Fragment) OS=Mus musculus GN=Cdca8 PE=2 SV=1 - [E9Q2C7_MOUSE]	10.48	4	1	1	2	1.48	1.07	0.72	1.14	0.77	1.09	0.74	0.86	0.58	0.91	0.61	1.10	0.74	12.10	9.70
Q9JHI5	Isovaleryl-CoA dehydrogenase, mitochondrial OS=Mus musculus GN=Ivd PE=1 SV=1 - [IVD_MOUSE]	24.29	1	10	10	30	1.05	1.02	0.93	1.09	1.06	0.90	0.87	0.86	0.82	0.94	0.99	1.03	0.99	46.30	8.29
Q64519	Syndecan-3 OS=Mus musculus GN=Sdc3 PE=2 SV=2 - [SDC3_MOUSE]	3.85	1	1	1	1	1.47	1.19	0.81	0.94	0.64	0.96	0.65	0.86	0.58	1.01	0.69	1.17	0.80	45.97	4.59
Q9DC63-3	Isoform 3 of F-box only protein 3 OS=Mus musculus GN=Fbxo3 - [FBX3_MOUSE]	10.00	4	3	3	7	0.96	1.09	1.08	0.89	0.98	1.09	0.93	0.86	0.92	1.01	1.04	1.11	1.10	46.87	6.74
P62880	Guanine nucleotide-binding protein G(I)/G(S)/G(T) subunit beta-2 OS=Mus musculus GN=Gnb2 PE=1 SV=3 - [GBB2_MOUSE]	59.71	6	6	15	130	1.04	1.02	0.93	1.03	1.02	0.92	0.87	0.86	0.86	0.93	0.87	1.01	0.98	37.31	6.00
D3Z315	Coatomer subunit epsilon (Fragment) OS=Mus musculus GN=Cope PE=2 SV=1 - [D3Z315_MOUSE]	20.26	4	3	3	6	1.11	1.08	1.10	1.02	1.04	0.90	0.81	0.86	0.82	0.83	0.85	0.91	0.85	25.35	4.70
P70372	ELAV-like protein 1 OS=Mus musculus GN=Elavl1 PE=1 SV=2 - [ELAV1_MOUSE]	21.47	1	6	6	20	0.97	1.05	1.13	0.98	1.03	0.86	0.88	0.86	0.88	0.85	0.90	0.97	1.04	36.15	9.04
F6U329	Sodium channel protein type 8 subunit alpha OS=Mus musculus GN=Scn8a PE=3 SV=2 - [F6U329_MOUSE]	7.28	14	8	11	22	0.93	1.00	1.04	1.16	1.26	0.84	0.90	0.86	0.93	0.91	0.98	0.87	0.90	225.02	6.30
O35136	Neural cell adhesion molecule 2 OS=Mus musculus GN=Ncam2 PE=1 SV=1 - [NCAM2_MOUSE]	35.01	5	24	24	74	1.05	0.96	0.99	1.08	1.02	0.96	0.93	0.86	0.85	0.97	0.90	1.00	0.95	93.15	6.07
Q8C6I5	Cysteine desulfurase, mitochondrial OS=Mus musculus GN=Nfs1 PE=2 SV=1 - [Q8C6I5_MOUSE]	26.14	6	10	10	15	1.03	1.05	0.94	1.02	0.92	0.84	0.90	0.86	0.82	0.90	0.82	0.88	0.90	50.54	8.16
Q9Z2W9	Glutamate receptor 3 OS=Mus musculus GN=Gria3 PE=1 SV=2 - [GRIA3_MOUSE]	29.62	2	19	24	75	0.96	1.11	1.03	1.09	1.14	0.84	0.84	0.86	0.88	0.81	0.83	0.87	0.88	100.46	8.38

Q8BH04	Phosphoenolpyruvate carboxykinase [GTP], mitochondrial OS=Mus musculus GN=Pck2 PE=2 SV=1 - [PCKGM_MOUSE]	14.37	1	7	7	13	0.99	1.10	1.08	1.09	1.07	0.96	0.94	0.86	0.91	1.05	1.07	1.02	1.01	70.48	7.28
Q99LC3	NADH dehydrogenase [ubiquinone] 1 alpha subcomplex subunit 10, mitochondrial OS=Mus musculus GN=Ndufa10 PE=1 SV=1 - [NDUAA_MOUSE]	44.51	1	16	16	69	0.91	0.84	0.96	1.06	1.14	0.90	1.03	0.86	0.95	0.96	1.01	0.93	1.03	40.58	7.78
P61028	Ras-related protein Rab 8B OS=Mus musculus GN=Rab8b PE=1 SV=1 - [RAB8B_MOUSE]	29.47	11	1	7	63	1.09	1.23	1.13	1.11	1.02	0.80	0.73	0.86	0.79	0.77	0.71	1.05	0.97	23.59	9.07
Q3U3R4-3	Isoform 3 of Lipase maturation factor 1 OS=Mus musculus GN=Lmf1 - [LMF1_MOUSE]	8.12	4	2	2	2	0.99	1.35	1.36	1.05	1.01	0.79	0.79	0.86	0.79	1.00	1.01	1.02	1.05	41.42	9.14
Q8BVG4-	Isoform 2 of Dipeptidyl peptidase 9 OS=Mus musculus GN=Dpp9 - [DPP9_MOUSE]	9.13	2	5	6	13	1.00	0.89	1.00	1.08	1.08	0.92	0.91	0.86	0.92	1.01	1.06	1.00	1.07	80.54	6.58
O88444	Adenylate cyclase type 1 OS=Mus musculus GN=Adcy1 PE=2 SV=2 - [ADCY1_MOUSE]	13.60	1	10	10	20	0.99	1.08	1.11	1.08	1.20	0.88	0.93	0.86	0.91	0.86	1.01	0.90	0.96	123.29	8.47
Q80WS3	rRNA/tRNA 2'-O-methyltransferase fibrillarin-like protein 1 OS=Mus musculus GN=Fbll1 PE=2 SV=1 - [FBLL1_MOUSE]	15.61	1	3	5	11	0.97	0.90	0.83	0.98	1.01	0.99	0.98	0.86	0.91	0.89	0.87	1.01	0.93	33.32	10.15
Q9ESW4	Acylglycerol kinase, mitochondrial OS=Mus musculus GN=Agk PE=1 SV=1 - [AGK_MOUSE]	23.52	1	9	9	26	0.94	1.03	1.07	1.15	1.21	0.94	0.94	0.86	0.90	0.91	0.98	1.00	1.03	46.95	8.40
Q9ERF3	WD repeat-containing protein 61 OS=Mus musculus GN=Wdr61 PE=2 SV=1 - [WDR61_MOUSE]	23.61	4	4	4	7	1.04	0.93	0.91	1.05	0.98	1.12	1.02	0.86	0.91	0.93	0.89	0.98	1.00	33.75	5.36
E9QAQ3	Rho GTPase-activating protein 26 OS=Mus musculus GN=Arhgap26 PE=2 SV=1 - [E9QAQ3_MOUSE]	7.51	4	5	5	8	0.89	1.13	1.24	1.09	1.24	0.87	0.97	0.86	0.97	1.04	1.10	0.87	1.04	85.90	6.74
P05132	cAMP-dependent protein kinase catalytic subunit alpha OS=Mus musculus GN=Prkaca PE=1 SV=3 - [KAPCA_MOUSE]	40.46	2	8	13	49	1.05	0.91	0.87	1.17	1.09	0.91	0.85	0.86	0.85	0.82	0.84	1.01	0.95	40.54	8.79

Q60771	Claudin-11 OS=Mus musculus GN=Cldn11 PE=1 SV=1 - [CLD11_MOUSE]	10.63	1	3	3	40	0.99	1.26	1.27	1.01	1.00	0.94	0.91	0.86	0.86	0.85	0.84	0.90	0.93	22.10	7.91
Q9D1M0	Protein SEC13 homolog OS=Mus musculus GN=Sec13 PE=2 SV=3 - [SEC13_MOUSE]	25.16	1	7	7	20	1.03	0.95	0.93	0.95	0.87	0.90	0.78	0.86	0.86	0.85	0.84	0.90	0.87	35.54	5.38
P61164	Alpha-centractin OS=Mus musculus GN=Actr1a PE=2 SV=1 - [ACTZ_MOUSE]	32.98	1	5	10	38	0.92	1.03	1.18	1.05	1.16	0.98	1.21	0.86	0.92	1.00	1.11	0.95	1.28	42.59	6.64
Q9CR10	Oxidoreductase-like domain-containing protein 1 OS=Mus musculus GN=Oxld1 PE=2 SV=1 - [OXLD1_MOUSE]	14.93	2	2	2	4	0.93	0.65	0.66	0.75	0.86	1.05	0.99	0.86	0.98	0.90	0.96	0.88	0.95	22.16	8.05
D3Z5I7	Inactive dipeptidyl peptidase 10 OS=Mus musculus GN=Dpp10 PE=2 SV=1 - [D3Z5I7_MOUSE]	27.35	3	16	17	33	1.12	1.03	0.91	1.09	0.94	0.92	0.82	0.86	0.76	0.91	0.86	0.91	0.84	85.31	6.42
M0QWG6	Retinal rod rhodopsin-sensitive cGMP 3',5'-cyclic phosphodiesterase subunit delta OS=Mus musculus GN=Pde6d PE=4 SV=1 - [M0QWG6_MOUSE]	25.00	2	2	2	3	1.07	0.95	0.89	1.13	1.05	0.92	0.86	0.86	0.80	0.96	0.90	1.02	0.96	14.34	6.14
O89112	LanC-like protein 1 OS=Mus musculus GN=Lancl1 PE=1 SV=1 - [LANC1_MOUSE]	16.79	2	5	5	13	0.97	1.08	1.11	1.03	1.06	0.89	0.97	0.86	0.89	0.92	0.94	1.02	1.03	45.31	7.77
E0CYG3	Sperm flagellar protein 2 OS=Mus musculus GN=Spef2 PE=2 SV=1 - [E0CYG3_MOUSE]	0.70	3	1	1	3	1.02	0.77	0.75	0.86	0.84	0.97	0.94	0.86	0.84	0.95	0.93	0.81	0.79	197.85	5.86
F5H8M6	MCG9349 OS=Mus musculus GN=Rps18-ps3 PE=3 SV=2 - [F5H8M6_MOUSE]	39.16	1	1	6	40	0.46	0.79	1.72	0.92	2.02	0.84	1.83	0.86	1.87	1.37	3.02	0.84	1.84	16.72	10.96
E9Q3K3	Arf-GAP with GTPase, ANK repeat and PH domain-containing protein 1 OS=Mus musculus GN=Agap1 PE=2 SV=1 - [E9Q3K3_MOUSE]	18.91	2	8	10	37	1.06	1.07	1.00	1.02	1.00	0.96	0.93	0.86	0.86	0.97	0.89	1.06	0.98	88.97	8.03
Q9EP69	Phosphatidylinositide phosphatase SAC1 OS=Mus musculus GN=Sacm1l PE=2 SV=1 - [SAC1_MOUSE]	19.08	1	10	10	23	0.96	1.01	1.13	1.18	1.22	0.87	0.88	0.86	0.87	0.93	0.96	0.99	1.04	66.90	7.30
P47962	60S ribosomal protein L5 OS=Mus musculus GN=Rpl5 PE=1 SV=3 - [RL5_MOUSE]	27.61	2	8	8	28	0.95	0.85	0.88	1.01	1.07	0.90	0.97	0.86	0.91	0.88	1.01	0.95	1.04	34.38	9.77

F7BAB2	Protein Tmem132b OS=Mus musculus GN=Tmem132b PE=4 SV=1 - [F7BAB2_MOUSE]	18.18	1	14	14	36	1.00	1.07	1.07	0.93	0.89	0.79	0.76	0.86	0.85	0.77	0.81	0.83	0.82	119.25	4.87
P60670	Nuclear protein localization protein 4 homolog OS=Mus musculus GN=Nploc4 PE=1 SV=3 - [NPL4_MOUSE]	10.53	2	5	5	8	1.05	0.90	0.92	1.04	1.02	0.91	0.77	0.86	0.79	0.93	0.78	0.94	0.82	67.97	6.46
Q60714	Long-chain fatty acid transport protein 1 OS=Mus musculus GN=Slc27a1 PE=1 SV=1 - [S27A1_MOUSE]	5.26	1	3	3	5	1.06	1.30	1.22	1.02	0.91	0.85	0.79	0.86	0.85	0.89	0.80	1.06	0.99	71.23	8.38
Q8R420	ATP-binding cassette sub-family A member 3 OS=Mus musculus GN=Abca3 PE=2 SV=3 - [ABCA3_MOUSE]	1.41	2	2	2	4	0.95	1.20	1.26	1.10	1.16	0.88	0.92	0.86	0.90	1.01	1.07	0.91	0.96	191.85	7.17
A2AUY4	Protein Baz2b OS=Mus musculus GN=Baz2b PE=4 SV=1 - [A2AUY4_MOUSE]	2.07	1	1	1	4	0.71	0.45	0.63	0.99	1.39	0.65	0.90	0.86	1.20	0.70	0.98	0.51	0.72	233.86	6.55
Q80YD6	Secretion-regulating guanine nucleotide exchange factor OS=Mus musculus GN=Sergef PE=2 SV=2 - [SRGEF_MOUSE]	4.96	2	2	2	4	0.94	1.36	1.44	0.93	0.99	1.04	1.11	0.86	0.91	1.22	1.31	0.98	1.05	49.19	6.19
A2ALI5	Adherens junction-associated protein 1 OS=Mus musculus GN=Ajap1 PE=2 SV=1 - [AJAP1_MOUSE]	4.37	1	1	1	2	1.14	1.60	1.40	1.18	1.03	1.04	0.90	0.86	0.75	1.03	0.91	1.08	0.95	44.74	9.42
P27601	Guanine nucleotide-binding protein subunit alpha-13 OS=Mus musculus GN=Gna13 PE=1 SV=1 - [GNA13_MOUSE]	42.97	6	13	14	48	1.11	1.06	0.95	1.11	0.97	0.97	0.89	0.86	0.83	1.03	0.94	0.98	0.92	44.03	8.21
Q9CSU0-	Isoform 2 of Regulation of nuclear pre-mRNA domain-containing protein 1B OS=Mus musculus GN=Rprd1b - [RPR1B_MOUSE]	15.65	5	3	3	6	0.90	0.76	0.98	0.93	1.02	0.79	0.83	0.86	0.96	0.86	0.97	0.84	0.95	33.49	5.64
E9Q2W9	Alpha-actinin-4 (Fragment) OS=Mus musculus GN=Actn4 PE=2 SV=1 - [E9Q2W9_MOUSE]	59.65	2	1	27	162	0.95	0.89	0.94	1.03	1.08	0.90	0.95	0.86	0.90	0.88	0.94	0.82	0.86	59.86	6.01

E9PY27	Cytosolic endo-beta-N-acetylglucosaminidase OS=Mus musculus GN=Engase PE=4 SV=1 - [E9PY27_MOUSE]	12.50	3	1	1	5	1.11	1.04	0.93	1.02	0.92	0.87	0.75	0.86	0.78	0.86	0.78	0.95	0.91	11.19	11.31
Q9CQT2	RNA-binding protein 7 OS=Mus musculus GN=Rbm7 PE=1 SV=1 - [RBM7_MOUSE]	11.70	1	3	3	4	1.06	1.02	0.96	0.88	0.84	0.81	0.76	0.86	0.78	1.06	0.97	0.91	0.86	30.13	9.38
O55135	Eukaryotic translation initiation factor 6 OS=Mus musculus GN=Eif6 PE=1 SV=2 - [IF6_MOUSE]	21.22	2	3	3	9	1.01	1.13	1.02	1.17	1.03	0.96	0.86	0.86	0.84	0.94	0.86	1.05	0.97	26.49	4.74
Q9Z2G6	Protein sel-1 homolog 1 OS=Mus musculus GN=Sel1l PE=2 SV=2 - [SE1L1_MOUSE]	13.54	3	6	6	13	1.18	1.11	1.09	1.02	1.00	0.88	0.84	0.86	0.77	0.88	0.74	1.14	0.98	88.28	5.57
Q8K411-2	Isoform 2 of Presequence protease, mitochondrial OS=Mus musculus GN=Pitrm1 - [PREP_MOUSE]	12.85	3	12	12	21	0.97	1.00	0.92	1.02	1.00	0.85	0.87	0.86	0.90	0.90	0.90	0.90	0.93	117.14	7.12
Q3UVG3	Protein FAM91A1 OS=Mus musculus GN=Fam91a1 PE=1 SV=1 - [F91A1_MOUSE]	3.94	1	3	3	4	1.06	1.04	0.98	1.13	1.11	0.91	0.86	0.86	0.81	0.96	0.93	1.20	1.21	93.40	6.35
Q9EPN1-3	Isoform 3 of Neurobeachin OS=Mus musculus GN=Nbea - [NBEA_MOUSE]	14.12	4	22	27	54	0.98	1.03	1.07	1.10	1.10	0.94	0.95	0.86	0.90	0.94	0.94	0.97	1.05	323.00	6.15
Q9CRA7	ATP synthase subunit s, mitochondrial OS=Mus musculus GN=Atp5s PE=2 SV=1 - [ATP5S_MOUSE]	13.50	1	1	1	2	1.01	0.73	0.72	0.87	0.86	0.98	0.96	0.86	0.85	0.96	0.95	1.03	1.02	23.26	8.06
Q8BUN5	Mothers against decapentaplegic homolog 3 OS=Mus musculus GN=Smad3 PE=1 SV=2 - [SMAD3_MOUSE]	8.71	4	1	3	5	1.02	1.09	1.06	0.99	0.96	0.85	0.83	0.86	0.84	0.93	0.91	0.96	0.94	48.05	7.15
Q80UM7	Mannosyl-oligosaccharide glucosidase OS=Mus musculus GN=Mogs PE=2 SV=1 - [MOGS_MOUSE]	16.07	1	9	9	13	0.89	1.20	1.24	0.96	1.04	0.77	0.83	0.86	0.90	0.74	0.77	0.85	0.92	91.77	9.00
Q3TDN2	FAS-associated factor 2 OS=Mus musculus GN=Faf2 PE=2 SV=2 - [FAF2_MOUSE]	23.37	5	6	6	11	1.04	1.03	0.99	1.04	1.08	0.87	0.87	0.86	0.88	0.95	0.94	1.05	1.00	52.44	5.47
Q14C51	Pentatricopeptide repeat domain-containing protein 3, mitochondrial OS=Mus musculus GN=Ptcd3 PE=2 SV=2 - [PTCD3_MOUSE]	5.55	1	3	3	6	0.91	0.91	1.01	1.06	1.09	0.86	0.96	0.86	0.94	0.91	1.00	0.85	0.91	77.75	5.88

A8Y5H7	Protein Sec14l1 OS=Mus musculus GN=Sec14l1 PE=2 SV=1 - [A8Y5H7_MOUSE]	1.26	3	1	1	2	0.81	0.97	1.21	0.93	1.16	0.83	1.02	0.86	1.06	0.82	1.02	0.78	0.97	81.16	6.34
Q80ZJ1	Ras-related protein Rap 2a OS=Mus musculus GN=Rap2a PE=1 SV=2 - [RAP2A_MOUSE]	58.47	3	3	8	37	1.09	0.98	0.88	1.04	0.97	0.90	0.87	0.86	0.81	0.86	0.80	0.88	0.83	20.63	4.82
P50516	V-type proton ATPase catalytic subunit A OS=Mus musculus GN=Atp6v1a PE=1 SV=2 - [VATA_MOUSE]	69.53	5	36	36	417	0.94	1.00	1.06	1.03	1.11	0.89	0.94	0.86	0.93	0.92	0.97	0.90	0.99	68.28	5.58
P97393	Rho GTPase-activating protein 5 OS=Mus musculus GN=Arhgap5 PE=2 SV=2 - [RHG05_MOUSE]	9.66	2	11	11	19	0.94	0.99	1.04	1.05	0.99	0.93	0.91	0.86	0.94	1.01	0.94	1.03	0.93	172.00	6.34
Q6ZQ38	Cullin-associated NEDD8-dissociated protein 1 OS=Mus musculus GN=Cand1 PE=2 SV=2 - [CAND1_MOUSE]	31.22	3	30	30	77	1.00	0.92	0.90	1.10	1.12	0.90	0.88	0.86	0.86	0.96	0.96	1.01	1.00	136.24	5.78
P57746	V-type proton ATPase subunit D OS=Mus musculus GN=Atp6v1d PE=1 SV=1 - [VATD_MOUSE]	59.11	1	14	14	37	0.94	1.01	1.06	1.02	1.04	0.94	0.92	0.86	0.95	0.86	0.94	0.96	1.00	28.35	9.45
P20917-2	Isoform S-MAG of Myelin-associated glycoprotein OS=Mus musculus GN=Mag - [MAG_MOUSE]	32.13	1	1	14	72	0.90	0.85	0.94	0.95	1.06	0.92	1.02	0.86	0.96	0.81	0.90	0.80	0.89	64.23	5.08
Q9R078	5'-AMP-activated protein kinase subunit beta-1 OS=Mus musculus GN=Prkab1 PE=1 SV=2 - [AAKB1_MOUSE]	18.89	1	3	3	6	1.00	0.84	0.91	1.00	1.04	0.97	0.96	0.86	0.86	0.92	0.93	0.88	0.93	30.29	6.23
Q05CH9	E3 ubiquitin-protein ligase RNF123 (Fragment) OS=Mus musculus GN=Rnf123 PE=2 SV=1 - [Q05CH9_MOUSE]	3.63	7	2	2	2	1.03	1.15	1.12	1.11	1.08	0.89	0.86	0.86	0.84	0.99	0.97	0.99	0.97	140.56	6.74
Q8BWY3	Eukaryotic peptide chain release factor subunit 1 OS=Mus musculus GN=Etf1 PE=1 SV=4 - [ERF1_MOUSE]	8.92	1	4	4	8	1.11	1.13	1.04	0.99	0.89	0.94	0.94	0.86	0.82	0.96	0.87	1.01	1.01	49.00	5.71
Q8CHP8	Phosphoglycolate phosphatase OS=Mus musculus GN=Pgp PE=2 SV=1 - [PGP_MOUSE]	26.48	1	6	6	25	0.90	1.21	1.42	1.02	1.10	0.91	1.04	0.86	1.00	0.87	0.96	0.91	0.96	34.52	5.35

Q6A099	MKIAA0248 protein (Fragment) OS=Mus musculus GN=Gbf1 PE=2 SV=1 - [Q6A099_MOUSE]	1.11	3	2	2	5	1.07	1.26	1.22	1.23	1.14	0.91	0.87	0.86	0.83	1.14	0.97	1.09	1.06	200.06	5.80
E9Q1S3	Protein transport protein Sec23A OS=Mus musculus GN=Sec23a PE=2 SV=1 - [E9Q1S3_MOUSE]	27.72	5	13	16	29	1.13	1.11	1.13	1.07	0.99	0.90	0.89	0.86	1.00	0.89	0.86	0.96	0.87	82.90	7.46
P46460	Vesicle-fusing ATPase OS=Mus musculus GN=Nsf PE=1 SV=2 - [NSF_MOUSE]	72.98	3	46	47	356	0.95	0.92	0.96	1.05	1.10	0.89	0.93	0.86	0.90	0.92	0.99	0.96	1.00	82.56	6.95
Q9WUM3	Coronin-1B OS=Mus musculus GN=Coro1b PE=1 SV=1 - [COR1B_MOUSE]	18.60	2	9	9	17	1.06	1.11	1.04	1.00	0.90	0.85	0.81	0.86	0.79	0.88	0.79	1.03	0.93	53.88	5.78
Q99MV7-	Isoform 3 of RING finger protein 17 OS=Mus musculus GN=Rnf17 - [RNF17_MOUSE]	1.04	3	1	1	1	1.19	9.85	8.28	1.30	1.10	1.27	1.07	0.86	0.72	0.72	0.60	0.48	0.41	121.09	5.48
E9Q2V5	Protein BC037034 OS=Mus musculus GN=BC037034 PE=4 SV=1 - [E9Q2V5_MOUSE]	4.83	3	2	2	4	1.11	1.19	1.07	1.14	1.02	0.96	0.85	0.86	0.77	1.01	0.91	1.10	0.99	62.71	8.68
Q8K3E5-2	Isoform 2 of Jouberin OS=Mus musculus GN=Ahi1 - [AHI1_MOUSE]	5.97	4	5	5	8	1.63	1.32	0.82	0.96	0.62	0.83	0.56	0.86	0.56	1.24	0.86	1.42	0.89	114.86	6.89
Q8K2P6	Rieske domain-containing protein OS=Mus musculus GN=Rfesd PE=1 SV=1 - [RFESD_MOUSE]	7.64	1	1	1	3	1.01	0.79	0.78	0.87	0.86	0.80	0.78	0.86	0.85	0.65	0.65	0.71	0.70	17.95	6.86
Q8CGC7	Bifunctional glutamate/proline--tRNA ligase OS=Mus musculus GN=Eprs PE=1 SV=4 - [SYEP_MOUSE]	28.90	1	34	34	70	0.98	0.98	1.01	1.08	1.10	0.95	0.95	0.86	0.88	0.93	0.98	0.94	0.97	169.97	7.66
Q64514-2	Isoform Short of Tripeptidyl-peptidase 2 OS=Mus musculus GN=Tpp2 - [TPP2_MOUSE]	19.06	2	20	20	38	0.97	0.97	0.98	1.00	1.03	0.94	0.93	0.86	0.90	0.94	0.98	0.93	0.98	138.38	6.38
Q8C4T8	G protein-activated inward rectifier potassium channel 2 OS=Mus musculus GN=Kcnj6 PE=2 SV=1 - [Q8C4T8_MOUSE]	10.14	4	3	3	6	0.92	0.92	1.00	1.18	1.14	0.79	0.96	0.86	0.93	0.88	1.06	0.73	0.87	47.45	5.52
P35293	Ras-related protein Rab 18 OS=Mus musculus GN=Rab18 PE=2 SV=2 - [RAB18_MOUSE]	59.71	1	10	10	17	1.05	1.03	0.98	1.12	1.06	0.91	0.91	0.86	0.84	0.95	0.91	0.95	0.90	23.02	5.36

O88741	Ganglioside-induced differentiation-associated protein 1 OS=Mus musculus GN=Gdap1 PE=1 SV=1 - [GDAP1_MOUSE]	37.71	2	9	10	22	0.97	1.04	0.98	1.07	1.07	0.82	0.79	0.86	0.81	0.86	0.89	0.88	0.84	41.28	8.37
Q61334	B-cell receptor-associated protein 29 OS=Mus musculus GN=Bcap29 PE=1 SV=1 - [BAP29_MOUSE]	9.58	1	2	2	3	0.98	0.96	0.98	1.18	1.20	0.88	0.89	0.86	0.88	0.92	0.94	0.98	1.01	27.95	9.72
Q8BP78	Protein FRA10AC1 homolog OS=Mus musculus GN=Fra10ac1 PE=1 SV=3 - [F10C1_MOUSE]	8.57	1	2	2	4	0.85	0.73	0.86	0.88	1.04	0.74	0.86	0.86	1.01	0.76	0.89	0.64	0.75	37.18	7.28
D3YXJ0	Protein Dgkh OS=Mus musculus GN=Dgkh PE=4 SV=1 - [D3YXJ0_MOUSE]	11.25	1	9	9	16	1.00	0.92	0.90	1.08	1.07	0.90	0.87	0.86	0.85	1.00	1.02	1.01	1.00	127.28	6.33
Q9JM63	ATP-sensitive inward rectifier potassium channel 10 OS=Mus musculus GN=Kcnj10 PE=1 SV=1 - [IRK10_MOUSE]	10.82	1	3	3	6	1.32	1.01	0.76	1.02	0.74	0.83	0.60	0.86	0.65	0.88	0.67	0.83	0.62	42.41	8.29
Q9JJF3	Bifunctional lysine-specific demethylase and histidyl-hydroxylase NO66 OS=Mus musculus GN=No66 PE=1 SV=2 - [NO66_MOUSE]	4.15	1	2	2	3	1.01	0.93	0.92	1.11	1.10	1.02	1.01	0.86	0.85	1.06	1.05	0.66	0.66	67.51	8.66
P62827	GTP-binding nuclear protein Ran OS=Mus musculus GN=Ran PE=1 SV=3 - [RAN_MOUSE]	42.13	3	8	8	28	0.92	0.99	0.97	1.04	1.09	0.91	0.94	0.86	0.88	0.91	0.95	0.95	1.03	24.41	7.49
P67871	Casein kinase II subunit beta OS=Mus musculus GN=Csnk2b PE=1 SV=1 - [CSK2B_MOUSE]	51.16	7	10	10	41	0.86	0.94	1.05	1.00	1.11	0.90	0.95	0.86	0.91	0.93	0.94	0.92	1.03	24.93	5.55
Q99M85	Transcriptional repressor scratch 1 OS=Mus musculus GN=Scrt1 PE=1 SV=1 - [SCRT1_MOUSE]	5.17	1	1	1	2	0.87	0.95	1.10	0.88	1.02	0.88	1.01	0.86	0.99	0.91	1.06	0.76	0.87	35.90	9.54
P40240	CD9 antigen OS=Mus musculus GN=Cd9 PE=1 SV=2 - [CD9_MOUSE]	16.37	1	3	3	7	0.98	1.15	1.13	0.98	0.93	0.85	1.13	0.86	0.98	0.91	1.02	0.98	1.05	25.24	7.23
Q9D0L8	mRNA cap guanine-N7 methyltransferase OS=Mus musculus GN=Rnmt PE=1 SV=1 - [MCES_MOUSE]	21.72	6	7	7	11	0.92	0.97	0.96	0.95	0.96	0.90	0.98	0.86	0.89	0.92	0.97	1.06	0.99	53.26	6.48

P21279	Guanine nucleotide-binding protein G(q) subunit alpha OS=Mus musculus GN=Gnaq PE=1 SV=4 - [GNAQ_MOUSE]	43.73	1	9	13	41	1.10	1.14	1.07	1.10	1.02	0.88	0.82	0.86	0.78	0.91	0.87	0.98	0.97	42.13	5.68
Q8R123	FAD synthase OS=Mus musculus GN=Flad1 PE=1 SV=1 - [FAD1_MOUSE]	6.91	3	3	3	3	1.04	0.92	0.98	1.19	1.14	1.01	0.94	0.86	0.92	0.95	0.96	1.00	1.07	54.73	6.64
P23819-4	Isoform 4 of Glutamate receptor 2 OS=Mus musculus GN=Gria2 - [GRIA2_MOUSE]	33.98	6	20	28	93	1.01	1.06	1.01	1.13	1.16	0.87	0.89	0.86	0.87	0.84	0.88	0.89	0.94	102.79	7.72
G5E8R3	Pyruvate carboxylase OS=Mus musculus GN=Pcx PE=3 SV=1 - [G5E8R3_MOUSE]	32.68	3	30	30	93	0.94	0.96	1.05	1.05	1.13	0.89	0.95	0.86	0.89	0.90	1.00	0.94	0.99	129.62	6.71
P14106	Complement C1q subcomponent subunit B OS=Mus musculus GN=C1qb PE=1 SV=2 - [C1QB_MOUSE]	21.74	1	4	5	12	0.99	1.04	1.08	0.92	0.95	0.80	0.78	0.86	0.86	0.86	0.85	0.95	1.00	26.70	8.15
P80315	T-complex protein 1 subunit delta OS=Mus musculus GN=Cct4 PE=1 SV=3 - [TCPD_MOUSE]	42.86	3	18	19	37	1.08	1.01	0.99	1.13	1.05	0.96	0.91	0.86	0.84	0.95	0.90	1.09	0.99	58.03	8.02
Q91XI1-2	Isoform 2 of tRNA-dihydrouridine(47) synthase [NAD(P)(+)]-like OS=Mus musculus GN=Dus3l - [DUS3L_MOUSE]	4.93	2	1	1	2	0.94	1.20	1.27	0.87	0.93	0.86	0.91	0.86	0.92	0.82	0.87	1.03	1.09	46.67	7.58
Q8VE19	WD repeat-containing protein mio OS=Mus musculus GN=Mios PE=2 SV=2 - [MIO_MOUSE]	6.17	1	4	4	6	0.92	1.20	1.26	0.95	1.14	0.87	0.94	0.86	0.87	0.90	0.94	0.93	1.04	98.27	6.71
A6H630	UPF0364 protein C6orf211 homolog OS=Mus musculus PE=2 SV=1 - [CF211_MOUSE]	11.39	5	4	4	8	1.02	1.05	1.02	1.09	1.09	0.88	0.95	0.86	0.85	0.91	0.88	0.98	1.03	50.52	5.92
Q8BJ71	Nuclear pore complex protein Nup93 OS=Mus musculus GN=Nup93 PE=2 SV=1 - [NUP93_MOUSE]	11.36	2	7	7	12	0.87	0.92	1.14	1.09	1.16	0.91	1.02	0.86	0.98	0.91	1.05	0.93	1.07	93.22	5.72
O70503	Estradiol 17-beta-dehydrogenase 12 OS=Mus musculus GN=Hsd17b12 PE=2 SV=1 - [DHB12_MOUSE]	20.19	2	5	5	9	0.96	0.86	0.99	1.14	1.17	0.86	0.89	0.86	0.90	0.90	0.94	0.87	0.83	34.72	9.52
O35386	Phytanoyl-CoA dioxygenase, peroxisomal OS=Mus musculus GN=Phyh PE=1 SV=1 - [PAHX_MOUSE]	4.14	1	1	1	2	1.02	1.21	1.17	0.93	0.91	0.98	0.96	0.86	0.84	0.93	0.91	0.96	0.94	38.58	7.53

Q8R180	ERO1-like protein alpha OS=Mus musculus GN=Ero1l PE=1 SV=2 - [ERO1A_MOUSE]	5.60	1	2	2	4	0.88	0.93	1.06	1.01	1.15	0.90	1.02	0.86	0.98	0.94	1.08	0.99	1.13	54.05	6.54
O35682	Myeloid-associated differentiation marker OS=Mus musculus GN=Myadm PE=2 SV=2 - [MYADM_MOUSE]	10.31	1	3	3	47	1.01	1.28	1.23	1.12	1.11	0.83	0.81	0.86	0.87	0.93	0.90	1.08	1.04	35.26	8.31
Q9CX34	Suppressor of G2 allele of SKP1 homolog OS=Mus musculus GN=Sugt1 PE=2 SV=3 - [SUGT1_MOUSE]	58.33	1	16	17	61	0.99	0.87	0.88	0.95	0.97	0.83	0.87	0.86	0.91	0.84	0.84	0.79	0.83	38.14	5.45
Q8CCJ9-2	Isoform 2 of PHD finger protein 20-like protein 1 OS=Mus musculus GN=Phf20l1 - [P20L1_MOUSE]	9.00	6	2	2	2	0.95	0.77	0.81	0.89	0.93	0.84	0.88	0.86	0.91	0.78	0.82	0.77	0.81	35.23	8.69
P28271	Cytoplasmic aconitate hydratase OS=Mus musculus GN=Aco1 PE=1 SV=3 - [ACOC_MOUSE]	20.81	2	15	15	25	0.96	0.99	1.09	1.02	1.09	0.88	0.94	0.86	0.89	0.99	0.98	0.94	1.08	98.06	7.50
Q9JLC4-2	Isoform 2 of VPS10 domain-containing receptor SorCS1 OS=Mus musculus GN=Sorcs1 - [SORC1_MOUSE]	5.41	8	5	5	11	0.91	0.98	1.07	0.84	0.90	0.76	0.77	0.87	0.93	0.82	0.77	0.85	0.79	127.79	7.28
Q9D6K8	FUN14 domain-containing protein 2 OS=Mus musculus GN=Fundc2 PE=2 SV=1 - [FUND2_MOUSE]	23.18	1	4	4	10	1.12	0.79	0.71	1.14	1.05	0.94	0.89	0.87	0.82	0.95	0.83	0.89	0.83	16.55	9.70
A3KFX0	Cytosolic 5'-nucleotidase 1A OS=Mus musculus GN=Nt5c1a PE=2 SV=1 - [5NT1A_MOUSE]	4.11	1	1	1	2	1.11	0.99	0.89	1.07	0.97	0.98	0.88	0.87	0.78	0.89	0.81	1.01	0.91	40.65	7.06
Q91VA7	Isocitrate dehydrogenase 3 (NAD+) beta OS=Mus musculus GN=Idh3b PE=2 SV=1 - [Q91VA7_MOUSE]	38.02	1	12	12	77	0.95	0.91	0.98	1.04	1.07	0.89	0.94	0.87	0.92	0.89	0.96	0.91	0.98	42.17	8.60
P11983	T-complex protein 1 subunit alpha OS=Mus musculus GN=Tcp1 PE=1 SV=3 - [TCPA_MOUSE]	43.53	3	21	21	56	1.07	0.96	0.95	1.13	1.01	0.92	0.82	0.87	0.81	0.98	0.92	1.03	0.90	60.41	6.16
P32037	Solute carrier family 2, facilitated glucose transporter member 3 OS=Mus musculus GN=Slc2a3 PE=1 SV=1 - [GTR3_MOUSE]	14.60	1	8	8	41	1.02	1.00	1.03	1.15	1.18	0.86	0.87	0.87	0.90	0.95	0.94	1.02	0.98	53.44	4.98

O55142	60S ribosomal protein L35a OS=Mus musculus GN=Rpl35a PE=2 SV=2 - [RL35A_MOUSE]	6.36	1	1	1	1	1.01	1.01	1.00	0.96	0.96	0.90	0.89	0.87	0.86	0.92	0.91	0.86	0.86	12.55	10.89
Q61151	Serine/threonine-protein phosphatase 2A 56 kDa regulatory subunit epsilon isoform OS=Mus musculus GN=Ppp2r5e PE=2 SV=3 - [2A5E_MOUSE]	21.84	1	7	9	19	1.03	0.97	0.92	1.12	1.03	1.01	0.92	0.87	0.87	0.97	0.98	1.08	1.05	54.68	6.95
Q60675	Laminin subunit alpha-2 OS=Mus musculus GN=Lama2 PE=1 SV=1 - [LAMA2_MOUSE]	1.45	2	4	4	8	0.85	1.06	1.24	0.88	1.00	0.83	0.98	0.87	1.01	0.86	0.99	0.76	0.87	342.46	6.09
P97390	Vacuolar protein sorting-associated protein 45 OS=Mus musculus GN=Vps45 PE=1 SV=1 - [VPS45_MOUSE]	12.28	1	7	7	15	1.01	0.96	0.97	1.06	1.11	0.83	0.83	0.87	0.89	0.95	0.86	0.94	0.95	65.01	8.25
Q02248	Catenin beta-1 OS=Mus musculus GN=Ctnnb1 PE=1 SV=1 - [CTNB1_MOUSE]	34.19	8	17	20	64	0.94	0.94	1.04	1.04	1.10	0.95	0.98	0.87	0.97	0.94	0.99	1.03	1.06	85.42	5.86
Q06185	ATP synthase subunit e, mitochondrial OS=Mus musculus GN=Atp5i PE=1 SV=2 - [ATP5I_MOUSE]	52.11	2	3	3	18	0.95	0.87	1.06	1.03	1.04	0.88	0.95	0.87	0.99	0.92	1.03	0.87	0.93	8.23	9.35
Q61598	Rab GDP dissociation inhibitor beta OS=Mus musculus GN=Gdi2 PE=1 SV=1 - [GDIB_MOUSE]	74.38	2	22	30	142	1.03	0.85	0.86	1.05	1.00	0.88	0.89	0.87	0.83	0.90	0.88	0.94	0.93	50.50	6.25
P17426-2	Isoform B of AP-2 complex subunit alpha-1 OS=Mus musculus GN=Ap2a1 - [AP2A1_MOUSE]	57.28	5	31	42	144	1.02	1.11	1.13	1.15	1.14	0.89	0.87	0.87	0.86	0.92	0.90	1.06	1.04	105.41	7.66
Q8R5K2-	Isoform 2 of Ubiquitin carboxyl-terminal hydrolase 33 OS=Mus musculus GN=Usp33 - [UBP33_MOUSE]	3.00	2	2	2	4	1.09	0.96	0.88	0.98	0.90	1.02	0.93	0.87	0.79	0.89	0.82	0.96	0.88	101.77	5.68
P45952	Medium-chain specific acyl-CoA dehydrogenase, mitochondrial OS=Mus musculus GN=Acadm PE=1 SV=1 - [ACADM_MOUSE]	17.58	2	6	6	18	1.03	1.18	1.09	1.07	1.09	0.97	0.93	0.87	0.86	1.01	0.98	1.06	1.01	46.45	8.37
P07146	Anionic trypsin-2 OS=Mus musculus GN=Prss2 PE=2 SV=1 - [TRY2_MOUSE]	8.13	1	1	1	2	0.97	1.14	1.18	0.91	0.94	0.96	0.98	0.87	0.89	0.89	0.92	0.84	0.87	26.19	4.63

A6PWX7	Ubiquinol-cytochrome c reductase complex chaperone CBP3 homolog OS=Mus musculus GN=Uqcc PE=2 SV=1 - [A6PWX7_MOUSE]	11.89	7	2	2	6	0.93	1.06	1.01	1.02	1.08	0.85	0.89	0.87	1.01	1.00	1.04	0.95	1.02	26.57	8.00
Q80UW2	F-box only protein 2 OS=Mus musculus GN=Fbxo2 PE=1 SV=1 - [FBX2_MOUSE]	57.24	1	11	11	53	0.94	0.93	1.07	0.94	1.00	0.78	0.81	0.87	0.93	0.76	0.84	0.79	0.82	33.66	4.28
Q5SW19	Clustered mitochondria protein homolog OS=Mus musculus GN=Cluh PE=2 SV=2 - [CLU_MOUSE]	3.95	3	4	4	7	0.98	1.21	1.19	1.05	1.03	0.99	1.01	0.87	0.90	0.97	0.98	0.99	1.00	147.97	6.02
Q8BH61	Coagulation factor XIII A chain OS=Mus musculus GN=F13a1 PE=2 SV=3 - [F13A_MOUSE]	2.32	1	1	1	2	1.00	0.73	0.73	1.06	1.06	0.95	0.94	0.87	0.86	0.99	0.99	0.97	0.97	83.15	5.92
Q924T2	28S ribosomal protein S2, mitochondrial OS=Mus musculus GN=Mrps2 PE=2 SV=1 - [RT02_MOUSE]	12.71	1	2	2	4	0.91	1.32	1.44	1.03	1.12	0.88	0.96	0.87	0.94	0.97	1.06	1.04	1.13	32.29	9.14
Q9DC70	NADH dehydrogenase [ubiquinone] iron-sulfur protein 7, mitochondrial OS=Mus musculus GN=Ndufs7 PE=1 SV=1 - [NDUS7_MOUSE]	29.91	1	6	6	12	0.96	0.84	0.92	1.13	1.17	0.98	0.98	0.87	0.95	0.89	0.97	0.92	0.99	24.67	9.92
Q9WTX6	Cullin-1 OS=Mus musculus GN=Cul1 PE=1 SV=1 - [CUL1_MOUSE]	13.27	3	9	9	15	1.02	1.05	0.94	1.05	1.01	0.82	0.88	0.87	0.87	0.93	0.86	0.95	0.96	89.63	8.00
Q9JJI8	60S ribosomal protein L38 OS=Mus musculus GN=Rpl38 PE=2 SV=3 - [RL38_MOUSE]	50.00	1	5	5	10	0.90	0.86	0.83	0.77	0.86	0.80	0.92	0.87	0.97	0.80	0.82	0.83	0.83	8.20	10.10
Q9DB41-[illegible]	Isoform 2 of Mitochondrial glutamate carrier 2 OS=Mus musculus GN=Slc25a18 - [GHC2_MOUSE]	16.99	2	3	5	8	0.98	1.16	1.25	1.14	1.16	0.75	0.76	0.87	0.86	0.87	0.91	0.88	0.96	33.28	9.29
P97300-3	Isoform 3 of Neuroplastin OS=Mus musculus GN=Nptn - [NPTN_MOUSE]	31.41	5	1	9	172	1.26	0.56	0.44	1.15	0.91	1.09	0.85	0.87	0.68	0.92	0.73	0.86	0.68	30.80	7.49
Q9D0J4	ADP-ribosylation factor-like protein 2 OS=Mus musculus GN=Arl2 PE=1 SV=1 - [ARL2_MOUSE]	15.76	1	2	2	3	1.07	1.07	0.99	1.19	1.11	0.98	0.91	0.87	0.81	0.95	0.89	1.14	1.06	20.85	5.96
Q61161	Mitogen-activated protein kinase kinase kinase kinase 2 OS=Mus musculus GN=Map4k2 PE=1 SV=1 - [M4K2_MOUSE]	4.14	2	2	2	4	0.92	1.13	1.23	1.13	1.23	0.98	1.06	0.87	0.94	1.07	1.16	1.21	1.32	91.21	6.46

Q9WV18	Gamma-aminobutyric acid type B receptor subunit 1 OS=Mus musculus GN=Gabbr1 PE=1 SV=1 - [GABR1_MOUSE]	11.56	4	9	9	23	0.93	0.96	1.02	1.12	1.13	0.89	1.00	0.87	0.94	0.91	1.01	0.84	0.88	108.15	8.21
Q3THK7	GMP synthase [glutamine-hydrolyzing] OS=Mus musculus GN=Gmps PE=1 SV=2 - [GUAA_MOUSE]	38.67	1	21	21	49	1.06	0.93	0.88	1.07	1.05	0.98	0.90	0.87	0.88	1.01	0.93	0.96	1.01	76.67	6.73
Q4PJX1-3	Isoform 3 of Protein odr-4 homolog OS=Mus musculus GN=Odr4 - [ODR4_MOUSE]	2.17	3	1	1	2	0.97	1.14	1.17	1.12	1.15	0.83	0.85	0.87	0.89	0.94	0.97	1.02	1.05	46.47	6.27
P27659	60S ribosomal protein L3 OS=Mus musculus GN=Rpl3 PE=2 SV=3 - [RL3_MOUSE]	29.78	3	12	12	30	0.94	0.93	0.95	0.98	0.97	0.85	0.87	0.87	0.95	0.97	1.00	0.86	0.98	46.08	10.21
Q8BSH3	Tropomyosin alpha-1 chain OS=Mus musculus GN=Tpm1 PE=2 SV=1 - [Q8BSH3_MOUSE]	65.14	3	6	26	348	1.14	0.64	0.52	0.92	0.80	2.02	1.41	0.87	0.83	0.92	0.94	1.02	0.71	32.68	4.74
Q8CJF9	Protein argonaute-3 OS=Mus musculus GN=Ago3 PE=2 SV=2 - [AGO3_MOUSE]	8.95	4	1	6	14	1.02	1.19	1.16	1.17	1.15	1.01	0.99	0.87	0.85	0.83	0.81	0.78	0.77	97.21	9.10
Q9DBR3-	Isoform 3 of Armadillo repeat-containing protein 8 OS=Mus musculus GN=Armc8 - [ARMC8_MOUSE]	12.32	4	3	3	8	1.04	1.17	1.25	1.01	0.98	0.85	0.80	0.87	0.94	0.87	0.95	1.07	1.11	39.72	7.58
Q3TDX8-	Isoform 2 of Cytochrome b5 reductase 4 OS=Mus musculus GN=Cyb5r4 - [NB5R4_MOUSE]	5.03	3	1	2	22	0.83	1.02	1.22	0.94	1.13	0.99	1.18	0.87	1.04	0.89	1.07	0.83	1.00	53.96	6.44
Q64387	Prepronociceptin OS=Mus musculus GN=Pnoc PE=2 SV=1 - [PNOC_MOUSE]	37.97	2	4	4	6	1.54	1.00	0.65	0.81	0.57	0.80	0.60	0.87	0.65	0.95	0.63	0.99	0.70	20.87	8.56
D3YU05	Glyceraldehyde-3-phosphate dehydrogenase OS=Mus musculus GN=Gm6316 PE=3 SV=1 - [D3YU05_MOUSE]	44.54	1	1	13	230	0.80	0.86	1.07	1.42	1.78	1.12	1.39	0.87	1.08	1.11	1.38	1.38	1.73	36.52	8.46
Q8VDC0	Probable leucine--tRNA ligase, mitochondrial OS=Mus musculus GN=Lars2 PE=2 SV=1 - [SYLM_MOUSE]	11.42	1	7	7	14	1.01	1.16	1.06	1.03	0.95	0.96	0.95	0.87	0.86	1.04	0.91	1.11	1.05	101.41	8.19

Q8BMF4	Dihydrolipoyllysine-residue acetyltransferase component of pyruvate dehydrogenase complex, mitochondrial OS=Mus musculus GN=Dlat PE=1 SV=2 - [ODP2_MOUSE]	39.72	1	19	19	148	1.02	0.92	0.92	1.01	1.01	0.89	0.87	0.87	0.85	0.90	0.91	0.83	0.81	67.90	8.57
Q9ER72	Cysteine--tRNA ligase, cytoplasmic OS=Mus musculus GN=Cars PE=1 SV=2 - [SYCC_MOUSE]	26.71	2	18	18	43	0.97	1.01	1.06	1.04	1.05	0.86	0.98	0.87	0.97	0.91	0.95	0.92	0.95	94.80	6.76
P51141	Segment polarity protein dishevelled homolog DVL-1 OS=Mus musculus GN=Dvl1 PE=1 SV=2 - [DVL1_MOUSE]	6.76	1	2	3	3	0.87	0.98	1.12	1.03	1.18	0.80	0.92	0.87	0.99	0.99	1.13	0.75	0.86	75.31	7.02
Q9QZE7	Translin-associated protein X OS=Mus musculus GN=Tsnax PE=1 SV=1 - [TSNAX_MOUSE]	37.93	1	9	9	15	1.02	1.24	1.21	1.11	1.10	0.87	0.91	0.87	0.90	0.92	0.89	1.07	1.05	32.91	6.55
Q6PB93	Polypeptide N-acetylgalactosaminyltransferase 2 OS=Mus musculus GN=Galnt2 PE=2 SV=1 - [GALT2_MOUSE]	8.07	2	3	3	4	0.88	0.89	1.01	0.98	1.06	0.92	1.03	0.87	0.98	0.81	0.89	0.84	0.95	64.47	8.53
A2AI19	Glutamate receptor ionotropic, NMDA 1 OS=Mus musculus GN=Grin1 PE=2 SV=1 - [A2AI19_MOUSE]	14.54	8	12	12	37	1.10	1.25	1.11	1.21	1.13	0.81	0.76	0.87	0.82	0.82	0.81	0.88	0.86	101.25	8.27
Q9ERE9	C-Jun-amino-terminal kinase-interacting protein 2 OS=Mus musculus GN=Mapk8ip2 PE=1 SV=1 - [JIP2_MOUSE]	3.49	1	2	2	3	0.88	0.88	1.00	0.84	0.95	1.03	1.16	0.87	0.99	0.92	1.05	1.08	1.23	89.84	4.42
Q9DCP2	Sodium-coupled neutral amino acid transporter 3 OS=Mus musculus GN=Slc38a3 PE=1 SV=1 - [S38A3_MOUSE]	9.50	1	4	4	11	1.05	0.90	0.92	1.01	1.02	0.85	0.92	0.87	0.93	0.92	0.96	0.77	0.85	55.56	7.09
Q8CFV9	Riboflavin kinase OS=Mus musculus GN=Rfk PE=1 SV=2 - [RIFK_MOUSE]	12.90	1	2	2	2	0.99	0.66	0.67	0.92	0.92	0.86	0.87	0.87	0.88	0.90	0.91	0.87	0.88	17.43	7.55
P31324	cAMP-dependent protein kinase type II-beta regulatory subunit OS=Mus musculus GN=Prkar2b PE=1 SV=3 - [KAP3_MOUSE]	63.94	2	18	20	136	0.91	1.13	1.18	1.03	1.10	0.92	0.97	0.87	1.00	0.93	1.04	1.07	1.15	46.14	4.98
Q8R3B7-2	Isoform 2 of Bromodomain-containing protein 8 OS=Mus musculus GN=Brd8 - [BRD8_MOUSE]	1.03	4	1	1	1	0.93	0.79	0.85	0.89	0.96	0.98	1.05	0.87	0.93	1.07	1.16	0.74	0.80	95.06	4.68

P58252	Elongation factor 2 OS=Mus musculus GN=Eef2 PE=1 SV=2 - [EF2_MOUSE]	41.26	1	30	31	125	0.96	0.96	0.99	1.06	1.09	0.89	0.91	0.87	0.89	0.91	0.91	0.93	0.93	95.25	6.83
E0CYH9	Carboxyl-terminal PDZ ligand of neuronal nitric oxide synthase protein (Fragment) OS=Mus musculus GN=Nos1ap PE=2 SV=1 - [E0CYH9_MOUSE]	20.61	5	2	2	2	0.90	0.87	0.97	0.94	1.05	0.87	0.97	0.87	0.97	0.82	0.92	0.85	0.95	25.42	9.16
Q8BMP6	Golgi resident protein GCP60 OS=Mus musculus GN=Acbd3 PE=1 SV=3 - [GCP60_MOUSE]	23.05	1	7	7	14	0.97	0.91	0.99	0.95	1.02	0.96	0.98	0.87	0.92	0.99	0.94	0.99	1.01	60.14	5.11
P14685	26S proteasome non-ATPase regulatory subunit 3 OS=Mus musculus GN=Psmd3 PE=1 SV=3 - [PSMD3_MOUSE]	38.11	2	17	17	43	1.00	1.00	1.04	1.09	1.07	0.91	0.88	0.87	0.87	0.91	0.92	0.87	0.92	60.68	8.44
B9EKN8	TRAF2 and NCK interacting kinase OS=Mus musculus GN=Tnik PE=2 SV=1 - [B9EKN8_MOUSE]	18.20	10	13	16	46	1.04	0.94	0.94	0.96	0.94	0.91	0.83	0.87	0.89	0.85	0.80	0.95	0.89	153.92	7.09
B1AXS6	Zinc finger MYM-type protein 3 OS=Mus musculus GN=Zmym3 PE=2 SV=1 - [B1AXS6_MOUSE]	4.30	7	3	3	8	1.06	0.83	0.87	0.93	0.97	0.91	0.86	0.87	0.80	0.97	0.82	0.93	0.94	141.97	6.04
Q8VD33	Small glutamine-rich tetratricopeptide repeat-containing protein beta OS=Mus musculus GN=Sgtb PE=2 SV=1 - [SGTB_MOUSE]	25.66	3	6	6	19	0.84	0.73	0.79	0.77	0.89	0.80	0.94	0.87	1.01	0.77	0.97	0.74	0.85	33.41	4.92
O35350	Calpain-1 catalytic subunit OS=Mus musculus GN=Capn1 PE=2 SV=1 - [CAN1_MOUSE]	8.42	1	5	5	8	0.97	0.74	0.88	1.02	1.09	0.85	0.87	0.87	0.88	0.91	0.91	0.85	0.85	82.05	5.87
Q9JHU4	Cytoplasmic dynein 1 heavy chain 1 OS=Mus musculus GN=Dync1h1 PE=1 SV=2 - [DYHC1_MOUSE]	38.85	3	151	152	427	0.96	1.02	1.07	1.12	1.16	0.89	0.90	0.87	0.90	0.95	0.97	1.02	1.07	531.71	6.42
Q9R1V4	Disintegrin and metalloproteinase domain-containing protein 11 OS=Mus musculus GN=Adam11 PE=1 SV=2 - [ADA11_MOUSE]	7.63	2	4	4	8	1.12	1.02	1.10	1.08	0.94	0.93	0.91	0.87	0.80	1.06	1.07	0.92	0.88	84.08	7.50
P12815	Programmed cell death protein 6 OS=Mus musculus GN=Pdcd6 PE=1 SV=2 - [PDCD6_MOUSE]	35.60	1	5	5	8	0.96	0.92	1.02	1.09	1.22	0.97	1.02	0.87	0.96	0.98	0.99	1.04	1.04	21.85	5.40

P47856-2	Isoform 2 of Glutamine--fructose-6-phosphate aminotransferase [isomerizing] 1 OS=Mus musculus GN=Gfpt1 - [GFPT1_MOUSE]	4.99	3	3	3	5	0.98	1.16	1.12	1.06	0.98	0.94	0.92	0.87	0.88	1.04	0.98	0.94	0.96	76.67	6.77
Q8C985-2	Isoform 2b of Neurexin-3-beta OS=Mus musculus GN=Nrxn3 - [NRX3B_MOUSE]	20.57	3	1	8	30	1.20	0.75	0.62	1.15	0.96	1.01	0.83	0.87	0.72	1.18	0.98	0.90	0.75	62.04	9.31
D6RGQ0	Complement factor H OS=Mus musculus GN=Cfh PE=2 SV=1 - [D6RGQ0_MOUSE]	8.65	8	8	8	13	0.87	1.10	1.30	0.73	0.86	0.80	0.93	0.87	0.98	0.86	0.99	0.82	0.89	124.97	6.83
O35864	COP9 signalosome complex subunit 5 OS=Mus musculus GN=Cops5 PE=1 SV=3 - [CSN5_MOUSE]	34.73	1	8	8	22	0.91	0.95	0.98	0.93	0.96	0.84	0.95	0.87	1.07	0.88	1.04	0.85	0.92	37.52	6.54
Q99K46	Ubiquitin carboxyl-terminal hydrolase 11 OS=Mus musculus GN=Usp11 PE=2 SV=4 - [UBP11_MOUSE]	4.23	1	1	3	6	1.18	1.19	1.01	0.99	0.84	0.82	0.69	0.87	0.74	0.97	0.82	0.98	0.83	105.32	4.96
E0CXE0	Protein Grip2 OS=Mus musculus GN=Grip2 PE=2 SV=1 - [E0CXE0_MOUSE]	0.97	3	1	1	1	0.90	1.02	1.13	1.02	1.13	0.86	0.95	0.87	0.97	0.82	0.91	0.89	0.99	88.00	7.01
J3QK23	Uncharacterized protein OS=Mus musculus GN=Gm9825 PE=4 SV=1 - [J3QK23_MOUSE]	25.18	3	5	5	17	1.04	0.87	0.82	1.05	1.00	0.80	0.74	0.87	0.89	0.87	0.79	0.86	0.77	31.72	11.90
Q3V117	ATP-citrate synthase OS=Mus musculus GN=Acly PE=2 SV=1 - [Q3V117_MOUSE]	43.51	3	36	36	106	0.97	1.02	1.07	1.06	1.09	0.91	0.90	0.87	0.90	0.97	1.02	1.06	1.11	120.72	7.44
D6RGB9	CUGBP Elav-like family member 6 OS=Mus musculus GN=Celf6 PE=2 SV=1 - [D6RGB9_MOUSE]	14.81	4	1	2	2	0.80	0.73	0.92	0.97	1.22	0.90	1.13	0.87	1.09	1.05	1.32	0.91	1.15	17.00	8.92
Q9WVQ1	Membrane-associated guanylate kinase, WW and PDZ domain-containing protein 2 OS=Mus musculus GN=Magi2 PE=1 SV=2 - [MAGI2_MOUSE]	28.71	4	25	26	59	0.86	0.96	1.12	0.93	1.09	0.84	0.92	0.87	1.02	0.79	0.92	0.78	0.95	140.83	6.19
O35963	Ras-related protein Rab 33B OS=Mus musculus GN=Rab33b PE=1 SV=1 - [RB33B_MOUSE]	23.58	11	3	5	52	1.10	1.00	0.90	1.05	1.00	0.94	0.83	0.87	0.82	1.00	0.91	1.02	0.88	25.75	7.69
P84089	Enhancer of rudimentary homolog OS=Mus musculus GN=Erh PE=1 SV=1 - [ERH_MOUSE]	44.23	3	5	5	36	0.95	0.70	0.76	0.89	0.94	0.85	0.94	0.87	0.91	0.80	0.82	0.72	0.83	12.25	5.92

D3YWB9	Contactin-associated protein-like 4 OS=Mus musculus GN=Cntnap4 PE=2 SV=1 - [D3YWB9_MOUSE]	4.66	2	5	5	7	1.02	1.13	1.09	0.99	0.95	0.94	0.88	0.87	0.86	0.97	0.96	1.07	1.02	144.67	7.02
D3YVE6	MCG18601 OS=Mus musculus GN=Rpl7a-ps10 PE=4 SV=1 - [D3YVE6_MOUSE]	22.93	11	6	6	18	1.01	0.86	0.88	1.04	0.97	0.88	0.87	0.87	0.81	0.97	0.90	0.96	0.91	30.02	10.45
A2AIX0	72 kDa inositol polyphosphate 5-phosphatase OS=Mus musculus GN=Inpp5e PE=4 SV=1 - [A2AIX0_MOUSE]	1.49	2	1	1	2	0.94	1.12	1.19	1.01	1.08	0.82	0.86	0.87	0.92	1.10	1.17	1.14	1.22	67.23	9.07
F7BQE4	Rho guanine nucleotide exchange factor 10 (Fragment) OS=Mus musculus GN=Arhgef10 PE=2 SV=1 - [F7BQE4_MOUSE]	1.42	3	1	1	2	0.93	1.00	1.08	0.76	0.82	0.84	0.90	0.87	0.94	0.88	0.95	0.90	0.97	109.34	8.00
D3YVW9	Ankyrin repeat domain-containing protein 45 (Fragment) OS=Mus musculus GN=Ankrd45 PE=2 SV=1 - [D3YVW9_MOUSE]	5.37	3	1	1	2	0.97	0.98	1.01	0.95	0.98	0.83	0.85	0.87	0.90	0.86	0.89	1.03	1.07	22.81	4.79
G3X956	FACT complex subunit SPT16 OS=Mus musculus GN=Supt16 PE=4 SV=1 - [G3X956_MOUSE]	7.45	2	7	7	15	1.05	1.11	0.99	1.02	0.98	0.93	0.89	0.87	0.87	0.90	0.83	1.02	0.97	119.76	5.66
P31938	Dual specificity mitogen-activated protein kinase kinase 1 OS=Mus musculus GN=Map2k1 PE=1 SV=2 - [MP2K1_MOUSE]	48.60	1	10	15	105	1.01	0.89	0.92	1.05	1.08	0.91	0.93	0.87	0.88	0.91	0.94	0.93	0.96	43.45	6.70
P63101	14-3-3 protein zeta/delta OS=Mus musculus GN=Ywhaz PE=1 SV=1 - [1433Z_MOUSE]	85.31	7	19	28	1089	0.94	0.94	1.01	0.99	1.07	0.89	0.95	0.87	0.92	0.87	0.93	0.83	0.89	27.75	4.79
Q8C436	Protein-lysine methyltransferase METTL21D OS=Mus musculus GN=Mettl21d PE=2 SV=2 - [MT21D_MOUSE]	3.07	1	1	1	1	0.86	0.84	0.98	0.95	1.10	0.86	1.00	0.87	1.01	0.81	0.94	0.90	1.05	25.52	4.73
Q8BYC6	Serine/threonine-protein kinase TAO3 OS=Mus musculus GN=Taok3 PE=1 SV=2 - [TAOK3_MOUSE]	4.23	3	1	4	6	0.99	0.99	1.01	0.91	0.93	0.92	0.93	0.87	0.88	0.73	0.75	0.87	0.88	105.27	7.36
Q922H2	[Pyruvate dehydrogenase [lipoamide]] kinase isozyme 3, mitochondrial OS=Mus musculus GN=Pdk3 PE=2 SV=1 - [PDK3_MOUSE]	20.96	1	5	6	17	1.01	1.08	1.00	1.04	1.03	1.00	0.96	0.87	0.89	0.92	0.96	1.08	1.04	47.89	8.82

P47239	Paired box protein Pax-7 OS=Mus musculus GN=Pax7 PE=1 SV=2 - [PAX7_MOUSE]	2.58	2	1	1	1	1.19	1.01	0.84	0.82	0.69	1.00	0.83	0.87	0.73	1.30	1.09	0.90	0.75	54.90	8.98
Q80Y81-2	Isoform 2 of Zinc phosphodiesterase ELAC protein 2 OS=Mus musculus GN=Elac2 - [RNZ2_MOUSE]	3.03	4	2	2	3	1.04	1.28	1.24	1.09	1.05	0.83	0.80	0.87	0.84	0.88	0.85	0.94	0.90	92.02	7.43
D3Z2Q3	Diablo homolog, mitochondrial OS=Mus musculus GN=Diablo PE=2 SV=1 - [D3Z2Q3_MOUSE]	17.24	3	2	2	3	0.94	1.11	1.18	0.93	0.99	0.98	1.04	0.87	0.93	0.85	0.90	0.90	0.96	19.78	8.82
Q0VE82	Copine-7 OS=Mus musculus GN=Cpne7 PE=2 SV=1 - [CPNE7_MOUSE]	8.98	1	3	4	7	1.04	1.04	0.98	1.11	1.03	1.00	0.90	0.87	0.84	1.03	0.99	1.20	1.13	61.85	5.40
Q9D061	Acyl-CoA-binding domain-containing protein 6 OS=Mus musculus GN=Acbd6 PE=1 SV=2 - [ACBD6_MOUSE]	37.59	3	10	10	37	0.85	0.79	0.91	0.83	0.95	0.88	0.99	0.87	1.03	0.84	0.92	0.84	0.94	30.87	5.11
E9PUM9	Protein Nrxn2 OS=Mus musculus GN=Nrxn2 PE=2 SV=1 - [E9PUM9_MOUSE]	11.39	8	10	17	66	1.19	1.15	1.01	1.03	0.88	0.99	0.81	0.87	0.69	0.99	0.92	0.94	0.87	183.90	5.82
Q8BI08	Protein MAL2 OS=Mus musculus GN=Mal2 PE=1 SV=1 - [MAL2_MOUSE]	6.29	1	1	1	2	1.19	1.26	1.05	1.04	0.87	0.90	0.75	0.87	0.73	0.88	0.74	0.85	0.72	19.08	6.49
D3Z3S1	Prolactin regulatory element binding, isoform CRA_b OS=Mus musculus GN=Preb PE=4 SV=1 - [D3Z3S1_MOUSE]	16.57	3	4	4	28	0.94	1.10	1.07	1.13	1.07	0.88	0.92	0.87	0.88	0.98	1.01	0.98	1.06	37.94	8.56
Q4VAE3	Transmembrane protein 65 OS=Mus musculus GN=Tmem65 PE=2 SV=1 - [TMM65_MOUSE]	24.79	1	5	5	13	1.01	0.95	0.96	1.09	1.11	0.84	0.80	0.87	0.86	0.85	0.83	0.91	0.90	24.90	7.78
Q9R190	Metastasis-associated protein MTA2 OS=Mus musculus GN=Mta2 PE=1 SV=1 - [MTA2_MOUSE]	8.53	1	5	5	9	0.90	0.91	0.99	1.04	1.12	0.98	1.10	0.87	0.98	0.90	0.99	1.09	1.16	74.98	9.67
Q2PFD7-5	Isoform 5 of PH and SEC7 domain-containing protein 3 OS=Mus musculus GN=Psd3 - [PSD3_MOUSE]	29.08	6	7	27	108	0.99	0.94	0.94	1.04	0.98	0.98	0.92	0.87	0.89	0.93	0.94	0.90	0.94	111.14	6.25
E9Q7T7	Protein Chadl OS=Mus musculus GN=Chadl PE=2 SV=1 - [E9Q7T7_MOUSE]	5.35	1	2	2	4	0.89	1.22	1.37	1.05	1.19	0.99	1.11	0.87	0.98	1.12	1.26	1.11	1.26	81.31	8.87

Q61194-2	Isoform 2 of Phosphatidylinositol 4-phosphate 3-kinase C2 domain-containing subunit alpha OS=Mus musculus GN=Pik3c2a - [P3C2A_MOUSE]	0.48	3	1	1	1	1.03	0.99	0.96	0.94	0.91	0.99	0.96	0.87	0.85	0.96	0.93	0.92	0.90	187.41	8.24
Q3UVU3	Zinc transporter 10 OS=Mus musculus GN=Slc30a10 PE=1 SV=1 - [ZNT10_MOUSE]	6.17	2	2	2	3	0.93	1.08	1.16	0.91	0.98	0.84	0.89	0.87	0.94	0.97	1.05	1.06	1.14	50.88	7.02
B2RXC1	Trafficking protein particle complex subunit 11 OS=Mus musculus GN=Trappc11 PE=2 SV=1 - [TPC11_MOUSE]	7.06	1	5	5	7	0.93	1.03	0.95	1.03	1.04	0.90	0.95	0.87	0.93	0.91	0.83	0.89	0.92	128.31	7.58
Q9D1E6	Tubulin-folding cofactor B OS=Mus musculus GN=Tbcb PE=1 SV=2 - [TBCB_MOUSE]	49.18	1	9	9	24	0.95	0.97	1.04	1.04	1.07	0.97	1.03	0.87	0.90	0.88	0.98	0.82	0.89	27.37	5.24
Q60900	ELAV-like protein 3 OS=Mus musculus GN=Elavl3 PE=1 SV=1 - [ELAV3_MOUSE]	38.42	2	9	12	22	0.95	0.91	0.99	0.99	1.06	0.93	0.97	0.87	0.95	0.85	0.87	0.99	1.07	39.51	9.28
Q8VDS3	Chromobox protein homolog 7 OS=Mus musculus GN=Cbx7 PE=1 SV=1 - [CBX7_MOUSE]	5.06	3	1	1	1	1.14	0.84	0.74	0.90	0.79	0.83	0.73	0.87	0.77	0.95	0.83	0.94	0.83	18.10	5.20
Q3TDQ1	Dolichyl-diphosphooligosaccharide--protein glycosyltransferase subunit STT3B OS=Mus musculus GN=Stt3b PE=1 SV=2 - [STT3B_MOUSE]	3.65	1	2	2	3	0.87	1.25	1.43	1.10	1.25	0.80	0.91	0.87	1.00	1.03	1.18	0.96	1.10	93.19	8.95
F8WJG3	Transformer-2 protein homolog beta OS=Mus musculus GN=Tra2b PE=2 SV=1 - [F8WJG3_MOUSE]	40.43	2	5	6	47	0.99	0.81	0.83	0.97	0.94	0.87	0.84	0.87	0.85	0.83	0.86	0.87	0.88	21.92	10.15
Q8BH55	Threonine synthase-like 1 OS=Mus musculus GN=Thnsl1 PE=2 SV=1 - [THNS1_MOUSE]	16.47	2	9	9	19	1.00	1.07	1.03	1.11	1.08	0.92	0.90	0.87	0.85	0.94	0.96	0.99	0.95	83.05	7.20
Q3U487	E3 ubiquitin-protein ligase HECTD3 OS=Mus musculus GN=Hectd3 PE=1 SV=2 - [HECD3_MOUSE]	4.18	1	3	3	4	0.96	1.14	0.73	1.12	1.16	0.96	0.92	0.87	0.91	0.95	0.94	1.00	1.04	97.29	5.47
P08414	Calcium/calmodulin-dependent protein kinase type IV OS=Mus musculus GN=Camk4 PE=1 SV=2 - [KCC4_MOUSE]	32.20	3	9	9	16	0.95	0.82	0.94	1.03	1.18	0.94	1.06	0.87	0.95	0.94	1.01	0.89	0.95	52.59	4.93

P83940	Transcription elongation factor B polypeptide 1 OS=Mus musculus GN=Tceb1 PE=1 SV=1 - [ELOC_MOUSE]	48.21	1	4	4	15	0.92	1.00	1.19	1.00	1.07	0.87	1.00	0.88	0.88	0.89	0.93	0.97	1.03	12.46	4.78
Q9D8E6	60S ribosomal protein L4 OS=Mus musculus GN=Rpl4 PE=1 SV=3 - [RL4_MOUSE]	29.12	1	10	10	37	0.94	0.94	0.99	1.03	1.08	0.95	0.93	0.88	0.86	0.88	0.93	0.99	1.01	47.12	11.00
Q6ZQI3	Malectin OS=Mus musculus GN=Mlec PE=2 SV=2 - [MLEC_MOUSE]	6.19	2	2	2	7	1.03	0.84	0.78	1.12	1.05	0.81	0.81	0.88	0.77	0.92	0.77	0.81	0.88	32.32	6.05
P70211-3	Isoform B of Netrin receptor DCC OS=Mus musculus GN=Dcc - [DCC_MOUSE]	1.61	3	2	2	4	0.96	1.17	1.22	0.91	0.95	0.93	0.97	0.88	0.91	0.94	0.98	0.96	1.00	149.25	6.62
P59108	Copine-2 OS=Mus musculus GN=Cpne2 PE=2 SV=1 - [CPNE2_MOUSE]	6.93	1	2	3	6	1.08	1.18	1.08	0.94	0.86	0.85	0.78	0.88	0.80	0.92	0.85	1.05	0.97	61.00	5.96
Q8CIE6	Coatomer subunit alpha OS=Mus musculus GN=Copa PE=1 SV=2 - [COPA_MOUSE]	14.38	3	13	13	28	0.94	1.02	1.14	1.12	1.19	0.91	0.95	0.88	0.91	0.93	1.04	1.02	1.11	138.34	7.65
Q80YA7	Dipeptidyl peptidase 8 OS=Mus musculus GN=Dpp8 PE=1 SV=1 - [DPP8_MOUSE]	5.49	1	2	3	7	1.60	1.23	0.93	1.15	0.71	0.79	0.70	0.88	0.63	0.83	0.67	1.06	0.67	102.12	5.81
Q8R313	Exocyst complex component 6 OS=Mus musculus GN=Exoc6 PE=1 SV=2 - [EXOC6_MOUSE]	2.24	2	1	1	2	1.03	1.16	1.13	1.06	1.04	0.92	0.89	0.88	0.85	0.97	0.95	0.94	0.91	93.02	6.15
F8VQD7	Receptor-type tyrosine-protein phosphatase gamma OS=Mus musculus GN=Ptprg PE=2 SV=1 - [F8VQD7_MOUSE]	4.37	4	5	5	9	1.08	1.06	1.03	1.09	1.05	0.92	0.83	0.88	0.82	0.84	0.92	0.91	0.91	161.19	6.37
Q8BZB2	Phosphopantothenoylcysteine decarboxylase OS=Mus musculus GN=Ppcdc PE=2 SV=1 - [COAC_MOUSE]	38.24	1	5	5	10	1.07	1.07	1.00	0.92	0.88	0.91	0.86	0.88	0.81	0.91	0.84	0.79	0.81	22.33	6.52
O35375-5	Isoform B0 of Neuropilin-2 OS=Mus musculus GN=Nrp2 - [NRP2_MOUSE]	5.77	6	4	4	10	1.22	1.20	0.99	1.03	0.87	0.85	0.70	0.88	0.65	1.08	0.88	1.34	1.10	101.35	5.81
Q8C080	Sorting nexin-16 OS=Mus musculus GN=Snx16 PE=2 SV=2 - [SNX16_MOUSE]	22.38	2	6	6	15	0.98	0.90	1.01	0.92	0.98	0.83	0.89	0.88	0.89	0.85	0.89	0.91	0.96	38.79	5.21
P16390	Potassium voltage-gated channel subfamily A member 3 OS=Mus musculus GN=Kcna3 PE=2 SV=3 - [KCNA3_MOUSE]	14.02	3	2	6	40	0.89	1.06	1.19	1.00	1.07	0.84	0.94	0.88	0.94	1.04	1.13	0.93	0.99	58.53	5.33

Q80U40-2	Isoform 2 of RIMS-binding protein 2 OS=Mus musculus GN=Rimbp2 - [RIMB2_MOUSE]	26.01	3	20	20	85	0.88	1.09	1.14	0.92	1.04	0.87	0.99	0.88	0.95	0.83	0.95	0.82	0.93	117.85	5.27
Q8BH66	Atlastin-1 OS=Mus musculus GN=Atl1 PE=1 SV=1 - [ATLA1_MOUSE]	37.46	1	18	19	49	0.96	1.02	1.02	1.11	1.16	0.90	0.96	0.88	0.92	0.95	1.04	0.91	0.99	63.34	6.49
P46978	Dolichyl-diphosphooligosaccharide--protein glycosyltransferase subunit STT3A OS=Mus musculus GN=Stt3a PE=1 SV=1 - [STT3A_MOUSE]	2.27	1	2	2	3	1.07	1.13	1.05	1.26	1.18	0.87	0.81	0.88	0.82	0.79	0.74	1.09	1.02	80.54	8.10
E9Q9M5	Ubiquitin carboxyl-terminal hydrolase OS=Mus musculus GN=Usp19 PE=2 SV=1 - [E9Q9M5_MOUSE]	3.85	4	3	3	5	0.99	1.02	1.00	1.03	1.02	0.89	0.90	0.88	0.86	0.85	0.84	0.89	0.88	143.36	6.23
Q8BPN8-2	Isoform 2 of DmX-like protein 2 OS=Mus musculus GN=Dmxl2 - [DMXL2_MOUSE]	24.49	6	53	53	126	0.99	1.01	1.01	1.12	1.10	0.89	0.87	0.88	0.90	0.92	0.94	1.01	1.01	336.80	6.46
Q91V12-2	Isoform A of Cytosolic acyl coenzyme A thioester hydrolase OS=Mus musculus GN=Acot7 - [BACH_MOUSE]	44.97	5	13	13	50	1.04	0.99	0.92	1.06	1.00	0.92	0.85	0.88	0.88	0.93	0.90	1.00	0.99	37.53	7.52
Q9JI59	Junctional adhesion molecule B OS=Mus musculus GN=Jam2 PE=1 SV=1 - [JAM2_MOUSE]	10.40	2	3	3	4	0.95	0.97	1.21	1.07	1.13	0.93	1.19	0.88	0.96	0.97	0.97	0.99	0.96	33.03	8.40
Q3TYA6	M-phase phosphoprotein 8 OS=Mus musculus GN=Mphosph8 PE=1 SV=1 - [MPP8_MOUSE]	6.18	1	3	3	8	0.98	0.98	1.00	0.90	0.90	1.06	1.16	0.88	0.87	1.08	1.22	0.92	0.92	97.41	5.85
Q5SSH7-2	Isoform 2 of Zinc finger ZZ-type and EF-hand domain-containing protein 1 OS=Mus musculus GN=Zzef1 - [ZZEF1_MOUSE]	1.04	3	2	2	8	0.93	1.21	1.38	1.11	1.22	0.87	0.89	0.88	0.92	0.95	0.98	1.00	1.03	324.23	6.07
G3UZW7	Protein mago nashi homolog 2 OS=Mus musculus GN=Magohb PE=2 SV=1 - [G3UZW7_MOUSE]	26.47	4	2	2	2	0.91	0.66	0.73	0.82	0.90	0.76	0.83	0.88	0.96	0.79	0.87	0.85	0.93	11.79	5.52
Q9EP84	G protein-coupled receptor kinase 6 OS=Mus musculus GN=Grk6 PE=2 SV=1 - [Q9EP84_MOUSE]	1.61	3	1	1	5	0.89	1.03	1.15	1.09	1.21	0.98	1.10	0.88	0.98	0.99	1.13	0.99	1.11	64.24	8.53

Q9D154	Leukocyte elastase inhibitor A OS=Mus musculus GN=Serpinb1a PE=1 SV=1 - [ILEUA_MOUSE]	31.93	7	11	11	20	1.08	0.92	0.90	1.06	1.05	0.95	0.89	0.88	0.81	0.94	0.93	0.99	0.93	42.55	6.21
P41105	60S ribosomal protein L28 OS=Mus musculus GN=Rpl28 PE=1 SV=2 - [RL28_MOUSE]	20.44	1	4	4	7	1.02	0.90	0.91	1.02	1.01	0.94	0.90	0.88	0.86	0.98	0.91	1.05	0.97	15.72	12.02
Q91WT9-	Isoform 2 of Cystathionine beta-synthase OS=Mus musculus GN=Cbs - [CBS_MOUSE]	7.13	2	3	3	4	0.98	1.23	1.25	1.05	1.05	1.12	1.14	0.88	1.00	0.81	0.81	0.93	0.98	60.15	6.61
Q9D1N9	39S ribosomal protein L21, mitochondrial OS=Mus musculus GN=Mrpl21 PE=2 SV=1 - [RM21_MOUSE]	14.83	2	2	2	4	0.98	0.90	0.91	0.88	0.89	0.85	0.86	0.88	0.89	0.89	0.91	0.95	0.97	23.35	9.99
Q8BZZ3	NEDD4-like E3 ubiquitin-protein ligase WWP1 OS=Mus musculus GN=Wwp1 PE=1 SV=2 - [WWP1_MOUSE]	3.05	1	1	1	2	1.08	0.99	0.92	1.32	1.23	0.92	0.85	0.88	0.81	1.29	1.20	1.13	1.05	104.63	6.38
Q9WV91	Prostaglandin F2 receptor negative regulator OS=Mus musculus GN=Ptgfrn PE=1 SV=2 - [FPRP_MOUSE]	8.19	1	5	5	7	0.97	1.01	1.04	1.07	1.14	1.05	0.97	0.88	0.93	0.85	0.90	0.98	1.07	98.66	6.61
P50171	Estradiol 17-beta-dehydrogenase 8 OS=Mus musculus GN=Hsd17b8 PE=1 SV=2 - [DHB8_MOUSE]	15.83	3	3	3	8	0.97	1.13	1.13	1.01	1.02	0.85	0.89	0.88	0.87	0.96	1.01	1.03	1.04	26.57	6.54
Q9CQA3	Succinate dehydrogenase [ubiquinone] iron-sulfur subunit, mitochondrial OS=Mus musculus GN=Sdhb PE=1 SV=1 - [DHSB_MOUSE]	43.62	2	13	13	72	0.91	0.83	0.87	0.91	0.93	0.84	0.90	0.88	0.92	0.85	0.89	0.87	0.93	31.79	8.68
Q8BJU0-2	Isoform 2 of Small glutamine-rich tetratricopeptide repeat-containing protein alpha OS=Mus musculus GN=Sgta - [SGTA_MOUSE]	29.62	2	9	9	37	0.85	0.76	0.81	0.85	0.98	0.82	0.93	0.88	0.98	0.83	0.95	0.75	0.86	34.17	5.06
P97318-8	Isoform DAB553 of Disabled homolog 1 OS=Mus musculus GN=Dab1 - [DAB1_MOUSE]	6.69	9	3	3	6	0.97	1.29	1.30	0.94	0.98	0.81	0.81	0.88	0.89	0.75	0.80	0.86	0.88	59.38	5.36
D3YZ62	Unconventional myosin-Va OS=Mus musculus GN=Myo5a PE=2 SV=1 - [D3YZ62_MOUSE]	37.09	12	56	61	142	0.92	0.96	1.04	1.11	1.18	0.92	0.95	0.88	0.95	0.98	1.02	1.02	1.10	212.20	8.62
D3Z3R1	60S ribosomal protein L36 OS=Mus musculus GN=Gm5745 PE=3 SV=1 - [D3Z3R1_MOUSE]	30.77	5	4	4	8	1.00	0.92	0.91	1.15	1.24	1.18	1.17	0.88	1.01	1.13	1.20	1.01	0.99	12.12	11.36

P70168	Importin subunit beta-1 OS=Mus musculus GN=Kpnb1 PE=1 SV=2 - [IMB1_MOUSE]	23.06	1	15	15	30	0.97	1.04	1.10	1.11	1.08	0.93	0.90	0.88	0.88	0.97	1.03	1.00	1.04	97.12	4.78
P47757	F-actin-capping protein subunit beta OS=Mus musculus GN=Capzb PE=1 SV=3 - [CAPZB_MOUSE]	54.15	4	1	15	111	0.77	1.00	1.30	0.71	0.92	0.81	1.05	0.88	1.14	0.86	1.12	0.94	1.22	31.33	5.74
O54734	Dolichyl-diphosphooligosaccharide--protein glycosyltransferase 48 kDa subunit OS=Mus musculus GN=Ddost PE=1 SV=2 - [OST48_MOUSE]	22.90	1	6	6	22	1.00	1.14	1.20	1.11	1.04	0.86	0.86	0.88	0.86	0.88	0.89	1.00	1.01	49.00	5.83
Q9D7S7-2	Isoform 2 of 60S ribosomal protein L22-like 1 OS=Mus musculus GN=Rpl22l1 - [RL22L_MOUSE]	29.75	2	2	2	7	1.09	1.21	1.09	0.94	0.90	0.70	0.62	0.88	0.78	0.81	0.85	0.98	0.98	14.33	9.45
Q9D0F3	Protein ERGIC-53 OS=Mus musculus GN=Lman1 PE=2 SV=1 - [LMAN1_MOUSE]	12.19	1	4	4	6	1.05	1.31	1.33	0.98	0.91	0.88	0.83	0.88	0.84	0.86	0.82	0.76	0.72	57.75	6.34
F6VQ81	Tumor protein D54 (Fragment) OS=Mus musculus GN=Tpd52l2 PE=4 SV=1 - [F6VQ81_MOUSE]	69.57	1	1	11	61	1.06	0.97	0.91	0.69	0.66	1.03	0.97	0.88	0.83	0.88	0.83	0.73	0.69	17.72	8.19
Q9CQL4	39S ribosomal protein L20, mitochondrial OS=Mus musculus GN=Mrpl20 PE=1 SV=1 - [RM20_MOUSE]	8.72	1	1	1	1	5.20	27.70	5.32	1.04	0.20	1.40	0.27	0.88	0.17	0.95	0.18	0.93	0.18	17.58	11.11
Q9WTS2	Alpha-(1,6)-fucosyltransferase OS=Mus musculus GN=Fut8 PE=2 SV=2 - [FUT8_MOUSE]	1.39	1	1	1	1	1.02	1.17	1.15	1.20	1.18	0.87	0.85	0.88	0.86	0.97	0.95	0.93	0.91	66.52	7.52
Q64487-5	Isoform E of Receptor-type tyrosine-protein phosphatase delta OS=Mus musculus GN=Ptprd - [PTPRD_MOUSE]	34.01	18	2	43	146	1.05	1.15	1.09	1.00	0.89	0.88	0.83	0.88	0.83	0.76	0.74	0.75	0.72	213.75	6.60
Q8JZM0	Dimethyladenosine transferase 1, mitochondrial OS=Mus musculus GN=Tfb1m PE=2 SV=1 - [TFB1M_MOUSE]	2.61	1	1	1	2	1.01	1.46	1.44	1.10	1.09	0.84	0.83	0.88	0.86	0.97	0.95	1.09	1.08	38.94	9.47
F6WDS8	Regulator of G-protein-signaling 6 OS=Mus musculus GN=Rgs6 PE=4 SV=1 - [F6WDS8_MOUSE]	54.17	3	13	16	35	0.90	0.91	1.01	1.05	1.13	0.94	1.02	0.88	1.02	0.93	0.95	0.96	1.04	52.88	6.67
Q9CZC8	Secernin-1 OS=Mus musculus GN=Scrn1 PE=1 SV=1 - [SCRN1_MOUSE]	58.94	2	19	19	217	0.85	0.83	1.01	0.82	0.95	0.79	0.92	0.88	0.98	0.75	0.89	0.77	0.92	46.30	4.79

Q2TPA8	Hydroxysteroid dehydrogenase-like protein 2 OS=Mus musculus GN=Hsdl2 PE=2 SV=1 - [HSDL2_MOUSE]	13.27	2	4	4	12	0.98	0.80	0.86	1.04	1.12	0.96	1.00	0.88	0.87	0.95	1.00	0.97	1.02	54.17	6.74
Q91YQ5	Dolichyl-diphosphooligosaccharide--protein glycosyltransferase subunit 1 OS=Mus musculus GN=Rpn1 PE=2 SV=1 - [RPN1_MOUSE]	20.89	1	11	11	25	1.01	1.14	1.08	1.14	1.09	0.84	0.86	0.88	0.82	0.98	0.92	0.95	0.93	68.49	6.46
Q61508-2	Isoform Short of Extracellular matrix protein 1 OS=Mus musculus GN=Ecm1 - [ECM1_MOUSE]	3.23	3	1	1	2	0.74	0.93	1.25	0.84	1.14	0.95	1.28	0.88	1.18	1.05	1.41	1.06	1.44	48.33	6.07
Q3UUG6-	Isoform 2 of TBC1 domain family member 24 OS=Mus musculus GN=Tbc1d24 - [TBC24_MOUSE]	11.35	2	6	6	13	1.01	0.99	1.07	1.06	1.12	0.97	0.92	0.88	0.91	0.97	0.91	1.02	1.04	62.59	7.11
Q62383	Transcription elongation factor SPT6 OS=Mus musculus GN=Supt6h PE=1 SV=2 - [SPT6H_MOUSE]	0.52	1	1	1	2	0.93	1.31	1.40	0.93	1.00	0.92	0.98	0.88	0.94	0.96	1.03	1.10	1.18	198.96	4.93
Q9EQ08	Heparan N-sulfatase OS=Mus musculus GN=Sgsh PE=2 SV=1 - [Q9EQ08_MOUSE]	12.55	2	4	4	8	1.10	1.04	0.94	0.81	0.74	0.78	0.72	0.88	0.77	0.92	0.83	0.86	0.80	56.69	6.37
A6PWC3	Nardilysin OS=Mus musculus GN=Nrd1 PE=2 SV=1 - [A6PWC3_MOUSE]	10.92	4	9	9	24	1.08	1.16	1.07	0.97	0.97	0.97	0.94	0.88	0.92	0.91	0.89	0.92	0.92	127.69	4.87
D3YTT4	Isobutyryl-CoA dehydrogenase, mitochondrial OS=Mus musculus GN=Acad8 PE=2 SV=1 - [D3YTT4_MOUSE]	13.56	2	5	5	8	0.89	1.11	1.34	0.78	0.93	0.91	0.86	0.88	0.91	1.02	1.08	0.90	0.99	45.05	8.02
Q9QXK3-	Isoform 4 of Coatomer subunit gamma-2 OS=Mus musculus GN=Copg2 - [COPG2_MOUSE]	10.11	7	6	8	14	0.91	1.08	1.14	1.09	1.17	0.92	0.98	0.88	0.96	0.97	1.08	0.97	1.00	97.49	5.86
O35382	Exocyst complex component 4 OS=Mus musculus GN=Exoc4 PE=1 SV=2 - [EXOC4_MOUSE]	15.90	3	11	11	27	0.94	1.12	1.28	1.08	1.20	0.88	0.91	0.88	0.96	0.97	1.02	0.99	1.07	110.48	6.49
Q5SUR0	Phosphoribosylformylglycinamidine synthase OS=Mus musculus GN=Pfas PE=2 SV=1 - [PUR4_MOUSE]	12.49	3	8	8	19	1.08	1.00	1.05	1.03	0.99	0.99	0.97	0.88	0.89	0.99	1.03	1.07	0.97	144.54	5.67

Q3UVW2	Gamma-aminobutyric acid receptor subunit gamma-2 OS=Mus musculus GN=Gabrg2 PE=2 SV=1 - [Q3UVW2_MOUSE]	9.66	4	3	3	5	0.98	0.89	0.91	1.06	1.09	0.84	0.86	0.88	0.90	0.83	0.90	0.91	0.90	51.77	8.37
Q6ZPQ6-2	Isoform 2 of Membrane-associated phosphatidylinositol transfer protein 2 OS=Mus musculus GN=Pitpnm2 - [PITM2_MOUSE]	14.52	3	13	13	22	0.94	1.03	1.13	1.03	1.09	0.90	0.94	0.88	0.93	0.91	1.01	0.93	1.03	142.20	7.71
Q9Z204-2	Isoform C1 of Heterogeneous nuclear ribonucleoproteins C1/C2 OS=Mus musculus GN=Hnrnpc - [HNRPC_MOUSE]	44.00	3	1	10	45	0.88	0.94	1.02	0.85	0.96	0.88	1.05	0.88	1.08	0.93	1.15	0.92	1.07	33.04	5.03
P56399	Ubiquitin carboxyl-terminal hydrolase 5 OS=Mus musculus GN=Usp5 PE=1 SV=1 - [UBP5_MOUSE]	50.82	4	34	34	96	0.95	0.93	0.94	1.10	1.11	0.95	0.94	0.88	0.93	0.95	1.00	0.95	0.98	95.77	5.01
O35450	FK506-binding protein-like OS=Mus musculus GN=Fkbpl PE=2 SV=1 - [FKBPL_MOUSE]	2.88	1	1	1	1	0.98	0.87	0.89	1.04	1.05	0.85	0.86	0.88	0.89	0.93	0.95	0.90	0.91	38.28	7.74
E9Q7E1	Coiled-coil domain-containing protein 150 OS=Mus musculus GN=Ccdc150 PE=2 SV=1 - [E9Q7E1_MOUSE]	6.03	4	1	2	4	0.79	0.80	1.02	0.98	1.24	0.79	1.00	0.88	1.11	0.79	1.00	0.93	1.18	66.26	5.81
A2A4I8	Membrane primary amine oxidase (Fragment) OS=Mus musculus GN=Aoc3 PE=2 SV=1 - [A2A4I8_MOUSE]	2.55	3	1	1	2	0.93	1.15	1.24	0.88	0.95	0.78	0.84	0.88	0.95	0.92	0.99	0.74	0.80	52.82	5.63
B1B1D8	39S ribosomal protein L2, mitochondrial OS=Mus musculus GN=Mrpl2 PE=2 SV=1 - [B1B1D8_MOUSE]	13.49	2	2	2	4	0.91	1.06	1.17	0.82	0.90	0.90	0.99	0.88	0.97	0.79	0.87	0.96	1.06	33.08	11.06
Q9WVJ2	26S proteasome non-ATPase regulatory subunit 13 OS=Mus musculus GN=Psmd13 PE=1 SV=1 - [PSD13_MOUSE]	14.89	5	4	4	9	1.08	0.95	0.88	1.20	1.02	0.92	0.86	0.88	0.79	1.00	0.88	1.18	1.20	42.78	5.71
P59279	Ras-related protein Rab-2B OS=Mus musculus GN=Rab2b PE=2 SV=1 - [RAB2B_MOUSE]	53.70	4	4	11	60	0.96	1.03	0.82	1.17	1.09	0.90	0.97	0.88	0.92	0.93	0.89	0.93	0.97	24.18	6.68

Q6ZQK5-	Isoform 2 of Arf-GAP with coiled-coil, ANK repeat and PH domain-containing protein 2 OS=Mus musculus GN=Acap2 - [ACAP2_MOUSE]	9.57	2	6	7	10	0.99	0.95	1.02	1.00	1.02	0.87	0.92	0.88	0.92	0.87	0.92	0.90	0.94	85.08	6.73
E9Q912	Protein Rap1gds1 OS=Mus musculus GN=Rap1gds1 PE=2 SV=1 - [E9Q912_MOUSE]	42.50	2	20	20	59	0.97	0.93	0.96	1.09	1.15	0.90	0.93	0.88	0.91	0.94	1.03	1.01	1.04	66.03	5.35
Q91V41	Ras-related protein Rab 14 OS=Mus musculus GN=Rab14 PE=1 SV=3 - [RAB14_MOUSE]	71.16	12	12	14	111	1.01	1.00	0.98	1.13	1.11	0.90	0.87	0.88	0.85	0.97	0.92	0.92	0.93	23.88	6.21
Q7TSY6-4	Isoform 4 of CUGBP Elav-like family member 4 OS=Mus musculus GN=Celf4 - [CELF4_MOUSE]	9.87	7	2	4	7	0.92	0.87	0.95	0.84	0.92	0.86	0.93	0.88	0.95	0.95	1.04	1.03	1.12	48.52	8.78
E0CY63	Claudin domain-containing protein 1 (Fragment) OS=Mus musculus GN=Cldn25 PE=2 SV=1 - [E0CY63_MOUSE]	18.70	3	2	2	4	1.05	1.12	1.06	1.03	0.98	0.88	0.83	0.88	0.83	0.90	0.85	0.98	0.93	14.29	4.70
Q04899	Cyclin-dependent kinase 18 OS=Mus musculus GN=Cdk18 PE=1 SV=1 - [CDK18_MOUSE]	15.08	49	1	6	13	1.20	1.85	1.54	1.28	1.06	1.34	1.11	0.88	0.73	1.30	1.08	1.57	1.31	51.81	8.51
P28651	Carbonic anhydrase-related protein OS=Mus musculus GN=Ca8 PE=1 SV=5 - [CAH8_MOUSE]	8.59	1	1	1	2	1.00	2.46	2.45	1.21	1.20	1.36	1.35	0.88	0.87	1.07	1.07	1.10	1.10	33.06	4.78
Q8BVF7	Gamma-secretase subunit APH-1A OS=Mus musculus GN=Aph1a PE=2 SV=2 - [APH1A_MOUSE]	3.02	1	1	1	1	1.56	1.65	1.05	1.43	0.91	0.98	0.63	0.88	0.56	1.22	0.78	1.11	0.71	28.97	7.90
D3Z4N1	Rhotekin (Fragment) OS=Mus musculus GN=Rtkn PE=2 SV=1 - [D3Z4N1_MOUSE]	7.83	3	1	2	4	0.89	0.79	0.89	0.87	0.98	0.84	0.94	0.88	0.99	0.72	0.81	0.97	1.09	24.03	6.35
O35066	Kinesin-like protein KIF3C OS=Mus musculus GN=Kif3c PE=2 SV=3 - [KIF3C_MOUSE]	11.81	1	3	7	12	1.01	1.03	1.01	0.97	0.96	0.96	0.94	0.88	0.87	1.01	0.99	0.98	0.96	89.92	8.06
P60764	Ras-related C3 botulinum toxin substrate 3 OS=Mus musculus GN=Rac3 PE=1 SV=1 - [RAC3_MOUSE]	23.96	9	1	5	24	1.15	1.16	1.02	1.20	1.10	1.03	0.89	0.88	0.82	1.10	1.02	1.17	1.05	21.37	8.15

Q9DC61	Mitochondrial-processing peptidase subunit alpha OS=Mus musculus GN=Pmpca PE=1 SV=1 - [MPPA_MOUSE]	21.37	2	7	7	15	0.89	0.86	0.99	0.96	1.11	0.93	1.01	0.88	1.04	1.01	1.15	0.99	1.15	58.24	6.83
P35278	Ras-related protein Rab 5C OS=Mus musculus GN=Rab5c PE=1 SV=2 - [RAB5C_MOUSE]	48.15	3	4	7	53	0.99	1.10	1.09	0.98	0.99	0.85	0.80	0.88	0.80	0.87	0.83	0.82	0.77	23.40	8.41
Q8BHW2	Protein OSCP1 OS=Mus musculus GN=Oscp1 PE=2 SV=1 - [OSCP1_MOUSE]	14.78	1	6	6	11	1.11	0.96	1.15	1.00	1.01	0.93	0.83	0.88	0.86	0.98	0.92	1.05	1.12	43.20	7.20
Q8VHM5	Heterogeneous nuclear ribonucleoprotein R OS=Mus musculus GN=Hnrnpr PE=2 SV=1 - [Q8VHM5_MOUSE]	41.46	4	19	24	104	0.95	0.77	0.81	0.92	0.96	0.88	0.91	0.88	0.93	0.83	0.86	0.79	0.83	70.84	8.13
Q8CI94	Glycogen phosphorylase, brain form OS=Mus musculus GN=Pygb PE=1 SV=3 - [PYGB_MOUSE]	51.36	1	28	37	171	1.06	1.04	0.96	1.13	1.05	0.89	0.85	0.88	0.82	0.97	0.89	1.07	1.01	96.67	6.73
P20934	Protein EVI2A OS=Mus musculus GN=Evi2a PE=1 SV=2 - [EVI2A_MOUSE]	8.97	1	1	1	1	0.86	1.00	1.16	0.81	0.93	0.69	0.79	0.88	1.02	0.89	1.03	0.98	1.14	24.07	9.52
D3Z495	Protein spire homolog 1 OS=Mus musculus GN=Spire1 PE=2 SV=1 - [D3Z495_MOUSE]	11.46	8	4	5	8	1.05	0.76	0.72	1.09	1.03	0.88	0.91	0.88	0.94	0.97	0.96	1.02	1.07	59.35	9.52
Q7TMC8	L-fucose kinase OS=Mus musculus GN=Fuk PE=2 SV=1 - [Q7TMC8_MOUSE]	2.75	1	3	3	4	0.81	1.14	1.56	1.16	1.32	0.82	1.01	0.88	1.09	1.03	1.27	1.10	1.32	119.19	6.60
P70288	Histone deacetylase 2 OS=Mus musculus GN=Hdac2 PE=1 SV=1 - [HDAC2_MOUSE]	17.83	2	2	5	16	0.91	0.90	0.96	0.96	1.06	0.91	1.04	0.88	0.94	0.94	1.03	0.93	1.08	55.27	5.91
P55096	ATP-binding cassette sub-family D member 3 OS=Mus musculus GN=Abcd3 PE=1 SV=2 - [ABCD3_MOUSE]	9.26	1	4	4	9	0.86	1.13	1.21	1.25	1.36	0.88	0.87	0.88	0.87	0.99	1.02	1.10	1.10	75.43	9.26
Q80SY5	Pre-mRNA-splicing factor 38B OS=Mus musculus GN=Prpf38b PE=1 SV=1 - [PR38B_MOUSE]	2.77	1	1	2	5	1.08	1.01	0.93	1.07	0.98	1.06	0.97	0.88	0.81	1.01	0.93	1.01	0.93	63.71	10.54
Q9QUR6	Prolyl endopeptidase OS=Mus musculus GN=Prep PE=2 SV=1 - [PPCE_MOUSE]	28.59	1	15	15	39	0.95	1.01	1.05	1.00	1.07	0.91	0.94	0.88	0.93	0.92	0.92	1.03	1.10	80.70	5.73

Q8C561-2	Isoform 2 of LMBR1 domain-containing protein 2 OS=Mus musculus GN=Lmbrd2 - [LMBD2_MOUSE]	10.79	3	4	4	12	1.10	1.20	1.00	1.29	1.17	0.87	0.77	0.88	0.84	1.06	1.00	1.01	0.91	75.69	7.50
Q9CZ44	NSFL1 cofactor p47 OS=Mus musculus GN=Nsfl1c PE=1 SV=1 - [NSF1C_MOUSE]	62.43	4	21	21	183	1.01	0.94	0.92	0.91	0.91	0.83	0.83	0.88	0.88	0.82	0.83	0.85	0.83	40.69	5.15
Q61081	Hsp90 co-chaperone Cdc37 OS=Mus musculus GN=Cdc37 PE=2 SV=1 - [CDC37_MOUSE]	30.61	1	10	10	64	0.92	0.79	0.93	0.89	0.97	0.84	0.94	0.88	0.95	0.86	0.95	0.77	0.87	44.56	5.34
Q9WV98	Mitochondrial import inner membrane translocase subunit Tim9 OS=Mus musculus GN=Timm9 PE=1 SV=1 - [TIM9_MOUSE]	56.18	1	4	4	38	0.94	0.72	0.74	0.83	0.89	0.80	0.88	0.88	0.93	0.77	0.81	0.76	0.85	10.34	7.21
Q0KL02-3	Isoform 3 of Triple functional domain protein OS=Mus musculus GN=Trio - [TRIO_MOUSE]	9.34	4	13	18	29	0.95	0.96	0.92	1.03	1.04	0.94	1.00	0.88	0.96	0.93	1.00	0.92	0.96	288.53	6.35
Q8BQZ4	Ral GTPase-activating protein subunit beta OS=Mus musculus GN=Ralgapb PE=1 SV=2 - [RLGPB_MOUSE]	2.83	5	3	3	6	0.90	1.21	1.41	1.18	1.22	0.91	1.06	0.88	0.98	0.97	1.07	1.02	1.13	165.09	6.77
Q9CQD1	Ras-related protein Rab 5A OS=Mus musculus GN=Rab5a PE=1 SV=1 - [RAB5A_MOUSE]	48.37	2	5	7	60	1.05	0.99	0.95	1.01	0.98	0.86	0.80	0.88	0.81	0.85	0.83	0.94	0.85	23.58	8.15
P49817	Caveolin-1 OS=Mus musculus GN=Cav1 PE=1 SV=1 - [CAV1_MOUSE]	25.28	7	3	3	4	0.89	0.83	1.00	0.90	0.99	0.94	1.01	0.88	1.06	0.91	1.04	0.70	0.90	20.53	6.02
Q8R5C5	Beta-centractin OS=Mus musculus GN=Actr1b PE=1 SV=1 - [ACTY_MOUSE]	41.22	3	6	11	47	0.93	0.92	0.96	1.00	1.04	0.99	1.06	0.88	0.89	0.90	0.95	0.84	0.95	42.25	6.40
Q4FE56	Ubiquitin carboxyl-terminal hydrolase OS=Mus musculus GN=Usp9x PE=2 SV=1 - [Q4FE56_MOUSE]	12.65	5	24	24	41	0.98	1.02	1.02	1.10	1.13	0.90	0.92	0.88	0.95	1.01	1.01	1.05	1.07	290.03	5.83
E9Q3A7	Protein Cdh19 OS=Mus musculus GN=Cdh19 PE=3 SV=1 - [E9Q3A7_MOUSE]	1.95	1	1	1	2	0.91	1.93	2.12	1.09	1.20	1.11	1.21	0.88	0.96	1.01	1.10	1.14	1.25	87.21	4.70
P97441	Zinc transporter 3 OS=Mus musculus GN=Slc30a3 PE=1 SV=1 - [ZNT3_MOUSE]	16.49	1	7	7	24	1.12	1.18	1.11	1.16	1.04	0.87	0.79	0.88	0.78	0.98	0.82	0.90	0.86	41.80	6.38

Q9QZV9	NTF2-related export protein 1 OS=Mus musculus GN=Nxt1 PE=1 SV=2 - [NXT1_MOUSE]	5.71	3	1	1	2	0.93	1.23	1.32	0.94	1.00	0.83	0.88	0.88	0.94	0.84	0.90	0.88	0.94	15.84	5.03
Q9EPE9	Probable cation-transporting ATPase 13A1 OS=Mus musculus GN=Atp13a1 PE=1 SV=2 - [AT131_MOUSE]	2.00	1	2	2	3	1.12	1.02	0.91	1.21	1.08	0.80	0.71	0.88	0.79	0.94	0.85	0.93	0.83	132.30	8.03
O88307	Sortilin-related receptor OS=Mus musculus GN=Sorl1 PE=2 SV=3 - [SORL_MOUSE]	7.95	1	14	14	24	0.91	1.10	1.22	0.96	1.06	0.90	0.93	0.88	0.93	0.89	1.00	0.81	0.95	246.93	5.54
Q9WVB4	Slit homolog 3 protein OS=Mus musculus GN=Slit3 PE=2 SV=2 - [SLIT3_MOUSE]	2.76	1	3	3	6	0.97	1.13	1.11	1.01	0.96	0.95	0.96	0.88	0.91	0.95	0.91	0.99	0.99	167.62	7.62
P62071	Ras-related protein R-Ras2 OS=Mus musculus GN=Rras2 PE=1 SV=1 - [RRAS2_MOUSE]	36.76	1	4	6	18	0.93	1.16	1.37	1.01	1.09	0.94	0.99	0.88	0.99	0.95	1.05	0.88	1.05	23.38	6.01
Q91W50	Cold shock domain-containing protein E1 OS=Mus musculus GN=Csde1 PE=2 SV=1 - [CSDE1_MOUSE]	27.69	1	19	19	43	0.98	0.84	0.94	0.92	0.97	0.93	0.97	0.88	0.87	0.85	0.98	0.88	0.93	88.73	6.37
O55106	Striatin OS=Mus musculus GN=Strn PE=1 SV=2 - [STRN_MOUSE]	33.46	3	17	18	50	0.94	0.97	1.08	0.99	1.08	1.02	1.04	0.88	0.96	1.03	1.11	1.04	1.10	85.91	5.27
Q920I9-2	Isoform 2 of WD repeat containing protein 7 OS=Mus musculus GN=Wdr7 - [WDR7_MOUSE]	24.02	2	23	23	50	0.93	0.97	1.10	1.01	1.17	0.89	0.97	0.88	0.94	0.93	0.97	0.95	1.11	160.22	7.01
O08800	Serpin B8 OS=Mus musculus GN=Serpinb8 PE=2 SV=2 - [SPB8_MOUSE]	4.81	1	2	2	3	0.91	1.04	1.14	1.03	1.12	0.82	0.90	0.88	0.96	0.83	0.91	0.76	0.83	42.12	6.46
Q5NCB5	Immunity-related GTPase family M protein 1 (Fragment) OS=Mus musculus GN=Irgm1 PE=2 SV=1 - [Q5NCB5_MOUSE]	3.82	3	1	1	2	1.22	1.34	1.09	1.24	1.01	0.95	0.77	0.88	0.72	1.01	0.83	1.07	0.88	32.32	6.42
Q99JY8	Lipid phosphate phosphohydrolase 3 OS=Mus musculus GN=Ppap2b PE=1 SV=1 - [LPP3_MOUSE]	19.55	1	7	7	26	1.15	0.99	0.94	1.17	0.97	0.92	0.80	0.88	0.78	0.99	0.92	1.01	0.97	35.19	9.07
Q52KR3-4	Isoform 4 of Protein prune homolog 2 OS=Mus musculus GN=Prune2 - [PRUN2_MOUSE]	9.52	5	2	2	7	1.13	1.26	1.10	1.12	0.98	1.10	1.00	0.88	0.77	1.06	0.97	1.15	1.09	33.56	4.97

Q80TA6-2	Isoform 2 of Myotubularin-related protein 12 OS=Mus musculus GN=Mtmr12 - [MTMRC_MOUSE]	4.81	2	2	2	4	0.88	0.98	1.11	1.00	1.13	0.96	1.08	0.88	1.00	0.96	1.08	0.91	1.03	49.86	6.67
Q91XL9-2	Isoform 2 of Oxysterol-binding protein-related protein 1 OS=Mus musculus GN=Osbpl1a - [OSBL1_MOUSE]	21.74	6	7	8	28	0.88	0.90	0.98	1.05	1.13	0.92	1.05	0.88	0.97	0.91	1.01	0.80	0.95	50.04	6.80
G3UW81	MCG125472 OS=Mus musculus GN=Cyp4f40 PE=3 SV=1 - [G3UW81_MOUSE]	2.48	4	1	1	1	0.93	1.05	1.13	1.15	1.25	0.73	0.79	0.88	0.95	0.88	0.95	1.00	1.08	60.17	8.09
P24527	Leukotriene A-4 hydrolase OS=Mus musculus GN=Lta4h PE=1 SV=4 - [LKHA4_MOUSE]	28.64	1	14	14	39	1.10	1.11	0.98	1.11	1.05	0.95	0.91	0.88	0.87	0.99	0.94	1.04	0.95	69.01	6.42
Q91V35	Protein tyrosin phosphatase receptor type alpha OS=Mus musculus GN=Ptpra PE=2 SV=1 - [Q91V35_MOUSE]	18.54	3	11	12	17	1.10	1.15	1.06	1.04	0.97	0.92	0.94	0.88	0.81	0.91	0.84	1.03	1.05	89.79	6.64
Q62420	Endophilin-A1 OS=Mus musculus GN=Sh3gl2 PE=1 SV=2 - [SH3G2_MOUSE]	68.47	7	16	23	339	0.86	0.79	0.97	0.82	0.98	0.80	0.94	0.88	1.02	0.74	0.87	0.74	0.89	39.93	5.39
Q0VBD0	Integrin beta OS=Mus musculus GN=Itgb8 PE=2 SV=1 - [Q0VBD0_MOUSE]	12.78	1	7	8	14	1.01	0.96	0.93	1.10	1.06	0.91	0.90	0.88	0.84	0.96	0.93	0.96	0.94	84.46	7.09
P80318	T-complex protein 1 subunit gamma OS=Mus musculus GN=Cct3 PE=1 SV=1 - [TCPG_MOUSE]	47.34	5	21	21	62	1.03	1.03	0.99	1.07	1.05	0.91	0.90	0.88	0.90	1.02	1.02	1.06	1.03	60.59	6.70
P14869	60S acidic ribosomal protein P0 OS=Mus musculus GN=Rplp0 PE=1 SV=3 - [RLA0_MOUSE]	50.47	3	12	12	25	0.97	1.02	1.13	1.06	1.10	0.89	0.91	0.88	0.91	0.98	1.00	0.99	0.91	34.19	6.25
Q99LE6	ATP-binding cassette sub-family F member 2 OS=Mus musculus GN=Abcf2 PE=2 SV=1 - [ABCF2_MOUSE]	8.44	1	5	5	10	1.04	0.88	0.92	1.01	1.01	0.93	0.89	0.88	0.89	0.91	0.89	0.93	0.99	71.74	7.05
Q9DC16	Endoplasmic reticulum-Golgi intermediate compartment protein 1 OS=Mus musculus GN=Ergic1 PE=1 SV=1 - [ERGI1_MOUSE]	7.93	2	2	2	4	0.97	0.86	0.89	1.04	1.08	0.89	0.92	0.88	0.91	0.98	1.01	0.90	0.93	32.54	7.06
P48774	Glutathione S-transferase Mu 5 OS=Mus musculus GN=Gstm5 PE=1 SV=1 - [GSTM5_MOUSE]	47.77	6	11	12	42	1.07	1.05	0.96	1.12	1.02	0.90	0.79	0.88	0.81	0.93	0.91	1.08	0.99	26.62	7.21

Q9D8N0	Elongation factor 1-gamma OS=Mus musculus GN=Eef1g PE=1 SV=3 - [EF1G_MOUSE]	45.08	1	16	16	60	0.97	0.98	0.95	1.00	1.05	0.92	0.95	0.88	0.89	0.90	0.90	0.95	1.00	50.03	6.74
Q6GYP7-5	Isoform 5 of Ral GTPase-activating protein subunit alpha-1 OS=Mus musculus GN=Ralgapa1 - [RGPA1_MOUSE]	2.89	4	3	3	4	0.85	0.86	0.94	1.00	1.11	0.96	1.13	0.89	1.04	0.93	1.07	0.85	1.01	117.10	5.91
O88441	Metaxin-2 OS=Mus musculus GN=Mtx2 PE=1 SV=1 - [MTX2_MOUSE]	17.11	1	4	4	7	0.91	0.92	0.99	0.98	1.02	0.87	0.94	0.89	0.89	0.95	0.93	1.13	1.14	29.74	5.63
Q9CZN8	Glutamyl-tRNA(Gln) amidotransferase subunit A, mitochondrial OS=Mus musculus GN=Qrsl1 PE=2 SV=1 - [GATA_MOUSE]	7.24	1	3	3	5	0.94	0.85	0.94	0.99	1.05	0.92	0.90	0.89	0.93	1.04	0.98	1.18	1.10	56.75	5.95
O55143	Sarcoplasmic/endoplasmic reticulum calcium ATPase 2 OS=Mus musculus GN=Atp2a2 PE=1 SV=2 - [AT2A2_MOUSE]	34.20	9	26	33	117	0.94	1.01	1.08	1.18	1.22	0.83	0.91	0.89	0.95	0.91	1.00	0.98	1.00	114.78	5.34
E9PZI9	OX-2 membrane glycoprotein OS=Mus musculus GN=Cd200 PE=2 SV=1 - [E9PZI9_MOUSE]	19.38	5	3	3	14	1.12	0.87	0.75	0.99	0.90	0.96	0.87	0.89	0.84	1.00	0.91	1.00	0.90	14.30	7.81
E9QKE4	Rab3 GTPase-activating protein non-catalytic subunit OS=Mus musculus GN=Rab3gap2 PE=2 SV=1 - [E9QKE4_MOUSE]	7.86	3	10	10	18	1.00	1.06	1.01	1.09	1.06	0.92	0.92	0.89	0.83	0.91	0.93	1.01	1.02	154.54	6.07
Q920R0	Alsin OS=Mus musculus GN=Als2 PE=1 SV=3 - [ALS2_MOUSE]	3.27	2	3	3	5	0.95	1.18	1.24	1.12	1.19	0.84	0.95	0.89	0.89	1.01	1.07	1.14	1.21	182.45	6.30
Q8K0G5	Protein TSSC1 OS=Mus musculus GN=Tssc1 PE=1 SV=2 - [TSSC1_MOUSE]	15.28	1	5	5	9	1.02	1.01	0.99	1.10	0.98	0.94	0.84	0.89	0.90	0.98	1.01	1.08	0.90	43.10	5.14
P06745	Glucose-6-phosphate isomerase OS=Mus musculus GN=Gpi PE=1 SV=4 - [G6PI_MOUSE]	59.86	1	28	28	239	1.02	0.92	0.93	1.10	1.08	0.93	0.91	0.89	0.89	0.95	0.93	1.00	0.99	62.73	8.13
Q9Z0V2	Potassium voltage-gated channel subfamily D member 2 OS=Mus musculus GN=Kcnd2 PE=1 SV=1 - [KCND2_MOUSE]	19.21	1	9	9	28	1.00	1.19	1.20	1.17	1.16	0.91	0.93	0.89	0.88	0.98	0.95	1.05	1.05	70.53	8.07
O35684	Neuroserpin OS=Mus musculus GN=Serpini1 PE=1 SV=1 - [NEUS_MOUSE]	11.71	2	4	4	6	0.97	0.88	0.91	0.97	0.95	0.92	0.84	0.89	0.82	0.78	0.87	0.75	0.71	46.32	4.72

P62908	40S ribosomal protein S3 OS=Mus musculus GN=Rps3 PE=1 SV=1 - [RS3_MOUSE]	57.20	2	12	12	39	0.95	0.93	0.85	1.04	1.11	0.91	0.91	0.89	0.90	0.97	0.95	0.94	0.96	26.66	9.66
G3UW90	Trichorhinophalangeal syndrome I (Human), isoform CRA_b OS=Mus musculus GN=Trps1 PE=4 SV=1 - [G3UW90_MOUSE]	0.94	2	1	1	1	0.94	0.93	0.99	0.94	1.00	1.00	1.06	0.89	0.94	0.90	0.96	0.89	0.95	140.85	7.64
Q8C0M2	Abhydrolase domain-containing protein 4 OS=Mus musculus GN=Abhd4 PE=2 SV=1 - [Q8C0M2_MOUSE]	3.77	3	1	1	2	0.92	1.03	1.12	0.97	1.06	0.86	0.93	0.89	0.96	0.97	1.06	0.98	1.07	36.03	8.27
Q69ZS6	Synaptic vesicle glycoprotein 2C OS=Mus musculus GN=Sv2c PE=1 SV=2 - [SV2C_MOUSE]	5.23	1	3	4	15	1.28	1.28	1.00	1.29	0.92	1.10	0.85	0.89	0.69	1.17	0.92	1.39	1.05	82.24	5.11
Q8BUN9	Protein Slc24a2 OS=Mus musculus GN=Slc24a2 PE=2 SV=1 - [Q8BUN9_MOUSE]	11.11	3	3	7	21	0.85	0.78	0.89	0.94	1.11	0.85	1.13	0.89	1.14	0.81	1.00	0.77	0.85	74.19	6.54
Q80XG9-2	Isoform 2 of Leucine-rich repeat transmembrane neuronal protein 4 OS=Mus musculus GN=Lrrtm4 - [LRRT4_MOUSE]	5.21	3	3	3	5	1.02	1.08	1.13	1.13	1.14	0.95	0.97	0.89	0.82	0.88	0.84	1.02	1.07	59.00	9.28
Q9CTY5	Calcium uptake protein 3, mitochondrial OS=Mus musculus GN=Micu3 PE=2 SV=2 - [MICU3_MOUSE]	12.05	2	5	5	23	1.08	1.18	1.18	1.05	1.10	1.04	0.99	0.89	0.91	0.87	0.81	1.05	1.06	59.77	7.15
P68040	Guanine nucleotide-binding protein subunit beta-2-like 1 OS=Mus musculus GN=Gnb2l1 PE=1 SV=3 - [GBLP_MOUSE]	62.46	1	14	14	33	0.96	0.92	0.92	1.06	1.07	0.84	0.87	0.89	0.88	0.87	0.92	0.96	1.04	35.05	7.69
E9PYJ8	Histone acetyltransferase p300 OS=Mus musculus GN=Ep300 PE=4 SV=1 - [E9PYJ8_MOUSE]	1.41	2	1	3	4	1.07	0.99	0.93	0.91	0.85	0.94	0.87	0.89	0.83	0.93	0.87	1.07	1.00	263.14	8.54
Q8BFR5	Elongation factor Tu, mitochondrial OS=Mus musculus GN=Tufm PE=1 SV=1 - [EFTU_MOUSE]	59.29	3	21	21	134	0.92	0.81	0.84	1.00	1.06	0.87	0.94	0.89	0.93	0.85	0.91	0.84	0.89	49.48	7.56
Q80WQ2	Protein VAC14 homolog OS=Mus musculus GN=Vac14 PE=1 SV=1 - [VAC14_MOUSE]	6.27	1	4	4	7	1.12	1.01	1.06	1.14	1.11	0.89	0.86	0.89	0.83	0.98	0.96	1.07	1.02	87.99	6.13

B0F2B4	Neuroligin 4-like OS=Mus musculus GN=Nlgn4l PE=1 SV=1 - [NLGN4_MOUSE]	8.57	1	4	6	14	0.99	1.14	1.21	1.12	1.15	1.06	1.06	0.89	0.95	0.93	0.99	0.83	0.89	97.29	6.32
Q8BU33	Acetolactate synthase-like protein OS=Mus musculus GN=Ilvbl PE=2 SV=1 - [ILVBL_MOUSE]	2.22	1	1	1	6	0.97	1.19	1.23	1.22	1.25	0.92	0.96	0.89	0.92	1.07	1.10	1.01	1.04	68.11	8.69
Q08331	Calretinin OS=Mus musculus GN=Calb2 PE=1 SV=3 - [CALB2_MOUSE]	60.15	1	16	17	120	1.72	0.95	0.59	0.98	0.56	0.99	0.56	0.89	0.51	1.15	0.69	1.37	0.81	31.35	5.02
Q8BHN3	Neutral alpha-glucosidase AB OS=Mus musculus GN=Ganab PE=1 SV=1 - [GANAB_MOUSE]	29.34	3	21	21	41	0.98	1.09	1.10	1.06	1.08	0.94	0.94	0.89	0.92	0.99	1.01	1.06	1.06	106.84	6.06
Q9JK81	UPF0160 protein MYG1, mitochondrial OS=Mus musculus GN=Myg1 PE=2 SV=1 - [MYG1_MOUSE]	8.42	3	3	3	5	1.07	1.07	1.06	1.01	1.00	1.00	0.99	0.89	0.88	1.09	1.09	1.01	0.95	42.70	7.02
Q8K0S0	Phytanoyl-CoA hydroxylase-interacting protein OS=Mus musculus GN=Phyhip PE=1 SV=1 - [PHYIP_MOUSE]	36.36	2	9	12	34	0.97	1.02	0.97	1.09	1.03	0.95	0.95	0.89	0.83	0.89	0.87	0.92	0.91	37.53	7.01
Q9Z188-2	Isoform 2 of Dual specificity tyrosine-phosphorylation-regulated kinase 1B OS=Mus musculus GN=Dyrk1b - [DYR1B_MOUSE]	2.89	6	2	2	3	1.14	1.05	0.91	1.00	0.87	0.98	0.85	0.89	0.77	0.95	0.83	1.15	1.01	64.87	9.03
F8WJD6	RNA-binding protein MEX3B OS=Mus musculus GN=Mex3b PE=4 SV=1 - [F8WJD6_MOUSE]	2.95	3	1	1	1	0.93	1.10	1.18	1.06	1.14	1.22	1.30	0.89	0.95	0.87	0.94	1.13	1.22	59.12	6.90
Q9CS42	Ribose-phosphate pyrophosphokinase 2 OS=Mus musculus GN=Prps2 PE=1 SV=4 - [PRPS2_MOUSE]	26.10	1	3	6	13	0.96	1.00	1.04	1.06	1.09	1.00	0.98	0.89	1.00	1.05	1.14	1.07	1.12	34.76	6.61
Q99KP6	Pre-mRNA-processing factor 19 OS=Mus musculus GN=Prpf19 PE=2 SV=1 - [PRP19_MOUSE]	22.22	4	8	8	21	1.00	0.95	1.06	1.06	0.98	1.00	1.01	0.89	0.90	1.01	0.98	1.00	1.00	55.20	6.61
D3YXA8	Solute carrier organic anion transporter family member 3A1 OS=Mus musculus GN=Slco3a1 PE=2 SV=1 - [D3YXA8_MOUSE]	2.66	3	1	1	1	0.75	1.38	1.83	0.81	1.08	0.87	1.16	0.89	1.18	0.83	1.10	0.81	1.08	64.33	7.43

Q6ZPE2	Myotubularin-related protein 5 OS=Mus musculus GN=Sbf1 PE=1 SV=2 - [MTMR5_MOUSE]	23.51	6	29	29	61	0.95	1.08	1.15	1.05	1.05	0.85	0.93	0.89	0.95	0.91	0.97	1.01	1.06	208.56	7.12
Q921Q7	Ras and Rab interactor 1 OS=Mus musculus GN=Rin1 PE=1 SV=1 - [RIN1_MOUSE]	15.47	1	9	9	17	0.97	1.09	1.12	1.07	1.12	0.94	0.93	0.89	0.90	1.02	1.04	1.11	1.13	82.96	8.72
Q99KZ6	Zinc finger protein 639 OS=Mus musculus GN=Znf639 PE=2 SV=1 - [ZN639_MOUSE]	1.24	1	1	1	1	1.04	0.76	0.73	0.93	0.89	1.08	1.03	0.89	0.85	0.81	0.78	0.70	0.67	55.64	5.83
P40124	Adenylyl cyclase-associated protein 1 OS=Mus musculus GN=Cap1 PE=1 SV=4 - [CAP1_MOUSE]	41.35	2	14	14	49	0.98	0.96	1.01	1.11	1.09	0.95	0.96	0.89	0.91	0.95	0.98	1.00	1.06	51.53	7.52
D3Z7R0	[3-methyl-2-oxobutanoate dehydrogenase [lipoamide]] kinase, mitochondrial (Fragment) OS=Mus musculus GN=Bckdk PE=2 SV=1 - [D3Z7R0_MOUSE]	5.14	2	1	1	2	0.85	1.09	1.29	1.13	1.34	0.94	1.11	0.89	1.05	0.90	1.06	0.91	1.08	24.34	9.51
Q80WG5	Leucine-rich repeat-containing protein 8A OS=Mus musculus GN=Lrrc8a PE=1 SV=1 - [LRC8A_MOUSE]	8.89	1	5	5	9	1.06	1.15	1.11	1.16	1.08	0.93	0.82	0.89	0.86	0.93	0.88	1.10	0.98	94.06	7.94
Q791T5-2	Isoform 2 of Mitochondrial carrier homolog 1 OS=Mus musculus GN=Mtch1 - [MTCH1_MOUSE]	16.13	3	6	6	18	1.08	1.11	1.07	1.11	1.00	0.79	0.71	0.89	0.81	0.93	0.83	1.05	0.98	39.91	9.48
F7C528	Protein Chd3 (Fragment) OS=Mus musculus GN=Chd3 PE=4 SV=1 - [F7C528_MOUSE]	1.17	3	2	2	3	1.03	0.85	0.82	0.99	0.96	1.04	1.00	0.89	0.86	0.96	0.93	0.93	0.93	213.90	7.53
B1AWN6	Protein Scn2a1 OS=Mus musculus GN=Scn2a1 PE=3 SV=1 - [B1AWN6_MOUSE]	13.91	12	19	25	74	1.08	1.05	1.00	1.11	1.03	0.89	0.84	0.89	0.81	0.97	0.95	0.96	0.89	227.80	5.67
P54923	[Protein ADP-ribosylarginine] hydrolase OS=Mus musculus GN=Adprh PE=2 SV=1 - [ADPRH_MOUSE]	35.36	1	8	8	16	0.97	1.08	1.12	1.11	1.12	0.92	0.94	0.89	0.91	1.00	1.04	1.03	0.99	40.04	5.76
Q9DBE8	Alpha-1,3/1,6-mannosyltransferase ALG2 OS=Mus musculus GN=Alg2 PE=2 SV=2 - [ALG2_MOUSE]	23.86	2	7	7	12	1.10	1.10	0.99	1.08	0.79	0.92	0.73	0.89	0.73	0.96	0.76	1.04	0.98	47.37	7.97

Q8C0A6	MAP7 domain-containing protein 2 (Fragment) OS=Mus musculus GN=Map7d2 PE=2 SV=1 - [Q8C0A6_MOUSE]	37.84	1	1	4	10	1.06	0.71	0.67	0.89	0.84	1.12	1.26	0.89	0.98	0.97	0.96	0.84	0.80	7.80	10.17
Q80VE5	Protein Tbc1d22b OS=Mus musculus GN=Tbc1d22b PE=2 SV=1 - [Q80VE5_MOUSE]	4.55	1	1	2	3	1.01	1.12	1.11	0.94	0.92	0.89	0.88	0.89	0.87	1.00	0.99	1.07	1.05	59.09	7.43
Q8VD75	Huntingtin-interacting protein 1 OS=Mus musculus GN=Hip1 PE=1 SV=2 - [HIP1_MOUSE]	10.11	1	8	9	21	0.99	0.93	0.94	1.12	1.14	1.02	1.00	0.89	0.91	1.00	1.01	0.95	1.04	115.13	5.43
Q8BJY1	26S proteasome non-ATPase regulatory subunit 5 OS=Mus musculus GN=Psmd5 PE=1 SV=4 - [PSMD5_MOUSE]	14.29	2	5	5	10	0.94	1.33	1.27	1.02	1.11	0.87	0.90	0.89	0.88	0.92	0.96	1.00	1.06	55.94	5.21
Q9JJL8	Serine--tRNA ligase, mitochondrial OS=Mus musculus GN=Sars2 PE=2 SV=2 - [SYSM_MOUSE]	12.55	1	4	4	6	0.96	1.50	1.70	0.98	1.05	0.98	1.05	0.89	0.95	0.90	0.96	1.00	1.00	58.28	7.90
Q99JZ4	GTP-binding protein SAR1a OS=Mus musculus GN=Sar1a PE=2 SV=1 - [Q99JZ4_MOUSE]	31.82	2	3	4	7	1.07	0.78	0.76	0.94	0.96	1.03	0.99	0.89	0.91	1.09	0.88	1.11	1.04	22.38	6.68
Q8VDG5	Phosphopantothenate--cysteine ligase OS=Mus musculus GN=Ppcs PE=2 SV=1 - [PPCS_MOUSE]	8.36	2	2	2	4	1.04	0.96	0.91	1.01	0.97	0.88	0.84	0.89	0.85	0.95	0.91	0.97	0.93	33.77	6.55
Q9ERE7	LDLR chaperone MESD OS=Mus musculus GN=Mesdc2 PE=1 SV=1 - [MESD_MOUSE]	48.21	3	11	11	75	1.21	0.87	0.71	1.00	0.81	0.87	0.75	0.89	0.77	0.79	0.67	0.87	0.74	25.19	6.34
Q8R3C6-2	Isoform 2 of Probable RNA-binding protein 19 OS=Mus musculus GN=Rbm19 - [RBM19_MOUSE]	1.73	2	1	1	1	1.00	0.72	0.72	0.82	0.82	0.95	0.95	0.89	0.89	0.95	0.96	0.86	0.86	57.90	5.55
Q69ZP3	Probable hydrolase PNKD OS=Mus musculus GN=Pnkd PE=2 SV=2 - [PNKD_MOUSE]	6.75	3	1	2	3	0.99	1.22	1.24	1.01	1.02	0.94	0.95	0.89	0.90	0.91	0.92	0.94	0.95	42.99	8.94
Q62426	Cystatin-B OS=Mus musculus GN=Cstb PE=1 SV=1 - [CYTB_MOUSE]	37.76	1	3	3	24	0.95	0.70	0.67	0.91	0.90	0.96	0.97	0.89	0.92	0.77	0.78	0.83	0.86	11.04	7.39
Q6QI06	Rapamycin-insensitive companion of mTOR OS=Mus musculus GN=Rictor PE=1 SV=2 - [RICTR_MOUSE]	0.82	1	1	1	3	0.98	1.36	1.39	1.17	1.19	1.05	1.07	0.89	0.90	0.94	0.96	1.13	1.16	191.45	7.23

F6SEU4	Ras/Rap GTPase-activating protein SynGAP OS=Mus musculus GN=Syngap1 PE=3 SV=2 - [SYGP1_MOUSE]	36.57	4	1	36	151	0.91	1.16	1.26	0.96	1.08	0.97	1.06	0.89	1.00	0.92	1.00	1.03	1.13	148.14	8.98
Q91YM2	Rho GTPase-activating protein 35 OS=Mus musculus GN=Arhgap35 PE=1 SV=3 - [RHG35_MOUSE]	16.68	1	18	18	31	0.98	1.01	1.10	1.04	1.04	0.90	0.89	0.89	0.92	0.97	0.98	1.02	1.10	170.29	6.61
Q99PV0	Pre-mRNA-processing-splicing factor 8 OS=Mus musculus GN=Prpf8 PE=1 SV=2 - [PRP8_MOUSE]	5.52	2	11	11	23	0.98	1.05	1.00	1.06	1.10	0.92	1.04	0.89	0.97	1.01	1.03	1.00	1.00	273.44	8.84
P08752	Guanine nucleotide-binding protein G(i) subunit alpha-2 OS=Mus musculus GN=Gnai2 PE=1 SV=5 - [GNAI2_MOUSE]	56.34	6	10	17	130	1.08	1.12	1.09	1.06	1.04	0.91	0.84	0.89	0.86	1.00	0.97	1.05	1.05	40.46	5.45
P57759	Endoplasmic reticulum resident protein 29 OS=Mus musculus GN=Erp29 PE=1 SV=2 - [ERP29_MOUSE]	45.80	4	10	10	38	1.03	0.85	0.81	0.85	0.85	0.82	0.80	0.89	0.92	0.77	0.80	0.83	0.86	28.81	6.15
Q60612	Adenosine receptor A1 OS=Mus musculus GN=Adora1 PE=2 SV=4 - [AA1R_MOUSE]	5.52	1	1	1	1	0.75	1.03	1.37	1.05	1.41	0.84	1.12	0.89	1.19	1.13	1.51	0.61	0.81	36.61	8.48
G3X928	SEC23-interacting protein OS=Mus musculus GN=Sec23ip PE=4 SV=1 - [G3X928_MOUSE]	10.52	2	9	9	16	1.03	0.94	0.86	1.03	1.00	0.93	0.92	0.89	0.92	0.96	0.95	1.05	0.97	110.69	5.72
Q9ESN4	Complement C1q-like protein 3 OS=Mus musculus GN=C1ql3 PE=1 SV=1 - [C1QL3_MOUSE]	28.24	2	4	4	16	0.93	0.83	0.88	1.01	1.08	0.87	0.93	0.89	0.89	0.92	0.96	0.92	0.93	26.67	6.79
F8WHL1	Zinc transporter 9 OS=Mus musculus GN=Slc30a9 PE=2 SV=1 - [F8WHL1_MOUSE]	8.78	3	4	4	6	1.03	1.23	1.38	1.29	1.20	0.84	0.76	0.89	0.96	1.00	1.00	0.93	0.92	60.64	8.70
Q8BWG8	Beta-arrestin-1 OS=Mus musculus GN=Arrb1 PE=1 SV=1 - [ARRB1_MOUSE]	52.39	9	14	15	39	1.03	1.02	0.95	0.96	0.93	0.94	0.90	0.89	0.88	0.97	0.89	0.90	0.88	46.94	6.28
Q80UP5	Ankyrin repeat domain-containing protein 13A OS=Mus musculus GN=Ankrd13a PE=1 SV=2 - [AN13A_MOUSE]	6.80	2	1	2	3	0.82	1.22	1.48	0.76	0.92	0.99	1.20	0.89	1.07	0.77	0.93	0.95	1.15	67.14	5.08
Q8R2K1-5	Isoform 5 of Fucose mutarotase OS=Mus musculus GN=Fuom - [FUCM_MOUSE]	41.13	8	3	3	6	0.66	0.84	1.05	0.90	1.12	0.80	1.06	0.89	1.28	0.86	1.19	0.71	1.02	13.71	5.10

Q60854	Serpin B6 OS=Mus musculus GN=Serpinb6 PE=2 SV=1 - [SPB6_MOUSE]	19.31	10	6	6	10	1.02	0.88	0.90	1.04	1.06	0.88	0.90	0.89	0.95	0.94	0.99	1.03	1.03	42.57	5.74
Q8QZV4	Serine/threonine-protein kinase 32C OS=Mus musculus GN=Stk32c PE=2 SV=1 - [ST32C_MOUSE]	17.01	2	5	5	10	0.95	0.92	1.01	1.04	1.06	0.89	1.02	0.89	0.90	0.97	1.05	0.94	0.96	55.23	6.16
P80314	T-complex protein 1 subunit beta OS=Mus musculus GN=Cct2 PE=1 SV=4 - [TCPB_MOUSE]	47.10	1	19	19	79	0.99	0.92	0.90	1.09	1.07	0.95	0.93	0.89	0.88	0.97	0.96	1.10	1.10	57.44	6.40
Q8BVW0	Neutral alpha-glucosidase C OS=Mus musculus GN=Ganc PE=2 SV=2 - [GANC_MOUSE]	1.34	2	1	1	1	0.86	1.19	1.38	0.92	1.07	0.79	0.92	0.89	1.03	1.10	1.28	1.39	1.63	101.94	6.32
Q9Z0E0	Neurochondrin OS=Mus musculus GN=Ncdn PE=1 SV=1 - [NCDN_MOUSE]	37.45	2	18	18	85	1.03	1.17	1.12	1.18	1.14	0.96	0.87	0.89	0.87	1.01	0.94	1.19	1.13	78.84	5.54
Q68ED2	Metabotropic glutamate receptor 7 OS=Mus musculus GN=Grm7 PE=1 SV=1 - [GRM7_MOUSE]	9.07	5	6	6	10	1.12	1.03	0.88	1.14	0.99	0.94	0.84	0.89	0.80	1.04	0.88	0.98	0.82	102.15	7.88
Q9D6J6	NADH dehydrogenase [ubiquinone] flavoprotein 2, mitochondrial OS=Mus musculus GN=Ndufv2 PE=1 SV=2 - [NDUV2_MOUSE]	61.29	5	13	14	218	0.90	0.82	0.93	0.89	0.96	0.84	0.92	0.89	0.96	0.80	0.86	0.84	0.89	27.27	7.40
A2AAN2	Signal recognition particle subunit SRP68 OS=Mus musculus GN=Srp68 PE=2 SV=1 - [A2AAN2_MOUSE]	10.39	2	4	4	7	0.93	1.10	1.22	1.16	1.32	0.95	1.22	0.89	0.95	1.06	1.05	0.99	1.14	65.91	7.27
P47941	Crk-like protein OS=Mus musculus GN=Crkl PE=1 SV=2 - [CRKL_MOUSE]	67.33	1	17	17	57	1.02	0.91	0.91	0.87	0.91	0.87	0.83	0.89	0.87	0.81	0.82	0.85	0.85	33.81	6.74
P54754	Ephrin type-B receptor 3 OS=Mus musculus GN=Ephb3 PE=1 SV=2 - [EPHB3_MOUSE]	11.28	5	6	9	16	0.95	1.13	1.15	1.10	1.13	0.93	1.01	0.89	0.92	0.90	1.01	0.89	0.99	109.59	6.28
Q8VDP4	DBIRD complex subunit KIAA1967 homolog OS=Mus musculus PE=1 SV=2 - [K1967_MOUSE]	24.84	1	16	16	27	0.93	0.92	0.94	0.95	1.11	1.00	1.02	0.89	1.10	1.01	0.99	1.03	1.05	102.94	5.25
P02802	Metallothionein-1 OS=Mus musculus GN=Mt1 PE=1 SV=1 - [MT1_MOUSE]	32.79	1	2	2	7	1.20	0.86	0.70	0.97	0.80	1.02	0.85	0.89	0.79	0.88	0.70	0.89	0.75	6.01	7.96
A2AGS2	Dual-specificity mitogen-activated protein kinase kinase 6 OS=Mus musculus GN=Map2k6 PE=2 SV=1 - [A2AGS2_MOUSE]	20.48	4	5	5	10	0.96	0.85	0.99	1.09	1.14	0.84	0.91	0.89	0.95	0.89	1.00	0.99	1.08	32.75	6.92

Q8BGT8	Phytanoyl-CoA hydroxylase-interacting protein-like OS=Mus musculus GN=Phyhipl PE=2 SV=1 - [PHIPL_MOUSE]	45.33	5	12	15	51	1.04	0.93	0.95	1.01	0.97	0.91	0.88	0.89	0.80	0.87	0.89	0.88	0.90	42.31	6.35
E9QAF8	Protein Ccnt2 OS=Mus musculus GN=Ccnt2 PE=2 SV=1 - [E9QAF8_MOUSE]	2.59	2	1	1	2	1.04	0.90	0.86	0.96	0.92	1.00	0.95	0.89	0.85	0.91	0.88	0.88	0.85	72.89	9.20
Q3UMY5-	Isoform 4 of Echinoderm microtubule-associated protein-like 4 OS=Mus musculus GN=Eml4 - [EMAL4_MOUSE]	11.30	5	8	8	12	0.93	0.74	0.77	0.95	1.00	0.95	0.96	0.89	0.97	0.82	0.89	0.91	0.97	97.50	6.01
O08848	60 kDa SS-A/Ro ribonucleoprotein OS=Mus musculus GN=Trove2 PE=1 SV=1 - [RO60_MOUSE]	14.87	1	6	6	13	1.05	1.04	0.99	1.06	1.02	0.91	0.91	0.89	0.85	0.91	0.87	0.93	0.92	60.09	7.90
F7CBI4	Arf-GAP with Rho-GAP domain, ANK repeat and PH domain-containing protein 2 (Fragment) OS=Mus musculus GN=Arap2 PE=4 SV=1 - [F7CBI4_MOUSE]	5.94	3	1	1	2	0.93	0.91	0.97	0.95	1.01	0.78	0.84	0.89	0.95	0.87	0.94	0.91	0.97	23.00	4.91
Q8BWW3	Phosphoacetylglucosamine mutase OS=Mus musculus GN=Pgm3 PE=2 SV=1 - [Q8BWW3_MOUSE]	9.18	2	4	4	6	1.09	1.00	0.94	1.23	1.09	0.88	0.76	0.89	0.83	0.95	0.88	0.98	0.98	54.95	6.29
P61205	ADP-ribosylation factor 3 OS=Mus musculus GN=Arf3 PE=2 SV=2 - [ARF3_MOUSE]	58.56	6	6	11	93	1.03	1.07	1.03	1.06	1.03	0.96	0.90	0.89	0.83	0.94	0.90	0.97	0.99	20.59	7.43
Q8BXA1	Golgi integral membrane protein 4 OS=Mus musculus GN=Golim4 PE=1 SV=1 - [GOLI4_MOUSE]	2.60	2	1	1	2	0.93	1.03	1.11	0.80	0.86	0.81	0.87	0.89	0.96	0.73	0.79	0.89	0.96	76.74	4.83
E9QLB2	Lysophospholipase-like protein 1 OS=Mus musculus GN=Lyplal1 PE=4 SV=1 - [E9QLB2_MOUSE]	9.62	2	2	2	6	1.14	0.94	0.82	1.03	0.90	0.89	0.77	0.89	0.78	0.98	0.86	1.01	0.89	26.32	7.88
Q920E5	Farnesyl pyrophosphate synthase OS=Mus musculus GN=Fdps PE=2 SV=1 - [FPPS_MOUSE]	21.81	1	8	8	14	1.09	0.96	0.97	1.10	0.99	0.88	0.84	0.89	0.86	0.95	0.91	1.07	0.93	40.56	5.66
F6RTE1	Serine/arginine-rich-splicing factor 9 (Fragment) OS=Mus musculus GN=Srsf9 PE=2 SV=1 - [F6RTE1_MOUSE]	19.62	1	1	3	5	1.20	0.87	0.72	0.80	0.67	1.04	0.86	0.89	0.74	0.89	0.75	0.67	0.56	17.73	6.54

Q8K0C4	Lanosterol 14-alpha demethylase OS=Mus musculus GN=Cyp51a1 PE=2 SV=1 - [CP51A_MOUSE]	4.37	1	2	2	6	1.02	1.27	1.24	1.02	1.05	0.91	0.99	0.89	0.87	0.92	0.90	0.92	1.00	56.74	8.41
Q8R1J9-2	Isoform 2 of Torsin-2A OS=Mus musculus GN=Tor2a - [TOR2A_MOUSE]	10.06	3	1	1	2	0.94	0.97	1.03	0.96	1.02	1.12	1.19	0.89	0.94	1.03	1.10	1.10	1.18	18.08	5.29
P45878	Peptidyl-prolyl cis-trans isomerase FKBP2 OS=Mus musculus GN=Fkbp2 PE=1 SV=1 - [FKBP2_MOUSE]	68.57	1	9	9	50	1.02	0.76	0.72	0.92	0.88	0.87	0.85	0.89	0.88	0.81	0.76	0.73	0.72	15.33	8.88
Q99MN1	Lysine--tRNA ligase OS=Mus musculus GN=Kars PE=1 SV=1 - [SYK_MOUSE]	19.16	2	9	9	22	1.00	0.93	0.92	1.01	1.03	0.93	0.92	0.89	0.87	0.93	0.92	0.97	0.95	67.80	5.94
P0CG14	Chromosome transmission fidelity protein 8 homolog isoform 2 OS=Mus musculus GN=Chtf8 PE=2 SV=1 - [CTF8A_MOUSE]	7.32	1	2	2	2	0.98	1.10	1.12	0.94	0.96	0.77	0.78	0.89	0.91	0.88	0.90	0.66	0.67	52.21	12.32
O88630	Golgi SNAP receptor complex member 1 OS=Mus musculus GN=Gosr1 PE=1 SV=2 - [GOSR1_MOUSE]	8.80	1	2	2	3	0.98	0.98	1.00	0.96	0.98	1.03	1.04	0.89	0.91	0.87	0.89	0.84	0.86	28.47	9.29
A2A8L5	Receptor-type tyrosine-protein phosphatase F OS=Mus musculus GN=Ptprf PE=1 SV=1 - [PTPRF_MOUSE]	10.59	3	8	12	25	1.08	1.18	1.16	0.98	0.96	0.90	0.86	0.89	0.82	0.89	0.84	0.96	0.90	211.36	6.65
Q91YP2	Neurolysin, mitochondrial OS=Mus musculus GN=Nln PE=2 SV=1 - [NEUL_MOUSE]	5.26	1	3	3	5	1.04	1.09	1.00	0.96	1.25	0.80	0.97	0.89	1.05	0.88	0.87	0.93	1.08	80.38	6.44
Q3UN00	P2X purinoceptor OS=Mus musculus GN=P2rx7 PE=2 SV=1 - [Q3UN00_MOUSE]	2.55	3	1	1	2	1.00	1.14	1.14	0.92	0.92	0.93	0.92	0.89	0.89	0.89	0.89	1.16	1.16	49.34	8.06
Q00612	Glucose-6-phosphate 1-dehydrogenase X OS=Mus musculus GN=G6pdx PE=1 SV=3 - [G6PD1_MOUSE]	30.49	4	13	13	31	0.95	0.87	0.97	1.04	1.05	0.91	0.91	0.89	0.88	0.93	0.94	0.92	1.01	59.22	6.49
Q6P5D3-2	Isoform 2 of Putative ATP-dependent RNA helicase DHX57 OS=Mus musculus GN=Dhx57 - [DHX57_MOUSE]	2.02	4	2	2	2	1.08	1.14	1.05	0.95	0.88	0.93	0.86	0.89	0.82	0.96	0.89	1.35	1.25	149.78	7.75
Q0V8T9	Contactin-associated protein like 5-1 OS=Mus musculus GN=Cntnap5a PE=2 SV=1 - [CTP5A_MOUSE]	8.28	5	7	7	11	1.13	1.17	1.02	1.02	0.85	0.99	0.85	0.89	0.78	1.03	0.89	1.03	0.90	145.62	6.28

Q9ERA6	Tuftelin-interacting protein 11 OS=Mus musculus GN=Tfip11 PE=1 SV=1 - [TFP11_MOUSE]	10.98	3	4	5	10	0.90	0.92	1.11	0.97	1.08	0.82	0.95	0.89	0.98	0.91	1.08	0.90	0.99	96.24	5.90
G5E8J3	Bromodomain and WD repeat domain containing 2, isoform CRA_a OS=Mus musculus GN=Wdr11 PE=4 SV=1 - [G5E8J3_MOUSE]	2.53	2	3	3	4	0.97	1.18	1.22	1.09	1.13	0.92	0.95	0.89	0.92	1.01	1.04	1.18	1.22	135.88	7.11
Q3TT92	Dihydropyrimidinase-related protein 3 OS=Mus musculus GN=Dpysl3 PE=2 SV=1 - [Q3TT92_MOUSE]	65.32	5	20	25	449	0.94	0.87	0.97	0.90	0.98	0.85	0.91	0.89	0.86	0.86	0.91	0.82	0.91	61.74	6.49
P42932	T-complex protein 1 subunit theta OS=Mus musculus GN=Cct8 PE=1 SV=3 - [TCPQ_MOUSE]	44.16	6	22	22	62	1.00	0.97	0.95	1.08	1.06	0.94	0.91	0.89	0.89	1.01	0.99	1.00	0.99	59.52	5.62
Q08879-2	Isoform C of Fibulin-1 OS=Mus musculus GN=Fbln1 - [FBLN1_MOUSE]	1.46	2	1	1	2	1.04	1.15	1.11	0.93	0.90	0.87	0.83	0.89	0.85	1.00	0.97	1.16	1.12	75.23	5.27
Q91VU6-[illegible]	Isoform 2 of DDB1- and CUL4-associated factor 11 OS=Mus musculus GN=Dcaf11 - [DCA11_MOUSE]	6.93	5	3	3	5	0.93	1.22	1.30	1.01	1.09	0.94	0.97	0.89	1.09	0.98	1.05	1.03	1.24	57.42	7.20
Q8BG40	Katanin p80 WD40 repeat-containing subunit B1 OS=Mus musculus GN=Katnb1 PE=1 SV=1 - [KTNB1_MOUSE]	18.69	1	8	8	16	0.94	1.08	1.14	1.07	1.07	0.96	1.03	0.89	0.93	0.96	1.00	1.03	1.02	72.59	7.27
B7ZMP1	Probable Xaa-Pro aminopeptidase 3 OS=Mus musculus GN=Xpnpep3 PE=2 SV=1 - [XPP3_MOUSE]	4.15	1	1	1	1	1.24	1.19	0.96	0.79	0.64	1.07	0.86	0.89	0.72	0.91	0.73	1.35	1.09	56.64	7.68
P50136	2-oxoisovalerate dehydrogenase subunit alpha, mitochondrial OS=Mus musculus GN=Bckdha PE=1 SV=1 - [ODBA_MOUSE]	16.97	2	5	5	9	0.97	0.90	1.03	1.01	1.08	0.94	0.96	0.89	1.02	1.02	1.08	1.07	1.10	50.34	8.06
Q8VHQ9	Acyl-coenzyme A thioesterase 11 OS=Mus musculus GN=Acot11 PE=2 SV=1 - [ACO11_MOUSE]	18.86	6	8	8	23	1.03	0.99	0.94	1.12	1.11	0.94	0.89	0.89	0.90	0.98	0.89	0.92	0.92	67.31	6.80
P14069	Protein S100-A6 OS=Mus musculus GN=S100a6 PE=1 SV=3 - [S10A6_MOUSE]	8.99	1	1	1	3	0.91	0.94	1.03	0.81	0.89	0.87	0.95	0.89	0.98	0.92	1.01	1.04	1.15	10.04	5.48

Q9Z2I9	Succinyl-CoA ligase [ADP-forming] subunit beta, mitochondrial OS=Mus musculus GN=Sucla2 PE=1 SV=2 - [SUCB1_MOUSE]	42.98	1	21	21	81	1.03	0.93	0.87	1.03	1.05	0.93	0.94	0.89	0.88	0.95	0.90	0.99	0.98	50.08	7.01
Q3U0M1-	Isoform 4 of Trafficking protein particle complex subunit 9 OS=Mus musculus GN=Trappc9 - [TPPC9_MOUSE]	6.90	7	4	4	7	1.02	1.07	1.22	1.09	1.08	0.81	0.83	0.89	0.85	0.94	0.97	1.09	1.07	78.25	7.74
Q8BMI0	F-box only protein 38 OS=Mus musculus GN=Fbxo38 PE=1 SV=1 - [FBX38_MOUSE]	0.75	1	1	1	1	1.05	0.89	0.85	1.05	1.00	1.00	0.95	0.89	0.85	0.92	0.88	0.95	0.91	133.84	6.09
Q00493	Carboxypeptidase E OS=Mus musculus GN=Cpe PE=1 SV=2 - [CBPE_MOUSE]	27.31	1	9	9	22	1.06	0.96	0.89	0.97	0.95	1.05	0.94	0.89	0.82	1.03	0.97	1.07	1.01	53.22	5.19
Q8R1F6-2	Isoform 2 of Protein HID1 OS=Mus musculus GN=Hid1 - [HID1_MOUSE]	2.54	2	2	2	3	0.88	1.03	1.18	1.02	1.16	0.92	1.04	0.89	1.02	1.02	1.17	0.99	1.13	88.60	5.94
Q80TZ3	Putative tyrosine-protein phosphatase auxilin OS=Mus musculus GN=Dnajc6 PE=2 SV=2 - [AUXI_MOUSE]	38.49	5	22	24	85	0.97	0.84	0.94	0.99	1.07	0.87	0.90	0.89	0.90	0.91	0.99	0.84	0.87	102.23	7.23
Q9DC53	Copine-8 OS=Mus musculus GN=Cpne8 PE=2 SV=2 - [CPNE8_MOUSE]	22.70	3	3	10	20	1.06	0.90	0.95	1.23	1.07	0.94	0.92	0.89	0.84	0.71	0.69	0.80	0.77	63.08	5.96
P59017	Bcl-2-like protein 13 OS=Mus musculus GN=Bcl2l13 PE=1 SV=2 - [B2L13_MOUSE]	13.82	1	4	4	6	0.82	0.84	1.18	0.74	1.04	0.94	1.03	0.89	1.04	0.79	0.93	0.83	0.97	46.69	4.59
Q8BJL0	SWI/SNF-related matrix-associated actin-dependent regulator of chromatin subfamily A-like protein 1 OS=Mus musculus GN=Smarcal1 PE=2 SV=1 - [SMAL1_MOUSE]	2.97	1	1	1	2	0.81	0.84	1.03	0.89	1.09	0.91	1.11	0.89	1.09	0.82	1.01	0.83	1.02	100.78	8.90
Q3B7Z2	Oxysterol-binding protein 1 OS=Mus musculus GN=Osbp PE=1 SV=3 - [OSBP1_MOUSE]	28.70	2	14	16	45	1.03	0.93	0.95	1.07	0.97	1.00	0.98	0.89	0.88	0.99	0.94	1.01	0.93	88.74	7.20
Q8CHW4	Translation initiation factor eIF-2B subunit epsilon OS=Mus musculus GN=Eif2b5 PE=1 SV=1 - [EI2BE_MOUSE]	5.44	1	3	3	7	0.95	1.23	1.16	1.06	1.10	0.91	0.98	0.89	0.87	0.97	1.07	0.99	0.95	80.04	5.07
Q8BFT9	Synaptic vesicle 2-related protein OS=Mus musculus GN=Svop PE=1 SV=1 - [SVOP_MOUSE]	13.50	2	5	5	23	1.04	1.35	1.49	1.08	1.07	0.85	0.78	0.89	0.90	0.96	0.92	1.06	0.93	60.73	5.85

P61967	AP-1 complex subunit sigma-1A OS=Mus musculus GN=Ap1s1 PE=1 SV=1 - [AP1S1_MOUSE]	21.52	7	2	3	6	0.91	1.07	1.18	1.00	1.17	0.73	0.88	0.89	0.98	1.04	1.14	0.83	1.01	18.72	5.73
P56382	ATP synthase subunit epsilon, mitochondrial OS=Mus musculus GN=Atp5e PE=2 SV=2 - [ATP5E_MOUSE]	44.23	1	3	3	13	0.98	1.02	1.07	0.97	0.99	0.86	0.90	0.89	0.89	0.87	0.87	0.94	1.01	5.83	10.01
Q8K2Y7	39S ribosomal protein L47, mitochondrial OS=Mus musculus GN=Mrpl47 PE=2 SV=2 - [RM47_MOUSE]	3.97	1	1	1	1	0.90	0.86	0.96	0.94	1.05	0.84	0.93	0.89	0.99	0.95	1.05	0.83	0.92	29.71	10.21
Q9CVB6	Actin-related protein 2/3 complex subunit 2 OS=Mus musculus GN=Arpc2 PE=1 SV=3 - [ARPC2_MOUSE]	62.67	2	14	14	42	1.10	0.87	0.79	1.04	1.01	0.94	0.88	0.89	0.88	0.90	0.86	1.01	0.98	34.34	7.36
Q8BVE3	V-type proton ATPase subunit H OS=Mus musculus GN=Atp6v1h PE=1 SV=1 - [VATH_MOUSE]	42.24	1	18	18	78	1.06	0.97	0.93	1.14	1.01	0.97	0.91	0.89	0.88	0.94	0.95	0.98	0.90	55.82	6.61
Q91ZA3	Propionyl-CoA carboxylase alpha chain, mitochondrial OS=Mus musculus GN=Pcca PE=2 SV=2 - [PCCA_MOUSE]	36.74	4	20	20	47	0.99	1.07	0.99	1.06	1.07	0.89	0.85	0.89	0.89	0.97	0.95	0.94	0.96	79.87	7.25
P16330	2',3'-cyclic-nucleotide 3'-phosphodiesterase OS=Mus musculus GN=Cnp PE=1 SV=3 - [CN37_MOUSE]	80.95	6	37	38	406	0.93	0.92	0.99	1.04	1.11	0.93	0.98	0.89	0.96	0.90	0.96	0.92	1.01	47.09	8.97
Q8K1S4-2	Isoform 2 of Netrin receptor UNC5A OS=Mus musculus GN=Unc5a - [UNC5A_MOUSE]	6.89	2	4	4	12	1.06	1.17	1.12	1.11	1.06	0.90	0.85	0.89	0.89	0.96	0.96	0.98	0.91	92.87	6.71
Q9Z0L0	Trophoblast glycoprotein OS=Mus musculus GN=Tpbg PE=1 SV=3 - [TPBG_MOUSE]	13.85	1	4	4	7	0.80	1.39	1.74	0.92	1.14	0.83	1.07	0.89	1.15	0.86	1.03	0.94	1.24	46.42	6.83
Q9Z2B2-2	Isoform 2 of Brain mitochondrial carrier protein 1 OS=Mus musculus GN=Slc25a14 - [UCP5_MOUSE]	3.42	2	1	1	2	1.09	1.21	1.10	1.08	0.99	0.88	0.80	0.89	0.81	1.00	0.91	1.02	0.93	36.02	9.69
Q9Z1N5	Spliceosome RNA helicase Ddx39b OS=Mus musculus GN=Ddx39b PE=1 SV=1 - [DX39B_MOUSE]	27.10	5	10	10	37	1.00	0.89	0.79	1.05	1.00	0.94	0.96	0.89	0.88	0.96	0.95	0.96	1.01	49.00	5.67
Q9JIA1	Leucine-rich glioma-inactivated protein 1 OS=Mus musculus GN=Lgi1 PE=1 SV=1 - [LGI1_MOUSE]	34.65	2	17	17	59	0.95	0.88	0.95	1.07	1.08	0.91	0.90	0.89	0.94	0.88	0.90	0.90	0.99	63.60	8.02

P00493	Hypoxanthine-guanine phosphoribosyltransferase OS=Mus musculus GN=Hprt1 PE=1 SV=3 - [HPRT_MOUSE]	76.15	1	12	12	60	1.18	0.96	0.75	0.95	0.81	1.02	0.83	0.89	0.76	0.99	0.83	0.99	0.79	24.55	6.68
Q9EPU4	Cleavage and polyadenylation specificity factor subunit 1 OS=Mus musculus GN=Cpsf1 PE=1 SV=1 - [CPSF1_MOUSE]	5.34	1	7	7	14	1.08	1.09	1.04	1.05	0.93	0.90	0.76	0.89	0.84	0.91	0.85	1.02	0.98	160.72	6.39
P70459	ETS domain-containing transcription factor ERF OS=Mus musculus GN=Erf PE=2 SV=1 - [ERF_MOUSE]	4.36	1	2	2	3	0.97	1.16	1.19	1.07	1.10	0.95	0.97	0.89	0.92	0.86	0.89	1.01	1.04	59.01	7.28
Q8K3G9	DCC-interacting protein 13-beta OS=Mus musculus GN=Appl2 PE=2 SV=1 - [DP13B_MOUSE]	5.14	2	2	3	5	0.96	1.05	1.09	0.94	0.98	1.00	1.04	0.89	0.93	1.02	1.07	0.95	0.99	73.81	5.03
Q9CQ86	Migration and invasion enhancer 1 OS=Mus musculus GN=Mien1 PE=1 SV=1 - [MIEN1_MOUSE]	41.74	1	5	5	24	1.16	0.91	0.86	0.90	0.80	1.01	0.86	0.89	0.80	0.85	0.74	0.88	0.78	12.29	4.51
P07901	Heat shock protein HSP 90-alpha OS=Mus musculus GN=Hsp90aa1 PE=1 SV=4 - [HS90A_MOUSE]	64.53	4	31	45	610	1.03	0.89	0.87	1.07	1.02	0.92	0.88	0.89	0.87	0.92	0.90	0.94	0.90	84.73	5.01
Q5SS00	DBF4-type zinc finger-containing protein 2 homolog OS=Mus musculus GN=Zdbf2 PE=2 SV=1 - [ZDBF2_MOUSE]	1.76	1	2	2	6	1.30	1.16	0.88	0.97	0.91	1.05	0.87	0.89	0.81	1.23	0.94	1.19	0.93	273.58	5.22
Q9DBG9	Tax1-binding protein 3 OS=Mus musculus GN=Tax1bp3 PE=1 SV=1 - [TX1B3_MOUSE]	28.23	1	2	2	5	0.94	1.37	1.46	0.94	1.00	0.95	1.01	0.89	0.95	0.97	1.04	0.79	0.85	13.71	8.48
Q91WD5	NADH dehydrogenase [ubiquinone] iron-sulfur protein 2, mitochondrial OS=Mus musculus GN=Ndufs2 PE=1 SV=1 - [NDUS2_MOUSE]	49.68	3	17	17	84	0.95	1.02	1.07	1.07	1.14	0.90	0.90	0.89	0.91	0.95	1.00	1.02	1.11	52.59	6.99
Q3TPX4	Exocyst complex component 5 OS=Mus musculus GN=Exoc5 PE=1 SV=2 - [EXOC5_MOUSE]	6.92	3	4	4	6	1.01	1.13	1.27	1.25	1.24	0.95	1.02	0.89	0.88	1.00	0.99	1.13	1.18	81.69	6.71
P62631	Elongation factor 1-alpha 2 OS=Mus musculus GN=Eef1a2 PE=1 SV=1 - [EF1A2_MOUSE]	60.69	1	12	21	196	0.99	0.96	0.99	0.98	1.01	0.92	0.95	0.89	0.93	0.93	0.94	0.92	0.96	50.42	9.03
Q99L27	GMP reductase 2 OS=Mus musculus GN=Gmpr2 PE=2 SV=2 - [GMPR2_MOUSE]	6.61	1	1	2	3	0.95	1.88	1.98	1.00	1.05	0.69	0.73	0.89	0.94	0.87	0.92	0.88	0.93	37.99	7.44

P29416	Beta-hexosaminidase subunit alpha OS=Mus musculus GN=Hexa PE=2 SV=2 - [HEXA_MOUSE]	11.74	1	5	5	9	0.96	1.20	1.30	1.14	1.12	1.04	0.92	0.89	0.76	0.95	0.92	1.10	1.01	60.57	6.54
P28571-1	Isoform GlyT-1A of Sodium- and chloride-dependent glycine transporter 1 OS=Mus musculus GN=Slc6a9 - [SC6A9_MOUSE]	10.58	8	5	5	21	1.11	1.14	0.98	1.11	1.00	0.93	0.83	0.89	0.81	1.00	0.92	1.01	0.92	70.51	7.91
Q9QUJ7-2	Isoform Short of Long-chain-fatty-acid--CoA ligase 4 OS=Mus musculus GN=Acsl4 - [ACSL4_MOUSE]	8.81	2	4	4	31	0.87	1.26	1.36	1.15	1.23	0.99	1.06	0.89	1.06	1.06	1.21	1.03	1.13	74.29	8.00
Q80U63	Mitofusin-2 OS=Mus musculus GN=Mfn2 PE=1 SV=3 - [MFN2_MOUSE]	20.48	4	11	11	19	1.05	1.12	1.07	1.10	1.05	0.82	0.79	0.89	0.84	0.91	0.82	0.96	0.88	86.13	6.77
A2AN08-3	Isoform 3 of E3 ubiquitin-protein ligase UBR4 OS=Mus musculus GN=Ubr4 - [UBR4_MOUSE]	3.24	5	14	14	21	0.98	1.08	1.06	1.11	1.12	0.91	0.92	0.89	0.93	0.94	0.96	1.08	1.11	569.94	6.06
Q8BYM5	Neuroligin-3 OS=Mus musculus GN=Nlgn3 PE=1 SV=2 - [NLGN3_MOUSE]	12.73	4	5	8	23	1.02	1.03	1.13	1.07	1.05	0.91	0.87	0.89	0.95	0.95	0.96	0.89	0.94	91.10	5.80
Q3TCJ1	BRISC complex subunit Abro1 OS=Mus musculus GN=Fam175b PE=2 SV=1 - [F175B_MOUSE]	12.05	2	4	4	8	0.96	0.99	1.02	1.06	1.21	0.93	0.96	0.89	0.96	0.99	1.06	1.00	1.12	46.91	6.18
Q8BHL5-2	Isoform 2 of Engulfment and cell motility protein 2 OS=Mus musculus GN=Elmo2 - [ELMO2_MOUSE]	15.42	7	7	9	20	0.97	1.10	1.19	1.09	1.12	0.89	0.91	0.89	0.95	0.87	0.95	0.99	1.12	82.49	5.96
Q80UY2-2	Isoform 2 of E3 ubiquitin-protein ligase KCMF1 OS=Mus musculus GN=Kcmf1 - [KCMF1_MOUSE]	3.04	2	1	1	2	1.04	0.83	0.79	0.88	0.85	1.10	1.05	0.89	0.86	0.94	0.90	0.98	0.94	32.12	5.49
Q8K3W0	BRCA1-A complex subunit BRE OS=Mus musculus GN=Bre PE=1 SV=2 - [BRE_MOUSE]	12.53	7	4	4	4	1.03	1.21	1.17	1.13	1.10	1.00	1.03	0.89	0.91	1.01	1.00	1.05	1.08	43.52	5.94
Q810U3	Neurofascin OS=Mus musculus GN=Nfasc PE=1 SV=1 - [NFASC_MOUSE]	39.76	1	4	38	201	1.01	1.03	1.11	1.06	1.02	0.93	0.91	0.89	0.85	0.96	0.89	0.99	0.94	137.89	6.19
Q80Y98-3	Isoform 3 of Phospholipase DDHD2 OS=Mus musculus GN=Ddhd2 - [DDHD2_MOUSE]	13.60	3	5	5	6	1.04	1.21	1.06	0.98	0.93	1.14	1.06	0.89	0.93	1.22	1.13	1.07	1.03	64.51	5.14
Q9CQF8	Ribosomal protein 63, mitochondrial OS=Mus musculus GN=Mrp63 PE=2 SV=1 - [RT63_MOUSE]	20.59	1	2	2	4	0.89	0.75	0.85	0.82	0.95	0.81	0.91	0.89	1.03	0.82	0.93	0.71	0.78	11.94	10.15

O08842	GDNF family receptor alpha-2 OS=Mus musculus GN=Gfra2 PE=2 SV=1 - [GFRA2_MOUSE]	14.47	3	6	6	11	0.93	0.96	1.04	1.02	1.05	0.91	0.94	0.89	0.93	0.80	0.83	0.90	1.00	51.56	7.75
P39688	Tyrosine-protein kinase Fyn OS=Mus musculus GN=Fyn PE=1 SV=4 - [FYN_MOUSE]	23.09	55	5	11	23	1.12	1.07	0.90	1.13	0.94	0.89	0.80	0.89	0.79	1.01	0.86	1.02	0.96	60.64	6.67
Q920N7	Synaptotagmin-12 OS=Mus musculus GN=Syt12 PE=2 SV=1 - [SYT12_MOUSE]	26.60	1	11	11	19	1.03	1.04	1.10	1.19	1.18	0.87	0.86	0.89	0.90	0.93	0.89	0.88	0.91	46.65	5.64
O88851	Putative hydrolase RBBP9 OS=Mus musculus GN=Rbbp9 PE=1 SV=2 - [RBBP9_MOUSE]	40.32	1	5	5	11	0.90	0.94	1.18	1.06	1.27	0.91	1.01	0.89	0.92	0.95	1.06	0.96	1.10	20.90	5.97
Q8VEK0	Cell cycle control protein 50A OS=Mus musculus GN=Tmem30a PE=1 SV=1 - [CC50A_MOUSE]	18.68	2	6	6	18	1.03	1.00	0.98	1.09	1.00	1.02	0.97	0.89	0.91	1.02	0.97	1.04	1.06	41.03	8.37
F6RUA3	Striatin-interacting proteins 2 (Fragment) OS=Mus musculus GN=Strip2 PE=4 SV=1 - [F6RUA3_MOUSE]	2.33	5	1	2	3	1.14	0.99	0.87	1.13	0.99	0.85	0.74	0.90	0.78	0.84	0.74	1.26	1.11	88.86	5.40
D3Z0Q6	Glycogen [starch] synthase, muscle OS=Mus musculus GN=Gys1 PE=2 SV=1 - [D3Z0Q6_MOUSE]	4.90	2	2	2	4	1.01	1.07	1.05	1.01	1.00	0.81	0.80	0.90	0.88	1.06	1.05	0.92	0.91	76.58	6.47
Q8BIK4	Dedicator of cytokinesis protein 9 OS=Mus musculus GN=Dock9 PE=1 SV=2 - [DOCK9_MOUSE]	4.72	5	6	6	9	0.96	1.00	1.00	1.14	1.20	0.80	0.89	0.90	0.91	0.90	1.01	0.88	0.95	235.16	7.25
Q91YW3	DnaJ homolog subfamily C member 3 OS=Mus musculus GN=Dnajc3 PE=1 SV=1 - [DNJC3_MOUSE]	13.49	1	5	5	11	0.97	0.93	0.97	0.89	0.91	0.85	0.89	0.90	0.88	0.86	0.88	0.85	0.86	57.43	5.85
Q61142-2	Isoform 2 of Spindlin-1 OS=Mus musculus GN=Spin1 - [SPIN1_MOUSE]	45.42	2	9	9	21	0.82	0.80	1.01	0.90	1.08	0.85	1.09	0.90	0.95	0.82	0.95	0.72	0.86	27.12	5.53
Q60709	Amyloid-like protein 2 OS=Mus musculus GN=Aplp2 PE=2 SV=1 - [Q60709_MOUSE]	21.44	2	2	15	40	1.02	0.90	0.98	0.92	1.04	0.79	0.86	0.90	0.90	0.95	0.93	0.84	0.85	85.19	4.73
F8WI56	Disco-interacting protein 2 homolog A OS=Mus musculus GN=Dip2a PE=2 SV=1 - [F8WI56_MOUSE]	7.55	3	6	9	16	1.05	1.12	1.09	1.03	0.95	0.95	0.95	0.90	0.98	0.92	0.96	0.94	0.99	169.41	7.77

E9PUE7	Active breakpoint cluster region-related protein OS=Mus musculus GN=Abr PE=2 SV=1 - [E9PUE7_MOUSE]	28.41	9	17	18	52	1.00	0.98	1.04	1.08	1.09	0.96	0.92	0.90	0.87	0.96	0.96	0.96	0.92	92.43	7.08
Q6PDC8-3	Isoform 3 of Major facilitator superfamily domain-containing protein 4 OS=Mus musculus GN=Mfsd4 - [MFSD4_MOUSE]	3.35	2	1	1	2	0.88	0.96	1.09	1.16	1.31	0.93	1.05	0.90	1.01	0.93	1.05	0.85	0.97	39.37	7.40
A6H584	Collagen alpha-5(VI) chain OS=Mus musculus GN=Col6a5 PE=1 SV=2 - [CO6A5_MOUSE]	0.23	2	1	1	2	0.94	1.09	1.16	0.95	1.01	0.96	1.01	0.90	0.95	1.06	1.13	0.93	0.99	289.44	6.55
A6PWE1	Coilin (Fragment) OS=Mus musculus GN=Coil PE=2 SV=1 - [A6PWE1_MOUSE]	3.47	3	2	2	2	0.79	0.81	1.03	0.64	0.80	0.88	1.11	0.90	1.13	1.19	1.51	0.96	1.21	58.82	9.51
Q9D1G1	Ras-related protein Rab-1B OS=Mus musculus GN=Rab1b PE=1 SV=1 - [RAB1B_MOUSE]	63.18	10	4	10	116	1.19	0.99	0.87	1.08	0.99	0.94	0.87	0.90	0.86	0.87	0.80	0.97	0.86	22.17	5.73
Q8BNE1-2	Isoform 2 of Protein FAM115A OS=Mus musculus GN=Fam115a - [F115A_MOUSE]	3.80	3	2	2	3	0.94	1.22	1.29	0.99	1.05	0.97	1.02	0.90	0.95	0.95	1.01	1.15	1.22	102.44	6.93
P83887	Tubulin gamma-1 chain OS=Mus musculus GN=Tubg1 PE=1 SV=1 - [TBG1_MOUSE]	24.17	1	1	6	21	0.91	1.47	1.62	0.94	1.03	1.00	1.09	0.90	0.98	0.91	1.01	0.97	1.07	51.07	6.02
Q5FWH7-	Isoform 2 of Zinc transporter ZIP12 OS=Mus musculus GN=Slc39a12 - [S39AC_MOUSE]	8.12	3	4	4	7	1.07	1.27	1.15	1.05	0.96	0.87	0.79	0.90	0.81	0.78	0.70	0.92	0.85	72.39	5.66
B2RSH2	Guanine nucleotide-binding protein G(i) subunit alpha-1 OS=Mus musculus GN=Gnai1 PE=2 SV=1 - [GNAI1_MOUSE]	51.13	6	7	14	159	1.01	1.08	1.11	1.14	1.10	0.93	0.91	0.90	0.89	0.94	0.92	1.05	1.02	40.34	5.97
Q6PDI5	Proteasome-associated protein ECM29 homolog OS=Mus musculus GN=Ecm29 PE=1 SV=3 - [ECM29_MOUSE]	3.42	7	4	4	7	1.02	0.92	0.97	1.12	1.15	0.90	0.87	0.90	0.91	0.94	0.96	1.01	0.99	203.57	7.06
Q9CR95	Adaptin ear-binding coat-associated protein 1 OS=Mus musculus GN=Necap1 PE=1 SV=2 - [NECP1_MOUSE]	49.09	1	11	12	44	0.95	0.92	1.01	0.86	0.94	0.90	0.93	0.90	0.93	0.84	0.87	0.80	0.81	29.62	6.38

Q80U19	Disheveled-associated activator of morphogenesis 2 OS=Mus musculus GN=Daam2 PE=2 SV=4 - [DAAM2_MOUSE]	9.51	2	8	9	18	0.94	1.02	1.25	1.15	1.20	0.94	0.98	0.90	0.94	1.08	1.13	1.05	1.14	128.29	6.92
P97449	Aminopeptidase N OS=Mus musculus GN=Anpep PE=1 SV=4 - [AMPN_MOUSE]	12.42	1	11	11	19	1.04	0.91	0.85	1.00	0.95	0.79	0.75	0.90	0.83	0.86	0.83	0.88	0.80	109.58	5.90
Q641P0	Actin-related protein 3B OS=Mus musculus GN=Actr3b PE=2 SV=1 - [ARP3B_MOUSE]	42.82	2	12	15	48	0.91	0.96	1.02	1.08	1.19	0.96	1.08	0.90	0.96	0.90	0.99	0.94	1.07	47.55	6.02
Q9CQG1-	Isoform 2 of Cation transport regulator-like protein 2 OS=Mus musculus GN=Chac2 - [CHAC2_MOUSE]	7.83	2	1	1	2	0.95	1.21	1.27	0.84	0.88	0.69	0.73	0.90	0.94	0.86	0.90	1.01	1.06	18.92	6.04
O89023	Tripeptidyl-peptidase 1 OS=Mus musculus GN=Tpp1 PE=1 SV=2 - [TPP1_MOUSE]	7.47	1	3	3	7	1.00	0.90	0.91	1.02	1.05	1.00	1.00	0.90	0.91	1.09	1.09	1.08	1.08	61.30	6.57
A2AA71	Protein transport protein Sec24A OS=Mus musculus GN=Sec24a PE=2 SV=1 - [A2AA71_MOUSE]	2.66	2	2	2	3	1.03	1.37	1.33	1.10	1.07	1.33	1.29	0.90	0.87	0.97	0.95	1.09	1.07	118.58	7.83
Q6A0A9	Constitutive coactivator of PPAR-gamma-like protein 1 OS=Mus musculus GN=FAM120A PE=1 SV=2 - [F120A_MOUSE]	10.88	1	8	8	21	0.91	0.87	0.89	1.03	1.12	0.92	1.03	0.90	0.98	0.95	1.07	0.96	1.06	121.57	8.92
E9PWX1	Dipeptidyl aminopeptidase-like protein 6 OS=Mus musculus GN=Dpp6 PE=2 SV=1 - [E9PWX1_MOUSE]	36.79	4	24	25	73	1.13	1.01	0.85	1.10	0.93	0.94	0.78	0.90	0.75	0.99	0.86	0.98	0.88	97.16	6.48
O35127	Protein C10 OS=Mus musculus GN=Grcc10 PE=2 SV=1 - [C10_MOUSE]	57.94	1	5	5	9	0.95	0.66	0.77	0.87	0.93	0.82	0.94	0.90	1.02	0.84	0.89	0.81	0.88	13.19	5.14
P62838	Ubiquitin-conjugating enzyme E2 D2 OS=Mus musculus GN=Ube2d2 PE=1 SV=1 - [UB2D2_MOUSE]	40.82	3	1	4	17	0.79	1.15	1.45	0.93	1.17	0.87	1.10	0.90	1.13	0.85	1.08	0.91	1.15	16.72	7.83
Q8K406	Leucine-rich repeat LGI family member 3 OS=Mus musculus GN=Lgi3 PE=1 SV=1 - [LGI3_MOUSE]	17.34	1	7	7	17	0.89	0.97	0.99	1.04	1.11	0.93	0.92	0.90	1.01	0.90	1.04	0.86	0.92	61.78	8.16

F6V6T4	Transmembrane emp24 domain-containing protein 2 (Fragment) OS=Mus musculus GN=Tmed2 PE=3 SV=1 - [F6V6T4_MOUSE]	28.41	3	3	3	5	1.07	1.16	1.03	1.19	1.37	0.74	0.74	0.90	0.84	0.85	0.80	0.65	0.54	20.01	9.07
Q50L41	Cytosolic phospholipase A2 zeta OS=Mus musculus GN=Pla2g4f PE=2 SV=3 - [PA24F_MOUSE]	1.52	1	1	1	1	0.99	1.06	1.08	1.03	1.04	0.77	0.78	0.90	0.91	0.86	0.88	1.07	1.09	96.30	5.73
P51432	1-phosphatidylinositol 4,5-bisphosphate phosphodiesterase beta-3 OS=Mus musculus GN=Plcb3 PE=2 SV=2 - [PLCB3_MOUSE]	6.24	3	4	5	10	0.98	1.30	1.32	1.03	1.07	0.92	1.00	0.90	0.87	1.01	1.02	1.11	1.13	139.40	5.94
P84309	Adenylate cyclase type 5 OS=Mus musculus GN=Adcy5 PE=1 SV=2 - [ADCY5_MOUSE]	13.15	3	10	11	27	1.04	1.13	1.06	1.29	1.28	1.02	0.98	0.90	0.82	1.25	1.18	1.42	1.30	139.03	7.06
O88533	Aromatic-L-amino-acid decarboxylase OS=Mus musculus GN=Ddc PE=2 SV=1 - [DDC_MOUSE]	8.13	2	4	4	6	1.26	1.14	0.92	1.19	1.00	0.97	0.80	0.90	0.73	1.10	0.92	1.47	1.17	53.84	6.60
Q99L04	Dehydrogenase/reductase SDR family member 1 OS=Mus musculus GN=Dhrs1 PE=2 SV=1 - [DHRS1_MOUSE]	33.55	1	7	7	14	0.94	1.04	1.07	0.97	1.13	0.95	1.03	0.90	1.00	0.93	0.98	1.06	1.04	33.98	8.35
Q8JZR0	Long-chain-fatty-acid--CoA ligase 5 OS=Mus musculus GN=Acsl5 PE=2 SV=1 - [ACSL5_MOUSE]	6.30	2	2	3	7	0.89	0.95	1.02	1.05	1.18	0.96	1.08	0.90	0.96	0.94	1.15	0.87	1.08	76.16	7.09
Q921M7	Protein FAM49B OS=Mus musculus GN=Fam49b PE=2 SV=1 - [FA49B_MOUSE]	41.36	1	9	11	53	1.01	1.14	1.18	1.13	1.08	0.93	0.95	0.90	0.89	1.01	0.99	1.08	1.15	36.75	6.06
P08032	Spectrin alpha chain, erythrocytic 1 OS=Mus musculus GN=Spta1 PE=2 SV=3 - [SPTA1_MOUSE]	1.28	1	2	3	17	0.92	0.95	0.96	0.45	0.49	0.82	0.88	0.90	0.97	1.07	1.16	0.67	0.73	279.69	5.03
Q3UPL0	Protein transport protein Sec31A OS=Mus musculus GN=Sec31a PE=1 SV=2 - [SC31A_MOUSE]	20.41	3	18	19	44	0.97	1.01	1.08	1.05	1.10	1.02	1.03	0.90	0.94	1.00	0.98	0.95	1.03	133.49	6.76
D3Z5M2	Protein Gm10110 OS=Mus musculus GN=Gm10110 PE=4 SV=1 - [D3Z5M2_MOUSE]	25.20	5	7	13	35	0.89	0.80	0.89	0.95	1.07	0.86	0.94	0.90	1.00	0.93	0.98	0.86	0.99	67.72	9.50
P70122	Ribosome maturation protein SBDS OS=Mus musculus GN=Sbds PE=1 SV=4 - [SBDS_MOUSE]	38.80	2	8	8	16	0.89	0.83	0.96	1.07	1.10	0.90	1.01	0.90	1.00	0.90	0.95	0.95	1.07	28.76	8.76

Q9Z0V7	Mitochondrial import inner membrane translocase subunit Tim17-B OS=Mus musculus GN=Timm17b PE=2 SV=1 - [TI17B_MOUSE]	8.72	1	1	1	3	0.81	1.05	1.29	0.91	1.12	0.93	1.13	0.90	1.10	0.76	0.94	0.92	1.13	18.34	9.03
Q9JHQ5	Leucine zipper transcription factor-like protein 1 OS=Mus musculus GN=Lztfl1 PE=2 SV=1 - [LZTL1_MOUSE]	41.14	4	11	11	31	0.88	0.70	0.85	0.80	0.89	0.84	0.92	0.90	1.00	0.76	0.91	0.73	0.87	34.75	5.17
P62814	V-type proton ATPase subunit B, brain isoform OS=Mus musculus GN=Atp6v1b2 PE=1 SV=1 - [VATB2_MOUSE]	83.37	2	31	31	304	0.96	1.07	1.15	1.06	1.11	0.89	0.92	0.90	0.94	0.93	0.97	0.94	0.99	56.51	5.81
Q91Z49-2	Isoform 2 of UAP56-interacting factor OS=Mus musculus GN=Fyttd1 - [UIF_MOUSE]	7.77	2	2	2	4	0.97	0.97	1.01	1.07	1.11	0.96	0.99	0.90	0.93	0.96	0.99	1.04	1.08	32.27	11.87
Q3U7R1	Extended synaptotagmin-1 OS=Mus musculus GN=Esyt1 PE=2 SV=2 - [ESYT1_MOUSE]	6.04	2	6	6	8	0.83	0.98	1.19	1.04	1.17	0.85	1.01	0.90	1.00	0.92	1.03	0.86	1.05	121.48	5.95
D3YWC2	ADP-ribosylation factor-like 6 interacting protein 4 OS=Mus musculus GN=Arl6ip4 PE=4 SV=1 - [D3YWC2_MOUSE]	18.50	3	3	3	6	0.77	0.90	1.15	0.89	1.07	0.94	1.06	0.90	1.19	0.82	1.00	0.70	0.89	22.59	11.30
G3UW82	MCG140437, isoform CRA_d OS=Mus musculus GN=Myh2 PE=4 SV=1 - [G3UW82_MOUSE]	30.23	3	3	53	175	0.99	2.08	1.58	0.83	0.97	7.56	8.79	0.90	0.95	0.87	0.99	1.11	1.08	223.08	5.77
Q5PR73	GTP-binding protein Di-Ras2 OS=Mus musculus GN=Diras2 PE=2 SV=1 - [DIRA2_MOUSE]	42.71	1	6	8	26	0.90	0.95	1.01	1.05	1.15	0.92	0.99	0.90	0.93	0.88	0.95	0.93	1.01	22.48	8.76
Q9R069	Basal cell adhesion molecule OS=Mus musculus GN=Bcam PE=2 SV=1 - [BCAM_MOUSE]	16.40	2	7	7	16	0.98	0.80	0.82	0.91	0.95	0.87	0.89	0.90	0.96	0.87	0.84	0.75	0.76	67.63	6.25
Q8CJH3	Plexin-B1 OS=Mus musculus GN=Plxnb1 PE=1 SV=2 - [PLXB1_MOUSE]	7.13	1	10	10	17	1.04	1.17	1.15	1.10	1.06	0.91	0.86	0.90	0.89	0.99	0.94	1.01	1.00	231.23	5.55
P28663	Beta-soluble NSF attachment protein OS=Mus musculus GN=Napb PE=1 SV=2 - [SNAB_MOUSE]	66.11	2	13	16	108	1.07	1.09	1.01	1.11	1.05	0.99	0.95	0.90	0.89	1.00	0.95	1.08	1.07	33.54	5.47
Q62203	Splicing factor 3A subunit 2 OS=Mus musculus GN=Sf3a2 PE=2 SV=2 - [SF3A2_MOUSE]	9.05	3	2	2	3	0.89	0.60	0.67	0.89	1.01	0.95	1.06	0.90	1.01	0.78	0.88	0.82	0.92	49.88	9.54

Q91ZJ5	UTP--glucose-1-phosphate uridylyltransferase OS=Mus musculus GN=Ugp2 PE=2 SV=3 - [UGPA_MOUSE]	37.99	2	15	15	40	1.01	0.94	0.97	1.10	1.10	0.95	0.94	0.90	0.90	0.97	0.98	1.04	1.09	56.94	7.61
Q9JIF7	Coatomer subunit beta OS=Mus musculus GN=Copb1 PE=1 SV=1 - [COPB_MOUSE]	9.76	1	7	7	18	0.98	1.09	1.13	1.04	1.13	0.85	0.89	0.90	0.87	1.01	0.92	0.99	1.00	107.00	6.00
B1ART1	Protein Vps13d OS=Mus musculus GN=Vps13d PE=2 SV=1 - [B1ART1_MOUSE]	0.71	3	2	2	4	1.04	1.32	1.27	1.00	0.97	0.94	0.91	0.90	0.86	1.06	1.03	1.15	1.11	487.02	6.49
Q9Z1Q9	Valine--tRNA ligase OS=Mus musculus GN=Vars PE=2 SV=1 - [SYVC_MOUSE]	23.83	6	24	24	46	0.91	0.99	1.06	1.09	1.19	0.93	1.02	0.90	0.97	0.96	1.05	0.93	1.03	140.13	7.77
B2RR83	Probable ATP-dependent RNA helicase YTHDC2 OS=Mus musculus GN=Ythdc2 PE=2 SV=1 - [YTDC2_MOUSE]	1.31	1	1	1	1	0.86	1.05	1.21	0.96	1.11	1.00	1.15	0.90	1.04	0.70	0.81	1.02	1.19	160.99	8.51
Q3V3R1	Monofunctional C1-tetrahydrofolate synthase, mitochondrial OS=Mus musculus GN=Mthfd1l PE=1 SV=2 - [C1TM_MOUSE]	13.00	1	10	10	21	0.89	1.03	1.19	1.15	1.26	0.93	1.01	0.90	0.99	0.95	0.99	0.94	1.03	105.66	7.02
Q9EQ20	Methylmalonate-semialdehyde dehydrogenase [acylating], mitochondrial OS=Mus musculus GN=Aldh6a1 PE=1 SV=1 - [MMSA_MOUSE]	53.64	1	21	21	59	1.03	1.08	1.02	1.15	1.09	0.98	0.92	0.90	0.89	0.95	0.98	1.05	0.96	57.88	8.07
Q9Z110-2	Isoform Short of Delta-1-pyrroline-5-carboxylate synthase OS=Mus musculus GN=Aldh18a1 - [P5CS_MOUSE]	16.90	2	11	11	20	0.96	1.12	1.17	1.05	1.08	0.91	0.87	0.90	0.91	0.90	0.96	1.02	1.04	87.00	7.55
Q3ULJ0-2	Isoform 2 of Glycerol-3-phosphate dehydrogenase 1-like protein OS=Mus musculus GN=Gpd1l - [GPD1L_MOUSE]	54.69	4	15	15	45	0.99	1.07	1.04	1.11	1.12	0.96	0.94	0.90	0.89	0.96	0.94	0.96	0.95	34.61	6.87
Q3TN85	UV excision repair protein RAD23 homolog A (Fragment) OS=Mus musculus GN=Rad23a PE=2 SV=1 - [Q3TN85_MOUSE]	36.34	4	7	9	26	1.11	1.09	0.95	0.94	0.84	0.97	0.82	0.90	0.87	0.85	0.76	0.90	0.80	34.81	4.69
A2AG47	Protein Maged2 (Fragment) OS=Mus musculus GN=Maged2 PE=2 SV=1 - [A2AG47_MOUSE]	3.76	3	1	1	1	0.96	1.52	1.58	1.05	1.09	0.96	1.00	0.90	0.93	1.00	1.04	1.05	1.09	22.70	9.92

P50396	Rab GDP dissociation inhibitor alpha OS=Mus musculus GN=Gdi1 PE=1 SV=3 - [GDIA_MOUSE]	72.48	3	25	31	281	1.04	0.94	0.91	1.03	0.99	0.94	0.89	0.90	0.88	0.94	0.91	0.96	0.92	50.49	5.08
Q3V3Q7	Phosphofurin acidic cluster sorting protein 2 OS=Mus musculus GN=Pacs2 PE=1 SV=2 - [PACS2_MOUSE]	5.57	2	2	3	4	1.18	1.32	1.17	1.06	0.89	0.94	0.79	0.90	0.76	0.87	0.74	1.12	0.95	94.87	6.51
Q04736	Tyrosine-protein kinase Yes OS=Mus musculus GN=Yes1 PE=1 SV=3 - [YES_MOUSE]	16.27	54	2	9	22	1.11	1.33	1.20	1.28	1.15	0.89	0.80	0.90	0.81	1.16	1.04	1.11	1.00	60.59	6.64
P47738	Aldehyde dehydrogenase, mitochondrial OS=Mus musculus GN=Aldh2 PE=1 SV=1 - [ALDH2_MOUSE]	43.55	4	16	17	49	1.03	1.00	1.01	1.07	1.07	0.93	0.94	0.90	0.94	0.96	0.96	0.99	1.01	56.50	7.62
Q8CFX0	Protein Wdr17 OS=Mus musculus GN=Wdr17 PE=2 SV=1 - [Q8CFX0_MOUSE]	0.94	5	1	1	2	0.92	1.22	1.33	0.99	1.08	0.93	1.01	0.90	0.98	1.01	1.10	0.96	1.04	141.52	6.81
E9Q6P5	Protein Ttc7b OS=Mus musculus GN=Ttc7b PE=2 SV=1 - [E9Q6P5_MOUSE]	17.56	1	12	12	31	0.96	1.03	1.06	1.09	1.17	0.83	0.89	0.90	0.89	0.87	0.90	0.92	0.92	94.14	6.90
Q9CWV6	PRKR-interacting protein 1 OS=Mus musculus GN=Prkrip1 PE=1 SV=2 - [PKRI1_MOUSE]	4.84	1	1	1	1	0.83	1.10	1.32	0.79	0.94	0.71	0.85	0.90	1.08	0.81	0.97	0.61	0.74	21.48	9.42
Q8R1S0	Ubiquinone biosynthesis monooxygenase COQ6 OS=Mus musculus GN=Coq6 PE=2 SV=3 - [COQ6_MOUSE]	2.73	1	1	1	2	0.90	1.20	1.34	0.96	1.07	0.83	0.91	0.90	1.00	0.99	1.10	1.14	1.27	51.36	7.17
Q9QYA2	Mitochondrial import receptor subunit TOM40 homolog OS=Mus musculus GN=Tomm40 PE=1 SV=3 - [TOM40_MOUSE]	9.70	1	2	2	5	1.07	1.16	1.08	1.29	1.20	0.85	0.79	0.90	0.84	0.94	0.88	1.20	1.12	37.87	7.74
Q69ZN6-2	Isoform 2 of N-acetylglucosamine-1-phosphotransferase subunits alpha/beta OS=Mus musculus GN=Gnptab - [GNPTA_MOUSE]	0.67	2	1	1	4	0.83	1.15	1.39	1.02	1.23	0.88	1.06	0.90	1.09	0.90	1.09	0.98	1.19	135.16	7.59
Q9D0F9	Phosphoglucomutase-1 OS=Mus musculus GN=Pgm1 PE=1 SV=4 - [PGM1_MOUSE]	51.96	3	25	25	87	1.06	1.05	0.96	1.10	1.06	0.96	0.89	0.90	0.82	1.04	0.99	1.07	1.00	61.38	6.57
Q3UH93	Plexin-D1 OS=Mus musculus GN=Plxnd1 PE=1 SV=1 - [PLXD1_MOUSE]	9.40	2	13	13	23	1.02	1.09	1.07	1.15	1.13	0.95	0.91	0.90	0.86	0.99	0.95	1.02	0.98	211.47	7.11

Q8BP47	Asparagine--tRNA ligase, cytoplasmic OS=Mus musculus GN=Nars PE=1 SV=2 - [SYNC_MOUSE]	28.80	1	14	15	52	0.93	1.05	1.12	1.05	1.13	0.88	0.95	0.90	0.96	0.94	1.04	0.95	1.04	64.24	5.86
Q9D880	Mitochondrial import inner membrane translocase subunit TIM50 OS=Mus musculus GN=Timm50 PE=1 SV=1 - [TIM50_MOUSE]	17.85	1	5	5	16	0.98	1.32	1.31	1.25	1.29	0.87	0.91	0.90	0.87	0.98	1.04	1.06	1.04	39.75	8.13
Q8R059	UDP-glucose 4-epimerase OS=Mus musculus GN=Gale PE=2 SV=1 - [GALE_MOUSE]	2.88	1	1	1	1	1.13	0.78	0.69	1.07	0.95	0.93	0.82	0.90	0.80	1.08	0.96	0.95	0.84	38.20	6.74
D3Z7P3	Glutaminase kidney isoform, mitochondrial OS=Mus musculus GN=Gls PE=1 SV=1 - [GLSK_MOUSE]	54.30	9	25	25	127	0.96	0.96	1.02	1.05	1.10	0.88	0.97	0.90	0.97	0.93	1.01	0.94	1.00	73.92	7.99
A2BEE9	Disks large homolog 3 OS=Mus musculus GN=Dlg3 PE=2 SV=1 - [A2BEE9_MOUSE]	44.67	4	26	28	87	0.94	1.03	1.04	0.95	1.02	0.88	0.92	0.90	0.93	0.85	0.92	0.85	0.93	92.06	6.86
A2AGT5-	Isoform 2 of Cytoskeleton-associated protein 5 OS=Mus musculus GN=Ckap5 - [CKAP5_MOUSE]	12.02	4	19	19	32	0.98	0.95	1.02	1.09	1.10	0.98	1.01	0.90	0.95	1.01	1.03	1.03	1.05	218.57	8.21
Q61035	Histidine--tRNA ligase, cytoplasmic OS=Mus musculus GN=Hars PE=2 SV=2 - [SYHC_MOUSE]	27.11	1	9	12	24	1.12	0.91	0.92	1.06	0.98	0.95	0.90	0.90	0.85	0.90	0.80	0.97	0.96	57.40	6.00
Q99JP6	Homer protein homolog 3 OS=Mus musculus GN=Homer3 PE=1 SV=2 - [HOME3_MOUSE]	4.49	4	1	2	2	0.89	1.04	1.17	0.80	0.90	0.87	0.98	0.90	1.01	0.78	0.88	0.88	1.00	39.67	5.36
F7B9G4	Glutamyl aminopeptidase (Fragment) OS=Mus musculus GN=Enpep PE=4 SV=1 - [F7B9G4_MOUSE]	3.59	2	1	1	2	1.09	1.50	1.37	1.23	1.12	0.85	0.78	0.90	0.82	1.01	0.92	1.10	1.01	57.43	5.21
Q3UHE1-	Isoform 2 of Membrane associated phosphatidylinositol transfer protein 3 OS=Mus musculus GN=Pitpnm3 - [PITM3_MOUSE]	9.60	2	7	7	14	0.81	0.94	1.16	0.79	1.02	0.92	1.13	0.90	1.43	0.89	1.15	0.91	1.16	104.71	7.12
E9Q035	Protein Gm20425 OS=Mus musculus GN=Gm20425 PE=4 SV=1 - [E9Q035_MOUSE]	45.09	8	4	44	270	1.00	1.10	1.05	0.98	1.04	0.90	0.82	0.90	0.83	0.97	0.95	0.88	0.93	107.73	7.77
Q6ZWR6-	Isoform 4 of Nesprin-1 OS=Mus musculus GN=Syne1 - [SYNE1_MOUSE]	2.28	4	14	17	33	0.91	1.04	1.13	1.10	1.21	0.93	1.01	0.90	0.98	0.95	1.09	0.97	1.03	1009.04	5.59

Q9Z0S1	3'(2'),5'-bisphosphate nucleotidase 1 OS=Mus musculus GN=Bpnt1 PE=1 SV=2 - [BPNT1_MOUSE]	43.18	3	9	9	20	0.95	1.00	1.05	1.03	1.07	0.86	0.92	0.90	0.92	0.95	0.92	1.07	1.07	33.18	5.82
Q9DCD2	Pre-mRNA-splicing factor SYF1 OS=Mus musculus GN=Xab2 PE=2 SV=1 - [SYF1_MOUSE]	2.81	2	2	2	2	0.75	1.07	1.43	0.99	1.33	0.92	1.22	0.90	1.20	0.86	1.15	0.96	1.29	99.92	6.23
Q80ZX0	Protein Sec24b OS=Mus musculus GN=Sec24b PE=2 SV=1 - [Q80ZX0_MOUSE]	7.67	3	6	6	13	1.00	1.03	1.00	1.07	1.03	0.92	1.03	0.90	0.91	1.03	1.02	1.03	0.96	135.47	6.96
P29387	Guanine nucleotide-binding protein subunit beta-4 OS=Mus musculus GN=Gnb4 PE=2 SV=4 - [GBB4_MOUSE]	21.18	1	2	6	35	0.99	1.03	1.04	1.11	1.03	0.95	0.94	0.90	0.86	0.89	0.96	1.04	0.95	37.36	6.16
Q9WTX8-	Isoform 2 of Mitotic spindle assembly checkpoint protein MAD1 OS=Mus musculus GN=Mad1l1 - [MD1L1_MOUSE]	5.06	2	2	2	3	1.01	1.33	1.32	0.98	0.97	1.22	1.21	0.90	0.89	1.02	1.01	1.08	1.07	60.11	5.66
A2ACG7	Dolichyl-diphosphooligosaccharide--protein glycosyltransferase subunit 2 OS=Mus musculus GN=Rpn2 PE=2 SV=1 - [A2ACG7_MOUSE]	28.46	2	10	10	19	1.10	1.12	1.01	1.14	0.99	0.85	0.74	0.90	0.79	0.92	0.83	1.04	0.81	67.46	6.23
P21278	Guanine nucleotide-binding protein subunit alpha-11 OS=Mus musculus GN=Gna11 PE=1 SV=1 - [GNA11_MOUSE]	44.01	1	9	13	32	1.08	1.29	1.21	1.14	1.06	0.94	0.87	0.90	0.87	0.99	0.89	1.11	1.07	42.00	5.97
Q80U56	Late secretory pathway protein AVL9 homolog OS=Mus musculus GN=Avl9 PE=2 SV=2 - [AVL9_MOUSE]	15.41	1	7	7	15	1.05	1.19	1.16	1.10	1.04	0.99	0.94	0.90	0.81	0.93	1.02	1.07	1.00	72.14	6.14
Q9D906	Ubiquitin-like modifier-activating enzyme ATG7 OS=Mus musculus GN=Atg7 PE=1 SV=1 - [ATG7_MOUSE]	9.31	1	6	6	8	0.91	1.14	1.24	1.08	1.16	0.84	0.93	0.90	0.83	0.87	0.90	1.00	1.05	77.47	6.40
Q8BGB5	LIM domain-containing protein 2 OS=Mus musculus GN=Limd2 PE=2 SV=1 - [LIMD2_MOUSE]	26.56	1	4	4	7	1.06	0.80	0.75	0.83	0.81	1.11	1.01	0.90	0.86	0.97	0.90	0.94	0.89	14.23	9.04

Q6R0H7	Guanine nucleotide-binding protein G(s) subunit alpha isoforms XLas OS=Mus musculus GN=Gnas PE=2 SV=1 - [GNAS1_MOUSE]	9.00	13	7	10	61	1.01	1.18	1.24	1.07	1.10	0.91	0.99	0.90	0.90	1.01	1.04	0.98	1.01	121.43	4.81
P30548	Substance-P receptor OS=Mus musculus GN=Tacr1 PE=2 SV=2 - [NK1R_MOUSE]	3.69	1	1	1	2	1.41	2.93	2.08	1.32	0.94	1.15	0.81	0.90	0.64	1.44	1.03	2.06	1.47	46.29	7.64
P62242	40S ribosomal protein S8 OS=Mus musculus GN=Rps8 PE=1 SV=2 - [RS8_MOUSE]	38.46	1	7	7	17	1.03	0.90	0.95	1.06	1.04	0.98	0.93	0.90	0.93	1.02	0.98	1.03	1.05	24.19	10.32
P18872	Guanine nucleotide-binding protein G(o) subunit alpha OS=Mus musculus GN=Gnao1 PE=1 SV=3 - [GNAO_MOUSE]	63.28	9	20	22	379	0.99	1.09	1.06	1.09	1.12	0.92	0.94	0.90	0.92	0.95	0.97	1.02	1.05	40.06	5.53
Q8K4Z5	Splicing factor 3A subunit 1 OS=Mus musculus GN=Sf3a1 PE=1 SV=1 - [SF3A1_MOUSE]	24.15	1	14	14	29	1.03	0.84	0.88	0.92	0.83	0.92	0.92	0.90	0.96	0.89	0.92	0.95	0.93	88.49	5.22
Q7TN98-5	Isoform 5 of Cytoplasmic polyadenylation element-binding protein 4 OS=Mus musculus GN=Cpeb4 - [CPEB4_MOUSE]	18.75	7	4	8	16	0.93	0.80	0.85	0.97	1.01	1.01	1.07	0.90	0.94	0.83	0.91	0.92	1.01	77.22	6.90
E9PYG6	Protein Rasa1 OS=Mus musculus GN=Rasa1 PE=2 SV=1 - [E9PYG6_MOUSE]	9.73	1	6	6	13	1.08	1.13	1.14	1.14	1.06	0.89	0.89	0.90	0.82	1.12	1.07	1.05	1.05	115.36	6.60
Q2TBE6	Phosphatidylinositol 4-kinase type 2-alpha OS=Mus musculus GN=Pi4k2a PE=1 SV=1 - [P4K2A_MOUSE]	12.73	1	4	4	6	0.88	1.19	1.37	1.08	1.23	0.91	1.02	0.90	1.00	0.92	1.02	0.95	1.05	54.22	8.05
Q8C1B7-3	Isoform 3 of Septin-11 OS=Mus musculus GN=Sept11 - [SEP11_MOUSE]	45.18	3	8	20	103	0.94	0.90	0.91	0.95	0.99	0.89	0.90	0.90	0.96	0.90	0.96	0.84	0.87	48.93	6.81
Q8BSK8-2	Isoform Alpha II of Ribosomal protein S6 kinase beta-1 OS=Mus musculus GN=Rps6kb1 - [KS6B1_MOUSE]	3.98	4	2	2	2	1.04	1.47	1.41	1.02	0.98	1.20	1.15	0.90	0.86	1.11	1.20	0.85	0.82	56.12	6.44
F8WIB1	ADP-ribosylation factor-like protein 1 (Fragment) OS=Mus musculus GN=Arl1 PE=2 SV=1 - [F8WIB1_MOUSE]	25.36	2	2	2	5	1.13	1.13	0.94	1.12	1.01	1.05	0.90	0.90	0.75	0.96	0.87	0.95	1.02	15.64	5.60

P68181-2	Isoform 2 of cAMP-dependent protein kinase catalytic subunit beta OS=Mus musculus GN=Prkacb - [KAPCB_MOUSE]	51.48	4	7	12	49	1.09	0.91	0.85	1.15	1.04	0.91	0.83	0.90	0.82	1.01	0.96	1.07	1.01	39.42	8.56
P35235-2	Isoform 2 of Tyrosine-protein phosphatase non-receptor type 11 OS=Mus musculus GN=Ptpn11 - [PTN11_MOUSE]	31.03	2	14	14	45	1.05	1.03	0.99	1.01	1.02	0.95	0.95	0.90	0.86	0.95	0.91	0.94	0.92	67.99	7.30
Q9DCL9	Multifunctional protein ADE2 OS=Mus musculus GN=Paics PE=1 SV=4 - [PUR6_MOUSE]	35.29	3	13	13	26	1.01	1.11	0.96	1.11	0.96	0.96	0.89	0.90	0.90	0.98	0.90	1.07	1.02	46.98	7.23
P51655	Glypican-4 OS=Mus musculus GN=Gpc4 PE=2 SV=2 - [GPC4_MOUSE]	17.24	3	4	6	19	1.04	0.98	0.96	1.10	1.06	0.99	0.91	0.90	0.87	1.02	1.01	1.20	1.21	62.55	6.33
E9QMW9	Polypyrimidine tract-binding protein 1 OS=Mus musculus GN=Ptbp1 PE=2 SV=1 - [E9QMW9_MOUSE]	11.05	5	1	1	2	1.47	0.96	0.65	1.16	0.79	1.08	0.73	0.90	0.61	1.09	0.74	1.17	0.80	37.12	8.13
O54983	Thiomorpholine-carboxylate dehydrogenase OS=Mus musculus GN=Crym PE=1 SV=1 - [CRYM_MOUSE]	36.42	1	10	10	39	1.05	1.16	1.07	1.09	1.10	0.93	0.89	0.90	0.88	1.08	1.03	1.27	1.20	33.50	5.67
Q0VBP7	Uncharacterized protein KIAA1644 OS=Mus musculus GN=Kiaa1644 PE=2 SV=1 - [K1644_MOUSE]	14.65	2	1	1	4	0.74	1.01	1.36	1.00	1.36	0.96	1.30	0.90	1.22	1.12	1.52	0.82	1.11	22.47	8.79
P60122	RuvB-like 1 OS=Mus musculus GN=Ruvbl1 PE=1 SV=1 - [RUVB1_MOUSE]	24.12	3	9	9	20	1.00	0.88	0.93	1.03	1.07	0.96	0.90	0.90	0.91	0.96	0.95	0.93	0.94	50.18	6.42
Q9CQI6	Coactosin-like protein OS=Mus musculus GN=Cotl1 PE=1 SV=3 - [COTL1_MOUSE]	64.79	1	8	8	28	1.01	1.04	1.03	1.00	1.01	0.90	0.87	0.90	0.90	0.59	0.63	0.85	0.84	15.93	5.40
Q8BFU8	Vesicular glutamate transporter 3 OS=Mus musculus GN=Slc17a8 PE=2 SV=1 - [VGLU3_MOUSE]	5.66	2	2	2	3	0.96	1.05	1.10	0.98	1.02	0.97	1.01	0.90	0.94	1.05	1.09	1.15	1.20	66.11	6.46
Q91XM9	Disks large homolog 2 OS=Mus musculus GN=Dlg2 PE=1 SV=2 - [DLG2_MOUSE]	52.82	11	28	33	139	0.97	0.91	0.95	0.97	1.01	0.90	0.92	0.90	0.94	0.85	0.89	0.89	0.93	94.82	6.24
Q03265	ATP synthase subunit alpha, mitochondrial OS=Mus musculus GN=Atp5a1 PE=1 SV=1 - [ATPA_MOUSE]	66.73	3	39	39	755	0.97	1.09	1.14	1.08	1.12	0.90	0.92	0.90	0.93	0.97	0.99	0.96	1.00	59.72	9.19
Q61696	Heat shock 70 kDa protein 1A OS=Mus musculus GN=Hspa1a PE=1 SV=2 - [HS71A_MOUSE]	53.35	1	1	25	255	0.66	1.29	1.94	0.95	1.42	0.99	1.49	0.90	1.35	1.01	1.52	1.06	1.59	70.04	5.72

P97300	Neuroplastin OS=Mus musculus GN=Nptn PE=1 SV=3 - [NPTN_MOUSE]	34.01	5	7	15	235	1.03	1.05	1.05	1.14	1.14	0.94	0.92	0.90	0.89	0.99	0.93	0.98	0.98	44.35	7.74
E0CZ04	Gamma-glutamylaminecyclotransferase (Fragment) OS=Mus musculus GN=Ggact PE=2 SV=1 - [E0CZ04_MOUSE]	29.52	3	2	2	3	1.06	0.93	0.87	0.94	0.88	0.95	0.89	0.90	0.85	0.99	0.93	1.11	1.04	11.93	5.39
D3Z5P0	Serine/threonine-protein kinase BRSK1 OS=Mus musculus GN=Brsk1 PE=2 SV=1 - [D3Z5P0_MOUSE]	22.90	6	8	11	20	0.93	1.01	1.13	1.04	1.12	0.97	1.06	0.90	0.98	0.96	1.13	0.94	0.99	77.15	9.17
J3QMQ2	Uncharacterized protein OS=Mus musculus GN=Gm10784 PE=4 SV=1 - [J3QMQ2_MOUSE]	10.13	3	1	1	3	1.14	1.07	0.93	1.10	0.96	0.92	0.80	0.90	0.79	0.91	0.80	1.00	0.88	8.70	7.18
F6TU77	Protein Fam184a (Fragment) OS=Mus musculus GN=Fam184a PE=2 SV=1 - [F6TU77_MOUSE]	9.17	5	6	6	15	1.01	0.86	0.90	0.87	0.92	0.92	0.98	0.90	0.94	0.86	0.84	0.84	0.83	117.03	5.74
Q9WV27	Sodium/potassium-transporting ATPase subunit alpha-4 OS=Mus musculus GN=Atp1a4 PE=1 SV=3 - [AT1A4_MOUSE]	13.28	2	1	15	471	0.84	1.06	1.34	0.99	1.21	0.80	0.96	0.90	1.04	0.94	1.03	0.79	0.98	114.81	5.71
Q7TQG1	Pleckstrin homology domain-containing family A member 6 OS=Mus musculus GN=Plekha6 PE=1 SV=1 - [PKHA6_MOUSE]	32.65	2	24	24	61	0.93	0.96	1.00	0.91	0.97	0.84	0.85	0.90	0.93	0.80	0.85	0.84	0.90	131.35	8.97
Q64332-2	Isoform IIb of Synapsin-2 OS=Mus musculus GN=Syn2 - [SYN2_MOUSE]	66.60	1	1	23	423	1.07	0.93	0.87	1.05	1.11	1.04	1.02	0.90	0.84	1.03	1.03	1.02	1.06	52.42	7.72
P11499	Heat shock protein HSP 90-beta OS=Mus musculus GN=Hsp90ab1 PE=1 SV=3 - [HS90B_MOUSE]	58.15	5	25	41	539	1.01	0.95	0.94	1.11	1.08	0.90	0.89	0.90	0.91	0.96	0.94	0.96	0.96	83.23	5.03
A3KGT2	CUGBP Elav-like family member 2 (Fragment) OS=Mus musculus GN=Celf2 PE=2 SV=1 - [A3KGT2_MOUSE]	52.00	13	8	9	31	0.93	0.98	1.06	0.97	1.04	0.88	1.01	0.90	1.03	0.87	1.03	0.96	1.03	22.52	7.69
Q5H8C4-2	Isoform 2 of Vacuolar protein sorting-associated protein 13A OS=Mus musculus GN=Vps13a - [VP13A_MOUSE]	2.12	2	4	5	10	1.06	1.08	0.98	1.10	1.03	0.95	0.90	0.90	0.90	0.95	0.90	1.05	1.01	346.50	6.02

Q9D3D0	Alpha-tocopherol transfer protein-like OS=Mus musculus GN=Ttpal PE=2 SV=3 - [TTPAL_MOUSE]	6.41	2	2	2	3	0.94	0.70	0.74	0.99	1.05	0.96	1.02	0.90	0.95	0.96	1.02	0.94	1.00	38.81	6.44
Q80ZD8	Amphoterin-induced protein 1 OS=Mus musculus GN=Amigo1 PE=1 SV=1 - [AMGO1_MOUSE]	15.04	3	6	6	14	0.93	0.76	0.82	1.09	1.08	0.98	0.98	0.90	0.91	0.83	0.86	0.79	0.80	55.31	6.86
P47809	Dual specificity mitogen-activated protein kinase kinase 4 OS=Mus musculus GN=Map2k4 PE=1 SV=2 - [MP2K4_MOUSE]	26.95	5	9	9	20	0.97	0.94	0.90	1.08	1.10	0.95	0.96	0.90	0.94	0.92	0.96	0.96	0.96	44.09	8.07
Q000W5	Guanylate-binding protein 10 OS=Mus musculus GN=Gbp10 PE=2 SV=1 - [Q000W5_MOUSE]	4.91	1	1	1	1	0.95	1.16	1.22	0.90	0.95	0.95	0.99	0.90	0.95	1.01	1.06	0.93	0.98	70.36	6.46
Q8VE37	Regulator of chromosome condensation OS=Mus musculus GN=Rcc1 PE=1 SV=1 - [RCC1_MOUSE]	6.65	2	2	2	5	0.94	0.81	0.86	0.96	1.02	0.96	1.02	0.90	0.96	0.97	1.04	0.95	1.02	44.90	8.10
Q5PPR2	Exocyst complex component 1 OS=Mus musculus GN=Exoc1 PE=2 SV=1 - [Q5PPR2_MOUSE]	10.35	3	9	9	14	1.00	1.13	1.18	1.13	1.13	0.94	0.93	0.90	0.86	1.04	0.97	1.07	1.12	100.13	6.52
Q80W47	WD repeat domain phosphoinositide-interacting protein 2 OS=Mus musculus GN=Wipi2 PE=1 SV=1 - [WIPI2_MOUSE]	17.75	2	4	4	7	0.95	0.92	0.89	1.02	1.06	0.86	0.94	0.90	0.88	1.04	0.98	1.14	1.25	48.45	5.86
Q8BGH2	Sorting and assembly machinery component 50 homolog OS=Mus musculus GN=Samm50 PE=1 SV=1 - [SAM50_MOUSE]	21.11	1	8	8	14	1.07	1.03	0.94	1.20	1.12	0.88	0.85	0.90	0.82	0.94	0.81	1.06	0.97	51.83	6.80
Q8K4K6	Pantothenate kinase 1 OS=Mus musculus GN=Pank1 PE=1 SV=1 - [PANK1_MOUSE]	6.02	2	2	2	4	1.07	0.80	0.75	1.15	1.07	1.04	0.97	0.90	0.84	0.97	0.91	1.02	0.95	60.05	8.03
Q80XC3	USP6 N-terminal-like protein OS=Mus musculus GN=Usp6nl PE=1 SV=2 - [US6NL_MOUSE]	3.54	2	2	2	7	0.89	1.02	1.11	0.95	0.96	0.97	1.01	0.90	1.07	0.91	1.05	0.91	1.06	93.52	9.13
Q99J45	Nuclear receptor-binding protein OS=Mus musculus GN=Nrbp1 PE=1 SV=1 - [NRBP_MOUSE]	6.36	2	2	2	6	0.98	0.97	0.77	0.97	1.03	0.95	0.97	0.90	1.01	0.88	0.90	0.78	0.76	59.83	5.08

O08797	Protein Serpinb9 OS=Mus musculus GN=Serpinb9 PE=2 SV=1 - [O08797_MOUSE]	11.23	6	4	4	7	1.18	0.98	0.82	1.02	0.89	0.83	0.68	0.90	0.76	0.93	0.76	1.02	0.85	42.23	5.40
G3X9N6	Oxysterol-binding protein OS=Mus musculus GN=Osbpl8 PE=3 SV=1 - [G3X9N6_MOUSE]	17.24	4	11	11	29	0.94	0.97	1.06	1.26	1.27	0.92	1.01	0.90	1.00	0.98	1.04	1.08	1.15	96.92	7.55
Q9JIA6	Galactokinase OS=Mus musculus GN=Galk1 PE=2 SV=1 - [Q9JIA6_MOUSE]	9.18	2	3	3	7	0.88	1.18	1.25	1.00	1.13	0.83	0.93	0.90	1.02	1.02	1.16	1.00	1.07	42.27	5.26
Q6P5E8	Diacylglycerol kinase theta OS=Mus musculus GN=Dgkq PE=1 SV=1 - [DGKQ_MOUSE]	7.07	3	6	6	12	1.05	1.31	1.06	1.07	0.96	0.93	0.88	0.90	0.87	0.99	0.94	1.14	1.10	102.19	7.52
P04247	Myoglobin OS=Mus musculus GN=Mb PE=1 SV=3 - [MYG_MOUSE]	20.78	1	2	2	2	0.73	0.74	0.68	0.75	1.02	1.34	1.82	0.90	1.23	0.92	0.85	1.03	0.96	17.06	7.62
Q80TL7-2	Isoform 2 of Protein MON2 homolog OS=Mus musculus GN=Mon2 - [MON2_MOUSE]	1.35	3	2	2	4	0.90	0.67	0.77	1.07	1.16	0.83	0.91	0.90	1.00	0.93	0.97	0.94	1.05	188.16	6.09
O09111	NADH dehydrogenase [ubiquinone] 1 beta subcomplex subunit 11, mitochondrial OS=Mus musculus GN=Ndufb11 PE=1 SV=2 - [NDUBB_MOUSE]	45.03	1	5	5	41	0.93	0.92	0.98	1.02	1.10	0.96	1.02	0.90	0.95	0.99	1.01	0.87	0.98	17.43	5.22
Q3ULD5	Methylcrotonoyl-CoA carboxylase beta chain, mitochondrial OS=Mus musculus GN=Mccc2 PE=2 SV=1 - [MCCB_MOUSE]	22.02	1	8	8	15	0.98	1.06	1.10	1.03	1.05	1.02	0.94	0.90	0.94	1.06	1.04	1.09	1.05	61.34	8.00
P35762	CD81 antigen OS=Mus musculus GN=Cd81 PE=1 SV=2 - [CD81_MOUSE]	27.54	1	3	3	42	1.00	1.05	1.03	1.09	1.05	0.83	0.88	0.90	0.89	0.88	0.88	0.84	0.85	25.80	5.83
Q9WVL0	Maleylacetoacetate isomerase OS=Mus musculus GN=Gstz1 PE=1 SV=1 - [MAAI_MOUSE]	26.39	1	4	4	9	1.11	1.01	0.89	1.27	1.21	1.05	0.97	0.90	0.88	1.12	0.87	0.99	0.96	24.26	7.85
G3X8Y3	N-alpha-acetyltransferase 15, NatA auxiliary subunit OS=Mus musculus GN=Naa15 PE=4 SV=1 - [G3X8Y3_MOUSE]	10.98	4	9	9	12	1.00	0.97	1.02	1.07	1.08	0.93	0.94	0.90	0.93	0.94	0.96	1.04	1.04	101.03	7.52
Q8JZR6-2	Isoform 2 of Electroneutral sodium bicarbonate exchanger 1 OS=Mus musculus GN=Slc4a8 - [S4A8_MOUSE]	13.40	2	8	9	18	0.90	0.94	1.00	1.04	1.12	0.77	0.85	0.90	1.06	0.83	0.94	0.88	0.94	116.61	6.83

B1ASU0	Interleukin-1 receptor accessory protein-like 1 OS=Mus musculus GN=Il1rapl1 PE=4 SV=1 - [B1ASU0_MOUSE]	13.07	3	7	7	25	0.97	1.13	1.22	1.14	1.15	0.89	0.92	0.90	0.96	0.94	0.99	0.95	0.93	79.73	6.32
Q9JM52	Misshapen-like kinase 1 OS=Mus musculus GN=Mink1 PE=1 SV=3 - [MINK1_MOUSE]	24.39	9	16	20	69	0.97	1.05	1.05	0.92	0.98	0.85	0.91	0.90	0.92	0.92	0.97	0.94	1.01	147.20	7.44
Q921F2	TAR DNA-binding protein 43 OS=Mus musculus GN=Tardbp PE=1 SV=1 - [TADBP_MOUSE]	19.81	4	1	6	12	0.77	1.01	1.30	0.83	1.07	0.73	0.93	0.90	1.17	0.85	1.10	0.97	1.25	44.52	6.70
Q91XD6	Vacuolar protein-sorting-associated protein 36 OS=Mus musculus GN=Vps36 PE=1 SV=1 - [VPS36_MOUSE]	16.06	1	6	6	12	0.93	1.04	1.13	1.05	1.08	0.95	0.91	0.90	0.92	0.98	1.01	1.01	1.12	43.71	7.15
E9QLP0	Tubulin polyglutamylase complex subunit 2 OS=Mus musculus GN=Tpgs2 PE=2 SV=1 - [E9QLP0_MOUSE]	7.09	2	1	1	5	0.84	0.69	0.82	1.01	1.17	0.87	1.00	0.90	1.08	0.85	1.01	0.82	0.95	33.21	7.24
Q99KI0	Aconitate hydratase, mitochondrial OS=Mus musculus GN=Aco2 PE=1 SV=1 - [ACON_MOUSE]	59.49	1	43	43	378	0.96	0.97	1.03	1.04	1.08	0.94	0.98	0.90	0.97	0.96	0.99	0.95	1.00	85.41	7.93
P35585	AP-1 complex subunit mu-1 OS=Mus musculus GN=Ap1m1 PE=1 SV=3 - [AP1M1_MOUSE]	35.46	4	12	12	29	0.98	1.10	1.08	0.99	0.97	0.88	0.88	0.90	0.92	0.91	0.90	1.00	0.94	48.51	7.30
Q3TY99	Transducin-like enhancer protein 3 OS=Mus musculus GN=Tle3 PE=2 SV=1 - [Q3TY99_MOUSE]	2.39	4	1	1	2	1.00	1.28	1.27	0.95	0.95	0.96	0.96	0.91	0.90	0.92	0.92	1.06	1.07	76.45	7.24
F8VPK5	Rho-associated protein kinase 2 OS=Mus musculus GN=Rock2 PE=2 SV=1 - [F8VPK5_MOUSE]	42.44	3	43	48	144	0.92	1.00	1.10	0.96	1.01	0.86	0.94	0.91	1.02	0.86	0.96	0.87	0.93	160.51	5.99
O88342	WD repeat-containing protein 1 OS=Mus musculus GN=Wdr1 PE=1 SV=3 - [WDR1_MOUSE]	47.69	1	22	22	118	0.97	0.93	0.94	0.95	0.98	0.89	0.88	0.91	0.89	0.88	0.88	0.92	0.96	66.36	6.60
Q9Z0P5-2	Isoform 2 of Twinfilin-2 OS=Mus musculus GN=Twf2 - [TWF2_MOUSE]	42.36	2	9	9	48	1.04	0.90	0.92	1.04	1.03	0.88	0.91	0.91	0.89	0.83	0.83	0.85	0.87	39.29	6.70

Q8R3B1	1-phosphatidylinositol 4,5-bisphosphate phosphodiesterase delta 1 OS=Mus musculus GN=Plcd1 PE=2 SV=2 - [PLCD1_MOUSE]	15.34	1	8	8	24	0.90	1.05	1.21	1.03	1.12	0.88	0.94	0.91	0.98	0.93	0.96	0.97	1.04	85.82	6.21
Q99PU8-2	Isoform 2 of Putative ATP-dependent RNA helicase DHX30 OS=Mus musculus GN=Dhx30 - [DHX30_MOUSE]	9.88	7	10	10	16	0.98	1.06	1.11	1.10	1.12	0.97	0.99	0.91	0.91	0.99	1.03	1.03	1.10	133.98	8.82
Q80UP3	Diacylglycerol kinase zeta OS=Mus musculus GN=Dgkz PE=1 SV=2 - [DGKZ_MOUSE]	17.98	4	12	12	24	0.95	0.95	1.04	0.90	0.98	0.93	0.96	0.91	1.00	0.89	0.94	0.89	0.97	103.97	8.09
F6RJC1	Teneurin-3 (Fragment) OS=Mus musculus GN=Tenm3 PE=4 SV=1 - [F6RJC1_MOUSE]	2.21	5	4	4	7	1.13	1.16	1.07	1.12	0.98	0.96	0.89	0.91	0.80	1.01	0.89	1.05	0.93	262.60	6.09
Q8K358	Phosphatidylinositol glycan anchor biosynthesis class U protein OS=Mus musculus GN=Pigu PE=2 SV=4 - [PIGU_MOUSE]	2.30	2	1	1	2	1.04	0.95	0.91	1.19	1.14	0.83	0.79	0.91	0.87	0.93	0.90	1.01	0.97	49.77	7.72
Q9EQP2	EH domain-containing protein 4 OS=Mus musculus GN=Ehd4 PE=1 SV=1 - [EHD4_MOUSE]	26.99	1	9	11	18	0.99	0.97	0.93	1.07	1.04	1.00	0.97	0.91	0.89	1.09	1.05	1.10	1.02	61.44	6.76
Q9WV60	Glycogen synthase kinase-3 beta OS=Mus musculus GN=Gsk3b PE=1 SV=2 - [GSK3B_MOUSE]	38.10	2	7	10	31	1.01	1.04	1.00	1.05	1.02	0.97	0.92	0.91	0.94	1.05	1.00	1.06	1.01	46.68	8.78
E0CZ69	FYVE, RhoGEF and PH domain-containing protein 4 (Fragment) OS=Mus musculus GN=Fgd4 PE=2 SV=1 - [E0CZ69_MOUSE]	4.83	6	2	2	2	0.92	1.26	1.26	0.92	0.99	0.94	1.02	0.91	0.98	0.98	1.06	0.87	0.87	43.71	5.22
Q71LX4	Talin-2 OS=Mus musculus GN=Tln2 PE=1 SV=3 - [TLN2_MOUSE]	29.18	2	41	48	91	0.97	1.07	1.07	1.10	1.14	0.95	0.97	0.91	0.95	1.00	1.03	1.08	1.11	253.46	5.80
Q6VNB8	WD repeat and FYVE domain-containing protein 3 OS=Mus musculus GN=Wdfy3 PE=1 SV=1 - [WDFY3_MOUSE]	2.68	4	7	7	13	0.93	1.13	1.14	1.06	1.13	0.87	1.00	0.91	1.04	0.97	1.13	1.05	1.25	392.09	6.80
Q61411	GTPase HRas OS=Mus musculus GN=Hras1 PE=1 SV=2 - [RASH_MOUSE]	66.67	5	5	10	51	1.06	0.99	0.94	1.10	1.01	0.97	0.94	0.91	0.88	0.92	0.85	0.88	0.89	21.28	5.31

Q8BR88	Probable phospholipid-transporting ATPase IA OS=Mus musculus GN=Atp8a1 PE=2 SV=1 - [Q8BR88_MOUSE]	24.57	8	23	25	77	1.17	1.22	1.04	1.17	0.97	0.94	0.80	0.91	0.76	0.99	0.85	1.07	0.90	131.33	6.84
Q9Z0Y1	Dynactin subunit 3 OS=Mus musculus GN=Dctn3 PE=2 SV=2 - [DCTN3_MOUSE]	23.66	2	4	4	10	0.84	1.01	1.10	0.94	1.07	0.93	1.08	0.91	1.02	0.92	1.01	0.91	1.07	20.97	6.06
Q9EPR4	Solute carrier family 23 member 2 OS=Mus musculus GN=Slc23a2 PE=1 SV=2 - [S23A2_MOUSE]	10.65	3	5	5	13	0.91	0.89	0.99	1.13	1.24	0.96	0.99	0.91	0.96	1.01	1.08	0.92	1.03	70.00	7.50
O54865	Guanylate cyclase soluble subunit beta-1 OS=Mus musculus GN=Gucy1b3 PE=2 SV=1 - [GCYB1_MOUSE]	24.52	1	13	13	32	0.97	1.03	1.08	1.12	1.12	0.85	0.85	0.91	0.95	0.96	0.98	1.05	1.06	70.55	5.31
Q7TT37	Elongator complex protein 1 OS=Mus musculus GN=Ikbkap PE=2 SV=2 - [ELP1_MOUSE]	5.10	1	5	5	8	0.98	1.10	1.11	1.16	1.09	0.82	0.70	0.91	0.89	0.98	0.93	1.05	1.15	149.49	6.00
P30416	Peptidyl-prolyl cis-trans isomerase FKBP4 OS=Mus musculus GN=Fkbp4 PE=1 SV=5 - [FKBP4_MOUSE]	23.36	4	9	9	43	0.97	1.04	1.08	0.97	0.97	0.89	0.93	0.91	0.90	0.94	0.93	0.95	0.96	51.54	5.72
Q8BLF1	Neutral cholesterol ester hydrolase 1 OS=Mus musculus GN=Nceh1 PE=1 SV=1 - [NCEH1_MOUSE]	20.59	2	8	8	21	0.95	1.06	1.05	1.11	1.15	0.92	0.92	0.91	0.86	0.88	0.95	0.94	0.99	45.71	7.05
O35409	Glutamate carboxypeptidase 2 OS=Mus musculus GN=Folh1 PE=2 SV=2 - [FOLH1_MOUSE]	15.96	2	8	8	14	1.10	0.98	0.96	1.08	0.96	0.87	0.82	0.91	0.82	0.95	0.84	0.92	0.85	84.52	8.10
O35598	Disintegrin and metalloproteinase domain-containing protein 10 OS=Mus musculus GN=Adam10 PE=1 SV=2 - [ADA10_MOUSE]	10.28	2	6	6	13	1.06	1.10	1.03	1.05	1.03	0.87	0.81	0.91	0.88	0.89	0.85	1.01	0.98	83.91	7.94
Q9CWG8-	Isoform 2 of NADH dehydrogenase [ubiquinone] complex I, assembly factor 7 OS=Mus musculus GN=Ndufaf7 - [NDUF7_MOUSE]	7.05	2	2	2	4	0.96	0.91	0.95	1.02	1.06	0.93	0.97	0.91	0.94	0.91	0.95	1.00	1.05	42.50	6.99
Q9EQF6	Dihydropyrimidinase-related protein 5 OS=Mus musculus GN=Dpysl5 PE=1 SV=1 - [DPYL5_MOUSE]	53.37	2	19	19	54	1.09	1.03	1.02	1.05	0.98	0.99	0.95	0.91	0.90	1.01	0.95	1.06	1.02	61.48	7.09

P49442	Inositol polyphosphate 1-phosphatase OS=Mus musculus GN=Inpp1 PE=1 SV=2 - [INPP_MOUSE]	27.27	4	8	8	21	0.89	0.85	0.99	1.04	1.13	0.98	1.11	0.91	1.07	0.97	1.19	0.91	1.09	43.32	5.01
E9QNG1	Intersectin-2 OS=Mus musculus GN=Itsn2 PE=2 SV=1 - [E9QNG1_MOUSE]	13.51	6	13	18	45	0.95	0.96	1.06	0.94	0.99	0.89	0.96	0.91	1.03	0.88	0.92	0.93	0.97	188.66	8.25
G3UYD0	General transcription factor II-I OS=Mus musculus GN=Gtf2i PE=2 SV=1 - [G3UYD0_MOUSE]	12.27	14	8	8	9	0.96	0.97	1.01	1.01	1.02	0.98	0.98	0.91	0.99	0.97	1.05	0.97	1.00	103.01	8.81
Q8BXL9-	Isoform 3 of Intermediate filament family orphan 1 OS=Mus musculus GN=Iffo1 - [IFFO1_MOUSE]	4.35	8	1	1	4	1.18	0.89	0.75	1.03	0.87	0.87	0.73	0.91	0.77	1.07	0.90	0.86	0.73	42.19	4.61
Q3UGX2	Spectrin beta 1 OS=Mus musculus GN=Sptb PE=2 SV=1 - [Q3UGX2_MOUSE]	28.34	6	42	49	134	0.91	1.00	1.08	1.05	1.16	0.93	1.00	0.91	1.00	0.96	1.08	1.02	1.18	267.93	5.49
Q3TWV4	AP-2 complex subunit mu OS=Mus musculus GN=Ap2m1 PE=2 SV=1 - [Q3TWV4_MOUSE]	43.65	2	20	20	92	1.00	1.05	1.06	1.14	1.14	0.87	0.85	0.91	0.87	0.98	0.97	1.00	1.01	49.36	9.54
Q9CQW1	Synaptobrevin homolog YKT6 OS=Mus musculus GN=Ykt6 PE=2 SV=1 - [YKT6_MOUSE]	58.59	1	10	10	19	1.00	0.96	0.92	1.08	1.08	0.95	0.97	0.91	0.95	0.92	1.02	0.91	0.92	22.30	6.35
Q60738	Zinc transporter 1 OS=Mus musculus GN=Slc30a1 PE=2 SV=1 - [ZNT1_MOUSE]	12.92	1	4	4	6	1.03	0.88	0.85	1.06	1.03	0.92	0.85	0.91	0.86	0.97	0.90	0.96	0.96	54.68	6.62
E9Q585	Protein Slc4a1ap OS=Mus musculus GN=Slc4a1ap PE=2 SV=1 - [E9Q585_MOUSE]	13.71	3	6	6	10	0.90	0.66	0.76	0.86	0.95	0.98	1.12	0.91	0.99	0.95	1.05	0.84	0.92	79.62	5.10
Q9CYH5	Glucose-fructose oxidoreductase domain-containing protein 2 OS=Mus musculus GN=Gfod2 PE=2 SV=1 - [GFOD2_MOUSE]	8.83	1	1	1	1	1.54	1.07	0.70	1.23	0.80	1.41	0.92	0.91	0.59	1.37	0.89	0.79	0.52	42.21	6.25
Q3UHI4	Protein TMED8 OS=Mus musculus GN=Tmed8 PE=1 SV=1 - [TMED8_MOUSE]	18.71	1	3	3	9	0.96	0.89	0.97	0.94	0.97	0.88	0.92	0.91	1.01	0.93	0.98	1.01	1.07	35.76	4.74
Q8BGN8	Synaptoporin OS=Mus musculus GN=Synpr PE=1 SV=1 - [SYNPR_MOUSE]	14.34	3	3	4	8	0.99	0.99	1.00	1.01	1.13	0.88	0.82	0.91	0.94	0.89	0.91	0.99	1.00	29.21	6.30
Q9Z2I4-2	Isoform 2 of Roundabout homolog 3 OS=Mus musculus GN=Robo3 - [ROBO3_MOUSE]	3.50	4	1	2	2	1.21	0.90	0.75	0.90	0.74	0.98	0.81	0.91	0.75	1.14	0.95	1.18	0.98	143.35	7.03

F8VQF9	Trafficking protein particle complex subunit 10 OS=Mus musculus GN=Trappc10 PE=4 SV=1 - [F8VQF9_MOUSE]	3.58	2	3	3	4	0.92	1.15	1.28	1.02	1.11	0.87	0.90	0.91	0.96	1.03	1.01	0.99	1.08	141.40	5.92
B7ZWL1	CCR4-NOT transcription complex subunit 1 OS=Mus musculus GN=Cnot1 PE=2 SV=1 - [B7ZWL1_MOUSE]	3.65	4	8	8	16	0.96	0.92	0.89	1.14	1.18	0.97	0.98	0.91	0.91	0.99	1.02	1.12	1.05	260.79	7.02
Q9D6Z1	Nucleolar protein 56 OS=Mus musculus GN=Nop56 PE=1 SV=2 - [NOP56_MOUSE]	20.00	8	9	9	19	1.02	1.09	0.98	1.06	1.05	0.92	1.01	0.91	0.94	0.97	0.96	0.97	0.95	64.42	9.14
Q9QZH6	Evolutionarily conserved signaling intermediate in Toll pathway, mitochondrial OS=Mus musculus GN=Ecsit PE=1 SV=2 - [ECSIT_MOUSE]	6.90	2	3	3	5	1.02	1.22	1.15	1.16	1.11	0.94	0.93	0.91	0.89	0.91	0.92	1.04	1.01	49.77	6.60
Q8BH44	Coronin-2B OS=Mus musculus GN=Coro2b PE=2 SV=2 - [COR2B_MOUSE]	35.00	4	13	13	24	0.95	0.89	1.00	1.00	1.08	0.96	0.97	0.91	0.98	0.89	0.90	0.88	0.99	54.90	8.27
O89017	Legumain OS=Mus musculus GN=Lgmn PE=1 SV=1 - [LGMN_MOUSE]	18.62	1	6	6	15	0.89	0.86	0.90	0.81	0.92	0.86	0.99	0.91	0.98	0.79	0.85	0.73	0.84	49.34	6.39
Q9CQE7	Endoplasmic reticulum-Golgi intermediate compartment protein 3 OS=Mus musculus GN=Ergic3 PE=2 SV=1 - [ERGI3_MOUSE]	8.09	4	3	3	5	0.98	1.24	1.27	1.15	1.15	0.89	0.92	0.91	0.92	0.94	0.95	0.97	0.87	43.18	6.47
P61079	Ubiquitin-conjugating enzyme E2 D3 OS=Mus musculus GN=Ube2d3 PE=1 SV=1 - [UB2D3_MOUSE]	40.82	3	1	4	17	0.86	0.98	1.13	0.98	1.13	0.96	1.10	0.91	1.05	0.96	1.11	0.87	1.01	16.68	7.80
Q6NXH3-	Isoform 3 of Vasculin OS=Mus musculus GN=Gpbp1 - [GPBP1_MOUSE]	13.59	3	3	3	4	0.86	0.76	0.88	0.85	0.99	1.06	1.22	0.91	1.05	0.95	1.11	0.82	0.95	41.28	5.91
G3UXL2	Ribose-phosphate pyrophosphokinase OS=Mus musculus GN=Prps1l3 PE=2 SV=1 - [G3UXL2_MOUSE]	34.59	2	6	9	19	1.05	1.11	1.06	1.08	0.93	0.92	0.85	0.91	0.85	1.04	0.94	1.30	1.19	34.80	6.98
O55013	Trafficking protein particle complex subunit 3 OS=Mus musculus GN=Trappc3 PE=1 SV=1 - [TPPC3_MOUSE]	24.44	1	6	6	13	1.08	1.19	1.15	0.90	0.98	0.85	0.83	0.91	0.77	0.93	0.87	0.95	0.92	20.29	4.96

E9Q175	Unconventional myosin-VI OS=Mus musculus GN=Myo6 PE=2 SV=1 - [E9Q175_MOUSE]	15.40	5	16	16	30	0.96	0.98	1.06	1.05	1.09	0.91	0.95	0.91	0.99	0.96	1.00	1.01	1.12	144.68	8.51
Q9JI19	Acidic fibroblast growth factor intracellular-binding protein OS=Mus musculus GN=Fibp PE=2 SV=1 - [FIBP_MOUSE]	2.24	2	1	1	1	0.99	1.09	1.10	1.11	1.13	1.05	1.06	0.91	0.91	1.13	1.14	1.03	1.04	41.18	6.76
Q9Z268	RasGAP-activating-like protein 1 OS=Mus musculus GN=Rasal1 PE=2 SV=2 - [RASL1_MOUSE]	28.41	2	15	15	48	1.01	1.20	1.23	1.21	1.13	0.99	0.97	0.91	0.92	1.03	1.04	1.13	1.13	89.34	6.37
O09172	Glutamate--cysteine ligase regulatory subunit OS=Mus musculus GN=Gclm PE=2 SV=1 - [GSH0_MOUSE]	26.64	3	5	5	12	0.90	1.08	1.18	1.18	1.21	0.97	0.94	0.91	0.93	1.11	1.22	1.06	1.19	30.52	5.52
Q61809	Leucine-rich repeat neuronal protein 1 OS=Mus musculus GN=Lrrn1 PE=2 SV=1 - [LRRN1_MOUSE]	2.09	1	1	1	2	1.48	1.32	0.89	1.20	0.81	0.91	0.61	0.91	0.61	1.16	0.78	1.24	0.84	80.50	6.15
Q9CQJ1	HIG1 domain family member 2A OS=Mus musculus GN=Higd2a PE=2 SV=1 - [HIG2A_MOUSE]	19.81	1	1	1	1	0.72	0.50	0.68	0.88	1.21	0.53	0.73	0.91	1.25	0.75	1.03	0.54	0.75	11.36	9.57
Q8K0D5	Elongation factor G, mitochondrial OS=Mus musculus GN=Gfm1 PE=2 SV=1 - [EFGM_MOUSE]	15.98	2	9	9	13	0.94	0.91	0.99	1.01	1.10	0.91	0.93	0.91	0.94	0.87	0.98	0.97	0.99	83.50	6.92
F8WHV1	Mitotic spindle-associated MMXD complex subunit MIP18 OS=Mus musculus GN=Fam96b PE=2 SV=1 - [F8WHV1_MOUSE]	31.62	2	2	2	5	0.89	0.93	0.89	0.96	0.94	1.16	1.31	0.91	1.02	1.05	1.30	1.14	1.43	14.99	5.45
Q02257	Junction plakoglobin OS=Mus musculus GN=Jup PE=1 SV=3 - [PLAK_MOUSE]	12.08	1	4	7	16	0.92	1.04	1.11	1.30	1.48	0.85	0.85	0.91	0.84	0.88	1.01	1.04	1.20	81.75	6.14
B9EJI9	Transmembrane protein 229A OS=Mus musculus GN=Tmem229a PE=2 SV=1 - [T229A_MOUSE]	3.23	1	1	1	2	1.01	1.20	1.18	1.21	1.20	0.91	0.89	0.91	0.90	0.96	0.95	0.98	0.97	41.50	9.35
E0CYX9	Coiled-coil domain-containing protein 127 (Fragment) OS=Mus musculus GN=Ccdc127 PE=2 SV=1 - [E0CYX9_MOUSE]	15.55	4	3	3	6	1.01	1.20	1.17	1.11	1.10	0.82	0.79	0.91	0.88	1.03	1.01	1.17	1.15	27.72	8.76

Q6P6P5	Slc12a6 protein OS=Mus musculus GN=Gm21985 PE=2 SV=1 - [Q6P6P5_MOUSE]	13.65	5	7	11	42	1.04	0.99	0.87	0.97	0.98	0.97	0.91	0.91	0.86	0.93	0.87	0.82	0.76	122.59	7.49
P20352	Tissue factor OS=Mus musculus GN=F3 PE=1 SV=2 - [TF_MOUSE]	5.78	1	1	1	3	0.86	0.94	1.09	0.85	0.98	0.95	1.10	0.91	1.05	1.00	1.16	0.84	0.98	32.91	9.32
Q9DBL1	Short/branched chain specific acyl-CoA dehydrogenase, mitochondrial OS=Mus musculus GN=Acadsb PE=1 SV=1 - [ACDSB_MOUSE]	15.97	2	5	5	10	1.03	1.06	1.22	1.12	1.03	0.80	0.81	0.91	0.76	0.94	0.92	1.03	0.99	47.84	7.87
P98203-4	Isoform 4 of Armadillo repeat protein deleted in velo-cardio-facial syndrome homolog OS=Mus musculus GN=Arvcf - [ARVC_MOUSE]	10.76	5	5	5	11	1.02	1.10	1.16	1.09	1.02	0.93	0.92	0.91	0.98	0.97	1.03	0.99	1.03	97.11	6.79
B1ARV6	Protein Gm13125 OS=Mus musculus GN=Gm13125 PE=4 SV=1 - [B1ARV6_MOUSE]	3.56	1	1	1	1	0.64	0.53	0.84	0.92	1.45	0.88	1.38	0.91	1.43	0.87	1.37	0.75	1.19	55.07	8.34
P10852	4F2 cell-surface antigen heavy chain OS=Mus musculus GN=Slc3a2 PE=1 SV=1 - [4F2_MOUSE]	37.45	2	18	18	81	1.01	1.05	0.93	1.12	1.06	0.94	0.84	0.91	0.90	0.96	0.93	0.96	0.90	58.30	5.91
F8WHJ1	FTS and Hook-interacting protein OS=Mus musculus GN=Fam160a2 PE=2 SV=1 - [F8WHJ1_MOUSE]	10.45	6	6	6	11	0.89	1.12	1.26	1.06	1.08	1.00	1.09	0.91	1.02	1.06	1.07	1.13	1.28	99.09	6.44
Q8CIB5	Fermitin family homolog 2 OS=Mus musculus GN=Fermt2 PE=1 SV=1 - [FERM2_MOUSE]	18.38	2	8	9	18	0.98	1.11	1.12	1.04	1.02	0.93	0.95	0.91	0.84	0.92	0.85	0.98	0.92	77.75	6.70
P63168	Dynein light chain 1, cytoplasmic OS=Mus musculus GN=Dynll1 PE=1 SV=1 - [DYL1_MOUSE]	67.42	2	3	7	73	0.91	0.82	0.97	0.86	0.92	0.85	0.91	0.91	0.99	0.85	0.81	0.85	0.97	10.36	7.40
Q9QYY8	Spastin OS=Mus musculus GN=Spast PE=2 SV=3 - [SPAST_MOUSE]	5.21	1	2	2	4	0.91	1.04	1.15	0.94	1.03	0.90	0.99	0.91	1.00	0.87	0.96	1.06	1.16	66.41	9.69
P80313	T-complex protein 1 subunit eta OS=Mus musculus GN=Cct7 PE=1 SV=1 - [TCPH_MOUSE]	31.62	1	14	14	40	0.99	0.87	0.90	1.11	1.10	0.92	0.93	0.91	0.90	0.96	0.98	1.00	1.02	59.61	7.84
E9Q0M7	Protein Atp11b OS=Mus musculus GN=Atp11b PE=2 SV=1 - [E9Q0M7_MOUSE]	2.38	3	1	2	4	1.16	1.13	0.97	1.24	1.06	0.89	0.76	0.91	0.78	1.33	1.15	1.14	0.98	95.26	6.24

P63321	Ras-related protein Ral-A OS=Mus musculus GN=Rala PE=1 SV=1 - [RALA_MOUSE]	51.94	1	3	8	112	1.06	0.86	0.80	1.06	1.01	0.96	0.90	0.91	0.87	0.89	0.84	0.85	0.77	23.54	7.11
E0CXE3	Contactin-6 OS=Mus musculus GN=Cntn6 PE=2 SV=1 - [E0CXE3_MOUSE]	3.45	3	2	2	4	1.02	1.05	1.03	1.04	1.03	0.97	0.95	0.91	0.89	1.00	0.98	1.01	0.99	105.45	6.58
Q3TDK6	Protein rogdi homolog OS=Mus musculus GN=Rogdi PE=2 SV=2 - [ROGDI_MOUSE]	47.74	3	10	10	33	0.99	1.05	1.03	1.03	1.05	0.86	0.85	0.91	0.94	0.94	0.92	0.99	0.97	32.08	8.18
Q8BU88	39S ribosomal protein L22, mitochondrial OS=Mus musculus GN=Mrpl22 PE=2 SV=1 - [RM22_MOUSE]	5.83	1	1	1	2	0.89	1.04	1.16	0.82	0.92	0.81	0.90	0.91	1.01	0.84	0.94	0.85	0.95	23.79	9.86
Q9DC51	Guanine nucleotide-binding protein G(k) subunit alpha OS=Mus musculus GN=Gnai3 PE=1 SV=3 - [GNAI3_MOUSE]	40.68	6	5	11	72	1.04	1.24	1.10	1.17	1.08	0.90	0.89	0.91	0.84	1.07	1.00	1.00	0.99	40.51	5.69
Q9EPR2-2	Isoform 2 of Group XIIA secretory phospholipase A2 OS=Mus musculus GN=Pla2g12a - [PG12A_MOUSE]	9.80	2	1	1	1	0.76	0.96	1.26	0.75	0.98	0.87	1.14	0.91	1.19	0.73	0.96	0.76	1.00	17.19	6.49
Q9QZD9	Eukaryotic translation initiation factor 3 subunit I OS=Mus musculus GN=Eif3i PE=1 SV=1 - [EIF3I_MOUSE]	20.92	2	7	7	18	0.94	0.86	0.91	0.98	1.01	0.94	0.96	0.91	0.95	0.87	0.95	0.93	0.96	36.44	5.64
Q9CQF9	Prenylcysteine oxidase OS=Mus musculus GN=Pcyox1 PE=1 SV=1 - [PCYOX_MOUSE]	30.30	3	10	10	28	1.13	0.97	0.89	1.08	0.98	0.90	0.82	0.91	0.82	0.89	0.81	0.96	0.86	56.46	6.92
Q6PIC6	Sodium/potassium-transporting ATPase subunit alpha-3 OS=Mus musculus GN=Atp1a3 PE=1 SV=1 - [AT1A3_MOUSE]	62.69	5	44	70	2117	1.02	1.05	1.01	1.10	1.06	0.91	0.89	0.91	0.89	0.97	0.93	0.97	0.96	111.62	5.41
Q9CQA5	Mediator of RNA polymerase II transcription subunit 4 OS=Mus musculus GN=Med4 PE=2 SV=1 - [MED4_MOUSE]	8.52	2	2	2	4	0.83	0.88	1.06	0.81	0.97	0.87	1.03	0.91	1.09	0.78	0.94	0.97	1.16	29.76	5.05
F6Y616	Polyadenylate-binding protein-interacting protein 1 OS=Mus musculus GN=Paip1 PE=2 SV=2 - [F6Y616_MOUSE]	27.89	5	3	10	32	0.93	0.81	0.92	0.97	1.04	0.92	0.99	0.91	1.02	0.88	1.02	0.79	0.91	53.93	4.78

Q9DCU6	39S ribosomal protein L4, mitochondrial OS=Mus musculus GN=Mrpl4 PE=2 SV=1 - [RM04_MOUSE]	3.74	1	1	1	2	0.90	0.90	1.00	1.17	1.29	1.09	1.21	0.91	1.01	0.95	1.05	0.86	0.95	33.05	9.82
Q810B6	Ankyrin repeat and FYVE domain-containing protein 1 OS=Mus musculus GN=Ankfy1 PE=2 SV=2 - [ANFY1_MOUSE]	4.53	2	4	4	5	0.89	0.93	1.05	1.00	1.01	0.91	1.00	0.91	0.97	1.01	1.17	1.01	1.08	128.57	5.91
B1AXI2	Rho guanine nucleotide exchange factor 9 OS=Mus musculus GN=Arhgef9 PE=2 SV=1 - [B1AXI2_MOUSE]	7.25	7	3	3	6	0.97	1.12	1.13	1.08	1.10	0.98	0.98	0.91	1.01	1.01	1.09	1.04	1.04	49.38	7.03
Q8BYR5-	Isoform 6 of Calcium-dependent secretion activator 2 OS=Mus musculus GN=Cadps2 - [CAPS2_MOUSE]	9.11	11	3	6	14	0.93	1.05	1.07	1.09	1.17	0.90	0.91	0.91	0.98	0.87	0.94	0.95	1.02	87.41	6.77
D3Z2Q2	Syntaxin-binding protein 5 OS=Mus musculus GN=Stxbp5 PE=2 SV=1 - [D3Z2Q2_MOUSE]	22.47	5	16	16	73	1.06	1.01	0.90	1.11	1.04	0.94	0.89	0.91	0.90	0.97	0.94	1.00	0.94	121.63	6.96
E9QNH6	Unconventional myosin-Ib OS=Mus musculus GN=Myo1b PE=2 SV=1 - [E9QNH6_MOUSE]	5.29	6	4	4	8	0.99	0.99	0.98	1.07	1.18	1.04	0.99	0.91	0.88	1.02	0.91	1.04	1.05	124.95	9.14
H3BJU7	Rho guanine nucleotide exchange factor 2 OS=Mus musculus GN=Arhgef2 PE=2 SV=1 - [H3BJU7_MOUSE]	31.17	17	22	22	52	0.95	1.05	1.16	1.05	1.17	0.93	1.02	0.91	0.99	0.95	1.02	1.00	1.03	108.52	6.87
Q9Z1G4-3	Isoform A1-III of V-type proton ATPase 116 kDa subunit a isoform 1 OS=Mus musculus GN=Atp6v0a1 - [VPP1_MOUSE]	29.57	12	23	24	199	1.01	1.05	1.07	1.08	1.09	0.86	0.86	0.91	0.92	0.95	0.93	0.92	0.95	95.56	6.77
Q07797	Galectin-3-binding protein OS=Mus musculus GN=Lgals3bp PE=1 SV=1 - [LG3BP_MOUSE]	4.51	2	2	2	5	0.89	0.95	1.05	0.79	0.87	0.77	0.85	0.91	1.03	0.74	0.84	0.67	0.74	64.45	5.14
Q9CQ22	Ragulator complex protein LAMTOR1 OS=Mus musculus GN=Lamtor1 PE=1 SV=1 - [LTOR1_MOUSE]	29.81	1	3	3	10	1.05	1.16	0.97	0.94	0.81	1.10	0.94	0.91	0.86	0.98	0.82	0.94	0.84	17.74	5.15
F8VPN4	Protein Agl OS=Mus musculus GN=Agl PE=4 SV=1 - [F8VPN4_MOUSE]	13.19	5	14	14	23	1.12	1.11	1.04	1.11	1.04	0.94	0.91	0.91	0.85	0.95	0.91	1.03	0.98	174.18	6.74

O55033	Cytoplasmic protein NCK2 OS=Mus musculus GN=Nck2 PE=1 SV=1 - [NCK2_MOUSE]	52.37	1	12	14	37	0.94	0.93	0.98	0.87	0.97	0.85	0.93	0.91	0.95	0.82	0.89	0.80	0.88	42.85	6.95
Q9CQZ5	NADH dehydrogenase [ubiquinone] 1 alpha subcomplex subunit 6 OS=Mus musculus GN=Ndufa6 PE=1 SV=1 - [NDUA6_MOUSE]	45.04	1	6	6	12	0.94	0.87	0.89	1.05	1.12	0.92	0.98	0.91	0.97	0.96	1.03	0.98	1.04	15.27	10.11
Q8K273	Membrane magnesium transporter 1 OS=Mus musculus GN=Mmgt1 PE=2 SV=1 - [MMGT1_MOUSE]	18.32	1	1	1	1	0.83	0.71	0.86	1.18	1.42	0.96	1.16	0.91	1.10	0.82	1.00	0.90	1.10	14.67	9.16
Q61093	Cytochrome b-245 heavy chain OS=Mus musculus GN=Cybb PE=2 SV=1 - [CY24B_MOUSE]	2.63	1	1	1	2	1.45	1.25	0.86	1.27	0.88	1.41	0.96	0.91	0.63	1.10	0.76	1.33	0.92	65.26	7.72
A2AE27	AMP deaminase 2 OS=Mus musculus GN=Ampd2 PE=4 SV=1 - [A2AE27_MOUSE]	11.41	5	9	9	14	1.02	0.99	1.01	0.95	0.90	0.99	0.89	0.91	0.82	0.99	0.92	1.06	0.98	94.64	6.51
B1AXC8	Coiled-coil-helix-coiled-coil-helix domain-containing protein 7 (Fragment) OS=Mus musculus GN=Chchd7 PE=2 SV=1 - [B1AXC8_MOUSE]	64.56	4	1	5	17	0.77	0.90	1.15	0.83	1.07	0.95	1.22	0.91	1.17	0.96	1.25	0.97	1.25	9.32	8.19
O35239	Tyrosine-protein phosphatase non-receptor type 9 OS=Mus musculus GN=Ptpn9 PE=2 SV=2 - [PTN9_MOUSE]	1.85	1	1	1	2	0.92	1.18	1.28	1.02	1.12	0.86	0.93	0.91	0.99	1.02	1.12	1.04	1.14	67.93	8.10
Q8VCW8	Acyl-CoA synthetase family member 2, mitochondrial OS=Mus musculus GN=Acsf2 PE=2 SV=1 - [ACSF2_MOUSE]	21.79	1	11	11	24	0.96	0.90	0.94	1.00	1.04	0.93	0.89	0.91	0.98	1.00	1.02	1.01	1.12	67.91	8.18
P35802	Neuronal membrane glycoprotein M6-a OS=Mus musculus GN=Gpm6a PE=1 SV=1 - [GPM6A_MOUSE]	17.63	1	7	7	32	1.06	1.12	1.10	1.15	1.07	0.97	0.92	0.91	0.89	0.93	0.88	1.03	1.03	31.13	5.27
Q8CBY8-	Isoform 2 of Dynactin subunit 4 OS=Mus musculus GN=Dctn4 - [DCTN4_MOUSE]	42.61	2	12	12	23	1.09	1.00	1.00	1.04	1.03	0.91	0.84	0.91	0.86	0.96	0.92	1.02	1.00	52.21	7.69

Q8BPM0-	Isoform 2 of Disheveled-associated activator of morphogenesis 1 OS=Mus musculus GN=Daam1 - [DAAM1_MOUSE]	7.96	6	6	8	17	0.90	1.06	1.17	1.15	1.26	0.91	0.97	0.91	0.95	1.01	1.06	0.98	1.14	122.20	7.46
D3YUM1	NADH dehydrogenase [ubiquinone] flavoprotein 1, mitochondrial OS=Mus musculus GN=Ndufv1 PE=2 SV=1 - [D3YUM1_MOUSE]	52.09	6	17	17	56	0.97	1.03	1.06	1.03	1.07	0.93	0.97	0.91	0.91	0.95	1.00	1.05	1.06	49.88	8.07
Q9ESN6	Tripartite motif-containing protein 2 OS=Mus musculus GN=Trim2 PE=1 SV=1 - [TRIM2_MOUSE]	36.96	7	19	22	68	1.00	0.78	0.78	0.94	0.93	0.89	0.85	0.91	0.87	0.84	0.84	0.87	0.84	81.39	6.96
Q6P5B5	Fragile X mental retardation syndrome-related protein 2 OS=Mus musculus GN=Fxr2 PE=2 SV=1 - [Q6P5B5_MOUSE]	26.71	2	8	12	29	0.90	1.10	1.17	0.91	1.01	0.95	1.05	0.91	0.93	0.94	1.02	0.84	0.98	74.21	6.23
Q6GQT9	Nodal modulator 1 OS=Mus musculus GN=Nomo1 PE=1 SV=1 - [NOMO1_MOUSE]	17.13	1	14	14	37	1.03	1.04	1.03	1.02	0.98	0.86	0.85	0.91	0.87	0.89	0.87	0.91	0.87	133.34	6.09
Q62074	Protein kinase C iota type OS=Mus musculus GN=Prkci PE=1 SV=3 - [KPCI_MOUSE]	1.68	1	1	1	1	0.92	1.18	1.28	1.13	1.23	1.03	1.11	0.91	0.99	1.06	1.16	0.99	1.08	68.16	5.85
B1AXJ6	Low-density lipoprotein receptor-related protein 8 OS=Mus musculus GN=Lrp8 PE=2 SV=1 - [B1AXJ6_MOUSE]	5.19	7	2	2	5	0.97	1.07	1.11	0.91	0.83	0.93	0.85	0.91	0.83	1.00	0.92	0.88	0.81	77.24	5.00
Q9Z1Z0	General vesicular transport factor p115 OS=Mus musculus GN=Uso1 PE=1 SV=2 - [USO1_MOUSE]	29.20	4	21	21	51	0.95	0.93	0.97	1.01	1.06	0.90	0.92	0.91	0.96	0.96	1.01	0.93	0.97	106.92	4.93
B1AX58	Plastin-3 OS=Mus musculus GN=Pls3 PE=2 SV=1 - [B1AX58_MOUSE]	31.77	2	13	18	47	1.00	0.85	0.86	1.05	1.10	0.99	1.01	0.91	0.99	0.99	0.96	0.95	0.94	71.70	5.62
Q8VBY2	Calcium/calmodulin-dependent protein kinase kinase 1 OS=Mus musculus GN=Camkk1 PE=1 SV=1 - [KKCC1_MOUSE]	24.95	2	9	9	18	0.96	0.97	0.90	1.12	0.99	0.94	0.85	0.91	0.94	0.91	0.88	1.09	0.93	55.80	5.99
F6QYT9	Kalirin (Fragment) OS=Mus musculus GN=Kalrn PE=4 SV=1 - [F6QYT9_MOUSE]	7.89	16	10	15	22	0.88	1.15	1.26	1.02	1.05	0.96	1.06	0.91	0.97	0.87	0.98	0.90	0.97	270.59	6.11

Q9QXW9	Large neutral amino acids transporter small subunit 2 OS=Mus musculus GN=Slc7a8 PE=1 SV=1 - [LAT2_MOUSE]	1.69	1	1	1	2	1.27	1.02	0.80	1.20	0.94	0.98	0.77	0.91	0.71	0.92	0.72	1.12	0.89	57.84	6.67
Q9JME5	AP-3 complex subunit beta-2 OS=Mus musculus GN=Ap3b2 PE=1 SV=2 - [AP3B2_MOUSE]	25.88	2	20	22	54	0.98	1.05	1.08	1.10	1.14	0.87	0.89	0.91	0.94	0.95	0.97	1.02	1.08	119.12	5.63
Q3TIR3	Synembryn-A OS=Mus musculus GN=Ric8a PE=1 SV=2 - [RIC8A_MOUSE]	10.00	1	3	3	6	0.81	1.17	1.34	1.11	1.37	0.95	1.16	0.91	1.03	0.95	1.18	1.11	1.27	59.81	5.68
O35841	Apoptosis inhibitor 5 OS=Mus musculus GN=Api5 PE=2 SV=2 - [API5_MOUSE]	11.11	1	4	4	10	1.00	1.02	1.07	0.89	1.10	0.96	0.92	0.91	0.94	0.91	1.01	0.95	0.98	56.75	5.92
Q9R1R8	Retinol dehydrogenase 11 OS=Mus musculus GN=Rdh11 PE=2 SV=1 - [Q9R1R8_MOUSE]	7.00	2	2	2	5	0.93	0.79	0.86	1.17	1.20	0.95	0.91	0.91	0.97	1.03	1.00	1.00	1.00	33.22	8.79
Q9Z103	Activity-dependent neuroprotector homeobox protein OS=Mus musculus GN=Adnp PE=2 SV=1 - [ADNP_MOUSE]	3.99	2	2	2	7	1.06	1.42	1.14	0.84	0.79	1.07	0.92	0.91	0.86	1.03	0.91	0.99	0.91	92.01	6.06
Q8BVQ5	Protein phosphatase methylesterase 1 OS=Mus musculus GN=Ppme1 PE=1 SV=5 - [PPME1_MOUSE]	32.12	1	12	12	23	0.94	0.91	0.90	1.09	1.14	0.97	1.01	0.91	0.97	0.93	1.00	0.94	0.96	42.23	5.97
H3BJ77	FUN14 domain containing 1, isoform CRA_b OS=Mus musculus GN=Fundc1 PE=4 SV=1 - [H3BJ77_MOUSE]	15.18	2	1	1	2	1.10	0.92	0.83	1.21	1.09	0.96	0.86	0.91	0.82	0.96	0.87	1.07	0.97	12.04	9.66
P14576	Signal recognition particle 54 kDa protein OS=Mus musculus GN=Srp54 PE=1 SV=2 - [SRP54_MOUSE]	13.89	2	5	5	9	0.96	0.83	0.84	1.09	1.07	0.96	1.00	0.91	0.90	0.99	0.99	0.98	0.98	55.68	8.75
P28867	Protein kinase C delta type OS=Mus musculus GN=Prkcd PE=1 SV=3 - [KPCD_MOUSE]	20.33	10	9	10	22	0.89	0.87	1.11	0.98	1.20	0.93	1.15	0.91	1.00	0.87	0.99	0.85	0.98	77.50	7.39
Q8BVG8	N-acetyltransferase 14 OS=Mus musculus GN=Nat14 PE=2 SV=1 - [NAT14_MOUSE]	26.70	1	4	4	8	0.93	0.81	0.98	1.18	1.28	0.85	0.91	0.91	0.97	1.05	1.12	1.00	1.14	21.76	10.74

Q3UHL1	CaM kinase-like vesicle-associated protein OS=Mus musculus GN=Camkv PE=1 SV=2 - [CAMKV_MOUSE]	60.55	1	26	26	92	1.03	1.02	1.01	1.08	1.00	0.93	0.89	0.91	0.87	0.99	0.94	1.01	0.96	54.79	5.54
Q810A3	Tetratricopeptide repeat protein 9C OS=Mus musculus GN=Ttc9c PE=2 SV=1 - [TTC9C_MOUSE]	12.28	2	2	2	5	1.17	0.88	0.97	1.05	0.86	1.06	1.04	0.91	1.01	1.02	1.00	1.07	1.07	19.99	9.29
P0C913	Overexpressed in colon carcinoma 1 protein homolog OS=Mus musculus PE=3 SV=1 - [OCC1_MOUSE]	55.56	1	2	2	4	0.99	0.68	0.71	0.82	0.68	1.01	0.84	0.91	0.92	0.95	0.90	0.77	0.78	6.45	6.57
O35943	Frataxin, mitochondrial OS=Mus musculus GN=Fxn PE=1 SV=1 - [FRDA_MOUSE]	19.81	1	3	3	6	1.21	0.73	0.63	0.93	0.83	1.16	0.82	0.91	0.77	1.07	0.85	0.92	0.82	22.91	8.02
Q5RJH3	Cadherin-12 OS=Mus musculus GN=Cdh12 PE=2 SV=1 - [CAD12_MOUSE]	1.51	1	1	1	1	1.20	1.27	1.05	0.91	0.76	0.96	0.80	0.91	0.76	0.87	0.73	0.67	0.56	88.41	4.83
P47802	Metaxin-1 OS=Mus musculus GN=Mtx1 PE=1 SV=1 - [MTX1_MOUSE]	18.30	8	5	5	9	1.04	1.32	1.16	1.19	1.09	0.81	0.78	0.91	0.83	0.96	0.86	1.02	0.98	35.60	6.18
D3YYW1	Transmembrane 9 superfamily member 1 (Fragment) OS=Mus musculus GN=Tm9sf1 PE=2 SV=1 - [D3YYW1_MOUSE]	10.17	6	1	1	2	1.07	1.15	1.07	1.07	1.00	0.91	0.84	0.91	0.85	0.95	0.88	1.04	0.96	13.50	7.44
O54774	AP-3 complex subunit delta-1 OS=Mus musculus GN=Ap3d1 PE=1 SV=1 - [AP3D1_MOUSE]	22.77	1	17	17	45	0.95	0.96	1.03	1.10	1.03	0.92	0.94	0.91	0.87	0.91	0.90	0.99	0.97	135.00	7.37
P35123	Ubiquitin carboxyl-terminal hydrolase 4 OS=Mus musculus GN=Usp4 PE=1 SV=3 - [UBP4_MOUSE]	8.32	1	5	7	15	0.90	1.12	1.12	1.03	1.12	0.93	1.03	0.91	0.91	0.87	0.89	0.95	0.99	108.27	5.64
Q91W29	Cytochrome c oxidase subunit 4 isoform 2, mitochondrial OS=Mus musculus GN=Cox4i2 PE=2 SV=1 - [COX42_MOUSE]	6.40	1	1	1	3	0.87	0.75	0.86	0.87	1.00	0.94	1.07	0.91	1.04	0.91	1.04	0.77	0.88	20.19	9.47
Q8CIG8	Protein arginine N-methyltransferase 5 OS=Mus musculus GN=Prmt5 PE=1 SV=3 - [ANM5_MOUSE]	18.21	2	9	9	16	1.07	1.08	0.94	1.14	1.07	0.95	0.90	0.91	0.85	0.99	0.90	1.08	0.92	72.63	6.42
Q9R0S3	Matrix metalloproteinase-17 OS=Mus musculus GN=Mmp17 PE=1 SV=3 - [MMP17_MOUSE]	15.22	1	4	4	10	1.10	1.02	0.97	0.89	0.75	1.07	1.03	0.91	0.96	1.04	1.01	1.09	1.21	64.29	6.38

Q60929-2	Isoform 2 of Myocyte-specific enhancer factor 2A OS=Mus musculus GN=Mef2a - [MEF2A_MOUSE]	11.22	4	2	3	6	1.00	1.14	1.14	1.04	1.03	0.95	1.05	0.91	0.91	1.00	0.99	0.89	0.89	52.59	8.69
Q8BUY8	G-protein coupled receptor-associated sorting protein 2 OS=Mus musculus GN=Gprasp2 PE=2 SV=2 - [GASP2_MOUSE]	6.30	1	4	4	14	1.20	1.25	1.22	0.96	0.90	0.92	0.82	0.91	0.82	0.96	0.80	1.09	0.92	92.74	5.11
B1AQZ0	Septin-8 OS=Mus musculus GN=Sept8 PE=2 SV=1 - [B1AQZ0_MOUSE]	44.63	5	2	19	95	0.93	0.86	0.91	1.04	1.11	0.95	0.98	0.91	1.13	0.98	1.09	0.97	1.05	55.84	6.10
P54830	Tyrosine-protein phosphatase non-receptor type 5 OS=Mus musculus GN=Ptpn5 PE=2 SV=2 - [PTN5_MOUSE]	17.19	4	6	6	10	1.03	1.13	1.04	1.25	1.09	1.01	0.97	0.91	0.94	1.13	1.04	1.23	1.15	60.78	5.20
Q61578	NADPH:adrenodoxin oxidoreductase, mitochondrial OS=Mus musculus GN=Fdxr PE=2 SV=1 - [ADRO_MOUSE]	12.35	1	4	4	4	0.88	1.32	1.35	1.09	1.25	0.98	1.12	0.91	1.08	1.07	1.14	1.01	1.15	54.17	8.66
Q99LG1	Transmembrane protein 51 OS=Mus musculus GN=Tmem51 PE=1 SV=1 - [TMM51_MOUSE]	19.68	1	2	2	3	1.08	1.00	0.93	0.80	0.75	0.99	0.92	0.91	0.85	1.32	1.23	1.27	1.18	27.38	6.95
Q68EF8	Rap guanine nucleotide exchange factor-like 1 OS=Mus musculus GN=Rapgefl1 PE=2 SV=2 - [RPGFL_MOUSE]	9.67	1	3	3	8	0.82	0.81	1.07	0.95	1.14	0.83	0.94	0.91	1.09	0.92	1.13	0.91	0.97	73.65	6.21
P31750	RAC-alpha serine/threonine-protein kinase OS=Mus musculus GN=Akt1 PE=1 SV=2 - [AKT1_MOUSE]	11.46	4	3	5	14	1.02	1.00	0.98	1.04	1.00	1.02	0.97	0.91	0.90	0.92	0.93	0.94	0.92	55.67	5.90
E9QAN4	Kinesin-like protein KIF1A OS=Mus musculus GN=Kif1a PE=2 SV=1 - [E9QAN4_MOUSE]	21.20	7	19	27	51	0.98	0.87	0.99	1.03	1.05	0.90	0.94	0.91	0.88	0.96	1.03	0.95	1.00	190.86	6.19
Q9Z0J4-2	Isoform N-NOS-2 of Nitric oxide synthase, brain OS=Mus musculus GN=Nos1 - [NOS1_MOUSE]	15.79	6	14	14	24	1.20	1.18	1.05	1.08	0.93	0.94	0.86	0.91	0.76	1.03	0.87	1.23	1.08	148.34	7.43
Q8R2R9	AP-3 complex subunit mu-2 OS=Mus musculus GN=Ap3m2 PE=2 SV=1 - [AP3M2_MOUSE]	24.40	1	7	8	17	0.94	0.99	1.06	1.16	1.24	0.90	0.85	0.91	0.98	0.84	1.01	0.99	1.02	46.89	7.56
Q9D8Y1	Transmembrane protein 126A OS=Mus musculus GN=Tmem126a PE=2 SV=1 - [T126A_MOUSE]	23.98	1	2	3	4	0.90	0.86	0.96	0.97	1.08	0.93	1.03	0.91	1.01	1.01	1.12	0.88	0.98	21.53	9.41

Q80X98	DEAH (Asp-Glu-Ala-His) box polypeptide 38 OS=Mus musculus GN=Dhx38 PE=2 SV=1 - [Q80X98_MOUSE]	3.50	1	3	3	5	1.21	1.08	1.26	0.80	1.03	1.16	1.40	0.91	1.04	1.62	2.35	1.43	0.98	140.54	6.49
Q5U5V2-2	Isoform 2 of Hydroxylysine kinase OS=Mus musculus GN=Agphd1 - [AGPD1_MOUSE]	7.98	2	1	1	2	1.10	1.05	0.95	1.12	1.02	0.92	0.83	0.91	0.83	0.90	0.82	1.41	1.28	17.81	6.32
Q80UM5	NEL-like 2 (Chicken) OS=Mus musculus GN=Nell2 PE=2 SV=1 - [Q80UM5_MOUSE]	17.09	2	12	12	34	1.00	1.01	1.04	0.95	0.99	0.85	0.85	0.92	0.93	0.83	0.83	0.85	0.84	91.37	5.82
Q02053	Ubiquitin-like modifier-activating enzyme 1 OS=Mus musculus GN=Uba1 PE=1 SV=1 - [UBA1_MOUSE]	47.16	2	33	33	170	0.97	1.04	1.09	1.04	1.10	0.94	0.98	0.92	0.95	0.95	0.99	1.01	1.06	117.73	5.66
Q9R1T4-2	Isoform I of Septin-6 OS=Mus musculus GN=Sept6 - [SEPT6_MOUSE]	56.91	4	11	21	126	0.95	0.75	0.84	0.92	0.96	0.89	0.90	0.92	0.92	0.86	0.90	0.87	0.90	48.73	6.55
Q8R104	NAD-dependent protein deacetylase sirtuin-3 OS=Mus musculus GN=Sirt3 PE=2 SV=1 - [SIR3_MOUSE]	11.28	3	2	2	4	0.94	0.97	1.03	1.03	1.10	0.84	0.89	0.92	0.97	0.92	0.98	0.86	0.92	28.80	6.23
Q9EPL8	Importin-7 OS=Mus musculus GN=Ipo7 PE=1 SV=2 - [IPO7_MOUSE]	6.17	1	5	5	16	0.91	1.04	1.11	1.16	1.26	0.83	0.94	0.92	1.02	0.94	1.00	1.00	1.08	119.41	4.82
E9Q242	Adenylosuccinate lyase OS=Mus musculus GN=Adsl PE=2 SV=1 - [E9Q242_MOUSE]	17.27	4	6	6	19	0.97	1.19	1.08	1.15	1.21	0.97	0.92	0.92	0.85	1.04	1.05	1.15	1.17	53.10	7.27
P47754	F-actin-capping protein subunit alpha-2 OS=Mus musculus GN=Capza2 PE=1 SV=3 - [CAZA2_MOUSE]	63.29	2	11	13	87	1.04	0.95	0.93	0.99	0.97	0.92	0.89	0.92	0.92	0.89	0.89	0.97	0.96	32.95	5.85
Q8BGR6	ADP-ribosylation factor-like protein 15 OS=Mus musculus GN=Arl15 PE=2 SV=1 - [ARL15_MOUSE]	24.51	1	4	4	6	0.89	0.80	0.84	0.95	1.06	1.05	1.01	0.92	1.04	0.82	0.99	0.86	0.93	22.89	5.29
Q80TM9-3	Isoform 3 of Nischarin OS=Mus musculus GN=Nisch - [NISCH_MOUSE]	3.71	10	4	4	8	1.17	0.95	0.89	1.14	1.04	0.99	0.86	0.92	0.83	1.04	0.92	1.10	1.00	147.52	4.98
Q62108-3	Isoform 3 of Disks large homolog 4 OS=Mus musculus GN=Dlg4 - [DLG4_MOUSE]	46.05	9	22	27	96	0.98	1.00	1.05	1.16	1.18	0.89	0.89	0.92	0.94	0.90	0.92	0.92	0.98	80.05	5.92
Q9JII5-2	Isoform 2 of DAZ-associated protein 1 OS=Mus musculus GN=Dazap1 - [DAZP1_MOUSE]	27.90	4	7	7	39	0.95	0.79	0.88	0.88	1.01	0.84	0.89	0.92	0.97	0.76	0.75	0.82	0.87	43.06	8.56

Q64299	Protein NOV homolog OS=Mus musculus GN=Nov PE=2 SV=1 - [NOV_MOUSE]	22.32	1	7	7	12	1.15	1.03	0.89	1.05	0.87	1.21	1.03	0.92	0.91	0.99	0.92	0.98	0.87	38.90	7.64
Q3UXL4	Centrosomal protein kizuna OS=Mus musculus GN=Plk1s1 PE=1 SV=1 - [KIZ_MOUSE]	4.03	1	2	2	3	1.04	0.77	0.73	0.99	0.94	1.07	1.02	0.92	0.87	0.98	0.93	0.94	0.91	76.49	5.62
P12660	Purkinje cell protein 2 OS=Mus musculus GN=Pcp2 PE=2 SV=2 - [PCP2_MOUSE]	30.83	2	2	2	3	1.23	0.97	0.79	1.26	1.02	1.26	1.02	0.92	0.74	1.50	1.22	1.62	1.32	13.05	5.14
Q8VDM4	26S proteasome non-ATPase regulatory subunit 2 OS=Mus musculus GN=Psmd2 PE=1 SV=1 - [PSMD2_MOUSE]	37.67	2	26	26	56	0.99	0.99	0.99	1.05	1.07	0.95	0.90	0.92	0.94	1.00	0.99	0.98	0.97	100.14	5.17
Q60597	2-oxoglutarate dehydrogenase, mitochondrial OS=Mus musculus GN=Ogdh PE=1 SV=3 - [ODO1_MOUSE]	45.94	5	29	35	123	0.98	0.96	0.90	1.11	1.07	0.94	0.92	0.92	0.96	0.95	0.94	1.03	1.02	116.37	6.83
Q7TSE6	Serine/threonine-protein kinase 38-like OS=Mus musculus GN=Stk38l PE=1 SV=2 - [ST38L_MOUSE]	7.54	2	3	3	7	0.82	1.00	1.21	1.15	1.37	0.83	1.00	0.92	1.11	0.94	1.15	0.95	1.16	53.74	6.96
Q9WVS4-	Isoform 2 of MAPK/MAK/MRK overlapping kinase OS=Mus musculus GN=Mok - [MOK_MOUSE]	3.93	3	1	1	1	0.83	1.36	1.63	1.00	1.20	0.96	1.15	0.92	1.10	1.06	1.27	1.15	1.38	20.85	9.28
O55029	Coatomer subunit beta' OS=Mus musculus GN=Copb2 PE=2 SV=2 - [COPB2_MOUSE]	22.65	1	12	12	34	1.03	1.02	0.93	1.00	0.99	0.88	0.88	0.92	0.94	0.97	0.95	0.95	0.94	102.38	5.30
Q8R0F5	RNA-binding motif protein, X-linked 2 OS=Mus musculus GN=Rbmx2 PE=1 SV=1 - [RBMX2_MOUSE]	3.99	1	1	1	2	1.01	0.85	0.84	0.81	0.80	0.92	0.91	0.92	0.90	0.74	0.73	0.75	0.74	37.51	9.72
E9QN99	Alpha/beta hydrolase domain-containing protein 14B OS=Mus musculus GN=Abhd14b PE=4 SV=1 - [E9QN99_MOUSE]	21.43	2	4	4	9	1.06	1.19	1.11	0.93	0.91	0.78	0.75	0.92	0.84	0.88	0.85	0.94	0.87	22.44	6.52
A2AQE4	COP9 signalosome complex subunit 2 OS=Mus musculus GN=Cops2 PE=2 SV=1 - [A2AQE4_MOUSE]	38.81	3	11	11	26	1.02	0.88	0.95	1.09	1.07	0.90	0.91	0.92	0.93	0.92	0.93	1.02	0.99	46.88	5.16

H9KUX8	Baculoviral IAP repeat-containing protein 6 OS=Mus musculus GN=Birc6 PE=2 SV=1 - [H9KUX8_MOUSE]	0.99	4	3	3	3	0.82	1.09	1.33	1.10	1.11	0.85	1.06	0.92	1.14	0.94	1.21	1.04	1.26	529.08	6.06
P49935	Pro-cathepsin H OS=Mus musculus GN=Ctsh PE=2 SV=2 - [CATH_MOUSE]	8.41	1	2	2	5	1.25	0.83	0.66	1.04	0.83	1.00	0.85	0.92	0.76	1.01	0.86	0.80	0.67	37.15	8.40
Q5F2E8	Serine/threonine-protein kinase TAO1 OS=Mus musculus GN=Taok1 PE=1 SV=1 - [TAOK1_MOUSE]	14.99	2	11	14	27	0.95	1.08	1.11	0.91	0.98	0.88	0.95	0.92	1.03	0.93	0.95	0.99	1.07	115.98	7.55
Q8K0U4	Heat shock 70 kDa protein 12A OS=Mus musculus GN=Hspa12a PE=1 SV=1 - [HS12A_MOUSE]	52.59	1	29	33	131	1.04	0.98	0.96	1.05	1.01	0.91	0.90	0.92	0.89	0.91	0.91	0.94	0.93	74.82	6.77
G3UZI7	Rho GTPase activating protein 6, isoform CRA_b OS=Mus musculus GN=Arhgap6 PE=4 SV=1 - [G3UZI7_MOUSE]	6.07	7	2	2	4	1.30	0.90	0.69	0.92	0.71	1.08	0.83	0.92	0.70	1.42	1.10	1.55	1.20	53.56	6.74
Q9CWF6	Bardet-Biedl syndrome 2 protein homolog OS=Mus musculus GN=Bbs2 PE=2 SV=1 - [BBS2_MOUSE]	1.80	1	1	1	2	0.89	1.08	1.21	0.90	1.02	0.98	1.10	0.92	1.03	0.99	1.12	1.00	1.12	79.88	5.86
Q9Z1F9	SUMO-activating enzyme subunit 2 OS=Mus musculus GN=Uba2 PE=2 SV=1 - [SAE2_MOUSE]	28.84	4	13	13	32	1.00	1.13	1.09	1.01	1.03	0.99	0.92	0.92	0.93	0.98	1.03	0.97	1.00	70.52	5.24
P26040	Ezrin OS=Mus musculus GN=Ezr PE=1 SV=3 - [EZRI_MOUSE]	42.32	1	15	26	80	0.95	0.78	0.81	0.92	0.98	0.91	1.01	0.92	1.01	0.87	0.94	0.82	0.85	69.36	6.10
P23242	Gap junction alpha-1 protein OS=Mus musculus GN=Gja1 PE=1 SV=2 - [CXA1_MOUSE]	30.10	1	8	8	43	1.03	0.97	1.01	1.05	1.03	0.91	0.96	0.92	0.86	0.97	1.06	1.04	1.11	42.98	8.76
P10126	Elongation factor 1-alpha 1 OS=Mus musculus GN=Eef1a1 PE=1 SV=3 - [EF1A1_MOUSE]	67.97	3	10	20	183	1.04	0.91	0.95	1.03	0.98	0.87	0.90	0.92	0.89	0.94	0.92	0.94	0.99	50.08	9.01
O08581	Potassium channel subfamily K member 1 OS=Mus musculus GN=Kcnk1 PE=1 SV=2 - [KCNK1_MOUSE]	5.65	1	1	1	1	0.97	1.66	1.70	0.81	0.83	0.94	0.96	0.92	0.94	0.72	0.74	0.86	0.88	38.18	6.07
Q8K010	5-oxoprolinase OS=Mus musculus GN=Oplah PE=2 SV=1 - [OPLA_MOUSE]	10.87	3	10	10	18	1.02	1.12	1.05	1.06	1.04	1.00	0.97	0.92	0.91	1.03	1.00	1.11	1.03	137.52	6.28
P14115	60S ribosomal protein L27a OS=Mus musculus GN=Rpl27a PE=2 SV=5 - [RL27A_MOUSE]	8.11	1	1	1	4	1.06	1.06	1.00	1.13	1.06	0.95	0.90	0.92	0.86	1.19	1.12	1.24	1.17	16.60	11.12

B1AWE0	Clathrin light chain A OS=Mus musculus GN=Clta PE=2 SV=1 - [B1AWE0_MOUSE]	33.33	4	1	9	138	1.23	1.09	0.87	1.04	0.85	1.04	0.98	0.92	1.01	1.08	0.91	0.90	0.84	23.46	4.50
O35678	Monoglyceride lipase OS=Mus musculus GN=Mgll PE=1 SV=1 - [MGLL_MOUSE]	35.97	5	9	9	38	0.93	0.96	1.06	1.02	1.12	0.98	1.00	0.92	1.00	0.90	1.01	0.94	1.04	33.37	7.15
Q792F9	Alpha-4 integrin OS=Mus musculus GN=Itga4 PE=2 SV=1 - [Q792F9_MOUSE]	2.81	2	1	1	1	0.76	0.64	0.84	0.73	0.96	0.82	1.07	0.92	1.20	0.74	0.97	0.73	0.97	115.01	6.81
Q99NB1	Acetyl-coenzyme A synthetase 2-like, mitochondrial OS=Mus musculus GN=Acss1 PE=1 SV=1 - [ACS2L_MOUSE]	12.17	1	7	7	14	1.20	1.20	1.06	1.07	0.96	0.97	0.84	0.92	0.81	1.06	0.96	1.13	0.97	74.58	6.98
Q9CWX9	Probable ATP-dependent RNA helicase DDX47 OS=Mus musculus GN=Ddx47 PE=2 SV=2 - [DDX47_MOUSE]	3.52	1	1	1	3	0.93	0.88	0.95	0.98	1.06	0.76	0.81	0.92	0.99	0.89	0.96	0.87	0.94	50.61	9.10
Q80UI5	Phosphatidylinositol 3-kinase regulatory subunit alpha OS=Mus musculus GN=Pik3r1 PE=2 SV=1 - [Q80UI5_MOUSE]	8.37	2	4	4	7	0.86	1.06	1.14	0.93	1.11	0.87	0.97	0.92	1.00	0.80	0.94	0.85	1.00	53.37	6.44
Q6NSR8	Probable aminopeptidase NPEPL1 OS=Mus musculus GN=Npepl1 PE=2 SV=1 - [PEPL1_MOUSE]	26.91	2	8	9	15	1.06	0.81	0.84	0.85	0.88	0.94	0.87	0.92	0.85	0.94	0.88	0.75	0.73	55.90	6.84
Q9R1C6	Diacylglycerol kinase epsilon OS=Mus musculus GN=Dgke PE=2 SV=1 - [DGKE_MOUSE]	14.54	2	7	7	19	0.99	1.11	1.10	0.93	0.95	0.87	0.85	0.92	0.90	0.91	0.93	0.96	1.04	63.59	7.44
F8VQC9	NBCn1-G OS=Mus musculus GN=Slc4a7 PE=2 SV=1 - [F8VQC9_MOUSE]	6.28	3	2	6	16	1.04	1.22	1.17	1.08	1.04	0.88	0.84	0.92	0.88	1.13	1.09	1.02	0.98	127.14	6.44
Q8BHJ7	Gamma-aminobutyric acid receptor subunit alpha-5 OS=Mus musculus GN=Gabra5 PE=2 SV=1 - [GBRA5_MOUSE]	10.80	1	2	4	10	1.00	0.97	0.96	1.12	1.12	1.16	1.15	0.92	0.91	0.92	0.92	0.92	0.92	52.24	9.01
O88792	Junctional adhesion molecule A OS=Mus musculus GN=F11r PE=1 SV=2 - [JAM1_MOUSE]	8.67	1	2	2	4	1.09	1.00	0.92	1.11	1.03	0.82	0.75	0.92	0.84	0.90	0.83	0.93	0.86	32.40	6.77
Q8BMD2-	Isoform 3 of Zinc finger protein DZIP1 OS=Mus musculus GN=Dzip1 - [DZIP1_MOUSE]	2.24	4	1	2	4	0.73	0.56	0.77	0.55	0.76	0.97	1.33	0.92	1.26	1.02	1.41	0.55	0.76	71.02	5.76

Q9QZ06	Toll-interacting protein OS=Mus musculus GN=Tollip PE=1 SV=1 - [TOLIP_MOUSE]	35.77	4	8	8	29	0.93	0.93	1.05	0.95	1.00	0.93	0.98	0.92	1.01	0.92	0.97	0.96	0.95	30.32	5.17
Q4VA93	Protein kinase C OS=Mus musculus GN=Prkca PE=2 SV=1 - [Q4VA93_MOUSE]	39.58	7	14	22	68	1.00	0.96	0.99	1.04	1.11	0.96	0.95	0.92	1.00	0.95	1.00	0.87	0.91	76.77	7.05
A2ANL9	Teneurin-1 OS=Mus musculus GN=Tenm1 PE=2 SV=1 - [A2ANL9_MOUSE]	3.78	3	5	7	15	1.05	1.12	1.04	1.13	1.13	0.98	0.90	0.92	0.87	1.05	1.02	0.99	0.92	304.66	6.32
P14131	40S ribosomal protein S16 OS=Mus musculus GN=Rps16 PE=2 SV=4 - [RS16_MOUSE]	37.67	1	6	6	14	0.92	0.95	1.05	1.00	1.08	1.00	0.99	0.92	0.95	0.93	1.03	0.93	1.02	16.44	10.21
Q925J9-4	Isoform 4 of Mediator of RNA polymerase II transcription subunit 1 OS=Mus musculus GN=Med1 - [MED1_MOUSE]	2.18	5	2	2	3	0.95	0.88	0.93	0.94	0.99	1.00	1.05	0.92	0.97	0.92	0.97	0.85	0.89	165.35	8.78
Q9JKY7	Cytochrome P450 CYP2D22 OS=Mus musculus GN=Cyp2d22 PE=2 SV=1 - [Q9JKY7_MOUSE]	5.00	4	2	2	2	0.89	1.29	1.44	0.95	1.06	1.02	1.13	0.92	1.02	1.06	1.19	1.02	1.14	56.43	6.98
P11438	Lysosome-associated membrane glycoprotein 1 OS=Mus musculus GN=Lamp1 PE=1 SV=2 - [LAMP1_MOUSE]	12.07	1	4	4	15	1.17	1.07	0.91	1.04	0.93	1.02	0.89	0.92	0.79	0.96	0.83	0.95	0.85	43.84	8.40
Q3TKD0	Transportin-1 (Fragment) OS=Mus musculus GN=Tnpo1 PE=2 SV=1 - [Q3TKD0_MOUSE]	3.84	3	1	2	19	1.03	1.25	1.22	1.01	1.07	0.92	0.91	0.92	0.91	1.13	1.09	0.96	0.98	91.68	4.92
Q8CAQ8-	Isoform 2 of Mitochondrial inner membrane protein OS=Mus musculus GN=Immt - [IMMT_MOUSE]	57.77	4	3	37	130	0.99	0.93	0.96	1.06	1.07	0.87	0.90	0.92	0.96	0.88	0.91	0.80	0.79	82.88	6.61
Q69ZA1-2	Isoform 2 of Cyclin-dependent kinase 13 OS=Mus musculus GN=Cdk13 - [CDK13_MOUSE]	2.27	50	1	2	5	1.34	1.91	1.41	1.01	0.75	1.12	0.83	0.92	0.68	1.64	1.22	1.11	0.83	158.04	9.70
P28740-2	Isoform 2 of Kinesin-like protein KIF2A OS=Mus musculus GN=Kif2a - [KIF2A_MOUSE]	29.89	3	1	18	38	0.87	1.37	1.57	1.23	1.41	1.16	1.32	0.92	1.05	1.36	1.56	1.44	1.65	74.80	6.46
Q9QUG9	RAS guanyl-releasing protein 2 OS=Mus musculus GN=Rasgrp2 PE=1 SV=2 - [GRP2_MOUSE]	4.61	2	2	2	3	0.89	0.72	0.81	1.38	1.54	0.99	1.10	0.92	1.03	1.06	1.19	1.36	1.53	69.40	7.66

Q9DB27	Malignant T-cell-amplified sequence 1 OS=Mus musculus GN=Mcts1 PE=2 SV=1 - [MCTS1_MOUSE]	46.96	3	6	6	15	1.00	0.81	0.81	1.04	1.02	0.98	0.93	0.92	0.89	0.92	0.85	1.00	0.98	20.54	8.82
E9PZ88	Alpha-mannosidase 2C1 OS=Mus musculus GN=Man2c1 PE=2 SV=1 - [E9PZ88_MOUSE]	11.81	5	7	7	10	0.99	0.90	0.93	1.08	1.05	0.83	0.96	0.92	0.97	0.89	1.01	0.84	0.91	104.61	6.46
Q9R1V6-1	Isoform 12 of Disintegrin and metalloproteinase domain-containing protein 22 OS=Mus musculus GN=Adam22 - [ADA22_MOUSE]	40.12	34	30	30	81	0.99	1.03	1.10	1.06	1.11	0.91	0.92	0.92	0.91	0.95	0.97	0.92	0.94	106.54	7.90
Q8CHX7	Raftlin-2 OS=Mus musculus GN=Rftn2 PE=2 SV=3 - [RFTN2_MOUSE]	28.20	2	9	9	22	1.11	1.16	1.08	1.09	1.01	0.92	0.91	0.92	0.85	1.03	0.93	0.94	0.93	54.94	5.50
Q9Z2X1-2	Isoform 2 of Heterogeneous nuclear ribonucleoprotein F OS=Mus musculus GN=Hnrnpf - [HNRPF_MOUSE]	25.82	6	5	7	33	0.78	0.68	0.87	0.84	1.04	0.94	1.26	0.92	1.26	0.90	1.07	0.75	0.96	43.66	5.48
Q80W54	CAAX prenyl protease 1 homolog OS=Mus musculus GN=Zmpste24 PE=1 SV=2 - [FACE1_MOUSE]	6.74	2	2	2	5	1.00	0.91	0.82	0.94	0.99	0.89	0.97	0.92	0.92	0.94	0.94	1.11	1.11	54.70	6.95
Q8JZY2	COMM domain-containing protein 10 OS=Mus musculus GN=Commd10 PE=2 SV=1 - [COMDA_MOUSE]	9.90	1	2	2	5	1.03	0.82	0.81	1.06	1.07	0.93	0.92	0.92	0.93	0.94	0.91	0.85	0.83	22.80	6.65
Q91W53	Golgin subfamily A member 7 OS=Mus musculus GN=Golga7 PE=2 SV=1 - [GOGA7_MOUSE]	8.03	2	1	1	2	0.88	0.94	1.06	0.86	0.98	0.92	1.04	0.92	1.04	0.89	1.01	0.85	0.97	15.78	7.05
Q69Z26	Contactin-4 OS=Mus musculus GN=Cntn4 PE=2 SV=2 - [CNTN4_MOUSE]	16.28	6	11	12	21	0.99	0.94	1.04	1.03	1.09	0.97	1.03	0.92	0.94	0.93	0.96	0.94	1.02	113.42	7.36
E9Q1W7	TBC domain-containing protein kinase-like protein OS=Mus musculus GN=Tbck PE=2 SV=1 - [E9Q1W7_MOUSE]	3.81	3	3	3	11	0.86	1.02	1.31	1.08	1.22	0.91	0.98	0.92	1.01	0.90	1.06	1.06	1.26	100.58	6.43
Q8K2X8	General transcription factor IIH subunit 5 OS=Mus musculus GN=Gtf2h5 PE=1 SV=1 - [TF2H5_MOUSE]	36.62	1	2	2	4	0.87	0.75	0.86	0.82	0.94	1.02	1.17	0.92	1.05	0.80	0.92	0.80	0.92	8.03	4.65

O88746-2	Isoform 2 of Target of Myb protein 1 OS=Mus musculus GN=Tom1 - [TOM1_MOUSE]	67.86	1	1	7	18	1.00	0.99	0.99	1.06	1.07	0.87	0.87	0.92	0.92	1.01	1.01	1.01	1.02	17.78	4.78
Q99N50-7	Isoform 7 of Synaptotagmin-like protein 2 OS=Mus musculus GN=Sytl2 - [SYTL2_MOUSE]	3.92	10	1	1	1	0.96	1.70	1.76	0.86	0.89	0.73	0.76	0.92	0.95	0.73	0.76	0.96	1.00	29.55	9.36
Q9D554	Splicing factor 3A subunit 3 OS=Mus musculus GN=Sf3a3 PE=2 SV=2 - [SF3A3_MOUSE]	17.76	1	7	7	13	0.93	1.04	1.01	0.87	0.90	1.02	1.10	0.92	1.07	0.92	0.90	1.00	1.06	58.81	5.34
Q62421	Endophilin-A3 OS=Mus musculus GN=Sh3gl3 PE=2 SV=1 - [SH3G3_MOUSE]	41.79	5	2	11	31	0.78	0.71	1.15	0.89	1.45	1.14	1.70	0.92	1.16	0.92	1.12	1.05	1.63	38.91	5.12
F2Z3X6	3-phosphoinositide-dependent protein kinase 1 OS=Mus musculus GN=Pdpk1 PE=2 SV=1 - [F2Z3X6_MOUSE]	18.61	5	7	7	12	1.02	1.00	0.95	1.06	1.04	0.94	0.95	0.92	0.91	1.03	1.00	1.12	1.07	60.90	7.39
Q641K1-2	Isoform 2 of Cytosolic carboxypeptidase 1 OS=Mus musculus GN=Agtpbp1 - [CBPC1_MOUSE]	4.94	6	5	5	7	0.95	1.06	1.12	1.05	1.19	0.87	0.98	0.92	1.08	1.03	1.17	0.93	1.06	132.14	7.53
O54991	Contactin-associated protein 1 OS=Mus musculus GN=Cntnap1 PE=1 SV=2 - [CNTP1_MOUSE]	21.59	1	23	23	63	1.02	1.19	1.17	1.09	1.05	0.90	0.88	0.92	0.92	0.94	0.92	1.01	1.06	156.21	7.06
E9Q8N8	Electrogenic sodium bicarbonate cotransporter 1 OS=Mus musculus GN=Slc4a4 PE=2 SV=1 - [E9Q8N8_MOUSE]	19.93	5	3	14	59	1.05	1.35	1.03	1.02	0.91	0.87	0.76	0.92	0.87	0.87	0.80	0.92	0.76	123.01	6.89
Q8R555	Cartilage acidic protein 1 OS=Mus musculus GN=Crtac1 PE=2 SV=1 - [CRAC1_MOUSE]	16.10	1	7	7	11	1.10	1.12	1.18	1.02	1.01	0.93	0.90	0.92	0.81	0.92	0.89	1.02	1.01	70.28	5.14
P51175	Protoporphyrinogen oxidase OS=Mus musculus GN=Ppox PE=2 SV=1 - [PPOX_MOUSE]	2.52	1	1	1	2	0.98	1.26	1.28	1.09	1.11	0.99	1.01	0.92	0.93	1.11	1.13	1.09	1.11	50.84	8.84
P21447	Multidrug resistance protein 1A OS=Mus musculus GN=Abcb1a PE=1 SV=3 - [MDR1A_MOUSE]	14.18	5	13	13	21	0.97	1.13	1.10	1.11	1.13	0.91	0.93	0.92	0.96	0.99	1.04	0.85	0.89	140.56	8.85
B1AYD3	Synapse differentiation-inducing gene protein 1 (Fragment) OS=Mus musculus GN=Syndig1 PE=2 SV=1 - [B1AYD3_MOUSE]	41.10	5	2	2	3	0.94	2.22	2.37	1.00	1.06	0.81	0.86	0.92	0.98	0.89	0.95	1.12	1.20	7.86	8.63

O88705	Potassium/sodium hyperpolarization-activated cyclic nucleotide-gated channel 3 OS=Mus musculus GN=Hcn3 PE=1 SV=1 - [HCN3_MOUSE]	3.08	1	2	2	3	1.12	1.26	1.13	0.95	0.85	1.01	0.90	0.92	0.82	1.19	1.06	1.17	1.05	86.59	9.86
Q5SVG5	AP-1 complex subunit beta-1 OS=Mus musculus GN=Ap1b1 PE=2 SV=1 - [Q5SVG5_MOUSE]	45.74	3	16	38	116	1.02	0.98	1.02	1.17	1.03	0.96	0.91	0.92	0.91	0.93	0.90	1.06	0.99	101.13	5.22
Q8R3F5	Malonyl-CoA-acyl carrier protein transacylase, mitochondrial OS=Mus musculus GN=Mcat PE=2 SV=3 - [FABD_MOUSE]	10.50	1	3	3	5	0.88	1.03	1.17	1.02	1.16	0.89	0.89	0.92	1.04	0.88	1.00	0.90	1.03	41.90	8.10
P97370	Sodium/potassium-transporting ATPase subunit beta-3 OS=Mus musculus GN=Atp1b3 PE=1 SV=1 - [AT1B3_MOUSE]	39.21	1	7	7	16	0.99	1.01	1.01	1.10	1.13	0.86	0.95	0.92	0.91	0.89	0.93	0.96	0.91	31.76	8.51
P63001	Ras-related C3 botulinum toxin substrate 1 OS=Mus musculus GN=Rac1 PE=1 SV=1 - [RAC1_MOUSE]	35.94	10	4	8	30	1.00	0.83	0.83	1.07	1.16	1.00	1.03	0.92	0.94	0.89	0.92	0.88	0.91	21.44	8.50
P48318	Glutamate decarboxylase 1 OS=Mus musculus GN=Gad1 PE=2 SV=2 - [DCE1_MOUSE]	25.46	3	10	11	36	1.08	1.22	0.96	1.15	1.01	0.99	0.85	0.92	0.83	1.01	0.88	1.05	0.92	66.61	7.17
Q9Z1A1	Protein Tfg OS=Mus musculus GN=Tfg PE=2 SV=1 - [Q9Z1A1_MOUSE]	40.05	7	13	13	73	0.95	0.95	0.94	0.92	0.94	0.89	0.91	0.92	0.94	0.85	0.90	0.87	0.95	42.99	5.10
P16388	Potassium voltage-gated channel subfamily A member 1 OS=Mus musculus GN=Kcna1 PE=2 SV=1 - [KCNA1_MOUSE]	21.21	3	5	8	47	0.93	1.08	1.18	1.12	1.21	0.90	0.93	0.92	0.99	0.83	0.88	0.86	0.90	56.37	5.14
Q8K2Y9-2	Isoform 2 of Malcavernin OS=Mus musculus GN=Ccm2 - [CCM2_MOUSE]	24.89	6	9	9	25	0.90	0.94	1.02	0.84	0.91	0.85	0.92	0.92	0.99	0.86	0.94	0.95	1.03	48.22	5.39
Q8BFZ2-2	Isoform 2 of Lipid phosphate phosphatase-related protein type 1 OS=Mus musculus GN=Lppr1 - [LPPR1_MOUSE]	3.05	2	1	1	2	1.31	1.29	0.98	1.35	1.02	1.03	0.78	0.92	0.70	1.29	0.98	1.19	0.91	32.72	7.01
P45376	Aldose reductase OS=Mus musculus GN=Akr1b1 PE=1 SV=3 - [ALDR_MOUSE]	39.24	5	11	12	31	1.05	1.02	0.94	1.06	1.03	0.91	0.87	0.92	0.86	1.00	0.90	0.98	0.88	35.71	7.18

P35363	5-hydroxytryptamine receptor 2A OS=Mus musculus GN=Htr2a PE=2 SV=1 - [5HT2A_MOUSE]	3.61	1	1	1	1	0.86	0.68	0.79	1.12	1.30	0.86	0.99	0.92	1.07	0.86	1.00	1.02	1.19	52.81	6.99
O88544	COP9 signalosome complex subunit 4 OS=Mus musculus GN=Cops4 PE=1 SV=1 - [CSN4_MOUSE]	43.10	4	13	14	35	1.06	1.03	0.92	1.09	1.03	0.98	0.90	0.92	0.88	1.02	0.97	0.97	0.93	46.26	5.83
P16858	Glyceraldehyde-3-phosphate dehydrogenase OS=Mus musculus GN=Gapdh PE=1 SV=2 - [G3P_MOUSE]	88.59	6	16	29	804	1.01	0.88	0.87	1.06	1.04	1.01	1.01	0.92	0.92	0.96	0.97	1.00	1.01	35.79	8.25
Q8K2C6	NAD-dependent protein deacylase sirtuin-5, mitochondrial OS=Mus musculus GN=Sirt5 PE=1 SV=1 - [SIR5_MOUSE]	17.42	1	5	5	10	0.96	1.00	1.05	0.95	0.93	0.85	0.89	0.92	0.88	0.78	0.90	0.81	0.87	34.11	8.53
Q9ESN9-4	Isoform 1d of C-Jun-amino-terminal kinase-interacting protein 3 OS=Mus musculus GN=Mapk8ip3 - [JIP3_MOUSE]	20.36	11	19	21	53	0.99	0.84	0.79	0.93	0.94	0.91	0.92	0.92	0.92	0.93	0.95	0.89	0.93	147.38	5.45
Q9D0G0	28S ribosomal protein S30, mitochondrial OS=Mus musculus GN=Mrps30 PE=2 SV=1 - [RT30_MOUSE]	9.28	1	3	3	5	1.00	0.87	0.92	0.92	1.01	0.93	0.90	0.92	0.92	0.97	0.89	0.91	1.04	49.91	9.38
P56392	Cytochrome c oxidase subunit 7A1, mitochondrial OS=Mus musculus GN=Cox7a1 PE=2 SV=1 - [CX7A1_MOUSE]	16.25	1	1	1	2	1.00	0.78	0.78	0.94	0.94	1.14	1.14	0.92	0.92	0.93	0.93	0.82	0.82	8.98	9.79
Q80XI6	Mitogen-activated protein kinase kinase kinase 11 OS=Mus musculus GN=Map3k11 PE=1 SV=1 - [M3K11_MOUSE]	2.71	2	1	2	3	0.98	1.22	1.24	0.99	1.00	1.00	1.01	0.92	0.93	0.90	0.91	1.01	1.03	93.17	8.43
Q9D6C8	Copine-4 OS=Mus musculus GN=Cpne4 PE=2 SV=1 - [Q9D6C8_MOUSE]	18.95	2	6	7	15	1.04	0.96	0.84	1.09	1.09	1.04	1.00	0.92	0.87	0.99	0.95	1.03	1.00	52.75	5.64
Q3TET1	Kinesin-like protein KIF3A OS=Mus musculus GN=Kif3a PE=2 SV=1 - [Q3TET1_MOUSE]	11.10	10	6	7	13	0.92	1.08	1.14	1.02	1.07	0.92	0.98	0.92	1.00	0.88	1.03	0.97	1.06	79.40	6.25
Q8N7N5	DDB1- and CUL4-associated factor 8 OS=Mus musculus GN=Dcaf8 PE=1 SV=1 - [DCAF8_MOUSE]	9.48	2	5	5	10	0.85	0.97	1.11	0.88	1.04	0.94	1.05	0.92	1.03	0.90	0.96	0.89	0.98	65.99	5.87

P14231	Sodium/potassium-transporting ATPase subunit beta-2 OS=Mus musculus GN=Atp1b2 PE=1 SV=2 - [AT1B2_MOUSE]	37.24	2	9	9	110	1.03	1.00	1.05	1.14	1.11	0.89	0.87	0.92	0.91	0.99	0.96	0.93	0.95	33.32	8.31
P51906	Excitatory amino acid transporter 3 OS=Mus musculus GN=Slc1a1 PE=1 SV=2 - [EAA3_MOUSE]	8.60	1	2	2	3	1.05	1.42	1.35	1.39	1.32	1.06	1.00	0.92	0.88	0.90	0.86	1.15	1.10	56.66	6.11
Q61292	Laminin subunit beta-2 OS=Mus musculus GN=Lamb2 PE=2 SV=2 - [LAMB2_MOUSE]	10.84	1	16	16	34	0.92	0.94	1.03	0.91	1.00	0.87	0.94	0.92	1.07	0.92	1.01	0.83	0.96	196.45	6.67
Q3UHK6	Teneurin-4 OS=Mus musculus GN=Tenm4 PE=1 SV=2 - [TEN4_MOUSE]	11.37	6	18	21	42	1.07	1.10	0.96	1.06	1.02	0.86	0.84	0.92	0.89	0.89	0.86	0.99	0.94	308.23	6.57
P12960	Contactin-1 OS=Mus musculus GN=Cntn1 PE=1 SV=1 - [CNTN1_MOUSE]	55.10	1	52	52	435	1.00	1.03	1.02	0.99	0.99	0.91	0.90	0.92	0.92	0.92	0.89	0.93	0.95	113.32	6.16
P16675	Lysosomal protective protein OS=Mus musculus GN=Ctsa PE=1 SV=1 - [PPGB_MOUSE]	9.28	3	3	3	5	0.82	1.12	1.38	1.11	1.23	0.92	1.01	0.92	1.12	1.01	1.19	0.91	1.11	53.81	5.86
Q7TMK9	Heterogeneous nuclear ribonucleoprotein Q OS=Mus musculus GN=Syncrip PE=1 SV=2 - [HNRPQ_MOUSE]	44.46	5	1	24	112	0.93	0.64	0.68	0.65	0.70	0.83	0.89	0.92	0.98	0.58	0.62	0.70	0.75	69.59	8.59
P20491	High affinity immunoglobulin epsilon receptor subunit gamma OS=Mus musculus GN=Fcer1g PE=1 SV=1 - [FCERG_MOUSE]	12.79	1	1	1	2	1.08	1.03	0.95	0.98	0.91	0.90	0.83	0.92	0.85	0.96	0.89	0.88	0.81	9.65	7.97
Q8K212	Phosphofurin acidic cluster sorting protein 1 OS=Mus musculus GN=Pacs1 PE=1 SV=2 - [PACS1_MOUSE]	20.29	1	13	14	55	1.11	1.01	0.93	1.10	1.00	0.94	0.89	0.92	0.84	0.96	0.91	1.03	0.94	104.76	7.74
E9QLL2	Dynamin-3 OS=Mus musculus GN=Dnm3 PE=2 SV=1 - [E9QLL2_MOUSE]	48.67	12	18	33	119	0.95	1.10	1.18	1.09	1.17	0.92	1.01	0.92	1.02	0.97	1.07	1.00	1.11	97.21	8.56
Q99KV1	DnaJ homolog subfamily B member 11 OS=Mus musculus GN=Dnajb11 PE=1 SV=1 - [DJB11_MOUSE]	30.73	1	7	7	24	0.91	0.77	0.81	0.92	1.00	0.90	0.96	0.92	0.95	0.83	0.89	0.87	0.92	40.53	6.32
E9PYH3	Ethanolamine-phosphate phospho-lyase OS=Mus musculus GN=Agxt2l1 PE=2 SV=1 - [E9PYH3_MOUSE]	2.23	2	1	1	2	1.01	0.77	0.76	0.88	0.87	0.73	0.71	0.92	0.91	0.88	0.86	0.60	0.60	54.83	6.87

Q6PHN9	Ras-related protein Rab 35 OS=Mus musculus GN=Rab35 PE=1 SV=1 - [RAB35_MOUSE]	48.26	10	7	9	73	1.09	1.09	0.99	1.09	1.01	0.89	0.88	0.92	0.83	0.90	0.88	1.01	1.00	23.01	8.29
Q8R1N4	NudC domain-containing protein 3 OS=Mus musculus GN=Nudcd3 PE=2 SV=3 - [NUDC3_MOUSE]	20.39	5	5	5	11	0.89	0.91	1.03	0.97	1.10	0.88	0.97	0.92	1.03	0.94	1.12	0.94	1.08	40.86	5.26
Q3U2A8	Valine--tRNA ligase, mitochondrial OS=Mus musculus GN=Vars2 PE=2 SV=2 - [SYVM_MOUSE]	6.70	4	5	5	8	1.00	1.04	1.04	1.08	1.08	1.05	1.02	0.92	0.98	1.10	1.04	1.06	1.19	118.39	7.14
Q9D2E2-2	Isoform 2 of Target of EGR1 protein 1 OS=Mus musculus GN=Toe1 - [TOE1_MOUSE]	3.65	2	1	1	1	0.91	1.03	1.13	0.89	0.98	0.81	0.89	0.92	1.01	0.96	1.06	0.90	0.99	37.13	6.77
P62700	Protein yippee-like 5 OS=Mus musculus GN=Ypel5 PE=2 SV=1 - [YPEL5_MOUSE]	21.49	1	2	2	4	0.95	1.14	1.20	0.92	0.97	0.86	0.90	0.92	0.97	0.86	0.90	0.92	0.97	13.83	7.31
Q6PIE5	Sodium/potassium-transporting ATPase subunit alpha-2 OS=Mus musculus GN=Atp1a2 PE=1 SV=1 - [AT1A2_MOUSE]	55.59	9	37	62	1114	0.98	1.01	1.05	1.08	1.10	0.90	0.90	0.92	0.92	0.97	0.96	0.90	0.94	112.15	5.55
K3W4T7	Neurexin-1 OS=Mus musculus GN=Nrxn1 PE=4 SV=1 - [K3W4T7_MOUSE]	35.02	14	29	35	157	1.03	0.98	0.98	1.03	0.99	0.92	0.88	0.92	0.89	0.90	0.89	0.92	0.91	134.37	6.19
D3YTZ7	Josephin-2 OS=Mus musculus GN=Josd2 PE=2 SV=1 - [D3YTZ7_MOUSE]	8.22	2	1	1	2	1.16	1.48	1.27	1.06	0.92	1.06	0.91	0.92	0.80	0.92	0.79	1.02	0.88	16.11	5.49
Q68FG2	Protein Sptbn2 OS=Mus musculus GN=Sptbn2 PE=2 SV=1 - [Q68FG2_MOUSE]	58.71	2	100	111	462	0.91	1.01	1.12	1.05	1.14	0.92	1.00	0.92	1.02	0.93	1.03	0.99	1.09	270.76	5.86
Q6PAJ1	Breakpoint cluster region protein OS=Mus musculus GN=Bcr PE=1 SV=3 - [BCR_MOUSE]	15.20	1	12	13	28	1.09	1.09	1.01	1.11	1.03	0.99	0.92	0.92	0.90	1.14	1.01	1.23	1.10	142.98	6.92
Q5SZV5	Dyslexia-associated protein KIAA0319 homolog OS=Mus musculus GN=Kiaa0319 PE=2 SV=1 - [K0319_MOUSE]	1.11	1	1	1	1	1.03	1.14	1.10	1.22	1.18	1.10	1.06	0.92	0.89	0.96	0.93	1.40	1.35	117.91	5.17
Q5SX99	Leucine-rich repeat-containing protein 48 OS=Mus musculus GN=Lrrc48 PE=2 SV=1 - [Q5SX99_MOUSE]	5.77	4	1	1	1	1.37	0.61	0.45	0.96	0.70	1.15	0.84	0.92	0.67	0.88	0.64	1.04	0.76	23.77	4.70

Q69ZF3	Non-lysosomal glucosylceramidase OS=Mus musculus GN=Gba2 PE=1 SV=2 - [GBA2_MOUSE]	14.16	2	9	9	18	1.04	1.09	1.09	1.06	1.02	0.95	0.87	0.92	0.88	0.93	1.02	1.05	1.04	103.23	5.59
Q8C878	NEDD8-activating enzyme E1 catalytic subunit OS=Mus musculus GN=Uba3 PE=1 SV=2 - [UBA3_MOUSE]	35.50	3	11	11	27	1.02	0.88	0.96	1.06	1.06	0.99	0.96	0.92	0.90	0.98	0.96	0.93	0.95	51.70	5.45
Q922H4	Mannose-1-phosphate guanyltransferase alpha OS=Mus musculus GN=Gmppa PE=2 SV=1 - [GMPPA_MOUSE]	11.43	6	3	3	5	1.00	1.07	1.07	1.03	1.05	0.88	0.89	0.92	0.89	0.87	0.89	0.88	0.88	46.22	7.62
Q3V2N7	Protein AI413582 OS=Mus musculus GN=AI413582 PE=4 SV=1 - [Q3V2N7_MOUSE]	6.86	1	1	1	2	0.97	0.68	0.70	0.80	0.83	0.96	0.99	0.92	0.95	0.86	0.89	0.91	0.94	11.55	6.95
Q8VCU2	Glycosylphosphatidylinositol specific phospholipase D1 OS=Mus musculus GN=Gpld1 PE=2 SV=1 - [Q8VCU2_MOUSE]	4.51	2	3	3	6	1.02	1.18	1.12	1.01	0.98	0.92	0.89	0.92	0.90	1.01	0.99	1.01	0.93	93.56	6.80
Q8C8R3-2	Isoform 2 of Ankyrin-2 OS=Mus musculus GN=Ank2 - [ANK2_MOUSE]	58.61	2	5	159	1021	1.03	1.11	1.01	1.05	1.05	1.04	1.02	0.92	0.92	1.00	0.94	1.01	0.98	428.50	5.16
P63280	SUMO-conjugating enzyme UBC9 OS=Mus musculus GN=Ube2i PE=1 SV=1 - [UBC9_MOUSE]	41.14	4	3	3	6	0.96	0.86	0.90	1.05	1.11	0.83	0.81	0.92	1.07	0.80	0.79	0.82	1.01	18.00	8.66
Q99LC2	Cleavage stimulation factor subunit 1 OS=Mus musculus GN=Cstf1 PE=2 SV=1 - [CSTF1_MOUSE]	9.28	2	2	2	4	0.80	1.08	1.35	0.95	1.19	0.95	1.19	0.92	1.15	0.86	1.08	0.89	1.11	48.35	6.58
Q9JKF1	Ras GTPase-activating-like protein IQGAP1 OS=Mus musculus GN=Iqgap1 PE=1 SV=2 - [IQGA1_MOUSE]	1.69	1	2	2	9	0.88	1.12	1.25	1.01	1.15	0.82	0.96	0.92	1.02	0.90	1.12	0.90	1.06	188.62	6.48
Q99J56	Derlin-1 OS=Mus musculus GN=Derl1 PE=2 SV=1 - [DERL1_MOUSE]	9.96	1	2	2	4	0.65	1.05	1.61	0.97	1.50	0.69	1.05	0.92	1.42	0.68	1.05	0.67	1.03	28.82	9.51
Q5SVR0-2	Isoform 2 of TBC1 domain family member 9B OS=Mus musculus GN=Tbc1d9b - [TBC9B_MOUSE]	2.57	2	2	2	6	1.02	1.26	1.23	1.10	1.04	0.96	0.93	0.92	0.93	0.95	1.01	1.00	0.98	139.84	5.35
Q8BIZ1-2	Isoform 2 of Ankyrin repeat and sterile alpha motif domain-containing protein 1B OS=Mus musculus GN=Anks1b - [ANS1B_MOUSE]	26.29	5	2	10	20	0.97	1.16	1.19	1.14	1.16	0.97	0.97	0.92	0.87	0.99	1.00	0.99	1.03	48.31	7.97

Q7TMY8-	Isoform 3 of E3 ubiquitin-protein ligase HUWE1 OS=Mus musculus GN=Huwe1 - [HUWE1_MOUSE]	7.61	6	22	22	49	0.97	1.11	1.15	1.10	1.14	0.93	0.99	0.92	0.97	1.00	1.01	1.10	1.12	480.60	5.20
Q8BRK8	5'-AMP-activated protein kinase catalytic subunit alpha-2 OS=Mus musculus GN=Prkaa2 PE=1 SV=3 - [AAPK2_MOUSE]	10.87	1	3	5	8	0.92	1.05	1.21	1.08	1.18	0.87	1.06	0.92	1.07	0.96	1.04	0.87	0.96	61.98	7.87
Q9JI90	E3 ubiquitin-protein ligase RNF14 OS=Mus musculus GN=Rnf14 PE=2 SV=2 - [RNF14_MOUSE]	14.23	3	6	6	15	0.96	0.84	0.88	0.93	0.95	0.99	0.97	0.92	0.99	0.87	0.90	0.91	0.91	54.89	4.70
Q8R2Y2-2	Isoform 2 of Cell surface glycoprotein MUC18 OS=Mus musculus GN=Mcam - [MUC18_MOUSE]	19.47	2	9	9	17	0.92	1.09	1.02	0.99	0.97	0.93	1.02	0.92	1.12	0.98	1.08	0.99	1.02	66.86	5.82
A2AHT3	Calsenilin isoform 4 OS=Mus musculus GN=Kcnip3 PE=2 SV=1 - [A2AHT3_MOUSE]	17.61	4	1	4	8	0.99	0.87	0.87	0.78	0.79	0.91	0.91	0.92	0.93	0.91	0.92	1.01	1.02	32.11	5.40
Q9Z1G3	V-type proton ATPase subunit C 1 OS=Mus musculus GN=Atp6v1c1 PE=1 SV=4 - [VATC1_MOUSE]	58.12	1	21	21	80	0.97	0.97	0.94	1.09	1.09	0.98	0.97	0.92	0.91	0.98	1.04	1.04	1.02	43.86	7.46
Q6PGN3-2	Isoform 2 of Serine/threonine-protein kinase DCLK2 OS=Mus musculus GN=Dclk2 - [DCLK2_MOUSE]	28.21	6	13	13	29	0.99	0.83	0.83	1.03	1.05	0.96	1.00	0.92	0.94	0.97	0.99	1.07	1.10	82.80	8.44
B1AY85	Plexin domain-containing protein 2 OS=Mus musculus GN=Plxdc2 PE=2 SV=1 - [B1AY85_MOUSE]	3.98	2	2	2	3	1.01	1.03	1.02	0.93	0.92	0.91	0.89	0.92	0.91	0.92	0.91	1.02	1.01	59.35	6.60
Q9JLZ3	Methylglutaconyl-CoA hydratase, mitochondrial OS=Mus musculus GN=Auh PE=2 SV=1 - [AUHM_MOUSE]	29.62	7	10	10	51	0.96	1.02	0.98	0.96	1.01	0.95	1.02	0.92	0.95	0.92	0.97	0.94	1.02	33.37	9.51
F8WHP5	Phospholipase DDHD1 OS=Mus musculus GN=Ddhd1 PE=2 SV=1 - [F8WHP5_MOUSE]	6.07	8	4	4	6	0.96	1.05	1.08	1.03	1.09	0.99	0.95	0.92	0.93	1.01	0.99	1.09	1.12	94.73	5.69
B7ZC46	Septin-8 OS=Mus musculus GN=Sept8 PE=2 SV=1 - [B7ZC46_MOUSE]	44.77	2	1	18	86	1.24	0.87	0.70	1.09	0.87	0.98	0.78	0.92	0.74	0.86	0.69	0.96	0.77	50.86	6.09
Q91YM4-	Isoform 2 of Protein TBRG4 OS=Mus musculus GN=Tbrg4 - [TBRG4_MOUSE]	4.50	5	2	2	3	1.04	1.00	0.96	1.29	1.24	0.89	0.85	0.92	0.88	0.95	0.92	1.00	0.96	68.10	8.32

Q91ZR1	Ras-related protein Rab 4B OS=Mus musculus GN=Rab4b PE=2 SV=2 - [RAB4B_MOUSE]	40.38	10	4	7	58	1.09	1.26	1.16	1.07	1.02	0.85	0.78	0.92	0.87	1.01	0.91	1.05	0.93	23.61	6.06
A2AEG6	Glycoprotein m6b, isoform CRA_g OS=Mus musculus GN=Gpm6b PE=4 SV=1 - [A2AEG6_MOUSE]	23.78	10	7	7	21	0.96	1.12	1.07	1.21	1.21	0.92	0.97	0.92	0.95	0.92	0.94	0.95	0.96	36.17	6.14
O35083	1-acyl-sn-glycerol-3-phosphate acyltransferase alpha OS=Mus musculus GN=Agpat1 PE=2 SV=1 - [PLCA_MOUSE]	18.95	1	4	4	7	0.95	1.13	1.11	1.11	1.17	0.89	1.00	0.93	0.91	1.00	1.00	1.05	1.05	31.69	9.14
Q9D9E0-2	Isoform 2 of Solute carrier family 22 member 17 OS=Mus musculus GN=Slc22a17 - [S22AH_MOUSE]	5.46	2	1	1	4	1.10	1.18	1.08	1.40	1.27	0.96	0.87	0.93	0.84	1.00	0.91	1.16	1.06	39.89	8.43
Q8K0Y7	Uncharacterized protein C9orf142 homolog OS=Mus musculus PE=2 SV=1 - [CI142_MOUSE]	8.78	1	1	1	2	1.25	0.94	0.75	0.83	0.66	0.92	0.73	0.93	0.74	0.95	0.76	0.97	0.77	21.96	5.66
A2AAE1-	Isoform 4 of Uncharacterized protein KIAA1109 OS=Mus musculus GN=Kiaa1109 - [K1109_MOUSE]	0.45	5	1	1	2	0.76	0.94	1.23	1.16	1.52	0.84	1.10	0.93	1.21	0.89	1.17	0.90	1.18	510.03	6.62
Q8CIE2-2	Isoform 2 of Zinc finger MIZ domain-containing protein 2 OS=Mus musculus GN=Zmiz2 - [ZMIZ2_MOUSE]	1.01	5	1	1	2	0.91	0.90	0.98	0.99	1.08	0.94	1.02	0.93	1.01	0.93	1.02	1.04	1.14	93.81	7.14
E9Q0N0	Intersectin-1 OS=Mus musculus GN=Itsn1 PE=2 SV=1 - [E9Q0N0_MOUSE]	23.56	11	1	35	86	0.77	0.57	0.73	0.87	1.13	0.81	1.05	0.93	1.19	0.95	1.24	0.80	1.04	194.78	7.91
P61290	Proteasome activator complex subunit 3 OS=Mus musculus GN=Psme3 PE=1 SV=1 - [PSME3_MOUSE]	22.05	3	5	5	8	1.03	0.93	0.99	0.98	0.94	0.91	0.97	0.93	0.98	0.77	0.99	0.95	1.12	29.49	5.95
P33622	Apolipoprotein C-III OS=Mus musculus GN=Apoc3 PE=2 SV=2 - [APOC3_MOUSE]	19.19	2	1	1	2	0.67	1.01	1.49	0.56	0.82	0.68	1.01	0.93	1.37	1.90	2.82	1.23	1.83	10.98	4.75
P19324	Serpin H1 OS=Mus musculus GN=Serpinh1 PE=1 SV=3 - [SERPH_MOUSE]	10.31	1	3	3	5	0.89	1.00	1.11	1.11	1.20	0.95	1.06	0.93	1.05	0.99	1.12	1.09	1.22	46.50	8.82
Q9ERL9	Guanylate cyclase soluble subunit alpha-3 OS=Mus musculus GN=Gucy1a3 PE=2 SV=2 - [GCYA3_MOUSE]	5.93	1	3	3	7	1.10	1.12	0.84	1.21	1.07	0.93	0.78	0.93	0.85	1.11	1.03	1.07	0.99	77.54	7.17

Q7TS64	Adrenergic receptor kinase, beta 1 OS=Mus musculus GN=Adrbk1 PE=2 SV=1 - [Q7TS64_MOUSE]	23.18	7	8	12	26	0.98	1.04	1.05	1.02	1.04	0.96	0.93	0.93	0.95	0.96	0.99	0.92	0.97	75.11	7.17
E9Q3L2	Protein Pi4ka OS=Mus musculus GN=Pi4ka PE=4 SV=1 - [E9Q3L2_MOUSE]	13.85	5	22	22	54	0.95	1.10	1.04	1.13	1.19	0.88	0.88	0.93	0.91	0.90	0.95	1.09	1.07	231.21	6.87
P48320	Glutamate decarboxylase 2 OS=Mus musculus GN=Gad2 PE=2 SV=1 - [DCE2_MOUSE]	18.97	1	7	8	24	1.12	0.98	0.88	1.02	0.94	0.91	0.82	0.93	0.82	1.09	1.04	1.05	0.98	65.18	6.90
P54071	Isocitrate dehydrogenase [NADP], mitochondrial OS=Mus musculus GN=Idh2 PE=1 SV=3 - [IDHP_MOUSE]	37.83	2	16	17	41	1.03	0.88	0.95	1.10	1.23	0.96	0.98	0.93	0.89	1.06	1.00	1.09	1.03	50.87	8.69
D3Z484	PIH1 domain-containing protein 1 (Fragment) OS=Mus musculus GN=Pih1d1 PE=2 SV=1 - [D3Z484_MOUSE]	11.03	2	1	1	1	0.94	0.86	0.91	0.75	0.79	1.08	1.14	0.93	0.98	0.94	1.00	0.88	0.93	14.94	4.49
Q8C341	SUN domain-containing ossification factor OS=Mus musculus GN=Suco PE=1 SV=3 - [SUCO_MOUSE]	0.96	2	1	1	2	0.97	1.14	1.18	1.23	1.27	0.93	0.96	0.93	0.95	1.08	1.12	1.07	1.11	139.08	5.02
E9PY16	Protein Adap1 OS=Mus musculus GN=Adap1 PE=2 SV=1 - [E9PY16_MOUSE]	33.69	2	10	10	23	0.99	0.86	0.88	1.07	1.09	0.98	0.95	0.93	0.93	0.97	0.96	1.01	0.97	43.34	8.65
Q9CQ40	39S ribosomal protein L49, mitochondrial OS=Mus musculus GN=Mrpl49 PE=2 SV=1 - [RM49_MOUSE]	44.58	1	6	6	15	0.98	0.80	0.89	0.92	0.97	0.83	0.85	0.93	0.89	0.80	0.79	0.81	0.81	19.12	9.50
P41242-3	Isoform 3 of Megakaryocyte-associated tyrosine-protein kinase OS=Mus musculus GN=Matk - [MATK_MOUSE]	6.45	7	2	2	3	0.91	0.78	0.86	0.91	1.00	1.00	1.09	0.93	1.01	0.93	1.02	0.96	1.05	51.46	8.40
Q8CJ61	CKLF-like MARVEL transmembrane domain-containing protein 4 OS=Mus musculus GN=Cmtm4 PE=2 SV=1 - [CKLF4_MOUSE]	4.81	1	1	1	2	0.97	1.02	1.05	1.03	1.06	0.99	1.01	0.93	0.95	0.99	1.02	0.90	0.93	22.91	5.59
P46097	Synaptotagmin-2 OS=Mus musculus GN=Syt2 PE=1 SV=1 - [SYT2_MOUSE]	48.34	1	11	17	120	0.98	1.09	1.17	1.02	1.08	0.86	0.88	0.93	0.95	0.94	0.90	0.82	0.87	47.23	7.99

A2APY7-2	Isoform 2 of NADH dehydrogenase [ubiquinone] 1 alpha subcomplex assembly factor 5 OS=Mus musculus GN=Ndufaf5 - [NDUF5_MOUSE]	7.05	2	1	1	2	0.92	1.14	1.24	1.02	1.11	0.97	1.06	0.93	1.01	1.17	1.28	1.03	1.13	17.51	5.52
Q8BGU5	Cyclin-Y OS=Mus musculus GN=Ccny PE=1 SV=1 - [CCNY_MOUSE]	18.77	4	5	5	12	0.93	1.05	1.12	1.16	1.09	1.02	0.99	0.93	1.03	1.02	1.02	1.12	1.23	39.37	7.20
Q8R071	Inositol-trisphosphate 3-kinase A OS=Mus musculus GN=Itpka PE=2 SV=1 - [IP3KA_MOUSE]	33.99	1	11	12	39	0.93	1.07	1.21	1.06	1.12	0.97	1.04	0.93	0.94	0.94	0.99	0.95	1.00	50.90	7.72
Q6P1B1	Xaa-Pro aminopeptidase 1 OS=Mus musculus GN=Xpnpep1 PE=2 SV=1 - [XPP1_MOUSE]	27.13	1	12	12	29	0.99	0.92	1.00	1.07	1.10	1.08	1.03	0.93	0.94	0.98	1.00	1.04	1.05	69.55	5.54
H3BIW6	1-phosphatidylinositol 4,5-bisphosphate phosphodiesterase eta-2 OS=Mus musculus GN=Plch2 PE=2 SV=1 - [H3BIW6_MOUSE]	17.59	19	14	15	30	1.00	1.05	1.09	1.02	1.01	0.98	0.92	0.93	0.93	1.02	1.00	1.04	1.10	125.70	6.79
P46096	Synaptotagmin-1 OS=Mus musculus GN=Syt1 PE=1 SV=1 - [SYT1_MOUSE]	57.48	1	15	28	412	0.98	0.95	1.00	1.01	1.02	0.94	0.97	0.93	0.97	0.94	0.97	0.95	0.98	47.39	8.53
Q8C0E2	Vacuolar protein sorting-associated protein 26B OS=Mus musculus GN=Vps26b PE=1 SV=1 - [VP26B_MOUSE]	38.99	2	11	11	32	1.04	1.01	0.96	0.96	0.91	0.88	0.85	0.93	0.87	0.88	0.87	0.93	0.91	39.10	7.37
Q9CPR5	39S ribosomal protein L15, mitochondrial OS=Mus musculus GN=Mrpl15 PE=1 SV=1 - [RM15_MOUSE]	6.44	2	2	2	3	0.97	0.85	0.87	1.09	1.12	0.92	0.94	0.93	0.95	1.01	1.04	0.94	0.97	33.52	10.07
P70698	CTP synthase 1 OS=Mus musculus GN=Ctps1 PE=1 SV=2 - [PYRG1_MOUSE]	8.29	1	4	4	5	0.91	1.08	1.08	1.08	1.22	0.84	0.92	0.93	0.95	0.93	0.96	0.99	1.08	66.64	6.58
O88962	7-alpha-hydroxycholest-4-en-3-one 12-alpha-hydroxylase OS=Mus musculus GN=Cyp8b1 PE=2 SV=1 - [CP8B1_MOUSE]	1.80	1	1	1	1	0.73	0.88	1.20	0.71	0.97	1.11	1.52	0.93	1.26	1.04	1.42	1.30	1.78	57.67	8.84
Q9JMB0	G kinase-anchoring protein 1 OS=Mus musculus GN=Gkap1 PE=1 SV=1 - [GKAP1_MOUSE]	12.02	1	4	4	6	0.97	0.92	0.95	0.90	1.12	1.02	1.09	0.93	0.98	1.15	1.00	0.88	1.02	41.74	8.82
P12023	Amyloid beta A4 protein OS=Mus musculus GN=App PE=1 SV=3 - [A4_MOUSE]	32.34	2	1	20	201	0.91	0.68	0.75	0.83	0.91	0.92	1.00	0.93	1.02	0.95	1.04	0.84	0.92	86.67	4.79

O70566	Protein diaphanous homolog 2 OS=Mus musculus GN=Diaph2 PE=1 SV=2 - [DIAP2_MOUSE]	5.65	3	5	7	12	0.98	0.91	1.06	1.14	1.08	0.91	0.90	0.93	0.96	1.00	1.03	0.97	1.12	124.79	6.92
Q8BLV3-	Isoform 2 of Sodium/hydrogen exchanger 7 OS=Mus musculus GN=Slc9a7 - [SL9A7_MOUSE]	3.65	2	2	2	4	1.13	1.48	1.30	1.14	1.01	0.93	0.82	0.93	0.82	1.06	0.94	1.12	0.99	60.76	8.82
Q8CHT0	Delta-1-pyrroline-5-carboxylate dehydrogenase, mitochondrial OS=Mus musculus GN=Aldh4a1 PE=1 SV=3 - [AL4A1_MOUSE]	19.75	1	7	7	16	0.98	0.91	0.97	1.06	1.08	0.97	0.96	0.93	0.95	1.02	0.99	1.02	0.97	61.80	8.24
P17439	Glucosylceramidase OS=Mus musculus GN=Gba PE=1 SV=1 - [GLCM_MOUSE]	2.52	1	1	1	2	1.11	1.09	0.99	1.07	0.96	0.91	0.82	0.93	0.84	0.92	0.83	0.87	0.79	57.59	7.75
Q9D3A9	Protein tweety homolog 1 OS=Mus musculus GN=Ttyh1 PE=1 SV=1 - [TTYH1_MOUSE]	12.89	6	5	5	11	1.06	1.28	1.16	1.11	0.98	0.92	0.79	0.93	0.85	0.94	0.90	1.07	1.02	49.00	5.03
Q923T9-3	Isoform 3 of Calcium/calmodulin-dependent protein kinase type II subunit gamma OS=Mus musculus GN=Camk2g - [KCC2G_MOUSE]	38.59	3	8	15	67	0.92	1.01	1.07	1.12	1.21	0.93	0.95	0.93	1.00	0.91	0.96	0.86	0.94	55.93	7.18
Q8BWM0	Prostaglandin E synthase 2 OS=Mus musculus GN=Ptges2 PE=1 SV=3 - [PGES2_MOUSE]	18.75	1	5	5	6	0.87	0.82	0.97	1.04	1.13	0.88	0.97	0.93	0.99	0.89	1.03	0.99	1.06	43.30	9.00
Q99LM2	CDK5 regulatory subunit-associated protein 3 OS=Mus musculus GN=Cdk5rap3 PE=2 SV=1 - [CK5P3_MOUSE]	6.16	1	3	3	5	1.01	1.13	1.17	1.01	1.01	0.95	0.98	0.93	0.90	1.06	1.06	1.02	1.02	56.95	4.83
E9Q1T3	Mitogen-activated protein kinase kinase kinase kinase OS=Mus musculus GN=Map4k5 PE=2 SV=1 - [E9Q1T3_MOUSE]	3.85	4	2	2	7	0.96	1.19	1.25	1.07	1.20	0.91	0.92	0.93	0.99	0.98	1.04	1.06	1.03	87.40	8.07
P23591	GDP-L-fucose synthase OS=Mus musculus GN=Tsta3 PE=2 SV=3 - [FCL_MOUSE]	14.64	1	3	3	5	1.07	1.11	1.03	1.02	0.95	1.04	0.97	0.93	0.86	0.96	0.89	1.00	0.94	35.86	6.74
Q7TPW1	Nexilin OS=Mus musculus GN=Nexn PE=1 SV=3 - [NEXN_MOUSE]	14.00	1	6	6	10	0.91	1.05	1.04	0.87	0.95	1.08	1.08	0.93	1.02	1.18	1.12	1.24	1.31	72.06	5.01
Q9CXU4	MCG11298 OS=Mus musculus GN=Timm23 PE=2 SV=1 - [Q9CXU4_MOUSE]	4.78	2	1	1	2	1.04	1.12	1.07	1.25	1.20	0.72	0.69	0.93	0.89	0.95	0.91	1.04	1.00	21.89	8.60

Q8K268	ATP-binding cassette sub-family F member 3 OS=Mus musculus GN=Abcf3 PE=1 SV=1 - [ABCF3_MOUSE]	5.92	1	3	3	5	1.01	1.28	1.27	1.00	0.99	0.88	0.86	0.93	0.92	0.96	1.02	1.02	0.90	79.82	6.16
P97807-2	Isoform Cytoplasmic of Fumarate hydratase, mitochondrial OS=Mus musculus GN=Fh - [FUMH_MOUSE]	67.67	3	23	23	87	1.00	0.95	0.98	1.06	1.03	0.97	0.98	0.93	0.93	0.93	0.94	0.90	0.94	50.02	7.94
P62702	40S ribosomal protein S4, X isoform OS=Mus musculus GN=Rps4x PE=2 SV=2 - [RS4X_MOUSE]	31.18	2	7	7	18	1.12	1.07	0.99	1.14	1.05	1.03	0.95	0.93	0.91	1.08	0.98	1.14	1.09	29.58	10.15
Q6P5G6	UBX domain-containing protein 7 OS=Mus musculus GN=Ubxn7 PE=1 SV=2 - [UBXN7_MOUSE]	18.42	1	1	6	10	0.94	0.99	1.05	0.87	0.93	0.96	1.02	0.93	0.98	0.94	1.00	1.07	1.14	52.13	5.03
F7CBP1	Eukaryotic translation initiation factor 4 gamma 2 OS=Mus musculus GN=Eif4g2 PE=4 SV=1 - [F7CBP1_MOUSE]	6.11	4	5	5	19	0.97	1.16	1.20	1.09	1.13	0.92	0.95	0.93	0.94	0.98	1.00	0.94	1.04	97.83	6.99
Q8BY89-2	Isoform 2 of Choline transporter-like protein 2 OS=Mus musculus GN=Slc44a2 - [CTL2_MOUSE]	4.97	2	3	3	5	1.08	1.32	1.12	1.19	1.06	0.96	0.96	0.93	0.93	1.06	1.07	1.05	1.06	79.87	8.66
Q9DAI2	Rab-like protein 5 OS=Mus musculus GN=Rabl5 PE=2 SV=1 - [RABL5_MOUSE]	29.73	1	4	4	6	0.75	0.80	1.08	0.72	1.06	0.94	1.26	0.93	1.14	0.84	0.89	0.79	1.02	20.82	5.25
Q8R4X3	RNA-binding protein 12 OS=Mus musculus GN=Rbm12 PE=1 SV=3 - [RBM12_MOUSE]	5.34	2	4	4	9	0.92	0.82	0.76	0.89	0.90	0.78	0.78	0.93	0.93	0.82	0.94	0.84	0.93	102.73	8.32
Q0VBL1	Tigger transposable element-derived protein 2 OS=Mus musculus GN=Tigd2 PE=2 SV=1 - [TIGD2_MOUSE]	1.14	1	1	1	1	1.11	1.46	1.32	0.84	0.76	0.87	0.78	0.93	0.83	1.03	0.93	0.92	0.83	59.61	9.22
O09167	60S ribosomal protein L21 OS=Mus musculus GN=Rpl21 PE=2 SV=3 - [RL21_MOUSE]	24.38	4	3	3	6	0.78	0.78	0.88	0.99	1.30	0.92	1.07	0.93	0.97	1.06	1.17	0.92	1.24	18.55	10.49
D3Z7E5	Glycogen synthase kinase-3 alpha OS=Mus musculus GN=Gsk3a PE=2 SV=1 - [D3Z7E5_MOUSE]	30.25	8	7	10	28	0.94	1.12	1.00	0.97	1.01	0.97	0.96	0.93	0.91	0.98	1.01	0.96	1.01	51.21	8.91

Q9D7N9	Adipocyte plasma membrane-associated protein OS=Mus musculus GN=Apmap PE=1 SV=1 - [APMAP_MOUSE]	13.73	1	5	5	10	0.93	1.23	1.31	0.98	1.04	0.82	0.88	0.93	1.00	0.90	0.96	0.90	0.94	46.40	6.32
Q91XU3	Phosphatidylinositol 5-phosphate 4-kinase type-2 gamma OS=Mus musculus GN=Pip4k2c PE=2 SV=1 - [PI42C_MOUSE]	12.35	1	5	5	13	0.96	0.85	0.91	1.01	1.10	0.96	0.98	0.93	0.95	0.85	0.86	0.99	0.97	47.31	6.89
Q9ESX5	H/ACA ribonucleoprotein complex subunit 4 OS=Mus musculus GN=Dkc1 PE=1 SV=4 - [DKC1_MOUSE]	11.79	2	4	4	8	1.01	1.46	1.67	1.09	1.04	1.02	0.95	0.93	0.95	0.99	0.94	1.08	1.00	57.37	9.28
Q921G7	Electron transfer flavoprotein-ubiquinone oxidoreductase, mitochondrial OS=Mus musculus GN=Etfdh PE=1 SV=1 - [ETFD_MOUSE]	28.25	2	14	14	29	1.04	0.98	0.93	1.12	1.07	0.92	0.92	0.93	0.86	1.01	1.02	0.95	0.94	68.05	7.58
Q8CDG3	Deubiquitinating protein VCIP135 OS=Mus musculus GN=Vcpip1 PE=2 SV=1 - [VCIP1_MOUSE]	24.18	2	19	19	47	0.95	0.98	1.04	0.99	1.03	0.97	1.02	0.93	0.99	0.95	1.01	0.97	1.00	134.42	7.17
Q9D8B7	Junctional adhesion molecule C OS=Mus musculus GN=Jam3 PE=1 SV=2 - [JAM3_MOUSE]	21.94	2	7	7	19	0.99	1.24	1.24	1.01	1.05	0.92	0.96	0.93	0.92	0.95	0.93	1.01	1.01	34.82	7.03
D3Z497	Uncharacterized protein OS=Mus musculus GN=Gm7075 PE=4 SV=1 - [D3Z497_MOUSE]	18.75	2	2	2	2	1.02	0.80	0.77	0.89	0.86	1.02	0.99	0.93	0.90	0.89	0.87	0.77	0.75	12.64	6.70
Q8C079-3	Isoform 3 of Striatin-interacting protein 1 OS=Mus musculus GN=Strip1 - [STRP1_MOUSE]	13.78	4	7	8	16	0.96	1.03	1.14	1.11	1.16	0.92	0.95	0.93	0.92	1.08	1.12	1.07	1.07	86.83	5.66
Q9CQ62	2,4-dienoyl-CoA reductase, mitochondrial OS=Mus musculus GN=Decr1 PE=1 SV=1 - [DECR_MOUSE]	14.63	1	4	4	12	1.14	0.84	0.88	1.01	0.91	0.87	0.80	0.93	0.93	0.99	1.01	1.15	1.07	36.19	8.95
F7D0T8	RING finger protein 157 (Fragment) OS=Mus musculus GN=Rnf157 PE=4 SV=1 - [F7D0T8_MOUSE]	1.98	5	1	1	3	0.86	0.81	0.94	0.89	1.03	0.97	1.12	0.93	1.07	0.88	1.03	0.97	1.13	54.80	4.61
Q91VD9	NADH-ubiquinone oxidoreductase 75 kDa subunit, mitochondrial OS=Mus musculus GN=Ndufs1 PE=1 SV=2 - [NDUS1_MOUSE]	59.56	1	33	33	151	0.94	0.96	1.00	1.00	1.05	0.92	0.98	0.93	0.97	0.93	0.96	0.97	1.07	79.73	5.72

Q8K596	Protein Slc8a2 OS=Mus musculus GN=Slc8a2 PE=2 SV=1 - [Q8K596_MOUSE]	37.02	5	28	29	160	0.97	1.08	1.10	1.08	1.10	0.91	0.91	0.93	0.97	0.94	0.97	1.03	1.03	100.65	5.12
A2ACM0	Regulatory-associated protein of mTOR OS=Mus musculus GN=Rptor PE=4 SV=1 - [A2ACM0_MOUSE]	0.67	2	1	1	1	1.07	1.07	1.00	1.00	0.93	0.86	0.79	0.93	0.86	0.96	0.89	1.04	0.97	149.40	6.87
B1AXZ0	ELAV-like protein 2 OS=Mus musculus GN=Elavl2 PE=2 SV=1 - [B1AXZ0_MOUSE]	40.75	9	5	11	26	0.99	1.04	0.98	0.90	0.91	0.88	0.86	0.93	0.87	0.83	0.82	0.92	0.87	37.98	9.04
P63082	V-type proton ATPase 16 kDa proteolipid subunit OS=Mus musculus GN=Atp6v0c PE=2 SV=1 - [VATL_MOUSE]	54.84	3	3	3	5	0.99	0.89	0.90	1.12	1.13	0.88	0.88	0.93	0.94	0.94	0.95	0.99	1.00	15.80	9.13
A8DUK4	Beta-globin OS=Mus musculus GN=Hbb-b1 PE=2 SV=1 - [A8DUK4_MOUSE]	89.80	5	14	14	188	0.84	1.01	1.18	0.54	0.65	0.83	0.96	0.93	1.14	1.14	1.30	0.70	0.83	15.74	7.69
Q5XJF6	Ribosomal protein OS=Mus musculus GN=Rpl10a PE=2 SV=1 - [Q5XJF6_MOUSE]	26.73	4	6	6	25	0.95	0.89	0.92	1.06	1.12	0.91	1.01	0.93	1.03	0.96	1.06	1.01	1.06	24.82	9.94
Q8K2P7-2	Isoform 2 of Sodium-coupled neutral amino acid transporter 1 OS=Mus musculus GN=Slc38a1 - [S38A1_MOUSE]	3.20	2	1	1	2	1.35	0.76	0.56	0.97	0.72	1.18	0.87	0.93	0.69	0.95	0.70	1.26	0.93	38.09	6.33
Q9CPV4	Glyoxalase domain-containing protein 4 OS=Mus musculus GN=Glod4 PE=2 SV=1 - [GLOD4_MOUSE]	78.86	8	19	19	84	1.00	0.99	0.95	1.01	1.00	0.96	0.95	0.93	0.91	0.95	0.94	0.95	0.94	33.30	5.47
Q8VD73	Potassium voltage-gated channel, shaker-related subfamily, beta member 3 OS=Mus musculus GN=Kcnab3 PE=2 SV=1 - [Q8VD73_MOUSE]	11.14	2	3	3	5	0.79	0.98	1.20	0.98	1.24	0.83	0.92	0.93	1.12	0.92	1.17	0.84	1.03	43.75	8.54
O54984	ATPase Asna1 OS=Mus musculus GN=Asna1 PE=1 SV=2 - [ASNA_MOUSE]	27.87	1	7	7	17	1.00	0.75	0.77	0.98	0.94	0.94	0.91	0.93	0.86	0.92	0.92	0.86	0.85	38.80	4.91
Q8C078	Calcium/calmodulin-dependent protein kinase kinase 2 OS=Mus musculus GN=Camkk2 PE=1 SV=2 - [KKCC2_MOUSE]	45.75	4	3	17	56	0.83	0.99	1.14	0.96	1.12	0.94	1.14	0.93	1.07	0.84	0.95	0.87	1.00	64.58	5.90

Q8BG92	Clavesin-2 OS=Mus musculus GN=Clvs2 PE=2 SV=1 - [CLVS2_MOUSE]	19.27	2	3	5	9	0.88	1.08	1.32	1.06	1.26	0.96	0.96	0.93	0.96	0.89	0.90	1.00	1.01	37.93	6.20
Q61655	ATP-dependent RNA helicase DDX19A OS=Mus musculus GN=Ddx19a PE=2 SV=2 - [DD19A_MOUSE]	21.97	5	8	8	18	1.00	0.92	0.97	0.98	1.02	0.92	0.92	0.93	0.95	0.96	0.99	0.96	1.11	53.90	6.67
Q6DFW4	Nucleolar protein 58 OS=Mus musculus GN=Nop58 PE=1 SV=1 - [NOP58_MOUSE]	7.28	1	3	3	5	1.04	1.00	0.90	1.14	1.09	1.01	0.96	0.93	0.89	1.00	0.96	0.94	0.90	60.30	8.34
Q8BWF0	Succinate-semialdehyde dehydrogenase, mitochondrial OS=Mus musculus GN=Aldh5a1 PE=1 SV=1 - [SSDH_MOUSE]	51.24	1	21	21	61	1.04	0.98	1.05	1.05	1.09	0.98	0.94	0.93	0.94	1.01	0.99	0.99	0.99	55.93	8.25
Q8BXA0	Leucine-rich repeat and fibronectin type-III domain-containing protein 5 OS=Mus musculus GN=Lrfn5 PE=1 SV=1 - [LRFN5_MOUSE]	7.23	2	3	3	5	0.99	1.03	1.11	1.05	1.16	0.84	0.84	0.93	1.18	1.05	1.04	0.95	0.97	79.32	7.24
Q9JJ28	Protein flightless-1 homolog OS=Mus musculus GN=Flii PE=1 SV=1 - [FLII_MOUSE]	4.25	1	4	4	9	0.97	1.09	1.06	1.01	1.08	0.91	0.94	0.93	0.97	0.98	1.04	1.04	1.02	144.71	6.06
Q8C015	Serine/threonine-protein kinase PAK 7 OS=Mus musculus GN=Pak7 PE=1 SV=1 - [PAK7_MOUSE]	21.14	2	10	12	25	0.88	1.06	1.28	1.03	1.11	1.01	1.12	0.93	1.11	0.90	1.03	0.88	0.98	80.90	8.21
Q8BG94	COMM domain-containing protein 7 OS=Mus musculus GN=Commd7 PE=2 SV=1 - [COMD7_MOUSE]	26.00	2	5	5	9	0.96	0.96	0.89	0.95	1.01	0.99	1.01	0.93	0.93	0.94	0.98	1.05	1.00	22.64	5.94
E9QNN1	ATP-dependent RNA helicase A OS=Mus musculus GN=Dhx9 PE=2 SV=1 - [E9QNN1_MOUSE]	20.81	4	20	20	61	1.04	1.04	1.09	1.07	1.05	0.98	0.99	0.93	0.94	0.98	0.97	1.06	1.11	149.60	6.83
Q91VR5	ATP-dependent RNA helicase DDX1 OS=Mus musculus GN=Ddx1 PE=1 SV=1 - [DDX1_MOUSE]	30.27	1	17	17	31	0.92	0.87	0.95	0.96	1.03	0.96	1.00	0.93	1.02	0.92	0.98	0.93	1.00	82.45	7.21
E9PVA8	Protein Gcn1l1 OS=Mus musculus GN=Gcn1l1 PE=2 SV=1 - [E9PVA8_MOUSE]	4.04	2	8	8	12	0.90	0.98	1.02	1.01	1.19	0.91	1.00	0.93	0.98	1.00	1.11	1.01	1.13	292.83	7.36
F8WGT1	Adenosylhomocysteinase OS=Mus musculus GN=Ahcyl2 PE=2 SV=1 - [F8WGT1_MOUSE]	30.23	9	2	21	82	0.96	0.96	1.69	1.00	1.05	1.01	1.05	0.93	0.98	1.04	1.09	0.83	0.87	66.73	7.36

P62281	40S ribosomal protein S11 OS=Mus musculus GN=Rps11 PE=2 SV=3 - [RS11_MOUSE]	27.85	1	6	6	15	0.96	1.12	1.15	1.05	1.11	0.95	1.00	0.93	1.01	0.98	1.03	0.98	1.10	18.42	10.30
Q9D186	Apolipoprotein O OS=Mus musculus GN=Apoo PE=2 SV=1 - [Q9D186_MOUSE]	31.71	3	4	4	16	1.08	1.12	1.00	1.08	0.99	0.96	0.90	0.93	0.89	1.03	0.92	0.97	0.94	18.79	8.37
Q922B1	O-acetyl-ADP-ribose deacetylase MACROD1 OS=Mus musculus GN=Macrod1 PE=2 SV=2 - [MACD1_MOUSE]	20.74	1	5	5	11	0.86	0.89	1.05	0.92	1.04	0.92	1.02	0.93	1.05	0.89	0.99	0.97	0.92	35.27	8.85
O54750	Cytochrome P450 2J6 OS=Mus musculus GN=Cyp2j6 PE=2 SV=2 - [CP2J6_MOUSE]	3.79	9	2	2	4	1.02	1.17	1.15	1.01	0.99	0.87	0.85	0.93	0.91	0.98	0.96	1.12	1.10	57.75	8.44
Q9JLN9	Serine/threonine-protein kinase mTOR OS=Mus musculus GN=Mtor PE=1 SV=2 - [MTOR_MOUSE]	9.65	2	17	17	35	1.01	1.20	1.13	1.13	1.13	0.91	0.92	0.93	0.88	0.99	1.00	1.02	1.03	288.60	7.17
Q9JJV2	Profilin-2 OS=Mus musculus GN=Pfn2 PE=1 SV=3 - [PROF2_MOUSE]	55.71	4	7	7	106	0.89	0.96	1.11	1.01	1.11	0.90	0.97	0.93	0.97	0.92	1.00	0.93	1.07	15.02	6.99
Q9Z1B3	1-phosphatidylinositol 4,5-bisphosphate phosphodiesterase beta-1 OS=Mus musculus GN=Plcb1 PE=1 SV=2 - [PLCB1_MOUSE]	59.46	9	60	60	238	0.97	1.01	1.01	1.04	1.08	0.98	1.02	0.93	0.93	0.95	0.98	0.93	0.97	138.31	6.13
Q91V64	Isochorismatase domain-containing protein 1 OS=Mus musculus GN=Isoc1 PE=2 SV=1 - [ISOC1_MOUSE]	13.47	1	3	3	5	1.12	1.14	1.03	1.19	1.07	1.04	0.94	0.93	0.84	1.16	1.04	1.22	1.10	32.01	7.39
Q3UEB3-	Isoform 3 of Poly(U)-binding-splicing factor PUF60 OS=Mus musculus GN=Puf60 - [PUF60_MOUSE]	26.85	3	10	10	21	0.96	1.10	1.20	0.99	1.00	0.96	0.97	0.93	0.93	0.99	1.01	0.90	0.97	53.99	5.33
Q3TKT4	Transcription activator BRG1 OS=Mus musculus GN=Smarca4 PE=1 SV=1 - [SMCA4_MOUSE]	6.94	4	4	10	16	0.97	1.01	1.09	0.94	1.09	0.92	1.00	0.93	1.04	0.94	1.00	1.09	1.11	181.31	8.00
Q3UHD6-	Isoform 2 of Sorting nexin-27 OS=Mus musculus GN=Snx27 - [SNX27_MOUSE]	25.48	5	9	9	30	1.08	1.00	0.84	1.15	1.06	0.87	0.80	0.93	0.83	0.96	0.94	0.93	0.89	59.50	6.24
Q9EPU0-2	Isoform 2 of Regulator of nonsense transcripts 1 OS=Mus musculus GN=Upf1 - [RENT1_MOUSE]	23.99	2	19	19	37	1.02	1.02	0.98	1.05	0.98	0.97	0.97	0.93	0.94	0.95	0.98	0.94	0.96	122.58	6.68

Q9CRA5	Golgi phosphoprotein 3 OS=Mus musculus GN=Golph3 PE=2 SV=1 - [GOLP3_MOUSE]	4.36	1	1	1	3	0.98	0.85	0.87	1.12	1.15	1.02	1.04	0.93	0.95	1.11	1.13	0.87	0.89	33.73	6.44
D3Z074	Protein diaphanous homolog 1 OS=Mus musculus GN=Diap1 PE=2 SV=2 - [D3Z074_MOUSE]	3.69	5	4	4	7	0.97	0.96	0.92	1.08	1.04	0.95	1.00	0.93	0.94	1.01	1.04	0.99	1.01	135.74	5.34
Q8C129	Leucyl-cystinyl aminopeptidase OS=Mus musculus GN=Lnpep PE=1 SV=1 - [LCAP_MOUSE]	6.83	1	5	5	22	0.92	1.07	1.10	0.89	0.99	0.95	1.00	0.93	1.03	0.94	0.98	0.97	0.89	117.23	5.96
Q8QZS1	3-hydroxyisobutyryl-CoA hydrolase, mitochondrial OS=Mus musculus GN=Hibch PE=1 SV=1 - [HIBCH_MOUSE]	33.25	2	11	11	26	1.07	0.97	0.90	1.15	1.08	1.03	0.96	0.93	0.82	1.03	1.03	1.05	1.06	43.01	8.06
D3YVS7	Uncharacterized protein (Fragment) OS=Mus musculus GN=Cystm1 PE=2 SV=1 - [D3YVS7_MOUSE]	11.90	2	1	1	2	1.10	0.98	0.88	0.95	0.86	0.98	0.89	0.93	0.84	1.03	0.93	1.04	0.95	9.16	4.44
Q9EQH3	Vacuolar protein sorting-associated protein 35 OS=Mus musculus GN=Vps35 PE=1 SV=1 - [VPS35_MOUSE]	26.63	1	20	20	32	1.14	1.11	0.92	1.14	0.94	0.91	0.76	0.93	0.77	0.98	0.86	1.10	1.01	91.65	5.44
Q8C754	Vacuolar protein sorting-associated protein 52 homolog OS=Mus musculus GN=Vps52 PE=2 SV=1 - [VPS52_MOUSE]	17.98	3	8	8	12	1.04	1.16	0.99	1.22	1.18	0.97	0.91	0.93	0.93	0.85	0.94	1.08	1.14	81.99	5.90
Q60634-3	Isoform 3 of Flotillin-2 OS=Mus musculus GN=Flot2 - [FLOT2_MOUSE]	48.28	4	16	16	37	1.00	1.12	1.12	1.10	1.07	0.91	0.91	0.93	0.93	1.04	1.04	1.06	1.11	41.63	5.22
Q7TNR6	Immunoglobulin superfamily member 21 OS=Mus musculus GN=Igsf21 PE=2 SV=1 - [IGS21_MOUSE]	19.02	1	7	7	20	1.10	0.97	0.86	0.95	0.89	0.94	0.94	0.93	0.92	0.91	0.84	1.00	0.93	51.90	6.95
Q9CXR1	Dehydrogenase/reductase SDR family member 7 OS=Mus musculus GN=Dhrs7 PE=2 SV=2 - [DHRS7_MOUSE]	2.96	1	1	1	1	0.92	1.16	1.26	1.24	1.34	0.88	0.95	0.93	1.00	1.01	1.10	1.02	1.10	38.14	8.32
D3Z6I4	Crystallin, zeta (Quinone reductase)-like 1, isoform CRA_h OS=Mus musculus GN=Cryzl1 PE=4 SV=1 - [D3Z6I4_MOUSE]	10.51	6	3	3	5	1.01	0.95	0.91	1.08	1.07	0.95	0.95	0.93	0.90	1.02	0.99	1.04	1.01	36.98	6.09

Q7TSQ8	Pyruvate dehydrogenase phosphatase regulatory subunit, mitochondrial OS=Mus musculus GN=Pdpr PE=2 SV=1 - [PDPR_MOUSE]	11.16	2	8	8	16	1.07	0.94	0.96	1.06	1.05	0.98	0.92	0.93	0.90	0.98	0.93	0.95	0.97	99.17	6.35
E0CXA9	MOB-like protein phocein OS=Mus musculus GN=Mob4 PE=2 SV=1 - [E0CXA9_MOUSE]	35.78	4	5	5	8	1.01	1.06	1.01	1.06	0.98	0.88	0.88	0.93	0.89	0.86	0.85	0.95	0.97	23.50	6.92
Q9Z1K6	E3 ubiquitin-protein ligase ARIH2 OS=Mus musculus GN=Arih2 PE=2 SV=1 - [ARI2_MOUSE]	3.25	1	1	1	1	1.07	0.97	0.90	1.27	1.18	1.15	1.06	0.93	0.87	1.01	0.95	0.93	0.87	57.66	5.69
O55242-2	Isoform 2 of Sigma non-opioid intracellular receptor 1 OS=Mus musculus GN=Sigmar1 - [SGMR1_MOUSE]	4.17	2	1	1	2	0.98	1.19	1.21	1.11	1.14	1.06	1.08	0.93	0.95	0.95	0.97	1.03	1.05	21.62	5.94
Q8QZY9	Splicing factor 3B subunit 4 OS=Mus musculus GN=Sf3b4 PE=2 SV=1 - [SF3B4_MOUSE]	19.10	1	4	4	15	0.90	0.90	1.01	0.84	0.92	1.01	1.13	0.93	1.09	0.86	0.97	0.94	1.00	44.33	8.56
Q922F4	Tubulin beta-6 chain OS=Mus musculus GN=Tubb6 PE=1 SV=1 - [TBB6_MOUSE]	38.93	1	2	17	652	0.94	1.00	0.97	1.05	1.08	1.06	1.03	0.93	1.10	0.93	0.90	1.12	1.04	50.06	4.89
Q69ZK9	Neuroligin-2 OS=Mus musculus GN=Nlgn2 PE=1 SV=2 - [NLGN2_MOUSE]	21.17	2	11	14	38	1.01	0.96	0.92	1.04	1.04	0.89	0.84	0.93	0.89	0.94	0.95	0.95	0.94	90.93	6.18
Q8C2E7	WASH complex subunit strumpellin OS=Mus musculus GN=Kiaa0196 PE=2 SV=2 - [STRUM_MOUSE]	5.18	2	5	5	7	1.03	1.15	1.24	1.05	1.14	0.98	1.09	0.93	1.03	0.95	1.04	1.07	1.17	134.02	7.12
Q6KAR6	Exocyst complex component 3 OS=Mus musculus GN=Exoc3 PE=1 SV=2 - [EXOC3_MOUSE]	15.63	1	9	10	16	0.98	1.05	1.15	1.10	1.10	0.92	0.92	0.93	0.88	0.91	0.96	0.97	0.99	86.40	6.20
Q99N15	17beta-hydroxysteroid dehydrogenase type 10/short chain L-3-hydroxyacyl-CoA dehydrogenase OS=Mus musculus GN=Hsd17b10 PE=2 SV=1 - [Q99N15_MOUSE]	56.70	3	11	11	42	1.01	1.05	1.02	1.11	1.13	0.93	0.93	0.93	0.92	1.01	0.98	0.99	1.01	27.26	8.76
P62196	26S protease regulatory subunit 8 OS=Mus musculus GN=Psmc5 PE=1 SV=1 - [PRS8_MOUSE]	45.07	2	14	15	37	1.04	0.95	0.94	1.08	1.03	0.94	0.91	0.93	0.90	0.98	0.91	0.94	0.97	45.60	7.55

Q03137	Ephrin type-A receptor 4 OS=Mus musculus GN=Epha4 PE=1 SV=2 - [EPHA4_MOUSE]	22.11	6	13	15	45	0.97	1.14	1.10	1.12	1.21	0.89	0.94	0.93	0.98	0.97	1.00	0.97	0.97	109.74	6.51
Q8BL99-5	Isoform 5 of Protein dopey-1 OS=Mus musculus GN=Dopey1 - [DOP1_MOUSE]	0.42	4	1	1	2	1.16	1.40	1.20	1.00	0.86	0.95	0.81	0.93	0.80	0.99	0.86	1.31	1.13	268.12	6.21
Q8K3C3	Protein LZIC OS=Mus musculus GN=Lzic PE=1 SV=1 - [LZIC_MOUSE]	30.53	2	2	5	14	0.99	0.66	0.80	0.98	0.91	0.94	1.01	0.93	0.87	0.87	0.88	0.81	0.87	21.52	4.96
D3YVM8	Biogenesis of lysosome-related organelles complex 1 subunit 1 OS=Mus musculus GN=Bloc1s1 PE=2 SV=1 - [D3YVM8_MOUSE]	8.26	2	1	1	2	1.02	0.98	0.96	0.98	0.97	0.95	0.93	0.93	0.91	0.96	0.94	0.92	0.90	13.89	6.79
Q9CWB7	Glutaredoxin-like protein C5orf63 homolog OS=Mus musculus PE=1 SV=1 - [YD286_MOUSE]	33.91	1	3	3	5	1.45	0.83	0.63	0.94	0.66	1.00	0.65	0.93	0.70	0.84	0.58	0.97	0.76	13.42	9.13
Q80U28-3	Isoform 3 of MAP kinase-activating death domain protein OS=Mus musculus GN=Madd - [MADD_MOUSE]	14.83	28	17	17	34	0.96	1.11	1.17	1.13	1.20	0.93	0.94	0.93	0.99	0.99	1.04	1.08	1.15	173.17	6.16
Q9Z0N1	Eukaryotic translation initiation factor 2 subunit 3, X-linked OS=Mus musculus GN=Eif2s3x PE=1 SV=2 - [IF2G_MOUSE]	13.77	3	5	5	12	1.08	0.90	0.90	1.06	0.95	0.90	0.80	0.93	0.90	0.86	0.84	0.92	0.86	51.03	8.40
Q8C996	Transmembrane protein 163 OS=Mus musculus GN=Tmem163 PE=1 SV=1 - [TM163_MOUSE]	4.86	1	1	1	33	1.02	1.28	1.17	1.09	1.05	0.91	0.82	0.93	0.91	0.97	0.98	0.95	0.90	31.17	7.93
Q6PEE3	Ribonucleoside-diphosphate reductase subunit M2 B OS=Mus musculus GN=Rrm2b PE=1 SV=1 - [RIR2B_MOUSE]	7.12	4	2	2	4	0.92	1.10	1.18	1.02	1.10	0.93	1.01	0.93	1.01	0.93	1.01	0.95	1.03	40.78	5.01
Q3TXX4	Vesicular glutamate transporter 1 OS=Mus musculus GN=Slc17a7 PE=2 SV=2 - [VGLU1_MOUSE]	15.89	1	8	8	84	1.02	1.02	1.03	1.10	1.01	0.93	0.87	0.93	0.90	0.91	0.89	0.99	0.92	61.60	7.34
Q8BQU3	Protein ACN9 homolog, mitochondrial OS=Mus musculus GN=Acn9 PE=2 SV=1 - [ACN9_MOUSE]	20.80	1	3	3	4	1.06	0.84	0.88	0.89	0.80	0.88	0.83	0.93	0.88	0.73	0.74	0.82	0.86	14.46	9.07
P97490	Adenylate cyclase type 8 OS=Mus musculus GN=Adcy8 PE=2 SV=2 - [ADCY8_MOUSE]	0.64	1	1	1	1	0.95	1.04	1.09	1.06	1.12	0.86	0.90	0.93	0.98	0.96	1.02	1.06	1.12	140.01	6.93

Q8CCK0	Core histone macro-H2A.2 OS=Mus musculus GN=H2afy2 PE=1 SV=3 - [H2AW_MOUSE]	19.89	1	5	7	23	1.01	1.08	1.14	1.08	1.07	1.00	1.03	0.93	0.90	1.02	1.00	1.04	1.05	40.07	9.69
Q6ZWX6	Eukaryotic translation initiation factor 2 subunit 1 OS=Mus musculus GN=Eif2s1 PE=1 SV=3 - [IF2A_MOUSE]	33.02	1	9	9	19	1.04	1.14	1.11	1.05	0.99	0.98	0.96	0.93	0.96	0.98	0.97	0.98	0.97	36.09	5.08
B8JK39	Protein Itga9 OS=Mus musculus GN=Itga9 PE=2 SV=1 - [B8JK39_MOUSE]	0.87	1	1	1	1	0.96	1.14	1.18	0.92	0.95	0.93	0.96	0.93	0.96	0.93	0.97	0.97	1.01	114.34	6.07
P57780	Alpha-actinin-4 OS=Mus musculus GN=Actn4 PE=1 SV=1 - [ACTN4_MOUSE]	58.44	3	15	45	277	0.84	0.83	0.99	0.97	1.12	0.93	1.11	0.93	1.10	0.90	1.04	0.80	0.98	104.91	5.41
P59999	Actin-related protein 2/3 complex subunit 4 OS=Mus musculus GN=Arpc4 PE=1 SV=3 - [ARPC4_MOUSE]	48.21	2	7	7	16	0.96	1.06	1.02	1.07	1.10	0.92	0.93	0.93	0.79	0.89	0.85	1.06	1.01	19.65	8.43
Q922B2	Aspartate--tRNA ligase, cytoplasmic OS=Mus musculus GN=Dars PE=2 SV=2 - [SYDC_MOUSE]	41.52	2	17	17	62	1.07	1.01	1.05	1.06	1.00	0.96	0.90	0.93	0.86	0.98	0.92	1.03	0.98	57.11	6.49
Q9DCJ1	Target of rapamycin complex subunit LST8 OS=Mus musculus GN=Mlst8 PE=1 SV=1 - [LST8_MOUSE]	12.27	1	3	3	5	1.07	1.15	1.07	0.90	0.79	1.08	0.96	0.93	0.91	0.89	0.83	1.10	1.14	35.83	5.86
Q9D1C2	Protein chibby homolog 1 OS=Mus musculus GN=Cby1 PE=2 SV=1 - [CBY1_MOUSE]	14.17	6	1	2	2	1.25	1.03	0.83	0.93	0.75	0.89	0.71	0.93	0.75	0.99	0.80	0.85	0.68	14.53	9.35
G3UYP4	Potassium voltage-gated channel subfamily KQT member 5 OS=Mus musculus GN=Kcnq5 PE=2 SV=1 - [G3UYP4_MOUSE]	5.35	4	3	4	8	0.91	1.00	1.11	1.01	1.08	0.94	1.03	0.93	1.03	1.00	1.08	0.86	0.92	90.00	9.42
F8VPU2	FERM, RhoGEF and pleckstrin domain-containing protein 1 OS=Mus musculus GN=Farp1 PE=1 SV=1 - [FARP1_MOUSE]	15.17	2	9	10	30	1.04	1.07	0.96	1.03	1.00	0.91	0.89	0.93	0.94	0.89	0.88	0.96	0.94	118.80	7.88
Q8R574	Phosphoribosyl pyrophosphate synthase-associated protein 2 OS=Mus musculus GN=Prpsap2 PE=1 SV=1 - [KPRB_MOUSE]	38.48	2	7	9	20	0.99	1.05	1.02	1.07	1.05	1.00	0.97	0.93	0.96	1.12	1.07	1.14	1.16	40.85	7.17

Q8BT60	Copine-3 OS=Mus musculus GN=Cpne3 PE=1 SV=2 - [CPNE3_MOUSE]	8.07	1	3	4	7	1.11	1.13	1.02	1.09	1.01	0.92	0.83	0.93	0.84	0.97	0.90	1.03	0.95	59.55	5.78
P09528	Ferritin heavy chain OS=Mus musculus GN=Fth1 PE=1 SV=2 - [FRIH_MOUSE]	54.95	1	8	8	37	0.87	0.74	0.81	0.82	0.91	0.90	1.03	0.93	1.04	0.83	0.98	0.97	1.03	21.05	5.88
P06801	NADP-dependent malic enzyme OS=Mus musculus GN=Me1 PE=1 SV=2 - [MAOX_MOUSE]	27.27	1	10	10	18	1.07	1.04	0.91	1.09	1.01	0.94	0.91	0.93	0.86	0.92	0.89	1.00	0.94	63.91	7.44
Q9CRC8	Leucine-rich repeat-containing protein 40 OS=Mus musculus GN=Lrrc40 PE=2 SV=2 - [LRC40_MOUSE]	7.14	5	4	4	6	1.05	1.20	1.09	1.12	1.06	0.99	0.93	0.93	0.88	1.12	1.06	1.12	1.04	68.03	6.89
D3Z205	Tumor necrosis factor alpha-induced protein 8 (Fragment) OS=Mus musculus GN=Tnfaip8 PE=2 SV=1 - [D3Z205_MOUSE]	25.97	8	1	1	2	1.01	0.99	0.98	0.84	0.83	1.04	1.02	0.93	0.92	0.94	0.93	1.13	1.11	8.42	4.88
Q8BLE7	Vesicular glutamate transporter 2 OS=Mus musculus GN=Slc17a6 PE=1 SV=1 - [VGLU2_MOUSE]	17.35	1	6	6	30	1.10	0.88	0.86	1.20	1.08	0.95	0.84	0.93	0.89	1.07	1.05	1.00	0.91	64.52	6.68
Q68FL0	Slc8a1 protein OS=Mus musculus GN=Slc8a1 PE=2 SV=1 - [Q68FL0_MOUSE]	20.25	4	15	16	50	1.05	1.01	1.00	1.09	1.00	0.98	0.94	0.93	0.91	1.00	0.97	0.99	0.96	106.60	5.00
P70202	Latexin OS=Mus musculus GN=Lxn PE=1 SV=2 - [LXN_MOUSE]	17.57	1	3	3	17	0.97	1.03	1.03	1.02	1.07	0.92	0.93	0.93	0.93	0.89	0.89	0.90	0.94	25.48	5.74
Q3UIL6-3	Isoform 3 of Pleckstrin homology domain-containing family A member 7 OS=Mus musculus GN=Plekha7 - [PKHA7_MOUSE]	1.88	6	1	1	6	1.03	0.94	0.90	1.03	0.95	0.86	0.91	0.93	0.99	0.88	0.86	0.89	0.91	114.59	9.61
Q6P8X1	Sorting nexin-6 OS=Mus musculus GN=Snx6 PE=1 SV=2 - [SNX6_MOUSE]	20.69	1	8	10	27	0.94	0.88	0.92	0.97	1.00	0.97	1.01	0.93	1.05	0.90	1.00	0.88	0.93	46.62	6.16
P70206	Plexin-A1 OS=Mus musculus GN=Plxna1 PE=1 SV=1 - [PLXA1_MOUSE]	10.72	2	10	18	31	0.95	1.16	1.10	1.01	1.07	0.86	0.92	0.93	0.93	0.97	1.02	0.97	1.01	210.96	6.90
Q8C413	Diacylglycerol kinase gamma OS=Mus musculus GN=Dgkg PE=2 SV=1 - [Q8C413_MOUSE]	10.28	3	7	7	11	1.13	0.99	0.85	1.06	0.94	1.04	0.91	0.93	0.79	1.00	0.89	1.16	1.03	83.92	6.39
Q8BGQ7	Alanine--tRNA ligase, cytoplasmic OS=Mus musculus GN=Aars PE=1 SV=1 - [SYAC_MOUSE]	34.50	1	24	24	68	0.99	1.04	1.09	1.08	1.09	0.99	0.98	0.94	0.92	0.96	0.94	1.00	1.00	106.84	5.67

Q6NS60	F-box only protein 41 OS=Mus musculus GN=Fbxo41 PE=1 SV=3 - [FBX41_MOUSE]	11.91	1	9	10	21	0.97	1.06	1.10	1.22	1.21	1.02	1.01	0.94	0.96	0.99	0.99	0.98	1.06	94.27	8.24
O70589	Peripheral plasma membrane protein CASK OS=Mus musculus GN=Cask PE=1 SV=2 - [CSKP_MOUSE]	32.29	11	23	23	61	1.03	0.89	0.90	1.02	1.01	0.91	0.92	0.94	0.94	0.86	0.89	0.99	1.00	105.04	6.43
P08775	DNA-directed RNA polymerase II subunit RPB1 OS=Mus musculus GN=Polr2a PE=1 SV=3 - [RPB1_MOUSE]	2.39	1	3	3	5	1.06	1.02	0.95	1.10	1.04	0.89	0.85	0.94	0.86	0.97	0.94	1.00	0.93	217.04	7.37
Q8BX17	Gem-associated protein 5 OS=Mus musculus GN=Gemin5 PE=1 SV=2 - [GEMI5_MOUSE]	1.53	3	2	2	2	0.99	1.57	1.59	1.11	1.13	0.79	0.79	0.94	0.94	0.99	1.00	0.93	0.95	166.49	6.71
Q8BM41	Protein LSM14 homolog B OS=Mus musculus GN=Lsm14b PE=2 SV=1 - [Q8BM41_MOUSE]	16.72	1	1	4	11	0.96	0.60	0.62	0.87	0.90	0.95	0.98	0.94	0.97	0.85	0.89	0.78	0.81	33.23	9.74
Q9CZG9	PDZ domain-containing protein 11 OS=Mus musculus GN=Pdzd11 PE=1 SV=1 - [PDZ11_MOUSE]	38.57	1	3	4	7	0.96	0.64	0.66	0.98	1.08	1.01	1.08	0.94	0.97	0.92	0.94	0.81	0.84	16.17	7.14
Q8BGX2	Uncharacterized protein C19orf52 homolog OS=Mus musculus PE=2 SV=1 - [CS052_MOUSE]	19.17	1	3	3	6	1.04	1.33	1.31	0.95	0.92	1.06	0.98	0.94	0.90	1.05	1.02	1.13	1.05	29.40	6.73
Q99J77	N-acetylneuraminic acid synthase (Sialic acid synthase) OS=Mus musculus GN=Nans PE=2 SV=1 - [Q99J77_MOUSE]	18.38	1	4	4	9	0.95	0.81	0.93	0.89	0.97	1.07	1.07	0.94	0.96	0.96	0.96	1.01	1.12	40.00	7.06
Q9D051	Pyruvate dehydrogenase E1 component subunit beta, mitochondrial OS=Mus musculus GN=Pdhb PE=1 SV=1 - [ODPB_MOUSE]	69.08	1	20	20	156	0.95	0.89	0.99	1.02	1.10	0.97	1.02	0.94	0.98	0.97	1.03	1.01	1.12	38.91	6.87
G3UYZ1	Immunoglobulin superfamily member 8 OS=Mus musculus GN=Igsf8 PE=2 SV=1 - [G3UYZ1_MOUSE]	36.86	3	13	13	122	1.02	1.14	1.12	1.06	1.01	0.93	0.92	0.94	0.92	1.00	0.98	0.99	0.98	58.10	7.91
Q62172	RalA-binding protein 1 OS=Mus musculus GN=Ralbp1 PE=1 SV=4 - [RBP1_MOUSE]	15.59	1	7	7	12	0.89	0.87	1.06	0.97	1.09	0.91	1.05	0.94	1.13	0.96	1.10	0.91	1.09	75.00	5.92

Q99JI4	26S proteasome non-ATPase regulatory subunit 6 OS=Mus musculus GN=Psmd6 PE=1 SV=1 - [PSMD6_MOUSE]	20.57	1	8	8	22	1.08	1.07	1.09	1.15	1.08	0.95	0.86	0.94	0.89	1.03	0.99	1.11	1.05	45.51	5.52
Q91Z61	GTP-binding protein Di-Ras1 OS=Mus musculus GN=Diras1 PE=2 SV=1 - [DIRA1_MOUSE]	29.29	2	4	6	11	1.04	1.05	0.99	1.12	1.07	1.02	0.98	0.94	0.91	1.02	0.98	1.00	1.01	22.25	8.90
Q3UGY8	Brefeldin A-inhibited guanine nucleotide-exchange protein 3 OS=Mus musculus GN=Arfgef3 PE=1 SV=1 - [BIG3_MOUSE]	8.16	1	12	12	28	0.84	1.14	1.26	1.08	1.26	0.88	1.05	0.94	1.13	1.00	1.19	1.00	1.20	239.94	5.88
Q8VED2	Biogenesis of lysosome-related organelles complex 1 subunit 4 OS=Mus musculus GN=Bloc1s4 PE=1 SV=1 - [BL1S4_MOUSE]	14.88	1	1	1	1	1.10	0.84	0.77	0.82	0.75	1.11	1.00	0.94	0.85	1.04	0.95	0.73	0.66	23.10	5.14
D3YXA2	Lariat debranching enzyme (Fragment) OS=Mus musculus GN=Dbr1 PE=2 SV=1 - [D3YXA2_MOUSE]	4.33	2	1	1	2	1.11	1.19	1.07	0.99	0.89	0.91	0.82	0.94	0.84	1.02	0.92	0.97	0.87	26.17	7.21
P63073	Eukaryotic translation initiation factor 4E OS=Mus musculus GN=Eif4e PE=1 SV=1 - [IF4E_MOUSE]	25.35	1	5	5	12	0.93	0.69	0.74	0.99	1.02	1.04	1.02	0.94	0.98	0.86	0.95	0.90	0.99	25.04	6.15
P26516	26S proteasome non-ATPase regulatory subunit 7 OS=Mus musculus GN=Psmd7 PE=1 SV=2 - [PSMD7_MOUSE]	28.35	1	5	5	16	0.92	0.95	0.88	0.88	1.07	0.82	1.01	0.94	0.97	1.01	0.98	0.88	1.06	36.52	6.77
Q9Z247	Peptidyl-prolyl cis-trans isomerase FKBP9 OS=Mus musculus GN=Fkbp9 PE=1 SV=1 - [FKBP9_MOUSE]	4.56	1	2	2	2	0.95	0.96	1.01	0.98	1.04	0.83	0.87	0.94	0.98	0.95	1.01	0.99	1.04	62.96	5.21
Q9WUM4	Coronin-1C OS=Mus musculus GN=Coro1c PE=1 SV=2 - [COR1C_MOUSE]	41.56	8	15	16	51	0.97	0.83	0.94	0.94	0.99	0.89	0.95	0.94	0.94	0.92	0.95	0.90	0.97	53.09	7.08
Q8VC88	Grancalcin OS=Mus musculus GN=Gca PE=2 SV=1 - [GRAN_MOUSE]	5.00	1	1	1	2	1.05	1.03	0.98	1.17	1.11	1.01	0.96	0.94	0.89	0.96	0.91	0.99	0.95	24.65	5.07
Q9CWJ9	Bifunctional purine biosynthesis protein PURH OS=Mus musculus GN=Atic PE=1 SV=2 - [PUR9_MOUSE]	41.89	1	17	17	30	1.11	1.10	1.02	1.09	1.00	0.96	0.88	0.94	0.89	0.97	0.89	1.09	0.96	64.18	6.76

D3YW26	Protein Tada2b OS=Mus musculus GN=Tada2b PE=2 SV=1 - [D3YW26_MOUSE]	9.28	2	1	1	10	1.45	1.25	0.86	1.17	0.81	1.08	0.74	0.94	0.64	0.67	0.46	1.40	0.97	39.98	8.81
F8VPX1	Ubiquitin carboxyl-terminal hydrolase OS=Mus musculus GN=Usp7 PE=2 SV=1 - [F8VPX1_MOUSE]	24.48	7	19	19	48	1.02	1.06	1.03	1.05	1.07	0.97	0.97	0.94	0.93	0.95	0.94	1.01	0.97	128.39	5.55
Q8CAD1	Ras-binding protein DA-Raf OS=Mus musculus GN=Araf PE=2 SV=1 - [Q8CAD1_MOUSE]	22.58	3	3	3	8	1.00	1.12	1.09	1.00	1.00	0.98	0.97	0.94	0.92	1.05	1.07	1.00	1.01	20.87	8.47
Q9D1T0	Leucine-rich repeat and immunoglobulin-like domain-containing nogo receptor-interacting protein 1 OS=Mus musculus GN=Lingo1 PE=1 SV=1 - [LIGO1_MOUSE]	17.59	2	8	8	19	1.04	1.20	1.11	1.08	1.01	1.02	0.98	0.94	0.84	1.01	0.90	1.12	1.06	69.06	8.31
P68254	14-3-3 protein theta OS=Mus musculus GN=Ywhaq PE=1 SV=1 - [1433T_MOUSE]	68.98	6	13	19	500	1.00	0.93	0.97	0.97	0.99	0.97	0.98	0.94	0.95	0.95	0.96	0.91	0.93	27.76	4.78
Q61753	D-3-phosphoglycerate dehydrogenase OS=Mus musculus GN=Phgdh PE=1 SV=3 - [SERA_MOUSE]	32.27	2	15	15	40	1.01	1.11	1.00	1.11	1.11	1.00	0.96	0.94	0.91	1.08	1.06	1.14	1.14	56.55	6.54
Q8K1S2	Netrin receptor UNC5D OS=Mus musculus GN=Unc5d PE=2 SV=1 - [UNC5D_MOUSE]	1.05	1	1	1	1	1.00	0.87	0.87	1.03	1.03	0.91	0.90	0.94	0.93	0.91	0.91	1.00	1.01	106.28	6.14
P46664	Adenylosuccinate synthetase isozyme 2 OS=Mus musculus GN=Adss PE=1 SV=2 - [PURA2_MOUSE]	25.88	1	8	8	24	0.98	1.03	1.02	1.04	1.08	1.00	1.01	0.94	0.97	0.95	1.01	1.07	0.95	49.99	6.38
Q99JY9	Actin-related protein 3 OS=Mus musculus GN=Actr3 PE=1 SV=3 - [ARP3_MOUSE]	62.92	1	15	18	98	0.92	1.00	1.10	1.05	1.12	0.94	1.01	0.94	0.97	0.91	1.03	0.96	1.09	47.33	5.88
Q9WUB0	RanBP-type and C3HC4-type zinc finger-containing protein 1 OS=Mus musculus GN=Rbck1 PE=1 SV=2 - [HOIL1_MOUSE]	16.54	2	6	6	11	0.97	1.16	1.19	1.00	1.03	1.02	1.03	0.94	1.03	0.93	1.01	0.94	0.96	57.50	6.19

A2A4A6	1-phosphatidylinositol 4,5-bisphosphate phosphodiesterase gamma-1 OS=Mus musculus GN=Plcg1 PE=4 SV=1 - [A2A4A6_MOUSE]	5.58	4	6	6	11	1.07	1.08	1.03	1.03	0.95	0.92	0.85	0.94	0.81	0.99	0.84	1.09	0.98	148.44	5.99
Q9R171	Cerebellin-1 OS=Mus musculus GN=Cbln1 PE=1 SV=1 - [CBLN1_MOUSE]	17.10	1	2	4	11	1.25	0.99	0.78	1.01	0.78	0.95	0.69	0.94	0.69	1.04	0.75	0.98	0.78	21.10	7.28
Q5SUE8-2	Isoform 2 of Ankyrin repeat domain-containing protein 40 OS=Mus musculus GN=Ankrd40 - [ANR40_MOUSE]	10.25	4	2	3	5	1.02	0.89	1.00	0.98	0.99	0.86	1.00	0.94	0.88	0.78	0.91	0.91	0.90	35.74	5.16
E9QKZ2	Importin-9 OS=Mus musculus GN=Ipo9 PE=2 SV=1 - [E9QKZ2_MOUSE]	5.10	4	4	4	5	1.32	1.12	0.93	1.11	0.84	0.99	0.76	0.94	0.67	0.88	0.67	0.91	0.71	115.88	4.82
Q7TNF0	Double C2-like domain-containing protein alpha OS=Mus musculus GN=Doc2a PE=2 SV=1 - [DOC2A_MOUSE]	17.78	4	5	6	14	0.94	1.02	1.05	0.95	0.97	0.85	0.90	0.94	0.98	0.86	0.95	0.92	0.97	44.58	7.12
Q920Q6-3	Isoform 3 of RNA-binding protein Musashi homolog 2 OS=Mus musculus GN=Msi2 - [MSI2H_MOUSE]	20.21	5	4	4	6	0.91	0.69	0.94	0.98	1.05	0.82	0.90	0.94	1.03	0.90	1.05	0.97	1.07	30.87	9.10
Q91WK2	Eukaryotic translation initiation factor 3 subunit H OS=Mus musculus GN=Eif3h PE=1 SV=1 - [EIF3H_MOUSE]	28.41	1	9	9	14	1.03	0.85	0.86	1.05	0.99	1.03	0.90	0.94	0.90	0.95	0.88	0.97	0.90	39.81	6.67
Q9D1A2	Cytosolic non-specific dipeptidase OS=Mus musculus GN=Cndp2 PE=1 SV=1 - [CNDP2_MOUSE]	37.05	1	15	15	37	0.98	0.92	1.02	0.97	0.98	0.93	0.95	0.94	0.96	1.01	1.04	0.97	0.97	52.73	5.66
Q6PAK3	Protein arginine N-methyltransferase 8 OS=Mus musculus GN=Prmt8 PE=2 SV=2 - [ANM8_MOUSE]	10.91	1	2	4	13	0.93	1.03	1.00	1.13	1.20	0.98	1.04	0.94	1.04	0.94	1.00	0.90	0.99	45.25	6.93
Q80T41	Gamma-aminobutyric acid type B receptor subunit 2 OS=Mus musculus GN=Gabbr2 PE=2 SV=2 - [GABR2_MOUSE]	22.34	1	12	12	59	0.94	1.05	1.00	1.06	1.05	0.98	0.95	0.94	0.97	0.97	0.96	1.00	0.99	105.60	8.72

P68404	Protein kinase C beta type OS=Mus musculus GN=Prkcb PE=1 SV=4 - [KPCB_MOUSE]	46.20	5	1	27	117	0.73	0.74	1.00	1.04	1.42	0.86	1.16	0.94	1.27	0.94	1.29	0.75	1.03	76.70	7.01
A2ARW8	Protein Gm14399 OS=Mus musculus GN=Gm14399 PE=2 SV=1 - [A2ARW8_MOUSE]	9.84	40	1	2	2	0.98	0.73	0.75	0.88	0.90	0.94	0.96	0.94	0.95	0.97	0.99	0.79	0.81	70.85	9.29
Q9QUH0	Glutaredoxin-1 OS=Mus musculus GN=Glrx PE=1 SV=3 - [GLRX1_MOUSE]	57.94	1	4	4	34	0.91	0.87	0.89	0.79	0.83	0.98	1.00	0.94	0.93	0.89	0.90	0.84	0.82	11.86	8.37
D3Z4S3	Putative peptidyl-tRNA hydrolase PTRHD1 OS=Mus musculus GN=Ptrhd1 PE=3 SV=1 - [PTRD1_MOUSE]	39.29	1	5	5	15	0.90	0.86	0.93	0.91	0.96	1.00	1.03	0.94	0.97	0.92	0.96	0.84	0.97	16.03	9.32
Q8BG67	Protein EFR3 homolog A OS=Mus musculus GN=Efr3a PE=1 SV=1 - [EFR3A_MOUSE]	7.45	3	4	5	10	1.02	1.29	1.08	1.24	1.19	0.92	0.88	0.94	0.91	1.02	1.02	1.19	1.01	92.55	6.83
P31650	Sodium- and chloride-dependent GABA transporter 3 OS=Mus musculus GN=Slc6a11 PE=1 SV=2 - [S6A11_MOUSE]	8.61	2	6	6	31	1.16	1.15	0.88	1.14	0.92	0.89	0.79	0.94	0.79	1.17	1.01	0.99	0.85	69.91	6.98
Q8K1M3	Protein kinase, cAMP dependent regulatory, type II alpha OS=Mus musculus GN=Prkar2a PE=2 SV=1 - [Q8K1M3_MOUSE]	42.29	2	11	13	60	0.96	1.02	1.03	1.06	1.06	0.93	0.98	0.94	0.93	0.98	0.98	0.98	1.01	45.56	4.93
O88531	Palmitoyl-protein thioesterase 1 OS=Mus musculus GN=Ppt1 PE=2 SV=2 - [PPT1_MOUSE]	16.34	3	4	4	13	0.98	1.06	1.08	1.04	1.00	0.91	0.89	0.94	0.95	1.01	0.99	1.02	1.08	34.47	8.00
Q8VCE6	5'(3')-deoxyribonucleotidase, mitochondrial OS=Mus musculus GN=Nt5m PE=2 SV=1 - [NT5M_MOUSE]	15.91	1	3	3	4	1.09	0.85	0.78	1.00	0.92	1.04	0.95	0.94	0.86	1.07	0.98	1.00	0.92	25.59	8.35
Q9JKD3	Secretory carrier-associated membrane protein 5 OS=Mus musculus GN=Scamp5 PE=2 SV=1 - [SCAM5_MOUSE]	28.51	1	4	4	47	1.02	0.93	0.94	1.16	1.12	0.88	0.84	0.94	0.89	0.96	0.93	0.94	0.92	26.05	8.54
Q91VE0	Long-chain fatty acid transport protein 4 OS=Mus musculus GN=Slc27a4 PE=1 SV=1 - [S27A4_MOUSE]	15.55	1	7	7	13	0.99	1.06	1.20	1.08	1.11	0.87	0.88	0.94	0.95	0.96	0.95	1.06	1.04	72.27	8.59
P55264	Adenosine kinase OS=Mus musculus GN=Adk PE=1 SV=2 - [ADK_MOUSE]	36.57	2	10	10	18	1.06	0.88	0.81	1.06	1.03	0.96	0.93	0.94	0.95	0.87	0.91	0.94	1.01	40.12	6.21

Q920Q8-4	Isoform 4 of Influenza virus NS1A-binding protein homolog OS=Mus musculus GN=Ivns1abp - [NS1BP_MOUSE]	1.83	3	1	1	2	1.01	1.09	1.08	1.00	1.00	0.92	0.91	0.94	0.93	1.01	1.01	1.01	1.01	66.63	5.91
Q8BMQ2	General transcription factor 3C polypeptide 4 OS=Mus musculus GN=Gtf3c4 PE=1 SV=2 - [TF3C4_MOUSE]	4.65	4	2	2	3	0.93	1.22	1.31	0.85	0.99	1.04	1.12	0.94	1.01	1.09	1.08	1.05	1.14	91.55	6.73
E9QL23	Dystonin OS=Mus musculus GN=Dst PE=2 SV=1 - [E9QL23_MOUSE]	5.71	8	24	25	45	0.96	0.99	1.04	1.06	1.08	0.95	0.99	0.94	1.02	0.93	0.97	1.00	1.08	614.83	5.71
Q8CBH5-	Isoform 2 of Major facilitator superfamily domain-containing protein 6 OS=Mus musculus GN=Mfsd6 - [MFSD6_MOUSE]	4.78	4	3	3	5	1.20	1.04	0.87	0.97	0.81	0.97	0.77	0.94	0.79	1.02	0.87	0.99	0.83	85.70	5.72
P35486	Pyruvate dehydrogenase E1 component subunit alpha, somatic form, mitochondrial OS=Mus musculus GN=Pdha1 PE=1 SV=1 - [ODPA_MOUSE]	52.05	1	21	22	150	0.91	0.95	1.05	1.01	1.08	0.93	1.00	0.94	0.99	0.94	1.01	0.97	1.05	43.20	8.19
Q9CQ75	NADH dehydrogenase [ubiquinone] 1 alpha subcomplex subunit 2 OS=Mus musculus GN=Ndufa2 PE=1 SV=3 - [NDUA2_MOUSE]	58.59	1	6	6	42	0.86	1.01	1.17	0.87	1.03	0.90	1.06	0.94	1.13	0.91	1.06	0.97	1.11	10.91	9.99
P14094	Sodium/potassium-transporting ATPase subunit beta-1 OS=Mus musculus GN=Atp1b1 PE=1 SV=1 - [AT1B1_MOUSE]	48.03	1	16	16	266	1.01	0.98	0.94	1.10	1.11	0.92	0.92	0.94	0.94	0.96	0.96	0.95	0.95	35.17	8.65
J3QPB5	Nucleoporin NDC1 OS=Mus musculus GN=Tmem48 PE=4 SV=1 - [J3QPB5_MOUSE]	3.88	2	1	1	4	0.88	1.44	1.64	1.24	1.41	0.81	0.92	0.94	1.06	0.99	1.13	1.16	1.32	60.26	8.70
G3UYJ1	Mitochondrial translocator assembly and maintenance protein 41 homolog (Fragment) OS=Mus musculus GN=Tamm41 PE=2 SV=1 - [G3UYJ1_MOUSE]	10.48	3	1	1	1	1.15	1.25	1.08	1.05	0.91	0.77	0.66	0.94	0.81	0.98	0.85	1.28	1.12	14.04	7.34

Q80UJ7	Rab3 GTPase-activating protein catalytic subunit OS=Mus musculus GN=Rab3gap1 PE=2 SV=4 - [RB3GP_MOUSE]	16.11	1	13	13	26	0.93	1.04	1.16	1.07	1.16	0.90	0.95	0.94	1.00	1.01	1.08	1.05	1.11	110.13	5.73
O70172	Phosphatidylinositol 5-phosphate 4-kinase type-2 alpha OS=Mus musculus GN=Pip4k2a PE=1 SV=1 - [PI42A_MOUSE]	26.42	3	7	10	49	0.97	1.13	1.08	1.06	1.07	0.98	0.92	0.94	0.90	1.01	0.99	0.98	1.01	46.12	6.99
Q9QYS2	Metabotropic glutamate receptor 3 OS=Mus musculus GN=Grm3 PE=2 SV=1 - [GRM3_MOUSE]	25.37	1	17	18	56	0.96	1.01	1.03	1.13	1.17	0.94	0.96	0.94	0.96	0.97	0.98	0.95	0.97	99.05	7.75
Q9CQB5	CDGSH iron-sulfur domain-containing protein 2 OS=Mus musculus GN=Cisd2 PE=1 SV=1 - [CISD2_MOUSE]	21.48	2	2	2	8	1.01	0.85	0.85	1.07	1.01	1.03	0.99	0.94	0.93	1.04	1.06	0.93	0.90	15.23	9.51
Q14BI2	Metabotropic glutamate receptor 2 OS=Mus musculus GN=Grm2 PE=2 SV=2 - [GRM2_MOUSE]	19.72	1	12	14	34	0.89	1.08	1.26	1.21	1.33	0.85	0.96	0.94	1.06	0.87	1.03	0.90	1.07	95.83	8.06
E9Q8N5	CLIP-associating protein 2 OS=Mus musculus GN=Clasp2 PE=2 SV=1 - [E9Q8N5_MOUSE]	20.06	6	20	22	46	1.02	1.18	1.15	1.09	1.11	0.95	0.96	0.94	0.93	1.03	1.02	1.05	1.07	140.64	8.63
Q8R2U0-2	Isoform 2 of Nucleoporin SEH1 OS=Mus musculus GN=Seh1l - [SEH1_MOUSE]	9.24	2	2	2	5	0.94	1.17	1.16	1.14	1.13	1.22	1.31	0.94	0.96	0.90	1.01	0.95	1.08	39.40	7.88
P08113	Endoplasmin OS=Mus musculus GN=Hsp90b1 PE=1 SV=2 - [ENPL_MOUSE]	41.90	2	30	32	111	1.02	1.01	0.99	1.02	1.02	0.96	0.94	0.94	0.92	0.96	0.92	0.95	0.93	92.42	4.82
Q9QY81	Nuclear pore membrane glycoprotein 210 OS=Mus musculus GN=Nup210 PE=1 SV=2 - [PO210_MOUSE]	0.74	1	1	1	1	0.87	1.42	1.63	1.07	1.23	0.66	0.75	0.94	1.08	0.71	0.82	1.05	1.21	203.97	6.65
Q8BYM8	Probable cysteine--tRNA ligase, mitochondrial OS=Mus musculus GN=Cars2 PE=2 SV=2 - [SYCM_MOUSE]	7.44	2	3	3	6	1.01	1.22	1.20	1.02	1.00	0.99	1.00	0.94	0.94	1.10	1.09	1.08	1.07	61.23	8.18
Q9DBY8	Nuclear valosin-containing protein-like OS=Mus musculus GN=Nvl PE=1 SV=1 - [NVL_MOUSE]	0.70	1	1	1	1	0.86	0.94	1.09	1.02	1.17	0.93	1.07	0.94	1.08	1.02	1.18	1.05	1.22	94.42	6.35
Q3TYD4	Arylsulfatase G OS=Mus musculus GN=Arsg PE=2 SV=1 - [ARSG_MOUSE]	10.48	2	4	4	8	0.89	1.10	1.01	0.94	1.05	0.93	1.04	0.94	1.06	0.89	0.97	0.94	0.95	57.40	6.39

Q8CGZ0	Calcium homeostasis endoplasmic reticulum protein OS=Mus musculus GN=Cherp PE=1 SV=1 - [CHERP_MOUSE]	11.54	2	7	7	14	1.01	1.01	0.94	0.96	0.99	0.94	0.88	0.94	0.97	0.80	0.78	0.78	0.82	106.10	9.14
Q61136	Serine/threonine-protein kinase PRP4 homolog OS=Mus musculus GN=Prpf4b PE=1 SV=3 - [PRP4B_MOUSE]	7.45	1	6	7	11	0.86	0.80	0.98	0.85	0.90	0.96	0.98	0.94	1.08	0.95	1.15	1.05	1.06	116.90	10.23
Q149F3	Eukaryotic peptide chain release factor GTP-binding subunit ERF3B OS=Mus musculus GN=Gspt2 PE=1 SV=1 - [ERF3B_MOUSE]	30.70	2	4	16	50	1.21	1.05	0.77	0.95	0.85	1.00	0.73	0.94	0.79	0.94	0.77	1.13	0.90	69.10	5.20
D3YTP3	Protein Mtx3 OS=Mus musculus GN=Mtx3 PE=4 SV=2 - [D3YTP3_MOUSE]	25.00	1	5	5	15	1.16	0.97	0.86	1.13	0.98	0.93	0.83	0.94	0.88	0.92	0.79	0.95	0.92	34.87	7.99
P50429	Arylsulfatase B OS=Mus musculus GN=Arsb PE=2 SV=3 - [ARSB_MOUSE]	11.99	2	6	6	12	1.08	0.96	0.93	1.02	0.99	0.89	0.91	0.94	0.89	0.82	0.76	0.90	0.85	59.61	7.24
P61161	Actin-related protein 2 OS=Mus musculus GN=Actr2 PE=1 SV=1 - [ARP2_MOUSE]	50.51	1	14	14	69	0.95	1.03	1.00	1.06	1.07	0.95	0.99	0.94	0.97	0.95	1.03	1.02	1.07	44.73	6.74
P80317	T-complex protein 1 subunit zeta OS=Mus musculus GN=Cct6a PE=1 SV=3 - [TCPZ_MOUSE]	43.88	4	17	17	63	0.97	1.07	1.00	1.10	1.11	0.97	0.95	0.94	0.91	0.99	1.00	1.04	1.05	57.97	7.08
E9Q7G1	Protein Tmed7 OS=Mus musculus GN=Tmed7 PE=2 SV=1 - [E9Q7G1_MOUSE]	12.23	2	2	2	3	1.17	1.13	0.96	1.02	0.87	1.03	0.87	0.94	0.80	0.94	0.80	0.90	0.77	21.26	6.20
P62849-2	Isoform 2 of 40S ribosomal protein S24 OS=Mus musculus GN=Rps24 - [RS24_MOUSE]	26.15	3	3	3	9	1.03	1.05	1.19	1.00	1.11	0.97	0.91	0.94	0.95	1.00	1.00	1.13	1.10	15.06	10.89
E9QNF7	Contactin-associated protein-like 2 OS=Mus musculus GN=Cntnap2 PE=2 SV=1 - [E9QNF7_MOUSE]	21.92	3	20	20	48	0.99	1.09	1.17	1.03	1.04	0.91	0.91	0.94	0.94	0.95	0.99	0.98	1.02	148.16	6.76
Q9DAW6	U4/U6 small nuclear ribonucleoprotein Prp4 OS=Mus musculus GN=Prpf4 PE=2 SV=1 - [PRP4_MOUSE]	16.12	1	7	7	12	0.97	0.95	1.07	1.07	1.15	0.96	1.04	0.94	0.95	0.95	0.94	0.98	0.95	58.33	7.28
P70423	Cationic amino acid transporter 3 OS=Mus musculus GN=Slc7a3 PE=2 SV=1 - [CTR3_MOUSE]	2.75	1	1	1	2	1.47	1.33	0.91	1.21	0.82	1.22	0.83	0.94	0.64	1.16	0.79	1.15	0.79	67.42	7.37

Q8K0D7	Tail-anchored protein insertion receptor WRB OS=Mus musculus GN=Wrb PE=2 SV=1 - [WRB_MOUSE]	13.22	1	2	2	4	1.37	1.17	1.07	1.13	1.06	0.98	0.76	0.94	0.87	0.93	0.77	1.17	0.86	19.85	9.79
F6ZGI7	Molybdopterin synthase catalytic subunit OS=Mus musculus GN=Mocs2 PE=4 SV=1 - [F6ZGI7_MOUSE]	36.36	2	3	3	8	1.01	0.87	0.88	0.84	0.79	0.89	0.82	0.94	0.97	0.77	0.85	0.91	0.89	9.70	4.61
D3Z7R4	Synaptotagmin-1 (Fragment) OS=Mus musculus GN=Syt1 PE=2 SV=1 - [D3Z7R4_MOUSE]	32.63	1	2	9	131	1.07	0.64	0.59	1.03	0.95	1.03	0.93	0.94	0.83	0.79	0.73	0.75	0.70	26.28	8.24
Q9D6Y7-	Isoform 3 of Mitochondrial peptide methionine sulfoxide reductase OS=Mus musculus GN=Msra - [MSRA_MOUSE]	25.39	4	4	4	9	0.96	0.95	0.95	0.93	1.02	0.91	0.95	0.94	0.97	0.95	1.00	0.89	0.98	21.54	7.58
Q06138	Calcium-binding protein 39 OS=Mus musculus GN=Cab39 PE=1 SV=2 - [CAB39_MOUSE]	24.05	4	8	8	16	1.17	1.04	0.92	1.07	0.85	0.96	0.86	0.94	0.77	0.95	0.87	1.13	0.96	39.82	6.89
Q8BNW9	Kelch repeat and BTB domain-containing protein 11 OS=Mus musculus GN=Kbtbd11 PE=1 SV=3 - [KBTBB_MOUSE]	28.28	1	12	12	32	1.02	1.20	1.16	1.07	1.08	0.89	0.86	0.94	0.96	0.95	0.96	1.03	1.07	67.90	5.39
Q6P8N8	ATP-dependent Clp protease ATP-binding subunit clpX-like, mitochondrial OS=Mus musculus GN=Clpx PE=2 SV=1 - [Q6P8N8_MOUSE]	7.10	2	3	3	4	0.90	0.78	0.82	0.87	1.02	0.76	0.83	0.94	1.10	1.00	1.17	0.76	0.89	67.29	7.55
D6RET7	GRAM domain-containing protein 4 OS=Mus musculus GN=Gramd4 PE=2 SV=1 - [D6RET7_MOUSE]	2.79	3	1	1	2	0.99	1.07	1.08	1.15	1.16	0.88	0.89	0.94	0.95	1.04	1.05	1.08	1.09	37.57	8.13
E9QLA5	Inverted formin-2 OS=Mus musculus GN=Inf2 PE=4 SV=1 - [E9QLA5_MOUSE]	17.70	3	12	12	31	0.97	1.13	1.19	1.09	1.13	1.10	1.19	0.94	1.15	1.13	1.21	1.23	1.20	138.28	5.22
Q9D2N9	Vacuolar protein sorting-associated protein 33A OS=Mus musculus GN=Vps33a PE=1 SV=2 - [VP33A_MOUSE]	2.68	1	2	2	2	1.00	0.93	0.93	1.15	1.15	0.98	0.97	0.94	0.94	1.06	1.06	0.97	0.97	67.51	7.08
Q9WU78	Programmed cell death 6-interacting protein OS=Mus musculus GN=Pdcd6ip PE=1 SV=3 - [PDC6I_MOUSE]	41.77	3	28	28	81	0.94	0.87	0.90	1.01	1.07	0.92	0.99	0.94	0.97	0.96	1.01	0.92	1.03	95.96	6.52

Q9D0C4-2	Isoform 2 of tRNA (guanine(37)-N1)-methyltransferase OS=Mus musculus GN=Trmt5 - [TRM5_MOUSE]	2.05	2	1	1	1	1.16	1.29	1.11	0.99	0.85	0.90	0.77	0.94	0.81	0.85	0.73	0.78	0.67	55.43	8.32
Q8K4X7	1-acyl-sn-glycerol-3-phosphate acyltransferase delta OS=Mus musculus GN=Agpat4 PE=2 SV=1 - [PLCD_MOUSE]	5.82	1	3	3	4	0.97	1.18	1.21	1.16	1.19	0.98	1.01	0.94	0.96	1.06	1.12	1.07	1.28	43.78	8.38
Q9D7N3	28S ribosomal protein S9, mitochondrial OS=Mus musculus GN=Mrps9 PE=1 SV=3 - [RT09_MOUSE]	14.10	1	4	4	9	0.84	0.98	0.97	0.90	0.99	0.83	0.97	0.94	0.98	0.95	1.06	0.86	0.90	44.90	8.81
M0QWA1	Protein Csmd2 (Fragment) OS=Mus musculus GN=Csmd2 PE=4 SV=1 - [M0QWA1_MOUSE]	1.96	1	5	6	12	0.97	0.97	0.97	0.99	1.01	0.91	0.93	0.94	1.01	0.86	0.88	0.90	0.90	372.41	6.01
Q62277	Synaptophysin OS=Mus musculus GN=Syp PE=1 SV=2 - [SYPH_MOUSE]	36.62	2	11	12	74	1.22	1.15	1.01	1.18	0.97	0.89	0.74	0.94	0.81	0.99	0.81	1.10	0.97	34.00	4.94
E9QKT1	Ubiquitin-protein ligase E3A OS=Mus musculus GN=Ube3a PE=2 SV=1 - [E9QKT1_MOUSE]	14.02	2	9	9	22	1.01	1.15	1.14	1.08	1.10	0.94	0.90	0.94	0.97	0.94	0.97	1.04	1.01	99.76	5.08
Q9Z1R9	MCG124046 OS=Mus musculus GN=Prss1 PE=2 SV=1 - [Q9Z1R9_MOUSE]	12.20	2	1	2	219	1.47	0.93	0.63	1.00	0.67	1.05	0.69	0.94	0.64	0.87	0.57	0.98	0.67	26.12	4.94
Q3TJI8	Corticosteroid 11-beta-dehydrogenase isozyme 1 OS=Mus musculus GN=Hsd11b1 PE=2 SV=1 - [Q3TJI8_MOUSE]	8.02	2	2	2	3	0.90	0.91	1.01	0.77	0.86	0.87	0.96	0.94	1.04	0.63	0.70	0.74	0.83	28.67	8.72
Q6PD03	Serine/threonine-protein phosphatase 2A 56 kDa regulatory subunit alpha isoform OS=Mus musculus GN=Ppp2r5a PE=1 SV=1 - [2A5A_MOUSE]	12.55	1	5	6	8	0.94	0.99	1.18	1.04	1.13	0.88	0.91	0.94	1.02	1.01	1.08	1.03	1.04	56.31	7.03
E9PUA3	IQ motif and SEC7 domain-containing protein 1 OS=Mus musculus GN=Iqsec1 PE=2 SV=1 - [E9PUA3_MOUSE]	26.21	3	20	21	48	1.03	1.11	1.09	1.13	1.16	0.96	0.98	0.94	0.93	0.97	0.99	0.97	1.01	122.58	8.47
A2AQ07	Tubulin beta-1 chain OS=Mus musculus GN=Tubb1 PE=1 SV=1 - [TBB1_MOUSE]	16.19	1	2	7	173	0.85	0.99	1.17	1.12	1.32	1.04	1.23	0.94	1.11	0.75	0.88	0.71	0.84	50.41	5.07

Q64336	T-box brain protein 1 OS=Mus musculus GN=Tbr1 PE=2 SV=2 - [TBR1_MOUSE]	29.07	5	15	15	26	0.90	0.89	1.02	0.94	1.00	0.89	0.96	0.94	1.06	0.79	0.90	0.73	0.81	73.89	7.33
D3Z4D2	RUN and FYVE domain-containing protein 2 OS=Mus musculus GN=Rufy2 PE=2 SV=1 - [D3Z4D2_MOUSE]	4.22	4	1	2	7	0.97	1.03	1.06	1.15	1.18	1.03	1.06	0.94	0.97	1.02	1.06	1.07	1.11	46.13	5.11
Q6PDN3-	Isoform 3 of Myosin light chain kinase, smooth muscle OS=Mus musculus GN=Mylk - [MYLK_MOUSE]	12.90	16	9	10	15	0.98	0.96	1.01	0.96	0.99	0.77	0.81	0.94	1.02	1.01	1.04	0.83	0.91	113.63	5.38
G3UW55	MCG14937 OS=Mus musculus GN=A230065H16Rik PE=4 SV=1 - [G3UW55_MOUSE]	33.33	1	2	2	15	1.61	0.99	0.67	0.78	0.47	1.18	0.67	0.94	0.59	1.62	1.01	1.52	0.94	11.42	4.75
Q8C2Q8	ATP synthase subunit gamma OS=Mus musculus GN=Atp5c1 PE=2 SV=1 - [Q8C2Q8_MOUSE]	37.59	7	9	9	68	0.98	1.00	1.06	1.12	1.08	0.98	1.00	0.94	0.95	1.03	1.01	1.03	1.03	30.24	8.84
P48024	Eukaryotic translation initiation factor 1 OS=Mus musculus GN=Eif1 PE=2 SV=2 - [EIF1_MOUSE]	61.95	5	2	7	67	1.08	0.85	0.79	0.98	0.90	0.94	0.83	0.94	0.87	0.87	0.77	0.95	0.85	12.74	7.44
Q6NZM9	Histone deacetylase 4 OS=Mus musculus GN=Hdac4 PE=1 SV=1 - [HDAC4_MOUSE]	15.24	2	12	12	33	1.01	1.00	0.99	0.95	0.94	0.95	0.82	0.94	0.95	0.86	0.77	0.90	0.85	118.49	6.92
F8WHM5	Golgi apparatus protein 1 (Fragment) OS=Mus musculus GN=Glg1 PE=2 SV=1 - [F8WHM5_MOUSE]	23.90	4	24	24	50	1.07	1.07	0.99	1.02	0.99	0.97	0.90	0.94	0.90	0.97	0.89	1.02	0.94	132.26	6.93
Q8K021	Secretory carrier-associated membrane protein 1 OS=Mus musculus GN=Scamp1 PE=1 SV=1 - [SCAM1_MOUSE]	29.29	3	6	6	57	1.07	0.97	0.92	1.07	1.02	0.95	0.91	0.94	0.89	1.00	0.96	0.97	0.94	38.00	7.71
P15920	V-type proton ATPase 116 kDa subunit a isoform 2 OS=Mus musculus GN=Atp6v0a2 PE=1 SV=2 - [VPP2_MOUSE]	1.29	1	1	1	2	1.05	1.34	1.28	1.20	1.15	0.93	0.88	0.94	0.90	0.99	0.95	1.03	0.98	98.08	6.64
Q61330	Contactin-2 OS=Mus musculus GN=Cntn2 PE=2 SV=2 - [CNTN2_MOUSE]	27.60	1	19	19	59	0.96	1.01	1.05	1.02	1.07	0.98	1.00	0.94	0.98	0.98	0.98	0.97	0.97	113.15	7.62

Q3TXS7	26S proteasome non-ATPase regulatory subunit 1 OS=Mus musculus GN=Psmd1 PE=1 SV=1 - [PSMD1_MOUSE]	23.92	3	16	16	40	0.97	0.97	0.91	1.02	1.01	0.99	0.98	0.94	0.93	0.95	1.00	0.99	1.03	105.66	5.39
Q9DCM2	Glutathione S-transferase kappa 1 OS=Mus musculus GN=Gstk1 PE=1 SV=3 - [GSTK1_MOUSE]	12.39	1	2	2	5	1.04	0.94	0.91	1.07	1.06	1.02	1.00	0.94	0.91	1.06	1.05	1.06	1.03	25.69	8.88
Q5FWK3	Rho GTPase-activating protein 1 OS=Mus musculus GN=Arhgap1 PE=1 SV=1 - [RHG01_MOUSE]	35.31	2	10	10	24	1.04	1.06	1.05	1.10	1.10	0.88	0.91	0.94	0.97	0.94	1.02	1.06	1.03	50.38	6.44
Q91WR3-	Isoform 2 of Activating signal cointegrator 1 complex subunit 2 OS=Mus musculus GN=Ascc2 - [ASCC2_MOUSE]	6.21	3	2	2	3	1.14	1.11	0.97	1.10	0.97	1.12	0.98	0.94	0.83	0.89	0.78	0.97	0.86	80.93	4.94
A2APV2-	Isoform 3 of Formin-like protein 2 OS=Mus musculus GN=Fmnl2 - [FMNL2_MOUSE]	6.97	7	5	7	12	0.97	1.15	1.22	1.14	1.14	0.90	0.87	0.94	1.06	1.00	1.04	1.04	1.11	123.90	7.65
G5E832	Adenomatosis polyposis coli 2 OS=Mus musculus GN=Apc2 PE=4 SV=1 - [G5E832_MOUSE]	2.51	3	1	3	4	0.83	0.98	1.18	0.86	1.03	1.00	1.19	0.94	1.13	0.81	0.97	0.89	1.07	242.96	8.88
Q9JIW9	Ras-related protein Ral-B OS=Mus musculus GN=Ralb PE=2 SV=1 - [RALB_MOUSE]	49.03	2	4	9	88	1.14	0.83	0.72	1.02	0.93	0.94	0.85	0.94	0.92	0.95	0.85	0.94	0.92	23.33	6.62
Q80SW1	Putative adenosylhomocysteinase 2 OS=Mus musculus GN=Ahcyl1 PE=1 SV=1 - [SAHH2_MOUSE]	37.36	3	10	21	121	0.96	1.02	1.02	1.04	1.07	0.96	0.97	0.94	0.98	0.94	0.96	0.92	0.98	58.91	6.89
P28652	Calcium/calmodulin-dependent protein kinase type II subunit beta OS=Mus musculus GN=Camk2b PE=1 SV=2 - [KCC2B_MOUSE]	43.17	9	11	18	139	0.98	0.90	0.89	1.15	1.19	0.91	0.92	0.94	0.90	1.00	1.02	1.04	1.06	60.42	7.28
F8WHK3	cAMP and cAMP-inhibited cGMP 3',5'-cyclic phosphodiesterase 10A OS=Mus musculus GN=Pde10a PE=4 SV=1 - [F8WHK3_MOUSE]	16.48	6	9	9	17	1.20	1.51	1.25	1.52	1.29	1.13	0.97	0.94	0.76	1.29	1.17	1.65	1.40	81.17	6.76

A2AIR7	Voltage-dependent N-type calcium channel subunit alpha-1B OS=Mus musculus GN=Cacna1b PE=3 SV=1 - [A2AIR7_MOUSE]	8.51	7	14	15	39	1.04	1.20	1.25	1.16	1.10	1.01	0.92	0.94	0.92	1.05	0.96	0.97	0.96	261.39	8.59
Q8BM92	Cadherin-7 OS=Mus musculus GN=Cdh7 PE=2 SV=1 - [CADH7_MOUSE]	3.82	1	1	2	5	1.18	1.12	1.04	1.13	0.96	1.16	0.98	0.94	0.80	0.82	0.70	0.78	0.66	87.15	4.82
Q91Z31	Polypyrimidine tract-binding protein 2 OS=Mus musculus GN=Ptbp2 PE=1 SV=2 - [PTBP2_MOUSE]	24.29	2	6	6	15	0.99	0.76	0.77	1.03	0.92	0.92	1.12	0.94	1.00	1.03	0.94	0.97	0.95	57.45	8.66
Q8BTG3	T-complex protein 11-like protein 1 OS=Mus musculus GN=Tcp11l1 PE=2 SV=1 - [T11L1_MOUSE]	6.09	1	2	3	5	1.08	0.98	0.91	1.15	1.07	1.06	0.98	0.94	0.87	1.14	1.06	1.17	1.09	56.30	5.50
Q91V36	Nuclear receptor-binding protein 2 OS=Mus musculus GN=Nrbp2 PE=1 SV=2 - [NRBP2_MOUSE]	4.61	1	2	2	4	1.12	0.86	0.76	0.86	0.88	1.01	0.90	0.94	0.84	1.12	0.98	0.99	0.94	57.31	6.35
Q8JZP2	Synapsin-3 OS=Mus musculus GN=Syn3 PE=1 SV=2 - [SYN3_MOUSE]	35.92	2	11	12	48	1.02	1.02	0.99	1.00	1.01	0.95	0.97	0.94	0.94	0.94	0.98	0.96	1.02	63.27	9.38
Q6ZPF3-3	Isoform 3 of T-lymphoma invasion and metastasis-inducing protein 2 OS=Mus musculus GN=Tiam2 - [TIAM2_MOUSE]	11.31	3	4	4	14	1.14	0.86	0.86	0.95	0.91	1.02	0.97	0.94	0.95	1.04	0.96	0.91	0.89	70.10	6.33
P28665	Murinoglobulin-1 OS=Mus musculus GN=Mug1 PE=1 SV=3 - [MUG1_MOUSE]	4.81	2	4	4	10	0.82	1.10	1.23	0.65	0.76	0.87	1.01	0.94	1.08	0.94	1.04	0.68	0.80	165.19	6.42
E9PUD2	Dynamin-1-like protein OS=Mus musculus GN=Dnm1l PE=2 SV=1 - [E9PUD2_MOUSE]	55.06	1	2	33	96	0.92	0.93	0.97	1.14	1.23	0.99	1.06	0.94	0.96	0.97	1.07	0.98	1.02	79.48	7.08
P62812	Gamma-aminobutyric acid receptor subunit alpha-1 OS=Mus musculus GN=Gabra1 PE=1 SV=1 - [GBRA1_MOUSE]	27.47	2	8	12	57	0.98	0.99	1.04	1.11	1.10	0.86	0.85	0.94	0.96	0.94	0.95	0.96	1.00	51.72	9.31
Q9DB72	BTB/POZ domain-containing protein 17 OS=Mus musculus GN=Btbd17 PE=2 SV=1 - [BTBDH_MOUSE]	19.87	1	7	7	15	1.01	1.10	1.04	1.08	1.13	0.86	0.97	0.94	0.92	0.90	0.95	0.93	1.01	52.57	9.42
Q6P069-2	Isoform 2 of Sorcin OS=Mus musculus GN=Sri - [SORCN_MOUSE]	28.96	2	6	6	17	0.94	0.77	0.86	0.93	1.02	0.93	0.99	0.94	1.05	0.92	1.03	0.93	1.00	20.28	5.34

Q8R066	Complement C1q tumor necrosis factor-related protein 4 OS=Mus musculus GN=C1qtnf4 PE=2 SV=1 - [C1QT4_MOUSE]	7.98	1	2	2	4	1.14	1.11	0.98	1.04	0.91	0.90	0.79	0.94	0.83	1.07	0.95	0.96	0.85	35.04	8.98
Q91YR7	Pre-mRNA-processing factor 6 OS=Mus musculus GN=Prpf6 PE=2 SV=1 - [PRP6_MOUSE]	5.84	2	5	5	11	1.03	1.05	1.15	0.95	1.04	0.90	1.00	0.94	1.02	1.01	1.11	1.08	1.14	106.65	8.09
Q99J08	SEC14-like protein 2 OS=Mus musculus GN=Sec14l2 PE=2 SV=1 - [S14L2_MOUSE]	16.87	1	5	5	15	0.96	1.15	1.10	1.09	1.16	0.96	1.04	0.94	0.98	0.93	1.03	1.06	1.17	46.27	7.11
Q8C0L0	Thioredoxin-related transmembrane protein 4 OS=Mus musculus GN=Tmx4 PE=1 SV=2 - [TMX4_MOUSE]	20.60	3	5	5	18	0.96	1.11	1.11	1.14	1.12	1.01	0.97	0.94	0.92	1.09	0.91	0.97	0.96	37.11	4.37
Q6PGH0	Ubiquitin domain-containing protein 2 OS=Mus musculus GN=Ubtd2 PE=2 SV=1 - [UBTD2_MOUSE]	27.78	2	4	4	13	0.98	1.24	1.18	1.00	1.06	1.06	1.07	0.94	0.97	0.92	0.96	0.93	0.91	26.13	5.83
E9Q7L0	Protein Ogdhl OS=Mus musculus GN=Ogdhl PE=2 SV=1 - [E9Q7L0_MOUSE]	38.68	1	24	30	79	0.95	1.06	1.04	1.09	1.09	0.93	0.96	0.94	0.98	0.92	1.01	0.99	1.01	116.53	6.84
Q99N87	28S ribosomal protein S5, mitochondrial OS=Mus musculus GN=Mrps5 PE=2 SV=1 - [RT05_MOUSE]	12.04	2	5	5	8	0.98	0.94	0.95	1.03	1.01	0.95	0.97	0.94	0.94	0.92	0.91	0.98	1.00	48.18	10.14
Q9CQ79	Thioredoxin domain-containing protein 9 OS=Mus musculus GN=Txndc9 PE=1 SV=1 - [TXND9_MOUSE]	18.14	1	4	4	6	0.97	0.81	0.91	0.73	0.80	0.89	0.99	0.94	1.02	0.79	0.82	0.77	0.78	26.24	5.95
Q8VHN8	Protein syndesmos OS=Mus musculus GN=Nudt16l1 PE=1 SV=2 - [SDOS_MOUSE]	9.95	4	1	2	3	1.13	1.28	1.13	0.83	0.73	0.96	0.84	0.94	0.83	0.98	0.87	1.01	0.89	23.40	9.26
Q9Z2W1	Serine/threonine-protein kinase 25 OS=Mus musculus GN=Stk25 PE=1 SV=2 - [STK25_MOUSE]	18.08	2	2	6	11	0.97	0.79	0.81	0.97	0.99	0.99	1.01	0.94	0.97	1.02	1.05	0.99	1.02	48.13	6.81
Q9EP72	ER membrane protein complex subunit 7 OS=Mus musculus GN=Emc7 PE=2 SV=1 - [EMC7_MOUSE]	6.64	1	1	1	1	1.08	0.83	0.77	1.08	1.00	0.94	0.87	0.94	0.87	1.04	0.96	1.01	0.94	26.29	9.23
P52196	Thiosulfate sulfurtransferase OS=Mus musculus GN=Tst PE=1 SV=3 - [THTR_MOUSE]	28.28	1	7	7	11	0.93	0.78	0.90	0.95	1.05	0.88	1.10	0.94	0.93	0.88	0.91	0.95	1.10	33.44	7.85

Q8BP40	Lysophosphatidic acid phosphatase type 6 OS=Mus musculus GN=Acp6 PE=2 SV=1 - [PPA6_MOUSE]	11.72	1	3	3	6	0.97	1.03	1.09	1.03	1.05	0.89	0.93	0.94	0.98	1.00	1.06	1.02	0.97	47.59	7.72
Q9QYC0	Alpha-adducin OS=Mus musculus GN=Add1 PE=1 SV=2 - [ADDA_MOUSE]	47.35	4	2	26	231	1.15	0.94	0.80	0.85	0.80	1.09	1.15	0.94	1.05	1.05	0.96	1.18	0.76	80.60	5.90
Q3ZK22-3	Isoform 3 of Vezatin OS=Mus musculus GN=Vezt - [VEZA_MOUSE]	2.76	4	1	1	2	1.17	1.50	1.27	1.46	1.24	1.02	0.86	0.94	0.80	1.12	0.96	1.23	1.05	70.65	6.19
Q9DCJ5	NADH dehydrogenase [ubiquinone] 1 alpha subcomplex subunit 8 OS=Mus musculus GN=Ndufa8 PE=1 SV=3 - [NDUA8_MOUSE]	43.02	1	6	6	39	0.86	0.78	0.82	0.83	0.94	0.90	1.01	0.94	1.10	0.85	1.01	0.87	1.02	19.98	8.46
O89053	Coronin-1A OS=Mus musculus GN=Coro1a PE=1 SV=5 - [COR1A_MOUSE]	41.21	4	4	17	47	0.91	0.91	1.06	1.13	1.21	0.77	0.93	0.94	1.03	0.95	1.04	0.80	0.91	50.96	6.48
Q9WVA3	Mitotic checkpoint protein BUB3 OS=Mus musculus GN=Bub3 PE=2 SV=2 - [BUB3_MOUSE]	21.47	1	6	6	13	1.00	1.22	1.15	0.97	0.93	0.89	0.84	0.94	0.94	0.97	0.99	1.08	1.08	36.93	6.84
Q99JF5	Diphosphomevalonate decarboxylase OS=Mus musculus GN=Mvd PE=1 SV=2 - [MVD1_MOUSE]	12.47	1	2	2	8	1.02	1.06	1.04	0.93	0.91	0.85	0.90	0.94	0.92	1.05	1.27	1.06	1.03	44.04	6.30
P48722	Heat shock 70 kDa protein 4L OS=Mus musculus GN=Hspa4l PE=1 SV=2 - [HS74L_MOUSE]	55.61	5	36	39	123	0.97	0.98	0.94	1.04	1.06	0.95	0.96	0.94	0.95	0.94	0.95	0.98	0.98	94.32	5.74
O70374-2	Isoform 2 of Protein CBFA2T2 OS=Mus musculus GN=Cbfa2t2 - [MTG8R_MOUSE]	2.79	5	2	2	3	0.92	1.18	1.27	0.79	0.86	1.17	1.26	0.94	1.02	1.04	1.13	0.89	0.97	63.88	7.69
Q3UYG8	O-acetyl-ADP-ribose deacetylase MACROD2 OS=Mus musculus GN=Macrod2 PE=2 SV=1 - [MACD2_MOUSE]	10.53	3	5	5	11	1.15	0.89	0.68	1.14	0.95	1.07	0.89	0.94	0.73	0.96	0.74	1.20	0.91	52.11	4.75
Q8BPA8-3	Isoform 2 of Protein DPCD OS=Mus musculus GN=Dpcd - [DPCD_MOUSE]	8.04	2	1	1	1	1.01	0.79	0.78	1.03	1.01	1.00	0.98	0.94	0.93	1.00	0.99	0.91	0.90	12.38	6.16
D3Z451	MCG54087 OS=Mus musculus GN=Serpina3j PE=3 SV=1 - [D3Z451_MOUSE]	6.67	1	1	1	1	0.93	1.25	1.34	0.98	1.06	0.85	0.91	0.94	1.02	0.82	0.89	1.12	1.21	47.33	7.50

A6X940	Fermitin family homolog 2 (Fragment) OS=Mus musculus GN=Fermt2 PE=2 SV=1 - [A6X940_MOUSE]	8.14	1	1	2	4	1.00	0.79	0.79	1.04	1.04	0.90	0.89	0.94	0.94	0.98	0.98	0.90	0.90	28.89	8.65
Q922D8	C-1-tetrahydrofolate synthase, cytoplasmic OS=Mus musculus GN=Mthfd1 PE=1 SV=4 - [C1TC_MOUSE]	22.99	1	16	16	36	1.04	1.05	1.05	1.07	1.04	0.92	0.92	0.94	0.92	0.94	0.97	1.01	1.02	101.14	7.14
Q811Q9-2	Isoform 2 of Choline-phosphate cytidylyltransferase B OS=Mus musculus GN=Pcyt1b - [PCY1B_MOUSE]	10.32	2	2	3	6	0.92	1.02	1.11	1.00	1.09	1.13	1.22	0.94	1.03	0.88	0.96	0.95	1.04	38.74	7.55
Q01853	Transitional endoplasmic reticulum ATPase OS=Mus musculus GN=Vcp PE=1 SV=4 - [TERA_MOUSE]	74.94	9	44	45	352	0.98	1.02	1.06	1.01	1.05	0.97	0.98	0.94	0.99	0.94	0.99	0.97	1.00	89.27	5.26
Q811U4	Mitofusin-1 OS=Mus musculus GN=Mfn1 PE=1 SV=3 - [MFN1_MOUSE]	2.43	1	1	1	2	1.05	1.25	1.19	1.33	1.27	0.78	0.74	0.94	0.90	0.90	0.86	1.03	0.98	83.67	6.51
P24638	Lysosomal acid phosphatase OS=Mus musculus GN=Acp2 PE=2 SV=2 - [PPAL_MOUSE]	1.89	1	1	1	2	1.03	0.91	0.88	1.03	0.99	1.04	1.01	0.94	0.91	0.92	0.89	0.88	0.85	48.48	7.02
Q8CIQ7	Dedicator of cytokinesis protein 3 OS=Mus musculus GN=Dock3 PE=1 SV=1 - [DOCK3_MOUSE]	6.02	3	7	8	26	0.92	1.06	1.00	1.00	0.97	0.98	1.02	0.95	0.96	0.95	0.92	1.02	1.01	232.76	6.99
Q61553	Fascin OS=Mus musculus GN=Fscn1 PE=1 SV=4 - [FSCN1_MOUSE]	56.19	5	23	23	95	0.99	0.98	1.01	1.06	1.08	0.95	0.93	0.95	0.97	0.94	0.95	0.98	1.08	54.47	6.89
Q6PHU5	Sortilin OS=Mus musculus GN=Sort1 PE=1 SV=1 - [SORT_MOUSE]	15.15	3	9	9	24	0.95	0.95	1.05	1.02	1.04	0.94	1.08	0.95	0.99	0.93	0.96	0.92	0.98	91.14	5.88
Q60737	Casein kinase II subunit alpha OS=Mus musculus GN=Csnk2a1 PE=1 SV=2 - [CSK21_MOUSE]	48.34	3	14	14	63	0.98	0.95	0.92	1.11	1.11	0.95	0.98	0.95	0.99	0.91	0.95	1.03	0.98	45.10	7.74
Q9WVK4	EH domain-containing protein 1 OS=Mus musculus GN=Ehd1 PE=1 SV=1 - [EHD1_MOUSE]	58.99	1	12	22	64	0.94	0.93	0.99	1.04	1.18	0.90	1.06	0.95	1.03	0.98	1.16	1.03	1.11	60.56	6.83
P59016	Vacuolar protein sorting-associated protein 33B OS=Mus musculus GN=Vps33b PE=1 SV=1 - [VP33B_MOUSE]	7.62	1	4	4	5	1.04	1.10	1.08	1.13	1.16	1.08	0.84	0.95	0.91	0.92	1.00	1.27	1.09	70.48	6.86

G3X8V3	Phosphatidylethanolamine-binding protein 4 OS=Mus musculus GN=Pebp4 PE=4 SV=1 - [G3X8V3_MOUSE]	3.31	2	1	1	1	0.79	1.19	1.50	1.11	1.40	1.17	1.47	0.95	1.19	0.84	1.06	1.01	1.28	26.84	5.48
Q8BZA9	Fructose-2,6-bisphosphatase TIGAR OS=Mus musculus GN=Tigar PE=2 SV=1 - [TIGAR_MOUSE]	15.24	1	2	2	7	0.98	0.88	0.82	1.10	1.03	0.97	1.02	0.95	0.90	0.89	1.12	1.09	1.16	29.17	8.18
Q91XY0	MCG133388, isoform CRA_m OS=Mus musculus GN=Pcdhga8 PE=2 SV=1 - [Q91XY0_MOUSE]	7.51	9	3	5	18	1.01	1.08	1.12	0.98	0.97	1.00	0.99	0.95	0.90	1.01	1.00	0.96	1.00	101.41	5.11
Q9CRD2	ER membrane protein complex subunit 2 OS=Mus musculus GN=Emc2 PE=2 SV=1 - [EMC2_MOUSE]	14.81	1	4	4	10	0.91	1.00	1.28	0.96	1.08	0.85	0.96	0.95	1.04	0.85	0.99	0.93	1.04	34.91	6.81
D3Z568	NADH dehydrogenase (Ubiquinone) 1 beta subcomplex, 5, isoform CRA_e OS=Mus musculus GN=Ndufb5 PE=4 SV=1 - [D3Z568_MOUSE]	17.65	2	3	3	15	1.03	1.05	0.97	1.22	1.18	0.91	0.89	0.95	0.99	1.06	1.00	0.89	0.91	14.03	5.54
Q9D2V7	Coronin-7 OS=Mus musculus GN=Coro7 PE=2 SV=2 - [CORO7_MOUSE]	24.95	3	16	16	33	1.02	1.13	1.12	1.07	1.08	0.96	0.93	0.95	0.90	0.99	0.98	0.96	0.96	100.75	5.77
Q9DB05	Alpha-soluble NSF attachment protein OS=Mus musculus GN=Napa PE=1 SV=1 - [SNAA_MOUSE]	74.24	1	15	18	134	0.95	1.09	1.15	1.04	1.10	0.96	0.97	0.95	1.00	0.96	1.04	1.03	1.08	33.17	5.45
P58854	Gamma-tubulin complex component 3 OS=Mus musculus GN=Tubgcp3 PE=2 SV=2 - [GCP3_MOUSE]	0.88	1	1	1	2	0.93	1.19	1.27	1.16	1.24	0.91	0.97	0.95	1.01	1.00	1.07	1.13	1.21	103.40	8.32
Q07417	Short-chain specific acyl-CoA dehydrogenase, mitochondrial OS=Mus musculus GN=Acads PE=2 SV=2 - [ACADS_MOUSE]	12.38	2	4	4	7	0.98	0.91	0.87	1.06	1.10	0.83	0.92	0.95	0.93	0.94	0.93	0.97	1.07	44.86	8.47
E9QNX9	Tyrosine-protein kinase receptor OS=Mus musculus GN=Igf1r PE=2 SV=1 - [E9QNX9_MOUSE]	5.92	2	5	6	9	0.94	1.18	1.24	1.01	1.03	0.93	0.99	0.95	1.01	0.95	1.01	0.93	1.00	155.19	5.83
Q9JHW4	Selenocysteine-specific elongation factor OS=Mus musculus GN=Eefsec PE=2 SV=2 - [SELB_MOUSE]	2.74	1	1	1	4	0.86	1.39	1.61	0.69	0.80	1.04	1.20	0.95	1.10	0.97	1.14	1.20	1.40	63.50	8.29

Q9D1J1	Adaptin ear-binding coat-associated protein 2 OS=Mus musculus GN=Necap2 PE=1 SV=1 - [NECP2_MOUSE]	17.29	2	3	4	8	0.99	1.02	0.97	0.98	0.95	0.88	0.86	0.95	0.92	0.83	0.84	0.96	0.93	28.58	7.94
A2A9X5	5'(3')-deoxyribonucleotidase, cytosolic type OS=Mus musculus GN=Nt5c PE=2 SV=1 - [A2A9X5_MOUSE]	40.21	1	1	6	25	0.89	0.88	1.00	0.93	0.92	1.00	1.18	0.95	0.96	0.87	1.05	1.05	1.02	21.93	5.33
E9PVS5	Mitochondrial inner membrane protein (Fragment) OS=Mus musculus GN=Immt PE=2 SV=1 - [E9PVS5_MOUSE]	48.94	1	3	8	22	1.05	1.02	0.98	1.02	0.96	0.82	0.81	0.95	0.90	0.89	1.01	1.03	0.99	19.66	9.09
P53810	Phosphatidylinositol transfer protein alpha isoform OS=Mus musculus GN=Pitpna PE=1 SV=2 - [PIPNA_MOUSE]	59.78	4	13	15	61	0.95	0.93	0.99	1.01	1.04	0.99	1.02	0.95	0.99	0.92	0.96	0.91	1.01	31.87	6.37
Q6ZPU9	KIF1-binding protein OS=Mus musculus GN=Kbp PE=2 SV=2 - [KBP_MOUSE]	14.42	6	6	6	10	1.07	1.12	1.05	0.98	0.96	0.87	0.84	0.95	0.88	0.94	0.94	1.00	0.97	71.01	5.48
P10649	Glutathione S-transferase Mu 1 OS=Mus musculus GN=Gstm1 PE=1 SV=2 - [GSTM1_MOUSE]	69.27	10	12	16	97	1.06	1.00	0.94	1.05	0.99	0.99	0.93	0.95	0.92	0.99	0.93	1.11	1.08	25.95	7.94
Q8BG39	Synaptic vesicle glycoprotein 2B OS=Mus musculus GN=Sv2b PE=1 SV=1 - [SV2B_MOUSE]	20.64	1	11	12	123	1.00	1.12	1.12	1.04	1.05	0.92	0.87	0.95	0.91	0.96	0.95	1.02	1.01	77.41	5.57
Q80XE1	Synembryn-B OS=Mus musculus GN=Ric8b PE=1 SV=2 - [RIC8B_MOUSE]	1.73	2	1	1	1	1.04	1.07	1.02	1.00	0.95	0.87	0.83	0.95	0.90	0.95	0.91	0.99	0.95	58.65	5.72
Q99MR8	Methylcrotonoyl-CoA carboxylase subunit alpha, mitochondrial OS=Mus musculus GN=Mccc1 PE=2 SV=2 - [MCCA_MOUSE]	12.97	3	7	7	17	0.96	1.07	1.06	1.15	1.19	1.01	0.99	0.95	1.02	1.01	1.05	0.95	0.99	79.29	7.83
A2ALK6	Band 4.1-like protein 4B OS=Mus musculus GN=Epb4.1l4b PE=2 SV=1 - [A2ALK6_MOUSE]	0.89	1	1	1	1	1.22	1.02	0.83	1.01	0.82	0.94	0.77	0.95	0.77	0.96	0.79	0.91	0.74	99.72	9.17
Q80TL0	Protein phosphatase 1E OS=Mus musculus GN=Ppm1e PE=1 SV=2 - [PPM1E_MOUSE]	26.17	1	13	13	65	1.02	0.94	0.97	1.05	1.02	0.97	0.93	0.95	0.96	0.95	0.93	0.98	0.99	83.37	4.97
Q9EP97	Sentrin-specific protease 3 OS=Mus musculus GN=Senp3 PE=1 SV=1 - [SENP3_MOUSE]	3.17	1	1	1	2	0.99	1.31	1.32	1.07	1.08	1.01	1.02	0.95	0.95	1.16	1.17	1.18	1.20	64.36	8.75

Q8R502	Leucine-rich repeat-containing protein 8C OS=Mus musculus GN=Lrrc8c PE=1 SV=1 - [LRC8C_MOUSE]	5.48	1	3	3	8	0.94	1.03	1.14	1.14	1.16	0.86	0.94	0.95	0.99	0.92	1.03	0.97	1.09	92.31	7.81
Q99JY0	Trifunctional enzyme subunit beta, mitochondrial OS=Mus musculus GN=Hadhb PE=1 SV=1 - [ECHB_MOUSE]	35.16	2	13	13	30	1.00	0.95	1.02	1.07	1.05	0.93	0.95	0.95	0.94	1.01	1.02	1.02	1.05	51.35	9.38
Q91Y86	Mitogen-activated protein kinase 8 OS=Mus musculus GN=Mapk8 PE=1 SV=1 - [MK08_MOUSE]	31.25	16	4	10	16	0.97	0.89	0.89	0.96	0.83	0.96	1.02	0.95	0.99	0.78	0.81	0.80	0.80	44.20	7.69
P17665	Cytochrome c oxidase subunit 7C, mitochondrial OS=Mus musculus GN=Cox7c PE=1 SV=1 - [COX7C_MOUSE]	61.90	3	3	3	12	1.03	0.78	0.75	0.97	0.95	1.07	1.03	0.95	0.94	1.01	0.91	0.95	0.92	7.33	11.00
P40336	Vacuolar protein sorting-associated protein 26A OS=Mus musculus GN=Vps26a PE=2 SV=1 - [VP26A_MOUSE]	31.19	2	7	7	25	0.93	1.04	1.19	1.02	1.04	0.97	1.02	0.95	0.87	0.94	1.05	1.01	1.04	38.09	6.57
E9Q828	Protein Atp2b4 OS=Mus musculus GN=Atp2b4 PE=2 SV=1 - [E9Q828_MOUSE]	34.30	4	22	32	161	1.06	1.12	1.05	1.12	1.02	0.92	0.86	0.95	0.85	0.99	0.91	1.01	0.99	129.17	6.73
Q5SU72	E3 ubiquitin/ISG15 ligase TRIM25 OS=Mus musculus GN=Trim25 PE=2 SV=1 - [Q5SU72_MOUSE]	2.84	3	1	1	2	0.93	1.05	1.12	0.92	0.99	0.85	0.91	0.95	1.02	0.99	1.07	1.07	1.15	44.13	7.87
Q8BG18	N-terminal EF-hand calcium-binding protein 1 OS=Mus musculus GN=Necab1 PE=1 SV=1 - [NECA1_MOUSE]	24.72	1	5	5	13	1.00	0.86	0.87	1.09	1.11	0.97	0.98	0.95	0.94	0.89	0.90	0.98	1.01	40.91	4.89
A2RSX9	Arfip1 protein OS=Mus musculus GN=Arfip1 PE=2 SV=1 - [A2RSX9_MOUSE]	3.23	3	1	1	3	1.31	1.13	0.86	0.99	0.75	1.27	0.96	0.95	0.78	1.34	1.02	1.07	0.89	38.39	6.61
Q9CQC7	NADH dehydrogenase [ubiquinone] 1 beta subcomplex subunit 4 OS=Mus musculus GN=Ndufb4 PE=1 SV=3 - [NDUB4_MOUSE]	49.61	1	5	5	16	1.07	0.94	1.01	1.11	1.04	0.98	0.99	0.95	0.96	0.94	0.95	0.99	0.97	15.07	9.89
Q7TSH8	Uncharacterized protein KIAA0195 OS=Mus musculus GN=Kiaa0195 PE=1 SV=1 - [K0195_MOUSE]	1.62	1	2	2	3	1.07	1.07	1.00	1.07	1.00	0.97	0.90	0.95	0.88	0.94	0.88	1.00	0.94	151.70	6.46

P49025-3	Isoform 3 of Citron Rho-interacting kinase OS=Mus musculus GN=Cit - [CTRO_MOUSE]	26.93	8	42	42	98	0.98	1.02	0.99	1.01	1.02	0.93	0.92	0.95	0.96	0.89	0.91	0.86	0.87	183.33	6.61
Q99LS3	Phosphoserine phosphatase OS=Mus musculus GN=Psph PE=1 SV=1 - [SERB_MOUSE]	12.89	3	3	3	6	0.96	1.11	1.17	1.09	1.13	0.95	1.00	0.95	1.00	1.02	1.07	1.05	1.10	25.08	6.14
Q8VD62	UPF0696 protein C11orf68 homolog OS=Mus musculus GN=Bles03 PE=2 SV=1 - [CK068_MOUSE]	25.10	2	4	4	8	0.94	0.94	0.96	0.97	1.03	1.00	0.99	0.95	1.06	0.98	0.99	1.07	1.09	27.52	5.47
P63137-2	Isoform 2 of Gamma-aminobutyric acid receptor subunit beta-2 OS=Mus musculus GN=Gabrb2 - [GBRB2_MOUSE]	21.52	3	3	7	17	1.03	0.98	1.00	1.12	1.00	0.86	0.84	0.95	0.88	0.91	0.89	0.82	0.78	54.60	9.32
G8JL54	COMM domain-containing protein 1 (Fragment) OS=Mus musculus GN=Commd1 PE=2 SV=1 - [G8JL54_MOUSE]	11.83	3	2	2	5	1.10	1.83	1.67	0.98	0.82	0.94	0.90	0.95	0.99	0.88	0.74	1.00	0.86	10.30	5.19
Q3UQ44	Ras GTPase-activating-like protein IQGAP2 OS=Mus musculus GN=Iqgap2 PE=1 SV=2 - [IQGA2_MOUSE]	1.46	1	2	2	5	1.00	1.09	1.09	1.07	1.07	1.10	1.09	0.95	0.97	0.94	0.97	0.95	0.98	180.42	5.64
Q62507	Cochlin OS=Mus musculus GN=Coch PE=2 SV=2 - [COCH_MOUSE]	2.54	1	1	1	2	1.11	0.99	0.88	1.13	1.01	0.89	0.79	0.95	0.85	0.88	0.79	1.09	0.98	59.91	8.44
Q8K0T4	Katanin p60 ATPase-containing subunit A-like 1 OS=Mus musculus GN=Katnal1 PE=1 SV=1 - [KATL1_MOUSE]	17.83	7	6	6	12	0.91	0.85	0.98	0.96	1.07	0.99	1.14	0.95	1.06	0.99	1.09	1.04	1.18	55.13	7.09
Q9QUR8	Semaphorin-7A OS=Mus musculus GN=Sema7a PE=1 SV=1 - [SEM7A_MOUSE]	15.21	1	7	7	25	0.91	0.98	1.06	0.99	1.14	0.84	1.02	0.95	1.06	0.88	1.00	0.83	0.95	74.95	7.74
P38585	Tubulin--tyrosine ligase OS=Mus musculus GN=Ttl PE=2 SV=2 - [TTL_MOUSE]	4.77	1	1	1	3	0.93	1.32	1.42	1.51	1.62	1.17	1.25	0.95	1.02	1.10	1.18	1.17	1.26	43.11	6.46
P62717	60S ribosomal protein L18a OS=Mus musculus GN=Rpl18a PE=1 SV=1 - [RL18A_MOUSE]	25.57	2	5	5	12	1.06	0.98	0.92	1.10	1.02	0.99	0.94	0.95	0.88	0.96	0.92	1.09	1.06	20.72	10.71
Q3TA40	Protein 6430548M08Rik OS=Mus musculus GN=6430548M08Rik PE=2 SV=1 - [Q3TA40_MOUSE]	40.81	4	14	14	56	0.95	0.87	0.92	0.93	0.99	0.93	0.97	0.95	0.99	0.92	0.95	0.88	0.91	45.19	5.07

Q8BX70-2	Isoform 2 of Vacuolar protein sorting-associated protein 13C OS=Mus musculus GN=Vps13c - [VP13C_MOUSE]	4.28	4	10	13	24	1.04	0.83	0.96	1.11	1.11	0.94	0.89	0.95	0.96	0.96	0.94	0.93	1.00	405.57	6.61
Q03173-6	Isoform 6 of Protein enabled homolog OS=Mus musculus GN=Enah - [ENAH_MOUSE]	36.88	10	15	15	44	0.98	0.80	0.76	0.91	0.94	0.96	0.92	0.95	0.92	0.86	0.92	0.83	0.82	56.32	6.34
Q9WV34	MAGUK p55 subfamily member 2 OS=Mus musculus GN=Mpp2 PE=1 SV=1 - [MPP2_MOUSE]	44.57	2	19	21	76	0.93	1.08	1.19	1.05	1.15	0.96	1.04	0.95	1.02	0.94	0.99	0.98	1.07	61.52	6.44
A2A6Q8	Myosin light chain 4 (Fragment) OS=Mus musculus GN=Myl4 PE=2 SV=1 - [A2A6Q8_MOUSE]	60.94	4	11	11	37	0.85	0.90	0.96	0.81	0.95	1.03	1.20	0.95	1.18	0.85	1.08	0.70	0.82	21.12	5.03
Q8BXZ1	Protein disulfide-isomerase TMX3 OS=Mus musculus GN=Tmx3 PE=1 SV=2 - [TMX3_MOUSE]	22.15	1	7	7	18	1.05	1.02	0.95	1.19	1.00	0.99	0.84	0.95	0.80	1.03	0.92	1.08	0.85	51.82	5.16
D3Z553	Protein Churchill OS=Mus musculus GN=Churc1 PE=2 SV=1 - [D3Z553_MOUSE]	37.14	2	1	1	2	1.17	0.84	0.72	0.65	0.56	1.21	1.02	0.95	0.81	0.89	0.76	0.74	0.63	4.08	4.08
Q8VHJ5	Serine/threonine-protein kinase MARK1 OS=Mus musculus GN=Mark1 PE=1 SV=2 - [MARK1_MOUSE]	22.64	1	8	14	36	0.94	1.12	1.14	1.01	1.04	0.95	1.05	0.95	1.00	1.02	1.07	1.00	1.06	88.28	9.39
Q3U2G2	Heat shock 70 kDa protein 4 OS=Mus musculus GN=Hspa4 PE=2 SV=1 - [Q3U2G2_MOUSE]	61.28	2	35	38	184	0.99	0.95	0.95	1.05	1.07	0.99	0.97	0.95	0.95	0.96	1.00	0.98	0.99	94.15	5.21
Q9CYT6	Adenylyl cyclase-associated protein 2 OS=Mus musculus GN=Cap2 PE=1 SV=1 - [CAP2_MOUSE]	40.76	2	17	17	54	0.97	1.08	1.10	1.06	1.10	0.93	0.95	0.95	0.95	0.95	1.00	1.01	1.00	52.83	6.43
Q8C1Y8	Vacuolar fusion protein CCZ1 homolog OS=Mus musculus GN=Ccz1 PE=1 SV=1 - [CCZ1_MOUSE]	9.17	1	4	4	7	0.97	1.02	1.02	1.10	1.16	0.92	0.92	0.95	0.87	0.95	1.01	0.94	1.01	55.47	6.02
Q8BLU0	Fibronectin leucine rich transmembrane protein 2 OS=Mus musculus GN=Flrt2 PE=2 SV=1 - [Q8BLU0_MOUSE]	3.48	1	2	2	3	1.00	1.30	1.29	1.06	1.06	0.71	0.70	0.95	0.94	0.98	0.98	1.02	1.01	73.90	7.91
Q9R0M4	Podocalyxin OS=Mus musculus GN=Podxl PE=1 SV=2 - [PODXL_MOUSE]	10.74	3	3	4	10	0.88	1.04	1.62	1.00	1.29	1.07	1.19	0.95	1.24	1.06	1.19	0.91	1.01	53.36	4.97

Q9JKP5	Muscleblind-like protein 1 OS=Mus musculus GN=Mbnl1 PE=1 SV=1 - [MBNL1_MOUSE]	18.48	4	1	5	7	1.02	0.71	0.69	0.94	0.93	0.88	0.86	0.95	0.93	0.92	0.91	1.16	1.14	36.95	8.60
P12658	Calbindin OS=Mus musculus GN=Calb1 PE=1 SV=2 - [CALB1_MOUSE]	73.56	1	22	23	208	1.24	0.88	0.73	0.99	0.80	1.04	0.81	0.95	0.78	0.99	0.79	1.10	0.84	29.98	4.83
Q3URI6	Protein Zfp560 OS=Mus musculus GN=Zfp560 PE=2 SV=1 - [Q3URI6_MOUSE]	3.71	1	1	1	7	0.88	0.95	1.09	1.18	1.35	0.74	0.84	0.95	1.08	0.72	0.83	0.96	1.09	85.06	8.10
K3W4Q8	Basigin OS=Mus musculus GN=Bsg PE=4 SV=1 - [K3W4Q8_MOUSE]	39.91	4	7	7	44	1.03	0.94	0.95	1.02	1.01	0.98	0.98	0.95	0.92	0.93	0.88	0.90	0.85	24.10	5.36
Q9D964	Glycine amidinotransferase, mitochondrial OS=Mus musculus GN=Gatm PE=1 SV=1 - [GATM_MOUSE]	13.95	1	4	4	8	1.03	1.22	0.89	1.07	1.12	0.98	0.87	0.95	1.00	1.05	1.01	0.99	1.00	48.27	7.88
Q6GQS1	Calcium-binding mitochondrial carrier protein SCaMC-3 OS=Mus musculus GN=Slc25a23 PE=2 SV=1 - [SCMC3_MOUSE]	15.20	2	5	5	8	1.16	1.53	1.31	1.36	1.22	0.91	0.77	0.95	0.80	0.98	0.84	1.04	1.01	52.46	7.59
A2AEE7	Cadherin EGF LAG seven-pass G-type receptor 2 OS=Mus musculus GN=Celsr2 PE=4 SV=1 - [A2AEE7_MOUSE]	4.93	5	11	11	23	1.00	1.19	1.20	1.03	1.01	0.96	0.99	0.95	0.99	1.01	1.01	0.97	1.01	316.79	5.47
Q3UMB9	WASH complex subunit 7 OS=Mus musculus GN=Kiaa1033 PE=2 SV=2 - [WASH7_MOUSE]	1.62	1	2	2	3	0.79	0.71	0.90	0.79	1.00	0.78	0.99	0.95	1.20	0.72	0.92	0.61	0.78	136.28	7.37
Q8VCK3	Tubulin gamma-2 chain OS=Mus musculus GN=Tubg2 PE=1 SV=1 - [TBG2_MOUSE]	22.62	1	1	6	18	0.83	0.93	1.12	0.91	1.10	0.84	1.00	0.95	1.14	0.82	0.99	1.07	1.29	51.09	6.21
A2AJ14	Endoplasmic reticulum mannosyl-oligosaccharide 1,2-alpha-mannosidase (Fragment) OS=Mus musculus GN=Man1b1 PE=2 SV=1 - [A2AJ14_MOUSE]	4.62	2	1	1	1	0.78	1.12	1.43	1.10	1.40	0.85	1.08	0.95	1.21	1.08	1.38	0.91	1.16	26.79	9.70
G5E829	MCG13663, isoform CRA_a OS=Mus musculus GN=Atp2b1 PE=3 SV=1 - [G5E829_MOUSE]	38.69	1	23	42	254	0.93	1.15	1.16	1.14	1.16	0.90	0.98	0.95	1.00	0.97	1.04	0.90	0.99	134.66	5.91

E9PX52	Arf-GAP with SH3 domain, ANK repeat and PH domain-containing protein 2 OS=Mus musculus GN=Asap2 PE=2 SV=1 - [E9PX52_MOUSE]	15.50	1	2	11	27	0.88	1.12	1.27	1.01	1.14	0.99	1.12	0.95	1.07	0.89	1.01	1.10	1.25	111.09	6.80
Q91W86	Vacuolar protein sorting-associated protein 11 homolog OS=Mus musculus GN=Vps11 PE=1 SV=3 - [VPS11_MOUSE]	6.27	1	5	5	8	1.03	0.92	0.95	1.10	1.00	0.88	0.91	0.95	0.92	0.90	0.93	0.95	0.96	107.65	7.01
Q6PGC1	ATP-dependent RNA helicase Dhx29 OS=Mus musculus GN=Dhx29 PE=2 SV=1 - [DHX29_MOUSE]	1.47	1	1	2	3	0.94	1.05	1.12	1.07	1.14	0.86	0.90	0.95	1.00	1.03	1.09	1.06	1.12	153.88	7.94
Q9EPK2-3	Isoform 3 of Protein XRP2 OS=Mus musculus GN=Rp2 - [XRP2_MOUSE]	7.45	4	2	2	2	0.85	0.64	0.75	0.86	1.01	0.95	1.11	0.95	1.12	0.75	0.88	0.82	0.97	36.54	5.48
P52479	Ubiquitin carboxyl-terminal hydrolase 10 OS=Mus musculus GN=Usp10 PE=1 SV=3 - [UBP10_MOUSE]	7.07	3	4	4	8	1.13	1.14	1.17	1.10	1.00	1.00	1.00	0.95	0.88	1.04	0.99	1.23	1.05	86.97	5.17
Q9CQY6	Mitochondrial nucleoid factor 1 OS=Mus musculus GN=Mnf1 PE=1 SV=1 - [MNF1_MOUSE]	44.12	3	4	4	7	1.04	0.81	0.77	0.99	1.01	1.15	1.06	0.95	0.92	1.08	1.00	1.09	1.03	16.31	9.19
Q8CCJ3-1	Isoform 2 of E3 UFM1-protein ligase 1 OS=Mus musculus GN=Ufl1 - [UFL1_MOUSE]	2.66	3	1	2	5	1.22	1.76	1.45	1.03	0.85	0.94	0.77	0.95	0.78	1.00	0.82	0.93	0.77	80.17	6.68
Q9DB24	Protein Tceal6 OS=Mus musculus GN=Tceal6 PE=2 SV=1 - [Q9DB24_MOUSE]	54.50	1	2	12	205	1.27	0.41	0.30	0.92	0.74	1.18	0.90	0.95	0.76	0.86	0.63	0.85	0.67	22.56	5.55
Q8BQP9	Regulator of G-protein signaling 7-binding protein OS=Mus musculus GN=Rgs7bp PE=1 SV=1 - [R7BP_MOUSE]	20.62	1	3	3	6	0.93	1.22	1.24	1.14	1.22	0.86	0.90	0.95	1.01	1.13	1.09	0.97	1.04	29.00	8.46
Q9D1Q6	Endoplasmic reticulum resident protein 44 OS=Mus musculus GN=Erp44 PE=1 SV=1 - [ERP44_MOUSE]	38.42	1	12	12	26	0.88	0.77	0.82	0.77	0.91	0.81	0.94	0.95	1.04	0.79	0.94	0.75	0.94	46.82	5.27
A2A9S3	ELAV-like protein 4 OS=Mus musculus GN=Elavl4 PE=2 SV=1 - [A2A9S3_MOUSE]	40.73	11	5	11	32	1.06	1.09	1.08	0.98	0.98	0.83	0.82	0.95	0.88	0.91	0.89	1.04	1.01	39.24	9.42
G3UYF9	Prefoldin subunit 6 OS=Mus musculus GN=H2-Ke2 PE=2 SV=1 - [G3UYF9_MOUSE]	57.43	1	1	9	63	1.00	0.74	0.71	0.90	0.75	0.92	0.93	0.95	0.95	1.09	1.00	0.75	0.73	11.40	8.44

Q3UID0	SWI/SNF complex subunit SMARCC2 OS=Mus musculus GN=Smarcc2 PE=2 SV=1 - [Q3UID0_MOUSE]	18.32	8	17	17	44	0.90	0.92	0.95	0.88	0.98	1.00	1.01	0.95	1.02	0.89	0.97	0.99	0.98	124.60	5.47
B2KF50	Protein Uhrf1bp1 OS=Mus musculus GN=Uhrf1bp1 PE=2 SV=1 - [B2KF50_MOUSE]	1.89	1	1	2	3	0.99	0.91	0.91	1.10	1.11	1.05	1.05	0.95	0.95	1.06	1.07	1.22	1.23	156.91	6.21
B8JK33	Heterogeneous nuclear ribonucleoprotein M OS=Mus musculus GN=Hnrnpm PE=2 SV=1 - [B8JK33_MOUSE]	28.28	6	13	13	36	0.90	1.00	1.11	0.96	1.08	0.96	1.05	0.95	1.05	1.01	1.09	1.01	1.11	68.12	8.32
Q9Z1L5	Voltage-dependent calcium channel subunit alpha-2/delta-3 OS=Mus musculus GN=Cacna2d3 PE=1 SV=1 - [CA2D3_MOUSE]	19.62	1	16	16	33	0.99	1.03	0.99	1.12	1.08	0.92	0.94	0.95	0.91	0.96	0.95	1.01	1.02	122.70	5.73
Q8CH18-3	Isoform 3 of Cell division cycle and apoptosis regulator protein 1 OS=Mus musculus GN=Ccar1 - [CCAR1_MOUSE]	2.95	3	3	3	7	1.08	0.84	0.84	1.03	0.95	1.01	1.00	0.95	0.87	1.01	0.89	0.90	0.93	124.67	6.02
Q8R191	Synaptogyrin-3 OS=Mus musculus GN=Syngr3 PE=1 SV=1 - [SNG3_MOUSE]	45.41	1	6	6	19	1.13	1.38	1.05	1.18	0.99	0.88	0.82	0.95	0.87	0.98	0.90	1.14	1.01	24.55	8.18
Q8K4R4	Cytoplasmic phosphatidylinositol transfer protein 1 OS=Mus musculus GN=Pitpnc1 PE=1 SV=1 - [PITC1_MOUSE]	35.24	4	9	9	34	0.89	0.88	0.91	0.93	1.08	0.92	1.06	0.95	1.13	0.88	1.02	0.93	1.06	38.36	6.32
Q04735-2	Isoform Short of Cyclin-dependent kinase 16 OS=Mus musculus GN=Cdk16 - [CDK16_MOUSE]	10.85	50	1	5	9	0.90	0.85	0.95	1.11	1.24	0.96	1.07	0.95	1.06	0.97	1.08	0.91	1.02	52.69	7.17
Q62186	Translocon-associated protein subunit delta OS=Mus musculus GN=Ssr4 PE=2 SV=1 - [SSRD_MOUSE]	25.58	2	3	3	5	1.10	1.00	0.90	0.99	0.90	0.77	0.69	0.95	0.86	0.87	0.79	0.95	0.86	18.92	5.78
F8WJI3	Coiled-coil domain-containing protein 58 OS=Mus musculus GN=Ccdc58 PE=2 SV=1 - [F8WJI3_MOUSE]	54.48	3	6	6	19	1.03	0.88	0.81	1.01	0.90	0.99	0.90	0.95	0.93	0.86	0.83	0.91	0.81	15.59	8.60
B1ARA3	60S ribosomal protein L26 (Fragment) OS=Mus musculus GN=Rpl26 PE=2 SV=1 - [B1ARA3_MOUSE]	24.27	4	3	3	5	0.93	1.19	1.30	1.05	1.13	0.96	1.10	0.95	1.09	0.96	1.13	0.99	0.99	12.21	10.92

Q9QZQ8	Core histone macro-H2A.1 OS=Mus musculus GN=H2afy PE=1 SV=3 - [H2AY_MOUSE]	27.96	2	6	8	30	1.11	1.00	0.94	1.03	0.94	1.06	0.92	0.95	0.88	1.00	0.91	1.13	0.97	39.71	9.80
Q60996-3	Isoform 3 of Serine/threonine-protein phosphatase 2A 56 kDa regulatory subunit gamma isoform OS=Mus musculus GN=Ppp2r5c - [2A5G_MOUSE]	10.72	4	2	4	7	1.10	1.44	1.31	1.22	1.11	0.86	0.78	0.95	0.86	1.01	0.92	0.84	0.77	56.52	6.96
E9PYR7	Protein Mctp1 OS=Mus musculus GN=Mctp1 PE=2 SV=1 - [E9PYR7_MOUSE]	2.82	1	1	1	2	1.06	1.33	1.26	1.07	1.01	1.00	0.94	0.95	0.90	1.02	0.97	1.02	0.97	36.37	7.91
Q9WTU6-	Isoform Alpha-1 of Mitogen-activated protein kinase 9 OS=Mus musculus GN=Mapk9 - [MK09_MOUSE]	29.92	15	5	10	20	0.98	1.05	1.12	0.97	1.01	0.89	0.90	0.95	0.98	0.90	0.96	0.90	1.05	44.05	6.54
D3Z5F9	Protein YIF1B (Fragment) OS=Mus musculus GN=Yif1b PE=2 SV=1 - [D3Z5F9_MOUSE]	13.89	4	1	1	3	0.76	1.03	1.34	1.08	1.41	1.07	1.39	0.95	1.24	0.92	1.20	0.77	1.02	19.26	10.35
Q91XU0-	Isoform 2 of ATPase WRNIP1 OS=Mus musculus GN=Wrnip1 - [WRIP1_MOUSE]	8.10	2	4	4	5	0.96	1.08	1.11	0.92	0.92	0.93	0.95	0.95	0.98	0.92	0.95	0.81	0.83	61.37	5.96
A2APX7	Protein Scn1a OS=Mus musculus GN=Scn1a PE=2 SV=1 - [A2APX7_MOUSE]	6.81	8	8	14	42	0.93	0.90	0.93	1.08	1.11	0.83	0.87	0.95	0.98	0.79	0.88	0.78	0.87	225.85	5.99
P68369	Tubulin alpha-1A chain OS=Mus musculus GN=Tuba1a PE=1 SV=1 - [TBA1A_MOUSE]	77.83	3	15	39	2828	0.98	1.02	1.02	1.03	1.05	0.99	0.99	0.95	0.94	0.99	0.98	1.00	1.04	50.10	5.06
Q6P5U7	Leucine-rich repeat and WD repeat-containing protein KIAA1239 OS=Mus musculus GN=Kiaa1239 PE=2 SV=2 - [K1239_MOUSE]	5.97	2	7	8	15	1.06	1.21	1.22	1.11	1.09	0.95	0.93	0.95	0.95	0.97	0.99	1.09	1.08	197.29	6.15
Q810U4-5	Isoform 5 of Neuronal cell adhesion molecule OS=Mus musculus GN=Nrcam - [NRCAM_MOUSE]	49.62	2	1	8	112	1.02	0.82	0.77	0.96	0.94	0.99	0.95	0.95	1.00	0.94	0.90	0.83	0.81	28.95	8.43
A2AJA9	Uncharacterized protein C9orf172 homolog OS=Mus musculus GN=Gm996 PE=1 SV=1 - [CI172_MOUSE]	20.64	2	15	15	29	1.01	1.09	1.03	1.08	1.12	0.96	1.00	0.95	0.92	0.96	0.94	0.96	0.93	107.12	9.11
Q8CGF6	WD repeat-containing protein 47 OS=Mus musculus GN=Wdr47 PE=1 SV=2 - [WDR47_MOUSE]	18.91	1	11	11	19	1.02	1.09	1.02	1.00	1.00	0.94	0.90	0.95	0.87	0.99	0.93	1.00	0.99	102.25	5.97

Q64737	Trifunctional purine biosynthetic protein adenosine-3 OS=Mus musculus GN=Gart PE=2 SV=3 - [PUR2_MOUSE]	13.07	3	9	9	19	0.97	0.94	0.95	1.09	1.12	0.96	1.05	0.95	0.96	1.08	1.08	1.08	1.12	107.44	6.68
P49615	Cyclin-dependent kinase 5 OS=Mus musculus GN=Cdk5 PE=1 SV=1 - [CDK5_MOUSE]	41.78	49	8	10	24	1.08	1.01	0.90	1.11	1.00	1.02	0.96	0.95	0.92	0.95	0.90	1.11	0.99	33.27	7.66
Q6P4T2	U5 small nuclear ribonucleoprotein 200 kDa helicase OS=Mus musculus GN=Snrnp200 PE=2 SV=1 - [U520_MOUSE]	13.39	1	21	21	36	1.00	1.09	1.07	0.99	1.02	1.00	0.99	0.95	0.94	0.93	0.97	1.01	1.00	244.39	6.06
E9Q1T1	Calcium/calmodulin-dependent protein kinase type II subunit beta OS=Mus musculus GN=Camk2d PE=2 SV=1 - [E9Q1T1_MOUSE]	41.09	15	10	17	101	1.25	1.07	0.91	1.12	0.97	1.01	0.84	0.95	0.78	1.12	0.89	1.12	0.96	59.97	7.27
D3Z0B8	DCN1-like protein 1 (Fragment) OS=Mus musculus GN=Dcun1d1 PE=2 SV=1 - [D3Z0B8_MOUSE]	29.91	5	3	5	12	0.95	1.00	0.99	0.94	1.04	0.92	0.91	0.95	1.00	0.95	1.00	1.04	0.91	24.92	5.25
F8WGN6	Kinesin-like protein KIF21A OS=Mus musculus GN=Kif21a PE=2 SV=1 - [F8WGN6_MOUSE]	31.14	14	33	34	96	1.02	1.01	1.05	1.04	1.08	0.97	0.97	0.95	0.95	0.95	0.98	0.98	0.96	181.26	6.06
P39061-2	Isoform 3 of Collagen alpha-1(XVIII) chain OS=Mus musculus GN=Col18a1 - [COIA1_MOUSE]	1.29	4	1	1	2	1.08	1.29	1.19	1.02	0.95	0.89	0.82	0.95	0.88	1.17	1.08	1.13	1.05	134.12	6.00
O55192	Sodium-dependent noradrenaline transporter OS=Mus musculus GN=Slc6a2 PE=2 SV=2 - [SC6A2_MOUSE]	11.02	1	4	4	8	1.03	1.25	1.12	1.09	1.00	0.89	0.85	0.95	0.90	0.98	0.92	0.96	0.86	69.21	7.44
Q08274	Dystrophia myotonica WD repeat-containing protein OS=Mus musculus GN=Dmwd PE=2 SV=3 - [DMWD_MOUSE]	12.93	2	6	6	10	1.04	1.02	1.02	1.02	0.98	0.95	0.94	0.95	0.91	0.88	0.85	0.97	1.00	69.77	7.24
Q8CBE3	WD repeat-containing protein 37 OS=Mus musculus GN=Wdr37 PE=2 SV=1 - [WDR37_MOUSE]	18.35	7	8	8	33	0.97	0.98	0.99	0.99	1.03	0.95	0.98	0.95	0.97	0.97	1.00	1.03	1.08	55.01	7.23

E9Q5E0	Myocyte-specific enhancer factor 2D OS=Mus musculus GN=Mef2d PE=2 SV=1 - [E9Q5E0_MOUSE]	24.31	5	7	7	11	0.85	0.96	1.19	0.90	1.13	0.91	1.14	0.95	1.23	0.92	1.10	0.88	1.01	54.08	8.38
Q3U2V3-[illegible]	Isoform 2 of 8-oxo-dGDP phosphatase NUDT18 OS=Mus musculus GN=Nudt18 - [NUD18_MOUSE]	14.23	3	3	3	6	1.08	1.37	1.02	0.92	0.87	0.99	0.92	0.95	0.95	0.91	0.88	0.93	0.93	27.34	6.42
P68510	14-3-3 protein eta OS=Mus musculus GN=Ywhah PE=1 SV=2 - [1433F_MOUSE]	72.36	3	14	21	417	0.97	0.88	0.92	0.98	1.02	0.95	0.97	0.95	0.97	0.92	0.93	0.89	0.93	28.19	4.89
Q8K1M6-[illegible]	Isoform 3 of Dynamin-1-like protein OS=Mus musculus GN=Dnm1l - [DNM1L_MOUSE]	55.36	2	2	33	92	0.96	1.31	1.37	1.24	1.30	1.04	1.08	0.95	0.99	1.00	1.05	1.00	1.05	77.96	6.81
Q6NVF9	Cleavage and polyadenylation specificity factor subunit 6 OS=Mus musculus GN=Cpsf6 PE=1 SV=1 - [CPSF6_MOUSE]	20.87	5	9	9	17	0.96	0.85	0.86	0.95	0.99	0.95	0.97	0.95	0.98	0.90	0.91	0.96	1.02	59.12	7.15
Q921J2	GTP-binding protein Rheb OS=Mus musculus GN=Rheb PE=1 SV=1 - [RHEB_MOUSE]	17.93	1	3	3	14	0.96	0.80	0.87	0.99	1.01	0.94	0.89	0.95	0.91	0.91	0.91	0.97	1.00	20.44	5.92
D3Z4N2	Sphingosine-1-phosphate phosphatase 1 (Fragment) OS=Mus musculus GN=Spp1 PE=2 SV=1 - [D3Z4N2_MOUSE]	5.49	3	1	1	2	1.19	1.06	0.89	0.78	0.66	2.45	2.05	0.95	0.80	0.95	0.80	1.08	0.91	17.89	4.30
Q9QZI8	Serine incorporator 1 OS=Mus musculus GN=Serinc1 PE=1 SV=1 - [SERC1_MOUSE]	6.62	1	2	2	9	1.18	1.37	1.26	1.24	1.14	0.92	0.79	0.95	0.85	0.96	1.06	1.00	0.99	50.48	6.28
Q80YA9	Connector enhancer of kinase suppressor of ras 2 OS=Mus musculus GN=Cnksr2 PE=1 SV=1 - [CNKR2_MOUSE]	17.83	3	15	15	38	1.00	1.19	1.23	1.07	1.12	0.96	0.96	0.95	1.06	0.90	0.94	1.01	1.02	117.32	6.79
E9Q4D4	Polycystic kidney disease protein 1-like 2 OS=Mus musculus GN=Pkd1l2 PE=4 SV=2 - [E9Q4D4_MOUSE]	0.33	3	1	1	1	1.04	0.74	0.70	1.03	0.99	1.01	0.96	0.95	0.91	0.93	0.89	0.95	0.91	271.89	5.63
Q1RLL3	Copine-9 OS=Mus musculus GN=Cpne9 PE=2 SV=1 - [CPNE9_MOUSE]	16.09	1	2	7	14	0.97	1.31	1.34	1.15	1.17	1.02	1.07	0.95	1.01	0.85	0.90	0.88	0.89	61.81	5.40
A2ALS7	Protein Rap1gap (Fragment) OS=Mus musculus GN=Rap1gap PE=2 SV=1 - [A2ALS7_MOUSE]	29.63	1	1	8	22	1.11	0.83	0.75	1.04	0.94	0.99	0.89	0.95	0.86	1.08	0.98	1.14	1.04	43.37	6.01

Q6P3Y5-2	Isoform 2 of Zinc finger protein 280C OS=Mus musculus GN=Znf280c - [Z280C_MOUSE]	3.90	3	1	1	2	0.97	0.45	0.47	1.17	1.21	0.87	0.89	0.95	0.98	1.07	1.11	0.74	0.77	77.85	9.20
D3Z634	Latrophilin-3 OS=Mus musculus GN=Lphn3 PE=2 SV=1 - [D3Z634_MOUSE]	18.29	23	22	22	41	1.07	1.10	1.01	1.09	1.02	1.07	0.95	0.95	0.91	1.06	0.97	1.07	0.96	162.92	6.42
Q61249	Immunoglobulin-binding protein 1 OS=Mus musculus GN=Igbp1 PE=1 SV=1 - [IGBP1_MOUSE]	27.94	1	8	8	21	0.89	0.81	0.93	0.84	0.96	0.86	1.01	0.95	1.08	0.84	0.90	0.87	0.99	38.95	6.18
Q64324	Syntaxin-binding protein 2 OS=Mus musculus GN=Stxbp2 PE=2 SV=1 - [STXB2_MOUSE]	1.69	2	1	1	2	1.03	1.29	1.25	1.23	1.20	0.99	0.96	0.95	0.92	1.14	1.11	1.16	1.12	66.32	6.74
Q0VE29	Sterile alpha motif domain-containing protein 12 OS=Mus musculus GN=Samd12 PE=2 SV=1 - [SAM12_MOUSE]	26.71	2	3	3	5	1.08	0.78	0.72	0.96	0.93	0.92	0.90	0.95	0.87	0.99	0.92	0.94	0.87	18.23	9.10
Q8R1T1	Charged multivesicular body protein 7 OS=Mus musculus GN=Chmp7 PE=1 SV=1 - [CHMP7_MOUSE]	11.75	2	4	4	10	0.96	0.78	0.93	0.99	1.03	0.96	1.00	0.95	0.98	0.98	1.08	0.95	1.02	50.60	5.22
Q5DTN8	Janus kinase and microtubule-interacting protein 3 OS=Mus musculus GN=Jakmip3 PE=2 SV=2 - [JKIP3_MOUSE]	8.65	5	4	7	13	0.93	1.05	1.13	0.84	0.96	0.84	0.88	0.95	1.05	0.96	1.03	0.95	1.02	98.65	5.74
Q62261	Spectrin beta chain, non-erythrocytic 1 OS=Mus musculus GN=Sptbn1 PE=1 SV=2 - [SPTB2_MOUSE]	72.75	3	141	153	1268	0.96	0.97	1.02	1.02	1.05	0.97	1.00	0.95	0.99	0.96	1.00	0.99	1.03	274.05	5.58
Q9CQA1	Trafficking protein particle complex subunit 5 OS=Mus musculus GN=Trappc5 PE=1 SV=1 - [TPPC5_MOUSE]	11.17	1	2	2	4	1.05	0.82	0.78	1.02	0.96	0.88	0.83	0.95	0.90	0.95	0.90	1.40	1.33	20.78	9.66
P68368	Tubulin alpha-4A chain OS=Mus musculus GN=Tuba4a PE=1 SV=1 - [TBA4A_MOUSE]	80.58	2	13	36	2632	0.91	0.93	1.06	1.02	1.12	0.92	1.04	0.95	1.02	0.95	1.01	0.92	1.01	49.89	5.06
Q9D4J7-2	Isoform 2 of PHD finger protein 6 OS=Mus musculus GN=Phf6 - [PHF6_MOUSE]	10.56	3	3	3	4	1.18	1.03	1.02	0.93	0.83	0.79	0.71	0.95	0.92	0.88	0.84	1.04	0.85	32.26	8.59
G3UWJ4	28S ribosomal protein S29, mitochondrial (Fragment) OS=Mus musculus GN=Dap3 PE=2 SV=1 - [G3UWJ4_MOUSE]	6.00	7	1	1	2	1.08	0.97	0.90	1.17	1.09	0.96	0.88	0.95	0.88	1.10	1.02	1.14	1.06	23.11	9.48

Q8R0J7	Vacuolar protein sorting-associated protein 37B OS=Mus musculus GN=Vps37b PE=2 SV=1 - [VP37B_MOUSE]	39.30	1	6	6	20	0.90	0.76	0.90	0.85	0.94	1.09	0.98	0.95	1.04	0.93	0.96	0.84	0.94	31.04	7.05
O54941	SWI/SNF-related matrix-associated actin-dependent regulator of chromatin subfamily E member 1 OS=Mus musculus GN=Smarce1 PE=1 SV=1 - [SMCE1_MOUSE]	25.30	1	10	11	30	0.96	0.78	0.82	0.80	0.84	0.98	0.94	0.95	1.04	0.81	0.81	0.85	0.91	46.61	4.88
Q8K448	ATP-binding cassette sub-family A member 5 OS=Mus musculus GN=Abca5 PE=1 SV=2 - [ABCA5_MOUSE]	0.73	1	1	1	2	1.06	0.98	0.93	1.18	1.12	1.03	0.97	0.95	0.90	1.12	1.06	0.90	0.85	185.77	7.20
Q9JKB3	Y-box-binding protein 3 OS=Mus musculus GN=Ybx3 PE=1 SV=2 - [YBOX3_MOUSE]	25.48	5	3	5	12	1.04	1.04	1.00	1.05	1.01	1.00	0.96	0.95	0.92	0.92	0.88	0.99	0.96	38.79	9.69
F6YBB8	RNA-binding protein NOB1 (Fragment) OS=Mus musculus GN=Nob1 PE=4 SV=1 - [F6YBB8_MOUSE]	14.52	3	1	1	1	0.88	0.89	1.00	0.99	1.12	0.98	1.10	0.95	1.08	0.98	1.11	0.99	1.12	7.12	9.41
Q8BGS2	BolA-like protein 2 OS=Mus musculus GN=Bola2 PE=1 SV=1 - [BOLA2_MOUSE]	62.79	2	5	5	34	1.03	0.86	0.91	0.89	0.89	0.94	0.90	0.95	0.93	0.91	0.83	0.92	0.87	10.21	6.16
Q5SWU9	Acetyl-CoA carboxylase 1 OS=Mus musculus GN=Acaca PE=1 SV=1 - [ACACA_MOUSE]	3.88	4	9	9	13	1.00	1.06	1.12	1.07	0.98	0.91	0.91	0.95	1.00	0.94	1.03	1.04	1.02	265.09	6.39
Q9CQ60	6-phosphogluconolactonase OS=Mus musculus GN=Pgls PE=2 SV=1 - [6PGL_MOUSE]	43.19	4	8	8	21	1.00	1.22	1.14	1.08	1.06	0.94	0.91	0.95	0.94	1.01	0.93	0.91	0.98	27.24	5.85
Q8BJI1	Sodium-dependent neutral amino acid transporter SLC6A17 OS=Mus musculus GN=Slc6a17 PE=1 SV=1 - [S6A17_MOUSE]	16.64	2	9	9	48	1.01	1.13	1.06	1.11	1.09	0.93	0.87	0.95	0.94	0.95	0.97	0.95	0.96	81.02	6.23
Q0VBL3	Protein Rbm15 OS=Mus musculus GN=Rbm15 PE=2 SV=1 - [Q0VBL3_MOUSE]	6.44	1	5	5	7	0.96	1.19	1.25	0.96	1.00	0.89	1.05	0.95	0.99	0.98	1.05	1.07	1.21	105.66	10.08

P62715	Serine/threonine-protein phosphatase 2A catalytic subunit beta isoform OS=Mus musculus GN=Ppp2cb PE=1 SV=1 - [PP2AB_MOUSE]	63.75	1	2	15	168	0.95	1.02	1.04	0.98	1.07	0.87	0.91	0.95	1.01	0.94	1.03	0.88	0.92	35.55	5.43
Q9CZU3	Superkiller viralicidic activity 2-like 2 OS=Mus musculus GN=Skiv2l2 PE=2 SV=1 - [SK2L2_MOUSE]	3.46	1	2	2	3	1.07	1.21	1.13	0.97	0.90	0.84	0.78	0.95	0.89	0.96	0.90	1.00	0.94	117.56	6.40
Q76MZ3	Serine/threonine-protein phosphatase 2A 65 kDa regulatory subunit A alpha isoform OS=Mus musculus GN=Ppp2r1a PE=1 SV=3 - [2AAA_MOUSE]	46.18	12	24	24	93	0.99	1.14	1.17	1.12	1.13	0.96	0.95	0.95	0.98	1.03	1.03	1.15	1.12	65.28	5.11
O54781	SRSF protein kinase 2 OS=Mus musculus GN=Srpk2 PE=1 SV=2 - [SRPK2_MOUSE]	21.29	2	10	12	26	0.92	0.90	0.99	1.06	1.15	0.92	1.00	0.95	1.10	0.94	1.02	0.93	1.06	76.71	4.91
Q9D708	Protein S100a16 OS=Mus musculus GN=S100a16 PE=2 SV=1 - [Q9D708_MOUSE]	42.74	2	3	3	16	1.09	1.14	1.04	0.92	0.84	0.98	0.93	0.95	0.98	0.96	0.88	1.00	0.97	14.31	5.86
Q8BJW6-	Isoform 2 of Eukaryotic translation initiation factor 2A OS=Mus musculus GN=Eif2a - [EIF2A_MOUSE]	11.09	3	4	4	8	0.98	1.04	1.16	1.06	1.04	1.00	0.99	0.95	1.06	0.91	0.95	0.97	1.11	54.10	8.98
Q91WL8-	Isoform 2 of WW domain-containing oxidoreductase OS=Mus musculus GN=Wwox - [WWOX_MOUSE]	18.35	4	2	2	4	0.99	1.13	1.14	1.05	1.06	0.93	0.94	0.95	0.97	0.97	0.99	1.07	1.09	17.96	5.64
Q07813	Apoptosis regulator BAX OS=Mus musculus GN=Bax PE=1 SV=1 - [BAX_MOUSE]	17.71	1	2	2	3	0.92	0.83	0.90	0.84	0.91	1.01	1.09	0.95	1.03	0.80	0.87	0.86	0.93	21.38	4.98
Q3TLZ6	Glutamyl-tRNA(Gln) amidotransferase subunit B, mitochondrial OS=Mus musculus GN=Pet112 PE=2 SV=1 - [Q3TLZ6_MOUSE]	7.20	3	2	2	5	0.91	0.92	1.01	1.03	1.13	0.96	1.05	0.95	1.04	0.98	1.10	0.92	1.04	52.51	8.27
Q61282	Aggrecan core protein OS=Mus musculus GN=Acan PE=1 SV=2 - [PGCA_MOUSE]	17.82	1	27	28	97	0.94	0.89	0.94	0.86	0.90	0.92	0.96	0.95	0.96	0.88	0.91	0.83	0.89	221.81	4.30

G3X997	Metallo-beta-lactamase domain-containing protein 2 OS=Mus musculus GN=Mblac2 PE=4 SV=1 - [G3X997_MOUSE]	35.13	2	7	7	14	1.22	1.18	0.98	1.10	0.96	1.03	0.84	0.96	0.84	1.06	0.91	1.15	0.96	31.19	6.86
I7HLV2	60S ribosomal protein L10 (Fragment) OS=Mus musculus GN=Rpl10 PE=4 SV=1 - [I7HLV2_MOUSE]	33.33	4	5	5	23	1.02	0.98	1.07	0.99	1.01	0.92	0.91	0.96	0.92	0.98	0.97	1.03	1.05	23.06	10.01
F6XQM2	Pleckstrin homology domain-containing family O member 1 (Fragment) OS=Mus musculus GN=Plekho1 PE=2 SV=1 - [F6XQM2_MOUSE]	24.11	2	5	5	7	1.01	0.93	1.10	0.93	0.95	0.96	1.08	0.96	1.03	0.90	1.13	0.96	1.02	41.10	8.03
Q9JKY5	Huntingtin-interacting protein 1-related protein OS=Mus musculus GN=Hip1r PE=1 SV=2 - [HIP1R_MOUSE]	21.72	1	18	18	38	0.98	1.09	1.14	1.16	1.20	0.93	0.96	0.96	1.00	1.02	1.03	1.00	1.06	119.35	6.52
G3UW30	1,4-alpha-glucan-branching enzyme OS=Mus musculus GN=Gbe1 PE=4 SV=1 - [G3UW30_MOUSE]	6.21	3	3	3	5	1.04	1.15	1.11	1.11	1.15	0.93	0.96	0.96	0.94	1.01	1.02	1.04	0.96	75.60	6.55
Q9CR68	Cytochrome b-c1 complex subunit Rieske, mitochondrial OS=Mus musculus GN=Uqcrfs1 PE=1 SV=1 - [UCRI_MOUSE]	61.68	1	14	14	119	1.05	0.96	0.94	0.92	0.91	0.89	0.95	0.96	0.91	0.87	0.85	0.89	0.97	29.35	8.70
Q6I6G8	E3 ubiquitin-protein ligase HECW2 OS=Mus musculus GN=Hecw2 PE=2 SV=1 - [HECW2_MOUSE]	6.02	2	5	5	10	0.93	0.91	1.02	1.09	1.10	0.97	1.02	0.96	1.05	0.99	1.05	1.11	1.20	176.13	5.41
Q9D428	Golgin subfamily A member 7B OS=Mus musculus GN=GOLGA7B PE=2 SV=1 - [GOG7B_MOUSE]	18.56	1	3	3	7	1.02	1.10	1.09	1.00	0.97	0.89	0.87	0.96	0.92	0.86	0.88	0.75	0.79	18.32	6.01
Q9ERE2	Keratin, type II cuticular Hb1 (Fragment) OS=Mus musculus GN=Krt81 PE=2 SV=1 - [KRT81_MOUSE]	9.49	6	2	4	43	0.99	1.15	0.84	22.49	25.87	1.30	1.12	0.96	0.97	1.18	0.86	1.06	0.95	43.73	5.20
Q9CY64	Biliverdin reductase A OS=Mus musculus GN=Blvra PE=2 SV=1 - [BIEA_MOUSE]	39.32	4	10	10	15	1.04	0.98	0.91	1.09	1.01	0.97	0.94	0.96	0.90	0.93	0.88	1.05	0.99	33.50	7.02

Q8K337-3	Isoform 3 of Type II inositol 1,4,5-trisphosphate 5-phosphatase OS=Mus musculus GN=Inpp5b - [I5P2_MOUSE]	2.16	2	1	1	2	0.97	1.35	1.38	0.92	0.94	0.98	1.00	0.96	0.98	0.99	1.02	1.33	1.37	84.83	6.35
Q64237	Dopamine beta-hydroxylase OS=Mus musculus GN=Dbh PE=2 SV=1 - [DOPO_MOUSE]	3.38	2	2	2	4	1.11	1.24	1.12	1.11	1.00	0.95	0.85	0.96	0.86	0.97	0.88	1.11	1.00	70.14	6.10
D3Z5N5	1-phosphatidylinositol 3-phosphate 5-kinase OS=Mus musculus GN=Pikfyve PE=2 SV=2 - [D3Z5N5_MOUSE]	2.92	4	4	4	12	0.96	1.26	1.08	1.09	1.02	1.09	1.23	0.96	1.02	1.14	1.21	1.04	1.00	231.93	6.64
Q3KQM4	Splicing factor U2AF 65 kDa subunit OS=Mus musculus GN=U2af2 PE=2 SV=1 - [Q3KQM4_MOUSE]	36.48	2	8	8	22	1.08	0.83	0.79	0.97	0.90	0.82	0.79	0.96	0.92	0.84	0.85	0.92	0.85	33.88	5.03
P25444	40S ribosomal protein S2 OS=Mus musculus GN=Rps2 PE=1 SV=3 - [RS2_MOUSE]	36.52	11	10	10	25	0.95	0.82	0.87	1.14	1.18	1.02	1.04	0.96	1.02	1.00	1.04	1.00	1.03	31.21	10.24
Q8BVR6-	Isoform 3 of RING finger and SPRY domain-containing protein 1 OS=Mus musculus GN=Rspry1 - [RSPRY_MOUSE]	1.77	2	1	1	2	1.06	1.33	1.26	1.15	1.08	0.87	0.82	0.96	0.90	1.10	1.04	1.18	1.12	50.77	5.35
Q62086	Serum paraoxonase/arylesterase 2 OS=Mus musculus GN=Pon2 PE=1 SV=2 - [PON2_MOUSE]	4.80	1	1	1	2	1.26	1.10	0.88	1.12	0.89	0.81	0.64	0.96	0.76	0.96	0.77	1.24	0.99	39.59	5.83
P68404-2	Isoform Beta-II of Protein kinase C beta type OS=Mus musculus GN=Prkcb - [KPCB_MOUSE]	50.97	5	3	29	179	0.88	0.96	1.04	1.01	1.14	1.00	1.09	0.96	1.07	0.99	1.05	0.96	1.04	76.84	7.02
Q91YP0	L-2-hydroxyglutarate dehydrogenase, mitochondrial OS=Mus musculus GN=L2hgdh PE=2 SV=1 - [L2HDH_MOUSE]	24.35	1	9	9	20	0.97	1.26	1.17	1.07	1.00	0.87	0.90	0.96	0.93	1.01	0.98	0.95	1.03	50.87	8.29
D3YVP6	Glutathione S-transferase Mu 7 OS=Mus musculus GN=Gstm7 PE=2 SV=2 - [D3YVP6_MOUSE]	59.12	13	7	11	34	0.99	1.02	1.12	1.09	1.06	1.00	0.94	0.96	0.98	1.03	1.01	1.16	1.11	21.31	8.35
Q99K48	Non-POU domain-containing octamer-binding protein OS=Mus musculus GN=Nono PE=1 SV=3 - [NONO_MOUSE]	41.86	3	15	16	102	0.91	0.98	1.04	0.92	0.99	0.86	0.96	0.96	1.00	0.88	0.95	0.95	1.05	54.51	8.95

D3Z6K8	Ras-specific guanine nucleotide-releasing factor 2 OS=Mus musculus GN=Rasgrf2 PE=2 SV=2 - [D3Z6K8_MOUSE]	10.27	6	9	10	26	1.08	1.32	1.30	1.17	1.09	0.96	0.97	0.96	0.93	1.02	0.95	1.05	0.93	136.02	7.97
Q9DBR1-	Isoform 2 of 5'-3' exoribonuclease 2 OS=Mus musculus GN=Xrn2 - [XRN2_MOUSE]	2.43	2	2	2	2	1.01	1.42	1.41	0.91	0.90	1.00	0.99	0.96	0.95	0.98	0.97	0.99	0.99	108.04	7.25
F8WGL3	Cofilin-1 OS=Mus musculus GN=Cfl1 PE=2 SV=1 - [F8WGL3_MOUSE]	52.86	1	1	22	907	0.63	0.55	0.87	0.98	1.55	0.99	1.56	0.96	1.51	0.91	1.45	0.56	0.89	24.56	8.03
Q64511	DNA topoisomerase 2-beta OS=Mus musculus GN=Top2b PE=1 SV=2 - [TOP2B_MOUSE]	8.93	17	11	13	25	0.99	1.05	1.03	1.01	1.04	0.93	0.96	0.96	0.97	0.97	0.99	0.94	1.04	181.80	8.29
Q61699	Heat shock protein 105 kDa OS=Mus musculus GN=Hsph1 PE=1 SV=2 - [HS105_MOUSE]	49.53	5	31	34	143	0.93	0.92	0.97	1.04	1.11	0.94	0.99	0.96	1.01	0.99	1.05	0.97	1.03	96.35	5.53
D3Z3R3	Thymidylate kinase OS=Mus musculus GN=Dtymk PE=2 SV=1 - [D3Z3R3_MOUSE]	15.58	2	2	2	3	0.97	0.87	0.89	1.09	1.12	0.91	0.93	0.96	0.98	0.91	0.94	0.96	0.99	17.61	7.97
E9PZJ2	Interferon regulatory factor 9 OS=Mus musculus GN=Irf9 PE=2 SV=1 - [E9PZJ2_MOUSE]	7.74	1	1	1	1	0.97	0.69	0.71	1.05	1.08	1.12	1.15	0.96	0.98	1.00	1.04	0.89	0.92	51.45	7.53
O55137	Acyl-coenzyme A thioesterase 1 OS=Mus musculus GN=Acot1 PE=1 SV=1 - [ACOT1_MOUSE]	21.72	8	4	7	18	0.77	0.81	1.10	1.01	1.22	1.03	1.23	0.96	1.22	0.91	1.18	0.94	1.14	46.11	6.58
Q9WVI9-	Isoform JIP-1e of C-Jun-amino-terminal kinase-interacting protein 1 OS=Mus musculus GN=Mapk8ip1 - [JIP1_MOUSE]	2.11	5	1	1	2	0.93	0.99	1.06	0.83	0.90	0.91	0.98	0.96	1.03	1.08	1.17	0.89	0.96	67.96	5.05
Q6NS52-2	Isoform 2 of Diacylglycerol kinase beta OS=Mus musculus GN=Dgkb - [DGKB_MOUSE]	14.97	1	1	10	20	1.14	0.95	0.84	1.02	0.90	1.10	0.96	0.96	0.84	1.00	0.88	1.11	0.98	89.37	7.93
Q68FL6	Methionine--tRNA ligase, cytoplasmic OS=Mus musculus GN=Mars PE=2 SV=1 - [SYMC_MOUSE]	12.97	3	10	10	19	0.96	1.11	1.13	1.03	1.09	0.95	1.03	0.96	0.96	0.96	0.99	0.95	1.06	101.37	7.14
Q9Z2D1	Myotubularin-related protein 2 OS=Mus musculus GN=Mtmr2 PE=1 SV=3 - [MTMR2_MOUSE]	14.15	4	6	6	9	1.04	1.30	1.25	1.09	1.12	1.04	0.92	0.96	0.90	1.00	0.95	1.02	0.99	73.19	7.25

B2RY56	RNA-binding protein 25 OS=Mus musculus GN=Rbm25 PE=1 SV=2 - [RBM25_MOUSE]	15.16	1	10	10	24	0.90	0.81	0.93	0.92	1.02	0.95	1.03	0.96	1.18	0.82	0.98	0.82	0.92	99.49	6.32
Q9CWD8	Iron-sulfur protein NUBPL OS=Mus musculus GN=Nubpl PE=2 SV=2 - [NUBPL_MOUSE]	6.27	1	1	1	1	0.91	1.34	1.48	0.94	1.03	1.15	1.26	0.96	1.05	0.78	0.86	0.80	0.88	34.12	9.07
P30935	Somatostatin receptor type 3 OS=Mus musculus GN=Sstr3 PE=2 SV=2 - [SSR3_MOUSE]	3.27	1	1	1	1	1.25	1.25	1.00	1.19	0.95	1.01	0.80	0.96	0.76	1.10	0.88	1.10	0.88	47.19	8.59
P12849	cAMP-dependent protein kinase type I-beta regulatory subunit OS=Mus musculus GN=Prkar1b PE=2 SV=2 - [KAP1_MOUSE]	25.98	8	5	9	16	0.92	0.92	1.04	1.03	1.10	0.95	1.03	0.96	1.02	1.04	1.03	0.88	0.94	43.20	5.96
P59644	Phosphatidylinositol 4,5-bisphosphate 5-phosphatase A OS=Mus musculus GN=Inpp5j PE=1 SV=2 - [PI5PA_MOUSE]	10.47	2	7	7	17	0.93	0.93	0.97	1.00	1.12	1.01	1.22	0.96	1.10	0.96	1.12	0.95	1.00	107.48	9.36
Q9JJY3	Sphingomyelin phosphodiesterase 3 OS=Mus musculus GN=Smpd3 PE=1 SV=1 - [NSMA2_MOUSE]	10.38	1	6	6	11	1.04	1.12	1.05	1.26	1.17	0.95	0.91	0.96	0.87	1.18	1.10	1.23	1.08	71.15	5.88
Q9D394-4	Isoform 4 of Protein RUFY3 OS=Mus musculus GN=Rufy3 - [RUFY3_MOUSE]	35.65	3	1	15	59	0.72	0.64	1.01	0.89	1.24	0.88	1.15	0.96	1.26	0.91	1.10	0.87	1.14	57.42	5.85
D3Z0M9	MCG18410, isoform CRA_a OS=Mus musculus GN=Ddx23 PE=3 SV=1 - [D3Z0M9_MOUSE]	5.86	1	4	4	10	0.90	0.91	1.01	0.99	1.05	1.00	1.10	0.96	1.19	1.01	1.02	1.03	1.14	95.44	9.58
Q61627	Glutamate receptor ionotropic, delta-1 OS=Mus musculus GN=Grid1 PE=1 SV=2 - [GRID1_MOUSE]	4.16	1	4	4	6	1.04	0.96	0.92	1.04	0.97	1.05	0.94	0.96	0.96	1.01	0.97	1.03	0.97	112.14	6.70
Q3UMB5	Smith-Magenis syndrome chromosomal region candidate gene 8 protein homolog OS=Mus musculus GN=Smcr8 PE=1 SV=2 - [SMCR8_MOUSE]	4.60	2	3	3	4	1.08	1.27	1.15	1.11	1.05	0.97	0.89	0.96	0.86	1.07	0.96	1.09	0.99	104.89	5.40
E9QM38	Solute carrier family 12 member 2 OS=Mus musculus GN=Slc12a2 PE=4 SV=1 - [E9QM38_MOUSE]	15.26	2	11	11	41	0.98	0.98	0.99	1.00	1.01	0.92	0.95	0.96	0.95	0.96	1.00	0.95	0.96	130.59	6.52

P36916	Guanine nucleotide-binding protein-like 1 OS=Mus musculus GN=Gnl1 PE=1 SV=4 - [GNL1_MOUSE]	23.39	2	12	12	27	0.98	1.07	1.03	1.10	1.06	0.98	1.01	0.96	0.97	1.00	1.00	1.04	1.02	68.73	5.68
Q99K01-3	Isoform 3 of Pyridoxal-dependent decarboxylase domain-containing protein 1 OS=Mus musculus GN=Pdxdc1 - [PDXD1_MOUSE]	7.32	6	5	5	10	0.94	1.07	1.05	1.08	1.16	0.92	0.99	0.96	1.00	0.97	1.03	1.02	1.07	78.49	5.54
B0R058	Glycerophosphocholine phosphodiesterase GPCPD1 OS=Mus musculus GN=Gpcpd1 PE=2 SV=1 - [B0R058_MOUSE]	24.55	4	8	8	14	1.08	1.04	1.12	1.01	0.93	0.96	0.91	0.96	0.92	0.93	0.94	1.05	1.01	50.66	6.34
Q7TSV4	Phosphoglucomutase-2 OS=Mus musculus GN=Pgm2 PE=1 SV=1 - [PGM2_MOUSE]	7.10	1	3	3	5	0.93	0.83	0.89	0.94	1.06	0.89	0.99	0.96	1.02	0.87	0.92	0.84	0.95	68.70	6.14
Q8VDJ3	Vigilin OS=Mus musculus GN=Hdlbp PE=1 SV=1 - [VIGLN_MOUSE]	22.08	1	19	19	42	0.96	0.98	1.03	0.95	1.06	0.95	1.04	0.96	1.00	1.00	1.04	0.95	1.03	141.66	6.87
Q8BIZ1	Ankyrin repeat and sterile alpha motif domain-containing protein 1B OS=Mus musculus GN=Anks1b PE=1 SV=3 - [ANS1B_MOUSE]	8.90	8	2	10	22	1.22	1.12	0.84	1.09	0.90	1.03	0.91	0.96	0.85	1.10	0.91	1.14	1.02	138.96	6.29
F7ABZ6	Protein Dnah10 (Fragment) OS=Mus musculus GN=Dnah10 PE=4 SV=1 - [F7ABZ6_MOUSE]	0.37	2	1	2	2	0.96	1.03	1.07	0.97	1.01	1.03	1.07	0.96	1.00	0.92	0.96	0.90	0.94	502.47	5.94
B1ARU4	Microtubule-actin cross-linking factor 1 OS=Mus musculus GN=Macf1 PE=2 SV=1 - [B1ARU4_MOUSE]	15.00	18	84	86	168	0.93	1.00	1.03	1.07	1.15	0.95	1.02	0.96	1.01	0.99	1.05	1.01	1.10	831.13	5.41
D3Z494	MCG142264, isoform CRA_a OS=Mus musculus GN=Akr1b10 PE=4 SV=1 - [D3Z494_MOUSE]	29.17	7	3	6	10	1.20	0.99	0.83	1.41	1.17	1.08	0.90	0.96	0.80	1.07	0.89	1.13	0.95	32.73	7.01
O35393	Ephrin-B3 OS=Mus musculus GN=Efnb3 PE=1 SV=1 - [EFNB3_MOUSE]	16.18	1	3	3	7	0.96	1.06	0.99	0.95	0.90	0.98	1.01	0.96	0.90	1.01	0.96	1.04	0.92	35.86	8.25
Q91VA6	Polymerase delta-interacting protein 2 OS=Mus musculus GN=Poldip2 PE=2 SV=1 - [PDIP2_MOUSE]	24.73	2	6	6	9	0.99	0.97	1.00	1.05	1.05	0.87	0.87	0.96	0.89	0.98	0.91	0.86	0.93	41.84	8.63
Q07409	Contactin-3 OS=Mus musculus GN=Cntn3 PE=2 SV=2 - [CNTN3_MOUSE]	13.91	1	10	11	28	0.99	1.12	1.16	1.07	1.10	0.99	1.00	0.96	0.99	0.91	0.96	1.01	1.03	113.16	6.23

H3BKJ6	POU domain, class 6, transcription factor 1 OS=Mus musculus GN=Pou6f1 PE=4 SV=1 - [H3BKJ6_MOUSE]	37.93	5	2	2	6	0.78	0.69	0.89	0.83	0.95	1.20	1.48	0.96	1.17	0.96	1.11	0.79	0.92	14.67	4.31
Q8BFP9	[Pyruvate dehydrogenase [lipoamide]] kinase isozyme 1, mitochondrial OS=Mus musculus GN=Pdk1 PE=2 SV=2 - [PDK1_MOUSE]	25.58	1	8	8	17	0.98	1.13	1.12	1.00	1.15	0.91	0.99	0.96	0.92	1.05	1.00	1.04	1.05	48.96	8.19
Q80U87	Ubiquitin carboxyl-terminal hydrolase 8 OS=Mus musculus GN=Usp8 PE=1 SV=2 - [UBP8_MOUSE]	6.85	2	6	6	10	1.00	1.00	1.01	0.99	1.06	0.76	0.81	0.96	0.97	0.99	0.92	0.86	1.02	122.53	8.47
Q8BGC4	Zinc-binding alcohol dehydrogenase domain-containing protein 2 OS=Mus musculus GN=Zadh2 PE=2 SV=1 - [ZADH2_MOUSE]	10.34	1	3	3	6	0.85	1.24	1.46	1.12	1.09	0.91	0.92	0.96	0.98	0.94	1.05	0.90	0.98	40.50	7.42
Q80U95	Ubiquitin-protein ligase E3C OS=Mus musculus GN=Ube3c PE=2 SV=2 - [UBE3C_MOUSE]	3.05	1	3	3	4	1.06	1.26	1.20	1.21	1.14	0.98	0.92	0.96	0.91	1.07	1.02	1.26	1.16	123.90	6.39
Q8K2J7-2	Isoform 2 of RELT-like protein 1 OS=Mus musculus GN=Rell1 - [RELL1_MOUSE]	20.79	2	4	4	6	1.06	1.16	1.11	1.05	0.85	1.33	0.94	0.96	0.86	1.15	1.07	0.84	0.79	22.25	8.34
Q9ESP1	Stromal cell-derived factor 2-like protein 1 OS=Mus musculus GN=Sdf2l1 PE=2 SV=2 - [SDF2L_MOUSE]	25.79	1	3	3	7	0.83	0.80	0.92	0.92	1.06	1.00	1.23	0.96	1.16	1.03	1.15	0.93	1.03	23.63	7.42
F6U5Y4	Protein Auts2 (Fragment) OS=Mus musculus GN=Auts2 PE=4 SV=1 - [F6U5Y4_MOUSE]	6.90	4	1	1	1	1.15	1.36	1.18	1.01	0.87	1.00	0.87	0.96	0.83	1.03	0.89	1.16	1.01	16.18	10.26
Q60857	Sodium-dependent serotonin transporter OS=Mus musculus GN=Slc6a4 PE=1 SV=4 - [SC6A4_MOUSE]	2.54	1	1	1	2	1.12	1.36	1.21	1.14	1.01	1.24	1.10	0.96	0.85	1.43	1.28	1.43	1.28	70.00	6.18
Q0P538	B-cell leukemia/lymphoma 2 related protein A1c OS=Mus musculus GN=Bcl2a1c PE=2 SV=1 - [Q0P538_MOUSE]	15.63	1	1	1	2	1.00	0.74	0.74	1.00	1.00	0.77	0.77	0.96	0.95	0.87	0.86	0.95	0.95	14.78	6.79

B2KF34	Mitogen-activated protein kinase 14 (Fragment) OS=Mus musculus GN=Mapk14 PE=2 SV=1 - [B2KF34_MOUSE]	7.88	16	1	2	2	1.04	1.11	1.07	1.05	1.01	1.04	1.00	0.96	0.92	1.11	1.07	1.13	1.09	23.69	8.27
O08529	Calpain-2 catalytic subunit OS=Mus musculus GN=Capn2 PE=2 SV=4 - [CAN2_MOUSE]	36.00	1	17	17	31	1.05	0.90	0.91	1.08	1.05	0.92	0.90	0.96	1.00	0.97	0.91	1.01	1.07	79.82	4.96
Q8R3Q0	Store-operated calcium entry-associated regulatory factor OS=Mus musculus GN=Tmem66 PE=2 SV=2 - [SARAF_MOUSE]	8.68	1	2	2	3	1.03	1.72	1.67	1.23	1.20	0.97	0.94	0.96	0.93	1.13	1.10	0.97	0.95	35.83	8.19
Q9JK42	[Pyruvate dehydrogenase [lipoamide]] kinase isozyme 2, mitochondrial OS=Mus musculus GN=Pdk2 PE=1 SV=2 - [PDK2_MOUSE]	24.82	1	6	7	10	1.10	0.89	0.90	1.17	1.09	0.95	0.94	0.96	0.96	0.96	0.94	1.08	1.09	46.01	6.61
P26443	Glutamate dehydrogenase 1, mitochondrial OS=Mus musculus GN=Glud1 PE=1 SV=1 - [DHE3_MOUSE]	54.84	3	29	29	229	1.02	1.02	1.00	1.06	1.04	0.98	0.97	0.96	0.95	1.05	1.03	1.07	1.08	61.30	8.00
Q811I0	ATP synthase mitochondrial F1 complex assembly factor 1 OS=Mus musculus GN=Atpaf1 PE=2 SV=1 - [ATPF1_MOUSE]	13.27	7	3	3	5	1.09	1.14	1.00	1.05	0.96	1.10	1.02	0.96	0.86	0.93	0.81	1.10	0.97	36.33	8.19
P32233	Developmentally-regulated GTP-binding protein 1 OS=Mus musculus GN=Drg1 PE=1 SV=1 - [DRG1_MOUSE]	18.53	1	5	5	7	1.00	1.04	1.04	0.98	1.05	0.98	0.95	0.96	0.94	1.10	1.01	1.00	0.94	40.49	8.90
P16054	Protein kinase C epsilon type OS=Mus musculus GN=Prkce PE=1 SV=1 - [KPCE_MOUSE]	42.74	5	22	23	78	0.98	0.99	1.03	1.02	1.05	0.93	1.00	0.96	0.97	0.97	1.00	0.98	1.04	83.51	7.03
Q8VCA8	Secernin-2 OS=Mus musculus GN=Scrn2 PE=2 SV=1 - [SCRN2_MOUSE]	8.47	2	2	2	3	1.05	1.04	0.99	1.12	1.06	0.94	0.89	0.96	0.91	1.22	1.16	1.03	0.98	46.57	5.71
Q4VA53	Sister chromatid cohesion protein PDS5 homolog B OS=Mus musculus GN=Pds5b PE=1 SV=1 - [PDS5B_MOUSE]	4.22	4	4	5	11	0.95	1.08	1.02	1.23	1.23	1.01	0.96	0.96	1.04	1.03	1.20	1.10	0.96	164.32	8.50
Q9QUI0	Transforming protein RhoA OS=Mus musculus GN=Rhoa PE=1 SV=1 - [RHOA_MOUSE]	73.06	4	9	11	96	1.04	0.99	0.95	1.06	0.98	1.00	0.96	0.96	0.91	0.98	0.90	1.07	0.95	21.77	6.10

Q6V3W6-	Isoform 2 of Coiled-coil domain-containing protein lobo homolog OS=Mus musculus GN=Ccdc135 - [CC135_MOUSE]	8.14	2	1	1	1	0.64	0.57	0.89	0.91	1.41	0.74	1.14	0.96	1.49	0.44	0.68	0.66	1.03	33.76	5.19
P51791-2	Isoform 2 of H(+)/Cl(-) exchange transporter 3 OS=Mus musculus GN=Clcn3 - [CLCN3_MOUSE]	5.79	5	3	3	5	1.16	1.19	1.02	1.50	1.29	1.09	0.93	0.96	0.82	1.08	0.93	1.03	0.88	84.42	7.25
D3YTT7	40S ribosomal protein SA OS=Mus musculus GN=Rpsa-ps10 PE=3 SV=1 - [D3YTT7_MOUSE]	41.36	2	7	7	27	1.02	1.02	1.06	1.05	1.08	1.00	0.95	0.96	0.90	1.02	1.06	1.04	1.06	32.82	4.93
Q9DCT2	NADH dehydrogenase [ubiquinone] iron-sulfur protein 3, mitochondrial OS=Mus musculus GN=Ndufs3 PE=1 SV=2 - [NDUS3_MOUSE]	43.35	1	13	13	60	0.97	1.01	1.02	1.01	1.06	0.99	1.00	0.96	1.05	0.92	0.96	0.93	0.99	30.13	7.17
Q8BXQ0	Ethanolamine kinase 1 OS=Mus musculus GN=Etnk1 PE=2 SV=1 - [Q8BXQ0_MOUSE]	3.31	2	1	1	2	0.92	1.24	1.34	1.15	1.24	0.96	1.04	0.96	1.04	1.25	1.36	1.24	1.35	41.96	5.44
J3QNB1	La-related protein 1 OS=Mus musculus GN=Larp1 PE=4 SV=1 - [J3QNB1_MOUSE]	22.29	2	16	16	34	0.95	0.93	0.91	0.90	0.94	1.01	0.97	0.96	0.97	0.94	0.97	0.90	0.96	121.05	8.79
E0CXN5	Glycerol-3-phosphate dehydrogenase [NAD(+)], cytoplasmic OS=Mus musculus GN=Gpd1 PE=2 SV=1 - [E0CXN5_MOUSE]	37.12	2	11	11	34	0.87	0.87	0.94	0.99	1.15	0.98	1.11	0.96	1.12	0.90	1.02	0.93	1.06	35.21	7.59
Q80TL1	Adenylate cyclase type 2 OS=Mus musculus GN=Adcy2 PE=2 SV=2 - [ADCY2_MOUSE]	11.38	2	11	11	20	1.03	1.21	1.20	1.09	1.07	0.91	0.85	0.96	0.91	0.99	0.90	1.01	0.93	123.19	8.31
Q8BNU0-	Isoform 2 of Armadillo repeat-containing protein 6 OS=Mus musculus GN=Armc6 - [ARMC6_MOUSE]	31.37	3	6	6	16	1.12	1.07	1.01	1.12	1.04	1.02	0.90	0.96	0.85	1.01	0.88	1.05	0.91	29.64	6.52
D3Z7J6	Translation initiation factor eIF-2B subunit beta (Fragment) OS=Mus musculus GN=Eif2b2 PE=2 SV=1 - [D3Z7J6_MOUSE]	14.91	3	2	2	3	1.08	1.38	1.27	0.89	1.06	0.85	0.87	0.96	0.89	1.04	0.97	1.30	1.20	25.35	6.29

Q5DTL9	Sodium-driven chloride bicarbonate exchanger OS=Mus musculus GN=Slc4a10 PE=1 SV=2 - [S4A10_MOUSE]	26.57	1	19	23	95	0.92	1.05	1.17	1.12	1.26	0.95	0.97	0.96	1.10	0.94	1.05	0.89	1.00	125.74	6.51
P42225	Signal transducer and activator of transcription 1 OS=Mus musculus GN=Stat1 PE=1 SV=1 - [STAT1_MOUSE]	8.14	2	4	4	8	0.94	0.94	1.09	0.99	1.13	0.91	0.97	0.96	0.95	1.00	1.02	1.02	1.11	87.14	5.58
Q69ZX8	Actin-binding LIM protein 3 OS=Mus musculus GN=Ablim3 PE=1 SV=2 - [ABLM3_MOUSE]	18.04	1	10	12	35	0.92	0.93	1.00	0.87	0.93	0.90	0.90	0.96	1.06	0.94	0.94	0.92	1.02	77.58	8.54
Q8BIV3	Ran-binding protein 6 OS=Mus musculus GN=Ranbp6 PE=2 SV=3 - [RNBP6_MOUSE]	1.99	1	1	2	3	0.95	1.22	1.28	1.00	1.05	0.98	1.02	0.96	1.00	0.99	1.04	1.24	1.30	124.51	5.03
Q9Z275	Retinaldehyde-binding protein 1 OS=Mus musculus GN=Rlbp1 PE=2 SV=3 - [RLBP1_MOUSE]	25.55	1	5	5	12	0.94	0.83	0.92	0.99	1.10	1.12	1.11	0.96	1.02	0.98	1.04	0.92	0.99	36.39	5.06
Q9Z1T1	AP-3 complex subunit beta-1 OS=Mus musculus GN=Ap3b1 PE=1 SV=2 - [AP3B1_MOUSE]	3.08	1	1	3	4	0.66	0.98	1.48	1.06	1.61	0.83	1.25	0.96	1.45	1.02	1.54	0.94	1.43	122.66	5.66
Q91VB8	Alpha globin 1 OS=Mus musculus GN=Hba-a1 PE=2 SV=1 - [Q91VB8_MOUSE]	62.68	4	7	7	176	0.84	1.04	1.17	0.60	0.72	0.81	0.94	0.96	1.15	1.12	1.39	0.80	0.93	15.10	8.22
P57080	Ubiquitin carboxyl-terminal hydrolase 25 OS=Mus musculus GN=Usp25 PE=1 SV=2 - [UBP25_MOUSE]	1.33	1	1	2	4	0.94	1.25	1.32	1.00	1.06	0.80	0.85	0.96	1.02	1.02	1.09	1.18	1.26	121.34	5.31
Q6P1F6	Serine/threonine-protein phosphatase 2A 55 kDa regulatory subunit B alpha isoform OS=Mus musculus GN=Ppp2r2a PE=1 SV=1 - [2ABA_MOUSE]	42.51	9	15	15	68	1.02	0.93	0.95	1.04	1.04	1.03	0.98	0.96	1.00	0.96	1.00	1.01	1.01	51.66	6.20
P43006-2	Isoform Glt-1A of Excitatory amino acid transporter 2 OS=Mus musculus GN=Slc1a2 - [EAA2_MOUSE]	40.07	5	3	23	422	0.94	0.94	0.99	1.08	1.07	0.95	1.00	0.96	0.97	1.01	0.97	0.83	0.90	61.73	6.84
A2ARP1-3	Isoform 3 of Inositol hexakisphosphate and diphosphoinositol-pentakisphosphate kinase 1 OS=Mus musculus GN=Ppip5k1 - [VIP1_MOUSE]	4.45	9	5	5	8	1.00	1.15	1.15	1.06	1.06	1.01	0.92	0.96	0.96	1.04	1.06	1.12	1.11	157.45	5.33

G5E866	Splicing factor 3B subunit 1 OS=Mus musculus GN=Sf3b1 PE=4 SV=1 - [G5E866_MOUSE]	18.33	2	17	17	39	0.95	0.94	0.98	1.02	1.09	0.94	0.96	0.96	1.04	0.98	1.02	0.97	1.07	145.74	7.09
Q8C167-2	Isoform 2 of Prolyl endopeptidase-like OS=Mus musculus GN=Prepl - [PPCEL_MOUSE]	23.35	4	10	10	23	1.09	0.92	0.85	1.02	1.02	1.04	1.01	0.96	0.95	1.08	1.10	0.95	0.92	73.17	5.59
P62962	Profilin-1 OS=Mus musculus GN=Pfn1 PE=1 SV=2 - [PROF1_MOUSE]	71.43	3	8	8	89	1.01	0.99	0.98	1.05	1.04	1.00	0.98	0.96	0.92	0.99	0.98	1.07	1.08	14.95	8.28
Q922D4-2	Isoform 2 of Serine/threonine-protein phosphatase 6 regulatory subunit 3 OS=Mus musculus GN=Ppp6r3 - [PP6R3_MOUSE]	15.96	5	10	10	31	0.98	1.01	1.12	0.97	0.97	0.98	1.00	0.96	0.97	0.97	1.00	0.96	0.98	92.56	4.59
Q9D5V6	Synapse-associated protein 1 OS=Mus musculus GN=Syap1 PE=1 SV=1 - [SYAP1_MOUSE]	50.41	1	14	14	58	0.98	0.79	0.74	0.89	0.88	1.01	1.02	0.96	0.96	0.90	0.89	0.91	0.86	41.32	4.54
Q8JZW5	SH2 domain-containing protein 5 OS=Mus musculus GN=Sh2d5 PE=1 SV=2 - [SH2D5_MOUSE]	13.91	2	3	3	4	0.91	1.21	1.33	1.15	1.22	0.88	0.93	0.96	1.06	1.14	1.25	1.28	1.36	38.15	8.32
Q91V14	Solute carrier family 12 member 5 OS=Mus musculus GN=Slc12a5 PE=1 SV=2 - [S12A5_MOUSE]	27.33	3	21	26	136	1.02	1.03	1.00	1.10	1.11	0.86	0.89	0.96	0.94	0.93	0.93	0.90	0.89	126.19	6.74
Q3V116	Polyhomeotic-like protein 1 OS=Mus musculus GN=Phc1 PE=2 SV=1 - [Q3V116_MOUSE]	3.65	6	1	1	3	0.59	0.75	1.27	1.10	1.86	0.88	1.48	0.96	1.63	0.79	1.34	0.84	1.42	100.62	9.25
Q91W61	F-box/LRR-repeat protein 15 OS=Mus musculus GN=Fbxl15 PE=1 SV=2 - [FXL15_MOUSE]	4.00	1	1	1	2	0.95	1.28	1.34	1.12	1.17	1.02	1.06	0.96	1.01	1.24	1.30	1.16	1.21	33.10	7.43
Q8C570	mRNA export factor OS=Mus musculus GN=Rae1 PE=1 SV=1 - [RAE1L_MOUSE]	27.17	2	7	7	16	0.88	0.87	0.95	0.90	1.00	0.97	1.07	0.96	1.06	0.92	0.97	0.89	1.02	40.94	7.83
Q8K0F1	TBC1 domain family member 23 OS=Mus musculus GN=Tbc1d23 PE=2 SV=1 - [TBC23_MOUSE]	1.02	1	1	1	1	1.06	1.01	0.95	1.08	1.02	1.00	0.93	0.96	0.90	1.09	1.02	1.17	1.10	76.38	5.36
G3X9Y1	Synaptotagmin III, isoform CRA_a OS=Mus musculus GN=Syt3 PE=4 SV=1 - [G3X9Y1_MOUSE]	8.69	4	5	5	9	1.03	0.94	0.92	1.15	1.11	0.91	0.92	0.96	0.98	0.97	0.74	0.95	0.93	63.21	6.62

P59764	Dedicator of cytokinesis protein 4 OS=Mus musculus GN=Dock4 PE=1 SV=1 - [DOCK4_MOUSE]	6.32	1	7	7	17	1.06	1.19	1.21	1.10	1.04	1.04	1.02	0.96	0.79	1.08	1.04	1.35	1.24	226.41	7.65
Q8R5J9	PRA1 family protein 3 OS=Mus musculus GN=Arl6ip5 PE=1 SV=2 - [PRAF3_MOUSE]	19.68	1	3	3	10	0.98	1.10	1.25	1.11	1.14	0.93	1.01	0.96	0.99	1.05	1.12	1.07	1.12	21.54	9.61
Q8K097-2	Isoform 2 of Protein lifeguard 2 OS=Mus musculus GN=Faim2 - [LFG2_MOUSE]	12.13	2	2	2	12	1.02	1.02	1.09	0.97	1.00	1.09	1.09	0.96	0.96	0.98	0.99	0.95	0.97	34.24	7.46
Q60790	Ras GTPase-activating protein 3 OS=Mus musculus GN=Rasa3 PE=1 SV=2 - [RASA3_MOUSE]	2.88	1	2	2	4	0.98	1.07	1.09	1.18	1.20	1.06	1.07	0.96	0.98	1.04	1.06	1.45	1.48	95.93	7.34
Q8BIW1	Protein prune homolog OS=Mus musculus GN=Prune PE=2 SV=1 - [PRUNE_MOUSE]	44.49	1	14	14	37	1.03	0.94	0.93	1.02	1.02	0.92	0.89	0.96	0.94	0.95	0.93	0.92	0.90	50.21	5.11
Q60967	Bifunctional 3'-phosphoadenosine 5'-phosphosulfate synthase 1 OS=Mus musculus GN=Papss1 PE=2 SV=1 - [PAPS1_MOUSE]	4.49	1	2	2	4	1.03	0.75	0.72	1.11	1.08	1.04	1.01	0.96	0.93	0.96	0.94	0.98	0.95	70.75	6.77
G3UZY2	Thioredoxin (Fragment) OS=Mus musculus GN=Txn2 PE=3 SV=1 - [G3UZY2_MOUSE]	52.31	4	6	6	158	1.06	0.86	0.89	0.92	0.88	1.03	1.00	0.96	0.95	0.90	0.85	0.85	0.83	14.20	5.81
P61222	ATP-binding cassette sub-family E member 1 OS=Mus musculus GN=Abce1 PE=2 SV=1 - [ABCE1_MOUSE]	17.03	1	8	8	15	0.97	0.78	0.83	0.97	1.04	0.96	0.90	0.96	0.90	0.86	0.89	0.83	0.85	67.27	8.34
Q80W22	Threonine synthase-like 2 OS=Mus musculus GN=Thnsl2 PE=2 SV=1 - [THNS2_MOUSE]	10.35	2	4	4	6	1.17	1.06	0.95	0.99	0.90	0.90	0.77	0.96	0.82	0.93	0.81	1.04	0.90	54.15	6.39
Q810U4-3	Isoform 3 of Neuronal cell adhesion molecule OS=Mus musculus GN=Nrcam - [NRCAM_MOUSE]	41.43	3	3	38	374	0.95	0.91	0.95	1.06	1.04	0.83	0.92	0.96	0.94	0.90	1.05	0.85	0.98	138.95	5.92
O35344	Importin subunit alpha-4 OS=Mus musculus GN=Kpna3 PE=1 SV=1 - [IMA4_MOUSE]	11.13	1	3	5	8	0.94	0.72	0.73	0.98	1.11	1.00	1.05	0.96	1.02	1.03	0.98	0.99	0.93	57.74	4.94

P54116	Erythrocyte band 7 integral membrane protein OS=Mus musculus GN=Stom PE=1 SV=3 - [STOM_MOUSE]	2.82	1	1	1	2	1.00	1.38	1.38	1.09	1.09	0.92	0.91	0.96	0.96	1.21	1.21	1.23	1.24	31.36	6.93
Q9D6F4	Gamma-aminobutyric acid receptor subunit alpha-4 OS=Mus musculus GN=Gabra4 PE=2 SV=1 - [GBRA4_MOUSE]	6.16	1	3	3	5	0.92	1.04	1.23	1.00	1.20	1.04	1.09	0.96	1.01	0.99	1.08	1.11	1.21	60.84	9.32
Q8CGC4	Protein LSM14 homolog B OS=Mus musculus GN=Lsm14b PE=2 SV=3 - [LS14B_MOUSE]	17.66	1	2	5	13	0.97	0.76	0.79	0.79	0.82	0.82	0.84	0.96	0.99	0.68	0.71	0.66	0.68	42.28	9.63
Q8K1A6	Coiled-coil and C2 domain-containing protein 1A OS=Mus musculus GN=Cc2d1a PE=1 SV=2 - [C2D1A_MOUSE]	13.79	2	11	11	23	0.97	1.03	1.03	0.93	0.92	0.94	0.94	0.96	0.99	0.96	0.96	1.05	0.96	103.63	7.84
D3Z0A2	Protein arginine N-methyltransferase 1 OS=Mus musculus GN=Prmt1 PE=2 SV=1 - [D3Z0A2_MOUSE]	14.78	6	2	4	11	1.08	0.92	0.85	0.96	0.86	0.91	0.80	0.96	0.94	0.83	0.77	1.05	1.03	36.54	6.19
Q9WTN0	Geranylgeranyl pyrophosphate synthase OS=Mus musculus GN=Ggps1 PE=2 SV=1 - [GGPPS_MOUSE]	3.67	1	1	1	2	1.10	0.76	0.69	1.16	1.05	1.05	0.95	0.96	0.87	0.92	0.84	0.93	0.85	34.68	6.46
A2ALS5	Protein Rap1gap OS=Mus musculus GN=Rap1gap PE=2 SV=1 - [A2ALS5_MOUSE]	29.83	5	6	13	36	1.07	1.15	1.02	1.07	0.98	1.03	0.91	0.96	0.92	1.04	0.92	1.08	1.01	76.74	6.07
P07356	Annexin A2 OS=Mus musculus GN=Anxa2 PE=1 SV=2 - [ANXA2_MOUSE]	21.83	4	8	8	14	1.10	1.02	0.92	1.02	0.99	0.91	0.79	0.96	0.92	1.04	1.03	0.94	0.82	38.65	7.69
P24270	Catalase OS=Mus musculus GN=Cat PE=1 SV=4 - [CATA_MOUSE]	32.64	2	12	12	26	0.94	1.09	1.16	1.02	1.07	0.91	0.98	0.96	1.03	1.05	1.06	1.04	1.10	59.76	7.88
Q9CZ30	Obg-like ATPase 1 OS=Mus musculus GN=Ola1 PE=1 SV=1 - [OLA1_MOUSE]	37.37	4	12	12	35	0.93	0.80	0.84	1.04	1.02	1.02	1.04	0.96	0.97	0.98	0.98	0.90	0.93	44.70	7.81
F6S185	Phosphoinositide-3-kinase-interacting protein 1 (Fragment) OS=Mus musculus GN=Pik3ip1 PE=4 SV=1 - [F6S185_MOUSE]	9.66	3	1	1	2	0.99	1.32	1.33	1.09	1.10	0.93	0.93	0.96	0.97	0.84	0.85	0.97	0.98	18.78	5.60
Q9Z2N8	Actin-like protein 6A OS=Mus musculus GN=Actl6a PE=1 SV=2 - [ACL6A_MOUSE]	8.39	1	1	2	2	1.04	0.64	0.61	1.00	0.96	0.98	0.93	0.96	0.92	0.93	0.89	0.78	0.75	47.42	5.60

Q91WM1-	Isoform 2 of Spermatid perinuclear RNA-binding protein OS=Mus musculus GN=Strbp - [STRBP_MOUSE]	3.09	2	1	2	5	0.84	0.76	0.90	0.98	1.17	0.91	1.08	0.96	1.14	0.83	0.99	0.88	1.04	70.77	8.51
O35465	Peptidyl-prolyl cis-trans isomerase FKBP8 OS=Mus musculus GN=Fkbp8 PE=1 SV=2 - [FKBP8_MOUSE]	11.69	3	3	3	9	0.98	0.95	0.96	1.05	1.13	1.02	1.06	0.96	0.99	1.02	1.12	1.06	1.08	43.50	5.16
Q9D706	RNA polymerase II-associated protein 3 OS=Mus musculus GN=Rpap3 PE=1 SV=1 - [RPAP3_MOUSE]	18.33	1	9	9	23	1.03	0.97	0.96	0.95	0.95	1.00	0.96	0.96	0.94	0.93	0.91	0.92	0.91	74.05	7.99
Q9D0K2	Succinyl-CoA:3-ketoacid coenzyme A transferase 1, mitochondrial OS=Mus musculus GN=Oxct1 PE=1 SV=1 - [SCOT1_MOUSE]	55.58	2	20	20	167	1.02	1.00	0.98	1.08	1.06	1.00	0.99	0.96	0.96	0.99	0.98	1.06	1.06	55.95	8.53
Q8R570	Synaptosomal-associated protein 47 OS=Mus musculus GN=Snap47 PE=1 SV=1 - [SNP47_MOUSE]	49.15	6	15	15	38	1.01	1.07	1.03	1.01	1.01	1.01	1.00	0.96	0.98	1.00	1.01	0.97	0.97	46.50	5.76
P07759	Serine protease inhibitor A3K OS=Mus musculus GN=Serpina3k PE=1 SV=2 - [SPA3K_MOUSE]	16.27	9	5	5	8	0.82	0.78	1.04	0.56	0.64	0.78	0.94	0.96	1.11	0.83	0.91	0.45	0.61	46.85	5.16
G3UZ21	Ral guanine nucleotide dissociation stimulator-like 2 (Fragment) OS=Mus musculus GN=Rgl2 PE=4 SV=1 - [G3UZ21_MOUSE]	5.71	2	1	1	2	1.13	1.12	0.99	1.06	0.93	1.14	1.00	0.96	0.85	1.24	1.10	1.10	0.97	33.20	7.20
Q9CQJ8	NADH dehydrogenase [ubiquinone] 1 beta subcomplex subunit 9 OS=Mus musculus GN=Ndufb9 PE=1 SV=3 - [NDUB9_MOUSE]	49.16	1	6	6	49	0.92	1.11	1.09	1.09	1.08	0.97	0.97	0.96	1.00	1.00	0.98	1.03	1.04	21.97	7.80
Q6PD28	Protein Ppp2r5b OS=Mus musculus GN=Ppp2r5b PE=2 SV=1 - [Q6PD28_MOUSE]	4.63	1	2	2	4	0.87	1.12	1.28	1.07	1.24	1.07	1.23	0.96	1.11	0.96	1.11	1.10	1.27	57.31	6.84
Q99LB2	Dehydrogenase/reductase SDR family member 4 OS=Mus musculus GN=Dhrs4 PE=2 SV=3 - [DHRS4_MOUSE]	10.04	1	3	3	4	1.12	0.91	0.81	1.03	0.95	0.99	0.88	0.96	0.86	0.98	0.87	1.21	1.08	29.87	9.38

P67984	60S ribosomal protein L22 OS=Mus musculus GN=Rpl22 PE=2 SV=2 - [RL22_MOUSE]	50.78	1	4	4	44	1.04	1.08	1.02	1.05	1.05	0.84	0.83	0.96	0.97	0.93	0.90	0.87	0.83	14.75	9.19
A2AHX7	Bcl-2-like protein 1 (Fragment) OS=Mus musculus GN=Bcl2l1 PE=2 SV=1 - [A2AHX7_MOUSE]	44.87	8	3	3	7	1.27	1.07	0.81	1.08	0.86	1.12	0.89	0.96	0.70	1.07	0.75	0.98	0.82	8.60	4.61
Q9JIA7	Sphingosine kinase 2 OS=Mus musculus GN=Sphk2 PE=1 SV=2 - [SPHK2_MOUSE]	4.54	1	2	2	4	1.00	1.20	1.20	1.10	1.10	0.93	0.93	0.96	0.96	0.96	0.96	0.98	0.98	65.58	6.57
Q9EQQ2	Protein YIPF5 OS=Mus musculus GN=Yipf5 PE=2 SV=1 - [YIPF5_MOUSE]	3.50	1	1	1	2	0.90	1.25	1.38	1.06	1.18	0.77	0.85	0.96	1.07	0.92	1.02	0.97	1.08	27.85	4.36
E9Q9E0	Calcium uptake protein 1, mitochondrial (Fragment) OS=Mus musculus GN=Micu1 PE=2 SV=1 - [E9Q9E0_MOUSE]	22.22	5	4	4	7	0.97	0.99	1.02	1.11	1.20	0.88	0.92	0.96	1.02	0.98	1.03	1.11	1.10	26.70	6.06
Q64343	ATP-binding cassette sub-family G member 1 OS=Mus musculus GN=Abcg1 PE=1 SV=1 - [ABCG1_MOUSE]	1.35	1	1	1	2	0.99	1.26	1.27	1.07	1.08	0.92	0.92	0.96	0.97	0.98	0.99	1.05	1.06	73.98	7.06
D3YWY6	Mitochondrial pyruvate carrier 1 OS=Mus musculus GN=Mpc1 PE=2 SV=1 - [D3YWY6_MOUSE]	9.41	4	1	1	1	0.94	1.31	1.39	1.44	1.52	1.12	1.18	0.96	1.02	1.05	1.11	0.90	0.96	9.73	9.39
Q8VCH8	UBX domain-containing protein 4 OS=Mus musculus GN=Ubxn4 PE=1 SV=1 - [UBXN4_MOUSE]	7.31	1	2	2	6	1.08	0.94	0.87	1.02	0.96	1.04	0.94	0.96	0.97	1.09	0.87	1.06	0.98	56.44	6.61
P97384	Annexin A11 OS=Mus musculus GN=Anxa11 PE=1 SV=2 - [ANX11_MOUSE]	16.10	2	6	7	12	1.02	1.10	1.06	1.07	1.05	0.96	0.94	0.96	0.95	0.96	0.96	0.99	0.96	54.04	7.66
A2AKH7	Leucine-rich repeat-containing protein 57 (Fragment) OS=Mus musculus GN=Lrrc57 PE=2 SV=1 - [A2AKH7_MOUSE]	25.11	3	5	5	10	0.97	0.95	0.94	1.14	1.04	1.00	1.07	0.96	1.05	1.04	0.91	0.97	0.87	24.27	8.18
Q9Z2C4	Myotubularin-related protein 1 OS=Mus musculus GN=Mtmr1 PE=1 SV=1 - [MTMR1_MOUSE]	11.36	5	3	7	15	1.02	1.10	1.10	1.12	1.02	0.89	0.95	0.96	1.00	1.05	1.05	1.06	1.15	75.27	6.80
O89051	Integral membrane protein 2B OS=Mus musculus GN=Itm2b PE=2 SV=1 - [ITM2B_MOUSE]	7.14	1	2	2	2	1.17	0.88	0.75	1.14	0.97	0.92	0.78	0.96	0.82	0.94	0.80	1.02	0.87	30.24	5.30

Q99JN2	Kelch-like protein 22 OS=Mus musculus GN=Klhl22 PE=1 SV=1 - [KLH22_MOUSE]	3.47	3	2	2	3	1.32	1.37	1.04	1.19	0.90	1.00	0.75	0.96	0.73	0.99	0.75	1.06	0.80	71.64	5.71
Q91VZ6	Stromal membrane-associated protein 1 OS=Mus musculus GN=Smap1 PE=1 SV=1 - [SMAP1_MOUSE]	20.91	2	7	8	46	1.03	0.97	0.93	1.00	0.96	0.93	0.94	0.96	0.95	0.92	0.91	0.91	0.87	47.63	8.51
H3BJ89	Syntaxin-5 OS=Mus musculus GN=Stx5a PE=2 SV=1 - [H3BJ89_MOUSE]	45.61	6	2	2	6	1.00	1.29	1.32	1.14	1.14	0.91	0.94	0.96	1.00	1.09	1.08	1.21	1.17	6.68	4.42
Q3UHD2	Glucose-fructose oxidoreductase domain-containing protein 1 OS=Mus musculus GN=Gfod1 PE=2 SV=1 - [GFOD1_MOUSE]	3.85	1	1	1	2	0.93	1.08	1.16	1.19	1.28	0.87	0.94	0.96	1.04	0.85	0.92	0.95	1.02	43.26	5.92
Q8R4H2	Rho guanine nucleotide exchange factor 12 OS=Mus musculus GN=Arhgef12 PE=1 SV=2 - [ARHGC_MOUSE]	12.83	2	15	15	27	0.96	1.09	1.19	1.01	1.07	0.97	1.03	0.96	1.06	1.04	1.08	1.06	1.13	172.24	5.74
Q3TDT0	Tripartite motif-containing protein 3 OS=Mus musculus GN=Trim3 PE=2 SV=1 - [Q3TDT0_MOUSE]	22.73	5	11	14	30	1.01	0.97	0.98	0.98	1.01	1.01	1.03	0.96	0.97	0.97	0.99	0.95	0.99	78.22	8.16
B0V2N1-(	Isoform 6 of Receptor-type tyrosine-protein phosphatase S OS=Mus musculus GN=Ptprs - [PTPRS_MOUSE]	25.31	6	21	27	76	1.03	1.11	1.01	1.02	0.99	0.93	0.92	0.96	0.92	0.95	0.91	0.94	0.98	206.70	7.28
H7BWZ3	Actin-related protein 2/3 complex subunit 3 OS=Mus musculus GN=Arpc3 PE=2 SV=1 - [H7BWZ3_MOUSE]	30.59	4	6	6	25	1.00	1.03	1.13	1.13	1.14	0.95	0.91	0.96	0.91	0.96	0.94	1.00	1.08	19.62	8.60
Q8K394	Inactive phospholipase C-like protein 2 OS=Mus musculus GN=Plcl2 PE=1 SV=2 - [PLCL2_MOUSE]	21.19	1	15	16	36	0.91	0.83	0.93	1.04	1.12	0.97	1.01	0.96	1.01	0.95	1.00	0.95	0.99	125.69	6.92
G3X934	MCG115964 OS=Mus musculus GN=Wdr70 PE=4 SV=1 - [G3X934_MOUSE]	1.52	2	1	1	1	0.83	0.96	1.15	0.78	0.94	1.00	1.19	0.96	1.15	0.79	0.95	0.76	0.91	73.01	6.05
B0QZV3	Protein Slc9a6 OS=Mus musculus GN=Slc9a6 PE=2 SV=1 - [B0QZV3_MOUSE]	9.70	2	6	6	22	1.06	1.16	1.15	1.10	1.05	0.93	0.87	0.96	0.91	0.96	1.00	0.90	0.87	74.18	6.39

D3Z6B9	Mitochondrial 10-formyltetrahydrofolate dehydrogenase OS=Mus musculus GN=Aldh1l2 PE=2 SV=1 - [D3Z6B9_MOUSE]	7.16	2	2	5	7	1.07	1.16	1.08	1.03	0.96	0.97	0.90	0.96	0.89	1.15	1.07	1.14	1.06	88.84	5.58
Q9QYI3	DnaJ homolog subfamily C member 7 OS=Mus musculus GN=Dnajc7 PE=1 SV=2 - [DNJC7_MOUSE]	21.86	3	10	10	24	0.94	0.94	0.98	0.88	0.99	0.96	0.97	0.96	1.03	0.92	0.97	0.99	1.02	56.44	6.49
D3YTM1	Type I inositol 3,4-bisphosphate 4-phosphatase OS=Mus musculus GN=Inpp4a PE=2 SV=1 - [D3YTM1_MOUSE]	20.45	9	13	13	25	0.97	1.08	1.10	1.17	1.12	0.96	0.97	0.96	0.97	0.95	0.96	0.97	1.02	100.52	6.64
Q9CXC9	ETS translocation variant 5 OS=Mus musculus GN=Etv5 PE=2 SV=1 - [ETV5_MOUSE]	2.35	2	1	1	2	0.87	1.37	1.58	1.04	1.20	0.83	0.95	0.96	1.11	0.80	0.93	0.94	1.08	57.67	5.63
P46935	E3 ubiquitin-protein ligase NEDD4 OS=Mus musculus GN=Nedd4 PE=1 SV=3 - [NEDD4_MOUSE]	24.58	1	14	16	41	1.03	1.09	1.09	1.05	1.03	0.94	0.90	0.96	0.91	1.00	1.02	1.00	0.95	102.64	5.26
Q80XU2	Neuronal proto-oncogene tyrosine-protein kinase Src OS=Mus musculus GN=Src PE=2 SV=1 - [Q80XU2_MOUSE]	22.24	2	6	10	21	1.06	1.19	0.99	1.12	1.02	1.02	0.95	0.96	0.90	1.00	0.91	1.01	0.97	59.85	7.42
Q9R0Q6	Actin-related protein 2/3 complex subunit 1A OS=Mus musculus GN=Arpc1a PE=1 SV=1 - [ARC1A_MOUSE]	49.19	4	14	14	37	0.99	0.91	0.91	0.99	1.04	0.95	0.91	0.97	0.91	0.93	0.95	0.96	0.98	41.60	8.18
E9Q3M3	Dynactin subunit 1 OS=Mus musculus GN=Dctn1 PE=2 SV=1 - [E9Q3M3_MOUSE]	37.10	8	37	37	92	0.96	0.98	1.03	1.09	1.15	0.97	0.99	0.97	1.01	1.04	1.08	1.00	1.03	139.68	5.71
Q61235	Beta-2-syntrophin OS=Mus musculus GN=Sntb2 PE=1 SV=2 - [SNTB2_MOUSE]	2.69	1	1	1	2	0.87	1.14	1.31	1.00	1.15	0.78	0.89	0.97	1.10	1.02	1.18	1.04	1.20	56.35	8.69
Q8BGH4	Receptor expression-enhancing protein 1 OS=Mus musculus GN=Reep1 PE=1 SV=1 - [REEP1_MOUSE]	24.88	1	4	4	9	0.93	0.86	0.90	1.04	1.13	0.99	1.02	0.97	1.10	0.96	1.04	0.94	1.09	22.27	9.50
O70443	Guanine nucleotide-binding protein G(z) subunit alpha OS=Mus musculus GN=Gnaz PE=2 SV=4 - [GNAZ_MOUSE]	37.46	1	11	11	40	1.02	1.19	1.14	1.15	1.13	0.96	0.89	0.97	0.92	1.01	0.96	1.03	1.04	40.82	7.61

Q8CHT1-2	Isoform 2 of Ephexin-1 OS=Mus musculus GN=Ngef - [NGEF_MOUSE]	25.16	4	15	15	35	0.98	1.09	1.14	1.14	1.18	0.96	1.02	0.97	1.01	0.97	1.03	1.11	1.13	71.37	5.24
A2ADY9	Protein DDI1 homolog 2 OS=Mus musculus GN=Ddi2 PE=1 SV=1 - [DDI2_MOUSE]	23.81	1	7	7	12	0.90	0.83	0.84	0.98	0.98	0.96	1.01	0.97	1.06	0.93	1.01	0.88	0.99	44.56	5.05
Q8K0D0-2	Isoform 2 of Cyclin-dependent kinase 17 OS=Mus musculus GN=Cdk17 - [CDK17_MOUSE]	17.67	50	3	8	16	0.82	0.83	1.01	0.98	1.11	0.91	0.98	0.97	1.02	0.96	1.02	0.79	0.97	48.94	8.06
P60766	Cell division control protein 42 homolog OS=Mus musculus GN=Cdc42 PE=1 SV=2 - [CDC42_MOUSE]	48.17	8	2	9	60	1.07	1.12	1.03	1.06	1.08	0.97	0.97	0.97	0.89	0.96	0.90	1.03	0.98	21.25	6.55
Q3UMQ8	H/ACA ribonucleoprotein complex non-core subunit NAF1 OS=Mus musculus GN=Naf1 PE=1 SV=2 - [NAF1_MOUSE]	9.61	2	2	2	3	1.06	0.92	0.87	0.86	0.81	0.70	0.65	0.97	0.91	1.04	0.98	1.15	1.09	53.21	5.16
P16283	Anion exchange protein 3 OS=Mus musculus GN=Slc4a3 PE=1 SV=2 - [B3A3_MOUSE]	11.17	8	10	10	15	1.15	1.04	0.97	1.12	1.02	1.07	0.96	0.97	0.92	1.07	0.98	1.17	1.01	135.29	6.51
Q3V0G7-2	Isoform 2 of GTPase-activating Rap/Ran-GAP domain-like protein 3 OS=Mus musculus GN=Garnl3 - [GARL3_MOUSE]	3.22	3	2	2	4	0.93	0.87	0.94	0.99	1.06	1.02	1.09	0.97	1.03	0.92	0.99	1.01	1.08	110.46	7.25
Q8BNL5	BTB/POZ domain-containing protein KCTD6 OS=Mus musculus GN=Kctd6 PE=2 SV=1 - [KCTD6_MOUSE]	8.86	1	2	2	3	1.03	1.20	1.16	0.89	0.86	0.89	0.86	0.97	0.93	0.85	0.83	1.22	1.19	27.61	5.96
Q8BK67	Protein RCC2 OS=Mus musculus GN=Rcc2 PE=2 SV=1 - [RCC2_MOUSE]	15.19	2	7	7	17	1.08	1.04	0.92	1.14	1.08	0.99	0.90	0.97	0.91	1.05	0.91	1.03	1.02	55.95	8.72
D6RG85	FAD-dependent oxidoreductase domain-containing protein 1 OS=Mus musculus GN=Foxred1 PE=2 SV=1 - [D6RG85_MOUSE]	11.29	5	2	2	3	1.05	1.30	1.23	1.02	0.96	0.99	0.93	0.97	0.91	1.01	0.96	1.11	1.05	20.60	9.47
P62889	60S ribosomal protein L30 OS=Mus musculus GN=Rpl30 PE=2 SV=2 - [RL30_MOUSE]	30.43	1	3	3	9	1.06	0.93	0.88	0.95	1.05	0.89	0.86	0.97	0.91	0.94	0.89	1.04	0.98	12.78	9.63
Q9Z140	Copine-6 OS=Mus musculus GN=Cpne6 PE=1 SV=1 - [CPNE6_MOUSE]	40.39	3	15	16	61	1.14	1.18	1.07	1.05	0.87	1.03	0.87	0.97	0.91	1.05	0.90	1.14	0.98	61.74	5.59

Q80VL1	Tudor and KH domain-containing protein OS=Mus musculus GN=Tdrkh PE=1 SV=1 - [TDRKH_MOUSE]	14.82	1	5	5	10	0.99	1.04	0.88	1.01	0.99	1.03	0.88	0.97	0.94	1.01	0.87	0.95	0.91	62.10	4.94
Q9CPZ8	COX assembly mitochondrial protein homolog OS=Mus musculus GN=Cmc1 PE=3 SV=1 - [COXM1_MOUSE]	25.47	1	2	2	4	1.13	0.59	0.66	0.88	0.82	0.99	0.98	0.97	0.92	0.99	0.95	0.90	0.92	12.54	8.24
Q9CWR2	Histone-lysine N-methyltransferase SMYD3 OS=Mus musculus GN=Smyd3 PE=2 SV=1 - [SMYD3_MOUSE]	5.37	2	2	2	3	1.02	1.02	0.99	1.22	1.19	0.92	0.89	0.97	0.94	0.98	0.96	1.10	1.08	49.09	7.08
Q3UG37	Histone deacetylase 6 (Fragment) OS=Mus musculus GN=Hdac6 PE=2 SV=1 - [Q3UG37_MOUSE]	13.78	3	10	10	25	1.01	1.05	0.99	1.05	0.98	0.98	0.96	0.97	0.91	0.99	0.92	0.98	0.97	110.17	5.83
Q8CG76	Aflatoxin B1 aldehyde reductase member 2 OS=Mus musculus GN=Akr7a2 PE=1 SV=3 - [ARK72_MOUSE]	17.98	1	5	5	12	0.94	0.97	0.99	1.06	1.12	1.02	1.06	0.97	1.00	1.07	1.12	1.06	1.12	40.59	8.12
Q9QYH6	Melanoma-associated antigen D1 OS=Mus musculus GN=Maged1 PE=1 SV=1 - [MAGD1_MOUSE]	9.03	2	4	5	11	1.10	0.76	0.68	0.99	0.94	1.14	1.07	0.97	0.93	1.03	1.02	0.95	0.87	85.62	7.50
G3X9S2	Ectonucleotide pyrophosphatase/phosphodiesterase 1, isoform CRA_d OS=Mus musculus GN=Enpp1 PE=1 SV=1 - [G3X9S2_MOUSE]	1.33	3	1	1	2	1.09	1.30	1.19	1.19	1.10	1.02	0.93	0.97	0.89	1.14	1.05	0.86	0.79	102.96	6.57
A2AED3-	Isoform 2 of Fibronectin type III domain-containing protein 7 OS=Mus musculus GN=Fndc7 - [FNDC7_MOUSE]	2.47	4	1	1	1	1.35	0.87	0.64	0.99	0.74	1.23	0.90	0.97	0.71	0.99	0.73	1.18	0.87	34.53	4.96
G3X9T8	Ceruloplasmin OS=Mus musculus GN=Cp PE=4 SV=1 - [G3X9T8_MOUSE]	5.75	5	4	4	8	1.08	1.23	1.16	0.91	0.87	0.78	0.70	0.97	0.92	1.07	0.99	0.89	0.82	121.00	5.85
Q9D0B6	Protein PBDC1 OS=Mus musculus GN=Pbdc1 PE=2 SV=1 - [PBDC1_MOUSE]	33.33	2	6	6	10	0.91	0.76	0.84	0.85	0.93	0.95	0.91	0.97	0.95	0.80	0.78	0.74	0.81	22.21	4.55
P02798	Metallothionein-2 OS=Mus musculus GN=Mt2 PE=1 SV=2 - [MT2_MOUSE]	19.67	1	1	1	5	1.29	0.78	0.60	0.97	0.72	0.98	0.76	0.97	0.70	0.80	0.66	0.91	0.63	6.11	7.83

Q8C5H8-2	Isoform 2 of NAD kinase 2, mitochondrial OS=Mus musculus GN=Nadk2 - [NAKD2_MOUSE]	20.52	4	6	6	16	1.05	1.08	1.00	1.12	1.07	1.03	0.94	0.97	0.90	1.05	1.03	1.12	1.04	43.28	8.85
D3YYK0	Abhydrolase domain containing 11, isoform CRA_b OS=Mus musculus GN=Abhd11 PE=4 SV=1 - [D3YYK0_MOUSE]	3.52	2	1	1	1	1.01	1.05	1.04	1.07	1.06	0.97	0.96	0.97	0.96	0.93	0.92	1.13	1.12	24.87	8.24
Q9R0P3	S-formylglutathione hydrolase OS=Mus musculus GN=Esd PE=2 SV=1 - [ESTD_MOUSE]	34.75	8	7	7	23	1.06	0.95	0.92	1.16	0.96	0.93	0.86	0.97	0.95	1.01	0.95	1.01	0.94	31.30	7.12
Q61335	B-cell receptor-associated protein 31 OS=Mus musculus GN=Bcap31 PE=1 SV=4 - [BAP31_MOUSE]	24.49	1	6	6	11	1.05	0.86	0.89	1.15	1.09	0.94	1.01	0.97	0.95	0.98	0.98	0.96	0.88	27.94	8.70
A2A5K2	Phospholipid transfer protein OS=Mus musculus GN=Pltp PE=2 SV=1 - [A2A5K2_MOUSE]	6.58	2	1	1	3	0.78	0.85	1.08	0.99	1.26	0.82	1.05	0.97	1.23	0.87	1.12	0.76	0.98	48.99	6.47
Q8VCY8-	Isoform 2 of Lipid phosphate phosphatase-related protein type 2 OS=Mus musculus GN=Lppr2 - [LPPR2_MOUSE]	10.84	2	3	3	9	1.08	1.18	1.13	1.29	1.17	0.93	0.83	0.97	0.84	0.98	0.85	1.10	0.99	48.38	9.45
Q8BHL3	TBC1 domain family member 10B OS=Mus musculus GN=Tbc1d10b PE=1 SV=2 - [TB10B_MOUSE]	19.42	1	9	9	29	1.02	0.89	0.95	1.03	1.11	1.00	1.00	0.97	0.98	1.01	1.04	0.98	0.91	87.22	8.90
D3Z2J6	Thioredoxin-related transmembrane protein 2 OS=Mus musculus GN=Tmx2 PE=2 SV=1 - [D3Z2J6_MOUSE]	25.68	2	5	5	20	1.27	1.25	1.04	1.32	1.10	0.88	0.77	0.97	0.75	0.92	0.96	1.04	0.92	29.57	8.72
O88845	A-kinase anchor protein 10, mitochondrial OS=Mus musculus GN=Akap10 PE=1 SV=3 - [AKA10_MOUSE]	13.44	3	6	6	11	0.96	1.08	1.14	1.06	1.09	0.98	1.03	0.97	0.98	0.99	1.09	1.03	1.06	73.59	6.79
P84096	Rho-related GTP-binding protein RhoG OS=Mus musculus GN=Rhog PE=2 SV=1 - [RHOG_MOUSE]	60.73	8	8	9	40	1.02	1.03	1.07	1.10	1.07	1.05	0.94	0.97	0.94	1.05	0.97	1.11	1.08	21.29	8.12
Q3TES0	IQ motif and SEC7 domain-containing protein 3 OS=Mus musculus GN=Iqsec3 PE=1 SV=1 - [IQEC3_MOUSE]	8.54	2	7	7	12	0.92	0.91	0.97	0.97	1.05	0.87	0.81	0.97	0.90	1.01	1.03	0.95	1.00	129.04	6.19

F6ZFT1	Acyl carrier protein (Fragment) OS=Mus musculus GN=Ndufab1 PE=2 SV=1 - [F6ZFT1_MOUSE]	35.43	3	5	6	31	1.02	0.64	0.54	0.90	0.74	1.03	0.94	0.97	0.91	0.90	0.72	0.90	0.72	14.30	4.77
Q9R1Z7	6-pyruvoyl tetrahydrobiopterin synthase OS=Mus musculus GN=Pts PE=2 SV=2 - [PTPS_MOUSE]	49.31	1	5	5	13	1.26	0.84	0.79	0.88	0.79	1.02	0.82	0.97	0.84	0.94	0.83	0.93	0.85	16.18	6.52
E9PYL4	H(+)/Cl(-) exchange transporter 7 OS=Mus musculus GN=Clcn7 PE=2 SV=1 - [E9PYL4_MOUSE]	2.43	2	1	1	1	0.88	2.46	2.79	1.88	2.13	1.33	1.50	0.97	1.10	1.30	1.47	1.99	2.27	86.44	6.90
Q6ZWU9	40S ribosomal protein S27 OS=Mus musculus GN=Rps27 PE=1 SV=3 - [RS27_MOUSE]	28.57	4	2	2	6	0.98	0.90	1.02	1.07	1.00	1.00	1.04	0.97	1.06	0.94	1.03	1.05	1.07	9.45	9.45
Q91Z53	Glyoxylate reductase/hydroxypyruvate reductase OS=Mus musculus GN=Grhpr PE=1 SV=1 - [GRHPR_MOUSE]	42.07	3	11	11	25	1.04	1.04	1.10	1.07	1.02	0.99	0.93	0.97	0.94	1.02	1.03	1.07	1.21	35.31	7.65
Q9JMA1	Ubiquitin carboxyl-terminal hydrolase 14 OS=Mus musculus GN=Usp14 PE=1 SV=3 - [UBP14_MOUSE]	46.65	3	18	18	69	1.07	1.06	0.98	1.01	0.99	1.03	0.99	0.97	0.96	0.98	0.91	1.05	1.00	55.97	5.24
M0QWJ9	Protein Tmem178b OS=Mus musculus GN=Tmem178b PE=4 SV=1 - [M0QWJ9_MOUSE]	6.98	1	1	1	2	1.09	1.05	0.96	1.12	1.03	0.84	0.76	0.97	0.89	0.92	0.85	0.95	0.87	19.43	9.77
Q7TT50	Serine/threonine-protein kinase MRCK beta OS=Mus musculus GN=Cdc42bpb PE=1 SV=2 - [MRCKB_MOUSE]	12.26	18	14	18	34	0.97	0.86	1.02	0.98	1.08	0.87	0.90	0.97	0.95	0.99	0.96	0.96	1.01	194.63	6.46
Q8VC42	Uncharacterized protein C18orf8 homolog OS=Mus musculus GN=Mic1 PE=2 SV=1 - [MIC1_MOUSE]	4.41	1	2	2	4	0.94	0.92	0.97	1.07	1.13	0.94	1.00	0.97	1.02	0.93	0.99	0.93	0.98	74.87	7.83
A2ALL9	Protein Atp2b3 OS=Mus musculus GN=Atp2b3 PE=2 SV=1 - [A2ALL9_MOUSE]	33.50	3	19	34	177	1.04	1.05	1.05	1.19	1.17	0.90	0.90	0.97	0.97	0.96	0.91	1.00	0.96	128.66	6.04
Q9Z1R4	Uncharacterized protein C6orf47 homolog OS=Mus musculus GN=D17h6s53e PE=2 SV=1 - [CF047_MOUSE]	2.39	1	1	1	1	1.18	1.01	0.86	1.16	0.98	1.10	0.93	0.97	0.82	1.08	0.91	1.05	0.89	31.99	6.89
J3QPE8	MCG16555 OS=Mus musculus GN=Vdac3-ps1 PE=4 SV=1 - [J3QPE8_MOUSE]	47.00	3	9	10	57	1.10	1.12	0.99	1.18	1.04	1.03	0.94	0.97	0.87	1.02	0.92	1.12	1.02	30.68	8.66

Q91V89	Protein Ppp2r5d OS=Mus musculus GN=Ppp2r5d PE=2 SV=1 - [Q91V89_MOUSE]	24.75	1	10	11	30	1.00	0.95	0.95	1.14	1.14	1.07	1.04	0.97	1.00	1.02	1.03	1.07	1.08	68.95	7.96
G3UXW9	COP9 signalosome complex subunit 1 OS=Mus musculus GN=Gps1 PE=4 SV=1 - [G3UXW9_MOUSE]	22.81	5	8	8	17	0.98	1.01	0.95	0.97	0.99	1.00	0.98	0.97	1.00	1.00	0.97	1.06	1.04	58.82	6.74
Q9EQJ9-2	Isoform 2 of Membrane-associated guanylate kinase, WW and PDZ domain-containing protein 3 OS=Mus musculus GN=Magi3 - [MAGI3_MOUSE]	17.58	3	13	14	22	0.94	0.94	1.01	0.92	0.94	1.05	1.11	0.97	1.09	0.99	1.00	0.99	1.04	123.28	6.38
Q6PNC0	DmX-like protein 1 OS=Mus musculus GN=Dmxl1 PE=1 SV=1 - [DMXL1_MOUSE]	1.56	2	4	4	6	1.04	0.92	0.89	1.16	1.13	0.82	0.87	0.97	1.02	0.75	0.73	1.07	1.03	335.80	6.42
Q80YD1	ATP-dependent RNA helicase SUPV3L1, mitochondrial OS=Mus musculus GN=Supv3l1 PE=2 SV=1 - [SUV3_MOUSE]	3.47	1	2	2	4	1.37	1.71	1.24	0.99	0.72	0.93	0.68	0.97	0.70	0.87	0.63	1.07	0.78	86.95	7.84
Q920Q4-2	Isoform 2 of Vacuolar protein sorting-associated protein 16 homolog OS=Mus musculus GN=Vps16 - [VPS16_MOUSE]	11.69	3	3	3	8	0.99	1.35	1.04	1.18	1.13	0.89	0.86	0.97	0.96	1.12	1.03	1.19	1.07	47.88	8.56
Q75N73	Zinc transporter ZIP14 OS=Mus musculus GN=Slc39a14 PE=1 SV=1 - [S39AE_MOUSE]	1.84	1	1	1	2	0.97	0.93	0.95	1.22	1.25	0.92	0.94	0.97	0.99	1.06	1.09	1.02	1.05	53.93	5.27
Q9JI18	Low-density lipoprotein receptor-related protein 1B OS=Mus musculus GN=Lrp1b PE=2 SV=1 - [LRP1B_MOUSE]	2.11	3	7	8	18	0.99	1.02	1.00	1.01	1.00	1.00	0.99	0.97	0.93	0.97	1.02	1.08	1.04	513.30	5.39
Q6ZPJ3	Ubiquitin-conjugating enzyme E2 O OS=Mus musculus GN=Ube2o PE=1 SV=3 - [UBE2O_MOUSE]	26.24	1	22	22	74	1.01	1.02	1.01	1.00	1.00	0.95	0.93	0.97	0.96	0.97	0.97	1.01	1.02	140.75	5.06
P35282	Ras-related protein Rab-21 OS=Mus musculus GN=Rab21 PE=1 SV=4 - [RAB21_MOUSE]	23.42	1	4	4	16	1.13	1.09	0.99	1.01	0.92	0.87	0.82	0.97	0.85	1.03	0.92	1.16	1.03	24.09	7.94
E9PY43	Protein Erich1 OS=Mus musculus GN=Erich1 PE=2 SV=1 - [E9PY43_MOUSE]	3.92	1	1	1	2	0.99	1.16	1.18	1.10	1.11	1.09	1.10	0.97	0.98	0.94	0.95	1.02	1.03	37.33	5.45
Q3U9G9	Lamin-B receptor OS=Mus musculus GN=Lbr PE=1 SV=2 - [LBR_MOUSE]	2.72	1	2	2	3	1.09	1.06	0.97	1.05	0.96	0.83	0.76	0.97	0.88	1.04	0.95	0.91	0.84	71.39	9.36

Q8K2Q5	Coiled-coil-helix-coiled-coil-helix domain-containing protein 7 OS=Mus musculus GN=Chchd7 PE=2 SV=1 - [CHCH7_MOUSE]	58.82	4	2	6	19	1.00	0.87	0.96	0.94	0.96	1.07	1.11	0.97	0.79	0.86	0.87	0.78	0.86	10.09	8.51
B2Z892	6-phosphofructo-2-kinase/fructose-2, 6-bisphosphatase 2 variant 4 OS=Mus musculus GN=Pfkfb2 PE=2 SV=1 - [B2Z892_MOUSE]	7.04	18	3	3	7	1.11	0.98	0.88	1.02	0.93	1.09	0.94	0.97	0.85	1.04	0.90	1.01	0.94	57.30	8.16
Q91YS7	Dual-specificity mitogen-activated protein kinase kinase 2 OS=Mus musculus GN=Map2k2 PE=2 SV=1 - [Q91YS7_MOUSE]	23.75	3	4	9	29	1.06	1.04	1.01	1.10	1.04	0.99	0.96	0.97	0.92	0.93	1.01	1.19	1.00	44.27	7.05
P37040	NADPH--cytochrome P450 reductase OS=Mus musculus GN=Por PE=1 SV=2 - [NCPR_MOUSE]	33.92	5	16	16	32	1.01	1.06	1.02	1.14	1.14	0.91	0.89	0.97	0.95	1.02	0.99	0.98	1.03	77.00	5.53
Q3USB7	Inactive phospholipase C-like protein 1 OS=Mus musculus GN=Plcl1 PE=1 SV=3 - [PLCL1_MOUSE]	18.25	1	15	17	33	0.99	1.02	1.06	1.05	1.05	1.01	1.00	0.97	1.03	0.99	0.97	0.97	1.00	122.59	5.64
Q6PIP5	NudC domain-containing protein 1 OS=Mus musculus GN=Nudcd1 PE=2 SV=2 - [NUDC1_MOUSE]	12.54	1	5	5	10	0.93	1.00	1.10	1.04	1.12	0.87	0.88	0.97	0.87	0.89	0.85	0.97	0.84	66.66	5.30
Q69Z38	Pseudopodium-enriched atypical kinase 1 OS=Mus musculus GN=Peak1 PE=1 SV=4 - [PEAK1_MOUSE]	5.36	1	7	7	14	0.97	1.02	0.95	0.87	0.86	0.99	1.01	0.97	1.02	0.91	0.94	0.92	0.95	190.98	6.87
Q8BYA0	Tubulin-specific chaperone D OS=Mus musculus GN=Tbcd PE=2 SV=1 - [TBCD_MOUSE]	2.84	1	3	3	6	1.06	1.28	1.13	1.11	1.08	0.88	0.89	0.97	0.89	1.00	0.88	1.23	1.17	133.24	6.51
P05063	Fructose-bisphosphate aldolase C OS=Mus musculus GN=Aldoc PE=1 SV=4 - [ALDOC_MOUSE]	84.85	2	24	30	505	0.99	1.09	1.08	1.02	1.01	0.98	0.98	0.97	0.98	0.97	0.98	0.98	0.99	39.37	7.12
Q3TRB3	Palmitoyltransferase ZDHHC17 OS=Mus musculus GN=Zdhhc17 PE=2 SV=1 - [Q3TRB3_MOUSE]	3.52	3	1	1	2	0.99	1.09	1.09	1.15	1.16	1.12	1.12	0.97	0.97	1.05	1.06	0.91	0.92	28.44	6.07
Q792Y8	MCG15081 OS=Mus musculus GN=Gm10334 PE=3 SV=1 - [Q792Y8_MOUSE]	12.20	3	1	2	5	0.70	0.75	1.07	0.65	0.92	0.94	1.34	0.97	1.38	0.79	1.13	1.08	1.55	26.10	4.92

Q8BRR9	Calcium/calmodulin-dependent 3',5'-cyclic nucleotide phosphodiesterase 1A OS=Mus musculus GN=Pde1a PE=2 SV=1 - [Q8BRR9_MOUSE]	26.06	13	12	12	24	1.02	1.00	1.11	1.03	1.06	1.00	0.94	0.97	0.94	0.98	0.98	1.02	1.00	62.31	5.91
Q8R4T1	Cystin-1 OS=Mus musculus GN=Cys1 PE=1 SV=1 - [CYS1_MOUSE]	15.86	1	1	1	3	1.01	1.12	1.11	0.97	0.96	1.06	1.05	0.97	0.96	1.04	1.04	0.84	0.83	15.50	5.25
Q923D2	Flavin reductase (NADPH) OS=Mus musculus GN=Blvrb PE=2 SV=3 - [BLVRB_MOUSE]	33.50	3	5	5	10	0.88	0.86	1.06	0.92	1.01	0.86	0.99	0.97	1.08	0.86	0.96	0.83	0.97	22.18	7.01
Q8BVL3	Sorting nexin-17 OS=Mus musculus GN=Snx17 PE=1 SV=2 - [SNX17_MOUSE]	5.32	3	2	2	4	0.93	1.03	1.11	0.93	1.00	1.00	1.08	0.97	1.04	0.95	1.02	0.99	1.07	52.76	7.49
Q99MX7	Cat eye syndrome critical region protein 6 homolog OS=Mus musculus GN=Cecr6 PE=1 SV=1 - [CECR6_MOUSE]	8.04	1	2	2	6	0.91	1.08	1.12	1.07	1.15	1.01	1.08	0.97	1.08	0.97	1.02	0.92	1.01	58.12	9.01
Q9WTR1	Transient receptor potential cation channel subfamily V member 2 OS=Mus musculus GN=Trpv2 PE=1 SV=2 - [TRPV2_MOUSE]	8.20	1	4	4	15	0.99	1.05	1.00	1.15	1.02	0.91	0.83	0.97	0.97	0.97	0.94	0.92	0.88	85.91	6.99
Q5F2D9	Arrestin, beta 2 OS=Mus musculus GN=Arrb2 PE=4 SV=1 - [Q5F2D9_MOUSE]	6.40	3	1	2	6	1.18	0.98	0.83	1.06	0.90	1.03	0.87	0.97	0.82	1.06	0.90	1.12	0.95	45.70	7.83
A2ATK9	Family with sequence similarity 171, member A1 OS=Mus musculus GN=Fam171a1 PE=2 SV=1 - [A2ATK9_MOUSE]	14.46	4	8	9	18	1.08	1.03	0.95	0.94	0.86	1.06	0.97	0.97	0.94	0.92	0.85	1.00	0.85	97.90	6.40
F7B6K4	Pantothenate kinase 4 (Fragment) OS=Mus musculus GN=Pank4 PE=4 SV=1 - [F7B6K4_MOUSE]	6.55	3	2	3	5	1.05	1.59	1.51	1.23	1.17	1.03	0.97	0.97	0.92	1.13	1.08	1.17	1.12	53.24	6.40
Q80TJ1-2	Isoform 2 of Calcium-dependent secretion activator 1 OS=Mus musculus GN=Cadps - [CAPS1_MOUSE]	28.66	3	1	25	72	1.04	1.01	0.98	1.27	1.23	1.01	0.97	0.97	0.93	1.12	1.08	1.08	1.05	153.91	5.86
E9Q6C7	Latrophilin-2 OS=Mus musculus GN=Lphn2 PE=2 SV=1 - [E9Q6C7_MOUSE]	4.24	5	5	5	11	0.98	0.94	1.01	1.05	0.99	0.95	1.00	0.97	0.99	0.89	0.91	0.91	0.89	166.47	6.71

Q9D6E4	Tetratricopeptide repeat protein 9B OS=Mus musculus GN=Ttc9b PE=2 SV=1 - [TTC9B_MOUSE]	24.69	1	2	3	9	0.90	0.95	1.08	0.93	1.02	1.04	1.19	0.97	1.14	0.84	0.94	0.91	1.05	25.89	9.48
Q6P9N1	Hyccin OS=Mus musculus GN=Fam126a PE=1 SV=2 - [HYCCI_MOUSE]	8.25	1	2	3	12	0.99	1.08	1.06	0.96	1.00	1.00	1.04	0.97	0.98	1.06	1.12	1.22	1.15	57.27	7.88
Q7TQ95	Protein lunapark OS=Mus musculus GN=Lnp PE=1 SV=1 - [LNP_MOUSE]	29.41	5	8	8	22	1.07	0.92	0.84	1.02	0.94	1.10	0.98	0.97	0.94	1.00	0.98	1.04	0.96	47.47	5.27
Q61043-2	Isoform 2 of Ninein OS=Mus musculus GN=Nin - [NIN_MOUSE]	1.04	3	1	2	2	1.99	1.29	0.65	1.37	0.69	1.25	0.62	0.97	0.49	0.95	0.48	1.60	0.81	233.46	4.97
Q571H0	Nucleolar pre-ribosomal-associated protein 1 OS=Mus musculus GN=Urb1 PE=2 SV=2 - [NPA1P_MOUSE]	0.88	4	1	2	3	0.92	1.16	1.26	0.86	0.93	0.93	1.01	0.97	1.05	0.97	1.06	0.93	1.01	254.45	6.96
Q80ZS3	28S ribosomal protein S26, mitochondrial OS=Mus musculus GN=Mrps26 PE=2 SV=1 - [RT26_MOUSE]	25.00	1	4	4	8	1.00	0.85	0.88	1.02	1.01	1.07	1.12	0.97	0.97	0.99	0.98	1.05	1.05	23.43	9.96
Q3U1Y4-	Isoform 2 of DENN domain-containing protein 4B OS=Mus musculus GN=Dennd4b - [DEN4B_MOUSE]	1.24	3	1	1	2	0.93	1.25	1.35	1.24	1.34	1.13	1.22	0.97	1.04	1.21	1.30	1.26	1.36	134.05	8.02
Q8BHA1	Leucine-rich repeat-containing protein 24 OS=Mus musculus GN=Lrrc24 PE=2 SV=1 - [LRC24_MOUSE]	4.41	1	1	1	2	1.17	0.91	0.77	0.94	0.80	0.88	0.75	0.97	0.83	0.89	0.76	0.98	0.84	56.30	8.21
D3Z4M2	Ceramide synthase 2 (Fragment) OS=Mus musculus GN=Cers2 PE=2 SV=1 - [D3Z4M2_MOUSE]	6.92	4	1	1	2	1.17	1.27	1.09	1.35	1.16	1.07	0.91	0.97	0.83	1.05	0.90	0.94	0.81	15.50	9.64
Q6P1J1	Crmp1 protein OS=Mus musculus GN=Crmp1 PE=2 SV=1 - [Q6P1J1_MOUSE]	70.41	2	27	33	343	1.07	0.97	0.91	1.01	0.95	1.00	0.95	0.97	0.92	0.97	0.90	1.03	0.98	74.17	6.81
Q6PB44-2	Isoform 2 of Tyrosine-protein phosphatase non-receptor type 23 OS=Mus musculus GN=Ptpn23 - [PTN23_MOUSE]	11.78	2	13	13	36	0.96	1.09	1.14	1.10	1.18	0.95	0.97	0.97	1.00	1.01	1.09	1.05	1.05	184.84	6.76
B8QI35	Liprin-alpha 3 OS=Mus musculus GN=Ppfia3 PE=2 SV=1 - [B8QI35_MOUSE]	38.86	3	34	39	149	0.94	1.01	1.05	0.98	1.03	0.95	1.00	0.97	1.02	0.93	0.99	0.91	0.98	133.34	5.64
Q8C166	Copine-1 OS=Mus musculus GN=Cpne1 PE=1 SV=1 - [CPNE1_MOUSE]	13.81	9	5	5	12	1.02	1.16	1.22	1.15	1.09	0.93	0.78	0.97	0.98	1.04	0.99	1.06	1.04	58.85	5.66

Q91WG5	5'-AMP-activated protein kinase subunit gamma-2 OS=Mus musculus GN=Prkag2 PE=1 SV=2 - [AAKG2_MOUSE]	12.72	5	3	5	9	0.93	1.15	1.09	1.10	1.21	1.00	1.07	0.97	1.04	1.19	1.08	1.14	1.19	62.91	9.36
P97364	Selenide, water dikinase 2 OS=Mus musculus GN=Sephs2 PE=1 SV=3 - [SPS2_MOUSE]	8.63	1	2	2	6	1.05	1.21	1.15	0.98	0.96	0.85	0.81	0.97	0.88	0.88	0.85	0.93	0.95	47.80	6.06
Q3TZZ7	Extended synaptotagmin-2 OS=Mus musculus GN=Esyt2 PE=1 SV=1 - [ESYT2_MOUSE]	8.17	2	5	5	9	0.90	1.10	1.22	1.15	1.23	0.98	0.98	0.97	0.95	0.94	1.04	1.12	1.10	94.08	7.75
Q03141-2	Isoform 2 of MAP/microtubule affinity-regulating kinase 3 OS=Mus musculus GN=Mark3 - [MARK3_MOUSE]	22.50	3	5	12	24	0.95	1.17	1.23	1.00	1.06	0.92	0.97	0.97	1.02	0.95	0.97	0.97	1.07	81.35	9.44
P23116	Eukaryotic translation initiation factor 3 subunit A OS=Mus musculus GN=Eif3a PE=1 SV=5 - [EIF3A_MOUSE]	21.73	1	29	29	75	0.94	1.05	1.01	1.01	1.05	0.98	0.96	0.97	0.98	0.95	1.00	0.96	0.97	161.84	6.77
O55128	Histone deacetylase complex subunit SAP18 OS=Mus musculus GN=Sap18 PE=1 SV=1 - [SAP18_MOUSE]	17.65	5	3	3	5	0.94	0.72	0.72	0.96	1.02	0.98	1.04	0.97	1.07	0.90	0.96	0.86	0.97	17.58	9.35
Q99LG2	Transportin-2 OS=Mus musculus GN=Tnpo2 PE=2 SV=1 - [TNPO2_MOUSE]	9.81	2	3	4	7	1.09	1.17	1.13	1.05	0.93	0.85	0.78	0.97	0.79	1.01	0.82	1.06	0.98	100.39	4.98
J3QP81	CLIP-associating protein 1 OS=Mus musculus GN=Clasp1 PE=4 SV=1 - [J3QP81_MOUSE]	10.69	6	11	13	24	0.94	1.16	1.20	1.08	1.04	0.91	0.92	0.97	0.98	0.96	0.99	1.06	1.10	160.82	8.68
Q3U0K8-2	Isoform 2 of 2-oxoglutarate and iron-dependent oxygenase domain-containing protein 1 OS=Mus musculus GN=Ogfod1 - [OGFD1_MOUSE]	7.97	4	3	3	6	0.92	0.92	1.03	0.88	0.97	0.99	1.17	0.97	1.07	0.97	1.10	0.95	1.05	57.82	4.91
Q3UU96-2	Isoform 2 of Serine/threonine-protein kinase MRCK alpha OS=Mus musculus GN=Cdc42bpa - [MRCKA_MOUSE]	7.43	20	6	9	19	1.02	1.15	1.07	1.07	1.02	0.98	0.95	0.97	0.98	1.01	0.96	0.99	1.00	173.72	6.35
A2AWI9	Endophilin-B2 OS=Mus musculus GN=Sh3glb2 PE=4 SV=1 - [A2AWI9_MOUSE]	40.37	2	1	13	61	0.92	0.77	0.84	0.97	1.00	0.95	1.00	0.97	1.02	0.91	0.93	0.82	0.86	41.78	5.97

Q8BVY4	Protein Supt3 OS=Mus musculus GN=Supt3 PE=2 SV=1 - [Q8BVY4_MOUSE]	3.21	1	1	1	2	0.89	1.16	1.30	0.84	0.94	0.96	1.06	0.97	1.08	1.05	1.18	1.13	1.27	41.21	8.54
Q9CX66	Uncharacterized protein C12orf45 homolog OS=Mus musculus GN=D10Wsu102e PE=2 SV=1 - [CL045_MOUSE]	31.35	1	3	3	6	0.81	0.84	1.05	0.83	0.98	1.02	1.22	0.97	1.19	1.07	1.24	0.87	0.97	19.95	4.84
Q02105	Complement C1q subcomponent subunit C OS=Mus musculus GN=C1qc PE=2 SV=2 - [C1QC_MOUSE]	12.60	1	2	2	19	0.96	1.02	1.13	0.97	1.02	0.98	0.94	0.97	1.01	1.01	1.00	0.97	1.07	25.97	8.54
Q8VIG3	Radial spoke head 1 homolog OS=Mus musculus GN=Rsph1 PE=1 SV=2 - [RSPH1_MOUSE]	3.32	1	1	1	7	1.44	1.23	0.73	0.99	0.64	1.33	0.92	0.97	0.67	1.46	0.96	1.34	0.93	34.16	4.53
Q3UJU9	Regulator of microtubule dynamics protein 3 OS=Mus musculus GN=Rmdn3 PE=1 SV=2 - [RMD3_MOUSE]	28.09	4	9	9	18	0.95	1.03	1.03	1.06	1.09	0.87	0.84	0.97	1.01	0.95	1.00	0.92	1.00	52.00	5.21
P13597-2	Isoform 2 of Intercellular adhesion molecule 1 OS=Mus musculus GN=Icam1 - [ICAM1_MOUSE]	1.69	2	1	1	1	1.10	1.16	1.05	0.84	0.76	1.05	0.95	0.97	0.88	0.85	0.77	1.01	0.92	58.72	6.21
O09165	Calsequestrin-1 OS=Mus musculus GN=Casq1 PE=2 SV=3 - [CASQ1_MOUSE]	13.33	2	3	3	12	0.71	1.15	1.19	0.78	1.08	14.13	18.25	0.97	1.50	1.25	1.55	1.10	1.25	46.35	4.12
A7XUY5-	Isoform 2 of Selection and upkeep of intraepithelial T-cells protein 5 OS=Mus musculus GN=Skint5 - [SKIT5_MOUSE]	0.56	4	1	1	1	1.01	1.19	1.17	1.00	0.98	1.05	1.03	0.97	0.96	0.95	0.94	0.93	0.92	163.40	8.16
P54823	Probable ATP-dependent RNA helicase DDX6 OS=Mus musculus GN=Ddx6 PE=2 SV=1 - [DDX6_MOUSE]	36.85	1	11	11	44	0.98	1.09	1.00	1.02	1.01	0.99	1.00	0.97	0.95	0.96	0.98	0.97	1.01	54.16	8.66
Q8CAG6	Pleckstrin OS=Mus musculus GN=Plek PE=2 SV=1 - [Q8CAG6_MOUSE]	4.28	2	1	1	2	1.06	1.27	1.19	0.90	0.85	1.02	0.96	0.97	0.92	1.05	1.00	1.04	0.99	37.13	7.43
Q9D4I9	RAB23, member RAS oncogene family, isoform CRA_a OS=Mus musculus GN=Rab23 PE=2 SV=1 - [Q9D4I9_MOUSE]	48.10	3	9	9	17	1.15	1.00	0.93	1.07	0.93	1.05	0.95	0.97	0.80	1.06	0.93	1.15	0.96	26.76	7.30
Q8C6E0	Coiled-coil domain-containing protein 104 OS=Mus musculus GN=Ccdc104 PE=2 SV=1 - [CC104_MOUSE]	68.51	2	16	16	46	1.06	0.86	0.75	0.92	0.86	1.03	0.99	0.97	0.92	0.90	0.84	0.89	0.89	39.58	4.93

Q8K2U2	Alpha-ketoglutarate-dependent dioxygenase alkB homolog 6 OS=Mus musculus GN=Alkbh6 PE=2 SV=2 - [ALKB6_MOUSE]	3.40	2	1	1	3	1.30	1.00	0.77	1.03	0.85	1.16	0.89	0.97	0.72	0.99	0.77	1.04	0.86	26.39	7.55
Q921K2	Poly (ADP-ribose) polymerase family, member 1 OS=Mus musculus GN=Parp1 PE=2 SV=1 - [Q921K2_MOUSE]	15.78	3	11	11	22	0.95	0.82	0.89	0.98	1.04	1.02	1.09	0.97	1.01	0.93	0.98	0.89	1.01	112.65	8.95
P39053-4	Isoform 4 of Dynamin-1 OS=Mus musculus GN=Dnm1 - [DYN1_MOUSE]	70.14	15	3	55	507	0.97	1.18	1.11	1.04	1.08	1.00	0.96	0.97	1.02	0.96	0.99	1.08	1.11	97.36	7.17
Q8QZZ7	TP53RK-binding protein OS=Mus musculus GN=Tprkb PE=2 SV=1 - [TPRKB_MOUSE]	6.29	1	1	1	2	0.91	1.11	1.21	0.84	0.92	0.90	0.98	0.97	1.06	0.97	1.06	1.07	1.17	19.54	7.39
P70408	Cadherin-10 OS=Mus musculus GN=Cdh10 PE=2 SV=3 - [CAD10_MOUSE]	16.75	2	6	10	21	0.90	0.93	1.12	0.98	1.14	1.01	1.11	0.97	1.14	0.94	1.12	0.83	0.98	88.26	4.94
Q8VDN2	Sodium/potassium-transporting ATPase subunit alpha-1 OS=Mus musculus GN=Atp1a1 PE=1 SV=1 - [AT1A1_MOUSE]	53.47	1	31	54	1204	0.93	1.05	1.09	1.10	1.18	0.93	0.97	0.97	1.03	0.94	1.01	0.91	0.96	112.91	5.45
Q3V0K9	Plastin-1 OS=Mus musculus GN=Pls1 PE=2 SV=1 - [PLSI_MOUSE]	5.24	1	1	3	5	0.94	1.47	1.57	1.09	1.16	0.92	0.98	0.97	1.04	0.93	0.99	1.00	1.06	70.36	5.38
A2AWA9	Rab GTPase-activating protein 1 OS=Mus musculus GN=Rabgap1 PE=2 SV=1 - [RBGP1_MOUSE]	17.86	5	16	18	47	0.93	0.99	1.07	1.08	1.11	1.01	1.04	0.97	1.03	0.95	1.06	0.94	1.05	120.72	5.25
Q99L47	Hsc70-interacting protein OS=Mus musculus GN=St13 PE=2 SV=1 - [F10A1_MOUSE]	33.42	4	13	13	94	0.99	0.74	0.75	0.90	0.90	0.97	0.98	0.97	0.98	0.89	0.88	0.85	0.86	41.63	5.26
Q61290	Voltage-dependent R-type calcium channel subunit alpha-1E OS=Mus musculus GN=Cacna1e PE=1 SV=1 - [CAC1E_MOUSE]	11.49	2	17	17	40	1.04	1.22	1.17	1.09	1.05	0.97	0.97	0.97	1.01	0.96	0.97	1.06	1.08	257.07	8.18
P15209	BDNF/NT-3 growth factors receptor OS=Mus musculus GN=Ntrk2 PE=1 SV=1 - [NTRK2_MOUSE]	18.76	4	7	10	25	0.99	0.93	1.07	1.02	1.07	0.94	0.95	0.97	0.95	1.00	1.00	0.99	0.98	92.07	6.55
O88998	Noelin OS=Mus musculus GN=Olfm1 PE=1 SV=1 - [NOE1_MOUSE]	31.13	7	11	11	68	1.04	1.01	1.03	0.94	0.95	0.94	0.91	0.97	0.95	0.87	0.86	0.94	0.92	55.36	6.95

P56565	Protein S100-A1 OS=Mus musculus GN=S100a1 PE=1 SV=2 - [S10A1_MOUSE]	29.79	2	2	2	108	1.14	0.80	0.65	0.95	0.77	1.03	0.87	0.97	0.84	0.88	0.73	0.96	0.80	10.50	4.50
Q3TLS3	GDP-D-glucose phosphorylase 1 OS=Mus musculus GN=Gdpgp1 PE=2 SV=2 - [GDPP1_MOUSE]	10.36	1	3	3	8	1.09	1.02	0.90	1.13	1.04	0.95	0.85	0.97	0.92	0.93	0.86	1.04	0.98	42.49	6.19
E9Q7U2	Calcium-binding and coiled-coil domain-containing protein 1 OS=Mus musculus GN=Calcoco1 PE=2 SV=1 - [E9Q7U2_MOUSE]	41.86	3	21	21	57	0.90	0.90	1.02	0.89	1.00	0.98	1.14	0.97	1.14	0.86	0.99	0.89	0.99	70.89	4.86
Q61361	Brevican core protein OS=Mus musculus GN=Bcan PE=1 SV=2 - [PGCB_MOUSE]	38.96	1	27	27	296	1.02	1.01	0.96	0.99	0.97	0.95	0.95	0.97	0.94	0.93	0.96	0.96	0.95	95.75	4.93
E9PWG2	Protein Trappc8 OS=Mus musculus GN=Trappc8 PE=2 SV=1 - [E9PWG2_MOUSE]	3.55	2	4	4	6	1.05	1.01	0.96	1.02	0.98	0.92	0.84	0.97	0.98	0.98	0.93	1.16	1.17	160.81	7.01
Q9JIG8	PRA1 family protein 2 OS=Mus musculus GN=Praf2 PE=2 SV=1 - [PRAF2_MOUSE]	6.18	1	1	1	4	0.95	1.03	1.08	1.18	1.24	1.00	1.04	0.97	1.02	1.09	1.15	1.05	1.10	19.47	9.60
Q9WVG6	Isoform 2 of Histone-arginine methyltransferase CARM1 OS=Mus musculus GN=Carm1 - [CARM1_MOUSE]	3.93	4	2	2	5	1.02	1.14	1.12	1.07	1.05	0.99	0.96	0.97	0.95	1.07	1.05	1.08	1.06	63.42	6.61
F6TBK9	Supervillin (Fragment) OS=Mus musculus GN=Svil PE=4 SV=1 - [F6TBK9_MOUSE]	10.59	1	1	1	4	1.30	1.19	0.92	0.98	0.75	1.04	0.80	0.98	0.75	1.06	0.82	1.15	0.89	19.03	5.82
Q91Y09	Protein Pcdha9 OS=Mus musculus GN=Pcdhac2 PE=2 SV=1 - [Q91Y09_MOUSE]	3.08	9	2	2	4	1.10	1.20	1.09	1.14	1.04	1.11	1.00	0.98	0.88	1.05	0.96	1.06	0.96	109.42	5.47
Q8R3N1	Nucleolar protein 14 OS=Mus musculus GN=Nop14 PE=1 SV=2 - [NOP14_MOUSE]	1.40	1	1	1	1	0.87	0.87	1.00	0.92	1.06	0.96	1.10	0.98	1.11	1.03	1.18	0.88	1.01	98.71	7.59
Q924D1	Cytochrome P450 CYP2J9 OS=Mus musculus GN=Cyp2j9 PE=2 SV=1 - [Q924D1_MOUSE]	4.58	1	2	2	4	0.92	1.17	1.27	1.03	1.11	0.79	0.85	0.98	1.06	1.02	1.11	0.97	1.06	57.90	7.75
Q9WUR2-	Isoform 2 of Enoyl-CoA delta isomerase 2, mitochondrial OS=Mus musculus GN=Eci2 - [ECI2_MOUSE]	17.32	9	4	4	9	0.91	0.75	0.82	0.98	1.11	0.97	1.03	0.98	1.21	0.93	1.07	0.99	1.14	39.48	8.43
Q69ZR9-2	Isoform 2 of Protein FAM208A OS=Mus musculus GN=Fam208a - [F208A_MOUSE]	0.73	2	1	1	2	1.03	0.97	0.93	1.03	0.99	1.00	0.96	0.98	0.94	1.05	1.01	1.07	1.04	168.72	6.01

P27671	Ras-specific guanine nucleotide-releasing factor 1 OS=Mus musculus GN=Rasgrf1 PE=1 SV=2 - [RGRF1_MOUSE]	8.40	1	7	8	15	1.00	0.93	1.03	1.11	1.10	0.96	1.03	0.98	1.06	0.99	0.97	0.92	1.05	144.01	7.17
Q8C050-2	Isoform 1 of Ribosomal protein S6 kinase alpha-5 OS=Mus musculus GN=Rps6ka5 - [KS6A5_MOUSE]	3.13	2	2	2	7	1.08	1.42	1.29	1.12	1.05	1.13	1.04	0.98	0.96	1.21	1.11	1.19	1.02	89.46	7.25
P59114	Phosphorylated CTD-interacting factor 1 OS=Mus musculus GN=Pcif1 PE=1 SV=1 - [PCIF1_MOUSE]	3.40	1	2	2	3	0.96	0.94	0.97	1.10	1.14	1.09	1.13	0.98	1.01	1.00	1.05	1.11	1.16	80.45	7.64
O88696	Putative ATP-dependent Clp protease proteolytic subunit, mitochondrial OS=Mus musculus GN=Clpp PE=2 SV=1 - [CLPP_MOUSE]	22.06	1	4	4	7	1.01	1.06	1.15	1.18	1.05	1.10	1.05	0.98	1.02	1.05	1.03	1.18	1.11	29.78	7.47
Q7TPR4	Alpha-actinin-1 OS=Mus musculus GN=Actn1 PE=1 SV=1 - [ACTN1_MOUSE]	63.00	1	1	53	311	1.04	1.97	1.90	1.10	1.06	1.03	0.98	0.98	0.94	0.94	0.91	0.83	0.81	103.00	5.38
Q99LB6-2	Isoform 2 of Methionine adenosyltransferase 2 subunit beta OS=Mus musculus GN=Mat2b - [MAT2B_MOUSE]	28.17	3	7	7	22	0.94	1.02	1.11	1.02	1.03	0.80	0.82	0.98	0.95	0.87	0.85	0.90	0.95	36.19	6.60
Q4VBE8	WD repeat-containing protein 18 OS=Mus musculus GN=Wdr18 PE=1 SV=1 - [WDR18_MOUSE]	7.19	1	1	1	2	1.11	0.78	0.70	1.10	0.99	0.87	0.78	0.98	0.88	0.90	0.82	0.94	0.85	47.18	6.89
Q9CPX4	Ferritin OS=Mus musculus GN=Ftl1 PE=2 SV=1 - [Q9CPX4_MOUSE]	44.26	4	6	6	11	0.97	0.98	0.84	0.82	0.86	0.99	0.99	0.98	0.83	0.94	0.88	1.21	1.19	20.76	6.00
Q8BLR9-2	Isoform 2 of Hypoxia-inducible factor 1-alpha inhibitor OS=Mus musculus GN=Hif1an - [HIF1N_MOUSE]	4.18	2	1	1	2	0.98	1.09	1.11	1.19	1.22	0.96	0.97	0.98	0.99	0.97	0.99	1.22	1.25	30.41	6.55
D3YWE6	Protein Mn1 OS=Mus musculus GN=Mn1 PE=4 SV=1 - [D3YWE6_MOUSE]	1.93	1	1	1	4	0.90	1.18	1.32	0.97	1.08	1.14	1.27	0.98	1.09	1.06	1.19	1.12	1.25	133.99	6.74
Q8CCI5	RING1 and YY1-binding protein OS=Mus musculus GN=Rybp PE=1 SV=1 - [RYBP_MOUSE]	24.56	1	2	2	5	0.97	1.57	1.61	1.07	1.10	1.22	1.25	0.98	1.00	1.03	1.06	1.11	1.14	24.76	9.63
P46061	Ran GTPase-activating protein 1 OS=Mus musculus GN=Rangap1 PE=1 SV=2 - [RAGP1_MOUSE]	22.92	1	11	11	22	1.02	0.86	0.90	1.14	1.09	1.06	1.01	0.98	0.96	1.06	1.07	1.01	1.04	63.49	4.68

Q80VP1	Epsin-1 OS=Mus musculus GN=Epn1 PE=1 SV=3 - [EPN1_MOUSE]	25.39	5	11	11	75	1.00	0.82	0.78	0.90	0.89	0.94	0.90	0.98	0.97	0.83	0.78	0.81	0.81	60.17	4.81
B1AUN2	MCG14442 OS=Mus musculus GN=Eif2b3 PE=4 SV=1 - [B1AUN2_MOUSE]	13.50	3	4	4	8	1.05	1.21	1.05	1.06	1.05	0.92	0.90	0.98	0.92	1.04	1.03	1.07	1.06	50.46	6.74
P61358	60S ribosomal protein L27 OS=Mus musculus GN=Rpl27 PE=2 SV=2 - [RL27_MOUSE]	27.94	3	3	3	12	0.99	1.37	1.37	1.21	1.23	1.12	1.02	0.98	0.96	0.96	0.94	0.97	0.97	15.79	10.56
P53986	Monocarboxylate transporter 1 OS=Mus musculus GN=Slc16a1 PE=1 SV=1 - [MOT1_MOUSE]	7.91	1	3	3	7	0.99	1.10	1.03	1.21	1.25	0.93	0.89	0.98	0.96	0.99	1.01	0.89	0.91	53.23	7.47
P32848	Parvalbumin alpha OS=Mus musculus GN=Pvalb PE=1 SV=3 - [PRVA_MOUSE]	52.73	1	5	5	12	0.85	0.74	0.80	0.80	0.91	1.05	1.18	0.98	1.03	0.82	0.88	0.69	0.73	11.92	5.19
Q99L45	Eukaryotic translation initiation factor 2 subunit 2 OS=Mus musculus GN=Eif2s2 PE=1 SV=1 - [IF2B_MOUSE]	56.50	3	21	21	94	1.08	0.92	0.88	0.99	0.91	0.98	0.93	0.98	0.91	0.87	0.84	0.86	0.84	38.07	5.80
A2A699-2	Isoform 2 of Protein FAM171A2 OS=Mus musculus GN=Fam171a2 - [F1712_MOUSE]	21.63	2	8	9	25	0.99	1.02	1.00	1.01	1.03	1.03	1.07	0.98	1.05	1.02	1.05	0.95	0.96	67.77	7.23
P22682	E3 ubiquitin-protein ligase CBL OS=Mus musculus GN=Cbl PE=1 SV=3 - [CBL_MOUSE]	16.43	1	9	10	18	1.01	0.99	1.11	1.07	1.11	1.07	1.07	0.98	1.00	1.31	1.15	1.05	1.06	100.50	6.67
Q9CQ43	Deoxyuridine triphosphatase OS=Mus musculus GN=Dut PE=2 SV=1 - [Q9CQ43_MOUSE]	46.30	2	5	5	8	0.92	1.00	0.98	0.82	0.89	1.12	1.13	0.98	1.05	1.12	1.12	0.96	1.06	17.37	6.04
Q9CZ04	COP9 signalosome complex subunit 7a OS=Mus musculus GN=Cops7a PE=1 SV=2 - [CSN7A_MOUSE]	52.73	5	10	10	24	1.03	0.95	0.91	1.09	0.98	0.96	0.98	0.98	0.94	0.99	0.99	0.98	0.98	30.21	7.87
Q91WC0	Histone-lysine N-methyltransferase setd3 OS=Mus musculus GN=Setd3 PE=1 SV=1 - [SETD3_MOUSE]	10.27	6	4	4	6	1.01	1.13	1.11	1.13	1.17	0.96	0.95	0.98	0.97	1.02	1.03	1.14	1.14	67.13	5.60
Q8BXV2	BRI3-binding protein OS=Mus musculus GN=Bri3bp PE=2 SV=1 - [BRI3B_MOUSE]	11.07	1	2	2	4	1.21	1.21	0.99	1.10	0.90	1.06	0.87	0.98	0.80	1.04	0.86	1.13	0.93	28.25	9.52

Q8BNN1	Spermatogenesis-associated protein 2-like protein OS=Mus musculus GN=Spata2l PE=2 SV=1 - [SPA2L_MOUSE]	12.21	1	3	3	8	1.04	1.30	1.22	1.00	0.99	1.07	1.03	0.98	0.94	1.19	1.15	1.26	1.24	46.74	5.20
P62320	Small nuclear ribonucleoprotein Sm D3 OS=Mus musculus GN=Snrpd3 PE=1 SV=1 - [SMD3_MOUSE]	31.75	1	3	3	11	1.00	1.12	1.12	0.98	0.93	0.89	0.89	0.98	0.95	0.99	0.92	0.89	0.95	13.91	10.32
P0CL69	Zinc finger protein 703 OS=Mus musculus GN=Znf703 PE=1 SV=1 - [ZN703_MOUSE]	2.19	1	1	1	2	0.96	0.69	0.72	0.85	0.88	1.13	1.17	0.98	1.01	0.88	0.91	0.85	0.88	58.69	8.79
Q7TN99-3	Isoform 3 of Cytoplasmic polyadenylation element-binding protein 3 OS=Mus musculus GN=Cpeb3 - [CPEB3_MOUSE]	27.85	12	7	12	30	1.00	1.02	1.04	1.00	0.97	0.99	0.99	0.98	0.92	1.03	1.04	1.06	1.08	75.64	7.23
O09012-2	Isoform 2 of Peroxisomal targeting signal 1 receptor OS=Mus musculus GN=Pex5 - [PEX5_MOUSE]	8.80	3	4	4	8	1.07	1.11	1.18	0.91	0.85	0.98	1.01	0.98	0.92	1.01	0.98	0.97	0.90	66.76	4.55
Q9CXV9	DCN1-like protein 5 OS=Mus musculus GN=Dcun1d5 PE=2 SV=1 - [DCNL5_MOUSE]	6.75	1	1	1	2	0.95	1.22	1.28	0.77	0.81	1.03	1.08	0.98	1.02	0.98	1.04	0.90	0.95	27.56	5.44
Q8BX10	Serine/threonine-protein phosphatase PGAM5, mitochondrial OS=Mus musculus GN=Pgam5 PE=2 SV=1 - [PGAM5_MOUSE]	27.08	3	6	6	17	0.98	0.95	1.10	1.03	1.12	0.98	1.04	0.98	0.92	0.94	0.92	0.91	0.98	31.97	9.04
P33175	Kinesin heavy chain isoform 5A OS=Mus musculus GN=Kif5a PE=1 SV=3 - [KIF5A_MOUSE]	16.07	1	7	13	37	0.88	0.98	0.96	0.95	1.09	1.02	1.15	0.98	0.96	0.97	1.01	0.90	0.99	116.95	5.94
Q80WM4	Hyaluronan and proteoglycan link protein 4 OS=Mus musculus GN=Hapln4 PE=2 SV=2 - [HPLN4_MOUSE]	18.25	1	5	6	10	0.88	1.06	1.19	0.96	1.08	0.98	1.08	0.98	1.12	0.99	1.11	1.08	1.03	42.78	8.85
P62960	Nuclease-sensitive element-binding protein 1 OS=Mus musculus GN=Ybx1 PE=1 SV=3 - [YBOX1_MOUSE]	33.54	5	6	8	37	1.11	1.18	1.02	0.98	0.93	1.01	0.92	0.98	0.93	1.02	0.97	1.00	0.91	35.71	9.88
Q8R105	Vacuolar protein sorting-associated protein 37C OS=Mus musculus GN=Vps37c PE=2 SV=1 - [VP37C_MOUSE]	28.98	2	8	8	17	0.85	0.85	0.98	0.82	0.94	0.84	0.95	0.98	1.10	0.83	1.00	0.81	0.94	38.43	5.31

P15105	Glutamine synthetase OS=Mus musculus GN=Glul PE=1 SV=6 - [GLNA_MOUSE]	65.68	3	22	22	326	0.96	0.98	1.01	1.00	1.04	0.96	1.00	0.98	1.02	0.95	0.99	1.00	1.05	42.09	7.08
Q9D2K6	Protein D930007J09Rik OS=Mus musculus GN=D930007J09Rik PE=2 SV=1 - [Q9D2K6_MOUSE]	5.34	1	1	1	1	1.13	1.07	0.95	1.05	0.93	1.02	0.90	0.98	0.86	0.99	0.88	1.05	0.94	13.61	12.13
Q640R3	Hepatocyte cell adhesion molecule OS=Mus musculus GN=Hepacam PE=1 SV=2 - [HECAM_MOUSE]	32.06	1	10	10	52	1.02	1.00	0.97	0.97	0.99	0.94	0.94	0.98	0.90	0.93	0.92	0.90	0.90	46.34	9.42
Q9DCM0	Persulfide dioxygenase ETHE1, mitochondrial OS=Mus musculus GN=Ethe1 PE=1 SV=2 - [ETHE1_MOUSE]	26.38	1	5	5	10	0.97	1.06	1.07	0.97	1.04	0.97	1.03	0.98	1.02	0.92	1.05	1.06	1.20	27.72	7.23
Q6RHR9-	Isoform 3 of Membrane-associated guanylate kinase, WW and PDZ domain-containing protein 1 OS=Mus musculus GN=Magi1 - [MAGI1_MOUSE]	29.02	3	5	26	70	0.97	0.84	0.99	0.84	0.90	1.10	1.06	0.98	1.00	0.96	1.10	0.85	0.94	134.32	5.78
Q14BI1	Protein Slc24a2 OS=Mus musculus GN=Slc24a2 PE=2 SV=1 - [Q14BI1_MOUSE]	15.47	1	3	7	27	0.91	1.22	1.32	1.09	1.20	0.92	1.02	0.98	1.08	0.95	1.02	0.89	1.06	78.93	6.20
Q5U405	Transmembrane protease serine 13 OS=Mus musculus GN=Tmprss13 PE=2 SV=2 - [TMPSD_MOUSE]	1.47	2	1	1	9	2.00	0.64	0.33	1.02	0.48	1.10	0.58	0.98	0.51	0.78	0.41	1.05	0.52	59.77	9.01
A2A839	Protein 4.1 OS=Mus musculus GN=Epb4.1 PE=2 SV=1 - [A2A839_MOUSE]	16.28	10	4	7	15	0.89	0.96	0.99	0.95	1.20	0.95	1.07	0.98	1.10	1.04	1.25	0.94	1.03	72.38	6.62
Q8R1F1	Niban-like protein 1 OS=Mus musculus GN=Fam129b PE=1 SV=2 - [NIBL1_MOUSE]	1.47	1	1	1	2	1.00	1.33	1.32	1.20	1.20	0.88	0.88	0.98	0.97	0.99	0.99	1.22	1.22	84.76	5.94
E9PXB0	Calcium-independent phospholipase A2-gamma OS=Mus musculus GN=Pnpla8 PE=2 SV=1 - [E9PXB0_MOUSE]	22.04	4	10	10	17	0.97	0.99	1.09	1.01	1.01	1.01	1.01	0.98	0.98	0.94	0.94	0.94	0.95	62.31	7.84
Q3UVX5	Metabotropic glutamate receptor 5 OS=Mus musculus GN=Grm5 PE=2 SV=2 - [GRM5_MOUSE]	21.86	5	22	22	52	1.08	1.16	1.08	1.15	1.07	0.99	0.91	0.98	0.91	1.02	0.96	1.07	1.00	131.78	7.84

Q8C863-2	Isoform 2 of E3 ubiquitin-protein ligase Itchy OS=Mus musculus GN=Itch - [ITCH_MOUSE]	3.43	2	2	2	3	0.94	1.06	1.12	1.06	1.12	0.91	0.96	0.98	1.03	0.94	1.00	1.08	1.15	86.65	6.34
Q05BC3-2	Isoform 2 of Echinoderm microtubule-associated protein-like 1 OS=Mus musculus GN=Eml1 - [EMAL1_MOUSE]	9.96	5	8	8	16	0.95	0.96	1.05	0.95	1.04	0.96	1.02	0.98	1.04	0.86	0.99	0.85	0.91	86.36	7.46
O88848	ADP-ribosylation factor-like protein 6 OS=Mus musculus GN=Arl6 PE=2 SV=1 - [ARL6_MOUSE]	11.83	3	2	2	2	1.05	0.82	0.77	1.09	1.04	1.05	0.99	0.98	0.93	0.86	0.82	0.74	0.70	20.95	8.25
D5MCW4	Protein CutA OS=Mus musculus GN=Cuta PE=2 SV=1 - [D5MCW4_MOUSE]	9.09	2	1	1	1	0.65	0.84	1.28	0.88	1.36	1.05	1.61	0.98	1.50	0.86	1.33	0.98	1.51	16.44	6.29
Q99J31	Oligophrenin-1 OS=Mus musculus GN=Ophn1 PE=2 SV=1 - [OPHN1_MOUSE]	6.61	3	3	3	12	1.01	1.02	1.00	1.00	1.02	0.98	0.91	0.98	1.05	1.03	0.91	1.04	0.94	91.93	7.96
Q3U5Q7	UMP-CMP kinase 2, mitochondrial OS=Mus musculus GN=Cmpk2 PE=2 SV=2 - [CMPK2_MOUSE]	19.46	1	7	7	10	1.01	1.09	1.12	1.03	1.07	0.97	0.93	0.98	0.95	1.02	0.97	1.13	1.14	50.00	7.27
Q61025-2	Isoform 2 of Intraflagellar transport protein 20 homolog OS=Mus musculus GN=Ift20 - [IFT20_MOUSE]	27.36	2	2	2	4	0.95	1.29	1.35	0.80	0.77	1.04	1.21	0.98	1.08	1.03	1.08	0.78	0.83	12.01	5.60
F7DBY2	Merlin (Fragment) OS=Mus musculus GN=Nf2 PE=2 SV=1 - [F7DBY2_MOUSE]	7.86	5	1	1	2	0.91	0.80	0.88	0.97	1.06	0.96	1.04	0.98	1.07	1.02	1.12	1.01	1.11	16.03	9.58
Q3UVL4	Vacuolar protein sorting-associated protein 51 homolog OS=Mus musculus GN=Vps51 PE=2 SV=2 - [VPS51_MOUSE]	21.61	4	9	9	21	0.90	1.17	1.32	1.20	1.36	0.90	1.02	0.98	1.05	1.10	1.21	1.07	1.20	86.13	6.24
Q8C6I2	Succinate dehydrogenase assembly factor 2, mitochondrial OS=Mus musculus GN=Sdhaf2 PE=2 SV=1 - [SDHF2_MOUSE]	20.12	3	3	3	8	1.00	0.62	0.61	0.86	0.88	1.02	1.03	0.98	0.99	0.65	0.87	0.81	0.79	19.42	6.35
H3BL92	Protein Evi5l OS=Mus musculus GN=Evi5l PE=2 SV=1 - [H3BL92_MOUSE]	9.85	5	6	6	11	0.95	1.08	1.16	1.08	1.15	0.99	1.02	0.98	1.04	1.05	1.06	1.00	1.05	92.28	5.27
Q07076	Annexin A7 OS=Mus musculus GN=Anxa7 PE=2 SV=2 - [ANXA7_MOUSE]	22.68	1	10	10	24	1.04	1.05	1.07	1.12	1.11	0.98	0.93	0.98	0.96	1.00	0.98	1.09	1.10	49.89	6.18

P39087	Glutamate receptor ionotropic, kainate 2 OS=Mus musculus GN=Grik2 PE=1 SV=4 - [GRIK2_MOUSE]	5.51	8	3	4	5	1.03	1.02	1.09	1.16	1.27	0.81	0.99	0.98	0.90	0.97	1.01	0.99	0.96	102.42	7.77
Q9JHI7	Exosome complex component RRP45 OS=Mus musculus GN=Exosc9 PE=2 SV=1 - [EXOS9_MOUSE]	3.20	1	1	1	2	0.99	1.21	1.22	0.93	0.95	1.02	1.03	0.98	0.99	0.90	0.92	0.95	0.96	48.91	5.11
O35593	26S proteasome non-ATPase regulatory subunit 14 OS=Mus musculus GN=Psmd14 PE=1 SV=2 - [PSDE_MOUSE]	11.29	1	2	2	4	0.91	0.90	0.87	0.77	1.09	1.22	1.18	0.98	0.95	1.03	1.10	1.02	0.99	34.55	6.52
D3Z118	Islet cell autoantigen 1 OS=Mus musculus GN=Ica1 PE=2 SV=1 - [D3Z118_MOUSE]	13.76	8	6	6	14	0.98	0.99	0.96	1.05	1.10	0.93	0.97	0.98	1.03	1.06	1.03	1.02	1.02	52.90	6.20
Q61923	Potassium voltage-gated channel subfamily A member 6 OS=Mus musculus GN=Kcna6 PE=1 SV=1 - [KCNA6_MOUSE]	17.20	1	5	6	24	1.38	1.08	1.00	1.15	0.89	0.88	0.68	0.98	0.85	1.00	0.82	1.04	0.94	58.64	5.03
Q8BIJ7	RUN and FYVE domain-containing protein 1 OS=Mus musculus GN=Rufy1 PE=1 SV=1 - [RUFY1_MOUSE]	5.48	2	3	4	11	0.99	1.11	1.12	1.02	1.03	1.00	1.00	0.98	1.01	1.01	1.04	1.01	1.04	80.33	5.68
Q9JJZ2	Tubulin alpha-8 chain OS=Mus musculus GN=Tuba8 PE=1 SV=1 - [TBA8_MOUSE]	45.21	1	4	22	2144	0.91	1.03	1.07	0.96	1.02	0.87	1.06	0.98	1.03	0.90	0.99	0.91	0.99	50.02	5.10
Q8K1S1	Leucine-rich repeat LGI family member 4 OS=Mus musculus GN=Lgi4 PE=1 SV=1 - [LGI4_MOUSE]	4.10	1	2	2	2	1.07	1.10	1.03	1.19	1.11	0.99	0.92	0.98	0.91	1.10	1.03	1.05	0.98	59.34	7.78
Q8VDU5	SNF-related serine/threonine-protein kinase OS=Mus musculus GN=Snrk PE=1 SV=1 - [SNRK_MOUSE]	6.55	2	4	4	4	0.98	1.14	1.10	1.06	1.01	1.14	1.18	0.98	1.00	1.10	1.12	1.03	1.21	81.86	7.49
Q8VEH5	EPM2A-interacting protein 1 OS=Mus musculus GN=Epm2aip1 PE=2 SV=1 - [EPMIP_MOUSE]	20.79	1	9	9	16	1.10	1.13	1.07	1.09	1.08	0.92	0.90	0.98	0.98	1.12	1.02	1.10	1.13	70.05	5.87
D3Z2K2	28S ribosomal protein S14, mitochondrial OS=Mus musculus GN=Mrps14 PE=2 SV=1 - [D3Z2K2_MOUSE]	12.30	2	1	1	2	0.89	0.97	1.08	0.94	1.06	1.18	1.32	0.98	1.10	0.92	1.03	0.79	0.89	14.27	10.86

Q8K2K6-3	Isoform 3 of Arf-GAP domain and FG repeat-containing protein 1 OS=Mus musculus GN=Agfg1 - [AGFG1_MOUSE]	23.77	6	10	12	52	0.97	0.83	0.83	0.95	1.00	0.96	0.98	0.98	1.02	0.90	0.93	0.86	0.87	55.03	8.47
H3BJY0	Calcium-transporting ATPase type 2C member 1 (Fragment) OS=Mus musculus GN=Atp2c1 PE=2 SV=1 - [H3BJY0_MOUSE]	24.68	12	4	4	13	1.00	1.05	1.06	1.14	1.19	0.91	0.94	0.98	1.01	1.01	1.03	1.06	1.03	25.36	5.49
Q8CFI7	DNA-directed RNA polymerase II subunit RPB2 OS=Mus musculus GN=Polr2b PE=2 SV=2 - [RPB2_MOUSE]	4.94	1	4	4	7	1.02	1.11	1.13	1.03	1.08	0.85	0.89	0.98	0.94	0.86	0.91	0.88	0.87	133.82	6.87
Q68FF6	ARF GTPase-activating protein GIT1 OS=Mus musculus GN=Git1 PE=1 SV=1 - [GIT1_MOUSE]	36.75	14	18	18	48	0.92	1.18	1.29	1.08	1.15	0.93	1.02	0.98	1.01	1.06	1.16	1.09	1.15	85.25	6.93
Q9D1K2	V-type proton ATPase subunit F OS=Mus musculus GN=Atp6v1f PE=1 SV=2 - [VATF_MOUSE]	82.35	2	9	9	108	0.97	0.84	0.91	0.88	0.92	0.91	0.96	0.98	1.02	0.85	0.91	0.82	0.87	13.36	5.82
P0C027	Diphosphoinositol polyphosphate phosphohydrolase 3-alpha OS=Mus musculus GN=Nudt10 PE=1 SV=1 - [NUD10_MOUSE]	39.02	1	2	5	14	0.90	1.05	1.09	0.93	0.87	1.03	1.06	0.98	1.08	1.00	1.03	1.04	1.10	18.58	5.69
A2AI08	Taperin OS=Mus musculus GN=Tprn PE=1 SV=1 - [TPRN_MOUSE]	2.40	1	1	1	2	1.30	1.38	1.06	1.09	0.84	1.34	1.03	0.98	0.75	1.58	1.22	1.35	1.04	80.04	7.09
H3BJR6	Sodium channel subunit beta-3 OS=Mus musculus GN=Scn3b PE=2 SV=1 - [H3BJR6_MOUSE]	11.43	4	2	2	5	1.10	0.98	0.85	1.13	0.92	1.04	0.94	0.98	0.84	1.06	0.89	1.14	0.97	20.35	4.75
A6H5Z3	Exocyst complex component 6B OS=Mus musculus GN=Exoc6b PE=2 SV=1 - [EXC6B_MOUSE]	6.79	4	4	4	7	0.96	0.98	1.00	1.14	1.19	0.93	0.88	0.98	1.02	1.07	1.02	1.03	1.00	94.07	6.39
Q8C4Q6	Axin interactor, dorsalization-associated protein OS=Mus musculus GN=Aida PE=1 SV=1 - [AIDA_MOUSE]	9.84	2	2	2	6	1.07	1.22	1.14	1.17	1.04	1.38	1.22	0.98	1.01	1.26	1.12	1.36	1.21	34.87	6.74
Q3YAB1	Kv channel-interacting protein 2 OS=Mus musculus GN=Kcnip2 PE=2 SV=1 - [Q3YAB1_MOUSE]	20.45	12	4	4	9	1.02	1.35	1.39	0.91	0.90	1.01	1.15	0.98	1.08	1.00	0.99	1.06	1.21	25.63	4.61
P84084	ADP-ribosylation factor 5 OS=Mus musculus GN=Arf5 PE=2 SV=2 - [ARF5_MOUSE]	60.56	3	3	9	51	1.05	1.04	1.02	1.03	1.00	1.00	0.98	0.98	0.94	0.96	0.91	0.97	0.92	20.52	6.79

Q80Z38	SH3 and multiple ankyrin repeat domains protein 2 OS=Mus musculus GN=Shank2 PE=1 SV=2 - [SHAN2_MOUSE]	44.72	3	4	46	259	1.03	1.00	0.89	0.93	0.94	1.05	0.92	0.98	1.03	0.92	0.93	0.97	0.89	158.87	7.18
Q63844	Mitogen-activated protein kinase 3 OS=Mus musculus GN=Mapk3 PE=1 SV=5 - [MK03_MOUSE]	38.42	19	9	14	37	1.07	1.08	1.03	1.00	0.95	0.94	0.96	0.98	0.89	1.03	0.95	1.11	1.11	43.04	6.61
P97492	Regulator of G-protein signaling 14 OS=Mus musculus GN=Rgs14 PE=1 SV=2 - [RGS14_MOUSE]	26.14	1	10	10	19	1.01	1.12	1.09	1.07	0.97	1.03	1.02	0.98	0.89	1.09	1.02	1.14	1.05	59.81	7.43
A2AKG8-	Isoform 2 of Focadhesin OS=Mus musculus GN=Focad - [FOCAD_MOUSE]	2.69	3	1	1	1	1.12	1.24	1.10	1.03	0.92	1.01	0.89	0.98	0.87	0.99	0.88	1.17	1.04	37.62	5.97
Q921F4	Heterogeneous nuclear ribonucleoprotein L-like OS=Mus musculus GN=Hnrnpll PE=1 SV=3 - [HNRLL_MOUSE]	34.01	2	12	12	22	1.02	0.92	0.90	1.10	0.99	0.90	0.86	0.98	1.00	0.96	0.98	0.97	0.97	64.08	5.85
O08677-2	Isoform LMW of Kininogen-1 OS=Mus musculus GN=Kng1 - [KNG1_MOUSE]	9.72	7	4	4	8	0.89	0.78	0.80	0.47	0.53	1.02	1.14	0.98	1.02	1.11	1.17	0.64	0.66	47.87	6.09
P28828	Receptor-type tyrosine-protein phosphatase mu OS=Mus musculus GN=Ptprm PE=2 SV=2 - [PTPRM_MOUSE]	4.13	1	2	3	4	0.77	1.10	1.44	0.86	1.12	0.82	1.06	0.98	1.27	0.91	1.19	0.81	1.06	163.55	6.68
G3X8Q6	Kinesin family member C2 OS=Mus musculus GN=Kifc2 PE=3 SV=1 - [G3X8Q6_MOUSE]	2.02	2	1	1	4	1.04	1.28	1.23	1.05	1.01	1.11	1.06	0.98	0.94	0.98	0.95	1.21	1.17	85.55	6.16
Q9D4H8-2	Isoform 2 of Cullin-2 OS=Mus musculus GN=Cul2 - [CUL2_MOUSE]	3.68	3	2	2	3	1.06	1.24	1.17	1.26	1.19	1.01	0.95	0.98	0.92	1.19	1.13	1.12	1.06	82.22	6.80
Q9QXS1-(	Isoform PLEC-0,1C of Plectin OS=Mus musculus GN=Plec - [PLEC_MOUSE]	43.15	30	162	164	392	0.93	1.06	1.12	1.11	1.17	0.97	1.04	0.98	1.06	0.97	1.04	0.98	1.05	519.47	5.76
Q8BK72	28S ribosomal protein S27, mitochondrial OS=Mus musculus GN=Mrps27 PE=2 SV=2 - [RT27_MOUSE]	13.01	1	4	4	7	0.97	0.88	0.88	1.14	1.14	1.00	1.04	0.98	0.98	1.00	1.00	0.96	0.96	47.75	5.50
Q78PY7	Staphylococcal nuclease domain-containing protein 1 OS=Mus musculus GN=Snd1 PE=1 SV=1 - [SND1_MOUSE]	31.21	3	22	22	47	1.00	1.08	1.08	1.03	1.00	0.94	0.96	0.98	0.94	0.99	1.00	1.09	1.06	102.02	7.43

Q9CQU3	Protein RER1 OS=Mus musculus GN=Rer1 PE=1 SV=1 - [RER1_MOUSE]	5.10	1	1	1	1	0.91	1.27	1.40	1.11	1.22	0.88	0.96	0.98	1.08	0.93	1.03	0.86	0.95	22.97	9.51
P62301	40S ribosomal protein S13 OS=Mus musculus GN=Rps13 PE=1 SV=2 - [RS13_MOUSE]	28.48	1	4	4	16	1.00	0.88	0.84	1.05	1.04	1.09	1.06	0.98	1.00	1.01	0.98	1.03	0.99	17.21	10.54
E9QMJ1	Arf-GAP with SH3 domain, ANK repeat and PH domain-containing protein 1 OS=Mus musculus GN=Asap1 PE=2 SV=1 - [E9QMJ1_MOUSE]	22.21	13	17	18	47	0.96	0.96	1.02	0.99	1.06	0.97	1.01	0.98	1.07	0.89	0.98	0.93	1.00	123.46	7.06
P12023-2	Isoform APP695 of Amyloid beta A4 protein OS=Mus musculus GN=App - [A4_MOUSE]	36.55	1	1	20	201	0.90	0.78	0.87	0.90	1.01	1.03	1.14	0.98	1.09	0.88	0.98	0.76	0.84	78.39	4.83
Q61414	Keratin, type I cytoskeletal 15 OS=Mus musculus GN=Krt15 PE=1 SV=2 - [K1C15_MOUSE]	7.08	17	1	4	5	1.59	0.80	0.50	2.48	1.55	1.33	0.83	0.98	0.61	1.19	0.75	1.80	1.13	49.11	4.86
Q8K5B2	Multiple coagulation factor deficiency protein 2 homolog OS=Mus musculus GN=Mcfd2 PE=2 SV=1 - [MCFD2_MOUSE]	52.41	3	4	4	12	1.03	0.74	0.74	0.91	0.87	1.04	0.98	0.98	0.88	1.04	0.88	0.94	0.92	16.16	4.72
O88502	High affinity cAMP-specific and IBMX-insensitive 3',5'-cyclic phosphodiesterase 8A OS=Mus musculus GN=Pde8a PE=1 SV=1 - [PDE8A_MOUSE]	5.71	1	2	3	6	1.38	1.78	1.29	1.13	0.82	1.08	0.78	0.98	0.71	0.99	0.72	1.17	0.85	93.11	5.85
F8WIK5	Pleckstrin homology domain-containing family A member 2 (Fragment) OS=Mus musculus GN=Plekha2 PE=2 SV=1 - [F8WIK5_MOUSE]	6.53	2	1	1	1	0.68	1.48	2.18	0.86	1.27	0.79	1.16	0.98	1.45	1.15	1.71	0.78	1.15	37.61	8.95
Q8CF96	Synaptotagmin VIIbeta OS=Mus musculus GN=Syt7 PE=2 SV=1 - [Q8CF96_MOUSE]	33.33	2	2	13	36	1.27	0.94	0.74	0.89	0.84	1.22	0.93	0.98	0.74	1.22	0.83	1.23	0.81	50.64	9.11
Q9R1T2	SUMO-activating enzyme subunit 1 OS=Mus musculus GN=Sae1 PE=2 SV=1 - [SAE1_MOUSE]	40.86	2	11	11	20	1.02	0.97	0.95	1.14	1.03	1.04	1.00	0.98	0.97	0.98	0.95	1.04	0.99	38.60	5.36
A2AKX2	Netrin-G2 OS=Mus musculus GN=Ntng2 PE=2 SV=1 - [A2AKX2_MOUSE]	13.95	5	4	4	7	0.83	0.89	1.15	1.03	1.27	0.90	1.25	0.98	1.15	0.94	0.96	0.94	1.15	49.05	7.88

Q99K95	Protein RTF2 homolog OS=Mus musculus GN=Rtfdc1 PE=2 SV=1 - [RTF2_MOUSE]	8.79	1	3	3	11	1.02	0.76	0.75	1.01	0.95	1.02	1.04	0.98	0.98	0.90	0.85	0.80	0.82	33.91	8.81
P60335	Poly(rC)-binding protein 1 OS=Mus musculus GN=Pcbp1 PE=1 SV=1 - [PCBP1_MOUSE]	68.26	2	11	16	53	0.99	1.01	1.00	1.03	1.02	1.02	0.98	0.98	0.95	0.97	0.99	1.08	1.06	37.47	7.09
Q7TQI3	Ubiquitin thioesterase OTUB1 OS=Mus musculus GN=Otub1 PE=1 SV=2 - [OTUB1_MOUSE]	48.71	3	9	9	53	1.04	1.07	1.05	1.02	1.02	1.00	0.99	0.98	0.93	1.00	0.96	1.06	1.05	31.25	4.94
Q9DCB4-	Isoform 2 of cAMP-regulated phosphoprotein 21 OS=Mus musculus GN=Arpp21 - [ARP21_MOUSE]	50.00	4	1	4	38	1.16	1.30	1.12	0.88	0.76	1.07	0.92	0.98	0.85	1.00	0.87	1.27	1.10	9.53	4.92
Q5U4F6	WD repeat-containing protein 34 OS=Mus musculus GN=Wdr34 PE=2 SV=2 - [WDR34_MOUSE]	9.35	2	3	3	5	1.06	1.33	1.26	1.09	1.02	0.87	0.82	0.98	0.92	1.05	0.99	1.15	1.08	57.94	6.52
Q61990-3	Isoform 3 of Poly(rC)-binding protein 2 OS=Mus musculus GN=Pcbp2 - [PCBP2_MOUSE]	50.43	4	7	13	48	1.01	0.94	0.90	0.92	1.00	0.96	1.01	0.98	0.97	0.98	0.98	1.01	1.03	36.78	7.06
F6RJV6	LanC-like protein 2 (Fragment) OS=Mus musculus GN=Lancl2 PE=2 SV=1 - [F6RJV6_MOUSE]	41.50	2	14	14	49	1.05	1.03	1.01	1.05	1.04	0.97	0.93	0.98	0.88	0.99	0.92	1.13	0.97	49.71	7.14
Q9CZJ2	Heat shock 70 kDa protein 12B OS=Mus musculus GN=Hspa12b PE=1 SV=1 - [HS12B_MOUSE]	11.53	1	4	8	21	0.95	1.22	1.26	1.13	1.19	0.86	0.90	0.98	0.96	0.93	0.98	1.10	1.16	76.07	8.47
Q8QZY1	Eukaryotic translation initiation factor 3 subunit L OS=Mus musculus GN=Eif3l PE=1 SV=1 - [EIF3L_MOUSE]	23.58	1	10	10	21	1.08	1.11	1.03	1.15	1.03	1.01	0.88	0.98	0.90	0.97	0.85	1.04	1.00	66.57	6.44
Q8K0V4	CCR4-NOT transcription complex subunit 3 OS=Mus musculus GN=Cnot3 PE=1 SV=1 - [CNOT3_MOUSE]	8.52	2	5	5	14	0.91	0.91	1.03	0.91	0.98	1.01	1.16	0.98	1.10	0.97	1.09	0.98	1.02	81.90	6.20
Q8C8R3-7	Isoform 7 of Ankyrin-2 OS=Mus musculus GN=Ank2 - [ANK2_MOUSE]	48.68	2	2	16	118	1.31	0.94	0.70	1.05	0.81	1.12	0.83	0.98	0.71	0.92	0.66	1.08	0.79	49.51	9.48
E9Q9E1	Eukaryotic translation initiation factor 4 gamma 1 OS=Mus musculus GN=Eif4g1 PE=2 SV=1 - [E9Q9E1_MOUSE]	16.70	11	16	19	51	1.02	0.85	0.92	1.01	1.02	1.02	1.05	0.98	0.92	1.02	1.03	0.97	1.08	175.22	5.38

Q9D1C3	Protein preY, mitochondrial OS=Mus musculus GN=Pyurf PE=3 SV=1 - [PREY_MOUSE]	18.75	1	1	1	4	1.05	0.89	0.84	0.99	0.94	0.94	0.90	0.98	0.94	0.85	0.81	0.93	0.89	12.50	8.34
Q91WE1	Sorting nexin-15 OS=Mus musculus GN=Snx15 PE=2 SV=1 - [SNX15_MOUSE]	35.61	3	6	6	12	0.94	0.71	0.91	0.80	0.90	1.12	1.14	0.98	1.00	1.07	1.05	0.91	0.98	37.72	5.24
Q9CYA6	Zinc finger CCHC domain-containing protein 8 OS=Mus musculus GN=Zcchc8 PE=2 SV=3 - [ZCHC8_MOUSE]	22.71	1	8	8	10	0.85	0.88	1.03	0.87	1.04	1.02	1.30	0.98	1.21	1.00	1.17	1.03	1.23	77.98	4.73
Q9R118	Serine protease HTRA1 OS=Mus musculus GN=Htra1 PE=1 SV=2 - [HTRA1_MOUSE]	10.42	1	4	4	8	0.97	1.31	1.29	0.85	0.82	0.96	0.95	0.98	0.96	0.85	0.90	0.88	0.84	51.18	7.65
Q61771	Kinesin-like protein KIF3B OS=Mus musculus GN=Kif3b PE=1 SV=1 - [KIF3B_MOUSE]	21.69	1	9	13	26	1.00	0.99	0.94	0.93	0.94	1.05	1.04	0.98	1.02	0.86	1.00	1.01	1.00	85.24	7.69
E9PXG9	Vacuolar protein sorting-associated protein 53 homolog OS=Mus musculus GN=Vps53 PE=2 SV=1 - [E9PXG9_MOUSE]	5.73	6	3	3	4	0.87	1.07	1.01	1.07	1.23	0.94	1.14	0.98	0.93	1.01	0.91	1.12	0.93	91.06	6.61
Q3UFT3	GRB2-associated and regulator of MAPK protein OS=Mus musculus GN=Garem PE=1 SV=2 - [GAREM_MOUSE]	4.91	1	3	3	5	0.94	1.53	1.54	1.13	1.13	1.11	1.20	0.98	1.04	0.95	1.02	0.96	1.15	97.19	6.60
P10605	Cathepsin B OS=Mus musculus GN=Ctsb PE=1 SV=2 - [CATB_MOUSE]	37.46	1	10	10	47	0.98	1.00	0.99	0.96	1.01	1.01	1.04	0.98	0.99	0.90	0.97	0.93	1.05	37.26	5.91
Q9QXB9	Developmentally-regulated GTP-binding protein 2 OS=Mus musculus GN=Drg2 PE=1 SV=1 - [DRG2_MOUSE]	18.68	1	5	6	9	0.95	0.80	1.06	0.98	1.11	1.00	1.08	0.98	1.03	0.83	0.89	1.14	1.08	40.69	8.88
Q9CPW7	Zinc finger matrin-type protein 2 OS=Mus musculus GN=Zmat2 PE=2 SV=1 - [ZMAT2_MOUSE]	37.19	1	5	5	12	0.98	0.94	0.88	1.00	1.03	1.17	1.02	0.98	1.10	1.01	0.94	1.01	0.88	23.60	9.01
Q61271	Activin receptor type-1B OS=Mus musculus GN=Acvr1b PE=1 SV=1 - [ACV1B_MOUSE]	5.94	1	2	2	4	0.92	1.08	1.14	0.98	1.08	0.94	1.07	0.98	1.02	0.90	1.03	1.11	1.07	56.66	7.17
Q99M71	Mammalian ependymin-related protein 1 OS=Mus musculus GN=Epdr1 PE=2 SV=1 - [EPDR1_MOUSE]	34.38	2	7	7	36	1.07	0.89	0.82	0.88	0.82	0.95	0.91	0.98	0.91	0.92	0.85	0.92	0.84	25.47	7.58

Q64669	NAD(P)H dehydrogenase [quinone] 1 OS=Mus musculus GN=Nqo1 PE=1 SV=3 - [NQO1_MOUSE]	6.57	1	2	2	2	1.00	0.96	0.96	1.03	1.03	1.07	1.06	0.98	0.98	1.00	1.00	0.98	0.98	30.94	8.72
Q8JZM7	Parafibromin OS=Mus musculus GN=Cdc73 PE=2 SV=1 - [CDC73_MOUSE]	2.82	1	1	2	8	0.88	1.24	1.33	0.96	1.05	0.88	1.01	0.98	1.11	0.98	1.06	0.94	0.97	60.54	9.61
Q9JME9	V-set and transmembrane domain-containing protein 2B OS=Mus musculus GN=Vstm2b PE=2 SV=1 - [VTM2B_MOUSE]	4.21	1	1	1	1	1.18	1.19	1.01	0.89	0.76	1.27	1.07	0.98	0.83	0.96	0.82	1.05	0.89	30.14	7.15
J3QQ40	Torsin-1A-interacting protein 1 OS=Mus musculus GN=Tor1aip1 PE=4 SV=1 - [J3QQ40_MOUSE]	8.93	4	2	2	4	0.94	1.08	1.15	1.07	1.14	1.18	1.25	0.98	1.04	1.14	1.22	1.16	1.23	32.76	7.47
E9Q066	La-related protein 4 OS=Mus musculus GN=Larp4 PE=2 SV=1 - [E9Q066_MOUSE]	5.15	3	3	3	4	1.04	1.12	1.07	0.90	0.87	0.97	0.93	0.98	0.83	0.88	0.97	1.02	1.03	79.55	6.51
Q9DC77	Small muscular protein OS=Mus musculus GN=Smpx PE=2 SV=2 - [SMPX_MOUSE]	45.88	1	2	2	6	0.88	0.69	0.86	0.92	1.00	1.24	1.60	0.98	1.12	0.92	1.09	0.80	0.95	9.25	9.70
Q9D8S4	Oligoribonuclease, mitochondrial OS=Mus musculus GN=Rexo2 PE=1 SV=2 - [ORN_MOUSE]	28.27	1	7	7	19	0.93	0.73	0.78	0.76	0.90	1.04	1.13	0.98	1.05	0.96	1.08	0.85	0.94	26.72	7.15
P53798	Squalene synthase OS=Mus musculus GN=Fdft1 PE=2 SV=2 - [FDFT_MOUSE]	2.40	1	1	1	7	0.95	1.24	1.43	1.05	1.13	1.02	1.07	0.98	1.06	0.97	1.04	1.02	1.03	48.12	6.32
Q78T81	Protein FAM102A OS=Mus musculus GN=Fam102a PE=1 SV=1 - [F102A_MOUSE]	2.30	1	1	1	2	1.04	1.07	1.02	1.01	0.97	1.13	1.08	0.98	0.94	1.04	1.00	0.87	0.83	42.80	8.56
A2AN84	55 kDa erythrocyte membrane protein OS=Mus musculus GN=Mpp1 PE=2 SV=1 - [A2AN84_MOUSE]	25.22	6	10	10	13	0.93	0.95	1.06	0.98	1.03	1.01	1.05	0.98	1.04	1.06	1.14	0.96	1.00	50.50	9.19
Q9JJV4	Voltage-dependent calcium channel gamma-4 subunit OS=Mus musculus GN=Cacng4 PE=1 SV=1 - [CCG4_MOUSE]	8.26	1	1	1	2	1.21	5.02	4.14	1.06	0.87	0.84	0.69	0.98	0.81	0.99	0.82	1.37	1.13	36.51	9.11
P53811	Phosphatidylinositol transfer protein beta isoform OS=Mus musculus GN=Pitpnb PE=1 SV=2 - [PIPNB_MOUSE]	32.47	1	7	9	18	1.06	1.15	1.07	1.05	0.97	1.00	0.95	0.98	0.93	0.98	0.96	1.17	1.15	31.47	6.95

Q2YDW2	Isoform 2 of Protein misato homolog 1 OS=Mus musculus GN=Msto1 - [MSTO1_MOUSE]	3.15	4	1	1	2	0.97	0.96	0.99	1.11	1.14	0.99	1.01	0.98	1.01	1.08	1.11	1.17	1.21	49.26	6.07
P28658	Ataxin-10 OS=Mus musculus GN=Atxn10 PE=1 SV=2 - [ATX10_MOUSE]	17.89	1	7	7	16	1.24	0.92	0.81	1.13	0.90	0.99	0.83	0.98	0.84	1.03	0.91	1.06	0.94	53.67	5.25
Q8C3F2-3	Isoform 3 of Constitutive coactivator of PPAR-gamma-like protein 2 OS=Mus musculus GN=Fam120c - [F120C_MOUSE]	2.91	2	2	2	3	0.94	0.91	0.97	1.05	1.11	1.08	1.14	0.98	1.04	1.01	1.08	0.89	0.95	109.51	8.59
Q7TNL9	Coiled-coil-helix-coiled-coil-helix domain containing 10 OS=Mus musculus GN=Chchd10 PE=2 SV=1 - [Q7TNL9_MOUSE]	16.67	1	1	1	5	0.93	0.81	0.62	1.03	0.98	0.79	0.89	0.98	0.86	1.09	1.08	0.89	0.89	13.80	8.22
Q9R0N9	Synaptotagmin-9 OS=Mus musculus GN=Syt9 PE=1 SV=2 - [SYT9_MOUSE]	3.67	1	1	1	2	0.90	1.45	1.61	0.86	0.96	1.02	1.13	0.98	1.09	1.03	1.15	1.20	1.34	56.23	7.24
Q6P5D8	Structural maintenance of chromosomes flexible hinge domain-containing protein 1 OS=Mus musculus GN=Smchd1 PE=1 SV=2 - [SMHD1_MOUSE]	1.94	1	3	3	6	0.95	1.29	1.36	1.10	1.11	1.04	1.00	0.98	1.06	0.99	1.04	1.14	1.18	225.51	7.24
J3QN27	Calcium-activated potassium channel subunit alpha-1 OS=Mus musculus GN=Kcnma1 PE=4 SV=1 - [J3QN27_MOUSE]	9.89	14	8	8	21	1.04	1.14	1.13	1.13	1.03	0.98	1.00	0.99	0.98	0.98	0.95	0.95	0.92	127.14	6.62
Q80VP5-2	Isoform 2 of Probable peptide chain release factor C12orf65 homolog, mitochondrial OS=Mus musculus - [CL065_MOUSE]	14.84	2	1	1	1	0.88	1.24	1.40	0.77	0.88	1.12	1.26	0.99	1.11	1.12	1.27	1.02	1.16	13.66	9.03
B7FAU9	Filamin, alpha OS=Mus musculus GN=Flna PE=4 SV=1 - [B7FAU9_MOUSE]	19.93	8	31	34	68	0.97	1.07	1.04	0.97	0.97	0.84	0.85	0.99	0.96	1.04	1.09	0.89	0.91	280.33	6.01
Q8VHC3	Selenoprotein M OS=Mus musculus GN=Selm PE=1 SV=3 - [SELM_MOUSE]	54.48	1	5	5	13	0.94	0.90	0.92	0.91	0.86	1.04	1.04	0.99	1.05	0.95	1.02	0.92	0.97	16.37	5.54
Q8BK64	Activator of 90 kDa heat shock protein ATPase homolog 1 OS=Mus musculus GN=Ahsa1 PE=2 SV=2 - [AHSA1_MOUSE]	27.81	1	7	7	21	1.16	1.36	1.20	1.15	1.03	0.96	0.90	0.99	0.90	0.98	0.89	1.13	0.92	38.09	5.53

Q8CGP0	Histone H2B type 3-B OS=Mus musculus GN=Hist3h2bb PE=1 SV=3 - [H2B3B_MOUSE]	47.62	3	3	7	101	1.03	0.89	0.93	0.95	1.02	1.22	1.19	0.99	1.04	1.15	1.12	1.22	1.17	13.90	10.32
P47968	Ribose-5-phosphate isomerase OS=Mus musculus GN=Rpia PE=2 SV=2 - [RPIA_MOUSE]	9.57	1	2	2	4	0.95	0.98	1.03	0.93	0.98	0.98	1.03	0.99	1.04	0.95	1.00	0.99	1.05	32.43	8.02
E9PUC5	PH and SEC7 domain-containing protein 3 OS=Mus musculus GN=Psd3 PE=2 SV=1 - [E9PUC5_MOUSE]	55.59	2	1	21	101	1.02	0.73	0.72	0.98	0.96	1.00	0.98	0.99	0.96	0.87	0.85	0.87	0.86	42.27	9.60
B1AXV0	DOMON domain-containing protein FRRS1L OS=Mus musculus GN=Frrs1l PE=1 SV=1 - [FRS1L_MOUSE]	26.62	2	7	7	16	1.03	1.05	0.97	1.08	1.04	0.99	0.90	0.99	0.87	0.90	0.86	0.99	0.94	32.49	5.43
J3KMQ6	Protein 5730455P16Rik OS=Mus musculus GN=5730455P16Rik PE=4 SV=1 - [J3KMQ6_MOUSE]	5.09	2	1	1	2	0.95	0.95	1.00	0.99	1.04	0.99	1.04	0.99	1.04	1.00	1.06	1.10	1.17	44.38	5.05
E9QK48	Echinoderm microtubule-associated protein-like 2 OS=Mus musculus GN=Eml2 PE=2 SV=1 - [E9QK48_MOUSE]	10.23	5	7	7	11	0.89	0.99	1.23	0.94	1.03	0.82	0.93	0.99	0.96	1.04	1.05	0.84	0.98	90.74	6.98
P26638	Serine--tRNA ligase, cytoplasmic OS=Mus musculus GN=Sars PE=2 SV=3 - [SYSC_MOUSE]	47.66	4	18	18	41	1.02	0.98	1.02	1.12	1.12	1.01	0.99	0.99	0.94	0.97	0.90	1.01	0.98	58.35	6.30
P54818	Galactocerebrosidase OS=Mus musculus GN=Galc PE=1 SV=2 - [GALC_MOUSE]	2.34	1	1	1	2	0.98	1.13	1.15	1.13	1.14	0.97	0.99	0.99	1.00	1.10	1.12	1.08	1.10	77.21	6.74
E9QP09	Cullin-9 OS=Mus musculus GN=Cul9 PE=3 SV=1 - [E9QP09_MOUSE]	0.44	1	1	1	2	0.93	1.11	1.18	1.07	1.15	0.95	1.01	0.99	1.05	0.98	1.05	1.05	1.13	281.07	5.59
Q3V492	F-box only protein 22 OS=Mus musculus GN=Fbxo22 PE=2 SV=1 - [Q3V492_MOUSE]	5.69	2	2	2	2	1.02	1.41	1.38	1.11	1.08	0.91	0.88	0.99	0.96	0.91	0.89	1.03	1.01	32.99	6.84
O35226-2	Isoform Rpn10B of 26S proteasome non-ATPase regulatory subunit 4 OS=Mus musculus GN=Psmd4 - [PSMD4_MOUSE]	29.02	4	1	10	38	0.84	1.28	1.52	0.83	0.99	1.08	1.28	0.99	1.17	0.92	1.09	0.89	1.05	41.02	4.79

B2RXW8	Ppfia1 protein OS=Mus musculus GN=Ppfia1 PE=2 SV=1 - [B2RXW8_MOUSE]	8.86	1	2	10	28	0.90	0.85	0.95	0.94	1.05	0.96	1.06	0.99	1.09	0.99	1.10	0.99	1.10	140.05	6.14
P22005	Proenkephalin-A OS=Mus musculus GN=Penk PE=2 SV=2 - [PENK_MOUSE]	37.31	2	7	7	27	1.46	0.94	0.60	1.04	0.68	1.10	0.74	0.99	0.69	1.48	1.05	1.90	1.23	30.98	5.68
Q9QZ23	NFU1 iron-sulfur cluster scaffold homolog, mitochondrial OS=Mus musculus GN=Nfu1 PE=1 SV=2 - [NFU1_MOUSE]	29.41	1	3	6	36	0.99	0.92	0.89	0.90	0.92	1.01	1.01	0.99	1.05	0.96	1.02	0.91	0.89	28.55	5.03
Q61743	ATP-sensitive inward rectifier potassium channel 11 OS=Mus musculus GN=Kcnj11 PE=2 SV=1 - [IRK11_MOUSE]	2.82	1	1	1	2	0.94	1.00	1.06	1.05	1.12	0.86	0.91	0.99	1.05	1.01	1.07	0.81	0.86	43.53	8.19
Q8K366	Exosc10 protein OS=Mus musculus GN=Exosc10 PE=2 SV=1 - [Q8K366_MOUSE]	2.44	3	2	2	2	0.89	0.85	0.96	0.99	1.12	0.93	1.04	0.99	0.98	0.99	1.11	0.96	1.08	98.05	8.29
Q3UV70	[Pyruvate dehydrogenase [acetyl-transferring]]-phosphatase 1, mitochondrial OS=Mus musculus GN=Pdp1 PE=2 SV=1 - [PDP1_MOUSE]	12.45	4	5	5	8	0.97	1.05	1.03	0.97	0.99	0.91	0.99	0.99	0.94	0.94	0.95	1.17	1.15	61.14	6.67
O88712-2	Isoform 2 of C-terminal-binding protein 1 OS=Mus musculus GN=Ctbp1 - [CTBP1_MOUSE]	32.79	8	10	10	36	0.99	1.06	1.02	1.10	1.07	1.04	1.05	0.99	1.00	0.99	1.02	1.15	1.10	46.58	6.65
P19253	60S ribosomal protein L13a OS=Mus musculus GN=Rpl13a PE=1 SV=4 - [RL13A_MOUSE]	18.23	3	4	4	12	0.97	0.95	0.95	1.05	1.04	1.06	1.00	0.99	0.99	1.03	1.07	1.06	1.01	23.45	11.02
E9QMX4	MAP kinase-activated protein kinase 2 OS=Mus musculus GN=Rps6kc1 PE=2 SV=1 - [E9QMX4_MOUSE]	13.64	5	9	9	15	0.95	0.94	1.05	0.96	1.04	1.00	1.05	0.99	1.03	1.04	1.13	1.01	1.03	115.70	4.89
Q61738-4	Isoform Alpha-7X2A of Integrin alpha-7 OS=Mus musculus GN=Itga7 - [ITA7_MOUSE]	3.49	7	2	2	4	0.95	1.12	1.17	0.98	1.03	0.91	0.96	0.99	1.03	0.95	1.00	1.06	1.12	122.07	6.00
P97772	Metabotropic glutamate receptor 1 OS=Mus musculus GN=Grm1 PE=2 SV=2 - [GRM1_MOUSE]	5.17	2	5	5	7	0.99	1.15	1.10	1.10	0.95	0.95	0.88	0.99	0.99	0.86	0.93	0.95	0.97	133.13	6.86
Q6P2B1	Transportin-3 OS=Mus musculus GN=Tnpo3 PE=1 SV=1 - [TNPO3_MOUSE]	4.12	3	3	3	5	1.08	1.04	0.82	1.07	0.96	1.00	0.84	0.99	0.77	1.02	0.80	1.04	0.98	104.10	5.57

Q6NZD2	Sorting nexin 1 OS=Mus musculus GN=Snx1 PE=2 SV=1 - [Q6NZD2_MOUSE]	35.51	3	14	16	48	0.90	1.00	1.06	0.88	0.94	0.94	1.01	0.99	1.04	0.91	1.00	0.85	0.96	58.84	5.26
F6TCF9	BAG family molecular chaperone regulator 1 OS=Mus musculus GN=Bag1 PE=2 SV=1 - [F6TCF9_MOUSE]	31.55	3	9	9	13	1.08	0.70	0.72	0.83	0.81	1.06	1.02	0.99	0.98	0.89	0.85	0.84	0.82	39.67	8.46
P53702	Cytochrome c-type heme lyase OS=Mus musculus GN=Hccs PE=2 SV=2 - [CCHL_MOUSE]	17.28	2	4	4	6	0.95	1.05	0.97	1.04	1.05	1.05	1.11	0.99	0.95	1.04	1.07	1.04	1.08	30.96	7.12
Q8C5W0-	Isoform 2 of Calmin OS=Mus musculus GN=Clmn - [CLMN_MOUSE]	8.63	4	4	4	9	1.09	0.92	0.93	1.12	1.06	1.02	0.94	0.99	1.02	1.04	1.04	0.96	0.98	102.50	4.92
E0CXZ5	Deoxynucleoside triphosphate triphosphohydrolase SAMHD1 OS=Mus musculus GN=Samhd1 PE=2 SV=1 - [E0CXZ5_MOUSE]	9.33	6	1	1	2	1.06	1.03	0.97	1.03	0.96	0.95	0.89	0.99	0.93	0.98	0.93	1.08	1.02	16.62	4.74
Q9D394	Protein RUFY3 OS=Mus musculus GN=Rufy3 PE=1 SV=1 - [RUFY3_MOUSE]	38.59	3	2	16	64	1.13	1.04	0.95	1.13	0.92	1.03	0.91	0.99	0.89	1.12	0.99	1.14	1.05	52.97	5.49
F8WH29	Fibrocystin-L OS=Mus musculus GN=Pkhd1l1 PE=2 SV=1 - [F8WH29_MOUSE]	0.59	3	1	1	2	0.92	0.99	1.07	1.12	1.21	1.09	1.18	0.99	1.07	0.87	0.94	1.05	1.14	464.21	6.42
Q05CL8	La-related protein 7 OS=Mus musculus GN=Larp7 PE=1 SV=2 - [LARP7_MOUSE]	8.60	3	4	4	7	0.87	1.05	1.18	0.97	1.13	1.04	1.32	0.99	1.19	0.94	1.14	1.03	1.18	64.76	9.54
Q14B80	Potassium voltage-gated channel subfamily C member 2 OS=Mus musculus GN=Kcnc2 PE=1 SV=1 - [KCNC2_MOUSE]	12.31	2	4	5	8	0.92	1.03	1.10	1.05	1.11	0.89	0.94	0.99	1.05	0.90	0.96	0.89	0.97	70.46	7.69
Q8BJZ0	Zinc finger MYND domain-containing protein 11 OS=Mus musculus GN=Zmynd11 PE=2 SV=1 - [Q8BJZ0_MOUSE]	3.10	10	2	2	2	1.02	1.35	1.32	1.03	1.01	0.92	0.90	0.99	0.97	1.01	0.99	1.27	1.25	64.23	9.03
P41233	ATP-binding cassette sub-family A member 1 OS=Mus musculus GN=Abca1 PE=1 SV=4 - [ABCA1_MOUSE]	1.55	1	2	2	3	1.07	1.10	1.03	0.97	0.91	0.83	0.77	0.99	0.92	0.99	0.92	0.99	0.93	253.75	6.81

P70280	Vesicle-associated membrane protein 7 OS=Mus musculus GN=Vamp7 PE=1 SV=1 - [VAMP7_MOUSE]	5.45	1	1	1	2	1.25	4.58	3.65	1.32	1.05	1.04	0.83	0.99	0.79	1.19	0.96	1.25	1.00	24.95	8.60
Q99NE5-6	Isoform 6 of Regulating synaptic membrane exocytosis protein 1 OS=Mus musculus GN=Rims1 - [RIMS1_MOUSE]	38.88	13	10	37	90	0.98	0.98	0.95	1.02	1.04	0.91	0.99	0.99	0.97	0.95	0.97	0.93	0.94	144.84	9.14
D3Z6I3	Lipoyl synthase, mitochondrial OS=Mus musculus GN=Lias PE=2 SV=1 - [D3Z6I3_MOUSE]	19.03	2	3	3	5	0.90	1.35	2.11	0.80	0.89	1.01	1.07	0.99	1.25	1.00	1.07	0.96	1.25	32.15	8.19
P56480	ATP synthase subunit beta, mitochondrial OS=Mus musculus GN=Atp5b PE=1 SV=2 - [ATPB_MOUSE]	79.40	1	30	30	946	0.99	0.97	0.99	1.03	1.04	1.01	1.02	0.99	0.99	1.02	1.04	1.03	1.05	56.27	5.34
P45591	Cofilin-2 OS=Mus musculus GN=Cfl2 PE=1 SV=1 - [COF2_MOUSE]	77.71	1	11	14	184	0.82	0.67	0.77	0.69	0.83	0.91	1.11	0.99	1.12	0.75	0.88	0.72	0.94	18.70	7.88
P40630-2	Isoform Nuclear of Transcription factor A, mitochondrial OS=Mus musculus GN=Tfam - [TFAM_MOUSE]	26.51	3	6	6	30	1.00	0.87	0.81	0.91	0.93	0.91	0.90	0.99	0.96	0.90	0.92	0.91	0.85	25.02	9.66
F6XTP0	RING finger protein 121 (Fragment) OS=Mus musculus GN=Rnf121 PE=4 SV=1 - [F6XTP0_MOUSE]	10.00	2	1	1	2	0.95	1.11	1.18	1.08	1.14	0.97	1.02	0.99	1.04	0.95	1.00	0.97	1.03	19.80	6.42
Q505D7	Optic atrophy 3 protein homolog OS=Mus musculus GN=Opa3 PE=1 SV=1 - [OPA3_MOUSE]	10.61	1	1	2	5	1.13	1.28	1.13	1.38	1.21	0.83	0.73	0.99	0.87	0.90	0.79	0.94	0.83	20.10	8.34
Q9QZM0	Ubiquilin-2 OS=Mus musculus GN=Ubqln2 PE=1 SV=2 - [UBQL2_MOUSE]	28.84	1	8	11	113	1.11	1.09	1.06	0.93	0.78	1.00	0.86	0.99	0.84	0.96	0.86	1.07	0.91	67.31	5.22
Q9DCH4	Eukaryotic translation initiation factor 3 subunit F OS=Mus musculus GN=Eif3f PE=1 SV=2 - [EIF3F_MOUSE]	19.67	1	5	5	11	0.99	1.14	1.17	1.01	1.04	0.96	0.99	0.99	0.97	0.88	0.92	1.02	0.99	37.96	5.58
Q9QXA1-	Isoform 3 of Cysteine and histidine-rich protein 1 OS=Mus musculus GN=Cyhr1 - [CYHR1_MOUSE]	6.80	4	1	1	2	1.14	1.26	1.11	0.84	0.74	0.87	0.76	0.99	0.87	1.13	1.00	1.48	1.31	28.09	6.86
Q8R2U6	Diphosphoinositol polyphosphate phosphohydrolase 2 OS=Mus musculus GN=Nudt4 PE=1 SV=1 - [NUDT4_MOUSE]	40.22	1	3	5	13	0.90	0.95	1.00	0.82	0.89	0.95	1.06	0.99	1.10	0.82	0.92	0.73	0.79	20.14	6.35

Q8CGF7-2	Isoform 2 of Transcription elongation regulator 1 OS=Mus musculus GN=Tcerg1 - [TCRG1_MOUSE]	23.26	3	23	23	46	0.94	0.76	0.80	0.86	0.90	0.94	0.99	0.99	1.05	0.88	0.90	0.78	0.84	121.50	8.54
P70188-2	Isoform KAP3B of Kinesin-associated protein 3 OS=Mus musculus GN=Kifap3 - [KIFA3_MOUSE]	4.02	3	2	3	5	1.04	1.05	1.02	1.00	0.96	0.92	0.88	0.99	0.95	0.85	0.82	1.01	0.98	88.96	5.20
G3X8R0	Receptor accessory protein 5, isoform CRA_a OS=Mus musculus GN=Reep5 PE=4 SV=1 - [G3X8R0_MOUSE]	10.05	1	3	3	9	0.86	0.80	0.92	1.06	1.20	0.92	1.14	0.99	1.17	0.98	1.10	1.01	1.13	21.43	8.12
Q80VQ0	Aldehyde dehydrogenase family 3 member B1 OS=Mus musculus GN=Aldh3b1 PE=2 SV=1 - [AL3B1_MOUSE]	13.03	3	4	4	9	1.00	1.11	1.11	1.09	1.04	0.93	0.92	0.99	1.03	0.99	0.96	0.98	1.02	52.26	7.55
D3YZV2	Potassium voltage-gated channel subfamily C member 3 OS=Mus musculus GN=Kcnc3 PE=2 SV=1 - [D3YZV2_MOUSE]	8.45	6	4	5	7	0.85	1.08	1.29	0.97	1.10	0.92	1.08	0.99	1.19	0.87	1.05	0.88	1.02	81.89	6.98
P11798	Calcium/calmodulin-dependent protein kinase type II subunit alpha OS=Mus musculus GN=Camk2a PE=1 SV=2 - [KCC2A_MOUSE]	53.14	4	1	21	209	1.11	1.03	0.93	1.32	1.18	0.99	0.88	0.99	0.88	1.12	1.01	1.05	0.94	54.08	7.08
A2AP32	NADH dehydrogenase [ubiquinone] 1 beta subcomplex subunit 6 OS=Mus musculus GN=Ndufb6 PE=2 SV=1 - [A2AP32_MOUSE]	18.56	2	1	1	8	0.94	1.61	1.56	1.22	1.22	1.01	1.07	0.99	1.07	1.03	1.05	1.04	1.03	11.73	9.60
F8WHP8	ATP synthase subunit f, mitochondrial OS=Mus musculus GN=Atp5j2 PE=2 SV=1 - [F8WHP8_MOUSE]	31.58	2	3	3	7	0.95	1.20	1.34	1.16	1.26	0.90	0.94	0.99	0.99	1.01	1.15	1.01	1.06	9.00	9.88
Q9Z2M6	Ubiquitin-like protein 3 OS=Mus musculus GN=Ubl3 PE=1 SV=1 - [UBL3_MOUSE]	26.50	1	2	2	3	0.98	0.91	0.92	1.04	1.06	0.90	0.91	0.99	1.01	0.86	0.88	0.99	1.01	13.17	6.92
Q80VP0	Tectonin beta-propeller repeat-containing protein 1 OS=Mus musculus GN=Tecpr1 PE=2 SV=1 - [TCPR1_MOUSE]	9.86	2	9	9	15	1.04	0.94	0.94	1.10	1.15	1.06	1.06	0.99	1.00	0.99	0.99	1.01	1.02	130.18	6.48

A6X935-2	Isoform 2 of Inter alpha-trypsin inhibitor, heavy chain 4 OS=Mus musculus GN=Itih4 - [ITIH4_MOUSE]	1.66	4	1	1	1	0.78	1.31	1.68	0.78	1.00	0.68	0.87	0.99	1.27	1.28	1.64	1.17	1.51	100.33	6.55
G3UWV4	Serine/threonine-protein kinase BRSK2 OS=Mus musculus GN=Brsk2 PE=2 SV=1 - [G3UWV4_MOUSE]	21.09	16	8	11	30	0.93	0.99	1.05	1.01	1.12	0.90	0.94	0.99	1.04	0.92	0.97	0.92	0.96	77.73	8.82
Q9Z1S5-2	Isoform 2 of Neuronal-specific septin-3 OS=Mus musculus GN=Sept3 - [SEPT3_MOUSE]	56.08	2	19	19	99	1.04	0.81	0.85	1.03	0.98	1.03	1.01	0.99	0.93	0.97	0.94	0.95	0.95	38.68	6.81
Q91WG4	Elongator complex protein 2 OS=Mus musculus GN=Elp2 PE=1 SV=1 - [ELP2_MOUSE]	1.08	1	1	1	2	1.25	0.91	0.73	1.08	0.86	1.03	0.82	0.99	0.79	0.92	0.73	1.08	0.86	93.03	5.82
Q7TSZ8	Nucleus accumbens-associated protein 1 OS=Mus musculus GN=Nacc1 PE=1 SV=1 - [NACC1_MOUSE]	2.53	1	1	1	2	0.96	1.14	1.18	0.97	1.00	0.97	1.00	0.99	1.02	0.93	0.97	1.14	1.18	56.50	5.73
O35954	Membrane-associated phosphatidylinositol transfer protein 1 OS=Mus musculus GN=Pitpnm1 PE=1 SV=1 - [PITM1_MOUSE]	9.33	2	9	9	19	0.97	1.09	1.15	1.11	1.12	0.98	1.05	0.99	0.99	1.03	1.08	1.02	1.05	134.86	6.06
A3KGU5	Spectrin alpha chain, non-erythrocytic 1 OS=Mus musculus GN=Sptan1 PE=2 SV=1 - [A3KGU5_MOUSE]	80.83	4	3	215	5378	0.97	0.75	0.75	0.89	0.91	0.98	0.98	0.99	1.03	0.88	0.89	0.86	0.88	282.72	5.33
Q91Z67	SLIT-ROBO Rho GTPase-activating protein 2 OS=Mus musculus GN=Srgap2 PE=1 SV=2 - [SRGP2_MOUSE]	15.50	1	12	13	26	0.98	0.95	1.01	0.99	1.05	0.97	1.05	0.99	0.99	1.00	1.00	0.91	0.97	120.72	6.64
Q8CIV8	Tubulin-specific chaperone E OS=Mus musculus GN=Tbce PE=1 SV=1 - [TBCE_MOUSE]	8.59	2	3	3	4	1.06	1.06	0.95	1.26	1.18	1.10	0.98	0.99	0.93	1.04	1.04	1.04	0.96	59.05	6.29
O35887	Calumenin OS=Mus musculus GN=Calu PE=1 SV=1 - [CALU_MOUSE]	66.35	7	4	16	181	1.09	0.95	0.89	0.87	0.81	1.08	0.96	0.99	1.00	1.12	0.94	0.96	0.98	37.04	4.67
P63032	GTP-binding protein Rhes OS=Mus musculus GN=Rasd2 PE=2 SV=1 - [RHES_MOUSE]	4.89	1	1	1	2	0.87	1.17	1.35	1.17	1.34	0.96	1.11	0.99	1.14	1.13	1.30	1.31	1.52	30.18	9.01

B1AVZ0	Uracil phosphoribosyltransferase homolog OS=Mus musculus GN=Uprt PE=2 SV=1 - [UPP_MOUSE]	6.45	1	2	2	6	0.98	1.04	1.07	0.90	0.88	0.92	0.93	0.99	0.96	0.90	0.89	0.93	0.92	34.26	6.23
P47963	60S ribosomal protein L13 OS=Mus musculus GN=Rpl13 PE=2 SV=3 - [RL13_MOUSE]	34.60	2	7	7	22	0.96	1.14	1.09	1.02	1.02	0.93	1.02	0.99	0.98	0.99	1.02	1.00	1.06	24.29	11.55
Q9D1F0	CAAX box 1 homolog A (Human) OS=Mus musculus GN=Cxx1b PE=4 SV=1 - [Q9D1F0_MOUSE]	8.85	1	1	1	1	0.98	0.85	0.87	1.44	1.47	1.03	1.05	0.99	1.01	1.12	1.14	1.03	1.05	13.59	8.40
Q9D7M1	Glucose-induced degradation protein 8 homolog OS=Mus musculus GN=Gid8 PE=2 SV=1 - [GID8_MOUSE]	57.46	1	8	8	30	1.06	1.01	0.98	0.87	0.90	0.89	0.90	0.99	0.99	0.79	0.87	1.01	0.90	26.76	4.97
D3YTQ9	40S ribosomal protein S15 OS=Mus musculus GN=Rps15 PE=2 SV=1 - [D3YTQ9_MOUSE]	50.00	2	4	4	24	0.98	0.73	0.72	1.00	1.02	1.02	0.96	0.99	0.94	0.95	1.01	0.94	0.96	13.73	10.59
Q9D287	Pre-mRNA-splicing factor SPF27 OS=Mus musculus GN=Bcas2 PE=2 SV=1 - [SPF27_MOUSE]	36.00	2	6	6	15	0.92	0.82	0.94	0.87	0.93	1.05	1.14	0.99	1.16	0.88	1.01	0.80	0.92	26.11	5.66
Q61425	Hydroxyacyl-coenzyme A dehydrogenase, mitochondrial OS=Mus musculus GN=Hadh PE=1 SV=2 - [HCDH_MOUSE]	40.13	2	8	9	29	0.99	1.12	1.09	1.02	1.22	0.98	1.15	0.99	1.13	1.07	1.25	1.03	1.19	34.44	8.65
D3Z2D8	Transmembrane protein 63B (Fragment) OS=Mus musculus GN=Tmem63b PE=2 SV=1 - [D3Z2D8_MOUSE]	2.58	2	1	1	1	1.15	1.37	1.18	1.11	0.96	0.83	0.72	0.99	0.86	0.84	0.73	0.97	0.84	34.89	8.43
Q8K1C0-4	Isoform 4 of Protein angel homolog 2 OS=Mus musculus GN=Angel2 - [ANGE2_MOUSE]	8.49	5	1	1	2	1.03	1.49	1.44	1.23	1.19	0.91	0.88	0.99	0.96	1.19	1.16	1.17	1.14	24.05	8.07
Q8K4S1	1-phosphatidylinositol 4,5-bisphosphate phosphodiesterase epsilon-1 OS=Mus musculus GN=Plce1 PE=1 SV=3 - [PLCE1_MOUSE]	1.10	1	1	1	10	0.91	0.67	0.74	0.74	0.81	0.87	0.94	0.99	1.08	0.86	0.94	0.70	0.77	254.89	6.15
Q0GA42	Metal transporter CNNM1 OS=Mus musculus GN=Cnnm1 PE=1 SV=5 - [CNNM1_MOUSE]	10.83	2	5	8	13	1.08	1.17	1.22	1.06	0.96	0.82	0.91	0.99	0.91	1.01	0.97	1.05	1.00	103.92	6.51

P03958	Adenosine deaminase OS=Mus musculus GN=Ada PE=1 SV=3 - [ADA_MOUSE]	3.69	1	1	1	1	0.93	1.67	1.79	0.79	0.85	1.36	1.45	0.99	1.06	0.76	0.82	1.07	1.15	39.97	5.72
E9QLS6	Phosphatidylinositol 3-kinase catalytic subunit type 3 OS=Mus musculus GN=Pik3c3 PE=2 SV=1 - [E9QLS6_MOUSE]	3.54	4	2	2	4	1.15	1.08	0.93	1.30	1.13	1.17	1.01	0.99	0.86	1.21	1.05	1.19	1.03	96.81	7.20
Q920P5	Adenylate kinase isoenzyme 5 OS=Mus musculus GN=Ak5 PE=2 SV=2 - [KAD5_MOUSE]	29.18	2	12	12	24	1.03	1.04	1.01	0.95	0.95	1.06	1.06	0.99	1.04	0.97	0.95	1.06	1.02	63.28	5.29
P60755	MAM domain-containing glycosylphosphatidylinositol anchor protein 2 OS=Mus musculus GN=Mdga2 PE=2 SV=1 - [MDGA2_MOUSE]	7.06	4	4	4	8	0.95	1.04	1.04	1.08	1.08	0.99	0.98	0.99	0.98	0.94	0.99	0.93	0.94	106.62	7.02
Q9Z2C9	Myotubularin-related protein 7 OS=Mus musculus GN=Mtmr7 PE=2 SV=2 - [MTMR7_MOUSE]	8.18	5	4	4	8	1.05	1.40	1.34	1.10	1.04	0.91	0.86	0.99	1.04	1.00	0.91	1.28	1.15	75.56	6.43
Q99M51	Cytoplasmic protein NCK1 OS=Mus musculus GN=Nck1 PE=1 SV=1 - [NCK1_MOUSE]	29.97	2	11	13	27	0.96	0.75	0.78	0.84	0.89	0.91	0.97	0.99	1.01	0.79	0.87	0.76	0.82	42.86	6.47
O54946-2	Isoform B of DnaJ homolog subfamily B member 6 OS=Mus musculus GN=Dnajb6 - [DNJB6_MOUSE]	23.14	2	1	5	13	0.80	0.81	1.01	0.91	1.13	0.76	0.95	0.99	1.23	0.87	1.09	0.78	0.98	26.96	7.61
Q8C522	Endonuclease domain-containing 1 protein OS=Mus musculus GN=Endod1 PE=1 SV=2 - [ENDD1_MOUSE]	16.77	1	6	6	25	0.97	1.01	1.09	0.99	1.01	0.96	1.00	0.99	0.98	1.02	1.07	1.04	1.04	55.23	6.16
E9Q7S0	Synaptojanin-1 OS=Mus musculus GN=Synj1 PE=2 SV=1 - [E9Q7S0_MOUSE]	48.20	7	45	48	177	1.03	1.00	0.98	1.05	1.02	1.00	0.97	0.99	0.93	0.98	0.96	1.01	0.98	144.50	7.34
Q8BIX3	ARL14 effector protein OS=Mus musculus GN=Arl14ep PE=2 SV=1 - [AL14E_MOUSE]	3.26	1	1	1	2	0.94	0.84	0.89	0.89	0.94	0.95	1.00	0.99	1.05	0.92	0.97	0.95	1.01	30.99	8.24
A2AUR7	Ras suppressor protein 1 OS=Mus musculus GN=Rsu1 PE=4 SV=1 - [A2AUR7_MOUSE]	16.15	3	4	4	5	0.93	0.89	1.02	0.96	1.06	1.00	1.08	0.99	1.11	0.98	1.10	0.90	0.96	29.52	8.22
Q9QYB8	Beta-adducin OS=Mus musculus GN=Add2 PE=1 SV=4 - [ADDB_MOUSE]	58.21	3	34	34	209	0.98	0.94	0.91	0.99	1.03	1.02	1.02	0.99	1.02	0.95	0.99	0.96	0.96	80.59	6.21

P53996	Cellular nucleic acid-binding protein OS=Mus musculus GN=Cnbp PE=2 SV=2 - [CNBP_MOUSE]	35.96	1	1	5	26	1.09	1.01	0.92	0.94	0.86	1.06	0.96	0.99	0.91	0.89	0.81	0.81	0.74	19.58	7.58
Q61187	Tumor susceptibility gene 101 protein OS=Mus musculus GN=Tsg101 PE=1 SV=2 - [TS101_MOUSE]	21.23	2	8	8	13	1.08	0.91	0.82	1.03	1.08	1.06	0.87	0.99	0.81	0.92	0.83	0.95	0.84	44.10	6.71
D6RE34	Protein Cln6 OS=Mus musculus GN=Cln6 PE=2 SV=1 - [D6RE34_MOUSE]	42.50	2	1	1	2	1.03	1.31	1.27	1.11	1.07	0.94	0.91	0.99	0.96	0.99	0.96	1.09	1.06	4.31	9.98
Q8C8T7	Protein ELFN1 OS=Mus musculus GN=Elfn1 PE=2 SV=1 - [ELFN1_MOUSE]	5.07	1	2	4	14	1.13	0.86	0.94	0.95	0.88	1.20	1.15	0.99	1.03	1.13	1.09	0.94	0.93	90.76	8.18
P26041	Moesin OS=Mus musculus GN=Msn PE=1 SV=3 - [MOES_MOUSE]	51.30	1	18	30	74	0.99	1.03	1.07	1.03	1.03	0.96	0.97	0.99	0.95	1.01	1.02	1.04	0.99	67.72	6.60
P80316	T-complex protein 1 subunit epsilon OS=Mus musculus GN=Cct5 PE=1 SV=1 - [TCPE_MOUSE]	58.41	2	24	25	68	1.02	0.87	0.88	1.03	1.04	0.99	0.99	0.99	0.97	0.98	1.06	1.01	1.02	59.59	6.02
P20060	Beta-hexosaminidase subunit beta OS=Mus musculus GN=Hexb PE=2 SV=2 - [HEXB_MOUSE]	18.66	1	8	8	13	1.02	1.13	0.94	1.20	1.17	1.09	1.00	0.99	0.97	0.94	0.96	0.98	0.96	61.08	8.12
P67778	Prohibitin OS=Mus musculus GN=Phb PE=1 SV=1 - [PHB_MOUSE]	69.12	2	15	15	44	1.01	1.13	1.18	1.15	1.18	0.99	1.03	0.99	1.00	1.06	1.10	0.97	0.99	29.80	5.76
Q64520	Guanylate kinase OS=Mus musculus GN=Guk1 PE=1 SV=2 - [KGUA_MOUSE]	36.87	3	5	5	17	1.02	0.72	0.69	0.89	0.90	1.01	0.98	0.99	1.00	0.90	0.88	0.96	0.94	21.90	6.55
P60762-2	Isoform 2 of Mortality factor 4-like protein 1 OS=Mus musculus GN=Morf4l1 - [MO4L1_MOUSE]	4.33	2	1	1	1	1.02	1.16	1.13	1.10	1.07	0.85	0.82	0.99	0.97	1.04	1.02	1.11	1.09	37.21	9.19
E9PWJ0	CUB and sushi domain-containing protein 3 OS=Mus musculus GN=Csmd3 PE=2 SV=1 - [E9PWJ0_MOUSE]	0.62	4	1	2	3	1.03	0.95	0.92	0.98	0.95	0.89	0.86	0.99	0.96	0.91	0.88	1.01	0.98	386.93	5.91
K7N6Y7	Protein Vmn2r21 OS=Mus musculus GN=Vmn2r21 PE=3 SV=1 - [K7N6Y7_MOUSE]	5.39	3	2	2	5	0.82	1.00	1.22	1.21	1.48	0.74	0.78	0.99	1.38	0.80	1.47	0.31	0.38	95.89	7.68
Q9ERU9	E3 SUMO-protein ligase RanBP2 OS=Mus musculus GN=Ranbp2 PE=1 SV=2 - [RBP2_MOUSE]	10.91	1	23	24	49	0.93	1.00	1.06	1.11	1.21	0.97	1.02	0.99	1.08	0.97	1.06	0.97	1.04	340.91	6.18

F8WHW3	Transmembrane protein 55B OS=Mus musculus GN=Tmem55b PE=2 SV=1 - [F8WHW3_MOUSE]	15.88	5	3	3	6	1.04	1.13	0.97	1.06	0.93	1.07	0.81	0.99	0.79	1.13	0.86	1.09	1.03	29.31	8.75
B1AWZ5	Protein NipSnap homolog 3B OS=Mus musculus GN=Nipsnap3b PE=2 SV=1 - [B1AWZ5_MOUSE]	16.75	2	2	2	25	1.06	0.90	0.85	1.02	0.89	1.14	1.03	0.99	0.92	0.99	0.94	0.94	0.87	22.87	9.51
Q8BTE0	UPF0369 protein C6orf57 homolog OS=Mus musculus PE=2 SV=2 - [CF057_MOUSE]	25.00	1	4	4	30	1.12	1.05	0.98	0.80	0.76	1.05	1.03	0.99	1.00	0.87	0.84	1.07	0.95	11.88	9.50
Q5SVL6	Rap1 GTPase-activating protein 2 OS=Mus musculus GN=Rap1gap2 PE=1 SV=1 - [RPGP2_MOUSE]	27.53	3	12	12	30	0.97	1.01	0.99	1.04	1.07	1.04	1.11	0.99	1.01	1.01	1.07	0.98	1.03	78.20	6.43
Q80X90	Filamin-B OS=Mus musculus GN=Flnb PE=1 SV=3 - [FLNB_MOUSE]	14.22	5	25	28	52	0.99	0.89	0.91	0.98	1.00	0.89	0.98	0.99	1.01	0.96	1.04	0.97	0.97	277.65	5.71
Q148V7-2	Isoform 2 of LisH domain and HEAT repeat-containing protein KIAA1468 OS=Mus musculus GN=Kiaa1468 - [K1468_MOUSE]	26.17	5	19	19	38	1.01	1.16	1.14	1.07	1.06	0.99	0.97	0.99	1.03	0.98	1.04	0.97	1.02	131.81	5.29
D3YU58	Anaphase-promoting complex subunit 7 OS=Mus musculus GN=Anapc7 PE=2 SV=1 - [D3YU58_MOUSE]	2.58	2	1	1	1	0.68	0.64	0.94	1.02	1.50	0.95	1.39	0.99	1.46	1.03	1.53	0.77	1.14	56.13	8.51
Q91W67	Ubiquitin-like protein 7 OS=Mus musculus GN=Ubl7 PE=1 SV=2 - [UBL7_MOUSE]	10.26	1	3	3	5	0.83	0.79	0.88	0.79	0.95	0.98	1.13	0.99	1.18	0.76	0.92	0.85	0.98	40.41	5.01
Q99JW4	LIM and senescent cell antigen-like-containing domain protein 1 OS=Mus musculus GN=Lims1 PE=1 SV=3 - [LIMS1_MOUSE]	25.85	3	4	7	20	1.14	0.96	0.91	0.93	0.81	1.01	0.89	0.99	0.84	0.87	0.72	0.98	0.84	37.21	8.05
P13595	Neural cell adhesion molecule 1 OS=Mus musculus GN=Ncam1 PE=1 SV=3 - [NCAM1_MOUSE]	41.79	4	6	38	409	1.10	0.83	0.78	1.01	0.85	1.12	1.02	0.99	0.91	1.03	0.99	1.03	0.96	119.35	4.83
P58044	Isopentenyl-diphosphate Delta-isomerase 1 OS=Mus musculus GN=Idi1 PE=2 SV=1 - [IDI1_MOUSE]	37.44	5	8	8	33	1.11	0.88	0.79	0.94	0.84	1.06	0.95	0.99	0.90	0.88	0.82	0.82	0.79	26.27	6.16
P97801	Survival motor neuron protein OS=Mus musculus GN=Smn1 PE=1 SV=1 - [SMN_MOUSE]	6.25	1	1	1	5	0.87	1.52	1.44	0.90	0.87	0.82	0.93	0.99	1.27	1.10	1.42	1.23	1.21	31.23	7.62

Q3TYA4	Syntaxin-binding protein 6 OS=Mus musculus GN=Stxbp6 PE=2 SV=1 - [Q3TYA4_MOUSE]	13.81	4	3	3	6	1.01	1.18	1.17	1.18	1.08	1.04	0.91	0.99	0.92	0.99	0.95	1.02	1.01	23.67	9.19
Q3TH34	Protein FAM45A OS=Mus musculus GN=Fam45a PE=2 SV=1 - [Q3TH34_MOUSE]	7.63	2	1	1	2	0.84	0.96	1.14	0.94	1.11	1.08	1.27	0.99	1.17	1.01	1.20	0.91	1.08	26.58	5.45
Q80ZJ7	Sorting nexin-32 OS=Mus musculus GN=Snx32 PE=2 SV=1 - [SNX32_MOUSE]	5.20	1	1	3	7	1.09	1.39	1.27	1.12	1.02	1.02	0.93	0.99	0.90	1.00	0.92	1.27	1.16	46.62	6.96
D3YXA6	Tripartite motif-containing protein 46 OS=Mus musculus GN=Trim46 PE=2 SV=1 - [D3YXA6_MOUSE]	9.65	5	5	5	9	0.96	1.12	1.25	1.13	1.12	0.99	1.16	0.99	1.12	0.95	1.05	1.03	1.17	80.86	7.87
Q60649	Caseinolytic peptidase B protein homolog OS=Mus musculus GN=Clpb PE=1 SV=1 - [CLPB_MOUSE]	8.71	4	5	5	11	1.03	1.20	1.23	0.99	0.99	1.01	0.99	0.99	0.96	1.05	1.04	1.15	1.06	75.96	8.51
Q60829	Protein phosphatase 1 regulatory subunit 1B OS=Mus musculus GN=Ppp1r1b PE=1 SV=2 - [PPR1B_MOUSE]	71.13	2	9	9	310	0.96	1.00	1.04	0.91	0.93	1.07	1.08	0.99	1.02	1.09	1.20	1.26	1.30	21.77	4.65
B1AS04	Protein LZIC (Fragment) OS=Mus musculus GN=Lzic PE=2 SV=1 - [B1AS04_MOUSE]	40.00	2	1	4	10	1.31	0.82	0.63	1.02	0.77	1.03	0.78	0.99	0.75	0.87	0.66	0.97	0.74	7.72	4.51
Q80TN4	DnaJ homolog subfamily C member 16 OS=Mus musculus GN=Dnajc16 PE=1 SV=2 - [DJC16_MOUSE]	1.42	1	1	1	1	1.18	1.05	0.89	1.59	1.36	1.34	1.13	0.99	0.84	0.81	0.69	1.24	1.06	89.08	7.55
Q60902	Epidermal growth factor receptor substrate 15-like 1 OS=Mus musculus GN=Eps15l1 PE=1 SV=3 - [EP15R_MOUSE]	48.62	3	4	33	96	1.01	0.91	0.79	1.05	1.06	1.06	1.05	0.99	1.03	0.91	0.93	0.89	0.79	99.25	5.02
Q9CQV8-	Isoform Short of 14-3-3 protein beta/alpha OS=Mus musculus GN=Ywhab - [1433B_MOUSE]	74.59	5	9	19	550	0.98	0.96	0.97	1.00	1.00	1.00	0.99	0.99	1.00	0.95	0.93	0.99	1.01	27.84	4.83
Q9DB77	Cytochrome b-c1 complex subunit 2, mitochondrial OS=Mus musculus GN=Uqcrc2 PE=1 SV=1 - [QCR2_MOUSE]	60.26	1	21	21	207	1.01	1.05	1.01	1.05	1.02	1.00	0.98	0.99	0.97	1.03	0.97	1.04	1.03	48.21	9.25
Q8K4Z3	NAD(P)H-hydrate epimerase OS=Mus musculus GN=Apoa1bp PE=1 SV=1 - [NNRE_MOUSE]	36.52	1	8	8	28	0.95	0.84	0.87	1.09	1.11	1.11	1.11	0.99	1.11	1.10	1.12	1.04	1.09	30.95	7.69

Q64442	Sorbitol dehydrogenase OS=Mus musculus GN=Sord PE=1 SV=3 - [DHSO_MOUSE]	28.57	1	8	8	16	1.03	0.96	0.88	0.99	0.94	0.97	0.86	0.99	0.92	0.95	0.92	0.97	0.88	38.22	7.02
P60710	Actin, cytoplasmic 1 OS=Mus musculus GN=Actb PE=1 SV=1 - [ACTB_MOUSE]	84.27	11	9	29	1484	1.01	1.03	1.02	1.07	1.04	1.00	0.99	0.99	0.96	1.00	0.98	1.05	1.04	41.71	5.48
E9Q1G8	Septin-7 OS=Mus musculus GN=Sept7 PE=3 SV=2 - [E9Q1G8_MOUSE]	61.56	3	29	32	480	0.95	0.89	0.90	0.98	1.01	0.98	1.02	0.99	1.05	0.94	1.01	0.93	0.97	50.62	8.57
A2A4H9	Peptidyl-prolyl cis-trans isomerase FKBP10 OS=Mus musculus GN=Fkbp10 PE=2 SV=1 - [A2A4H9_MOUSE]	2.35	2	1	1	1	0.96	1.03	1.06	0.57	0.59	0.79	0.82	0.99	1.03	0.76	0.79	0.83	0.86	52.30	5.53
Q8VD65	Phosphoinositide 3-kinase regulatory subunit 4 OS=Mus musculus GN=Pik3r4 PE=1 SV=3 - [PI3R4_MOUSE]	2.95	1	3	3	4	1.08	1.01	0.90	1.08	1.00	0.91	0.84	1.00	0.92	0.95	0.89	1.07	0.95	152.50	7.12
Q6PFR5	Transformer-2 protein homolog alpha OS=Mus musculus GN=Tra2a PE=1 SV=1 - [TRA2A_MOUSE]	13.88	2	3	4	12	0.96	0.89	0.92	0.90	1.00	0.82	0.85	1.00	0.91	0.90	0.82	0.91	0.94	32.30	11.28
Q9JL26-2	Isoform 2 of Formin-like protein 1 OS=Mus musculus GN=Fmnl1 - [FMNL_MOUSE]	7.33	3	1	6	16	1.00	0.87	0.87	0.92	0.92	0.77	0.77	1.00	0.99	0.96	0.96	1.11	1.11	118.96	5.66
F7CWH3	Inositol hexakisphosphate kinase 1 OS=Mus musculus GN=Ip6k1 PE=2 SV=1 - [F7CWH3_MOUSE]	13.54	3	1	1	1	1.05	0.95	0.91	1.01	0.97	1.02	0.97	1.00	0.95	0.87	0.83	0.89	0.85	11.17	8.63
D3Z5W7	Glutathione S-transferase theta-1 (Fragment) OS=Mus musculus GN=Gstt1 PE=2 SV=1 - [D3Z5W7_MOUSE]	10.32	3	1	1	2	1.41	0.92	0.66	1.03	0.73	0.90	0.63	1.00	0.71	1.24	0.88	1.35	0.96	14.46	6.27
Q3U1Z5-2	Isoform 2 of G-protein-signaling modulator 3 OS=Mus musculus GN=Gpsm3 - [GPSM3_MOUSE]	14.88	2	1	1	3	0.85	0.92	1.08	0.89	1.05	0.94	1.10	1.00	1.17	0.94	1.10	0.91	1.07	13.48	4.94
P14152	Malate dehydrogenase, cytoplasmic OS=Mus musculus GN=Mdh1 PE=1 SV=3 - [MDHC_MOUSE]	55.69	3	20	20	228	0.97	0.94	0.94	1.03	1.05	1.01	1.01	1.00	1.02	1.00	1.02	1.00	1.02	36.49	6.58
Q91WJ8	Far upstream element-binding protein 1 OS=Mus musculus GN=Fubp1 PE=1 SV=1 - [FUBP1_MOUSE]	48.54	2	2	23	201	1.05	0.91	0.87	1.06	0.99	0.99	1.01	1.00	1.02	1.00	1.01	0.98	0.93	68.50	7.93

Q8VC85	U6 snRNA-associated Sm-like protein LSm1 OS=Mus musculus GN=Lsm1 PE=2 SV=1 - [LSM1_MOUSE]	36.09	1	4	4	8	1.00	1.06	0.95	0.96	0.93	1.02	0.99	1.00	0.99	0.97	0.95	1.11	0.99	15.23	5.38
E9PX61	Protein Gm15800 OS=Mus musculus GN=Gm15800 PE=2 SV=1 - [E9PX61_MOUSE]	1.53	2	5	5	8	0.93	1.08	1.25	0.95	1.03	0.90	0.97	1.00	0.97	0.88	0.96	0.87	1.11	452.28	6.18
P62245	40S ribosomal protein S15a OS=Mus musculus GN=Rps15a PE=2 SV=2 - [RS15A_MOUSE]	53.08	4	6	6	15	1.11	1.07	1.01	1.05	1.01	0.96	1.01	1.00	0.95	1.00	1.04	1.11	1.07	14.83	10.13
Q9DCS3	Trans-2-enoyl-CoA reductase, mitochondrial OS=Mus musculus GN=Mecr PE=2 SV=2 - [MECR_MOUSE]	41.29	2	7	7	11	0.83	1.03	1.18	1.11	1.27	1.15	1.26	1.00	1.19	1.15	1.31	1.10	1.39	40.32	9.07
Q8VI75	Importin-4 OS=Mus musculus GN=Ipo4 PE=1 SV=1 - [IPO4_MOUSE]	5.27	3	5	5	7	1.11	1.29	1.16	1.24	1.09	0.99	0.88	1.00	0.85	1.06	1.00	1.07	0.97	119.20	5.03
B1AU41	Alpha-1B adrenergic receptor (Fragment) OS=Mus musculus GN=Adra1b PE=2 SV=1 - [B1AU41_MOUSE]	2.81	2	1	1	1	0.77	1.07	1.39	1.04	1.35	0.91	1.18	1.00	1.29	0.97	1.26	0.99	1.29	51.09	9.54
Q8C3P7	N6-adenosine-methyltransferase 70 kDa subunit OS=Mus musculus GN=Mettl3 PE=2 SV=2 - [MTA70_MOUSE]	3.97	5	2	2	4	1.02	1.09	1.15	0.97	1.03	1.09	1.06	1.00	1.05	0.98	0.99	1.02	1.01	64.58	6.49
A8XY17	Vacuolar protein-sorting-associated protein 25 OS=Mus musculus GN=Vps25 PE=2 SV=1 - [A8XY17_MOUSE]	19.53	6	2	2	4	1.20	0.89	0.74	1.22	1.02	1.07	0.88	1.00	0.83	1.03	0.86	1.20	1.01	14.95	5.17
O70161-3	Isoform 3 of Phosphatidylinositol 4-phosphate 5-kinase type-1 gamma OS=Mus musculus GN=Pip5k1c - [PI51C_MOUSE]	36.06	9	16	17	89	0.97	0.99	1.00	1.01	1.03	1.05	1.06	1.00	1.06	1.03	1.05	0.98	0.99	69.46	5.73
Q91V57-3	Isoform 3 of N-chimaerin OS=Mus musculus GN=Chn1 - [CHIN_MOUSE]	19.05	6	3	3	7	0.95	1.25	1.32	1.04	1.10	1.06	1.15	1.00	1.07	1.06	1.16	1.03	1.09	24.08	5.49
P35279	Ras-related protein Rab 6A OS=Mus musculus GN=Rab6a PE=1 SV=4 - [RAB6A_MOUSE]	45.67	12	5	9	75	1.10	1.24	1.15	1.02	0.91	0.92	0.83	1.00	0.84	1.01	0.90	1.05	0.91	23.57	5.54
Q6V4S5	Protein sidekick-2 OS=Mus musculus GN=Sdk2 PE=2 SV=1 - [SDK2_MOUSE]	6.30	4	10	10	26	1.04	1.08	1.00	1.04	1.01	0.91	0.87	1.00	0.96	0.97	0.91	0.97	0.93	239.75	7.36

A2RSJ4	UHRF1-binding protein 1-like OS=Mus musculus GN=Uhrf1bp1l PE=2 SV=2 - [UH1BL_MOUSE]	7.28	1	7	8	14	0.86	1.02	1.14	0.98	1.19	1.18	1.36	1.00	1.15	1.15	1.33	1.03	1.11	161.84	6.61
Q8VDQ8	NAD-dependent protein deacetylase sirtuin-2 OS=Mus musculus GN=Sirt2 PE=1 SV=2 - [SIR2_MOUSE]	62.72	3	19	19	110	0.94	0.96	1.05	1.09	1.16	1.00	1.06	1.00	1.05	0.94	1.01	0.95	1.06	43.23	5.35
Q8K310	Matrin-3 OS=Mus musculus GN=Matr3 PE=1 SV=1 - [MATR3_MOUSE]	33.45	1	21	21	61	0.94	1.02	1.05	0.99	1.02	1.03	1.11	1.00	1.07	0.99	1.02	1.07	1.14	94.57	6.25
G3X909	Slit homolog 2 protein OS=Mus musculus GN=Slit2 PE=2 SV=1 - [G3X909_MOUSE]	3.41	6	2	2	4	1.07	1.22	1.13	1.01	0.94	0.96	0.89	1.00	0.93	0.95	0.89	1.03	0.96	95.43	8.54
Q9CXU9	Eukaryotic translation initiation factor 1b OS=Mus musculus GN=Eif1b PE=2 SV=2 - [EIF1B_MOUSE]	61.06	5	1	6	63	1.02	0.68	0.67	0.87	0.86	0.92	0.91	1.00	0.98	0.69	0.68	0.83	0.81	12.82	7.37
Q9CWF2	Tubulin beta-2B chain OS=Mus musculus GN=Tubb2b PE=1 SV=1 - [TBB2B_MOUSE]	74.38	2	1	28	1881	1.07	0.82	0.81	1.08	1.02	1.09	0.92	1.00	0.94	1.14	1.05	1.12	1.07	49.92	4.89
Q8K440-2	Isoform 2 of ATP-binding cassette sub-family A member 8-B OS=Mus musculus GN=Abca8b - [ABC8B_MOUSE]	0.83	2	1	1	2	0.82	1.39	1.69	1.01	1.23	0.87	1.05	1.00	1.21	0.85	1.04	0.86	1.05	176.05	7.03
B2RQ71	Dip2c protein OS=Mus musculus GN=Dip2c PE=2 SV=1 - [B2RQ71_MOUSE]	13.24	2	1	15	35	1.06	1.27	1.20	0.99	0.94	0.95	0.90	1.00	0.94	1.02	0.97	0.97	0.92	170.81	7.39
F6QP10	Sn1-specific diacylglycerol lipase beta (Fragment) OS=Mus musculus GN=Daglb PE=4 SV=2 - [F6QP10_MOUSE]	1.53	2	1	1	1	0.93	1.30	1.39	1.20	1.28	1.38	1.47	1.00	1.06	1.02	1.09	1.27	1.36	64.62	6.44
O08908	Phosphatidylinositol 3-kinase regulatory subunit beta OS=Mus musculus GN=Pik3r2 PE=1 SV=2 - [P85B_MOUSE]	7.62	1	3	3	6	0.95	1.14	1.20	1.07	1.06	1.04	1.09	1.00	0.99	1.00	1.03	1.01	1.00	81.22	6.07
Q63829	COMM domain-containing protein 3 OS=Mus musculus GN=Commd3 PE=2 SV=1 - [COMD3_MOUSE]	5.64	1	1	1	1	0.99	0.82	0.83	0.96	0.97	1.22	1.22	1.00	1.00	1.25	1.27	1.12	1.13	22.02	5.59

P70697	Uroporphyrinogen decarboxylase OS=Mus musculus GN=Urod PE=1 SV=2 - [DCUP_MOUSE]	14.99	1	4	4	6	0.77	1.01	1.23	1.11	1.24	0.96	1.04	1.00	1.21	1.12	1.22	1.04	1.18	40.67	6.65
Q8BTE5	Protein 1110001A16Rik OS=Mus musculus GN=1110001A16Rik PE=4 SV=2 - [Q8BTE5_MOUSE]	13.75	1	1	1	2	1.04	0.84	0.81	0.91	0.87	0.98	0.94	1.00	0.96	1.01	0.98	1.06	1.02	9.28	9.45
D3YZT4	Sphingosine-1-phosphate lyase 1 (Fragment) OS=Mus musculus GN=Sgpl1 PE=2 SV=1 - [D3YZT4_MOUSE]	7.54	3	1	1	3	0.99	1.14	1.16	1.30	1.31	0.93	0.93	1.00	1.00	1.01	1.02	1.06	1.07	22.96	8.84
P28659-2	Isoform 2 of CUGBP Elav-like family member 1 OS=Mus musculus GN=Celf1 - [CELF1_MOUSE]	10.79	4	3	4	7	0.92	1.12	1.15	1.00	1.08	0.96	1.06	1.00	1.08	0.99	1.11	1.12	1.21	51.56	8.47
A2A547	60S ribosomal protein L19 OS=Mus musculus GN=Rpl19 PE=2 SV=1 - [A2A547_MOUSE]	17.53	2	5	5	14	1.06	0.90	0.92	1.16	1.09	1.03	1.11	1.00	0.98	1.05	1.07	1.00	1.06	23.23	11.47
Q7TMW6	Cytosolic Fe-S cluster assembly factor NARFL OS=Mus musculus GN=Narfl PE=2 SV=2 - [NARFL_MOUSE]	10.71	2	4	4	6	0.99	1.21	1.19	1.09	1.04	0.95	0.94	1.00	1.01	1.06	1.07	1.03	1.01	53.07	6.48
Q9CX00	IST1 homolog OS=Mus musculus GN=Ist1 PE=2 SV=1 - [IST1_MOUSE]	15.75	1	6	6	8	1.06	0.93	0.91	0.98	0.94	0.88	0.92	1.00	0.95	0.97	0.94	0.95	0.99	39.44	5.44
Q9JI78	Peptide-N(4)-(N-acetyl-beta-glucosaminyl)asparagine amidase OS=Mus musculus GN=Ngly1 PE=1 SV=2 - [NGLY1_MOUSE]	5.38	1	3	3	4	0.94	1.17	1.15	0.93	0.99	0.98	1.03	1.00	0.96	1.03	1.04	1.04	1.10	74.23	6.89
Q61584-6	Isoform F of Fragile X mental retardation syndrome-related protein 1 OS=Mus musculus GN=Fxr1 - [FXR1_MOUSE]	22.38	7	8	11	19	1.01	1.06	1.09	1.05	1.01	1.02	0.94	1.00	0.91	1.06	0.97	1.01	0.96	73.08	6.98
Q99LD7	E3 ubiquitin-protein ligase makorin-1 OS=Mus musculus GN=Mkrn1 PE=2 SV=1 - [Q99LD7_MOUSE]	6.38	7	2	2	3	1.00	0.75	0.74	0.86	0.85	1.09	1.08	1.00	0.99	0.92	0.91	0.83	0.83	34.94	6.51
A2BGI8	Peptidyl-prolyl cis-trans isomerase H (Fragment) OS=Mus musculus GN=Ppih PE=2 SV=1 - [A2BGI8_MOUSE]	28.48	5	3	4	13	1.09	1.13	1.04	1.06	1.07	1.06	0.96	1.00	0.98	0.97	0.99	0.96	0.97	17.22	8.34

P35436	Glutamate receptor ionotropic, NMDA 2A OS=Mus musculus GN=Grin2a PE=1 SV=2 - [NMDE1_MOUSE]	9.08	1	9	9	16	0.95	0.97	1.08	1.03	1.11	0.90	0.93	1.00	1.01	0.91	0.95	0.93	0.90	165.32	7.01
E9Q6X0	Microtubule-associated protein RP/EB family member 2 OS=Mus musculus GN=Mapre2 PE=2 SV=1 - [E9Q6X0_MOUSE]	62.32	5	12	14	43	0.96	1.00	1.11	1.03	1.06	0.94	1.00	1.00	1.01	1.05	1.07	0.97	1.10	32.24	5.26
Q6X893	Choline transporter-like protein 1 OS=Mus musculus GN=Slc44a1 PE=1 SV=3 - [CTL1_MOUSE]	10.41	6	5	5	10	0.99	1.27	1.16	1.10	0.98	0.91	0.96	1.00	0.91	1.10	1.07	1.12	1.23	73.02	8.75
Q8BG58	Transmembrane prolyl 4-hydroxylase OS=Mus musculus GN=P4htm PE=2 SV=1 - [P4HTM_MOUSE]	5.77	1	2	2	4	0.99	1.17	1.18	1.12	1.13	1.22	1.22	1.00	1.00	1.07	1.08	1.07	1.08	57.01	6.09
Q9D967	Magnesium-dependent phosphatase 1 OS=Mus musculus GN=Mdp1 PE=1 SV=1 - [MGDP1_MOUSE]	54.27	1	6	6	14	0.99	0.86	0.93	0.91	0.97	0.96	0.96	1.00	1.00	0.96	0.90	0.95	0.96	18.57	6.80
Q6ZQ93-4	Isoform 4 of Ubiquitin carboxyl-terminal hydrolase 34 OS=Mus musculus GN=Usp34 - [UBP34_MOUSE]	1.82	7	2	2	12	0.92	0.71	0.77	0.92	1.01	0.99	1.08	1.00	1.09	0.99	1.09	0.87	0.96	275.37	5.52
Q91XW9	MCG133388, isoform CRA_f OS=Mus musculus GN=Pcdhgc5 PE=2 SV=1 - [Q91XW9_MOUSE]	9.85	5	4	6	23	0.88	1.09	1.23	1.03	1.17	1.14	1.21	1.00	1.19	1.07	1.21	0.93	1.06	101.83	5.27
Q8BTW3	Exosome complex component MTR3 OS=Mus musculus GN=Exosc6 PE=1 SV=1 - [EXOS6_MOUSE]	5.49	1	1	1	2	0.98	0.91	0.93	0.92	0.93	1.04	1.06	1.00	1.01	0.99	1.01	0.95	0.96	28.35	6.11
G5E924	Heterogeneous nuclear ribonucleoprotein L (Fragment) OS=Mus musculus GN=Hnrnpl PE=4 SV=1 - [G5E924_MOUSE]	42.11	5	16	16	111	0.91	0.95	0.98	0.96	1.02	0.96	1.07	1.00	1.07	0.94	0.99	0.93	1.00	66.89	8.18
O88413	Tubby-related protein 3 OS=Mus musculus GN=Tulp3 PE=1 SV=1 - [TULP3_MOUSE]	2.39	1	1	1	2	0.95	1.18	1.24	0.85	0.90	0.96	1.01	1.00	1.05	1.02	1.07	0.99	1.05	51.20	6.24
Q61137-2	Isoform 2 of Astrotactin-1 OS=Mus musculus GN=Astn1 - [ASTN1_MOUSE]	24.81	3	23	23	58	1.03	0.99	0.98	1.02	1.01	0.97	0.97	1.00	0.97	0.97	0.98	0.94	0.91	143.86	5.15

Q5DU31-	Isoform 2 of Interactor protein for cytohesin exchange factors 1 OS=Mus musculus GN=Ipcef1 - [ICEF1_MOUSE]	25.61	6	9	9	29	0.97	0.91	0.92	0.94	0.99	1.03	1.02	1.00	1.07	0.90	0.93	0.84	0.86	46.25	6.73
G3X8S2	BRF1 homolog, subunit of RNA polymerase III transcription initiation factor IIIB (S. cerevisiae) OS=Mus musculus GN=Brf1 PE=4 SV=1 - [G3X8S2_MOUSE]	1.63	2	1	1	2	1.08	0.94	0.87	0.99	0.91	1.08	0.99	1.00	0.92	1.10	1.02	1.03	0.95	73.76	5.30
Q91ZM2-	Isoform 5 of SH2B adapter protein 1 OS=Mus musculus GN=Sh2b1 - [SH2B1_MOUSE]	17.83	6	5	5	9	1.05	0.81	1.04	1.06	1.04	1.01	1.08	1.00	1.12	1.04	1.05	1.03	1.12	46.36	6.09
Q8R3L2-3	Isoform 3 of Transcription factor 25 OS=Mus musculus GN=Tcf25 - [TCF25_MOUSE]	8.35	5	3	3	17	0.96	0.99	1.09	0.99	1.02	0.89	0.87	1.00	1.03	0.80	0.81	0.94	0.97	69.26	7.25
P55144-3	Isoform 1 of Tyrosine-protein kinase receptor TYRO3 OS=Mus musculus GN=Tyro3 - [TYRO3_MOUSE]	8.79	3	5	6	13	0.98	1.27	1.27	1.03	1.06	0.94	0.98	1.00	1.02	1.03	1.08	1.03	1.07	95.98	5.60
Q924S7	Sprouty-related, EVH1 domain-containing protein 2 OS=Mus musculus GN=Spred2 PE=1 SV=1 - [SPRE2_MOUSE]	10.24	3	2	3	5	0.92	1.16	1.27	0.92	1.01	0.99	1.08	1.00	1.09	1.10	1.20	0.90	0.99	46.76	6.81
A2A9Z1	Dystrophin OS=Mus musculus GN=Dmd PE=2 SV=1 - [A2A9Z1_MOUSE]	17.55	5	7	8	33	1.00	1.14	1.05	0.98	1.01	1.12	1.06	1.00	1.07	1.04	1.05	0.92	0.94	68.88	6.62
Q3UFS0-2	Isoform 2 of Protein zyg-11 homolog B OS=Mus musculus GN=Zyg11b - [ZY11B_MOUSE]	3.36	3	1	1	1	0.92	0.97	1.05	0.89	0.96	0.81	0.87	1.00	1.08	0.80	0.87	1.10	1.19	50.07	7.20
Q9CR26	Vacuolar protein sorting-associated protein VTA1 homolog OS=Mus musculus GN=Vta1 PE=1 SV=1 - [VTA1_MOUSE]	12.30	2	3	3	6	0.91	0.98	1.05	0.94	1.00	1.02	1.11	1.00	1.10	1.02	1.09	1.07	1.14	33.89	6.13
P63080	Gamma-aminobutyric acid receptor subunit beta-3 OS=Mus musculus GN=Gabrb3 PE=2 SV=1 - [GBRB3_MOUSE]	21.99	3	4	7	29	0.99	1.24	1.14	1.13	1.15	0.93	0.90	1.00	1.01	0.99	1.02	1.11	0.91	54.13	9.10
Q3TEA8-	Isoform 2 of Heterochromatin protein 1-binding protein 3 OS=Mus musculus GN=Hp1bp3 - [HP1B3_MOUSE]	21.44	8	10	10	46	0.99	0.77	0.77	1.00	1.03	1.10	1.13	1.00	0.98	1.05	1.05	1.01	1.03	59.51	9.79

Q9WVB0	RNA-binding protein with multiple splicing OS=Mus musculus GN=Rbpms PE=2 SV=2 - [RBPMS_MOUSE]	7.61	2	1	1	5	1.09	1.28	1.17	0.93	0.85	1.05	0.96	1.00	0.91	1.00	0.92	1.00	0.94	21.80	8.07
Q61301-2	Isoform 2 of Catenin alpha-2 OS=Mus musculus GN=Ctnna2 - [CTNA2_MOUSE]	40.44	5	22	29	94	0.98	1.02	1.08	1.05	1.14	0.89	0.93	1.00	0.98	0.96	1.03	1.00	1.02	100.36	5.96
P35564	Calnexin OS=Mus musculus GN=Canx PE=1 SV=1 - [CALX_MOUSE]	35.87	1	20	20	122	1.01	0.97	0.97	1.04	1.03	1.06	1.04	1.00	0.99	1.03	1.02	1.01	1.00	67.24	4.64
G3UWG1	MCG115977 OS=Mus musculus GN=Gm10108 PE=3 SV=1 - [G3UWG1_MOUSE]	63.81	2	10	12	495	1.03	0.82	0.80	0.92	0.89	0.94	0.92	1.00	0.98	0.92	0.91	0.87	0.84	11.69	9.64
Q5SX39	Myosin-4 OS=Mus musculus GN=Myh4 PE=1 SV=1 - [MYH4_MOUSE]	39.50	3	17	65	208	1.00	1.21	1.13	0.98	0.91	10.37	11.11	1.00	1.09	1.15	1.09	1.08	1.07	222.72	5.74
Q9R0N3	Synaptotagmin-11 OS=Mus musculus GN=Syt11 PE=2 SV=2 - [SYT11_MOUSE]	10.23	1	3	3	5	1.15	1.06	1.11	1.14	1.11	0.93	0.94	1.00	0.88	0.95	0.94	0.93	0.88	48.30	9.11
P46471	26S protease regulatory subunit 7 OS=Mus musculus GN=Psmc2 PE=1 SV=5 - [PRS7_MOUSE]	50.12	2	17	17	62	0.95	1.13	1.20	1.03	1.13	0.98	1.05	1.00	1.04	1.07	1.16	1.02	1.10	48.62	5.95
Q9D8U8	Sorting nexin-5 OS=Mus musculus GN=Snx5 PE=1 SV=1 - [SNX5_MOUSE]	22.77	1	7	7	18	0.98	0.84	0.92	0.95	1.03	0.89	0.98	1.00	1.02	0.98	0.97	0.97	1.01	46.77	6.62
Q5SSM3-	Isoform 2 of Rho GTPase-activating protein 44 OS=Mus musculus GN=Arhgap44 - [RHG44_MOUSE]	15.31	5	8	8	15	0.94	0.94	0.98	1.06	1.15	0.96	1.04	1.00	1.05	0.98	1.08	0.99	1.02	83.22	6.73
D3YZU5	SH3 and multiple ankyrin repeat domains protein 1 OS=Mus musculus GN=Shank1 PE=2 SV=1 - [D3YZU5_MOUSE]	18.07	3	27	27	84	0.92	1.03	1.09	1.07	1.11	0.94	1.05	1.00	1.07	0.92	0.96	0.92	1.00	225.32	8.47
F7BUB1	NF-kappa-B inhibitor-interacting Ras-like protein 1 (Fragment) OS=Mus musculus GN=Nkiras1 PE=2 SV=1 - [F7BUB1_MOUSE]	10.17	2	1	1	1	0.96	1.06	1.10	1.05	1.09	1.01	1.05	1.00	1.04	0.95	0.99	1.00	1.05	13.61	4.96
P35951	Low-density lipoprotein receptor OS=Mus musculus GN=Ldlr PE=1 SV=2 - [LDLR_MOUSE]	1.51	1	1	1	2	1.05	1.14	1.07	0.91	0.86	0.95	0.90	1.00	0.94	1.10	1.04	0.98	0.93	94.89	5.02

E9Q0P6	Lys-63-specific deubiquitinase BRCC36 (Fragment) OS=Mus musculus GN=Brcc3 PE=2 SV=1 - [E9Q0P6_MOUSE]	38.00	4	1	1	2	1.09	1.44	1.31	1.02	0.93	1.06	0.97	1.00	0.91	1.08	0.98	1.04	0.95	5.81	9.69
Q8C3X4-2	Isoform 2 of Translation factor Guf1, mitochondrial OS=Mus musculus GN=Guf1 - [GUF1_MOUSE]	1.60	3	1	1	1	0.94	1.30	1.38	0.99	1.05	0.88	0.93	1.00	1.06	0.98	1.04	1.10	1.17	62.89	8.94
O08530	Sphingosine 1-phosphate receptor 1 OS=Mus musculus GN=S1pr1 PE=2 SV=3 - [S1PR1_MOUSE]	13.35	1	4	4	10	1.06	1.20	1.41	1.15	1.08	1.07	0.97	1.00	1.00	1.02	0.99	1.04	1.00	42.61	9.42
F6QA74	DNA-(apurinic or apyrimidinic site) lyase (Fragment) OS=Mus musculus GN=Apex1 PE=2 SV=1 - [F6QA74_MOUSE]	29.76	3	6	6	14	0.96	1.30	1.11	1.12	1.20	0.99	0.95	1.00	1.02	0.98	1.03	1.15	1.24	32.30	8.57
Q9CQ88	Tetraspanin-31 OS=Mus musculus GN=Tspan31 PE=1 SV=1 - [TSN31_MOUSE]	5.71	1	1	1	1	1.14	0.86	0.75	0.93	0.81	0.99	0.86	1.00	0.87	0.93	0.81	1.17	1.02	22.68	8.44
P62746	Rho-related GTP-binding protein RhoB OS=Mus musculus GN=Rhob PE=1 SV=1 - [RHOB_MOUSE]	66.84	1	6	9	106	1.00	0.93	0.93	0.92	0.97	0.99	1.00	1.00	1.00	0.99	0.99	0.94	0.95	22.11	5.24
A2A432-2	Isoform 2 of Cullin-4B OS=Mus musculus GN=Cul4b - [CUL4B_MOUSE]	12.43	4	7	11	23	1.16	1.12	1.11	1.10	1.01	0.98	0.91	1.00	0.91	1.06	0.95	1.12	1.06	107.32	8.48
H3BJE9	RING finger protein 141 (Fragment) OS=Mus musculus GN=Rnf141 PE=2 SV=1 - [H3BJE9_MOUSE]	17.11	3	1	1	4	0.87	1.18	1.35	0.95	1.10	0.91	1.05	1.00	1.15	0.82	0.95	0.84	0.97	8.39	5.83
Q8BMJ3	Eukaryotic translation initiation factor 1A, X-chromosomal OS=Mus musculus GN=Eif1ax PE=2 SV=3 - [IF1AX_MOUSE]	14.58	1	1	2	3	0.58	0.91	1.56	0.66	1.14	0.91	1.56	1.00	1.71	0.75	1.30	0.96	1.66	16.45	5.24
Q8BZ47	Zinc finger protein 609 OS=Mus musculus GN=Znf609 PE=1 SV=2 - [ZN609_MOUSE]	1.49	1	1	1	2	1.03	0.52	0.50	1.02	0.99	1.33	1.28	1.00	0.97	1.16	1.12	0.90	0.88	151.02	8.00
Q68EF0	Rab-3A-interacting protein OS=Mus musculus GN=Rab3ip PE=1 SV=1 - [RAB3I_MOUSE]	7.94	1	2	2	5	1.02	0.87	1.00	1.06	1.07	1.00	1.05	1.00	1.12	1.01	1.12	1.11	1.13	47.10	6.71

B1ATZ0	Hepatocyte growth factor-regulated tyrosine kinase substrate OS=Mus musculus GN=Hgs PE=2 SV=1 - [B1ATZ0_MOUSE]	27.55	6	20	20	73	0.94	0.94	1.03	0.93	1.00	0.96	1.02	1.00	1.06	0.92	0.97	0.94	1.00	85.72	7.47
Q9WUU7	Cathepsin Z OS=Mus musculus GN=Ctsz PE=2 SV=1 - [CATZ_MOUSE]	11.76	1	3	3	6	0.88	0.93	1.12	0.89	0.99	0.95	1.14	1.00	1.20	0.95	1.12	0.93	1.05	33.97	6.60
Q62470-3	Isoform 3 of Integrin alpha-3 OS=Mus musculus GN=Itga3 - [ITA3_MOUSE]	1.66	4	2	2	3	1.11	1.06	0.95	0.91	0.82	0.98	0.87	1.00	0.90	1.02	0.91	0.96	0.87	113.36	6.14
P84075	Neuron-specific calcium-binding protein hippocalcin OS=Mus musculus GN=Hpca PE=1 SV=2 - [HPCA_MOUSE]	79.27	4	6	19	286	0.96	0.88	0.93	0.91	0.97	1.00	1.02	1.00	1.06	0.85	0.85	1.00	1.03	22.41	4.97
P68037	Ubiquitin-conjugating enzyme E2 L3 OS=Mus musculus GN=Ube2l3 PE=2 SV=1 - [UB2L3_MOUSE]	63.64	2	6	6	43	1.02	0.76	0.80	0.89	0.90	0.99	1.01	1.00	1.02	0.93	0.93	0.88	0.91	17.85	8.51
Q8VE70	Programmed cell death protein 10 OS=Mus musculus GN=Pdcd10 PE=1 SV=1 - [PDC10_MOUSE]	17.92	3	4	4	10	1.01	1.13	1.12	1.07	1.03	0.96	0.92	1.00	0.95	0.93	0.90	0.92	0.89	24.70	8.19
Q6VVW5	Isoform 3 of Atrial natriuretic peptide receptor 2 OS=Mus musculus GN=Npr2 - [ANPRB_MOUSE]	3.11	4	1	1	2	1.02	2.05	2.00	1.08	1.05	0.95	0.92	1.00	0.97	1.04	1.02	1.02	1.00	46.41	6.07
Q8VBV7	COP9 signalosome complex subunit 8 OS=Mus musculus GN=Cops8 PE=1 SV=1 - [CSN8_MOUSE]	42.58	1	5	5	26	1.03	1.20	1.18	1.03	1.02	1.07	1.03	1.00	1.01	1.12	1.09	0.96	1.01	23.24	5.20
Q5SX40	Myosin-1 OS=Mus musculus GN=Myh1 PE=1 SV=1 - [MYH1_MOUSE]	40.01	5	10	68	228	0.95	1.14	1.24	0.83	0.80	10.19	10.51	1.00	0.86	1.06	0.88	1.03	1.05	223.20	5.76
Q9D115	Zinc finger protein 706 OS=Mus musculus GN=Znf706 PE=2 SV=1 - [ZN706_MOUSE]	14.47	1	1	1	2	0.88	0.84	0.95	0.77	0.87	1.05	1.19	1.00	1.13	0.94	1.06	0.86	0.97	8.49	10.01
P62307	Small nuclear ribonucleoprotein F OS=Mus musculus GN=Snrpf PE=2 SV=1 - [RUXF_MOUSE]	6.98	1	1	1	1	1.14	1.16	1.02	1.34	1.17	1.15	1.00	1.00	0.88	1.23	1.08	1.11	0.98	9.72	4.67
Q8BML1	Protein-methionine sulfoxide oxidase MICAL2 OS=Mus musculus GN=Mical2 PE=1 SV=1 - [MICA2_MOUSE]	3.33	4	1	3	7	0.87	1.12	1.28	0.75	0.86	1.15	1.31	1.00	1.15	0.96	1.11	0.89	1.03	110.00	8.10

E9QPI5	Sister chromatid cohesion protein PDS5 homolog A OS=Mus musculus GN=Pds5a PE=4 SV=1 - [E9QPI5_MOUSE]	1.50	2	1	1	4	0.81	0.98	1.21	1.19	1.47	0.94	1.15	1.00	1.24	1.23	1.53	1.08	1.33	150.13	7.85
Q80TR1	Latrophilin-1 OS=Mus musculus GN=Lphn1 PE=1 SV=2 - [LPHN1_MOUSE]	24.08	7	24	24	67	1.05	1.13	1.07	1.06	1.03	0.99	0.94	1.00	0.96	1.04	1.01	1.01	0.97	161.58	6.48
Q3TPE9-2	Isoform 2 of Ankyrin repeat and MYND domain-containing protein 2 OS=Mus musculus GN=Ankmy2 - [ANKY2_MOUSE]	10.00	2	3	3	6	0.97	0.87	0.95	1.03	0.94	0.95	0.97	1.00	1.07	1.01	1.03	1.00	1.08	33.60	8.12
P35980	60S ribosomal protein L18 OS=Mus musculus GN=Rpl18 PE=2 SV=3 - [RL18_MOUSE]	29.79	5	5	5	10	1.07	0.93	0.90	1.07	0.98	1.04	1.06	1.00	0.94	1.01	0.95	0.99	1.01	21.63	11.78
Q9D020-1	Isoform 1 of Cytosolic 5'-nucleotidase 3A OS=Mus musculus GN=Nt5c3a - [5NT3A_MOUSE]	13.13	2	3	3	6	0.98	0.96	0.98	1.08	1.11	1.03	1.05	1.00	1.18	0.96	0.99	1.10	1.13	33.77	5.69
Q9QZB9	Dynactin subunit 5 OS=Mus musculus GN=Dctn5 PE=1 SV=1 - [DCTN5_MOUSE]	6.04	1	1	1	1	1.14	1.08	0.95	0.97	0.85	0.92	0.80	1.00	0.88	0.94	0.83	0.93	0.82	20.13	8.02
Q8BVI4	Dihydropteridine reductase OS=Mus musculus GN=Qdpr PE=1 SV=2 - [DHPR_MOUSE]	50.62	4	8	8	36	0.99	0.93	1.01	1.02	1.04	1.07	1.07	1.00	1.03	1.10	1.07	1.07	1.15	25.55	7.81
F8VQK3	Protein Gucy1a2 OS=Mus musculus GN=Gucy1a2 PE=2 SV=1 - [F8VQK3_MOUSE]	11.10	1	7	7	11	0.94	1.21	1.13	1.19	1.32	0.89	0.92	1.00	1.10	1.03	1.05	1.11	1.10	81.78	6.87
Q8VDT9	39S ribosomal protein L50, mitochondrial OS=Mus musculus GN=Mrpl50 PE=2 SV=2 - [RM50_MOUSE]	37.74	1	4	4	15	1.06	0.86	0.78	0.97	0.90	0.99	0.96	1.00	0.98	0.98	0.83	0.92	1.02	18.20	9.33
O88520	Leucine-rich repeat protein SHOC-2 OS=Mus musculus GN=Shoc2 PE=2 SV=2 - [SHOC2_MOUSE]	3.61	1	2	2	4	1.09	1.40	1.29	1.13	1.04	1.04	0.95	1.00	0.92	1.14	1.05	1.28	1.18	64.85	8.46
Q6PJN8	DALR anticodon-binding domain-containing protein 3 OS=Mus musculus GN=Dalrd3 PE=2 SV=1 - [DALD3_MOUSE]	2.79	1	1	1	2	0.85	0.89	1.05	1.06	1.25	1.03	1.20	1.00	1.17	1.03	1.21	1.18	1.39	58.70	8.15
Q91VI7	Ribonuclease inhibitor OS=Mus musculus GN=Rnh1 PE=1 SV=1 - [RINI_MOUSE]	33.99	1	11	11	20	0.97	1.03	1.08	1.04	1.07	0.98	1.03	1.00	0.99	1.06	1.07	1.18	1.16	49.78	4.78

Q99MI1-2	Isoform 2 of ELKS/Rab6-interacting/CAST family member 1 OS=Mus musculus GN=Erc1 - [RB6I2_MOUSE]	43.75	2	2	44	164	0.93	1.06	1.03	0.84	0.83	1.33	1.12	1.00	0.99	0.98	0.90	0.95	0.93	111.86	6.49
A2A9I0	Golgi SNAP receptor complex member 2 OS=Mus musculus GN=Gosr2 PE=4 SV=1 - [A2A9I0_MOUSE]	16.36	2	2	2	3	0.86	1.01	1.17	0.98	1.15	1.00	1.16	1.00	1.17	0.97	1.13	1.00	1.16	19.56	9.50
Q6VN19	Ran-binding protein 10 OS=Mus musculus GN=Ranbp10 PE=1 SV=2 - [RBP10_MOUSE]	4.35	1	2	2	3	0.84	1.42	1.69	0.92	1.10	1.21	1.43	1.00	1.19	0.79	0.94	0.99	1.18	67.15	6.58
Q3UF75	Alpha-parvin OS=Mus musculus GN=Parva PE=2 SV=1 - [Q3UF75_MOUSE]	10.71	5	2	3	5	0.96	0.92	0.96	1.06	1.10	1.00	1.04	1.00	1.04	1.02	1.06	0.92	0.97	38.34	5.12
Q9R0K7	Plasma membrane calcium-transporting ATPase 2 OS=Mus musculus GN=Atp2b2 PE=1 SV=2 - [AT2B2_MOUSE]	44.32	2	33	48	353	0.92	1.02	1.11	1.09	1.17	0.96	1.03	1.00	1.05	0.92	1.03	0.85	0.96	132.50	5.96
Q9EP89	Serine beta-lactamase-like protein LACTB, mitochondrial OS=Mus musculus GN=Lactb PE=1 SV=1 - [LACTB_MOUSE]	21.23	1	8	8	13	0.95	0.98	1.04	1.01	1.10	0.91	1.13	1.00	1.02	1.06	1.13	1.00	1.09	60.67	8.90
D3Z7Q2	Uncharacterized protein OS=Mus musculus GN=Smim20 PE=2 SV=1 - [D3Z7Q2_MOUSE]	20.29	1	1	1	2	0.92	1.04	1.13	1.04	1.12	1.05	1.14	1.00	1.08	0.96	1.05	1.02	1.11	7.81	9.70
Q08642	Protein-arginine deiminase type-2 OS=Mus musculus GN=Padi2 PE=1 SV=2 - [PADI2_MOUSE]	12.63	1	7	7	15	1.07	1.37	1.17	1.25	1.13	0.99	1.07	1.00	0.97	1.08	0.94	1.13	1.14	76.20	5.53
E9Q9N6	Serine/threonine-protein kinase MARK2 OS=Mus musculus GN=Mark2 PE=2 SV=1 - [E9Q9N6_MOUSE]	21.50	18	7	12	24	0.98	0.89	0.99	1.06	1.06	1.00	1.01	1.00	1.04	1.01	1.02	0.96	0.99	79.04	9.64
Q8C0K8	Protein Uvrag OS=Mus musculus GN=Uvrag PE=2 SV=1 - [Q8C0K8_MOUSE]	3.01	1	1	1	2	0.82	1.80	2.19	1.20	1.46	1.07	1.30	1.00	1.22	0.96	1.17	0.99	1.21	77.48	7.97
O88587-2	Isoform Soluble of Catechol O-methyltransferase OS=Mus musculus GN=Comt - [COMT_MOUSE]	13.96	2	3	3	5	1.14	1.01	0.82	1.24	1.01	0.94	1.02	1.00	0.81	0.94	0.77	0.98	0.80	24.69	5.47

Q8BHS6	Armadillo repeat-containing X-linked protein 3 OS=Mus musculus GN=Armcx3 PE=1 SV=1 - [ARMX3_MOUSE]	5.01	1	1	1	2	0.98	1.14	1.16	1.11	1.12	1.21	1.22	1.00	1.02	1.26	1.28	1.36	1.38	42.59	8.68
Q8R1G1-2	Isoform 2 of Threonine aspartase 1 OS=Mus musculus GN=Tasp1 - [TASP1_MOUSE]	7.91	3	2	2	3	0.97	0.89	0.92	0.95	0.97	0.98	1.00	1.00	1.03	0.87	0.90	0.81	0.83	27.53	9.00
Q3TJD7	PDZ and LIM domain protein 7 OS=Mus musculus GN=Pdlim7 PE=2 SV=1 - [PDLI7_MOUSE]	10.07	7	4	4	5	1.02	0.92	0.94	0.97	0.96	0.71	0.78	1.00	0.96	1.08	1.07	0.79	0.84	50.09	8.47
F8WGE3	ATP-binding cassette sub-family A member 8-A OS=Mus musculus GN=Abca8a PE=2 SV=1 - [F8WGE3_MOUSE]	0.86	3	1	1	2	1.04	1.57	1.50	1.01	0.97	0.90	0.86	1.00	0.96	0.86	0.82	0.88	0.84	183.71	7.36
Q4KMS1	Tripartite motif-containing 44 OS=Mus musculus GN=Trim44 PE=2 SV=1 - [Q4KMS1_MOUSE]	19.13	4	4	4	9	0.83	0.81	0.97	0.75	0.96	1.08	1.30	1.00	1.25	1.01	1.28	0.89	1.12	38.11	4.18
Q9QXY6	EH domain-containing protein 3 OS=Mus musculus GN=Ehd3 PE=1 SV=2 - [EHD3_MOUSE]	61.12	1	15	25	91	0.92	0.96	1.04	1.03	1.08	0.98	1.04	1.00	1.05	0.99	1.07	0.99	1.06	60.78	6.46
Q9D819	Inorganic pyrophosphatase OS=Mus musculus GN=Ppa1 PE=1 SV=1 - [IPYR_MOUSE]	78.20	1	20	20	68	0.87	0.71	0.82	0.82	0.96	0.88	1.04	1.00	1.17	0.86	0.98	0.83	0.98	32.65	5.60
Q9ERS2	NADH dehydrogenase [ubiquinone] 1 alpha subcomplex subunit 13 OS=Mus musculus GN=Ndufa13 PE=1 SV=3 - [NDUAD_MOUSE]	53.47	1	7	7	31	1.04	1.04	1.01	1.14	1.12	1.07	1.05	1.00	1.06	1.15	1.12	1.04	1.08	16.85	9.48
G5E8K5-2	Isoform 2 of Ankyrin-3 OS=Mus musculus GN=Ank3 - [ANK3_MOUSE]	34.01	9	31	38	112	0.97	1.09	1.07	1.04	1.07	0.96	1.00	1.00	1.05	0.95	0.97	0.96	0.98	188.14	7.75
Q9QYI4	DnaJ homolog subfamily B member 12 OS=Mus musculus GN=Dnajb12 PE=2 SV=2 - [DJB12_MOUSE]	8.24	2	3	3	6	0.98	1.41	1.44	1.11	1.13	1.04	1.07	1.00	1.07	1.08	1.12	1.07	1.10	41.96	8.51
Q9Z127	Large neutral amino acids transporter small subunit 1 OS=Mus musculus GN=Slc7a5 PE=1 SV=2 - [LAT1_MOUSE]	11.52	1	4	4	11	0.97	1.20	1.23	1.02	1.07	0.87	0.97	1.00	0.99	0.94	0.97	0.89	0.90	55.84	7.90
P62911	60S ribosomal protein L32 OS=Mus musculus GN=Rpl32 PE=2 SV=2 - [RL32_MOUSE]	17.78	3	2	2	5	1.19	0.89	1.52	1.76	1.48	1.19	1.00	1.00	0.84	1.40	1.18	1.64	1.38	15.85	11.33

G3UXZ5	Proteasome activator complex subunit 1 (Fragment) OS=Mus musculus GN=Psme1 PE=2 SV=1 - [G3UXZ5_MOUSE]	36.55	5	9	9	36	1.04	0.84	0.77	0.85	0.82	1.07	1.00	1.00	0.96	0.99	0.96	1.07	1.00	27.39	5.60
O35926	Cyclin-dependent kinase 5 activator 2 OS=Mus musculus GN=Cdk5r2 PE=3 SV=2 - [CD5R2_MOUSE]	9.76	1	2	2	3	0.89	0.90	1.08	0.95	1.07	1.06	1.22	1.00	1.22	1.03	1.16	0.84	0.96	38.90	9.72
P34928	Apolipoprotein C-I OS=Mus musculus GN=Apoc1 PE=2 SV=1 - [APOC1_MOUSE]	10.23	1	1	1	2	0.67	0.95	1.42	0.54	0.80	0.73	1.09	1.00	1.50	1.81	2.72	1.10	1.65	9.69	9.09
Q8CAL5	Glypican-5 OS=Mus musculus GN=Gpc5 PE=2 SV=1 - [GPC5_MOUSE]	10.31	3	5	5	8	0.99	0.94	1.09	1.03	1.06	1.02	0.97	1.00	0.98	1.07	1.13	1.08	1.09	63.75	7.30
O70325-2	Isoform Cytoplasmic of Phospholipid hydroperoxide glutathione peroxidase, mitochondrial OS=Mus musculus GN=Gpx4 - [GPX41_MOUSE]	34.71	4	5	5	15	0.99	0.95	0.95	1.01	1.04	0.85	0.88	1.00	0.92	0.95	0.84	0.90	0.91	19.51	8.05
E9Q5Y2	Splicing factor U2AF 26 kDa subunit OS=Mus musculus GN=U2af1l4 PE=2 SV=1 - [E9Q5Y2_MOUSE]	14.92	7	1	2	5	1.00	0.97	0.97	0.77	0.77	0.78	0.78	1.00	1.00	1.01	1.02	1.03	1.03	21.33	8.98
Q9CPS6	Histidine triad nucleotide-binding protein 3 OS=Mus musculus GN=Hint3 PE=2 SV=1 - [HINT3_MOUSE]	23.64	3	3	3	8	0.90	0.84	0.97	1.03	1.15	0.93	1.02	1.00	1.11	1.02	1.03	1.01	1.14	18.78	6.33
F6XIS3	Mid1-interacting protein 1 (Fragment) OS=Mus musculus GN=Mid1ip1 PE=2 SV=1 - [F6XIS3_MOUSE]	17.65	2	1	1	2	0.93	1.12	1.20	0.94	1.01	1.11	1.19	1.00	1.08	1.22	1.31	1.06	1.14	5.85	4.55
E9Q8P5	PDZ and LIM domain protein 5 OS=Mus musculus GN=Pdlim5 PE=2 SV=2 - [E9Q8P5_MOUSE]	38.08	4	2	6	20	1.03	0.67	0.65	1.00	0.97	1.10	1.06	1.00	0.98	0.99	0.97	0.96	0.93	26.34	9.67
Q99KY4-	Isoform 2 of Cyclin-G-associated kinase OS=Mus musculus GN=Gak - [GAK_MOUSE]	10.19	4	9	11	34	1.01	1.09	0.92	1.05	0.96	1.08	0.95	1.00	1.01	1.00	0.91	1.00	0.95	138.26	5.96
Q6PAN7-	Isoform 2 of Proline-rich protein 18 OS=Mus musculus GN=Prr18 - [PRR18_MOUSE]	15.64	2	2	2	3	1.09	0.90	0.82	0.94	0.86	1.03	0.95	1.00	0.92	0.83	0.77	1.15	1.06	25.56	10.35
Q99JG2	Endothelin B receptor-like protein 2 OS=Mus musculus GN=Gpr37l1 PE=1 SV=2 - [ETBR2_MOUSE]	9.77	1	3	3	9	1.14	0.87	0.91	1.11	0.95	0.96	0.76	1.00	0.84	0.97	0.85	1.00	0.92	52.69	7.36

Q50H33-2	Isoform 2 of BTB/POZ domain-containing protein KCTD8 OS=Mus musculus GN=Kctd8 - [KCTD8_MOUSE]	9.83	3	1	3	9	0.93	1.05	1.09	0.99	0.98	0.86	0.94	1.00	1.10	0.93	1.04	0.85	0.82	37.96	8.22
Q9DCL8	Protein phosphatase inhibitor 2 OS=Mus musculus GN=Ppp1r2 PE=1 SV=3 - [IPP2_MOUSE]	69.42	6	10	10	80	1.06	0.72	0.66	0.92	0.86	1.09	1.03	1.00	0.99	1.04	0.97	0.99	0.97	23.11	4.83
Q9CXT8	Mitochondrial-processing peptidase subunit beta OS=Mus musculus GN=Pmpcb PE=2 SV=1 - [MPPB_MOUSE]	10.43	1	4	4	7	0.93	1.12	1.18	1.05	1.13	0.96	1.02	1.00	1.07	1.05	1.11	1.11	1.17	54.58	6.99
Q6NVF0-	Isoform 2 of Inositol polyphosphate 5-phosphatase OCRL-1 OS=Mus musculus GN=Ocrl - [OCRL_MOUSE]	13.20	2	7	7	12	1.08	1.17	1.03	1.10	1.02	1.11	1.01	1.00	0.95	1.18	1.06	1.13	1.00	83.96	6.60
Q9EQZ6-3	Isoform 3 of Rap guanine nucleotide exchange factor 4 OS=Mus musculus GN=Rapgef4 - [RPGF4_MOUSE]	12.39	7	8	8	15	1.11	1.14	1.17	1.08	1.03	0.97	1.03	1.00	0.99	0.96	0.93	1.09	1.02	113.42	6.83
Q9DBT3	Coiled-coil domain-containing protein 97 OS=Mus musculus GN=Ccdc97 PE=1 SV=1 - [CCD97_MOUSE]	17.65	1	3	3	6	0.81	1.05	1.29	0.66	0.81	0.94	1.39	1.00	1.30	1.06	1.31	0.92	1.13	38.70	4.60
O35286	Putative pre-mRNA-splicing factor ATP-dependent RNA helicase DHX15 OS=Mus musculus GN=Dhx15 PE=2 SV=2 - [DHX15_MOUSE]	18.74	1	11	11	36	0.99	1.04	1.08	1.08	1.08	0.99	0.99	1.00	1.01	1.00	0.99	1.08	1.12	90.95	7.46
Q6PE01	U5 small nuclear ribonucleoprotein 40 kDa protein OS=Mus musculus GN=Snrnp40 PE=2 SV=1 - [SNR40_MOUSE]	7.54	1	2	2	3	0.91	0.83	0.91	1.03	1.13	0.97	1.07	1.00	1.10	0.96	1.06	0.95	1.05	39.25	8.10
Q60817	Nascent polypeptide-associated complex subunit alpha OS=Mus musculus GN=Naca PE=1 SV=1 - [NACA_MOUSE]	35.81	2	4	4	31	1.02	0.89	0.95	0.90	0.90	0.93	0.91	1.00	1.00	0.87	0.86	0.85	0.92	23.37	4.56
E0CY60	Rho guanine nucleotide exchange factor 4 (Fragment) OS=Mus musculus GN=Arhgef4 PE=2 SV=1 - [E0CY60_MOUSE]	7.37	5	1	1	4	1.22	1.10	0.90	0.94	0.77	1.19	0.97	1.00	0.82	0.96	0.79	1.68	1.38	22.11	4.89

O70310	Glycylpeptide N-tetradecanoyltransferase 1 OS=Mus musculus GN=Nmt1 PE=1 SV=1 - [NMT1_MOUSE]	27.62	1	8	10	26	0.99	0.93	0.95	0.97	0.93	0.99	1.00	1.00	0.96	0.98	0.95	1.04	1.00	56.85	8.00
Q76KJ5	DNA-directed RNA polymerase I subunit RPA34 OS=Mus musculus GN=Cd3eap PE=1 SV=2 - [RPA34_MOUSE]	5.26	1	1	1	1	1.02	0.98	0.95	1.12	1.09	1.04	1.02	1.00	0.98	1.14	1.11	1.03	1.00	43.06	9.61
P42859	Huntingtin OS=Mus musculus GN=Htt PE=1 SV=2 - [HD_MOUSE]	4.97	3	11	11	21	1.01	1.25	1.22	1.10	1.12	0.89	0.91	1.01	0.94	1.04	1.02	1.10	1.05	344.47	6.29
Q9CQE3	28S ribosomal protein S17, mitochondrial OS=Mus musculus GN=Mrps17 PE=2 SV=1 - [RT17_MOUSE]	27.50	2	2	2	4	0.88	0.86	0.97	1.16	1.31	0.99	1.12	1.01	1.14	0.97	1.10	0.94	1.07	13.37	9.89
Q9D7X8	Gamma-glutamylcyclotransferase OS=Mus musculus GN=Ggct PE=2 SV=1 - [GGCT_MOUSE]	10.11	1	2	2	3	1.00	0.85	0.86	1.07	1.02	0.97	0.98	1.01	1.02	1.00	1.02	0.96	0.98	21.15	5.67
Q64332	Synapsin-2 OS=Mus musculus GN=Syn2 PE=1 SV=2 - [SYN2_MOUSE]	58.36	1	3	25	466	1.04	0.87	0.87	1.02	0.94	1.07	0.98	1.01	1.00	1.04	1.03	1.20	1.09	63.33	8.43
Q91YT7	Protein Ythdf2 OS=Mus musculus GN=Ythdf2 PE=2 SV=1 - [Q91YT7_MOUSE]	8.98	1	3	4	10	0.96	0.96	1.10	0.99	1.08	1.10	1.13	1.01	1.04	1.03	1.08	0.97	1.06	62.24	8.79
Q56A07	Sodium channel subunit beta-2 OS=Mus musculus GN=Scn2b PE=1 SV=1 - [SCN2B_MOUSE]	30.70	1	8	8	43	1.13	0.91	0.98	1.08	1.02	1.06	1.07	1.01	0.94	0.96	0.90	1.03	1.09	24.21	6.54
Q00896	Alpha-1-antitrypsin 1-3 OS=Mus musculus GN=Serpina1c PE=1 SV=2 - [A1AT3_MOUSE]	21.36	3	3	8	19	0.95	1.22	1.28	0.72	0.83	1.05	1.16	1.01	1.24	1.21	1.33	0.73	0.83	45.79	5.44
Q00560	Interleukin-6 receptor subunit beta OS=Mus musculus GN=Il6st PE=1 SV=2 - [IL6RB_MOUSE]	9.16	1	4	4	8	1.02	1.13	1.13	0.91	0.90	1.02	1.01	1.01	1.02	1.10	1.08	1.10	1.05	102.39	5.52
E9QJS0	28S ribosomal protein S10, mitochondrial OS=Mus musculus GN=Mrps10 PE=3 SV=1 - [E9QJS0_MOUSE]	11.25	5	1	1	2	1.06	1.01	0.95	1.08	1.02	0.94	0.88	1.01	0.95	0.89	0.84	0.89	0.85	18.69	8.13
E9Q9X4	Nitric oxide synthase OS=Mus musculus GN=Nos3 PE=2 SV=1 - [E9Q9X4_MOUSE]	1.63	2	1	1	1	1.22	1.25	1.02	1.10	0.90	0.88	0.71	1.01	0.82	1.04	0.86	1.16	0.95	122.02	7.52
P62852	40S ribosomal protein S25 OS=Mus musculus GN=Rps25 PE=2 SV=1 - [RS25_MOUSE]	24.00	1	4	4	7	0.98	0.71	0.70	1.04	0.95	1.06	0.96	1.01	1.01	1.02	1.00	0.86	0.88	13.73	10.11

P62918	60S ribosomal protein L8 OS=Mus musculus GN=Rpl8 PE=2 SV=2 - [RL8_MOUSE]	35.02	1	5	5	12	1.07	1.01	1.01	1.07	1.04	0.99	1.01	1.01	0.98	0.97	1.00	1.04	1.05	28.01	11.03
E9Q7E2	Protein Arid2 OS=Mus musculus GN=Arid2 PE=2 SV=1 - [E9Q7E2_MOUSE]	1.04	1	2	2	4	1.05	1.57	1.17	1.12	1.06	1.15	1.08	1.01	1.01	1.18	1.19	1.06	1.12	195.87	7.47
P10630	Eukaryotic initiation factor 4A-II OS=Mus musculus GN=Eif4a2 PE=2 SV=2 - [IF4A2_MOUSE]	51.35	6	9	18	80	1.01	0.93	0.91	1.16	1.17	1.00	0.99	1.01	0.98	0.99	0.98	1.08	0.98	46.37	5.48
B2RQS1	Striatin-3 OS=Mus musculus GN=Strn3 PE=2 SV=1 - [B2RQS1_MOUSE]	24.58	2	11	12	30	1.07	1.08	1.00	1.05	1.00	1.01	1.01	1.01	0.97	0.96	0.99	0.99	0.92	77.68	5.22
Q91YR5-2	Isoform 3 of Methyltransferase-like protein 13 OS=Mus musculus GN=Mettl13 - [MET13_MOUSE]	7.55	3	1	1	2	0.95	1.07	1.13	1.05	1.11	0.89	0.93	1.01	1.06	0.96	1.01	0.99	1.04	36.07	6.65
Q4VC33	Macrophage erythroblast attacher OS=Mus musculus GN=Maea PE=1 SV=1 - [MAEA_MOUSE]	5.56	2	2	2	8	0.96	1.25	1.20	1.01	1.06	0.95	0.93	1.01	0.96	0.90	0.89	1.00	1.07	45.31	8.69
Q3UHN9	Bifunctional heparan sulfate N-deacetylase/N-sulfotransferase 1 OS=Mus musculus GN=Ndst1 PE=1 SV=2 - [NDST1_MOUSE]	1.36	1	1	1	1	0.97	1.00	1.03	0.65	0.67	0.96	0.98	1.01	1.03	0.74	0.77	0.74	0.76	100.66	7.77
Q8R5M8-	Isoform 4 of Cell adhesion molecule 1 OS=Mus musculus GN=Cadm1 - [CADM1_MOUSE]	48.92	8	3	15	341	1.38	0.94	0.87	1.04	0.91	1.17	0.92	1.01	1.00	1.14	0.91	1.11	1.09	45.75	5.12
Q6P5F6	Zinc transporter ZIP10 OS=Mus musculus GN=Slc39a10 PE=1 SV=1 - [S39AA_MOUSE]	13.09	2	10	10	26	1.05	0.94	0.91	1.00	0.92	1.01	0.95	1.01	0.99	1.03	0.97	1.01	0.92	94.33	6.71
P22907	Porphobilinogen deaminase OS=Mus musculus GN=Hmbs PE=2 SV=2 - [HEM3_MOUSE]	15.79	2	4	4	6	1.01	0.92	0.98	0.98	0.97	1.05	1.06	1.01	0.96	1.05	1.04	1.09	1.05	39.32	6.87
Q9CQK7	RWD domain-containing protein 1 OS=Mus musculus GN=Rwdd1 PE=1 SV=1 - [RWDD1_MOUSE]	11.93	1	4	4	10	1.02	0.68	0.81	0.75	0.84	1.01	0.96	1.01	0.91	0.96	0.80	0.78	0.78	27.77	4.26
D3YZI9	Protein Pgbd5 OS=Mus musculus GN=Pgbd5 PE=2 SV=1 - [D3YZI9_MOUSE]	12.81	3	4	4	10	1.03	1.23	1.06	1.12	1.08	0.90	0.85	1.01	0.95	1.03	0.90	1.20	1.10	58.26	8.85
Q8BGZ1	Hippocalcin-like protein 4 OS=Mus musculus GN=Hpcal4 PE=2 SV=3 - [HPCL4_MOUSE]	83.25	2	11	14	156	1.11	0.85	0.80	0.94	0.84	1.03	0.92	1.01	0.91	0.99	0.93	1.10	1.00	22.20	4.89

P19783	Cytochrome c oxidase subunit 4 isoform 1, mitochondrial OS=Mus musculus GN=Cox4i1 PE=1 SV=2 - [COX41_MOUSE]	57.99	3	9	9	104	0.97	1.15	1.14	1.12	1.12	1.01	1.01	1.01	1.02	1.01	1.04	1.03	1.06	19.52	9.23
Q9R0P5	Destrin OS=Mus musculus GN=Dstn PE=1 SV=3 - [DEST_MOUSE]	73.33	1	12	13	72	0.85	0.75	0.90	0.74	0.87	0.87	1.00	1.01	1.22	0.81	0.97	0.86	1.03	18.51	7.97
P62500-2	Isoform 2 of TSC22 domain family protein 1 OS=Mus musculus GN=Tsc22d1 - [T22D1_MOUSE]	30.07	4	1	4	47	0.94	0.88	0.94	0.78	0.83	1.01	1.08	1.01	1.07	1.05	1.13	1.03	1.11	15.57	5.20
Q91ZX7	Prolow-density lipoprotein receptor-related protein 1 OS=Mus musculus GN=Lrp1 PE=1 SV=1 - [LRP1_MOUSE]	29.39	3	94	95	288	1.01	0.99	1.01	0.98	0.96	0.96	0.93	1.01	1.01	0.92	0.93	0.99	0.96	504.41	5.36
Q922S8	Kinesin-like protein KIF2C OS=Mus musculus GN=Kif2c PE=1 SV=1 - [KIF2C_MOUSE]	3.61	2	1	2	2	1.13	1.05	0.93	1.17	1.03	0.85	0.75	1.01	0.89	1.02	0.90	1.20	1.06	81.03	7.97
Q923Z0	G-protein coupled receptor family C group 5 member B OS=Mus musculus GN=Gprc5b PE=2 SV=1 - [GPC5B_MOUSE]	15.85	1	4	4	26	1.05	1.18	1.15	0.98	0.93	1.01	0.94	1.01	0.96	1.03	0.99	1.08	1.01	45.87	8.38
P09581	Macrophage colony-stimulating factor 1 receptor OS=Mus musculus GN=Csf1r PE=1 SV=3 - [CSF1R_MOUSE]	1.54	1	1	1	1	0.91	0.71	0.78	0.94	1.03	1.00	1.09	1.01	1.10	0.86	0.94	0.91	1.01	109.11	6.21
Q9DBC0	Selenoprotein O OS=Mus musculus GN=Selo PE=1 SV=4 - [SELO_MOUSE]	5.55	1	3	3	5	0.89	1.07	1.22	1.04	1.19	0.91	1.04	1.01	1.15	0.97	1.12	1.10	1.24	74.18	5.83
Q99MJ9	ATP-dependent RNA helicase DDX50 OS=Mus musculus GN=Ddx50 PE=2 SV=1 - [DDX50_MOUSE]	2.45	1	2	2	2	0.95	1.03	1.08	1.03	1.08	0.97	1.01	1.01	1.05	1.22	1.28	1.17	1.23	82.12	9.25
Q9CPQ3	Mitochondrial import receptor subunit TOM22 homolog OS=Mus musculus GN=Tomm22 PE=2 SV=3 - [TOM22_MOUSE]	47.18	1	4	4	7	1.08	1.14	1.01	1.10	1.06	0.91	0.95	1.01	1.02	0.97	0.98	1.07	1.04	15.53	4.34
P22437	Prostaglandin G/H synthase 1 OS=Mus musculus GN=Ptgs1 PE=2 SV=1 - [PGH1_MOUSE]	4.82	1	2	2	3	0.90	1.81	2.00	1.04	1.15	0.93	1.02	1.01	1.11	0.97	1.08	1.02	0.66	69.00	6.83
Q62101	Serine/threonine-protein kinase D1 OS=Mus musculus GN=Prkd1 PE=1 SV=2 - [KPCD1_MOUSE]	1.42	1	1	1	1	1.08	1.38	1.27	1.22	1.13	1.05	0.97	1.01	0.93	1.09	1.00	1.27	1.17	101.97	6.58

Q61749	Translation initiation factor eIF-2B subunit delta OS=Mus musculus GN=Eif2b4 PE=2 SV=2 - [EI2BD_MOUSE]	14.12	3	5	6	28	0.90	0.99	1.22	1.04	1.19	1.02	1.17	1.01	1.06	1.07	1.25	0.98	1.19	57.59	9.25
Q8K2M0	39S ribosomal protein L38, mitochondrial OS=Mus musculus GN=Mrpl38 PE=2 SV=2 - [RM38_MOUSE]	6.84	2	2	2	4	1.10	1.01	1.10	1.13	1.09	1.15	1.08	1.01	0.99	1.11	1.05	1.15	1.09	45.00	8.10
Q80VP9	Aspartate beta-hydroxylase domain-containing protein 2 OS=Mus musculus GN=Asphd2 PE=2 SV=1 - [ASPH2_MOUSE]	3.21	1	1	1	2	0.96	1.03	1.06	1.06	1.10	0.74	0.76	1.01	1.04	1.00	1.04	1.09	1.14	38.71	7.05
Q61418	H(+)/Cl(-) exchange transporter 4 OS=Mus musculus GN=Clcn4 PE=2 SV=2 - [CLCN4_MOUSE]	7.10	1	3	3	9	1.13	1.00	0.94	1.11	0.95	1.04	0.89	1.01	0.93	1.02	0.92	1.03	0.96	83.68	6.86
Q6PGB6-3	Isoform 3 of N-alpha-acetyltransferase 50 OS=Mus musculus GN=Naa50 - [NAA50_MOUSE]	25.58	5	3	3	5	1.02	0.83	0.79	1.02	0.96	1.03	1.00	1.01	0.99	0.93	0.97	0.89	0.84	14.89	8.56
D3Z2W1	Protein Dgki OS=Mus musculus GN=Dgki PE=2 SV=1 - [D3Z2W1_MOUSE]	18.38	3	11	11	21	1.01	1.07	1.08	0.97	1.02	1.00	1.02	1.01	1.03	0.99	1.04	1.10	1.13	115.89	7.85
F6Z458	Endoplasmic reticulum lectin 1 (Fragment) OS=Mus musculus GN=Erlec1 PE=2 SV=1 - [F6Z458_MOUSE]	9.66	2	1	1	1	0.99	1.65	1.66	1.28	1.29	1.24	1.25	1.01	1.01	1.00	1.01	1.03	1.04	27.25	7.49
Q3USC7	Ganglioside-induced differentiation-associated protein 1-like 1 OS=Mus musculus GN=Gdap1l1 PE=2 SV=1 - [Q3USC7_MOUSE]	28.61	3	6	7	25	1.02	1.01	0.96	1.07	1.07	1.04	0.99	1.01	0.96	0.90	0.95	1.07	1.01	41.89	6.48
Q9CX53	Gem-associated protein 6 OS=Mus musculus GN=Gemin6 PE=2 SV=2 - [GEMI6_MOUSE]	16.27	1	3	3	4	1.22	1.09	0.83	0.90	0.73	0.96	0.89	1.01	0.87	0.90	0.78	0.91	0.74	18.74	5.25
D3Z0F5	COP9 signalosome complex subunit 6 OS=Mus musculus GN=Cops6 PE=2 SV=1 - [D3Z0F5_MOUSE]	19.19	3	4	4	14	1.13	1.13	0.84	1.03	0.96	0.98	0.90	1.01	0.87	0.94	0.88	1.07	0.97	33.57	6.00
Q5SUH6	Clathrin interactor 1 OS=Mus musculus GN=Clint1 PE=4 SV=1 - [Q5SUH6_MOUSE]	29.80	4	16	16	63	1.07	0.86	0.81	0.95	0.88	1.00	0.92	1.01	0.94	0.95	0.89	0.88	0.80	69.71	6.65

Q6P5F7	Protein tweety homolog 3 OS=Mus musculus GN=Ttyh3 PE=1 SV=1 - [TTYH3_MOUSE]	10.31	2	4	4	6	1.04	1.15	1.08	1.06	1.07	0.97	0.93	1.01	1.02	1.07	1.08	0.99	1.01	57.68	5.85
Q9R0M6	Ras-related protein Rab-9A OS=Mus musculus GN=Rab9a PE=1 SV=1 - [RAB9A_MOUSE]	24.38	2	3	3	10	1.03	1.22	1.33	1.05	1.05	1.05	1.02	1.01	0.94	1.02	0.91	1.08	1.01	22.90	5.66
E9Q3E8	Pituitary adenylate cyclase-activating polypeptide type I receptor OS=Mus musculus GN=Adcyap1r1 PE=2 SV=1 - [E9Q3E8_MOUSE]	3.36	7	2	2	2	1.30	1.35	1.03	1.10	0.84	1.25	0.95	1.01	0.77	1.18	0.91	1.28	0.99	51.13	6.60
Q4JJC0	PAR3 splice variant (Fragment) OS=Mus musculus GN=Pard3 PE=2 SV=1 - [Q4JJC0_MOUSE]	3.32	19	1	1	5	1.20	1.07	0.89	1.11	0.92	1.25	1.03	1.01	0.83	1.32	1.10	1.32	1.09	46.09	9.06
A2AKI5	Integrin alpha-V light chain OS=Mus musculus GN=Itgav PE=2 SV=1 - [A2AKI5_MOUSE]	19.25	4	16	16	31	1.07	0.92	0.94	1.09	1.01	1.07	1.01	1.01	0.95	1.03	0.96	1.03	1.01	111.44	5.67
Q8BYI8	Uncharacterized protein KIAA1467 OS=Mus musculus GN=Kiaa1467 PE=2 SV=1 - [K1467_MOUSE]	10.90	2	4	4	7	0.94	0.94	0.99	1.00	1.00	1.11	1.10	1.01	1.04	0.97	1.10	0.97	1.03	66.99	5.03
Q8C7X2-	Isoform 2 of ER membrane protein complex subunit 1 OS=Mus musculus GN=Emc1 - [EMC1_MOUSE]	12.37	3	8	8	15	0.89	1.19	1.13	1.10	1.14	0.92	1.01	1.01	0.89	0.99	1.05	1.19	1.03	111.22	7.43
Q9JIS5	Synaptic vesicle glycoprotein 2A OS=Mus musculus GN=Sv2a PE=1 SV=1 - [SV2A_MOUSE]	24.39	1	15	15	232	1.00	1.20	1.17	1.07	1.12	0.92	0.91	1.01	0.99	0.96	1.00	0.98	0.97	82.59	5.57
Q8C7X8	Brain protein I3 OS=Mus musculus GN=Bri3 PE=4 SV=1 - [Q8C7X8_MOUSE]	16.00	3	1	1	1	1.19	1.20	1.01	1.31	1.10	0.95	0.79	1.01	0.85	1.08	0.91	1.11	0.94	11.06	8.32
Q8R554	OTU domain-containing protein 7A OS=Mus musculus GN=Otud7a PE=2 SV=1 - [OTU7A_MOUSE]	7.88	2	3	5	9	1.02	1.12	1.10	0.88	1.04	1.13	1.10	1.01	1.05	1.04	1.17	1.03	1.00	100.73	8.22
Q9D6F9	Tubulin beta-4A chain OS=Mus musculus GN=Tubb4a PE=1 SV=3 - [TBB4A_MOUSE]	75.68	1	6	27	1891	0.98	0.96	0.99	1.03	1.05	1.02	1.03	1.01	1.02	1.01	1.03	1.01	1.04	49.55	4.88

A2AHB7	Potassium channel subfamily T member 1 OS=Mus musculus GN=Kcnt1 PE=2 SV=1 - [A2AHB7_MOUSE]	4.19	6	3	3	8	0.96	1.12	1.16	0.96	1.00	0.94	0.96	1.01	1.21	1.05	1.17	1.01	1.11	137.27	7.46
P0C0A3	Charged multivesicular body protein 6 OS=Mus musculus GN=Chmp6 PE=2 SV=2 - [CHMP6_MOUSE]	22.50	3	3	4	10	1.02	0.96	1.02	1.01	1.07	0.91	0.90	1.01	1.06	0.97	1.05	0.89	0.96	23.40	5.44
O08810	116 kDa U5 small nuclear ribonucleoprotein component OS=Mus musculus GN=Eftud2 PE=2 SV=1 - [U5S1_MOUSE]	32.54	4	21	22	63	1.04	1.12	1.12	1.06	1.05	1.00	1.00	1.01	1.05	1.04	1.00	1.08	1.01	109.29	5.00
Q8BUV3	Gephyrin OS=Mus musculus GN=Gphn PE=1 SV=2 - [GEPH_MOUSE]	43.43	2	22	22	61	0.98	0.93	0.97	1.06	1.05	0.97	1.00	1.01	0.94	1.00	1.01	1.01	0.97	83.23	5.60
O55201	Transcription elongation factor SPT5 OS=Mus musculus GN=Supt5h PE=1 SV=1 - [SPT5H_MOUSE]	14.88	2	9	9	18	1.02	0.97	0.99	1.02	1.01	1.00	1.00	1.01	1.02	1.05	1.02	1.07	1.04	120.59	5.05
E9Q933	Transmembrane protein 11, mitochondrial OS=Mus musculus GN=Tmem11 PE=2 SV=1 - [E9Q933_MOUSE]	4.55	2	1	1	2	1.08	1.42	1.32	1.53	1.42	1.01	0.93	1.01	0.94	1.11	1.03	0.96	0.90	19.80	6.25
Q8K3H0	DCC-interacting protein 13-alpha OS=Mus musculus GN=Appl1 PE=1 SV=1 - [DP13A_MOUSE]	29.28	1	15	16	42	1.00	1.10	1.11	1.02	1.01	0.97	0.99	1.01	1.00	0.98	1.03	1.06	1.06	79.28	5.41
Q8BUY5	Translocase of inner mitochondrial membrane domain-containing protein 1 OS=Mus musculus GN=Timmdc1 PE=2 SV=1 - [TIDC1_MOUSE]	5.26	1	1	1	1	0.98	1.09	1.11	0.95	0.96	1.05	1.06	1.01	1.03	0.93	0.95	1.11	1.13	31.77	9.29
Q8VE52	Opioid growth factor receptor-like protein 1 OS=Mus musculus GN=Ogfrl1 PE=2 SV=2 - [OGRL1_MOUSE]	7.11	1	3	3	3	0.97	1.10	1.13	1.09	1.11	1.01	1.02	1.01	1.04	1.02	1.04	1.07	1.09	52.24	6.57
Q8R310-2	Isoform 2 of Transmembrane and coiled-coil domains protein 3 OS=Mus musculus GN=Tmcc3 - [TMCC3_MOUSE]	2.69	2	1	1	2	1.17	1.20	1.02	1.17	0.99	1.05	0.89	1.01	0.86	1.27	1.08	1.17	0.99	50.14	8.56

Q56A08	G patch domain and KOW motifs-containing protein OS=Mus musculus GN=Gpkow PE=2 SV=2 - [GPKOW_MOUSE]	1.84	1	1	1	2	0.98	1.20	1.22	0.93	0.94	1.16	1.18	1.01	1.03	0.96	0.98	0.87	0.89	53.80	8.07
Q05D44	Eukaryotic translation initiation factor 5B OS=Mus musculus GN=Eif5b PE=1 SV=2 - [IF2P_MOUSE]	14.72	1	13	13	32	1.04	0.91	0.87	1.04	0.99	1.03	0.96	1.01	0.96	1.00	0.98	1.03	0.96	137.53	5.59
G3UXX3	Sepiapterin reductase OS=Mus musculus GN=Spr PE=2 SV=1 - [G3UXX3_MOUSE]	51.14	5	9	9	31	0.99	1.05	0.94	1.09	1.07	1.02	1.02	1.01	1.01	1.11	1.14	1.13	1.19	23.34	6.21
P58158	Galactosylgalactosylxylosylprotein 3-beta-glucuronosyltransferase 3 OS=Mus musculus GN=B3gat3 PE=2 SV=1 - [B3GA3_MOUSE]	4.18	1	1	1	2	1.01	1.35	1.33	1.39	1.37	0.98	0.96	1.01	0.99	0.88	0.87	1.03	1.02	37.04	8.78
H3BKM2	Nuclear-interacting partner of ALK OS=Mus musculus GN=Zc3hc1 PE=2 SV=1 - [H3BKM2_MOUSE]	8.28	4	2	2	4	0.87	0.99	1.13	1.12	1.28	1.05	1.20	1.01	1.15	1.04	1.19	1.03	1.18	50.48	5.50
O55003	BCL2/adenovirus E1B 19 kDa protein-interacting protein 3 OS=Mus musculus GN=Bnip3 PE=1 SV=1 - [BNIP3_MOUSE]	3.74	1	1	1	1	1.08	0.89	0.83	0.94	0.88	1.02	0.94	1.01	0.94	0.84	0.78	0.88	0.82	20.97	7.14
Q3U6N9-2	Isoform 2 of UPF0488 protein C8orf33 homolog OS=Mus musculus - [CH033_MOUSE]	10.06	3	1	1	2	1.03	1.11	1.07	0.93	0.90	0.97	0.93	1.01	0.98	1.05	1.02	0.95	0.92	17.99	10.24
Q8C0T5	Signal-induced proliferation-associated 1-like protein 1 OS=Mus musculus GN=Sipa1l1 PE=1 SV=2 - [SI1L1_MOUSE]	19.42	4	24	25	75	0.96	0.95	1.01	0.98	1.02	1.00	1.07	1.01	1.07	0.96	1.03	0.94	0.98	196.91	8.13
P81117	Nucleobindin-2 OS=Mus musculus GN=Nucb2 PE=1 SV=2 - [NUCB2_MOUSE]	50.95	1	15	15	55	1.05	0.87	0.84	0.92	0.84	1.14	1.07	1.01	0.97	1.03	0.97	0.92	0.86	50.27	5.15
Q3UN90	LYR motif-containing protein 9 OS=Mus musculus GN=Lyrm9 PE=2 SV=1 - [LYRM9_MOUSE]	32.05	2	2	2	5	1.06	0.78	0.77	0.92	0.85	1.05	0.97	1.01	0.93	1.00	0.92	0.94	0.88	9.30	8.91
Q8BYL4	Tyrosine--tRNA ligase, mitochondrial OS=Mus musculus GN=Yars2 PE=2 SV=2 - [SYYM_MOUSE]	1.48	1	1	1	3	1.02	1.26	1.23	1.00	0.98	1.01	0.98	1.01	0.98	1.20	1.18	1.40	1.37	52.56	9.16

P60229	Eukaryotic translation initiation factor 3 subunit E OS=Mus musculus GN=Eif3e PE=1 SV=1 - [EIF3E_MOUSE]	16.40	1	5	5	7	0.92	1.09	1.59	1.01	1.05	0.96	1.13	1.01	1.10	0.82	1.05	1.13	1.23	52.19	6.04
Q9CQQ8	U6 snRNA-associated Sm-like protein LSm7 OS=Mus musculus GN=Lsm7 PE=3 SV=1 - [LSM7_MOUSE]	46.60	1	4	5	15	0.98	0.76	0.76	0.91	0.91	1.02	0.96	1.01	1.03	0.91	0.97	0.95	1.01	11.63	5.27
Q99J36	THUMP domain-containing protein 1 OS=Mus musculus GN=Thumpd1 PE=1 SV=1 - [THUM1_MOUSE]	6.29	1	3	3	8	1.07	0.92	0.85	1.01	0.95	1.08	0.98	1.01	0.89	0.98	0.88	0.99	1.00	38.86	6.07
Q80ZI6	E3 ubiquitin-protein ligase LRSAM1 OS=Mus musculus GN=Lrsam1 PE=2 SV=1 - [LRSM1_MOUSE]	6.60	1	4	4	7	1.20	1.09	1.11	1.09	1.02	1.02	1.06	1.01	0.72	1.09	0.90	1.12	0.83	83.92	6.14
Q8VDL4-	Isoform 3 of ADP-dependent glucokinase OS=Mus musculus GN=Adpgk - [ADPGK_MOUSE]	6.46	3	2	2	4	1.12	1.46	1.30	1.13	1.01	1.04	0.93	1.01	0.90	1.22	1.09	1.26	1.13	53.74	5.62
P50580	Proliferation-associated protein 2G4 OS=Mus musculus GN=Pa2g4 PE=1 SV=3 - [PA2G4_MOUSE]	45.94	2	14	14	45	0.95	0.98	1.08	1.05	1.09	1.01	1.04	1.01	1.06	0.96	1.00	0.95	1.00	43.67	6.86
P47911	60S ribosomal protein L6 OS=Mus musculus GN=Rpl6 PE=1 SV=3 - [RL6_MOUSE]	26.35	2	7	7	19	0.94	0.85	0.94	0.97	1.04	1.00	1.12	1.01	1.13	1.01	1.06	0.93	1.00	33.49	10.70
Q78RX3	Small integral membrane protein 12 OS=Mus musculus GN=Smim12 PE=3 SV=1 - [SIM12_MOUSE]	18.48	1	2	2	3	1.11	0.63	0.56	1.04	0.93	1.09	0.98	1.01	0.90	0.95	0.85	0.74	0.66	10.79	9.04
A2ADA6	Protein Acap3 OS=Mus musculus GN=Acap3 PE=2 SV=1 - [A2ADA6_MOUSE]	3.74	2	1	2	2	1.62	0.77	0.47	1.48	0.91	0.92	0.57	1.01	0.62	1.07	0.66	0.96	0.60	92.28	6.02
Q8BTU1	UPF0468 protein C16orf80 homolog OS=Mus musculus GN=Gtl3 PE=2 SV=1 - [CP080_MOUSE]	6.22	1	1	1	2	1.14	1.25	1.09	1.01	0.88	1.29	1.12	1.01	0.88	1.15	1.01	1.09	0.95	22.73	9.76
Q5XG73-2	Isoform 2 of Acyl-CoA-binding domain-containing protein 5 OS=Mus musculus GN=Acbd5 - [ACBD5_MOUSE]	6.36	4	3	3	4	0.96	1.29	1.34	1.00	1.02	0.98	1.06	1.01	1.05	1.02	1.07	1.08	1.12	52.34	5.29
F6S7W6	Serine/threonine-protein kinase SIK3 (Fragment) OS=Mus musculus GN=Sik3 PE=2 SV=1 - [F6S7W6_MOUSE]	11.52	7	10	10	24	0.96	1.11	1.01	1.08	1.11	1.02	1.00	1.01	1.03	1.07	1.06	1.04	1.06	144.70	6.81

Q9JKR6	Hypoxia up-regulated protein 1 OS=Mus musculus GN=Hyou1 PE=1 SV=1 - [HYOU1_MOUSE]	37.74	3	28	28	79	1.03	1.06	0.99	1.04	1.06	1.05	1.04	1.01	1.02	1.05	1.02	1.05	1.07	111.11	5.19
G3UWG2	T-cell lymphoma invasion and metastasis 1 OS=Mus musculus GN=Tiam1 PE=4 SV=1 - [G3UWG2_MOUSE]	3.46	4	4	4	7	1.39	1.17	0.85	0.89	0.73	1.07	0.80	1.01	0.84	1.10	0.85	1.02	0.81	177.34	6.64
Q9D9M2-	Isoform 2 of Ubiquitin carboxyl-terminal hydrolase 12 OS=Mus musculus GN=Usp12 - [UBP12_MOUSE]	11.60	2	1	2	5	1.34	0.68	0.50	1.23	0.91	1.17	0.87	1.01	0.75	1.06	0.79	1.17	0.87	29.14	5.40
P63141	Potassium voltage-gated channel subfamily A member 2 OS=Mus musculus GN=Kcna2 PE=1 SV=1 - [KCNA2_MOUSE]	23.85	3	5	9	46	1.09	1.16	1.07	1.15	1.06	0.99	0.90	1.01	0.92	0.88	0.83	0.94	0.87	56.66	4.86
P70232	Neural cell adhesion molecule L1-like protein OS=Mus musculus GN=Chl1 PE=1 SV=2 - [CHL1_MOUSE]	28.29	2	27	27	73	1.10	0.92	0.76	0.98	0.86	1.06	0.94	1.01	0.92	1.00	0.89	1.03	0.90	134.99	5.57
Q8BKY8	mTERF domain-containing protein 3, mitochondrial OS=Mus musculus GN=Mterfd3 PE=1 SV=1 - [MTER3_MOUSE]	3.38	1	1	1	2	0.84	1.05	1.25	1.10	1.30	0.97	1.15	1.01	1.20	1.17	1.39	1.03	1.23	43.43	8.94
Q91WQ3	Tyrosine--tRNA ligase, cytoplasmic OS=Mus musculus GN=Yars PE=2 SV=3 - [SYYC_MOUSE]	45.83	3	21	21	43	0.96	0.97	1.05	1.01	1.06	1.01	1.04	1.01	1.06	0.98	1.04	0.99	1.05	59.07	7.01
Q9D920	Loss of heterozygosity 12 chromosomal region 1 protein homolog OS=Mus musculus GN=Loh12cr1 PE=2 SV=1 - [L12R1_MOUSE]	6.15	2	1	1	2	1.05	0.97	0.92	1.06	1.00	1.07	1.01	1.01	0.96	0.94	0.89	1.06	1.01	22.11	6.55
Q99NB8	Ubiquilin-4 OS=Mus musculus GN=Ubqln4 PE=1 SV=1 - [UBQL4_MOUSE]	25.50	2	8	10	62	1.04	0.94	0.96	0.91	0.90	1.08	1.03	1.01	0.99	0.97	0.98	0.93	0.94	63.47	5.03
J3QP43	Protein 6330403A02Rik OS=Mus musculus GN=6330403A02Rik PE=4 SV=1 - [J3QP43_MOUSE]	21.28	2	2	2	57	1.07	1.08	0.94	1.08	0.97	1.14	1.06	1.01	0.93	1.10	1.04	1.20	1.12	15.01	6.51
Q9DAM7	UPF0444 transmembrane protein C12orf23 homolog OS=Mus musculus PE=2 SV=1 - [CL023_MOUSE]	17.39	1	1	1	2	1.36	0.85	0.62	1.30	0.95	1.32	0.97	1.01	0.74	1.21	0.89	1.19	0.88	11.54	9.32

Q6PGA0-	Isoform 2 of REST corepressor 3 OS=Mus musculus GN=Rcor3 - [RCOR3_MOUSE]	6.68	4	2	2	3	0.99	1.10	1.10	0.95	0.96	1.00	1.00	1.01	1.01	0.99	0.99	1.03	1.04	47.44	6.80
Q9CWP8	DNA polymerase delta subunit 4 OS=Mus musculus GN=Pold4 PE=2 SV=1 - [DPOD4_MOUSE]	9.35	1	1	1	2	0.93	0.68	0.73	0.91	0.97	0.89	0.95	1.01	1.08	1.03	1.11	1.01	1.09	12.40	6.52
Q9CQZ6	NADH dehydrogenase [ubiquinone] 1 beta subcomplex subunit 3 OS=Mus musculus GN=Ndufb3 PE=1 SV=1 - [NDUB3_MOUSE]	41.35	1	4	4	10	1.01	1.03	0.97	0.94	0.99	1.13	1.11	1.01	1.01	1.00	1.05	0.93	0.97	11.68	9.04
Q9WU79	Proline dehydrogenase 1, mitochondrial OS=Mus musculus GN=Prodh PE=2 SV=2 - [PROD_MOUSE]	10.18	4	4	4	6	1.00	0.91	1.03	1.26	1.23	0.91	0.90	1.01	1.01	1.04	1.02	0.97	0.99	67.99	8.24
Q8C3Q5-	Isoform 2 of Protein shisa-7 OS=Mus musculus GN=Shisa7 - [SHSA7_MOUSE]	24.40	3	9	9	35	1.06	1.01	0.98	0.99	0.96	1.11	1.03	1.01	0.96	1.03	1.02	1.00	1.02	56.39	9.86
P23475	X-ray repair cross-complementing protein 6 OS=Mus musculus GN=Xrcc6 PE=1 SV=5 - [XRCC6_MOUSE]	2.30	1	1	1	2	0.91	0.91	1.00	0.92	1.01	1.11	1.22	1.01	1.11	0.99	1.09	1.03	1.13	69.44	6.79
Q8VH51-	Isoform 3 of RNA-binding protein 39 OS=Mus musculus GN=Rbm39 - [RBM39_MOUSE]	15.26	6	4	4	7	0.89	0.94	1.11	1.15	1.20	0.97	1.03	1.01	1.09	1.02	1.23	1.01	1.19	39.79	6.55
Q9CXS4	Centromere protein V OS=Mus musculus GN=Cenpv PE=2 SV=2 - [CENPV_MOUSE]	60.32	3	10	10	26	0.89	0.90	1.07	0.94	1.09	0.97	1.10	1.01	1.16	0.97	1.11	0.92	1.08	27.52	9.79
P48036	Annexin A5 OS=Mus musculus GN=Anxa5 PE=1 SV=1 - [ANXA5_MOUSE]	57.99	1	16	16	53	1.00	1.06	1.02	1.03	1.02	0.98	0.96	1.01	0.99	1.04	1.05	1.05	1.03	35.73	4.96
E9Q3N1	High affinity cationic amino acid transporter 1 (Fragment) OS=Mus musculus GN=Slc7a1 PE=2 SV=1 - [E9Q3N1_MOUSE]	5.34	2	1	1	2	0.83	0.88	1.06	0.85	1.03	0.80	0.96	1.01	1.22	0.85	1.03	0.71	0.86	58.10	6.99
Q8VDD5	Myosin-9 OS=Mus musculus GN=Myh9 PE=1 SV=4 - [MYH9_MOUSE]	41.07	2	55	70	267	0.94	0.89	0.98	1.04	1.14	0.99	1.06	1.01	1.08	1.04	1.12	1.01	1.11	226.23	5.66
D3Z3A7	Low-density lipoprotein receptor-related protein 11 (Fragment) OS=Mus musculus GN=Lrp11 PE=2 SV=1 - [D3Z3A7_MOUSE]	7.02	4	2	2	4	1.06	0.75	0.70	0.94	0.88	0.82	0.77	1.01	0.95	0.72	0.68	0.94	0.89	44.36	7.66

Q6PDJ6	F-box only protein 42 OS=Mus musculus GN=Fbxo42 PE=1 SV=1 - [FBX42_MOUSE]	1.67	1	1	1	2	1.06	1.09	1.02	1.05	0.99	1.04	0.98	1.01	0.95	1.01	0.95	1.21	1.14	77.73	7.58
G3UYG6	PERQ amino acid-rich with GYF domain-containing protein 2 (Fragment) OS=Mus musculus GN=Gigyf2 PE=2 SV=1 - [G3UYG6_MOUSE]	16.26	12	14	14	29	0.97	1.00	1.04	0.92	0.93	0.88	0.90	1.01	0.97	0.90	0.91	0.89	0.92	148.49	5.57
Q6PDY2	2-aminoethanethiol dioxygenase OS=Mus musculus GN=Ado PE=1 SV=2 - [AEDO_MOUSE]	28.91	1	5	5	16	0.98	1.12	1.25	0.96	1.06	0.99	1.10	1.01	1.13	1.00	1.07	1.00	1.12	28.35	5.97
Q9D0A3	UPF0552 protein C15orf38 homolog OS=Mus musculus PE=2 SV=1 - [CO038_MOUSE]	49.56	1	10	10	86	1.03	0.96	0.90	0.93	0.87	1.07	1.01	1.01	1.01	0.95	0.93	0.92	0.88	25.18	5.19
Q9WTR2	Mitogen-activated protein kinase kinase kinase 6 OS=Mus musculus GN=Map3k6 PE=1 SV=4 - [M3K6_MOUSE]	1.08	1	1	1	1	1.21	1.86	1.54	1.37	1.13	1.02	0.84	1.01	0.84	1.11	0.92	1.10	0.92	142.96	6.68
Q9D8Z2	TP53-regulated inhibitor of apoptosis 1 OS=Mus musculus GN=Triap1 PE=2 SV=1 - [TRIA1_MOUSE]	55.26	1	4	4	16	0.99	0.68	0.65	0.95	0.97	1.07	1.09	1.01	1.07	0.99	0.97	0.93	1.00	8.75	5.48
Q9EQG9-	Isoform 2 of Collagen type IV alpha-3-binding protein OS=Mus musculus GN=Col4a3bp - [C43BP_MOUSE]	11.87	3	6	6	11	1.00	0.98	0.98	0.99	0.99	1.10	0.97	1.01	0.93	0.99	0.92	1.04	1.04	68.24	5.43
Q3UW66	Sulfurtransferase OS=Mus musculus GN=Mpst PE=2 SV=1 - [Q3UW66_MOUSE]	27.27	2	6	6	16	0.93	1.14	1.09	1.00	1.01	1.00	1.04	1.01	1.05	0.99	1.05	0.98	0.98	33.08	6.47
P32883-2	Isoform 2B of GTPase KRas OS=Mus musculus GN=Kras - [RASK_MOUSE]	62.23	5	4	9	39	1.08	0.95	0.84	1.12	0.99	1.07	0.95	1.01	0.93	1.03	0.93	1.01	0.78	21.47	8.12
Q9CXE2	B-cell CLL/lymphoma 7 protein family member A OS=Mus musculus GN=Bcl7a PE=2 SV=1 - [BCL7A_MOUSE]	26.67	3	2	4	15	1.02	1.38	1.54	0.95	1.09	1.09	1.10	1.01	1.20	1.00	1.00	1.07	1.27	22.77	5.06
Q62018-3	Isoform 3 of RNA polymerase-associated protein CTR9 homolog OS=Mus musculus GN=Ctr9 - [CTR9_MOUSE]	3.03	2	2	2	2	0.98	0.91	0.93	1.42	1.13	1.07	1.09	1.01	1.03	1.07	1.10	1.07	1.09	123.79	6.46

Q7TSH2	Phosphorylase b kinase regulatory subunit beta OS=Mus musculus GN=Phkb PE=1 SV=1 - [KPBB_MOUSE]	2.86	1	2	2	3	0.98	1.28	1.31	1.09	1.12	1.09	1.11	1.01	1.04	1.13	1.16	0.87	0.90	123.81	6.83
Q9WUN2	Serine/threonine-protein kinase TBK1 OS=Mus musculus GN=Tbk1 PE=1 SV=1 - [TBK1_MOUSE]	3.16	1	2	2	4	1.17	1.39	1.19	1.13	0.97	1.09	0.93	1.01	0.87	1.08	0.93	1.12	0.96	83.37	6.87
Q9EQI8	39S ribosomal protein L46, mitochondrial OS=Mus musculus GN=Mrpl46 PE=2 SV=1 - [RM46_MOUSE]	9.89	1	2	2	4	0.86	0.96	1.11	0.83	0.96	0.84	0.97	1.01	1.17	0.89	1.03	1.14	1.32	32.11	7.40
Q8K4I3	Rho guanine nucleotide exchange factor 6 OS=Mus musculus GN=Arhgef6 PE=1 SV=1 - [ARHG6_MOUSE]	4.15	4	1	3	5	1.07	0.91	0.85	1.16	1.08	0.88	0.81	1.01	0.94	0.98	0.91	1.09	1.01	87.00	5.87
Q6P9R2	Serine/threonine-protein kinase OSR1 OS=Mus musculus GN=Oxsr1 PE=1 SV=1 - [OXSR1_MOUSE]	14.80	4	3	6	11	0.94	0.98	1.08	1.09	1.00	0.95	0.95	1.01	0.92	1.07	1.03	1.01	1.04	58.18	6.43
Q8K4P8-4	Isoform 4 of E3 ubiquitin-protein ligase HECW1 OS=Mus musculus GN=Hecw1 - [HECW1_MOUSE]	4.13	4	1	1	5	1.09	1.31	1.25	1.31	1.20	0.94	0.86	1.01	0.93	0.88	0.81	1.06	0.97	35.45	9.26
Q6DFY2	Opioid binding protein/cell adhesion molecule-like OS=Mus musculus GN=Opcml PE=2 SV=1 - [Q6DFY2_MOUSE]	55.19	1	2	19	316	0.98	0.90	0.88	1.00	0.98	1.05	1.04	1.01	1.05	0.92	0.94	0.92	0.89	37.13	6.68
P55066	Neurocan core protein OS=Mus musculus GN=Ncan PE=2 SV=1 - [NCAN_MOUSE]	28.31	1	29	30	190	1.01	0.98	0.99	0.94	0.95	0.98	1.00	1.01	0.98	0.97	0.96	0.92	0.93	137.11	5.72
Q60673	Receptor-type tyrosine-protein phosphatase-like N OS=Mus musculus GN=Ptprn PE=2 SV=2 - [PTPRN_MOUSE]	13.38	2	8	8	19	1.25	1.23	0.98	0.99	0.90	1.27	0.79	1.01	0.72	1.26	0.99	1.47	1.01	106.02	7.09
Q14CH7	Alanine--tRNA ligase, mitochondrial OS=Mus musculus GN=Aars2 PE=2 SV=1 - [SYAM_MOUSE]	2.24	1	2	2	3	0.98	1.47	1.49	1.10	1.12	1.03	1.04	1.02	1.03	1.00	1.02	1.08	1.10	106.72	6.18
Q3TQZ7	Mitogen-activated protein kinase 10 OS=Mus musculus GN=Mapk10 PE=2 SV=1 - [Q3TQZ7_MOUSE]	35.17	19	4	12	24	1.25	0.99	0.94	1.33	0.87	1.14	0.93	1.02	0.93	0.95	0.91	0.97	1.00	47.97	7.69

A2RT62	F-box/LRR-repeat protein 16 OS=Mus musculus GN=Fbxl16 PE=2 SV=1 - [FXL16_MOUSE]	15.03	1	5	5	24	0.98	1.05	1.12	0.96	1.05	1.00	1.06	1.02	1.09	0.92	1.08	0.99	1.06	51.84	6.60
Q9CZJ6	Protein Mis18-alpha OS=Mus musculus GN=Mis18a PE=2 SV=1 - [MS18A_MOUSE]	7.35	1	1	1	1	0.91	1.34	1.47	0.95	1.04	1.02	1.11	1.02	1.11	0.96	1.06	1.02	1.12	22.93	5.27
A2RSQ0	DENN domain-containing protein 5B OS=Mus musculus GN=Dennd5b PE=1 SV=2 - [DEN5B_MOUSE]	1.18	1	1	1	2	0.97	1.08	1.12	1.20	1.24	0.88	0.91	1.02	1.05	1.16	1.20	1.39	1.44	144.54	6.68
Q99N96	39S ribosomal protein L1, mitochondrial OS=Mus musculus GN=Mrpl1 PE=1 SV=2 - [RM01_MOUSE]	11.31	2	3	3	5	1.01	0.94	0.97	0.99	1.01	1.06	1.08	1.02	1.02	0.98	1.00	1.06	1.09	37.57	8.72
Q8BQU6	Gap junction gamma-2 protein OS=Mus musculus GN=Gjc2 PE=1 SV=2 - [CXG2_MOUSE]	7.73	1	1	1	9	0.76	0.99	1.30	1.06	1.38	0.89	1.17	1.02	1.34	1.14	1.50	0.85	1.11	46.98	7.12
Q3U0D9-2	Isoform 2 of E3 ubiquitin-protein ligase HACE1 OS=Mus musculus GN=Hace1 - [HACE1_MOUSE]	6.21	6	4	4	8	0.98	1.21	1.17	1.09	1.08	1.08	0.86	1.02	0.81	0.93	0.84	1.16	1.06	94.51	5.60
P47791-2	Isoform Cytoplasmic of Glutathione reductase, mitochondrial OS=Mus musculus GN=Gsr - [GSHR_MOUSE]	51.27	2	14	14	65	1.01	0.89	0.92	0.98	0.91	0.95	0.86	1.02	0.96	0.96	0.93	1.01	1.05	51.04	7.39
Q8BTV1	Tumor suppressor candidate 3 OS=Mus musculus GN=Tusc3 PE=2 SV=1 - [TUSC3_MOUSE]	6.34	1	2	2	3	1.11	1.37	1.23	1.15	1.03	0.99	0.89	1.02	0.91	1.07	0.97	1.21	1.09	39.52	10.11
Q8K341-4	Isoform 4 of Alpha-tubulin N-acetyltransferase OS=Mus musculus GN=Atat1 - [ATAT_MOUSE]	26.73	9	7	7	14	0.92	1.01	1.09	0.98	1.08	0.97	1.03	1.02	0.99	1.04	1.11	1.01	1.03	37.50	10.10
P23927	Alpha-crystallin B chain OS=Mus musculus GN=Cryab PE=1 SV=2 - [CRYAB_MOUSE]	50.86	1	7	7	23	0.94	0.80	0.79	0.96	1.01	1.08	1.10	1.02	1.10	1.04	1.05	0.95	1.01	20.06	7.33
Q8R464	Cell adhesion molecule 4 OS=Mus musculus GN=Cadm4 PE=1 SV=1 - [CADM4_MOUSE]	35.57	1	12	12	97	0.98	0.95	1.02	0.91	0.95	0.99	1.03	1.02	1.03	0.97	1.01	0.95	1.00	42.70	6.30
E0CYE2	Inosine-5'-monophosphate dehydrogenase OS=Mus musculus GN=Impdh1 PE=2 SV=1 - [E0CYE2_MOUSE]	10.02	5	2	4	5	0.99	1.05	1.07	1.07	1.08	0.93	0.94	1.02	1.03	1.00	1.02	1.18	1.20	52.44	6.55

Q7TSS2-2	Isoform 2 of Ubiquitin-conjugating enzyme E2 Q1 OS=Mus musculus GN=Ube2q1 - [UB2Q1_MOUSE]	9.09	5	2	2	3	1.00	0.96	0.96	1.10	1.10	1.01	1.01	1.02	1.02	0.98	0.99	1.01	1.01	28.47	4.74
O08692	Myeloid bactenecin (F1) OS=Mus musculus GN=Ngp PE=2 SV=1 - [O08692_MOUSE]	19.16	1	2	2	5	0.80	0.81	0.92	0.99	1.08	1.10	1.26	1.02	1.13	1.15	1.43	1.46	1.62	19.32	5.31
D3Z6C3	40S ribosomal protein S3a OS=Mus musculus GN=Rps3a2 PE=3 SV=1 - [D3Z6C3_MOUSE]	45.83	3	13	13	39	0.96	0.96	0.95	0.95	0.99	1.01	1.04	1.02	1.03	0.95	0.99	0.95	0.97	29.81	9.73
Q3TB04	FERM domain-containing protein 4A OS=Mus musculus GN=Frmd4a PE=2 SV=1 - [Q3TB04_MOUSE]	8.96	6	4	4	7	1.00	1.28	1.29	1.09	1.04	1.02	1.03	1.02	1.02	1.11	1.12	1.12	1.14	80.74	9.20
P62267	40S ribosomal protein S23 OS=Mus musculus GN=Rps23 PE=2 SV=3 - [RS23_MOUSE]	8.39	1	1	1	4	0.98	0.89	0.90	1.08	1.10	1.01	1.03	1.02	1.03	0.97	0.99	0.98	0.99	15.80	10.49
Q09200	Beta-1,4 N-acetylgalactosaminyltransferase 1 OS=Mus musculus GN=B4galnt1 PE=2 SV=1 - [B4GN1_MOUSE]	3.75	1	2	2	3	0.87	1.33	1.53	1.07	1.23	0.95	1.09	1.02	1.17	0.99	1.14	1.11	1.28	59.18	8.59
P26231	Catenin alpha-1 OS=Mus musculus GN=Ctnna1 PE=1 SV=1 - [CTNA1_MOUSE]	22.74	1	7	16	45	0.93	0.91	0.91	0.88	1.09	0.96	1.02	1.02	1.13	1.02	1.13	0.91	0.96	100.04	6.23
Q80TE7	Leucine-rich repeat-containing protein 7 OS=Mus musculus GN=Lrrc7 PE=1 SV=2 - [LRRC7_MOUSE]	28.19	2	30	31	73	1.01	1.04	1.02	1.02	1.00	1.06	1.02	1.02	0.98	0.97	0.94	0.97	0.99	166.80	6.61
Q9JKB1	Ubiquitin carboxyl-terminal hydrolase isozyme L3 OS=Mus musculus GN=Uchl3 PE=1 SV=2 - [UCHL3_MOUSE]	56.09	2	10	10	51	1.01	0.77	0.82	0.92	0.94	0.94	0.95	1.02	1.00	0.92	0.91	0.93	0.97	26.14	5.05
F6RPJ9	Insulin-degrading enzyme (Fragment) OS=Mus musculus GN=Ide PE=3 SV=1 - [F6RPJ9_MOUSE]	12.16	2	11	11	17	0.95	1.05	1.16	1.10	1.19	0.89	0.91	1.02	1.03	0.83	0.89	0.94	1.01	114.19	6.27
Q9ERV1	Probable E3 ubiquitin-protein ligase makorin-2 OS=Mus musculus GN=Mkrn2 PE=2 SV=2 - [MKRN2_MOUSE]	12.02	1	5	5	7	0.97	1.03	1.06	0.74	0.91	0.96	1.09	1.02	1.02	0.94	1.02	0.95	0.97	46.57	7.34

Q9D727	Uncharacterized protein C6orf226 homolog OS=Mus musculus PE=4 SV=1 - [CF226_MOUSE]	37.72	1	2	2	4	1.09	1.03	0.94	0.89	0.82	1.24	1.13	1.02	0.93	1.14	1.05	1.14	1.05	12.01	6.29
E9QN14	SLIT-ROBO Rho GTPase-activating protein 3 OS=Mus musculus GN=Srgap3 PE=2 SV=1 - [E9QN14_MOUSE]	27.07	4	18	22	44	1.02	0.90	0.89	1.09	1.07	0.96	0.94	1.02	0.98	1.05	1.06	1.01	1.06	121.65	6.57
Q99LI7	Cleavage stimulation factor subunit 3 OS=Mus musculus GN=Cstf3 PE=1 SV=1 - [CSTF3_MOUSE]	1.81	1	1	1	2	1.07	1.18	1.10	1.00	0.93	0.97	0.90	1.02	0.95	0.94	0.88	1.11	1.04	82.82	8.12
H3BK80	PH and SEC7 domain-containing protein 2 OS=Mus musculus GN=Psd2 PE=2 SV=1 - [H3BK80_MOUSE]	3.61	4	2	2	4	1.06	0.86	0.81	0.90	0.84	0.90	0.84	1.02	0.95	0.99	0.94	0.83	0.78	59.88	4.74
E9PWM3	Protein Armcx4 OS=Mus musculus GN=Armcx4 PE=2 SV=1 - [E9PWM3_MOUSE]	3.35	1	5	5	7	1.14	1.12	1.06	0.96	0.84	1.01	0.89	1.02	0.92	0.96	0.81	0.97	0.76	242.81	4.87
P58468	Protein FAM207A OS=Mus musculus GN=Fam207a PE=1 SV=1 - [F207A_MOUSE]	7.31	1	1	1	2	1.21	1.38	1.14	0.95	0.78	0.96	0.79	1.02	0.84	0.94	0.78	0.90	0.75	24.80	11.00
O88428	Bifunctional 3'-phosphoadenosine 5'-phosphosulfate synthase 2 OS=Mus musculus GN=Papss2 PE=1 SV=2 - [PAPS2_MOUSE]	2.25	1	1	1	1	1.02	1.15	1.13	1.01	0.99	1.05	1.02	1.02	0.99	1.11	1.09	1.21	1.18	70.31	7.58
Q8BHC1	Ras-related protein Rab-39B OS=Mus musculus GN=Rab39b PE=2 SV=1 - [RB39B_MOUSE]	34.74	11	5	7	56	1.06	1.10	0.89	1.21	1.03	0.84	0.77	1.02	0.79	0.92	0.77	0.99	0.82	24.62	7.83
Q9DCR2	AP-3 complex subunit sigma-1 OS=Mus musculus GN=Ap3s1 PE=1 SV=2 - [AP3S1_MOUSE]	13.99	1	2	2	11	0.96	1.00	1.03	1.08	1.08	0.98	1.00	1.02	1.10	1.03	1.07	1.08	1.15	21.72	5.39
Q8BK63-2	Isoform 2 of Casein kinase I isoform alpha OS=Mus musculus GN=Csnk1a1 - [KC1A_MOUSE]	7.08	8	2	2	3	1.17	1.33	1.13	1.19	1.05	0.92	0.78	1.02	0.86	1.08	0.92	1.23	1.05	37.54	9.48
Q9JMK2	Casein kinase I isoform epsilon OS=Mus musculus GN=Csnk1e PE=1 SV=2 - [KC1E_MOUSE]	15.14	3	4	5	11	0.87	0.93	1.02	0.84	0.97	0.93	1.11	1.02	1.23	0.96	1.09	0.92	1.05	47.29	9.66
Q542I8	Integrin beta OS=Mus musculus GN=Itgb2 PE=2 SV=1 - [Q542I8_MOUSE]	8.05	6	4	5	9	1.02	1.25	1.24	1.01	1.06	0.97	0.94	1.02	0.99	1.12	1.10	1.13	1.11	84.84	7.05

Q78PG9	Coiled-coil domain-containing protein 25 OS=Mus musculus GN=Ccdc25 PE=1 SV=1 - [CCD25_MOUSE]	17.31	1	4	4	6	1.05	0.77	0.75	0.90	0.91	0.97	0.93	1.02	1.01	0.97	0.92	1.00	0.93	24.46	6.95
D3Z7D2	tRNA (guanine-N(7)-)-methyltransferase OS=Mus musculus GN=Mettl1 PE=2 SV=1 - [D3Z7D2_MOUSE]	6.14	2	1	1	2	0.90	0.97	1.07	1.14	1.26	0.97	1.07	1.02	1.12	1.14	1.26	1.09	1.21	25.82	9.04
Q61656	Probable ATP-dependent RNA helicase DDX5 OS=Mus musculus GN=Ddx5 PE=1 SV=2 - [DDX5_MOUSE]	40.72	5	18	24	68	0.96	0.91	0.97	0.99	1.06	1.06	1.16	1.02	1.11	1.00	1.03	1.03	1.07	69.25	8.92
D3Z0T8	DnaJ homolog subfamily C member 28 (Fragment) OS=Mus musculus GN=Dnajc28 PE=2 SV=1 - [D3Z0T8_MOUSE]	16.67	4	1	1	2	1.01	0.98	0.96	0.90	0.89	0.90	0.88	1.02	1.00	1.01	1.00	0.95	0.94	9.64	8.53
Q91W39	Nuclear receptor coactivator 5 OS=Mus musculus GN=Ncoa5 PE=1 SV=1 - [NCOA5_MOUSE]	20.90	2	4	8	19	0.86	0.99	1.16	0.95	1.12	1.05	1.21	1.02	1.18	1.03	1.20	0.94	1.13	65.28	9.82
Q8CH72	E3 ubiquitin-protein ligase TRIM32 OS=Mus musculus GN=Trim32 PE=1 SV=2 - [TRI32_MOUSE]	25.95	3	13	13	25	0.94	0.90	0.93	0.98	0.96	1.02	1.06	1.02	1.04	0.97	1.03	0.99	1.02	72.01	6.90
Q8K2H2	OTU domain-containing protein 6B OS=Mus musculus GN=Otud6b PE=2 SV=1 - [OTU6B_MOUSE]	27.55	3	5	5	8	1.16	1.18	1.13	0.86	0.88	1.17	0.99	1.02	0.95	1.12	0.96	1.15	1.01	33.74	5.53
P70699	Lysosomal alpha-glucosidase OS=Mus musculus GN=Gaa PE=1 SV=2 - [LYAG_MOUSE]	15.32	5	12	12	25	1.07	1.02	0.98	0.94	0.91	1.00	1.03	1.02	0.90	1.17	1.04	1.07	1.13	106.18	5.83
Q99LF4	tRNA-splicing ligase RtcB homolog OS=Mus musculus GN=D10Wsu52e PE=2 SV=1 - [RTCB_MOUSE]	47.33	1	18	18	36	0.90	0.94	1.01	1.01	1.07	1.00	1.10	1.02	1.14	0.99	1.06	0.99	1.05	55.21	7.23
P16546	Spectrin alpha chain, non-erythrocytic 1 OS=Mus musculus GN=Sptan1 PE=1 SV=4 - [SPTN1_MOUSE]	81.03	3	6	218	5377	0.94	0.81	0.84	0.83	0.92	0.95	1.03	1.02	1.08	0.90	0.96	0.90	0.93	284.42	5.33
Q8BG79-2	Isoform 2 of CWF19-like protein 2 OS=Mus musculus GN=Cwf19l2 - [C19L2_MOUSE]	2.48	2	1	1	1	1.10	0.85	0.77	1.28	1.17	1.13	1.03	1.02	0.93	0.90	0.83	0.78	0.72	56.24	8.75

Q8BHJ5	F-box-like/WD repeat-containing protein TBL1XR1 OS=Mus musculus GN=Tbl1xr1 PE=2 SV=1 - [TBL1R_MOUSE]	8.56	2	4	4	17	0.96	0.87	0.91	0.94	0.94	1.04	1.14	1.02	1.03	1.01	1.06	0.98	0.98	55.63	5.63
Q9D2C2-2	Isoform 2 of Protein SAAL1 OS=Mus musculus GN=Saal1 - [SAAL1_MOUSE]	3.23	2	1	1	1	1.13	0.83	0.73	1.12	0.99	0.68	0.60	1.02	0.90	0.89	0.79	0.71	0.63	48.20	4.53
Q8BYW1-	Isoform 2 of Rho GTPase-activating protein 25 OS=Mus musculus GN=Arhgap25 - [RHG25_MOUSE]	9.84	8	4	5	6	0.86	0.87	1.02	0.96	1.15	1.02	1.14	1.02	1.11	0.97	1.20	0.86	1.02	62.83	5.48
P11031	Activated RNA polymerase II transcriptional coactivator p15 OS=Mus musculus GN=Sub1 PE=1 SV=3 - [TCP4_MOUSE]	39.37	1	7	7	24	1.02	0.85	0.85	0.95	0.97	1.02	1.00	1.02	0.97	0.86	0.88	0.79	0.75	14.42	9.60
Q6PDL0	Cytoplasmic dynein 1 light intermediate chain 2 OS=Mus musculus GN=Dync1li2 PE=1 SV=2 - [DC1L2_MOUSE]	27.24	1	10	11	34	0.98	0.94	1.02	1.02	0.91	1.01	1.04	1.02	1.09	1.00	1.02	1.03	1.03	54.18	6.28
G5E897	KDEL (Lys-Asp-Glu-Leu) containing 2, isoform CRA_b OS=Mus musculus GN=Kdelc2 PE=4 SV=1 - [G5E897_MOUSE]	3.38	1	1	1	2	1.06	1.31	1.24	1.28	1.21	1.17	1.10	1.02	0.96	1.18	1.11	1.26	1.20	57.65	7.74
Q8R0F8	Acylpyruvase FAHD1, mitochondrial OS=Mus musculus GN=Fahd1 PE=1 SV=2 - [FAHD1_MOUSE]	72.25	1	10	10	107	1.02	0.73	0.74	0.90	0.89	0.98	0.97	1.02	1.00	0.93	0.92	0.84	0.83	25.16	7.69
B0QZP4	tRNA-splicing endonuclease subunit Sen54 (Fragment) OS=Mus musculus GN=Tsen54 PE=2 SV=1 - [B0QZP4_MOUSE]	6.54	4	1	1	2	0.92	1.35	1.47	0.96	1.05	1.10	1.19	1.02	1.11	1.06	1.16	1.00	1.09	17.30	8.29
Q9QYB1	Chloride intracellular channel protein 4 OS=Mus musculus GN=Clic4 PE=1 SV=3 - [CLIC4_MOUSE]	45.45	1	7	7	18	0.96	1.05	0.90	1.01	1.11	0.99	1.04	1.02	0.97	1.11	1.27	1.22	1.14	28.71	5.59
Q5KU39	Vacuolar protein sorting-associated protein 41 homolog OS=Mus musculus GN=Vps41 PE=2 SV=1 - [VPS41_MOUSE]	1.88	1	1	2	3	1.15	1.14	0.99	1.25	1.08	1.03	0.89	1.02	0.88	1.06	0.92	1.24	1.08	98.54	5.81

Q8BFW9	Solute carrier family 2, facilitated glucose transporter member 12 OS=Mus musculus GN=Slc2a12 PE=2 SV=1 - [GTR12_MOUSE]	3.38	1	1	1	1	0.90	0.69	0.76	0.77	0.85	0.95	1.05	1.02	1.12	0.79	0.88	0.81	0.89	67.29	8.07
E0CYQ2	NudC domain-containing protein 2 OS=Mus musculus GN=Nudcd2 PE=2 SV=1 - [E0CYQ2_MOUSE]	15.91	2	2	2	6	0.89	0.94	1.08	0.90	1.04	0.97	1.08	1.02	1.17	0.94	1.08	0.96	1.10	14.72	4.68
Q99KK9	Probable histidine--tRNA ligase, mitochondrial OS=Mus musculus GN=Hars2 PE=2 SV=1 - [SYHM_MOUSE]	10.89	2	2	5	8	1.00	1.04	1.04	1.10	1.11	1.09	1.09	1.02	1.02	0.99	0.99	0.94	0.95	56.95	8.32
Q3TRM4-	Isoform 4 of Neuropathy target esterase OS=Mus musculus GN=Pnpla6 - [PLPL6_MOUSE]	1.03	4	1	1	2	1.15	1.21	1.05	1.16	1.01	0.84	0.73	1.02	0.89	0.91	0.79	0.91	0.80	129.27	8.84
Q7M6Y3-	Isoform 6 of Phosphatidylinositol-binding clathrin assembly protein OS=Mus musculus GN=Picalm - [PICAL_MOUSE]	24.39	3	1	10	21	0.99	0.42	0.43	1.07	1.08	0.92	0.93	1.02	1.03	0.97	0.98	0.86	0.87	70.52	7.90
D3Z5Y5	Choline/ethanolaminephosphotransferase 1 (Fragment) OS=Mus musculus GN=Cept1 PE=2 SV=1 - [D3Z5Y5_MOUSE]	18.33	4	1	1	2	1.01	1.30	1.28	1.14	1.12	0.83	0.82	1.02	1.00	0.99	0.98	1.20	1.19	6.74	10.01
Q3UZA1-	Isoform 2 of CapZ-interacting protein OS=Mus musculus GN=Rcsd1 - [CPZIP_MOUSE]	21.47	2	6	6	14	0.99	1.18	1.20	0.91	0.92	1.21	1.17	1.02	1.02	1.00	0.98	0.91	0.90	40.83	5.05
O70582	Arachidonate 12-lipoxygenase, 12R-type OS=Mus musculus GN=Alox12b PE=2 SV=1 - [LX12B_MOUSE]	1.57	1	1	1	2	0.87	0.66	0.76	0.84	0.96	1.03	1.17	1.02	1.16	1.06	1.22	0.90	1.03	80.53	6.71
Q9JLI8	Squamous cell carcinoma antigen recognized by T-cells 3 OS=Mus musculus GN=Sart3 PE=2 SV=1 - [SART3_MOUSE]	5.72	2	5	5	10	0.93	0.98	0.99	1.08	1.07	0.98	1.03	1.02	1.07	0.84	0.93	0.98	1.04	109.55	5.24
Q9WVH9	Fibulin-5 OS=Mus musculus GN=Fbln5 PE=2 SV=1 - [FBLN5_MOUSE]	12.72	1	4	4	6	0.87	1.55	1.54	1.09	1.20	0.91	0.99	1.02	1.14	1.16	1.38	1.06	1.23	50.16	4.70
E9QME5	E3 ubiquitin-protein ligase TRIM33 OS=Mus musculus GN=Trim33 PE=2 SV=1 - [E9QME5_MOUSE]	1.69	4	2	2	2	1.06	1.40	1.31	0.91	0.85	0.86	0.81	1.02	0.96	0.91	0.86	1.04	0.98	121.61	6.73

Q7TPD3	Roundabout homolog 2 OS=Mus musculus GN=Robo2 PE=2 SV=2 - [ROBO2_MOUSE]	21.29	5	21	22	43	1.06	1.06	1.03	1.01	1.00	1.06	1.01	1.02	0.98	1.00	0.96	1.01	1.00	161.09	6.33
Q91X78	Erlin-1 OS=Mus musculus GN=Erlin1 PE=1 SV=1 - [ERLN1_MOUSE]	11.27	2	1	3	8	0.81	1.15	1.42	1.16	1.44	0.63	0.78	1.02	1.26	0.83	1.02	0.90	1.12	38.91	7.21
Q60936	Chaperone activity of bc1 complex-like, mitochondrial OS=Mus musculus GN=Adck3 PE=2 SV=2 - [ADCK3_MOUSE]	4.19	3	2	2	3	1.13	1.53	1.35	1.51	1.33	1.03	0.90	1.02	0.90	0.90	0.80	1.19	1.05	71.70	6.54
Q9CZX7	Transmembrane protein 55A OS=Mus musculus GN=Tmem55a PE=1 SV=1 - [TM55A_MOUSE]	4.28	1	1	1	2	1.02	1.03	1.00	1.08	1.06	0.83	0.81	1.02	1.00	0.86	0.84	1.02	1.00	28.02	8.68
P97352	Protein S100-A13 OS=Mus musculus GN=S100a13 PE=1 SV=1 - [S10AD_MOUSE]	41.84	1	5	5	19	1.10	0.69	0.69	0.85	0.76	1.00	0.93	1.02	0.88	0.90	0.87	0.85	0.83	11.15	6.13
Q8R2K3	Single-stranded DNA-binding protein OS=Mus musculus GN=Ssbp1 PE=2 SV=1 - [Q8R2K3_MOUSE]	56.76	3	8	8	31	1.04	0.85	0.77	0.89	0.84	0.98	0.96	1.02	0.98	0.97	0.98	0.94	0.99	17.15	9.79
Q6ZWY8	Thymosin beta-10 OS=Mus musculus GN=Tmsb10 PE=2 SV=3 - [TYB10_MOUSE]	45.45	3	1	3	178	1.04	0.49	0.47	0.95	0.91	1.26	1.20	1.02	0.98	0.97	0.93	0.88	0.85	5.02	5.36
Q64337	Sequestosome-1 OS=Mus musculus GN=Sqstm1 PE=1 SV=1 - [SQSTM_MOUSE]	40.27	4	8	8	24	1.10	1.06	0.90	0.98	0.99	1.05	1.02	1.02	0.93	0.92	0.88	1.08	0.93	48.13	5.21
Q9QZF2	Glypican-1 OS=Mus musculus GN=Gpc1 PE=1 SV=1 - [GPC1_MOUSE]	31.24	1	10	10	17	1.05	1.07	1.14	1.21	1.08	1.07	1.03	1.02	0.89	1.07	0.91	0.94	0.87	61.32	7.05
Q61609	Sodium-dependent phosphate transporter 1 OS=Mus musculus GN=Slc20a1 PE=1 SV=1 - [S20A1_MOUSE]	5.73	1	3	3	5	1.06	0.97	0.92	0.98	0.97	1.00	0.93	1.02	0.94	0.93	0.86	0.89	0.87	74.11	6.89
A2AGX3	PR domain-containing protein 11 OS=Mus musculus GN=Prdm11 PE=3 SV=1 - [PRD11_MOUSE]	1.77	1	1	1	1	0.95	0.69	0.72	0.80	0.84	1.06	1.11	1.02	1.07	0.81	0.85	0.75	0.79	63.34	6.55
P97326	Cadherin-6 OS=Mus musculus GN=Cdh6 PE=1 SV=2 - [CADH6_MOUSE]	6.08	1	1	3	7	0.99	1.00	1.00	0.98	0.99	0.96	0.96	1.02	1.03	0.96	0.97	1.02	1.03	88.30	5.00
Q8BGH7	CDC42 small effector protein 2 OS=Mus musculus GN=Cdc42se2 PE=1 SV=1 - [C42S2_MOUSE]	22.62	1	1	1	3	0.94	0.62	0.66	0.95	1.00	1.00	1.05	1.02	1.08	0.91	0.97	0.82	0.87	9.22	8.35

Q9ERD7	Tubulin beta-3 chain OS=Mus musculus GN=Tubb3 PE=1 SV=1 - [TBB3_MOUSE]	73.56	1	11	29	1253	0.98	1.04	1.07	1.06	1.08	1.02	1.03	1.02	1.04	1.03	1.05	1.06	1.07	50.39	4.93
Q8C0Y0-2	Isoform 2 of Serine/threonine-protein phosphatase 4 regulatory subunit 4 OS=Mus musculus GN=Ppp4r4 - [PP4R4_MOUSE]	1.52	3	1	1	2	0.95	1.13	1.19	1.11	1.17	1.06	1.11	1.02	1.07	1.06	1.12	1.01	1.06	90.42	6.57
Q9QYE3-	Isoform 6 of B-cell lymphoma/leukemia 11A OS=Mus musculus GN=Bcl11a - [BC11A_MOUSE]	32.98	3	1	4	10	0.86	1.15	1.34	0.87	1.02	0.97	1.13	1.02	1.19	1.04	1.22	1.15	1.34	21.06	7.15
Q9CRD4	Dysbindin domain-containing protein 2 OS=Mus musculus GN=Dbndd2 PE=1 SV=1 - [DBND2_MOUSE]	7.59	1	1	1	2	1.08	1.10	1.02	0.84	0.78	1.03	0.95	1.02	0.94	1.07	0.99	1.17	1.09	17.18	4.22
F8VPK3	Myc-associated zinc finger protein OS=Mus musculus GN=Maz PE=4 SV=1 - [F8VPK3_MOUSE]	1.89	3	1	1	1	0.89	0.93	1.04	0.94	1.06	1.08	1.20	1.02	1.14	1.02	1.15	0.99	1.12	48.71	8.95
P61087	Ubiquitin-conjugating enzyme E2 K OS=Mus musculus GN=Ube2k PE=1 SV=3 - [UBE2K_MOUSE]	40.50	5	7	7	14	0.97	0.99	1.02	0.97	1.02	1.04	1.08	1.02	1.15	0.98	1.20	1.00	1.15	22.39	5.44
Q60770	Syntaxin-binding protein 3 OS=Mus musculus GN=Stxbp3 PE=1 SV=1 - [STXB3_MOUSE]	9.63	2	5	5	13	0.92	1.09	1.18	1.04	1.11	0.96	1.04	1.02	1.01	1.04	1.11	1.08	1.13	67.90	8.02
F6SVV1	Protein Gm9493 OS=Mus musculus GN=Gm9493 PE=4 SV=1 - [F6SVV1_MOUSE]	36.46	3	6	6	10	0.98	0.90	0.87	1.08	1.11	1.05	1.12	1.02	0.96	1.04	1.08	0.97	0.98	21.87	10.02
P21237	Brain-derived neurotrophic factor OS=Mus musculus GN=Bdnf PE=1 SV=1 - [BDNF_MOUSE]	6.43	3	1	1	4	1.16	1.05	0.90	0.90	0.77	1.04	0.89	1.02	0.88	1.11	0.96	1.01	0.87	28.11	8.66
Q62092	Neuron-specific protein family member 1 OS=Mus musculus GN=Nsg1 PE=2 SV=3 - [NSG1_MOUSE]	4.32	1	1	1	1	1.06	1.07	1.01	0.93	0.88	1.06	1.00	1.02	0.96	1.08	1.02	1.14	1.08	20.92	6.21
Q9D6R2	Isocitrate dehydrogenase [NAD] subunit alpha, mitochondrial OS=Mus musculus GN=Idh3a PE=1 SV=1 - [IDH3A_MOUSE]	54.10	2	21	21	146	0.90	0.85	0.94	0.96	1.05	1.04	1.11	1.02	1.11	0.98	1.08	0.94	1.04	39.61	6.73

Q6PD24	Ankyrin repeat domain-containing protein 13D OS=Mus musculus GN=Ankrd13d PE=2 SV=1 - [AN13D_MOUSE]	7.34	3	2	3	7	0.95	1.06	1.01	0.89	0.83	1.11	1.12	1.02	1.04	1.13	1.14	0.96	0.98	58.37	5.26
E0CZ72	Kinesin-like protein KIF2A OS=Mus musculus GN=Kif2a PE=2 SV=1 - [E0CZ72_MOUSE]	33.11	7	3	20	64	0.85	0.97	1.17	0.93	0.96	0.94	1.09	1.02	1.10	1.13	1.30	0.97	1.13	83.81	6.42
Q9CPX6	Ubiquitin-like-conjugating enzyme ATG3 OS=Mus musculus GN=Atg3 PE=1 SV=1 - [ATG3_MOUSE]	30.89	1	6	6	19	0.97	0.92	0.87	0.98	1.01	1.07	1.10	1.02	1.03	1.05	1.08	1.18	1.05	35.77	4.72
P97825	Hematological and neurological expressed 1 protein OS=Mus musculus GN=Hn1 PE=1 SV=3 - [HN1_MOUSE]	37.66	1	4	4	42	1.11	1.03	0.90	0.91	0.79	1.07	0.94	1.02	0.97	0.98	0.89	1.01	0.90	16.07	5.31
P26883	Peptidyl-prolyl cis-trans isomerase FKBP1A OS=Mus musculus GN=Fkbp1a PE=1 SV=2 - [FKB1A_MOUSE]	73.15	2	8	8	216	1.12	0.85	0.74	0.91	0.84	1.09	0.99	1.02	0.91	0.98	0.89	0.98	0.89	11.92	8.16
E9QLH4	E3 ubiquitin-protein ligase TRIM9 OS=Mus musculus GN=Trim9 PE=2 SV=1 - [E9QLH4_MOUSE]	27.54	8	15	15	41	0.92	1.11	1.21	1.02	1.05	0.96	1.04	1.02	1.03	0.97	1.01	1.05	1.11	87.49	6.92
P42128	Forkhead box protein K1 OS=Mus musculus GN=Foxk1 PE=1 SV=2 - [FOXK1_MOUSE]	6.95	1	2	2	3	0.95	1.14	1.16	1.08	1.10	1.06	1.10	1.02	1.07	0.96	0.98	1.15	1.21	74.87	9.17
F7D5G9	Protein Ciz1 (Fragment) OS=Mus musculus GN=Ciz1 PE=4 SV=1 - [F7D5G9_MOUSE]	3.27	5	1	1	1	0.90	0.97	1.09	0.79	0.88	1.08	1.20	1.02	1.14	1.19	1.33	1.09	1.22	27.19	4.79
Q9CZW6	E3 ubiquitin-protein ligase RNF146 OS=Mus musculus GN=Rnf146 PE=1 SV=2 - [RN146_MOUSE]	11.42	2	3	3	6	0.94	0.86	1.04	0.85	0.94	1.02	1.07	1.02	1.11	1.01	1.09	0.99	1.03	38.91	5.20
Q80U35	Rho guanine nucleotide exchange factor 17 OS=Mus musculus GN=Arhgef17 PE=1 SV=2 - [ARHGH_MOUSE]	1.56	2	3	3	4	1.05	0.91	0.85	1.16	1.15	0.99	0.92	1.02	0.95	1.08	1.01	1.04	1.00	221.53	6.30
P38060	Hydroxymethylglutaryl-CoA lyase, mitochondrial OS=Mus musculus GN=Hmgcl PE=1 SV=2 - [HMGCL_MOUSE]	22.15	1	5	5	6	1.07	1.22	1.14	1.08	1.07	1.19	0.99	1.02	1.10	1.28	1.01	1.36	1.26	34.22	8.41

P55302	Alpha-2-macroglobulin receptor-associated protein OS=Mus musculus GN=Lrpap1 PE=1 SV=1 - [AMRP_MOUSE]	47.50	2	17	17	81	0.94	0.88	0.94	0.84	0.90	1.07	1.15	1.02	1.06	1.00	1.03	0.91	1.04	42.19	7.87
Q6P1B3	PILR alpha-associated neural protein OS=Mus musculus GN=Pianp PE=1 SV=1 - [PIANP_MOUSE]	21.22	3	3	3	7	1.06	0.74	0.70	1.00	0.94	1.25	1.17	1.02	0.96	1.10	1.04	1.27	1.29	29.73	8.72
H3BKN0	tRNA (cytosine(34)-C(5))-methyltransferase OS=Mus musculus GN=Nsun2 PE=2 SV=1 - [H3BKN0_MOUSE]	4.16	3	2	2	3	1.18	1.12	0.95	1.01	0.86	0.91	0.77	1.02	0.87	1.04	0.88	1.03	0.88	81.32	6.77
Q8BGY3	Leucine zipper protein 2 OS=Mus musculus GN=Luzp2 PE=2 SV=1 - [LUZP2_MOUSE]	24.35	1	6	7	13	1.05	1.06	0.88	0.91	0.87	1.06	0.96	1.02	1.01	0.94	0.93	0.93	0.89	39.00	8.50
Q91YE7-2	Isoform 2 of RNA-binding protein 5 OS=Mus musculus GN=Rbm5 - [RBM5_MOUSE]	7.99	2	5	5	29	0.90	0.79	0.91	0.93	0.98	1.06	1.16	1.02	1.16	0.99	1.20	0.88	0.97	92.18	6.21
B2RRE2	Myo18a protein OS=Mus musculus GN=Myo18a PE=2 SV=1 - [B2RRE2_MOUSE]	28.04	12	42	43	96	0.95	1.01	1.03	1.03	1.08	0.99	1.04	1.02	1.06	0.97	1.03	0.98	1.01	231.99	6.10
Q8VBT9	Tether containing UBX domain for GLUT4 OS=Mus musculus GN=Aspscr1 PE=1 SV=1 - [ASPC1_MOUSE]	14.73	12	5	5	10	0.91	1.05	1.17	1.00	1.14	1.02	1.10	1.02	1.06	1.06	1.22	0.99	1.14	59.76	6.96
Q6VH22	Intraflagellar transport protein 172 homolog OS=Mus musculus GN=Ift172 PE=1 SV=1 - [IF172_MOUSE]	2.00	1	1	1	3	1.49	1.76	1.18	1.15	0.77	1.33	0.89	1.02	0.69	0.96	0.64	1.26	0.85	197.42	6.02
P18242	Cathepsin D OS=Mus musculus GN=Ctsd PE=1 SV=1 - [CATD_MOUSE]	33.17	3	10	10	33	1.01	0.83	0.94	0.97	1.00	1.00	1.01	1.02	1.02	0.95	1.01	0.95	1.04	44.92	7.15
Q9Z2A9-2	Isoform 2 of Gamma-glutamyltransferase 5 OS=Mus musculus GN=Ggt5 - [GGT5_MOUSE]	2.19	3	1	1	1	1.31	1.44	1.10	1.14	0.87	1.31	1.00	1.02	0.78	1.13	0.86	1.26	0.97	49.33	7.84
H3BK68	tRNA (guanine(26)-N(2))-dimethyltransferase (Fragment) OS=Mus musculus GN=Trmt1 PE=2 SV=1 - [H3BK68_MOUSE]	8.60	7	1	1	3	1.03	1.17	1.14	0.94	0.91	1.02	0.99	1.02	0.99	1.07	1.05	1.15	1.12	24.15	5.66
Q9Z0M3	Cadherin-20 OS=Mus musculus GN=Cdh20 PE=2 SV=1 - [CAD20_MOUSE]	2.87	1	1	2	3	0.99	1.06	1.06	1.08	1.09	1.10	1.11	1.02	1.03	1.05	1.06	1.03	1.04	88.94	4.72

D3YZ18	High mobility group protein B1 (Fragment) OS=Mus musculus GN=Hmgb1 PE=2 SV=1 - [D3YZ18_MOUSE]	64.80	2	1	8	160	0.74	0.82	1.10	0.86	1.16	1.19	1.60	1.02	1.37	0.89	1.20	0.82	1.11	14.42	9.76
Q9DAK9	14 kDa phosphohistidine phosphatase OS=Mus musculus GN=Phpt1 PE=1 SV=1 - [PHP14_MOUSE]	56.45	1	7	7	43	0.97	0.75	0.79	0.87	0.92	0.99	1.06	1.02	1.08	0.90	0.89	0.83	0.91	13.99	5.53
Q8C0P5	Coronin-2A OS=Mus musculus GN=Coro2a PE=2 SV=1 - [COR2A_MOUSE]	6.87	2	3	4	6	1.01	1.36	1.28	1.16	1.11	0.92	0.95	1.02	1.01	0.95	0.90	1.06	1.00	59.54	7.71
Q8C008	Double zinc ribbon and ankyrin repeat-containing protein 1 OS=Mus musculus GN=Dzank1 PE=1 SV=2 - [DZAN1_MOUSE]	17.87	2	10	10	15	1.14	1.03	1.15	1.01	0.92	1.12	1.05	1.02	0.92	1.03	0.95	1.08	0.99	84.97	8.18
Q924D0	Reticulon-4-interacting protein 1, mitochondrial OS=Mus musculus GN=Rtn4ip1 PE=1 SV=2 - [RT4I1_MOUSE]	2.78	1	1	1	3	0.96	0.86	0.89	0.92	0.96	1.15	1.19	1.02	1.06	1.02	1.06	1.01	1.05	43.34	9.20
Q6PGF7	Exocyst complex component 8 OS=Mus musculus GN=Exoc8 PE=1 SV=1 - [EXOC8_MOUSE]	20.67	1	10	10	39	0.99	1.11	1.08	1.09	1.09	0.99	0.99	1.02	1.05	1.03	1.04	1.08	1.09	80.98	5.40
Q9DBC7	cAMP-dependent protein kinase type I-alpha regulatory subunit OS=Mus musculus GN=Prkar1a PE=1 SV=3 - [KAP0_MOUSE]	40.42	3	9	13	30	0.94	1.02	1.13	1.02	1.06	1.06	1.14	1.02	1.18	1.08	1.21	1.10	1.20	43.16	5.35
Q6VNS1	NT-3 growth factor receptor OS=Mus musculus GN=Ntrk3 PE=1 SV=1 - [NTRK3_MOUSE]	14.42	3	6	9	14	0.94	1.16	1.32	1.05	1.10	0.97	1.00	1.02	1.17	0.90	1.03	1.01	1.16	92.70	6.67
Q9CQ91	NADH dehydrogenase [ubiquinone] 1 alpha subcomplex subunit 3 OS=Mus musculus GN=Ndufa3 PE=1 SV=1 - [NDUA3_MOUSE]	50.00	2	4	4	13	1.02	1.07	1.22	1.09	1.04	0.97	0.99	1.02	0.91	1.04	0.95	1.06	1.00	9.32	8.47
Q80U57	Regulating synaptic membrane exocytosis protein 3 OS=Mus musculus GN=Rims3 PE=1 SV=2 - [RIMS3_MOUSE]	30.29	2	5	6	11	0.96	1.06	1.22	1.11	1.13	1.02	1.06	1.02	1.04	0.92	0.92	1.01	1.03	32.57	9.29
Q8VCN9	Tubulin-specific chaperone C OS=Mus musculus GN=Tbcc PE=2 SV=1 - [TBCC_MOUSE]	24.63	1	8	8	14	0.99	1.05	1.08	0.96	0.95	0.93	0.95	1.02	0.96	0.94	0.95	1.01	1.07	38.10	5.30

Q8BP01	Vimentin-type intermediate filament-associated coiled-coil protein OS=Mus musculus GN=Vmac PE=2 SV=1 - [VMAC_MOUSE]	4.60	1	1	1	1	0.71	0.96	1.35	0.81	1.14	0.99	1.39	1.02	1.44	1.33	1.87	1.18	1.66	19.01	5.39
O35615	Zinc finger protein ZFPM1 OS=Mus musculus GN=Zfpm1 PE=1 SV=1 - [FOG1_MOUSE]	2.21	1	1	1	1	1.08	0.77	0.71	1.10	1.02	1.31	1.21	1.02	0.94	1.11	1.03	1.18	1.10	105.92	6.89
Q3UKJ7-2	Isoform 2 of WD40 repeat-containing protein SMU1 OS=Mus musculus GN=Smu1 - [SMU1_MOUSE]	6.93	2	2	2	2	0.95	0.99	1.04	0.80	0.84	1.03	1.08	1.02	1.07	1.10	1.16	1.03	1.09	37.52	6.65
Q8BGR9	Ubiquitin-like domain-containing CTD phosphatase 1 OS=Mus musculus GN=Ublcp1 PE=1 SV=1 - [UBCP1_MOUSE]	5.97	2	2	2	4	0.89	1.01	1.14	1.01	1.14	0.95	1.06	1.03	1.15	0.89	1.01	0.85	0.96	36.81	6.46
Q9QYJ0	DnaJ homolog subfamily A member 2 OS=Mus musculus GN=Dnaja2 PE=1 SV=1 - [DNJA2_MOUSE]	42.23	1	12	12	63	0.95	0.83	0.79	0.95	0.98	1.00	0.97	1.03	1.01	0.89	0.91	0.99	0.89	45.72	6.48
Q8BWU5	Probable tRNA threonylcarbamoyladenosine biosynthesis protein Osgep OS=Mus musculus GN=Osgep PE=2 SV=2 - [OSGEP_MOUSE]	8.06	4	2	2	4	0.98	1.11	1.13	1.11	1.13	0.96	0.97	1.03	1.04	1.09	1.11	1.11	1.13	36.28	6.24
Q5SXA9	Protein KIBRA OS=Mus musculus GN=Wwc1 PE=1 SV=1 - [KIBRA_MOUSE]	0.63	1	1	1	1	1.05	1.02	0.97	0.92	0.87	0.97	0.92	1.03	0.97	0.98	0.93	1.09	1.04	124.03	5.97
A2AD03	Rab proteins geranylgeranyltransferase component A 1 OS=Mus musculus GN=Chm PE=2 SV=1 - [A2AD03_MOUSE]	15.86	4	7	7	13	1.09	0.99	0.96	1.07	1.01	1.05	0.93	1.03	0.97	1.00	1.01	1.13	1.04	73.62	4.69
Q8BXA5	Cleft lip and palate transmembrane protein 1-like protein OS=Mus musculus GN=Clptm1l PE=2 SV=1 - [CLP1L_MOUSE]	2.97	1	1	1	2	0.75	1.22	1.62	0.90	1.20	0.74	0.99	1.03	1.36	0.58	0.77	0.82	1.09	62.14	8.84
P60882	Multiple epidermal growth factor-like domains protein 8 OS=Mus musculus GN=Megf8 PE=2 SV=2 - [MEGF8_MOUSE]	0.86	1	2	2	4	1.03	1.54	1.48	1.00	0.97	0.95	0.92	1.03	0.99	1.01	0.98	1.07	1.04	297.29	6.80

Q922Q8	Leucine-rich repeat-containing protein 59 OS=Mus musculus GN=Lrrc59 PE=2 SV=1 - [LRC59_MOUSE]	31.27	1	8	8	17	0.88	0.87	1.11	0.97	1.08	1.06	1.09	1.03	1.08	1.10	1.06	0.82	1.01	34.86	9.52
Q9CQU0	Thioredoxin domain-containing protein 12 OS=Mus musculus GN=Txndc12 PE=2 SV=1 - [TXD12_MOUSE]	31.18	1	4	4	22	0.94	0.76	0.81	0.89	0.99	1.07	1.06	1.03	1.12	0.94	1.02	0.85	0.90	19.04	5.26
Q6PH08-4	Isoform 4 of ERC protein 2 OS=Mus musculus GN=Erc2 - [ERC2_MOUSE]	50.10	1	2	24	198	1.06	1.08	1.13	0.87	0.89	0.98	1.08	1.03	0.98	0.91	0.90	0.89	0.84	59.67	8.41
Q3UF95	Large proline-rich protein BAG6 OS=Mus musculus GN=Bag6 PE=2 SV=1 [Q3UF95_MOUSE]	15.23	8	11	11	26	0.96	1.20	1.27	1.10	1.17	1.06	1.02	1.03	1.03	1.12	1.10	1.16	1.17	119.07	5.82
Q8BNJ6	Neuropilin and tolloid-like protein 2 OS=Mus musculus GN=Neto2 PE=1 SV=1 - [NETO2_MOUSE]	4.00	2	2	2	2	0.89	0.99	1.11	0.99	1.12	1.02	1.15	1.03	1.15	1.05	1.18	1.34	1.51	59.33	6.77
Q9WUA6	Isoform 2 of RAC-gamma serine/threonine-protein kinase OS=Mus musculus GN=Akt3 - [AKT3_MOUSE]	8.09	2	1	3	6	1.08	1.00	0.93	0.99	0.92	0.95	0.88	1.03	0.95	0.96	0.90	1.14	1.06	54.60	6.70
Q9CXE7	Transmembrane emp24 domain-containing protein 5 OS=Mus musculus GN=Tmed5 PE=2 SV=1 - [TMED5_MOUSE]	10.04	2	2	2	3	0.93	0.79	0.85	1.03	1.11	1.00	1.07	1.03	1.10	0.94	1.02	0.93	1.00	26.16	4.93
Q9CZL5	Pterin-4-alpha-carbinolamine dehydratase 2 OS=Mus musculus GN=Pcbd2 PE=1 SV=2 - [PHS2_MOUSE]	41.91	1	5	5	16	1.21	0.80	0.71	0.95	0.77	1.04	0.84	1.03	0.79	1.03	0.86	0.90	0.76	14.82	9.16
B2RQR5	Small G protein signaling modulator 1 OS=Mus musculus GN=Sgsm1 PE=2 SV=1 - [B2RQR5_MOUSE]	7.04	7	5	5	11	1.13	1.35	1.15	1.06	0.95	1.10	0.98	1.03	0.91	1.22	1.11	1.36	1.11	123.19	5.66
P35550	rRNA 2'-O-methyltransferase fibrillarin OS=Mus musculus GN=Fbl PE=1 SV=2 - [FBRL_MOUSE]	22.94	1	4	6	7	1.12	0.80	0.89	1.23	0.99	0.99	0.87	1.03	1.04	1.05	0.94	1.05	0.85	34.29	10.24
Q925I8	OL-protocadherin isoform OS=Mus musculus GN=Pcdh10 PE=2 SV=1 - [Q925I8_MOUSE]	15.19	3	10	10	33	1.04	1.13	1.11	0.94	0.97	0.99	1.02	1.03	0.95	1.04	1.03	1.06	0.99	113.07	4.92

Q921L3	Transmembrane and coiled-coil domain-containing protein 1 OS=Mus musculus GN=Tmco1 PE=2 SV=1 - [TMCO1_MOUSE]	11.70	1	2	2	3	1.04	1.11	1.07	1.11	1.07	0.84	0.80	1.03	0.99	0.97	0.93	1.02	0.98	21.16	9.74
D3Z5Q9	Tropomodulin-4 (Fragment) OS=Mus musculus GN=Tmod4 PE=2 SV=1 - [D3Z5Q9_MOUSE]	11.41	2	2	2	5	0.97	1.33	1.37	0.71	0.73	23.98	24.59	1.03	1.05	1.05	1.09	1.30	1.35	20.98	4.41
Q9QXA5	U6 snRNA-associated Sm-like protein LSm4 OS=Mus musculus GN=Lsm4 PE=2 SV=1 - [LSM4_MOUSE]	16.06	3	2	2	5	1.04	0.99	0.99	0.81	0.80	1.04	0.94	1.03	0.98	0.92	0.85	0.84	0.78	15.07	10.05
Q9JJV5	Voltage-dependent calcium channel gamma-3 subunit OS=Mus musculus GN=Cacng3 PE=1 SV=2 - [CCG3_MOUSE]	13.97	1	2	4	7	0.95	0.70	0.74	1.08	1.14	1.14	1.20	1.03	1.08	0.87	0.92	0.90	0.95	35.49	9.48
G3X8R5	Glutamine-rich protein 1 OS=Mus musculus GN=Qrich1 PE=4 SV=1 - [G3X8R5_MOUSE]	1.16	2	1	1	3	1.29	0.73	0.57	1.03	0.80	1.12	0.86	1.03	0.79	1.09	0.84	1.12	0.87	86.47	5.87
P97952	Sodium channel subunit beta-1 OS=Mus musculus GN=Scn1b PE=2 SV=1 - [SCN1B_MOUSE]	21.56	1	6	6	23	1.21	1.12	0.98	1.17	1.03	1.08	0.99	1.03	0.87	1.00	0.86	1.08	0.93	24.63	4.83
P62264	40S ribosomal protein S14 OS=Mus musculus GN=Rps14 PE=2 SV=3 - [RS14_MOUSE]	22.52	3	3	3	16	0.90	1.00	0.94	0.96	0.99	0.98	1.07	1.03	0.96	0.99	0.95	1.09	1.08	16.26	10.05
Q9Z204	Heterogeneous nuclear ribonucleoproteins C1/C2 OS=Mus musculus GN=Hnrnpc PE=1 SV=1 - [HNRPC_MOUSE]	46.33	2	1	10	41	0.92	0.57	0.62	0.68	0.73	0.81	0.88	1.03	1.11	1.06	1.16	1.16	1.27	34.36	5.05
D3YU96	Cytohesin-2 OS=Mus musculus GN=Cyth2 PE=2 SV=1 - [D3YU96_MOUSE]	35.51	8	8	12	34	0.97	1.06	1.05	1.08	1.09	1.02	1.01	1.03	1.07	1.00	1.01	1.00	1.05	44.63	6.38
Q9D7H3	RNA 3'-terminal phosphate cyclase OS=Mus musculus GN=RtcA PE=2 SV=2 - [RTCA_MOUSE]	7.38	4	2	2	4	1.02	1.40	1.37	1.13	1.11	0.87	0.85	1.03	1.01	1.01	0.99	1.24	1.23	39.23	7.90
Q8R332-4	Isoform 4 of Nucleoporin p58/p45 OS=Mus musculus GN=Nupl1 - [NUPL1_MOUSE]	7.89	4	4	4	6	0.94	0.98	1.05	0.87	0.93	0.98	1.01	1.03	1.08	0.87	0.94	0.86	0.95	53.97	8.31
Q8BHN1-	Isoform 3 of Gamma-taxilin OS=Mus musculus GN=Txlng - [TXLNG_MOUSE]	3.79	2	1	2	4	1.07	0.92	0.86	0.90	0.83	1.12	1.04	1.03	0.95	0.91	0.85	1.04	0.97	54.98	8.50

E9Q7C0	Protein 1700030K09Rik OS=Mus musculus GN=1700030K09Rik PE=2 SV=1 - [E9Q7C0_MOUSE]	2.31	2	1	1	1	0.80	0.88	1.10	0.93	1.17	1.11	1.38	1.03	1.29	1.05	1.33	1.01	1.27	65.38	5.94
Q01279	Epidermal growth factor receptor OS=Mus musculus GN=Egfr PE=1 SV=1 - [EGFR_MOUSE]	3.80	3	4	4	6	1.02	1.23	1.14	0.99	0.95	0.92	0.85	1.03	0.96	1.03	1.01	1.13	1.06	134.77	6.86
O88844	Isocitrate dehydrogenase [NADP] cytoplasmic OS=Mus musculus GN=Idh1 PE=1 SV=2 - [IDHC_MOUSE]	42.75	2	14	15	50	1.02	0.87	0.90	1.05	1.02	1.11	1.04	1.03	1.01	1.08	1.10	1.10	1.01	46.64	7.17
G5E8V8	Phosphorylated adapter RNA export protein OS=Mus musculus GN=Phax PE=4 SV=1 - [G5E8V8_MOUSE]	25.62	3	7	7	17	1.03	0.97	0.94	1.03	0.86	1.07	0.99	1.03	0.88	0.93	0.83	0.98	0.80	40.84	6.02
D3Z0S1	Annexin (Fragment) OS=Mus musculus GN=Anxa4 PE=2 SV=1 - [D3Z0S1_MOUSE]	19.29	3	3	4	8	1.00	1.11	1.11	1.08	1.08	0.96	0.88	1.03	1.03	1.03	1.10	0.98	0.99	28.53	5.35
Q810A7	ATP-dependent RNA helicase DDX42 OS=Mus musculus GN=Ddx42 PE=1 SV=3 - [DDX42_MOUSE]	18.73	2	11	11	29	0.87	0.89	0.91	0.95	1.08	0.99	1.17	1.03	1.15	0.94	1.14	0.98	1.12	101.90	6.98
Q76LL6-3	Isoform 3 of FH1/FH2 domain-containing protein 3 OS=Mus musculus GN=Fhod3 - [FHOD3_MOUSE]	2.05	4	2	2	12	1.10	1.25	1.18	1.22	1.10	1.11	0.94	1.03	0.93	1.00	0.79	1.16	0.92	158.18	5.83
Q8R5A3	Amyloid beta A4 precursor protein-binding family B member 1-interacting protein OS=Mus musculus GN=Apbb1ip PE=1 SV=2 - [AB1IP_MOUSE]	3.88	1	1	3	4	1.01	0.85	0.84	0.97	0.96	0.92	0.90	1.03	1.01	0.94	0.93	0.86	0.85	74.27	5.35
Q8BME9	Cerebellin-4 OS=Mus musculus GN=Cbln4 PE=1 SV=1 - [CBLN4_MOUSE]	7.07	1	1	1	1	1.16	1.17	1.01	1.05	0.90	0.80	0.68	1.03	0.88	0.94	0.81	0.90	0.77	21.60	8.73
Q62167	ATP-dependent RNA helicase DDX3X OS=Mus musculus GN=Ddx3x PE=1 SV=3 - [DDX3X_MOUSE]	35.20	4	4	19	46	1.01	1.32	1.41	1.05	1.04	0.99	1.03	1.03	1.02	0.92	0.91	1.04	1.00	73.06	7.18
O35874	Neutral amino acid transporter A OS=Mus musculus GN=Slc1a4 PE=1 SV=1 - [SATT_MOUSE]	14.47	2	4	6	41	1.15	1.36	1.14	1.11	1.09	0.95	0.80	1.03	0.91	1.07	0.98	1.08	0.94	56.03	5.87
Q7TMM9	Tubulin beta-2A chain OS=Mus musculus GN=Tubb2a PE=1 SV=1 - [TBB2A_MOUSE]	84.04	2	3	30	1962	1.02	0.85	0.82	1.00	1.01	1.11	1.06	1.03	1.00	1.09	1.07	1.05	1.02	49.87	4.89

E9Q705	BolA-like protein 3 OS=Mus musculus GN=Bola3 PE=2 SV=1 - [E9Q705_MOUSE]	27.37	3	3	3	9	0.73	0.57	0.79	0.85	1.12	1.05	1.42	1.03	1.40	0.91	1.25	0.75	0.87	10.82	8.31
Q3TY86-3	Isoform 3 of Apoptosis-inducing factor 3 OS=Mus musculus GN=Aifm3 - [AIFM3_MOUSE]	10.40	3	4	4	5	0.99	0.89	0.82	1.12	1.00	0.99	1.06	1.03	1.27	0.98	1.07	1.04	1.07	55.00	9.25
Q9D8P7	General transcription factor 3C polypeptide 6 OS=Mus musculus GN=Gtf3c6 PE=2 SV=1 - [TF3C6_MOUSE]	7.05	1	1	1	2	0.89	0.53	0.60	0.88	0.99	1.26	1.41	1.03	1.15	0.95	1.07	0.86	0.96	25.53	4.22
P70333	Heterogeneous nuclear ribonucleoprotein H2 OS=Mus musculus GN=Hnrnph2 PE=1 SV=1 - [HNRH2_MOUSE]	40.98	2	6	13	107	0.93	0.90	0.97	0.98	1.07	0.99	1.07	1.03	1.07	0.97	1.04	0.94	1.07	49.25	6.30
Q91YL3	Uridine-cytidine kinase-like 1 OS=Mus musculus GN=Uckl1 PE=1 SV=1 - [UCKL1_MOUSE]	5.11	2	2	2	4	0.70	0.88	1.26	0.74	1.06	1.17	1.68	1.03	1.47	1.10	1.59	0.87	1.25	60.80	7.15
P86792	C-C motif chemokine 21b OS=Mus musculus GN=Ccl21b PE=1 SV=1 - [CC21B_MOUSE]	13.53	1	1	1	2	0.92	0.55	0.59	0.90	0.97	0.84	0.90	1.03	1.11	1.05	1.14	1.08	1.17	14.57	9.88
P97447	Four and a half LIM domains protein 1 OS=Mus musculus GN=Fhl1 PE=2 SV=3 - [FHL1_MOUSE]	44.29	9	10	10	89	1.16	0.88	0.77	0.91	0.80	1.17	1.03	1.03	0.90	1.08	0.97	1.02	0.93	31.87	8.37
P20801	Troponin C, skeletal muscle OS=Mus musculus GN=Tnnc2 PE=1 SV=2 - [TNNC2_MOUSE]	22.50	1	2	3	5	0.97	1.53	1.58	0.75	0.77	11.39	11.30	1.03	0.96	1.20	1.07	1.03	1.13	18.10	4.20
P58389	Serine/threonine-protein phosphatase 2A activator OS=Mus musculus GN=Ppp2r4 PE=1 SV=1 - [PTPA_MOUSE]	13.62	4	3	3	7	1.10	1.24	0.98	1.15	1.04	0.98	0.85	1.03	0.89	1.08	0.92	1.09	1.04	36.69	6.39
Q8BHN0	Protein phosphatase 1L OS=Mus musculus GN=Ppm1l PE=1 SV=1 - [PPM1L_MOUSE]	14.44	3	4	4	7	0.94	1.10	1.18	1.09	1.16	1.00	1.29	1.03	1.10	1.02	1.17	0.94	0.96	41.02	5.99
Q3V0I2	Proline-rich protein 7 OS=Mus musculus GN=Prr7 PE=2 SV=1 - [PRR7_MOUSE]	18.22	1	3	3	5	1.16	1.05	1.01	1.04	1.03	1.04	0.91	1.03	0.96	0.99	0.88	1.24	1.10	30.33	8.60
Q3UHG7-	Isoform 2 of Protein LCHN OS=Mus musculus GN=Lchn - [LCHN_MOUSE]	3.58	2	1	1	2	1.10	1.46	1.33	1.26	1.15	0.94	0.85	1.03	0.94	1.06	0.97	1.09	0.99	44.23	5.58
Q8C729	Protein FAM126B OS=Mus musculus GN=Fam126b PE=1 SV=1 - [F126B_MOUSE]	14.72	3	7	7	16	1.02	0.94	0.96	1.05	1.09	0.95	1.00	1.03	1.07	0.98	1.05	0.95	1.06	58.55	7.72

Q5DTT1	Uncharacterized protein KIAA2022 OS=Mus musculus GN=Kiaa2022 PE=2 SV=3 - [K2022_MOUSE]	0.66	1	1	1	1	1.50	0.80	0.54	0.94	0.63	1.14	0.76	1.03	0.68	1.03	0.69	1.25	0.84	167.00	5.97
D3YTS3	Protein D630045J12Rik OS=Mus musculus GN=D630045J12Rik PE=2 SV=1 - [D3YTS3_MOUSE]	10.60	2	2	12	28	1.02	1.08	1.08	1.13	1.09	1.00	0.96	1.03	0.99	1.01	0.97	0.89	0.84	179.10	6.34
Q9QWH1	Polyhomeotic-like protein 2 OS=Mus musculus GN=Phc2 PE=1 SV=1 - [PHC2_MOUSE]	7.65	3	3	3	7	1.14	1.07	0.94	0.95	0.76	1.25	1.07	1.03	0.89	0.98	0.80	1.02	0.83	89.74	8.75
Q59J78	Mimitin, mitochondrial OS=Mus musculus GN=Ndufaf2 PE=2 SV=1 - [MIMIT_MOUSE]	28.57	1	4	4	9	0.96	0.97	1.04	1.01	1.04	0.98	1.00	1.03	1.00	0.90	0.96	0.78	0.90	19.62	8.25
P28661-2	Isoform 2 of Septin-4 OS=Mus musculus GN=Sept4 - [SEPT4_MOUSE]	48.80	9	15	16	51	0.91	0.90	1.07	1.00	1.06	1.01	1.10	1.03	1.13	0.95	1.04	0.98	1.10	52.96	6.18
Q8CHG7	Rap guanine nucleotide exchange factor 2 OS=Mus musculus GN=Rapgef2 PE=1 SV=2 - [RPGF2_MOUSE]	29.08	6	27	27	61	0.99	0.98	0.95	1.05	1.08	1.03	1.07	1.03	1.07	1.04	1.09	1.07	1.08	166.31	6.61
Q6DFV5	Probable helicase with zinc finger domain OS=Mus musculus GN=Helz PE=1 SV=2 - [HELZ_MOUSE]	2.04	5	3	3	10	1.05	1.12	1.06	1.02	0.97	1.05	1.01	1.03	1.02	0.96	0.92	0.97	0.92	219.74	7.40
B7ZC24	Nuclear receptor coactivator 5 OS=Mus musculus GN=Ncoa5 PE=2 SV=1 - [B7ZC24_MOUSE]	15.02	1	1	5	12	0.92	1.32	1.42	0.96	1.04	0.92	0.99	1.03	1.11	1.00	1.09	1.02	1.11	48.05	9.69
F8WIE5	E3 ubiquitin-protein ligase HECTD1 OS=Mus musculus GN=Hectd1 PE=2 SV=1 - [F8WIE5_MOUSE]	3.68	2	6	6	11	1.00	1.19	1.20	1.07	1.13	0.94	1.01	1.03	0.89	0.98	0.99	1.04	0.96	289.05	5.36
E9Q2A6	Protein-tyrosine kinase 2-beta OS=Mus musculus GN=Ptk2b PE=2 SV=1 - [E9Q2A6_MOUSE]	39.61	4	31	32	91	0.98	0.94	0.97	1.01	1.01	1.07	1.04	1.03	0.98	1.05	1.05	0.99	0.99	111.03	6.04
Q8BLS7	Ankyrin repeat domain-containing protein SOWAHA OS=Mus musculus GN=Sowaha PE=2 SV=2 - [SWAHA_MOUSE]	1.82	1	1	1	2	1.01	1.15	1.13	1.05	1.03	1.03	1.02	1.03	1.01	1.02	1.01	1.25	1.24	57.71	9.82
Q923G2	DNA-directed RNA polymerases I, II, and III subunit RPABC3 OS=Mus musculus GN=Polr2h PE=2 SV=3 - [RPAB3_MOUSE]	26.67	1	3	3	5	1.10	1.26	1.04	0.99	0.87	1.00	0.91	1.03	0.93	1.00	0.84	1.00	0.91	17.13	4.68

Q99L13	3-hydroxyisobutyrate dehydrogenase, mitochondrial OS=Mus musculus GN=Hibadh PE=2 SV=1 - [3HIDH_MOUSE]	46.57	1	11	11	34	0.90	1.06	1.04	0.99	1.01	1.01	1.12	1.03	1.13	1.01	1.12	1.03	1.06	35.42	8.13
P01887	Beta-2-microglobulin OS=Mus musculus GN=B2m PE=1 SV=2 - [B2MG_MOUSE]	59.66	1	4	4	9	0.98	0.87	0.92	0.85	0.95	1.05	1.06	1.03	1.19	0.98	1.08	0.93	0.77	13.77	8.44
E9PXJ0	Neuronal tyrosine-phosphorylated phosphoinositide-3-kinase adapter 1 (Fragment) OS=Mus musculus GN=Nyap1 PE=2 SV=1 - [E9PXJ0_MOUSE]	5.32	4	3	3	6	0.91	1.04	1.16	1.04	1.08	1.02	1.15	1.03	1.15	1.09	1.19	1.15	1.26	82.75	9.19
Q91UZ1	Phospholipase C beta 4 OS=Mus musculus GN=Plcb4 PE=2 SV=1 - [Q91UZ1_MOUSE]	18.64	3	19	19	45	0.95	0.87	0.88	0.95	1.02	1.00	1.01	1.03	1.07	0.96	0.97	0.88	0.87	134.44	6.90
D3YVZ8	Zinc finger protein Pegasus (Fragment) OS=Mus musculus GN=Ikzf5 PE=2 SV=1 - [D3YVZ8_MOUSE]	45.00	4	1	1	1	1.02	0.68	0.67	0.89	0.88	1.15	1.12	1.03	1.01	0.89	0.87	0.80	0.79	2.41	4.61
Q9DCC8	Mitochondrial import receptor subunit TOM20 homolog OS=Mus musculus GN=Tomm20 PE=1 SV=1 - [TOM20_MOUSE]	27.59	1	3	3	11	1.10	0.96	0.88	0.98	0.97	1.08	1.08	1.03	1.01	1.13	1.01	1.06	1.05	16.27	8.60
Q5DU25	IQ motif and SEC7 domain-containing protein 2 OS=Mus musculus GN=Iqsec2 PE=1 SV=3 - [IQEC2_MOUSE]	12.45	5	12	14	52	1.08	1.07	1.08	1.14	1.07	1.03	0.95	1.03	0.99	0.99	0.95	1.03	0.97	161.69	8.56
Q3TXX3-	Isoform 2 of Protrudin OS=Mus musculus GN=Zfyve27 - [ZFY27_MOUSE]	2.94	2	1	1	2	0.94	1.08	1.15	0.97	1.03	0.93	0.98	1.03	1.09	1.03	1.10	1.13	1.21	45.38	5.35
Q00899	Transcriptional repressor protein YY1 OS=Mus musculus GN=Yy1 PE=1 SV=1 - [TYY1_MOUSE]	15.70	2	5	5	13	1.07	0.78	0.67	1.01	0.97	1.18	1.07	1.03	1.03	1.01	0.95	0.95	0.93	44.69	6.29
Q80TN7	Neuron navigator 3 OS=Mus musculus GN=Nav3 PE=1 SV=2 - [NAV3_MOUSE]	3.31	5	6	6	9	1.00	1.08	1.04	1.05	1.01	1.07	1.00	1.03	1.01	0.97	0.97	0.87	0.86	252.15	8.76
O08576	RUN domain-containing protein 3A OS=Mus musculus GN=Rundc3a PE=1 SV=1 - [RUN3A_MOUSE]	18.61	1	1	5	7	1.14	1.28	1.12	0.99	0.86	1.07	0.94	1.03	0.90	0.91	0.80	1.02	0.90	49.99	5.50

Q8K093	Thyrotropin-releasing hormone-degrading ectoenzyme OS=Mus musculus GN=Trhde PE=2 SV=1 - [TRHDE_MOUSE]	1.56	1	1	1	1	1.03	0.61	0.60	0.99	0.96	0.95	0.92	1.03	1.00	1.11	1.08	1.19	1.16	117.38	7.06
Q6QWF9	Calcium/calmodulin-dependent protein kinase II inhibitor 1 OS=Mus musculus GN=Camk2n1 PE=1 SV=1 - [CK2N1_MOUSE]	57.69	1	2	4	42	0.97	0.77	0.80	0.88	0.88	1.05	1.11	1.03	1.11	1.01	0.98	0.86	0.87	8.51	5.45
P14873	Microtubule-associated protein 1B OS=Mus musculus GN=Map1b PE=1 SV=2 - [MAP1B_MOUSE]	62.99	1	126	127	1470	1.05	0.88	0.85	0.95	0.90	1.06	1.01	1.03	0.99	1.02	0.97	0.99	0.95	270.09	4.83
Q8BZM0-	Isoform 2 of Kelch-like protein 12 OS=Mus musculus GN=Klhl12 - [KLH12_MOUSE]	4.07	2	1	1	1	1.67	7.35	4.39	1.27	0.76	1.57	0.93	1.03	0.61	1.31	0.79	1.03	0.62	60.53	5.44
O55091	Protein IMPACT OS=Mus musculus GN=Impact PE=1 SV=2 - [IMPCT_MOUSE]	52.20	2	13	13	42	1.05	0.93	0.92	0.99	0.93	0.97	0.88	1.03	0.90	1.02	0.94	1.10	1.02	36.25	5.05
B9EKR1	Protein Ptprz1 OS=Mus musculus GN=Ptprz1 PE=2 SV=1 - [B9EKR1_MOUSE]	13.80	1	29	29	486	1.04	0.89	0.86	0.90	0.88	1.05	1.01	1.03	0.98	0.93	0.88	0.90	0.86	254.25	4.88
Q9WV85	Nucleoside diphosphate kinase 3 OS=Mus musculus GN=Nme3 PE=2 SV=3 - [NDK3_MOUSE]	23.08	1	3	3	7	1.00	0.97	0.94	1.18	1.26	1.03	1.11	1.03	1.05	1.04	1.05	1.11	1.11	19.09	6.68
Q8VI51	VPS10 domain-containing receptor SorCS3 OS=Mus musculus GN=Sorcs3 PE=1 SV=1 - [SORC3_MOUSE]	11.40	1	10	10	17	1.11	1.04	0.94	1.01	0.96	0.99	0.90	1.03	1.07	1.04	1.03	0.92	0.95	135.90	6.49
P14733	Lamin-B1 OS=Mus musculus GN=Lmnb1 PE=1 SV=3 - [LMNB1_MOUSE]	52.89	2	27	31	146	0.95	0.98	1.02	0.89	0.96	1.01	1.06	1.03	1.10	0.97	1.04	0.93	1.02	66.74	5.16
Q8BGR8	GSK3-beta interaction protein OS=Mus musculus GN=Gskip PE=2 SV=1 - [GSKIP_MOUSE]	67.63	2	5	5	9	1.11	0.78	0.80	0.95	0.87	1.01	0.90	1.03	1.12	0.96	0.81	0.92	0.86	15.63	4.41
Q62318	Transcription intermediary factor 1-beta OS=Mus musculus GN=Trim28 PE=1 SV=3 - [TIF1B_MOUSE]	34.17	2	16	16	57	0.97	1.04	1.06	0.98	0.99	1.02	1.10	1.03	1.09	1.05	1.04	1.05	1.09	88.79	5.77
O35114	Lysosome membrane protein 2 OS=Mus musculus GN=Scarb2 PE=1 SV=3 - [SCRB2_MOUSE]	6.28	1	2	2	3	1.00	1.03	1.03	1.11	1.11	0.92	0.92	1.03	1.03	1.02	1.02	0.98	0.99	54.01	5.10

Q8K327	Chromosome alignment-maintaining phosphoprotein 1 OS=Mus musculus GN=Champ1 PE=1 SV=1 - [CHAP1_MOUSE]	20.82	1	13	13	21	0.97	0.85	0.90	0.87	0.89	1.07	1.11	1.03	1.11	0.98	1.03	1.00	1.01	87.51	7.85
H7BWY4	Disks large homolog 1 OS=Mus musculus GN=Dlg1 PE=2 SV=1 - [H7BWY4_MOUSE]	40.48	7	21	28	91	0.95	0.89	0.98	0.97	1.05	1.03	1.07	1.03	1.06	0.96	1.00	0.92	0.97	96.83	5.90
Q3TZU5	Protein kinase inhibitor beta, cAMP dependent, testis specific, isoform CRA_c OS=Mus musculus GN=Pkib PE=4 SV=1 - [Q3TZU5_MOUSE]	64.10	4	5	5	9	1.18	0.91	0.72	1.06	0.78	1.29	0.98	1.03	0.79	1.13	0.97	1.28	1.10	8.33	5.15
O88935	Synapsin-1 OS=Mus musculus GN=Syn1 PE=1 SV=2 - [SYN1_MOUSE]	83.71	3	42	44	1054	0.97	0.94	0.95	0.98	1.00	1.03	1.05	1.03	1.04	0.98	1.01	0.99	1.02	74.05	9.80
Q9R1X5	Multidrug resistance-associated protein 5 OS=Mus musculus GN=Abcc5 PE=1 SV=2 - [MRP5_MOUSE]	0.84	1	1	1	1	0.99	0.86	0.86	0.86	0.87	1.21	1.21	1.03	1.04	0.88	0.89	1.06	1.07	161.02	8.60
P63250	G protein-activated inward rectifier potassium channel 1 OS=Mus musculus GN=Kcnj3 PE=1 SV=1 - [IRK3_MOUSE]	8.58	1	4	4	6	0.86	1.06	1.14	0.99	1.15	0.90	0.93	1.03	1.13	0.88	1.08	0.82	0.89	56.54	8.37
P60766-1	Isoform 1 of Cell division control protein 42 homolog OS=Mus musculus GN=Cdc42 - [CDC42_MOUSE]	49.74	8	3	10	51	1.10	1.05	0.91	1.22	1.23	0.90	0.87	1.03	0.93	0.92	0.94	1.11	0.95	21.30	6.04
O54962	Barrier-to-autointegration factor OS=Mus musculus GN=Banf1 PE=1 SV=1 - [BAF_MOUSE]	49.44	1	3	3	24	0.96	0.81	0.85	0.89	0.89	1.04	1.02	1.03	1.12	1.02	1.04	0.92	1.06	10.10	6.09
O70194	Eukaryotic translation initiation factor 3 subunit D OS=Mus musculus GN=Eif3d PE=1 SV=2 - [EIF3D_MOUSE]	17.70	1	8	8	17	1.01	0.90	0.95	1.00	1.01	1.11	1.08	1.03	1.08	1.00	0.97	0.96	1.03	63.95	6.05
Q3UI43	BRISC and BRCA1-A complex member 1 OS=Mus musculus GN=Babam1 PE=2 SV=1 - [BABA1_MOUSE]	37.84	1	7	7	27	1.01	0.86	0.93	0.81	0.86	0.96	0.97	1.03	1.03	0.88	0.89	0.87	0.95	36.77	4.55
D3YWX2	YLP motif-containing protein 1 OS=Mus musculus GN=Ylpm1 PE=2 SV=2 - [D3YWX2_MOUSE]	23.33	8	36	37	101	0.98	0.95	0.99	0.95	0.97	1.03	1.08	1.03	1.05	0.95	0.96	0.92	0.96	240.90	6.68

B0R091	Protein Gm20056 OS=Mus musculus GN=Chp1 PE=2 SV=1 - [B0R091_MOUSE]	69.11	3	12	12	25	0.93	0.92	0.96	1.00	0.91	1.07	1.03	1.03	1.03	1.03	0.98	0.97	0.99	21.98	4.93
Q9QXN0-	Isoform 4 of Protein Shroom3 OS=Mus musculus GN=Shroom3 - [SHRM3_MOUSE]	1.83	9	1	3	9	0.93	1.25	1.35	1.08	1.16	0.99	1.07	1.03	1.11	1.03	1.12	0.95	1.02	195.32	7.90
P09813	Apolipoprotein A-II OS=Mus musculus GN=Apoa2 PE=1 SV=2 - [APOA2_MOUSE]	8.82	1	1	1	2	0.62	0.95	1.54	0.57	0.93	0.75	1.21	1.03	1.67	1.73	2.81	1.05	1.71	11.30	7.18
Q9JJA7-3	Isoform 3 of Cyclin-L2 OS=Mus musculus GN=Ccnl2 - [CCNL2_MOUSE]	6.88	3	1	1	1	1.02	1.14	1.11	0.84	0.82	1.24	1.20	1.03	1.01	0.99	0.97	0.88	0.86	23.99	9.01
A2A6U3	Septin-9 OS=Mus musculus GN=Sept9 PE=2 SV=1 - [A2A6U3_MOUSE]	43.89	6	20	21	54	0.90	1.03	1.13	0.98	1.03	1.02	1.02	1.03	1.12	0.94	1.03	0.91	0.99	63.73	8.32
Q9D1D4	Transmembrane emp24 domain-containing protein 10 OS=Mus musculus GN=Tmed10 PE=2 SV=1 - [TMEDA_MOUSE]	31.05	2	6	6	18	0.95	0.88	0.92	0.98	1.02	1.12	1.04	1.03	1.09	0.94	0.97	0.95	0.94	24.89	6.70
D3YWG7	DCN1-like protein 3 (Fragment) OS=Mus musculus GN=Dcun1d3 PE=2 SV=1 - [D3YWG7_MOUSE]	18.79	2	2	2	7	1.04	1.02	0.92	0.94	0.93	0.96	0.89	1.03	0.93	0.93	0.87	0.98	0.91	18.20	6.55
P58929	Glucocorticoid modulatory element-binding protein 2 OS=Mus musculus GN=Gmeb2 PE=2 SV=2 - [GMEB2_MOUSE]	1.70	1	1	1	1	0.94	1.32	1.40	0.85	0.91	0.93	0.99	1.03	1.10	1.04	1.11	1.09	1.17	56.60	5.31
Q9CQI7	U2 small nuclear ribonucleoprotein B'' OS=Mus musculus GN=Snrpb2 PE=2 SV=1 - [RU2B_MOUSE]	16.00	2	2	3	9	1.04	1.05	1.18	0.80	0.89	0.99	0.95	1.03	0.99	0.99	0.86	0.97	0.94	25.31	9.72
Q9R1K9	Centrin-2 OS=Mus musculus GN=Cetn2 PE=1 SV=1 - [CETN2_MOUSE]	68.02	3	11	11	44	1.12	0.93	0.86	0.94	0.79	1.14	0.97	1.03	0.91	1.15	0.96	0.96	0.94	19.78	5.00
Q9CPQ9	Fructose-bisphosphate aldolase OS=Mus musculus GN=Aldoart1 PE=2 SV=1 - [Q9CPQ9_MOUSE]	52.20	3	1	18	397	1.23	0.61	0.49	1.44	1.17	0.97	0.78	1.03	0.84	1.21	0.98	1.09	0.88	39.26	7.37
O08997	Copper transport protein ATOX1 OS=Mus musculus GN=Atox1 PE=2 SV=1 - [ATOX1_MOUSE]	58.82	1	4	4	97	1.11	1.01	0.85	0.96	0.89	1.16	1.03	1.03	0.96	0.98	0.95	0.95	0.89	7.33	6.51

P24369	Peptidyl-prolyl cis-trans isomerase B OS=Mus musculus GN=Ppib PE=2 SV=2 - [PPIB_MOUSE]	49.54	2	10	11	43	1.00	0.82	0.81	0.98	0.96	1.13	1.16	1.03	1.05	0.99	1.03	1.03	1.03	23.70	9.55
P10639	Thioredoxin OS=Mus musculus GN=Txn PE=1 SV=3 - [THIO_MOUSE]	65.71	1	7	7	82	1.04	0.84	0.79	0.96	0.91	1.07	1.03	1.03	1.01	0.93	0.91	0.95	0.91	11.67	4.92
Q6NZR5	Protein Skiv2l OS=Mus musculus GN=Skiv2l PE=2 SV=1 - [Q6NZR5_MOUSE]	3.62	1	3	3	10	0.95	1.14	0.99	0.98	0.99	1.00	1.05	1.03	0.96	1.04	1.09	1.05	1.10	137.44	6.28
Q9D902	General transcription factor IIE subunit 2 OS=Mus musculus GN=Gtf2e2 PE=2 SV=2 - [T2EB_MOUSE]	9.59	1	2	2	5	0.95	0.78	0.82	1.01	1.07	1.06	1.11	1.03	1.04	1.19	1.18	0.96	0.97	33.03	9.64
Q9CZY3	Ubiquitin-conjugating enzyme E2 variant 1 OS=Mus musculus GN=Ube2v1 PE=1 SV=1 - [UB2V1_MOUSE]	44.90	7	1	5	72	0.97	1.20	1.23	1.05	1.06	0.98	0.89	1.03	1.04	1.00	0.99	1.03	1.06	16.34	7.96
Q9Z2Q6	Septin-5 OS=Mus musculus GN=Sept5 PE=1 SV=2 - [SEPT5_MOUSE]	44.99	1	15	15	146	1.00	0.90	0.87	1.04	1.04	1.08	1.06	1.03	1.03	1.00	0.97	1.05	1.05	42.72	6.67
Q9CXX9	CUE domain-containing protein 2 OS=Mus musculus GN=Cuedc2 PE=2 SV=1 - [CUED2_MOUSE]	23.24	1	6	6	29	1.03	0.87	0.79	0.96	0.89	1.17	1.11	1.03	1.00	0.99	0.93	0.91	0.90	31.83	4.98
Q9WV38	Solute carrier family 2, facilitated glucose transporter member 5 OS=Mus musculus GN=Slc2a5 PE=2 SV=2 - [GTR5_MOUSE]	2.99	1	1	1	2	1.12	1.02	0.91	0.97	0.86	1.03	0.91	1.03	0.92	1.10	0.98	1.01	0.90	55.37	5.31
Q9D114	Guanosine-3',5'-bis(diphosphate) 3'-pyrophosphohydrolase MESH1 OS=Mus musculus GN=Hddc3 PE=2 SV=1 - [MESH1_MOUSE]	7.82	1	1	1	2	0.87	0.77	0.88	0.87	1.00	0.97	1.12	1.03	1.19	0.89	1.03	0.80	0.92	20.25	5.96
P99024	Tubulin beta-5 chain OS=Mus musculus GN=Tubb5 PE=1 SV=1 - [TBB5_MOUSE]	75.23	2	4	29	1915	1.01	1.06	1.03	1.03	1.03	1.05	1.04	1.03	1.02	1.07	1.08	1.11	1.10	49.64	4.89
Q6PAM0	5'-AMP-activated protein kinase subunit beta-2 OS=Mus musculus GN=Prkab2 PE=1 SV=1 - [AAKB2_MOUSE]	19.19	3	3	3	11	0.89	1.02	1.07	1.06	1.07	1.05	1.14	1.03	1.11	0.93	1.02	0.90	0.99	30.19	6.46

Q9D7A8	Armadillo repeat-containing protein 1 OS=Mus musculus GN=Armc1 PE=2 SV=1 - [ARMC1_MOUSE]	22.70	1	4	4	10	0.96	1.30	1.31	0.90	0.97	0.97	0.94	1.03	1.13	0.98	0.87	1.04	1.13	31.23	5.57
G3X8R2	Cytoplasmic FMR1-interacting protein 1 (Fragment) OS=Mus musculus GN=Sra1 PE=4 SV=1 - [G3X8R2_MOUSE]	39.01	2	5	5	13	0.92	0.83	0.85	0.91	0.97	1.03	1.10	1.03	1.04	0.94	0.87	0.94	1.00	24.59	6.02
A2AKF0	N-terminal EF-hand calcium-binding protein 3 (Fragment) OS=Mus musculus GN=Necab3 PE=2 SV=1 - [A2AKF0_MOUSE]	16.67	3	1	1	3	0.85	0.90	1.06	0.72	0.85	0.99	1.16	1.03	1.22	0.97	1.15	0.93	1.10	18.44	7.77
Q9Z1K5	E3 ubiquitin-protein ligase ARIH1 OS=Mus musculus GN=Arih1 PE=2 SV=3 - [ARI1_MOUSE]	12.61	3	6	6	9	1.02	1.11	1.08	0.98	1.00	0.90	0.89	1.03	1.00	1.02	1.04	0.92	1.02	63.97	5.08
Q8R0P4	Mth938 domain-containing protein OS=Mus musculus GN=Aamdc PE=1 SV=1 - [AAMDC_MOUSE]	40.16	9	5	5	15	1.02	1.05	1.02	0.96	0.94	1.09	1.06	1.03	1.05	1.04	1.00	1.01	1.00	13.24	7.99
Q9QX96	Sal-like protein 2 OS=Mus musculus GN=Sall2 PE=2 SV=2 - [SALL2_MOUSE]	1.39	2	1	1	1	1.18	1.25	1.07	0.93	0.79	1.29	1.09	1.03	0.88	1.04	0.89	1.08	0.92	104.88	6.28
G3X972	Protein Sec24c OS=Mus musculus GN=Sec24c PE=4 SV=1 - [G3X972_MOUSE]	12.23	1	10	10	19	1.06	1.23	1.20	1.17	1.17	1.03	0.98	1.03	0.94	0.96	0.92	1.03	0.95	118.51	6.84
A2AF31	Protein Tmsb15b2 OS=Mus musculus GN=Tmsb15b2 PE=2 SV=1 - [A2AF31_MOUSE]	48.89	3	2	2	189	1.05	0.73	0.68	0.88	0.93	1.19	1.07	1.03	1.03	1.01	0.96	0.99	0.90	5.24	6.00
Q9QYK9	Calcium/calmodulin-dependent protein kinase type 1B OS=Mus musculus GN=Pnck PE=2 SV=1 - [KCC1B_MOUSE]	7.58	2	2	2	6	1.14	1.27	0.92	1.05	0.92	1.09	0.94	1.03	0.79	1.26	0.99	1.24	1.06	38.49	6.47
Q91ZS8-5	Isoform 5 of Double-stranded RNA-specific editase 1 OS=Mus musculus GN=Adarb1 - [RED1_MOUSE]	1.62	5	1	1	1	1.08	1.33	1.23	1.08	1.00	1.16	1.07	1.03	0.96	1.09	1.01	1.44	1.34	74.12	9.35
P70208	Plexin-A3 OS=Mus musculus GN=Plxna3 PE=1 SV=2 - [PLXA3_MOUSE]	4.11	2	1	7	11	0.95	1.05	1.10	0.95	1.00	1.11	1.16	1.03	1.08	1.09	1.15	1.13	1.19	207.83	7.09
Q9WTR6	Cystine/glutamate transporter OS=Mus musculus GN=Slc7a11 PE=1 SV=1 - [XCT_MOUSE]	7.77	1	3	3	6	1.19	1.06	0.77	1.14	0.93	1.00	0.75	1.03	0.90	1.17	1.02	1.32	0.90	55.42	9.23

Q9EPL2	Calsyntenin-1 OS=Mus musculus GN=Clstn1 PE=1 SV=1 - [CSTN1_MOUSE]	25.54	2	18	18	46	0.99	0.80	0.83	0.95	0.98	1.03	1.02	1.03	1.08	0.93	0.94	0.83	0.88	108.83	4.92
Q8BGC0	HIV Tat-specific factor 1 homolog OS=Mus musculus GN=Htatsf1 PE=1 SV=1 - [HTSF1_MOUSE]	30.12	2	19	19	65	1.04	1.04	1.05	0.92	0.89	1.06	1.02	1.03	1.05	0.89	0.91	0.94	0.90	86.19	4.40
Q9R062	Glycogenin-1 OS=Mus musculus GN=Gyg1 PE=2 SV=3 - [GLYG_MOUSE]	26.43	3	5	5	8	1.10	1.15	0.94	1.14	0.99	1.01	0.90	1.03	0.90	1.01	0.87	0.99	0.87	37.38	5.29
Q9DCS9	NADH dehydrogenase [ubiquinone] 1 beta subcomplex subunit 10 OS=Mus musculus GN=Ndufb10 PE=1 SV=3 - [NDUBA_MOUSE]	53.98	2	10	10	54	1.07	0.87	0.85	0.97	0.89	0.98	0.93	1.03	1.00	0.93	0.84	0.98	0.95	21.01	8.03
Q9D1F4	Proline-rich AKT1 substrate 1 OS=Mus musculus GN=Akt1s1 PE=1 SV=1 - [AKTS1_MOUSE]	36.58	6	2	5	30	1.03	0.78	0.76	0.92	0.91	1.02	1.02	1.03	1.00	0.96	0.94	0.84	0.99	27.47	4.72
Q3UMF0-	Isoform 3 of Cordon-bleu protein-like 1 OS=Mus musculus GN=Cobll1 - [COBL1_MOUSE]	13.48	9	11	11	25	1.00	0.99	1.01	0.88	0.93	1.00	1.01	1.04	1.02	0.97	0.94	0.96	0.98	129.37	7.88
P53996-2	Isoform 2 of Cellular nucleic acid-binding protein OS=Mus musculus GN=Cnbp - [CNBP_MOUSE]	37.06	3	1	5	42	0.99	1.05	1.04	1.02	1.00	1.12	1.05	1.04	0.99	1.06	1.02	0.96	0.92	18.73	7.71
B8QI34	Liprin-alpha 2 OS=Mus musculus GN=Ppfia2 PE=2 SV=1 - [B8QI34_MOUSE]	23.63	2	16	23	55	1.10	1.02	0.98	0.94	0.90	1.07	0.99	1.04	0.97	1.03	0.96	1.13	1.04	143.24	6.11
F6R8U9	Sulfatase-modifying factor 2 (Fragment) OS=Mus musculus GN=Sumf2 PE=4 SV=1 - [F6R8U9_MOUSE]	12.39	2	2	2	6	1.10	0.94	0.96	0.92	0.87	1.05	0.83	1.04	0.88	1.00	0.88	1.07	1.07	25.63	7.44
B2RXS4	Plexin-B2 OS=Mus musculus GN=Plxnb2 PE=1 SV=1 - [PLXB2_MOUSE]	9.17	1	11	11	18	0.99	1.05	1.09	0.98	0.94	0.93	0.91	1.04	1.01	0.95	0.92	1.06	1.03	206.10	5.87
Q99LR1	Monoacylglycerol lipase ABHD12 OS=Mus musculus GN=Abhd12 PE=1 SV=2 - [ABD12_MOUSE]	16.58	4	5	5	14	1.03	1.11	1.05	1.10	1.14	0.92	0.89	1.04	1.01	1.03	1.00	0.96	0.95	45.24	8.72
Q9D9I4	TBC1 domain family member 20 OS=Mus musculus GN=Tbc1d20 PE=2 SV=1 - [TBC20_MOUSE]	6.47	1	2	2	4	0.95	1.11	1.16	1.16	1.22	1.03	1.08	1.04	1.08	1.19	1.25	1.04	1.09	45.80	6.96
O70318	Band 4.1-like protein 2 OS=Mus musculus GN=Epb41l2 PE=1 SV=2 - [E41L2_MOUSE]	36.94	1	28	32	122	0.99	0.98	0.95	0.97	0.98	1.05	1.01	1.04	0.97	1.03	1.02	0.98	0.98	109.87	5.43

G3UXN4	KN motif and ankyrin repeat domain-containing protein 3 OS=Mus musculus GN=Kank3 PE=2 SV=1 - [G3UXN4_MOUSE]	4.81	2	2	2	4	0.95	1.03	1.08	0.96	1.01	1.09	1.14	1.04	1.09	1.20	1.26	1.05	1.11	64.34	4.84
E9PXX7	Thioredoxin domain-containing protein 5 OS=Mus musculus GN=Txndc5 PE=2 SV=1 - [E9PXX7_MOUSE]	57.27	2	15	15	61	1.08	0.90	0.91	0.92	0.89	1.03	0.97	1.04	0.95	0.96	0.90	0.98	0.91	38.51	5.39
P63054	Purkinje cell protein 4 OS=Mus musculus GN=Pcp4 PE=2 SV=2 - [PCP4_MOUSE]	75.81	2	7	7	383	0.91	0.82	0.90	0.86	0.94	1.08	1.18	1.04	1.12	1.00	1.08	0.95	1.06	6.80	6.71
Q61548-3	Isoform 3 of Clathrin coat assembly protein AP180 OS=Mus musculus GN=Snap91 - [AP180_MOUSE]	32.49	8	22	24	227	1.01	0.92	0.90	1.03	1.00	1.02	0.98	1.04	1.03	1.05	1.04	1.09	1.01	88.77	4.91
Q99JB2	Stomatin-like protein 2, mitochondrial OS=Mus musculus GN=Stoml2 PE=1 SV=1 - [STML2_MOUSE]	30.03	4	8	8	16	1.00	1.17	1.27	0.97	0.96	1.12	1.16	1.04	1.05	1.11	1.15	1.00	0.99	38.36	8.87
G5E8G3	MCG9827 OS=Mus musculus GN=Opcml PE=4 SV=1 - [G5E8G3_MOUSE]	54.20	1	1	18	218	1.05	0.99	0.95	1.03	0.97	1.11	0.98	1.04	0.95	0.94	0.96	1.03	0.91	37.99	6.87
D3Z0L4	Coiled-coil-helix-coiled-coil-helix domain-containing protein 3, mitochondrial (Fragment) OS=Mus musculus GN=Chchd3 PE=2 SV=1 - [D3Z0L4_MOUSE]	43.75	2	1	11	117	0.82	0.75	0.84	0.83	0.98	1.05	1.26	1.04	1.30	0.92	1.11	0.87	1.02	22.44	6.42
F6SPK0	Ubiquitin-conjugating enzyme E2 J1 (Fragment) OS=Mus musculus GN=Ube2j1 PE=4 SV=1 - [F6SPK0_MOUSE]	7.63	1	1	1	2	1.13	1.18	1.05	1.07	0.95	1.09	0.97	1.04	0.92	0.95	0.84	1.11	0.99	28.90	7.25
Q8CF89	TGF-beta-activated kinase 1 and MAP3K7-binding protein 1 OS=Mus musculus GN=Tab1 PE=1 SV=2 - [TAB1_MOUSE]	1.79	1	1	1	2	0.98	1.02	1.03	1.09	1.11	0.99	1.00	1.04	1.05	0.96	0.98	0.99	1.01	54.58	5.52
Q5SV85-2	Isoform 2 of Synergin gamma OS=Mus musculus GN=Synrg - [SYNRG_MOUSE]	32.43	3	25	26	79	1.08	0.94	0.85	0.97	0.92	1.08	1.03	1.04	0.99	1.05	0.99	1.06	0.96	122.01	5.00

D3Z396	Neurotrimin OS=Mus musculus GN=Ntm PE=2 SV=1 - [D3Z396_MOUSE]	45.57	5	10	16	485	0.99	1.01	1.00	1.03	1.03	1.04	1.04	1.04	1.04	0.96	0.95	0.92	0.93	34.93	6.96
Q71M36	Chondroitin sulfate proteoglycan 5 OS=Mus musculus GN=Cspg5 PE=1 SV=2 - [CSPG5_MOUSE]	19.43	7	9	9	64	0.93	1.08	1.13	1.00	1.07	1.08	1.13	1.04	1.13	1.00	1.08	0.96	1.01	60.37	4.54
Q5NBX1-	Isoform 2 of Protein cordon-bleu OS=Mus musculus GN=Cobl - [COBL_MOUSE]	9.80	11	9	9	25	0.98	1.07	1.07	0.95	1.01	0.99	1.02	1.04	1.05	0.95	1.02	0.89	0.96	135.44	8.21
Q9WV03	Protein FAM50A OS=Mus musculus GN=Fam50a PE=2 SV=1 - [FA50A_MOUSE]	14.45	2	4	4	7	0.99	0.75	0.76	0.94	0.95	1.02	1.01	1.04	1.05	0.89	0.90	0.90	0.88	40.23	6.83
Q9WVC2	Isoform 2 of Ly-6/neurotoxin-like protein 1 OS=Mus musculus GN=Lynx1 - [LYNX1_MOUSE]	24.14	2	2	2	2	1.05	1.24	1.18	1.25	1.19	1.18	1.12	1.04	0.98	1.11	1.06	1.29	1.24	12.83	8.19
O70252	Heme oxygenase 2 OS=Mus musculus GN=Hmox2 PE=2 SV=1 - [HMOX2_MOUSE]	43.49	4	9	9	20	1.06	0.94	0.93	1.05	0.97	1.02	0.91	1.04	0.93	0.97	0.90	1.00	0.88	35.72	5.87
Q9JJF0	Nucleosome assembly protein 1-like 5 OS=Mus musculus GN=Nap1l5 PE=2 SV=1 - [NP1L5_MOUSE]	47.44	1	5	5	22	1.53	0.69	0.48	0.98	0.67	1.07	0.75	1.04	0.79	1.32	0.90	1.16	0.78	17.01	4.32
Q7TSK3-3	Isoform 3 of Protocadherin-8 OS=Mus musculus GN=Pcdh8 - [PCDH8_MOUSE]	5.28	3	3	3	5	1.07	1.18	1.05	1.04	0.91	1.11	0.94	1.04	0.97	1.08	1.02	1.25	1.08	101.82	5.44
E9PX29	Protein Sptbn4 OS=Mus musculus GN=Sptbn4 PE=2 SV=1 - [E9PX29_MOUSE]	22.96	7	41	45	106	0.92	0.95	1.03	0.93	1.01	1.05	1.10	1.04	1.13	0.98	1.06	1.02	1.09	288.65	6.00
Q14AR7	MCG6726, isoform CRA_f OS=Mus musculus GN=Mrgbp PE=2 SV=1 - [Q14AR7_MOUSE]	6.37	3	1	1	1	1.30	1.46	1.12	1.21	0.93	1.23	0.94	1.04	0.80	0.89	0.69	1.02	0.78	22.42	5.83
P50446	Keratin, type II cytoskeletal 6A OS=Mus musculus GN=Krt6a PE=2 SV=3 - [K2C6A_MOUSE]	8.14	7	1	5	9	1.34	1.35	1.01	1.23	0.92	1.18	0.88	1.04	0.77	0.84	0.63	1.02	0.77	59.30	7.94
Q6P3A8	2-oxoisovalerate dehydrogenase subunit beta, mitochondrial OS=Mus musculus GN=Bckdhb PE=2 SV=2 - [ODBB_MOUSE]	8.21	2	2	2	3	1.09	0.94	0.86	1.16	1.06	1.09	0.99	1.04	0.95	1.04	0.95	1.06	0.98	42.85	6.70

Q6P9Z1	SWI/SNF-related matrix-associated actin-dependent regulator of chromatin subfamily D member 3 OS=Mus musculus GN=Smarcd3 PE=1 SV=2 - [SMRD3_MOUSE]	10.14	3	2	3	7	1.04	1.27	1.04	1.10	1.06	1.15	1.01	1.04	1.01	1.24	1.08	1.26	1.15	54.95	9.35
Q6ZPZ3-2	Isoform 2 of Zinc finger CCCH domain-containing protein 4 OS=Mus musculus GN=Zc3h4 - [ZC3H4_MOUSE]	18.67	3	13	13	23	1.01	0.90	0.86	0.93	0.91	1.04	0.99	1.04	0.96	1.04	1.07	0.98	0.98	131.52	6.52
E9Q9B2	Protein N4bp2 (Fragment) OS=Mus musculus GN=N4bp2 PE=2 SV=1 - [E9Q9B2_MOUSE]	1.47	6	1	2	3	1.50	1.05	0.70	0.83	0.55	1.29	0.85	1.04	0.69	0.82	0.55	0.93	0.62	164.29	5.33
F7BP73	Solute carrier family 22 member 23 (Fragment) OS=Mus musculus GN=Slc22a23 PE=4 SV=1 - [F7BP73_MOUSE]	9.01	4	3	3	5	1.05	1.24	1.19	1.15	1.10	1.07	1.01	1.04	1.06	1.04	0.94	1.00	1.02	62.03	8.05
P09470	Angiotensin-converting enzyme OS=Mus musculus GN=Ace PE=1 SV=3 - [ACE_MOUSE]	1.75	2	2	2	4	1.00	1.09	1.09	1.14	1.13	0.98	0.97	1.04	1.03	1.18	1.18	1.15	1.15	150.82	6.55
Q91YJ2	Sorting nexin-4 OS=Mus musculus GN=Snx4 PE=2 SV=1 - [SNX4_MOUSE]	16.00	1	5	5	10	1.09	1.21	1.10	1.04	1.05	0.90	0.95	1.04	0.98	1.05	1.00	1.00	0.96	51.75	5.80
P56371	Ras-related protein Rab 4A OS=Mus musculus GN=Rab4a PE=1 SV=2 - [RAB4A_MOUSE]	26.15	10	2	5	51	1.17	1.75	1.49	1.17	1.00	0.99	0.84	1.04	0.88	1.03	0.88	1.17	1.00	24.39	6.07
D3YXK0	MCG4624, isoform CRA_c OS=Mus musculus GN=Jakmip2 PE=4 SV=2 - [D3YXK0_MOUSE]	15.73	1	10	11	18	0.97	0.91	0.90	0.91	0.99	0.97	1.12	1.04	0.97	0.84	0.89	0.99	1.07	96.26	5.97
Q9D338	39S ribosomal protein L19, mitochondrial OS=Mus musculus GN=Mrpl19 PE=2 SV=1 - [RM19_MOUSE]	17.12	1	3	3	4	0.96	1.03	0.93	1.03	0.90	1.24	1.12	1.04	0.86	1.33	1.14	1.16	0.94	33.56	9.44
Q61739-2	Isoform Alpha-6X1A of Integrin alpha-6 OS=Mus musculus GN=Itga6 - [ITA6_MOUSE]	2.98	3	3	3	4	0.95	0.97	0.81	1.01	1.06	0.93	0.92	1.04	1.00	0.91	0.96	0.93	0.90	119.53	6.96
Q8R0F6-2	Isoform 2 of Integrin-linked kinase-associated serine/threonine phosphatase 2C OS=Mus musculus GN=Ilkap - [ILKAP_MOUSE]	4.46	2	1	1	2	0.81	1.09	1.34	0.90	1.11	0.92	1.13	1.04	1.28	1.01	1.25	1.11	1.37	30.02	7.87

Q3UNX5	Acyl-coenzyme A synthetase ACSM3, mitochondrial OS=Mus musculus GN=Acsm3 PE=1 SV=2 - [ACSM3_MOUSE]	1.21	2	1	1	1	1.56	1.04	0.67	1.17	0.75	1.13	0.72	1.04	0.66	1.33	0.85	1.49	0.95	65.58	7.46
D3Z5J3	Sorbin and SH3 domain-containing protein 1 OS=Mus musculus GN=Sorbs1 PE=2 SV=1 - [D3Z5J3_MOUSE]	36.57	7	5	31	106	0.98	1.04	1.04	0.90	0.96	0.97	1.07	1.04	1.13	0.89	0.99	0.97	0.90	103.91	5.94
Q8VCS6	Mediator of RNA polymerase II transcription subunit 9 OS=Mus musculus GN=Med9 PE=2 SV=1 - [MED9_MOUSE]	10.56	1	1	1	2	1.46	1.82	1.24	1.23	0.84	1.38	0.94	1.04	0.71	1.12	0.77	0.91	0.62	15.70	7.44
P09240	Cholecystokinin OS=Mus musculus GN=Cck PE=1 SV=3 - [CCKN_MOUSE]	8.70	1	1	1	2	1.26	1.13	0.89	1.03	0.81	1.21	0.95	1.04	0.82	1.03	0.81	1.10	0.87	12.76	9.35
Q0VGB7	Serine/threonine-protein phosphatase 4 regulatory subunit 2 OS=Mus musculus GN=Ppp4r2 PE=1 SV=1 - [PP4R2_MOUSE]	9.83	1	3	3	5	0.91	0.77	0.79	0.87	1.06	1.00	1.09	1.04	1.16	0.92	0.95	0.86	0.93	46.45	4.56
Q3UFK1	Coiled-coil domain containing 28A OS=Mus musculus GN=Ccdc28a PE=2 SV=1 - [Q3UFK1_MOUSE]	46.74	4	5	5	13	1.20	1.17	1.14	1.06	0.89	1.23	0.94	1.04	1.09	1.16	0.92	1.06	0.83	20.29	8.12
Q7TSI3	Serine/threonine-protein phosphatase 6 regulatory subunit 1 OS=Mus musculus GN=Ppp6r1 PE=1 SV=1 - [PP6R1_MOUSE]	6.07	1	5	5	10	0.96	1.03	1.07	1.00	1.07	1.09	1.09	1.04	1.05	1.01	1.03	0.94	0.93	94.47	4.64
Q61097-2	Isoform 2 of Kinase suppressor of Ras 1 OS=Mus musculus GN=Ksr1 - [KSR1_MOUSE]	7.65	5	5	5	11	1.03	1.24	1.13	1.07	0.99	0.95	0.90	1.04	0.99	1.09	0.97	1.21	1.08	95.80	8.48
D3Z1V2	Regulator of G-protein-signaling 8 (Fragment) OS=Mus musculus GN=Rgs8 PE=2 SV=1 - [D3Z1V2_MOUSE]	6.52	3	1	1	2	1.22	1.45	1.19	1.26	1.04	1.11	0.91	1.04	0.85	1.20	0.99	1.34	1.10	16.01	8.92
Q64727	Vinculin OS=Mus musculus GN=Vcl PE=1 SV=4 - [VINC_MOUSE]	40.90	1	33	33	84	1.04	1.04	1.01	1.05	1.02	1.03	0.99	1.04	1.02	1.08	1.09	1.10	1.07	116.64	6.00
E9Q1U6	CBP80/20-dependent translation initiation factor OS=Mus musculus GN=Ctif PE=4 SV=1 - [E9Q1U6_MOUSE]	7.54	3	5	5	10	0.95	1.19	1.25	0.95	0.97	1.11	1.10	1.04	1.08	0.95	1.00	1.01	1.07	70.23	6.61

F7CZ64	Voltage-dependent calcium channel gamma-8 subunit OS=Mus musculus GN=Cacng8 PE=4 SV=2 - [F7CZ64_MOUSE]	20.57	2	4	4	17	1.05	0.93	0.88	1.07	1.02	1.05	1.01	1.04	0.97	1.09	1.03	1.04	0.98	43.39	9.20
Q9JL35	High mobility group nucleosome-binding domain-containing protein 5 OS=Mus musculus GN=Hmgn5 PE=1 SV=2 - [HMGN5_MOUSE]	50.99	1	12	12	27	1.10	0.62	0.62	1.00	0.81	1.21	0.98	1.04	0.94	0.99	0.91	0.87	0.75	45.32	4.37
Q9QZQ1	Afadin OS=Mus musculus GN=Mllt4 PE=1 SV=3 - [AFAD_MOUSE]	25.55	6	5	36	90	0.99	0.97	0.90	0.99	0.99	1.16	1.15	1.04	1.10	1.20	1.12	1.05	1.01	206.37	6.32
F8VPZ3	Ubiquitin carboxyl-terminal hydrolase OS=Mus musculus GN=Usp32 PE=2 SV=1 - [F8VPZ3_MOUSE]	2.00	2	2	2	3	0.95	1.12	1.17	0.95	1.00	1.14	1.19	1.04	1.09	1.01	1.06	0.99	1.04	181.62	6.51
D3Z645	Vacuolar protein sorting-associated protein 29 OS=Mus musculus GN=Vps29 PE=2 SV=1 - [D3Z645_MOUSE]	56.94	3	1	6	31	1.03	1.07	0.98	1.03	0.99	0.83	0.79	1.04	1.07	1.02	0.99	1.09	1.13	16.13	7.01
E9PVU7	cAMP-specific 3',5'-cyclic phosphodiesterase 4D OS=Mus musculus GN=Pde4d PE=2 SV=1 - [E9PVU7_MOUSE]	15.57	11	7	10	21	0.98	0.91	0.93	1.02	1.06	1.05	0.96	1.04	1.02	1.02	1.00	1.06	0.95	90.70	5.49
P70425	GTP-binding protein Rit2 OS=Mus musculus GN=Rit2 PE=1 SV=1 - [RIT2_MOUSE]	5.53	1	1	1	2	1.00	1.24	1.23	1.20	1.19	1.06	1.05	1.04	1.03	1.18	1.18	1.38	1.38	24.79	6.92
Q3TDD9	Protein phosphatase 1 regulatory subunit 21 OS=Mus musculus GN=Ppp1r21 PE=2 SV=2 - [PPR21_MOUSE]	20.26	3	11	11	23	0.93	0.97	0.94	1.06	1.15	0.96	1.09	1.04	1.06	1.07	1.09	1.08	1.11	88.28	6.90
Q03157	Amyloid-like protein 1 OS=Mus musculus GN=Aplp1 PE=1 SV=1 - [APLP1_MOUSE]	20.98	1	12	12	51	0.99	1.12	1.06	0.92	0.90	1.05	1.05	1.04	1.00	1.00	0.99	1.01	1.00	72.71	5.67
Q8R5A6	TBC1 domain family member 22A OS=Mus musculus GN=Tbc1d22a PE=2 SV=3 - [TB22A_MOUSE]	4.46	2	1	2	3	0.95	1.54	1.62	1.22	1.29	1.08	1.13	1.04	1.09	1.11	1.17	1.25	1.32	59.33	6.46
P50114	Protein S100-B OS=Mus musculus GN=S100b PE=2 SV=2 - [S100B_MOUSE]	79.35	1	5	5	350	1.00	0.67	0.67	0.86	0.87	1.06	1.06	1.04	1.05	0.97	0.97	0.90	0.88	10.72	4.55

Q8R0A5	Transcription elongation factor A protein-like 3 OS=Mus musculus GN=Tceal3 PE=1 SV=2 - [TCAL3_MOUSE]	54.50	3	2	12	197	1.46	0.43	0.33	1.05	0.85	1.29	0.92	1.04	0.66	0.95	0.64	0.82	0.64	22.45	5.44
P54775	26S protease regulatory subunit 6B OS=Mus musculus GN=Psmc4 PE=1 SV=2 - [PRS6B_MOUSE]	40.19	1	11	11	29	1.01	0.93	0.91	1.06	1.03	1.09	1.08	1.04	1.03	1.06	1.03	1.01	0.98	47.38	5.21
Q8BIL5-2	Isoform 2 of Protein Hook homolog 1 OS=Mus musculus GN=Hook1 - [HOOK1_MOUSE]	5.28	3	2	2	3	0.92	0.85	0.93	0.94	1.02	1.00	1.08	1.04	1.13	0.99	1.08	0.93	1.01	63.97	4.98
Q62442-2	Isoform 2 of Vesicle-associated membrane protein 1 OS=Mus musculus GN=Vamp1 - [VAMP1_MOUSE]	42.74	3	4	6	24	0.96	1.11	1.15	1.04	1.12	0.91	0.93	1.04	1.04	1.01	1.02	0.92	0.94	12.74	8.47
P26645	Myristoylated alanine-rich C-kinase substrate OS=Mus musculus GN=Marcks PE=1 SV=2 - [MARCS_MOUSE]	73.79	1	16	16	348	1.31	0.96	0.83	1.03	0.78	1.18	0.90	1.04	0.84	1.08	0.86	1.17	0.89	29.64	4.34
O09173	Homogentisate 1,2-dioxygenase OS=Mus musculus GN=Hgd PE=1 SV=2 - [HGD_MOUSE]	2.47	1	1	1	1	0.90	10.12	11.21	1.01	1.12	1.09	1.20	1.04	1.15	0.93	1.03	1.09	1.21	49.93	7.24
Q8VDM1	Zinc finger CCCH-type with G patch domain-containing protein OS=Mus musculus GN=Zgpat PE=2 SV=1 - [ZGPAT_MOUSE]	6.26	2	2	2	4	0.91	1.23	1.35	0.85	0.93	1.11	1.21	1.04	1.14	1.03	1.13	1.02	1.12	56.38	5.33
Q9JLB0	MAGUK p55 subfamily member 6 OS=Mus musculus GN=Mpp6 PE=1 SV=1 - [MPP6_MOUSE]	36.89	4	17	19	48	0.93	0.89	0.99	1.02	1.10	1.01	1.11	1.04	1.13	1.05	1.13	0.96	1.07	62.59	6.40
Q9CQ45	Neudesin OS=Mus musculus GN=Nenf PE=1 SV=1 - [NENF_MOUSE]	52.63	1	6	6	38	1.07	0.76	0.69	0.90	0.84	1.11	1.09	1.04	1.05	1.00	0.98	0.88	0.82	18.89	5.27
Q80V91	Probable E3 ubiquitin-protein ligase DTX3 OS=Mus musculus GN=Dtx3 PE=2 SV=2 - [DTX3_MOUSE]	13.83	3	3	3	10	1.17	1.03	0.97	1.02	0.90	0.97	0.89	1.04	0.96	0.99	0.85	0.97	0.83	37.96	8.73
P48758	Carbonyl reductase [NADPH] 1 OS=Mus musculus GN=Cbr1 PE=1 SV=3 - [CBR1_MOUSE]	54.15	1	11	12	56	1.07	1.04	1.00	1.10	1.00	1.08	1.02	1.04	0.97	1.07	1.04	1.17	1.07	30.62	8.31
Q6W8Q3	Purkinje cell protein 4-like protein 1 OS=Mus musculus GN=Pcp4l1 PE=2 SV=1 - [PC4L1_MOUSE]	60.29	1	4	4	45	0.98	0.70	0.76	0.84	0.89	1.04	1.16	1.04	1.13	1.14	1.17	1.08	1.10	7.50	5.52

P37804	Transgelin OS=Mus musculus GN=Tagln PE=1 SV=3 - [TAGL_MOUSE]	38.31	1	7	7	14	1.07	0.93	0.86	0.86	0.80	0.88	0.80	1.04	1.06	1.03	0.99	0.84	0.79	22.56	8.81
B7ZBW1	Potassium voltage-gated channel subfamily KQT member 2 OS=Mus musculus GN=Kcnq2 PE=2 SV=1 - [B7ZBW1_MOUSE]	17.61	27	9	9	18	0.99	1.06	1.05	1.08	1.02	0.99	0.94	1.04	0.99	1.10	1.12	1.09	1.06	93.88	9.28
Q9D0C1	E3 ubiquitin-protein ligase RNF115 OS=Mus musculus GN=Rnf115 PE=1 SV=1 - [RN115_MOUSE]	2.95	1	1	1	2	1.06	1.07	1.00	1.03	0.97	1.02	0.96	1.04	0.98	1.06	1.00	0.96	0.91	33.84	5.83
Q9D1I5	Methylmalonyl-CoA epimerase, mitochondrial OS=Mus musculus GN=Mcee PE=2 SV=1 - [MCEE_MOUSE]	54.49	1	5	5	27	0.90	0.77	0.86	0.83	0.93	1.03	1.14	1.04	1.16	0.88	1.04	0.79	0.89	19.01	9.09
Q9Z1W9	STE20/SPS1-related proline-alanine-rich protein kinase OS=Mus musculus GN=Stk39 PE=1 SV=1 - [STK39_MOUSE]	21.22	2	8	10	32	0.96	0.97	1.02	0.95	1.10	0.99	1.11	1.04	1.00	0.99	1.17	0.99	1.12	60.28	6.29
Q99JW1-2	Isoform 2 of Alpha/beta hydrolase domain-containing protein 17A OS=Mus musculus GN=Abhd17a - [AB17A_MOUSE]	9.76	2	1	1	4	1.04	1.26	1.21	1.10	1.06	0.99	0.95	1.04	1.00	1.03	1.00	0.98	0.95	26.93	6.95
P83093	Stromal interaction molecule 2 OS=Mus musculus GN=Stim2 PE=1 SV=2 - [STIM2_MOUSE]	16.62	3	10	10	16	0.92	1.08	1.20	1.01	1.07	1.04	1.00	1.04	1.11	0.96	0.96	0.89	0.94	83.87	6.79
Q9D1P0	39S ribosomal protein L13, mitochondrial OS=Mus musculus GN=Mrpl13 PE=2 SV=1 - [RM13_MOUSE]	27.53	2	4	4	8	0.93	1.03	1.09	1.04	1.14	1.08	1.18	1.04	1.14	1.07	1.20	0.96	1.15	20.66	9.35
A2AFR3-2	Isoform 2 of FERM and PDZ domain-containing protein 4 OS=Mus musculus GN=Frmpd4 - [FRPD4_MOUSE]	3.36	3	3	3	17	1.23	1.31	1.04	1.08	0.86	1.09	0.94	1.04	0.87	0.97	0.82	0.99	0.81	140.41	5.20
G3UYN6	PCTP-like protein (Fragment) OS=Mus musculus GN=Stard10 PE=4 SV=1 - [G3UYN6_MOUSE]	13.79	6	1	1	2	0.76	0.72	0.94	0.86	1.13	0.74	0.97	1.04	1.36	0.73	0.96	0.82	1.08	9.62	6.68
D3Z285	NFU1 iron-sulfur cluster scaffold homolog, mitochondrial (Fragment) OS=Mus musculus GN=Nfu1 PE=2 SV=1 - [D3Z285_MOUSE]	25.63	1	1	4	29	0.96	0.81	0.87	0.99	1.01	1.04	1.11	1.04	1.13	1.01	1.04	0.95	0.98	22.01	4.96

Q7TNY3	Calcipressin 1 large isoform OS=Mus musculus GN=Rcan1 PE=2 SV=1 - [Q7TNY3_MOUSE]	11.95	4	2	2	7	0.94	0.95	0.96	0.91	0.98	0.86	0.87	1.04	1.01	0.72	0.78	0.86	0.85	28.12	5.41
Q9Z0G0	PDZ domain-containing protein GIPC1 OS=Mus musculus GN=Gipc1 PE=1 SV=1 - [GIPC1_MOUSE]	30.03	2	6	6	20	0.95	0.97	1.00	0.99	1.05	1.09	1.19	1.04	1.17	1.06	1.13	0.98	1.05	36.11	5.91
Q8VE42	Ankyrin repeat domain-containing protein 49 OS=Mus musculus GN=Ankrd49 PE=1 SV=1 - [ANR49_MOUSE]	21.43	1	4	4	9	1.05	0.88	0.83	1.03	0.96	1.31	1.20	1.04	1.19	1.24	1.02	1.03	0.88	27.09	5.16
P62309	Small nuclear ribonucleoprotein G OS=Mus musculus GN=Snrpg PE=1 SV=1 - [RUXG_MOUSE]	25.00	1	2	2	7	0.93	0.98	1.08	0.92	1.01	0.97	1.04	1.04	1.08	0.83	0.89	0.93	0.96	8.49	8.88
Q6PAV2-	Isoform 2 of Probable E3 ubiquitin-protein ligase HERC4 OS=Mus musculus GN=Herc4 - [HERC4_MOUSE]	3.91	2	3	3	5	1.03	1.29	1.24	1.07	1.04	0.91	0.88	1.04	0.94	0.99	0.96	1.24	1.20	117.51	6.37
Q9Z0V1-2	Isoform 2 of Potassium voltage-gated channel subfamily D member 3 OS=Mus musculus GN=Kcnd3 - [KCND3_MOUSE]	8.65	3	3	3	7	1.18	0.99	0.76	1.08	0.90	1.09	0.82	1.04	0.83	1.13	0.91	0.94	0.74	71.36	8.25
Q8VCG3	WD repeat-containing protein 74 OS=Mus musculus GN=Wdr74 PE=2 SV=1 - [WDR74_MOUSE]	3.65	1	1	1	2	0.91	1.05	1.15	1.04	1.14	1.17	1.27	1.04	1.14	1.11	1.23	1.16	1.28	42.61	7.78
Q9D0D4	Probable dimethyladenosine transferase OS=Mus musculus GN=Dimt1 PE=2 SV=1 - [DIM1_MOUSE]	2.88	1	1	1	2	1.17	1.80	1.53	1.13	0.97	0.98	0.83	1.04	0.89	1.03	0.88	1.00	0.85	35.25	9.99
P05977	Myosin light chain 1/3, skeletal muscle isoform OS=Mus musculus GN=Myl1 PE=1 SV=2 - [MYL1_MOUSE]	75.00	4	9	12	86	1.01	1.42	1.42	0.81	0.96	15.39	19.49	1.04	1.05	1.02	0.94	1.04	1.05	20.58	5.03
Q8VE95	UPF0598 protein C8orf82 homolog OS=Mus musculus PE=2 SV=1 - [CH082_MOUSE]	4.13	1	1	1	1	1.03	1.08	1.04	1.21	1.17	1.01	0.97	1.04	1.00	1.24	1.20	1.22	1.18	24.31	8.70
D3Z0V7	TSC22 domain family protein 1 OS=Mus musculus GN=Tsc22d1 PE=2 SV=1 - [D3Z0V7_MOUSE]	14.17	6	8	11	61	0.85	0.77	0.94	0.86	1.01	1.14	1.24	1.04	1.25	0.98	1.14	0.93	1.19	102.02	5.77

Q9Z1D1	Eukaryotic translation initiation factor 3 subunit G OS=Mus musculus GN=Eif3g PE=1 SV=2 - [EIF3G_MOUSE]	37.19	1	9	9	57	1.03	0.94	0.92	0.88	0.84	1.04	0.95	1.04	1.01	0.90	0.89	0.92	0.86	35.62	5.90
Q80Z10-2	Isoform 2 of Astrotactin-2 OS=Mus musculus GN=Astn2 - [ASTN2_MOUSE]	7.72	3	7	7	14	0.91	1.24	1.46	1.09	1.20	0.93	1.00	1.04	1.08	0.93	1.02	0.85	0.95	148.80	6.02
E9Q0W8	Small nuclear ribonucleoprotein E OS=Mus musculus GN=Snrpe PE=2 SV=1 - [E9Q0W8_MOUSE]	21.15	3	1	1	1	1.03	1.13	1.09	0.87	0.84	0.94	0.90	1.04	1.00	1.01	0.98	1.03	1.00	6.16	9.85
Q6A0D4	Raftlin OS=Mus musculus GN=Rftn1 PE=1 SV=4 - [RFTN1_MOUSE]	9.03	1	2	2	8	1.21	1.33	1.10	1.17	0.86	1.05	0.87	1.04	0.78	1.21	0.94	1.19	1.07	61.50	7.68
Q8K0C8	Cytochrome c oxidase assembly protein COX19 OS=Mus musculus GN=Cox19 PE=3 SV=1 - [COX19_MOUSE]	34.78	1	3	3	5	1.02	0.84	0.87	0.94	0.99	1.15	1.12	1.04	1.08	1.00	1.01	0.93	0.90	10.81	8.07
Q8BHE5	Bone morphogenetic protein 3 OS=Mus musculus GN=Bmp3 PE=1 SV=1 - [BMP3_MOUSE]	2.78	1	1	1	2	1.14	0.98	0.86	1.02	0.90	1.09	0.95	1.04	0.91	0.95	0.84	0.94	0.83	52.74	9.38
O35098	Dihydropyrimidinase-related protein 4 OS=Mus musculus GN=Dpysl4 PE=1 SV=1 - [DPYL4_MOUSE]	46.50	4	16	18	119	1.01	1.05	1.07	1.04	1.02	1.02	1.03	1.04	0.99	1.01	0.99	0.99	1.05	61.92	6.98
Q4KMM3	Oxidation resistance protein 1 OS=Mus musculus GN=Oxr1 PE=1 SV=3 - [OXR1_MOUSE]	68.01	6	49	49	552	0.95	0.87	0.93	0.89	0.93	0.96	1.01	1.04	1.06	0.87	0.89	0.87	0.90	95.85	5.33
Q6PDG8-	Isoform 2 of Vacuolar fusion protein MON1 homolog A OS=Mus musculus GN=Mon1a - [MON1A_MOUSE]	5.86	2	1	1	4	0.96	1.00	1.03	1.12	1.17	1.23	1.27	1.04	1.08	0.98	1.02	1.06	1.10	51.13	6.06
Q8C178	Acyl-coenzyme A oxidase OS=Mus musculus GN=Acox3 PE=2 SV=1 - [Q8C178_MOUSE]	1.72	3	1	1	2	1.06	1.59	1.51	0.97	0.92	1.00	0.94	1.04	0.98	0.96	0.91	1.07	1.01	77.82	7.11
O70309	Integrin beta-5 OS=Mus musculus GN=Itgb5 PE=1 SV=2 - [ITB5_MOUSE]	4.14	5	2	3	5	1.05	1.39	1.32	1.09	1.04	0.94	0.89	1.04	0.99	1.09	1.04	1.19	1.13	87.85	6.16
Q7TQE6	Macoilin OS=Mus musculus GN=Tmem57 PE=2 SV=1 - [MACOI_MOUSE]	1.20	1	1	1	1	1.20	2.09	1.74	1.13	0.94	1.47	1.22	1.04	0.87	1.07	0.90	1.05	0.88	76.04	9.07
E9QM77	Ataxin-2 OS=Mus musculus GN=Atxn2 PE=2 SV=1 - [E9QM77_MOUSE]	16.95	7	16	16	45	0.89	0.75	0.82	0.87	0.97	0.98	1.08	1.04	1.17	0.89	1.06	0.81	0.92	136.38	9.54

A2AFJ1	Histone-binding protein RBBP7 OS=Mus musculus GN=Rbbp7 PE=2 SV=1 - [A2AFJ1_MOUSE]	27.40	5	5	8	67	0.80	0.80	0.98	0.80	0.99	0.94	1.21	1.04	1.30	0.85	1.04	0.82	1.02	46.88	5.07
Q8C4C4	Repulsive guidance molecule A OS=Mus musculus GN=Rgma PE=2 SV=1 - [Q8C4C4_MOUSE]	13.37	3	3	3	5	1.20	1.39	1.19	1.19	0.96	1.15	0.95	1.04	0.97	1.02	0.97	1.02	1.00	37.84	6.60
P70429-2	Isoform 1 of Ena/VASP-like protein OS=Mus musculus GN=Evl - [EVL_MOUSE]	35.37	4	10	10	27	0.98	0.89	0.87	0.99	0.92	0.98	0.98	1.04	0.97	0.99	0.94	0.98	0.95	42.10	8.35
P58404-2	Isoform 2 of Striatin-4 OS=Mus musculus GN=Strn4 - [STRN4_MOUSE]	33.86	3	14	15	41	0.97	1.03	1.04	0.99	1.00	1.01	1.09	1.04	1.09	1.01	1.01	0.97	0.94	80.93	5.38
P52480	Pyruvate kinase PKM OS=Mus musculus GN=Pkm PE=1 SV=4 - [KPYM_MOUSE]	80.98	4	2	44	824	1.12	1.10	0.87	1.18	1.01	1.06	0.96	1.04	0.94	0.92	0.95	1.06	0.92	57.81	7.47
D3Z601	Calsyntenin-3 OS=Mus musculus GN=Clstn3 PE=2 SV=1 - [D3Z601_MOUSE]	10.99	2	6	6	10	0.98	1.03	1.00	0.97	0.95	0.96	0.92	1.04	1.04	1.01	1.01	0.94	0.93	101.75	5.27
P97792	Coxsackievirus and adenovirus receptor homolog OS=Mus musculus GN=Cxadr PE=1 SV=1 - [CXAR_MOUSE]	19.18	3	5	5	9	1.12	0.95	0.87	1.08	0.99	1.09	1.00	1.04	0.94	0.99	0.92	0.93	0.85	39.92	6.96
Q6NV83-:	Isoform 3 of U2 snRNP-associated SURP motif-containing protein OS=Mus musculus GN=U2surp - [SR140_MOUSE]	4.16	3	3	3	4	0.98	0.81	0.76	1.05	1.07	1.02	1.02	1.04	1.09	1.02	1.13	1.25	1.17	113.19	8.35
A2AP93	Mitogen-activated protein kinase kinase kinase 7 OS=Mus musculus GN=Map3k7 PE=2 SV=1 - [A2AP93_MOUSE]	8.15	4	3	3	8	0.98	0.88	0.90	0.97	1.03	0.99	1.00	1.04	1.07	1.01	1.09	0.83	0.91	53.72	6.68
P32067	Lupus La protein homolog OS=Mus musculus GN=Ssb PE=2 SV=1 - [LA_MOUSE]	26.75	4	11	11	20	1.06	0.84	0.88	1.03	1.00	1.04	1.04	1.04	1.03	0.95	0.94	0.93	0.93	47.73	9.77
Q5SVQ0-	Isoform 4 of Histone acetyltransferase KAT7 OS=Mus musculus GN=Kat7 - [KAT7_MOUSE]	5.16	4	2	2	6	0.83	0.88	1.01	0.94	1.06	0.98	1.10	1.04	1.17	0.95	1.09	0.97	1.10	66.87	8.72
Q9JII6	Alcohol dehydrogenase [NADP(+)] OS=Mus musculus GN=Akr1a1 PE=1 SV=3 - [AK1A1_MOUSE]	40.92	2	11	11	36	0.96	1.07	1.11	1.01	1.08	1.00	1.06	1.04	1.09	1.04	1.06	1.07	1.12	36.56	7.39

Q61140	Breast cancer anti-estrogen resistance protein 1 OS=Mus musculus GN=Bcar1 PE=1 SV=2 - [BCAR1_MOUSE]	11.90	2	6	6	13	1.07	1.11	1.03	0.92	0.89	1.04	1.05	1.04	0.96	0.91	1.12	1.05	1.09	94.23	5.68
E0CX79	Zinc finger protein-like 1 (Fragment) OS=Mus musculus GN=Zfpl1 PE=2 SV=1 - [E0CX79_MOUSE]	13.18	5	2	2	3	1.01	1.34	1.33	0.98	0.96	0.95	0.97	1.04	1.00	1.01	1.05	1.22	1.24	14.50	8.37
P70445	Eukaryotic translation initiation factor 4E-binding protein 2 OS=Mus musculus GN=Eif4ebp2 PE=2 SV=1 - [4EBP2_MOUSE]	73.33	1	5	5	22	1.08	1.06	1.02	0.94	0.86	1.06	1.06	1.04	1.01	0.99	0.99	0.98	0.98	12.89	6.52
Q3TW96	UDP-N-acetylhexosamine pyrophosphorylase-like protein 1 OS=Mus musculus GN=Uap1l1 PE=2 SV=1 - [UAP1L_MOUSE]	13.02	2	6	6	9	1.03	1.13	1.11	1.07	1.03	1.02	0.95	1.04	1.04	1.01	1.01	1.10	0.97	56.58	5.43
O70433	Four and a half LIM domains protein 2 OS=Mus musculus GN=Fhl2 PE=1 SV=1 - [FHL2_MOUSE]	47.31	1	12	12	38	0.92	0.82	0.96	0.91	0.95	1.12	1.14	1.04	1.09	0.91	0.98	0.85	0.96	32.05	7.30
O55241	Orexin OS=Mus musculus GN=Hcrt PE=2 SV=1 - [OREX_MOUSE]	10.00	1	1	1	5	1.08	1.03	1.01	1.04	0.97	1.20	1.09	1.04	0.94	1.06	0.95	1.15	1.14	13.49	9.70
Q2WF71	Leucine-rich repeat and fibronectin type III domain-containing protein 1 OS=Mus musculus GN=Lrfn1 PE=1 SV=1 - [LRFN1_MOUSE]	7.96	3	4	4	7	1.05	1.17	1.05	1.22	1.09	1.00	0.88	1.04	0.93	1.13	1.03	0.97	0.88	81.89	7.59
Q8BZW2	Ankyrin repeat domain-containing protein SOWAHB OS=Mus musculus GN=Sowahb PE=2 SV=1 - [SWAHB_MOUSE]	2.37	1	1	1	2	0.95	0.78	0.81	0.99	1.04	1.00	1.05	1.04	1.10	0.96	1.01	0.88	0.92	83.49	8.27
Q9JKL5	Calcineurin B homologous protein 3 OS=Mus musculus GN=Tesc PE=1 SV=2 - [CHP3_MOUSE]	29.44	1	5	5	13	0.98	0.98	1.01	0.98	0.94	1.01	0.99	1.04	1.04	1.04	1.04	1.11	1.13	24.58	4.98
P19157	Glutathione S-transferase P 1 OS=Mus musculus GN=Gstp1 PE=1 SV=2 - [GSTP1_MOUSE]	83.81	3	10	10	137	1.03	1.00	0.96	1.00	1.01	1.03	1.02	1.04	1.03	1.08	1.07	1.14	1.15	23.59	7.87

Q9DAS9	Guanine nucleotide-binding protein G(I)/G(S)/G(O) subunit gamma-12 OS=Mus musculus GN=Gng12 PE=1 SV=3 - [GBG12_MOUSE]	58.33	1	4	4	36	1.05	1.13	1.07	1.00	1.02	1.04	1.01	1.04	0.99	1.04	0.99	1.08	1.03	7.99	8.97
Q9WV92	Band 4.1-like protein 3 OS=Mus musculus GN=Epb41l3 PE=1 SV=1 - [E41L3_MOUSE]	56.94	3	3	42	323	1.02	0.97	0.92	0.89	0.92	1.07	1.06	1.04	1.07	1.01	1.00	0.91	0.94	103.27	5.31
Q9R1Q8	Transgelin-3 OS=Mus musculus GN=Tagln3 PE=1 SV=1 - [TAGL3_MOUSE]	67.34	1	12	13	97	0.84	0.77	0.96	0.79	0.98	0.92	1.13	1.04	1.21	0.84	1.00	0.82	1.03	22.46	7.33
P68033	Actin, alpha cardiac muscle 1 OS=Mus musculus GN=Actc1 PE=1 SV=1 - [ACTC_MOUSE]	76.39	6	2	27	1024	0.83	0.93	1.01	1.05	1.21	0.70	0.82	1.04	1.16	1.24	1.49	0.85	1.03	41.99	5.39
F6YFN0	ERI1 exoribonuclease 3 (Fragment) OS=Mus musculus GN=Eri3 PE=2 SV=1 - [F6YFN0_MOUSE]	13.24	4	1	1	2	1.37	1.08	0.79	1.11	0.81	1.15	0.83	1.04	0.76	1.02	0.74	1.13	0.83	7.83	5.29
B7ZP47	Wapal protein OS=Mus musculus GN=Wapal PE=2 SV=1 - [B7ZP47_MOUSE]	1.09	2	1	1	3	0.94	0.77	0.81	0.95	1.00	1.05	1.11	1.04	1.11	1.11	1.18	1.00	1.07	133.27	5.47
O88597	Beclin-1 OS=Mus musculus GN=Becn1 PE=1 SV=3 - [BECN1_MOUSE]	13.39	4	5	5	18	1.06	1.25	1.05	1.08	1.03	1.19	1.09	1.05	1.01	1.16	1.07	1.04	1.00	51.56	4.93
Q08509	Epidermal growth factor receptor kinase substrate 8 OS=Mus musculus GN=Eps8 PE=1 SV=2 - [EPS8_MOUSE]	18.39	3	11	11	27	1.01	1.04	0.93	1.00	0.94	1.00	0.96	1.05	0.97	1.05	0.97	1.12	1.03	91.68	7.65
A2AES3	OTU domain-containing protein 5 OS=Mus musculus GN=Otud5 PE=2 SV=1 - [A2AES3_MOUSE]	10.06	5	3	3	6	1.02	1.00	0.92	0.86	0.90	1.01	0.93	1.05	0.97	1.11	1.09	0.95	1.00	55.89	5.95
Q80W93	Hydrocephalus-inducing protein OS=Mus musculus GN=Hydin PE=2 SV=2 - [HYDIN_MOUSE]	0.35	1	1	2	4	1.08	1.00	0.93	1.07	0.99	1.02	0.94	1.05	0.97	1.09	1.01	1.09	1.02	581.15	6.38
Q9Z2R6	Protein unc-119 homolog A OS=Mus musculus GN=Unc119 PE=1 SV=1 - [U119A_MOUSE]	5.42	2	1	1	1	1.04	1.09	1.04	0.99	0.96	1.00	0.96	1.05	1.00	0.93	0.89	1.00	0.97	26.99	6.15
E0CY91	Ubiquitin-like protein 5 OS=Mus musculus GN=Ubl5 PE=2 SV=1 - [E0CY91_MOUSE]	42.55	6	2	2	4	1.06	1.07	1.15	0.95	0.89	1.38	1.20	1.05	0.98	1.23	1.33	0.97	1.05	5.40	9.80

Q8C2Q3	RNA-binding protein 14 OS=Mus musculus GN=Rbm14 PE=1 SV=1 - [RBM14_MOUSE]	22.57	5	14	15	75	0.97	0.95	0.96	0.97	0.98	1.01	1.08	1.05	1.10	1.02	1.05	1.04	1.04	69.41	9.67
Q60841-3	Isoform 3 of Reelin OS=Mus musculus GN=Reln - [RELN_MOUSE]	0.67	3	2	2	3	1.14	0.91	0.79	0.89	0.78	0.94	0.82	1.05	0.91	1.16	1.02	1.06	0.93	382.95	5.55
Q9EQX4	Allograft inflammatory factor 1-like OS=Mus musculus GN=Aif1l PE=2 SV=1 - [AIF1L_MOUSE]	11.33	1	2	2	2	0.83	0.98	0.97	0.80	0.96	0.92	1.09	1.05	1.25	1.10	0.99	0.94	1.13	17.01	7.18
Q8BZ81-2	Isoform 2 of Leucine-rich repeat transmembrane neuronal protein 3 OS=Mus musculus GN=Lrrtm3 - [LRRT3_MOUSE]	2.33	2	1	1	2	1.16	1.02	0.88	1.30	1.12	1.04	0.89	1.05	0.90	1.07	0.93	1.11	0.96	58.48	9.61
P70460	Vasodilator-stimulated phosphoprotein OS=Mus musculus GN=Vasp PE=1 SV=4 - [VASP_MOUSE]	15.47	1	4	4	8	0.86	0.76	0.87	0.84	0.97	0.99	1.17	1.05	1.28	1.11	1.30	0.99	1.09	39.64	8.53
Q91YN5-2	Isoform AGX1 of UDP-N-acetylhexosamine pyrophosphorylase OS=Mus musculus GN=Uap1 - [UAP1_MOUSE]	7.13	4	3	3	4	0.97	1.23	1.26	0.89	0.94	0.87	0.89	1.05	1.07	1.01	0.94	1.07	1.07	56.86	6.54
Q9WUM5	Succinyl-CoA ligase [ADP/GDP-forming] subunit alpha, mitochondrial OS=Mus musculus GN=Suclg1 PE=1 SV=4 - [SUCA_MOUSE]	35.55	1	10	10	68	0.92	0.78	0.89	0.92	0.99	1.02	1.15	1.05	1.14	1.00	1.18	1.03	1.08	36.13	9.39
G5E8R8	MCG129950, isoform CRA_a OS=Mus musculus GN=Ubxn7 PE=4 SV=1 - [G5E8R8_MOUSE]	19.43	1	2	7	10	0.92	0.78	0.84	0.93	1.00	1.03	1.11	1.05	1.13	0.87	0.95	0.95	1.03	54.78	5.16
Q9DBZ5	Eukaryotic translation initiation factor 3 subunit K OS=Mus musculus GN=Eif3k PE=1 SV=1 - [EIF3K_MOUSE]	12.84	2	2	2	3	1.11	1.14	1.03	1.05	0.94	1.04	0.94	1.05	0.94	1.07	0.97	0.75	0.68	25.07	4.93
Q5SWZ5	Myosin phosphatase Rho-interacting protein OS=Mus musculus GN=Mprip PE=2 SV=1 - [Q5SWZ5_MOUSE]	17.94	2	6	29	63	0.96	1.05	1.05	0.97	1.02	1.12	1.16	1.05	1.07	0.93	0.98	1.01	1.05	257.13	5.59
P08003	Protein disulfide-isomerase A4 OS=Mus musculus GN=Pdia4 PE=1 SV=3 - [PDIA4_MOUSE]	49.37	1	26	26	101	1.03	0.82	0.82	0.93	0.91	1.03	1.01	1.05	1.02	1.01	0.95	0.89	0.89	71.94	5.31

Q8BYH8-	Isoform 2 of Chromodomain-helicase-DNA-binding protein 9 OS=Mus musculus GN=Chd9 - [CHD9_MOUSE]	0.45	16	1	2	2	0.99	1.03	1.03	0.87	0.87	1.14	1.14	1.05	1.05	1.08	1.09	1.02	1.03	321.69	7.39
G3X9V2	Catenin (Cadherin associated protein), delta 1, isoform CRA_a OS=Mus musculus GN=Ctnnd1 PE=4 SV=1 - [G3X9V2_MOUSE]	9.98	19	7	7	16	0.96	1.16	1.20	1.02	1.12	0.97	1.02	1.05	1.01	1.01	1.04	1.00	1.01	104.04	6.95
O35648	Centrin-3 OS=Mus musculus GN=Cetn3 PE=2 SV=1 - [CETN3_MOUSE]	7.19	1	1	1	6	1.14	1.20	1.05	0.97	0.84	1.12	0.82	1.05	0.92	1.11	0.80	0.95	0.81	19.51	4.78
Q4ACU6	SH3 and multiple ankyrin repeat domains protein 3 OS=Mus musculus GN=Shank3 PE=1 SV=2 - [SHAN3_MOUSE]	22.05	3	25	25	94	0.96	1.00	1.08	1.05	1.09	0.99	1.05	1.05	1.08	1.02	1.12	1.04	1.12	192.10	8.90
Q99M87	DnaJ homolog subfamily A member 3, mitochondrial OS=Mus musculus GN=Dnaja3 PE=1 SV=1 - [DNJA3_MOUSE]	26.25	3	10	10	18	0.95	0.87	0.99	0.96	1.02	0.93	1.04	1.05	1.05	0.90	0.91	0.88	0.93	52.41	9.22
Q6P8M1-	Isoform 2 of Putative deoxyribonuclease TATDN1 OS=Mus musculus GN=Tatdn1 - [TATD1_MOUSE]	7.48	2	2	2	2	1.02	1.27	1.24	1.08	1.05	1.10	1.07	1.05	1.02	1.00	0.98	1.01	0.99	33.23	6.18
Q3U422	NADH dehydrogenase [ubiquinone] flavoprotein 3, mitochondrial OS=Mus musculus GN=Ndufv3 PE=2 SV=1 - [Q3U422_MOUSE]	53.63	1	12	14	103	1.05	0.78	0.76	0.92	0.87	1.07	1.01	1.05	1.03	0.99	1.00	0.92	0.95	50.47	8.97
F6Q5Z1	Integrin-linked protein kinase (Fragment) OS=Mus musculus GN=Ilk PE=4 SV=1 - [F6Q5Z1_MOUSE]	6.42	2	1	1	2	0.94	1.03	1.10	1.05	1.12	0.92	0.97	1.05	1.11	1.09	1.16	0.91	0.97	24.99	9.41
Q9QZB1	Regulator of G-protein signaling 20 OS=Mus musculus GN=Rgs20 PE=1 SV=1 - [RGS20_MOUSE]	29.71	3	5	5	12	0.90	0.82	0.88	1.03	1.18	1.16	1.28	1.05	1.18	1.26	1.22	1.13	1.10	26.97	5.16
Q9DC71	28S ribosomal protein S15, mitochondrial OS=Mus musculus GN=Mrps15 PE=2 SV=2 - [RT15_MOUSE]	10.47	1	3	3	4	0.99	0.72	0.72	0.94	1.03	0.97	0.98	1.05	1.15	0.98	1.11	0.85	0.87	29.45	10.13
Q9DCV4	Regulator of microtubule dynamics protein 1 OS=Mus musculus GN=Rmdn1 PE=2 SV=2 - [RMD1_MOUSE]	8.52	2	2	2	3	0.90	1.00	1.11	1.06	1.18	0.76	0.84	1.05	1.16	0.87	0.97	0.97	1.08	34.98	8.70

Q8BH64	EH domain-containing protein 2 OS=Mus musculus GN=Ehd2 PE=1 SV=1 - [EHD2_MOUSE]	5.89	1	2	3	4	1.00	0.99	0.99	0.99	0.99	0.94	0.94	1.05	1.05	1.07	1.07	1.09	1.09	61.14	6.51
Q8K0E8	Fibrinogen beta chain OS=Mus musculus GN=Fgb PE=2 SV=1 - [FIBB_MOUSE]	6.03	2	2	3	6	0.75	1.09	1.45	0.62	0.82	0.73	0.97	1.05	1.39	1.32	1.76	0.84	1.11	54.72	7.08
Q04750	DNA topoisomerase 1 OS=Mus musculus GN=Top1 PE=1 SV=2 - [TOP1_MOUSE]	12.13	1	9	9	15	0.97	0.94	0.90	1.10	1.09	0.99	1.09	1.05	1.13	1.04	1.11	1.00	1.04	90.82	9.33
Q91VR8	Protein BRICK1 OS=Mus musculus GN=Brk1 PE=2 SV=1 - [BRK1_MOUSE]	65.33	1	7	7	20	0.96	0.96	0.96	0.94	0.89	1.03	1.03	1.05	1.08	0.90	0.96	0.94	0.96	8.76	5.45
Q8BYK6	YTH domain family protein 3 OS=Mus musculus GN=Ythdf3 PE=1 SV=2 - [YTHD3_MOUSE]	11.28	3	5	5	11	0.95	0.77	0.85	0.97	1.01	1.07	1.12	1.05	1.11	1.02	1.07	0.98	1.02	63.92	9.04
Q3TAS6-2	Isoform 2 of ER membrane protein complex subunit 10 OS=Mus musculus GN=Emc10 - [EMC10_MOUSE]	10.63	3	2	2	6	0.99	1.20	1.22	0.98	1.03	1.19	1.11	1.05	1.10	0.89	1.06	0.90	1.07	26.67	6.13
O54949	Serine/threonine-protein kinase NLK OS=Mus musculus GN=Nlk PE=1 SV=2 - [NLK_MOUSE]	4.74	17	2	3	4	0.99	1.28	1.29	1.08	1.09	1.22	1.22	1.05	1.05	0.93	0.93	1.13	1.14	58.28	8.13
Q6RHR9-	Isoform 2 of Membrane associated guanylate kinase, WW and PDZ domain-containing protein 1 OS=Mus musculus GN=Magi1 - [MAGI1_MOUSE]	24.57	1	1	22	57	1.12	1.65	1.47	0.95	0.85	1.10	0.98	1.05	0.93	1.12	1.01	1.08	0.97	127.57	6.16
Q80TR4	Slit homolog 1 protein OS=Mus musculus GN=Slit1 PE=1 SV=2 - [SLIT1_MOUSE]	2.02	4	2	2	4	1.13	1.13	1.00	1.06	0.94	1.10	0.98	1.05	0.93	1.10	0.98	1.16	1.03	167.31	6.80
P97445	Voltage-dependent P/Q-type calcium channel subunit alpha-1A OS=Mus musculus GN=Cacna1a PE=1 SV=2 - [CAC1A_MOUSE]	12.88	2	19	20	48	0.94	1.14	1.16	1.11	1.17	0.96	0.99	1.05	1.07	1.09	1.08	1.00	1.00	267.48	8.85
Q9CQ54	NADH dehydrogenase [ubiquinone] 1 subunit C2 OS=Mus musculus GN=Ndufc2 PE=1 SV=1 - [NDUC2_MOUSE]	28.33	1	5	5	18	1.10	1.11	0.99	1.16	1.26	1.03	1.04	1.05	0.98	0.99	1.00	1.01	1.08	14.15	9.20
E9PUA2	Terminal uridylyltransferase 7 OS=Mus musculus GN=Zcchc6 PE=4 SV=1 - [E9PUA2_MOUSE]	1.36	2	2	2	7	0.98	1.17	1.15	1.14	1.15	1.13	1.11	1.05	1.04	1.12	1.11	1.21	1.20	167.05	6.52

Q925T6-3	Isoform 3 of Glutamate receptor-interacting protein 1 OS=Mus musculus GN=Grip1 - [GRIP1_MOUSE]	5.13	13	4	4	7	0.98	0.91	0.85	1.12	1.11	0.96	0.98	1.05	1.06	1.04	0.98	0.92	0.95	111.31	6.00
Q80UU9	Membrane-associated progesterone receptor component 2 OS=Mus musculus GN=Pgrmc2 PE=1 SV=2 - [PGRC2_MOUSE]	64.52	1	10	12	30	1.08	1.15	0.95	1.11	0.99	1.08	0.98	1.05	0.98	1.08	1.00	1.01	0.97	23.32	5.15
Q8BGT6	MICAL-like protein 1 OS=Mus musculus GN=Micall1 PE=1 SV=3 - [MILK1_MOUSE]	17.36	3	13	13	34	0.92	0.80	0.81	0.83	0.93	1.07	1.12	1.05	1.10	0.95	1.09	0.94	1.01	94.02	6.68
Q3UCV8	Ubiquitin thioesterase otulin OS=Mus musculus GN=Fam105b PE=1 SV=1 - [OTUL_MOUSE]	1.99	1	1	1	1	0.98	1.06	1.08	1.01	1.03	1.05	1.07	1.05	1.07	1.09	1.12	1.04	1.07	40.29	5.68
D3YYY8	Protein Arhgef26 OS=Mus musculus GN=Arhgef26 PE=4 SV=1 - [D3YYY8_MOUSE]	0.81	1	1	1	1	1.22	1.31	1.08	0.93	0.76	0.93	0.76	1.05	0.86	0.98	0.81	0.98	0.81	97.33	8.72
E0CXZ1	FSD1-like protein (Fragment) OS=Mus musculus GN=Fsd1l PE=2 SV=2 - [E0CXZ1_MOUSE]	17.44	6	5	5	15	0.92	1.12	1.14	0.98	1.04	1.02	1.11	1.05	1.11	1.03	1.11	1.13	1.24	55.30	6.25
Q78J03	Methionine-R-sulfoxide reductase B2, mitochondrial OS=Mus musculus GN=Msrb2 PE=1 SV=1 - [MSRB2_MOUSE]	48.00	1	8	8	35	0.97	0.90	0.92	0.94	0.98	1.11	1.18	1.05	1.09	0.95	1.06	0.91	0.98	19.14	9.06
Q8C2K1	Differentially expressed in FDCP 6 OS=Mus musculus GN=Def6 PE=1 SV=1 - [DEFI6_MOUSE]	6.67	2	3	4	9	0.94	0.93	0.98	0.91	0.96	0.98	1.05	1.05	1.08	0.89	1.05	0.86	0.83	73.41	6.27
Q811P8	Rho GTPase-activating protein 32 OS=Mus musculus GN=Arhgap32 PE=1 SV=2 - [RHG32_MOUSE]	27.33	5	33	35	127	0.97	1.01	1.02	1.00	1.03	1.04	1.04	1.05	1.08	1.00	1.03	0.98	0.98	229.58	6.86
A2AQ19	RNA polymerase-associated protein RTF1 homolog OS=Mus musculus GN=Rtf1 PE=2 SV=1 - [RTF1_MOUSE]	15.38	1	11	11	17	0.98	0.90	0.86	0.94	0.95	1.08	1.09	1.05	1.09	1.02	1.09	0.94	0.93	80.75	8.16
D3Z6S7	Immunoglobulin superfamily member 11 OS=Mus musculus GN=Igsf11 PE=4 SV=1 - [D3Z6S7_MOUSE]	3.52	2	1	1	2	1.14	1.19	1.04	1.31	1.14	1.19	1.03	1.05	0.91	1.20	1.05	1.31	1.15	39.88	7.17

Q62141-2	Isoform 2 of Paired amphipathic helix protein Sin3b OS=Mus musculus GN=Sin3b - [SIN3B_MOUSE]	3.75	4	1	1	2	1.09	1.39	1.27	0.88	0.81	1.10	1.00	1.05	0.96	1.08	0.99	1.16	1.06	32.83	7.09
Q8BGB7	Enolase-phosphatase E1 OS=Mus musculus GN=Enoph1 PE=2 SV=1 - [ENOPH_MOUSE]	10.89	2	2	2	11	1.01	0.95	0.95	1.12	1.11	1.12	1.00	1.05	1.09	1.00	1.02	1.17	1.10	28.58	4.92
G3X9U9	Fission 1 (Mitochondrial outer membrane) homolog (Yeast), isoform CRA_c OS=Mus musculus GN=Fis1 PE=4 SV=1 - [G3X9U9_MOUSE]	26.21	3	3	3	6	0.90	0.88	0.94	1.01	1.08	1.01	1.13	1.05	1.11	0.99	1.05	1.03	1.14	16.25	9.25
Q9WTM5	RuvB-like 2 OS=Mus musculus GN=Ruvbl2 PE=2 SV=3 - [RUVB2_MOUSE]	16.63	2	6	6	13	0.95	1.09	1.16	1.13	1.09	0.87	0.90	1.05	1.00	1.07	1.11	1.16	1.16	51.08	5.64
Q8VCF1-	Isoform 2 of Soluble calcium-activated nucleotidase 1 OS=Mus musculus GN=Cant1 - [CANT1_MOUSE]	5.61	3	1	1	1	0.85	1.12	1.32	0.92	1.09	1.02	1.20	1.05	1.23	1.09	1.29	1.09	1.29	24.17	8.38
P47753	F-actin-capping protein subunit alpha-1 OS=Mus musculus GN=Capza1 PE=1 SV=4 - [CAZA1_MOUSE]	42.31	2	5	7	27	1.06	1.04	0.99	1.05	0.95	0.99	1.02	1.05	0.97	1.17	1.14	1.11	1.03	32.92	5.55
E9PZ58	Spermine synthase OS=Mus musculus GN=Sms PE=2 SV=1 - [E9PZ58_MOUSE]	27.91	3	6	6	9	1.03	0.97	1.01	1.08	1.01	1.01	1.07	1.05	1.09	0.95	1.06	1.08	1.12	28.64	5.30
P35822	Receptor-type tyrosine-protein phosphatase kappa OS=Mus musculus GN=Ptprk PE=1 SV=1 - [PTPRK_MOUSE]	7.89	1	7	8	17	0.96	0.99	1.05	0.89	0.95	1.06	1.06	1.05	1.10	0.91	1.08	0.89	1.00	164.08	5.95
Q9DA97	Septin-14 OS=Mus musculus GN=Sept14 PE=2 SV=3 - [SEP14_MOUSE]	8.14	1	1	2	8	0.96	0.69	0.71	0.91	0.94	1.09	1.12	1.05	1.08	0.95	0.99	0.77	0.80	49.78	7.27
Q99JX7	Nuclear RNA export factor 1 OS=Mus musculus GN=Nxf1 PE=1 SV=3 - [NXF1_MOUSE]	5.99	1	3	3	6	0.91	0.94	1.14	0.87	0.96	0.94	1.06	1.05	1.15	0.91	1.08	0.97	1.13	70.26	8.73
Q9D6K5	Synaptojanin-2-binding protein OS=Mus musculus GN=Synj2bp PE=1 SV=1 - [SYJ2B_MOUSE]	30.34	6	4	4	11	0.92	1.26	1.27	1.07	1.04	1.00	1.11	1.05	1.05	1.03	1.10	1.21	1.19	15.81	6.30
G3UW40	MCG4620, isoform CRA_b OS=Mus musculus GN=Mcc PE=4 SV=1 - [G3UW40_MOUSE]	11.71	2	7	7	14	0.93	0.78	0.84	0.83	0.88	0.99	1.04	1.05	1.07	0.85	0.93	0.84	0.85	92.76	5.48

Q8VED9	Galectin-related protein OS=Mus musculus GN=Lgalsl PE=1 SV=1 - [LEGL_MOUSE]	59.30	2	8	8	51	0.94	0.96	1.01	1.01	1.04	0.96	0.99	1.05	1.10	0.94	0.99	0.98	1.04	18.94	5.35
P70444	BH3-interacting domain death agonist OS=Mus musculus GN=Bid PE=1 SV=2 - [BID_MOUSE]	31.28	2	4	4	14	1.12	1.06	0.95	0.95	0.90	1.12	1.06	1.05	0.93	1.06	0.98	1.10	1.00	21.94	4.81
Q8K1J6	CCA tRNA nucleotidyltransferase 1, mitochondrial OS=Mus musculus GN=Trnt1 PE=2 SV=1 - [TRNT1_MOUSE]	12.67	2	4	4	7	1.03	1.22	1.12	1.10	1.05	1.02	0.98	1.05	1.05	0.91	0.91	1.00	0.96	49.86	8.56
P61264	Syntaxin-1B OS=Mus musculus GN=Stx1b PE=1 SV=1 - [STX1B_MOUSE]	60.42	1	18	19	507	1.06	0.99	0.96	0.97	0.92	1.11	1.05	1.05	1.02	1.02	0.95	0.96	0.90	33.22	5.38
P47746	Cannabinoid receptor 1 OS=Mus musculus GN=Cnr1 PE=2 SV=1 - [CNR1_MOUSE]	6.77	1	2	2	3	1.01	0.89	0.88	1.00	0.99	1.00	1.03	1.05	1.02	0.99	1.03	0.96	0.90	52.80	8.27
P00920	Carbonic anhydrase 2 OS=Mus musculus GN=Ca2 PE=1 SV=4 - [CAH2_MOUSE]	66.15	1	15	15	239	0.86	0.90	1.05	0.97	1.12	1.04	1.21	1.05	1.17	1.04	1.19	0.96	1.16	29.01	7.01
Q3UZP4	Small VCP/p97-interacting protein OS=Mus musculus GN=Svip PE=2 SV=1 - [SVIP_MOUSE]	14.29	1	1	1	3	1.02	0.70	0.69	0.81	0.79	1.11	1.09	1.05	1.03	0.63	0.62	0.96	0.95	8.36	8.91
D3YU12	NmrA-like family domain-containing protein 1 OS=Mus musculus GN=Nmral1 PE=2 SV=1 - [D3YU12_MOUSE]	21.74	5	6	6	12	1.05	0.99	0.95	1.01	0.89	1.10	1.04	1.05	1.00	1.04	0.98	1.13	1.05	33.06	6.80
O35188	Fractalkine OS=Mus musculus GN=Cx3cl1 PE=2 SV=3 - [X3CL1_MOUSE]	9.11	1	3	3	21	1.02	1.03	1.03	0.99	0.94	1.09	1.02	1.05	1.04	1.10	1.05	0.98	1.03	42.07	5.54
Q80XI3-2	Isoform 2 of Eukaryotic translation initiation factor 4 gamma 3 OS=Mus musculus GN=Eif4g3 - [IF4G3_MOUSE]	24.36	9	26	29	68	0.96	1.07	1.02	0.97	0.99	1.06	1.10	1.05	1.11	1.02	1.08	0.97	1.03	172.76	5.50
P62806	Histone H4 OS=Mus musculus GN=Hist1h4a PE=1 SV=2 - [H4_MOUSE]	54.37	1	9	9	118	1.06	0.99	0.92	1.08	1.00	1.01	0.94	1.05	1.00	1.07	0.99	1.07	0.98	11.36	11.36
Q8BV13	COP9 signalosome complex subunit 7b OS=Mus musculus GN=Cops7b PE=1 SV=1 - [CSN7B_MOUSE]	25.76	1	5	5	10	1.07	0.95	1.00	0.99	1.01	1.07	1.00	1.05	1.02	1.12	1.08	0.97	0.88	29.67	6.32

Q63810-2	Isoform 2 of Calcineurin subunit B type 1 OS=Mus musculus GN=Ppp3r1 - [CANB1_MOUSE]	77.50	3	15	15	549	1.08	0.78	0.85	0.91	0.88	1.10	1.03	1.05	0.99	0.92	0.89	0.91	0.92	18.20	4.92
Q9CR59	Growth arrest and DNA damage-inducible proteins-interacting protein 1 OS=Mus musculus GN=Gadd45gip1 PE=2 SV=1 - [G45IP_MOUSE]	32.88	1	6	6	16	1.03	0.99	1.04	1.03	1.10	1.16	1.14	1.05	1.13	1.17	1.17	1.26	1.17	25.80	10.27
Q8BX09-2	Isoform 2 of Retinoblastoma-binding protein 5 OS=Mus musculus GN=Rbbp5 - [RBBP5_MOUSE]	16.62	1	1	4	9	1.34	0.89	0.66	0.92	0.68	0.71	0.53	1.05	0.78	0.73	0.54	0.78	0.59	40.74	4.83
Q6ZQM0-	Isoform 4 of E3 ubiquitin-protein ligase rififylin OS=Mus musculus GN=Rffl - [RFFL_MOUSE]	6.48	5	1	1	5	0.94	1.13	1.20	0.93	0.99	1.10	1.17	1.05	1.12	1.00	1.07	1.17	1.25	33.04	5.83
Q78ZM0	Sorting nexin 3 OS=Mus musculus GN=Snx3 PE=2 SV=1 - [Q78ZM0_MOUSE]	41.98	4	7	8	38	0.96	0.89	0.95	0.96	0.95	1.00	1.01	1.05	1.08	0.93	1.01	0.93	1.06	18.75	8.66
Q8BX94	Oxysterol-binding protein-related protein 2 OS=Mus musculus GN=Osbpl2 PE=2 SV=1 - [OSBL2_MOUSE]	3.72	1	1	2	3	0.97	2.68	2.76	1.03	1.07	0.89	0.92	1.05	1.08	1.07	1.10	1.00	1.04	55.35	6.20
Q9CPW9-	Isoform 2 of Methionine aminopeptidase 1D, mitochondrial OS=Mus musculus GN=Metap1d - [MAP12_MOUSE]	6.45	2	1	1	2	0.94	1.41	1.49	0.93	0.98	1.16	1.22	1.05	1.11	1.04	1.10	0.98	1.04	24.04	5.26
O88291	DBIRD complex subunit ZNF326 OS=Mus musculus GN=Znf326 PE=1 SV=1 - [ZN326_MOUSE]	17.41	5	8	8	48	1.00	0.92	0.90	0.89	0.87	1.03	1.10	1.05	0.99	1.03	0.93	1.01	0.97	65.18	5.19
D3Z7V3	Sarcolemmal membrane-associated protein OS=Mus musculus GN=Slmap PE=2 SV=1 - [D3Z7V3_MOUSE]	24.56	13	16	16	29	1.00	0.94	1.04	1.04	1.06	1.07	1.11	1.05	1.13	1.06	1.19	1.01	1.12	90.67	5.25
Q3UHC7-	Isoform 2 of Disabled homolog 2-interacting protein OS=Mus musculus GN=Dab2ip - [DAB2P_MOUSE]	15.77	12	11	14	33	0.98	1.14	1.01	1.10	1.03	1.01	0.92	1.05	1.09	1.00	1.00	1.04	1.03	117.58	6.79
Q7M6Y3-	Isoform 5 of Phosphatidylinositol-binding clathrin assembly protein OS=Mus musculus GN=Picalm - [PICAL_MOUSE]	23.51	3	1	10	43	1.06	0.65	0.59	1.09	1.04	1.06	0.88	1.05	0.98	1.00	1.04	0.84	0.79	70.94	8.37
Q8CCX5-	Isoform 2 of Keratin-like protein KRT222 OS=Mus musculus GN=Krt222 - [KT222_MOUSE]	18.11	2	2	3	9	0.84	1.00	1.18	1.02	0.98	1.21	1.42	1.05	1.24	0.87	1.04	1.00	1.25	29.42	6.07

Q9QYI5	DnaJ homolog subfamily B member 2 OS=Mus musculus GN=Dnajb2 PE=2 SV=2 - [DNJB2_MOUSE]	36.10	2	8	9	32	1.09	1.02	0.91	1.00	0.91	1.05	0.94	1.05	0.97	0.98	0.91	1.04	0.98	30.60	4.98
Q8BGQ1	Spermatogenesis-defective protein 39 homolog OS=Mus musculus GN=Vipas39 PE=1 SV=1 - [SPE39_MOUSE]	18.74	2	8	8	14	1.11	1.03	0.90	1.07	0.99	1.14	1.07	1.05	0.97	1.02	0.94	1.07	0.99	56.59	7.17
P59672	Ankyrin repeat and SAM domain-containing protein 1A OS=Mus musculus GN=Anks1a PE=1 SV=3 - [ANS1A_MOUSE]	0.87	3	1	1	2	0.95	1.03	1.08	0.96	1.02	1.08	1.14	1.05	1.11	0.91	0.97	0.91	0.96	125.16	6.05
Q80VD1	Protein FAM98B OS=Mus musculus GN=Fam98b PE=2 SV=1 - [FA98B_MOUSE]	19.58	1	5	6	14	1.06	1.01	0.92	1.02	0.99	1.10	1.09	1.05	1.04	1.07	1.07	1.09	1.02	45.32	8.50
Q3UM45	Protein phosphatase 1 regulatory subunit 7 OS=Mus musculus GN=Ppp1r7 PE=1 SV=2 - [PP1R7_MOUSE]	54.85	2	18	18	75	0.91	0.88	1.02	0.90	0.99	0.98	1.10	1.05	1.14	0.96	1.10	0.99	1.11	41.27	4.92
Q91WS0	CDGSH iron-sulfur domain-containing protein 1 OS=Mus musculus GN=Cisd1 PE=1 SV=1 - [CISD1_MOUSE]	64.81	1	6	6	63	1.00	0.91	0.91	1.13	1.13	1.05	1.08	1.05	1.06	1.11	1.11	0.99	1.01	12.09	9.06
F6Y6Q2	Phosphoethanolamine/ phosphocholine phosphatase (Fragment) OS=Mus musculus GN=Phospho1 PE=2 SV=1 - [F6Y6Q2_MOUSE]	25.53	2	1	1	2	0.97	0.86	0.88	0.98	1.00	1.06	1.09	1.05	1.08	1.10	1.13	0.92	0.95	5.21	4.34
Q9JHK4	Geranylgeranyl transferase type-2 subunit alpha OS=Mus musculus GN=Rabggta PE=1 SV=1 - [PGTA_MOUSE]	26.63	2	11	11	17	1.01	1.06	0.98	1.15	1.12	0.86	0.87	1.05	0.90	0.98	0.99	1.26	1.07	64.95	5.80
Q5U4C1	G-protein coupled receptor-associated sorting protein 1 OS=Mus musculus GN=Gprasp1 PE=2 SV=1 - [GASP1_MOUSE]	9.58	2	9	9	14	1.11	1.07	0.98	1.09	0.91	1.15	0.96	1.05	0.92	1.12	0.93	1.12	0.94	151.65	5.08
E9PVP5	Molybdopterin synthase catalytic subunit OS=Mus musculus GN=Mocs2 PE=2 SV=1 - [E9PVP5_MOUSE]	9.55	3	1	1	2	1.29	0.74	0.57	1.06	0.82	0.86	0.66	1.05	0.81	0.88	0.69	0.88	0.68	17.10	4.96
A2AJT4	Arginine/serine-rich protein PNISR OS=Mus musculus GN=Pnisr PE=1 SV=1 - [PNISR_MOUSE]	6.71	6	4	5	6	1.06	0.90	0.80	0.94	0.94	0.96	0.92	1.05	0.99	0.97	0.90	0.94	0.83	92.06	10.01

Q8VCC9	Spondin-1 OS=Mus musculus GN=Spon1 PE=2 SV=1 - [SPON1_MOUSE]	11.77	2	8	8	14	1.11	1.00	0.88	1.02	0.93	0.98	0.92	1.05	0.93	1.06	0.99	1.04	0.98	90.76	6.02
Q5DTM8	E3 ubiquitin-protein ligase BRE1A OS=Mus musculus GN=Rnf20 PE=1 SV=2 - [BRE1A_MOUSE]	17.47	5	15	15	35	1.02	1.02	1.00	0.88	0.90	0.94	0.97	1.05	1.04	0.83	0.86	0.95	0.95	113.45	5.96
Q8K482	EMILIN-2 OS=Mus musculus GN=Emilin2 PE=1 SV=1 - [EMIL2_MOUSE]	0.65	1	1	1	1	1.22	1.19	0.97	0.94	0.76	1.35	1.10	1.05	0.86	1.26	1.04	1.11	0.91	117.24	5.62
Q61285	ATP-binding cassette sub-family D member 2 OS=Mus musculus GN=Abcd2 PE=1 SV=1 - [ABCD2_MOUSE]	3.37	1	2	2	3	0.93	1.19	1.28	0.98	1.05	0.98	1.05	1.05	1.13	0.88	0.95	1.01	1.09	83.43	9.09
Q8CD60	UPF0583 protein C15orf59 homolog OS=Mus musculus PE=2 SV=1 - [CO059_MOUSE]	7.88	1	2	2	4	0.98	0.87	0.89	0.91	0.94	1.07	1.09	1.05	1.08	0.99	1.01	1.00	1.02	31.79	4.87
Q9DA19-	Isoform 2 of Corepressor interacting with RBPJ 1 OS=Mus musculus GN=Cir1 - [CIR1_MOUSE]	25.47	2	2	2	4	1.01	0.75	0.74	0.99	0.98	1.00	0.99	1.05	1.04	0.98	0.97	0.89	0.88	12.66	8.50
Q9CRA9-	Isoform 2 of FGFR1 oncogene partner 2 homolog OS=Mus musculus GN=Fgfr1op2 - [FGOP2_MOUSE]	43.26	3	9	9	25	0.91	0.96	1.12	0.86	0.97	1.01	1.11	1.05	1.22	1.01	1.08	0.93	1.06	24.93	5.57
E9QB01	Neural cell adhesion molecule 1 OS=Mus musculus GN=Ncam1 PE=2 SV=1 - [E9QB01_MOUSE]	47.41	5	2	34	404	0.93	0.95	1.28	0.88	0.97	0.92	1.08	1.05	1.22	1.06	1.29	0.95	1.03	93.43	4.91
Q7TSG2	RNA polymerase II subunit A C-terminal domain phosphatase OS=Mus musculus GN=Ctdp1 PE=1 SV=1 - [CTDP1_MOUSE]	6.77	3	4	4	7	0.87	0.93	1.10	0.95	1.08	1.19	1.35	1.05	1.24	1.11	1.24	1.13	1.27	104.49	5.39
Q5DTT2	PH and SEC7 domain-containing protein 1 OS=Mus musculus GN=Psd PE=1 SV=2 - [PSD1_MOUSE]	7.81	2	3	7	11	1.10	1.15	0.81	1.20	1.11	1.01	0.94	1.05	0.95	0.84	0.87	1.07	0.85	109.62	7.05
Q91YJ3	Thymocyte nuclear protein 1 OS=Mus musculus GN=Thyn1 PE=1 SV=1 - [THYN1_MOUSE]	38.05	1	8	8	11	0.98	0.80	0.84	0.93	0.98	1.09	1.11	1.05	1.08	1.03	1.05	1.05	1.15	26.16	9.11
Q8CHP5-	Isoform 2 of Partner of Y14 and mago OS=Mus musculus GN=Wibg - [WIBG_MOUSE]	56.44	2	6	6	18	1.05	0.99	0.92	1.00	0.97	1.20	1.04	1.05	1.02	1.00	0.97	1.01	0.95	22.67	9.48

P61406	Telomerase-binding protein EST1A OS=Mus musculus GN=Smg6 PE=2 SV=1 - [EST1A_MOUSE]	0.92	1	1	1	2	0.91	0.95	1.04	0.88	0.96	1.10	1.19	1.05	1.15	0.96	1.05	0.91	1.00	160.40	6.77
A2AHC3	Calmodulin-regulated spectrin-associated protein 1 OS=Mus musculus GN=Camsap1 PE=1 SV=1 - [CAMP1_MOUSE]	20.18	6	21	21	39	1.01	0.93	1.01	1.03	1.00	1.07	1.10	1.05	1.04	1.07	1.07	1.00	0.98	175.78	6.95
P63239	Neuroendocrine convertase 1 OS=Mus musculus GN=Pcsk1 PE=1 SV=1 - [NEC1_MOUSE]	4.78	1	3	3	12	1.23	0.80	0.67	0.96	0.77	1.18	0.98	1.05	0.86	1.15	0.93	1.08	0.90	84.12	6.43
Q9DC22	DDB1- and CUL4-associated factor 6 OS=Mus musculus GN=Dcaf6 PE=1 SV=1 - [DCAF6_MOUSE]	5.59	1	4	4	7	0.89	1.17	1.27	1.06	1.15	0.99	1.14	1.05	1.14	0.99	1.17	1.05	1.14	97.53	5.22
Q8VEK3	Heterogeneous nuclear ribonucleoprotein U OS=Mus musculus GN=Hnrnpu PE=1 SV=1 - [HNRPU_MOUSE]	30.00	2	17	17	111	0.90	0.99	1.08	0.99	1.07	1.06	1.13	1.05	1.14	1.00	1.09	1.01	1.13	87.86	6.24
Q8BFU3	RING finger protein 214 OS=Mus musculus GN=Rnf214 PE=2 SV=1 - [RN214_MOUSE]	45.21	6	25	26	83	1.01	0.91	0.89	0.92	0.90	0.96	0.94	1.05	0.96	0.93	0.90	0.93	0.89	73.58	6.23
Q9D024	Coiled-coil domain-containing protein 47 OS=Mus musculus GN=Ccdc47 PE=2 SV=2 - [CCD47_MOUSE]	25.88	5	11	11	25	1.04	0.94	0.91	0.99	0.93	1.08	0.98	1.05	0.97	1.02	0.96	1.01	0.96	55.81	4.84
P83870	PHD finger-like domain-containing protein 5A OS=Mus musculus GN=Phf5a PE=1 SV=1 - [PHF5A_MOUSE]	49.09	1	5	5	11	1.01	0.92	0.91	0.88	0.95	1.07	1.03	1.05	1.08	0.91	0.93	0.99	1.01	12.40	8.41
Q8BVI5	Syntaxin-16 OS=Mus musculus GN=Stx16 PE=1 SV=3 - [STX16_MOUSE]	33.74	6	8	8	15	1.03	1.14	1.08	1.07	1.09	0.93	0.96	1.05	1.00	1.04	1.05	1.01	1.05	37.06	5.86
A2AWF8	Receptor-type tyrosine-protein phosphatase eta OS=Mus musculus GN=Ptprj PE=2 SV=1 - [A2AWF8_MOUSE]	4.55	4	4	4	7	1.12	0.92	0.97	1.00	1.01	1.04	0.95	1.05	0.93	1.07	0.96	0.92	0.89	128.63	5.39
Q80XQ2	TBC1 domain family member 5 OS=Mus musculus GN=Tbc1d5 PE=1 SV=2 - [TBCD5_MOUSE]	10.06	1	6	6	11	1.04	0.85	1.04	1.04	1.04	0.98	1.04	1.05	1.02	0.98	1.03	1.00	1.06	91.78	6.79
P52800	Ephrin-B2 OS=Mus musculus GN=Efnb2 PE=1 SV=1 - [EFNB2_MOUSE]	28.87	3	6	6	17	1.05	0.95	0.98	1.03	0.96	1.09	1.03	1.05	1.01	1.01	1.00	1.04	0.98	37.18	8.97

Q80TR0	MKIAA0827 protein (Fragment) OS=Mus musculus GN=Nfat5 PE=2 SV=1 - [Q80TR0_MOUSE]	3.58	1	1	2	6	1.16	1.18	1.02	1.16	1.00	1.08	0.93	1.05	0.91	1.13	0.98	1.17	1.01	111.58	4.67
O88952	Protein lin-7 homolog C OS=Mus musculus GN=Lin7c PE=1 SV=2 - [LIN7C_MOUSE]	59.90	2	5	11	100	0.95	0.98	0.97	0.84	0.90	1.01	1.03	1.06	1.07	1.03	0.99	1.00	1.00	21.82	8.43
P09411	Phosphoglycerate kinase 1 OS=Mus musculus GN=Pgk1 PE=1 SV=4 - [PGK1_MOUSE]	78.42	4	29	29	205	0.97	0.90	0.93	1.06	1.03	1.07	1.08	1.06	1.08	1.02	1.04	1.02	1.03	44.52	7.90
Q3UHK1	Proton myo-inositol cotransporter OS=Mus musculus GN=Slc2a13 PE=2 SV=2 - [MYCT_MOUSE]	10.99	1	6	6	17	0.96	1.09	1.11	1.07	1.14	0.91	0.93	1.06	0.96	1.19	1.22	1.16	1.01	69.02	6.86
Q3V1N5	Protein Heca OS=Mus musculus GN=Heca PE=2 SV=1 - [Q3V1N5_MOUSE]	2.57	1	1	1	6	0.96	1.21	1.27	1.04	1.12	1.04	1.08	1.06	1.10	0.91	1.03	1.04	1.05	59.08	8.60
D3YZ09	H/ACA ribonucleoprotein complex subunit 1 (Fragment) OS=Mus musculus GN=Gar1 PE=2 SV=1 - [D3YZ09_MOUSE]	17.17	5	3	3	5	1.10	0.89	0.77	0.97	0.88	1.10	0.98	1.06	1.00	0.97	0.84	1.07	0.93	20.44	10.14
Q3TC93	HCLS1-binding protein 3 OS=Mus musculus GN=Hs1bp3 PE=1 SV=2 - [H1BP3_MOUSE]	19.75	1	4	4	8	1.02	1.08	1.18	1.03	1.01	1.19	1.16	1.06	1.03	1.04	1.08	1.01	1.01	43.66	4.98
Q6PGH2	Hematological and neurological expressed 1-like protein OS=Mus musculus GN=Hn1l PE=2 SV=1 - [HN1L_MOUSE]	46.32	2	5	5	12	1.21	0.94	0.77	0.91	0.73	1.15	0.93	1.06	0.87	1.12	0.92	1.10	0.94	20.01	8.62
Q3U2E2	Zinc finger MYM-type protein 5 OS=Mus musculus GN=Zmym5 PE=2 SV=2 - [ZMYM5_MOUSE]	0.96	1	1	1	1	1.02	0.97	0.95	1.22	1.19	1.12	1.09	1.06	1.03	1.20	1.18	0.96	0.94	70.03	7.97
Q9D2U5	LSM domain-containing protein 1 OS=Mus musculus GN=Lsmd1 PE=2 SV=1 - [LSMD1_MOUSE]	7.20	1	1	1	1	0.74	1.07	1.45	0.96	1.30	1.15	1.55	1.06	1.43	0.83	1.13	0.93	1.27	13.42	5.50
Q8CFR5	Dystrobrevin alpha OS=Mus musculus GN=Dtna PE=2 SV=1 - [Q8CFR5_MOUSE]	21.41	19	11	11	29	1.02	1.13	1.08	1.03	0.97	1.12	1.14	1.06	1.11	1.14	1.10	1.07	1.05	76.90	6.89
Q5EBG8	Uncharacterized protein C1orf50 homolog OS=Mus musculus PE=2 SV=1 - [CA050_MOUSE]	35.68	1	4	4	8	1.07	1.00	1.02	0.94	0.92	1.09	1.00	1.06	0.99	1.04	0.96	0.99	1.02	21.85	5.53

B2RQC7	DIP2 disco-interacting protein 2 homolog B (Drosophila) OS=Mus musculus GN=Dip2b PE=2 SV=1 - [B2RQC7_MOUSE]	12.84	3	1	15	31	0.85	0.98	1.16	0.94	1.11	0.94	1.10	1.06	1.24	1.10	1.30	1.04	1.23	146.60	8.03
E9PVV3	Potassium voltage-gated channel subfamily C member 1 OS=Mus musculus GN=Kcnc1 PE=2 SV=1 - [E9PVV3_MOUSE]	8.38	2	3	3	9	0.91	1.23	1.31	1.03	1.15	1.05	1.08	1.06	1.14	0.92	0.92	1.04	1.08	65.81	6.18
P09450	Transcription factor jun-B OS=Mus musculus GN=Junb PE=1 SV=1 - [JUNB_MOUSE]	10.17	1	2	2	2	1.03	0.94	0.91	1.04	1.01	1.21	1.17	1.06	1.02	1.06	1.03	1.09	1.06	35.74	9.22
P07628	Kallikrein 1-related peptidase b8 OS=Mus musculus GN=Klk1b8 PE=2 SV=1 - [K1KB8_MOUSE]	14.56	1	1	1	1	0.71	0.79	1.12	0.84	1.18	0.99	1.39	1.06	1.49	1.19	1.69	1.02	1.45	28.51	8.00
Q9JKK7	Tropomodulin-2 OS=Mus musculus GN=Tmod2 PE=1 SV=2 - [TMOD2_MOUSE]	67.81	3	17	17	180	0.97	0.84	0.84	0.86	0.90	1.03	1.08	1.06	1.10	0.91	0.93	0.88	0.90	39.49	5.35
E9Q3U1	Ubiquitin-conjugating enzyme E2 H OS=Mus musculus GN=Ube2h PE=2 SV=1 - [E9Q3U1_MOUSE]	19.74	4	2	2	4	0.98	0.93	0.85	1.02	1.04	1.12	1.11	1.06	1.07	0.97	0.93	0.89	0.86	17.16	4.67
Q9Z1T2	Thrombospondin-4 OS=Mus musculus GN=Thbs4 PE=1 SV=1 - [TSP4_MOUSE]	6.13	2	5	5	10	1.04	1.12	1.12	1.07	1.01	1.03	1.02	1.06	1.01	0.98	0.92	1.05	1.00	106.30	4.67
O35737	Heterogeneous nuclear ribonucleoprotein H OS=Mus musculus GN=Hnrnph1 PE=1 SV=3 - [HNRH1_MOUSE]	33.18	2	3	11	78	0.88	1.03	1.12	0.89	1.10	0.98	1.27	1.06	1.22	0.93	1.09	0.92	1.11	49.17	6.30
E9Q7Z8	Protein Ythdc1 OS=Mus musculus GN=Ythdc1 PE=2 SV=1 - [E9Q7Z8_MOUSE]	5.07	4	4	4	11	0.86	0.72	0.77	0.94	1.09	1.10	1.34	1.06	1.26	1.03	1.14	0.82	0.91	82.70	5.91
Q9CQF3	Cleavage and polyadenylation specificity factor subunit 5 OS=Mus musculus GN=Nudt21 PE=2 SV=1 - [CPSF5_MOUSE]	13.22	1	3	3	7	0.83	0.90	0.93	1.05	1.16	1.03	1.23	1.06	1.27	1.02	1.30	0.95	1.15	26.22	8.82
O08579	Emerin OS=Mus musculus GN=Emd PE=1 SV=1 - [EMD_MOUSE]	23.17	4	5	5	15	0.89	0.79	0.94	0.96	1.19	1.01	1.35	1.06	1.20	0.95	1.14	0.85	1.08	29.42	5.01

Q8K194	U4/U6.U5 small nuclear ribonucleoprotein 27 kDa protein OS=Mus musculus GN=Snrnp27 PE=2 SV=1 - [SNR27_MOUSE]	19.35	2	4	4	9	0.99	0.78	0.80	1.04	1.09	1.08	0.93	1.06	0.99	1.03	1.04	0.91	0.88	18.87	11.62
P59048	p53 and DNA damage-regulated protein 1 OS=Mus musculus GN=Pdrg1 PE=2 SV=1 - [PDRG1_MOUSE]	9.77	1	1	1	2	0.81	0.83	1.03	1.14	1.41	1.53	1.89	1.06	1.30	1.09	1.35	0.80	0.99	15.37	8.10
Q8BTS4	Nuclear pore complex protein Nup54 OS=Mus musculus GN=Nup54 PE=1 SV=1 - [NUP54_MOUSE]	6.08	1	2	2	4	1.04	1.03	0.99	1.07	1.02	1.05	1.01	1.06	1.01	1.02	0.98	0.99	0.95	55.70	7.02
E9Q6R7	Protein Utrn OS=Mus musculus GN=Utrn PE=2 SV=1 - [E9Q6R7_MOUSE]	1.49	3	3	5	15	0.89	1.04	1.17	0.96	1.05	1.00	1.10	1.06	1.15	1.06	1.17	0.98	1.08	392.46	5.33
D3Z7G3	Protein 2010107G23Rik (Fragment) OS=Mus musculus GN=2010107G23Rik PE=2 SV=1 - [D3Z7G3_MOUSE]	14.14	4	1	1	4	0.87	1.15	1.44	1.08	1.32	0.86	1.23	1.06	1.38	1.00	1.25	0.84	1.21	10.84	11.15
Q922R1	UPF0183 protein C16orf70 homolog OS=Mus musculus PE=2 SV=2 - [CP070_MOUSE]	7.11	3	2	2	4	1.07	1.27	1.19	1.36	1.28	0.87	0.82	1.06	0.99	1.14	1.07	1.25	1.18	47.39	7.74
Q9CQB4	Cytochrome b-c1 complex subunit 7 OS=Mus musculus GN=Uqcrb PE=3 SV=1 - [Q9CQB4_MOUSE]	54.05	2	9	9	174	0.97	0.80	0.83	0.86	0.89	1.06	1.08	1.06	1.10	0.95	0.99	0.90	0.94	13.55	9.11
Q9CPX7	28S ribosomal protein S16, mitochondrial OS=Mus musculus GN=Mrps16 PE=2 SV=1 - [RT16_MOUSE]	20.74	1	2	2	4	0.77	0.85	1.10	0.96	1.25	0.98	1.27	1.06	1.37	1.02	1.32	0.94	1.23	15.18	9.67
Q569Z5	Probable ATP-dependent RNA helicase DDX46 OS=Mus musculus GN=Ddx46 PE=1 SV=2 - [DDX46_MOUSE]	9.11	3	9	9	19	0.95	1.12	1.13	1.04	1.05	1.00	1.01	1.06	1.07	0.98	1.07	1.09	1.05	117.38	9.26
P59281-2	Isoform 2 of Rho GTPase-activating protein 39 OS=Mus musculus GN=Arhgap39 - [RHG39_MOUSE]	16.08	5	12	12	25	0.99	1.13	1.18	1.09	1.11	1.07	1.18	1.06	1.09	1.10	1.14	1.05	1.14	121.40	7.52
Q8CBY0	Glutamyl-tRNA(Gln) amidotransferase subunit C, mitochondrial OS=Mus musculus GN=Gatc PE=2 SV=1 - [GATC_MOUSE]	29.68	1	3	3	14	1.00	0.82	0.99	0.92	1.03	1.22	1.13	1.06	1.07	0.92	0.92	0.88	0.84	16.66	5.16

Q61387	Cytochrome c oxidase subunit 7A-related protein, mitochondrial OS=Mus musculus GN=Cox7a2l PE=2 SV=1 - [COX7R_MOUSE]	48.65	2	3	3	4	0.92	1.15	1.14	1.21	1.17	1.22	1.18	1.06	1.24	1.19	1.21	1.14	1.19	12.39	9.60
Q0VAW6	Protein 8030462N17Rik OS=Mus musculus GN=8030462N17Rik PE=2 SV=1 - [Q0VAW6_MOUSE]	16.29	3	5	5	21	1.02	0.78	0.82	1.01	1.00	1.18	1.10	1.06	1.14	1.04	0.98	0.92	0.97	42.75	4.96
Q8VC66	Afadin- and alpha-actinin-binding protein OS=Mus musculus GN=Ssx2ip PE=1 SV=1 - [ADIP_MOUSE]	6.83	1	3	3	5	0.96	1.16	1.10	0.82	0.87	1.04	1.10	1.06	1.10	1.03	1.07	1.06	1.13	70.91	6.65
Q9JKL4	NADH dehydrogenase [ubiquinone] 1 alpha subcomplex assembly factor 3 OS=Mus musculus GN=Ndufaf3 PE=2 SV=1 - [NDUF3_MOUSE]	5.95	1	1	1	2	1.07	1.00	0.94	1.02	0.95	1.12	1.05	1.06	0.99	0.87	0.81	1.00	0.94	20.72	8.05
P34152-3	Isoform 3 of Focal adhesion kinase 1 OS=Mus musculus GN=Ptk2 - [FAK1_MOUSE]	16.25	9	11	12	38	0.90	0.95	1.12	0.98	1.13	1.02	1.11	1.06	1.19	1.00	1.11	0.98	1.03	119.17	6.62
Q9CZ13	Cytochrome b-c1 complex subunit 1, mitochondrial OS=Mus musculus GN=Uqcrc1 PE=1 SV=2 - [QCR1_MOUSE]	47.92	1	15	15	189	0.98	1.09	1.08	1.01	1.02	1.04	1.06	1.06	1.09	1.05	1.11	1.08	1.11	52.82	6.21
Q68FL1	Bai3 protein (Fragment) OS=Mus musculus GN=Bai3 PE=2 SV=1 - [Q68FL1_MOUSE]	11.39	3	9	10	29	1.10	1.04	1.02	1.16	1.07	1.05	1.00	1.06	0.98	1.02	0.98	1.04	0.97	143.85	6.67
Q8CI43	Myosin light chain 6B OS=Mus musculus GN=Myl6b PE=2 SV=1 - [MYL6B_MOUSE]	65.70	1	10	11	50	0.98	0.82	0.78	0.91	0.95	1.15	1.18	1.06	1.09	1.03	1.01	0.91	0.94	22.73	5.53
B2RXE2	Sodium/hydrogen exchanger OS=Mus musculus GN=Slc9a5 PE=2 SV=1 - [B2RXE2_MOUSE]	2.12	1	1	1	2	1.06	1.43	1.34	1.12	1.06	1.16	1.08	1.06	0.99	1.00	0.94	1.14	1.07	98.95	7.31
Q921H9	Sel1 repeat-containing protein 1 OS=Mus musculus GN=Selrc1 PE=1 SV=1 - [SELR1_MOUSE]	35.50	1	8	8	19	0.98	0.81	0.84	0.92	0.96	1.03	1.09	1.06	1.12	0.96	0.97	0.88	0.92	25.60	6.29
Q8CIN4	Serine/threonine-protein kinase PAK 2 OS=Mus musculus GN=Pak2 PE=1 SV=1 - [PAK2_MOUSE]	52.29	1	11	18	77	1.05	0.90	0.83	0.95	0.95	1.03	0.95	1.06	1.06	0.99	0.92	0.96	0.96	57.89	5.77
F8VPP8	Protein Zc3h7b OS=Mus musculus GN=Zc3h7b PE=2 SV=1 - [F8VPP8_MOUSE]	1.02	1	1	1	3	0.95	1.35	1.56	1.05	1.10	1.00	0.89	1.06	1.11	1.05	1.10	0.97	1.02	110.22	7.42

Q8R1Q8	Cytoplasmic dynein 1 light intermediate chain 1 OS=Mus musculus GN=Dync1li1 PE=1 SV=1 - [DC1L1_MOUSE]	53.15	1	22	23	114	0.96	0.88	0.88	0.92	0.96	1.01	1.03	1.06	1.08	0.96	1.00	0.98	1.03	56.58	6.42
B1ARW6	Metal regulatory transcription factor 1 (Fragment) OS=Mus musculus GN=Mtf1 PE=2 SV=1 - [B1ARW6_MOUSE]	7.75	2	1	1	2	1.08	0.88	0.81	0.97	0.90	1.01	0.93	1.06	0.97	0.91	0.84	1.08	1.00	15.84	4.35
E9Q456	Tropomyosin alpha-1 chain OS=Mus musculus GN=Tpm1 PE=2 SV=1 - [E9Q456_MOUSE]	52.42	2	1	16	346	0.97	1.18	1.12	0.90	0.91	3.90	3.75	1.06	1.09	1.12	1.07	0.95	0.98	28.65	4.77
A2AAY5	SH3 and PX domain-containing protein 2B OS=Mus musculus GN=Sh3pxd2b PE=1 SV=1 - [SPD2B_MOUSE]	1.87	1	2	2	9	0.96	1.20	1.18	0.98	1.01	0.99	1.08	1.06	1.06	1.02	1.05	1.02	1.03	101.45	8.66
P97457	Myosin regulatory light chain 2, skeletal muscle isoform OS=Mus musculus GN=Mylpf PE=1 SV=3 - [MLRS_MOUSE]	76.92	2	12	12	32	1.11	1.25	1.13	1.02	0.96	11.42	8.29	1.06	1.05	1.24	1.16	1.12	1.06	18.94	4.92
Q02357-6	Isoform 6 of Ankyrin-1 OS=Mus musculus GN=Ank1 - [ANK1_MOUSE]	17.72	17	19	21	46	1.00	1.13	1.08	0.97	1.00	1.05	0.97	1.06	1.05	1.04	1.00	1.04	0.97	209.42	6.74
Q810B8	SLIT and NTRK-like protein 4 OS=Mus musculus GN=Slitrk4 PE=2 SV=2 - [SLIK4_MOUSE]	4.90	1	3	3	6	1.20	1.42	1.04	1.20	0.89	1.14	0.92	1.06	0.90	1.17	0.96	1.14	0.92	94.48	7.80
P14211	Calreticulin OS=Mus musculus GN=Calr PE=1 SV=1 - [CALR_MOUSE]	69.95	1	25	25	231	0.94	0.75	0.81	0.86	0.92	1.06	1.12	1.06	1.14	1.00	1.05	0.92	1.01	47.96	4.49
Q9JI11	Serine/threonine-protein kinase 4 OS=Mus musculus GN=Stk4 PE=1 SV=1 - [STK4_MOUSE]	8.83	4	1	3	5	0.80	0.87	1.09	0.84	1.04	1.03	1.28	1.06	1.32	1.11	1.38	1.02	1.28	55.51	5.19
Q8CI33-2	Isoform 2 of CWF19-like protein 1 OS=Mus musculus GN=Cwf19l1 - [C19L1_MOUSE]	3.51	2	1	1	2	1.04	1.17	1.13	1.05	1.01	1.12	1.07	1.06	1.02	1.10	1.06	1.08	1.04	45.09	7.68
Q9DCM4	Dynein light chain 4, axonemal OS=Mus musculus GN=Dnal4 PE=2 SV=2 - [DNAL4_MOUSE]	7.62	2	1	1	1	1.00	1.17	1.17	1.00	1.00	1.10	1.09	1.06	1.06	1.09	1.09	1.15	1.16	12.01	5.49
Q6IR34-3	Isoform 3 of G-protein-signaling modulator 1 OS=Mus musculus GN=Gpsm1 - [GPSM1_MOUSE]	18.92	10	8	8	14	1.04	0.83	0.78	0.97	0.90	1.28	1.14	1.06	1.01	1.28	1.27	1.50	1.26	71.88	6.15

Q8VBX6-	Isoform 2 of Multiple PDZ domain protein OS=Mus musculus GN=Mpdz - [MPDZ_MOUSE]	4.10	11	5	5	10	1.00	1.14	1.07	0.96	1.04	1.09	1.05	1.06	1.05	1.00	1.07	1.01	1.09	214.93	5.01
Q9Z1X4	Interleukin enhancer-binding factor 3 OS=Mus musculus GN=Ilf3 PE=1 SV=2 - [ILF3_MOUSE]	20.27	4	11	12	42	0.98	1.06	1.16	1.05	1.12	1.02	1.14	1.06	1.06	1.00	1.03	1.02	1.09	95.96	8.76
Q9QXV8	Protein sprouty homolog 2 OS=Mus musculus GN=Spry2 PE=2 SV=1 - [SPY2_MOUSE]	5.08	1	1	1	2	0.98	0.91	0.93	0.83	0.85	1.05	1.07	1.06	1.08	0.69	0.70	1.12	1.14	34.60	8.29
Q8C1B1	Calmodulin-regulated spectrin-associated protein 2 OS=Mus musculus GN=Camsap2 PE=1 SV=3 - [CAMP2_MOUSE]	21.15	5	23	24	57	0.94	0.97	0.95	0.95	1.05	1.01	1.03	1.06	1.11	1.00	1.05	0.98	1.04	164.23	6.86
F8WI57	Actin-like protein 6B OS=Mus musculus GN=Actl6b PE=2 SV=1 - [F8WI57_MOUSE]	12.07	4	3	4	7	1.10	0.95	0.92	0.88	0.91	1.17	1.04	1.06	1.00	1.03	1.02	1.13	1.06	41.66	5.36
Q9QUR7	Peptidyl-prolyl cis-trans isomerase NIMA-interacting 1 OS=Mus musculus GN=Pin1 PE=1 SV=1 - [PIN1_MOUSE]	56.97	1	9	9	79	0.96	0.79	0.79	0.89	0.92	1.11	1.12	1.06	1.13	0.98	1.04	0.85	0.94	18.36	8.79
E0CYH4	WD repeat-containing protein 26 OS=Mus musculus GN=Wdr26 PE=2 SV=1 - [E0CYH4_MOUSE]	13.44	3	5	5	14	1.03	1.25	1.21	1.04	0.98	0.92	0.86	1.06	0.98	1.03	0.99	1.06	1.03	68.84	6.15
Q8CHU3	Epsin-2 OS=Mus musculus GN=Epn2 PE=1 SV=1 - [EPN2_MOUSE]	30.92	4	9	12	109	1.01	0.81	0.93	0.93	0.91	1.01	1.07	1.06	1.07	0.93	0.92	0.90	0.94	63.43	8.16
Q922J3	CAP-Gly domain-containing linker protein 1 OS=Mus musculus GN=Clip1 PE=1 SV=1 - [CLIP1_MOUSE]	58.45	3	6	80	343	1.14	0.98	0.82	0.95	0.86	1.17	1.09	1.06	0.99	1.00	1.04	0.96	0.87	155.72	5.24
Q9JKV1	Proteasomal ubiquitin receptor ADRM1 OS=Mus musculus GN=Adrm1 PE=1 SV=2 - [ADRM1_MOUSE]	14.50	2	5	5	9	0.99	1.11	1.02	0.93	0.99	1.15	1.19	1.06	1.17	1.06	1.18	1.17	1.19	42.03	5.07
Q91ZV7	Plexin domain-containing protein 1 OS=Mus musculus GN=Plxdc1 PE=1 SV=2 - [PLDX1_MOUSE]	4.80	2	2	2	2	0.78	1.27	1.62	0.85	1.08	1.21	1.54	1.06	1.35	1.04	1.33	0.90	1.16	55.60	6.62
Q9WV92-	Isoform 7 of Band 4.1-like protein 3 OS=Mus musculus GN=Epb41l3 - [E41L3_MOUSE]	54.31	1	1	37	260	1.08	1.01	0.94	0.91	0.85	1.09	1.01	1.06	0.98	1.13	1.05	0.82	0.77	91.79	5.50

Q8R0H9	ADP-ribosylation factor-binding protein GGA1 OS=Mus musculus GN=Gga1 PE=1 SV=1 - [GGA1_MOUSE]	11.02	1	6	6	13	1.03	0.97	0.94	1.06	0.99	1.04	0.98	1.06	1.08	1.00	0.95	0.98	0.91	69.93	5.27
Q9R060	Cytosolic Fe-S cluster assembly factor NUBP1 OS=Mus musculus GN=Nubp1 PE=1 SV=1 - [NUBP1_MOUSE]	5.63	1	1	1	1	1.19	1.31	1.10	0.55	0.46	1.21	1.01	1.06	0.89	1.59	1.34	1.74	1.46	34.06	5.90
Q99NF7	Ppm1b protein OS=Mus musculus GN=Ppm1b PE=2 SV=1 - [Q99NF7_MOUSE]	22.64	5	1	9	29	0.89	0.81	0.91	0.76	0.85	0.97	1.08	1.06	1.18	1.03	1.16	1.26	1.41	52.08	5.11
Q8C3X2	Coiled-coil domain-containing protein 90B, mitochondrial OS=Mus musculus GN=Ccdc90b PE=2 SV=1 - [CC90B_MOUSE]	13.28	5	4	4	6	1.11	0.94	0.82	1.00	0.82	1.13	1.03	1.06	0.90	1.05	0.86	1.04	0.92	29.58	8.53
Q9Z2I8-2	Isoform 2 of Succinyl-CoA ligase [GDP-forming] subunit beta, mitochondrial OS=Mus musculus GN=Suclg2 - [SUCB2_MOUSE]	23.44	2	8	8	12	1.01	0.91	0.83	1.11	1.03	1.07	1.05	1.06	1.07	1.04	1.04	1.03	1.04	41.32	5.44
P97762	Retinitis pigmentosa 9 protein homolog OS=Mus musculus GN=rp9 PE=1 SV=1 - [RP9_MOUSE]	3.76	1	1	1	1	1.10	0.97	0.88	0.83	0.75	0.91	0.82	1.06	0.96	1.15	1.05	0.85	0.78	25.25	9.83
Q3UVR3-	Isoform 2 of Tau-tubulin kinase 2 OS=Mus musculus GN=Ttbk2 - [TTBK2_MOUSE]	2.91	4	3	3	4	0.96	0.97	1.01	0.92	0.95	1.17	1.21	1.06	1.10	1.19	1.28	1.27	1.51	132.17	6.52
Q99LH9	SH3 domain-binding protein 5-like OS=Mus musculus GN=Sh3bp5l PE=1 SV=1 - [3BP5L_MOUSE]	15.05	1	4	4	12	1.18	0.86	0.80	0.77	0.72	1.13	0.88	1.06	0.90	0.94	0.87	0.98	0.80	43.35	6.00
Q99P88	Nuclear pore complex protein Nup155 OS=Mus musculus GN=Nup155 PE=2 SV=1 - [NU155_MOUSE]	1.22	1	1	1	2	1.46	0.87	0.59	1.07	0.73	1.36	0.93	1.06	0.72	1.11	0.76	1.19	0.81	155.02	6.15
Q9Z211	Peroxisomal membrane protein 11A OS=Mus musculus GN=Pex11a PE=2 SV=1 - [PX11A_MOUSE]	5.28	1	1	1	1	0.74	1.23	1.66	0.84	1.13	1.25	1.67	1.06	1.43	1.14	1.54	0.61	0.83	28.14	9.83
P21107-2	Isoform 2 of Tropomyosin alpha-3 chain OS=Mus musculus GN=Tpm3 - [TPM3_MOUSE]	58.06	3	8	22	411	1.05	0.98	0.88	0.98	0.84	1.12	1.00	1.06	0.98	1.03	0.99	0.99	0.93	29.00	4.78
D3YUS5	Protein Rasal2 OS=Mus musculus GN=Rasal2 PE=2 SV=1 - [D3YUS5_MOUSE]	18.32	6	13	15	38	0.95	0.98	1.12	0.91	0.96	0.99	1.08	1.06	1.09	0.95	1.09	0.97	1.10	141.92	7.80

Q61029-3	Isoform Epsilon of Lamina-associated polypeptide 2, isoforms beta/delta/epsilon/gamma OS=Mus musculus GN=Tmpo - [LAP2B_MOUSE]	44.66	5	5	14	43	0.94	1.10	0.97	0.98	0.95	1.08	1.16	1.06	1.16	1.03	1.14	0.98	0.97	46.02	9.22
F8VQA4	Peptidyl-glycine alpha-amidating monooxygenase OS=Mus musculus GN=Pam PE=2 SV=1 - [F8VQA4_MOUSE]	22.60	6	16	16	65	1.30	0.99	0.74	0.95	0.71	1.01	0.78	1.06	0.82	1.06	0.84	1.07	0.81	108.76	6.60
Q8R050-2	Isoform 2 of Eukaryotic peptide chain release factor GTP-binding subunit ERF3A OS=Mus musculus GN=Gspt1 - [ERF3A_MOUSE]	30.87	3	4	16	51	1.01	0.99	0.99	0.98	0.91	0.83	0.82	1.06	1.05	0.92	0.85	1.02	1.13	68.48	5.21
Q9JKC6	Cell cycle exit and neuronal differentiation protein 1 OS=Mus musculus GN=Cend1 PE=1 SV=1 - [CEND_MOUSE]	73.15	1	8	8	148	1.01	0.82	0.81	0.95	1.00	1.21	1.22	1.06	1.11	1.07	1.09	0.93	0.94	14.98	8.97
Q9ES00	Ubiquitin conjugation factor E4 B OS=Mus musculus GN=Ube4b PE=1 SV=3 - [UBE4B_MOUSE]	2.13	4	1	2	9	1.14	1.35	1.18	1.11	0.97	1.20	1.05	1.06	0.93	1.09	0.96	1.42	1.25	133.23	6.07
Q78YZ6-3	Isoform 3 of Short coiled-coil protein OS=Mus musculus GN=Scoc - [SCOC_MOUSE]	56.79	3	3	3	18	1.05	0.78	0.84	0.89	0.93	1.05	1.11	1.06	1.05	1.01	1.04	0.96	1.02	9.28	4.75
Q68FH0-2	Isoform 2 of Plakophilin-4 OS=Mus musculus GN=Pkp4 - [PKP4_MOUSE]	21.71	6	17	20	44	0.97	0.97	1.07	1.01	1.04	1.00	1.02	1.06	1.02	1.01	1.01	1.07	1.07	126.70	8.95
F7AI27	Leucine-rich repeat-containing protein 16A (Fragment) OS=Mus musculus GN=Lrrc16a PE=2 SV=1 - [F7AI27_MOUSE]	4.40	4	2	3	5	0.98	0.93	0.94	0.90	0.92	1.12	1.13	1.06	1.08	1.06	1.08	1.11	1.13	102.53	7.94
Q91V24	ATP-binding cassette sub-family A member 7 OS=Mus musculus GN=Abca7 PE=1 SV=1 - [ABCA7_MOUSE]	2.69	2	4	4	9	1.01	0.95	0.92	1.14	1.13	0.92	0.95	1.06	1.02	1.10	1.03	0.99	1.01	236.73	7.28
Q8K4E0	Alstrom syndrome protein 1 homolog OS=Mus musculus GN=Alms1 PE=1 SV=2 - [ALMS1_MOUSE]	0.46	1	1	1	2	1.08	0.96	0.89	1.07	0.99	1.17	1.08	1.06	0.98	1.00	0.93	1.04	0.96	359.99	6.39
P01831	Thy-1 membrane glycoprotein OS=Mus musculus GN=Thy1 PE=1 SV=1 - [THY1_MOUSE]	32.10	1	5	5	347	1.00	1.12	1.12	0.95	0.95	1.05	1.04	1.06	1.07	1.03	1.03	1.04	1.03	18.07	8.97

P70170-3	Isoform SUR2C of ATP-binding cassette sub-family C member 9 OS=Mus musculus GN=Abcc9 - [ABCC9_MOUSE]	0.46	5	1	1	1	1.02	1.05	1.03	1.13	1.11	1.43	1.40	1.06	1.04	1.13	1.11	1.34	1.32	169.98	7.24
E9PZA8	Synaptotagmin-7 OS=Mus musculus GN=Syt7 PE=2 SV=1 - [E9PZA8_MOUSE]	31.92	2	3	14	37	0.85	0.98	1.03	0.90	0.98	0.94	1.04	1.06	1.21	1.05	1.25	1.02	1.04	63.06	9.44
Q8K3W3	Protein CASC3 OS=Mus musculus GN=Casc3 PE=1 SV=3 - [CASC3_MOUSE]	11.75	1	7	7	13	1.08	0.85	0.91	1.04	0.90	1.15	1.07	1.06	1.04	1.09	1.02	1.07	0.92	75.72	6.44
F8WHU6	Cadherin-9 OS=Mus musculus GN=Cdh9 PE=2 SV=1 - [F8WHU6_MOUSE]	11.04	2	4	7	12	0.97	1.31	1.44	0.86	0.88	1.11	0.98	1.06	1.09	0.93	0.96	0.97	0.99	88.47	4.96
Q9D0E3	LysM and putative peptidoglycan-binding domain-containing protein 1 OS=Mus musculus GN=Lysmd1 PE=1 SV=1 - [LYSM1_MOUSE]	53.54	1	7	8	26	0.92	0.95	0.97	0.85	0.98	1.07	1.19	1.06	1.13	0.98	1.05	0.82	1.01	24.80	8.92
Q91ZR2	Sorting nexin-18 OS=Mus musculus GN=Snx18 PE=2 SV=1 - [SNX18_MOUSE]	1.95	2	1	1	3	1.10	1.16	1.05	1.03	0.94	1.14	1.04	1.06	0.96	1.05	0.95	1.08	0.99	67.86	6.67
D3YZT0	60S ribosomal protein L9 (Fragment) OS=Mus musculus GN=Rpl9 PE=2 SV=1 - [D3YZT0_MOUSE]	43.75	4	3	3	6	1.10	1.20	1.17	1.28	0.96	1.01	0.82	1.06	0.85	0.88	0.81	1.00	0.98	9.27	10.74
E9PWB1	Pleckstrin homology-like domain family B member 1 OS=Mus musculus GN=Phldb1 PE=2 SV=1 - [E9PWB1_MOUSE]	7.93	12	7	7	10	0.96	0.99	1.06	0.96	1.02	1.06	1.12	1.06	1.10	1.10	1.13	1.09	1.17	144.95	8.88
P09242	Alkaline phosphatase, tissue-nonspecific isozyme OS=Mus musculus GN=Alpl PE=1 SV=2 - [PPBT_MOUSE]	13.55	2	6	6	14	0.99	1.07	0.96	1.05	1.04	1.04	1.08	1.06	1.04	1.09	1.02	0.90	0.86	57.48	7.01
Q8R4N0	Citrate lyase subunit beta-like protein, mitochondrial OS=Mus musculus GN=Clybl PE=2 SV=2 - [CLYBL_MOUSE]	32.54	1	9	9	20	1.00	0.87	0.81	0.95	0.97	1.10	1.10	1.06	1.08	1.02	1.03	1.13	1.03	37.52	8.54
Q4LDD4-	Isoform 3 of Arf-GAP with Rho-GAP domain, ANK repeat and PH domain-containing protein 1 OS=Mus musculus GN=Arap1 - [ARAP1_MOUSE]	0.67	3	1	1	1	0.87	1.19	1.37	1.01	1.16	0.94	1.07	1.06	1.22	1.18	1.36	1.21	1.39	134.55	6.24

G3UZ30	Protein phosphatase 1 regulatory subunit 11 (Fragment) OS=Mus musculus GN=Ppp1r11 PE=2 SV=2 - [G3UZ30_MOUSE]	53.33	3	4	4	158	1.05	0.76	0.73	0.95	0.89	1.18	1.08	1.06	1.01	1.03	1.00	0.96	0.89	13.83	8.59
P62748	Hippocalcin-like protein 1 OS=Mus musculus GN=Hpcal1 PE=2 SV=2 - [HPCL1_MOUSE]	74.61	1	4	16	255	1.58	0.82	0.59	0.94	0.59	1.07	0.68	1.06	0.65	1.12	0.72	1.39	0.91	22.32	5.50
E9Q5C9	Protein Nolc1 OS=Mus musculus GN=Nolc1 PE=2 SV=1 - [E9Q5C9_MOUSE]	19.23	1	15	15	35	1.05	0.85	0.83	1.06	0.98	0.98	0.95	1.06	1.02	1.07	1.01	0.91	0.86	73.65	9.36
Q9CWK8	Sorting nexin-2 OS=Mus musculus GN=Snx2 PE=1 SV=2 - [SNX2_MOUSE]	27.94	1	12	14	28	1.02	1.12	1.09	1.13	1.06	1.06	0.96	1.06	0.99	1.02	1.02	1.06	0.97	58.43	5.12
Q9CQG2-	Isoform 2 of Methyltransferase-like protein 16 OS=Mus musculus GN=Mettl16 - [MET16_MOUSE]	3.63	3	1	1	2	1.01	1.17	1.16	1.41	1.40	0.95	0.93	1.06	1.05	1.16	1.15	1.19	1.18	58.77	7.37
G5E8E0	Regulator of G-protein signaling 17, isoform CRA_b OS=Mus musculus GN=Rgs17 PE=4 SV=1 - [G5E8E0_MOUSE]	23.91	3	4	4	7	1.00	1.13	1.16	1.05	1.00	1.09	1.10	1.06	1.06	1.03	1.05	1.08	1.10	26.61	6.01
Q8BXR1	Probable cationic amino acid transporter OS=Mus musculus GN=Slc7a14 PE=2 SV=1 - [S7A14_MOUSE]	15.30	2	9	9	26	1.28	1.01	0.95	1.21	0.90	0.95	0.76	1.06	0.89	1.14	0.97	1.22	0.97	83.93	5.35
E9QK04	Neogenin OS=Mus musculus GN=Neo1 PE=2 SV=1 - [E9QK04_MOUSE]	17.90	6	18	18	45	1.06	1.07	0.99	0.99	0.96	1.02	0.98	1.06	1.02	1.06	0.98	1.03	0.98	162.82	6.49
O88745	Scrapie-responsive protein 1 OS=Mus musculus GN=Scrg1 PE=2 SV=1 - [SCRG1_MOUSE]	31.63	1	3	3	8	1.01	0.76	0.69	0.99	0.91	1.10	1.10	1.06	1.07	0.83	0.83	0.99	1.01	11.17	7.91
Q62417	Sorbin and SH3 domain-containing protein 1 OS=Mus musculus GN=Sorbs1 PE=1 SV=2 - [SRBS1_MOUSE]	26.82	1	5	31	103	0.96	0.81	0.80	1.06	1.11	1.00	1.11	1.06	1.08	0.98	0.98	0.97	1.02	142.98	8.25
F6RDS0	E3 ubiquitin-protein ligase UBR3 (Fragment) OS=Mus musculus GN=Ubr3 PE=4 SV=1 - [F6RDS0_MOUSE]	2.32	4	1	1	2	0.90	1.11	1.23	1.06	1.17	1.07	1.17	1.06	1.17	1.10	1.22	1.18	1.31	56.25	5.34
Q8CIP5	Protein dispatched homolog 2 OS=Mus musculus GN=Disp2 PE=2 SV=1 - [DISP2_MOUSE]	2.53	1	2	2	6	1.18	1.42	1.32	1.14	1.05	1.17	0.76	1.06	0.80	1.20	0.86	1.15	0.94	147.85	6.76

Q99JP0	Mitogen-activated protein kinase kinase kinase kinase 3 OS=Mus musculus GN=Map4k3 PE=1 SV=4 - [M4K3_MOUSE]	1.34	2	1	1	3	0.95	0.96	1.00	0.87	0.91	1.14	1.19	1.06	1.11	0.89	0.94	1.07	1.12	101.05	7.64
P56388-2	Isoform Short of Cocaine- and amphetamine-regulated transcript protein OS=Mus musculus GN=Cartpt - [CART_MOUSE]	37.07	2	4	4	12	1.84	1.01	0.51	0.95	0.46	1.25	0.60	1.06	0.57	1.59	0.87	1.79	0.90	12.80	8.25
Q61112	45 kDa calcium-binding protein OS=Mus musculus GN=Sdf4 PE=2 SV=1 - [CAB45_MOUSE]	48.48	2	16	16	58	0.99	0.70	0.73	0.78	0.82	1.18	1.21	1.06	1.13	0.92	0.95	0.81	0.81	42.04	4.96
P62983	Ubiquitin-40S ribosomal protein S27a OS=Mus musculus GN=Rps27a PE=1 SV=2 - [RS27A_MOUSE]	62.18	12	15	15	186	1.24	0.90	0.72	0.97	0.80	1.11	0.90	1.06	0.85	1.00	0.79	1.04	0.83	17.94	9.64
Q8K221	Arfaptin-2 OS=Mus musculus GN=Arfip2 PE=2 SV=2 - [ARFP2_MOUSE]	10.85	2	3	3	6	1.21	1.21	1.04	1.06	0.91	1.21	1.02	1.06	0.90	1.23	1.04	1.10	0.98	37.75	5.87
Q6URW6	Isoform 2 of Myosin-14 OS=Mus musculus GN=Myh14 - [MYH14_MOUSE]	22.24	5	27	36	86	0.98	1.09	1.07	1.03	1.10	0.99	0.97	1.06	1.07	1.07	1.10	1.10	1.14	227.62	5.55
Q8BGW1	Alpha-ketoglutarate-dependent dioxygenase FTO OS=Mus musculus GN=Fto PE=1 SV=1 - [FTO_MOUSE]	18.33	5	6	6	8	1.03	1.07	0.94	0.99	0.94	0.99	0.91	1.06	0.87	0.89	0.95	1.04	0.97	57.97	5.12
Q9WTL7	Acyl-protein thioesterase 2 OS=Mus musculus GN=Lypla2 PE=1 SV=1 - [LYPA2_MOUSE]	47.62	1	6	7	21	0.93	0.84	0.88	1.01	1.06	1.10	1.14	1.06	1.19	1.08	1.24	0.95	1.19	24.78	7.23
Q14AI6	RNA pseudouridylate synthase domain-containing protein 3 OS=Mus musculus GN=Rpusd3 PE=2 SV=1 - [RUSD3_MOUSE]	4.36	5	2	2	3	0.94	1.11	1.19	1.01	1.08	1.04	1.11	1.06	1.13	0.99	1.06	1.12	1.20	38.05	9.52
P68372	Tubulin beta-4B chain OS=Mus musculus GN=Tubb4b PE=1 SV=1 - [TBB4B_MOUSE]	75.06	3	1	30	2072	0.99	0.86	0.90	1.01	1.07	1.08	1.10	1.06	1.11	1.08	1.10	1.01	1.05	49.80	4.89
Q9QYF9	Protein NDRG3 OS=Mus musculus GN=Ndrg3 PE=1 SV=1 - [NDRG3_MOUSE]	37.07	3	8	9	51	0.97	1.09	1.11	1.03	1.07	1.05	1.07	1.06	1.04	1.04	1.11	1.09	1.03	41.53	5.25
F8WH20	Oxysterol-binding protein OS=Mus musculus GN=Osbpl3 PE=2 SV=1 - [F8WH20_MOUSE]	8.67	4	5	5	9	0.98	0.87	0.89	0.95	0.97	1.05	1.01	1.06	1.13	0.99	1.05	0.93	0.91	92.96	6.54

Q6P542	ATP-binding cassette sub-family F member 1 OS=Mus musculus GN=Abcf1 PE=1 SV=1 - [ABCF1_MOUSE]	16.49	1	9	9	18	1.00	0.89	1.13	1.12	1.10	1.00	1.13	1.06	1.18	1.02	1.03	0.95	0.97	94.89	6.51
Q91WK5	Glycine cleavage system H protein, mitochondrial OS=Mus musculus GN=Gcsh PE=1 SV=2 - [GCSH_MOUSE]	17.65	1	2	2	29	1.10	0.67	0.66	0.95	0.85	1.11	0.99	1.06	0.97	0.98	0.86	0.94	0.82	18.63	4.75
Q8CAK3	UPF0515 protein C19orf66 homolog OS=Mus musculus PE=2 SV=1 - [CS066_MOUSE]	32.76	2	8	8	27	1.05	0.86	0.86	0.89	0.89	1.09	0.98	1.06	1.01	0.99	0.97	0.94	0.91	32.99	7.28
Q6PHZ8	Kv channel-interacting protein 4 OS=Mus musculus GN=Kcnip4 PE=1 SV=1 - [KCIP4_MOUSE]	39.20	11	6	6	27	0.96	0.84	0.79	0.88	0.94	1.04	1.00	1.06	1.06	0.92	0.86	0.87	0.88	28.74	5.21
D3YZR8	Bcl2 antagonist of cell death OS=Mus musculus GN=Bad PE=2 SV=1 - [D3YZR8_MOUSE]	50.00	5	5	5	14	1.00	1.01	1.18	0.89	0.89	1.15	1.19	1.06	1.06	0.99	1.08	0.95	0.95	19.33	6.11
A2AIH8	Pirin (Fragment) OS=Mus musculus GN=Pir PE=2 SV=1 - [A2AIH8_MOUSE]	7.51	2	2	2	2	0.87	0.73	0.84	0.83	0.96	1.13	1.30	1.06	1.22	1.06	1.22	0.98	1.13	27.93	6.92
Q8CC31	Mothers against decapentaplegic homolog 1 OS=Mus musculus GN=Smad1 PE=2 SV=1 - [Q8CC31_MOUSE]	12.77	4	2	2	4	1.04	1.00	0.96	0.97	0.93	0.88	0.84	1.06	1.02	1.08	1.04	1.14	1.09	26.43	8.29
P56391	Cytochrome c oxidase subunit 6B1 OS=Mus musculus GN=Cox6b1 PE=1 SV=2 - [CX6B1_MOUSE]	75.58	1	8	8	350	1.00	0.97	0.96	0.97	0.97	0.98	0.99	1.06	1.06	0.95	0.96	0.99	1.02	10.06	8.72
A3KG57	Serine/arginine-rich-splicing factor 12 (Fragment) OS=Mus musculus GN=Srsf12 PE=2 SV=1 - [A3KG57_MOUSE]	20.69	2	3	5	14	1.12	1.21	1.01	0.93	0.88	1.13	1.00	1.06	1.09	1.18	1.03	1.13	1.01	30.35	11.58
Q8R429	Sarcoplasmic/endoplasmic reticulum calcium ATPase 1 OS=Mus musculus GN=Atp2a1 PE=2 SV=1 - [AT2A1_MOUSE]	8.85	1	1	8	22	0.70	1.36	1.92	0.70	0.99	3.62	5.12	1.06	1.51	1.15	1.64	0.71	1.01	109.35	5.22
Q99KU0	Vacuole membrane protein 1 OS=Mus musculus GN=Vmp1 PE=2 SV=2 - [VMP1_MOUSE]	9.11	2	2	2	3	1.14	1.30	1.14	1.22	1.07	1.00	0.87	1.06	0.93	1.07	0.94	1.08	0.95	45.93	6.95
D3Z111	Acyl-protein thioesterase 1 OS=Mus musculus GN=Lypla1 PE=2 SV=1 - [D3Z111_MOUSE]	13.27	7	2	2	4	0.86	0.90	1.04	1.05	1.22	1.00	1.15	1.07	1.23	1.01	1.17	0.94	1.09	21.14	6.18

Q8CFP6	DnaJ homolog subfamily C member 27 OS=Mus musculus GN=Dnajc27 PE=2 SV=1 - [DJC27_MOUSE]	8.42	1	2	2	2	1.23	1.13	0.92	1.31	1.07	1.29	1.04	1.07	0.86	0.95	0.77	1.01	0.83	30.81	8.47
Q6P0X2-2	Isoform 2 of Zinc finger protein 511 OS=Mus musculus GN=Znf511 - [ZN511_MOUSE]	5.20	1	1	1	2	1.05	0.93	0.89	0.99	0.95	1.08	1.03	1.07	1.01	0.99	0.95	1.15	1.10	28.27	7.15
Q9CR16	Peptidyl-prolyl cis-trans isomerase D OS=Mus musculus GN=Ppid PE=1 SV=3 - [PPID_MOUSE]	47.03	1	17	18	61	0.98	0.82	0.84	0.94	0.93	1.05	1.08	1.07	1.06	1.05	0.99	0.97	0.99	40.72	7.43
B0QZX9	Dephospho-CoA kinase domain-containing protein (Fragment) OS=Mus musculus GN=Dcakd PE=2 SV=1 - [B0QZX9_MOUSE]	11.43	2	1	1	1	1.37	1.23	0.90	1.24	0.91	1.22	0.89	1.07	0.78	0.98	0.72	1.04	0.76	11.85	8.21
Q8CI75	DIS3-like exonuclease 2 OS=Mus musculus GN=Dis3l2 PE=1 SV=1 - [DI3L2_MOUSE]	7.82	2	5	5	9	0.91	1.04	1.14	1.00	1.12	0.91	1.12	1.07	1.15	0.99	1.14	0.95	1.00	97.71	5.90
Q505B7-2	Isoform 2 of Protein archease OS=Mus musculus GN=Zbtb8os - [ARCH_MOUSE]	4.79	3	1	1	3	0.88	0.76	0.86	0.79	0.90	1.10	1.24	1.07	1.21	0.94	1.08	0.97	1.11	17.20	4.68
H7BWY2	AP-3 complex subunit mu-1 OS=Mus musculus GN=Ap3m1 PE=2 SV=1 - [H7BWY2_MOUSE]	7.69	2	1	2	2	0.64	0.75	1.17	1.22	1.90	0.73	1.14	1.07	1.66	0.49	0.77	0.95	1.49	41.00	8.25
P97379	Ras GTPase-activating protein-binding protein 2 OS=Mus musculus GN=G3bp2 PE=1 SV=2 - [G3BP2_MOUSE]	33.61	2	12	12	38	1.03	0.83	0.83	0.87	0.89	1.12	1.07	1.07	1.06	0.96	0.96	0.98	0.99	54.05	5.62
P59326	YTH domain family protein 1 OS=Mus musculus GN=Ythdf1 PE=2 SV=1 - [YTHD1_MOUSE]	10.20	2	4	5	17	0.88	0.92	1.10	0.96	1.06	1.03	1.10	1.07	1.18	0.92	1.02	0.91	1.10	60.84	8.95
Q5XJY5	Coatomer subunit delta OS=Mus musculus GN=Arcn1 PE=2 SV=2 - [COPD_MOUSE]	14.87	1	6	6	12	1.00	0.90	0.94	1.03	1.05	0.97	0.97	1.07	1.03	1.06	1.08	1.09	1.02	57.19	6.21
Q6DID3	Protein SCAF8 OS=Mus musculus GN=Scaf8 PE=1 SV=1 - [SCAF8_MOUSE]	5.91	1	4	4	6	0.90	0.75	0.83	1.02	1.01	1.08	1.12	1.07	1.16	0.94	0.98	0.91	0.95	139.49	8.24
P62774	Myotrophin OS=Mus musculus GN=Mtpn PE=1 SV=2 - [MTPN_MOUSE]	83.05	1	9	9	73	1.02	0.77	0.72	0.89	0.92	1.12	1.11	1.07	0.99	0.94	0.93	0.86	0.88	12.85	5.52
G3X9K4	SAPS domain family, member 2, isoform CRA_a OS=Mus musculus GN=Ppp6r2 PE=4 SV=1 - [G3X9K4_MOUSE]	7.91	2	5	5	17	0.92	1.14	1.10	0.96	1.04	0.94	1.03	1.07	1.05	0.97	1.00	0.97	0.98	100.40	4.82

Q8BI84	Melanoma inhibitory activity protein 3 OS=Mus musculus GN=Mia3 PE=1 SV=2 - [MIA3_MOUSE]	17.15	5	23	23	43	0.99	1.02	1.00	1.02	1.00	1.01	1.03	1.07	1.08	1.04	1.04	0.98	0.97	213.54	4.75
A2AR60	Protein CASC4 (Fragment) OS=Mus musculus GN=Casc4 PE=2 SV=1 - [A2AR60_MOUSE]	14.61	5	3	3	7	1.03	0.87	0.84	0.98	0.95	1.11	1.06	1.07	1.01	1.01	1.01	0.95	0.98	30.54	6.24
P84086	Complexin-2 OS=Mus musculus GN=Cplx2 PE=1 SV=1 - [CPLX2_MOUSE]	66.42	1	5	8	175	0.94	0.71	0.81	0.89	0.96	1.08	1.11	1.07	1.15	1.04	1.11	0.87	1.00	15.38	5.08
F6VJ09	Breast carcinoma-amplified sequence 3 homolog (Fragment) OS=Mus musculus GN=Bcas3 PE=4 SV=1 - [F6VJ09_MOUSE]	23.26	1	1	2	5	1.01	0.79	0.78	0.84	0.84	1.12	1.11	1.07	1.05	0.92	0.92	0.84	0.83	9.40	9.57
Q9ESE1-3	Isoform 3 of Lipopolysaccharide-responsive and beige-like anchor protein OS=Mus musculus GN=Lrba - [LRBA_MOUSE]	2.64	4	1	6	11	1.33	2.16	1.63	1.11	0.83	0.89	0.66	1.07	0.80	1.18	0.89	1.34	1.01	286.27	5.66
Q61879	Myosin-10 OS=Mus musculus GN=Myh10 PE=1 SV=2 - [MYH10_MOUSE]	48.18	7	73	91	468	0.94	0.93	1.00	1.07	1.13	1.02	1.08	1.07	1.09	1.03	1.11	1.03	1.11	228.85	5.54
Q923D4	Splicing factor 3B subunit 5 OS=Mus musculus GN=Sf3b5 PE=2 SV=1 - [SF3B5_MOUSE]	55.81	1	4	4	10	0.86	0.91	1.03	0.91	1.03	1.10	1.13	1.07	1.15	0.94	1.04	0.89	1.04	10.11	6.35
Q91YD9	Neural Wiskott-Aldrich syndrome protein OS=Mus musculus GN=Wasl PE=1 SV=1 - [WASL_MOUSE]	42.71	2	15	15	81	1.04	0.95	0.94	1.00	0.95	1.00	0.99	1.07	1.03	0.98	0.97	0.92	0.89	54.24	7.93
Q8CH77-2	Isoform 2 of Neuron navigator 1 OS=Mus musculus GN=Nav1 - [NAV1_MOUSE]	11.68	4	15	15	35	1.08	0.88	0.80	1.02	0.93	1.10	1.08	1.07	1.01	1.13	1.08	1.00	0.93	195.78	8.13
Q8VCQ3	Nuclear receptor-binding factor 2 OS=Mus musculus GN=Nrbf2 PE=1 SV=1 - [NRBF2_MOUSE]	33.45	1	6	6	18	1.05	0.88	0.88	0.90	0.89	1.13	1.14	1.07	1.04	0.94	0.97	0.97	0.89	32.48	5.76
Q8K1Z0	Ubiquinone biosynthesis protein COQ9, mitochondrial OS=Mus musculus GN=Coq9 PE=1 SV=1 - [COQ9_MOUSE]	22.04	2	5	5	53	0.93	1.02	1.02	0.86	0.94	1.06	1.13	1.07	1.16	0.99	1.02	0.94	1.07	35.06	5.92
Q8BMP4	G-protein coupled estrogen receptor 1 OS=Mus musculus GN=Gper PE=2 SV=2 - [GPER_MOUSE]	7.20	1	1	1	1	1.09	0.85	0.78	0.99	0.91	1.07	0.97	1.07	0.97	1.06	0.97	1.09	1.00	42.45	7.65

Q04447	Creatine kinase B-type OS=Mus musculus GN=Ckb PE=1 SV=1 - [KCRB_MOUSE]	86.09	1	25	26	1392	0.95	1.01	1.06	1.00	1.04	1.06	1.10	1.07	1.12	1.02	1.07	1.02	1.09	42.69	5.67
Q8R395	COMM domain-containing protein 5 OS=Mus musculus GN=Commd5 PE=2 SV=1 - [COMD5_MOUSE]	7.59	1	1	1	2	1.35	1.82	1.34	1.39	1.03	1.23	0.91	1.07	0.79	1.24	0.92	2.06	1.53	24.48	8.06
E9Q740	Signal recognition particle subunit SRP72 OS=Mus musculus GN=Srp72 PE=2 SV=1 - [E9Q740_MOUSE]	13.11	2	4	5	9	0.77	1.05	1.17	1.20	1.21	0.82	1.00	1.07	1.39	1.06	1.12	0.98	1.13	67.89	9.38
Q8BY87-2	Isoform 2 of Ubiquitin carboxyl-terminal hydrolase 47 OS=Mus musculus GN=Usp47 - [UBP47_MOUSE]	8.41	2	9	10	26	1.06	1.19	1.18	1.09	1.00	1.06	0.97	1.07	1.01	1.06	0.99	1.11	1.01	154.74	5.08
Q8VDM6	Heterogeneous nuclear ribonucleoprotein U-like protein 1 OS=Mus musculus GN=Hnrnpul1 PE=1 SV=1 - [HNRL1_MOUSE]	13.74	3	7	7	30	0.99	1.02	0.83	1.02	0.93	1.02	0.91	1.07	1.05	1.04	1.05	1.11	1.01	95.94	6.58
H3BJ08	Protein 1700037C18Rik (Fragment) OS=Mus musculus GN=1700037C18Rik PE=2 SV=1 - [H3BJ08_MOUSE]	11.11	3	1	1	1	1.19	0.96	0.80	1.18	1.00	1.08	0.91	1.07	0.90	1.03	0.87	1.10	0.93	15.18	8.37
E0CXF0	E2F-associated phosphoprotein OS=Mus musculus GN=Eapp PE=2 SV=1 - [E0CXF0_MOUSE]	12.18	3	1	1	2	1.11	0.87	0.78	0.98	0.88	1.02	0.92	1.07	0.96	0.90	0.82	0.75	0.68	17.36	4.48
Q80U78-2	Isoform 2 of Pumilio homolog 1 OS=Mus musculus GN=Pum1 - [PUM1_MOUSE]	9.02	8	7	8	15	0.96	1.04	1.09	1.05	1.04	1.09	1.14	1.07	1.17	1.03	1.04	1.11	1.16	126.26	6.86
Q9D7V9	N-acylethanolamine-hydrolyzing acid amidase OS=Mus musculus GN=Naaa PE=2 SV=2 - [NAAA_MOUSE]	4.97	2	2	2	2	1.20	1.14	0.94	0.88	0.73	1.09	0.91	1.07	0.89	1.04	0.87	1.04	0.86	40.05	6.46
Q9WTP6-	Isoform 2 of Adenylate kinase 2, mitochondrial OS=Mus musculus GN=Ak2 - [KAD2_MOUSE]	44.83	3	8	8	19	0.90	0.85	0.91	1.05	1.05	1.02	1.09	1.07	1.12	1.08	1.14	1.08	1.08	25.59	7.42
A2AUD5	Tumor protein D54 OS=Mus musculus GN=Tpd52l2 PE=2 SV=1 - [A2AUD5_MOUSE]	66.38	3	4	14	82	1.04	0.86	0.84	0.86	0.87	1.10	1.08	1.07	0.98	1.13	1.08	1.03	0.99	25.05	6.55
Q99LH4	Zinc finger protein 672 OS=Mus musculus GN=Znf672 PE=2 SV=1 - [ZN672_MOUSE]	6.84	1	1	1	2	0.73	0.79	1.08	0.66	0.90	1.28	1.73	1.07	1.45	1.18	1.61	0.58	0.79	52.12	9.36

F8WJ31	Zinc finger and SCAN domain-containing protein 26 OS=Mus musculus GN=Zscan26 PE=2 SV=1 - [F8WJ31_MOUSE]	10.00	3	3	3	7	1.02	0.89	0.85	0.97	0.93	1.16	1.19	1.07	1.09	1.08	1.06	1.08	1.06	38.79	8.78
Q00623	Apolipoprotein A-I OS=Mus musculus GN=Apoa1 PE=1 SV=2 - [APOA1_MOUSE]	32.20	1	10	10	22	0.79	1.01	1.39	0.59	0.72	0.94	1.21	1.07	1.35	1.58	1.93	0.84	1.06	30.60	5.73
Q3UH60	Disco-interacting protein 2 homolog B OS=Mus musculus GN=Dip2b PE=1 SV=1 - [DIP2B_MOUSE]	14.49	3	5	19	43	0.93	1.17	1.13	1.01	1.12	1.12	1.31	1.07	1.13	1.05	1.26	0.96	1.00	171.02	8.09
Q8C854-3	Isoform 2 of Myelin expression factor 2 OS=Mus musculus GN=Myef2 - [MYEF2_MOUSE]	25.96	8	13	13	38	0.92	0.92	1.05	1.01	1.08	1.06	1.17	1.07	1.19	1.05	1.13	0.98	1.07	61.69	8.87
Q9CW03	Structural maintenance of chromosomes protein 3 OS=Mus musculus GN=Smc3 PE=1 SV=2 - [SMC3_MOUSE]	9.78	1	10	10	27	1.00	1.17	1.19	1.01	1.05	1.01	1.02	1.07	1.26	1.16	1.15	1.28	1.20	141.47	7.18
F7CD65	Epsin-2 (Fragment) OS=Mus musculus GN=Epn2 PE=4 SV=1 - [F7CD65_MOUSE]	45.95	1	1	4	38	0.96	1.16	1.20	0.87	0.90	0.95	0.98	1.07	1.10	0.94	0.98	1.13	1.17	16.35	7.74
Q9EQ80	NIF3-like protein 1 OS=Mus musculus GN=Nif3l1 PE=1 SV=4 - [NIF3L_MOUSE]	18.62	2	5	5	11	1.01	1.24	1.29	1.06	1.06	0.90	0.92	1.07	1.05	1.08	1.11	1.12	1.16	41.72	6.76
E9Q0A3	Protein Arhgef11 (Fragment) OS=Mus musculus GN=Arhgef11 PE=2 SV=1 - [E9Q0A3_MOUSE]	5.89	3	5	5	21	1.13	1.18	1.00	1.17	1.03	1.00	0.94	1.07	1.08	1.03	0.97	1.13	1.08	163.51	5.78
D3Z6S1	Transmembrane protein 214 OS=Mus musculus GN=Tmem214 PE=2 SV=1 - [D3Z6S1_MOUSE]	2.65	2	1	2	2	1.04	0.85	0.82	1.12	1.08	1.01	0.97	1.07	1.03	1.13	1.09	1.05	1.02	71.26	9.41
P56212-2	Isoform ARPP-16 of cAMP-regulated phosphoprotein 19 OS=Mus musculus GN=Arpp19 - [ARP19_MOUSE]	62.50	5	5	6	112	0.92	0.79	0.85	0.93	0.98	1.07	1.10	1.07	1.09	0.98	1.08	0.92	0.98	10.60	9.70
P70271	PDZ and LIM domain protein 4 OS=Mus musculus GN=Pdlim4 PE=1 SV=3 - [PDLI4_MOUSE]	30.30	3	7	7	18	1.11	0.99	0.97	1.04	0.92	1.09	1.01	1.07	0.98	1.02	1.00	1.11	1.05	35.53	8.05
Q8R146-2	Isoform 2 of Acylamino-acid-releasing enzyme OS=Mus musculus GN=Apeh - [APEH_MOUSE]	8.37	2	4	4	8	1.21	1.24	1.05	1.21	0.97	1.10	0.90	1.07	0.88	1.13	0.96	1.27	1.03	79.91	5.54

P62077	Mitochondrial import inner membrane translocase subunit Tim8 B OS=Mus musculus GN=Timm8b PE=3 SV=1 - [TIM8B_MOUSE]	56.63	1	4	4	11	0.98	0.95	0.93	0.95	1.00	1.05	1.09	1.07	1.17	1.02	1.05	1.03	1.01	9.28	5.12
Q8CG72	Poly(ADP-ribose) glycohydrolase ARH3 OS=Mus musculus GN=Adprhl2 PE=1 SV=1 - [ARHL2_MOUSE]	12.97	2	4	4	9	1.05	1.09	1.24	1.06	1.04	0.99	0.94	1.07	1.07	1.07	0.94	1.16	1.16	39.39	4.96
D3YXN7	Tetraspanin-9 (Fragment) OS=Mus musculus GN=Tspan9 PE=2 SV=1 - [D3YXN7_MOUSE]	8.72	2	1	1	7	0.92	1.03	1.12	1.15	1.25	1.09	1.19	1.07	1.16	1.03	1.12	1.08	1.17	19.09	4.93
Q0KK55-3	Isoform 3 of Protein very KIND OS=Mus musculus GN=Kndc1 - [VKIND_MOUSE]	1.62	3	1	1	2	0.97	1.43	1.48	1.10	1.14	0.91	0.93	1.07	1.10	0.93	0.97	1.01	1.05	70.10	9.25
Q8K352	SAM and SH3 domain-containing protein 3 OS=Mus musculus GN=Sash3 PE=2 SV=2 - [SASH3_MOUSE]	5.00	1	2	2	3	0.98	1.14	1.15	0.83	0.84	1.03	1.04	1.07	1.08	0.99	1.01	1.11	1.13	41.58	5.22
Q3UHG5	Tetraspanin-7 OS=Mus musculus GN=Tspan7 PE=2 SV=1 - [Q3UHG5_MOUSE]	9.57	2	2	2	59	1.07	1.44	1.32	1.06	1.09	0.93	0.87	1.07	1.20	1.12	1.13	1.11	1.10	25.40	7.58
E9QKH8	Catenin delta-2 OS=Mus musculus GN=Ctnnd2 PE=2 SV=1 - [E9QKH8_MOUSE]	35.55	3	31	33	133	1.01	1.08	1.07	1.01	1.00	1.03	1.03	1.07	1.07	1.07	1.05	1.06	1.04	134.86	7.65
F6ZFU0	Elongation factor 1-delta (Fragment) OS=Mus musculus GN=Eef1d PE=2 SV=1 - [F6ZFU0_MOUSE]	49.64	5	2	23	112	1.44	0.97	0.66	0.92	0.63	1.37	0.97	1.07	0.90	1.12	0.70	1.22	0.80	61.35	7.39
P21550	Beta-enolase OS=Mus musculus GN=Eno3 PE=1 SV=3 - [ENOB_MOUSE]	26.27	5	1	10	519	0.87	1.08	1.23	0.89	1.02	1.69	1.92	1.07	1.22	1.14	1.30	0.99	1.13	47.00	7.18
Q3TYD6	Serine/threonine-protein kinase LMTK2 OS=Mus musculus GN=Lmtk2 PE=1 SV=3 - [LMTK2_MOUSE]	15.98	1	14	14	31	0.96	0.99	1.03	1.00	1.02	1.12	1.11	1.07	1.10	0.96	0.99	0.91	0.92	160.41	4.51
A3KG01	Eph receptor B2 OS=Mus musculus GN=Ephb2 PE=2 SV=2 - [A3KG01_MOUSE]	7.40	5	4	5	12	1.03	1.45	1.53	1.20	1.26	1.02	0.98	1.07	1.04	0.95	0.92	0.98	0.95	109.83	5.71
P16014	Secretogranin-1 OS=Mus musculus GN=Chgb PE=1 SV=2 - [SCG1_MOUSE]	36.19	1	22	22	144	1.19	0.90	0.73	0.91	0.78	1.13	0.94	1.07	0.90	1.03	0.86	0.99	0.81	77.92	5.07

O35495-3	Isoform 3 of Cyclin-dependent kinase 14 OS=Mus musculus GN=Cdk14 - [CDK14_MOUSE]	22.62	55	6	7	19	1.05	1.03	0.98	1.10	0.98	1.03	0.96	1.07	1.06	1.05	1.00	0.94	0.92	41.35	8.88
Q9D219	B-cell CLL/lymphoma 9 protein OS=Mus musculus GN=Bcl9 PE=1 SV=3 - [BCL9_MOUSE]	7.16	1	6	6	12	1.10	0.90	0.87	0.83	0.82	1.12	1.07	1.07	1.01	1.06	1.05	0.97	1.00	148.87	8.91
Q9Z2D6	Methyl-CpG-binding protein 2 OS=Mus musculus GN=Mecp2 PE=1 SV=1 - [MECP2_MOUSE]	53.10	4	20	20	108	1.05	0.94	0.91	1.02	0.97	1.08	1.06	1.07	1.03	1.08	1.07	1.05	0.98	52.28	9.96
P63044	Vesicle-associated membrane protein 2 OS=Mus musculus GN=Vamp2 PE=1 SV=2 - [VAMP2_MOUSE]	43.10	2	2	6	133	1.03	1.20	1.16	1.00	0.98	0.98	0.95	1.07	1.00	1.06	0.98	1.13	1.09	12.68	8.13
F6ZDT4	Ribonuclease H2 subunit C (Fragment) OS=Mus musculus GN=Rnaseh2c PE=4 SV=1 - [F6ZDT4_MOUSE]	22.73	2	2	2	5	1.43	1.02	0.69	0.95	0.73	1.12	0.78	1.07	0.74	0.93	0.65	1.13	0.79	9.33	4.81
P51830	Adenylate cyclase type 9 OS=Mus musculus GN=Adcy9 PE=1 SV=1 - [ADCY9_MOUSE]	20.62	2	19	20	36	0.97	1.16	1.27	1.09	1.15	0.94	0.99	1.07	1.08	1.00	1.04	0.90	0.99	150.86	7.21
Q9WVA4	Transgelin-2 OS=Mus musculus GN=Tagln2 PE=1 SV=4 - [TAGL2_MOUSE]	62.81	1	9	10	40	0.94	0.97	0.98	0.85	0.94	1.07	1.11	1.07	1.19	1.03	1.14	1.03	1.19	22.38	8.24
Q05186	Reticulocalbin-1 OS=Mus musculus GN=Rcn1 PE=1 SV=1 - [RCN1_MOUSE]	42.15	1	11	11	43	1.25	0.89	0.73	0.97	0.77	1.09	0.92	1.07	0.93	1.16	0.95	1.24	1.04	38.09	4.84
Q8K2C7	Protein OS-9 OS=Mus musculus GN=Os9 PE=1 SV=2 - [OS9_MOUSE]	16.67	5	6	7	20	0.92	0.91	1.04	0.85	0.90	1.01	1.09	1.07	1.01	0.90	0.92	0.85	0.86	76.06	4.84
Q64433	10 kDa heat shock protein, mitochondrial OS=Mus musculus GN=Hspe1 PE=1 SV=2 - [CH10_MOUSE]	71.57	1	8	8	207	1.04	0.76	0.73	0.92	0.87	1.13	1.07	1.07	1.02	1.01	0.97	0.92	0.87	10.96	8.35
Q9JL62	Glycolipid transfer protein OS=Mus musculus GN=Gltp PE=2 SV=3 - [GLTP_MOUSE]	26.79	3	6	6	10	1.01	0.95	0.80	1.13	1.08	1.11	1.04	1.07	1.01	1.03	1.02	1.04	1.04	23.67	7.39
O88834	SH2 domain-containing adapter protein D OS=Mus musculus GN=Shd PE=1 SV=1 - [SHD_MOUSE]	25.66	2	6	6	19	1.04	0.89	0.88	1.03	0.88	1.07	1.05	1.07	0.99	0.86	0.78	0.92	0.89	38.46	5.05
G3X9J0	MCG122846 OS=Mus musculus GN=Sipa1l3 PE=4 SV=1 - [G3X9J0_MOUSE]	7.83	3	10	11	17	1.02	0.96	0.92	1.02	0.96	1.17	1.14	1.07	1.02	1.05	1.06	1.13	1.17	194.94	8.32

Q9QXT8	Calsenilin OS=Mus musculus GN=Kcnip3 PE=1 SV=2 - [CSEN_MOUSE]	20.70	3	1	4	9	0.97	1.10	1.13	0.90	0.92	1.08	1.11	1.07	1.10	1.05	1.08	1.08	1.11	29.44	5.69
Q9CXV1	Succinate dehydrogenase [ubiquinone] cytochrome b small subunit, mitochondrial OS=Mus musculus GN=Sdhd PE=2 SV=2 - [DHSD_MOUSE]	21.38	1	1	1	2	1.36	0.99	0.73	1.59	1.17	0.98	0.72	1.07	0.79	1.13	0.84	0.97	0.71	17.00	9.10
Q3ULB1	Testin OS=Mus musculus GN=Tes PE=2 SV=1 - [Q3ULB1_MOUSE]	6.10	4	2	2	3	1.08	1.50	1.39	0.94	0.87	1.04	0.95	1.07	0.99	1.00	0.93	1.10	1.02	46.56	7.87
Q7TSK2-2	Isoform 2 of Seizure protein 6 OS=Mus musculus GN=Sez6 - [SEZ6_MOUSE]	6.24	5	4	4	18	1.11	1.00	0.98	1.01	0.94	0.98	0.96	1.07	0.98	1.03	0.97	1.12	1.01	106.00	5.36
Q6ZPS6	Ankyrin repeat and IBR domain-containing protein 1 OS=Mus musculus GN=Ankib1 PE=1 SV=2 - [AKIB1_MOUSE]	7.83	1	5	5	9	1.07	1.17	1.27	1.07	1.01	1.15	1.09	1.07	0.91	1.21	1.10	0.99	0.97	121.80	5.16
Q61908	Cx9C motif-containing protein 4 OS=Mus musculus GN=Cmc4 PE=2 SV=1 - [CMC4_MOUSE]	60.29	1	5	5	13	1.04	0.97	0.97	1.02	1.01	1.01	0.90	1.07	1.07	1.04	1.02	0.99	0.94	7.74	8.38
Q64191	N(4)-(beta-N-acetylglucosaminyl)-L-asparaginase OS=Mus musculus GN=Aga PE=2 SV=1 - [ASPG_MOUSE]	4.34	1	1	1	1	1.09	1.32	1.21	0.90	0.83	1.36	1.25	1.07	0.98	1.25	1.15	1.69	1.55	37.00	6.44
Q08943	FACT complex subunit SSRP1 OS=Mus musculus GN=Ssrp1 PE=1 SV=2 - [SSRP1_MOUSE]	15.96	3	8	8	47	1.00	1.06	1.17	1.06	1.03	0.99	0.97	1.07	1.08	0.97	0.99	0.94	0.99	80.81	6.76
P97376	Protein FRG1 OS=Mus musculus GN=Frg1 PE=1 SV=2 - [FRG1_MOUSE]	5.04	1	1	1	1	0.96	1.05	1.08	0.93	0.96	1.18	1.22	1.07	1.11	1.04	1.08	1.16	1.21	29.11	8.92
A2A791-2	Isoform 2 of Zinc finger MYM-type protein 4 OS=Mus musculus GN=Zmym4 [ZMYM4_MOUSE]	0.62	2	1	1	1	1.11	0.86	0.78	0.94	0.85	1.20	1.08	1.07	0.96	1.06	0.95	1.04	0.94	162.74	6.54
Q9CY58-3	Isoform 3 of Plasminogen activator inhibitor 1 RNA-binding protein OS=Mus musculus GN=Serbp1 - [PAIRB_MOUSE]	48.19	3	1	16	95	1.53	0.69	0.45	0.85	0.56	0.88	0.58	1.07	0.70	1.09	0.71	1.15	0.76	42.21	8.44
G5E8T2	E3 ubiquitin-protein ligase TTC3 OS=Mus musculus GN=Ttc3 PE=4 SV=1 - [G5E8T2_MOUSE]	4.75	8	7	7	12	1.11	0.91	0.87	1.00	0.89	1.14	1.00	1.07	1.02	1.14	1.01	1.04	0.94	223.77	6.25

B1B0F0	U2 small nuclear ribonucleoprotein auxiliary factor 35 kDa subunit-related protein 2 (Fragment) OS=Mus musculus GN=Zrsr2 PE=2 SV=1 - [B1B0F0_MOUSE]	7.08	5	1	1	1	1.07	0.88	0.82	1.14	1.07	0.96	0.89	1.07	0.99	0.88	0.82	0.72	0.68	13.71	9.45
P48428	Tubulin-specific chaperone A OS=Mus musculus GN=Tbca PE=2 SV=3 - [TBCA_MOUSE]	49.07	1	8	8	88	0.94	0.75	0.84	0.92	0.99	1.08	1.09	1.07	1.17	1.02	1.14	0.97	1.02	12.75	5.27
Q9ET38-2	Isoform 2 of Claudin-19 OS=Mus musculus GN=Cldn19 - [CLD19_MOUSE]	8.06	2	1	1	1	1.00	1.00	0.99	0.86	0.86	1.10	1.10	1.07	1.07	1.05	1.05	1.07	1.08	22.15	7.59
Q4KL52	Zfyve19 protein OS=Mus musculus GN=Zfyve19 PE=2 SV=1 - [Q4KL52_MOUSE]	32.42	2	7	7	37	1.08	0.90	0.89	0.90	0.86	1.14	1.08	1.07	1.01	1.06	1.05	0.99	0.98	36.30	7.81
Q04888	Transcription factor SOX-10 OS=Mus musculus GN=Sox10 PE=2 SV=2 - [SOX10_MOUSE]	1.93	1	1	1	2	1.24	1.36	1.09	0.98	0.79	1.36	1.09	1.07	0.86	1.13	0.91	1.44	1.16	49.92	6.60
D3Z108	Transmembrane and ubiquitin-like domain-containing protein 1 (Fragment) OS=Mus musculus GN=Tmub1 PE=2 SV=1 - [D3Z108_MOUSE]	10.29	4	2	2	4	0.98	1.55	1.58	1.14	1.17	0.90	0.91	1.07	1.10	1.04	1.07	1.32	1.35	21.57	4.82
G3XA57	Protein Rab11fip2 OS=Mus musculus GN=Rab11fip2 PE=4 SV=1 - [G3XA57_MOUSE]	36.52	5	14	14	36	0.97	0.90	0.89	0.94	1.00	1.05	1.12	1.07	1.13	1.00	1.06	1.03	1.07	58.17	9.45
Q5SW75	Protein phosphatase Slingshot homolog 2 OS=Mus musculus GN=Ssh2 PE=1 SV=2 - [SSH2_MOUSE]	4.57	3	4	4	6	0.91	0.92	1.00	0.96	1.01	1.09	1.07	1.07	1.11	1.07	1.07	0.91	1.03	158.13	5.67
Q9JHU2	Palmdelphin OS=Mus musculus GN=Palmd PE=1 SV=1 - [PALMD_MOUSE]	48.82	3	19	19	70	1.07	1.03	0.97	0.97	0.90	1.15	1.05	1.07	1.05	1.03	1.00	1.00	0.94	62.66	5.66
Q8BNY6	Neuronal calcium sensor 1 OS=Mus musculus GN=Ncs1 PE=2 SV=3 - [NCS1_MOUSE]	27.89	1	5	5	12	1.13	0.73	0.61	1.06	0.76	1.10	0.83	1.07	0.75	1.04	0.68	1.05	0.83	21.86	4.83
Q923M0	Protein phosphatase 1 regulatory subunit 16A OS=Mus musculus GN=Ppp1r16a PE=1 SV=1 - [PP16A_MOUSE]	7.25	2	3	3	4	0.99	1.18	1.28	1.00	1.00	1.08	1.14	1.07	1.09	1.03	1.00	1.08	1.02	57.49	5.81
P50608	Fibromodulin OS=Mus musculus GN=Fmod PE=2 SV=1 - [FMOD_MOUSE]	2.13	1	1	1	1	0.93	1.14	1.22	0.86	0.92	0.97	1.03	1.07	1.15	1.09	1.17	0.90	0.97	43.03	6.04

E9Q411	Protein Nbas OS=Mus musculus GN=Nbas PE=2 SV=1 - [E9Q411_MOUSE]	0.47	1	1	1	1	1.03	1.20	1.16	1.15	1.12	0.90	0.87	1.07	1.04	1.06	1.03	0.93	0.90	265.61	5.95
Q9WU28	Prefoldin subunit 5 OS=Mus musculus GN=Pfdn5 PE=1 SV=1 - [PFD5_MOUSE]	60.39	6	7	7	44	1.05	0.79	0.79	0.86	0.84	1.00	0.98	1.07	1.02	0.91	0.93	0.89	0.87	17.35	6.33
Q9CQZ7	DNA-directed RNA polymerase III subunit RPC10 OS=Mus musculus GN=Polr3k PE=2 SV=1 - [RPC10_MOUSE]	21.30	1	2	2	7	1.07	1.21	1.20	0.98	1.00	1.13	1.11	1.07	1.02	1.11	1.05	1.10	1.04	12.32	7.83
Q9JJY4	Probable ATP-dependent RNA helicase DDX20 OS=Mus musculus GN=Ddx20 PE=1 SV=2 - [DDX20_MOUSE]	1.33	1	1	1	1	0.56	0.82	1.45	0.88	1.56	0.85	1.50	1.07	1.90	1.05	1.86	0.73	1.30	91.65	6.74
O88704	Potassium/sodium hyperpolarization-activated cyclic nucleotide-gated channel 1 OS=Mus musculus GN=Hcn1 PE=1 SV=1 - [HCN1_MOUSE]	6.37	3	3	4	39	1.02	1.25	1.11	1.02	0.95	1.04	0.95	1.07	1.11	1.08	1.00	0.98	1.01	102.37	8.37
Q8K0T0	Reticulon-1 OS=Mus musculus GN=Rtn1 PE=1 SV=1 - [RTN1_MOUSE]	39.36	1	18	21	278	1.06	0.95	0.95	1.02	0.97	1.12	1.01	1.07	1.01	1.10	1.06	1.07	1.06	83.52	4.58
P62313	U6 snRNA-associated Sm-like protein LSm6 OS=Mus musculus GN=Lsm6 PE=2 SV=1 - [LSM6_MOUSE]	33.75	1	3	3	10	1.04	0.88	0.87	0.91	0.83	1.11	1.06	1.07	1.01	1.06	0.96	0.97	0.86	9.12	9.58
P30875	Somatostatin receptor type 2 OS=Mus musculus GN=Sstr2 PE=1 SV=1 - [SSR2_MOUSE]	2.98	1	1	1	1	1.19	1.08	0.91	1.22	1.02	0.86	0.72	1.07	0.90	1.06	0.89	0.97	0.82	41.19	8.97
Q3TJ55	Syntaxin-2 OS=Mus musculus GN=Stx2 PE=2 SV=1 - [Q3TJ55_MOUSE]	12.50	4	2	3	16	1.08	1.23	1.13	1.03	0.95	1.03	1.00	1.07	1.02	0.99	0.91	1.12	1.17	33.18	6.21
E9QA15	Protein Cald1 OS=Mus musculus GN=Cald1 PE=2 SV=1 - [E9QA15_MOUSE]	27.86	4	3	20	94	1.11	1.19	1.11	0.91	0.84	1.00	0.88	1.07	1.00	1.15	1.05	0.91	0.86	89.22	5.50
Q9QXJ1-2	Isoform 2 of Amyloid beta A4 precursor protein-binding family B member 1 OS=Mus musculus GN=Apbb1 - [APBB1_MOUSE]	11.72	2	5	5	11	1.08	1.16	0.99	1.16	0.99	1.17	1.07	1.07	0.98	1.02	0.97	1.07	1.05	77.07	4.97
D6RDD4	GDNF family receptor alpha-1 OS=Mus musculus GN=Gfra1 PE=2 SV=1 - [D6RDD4_MOUSE]	3.82	3	2	2	3	1.13	0.90	0.79	1.02	0.90	1.17	1.03	1.07	0.94	1.30	1.15	1.25	1.11	47.19	8.02

Q8BM72	Heat shock 70 kDa protein 13 OS=Mus musculus GN=Hspa13 PE=2 SV=1 - [HSP13_MOUSE]	5.10	3	2	2	4	1.02	1.30	1.28	1.23	1.20	1.03	1.01	1.07	1.05	1.21	1.19	1.26	1.24	51.68	5.63
Q6XLQ8	Calumenin OS=Mus musculus GN=Calu PE=2 SV=1 - [Q6XLQ8_MOUSE]	69.84	5	5	17	188	1.08	0.74	0.75	0.93	0.93	1.11	1.01	1.07	0.98	0.93	1.00	0.91	0.81	37.09	4.59
Q9CWG9	Biogenesis of lysosome-related organelles complex 1 subunit 2 OS=Mus musculus GN=Bloc1s2 PE=1 SV=1 - [BL1S2_MOUSE]	37.76	1	7	7	19	1.12	0.88	0.80	0.90	0.90	1.17	0.97	1.07	0.93	1.05	0.96	1.11	0.98	16.29	4.88
E9Q4M4	Coiled-coil-helix-coiled-coil-helix domain-containing protein 6, mitochondrial OS=Mus musculus GN=Chchd6 PE=2 SV=1 - [E9Q4M4_MOUSE]	46.12	2	13	13	70	0.98	0.97	1.02	0.93	0.99	1.12	1.16	1.07	1.13	1.08	1.10	1.02	1.07	26.37	8.62
P59913	Protein-L-isoaspartate O-methyltransferase domain-containing protein 1 OS=Mus musculus GN=Pcmtd1 PE=2 SV=1 - [PCMD1_MOUSE]	5.32	1	2	2	2	1.10	1.25	1.13	1.14	1.04	1.01	0.91	1.08	0.97	1.07	0.97	1.20	1.09	40.67	5.66
Q80U93	Nuclear pore complex protein Nup214 OS=Mus musculus GN=Nup214 PE=1 SV=2 - [NU214_MOUSE]	4.89	6	7	8	14	0.86	1.01	1.17	0.99	1.03	1.06	1.14	1.08	1.26	1.06	1.19	0.95	1.04	212.85	7.08
O08644	Ephrin type-B receptor 6 OS=Mus musculus GN=Ephb6 PE=2 SV=4 - [EPHB6_MOUSE]	2.07	2	1	2	3	0.88	1.22	1.38	1.22	1.38	0.99	1.12	1.08	1.22	1.01	1.15	1.05	1.19	110.04	6.83
Q99N84	28S ribosomal protein S18b, mitochondrial OS=Mus musculus GN=Mrps18b PE=2 SV=1 - [RT18B_MOUSE]	27.56	5	3	3	5	0.96	0.76	0.79	0.92	0.95	0.89	0.93	1.08	1.22	0.98	1.02	0.86	0.90	28.68	8.35
Q6P3D0	U8 snoRNA-decapping enzyme OS=Mus musculus GN=Nudt16 PE=1 SV=1 - [NUD16_MOUSE]	17.44	2	2	3	5	1.30	1.12	0.86	0.95	0.73	0.91	0.70	1.08	0.82	1.02	0.78	1.12	0.86	21.81	7.12
Q3UXZ6	Protein FAM81A OS=Mus musculus GN=Fam81a PE=2 SV=2 - [FA81A_MOUSE]	23.35	5	8	9	15	1.09	1.07	1.08	1.08	1.02	1.18	1.02	1.08	0.98	1.11	0.98	1.01	0.98	41.68	8.75
P56213	FAD-linked sulfhydryl oxidase ALR OS=Mus musculus GN=Gfer PE=2 SV=2 - [ALR_MOUSE]	33.33	1	6	6	17	0.95	0.87	1.03	0.90	1.02	1.03	1.13	1.08	1.10	0.97	1.05	0.83	1.08	22.86	7.93

D6RGB6	Uncharacterized protein OS=Mus musculus GN=Vps9d1 PE=2 SV=1 - [D6RGB6_MOUSE]	8.15	4	1	1	2	0.88	1.03	1.17	1.04	1.18	1.02	1.15	1.08	1.21	1.09	1.24	1.10	1.25	25.62	9.29
Q9WV30	Nuclear factor of activated T-cells 5 OS=Mus musculus GN=Nfat5 PE=2 SV=1 - [NFAT5_MOUSE]	2.53	4	1	2	6	1.01	1.04	1.03	1.09	1.08	1.06	1.05	1.08	1.07	1.04	1.03	0.98	0.97	132.11	5.35
Q99N28	Cell adhesion molecule 3 OS=Mus musculus GN=Cadm3 PE=1 SV=1 - [CADM3_MOUSE]	39.90	2	11	11	78	1.00	0.99	0.98	1.03	1.06	0.99	0.99	1.08	1.07	0.99	1.01	0.99	1.00	42.94	5.80
Q8R404	Protein QIL1 OS=Mus musculus GN=Qil1 PE=2 SV=1 - [QIL1_MOUSE]	42.86	1	4	4	9	1.06	1.09	1.11	1.16	1.07	1.20	1.12	1.08	0.99	1.24	1.09	1.21	1.05	13.36	8.63
Q99KR6	E3 ubiquitin-protein ligase RNF34 OS=Mus musculus GN=Rnf34 PE=1 SV=1 - [RNF34_MOUSE]	14.36	1	4	4	8	1.01	0.84	0.91	0.81	0.86	1.08	1.11	1.08	1.17	0.87	0.96	0.97	1.01	42.00	4.84
P70388-4	Isoform 4 of DNA repair protein RAD50 OS=Mus musculus GN=Rad50 - [RAD50_MOUSE]	2.42	6	1	1	1	0.97	1.11	1.14	0.92	0.94	1.26	1.29	1.08	1.10	0.89	0.91	0.99	1.03	57.55	8.16
F8VQ11	Rho GTPase-activating protein 23 OS=Mus musculus GN=Arhgap23 PE=2 SV=1 - [F8VQ11_MOUSE]	7.99	4	7	8	14	1.06	1.06	1.00	0.96	0.93	0.96	1.03	1.08	1.01	1.03	1.08	1.06	1.08	139.22	9.06
Q922G2	Protein FAM76A OS=Mus musculus GN=Fam76a PE=2 SV=1 - [FA76A_MOUSE]	17.92	3	5	5	9	0.94	0.94	0.94	0.96	0.98	1.05	1.10	1.08	1.04	1.01	1.04	0.90	0.96	35.07	9.19
Q8C0D7-4	Isoform 4 of Inhibitor of growth protein 4 OS=Mus musculus GN=Ing4 - [ING4_MOUSE]	6.02	5	1	1	1	1.05	1.03	0.97	1.00	0.95	1.17	1.11	1.08	1.02	1.07	1.02	1.29	1.23	19.16	8.47
Q69ZW3-	Isoform 2 of EH domain-binding protein 1 OS=Mus musculus GN=Ehbp1 - [EHBP1_MOUSE]	20.48	3	16	16	31	1.05	0.91	0.89	1.00	0.94	1.07	1.04	1.08	0.99	1.05	0.98	1.04	1.05	136.15	5.39
E9PZ19	Protein Igsf9b OS=Mus musculus GN=Igsf9b PE=2 SV=1 - [E9PZ19_MOUSE]	6.55	2	6	6	15	1.04	1.27	1.22	1.15	1.08	0.99	1.02	1.08	1.06	0.98	0.99	0.85	0.91	144.86	6.70
Q920M5	Coronin-6 OS=Mus musculus GN=Coro6 PE=2 SV=1 - [CORO6_MOUSE]	4.03	5	1	2	3	0.88	1.33	1.51	1.06	1.20	0.93	1.05	1.08	1.22	1.01	1.15	1.13	1.29	52.57	5.96
Q8VDP2	UPF0428 protein CXorf56 homolog OS=Mus musculus PE=2 SV=1 - [CX056_MOUSE]	27.93	2	5	5	11	0.96	0.91	0.95	0.99	1.02	1.11	1.16	1.08	1.22	1.05	1.11	1.04	1.13	25.58	8.73

E9PUF7	Rho guanine nucleotide exchange factor 1 OS=Mus musculus GN=Arhgef1 PE=2 SV=1 - [E9PUF7_MOUSE]	5.33	7	4	4	7	1.03	1.21	1.17	1.13	1.10	1.12	1.10	1.08	1.07	1.09	1.06	1.13	1.09	108.63	6.09
D3Z6G3	Microtubule-associated protein RP/EB family member 3 OS=Mus musculus GN=Mapre3 PE=2 SV=1 - [D3Z6G3_MOUSE]	38.72	2	1	10	47	0.97	1.02	1.05	1.08	1.11	1.14	1.18	1.08	1.11	1.12	1.16	1.15	1.19	30.35	5.30
Q8VHP6	Cadherin-related family member 1 OS=Mus musculus GN=Cdhr1 PE=1 SV=1 - [CDHR1_MOUSE]	2.91	1	1	1	2	0.88	0.82	0.94	0.96	1.09	1.02	1.16	1.08	1.22	1.02	1.17	0.92	1.05	93.97	5.73
E9Q8E3	Protein Abi3bp OS=Mus musculus GN=Abi3bp PE=2 SV=1 - [E9Q8E3_MOUSE]	3.41	4	2	2	4	0.66	0.93	1.42	0.80	1.22	0.99	1.51	1.08	1.63	1.17	1.79	0.96	1.46	99.71	9.51
Q9D1L0	Coiled-coil-helix-coiled-coil-helix domain-containing protein 2, mitochondrial OS=Mus musculus GN=Chchd2 PE=1 SV=1 - [CHCH2_MOUSE]	30.72	3	4	4	72	0.98	0.98	1.10	0.86	0.94	1.08	1.10	1.08	1.08	1.00	1.00	0.92	0.96	15.65	9.66
Q8C0V9-3	Isoform 3 of FERM domain-containing protein 6 OS=Mus musculus GN=Frmd6 - [FRMD6_MOUSE]	1.78	3	1	1	2	0.95	1.41	1.49	1.17	1.23	1.09	1.14	1.08	1.13	1.12	1.19	1.18	1.25	64.18	8.31
E0CYM8	Tyrosine-protein phosphatase non-receptor type substrate 1 OS=Mus musculus GN=Sirpa PE=4 SV=1 - [E0CYM8_MOUSE]	49.32	6	1	19	181	2.02	3.34	1.65	0.77	0.38	2.01	0.99	1.08	0.53	1.98	0.98	1.23	0.61	56.38	8.28
Q80TH2-1	Isoform 1 of Protein LAP2 OS=Mus musculus GN=Erbb2ip - [LAP2_MOUSE]	12.06	4	12	13	24	1.01	1.12	1.24	1.00	1.00	1.06	1.07	1.08	1.07	1.07	1.07	1.10	1.15	154.23	5.58
Q8C145	Zinc transporter ZIP6 OS=Mus musculus GN=Slc39a6 PE=1 SV=1 - [S39A6_MOUSE]	16.47	2	8	8	34	1.15	0.96	0.93	1.01	0.85	1.11	0.94	1.08	0.94	1.10	0.97	1.16	0.99	86.33	6.84
P51949	CDK-activating kinase assembly factor MAT1 OS=Mus musculus GN=Mnat1 PE=2 SV=2 - [MAT1_MOUSE]	21.04	1	5	5	12	0.97	0.81	0.78	0.85	0.89	1.09	1.12	1.08	1.07	1.01	0.99	0.95	0.98	35.83	5.82
H3BKF6	Homeobox protein cut-like 1 OS=Mus musculus GN=Cux1 PE=2 SV=1 - [H3BKF6_MOUSE]	5.78	19	7	7	13	0.87	0.83	0.99	0.89	1.05	1.08	1.27	1.08	1.28	0.96	1.10	0.82	1.02	164.40	6.11

E9Q5E1	Protocadherin-19 OS=Mus musculus GN=Pcdh19 PE=2 SV=1 - [E9Q5E1_MOUSE]	4.92	3	5	5	9	1.26	1.43	1.18	1.16	0.98	1.21	0.97	1.08	0.97	1.19	0.94	1.28	1.04	120.75	5.40
Q3UYC0	Protein phosphatase 1H OS=Mus musculus GN=Ppm1h PE=1 SV=1 - [PPM1H_MOUSE]	38.01	7	15	15	32	0.93	1.08	1.15	1.00	1.09	1.06	1.22	1.08	1.20	1.05	1.13	1.11	1.17	56.34	6.73
G5E8J6	Histidine rich calcium binding protein, isoform CRA_a OS=Mus musculus GN=Hrc PE=4 SV=1 - [G5E8J6_MOUSE]	4.47	1	2	2	4	0.75	1.22	1.63	0.49	0.65	6.25	8.29	1.08	1.43	1.10	1.47	0.90	1.20	85.12	4.63
Q3UE37	Ubiquitin-conjugating enzyme E2 Z OS=Mus musculus GN=Ube2z PE=1 SV=2 - [UBE2Z_MOUSE]	14.04	1	4	4	5	1.12	0.93	0.83	1.16	1.00	0.96	0.89	1.08	0.97	0.95	0.87	1.19	1.12	38.34	5.62
O35454	Chloride transport protein 6 OS=Mus musculus GN=Clcn6 PE=1 SV=1 - [CLCN6_MOUSE]	7.47	3	4	4	5	1.24	1.28	1.11	1.15	0.96	1.01	0.88	1.08	0.94	1.31	1.06	0.78	0.69	96.92	7.02
Q9R0L6	Pericentriolar material 1 protein OS=Mus musculus GN=Pcm1 PE=1 SV=2 - [PCM1_MOUSE]	5.58	2	9	9	24	1.02	0.96	1.03	0.93	0.89	1.09	1.07	1.08	1.11	1.02	1.00	0.99	1.08	228.71	5.01
Q689Z5	Protein strawberry notch homolog 1 OS=Mus musculus GN=Sbno1 PE=1 SV=2 - [SBNO1_MOUSE]	0.58	3	1	1	1	0.91	0.92	1.01	1.13	1.24	0.94	1.02	1.08	1.18	0.95	1.04	1.06	1.17	153.64	8.07
M0QWP1	Agrin OS=Mus musculus GN=Agrn PE=4 SV=1 - [M0QWP1_MOUSE]	15.59	4	22	22	51	0.99	1.01	1.06	0.98	0.95	0.99	1.00	1.08	1.01	0.96	0.97	0.97	0.97	216.51	6.15
B9EHJ3	Tight junction protein ZO-1 OS=Mus musculus GN=Tjp1 PE=2 SV=1 - [B9EHJ3_MOUSE]	34.48	2	39	39	132	0.95	0.92	0.96	0.91	0.95	1.06	1.10	1.08	1.16	0.97	1.04	0.91	0.94	188.74	6.67
Q149L6-2	Isoform 2 of DnaJ homolog subfamily B member 14 OS=Mus musculus GN=Dnajb14 - [DJB14_MOUSE]	9.73	3	3	3	5	1.05	0.89	0.80	1.10	0.99	0.98	0.88	1.08	0.97	1.03	0.92	1.16	1.05	36.69	8.56
P49443	Protein phosphatase 1A OS=Mus musculus GN=Ppm1a PE=1 SV=1 - [PPM1A_MOUSE]	48.43	1	13	16	61	0.94	0.84	0.93	0.90	0.98	1.12	1.19	1.08	1.15	1.00	1.10	0.93	1.08	42.41	5.36
E9PUQ5	Golgin subfamily A member 2 OS=Mus musculus GN=Golga2 PE=2 SV=1 - [E9PUQ5_MOUSE]	28.75	8	20	20	64	0.99	0.82	0.94	0.91	0.92	1.00	1.03	1.08	1.12	0.93	0.93	0.83	0.92	116.21	5.05

Q80TL4	Protein KIAA1045 OS=Mus musculus GN=Kiaa1045 PE=1 SV=2 - [K1045_MOUSE]	42.00	2	4	16	97	0.97	0.98	1.00	1.05	1.07	1.13	1.22	1.08	1.13	1.02	1.04	0.97	1.00	45.19	5.77
Q8BYI9	Tenascin-R OS=Mus musculus GN=Tnr PE=1 SV=2 - [TENR_MOUSE]	43.00	2	43	43	500	0.99	0.98	0.99	0.94	0.96	1.04	1.05	1.08	1.09	1.00	1.01	0.99	1.01	149.49	4.94
A2A5R8	Double-stranded RNA-binding protein Staufen homolog 1 OS=Mus musculus GN=Stau1 PE=2 SV=1 - [A2A5R8_MOUSE]	15.26	4	6	6	13	0.93	0.83	0.87	0.94	0.99	1.02	1.02	1.08	1.14	0.99	1.05	0.88	0.92	53.71	9.55
E9QAT4	Protein Sec16a OS=Mus musculus GN=Sec16a PE=2 SV=1 - [E9QAT4_MOUSE]	13.36	3	18	18	41	1.13	1.12	0.98	1.02	0.92	1.10	1.01	1.08	0.94	1.06	1.00	1.03	0.91	254.05	5.81
Q9ES28-3	Isoform C of Rho guanine nucleotide exchange factor 7 OS=Mus musculus GN=Arhgef7 - [ARHG7_MOUSE]	37.74	10	20	22	52	0.96	0.96	1.02	0.93	1.00	0.98	1.02	1.08	1.10	0.97	1.03	0.97	1.06	88.89	6.28
Q921U8-2	Isoform L2 of Smoothelin OS=Mus musculus GN=Smtn - [SMTN_MOUSE]	4.45	8	3	3	4	0.97	1.22	1.31	0.97	0.98	0.93	0.93	1.08	1.10	1.24	1.25	1.08	1.09	100.07	8.81
A2ATT5	ADP-sugar pyrophosphatase (Fragment) OS=Mus musculus GN=Nudt5 PE=2 SV=1 - [A2ATT5_MOUSE]	26.71	2	2	2	4	0.75	0.99	1.32	0.88	1.17	0.99	1.31	1.08	1.42	0.98	1.30	1.02	1.35	17.67	5.40
Q812E0	Cytoplasmic polyadenylation element-binding protein 2 OS=Mus musculus GN=Cpeb2 PE=1 SV=1 - [CPEB2_MOUSE]	26.10	4	5	10	19	1.01	1.47	1.44	1.05	1.02	1.02	1.12	1.08	1.05	1.05	0.96	0.97	0.99	58.40	7.50
Q9JJR8	Transmembrane protein 9B OS=Mus musculus GN=Tmem9b PE=1 SV=1 - [TMM9B_MOUSE]	15.58	4	3	3	5	1.00	1.18	1.18	1.13	1.21	1.00	0.97	1.08	1.05	1.04	1.01	1.14	1.00	22.59	8.18
G5E896	Enhancer of mRNA decapping 4, isoform CRA_b OS=Mus musculus GN=Edc4 PE=4 SV=1 - [G5E896_MOUSE]	4.91	5	5	5	7	1.15	1.41	1.22	1.26	1.10	1.08	0.94	1.08	0.93	1.09	0.95	1.09	0.96	152.39	5.82
Q7TMY4-	Isoform 2 of THO complex subunit 7 homolog OS=Mus musculus GN=Thoc7 - [THOC7_MOUSE]	8.76	2	1	1	1	1.07	0.92	0.86	1.07	1.00	1.01	0.94	1.08	1.01	1.04	0.98	1.12	1.05	15.94	7.81
Q9WVE8	Protein kinase C and casein kinase substrate in neurons protein 2 OS=Mus musculus GN=Pacsin2 PE=1 SV=1 - [PACN2_MOUSE]	30.66	1	13	14	114	0.97	0.81	0.85	0.81	0.91	0.96	0.98	1.08	1.05	0.89	0.89	0.87	0.91	55.80	5.20

E9Q9U5	Protein Ildr2 OS=Mus musculus GN=Ildr2 PE=2 SV=1 - [E9Q9U5_MOUSE]	21.94	1	10	10	16	1.04	1.12	1.12	0.96	0.90	1.06	0.99	1.08	1.00	1.02	0.99	1.03	1.08	73.19	7.68
Q9WV55	Vesicle-associated membrane protein-associated protein A OS=Mus musculus GN=Vapa PE=1 SV=2 - [VAPA_MOUSE]	71.89	1	14	15	60	0.97	1.06	0.96	1.00	1.04	1.11	1.07	1.08	1.03	1.06	1.06	0.97	0.99	27.84	8.40
F6TP58	Protein Dchs1 (Fragment) OS=Mus musculus GN=Dchs1 PE=4 SV=1 - [F6TP58_MOUSE]	0.98	3	2	2	3	0.97	1.06	1.10	1.03	1.07	1.11	1.14	1.08	1.11	1.07	1.10	1.00	1.03	271.59	5.14
Q5U3K5	Rab-like protein 6 OS=Mus musculus GN=Rabl6 PE=1 SV=2 - [RABL6_MOUSE]	35.31	1	20	20	47	1.05	1.06	0.97	1.09	1.00	1.08	0.99	1.08	1.06	1.13	1.07	1.07	1.07	79.78	5.53
E9PZB3	Protein Gm5093 OS=Mus musculus GN=Gm5093 PE=3 SV=1 - [E9PZB3_MOUSE]	5.17	5	1	1	1	0.94	0.71	0.75	0.81	0.86	0.78	0.82	1.08	1.14	0.96	1.02	0.95	1.01	19.86	9.51
Q80ZU5	Coiled-coil domain-containing protein 181 OS=Mus musculus GN=Ccdc181 PE=2 SV=1 - [CC181_MOUSE]	3.54	1	1	2	6	1.44	1.20	0.83	1.07	0.74	1.15	0.79	1.08	0.75	1.05	0.73	1.21	0.84	59.21	5.33
Q91XH6	Vesicle transport through interaction with t-SNAREs 1B homolog OS=Mus musculus GN=Vti1b PE=2 SV=1 - [Q91XH6_MOUSE]	40.09	4	7	7	21	1.03	1.10	1.07	0.95	0.94	1.08	1.05	1.08	1.08	1.04	1.01	0.95	0.99	26.71	9.04
P19536	Cytochrome c oxidase subunit 5B, mitochondrial OS=Mus musculus GN=Cox5b PE=1 SV=1 - [COX5B_MOUSE]	74.22	2	9	9	354	0.98	0.72	0.72	0.88	0.90	1.11	1.12	1.08	1.10	0.98	1.00	0.91	0.93	13.80	8.38
D3YYP5	Non-syndromic hearing impairment protein 5 homolog OS=Mus musculus GN=Dfna5 PE=2 SV=2 - [D3YYP5_MOUSE]	9.52	3	2	2	3	0.88	1.38	1.57	1.23	1.40	1.02	1.16	1.08	1.23	1.02	1.16	1.35	1.55	44.25	5.31
F7CK47	Microtubule-associated protein (Fragment) OS=Mus musculus GN=Map4 PE=4 SV=1 - [F7CK47_MOUSE]	18.46	1	4	22	107	1.06	0.96	0.92	1.00	0.90	1.26	1.20	1.08	0.95	1.05	1.01	0.94	0.95	152.69	9.28
Q6ZPJ0	Testis-expressed sequence 2 protein OS=Mus musculus GN=Tex2 PE=1 SV=2 - [TEX2_MOUSE]	4.43	3	3	3	5	0.98	1.09	1.08	1.01	1.06	0.99	0.95	1.08	1.06	1.07	1.08	0.95	0.94	125.15	5.80

Q8CH09	SURP and G-patch domain-containing protein 2 OS=Mus musculus GN=Sugp2 PE=2 SV=2 - [SUGP2_MOUSE]	6.47	4	6	6	9	0.99	1.21	1.27	1.09	1.15	1.07	1.10	1.08	1.08	1.06	1.15	1.06	1.09	118.03	8.31
Q91XD2	LIM and senescent cell antigen-like-containing domain protein 2 OS=Mus musculus GN=Lims2 PE=1 SV=1 - [LIMS2_MOUSE]	13.20	1	1	4	9	1.16	0.92	0.80	0.92	0.80	1.12	0.96	1.08	0.93	1.01	0.88	0.97	0.84	39.00	7.97
P83882	60S ribosomal protein L36a OS=Mus musculus GN=Rpl36a PE=2 SV=2 - [RL36A_MOUSE]	16.04	1	2	2	8	1.13	0.82	0.72	0.92	0.76	1.12	0.98	1.08	0.94	0.96	0.85	0.87	0.81	12.43	10.58
Q9JKF7	39S ribosomal protein L39, mitochondrial OS=Mus musculus GN=Mrpl39 PE=2 SV=4 - [RM39_MOUSE]	3.57	1	2	2	4	1.03	1.42	1.37	1.01	0.98	1.00	0.97	1.08	1.04	1.16	1.13	1.17	1.14	38.52	7.94
Q60902-4	Isoform 4 of Epidermal growth factor receptor substrate 15-like 1 OS=Mus musculus GN=Eps15l1 - [EP15R_MOUSE]	51.11	1	1	30	89	1.00	0.93	0.93	0.97	0.96	0.93	0.93	1.08	1.07	0.96	0.96	0.92	0.92	84.70	5.02
O54946	DnaJ homolog subfamily B member 6 OS=Mus musculus GN=Dnajb6 PE=1 SV=4 - [DNJB6_MOUSE]	23.56	3	3	7	25	1.02	1.35	1.36	1.08	1.10	1.06	1.04	1.08	0.98	1.21	1.37	0.96	0.96	39.78	9.36
P62830	60S ribosomal protein L23 OS=Mus musculus GN=Rpl23 PE=1 SV=1 - [RL23_MOUSE]	36.43	2	4	4	7	1.17	0.98	0.93	1.00	0.97	1.19	1.14	1.08	1.11	1.14	1.08	1.20	1.03	14.86	10.51
A2AQ25	Sickle tail protein OS=Mus musculus GN=Skt PE=1 SV=1 - [SKT_MOUSE]	21.12	14	31	31	71	0.89	0.93	1.00	1.01	1.17	1.06	1.10	1.08	1.17	0.95	1.08	0.90	1.02	212.90	7.77
Q9CPS8	Small membrane A-kinase anchor protein OS=Mus musculus PE=1 SV=1 - [SMAKA_MOUSE]	12.26	1	1	1	3	1.24	0.90	0.72	1.02	0.82	1.44	1.15	1.08	0.87	1.03	0.83	1.02	0.82	11.92	4.25
Q921W0	Charged multivesicular body protein 1a OS=Mus musculus GN=Chmp1a PE=1 SV=1 - [CHM1A_MOUSE]	12.24	1	3	3	9	0.98	0.92	0.99	0.95	0.89	1.15	1.17	1.08	0.95	1.03	0.97	0.99	0.87	21.59	8.06
Q922U1	U4/U6 small nuclear ribonucleoprotein Prp3 OS=Mus musculus GN=Prpf3 PE=1 SV=1 - [PRPF3_MOUSE]	15.52	2	7	7	10	1.02	1.00	0.94	0.96	0.94	1.23	1.11	1.08	1.16	0.98	0.95	0.94	0.98	77.41	9.50
P69566	Ran-binding protein 9 OS=Mus musculus GN=Ranbp9 PE=1 SV=1 - [RANB9_MOUSE]	13.32	3	5	5	14	1.02	1.01	1.15	1.06	1.02	0.96	0.95	1.08	1.03	1.02	0.95	1.13	1.15	70.97	6.84

E9QPH0	cGMP-dependent protein kinase OS=Mus musculus GN=Prkg2 PE=2 SV=1 - [E9QPH0_MOUSE]	3.14	3	2	2	3	0.97	1.12	1.15	0.95	0.97	1.14	1.16	1.08	1.11	0.88	0.91	1.12	1.15	84.16	8.40
P60191	Regulating synaptic membrane exocytosis protein 4 OS=Mus musculus GN=Rims4 PE=2 SV=1 - [RIMS4_MOUSE]	6.69	1	1	1	2	0.87	1.02	1.17	1.06	1.22	1.05	1.21	1.08	1.24	1.04	1.19	1.02	1.17	29.31	5.80
Q8R2V3	Zinc finger protein 445 OS=Mus musculus GN=Znf445 PE=2 SV=2 - [ZN445_MOUSE]	3.14	1	1	1	2	1.00	0.87	0.87	0.87	0.87	0.94	0.93	1.08	1.08	1.01	1.01	0.91	0.92	114.70	9.32
H7BWX9	Small ubiquitin-related modifier 2 OS=Mus musculus GN=Sumo2 PE=2 SV=1 - [H7BWX9_MOUSE]	41.51	4	1	2	18	1.06	0.76	0.72	0.94	0.89	1.13	1.06	1.08	1.02	1.00	0.94	0.92	0.87	6.01	8.91
Q9WU60	Attractin OS=Mus musculus GN=Atrn PE=2 SV=3 - [ATRN_MOUSE]	1.61	1	2	2	4	1.10	1.08	0.98	1.08	0.98	0.97	0.87	1.08	0.98	0.90	0.82	0.87	0.80	157.95	7.27
O88491	Histone-lysine N-methyltransferase, H3 lysine-36 and H4 lysine-20 specific OS=Mus musculus GN=Nsd1 PE=1 SV=1 - [NSD1_MOUSE]	0.35	2	1	1	2	1.04	1.14	1.09	0.95	0.92	1.02	0.97	1.08	1.03	1.01	0.97	0.97	0.93	283.91	7.90
Q9DCZ1	GMP reductase 1 OS=Mus musculus GN=Gmpr PE=2 SV=1 - [GMPR1_MOUSE]	10.72	2	2	3	5	0.93	0.94	0.79	1.73	1.86	0.95	1.02	1.08	1.16	1.15	1.24	0.90	0.97	37.46	7.09
P35922-9	Isoform ISO9 of Fragile X mental retardation protein 1 homolog OS=Mus musculus GN=Fmr1 - [FMR1_MOUSE]	20.95	18	7	10	18	0.88	0.99	1.08	0.99	1.12	1.01	1.12	1.08	1.19	0.87	1.01	1.01	1.09	64.06	8.53
Q62313	Trans-Golgi network integral membrane protein 1 OS=Mus musculus GN=Tgoln1 PE=1 SV=1 - [TGON1_MOUSE]	8.50	2	2	2	3	1.09	0.90	0.82	1.01	0.93	1.03	0.94	1.08	0.99	1.00	0.92	1.01	0.93	37.83	5.34
Q8CBH7-	Isoform 2 of E3 ubiquitin-protein ligase MARCH11 OS=Mus musculus GN=March11 - [MARHB_MOUSE]	5.56	3	1	1	2	1.01	0.98	0.97	0.96	0.95	1.00	0.99	1.08	1.07	1.11	1.10	1.06	1.05	27.14	7.33
F2Z3U3	Protein Raph1 (Fragment) OS=Mus musculus GN=Raph1 PE=2 SV=1 - [F2Z3U3_MOUSE]	17.71	5	8	10	24	0.96	1.05	0.96	1.06	1.02	1.01	1.02	1.08	1.03	1.01	0.98	1.03	1.01	66.93	6.07
Q9WTV7	E3 ubiquitin-protein ligase RLIM OS=Mus musculus GN=Rlim PE=1 SV=2 - [RNF12_MOUSE]	2.00	1	1	1	2	0.90	0.82	0.91	0.73	0.81	1.02	1.12	1.08	1.20	1.02	1.14	0.92	1.02	66.34	6.90

Q6PH08	ERC protein 2 OS=Mus musculus GN=Erc2 PE=1 SV=2 - [ERC2_MOUSE]	52.87	6	29	56	451	0.99	0.88	0.88	0.92	0.91	1.07	1.06	1.08	1.06	0.99	0.98	0.91	0.92	110.57	7.03
Q6P6I6	DNA-directed RNA polymerase II subunit GRINL1A OS=Mus musculus GN=Polr2m PE=2 SV=2 - [GRL1A_MOUSE]	10.66	3	3	3	7	1.23	1.02	0.83	0.88	0.83	1.16	1.11	1.08	0.87	1.10	0.89	1.06	0.73	41.21	6.55
P46938-2	Isoform 2 of Yorkie homolog OS=Mus musculus GN=Yap1 - [YAP1_MOUSE]	24.36	6	6	6	15	0.99	1.05	1.13	1.01	1.00	1.08	1.11	1.08	1.24	1.11	1.13	0.92	1.09	50.67	5.00
Q6DFV7	Nuclear receptor coactivator 7 OS=Mus musculus GN=Ncoa7 PE=2 SV=2 - [NCOA7_MOUSE]	27.57	6	18	18	40	0.99	0.84	0.79	0.95	0.93	1.09	1.07	1.08	1.04	1.01	0.95	0.95	0.92	106.29	5.43
P0C7M9	C-type lectin domain family 2 member L OS=Mus musculus GN=Clec2l PE=2 SV=1 - [CLC2L_MOUSE]	4.27	1	1	1	2	1.09	1.47	1.35	1.17	1.07	0.92	0.84	1.08	0.99	1.08	1.00	1.35	1.24	23.64	7.20
Q3UGS4	Protein FAM195B OS=Mus musculus GN=Fam195b PE=1 SV=1 - [F195B_MOUSE]	58.76	1	5	5	24	1.01	0.92	0.99	0.94	0.97	1.15	1.16	1.08	1.08	0.96	1.04	0.89	0.93	11.09	9.14
Q61165	Sodium/hydrogen exchanger 1 OS=Mus musculus GN=Slc9a1 PE=1 SV=1 - [SL9A1_MOUSE]	17.93	1	8	8	39	1.07	1.05	1.00	1.08	1.00	1.09	1.01	1.08	0.98	1.08	1.03	1.00	0.95	91.41	7.12
Q9R1E0	Forkhead box protein O1 OS=Mus musculus GN=Foxo1 PE=1 SV=2 - [FOXO1_MOUSE]	2.30	1	1	1	2	1.08	0.98	0.91	0.93	0.86	1.19	1.10	1.08	1.00	1.08	1.00	1.46	1.36	69.47	6.95
Q78JW9	Ubiquitin domain-containing protein UBFD1 OS=Mus musculus GN=Ubfd1 PE=1 SV=2 - [UBFD1_MOUSE]	27.99	1	7	7	28	1.12	0.74	0.75	0.91	0.89	1.06	0.97	1.08	0.97	1.01	0.98	0.90	0.87	40.12	8.85
A2AU91	Tumor suppressor p53-binding protein 1 OS=Mus musculus GN=Trp53bp1 PE=2 SV=1 - [A2AU91_MOUSE]	32.76	11	35	35	91	1.07	0.97	0.89	1.04	0.96	1.15	0.98	1.08	1.07	1.08	0.99	1.03	0.94	212.60	4.63
Q80TS7	Dendrin OS=Mus musculus GN=Ddn PE=1 SV=3 - [DEND_MOUSE]	5.49	1	3	3	6	0.97	1.06	0.99	1.07	1.13	1.05	0.99	1.08	1.01	0.95	0.89	0.90	0.92	76.36	9.98
O89001	Carboxypeptidase D OS=Mus musculus GN=Cpd PE=1 SV=2 - [CBPD_MOUSE]	1.82	1	3	3	5	1.15	1.29	1.26	1.13	0.98	0.96	0.87	1.08	0.84	0.96	0.84	1.01	0.86	152.31	6.18
Q9Z2Y3-5	Isoform 5 of Homer protein homolog 1 OS=Mus musculus GN=Homer1 - [HOME1_MOUSE]	46.77	5	1	9	83	0.97	1.46	1.50	1.32	1.36	1.21	1.24	1.08	1.11	0.92	0.95	1.17	1.21	20.90	7.62

E9PV14	Band 4.1-like protein 1 (Fragment) OS=Mus musculus GN=Epb4.1l1 PE=2 SV=1 - [E9PV14_MOUSE]	36.09	1	12	28	98	1.04	1.05	1.00	0.90	0.82	1.03	0.92	1.08	1.08	0.95	0.90	0.99	0.89	117.10	5.92
P62627	Dynein light chain roadblock-type 1 OS=Mus musculus GN=Dynlrb1 PE=1 SV=3 - [DLRB1_MOUSE]	73.96	2	7	7	71	1.04	0.87	0.79	0.94	0.93	1.12	1.05	1.08	1.03	1.00	0.97	0.94	0.96	10.98	7.25
Q8BKQ3	Testican-1 OS=Mus musculus GN=Spock1 PE=2 SV=1 - [Q8BKQ3_MOUSE]	29.61	2	8	8	15	0.90	0.88	0.99	0.94	1.07	1.14	1.27	1.08	1.25	1.01	1.24	0.94	0.96	49.22	6.01
Q8CE90-4	Isoform 4 of Dual specificity mitogen-activated protein kinase kinase 7 OS=Mus musculus GN=Map2k7 - [MP2K7_MOUSE]	5.20	11	1	2	3	0.70	4.45	6.36	1.11	1.58	0.89	1.27	1.08	1.55	1.13	1.61	1.11	1.59	39.35	8.63
P59708	Pre-mRNA branch site protein p14 OS=Mus musculus GN=Sf3b14 PE=2 SV=1 - [PM14_MOUSE]	11.20	1	1	1	3	0.94	0.85	0.90	1.03	1.09	0.98	1.03	1.08	1.14	0.99	1.05	1.02	1.09	14.58	9.38
Q8C052	Microtubule-associated protein 1S OS=Mus musculus GN=Map1s PE=1 SV=2 - [MAP1S_MOUSE]	8.94	1	7	7	12	1.04	1.14	1.11	1.01	1.03	1.03	1.07	1.08	1.08	1.03	1.09	1.16	1.13	102.88	7.02
Q3TXN1	Prostaglandin reductase 2 OS=Mus musculus GN=Ptgr2 PE=2 SV=1 - [Q3TXN1_MOUSE]	25.08	4	5	5	16	0.96	1.04	1.14	0.95	0.98	1.06	1.03	1.08	1.07	1.07	1.06	1.03	1.12	34.25	5.50
H3BJI4	RWD domain containing 4A, isoform CRA_b OS=Mus musculus GN=Rwdd4a PE=4 SV=1 - [H3BJI4_MOUSE]	45.45	3	2	2	6	1.03	0.98	0.90	0.92	0.93	1.06	1.23	1.08	1.16	1.02	1.06	0.94	0.92	5.03	4.64
P63017	Heat shock cognate 71 kDa protein OS=Mus musculus GN=Hspa8 PE=1 SV=1 - [HSP7C_MOUSE]	78.33	4	30	46	1182	0.93	0.81	0.91	0.95	1.03	1.05	1.14	1.08	1.14	1.02	1.11	0.96	1.05	70.83	5.52
Q9D6J5	NADH dehydrogenase [ubiquinone] 1 beta subcomplex subunit 8, mitochondrial OS=Mus musculus GN=Ndufb8 PE=1 SV=1 - [NDUB8_MOUSE]	46.77	4	6	6	35	1.07	1.26	1.22	1.11	1.07	1.04	1.04	1.08	1.06	1.13	1.06	1.14	1.04	21.86	6.64

E9QKA4	Serine/arginine repetitive matrix protein 1 OS=Mus musculus GN=Srrm1 PE=2 SV=1 - [E9QKA4_MOUSE]	9.14	8	4	8	14	0.94	1.07	1.05	0.94	0.98	0.97	1.01	1.08	1.02	0.99	1.03	0.88	0.93	101.10	11.94
Q2TA57	Aspartate beta-hydroxylase domain-containing protein 1 OS=Mus musculus GN=Asphd1 PE=2 SV=2 - [ASPH1_MOUSE]	10.00	1	2	2	2	1.06	1.07	1.00	1.28	1.20	0.91	0.85	1.09	0.96	0.95	0.90	0.93	0.88	38.24	8.25
Q9JKC7	AP-4 complex subunit mu-1 OS=Mus musculus GN=Ap4m1 PE=2 SV=1 - [AP4M1_MOUSE]	3.34	1	1	1	1	0.54	1.32	2.45	0.98	1.83	0.92	1.70	1.09	2.01	1.06	1.97	0.95	1.78	49.48	6.64
P97742	Carnitine O-palmitoyltransferase 1, liver isoform OS=Mus musculus GN=Cpt1a PE=1 SV=4 - [CPT1A_MOUSE]	3.88	1	2	2	3	1.36	1.39	1.02	1.38	1.01	1.05	0.77	1.09	0.80	1.21	0.89	1.48	1.09	88.19	8.62
Q3UKC1	Tax1-binding protein 1 homolog OS=Mus musculus GN=Tax1bp1 PE=1 SV=2 - [TAXB1_MOUSE]	11.43	3	8	8	11	0.99	0.97	1.03	1.01	1.06	0.94	1.00	1.09	1.03	1.04	1.11	0.90	1.04	93.57	5.33
A2AMY5	Ubiquitin-associated protein 2 OS=Mus musculus GN=Ubap2 PE=2 SV=1 - [A2AMY5_MOUSE]	13.26	3	10	10	19	1.01	0.85	0.79	0.96	0.89	1.11	1.06	1.09	1.12	1.03	1.02	0.93	0.87	117.76	7.72
Q921H8	3-ketoacyl-CoA thiolase A, peroxisomal OS=Mus musculus GN=Acaa1a PE=2 SV=1 - [THIKA_MOUSE]	49.06	7	13	13	29	0.92	0.91	0.96	0.99	1.04	1.05	1.09	1.09	1.14	1.05	1.18	1.05	1.18	43.93	8.44
E9QKY7	Transcription factor 20 OS=Mus musculus GN=Tcf20 PE=2 SV=1 - [E9QKY7_MOUSE]	8.75	4	13	14	24	1.03	0.97	0.91	1.06	1.00	1.10	1.10	1.09	1.06	1.08	1.07	1.15	1.10	213.43	9.03
Q3UGF1-	Isoform 3 of WD repeat containing protein 19 OS=Mus musculus GN=Wdr19 - [WDR19_MOUSE]	0.69	3	1	1	1	1.00	0.83	0.83	0.93	0.93	1.18	1.17	1.09	1.08	0.96	0.96	1.10	1.10	115.07	6.29
Q9ET77	Junctophilin-3 OS=Mus musculus GN=Jph3 PE=1 SV=1 - [JPH3_MOUSE]	11.02	1	6	6	12	0.98	1.03	1.09	0.94	0.96	1.08	1.11	1.09	1.08	0.97	1.01	0.97	1.00	81.18	9.22
E9Q9B7	Protein Kidins220 OS=Mus musculus GN=Kidins220 PE=2 SV=1 - [E9Q9B7_MOUSE]	7.14	1	10	10	14	1.04	1.14	1.06	1.07	1.03	0.97	0.96	1.09	0.99	1.11	1.06	1.06	1.01	199.06	6.67
O54998	Peptidyl-prolyl cis-trans isomerase FKBP7 OS=Mus musculus GN=Fkbp7 PE=1 SV=1 - [FKBP7_MOUSE]	11.01	2	3	3	3	1.13	0.84	0.81	0.91	0.84	1.16	1.02	1.09	0.85	1.00	0.77	0.73	0.64	24.90	6.04

Q8VDN4	Coiled-coil domain-containing protein 92 OS=Mus musculus GN=Ccdc92 PE=1 SV=1 - [CCD92_MOUSE]	42.36	3	13	13	32	1.00	0.91	0.94	0.90	0.92	1.10	1.04	1.09	1.16	1.04	1.06	0.93	0.93	35.19	9.38
F6XC25	Coiled-coil and C2 domain-containing protein 1B (Fragment) OS=Mus musculus GN=Cc2d1b PE=4 SV=1 - [F6XC25_MOUSE]	11.95	2	6	6	8	0.97	1.02	1.07	0.95	0.94	0.94	0.92	1.09	1.08	1.01	1.05	1.13	1.03	84.96	5.87
O54786-2	Isoform ICAD-S of DNA fragmentation factor subunit alpha OS=Mus musculus GN=Dffa - [DFFA_MOUSE]	30.94	1	1	6	17	0.85	1.10	1.29	0.83	0.97	1.09	1.28	1.09	1.27	0.92	1.09	0.62	0.74	29.16	4.59
Q3TYF1	NGFI-A-binding protein 2 OS=Mus musculus GN=Nab2 PE=2 SV=1 - [Q3TYF1_MOUSE]	1.95	2	1	1	1	1.11	1.17	1.06	1.00	0.90	1.01	0.91	1.09	0.98	0.99	0.89	1.04	0.94	49.70	6.28
P02469	Laminin subunit beta-1 OS=Mus musculus GN=Lamb1 PE=1 SV=3 - [LAMB1_MOUSE]	1.18	2	2	2	3	0.94	0.87	0.93	0.92	0.98	1.09	1.15	1.09	1.15	1.14	1.21	0.99	1.05	196.96	4.94
A2AV92	PHD finger protein 20 (Fragment) OS=Mus musculus GN=Phf20 PE=2 SV=1 - [A2AV92_MOUSE]	9.73	3	1	1	1	1.04	1.91	1.83	0.98	0.94	1.23	1.18	1.09	1.04	0.96	0.92	1.02	0.98	13.66	6.68
Q8BGY2	Eukaryotic translation initiation factor 5A-2 OS=Mus musculus GN=Eif5a2 PE=2 SV=3 - [IF5A2_MOUSE]	37.91	1	8	8	57	1.00	0.79	0.79	0.90	0.88	1.13	1.03	1.09	1.08	0.97	0.98	0.89	0.87	16.78	5.58
P99029-2	Isoform Cytoplasmic+peroxisomal of Peroxiredoxin-5, mitochondrial OS=Mus musculus GN=Prdx5 - [PRDX5_MOUSE]	74.69	4	13	13	117	0.90	0.81	0.91	0.95	1.04	1.04	1.19	1.09	1.22	1.00	1.10	0.98	1.11	17.00	7.87
Q00PI9	Heterogeneous nuclear ribonucleoprotein U-like protein 2 OS=Mus musculus GN=Hnrnpul2 PE=1 SV=2 - [HNRL2_MOUSE]	30.20	1	18	18	61	0.95	0.84	1.05	0.97	1.03	1.07	1.10	1.09	1.14	0.99	1.08	1.06	1.13	84.89	4.89
E9PYH0	Versican core protein OS=Mus musculus GN=Vcan PE=2 SV=1 - [E9PYH0_MOUSE]	9.45	6	4	25	317	1.13	0.91	0.74	0.88	0.78	1.16	0.95	1.09	0.84	0.94	0.79	0.97	0.87	366.67	4.63
P56375	Acylphosphatase-2 OS=Mus musculus GN=Acyp2 PE=2 SV=2 - [ACYP2_MOUSE]	43.40	1	4	4	18	1.09	0.86	0.81	0.91	0.87	1.13	0.97	1.09	0.94	0.97	0.83	1.00	0.89	11.87	9.26

E9Q132	60S ribosomal protein L24 OS=Mus musculus GN=Rpl24 PE=2 SV=1 - [E9Q132_MOUSE]	21.05	3	4	4	9	1.04	1.06	1.08	1.02	1.01	0.94	0.96	1.09	1.08	0.98	1.00	1.06	1.08	15.34	10.87
Q8R3D1	TBC1 domain family member 13 OS=Mus musculus GN=Tbc1d13 PE=2 SV=1 - [TBC13_MOUSE]	4.25	1	1	1	2	1.23	0.89	0.72	1.01	0.82	1.12	0.91	1.09	0.88	0.97	0.79	0.96	0.78	46.42	5.36
Q8K382	DENN domain-containing protein 1A OS=Mus musculus GN=Dennd1a PE=1 SV=2 - [DEN1A_MOUSE]	10.93	2	6	6	13	1.06	1.31	1.23	1.02	1.03	1.05	0.92	1.09	1.07	1.03	0.97	1.02	0.94	111.47	6.79
F7CC56	RB1-inducible coiled-coil protein 1 (Fragment) OS=Mus musculus GN=Rb1cc1 PE=4 SV=1 - [F7CC56_MOUSE]	2.25	4	4	4	5	0.96	1.02	1.11	0.97	1.07	1.00	1.08	1.09	1.05	1.05	1.04	1.00	1.07	168.86	5.52
Q9DB15	39S ribosomal protein L12, mitochondrial OS=Mus musculus GN=Mrpl12 PE=1 SV=2 - [RM12_MOUSE]	54.23	1	8	8	49	1.01	0.89	0.88	0.94	0.94	1.09	1.15	1.09	1.08	0.99	0.98	0.95	0.95	21.69	9.29
Q4VAA2	Protein CDV3 OS=Mus musculus GN=Cdv3 PE=1 SV=2 - [CDV3_MOUSE]	74.02	3	20	20	171	1.08	0.88	0.78	0.96	0.89	1.12	1.04	1.09	1.02	1.02	0.94	0.95	0.88	29.71	6.10
Q8BIF9	Zinc finger protein 787 OS=Mus musculus GN=Znf787 PE=2 SV=3 - [ZN787_MOUSE]	3.94	1	1	1	1	1.12	2.40	2.13	1.31	1.17	1.31	1.17	1.09	0.97	1.19	1.06	1.50	1.34	40.45	8.24
D3Z1N9	MCG9889 OS=Mus musculus GN=Gm10709 PE=4 SV=1 - [D3Z1N9_MOUSE]	24.38	6	4	4	8	1.10	0.85	0.76	1.06	0.97	1.15	1.07	1.09	1.00	1.15	1.07	0.90	0.82	17.64	11.84
A2AJ20	Neural proliferation differentiation and control protein 1 (Fragment) OS=Mus musculus GN=Npdc1 PE=2 SV=1 - [A2AJ20_MOUSE]	13.42	4	2	2	4	1.33	0.96	0.72	1.07	0.81	1.09	0.81	1.09	0.81	1.07	0.80	1.15	0.86	24.53	8.73
Q91V76	Ester hydrolase C11orf54 homolog OS=Mus musculus PE=2 SV=1 - [CK054_MOUSE]	20.95	1	6	6	10	0.90	0.73	0.83	1.00	1.09	1.09	1.18	1.09	1.16	1.11	1.22	1.07	1.09	34.97	6.29
Q9CXF4	TBC1 domain family member 15 OS=Mus musculus GN=Tbc1d15 PE=1 SV=1 - [TBC15_MOUSE]	2.24	1	1	1	2	0.86	1.24	1.44	1.10	1.28	0.95	1.10	1.09	1.26	1.13	1.32	1.09	1.28	76.48	5.30
A2AJ95	NMDA receptor synaptonuclear-signaling and neuronal migration factor OS=Mus musculus GN=Nsmf PE=2 SV=1 - [A2AJ95_MOUSE]	10.02	9	3	3	8	0.88	1.01	1.16	1.01	1.15	0.95	1.25	1.09	1.20	1.02	1.16	1.05	1.27	53.92	8.51

D3Z1M4	Calcium-binding protein 1 OS=Mus musculus GN=Cabp1 PE=2 SV=1 - [D3Z1M4_MOUSE]	35.14	4	11	11	35	0.93	1.02	1.05	0.85	0.93	1.10	1.27	1.09	1.21	0.88	0.97	0.86	0.90	37.89	8.28
A2AUK5	Band 4.1-like protein 1 OS=Mus musculus GN=Epb4.1l1 PE=4 SV=1 - [A2AUK5_MOUSE]	45.73	7	18	34	242	1.05	0.89	0.84	0.97	0.89	1.09	1.03	1.09	0.99	1.08	0.97	0.99	0.93	98.25	5.66
A2A543	Voltage-dependent L-type calcium channel subunit beta-1 OS=Mus musculus GN=Cacnb1 PE=2 SV=1 - [A2A543_MOUSE]	25.80	7	6	10	26	1.04	1.09	1.10	1.06	1.00	1.10	1.12	1.09	0.99	1.15	1.14	1.10	1.13	65.44	6.62
A2AWI7	Endophilin-B2 OS=Mus musculus GN=Sh3glb2 PE=2 SV=1 - [A2AWI7_MOUSE]	43.56	3	2	14	66	0.84	1.13	1.15	0.88	1.02	1.05	1.29	1.09	1.32	1.05	1.19	1.01	1.12	44.88	5.58
Q9Z131-3	Isoform 3 of SH3 domain-binding protein 5 OS=Mus musculus GN=Sh3bp5 - [3BP5_MOUSE]	8.33	3	3	3	5	0.93	0.98	1.22	0.91	0.99	1.02	1.09	1.09	1.34	0.99	1.16	1.20	1.46	49.90	7.83
Q9D4C5	ELL-associated factor 1 OS=Mus musculus GN=Eaf1 PE=1 SV=2 - [EAF1_MOUSE]	8.96	1	1	1	3	0.99	0.67	0.68	0.75	0.76	1.12	1.13	1.09	1.10	1.01	1.02	1.18	1.20	28.95	5.40
P15208	Insulin receptor OS=Mus musculus GN=Insr PE=1 SV=2 - [INSR_MOUSE]	2.55	1	2	3	6	1.10	1.05	0.96	1.09	0.99	1.05	0.96	1.09	0.99	1.07	0.98	1.08	0.99	155.51	5.95
Q7TQH0	Ataxin-2-like protein OS=Mus musculus GN=Atxn2l PE=1 SV=1 - [ATX2L_MOUSE]	30.22	3	1	22	73	1.12	1.19	1.06	1.06	0.95	1.11	0.99	1.09	0.97	1.15	1.03	1.20	1.07	110.58	8.85
Q9EP53-4	Isoform 4 of Hamartin OS=Mus musculus GN=Tsc1 - [TSC1_MOUSE]	5.92	4	4	4	7	0.96	1.06	1.14	1.00	1.10	0.93	0.95	1.09	1.11	1.07	1.07	1.09	1.18	127.30	6.40
E9Q0T8	Protein Dnah7a OS=Mus musculus GN=Dnah7a PE=4 SV=2 - [E9Q0T8_MOUSE]	0.30	2	1	1	1	0.81	1.08	1.33	0.79	0.98	1.24	1.53	1.09	1.34	1.15	1.42	0.85	1.05	460.61	6.20
Q61166	Microtubule-associated protein RP/EB family member 1 OS=Mus musculus GN=Mapre1 PE=1 SV=3 - [MARE1_MOUSE]	42.91	1	6	9	31	0.96	0.82	0.79	0.96	0.95	1.14	1.18	1.09	1.14	1.04	1.11	0.98	1.05	30.00	5.22
Q8R205	Zinc finger CCCH domain-containing protein 10 OS=Mus musculus GN=Zc3h10 PE=2 SV=1 - [ZC3HA_MOUSE]	2.30	1	1	1	2	1.08	0.93	0.86	0.83	0.76	1.19	1.09	1.09	1.00	1.13	1.05	1.03	0.95	46.09	7.62
Q9QY42-[illegible]	Isoform 2 of Probable G-protein coupled receptor 37 OS=Mus musculus GN=Gpr37 - [GPR37_MOUSE]	3.72	3	1	1	2	1.13	1.61	1.41	1.15	1.01	0.98	0.86	1.09	0.96	1.02	0.90	1.05	0.93	39.25	6.19

Q9CQ01	Ribonuclease T2 OS=Mus musculus GN=Rnaset2 PE=2 SV=1 - [RNT2_MOUSE]	2.70	1	1	1	2	1.04	0.76	0.73	0.92	0.88	1.01	0.96	1.09	1.04	1.07	1.03	1.12	1.07	29.59	6.37
F6VQZ6	Protein shisa-6 homolog (Fragment) OS=Mus musculus GN=Shisa6 PE=4 SV=1 - [F6VQZ6_MOUSE]	16.57	2	7	7	15	1.03	1.17	0.98	0.97	0.93	1.11	1.02	1.09	1.09	1.07	1.00	1.20	1.00	56.20	9.19
Q7TPD0-2	Isoform 2 of Integrator complex subunit 3 OS=Mus musculus GN=Ints3 - [INT3_MOUSE]	18.79	2	3	3	4	0.99	1.03	0.93	0.94	0.96	1.11	1.11	1.09	1.04	1.13	1.11	0.99	1.08	36.63	8.51
Q8BJF9	Charged multivesicular body protein 2b OS=Mus musculus GN=Chmp2b PE=2 SV=1 - [CHM2B_MOUSE]	20.66	1	4	5	12	1.07	0.87	0.84	0.98	0.89	1.09	0.98	1.09	1.06	0.94	0.94	0.94	0.87	23.92	8.78
A2AR02	Peptidyl-prolyl cis-trans isomerase G OS=Mus musculus GN=Ppig PE=1 SV=1 - [PPIG_MOUSE]	4.79	2	3	3	9	1.00	0.96	0.88	0.96	1.00	0.94	1.00	1.09	1.16	0.97	1.01	1.10	1.03	88.27	10.27
Q3U9F6	RING finger protein 166 OS=Mus musculus GN=Rnf166 PE=2 SV=1 - [RN166_MOUSE]	7.59	1	1	1	3	0.99	1.00	1.01	0.99	1.00	0.98	0.98	1.09	1.10	1.06	1.07	0.94	0.95	26.05	8.06
E9Q616	Protein Ahnak OS=Mus musculus GN=Ahnak PE=2 SV=1 - [E9Q616_MOUSE]	58.29	2	156	158	412	1.02	0.80	0.77	0.89	0.88	1.04	1.01	1.09	1.09	1.06	1.03	0.85	0.82	603.87	6.30
P58686	UPF0184 protein C9orf16 homolog OS=Mus musculus PE=2 SV=1 - [CI016_MOUSE]	10.84	1	1	1	2	0.83	1.01	1.21	0.92	1.11	0.87	1.04	1.09	1.31	0.94	1.13	1.04	1.25	9.05	4.26
Q3TQI7	Uncharacterized protein C9orf78 homolog OS=Mus musculus PE=1 SV=2 - [CI078_MOUSE]	32.87	1	9	9	28	0.97	0.83	0.84	0.91	0.94	1.09	1.09	1.09	1.10	0.92	1.01	0.96	0.95	33.52	6.34
Q9CYG7-	Isoform 2 of Mitochondrial import receptor subunit TOM34 OS=Mus musculus GN=Tomm34 - [TOM34_MOUSE]	32.36	1	1	7	21	1.04	0.97	0.93	0.98	0.95	1.10	1.05	1.09	1.05	0.96	0.93	0.99	0.96	34.26	9.07
P62869	Transcription elongation factor B polypeptide 2 OS=Mus musculus GN=Tceb2 PE=1 SV=1 - [ELOB_MOUSE]	84.75	1	8	8	27	1.01	0.91	0.86	0.96	0.95	1.05	1.10	1.09	1.04	0.99	0.93	1.00	1.04	13.16	5.01
E9Q6U4	Protein Pfdn4 OS=Mus musculus GN=Pfdn4 PE=2 SV=1 - [E9Q6U4_MOUSE]	40.00	5	6	6	34	1.01	0.82	0.82	0.89	0.85	1.12	1.09	1.09	1.05	1.00	0.97	1.00	0.90	14.72	4.50
Q9DA03	Complex III assembly factor LYRM7 OS=Mus musculus GN=Lyrm7 PE=2 SV=2 - [LYRM7_MOUSE]	25.96	1	2	2	3	0.94	0.78	0.83	0.86	0.92	0.95	1.01	1.09	1.16	0.97	1.04	0.89	0.95	12.04	9.45

B1AVF2	TSC22 domain family protein 3 OS=Mus musculus GN=Tsc22d3 PE=2 SV=1 - [B1AVF2_MOUSE]	45.13	4	2	4	21	1.02	0.81	0.77	0.86	0.86	1.08	1.06	1.09	1.09	0.95	0.96	0.91	0.92	12.55	4.68
Q7TME0	Lipid phosphate phosphatase-related protein type 4 OS=Mus musculus GN=Lppr4 PE=1 SV=2 - [LPPR4_MOUSE]	27.15	1	16	16	70	1.02	1.14	1.12	1.03	1.03	1.11	1.08	1.09	1.09	1.05	1.04	1.06	1.05	83.24	8.84
Q8K2F8	Protein LSM14 homolog A OS=Mus musculus GN=Lsm14a PE=1 SV=1 - [LS14A_MOUSE]	20.56	3	10	10	16	1.09	0.95	1.04	0.98	0.92	1.00	0.97	1.09	1.05	1.03	0.95	0.90	0.98	50.52	9.52
Q61016	Guanine nucleotide-binding protein G(I)/G(S)/G(O) subunit gamma-7 OS=Mus musculus GN=Gng7 PE=2 SV=2 - [GBG7_MOUSE]	57.35	3	3	3	28	0.98	1.10	1.13	0.94	0.98	1.15	1.14	1.09	1.14	1.25	1.26	1.41	1.41	7.47	8.51
P61089	Ubiquitin-conjugating enzyme E2 N OS=Mus musculus GN=Ube2n PE=1 SV=1 - [UBE2N_MOUSE]	55.26	2	8	8	33	0.94	0.71	0.77	0.95	1.00	0.94	1.02	1.09	1.06	1.00	0.99	0.88	0.90	17.13	6.57
Q6GV12	3-ketodihydrosphingosine reductase OS=Mus musculus GN=Kdsr PE=2 SV=1 - [KDSR_MOUSE]	4.22	1	1	1	2	0.95	1.35	1.41	1.02	1.07	1.01	1.05	1.09	1.14	0.91	0.96	1.08	1.14	35.93	7.90
Q8BH27	Multiple epidermal growth factor-like domains protein 9 OS=Mus musculus GN=Megf9 PE=2 SV=1 - [MEGF9_MOUSE]	8.17	1	5	5	13	1.09	0.94	0.85	1.15	1.07	1.24	1.09	1.09	1.01	1.09	0.97	1.04	0.89	62.79	5.47
F6ZQA3	Protein Numa1 (Fragment) OS=Mus musculus GN=Numa1 PE=2 SV=1 - [F6ZQA3_MOUSE]	31.85	1	1	15	31	0.84	1.00	1.18	0.93	1.10	0.97	1.14	1.09	1.29	0.99	1.17	1.08	1.29	82.83	9.44
O35075	Down syndrome critical region protein 3 homolog OS=Mus musculus GN=Dscr3 PE=2 SV=1 - [DSCR3_MOUSE]	5.72	1	1	1	4	1.20	1.42	1.18	1.25	1.04	1.22	1.01	1.09	0.91	1.13	0.94	1.31	1.10	32.95	7.68
F8WI35	Histone H3 OS=Mus musculus GN=H3f3a PE=2 SV=1 - [F8WI35_MOUSE]	36.30	6	1	8	56	1.02	0.81	0.79	1.06	1.04	1.08	1.06	1.09	1.07	1.17	1.15	1.11	1.09	15.19	11.40
Q9CQX2	Cytochrome b5 type B OS=Mus musculus GN=Cyb5b PE=1 SV=1 - [CYB5B_MOUSE]	41.10	1	4	4	42	1.06	1.10	1.06	1.03	0.88	1.13	1.07	1.09	1.06	0.93	0.95	1.03	1.04	16.31	4.89
P05555-2	Isoform 2 of Integrin alpha-M OS=Mus musculus GN=Itgam - [ITAM_MOUSE]	2.80	7	2	2	3	1.08	1.25	1.16	0.95	0.89	0.92	0.85	1.09	1.01	1.40	1.30	1.22	1.14	115.12	7.64

O35607	Bone morphogenetic protein receptor type-2 OS=Mus musculus GN=Bmpr2 PE=2 SV=1 - [BMPR2_MOUSE]	16.09	1	12	12	27	1.10	1.16	1.09	1.03	0.99	1.01	1.04	1.09	1.02	1.05	1.04	1.05	0.99	114.95	6.23
Q9CQZ1	Heat shock factor-binding protein 1 OS=Mus musculus GN=Hsbp1 PE=2 SV=1 - [HSBP1_MOUSE]	72.37	1	5	5	26	1.00	0.60	0.59	0.90	1.02	1.07	1.06	1.09	1.08	1.05	1.10	0.83	0.93	8.61	4.31
Q9DB29	Isoamyl acetate-hydrolyzing esterase 1 homolog OS=Mus musculus GN=Iah1 PE=2 SV=1 - [IAH1_MOUSE]	16.47	1	3	3	12	0.92	1.05	1.11	1.02	1.15	1.00	1.17	1.09	1.28	1.02	1.19	1.19	1.39	27.96	5.52
Q6PCZ4	Melanoma-associated antigen E1 OS=Mus musculus GN=Magee1 PE=1 SV=1 - [MAGE1_MOUSE]	4.79	1	3	3	10	1.13	1.15	0.91	1.00	0.88	1.04	0.97	1.09	1.06	1.10	0.98	1.02	1.02	101.57	6.67
Q9D164-2	Isoform 2 of FXYD domain-containing ion transport regulator 6 OS=Mus musculus GN=Fxyd6 - [FXYD6_MOUSE]	26.88	2	2	2	10	1.43	0.90	0.66	0.93	0.70	1.31	0.91	1.09	0.72	1.05	0.87	1.10	0.78	10.30	5.11
Q9JLV1	BAG family molecular chaperone regulator 3 OS=Mus musculus GN=Bag3 PE=1 SV=2 - [BAG3_MOUSE]	26.69	1	9	9	24	0.96	0.82	0.80	0.88	0.94	1.10	1.23	1.09	1.16	0.94	0.98	0.91	0.88	61.82	7.27
P51163	Uroporphyrinogen-III synthase OS=Mus musculus GN=Uros PE=2 SV=1 - [HEM4_MOUSE]	23.40	2	4	4	7	0.90	0.87	0.96	0.82	0.93	0.91	0.97	1.09	1.21	0.92	1.02	0.92	1.02	28.49	6.54
Q61036	Serine/threonine-protein kinase PAK 3 OS=Mus musculus GN=Pak3 PE=1 SV=2 - [PAK3_MOUSE]	30.05	5	5	14	70	1.09	0.92	0.85	0.97	0.87	1.09	1.04	1.09	1.00	1.04	0.96	1.03	0.96	62.36	5.40
P15116	Cadherin-2 OS=Mus musculus GN=Cdh2 PE=1 SV=2 - [CADH2_MOUSE]	33.33	2	17	18	84	1.03	0.99	0.94	0.99	0.93	1.10	1.02	1.09	1.03	1.06	0.98	1.07	1.02	99.73	4.78
P62484	Abl interactor 2 OS=Mus musculus GN=Abi2 PE=1 SV=1 - [ABI2_MOUSE]	32.51	1	7	11	43	0.97	0.99	0.95	0.96	1.01	1.11	1.17	1.09	1.12	1.06	1.10	0.94	0.99	49.36	6.01
Q9CR98	Protein FAM136A OS=Mus musculus GN=Fam136a PE=1 SV=1 - [F136A_MOUSE]	53.62	2	7	7	43	1.06	0.87	0.84	0.95	0.90	1.10	1.03	1.09	1.02	1.10	1.02	1.03	0.98	15.66	7.61
Q9CXW2	28S ribosomal protein S22, mitochondrial OS=Mus musculus GN=Mrps22 PE=2 SV=1 - [RT22_MOUSE]	14.76	1	4	4	5	0.94	1.04	0.99	0.90	0.98	0.93	0.97	1.09	0.96	0.91	1.00	1.02	0.89	41.17	8.56

D3YW10	Ubiquitin-conjugating enzyme E2 E2 (Fragment) OS=Mus musculus GN=Ube2e2 PE=2 SV=1 - [D3YW10_MOUSE]	23.15	10	2	2	6	0.93	0.92	0.96	1.04	1.12	1.01	1.09	1.09	1.12	0.95	1.00	1.06	1.14	11.63	5.07
Q9D6T9	28S ribosomal protein S36, mitochondrial OS=Mus musculus GN=Mrps36 PE=4 SV=1 - [Q9D6T9_MOUSE]	56.00	4	6	6	73	0.99	0.69	0.72	0.90	0.92	1.13	1.14	1.09	1.09	0.95	1.00	0.87	0.84	10.94	9.99
Q78F42	Protein Zfp324 OS=Mus musculus GN=Zfp324 PE=2 SV=1 - [Q78F42_MOUSE]	5.83	1	1	2	2	1.07	0.89	0.83	0.97	0.91	1.07	1.00	1.09	1.02	1.01	0.95	0.88	0.83	64.32	9.55
P47955	60S acidic ribosomal protein P1 OS=Mus musculus GN=Rplp1 PE=1 SV=1 - [RLA1_MOUSE]	66.67	2	3	3	31	0.84	0.80	0.90	0.88	0.96	1.28	1.31	1.09	1.22	0.97	1.10	1.05	1.12	11.47	4.32
Q8R3V6	CUE domain-containing protein 1 OS=Mus musculus GN=Cuedc1 PE=1 SV=2 - [CUED1_MOUSE]	15.46	6	4	4	10	1.02	0.93	0.89	0.95	0.93	1.18	1.18	1.09	1.13	1.03	1.10	1.10	1.05	42.75	5.58
Q9CRC6	UPF0693 protein C10orf32 homolog OS=Mus musculus PE=1 SV=1 - [CJ032_MOUSE]	60.95	1	4	4	18	1.06	0.99	0.91	0.93	0.85	1.22	1.18	1.09	1.15	1.12	1.17	1.12	1.10	11.54	6.79
Q9DCX2	ATP synthase subunit d, mitochondrial OS=Mus musculus GN=Atp5h PE=1 SV=3 - [ATP5H_MOUSE]	89.44	3	17	19	259	0.96	0.77	0.80	0.87	0.91	1.10	1.16	1.09	1.17	0.99	1.07	0.96	1.01	18.74	5.69
Q8VI59	Pecanex-like protein 3 OS=Mus musculus GN=Pcnxl3 PE=1 SV=2 - [PCX3_MOUSE]	0.94	1	1	1	1	4.37	56.42	12.88	2.57	0.59	2.53	0.58	1.09	0.25	1.00	0.23	1.12	0.26	221.43	6.62
Q6ZWQ5	Sorting nexin 12, isoform CRA_c OS=Mus musculus GN=Snx12 PE=2 SV=1 - [Q6ZWQ5_MOUSE]	61.11	4	8	9	31	0.94	0.96	1.07	0.98	1.04	1.04	1.07	1.09	1.18	0.99	1.08	1.04	1.13	18.87	8.44
G3UXB9	T-cell leukemia homeobox protein 1 OS=Mus musculus GN=Tlx1 PE=2 SV=1 - [G3UXB9_MOUSE]	10.99	4	1	1	1	0.35	0.78	2.22	0.81	2.31	1.07	3.03	1.09	3.10	1.28	3.65	1.93	5.50	10.47	8.00
Q80XP9-2	Isoform 2 of Serine/threonine-protein kinase WNK3 OS=Mus musculus GN=Wnk3 - [WNK3_MOUSE]	6.32	5	5	8	14	1.13	0.84	0.84	0.95	0.93	1.05	1.02	1.09	1.08	1.07	1.01	1.02	1.04	188.31	5.62
H3BKL6	cTAGE family member 5 OS=Mus musculus GN=Ctage5 PE=2 SV=1 - [H3BKL6_MOUSE]	10.27	14	6	6	10	1.06	1.09	1.07	1.13	1.10	1.07	1.11	1.09	1.15	1.09	1.13	1.00	1.07	79.35	5.21

Q61581	Insulin-like growth factor-binding protein 7 OS=Mus musculus GN=Igfbp7 PE=2 SV=3 - [IBP7_MOUSE]	3.20	3	1	1	1	0.83	0.57	0.69	0.79	0.95	0.67	0.80	1.09	1.31	1.04	1.25	0.89	1.07	28.95	8.31
Q9CRB6	Tubulin polymerization-promoting protein family member 3 OS=Mus musculus GN=Tppp3 PE=1 SV=1 - [TPPP3_MOUSE]	53.41	1	12	12	52	1.24	0.84	0.67	0.91	0.72	1.18	0.96	1.09	0.86	1.22	0.99	1.14	0.92	18.95	9.11
Q8VCD6	Receptor expression-enhancing protein 2 OS=Mus musculus GN=Reep2 PE=2 SV=2 - [REEP2_MOUSE]	27.17	1	6	6	13	0.82	1.11	1.32	1.03	1.19	1.09	1.26	1.09	1.31	1.10	1.35	1.02	1.24	28.42	9.41
P09103	Protein disulfide-isomerase OS=Mus musculus GN=P4hb PE=1 SV=2 - [PDIA1_MOUSE]	69.55	3	33	33	288	1.00	0.81	0.78	0.90	0.90	1.10	1.09	1.09	1.07	0.99	1.01	0.94	0.94	57.02	4.88
Q8C0Q2	Zinc fingers and homeoboxes protein 3 OS=Mus musculus GN=Zhx3 PE=2 SV=2 - [ZHX3_MOUSE]	7.89	1	5	5	10	1.01	0.99	1.03	0.95	0.93	1.11	1.09	1.09	1.09	1.03	0.99	1.03	1.07	104.28	6.57
Q9Z2M7	Phosphomannomutase 2 OS=Mus musculus GN=Pmm2 PE=2 SV=1 - [PMM2_MOUSE]	29.75	1	5	6	18	0.97	0.94	0.96	0.99	1.05	1.14	1.16	1.09	1.11	1.09	1.12	1.15	1.19	27.64	6.42
A2A690	Protein TANC2 OS=Mus musculus GN=Tanc2 PE=1 SV=1 - [TANC2_MOUSE]	10.48	4	14	14	38	0.96	1.17	1.21	1.01	1.07	1.09	1.09	1.09	1.12	1.11	1.14	1.10	1.14	220.13	7.97
P55284	Cadherin-5 OS=Mus musculus GN=Cdh5 PE=1 SV=2 - [CADH5_MOUSE]	3.19	1	2	2	2	0.95	1.18	1.25	0.96	1.02	0.79	0.84	1.09	1.15	0.94	0.99	0.63	0.67	87.85	5.30
F6YRW4	F-box and leucine-rich repeat protein 11 OS=Mus musculus GN=Kdm2a PE=4 SV=2 - [F6YRW4_MOUSE]	2.84	3	2	2	2	0.94	0.92	0.98	1.10	1.17	1.02	1.08	1.09	1.15	1.08	1.15	0.96	1.01	132.58	7.52
Q6PCN3	Tau-tubulin kinase 1 OS=Mus musculus GN=Ttbk1 PE=2 SV=3 - [TTBK1_MOUSE]	5.73	1	4	4	7	0.91	0.88	0.86	1.05	1.17	1.16	1.30	1.09	1.16	1.02	1.19	1.10	1.14	141.53	5.62
Q6P2K6-2	Isoform 2 of Serine/threonine-protein phosphatase 4 regulatory subunit 3A OS=Mus musculus GN=Smek1 - [P4R3A_MOUSE]	4.25	3	1	1	1	0.95	0.80	0.83	0.90	0.95	0.92	0.96	1.09	1.15	1.01	1.07	0.92	0.97	29.16	4.87
Q6PDX6-4	Isoform 4 of E3 ubiquitin-protein ligase Rnf220 OS=Mus musculus GN=Rnf220 - [RN220_MOUSE]	40.00	5	8	8	22	1.08	0.98	1.01	0.95	0.93	1.14	1.05	1.09	1.02	0.94	0.94	0.97	0.95	27.47	4.96

Q9CPT3	N-acylneuraminate-9-phosphatase OS=Mus musculus GN=Nanp PE=1 SV=1 - [NANP_MOUSE]	7.26	1	1	1	2	1.22	0.99	0.81	1.27	1.04	0.98	0.80	1.09	0.90	1.02	0.84	1.18	0.97	27.79	6.07
E9Q6H8	Protein Plekha5 OS=Mus musculus GN=Plekha5 PE=2 SV=1 - [E9Q6H8_MOUSE]	7.17	1	6	6	15	0.98	1.02	1.06	1.09	1.13	1.02	1.08	1.09	1.13	1.01	1.11	1.07	1.16	144.26	7.39
P62761	Visinin-like protein 1 OS=Mus musculus GN=Vsnl1 PE=1 SV=2 - [VISL1_MOUSE]	76.96	1	13	16	439	1.06	0.87	0.84	0.97	0.92	1.07	1.03	1.09	1.04	0.98	0.92	1.00	0.93	22.13	5.15
G3UWW1	Microtubule-associated protein (Fragment) OS=Mus musculus GN=Map2 PE=2 SV=1 - [G3UWW1_MOUSE]	52.08	1	2	20	737	1.10	1.09	0.99	0.88	0.80	1.09	0.99	1.10	0.99	0.98	0.90	0.80	0.73	36.61	10.42
Q60989	E3 ubiquitin-protein ligase XIAP OS=Mus musculus GN=Xiap PE=1 SV=2 - [XIAP_MOUSE]	12.70	2	4	4	8	1.00	0.76	0.75	0.88	0.87	1.03	0.99	1.10	1.00	1.00	1.03	0.93	1.01	56.04	6.23
Q9Z0F8	Disintegrin and metalloproteinase domain-containing protein 17 OS=Mus musculus GN=Adam17 PE=1 SV=3 - [ADA17_MOUSE]	3.02	2	2	2	3	1.03	0.99	0.95	1.00	0.96	1.05	1.01	1.10	1.06	0.99	0.96	0.88	0.85	93.00	5.94
P70671	Interferon regulatory factor 3 OS=Mus musculus GN=Irf3 PE=1 SV=1 - [IRF3_MOUSE]	3.34	1	1	1	2	1.25	1.78	1.42	1.26	1.01	1.11	0.89	1.10	0.88	1.23	0.99	1.49	1.20	46.82	5.43
P15066	Transcription factor jun-D OS=Mus musculus GN=Jund PE=1 SV=1 - [JUND_MOUSE]	14.66	1	3	4	8	0.98	0.85	0.81	0.91	0.86	1.10	1.08	1.10	1.12	1.03	0.99	1.14	1.09	34.88	7.44
Q9CR50	RING finger and CHY zinc finger domain-containing protein 1 OS=Mus musculus GN=Rchy1 PE=1 SV=1 - [ZN363_MOUSE]	6.51	2	2	2	4	1.04	0.69	0.71	0.82	0.85	1.07	1.09	1.10	1.02	0.91	0.93	0.90	0.93	29.99	7.18
Q9D8S9	BolA-like protein 1 OS=Mus musculus GN=Bola1 PE=1 SV=1 - [BOLA1_MOUSE]	48.91	1	4	4	22	1.02	0.93	0.89	0.93	0.90	1.13	1.13	1.10	1.13	1.02	1.01	0.93	0.91	14.37	8.76
Q80W00-	Isoform 2 of Serine/threonine-protein phosphatase 1 regulatory subunit 10 OS=Mus musculus GN=Ppp1r10 - [PP1RA_MOUSE]	13.27	2	8	8	11	0.99	0.92	0.90	0.93	0.90	1.11	1.10	1.10	1.10	1.05	0.99	0.95	0.96	92.99	9.16

P10637-5	Isoform Tau-D of Microtubule-associated protein tau OS=Mus musculus GN=Mapt - [TAU_MOUSE]	77.69	6	1	31	707	1.09	1.12	1.07	0.85	0.85	1.21	1.13	1.10	1.02	0.93	0.87	1.09	1.00	38.94	9.50
Q9CVD2	Ataxin-3 OS=Mus musculus GN=Atxn3 PE=1 SV=2 - [ATX3_MOUSE]	25.07	5	7	7	25	0.96	0.94	1.02	0.90	0.96	1.00	1.08	1.10	1.09	1.04	1.05	1.07	1.03	40.51	4.83
Q60598	Src substrate cortactin OS=Mus musculus GN=Cttn PE=1 SV=2 - [SRC8_MOUSE]	43.96	2	22	22	224	1.02	0.83	0.84	0.93	0.92	1.13	1.08	1.10	1.09	1.02	0.99	0.94	0.93	61.21	5.40
E9QAD1	Nuclear receptor-interacting protein 2 (Fragment) OS=Mus musculus GN=Nrip2 PE=2 SV=1 - [E9QAD1_MOUSE]	20.09	4	3	3	8	1.34	1.14	0.95	0.93	0.74	1.21	0.94	1.10	0.94	1.34	1.05	1.04	0.86	24.01	8.10
F7A8R0	N-alpha-acetyltransferase 35, NatC auxiliary subunit (Fragment) OS=Mus musculus GN=Naa35 PE=2 SV=1 - [F7A8R0_MOUSE]	6.97	5	2	2	3	1.19	1.24	1.04	1.18	0.99	1.03	0.86	1.10	0.92	1.13	0.95	1.25	1.05	45.39	5.36
P80560	Receptor-type tyrosine-protein phosphatase N2 OS=Mus musculus GN=Ptprn2 PE=1 SV=2 - [PTPR2_MOUSE]	18.18	1	14	14	62	1.20	1.03	0.79	1.04	0.81	1.15	0.93	1.10	0.89	1.15	0.92	1.16	0.94	111.43	5.95
Q9CR29	Coiled-coil domain-containing protein 43 OS=Mus musculus GN=Ccdc43 PE=1 SV=1 - [CCD43_MOUSE]	35.14	3	9	9	28	1.21	0.78	0.70	1.01	0.81	1.20	0.98	1.10	0.89	1.07	0.89	1.12	0.83	25.03	4.97
P16092-6	Isoform 6 of Fibroblast growth factor receptor 1 OS=Mus musculus GN=Fgfr1 - [FGFR1_MOUSE]	2.07	53	1	2	6	1.13	1.17	1.04	1.01	0.89	1.10	0.97	1.10	0.97	1.08	0.96	1.06	0.95	91.61	6.21
Q8R0A6	V-set and transmembrane domain-containing protein 2A OS=Mus musculus GN=Vstm2a PE=2 SV=2 - [VTM2A_MOUSE]	18.22	2	2	2	3	1.10	0.73	0.85	0.97	0.91	1.28	1.15	1.10	1.02	0.85	0.78	0.89	0.87	25.86	8.27
B1AQX6	SRC kinase-signaling inhibitor 1 OS=Mus musculus GN=Srcin1 PE=2 SV=1 - [B1AQX6_MOUSE]	48.72	4	2	43	157	1.09	1.30	1.19	1.14	1.04	1.10	1.00	1.10	1.01	0.90	0.83	0.92	0.84	126.91	9.36
E9PXF0	Protein Pcdh17 OS=Mus musculus GN=Pcdh17 PE=2 SV=1 - [E9PXF0_MOUSE]	12.45	1	9	9	21	1.17	1.08	0.89	1.05	0.88	1.13	0.92	1.10	0.93	1.25	1.07	1.24	1.00	126.07	5.21

Q3TGF2	Protein FAM107B OS=Mus musculus GN=Fam107b PE=2 SV=2 - [F107B_MOUSE]	28.24	5	3	4	8	1.14	0.96	0.83	0.87	0.74	1.15	1.02	1.10	0.93	1.08	0.95	0.92	0.80	15.56	8.31
Q8VD12-	Isoform 2 of Zinc finger protein 385A OS=Mus musculus GN=Znf385a - [Z385A_MOUSE]	4.37	2	1	1	2	1.09	1.29	1.18	1.23	1.13	1.01	0.92	1.10	1.00	1.00	0.91	0.85	0.78	38.31	9.74
E0CXA7	Inhibitor of growth protein 2 (Fragment) OS=Mus musculus GN=Ing2 PE=2 SV=1 - [E0CXA7_MOUSE]	11.72	3	1	1	2	1.03	1.12	1.09	1.17	1.14	1.15	1.11	1.10	1.07	1.23	1.20	1.28	1.25	16.78	7.59
Q8R1C0	Potassium voltage-gated channel subfamily C member 4 OS=Mus musculus GN=Kcnc4 PE=2 SV=1 - [KCNC4_MOUSE]	8.28	1	2	3	4	1.03	1.31	1.27	1.25	1.21	1.00	0.96	1.10	1.06	1.18	1.14	1.02	0.99	68.61	7.05
Q9JKS4-3	Isoform 3 of LIM domain-binding protein 3 OS=Mus musculus GN=Ldb3 - [LDB3_MOUSE]	14.37	7	7	7	13	0.94	1.05	1.10	0.98	0.98	1.86	2.03	1.10	1.03	1.05	1.21	0.99	1.00	70.71	7.96
Q78HU3	Multivesicular body subunit 12A OS=Mus musculus GN=Mvb12a PE=1 SV=1 - [MB12A_MOUSE]	3.69	1	1	1	2	0.89	0.73	0.81	0.96	1.07	0.98	1.09	1.10	1.23	0.93	1.05	0.92	1.04	28.69	9.11
Q5XG69	Soluble lamin-associated protein of 75 kDa OS=Mus musculus GN=Fam169a PE=1 SV=3 - [F169A_MOUSE]	15.64	1	8	8	11	0.98	1.00	1.02	1.05	0.94	1.01	0.90	1.10	1.09	1.03	1.09	1.01	0.98	73.19	4.68
P21995	Embigin OS=Mus musculus GN=Emb PE=1 SV=2 - [EMB_MOUSE]	9.39	1	3	3	6	1.00	1.38	1.31	1.16	1.16	1.14	1.07	1.10	1.17	1.16	1.27	1.41	1.43	37.04	6.02
G5E843	Roundabout homolog 1 OS=Mus musculus GN=Robo1 PE=4 SV=1 - [G5E843_MOUSE]	7.88	2	8	9	18	1.09	1.16	1.01	0.97	0.86	1.17	1.06	1.10	1.00	1.15	1.12	1.25	1.12	176.27	6.19
P60867	40S ribosomal protein S20 OS=Mus musculus GN=Rps20 PE=1 SV=1 - [RS20_MOUSE]	19.33	1	2	2	14	1.07	1.14	1.06	0.95	0.99	1.11	1.07	1.10	1.02	1.03	1.02	0.96	0.98	13.36	9.94
P97315	Cysteine and glycine-rich protein 1 OS=Mus musculus GN=Csrp1 PE=1 SV=3 - [CSRP1_MOUSE]	67.88	1	13	13	325	1.06	0.84	0.77	0.91	0.85	1.13	1.05	1.10	1.03	0.98	0.95	0.94	0.90	20.57	8.57
Q3UFK8	FERM domain-containing protein 8 OS=Mus musculus GN=Frmd8 PE=1 SV=2 - [FRMD8_MOUSE]	4.29	1	2	2	3	1.00	1.06	1.06	0.98	0.98	1.01	1.00	1.10	1.10	1.03	1.03	1.18	1.18	51.80	6.44

A2AQT8	Methylmalonic aciduria and homocystinuria type D homolog, mitochondrial (Fragment) OS=Mus musculus GN=Mmadhc PE=2 SV=1 - [A2AQT8_MOUSE]	7.82	3	1	1	2	0.89	1.06	1.19	0.90	1.00	1.05	1.17	1.10	1.23	1.11	1.25	0.84	0.94	19.79	5.19
O88532	Zinc finger RNA-binding protein OS=Mus musculus GN=Zfr PE=1 SV=2 - [ZFR_MOUSE]	21.23	5	15	15	34	0.94	0.97	1.08	1.04	1.07	1.02	1.11	1.10	1.18	1.02	1.03	0.99	1.04	116.79	9.04
Q6IRU5	Clathrin light chain B OS=Mus musculus GN=Cltb PE=2 SV=1 - [CLCB_MOUSE]	43.67	3	1	14	314	1.00	0.77	0.77	0.90	0.91	1.12	1.10	1.10	1.10	0.98	1.03	0.89	0.90	25.16	4.63
E9QP99	Golgin subfamily A member 3 OS=Mus musculus GN=Golga3 PE=2 SV=1 - [E9QP99_MOUSE]	39.41	4	43	43	120	1.03	0.93	0.93	0.91	0.88	1.03	0.99	1.10	1.07	1.01	0.93	0.93	0.91	167.19	5.38
Q8BGT7	Survival of motor neuron-related-splicing factor 30 OS=Mus musculus GN=Smndc1 PE=2 SV=1 - [SPF30_MOUSE]	21.85	1	3	3	8	1.11	0.73	0.61	1.04	0.80	1.15	1.09	1.10	1.02	0.94	0.99	1.00	0.81	26.74	7.24
Q60648	Ganglioside GM2 activator OS=Mus musculus GN=Gm2a PE=1 SV=2 - [SAP3_MOUSE]	24.35	1	4	4	5	1.02	0.91	0.82	0.94	0.91	1.18	1.11	1.10	1.01	0.98	1.02	0.90	0.88	20.81	5.90
A2A7B5	Protein Prdm2 OS=Mus musculus GN=Prdm2 PE=2 SV=1 - [A2A7B5_MOUSE]	2.40	1	2	2	3	1.13	1.26	1.12	1.06	0.94	1.06	0.94	1.10	0.97	1.08	0.96	1.07	0.95	187.09	8.00
O35972	39S ribosomal protein L23, mitochondrial OS=Mus musculus GN=Mrpl23 PE=2 SV=1 - [RM23_MOUSE]	29.45	1	3	3	5	0.94	1.25	1.33	1.14	1.21	1.03	1.09	1.10	1.17	1.10	1.22	1.12	1.20	17.11	9.76
Q9D832	DnaJ homolog subfamily B member 4 OS=Mus musculus GN=Dnajb4 PE=2 SV=1 - [DNJB4_MOUSE]	27.89	1	7	8	15	1.12	0.92	0.83	0.98	0.92	1.14	0.98	1.10	1.02	1.03	0.97	0.97	0.93	37.76	8.59
Q8C7E9	Cleavage stimulation factor subunit 2 tau variant OS=Mus musculus GN=Cstf2t PE=1 SV=2 - [CSTFT_MOUSE]	19.94	1	1	8	26	0.83	0.94	1.14	0.85	1.03	1.06	1.27	1.10	1.32	0.97	1.18	0.88	1.07	65.82	7.25
G5E8J8	MCG14253, isoform CRA_a OS=Mus musculus GN=Wiz PE=4 SV=1 - [G5E8J8_MOUSE]	1.68	5	1	1	2	1.12	1.31	1.17	0.97	0.86	1.08	0.96	1.10	0.98	1.11	0.99	1.19	1.06	102.87	9.01
Q99M07	Cytochrome c oxidase assembly factor 5 OS=Mus musculus GN=Coa5 PE=2 SV=1 - [COA5_MOUSE]	13.51	1	1	1	2	1.04	0.63	0.61	1.05	1.01	1.09	1.05	1.10	1.06	1.10	1.06	0.96	0.93	8.35	8.70

Q923W1	Trimethylguanosine synthase OS=Mus musculus GN=Tgs1 PE=1 SV=2 - [TGS1_MOUSE]	2.11	2	2	2	3	1.13	1.25	1.11	1.03	0.91	1.06	0.93	1.10	0.97	1.20	1.07	1.24	1.10	96.73	4.97
Q9D3D9	ATP synthase subunit delta, mitochondrial OS=Mus musculus GN=Atp5d PE=1 SV=1 - [ATPD_MOUSE]	58.33	2	4	4	244	0.96	0.95	0.99	0.90	0.94	1.10	1.15	1.10	1.17	1.03	1.05	0.96	1.00	17.59	5.08
Q3UUF8	Ankyrin repeat domain-containing protein 34B OS=Mus musculus GN=Ankrd34b PE=1 SV=1 - [AN34B_MOUSE]	6.30	1	3	3	7	1.07	0.86	0.78	1.03	0.94	1.04	0.96	1.10	1.00	1.10	1.02	1.20	1.10	55.38	8.02
Q8BTJ4-2	Isoform 2 of Bis(5'-adenosyl)-triphosphatase enpp4 OS=Mus musculus GN=Enpp4 - [ENPP4_MOUSE]	9.46	4	3	4	8	1.00	0.85	0.85	0.81	0.81	0.80	0.79	1.10	1.09	0.70	0.70	0.98	0.98	47.87	6.73
Q8BUR3-	Isoform 2 of Forkhead box protein J3 OS=Mus musculus GN=Foxj3 - [FOXJ3_MOUSE]	5.94	2	3	3	6	0.96	0.85	0.87	0.80	0.98	1.04	1.19	1.10	1.17	0.90	1.07	0.90	1.04	65.49	7.17
Q7TNG8	Probable D-lactate dehydrogenase, mitochondrial OS=Mus musculus GN=Ldhd PE=1 SV=1 - [LDHD_MOUSE]	3.10	1	1	1	2	0.92	1.77	1.91	1.61	1.74	1.17	1.25	1.10	1.18	1.48	1.60	1.09	1.18	51.82	6.62
Q9WUD1	STIP1 homology and U box-containing protein 1 OS=Mus musculus GN=Stub1 PE=1 SV=1 - [CHIP_MOUSE]	63.49	1	16	16	47	0.90	0.81	0.97	0.84	0.92	1.07	1.19	1.10	1.20	0.97	1.03	0.91	1.00	34.89	6.01
Q91Z69	SLIT-ROBO Rho GTPase-activating protein 1 OS=Mus musculus GN=Srgap1 PE=1 SV=2 - [SRGP1_MOUSE]	7.44	3	1	7	9	0.72	0.95	1.31	0.98	1.36	1.16	1.61	1.10	1.52	1.06	1.48	0.97	1.35	121.35	6.74
D3Z3D9	Beta-chimaerin OS=Mus musculus GN=Chn2 PE=2 SV=1 - [D3Z3D9_MOUSE]	8.67	2	1	1	2	0.89	1.23	1.37	1.11	1.23	1.28	1.43	1.10	1.22	1.17	1.31	1.12	1.25	22.36	8.37
Q3UHJ1	TNF receptor-associated factor 3 OS=Mus musculus GN=Traf3 PE=2 SV=1 - [Q3UHJ1_MOUSE]	10.52	3	5	5	11	1.16	1.00	0.96	1.05	0.85	1.04	0.83	1.10	0.88	1.13	0.97	1.13	1.05	61.52	8.03
P04370-4	Isoform 4 of Myelin basic protein OS=Mus musculus GN=Mbp - [MBP_MOUSE]	68.72	5	2	22	1095	0.94	0.84	0.90	1.03	1.09	1.23	1.31	1.10	1.21	1.11	1.19	1.14	1.23	21.49	11.24
Q99JF8	PC4 and SFRS1-interacting protein OS=Mus musculus GN=Psip1 PE=1 SV=1 - [PSIP1_MOUSE]	47.92	3	4	24	152	1.03	0.97	0.86	1.11	1.01	1.13	1.00	1.10	1.11	1.17	1.04	1.13	1.11	59.66	9.13

Q9CPW4	Actin-related protein 2/3 complex subunit 5 OS=Mus musculus GN=Arpc5 PE=2 SV=3 - [ARPC5_MOUSE]	52.32	2	8	8	38	1.10	1.02	0.93	1.05	1.01	1.22	1.07	1.10	0.99	1.12	1.04	1.05	1.00	16.28	5.67
Q9CQP3	Coiled-coil-helix-coiled-coil-helix domain-containing protein 5 OS=Mus musculus GN=Chchd5 PE=2 SV=1 - [CHCH5_MOUSE]	23.64	1	2	2	6	1.11	0.90	0.71	1.05	0.84	1.10	1.01	1.10	0.99	0.99	0.87	0.97	0.85	12.32	7.31
Q9CZ83	39S ribosomal protein L55, mitochondrial OS=Mus musculus GN=Mrpl55 PE=2 SV=1 - [RM55_MOUSE]	24.41	2	2	2	19	1.03	1.25	1.08	0.96	0.96	1.03	0.95	1.10	0.96	0.96	0.99	1.07	1.03	15.11	10.51
O70251	Elongation factor 1-beta OS=Mus musculus GN=Eef1b PE=1 SV=5 - [EF1B_MOUSE]	67.11	5	11	13	210	1.00	0.81	0.87	0.90	0.94	1.14	1.16	1.10	1.13	1.02	1.02	0.90	0.97	24.68	4.69
Q60575-2	Isoform 2 of Kinesin-like protein KIF1B OS=Mus musculus GN=Kif1b - [KIF1B_MOUSE]	7.06	6	2	10	25	1.10	1.37	1.24	0.96	0.87	1.13	1.02	1.10	1.00	1.11	1.01	1.07	0.97	198.80	5.59
G3UX26	Voltage-dependent anion-selective channel protein 2 (Fragment) OS=Mus musculus GN=Vdac2 PE=4 SV=1 - [G3UX26_MOUSE]	61.48	4	12	12	134	1.07	1.09	1.03	1.21	1.15	1.14	1.07	1.10	1.05	1.15	1.10	1.24	1.13	30.43	7.58
G3X9N3	MCG16539 OS=Mus musculus GN=Pnmal2 PE=4 SV=1 - [G3X9N3_MOUSE]	21.33	1	11	11	25	1.05	1.04	0.83	1.10	0.97	1.17	1.01	1.10	1.11	1.11	1.04	1.12	1.08	71.71	5.17
Q6WKZ8	E3 ubiquitin-protein ligase UBR2 OS=Mus musculus GN=Ubr2 PE=1 SV=2 - [UBR2_MOUSE]	0.74	2	1	1	2	0.87	0.95	1.10	1.13	1.30	0.84	0.96	1.10	1.26	0.88	1.01	1.15	1.33	199.03	6.33
B0QZH9	PHD finger protein 23 (Fragment) OS=Mus musculus GN=Phf23 PE=2 SV=1 - [B0QZH9_MOUSE]	6.74	4	1	1	2	1.11	1.19	1.07	1.09	0.98	1.10	0.98	1.10	0.99	1.25	1.13	1.33	1.20	20.86	10.08
Q9DAU1	Protein canopy homolog 3 OS=Mus musculus GN=Cnpy3 PE=1 SV=1 - [CNPY3_MOUSE]	38.41	3	9	9	44	1.06	0.85	0.90	0.97	0.92	1.12	1.07	1.10	1.05	1.03	0.95	0.92	0.87	30.52	5.62
B7ZC84	Serine/threonine-protein kinase tousled-like 2 (Fragment) OS=Mus musculus GN=Tlk2 PE=2 SV=1 - [B7ZC84_MOUSE]	8.60	6	1	1	1	1.00	0.90	0.90	0.99	0.99	1.06	1.06	1.10	1.10	1.04	1.04	1.08	1.08	10.38	7.34

Q9DB60	Prostamide/prostaglandin F synthase OS=Mus musculus GN=Fam213b PE=1 SV=1 - [PGFS_MOUSE]	16.42	2	3	3	5	0.59	0.99	1.68	0.85	1.13	0.83	1.00	1.10	0.95	1.08	1.10	0.93	1.26	21.66	6.74
Q60772	Cyclin-dependent kinase 4 inhibitor C OS=Mus musculus GN=Cdkn2c PE=2 SV=1 - [CDN2C_MOUSE]	7.74	1	1	1	2	1.11	0.89	0.80	0.73	0.65	1.22	1.09	1.10	0.98	1.12	1.01	0.92	0.83	18.06	6.00
P62855	40S ribosomal protein S26 OS=Mus musculus GN=Rps26 PE=2 SV=3 - [RS26_MOUSE]	20.87	1	2	2	8	1.03	1.10	1.07	0.99	1.14	1.08	1.20	1.10	1.08	1.10	1.07	0.99	1.09	13.01	11.00
Q9CQ56-2	Isoform 2 of Vesicle transport protein USE1 OS=Mus musculus GN=Use1 - [USE1_MOUSE]	12.11	3	2	2	4	0.86	1.09	1.26	1.00	1.15	1.03	1.18	1.10	1.27	1.17	1.35	1.06	1.23	21.46	9.54
P21661	Neuroendocrine convertase 2 OS=Mus musculus GN=Pcsk2 PE=2 SV=1 - [NEC2_MOUSE]	14.76	1	7	7	19	1.00	0.92	0.92	0.98	0.99	1.04	1.04	1.10	1.05	0.99	1.03	0.96	0.91	70.74	6.42
E9PUH0	Protein A430005L14Rik OS=Mus musculus GN=A430005L14Rik PE=2 SV=1 - [E9PUH0_MOUSE]	17.71	2	3	3	5	0.98	0.93	0.95	1.06	0.98	1.31	1.21	1.10	1.29	0.87	1.00	0.91	0.97	20.90	5.15
Q9JKV5	Secretory carrier-associated membrane protein 4 OS=Mus musculus GN=Scamp4 PE=2 SV=1 - [SCAM4_MOUSE]	4.78	1	1	1	4	1.18	1.77	1.50	1.41	1.19	1.18	1.00	1.10	0.93	1.11	0.95	1.02	0.87	25.33	8.66
Q99PL6	UBX domain-containing protein 6 OS=Mus musculus GN=Ubxn6 PE=1 SV=1 - [UBXN6_MOUSE]	26.02	3	10	10	26	0.96	1.01	1.00	0.97	1.07	1.13	1.13	1.10	1.18	1.10	1.22	1.06	1.11	49.77	8.54
A2BDX3	Adenylyltransferase and sulfurtransferase MOCS3 OS=Mus musculus GN=Mocs3 PE=2 SV=1 - [MOCS3_MOUSE]	3.48	1	1	1	2	1.02	1.11	1.09	1.13	1.11	0.85	0.84	1.10	1.08	0.93	0.91	1.17	1.15	49.34	7.49
E9Q389	Gametogenetin-binding protein 2 (Fragment) OS=Mus musculus GN=Ggnbp2 PE=2 SV=1 - [E9Q389_MOUSE]	11.90	6	1	1	7	0.96	1.21	1.24	1.09	1.19	0.91	1.03	1.10	1.13	0.87	0.91	1.12	1.22	9.64	5.08
Q9D7S9	Charged multivesicular body protein 5 OS=Mus musculus GN=Chmp5 PE=2 SV=1 - [CHMP5_MOUSE]	40.64	1	6	6	21	1.07	0.90	0.84	0.94	0.87	1.20	1.10	1.10	1.00	1.04	1.02	0.99	0.95	24.56	4.79

G3UZJ2	Microtubule-associated protein (Fragment) OS=Mus musculus GN=Map2 PE=2 SV=1 - [G3UZJ2_MOUSE]	73.86	1	2	22	748	1.20	0.94	0.88	1.06	0.82	1.13	0.93	1.10	0.93	1.07	0.86	1.05	0.89	25.74	11.09
Q80T85	DDB1- and CUL4-associated factor 5 OS=Mus musculus GN=Dcaf5 PE=1 SV=2 - [DCAF5_MOUSE]	2.96	1	2	2	6	0.92	0.90	1.07	0.87	1.01	1.09	1.15	1.10	1.30	0.92	1.03	0.79	0.83	103.61	5.80
Q8BKI2	Trinucleotide repeat-containing gene 6B protein OS=Mus musculus GN=Tnrc6b PE=2 SV=2 - [TNR6B_MOUSE]	20.17	2	22	22	69	1.01	0.94	0.91	0.96	0.97	1.07	1.06	1.10	1.03	0.99	0.99	0.94	0.95	191.85	6.30
O88271	Craniofacial development protein 1 OS=Mus musculus GN=Cfdp1 PE=1 SV=1 - [CFDP1_MOUSE]	35.59	1	10	10	33	1.03	0.83	0.79	1.02	0.99	1.14	1.10	1.10	1.11	1.06	1.00	1.06	1.01	32.90	4.86
Q5F2E7	Nuclear fragile X mental retardation-interacting protein 2 OS=Mus musculus GN=Nufip2 PE=1 SV=1 - [NUFP2_MOUSE]	29.91	2	13	13	39	0.98	0.75	0.80	0.92	0.94	1.15	1.17	1.10	1.13	1.02	0.98	0.87	0.92	75.61	8.70
A2A5R2	Brefeldin A-inhibited guanine nucleotide-exchange protein 2 OS=Mus musculus GN=Arfgef2 PE=1 SV=1 - [BIG2_MOUSE]	9.26	4	9	9	15	0.96	1.34	1.42	1.29	1.33	0.95	0.99	1.10	1.14	1.14	1.22	1.25	1.29	202.11	6.55
Q8VCI5	Peroxisomal biogenesis factor 19 OS=Mus musculus GN=Pex19 PE=1 SV=1 - [PEX19_MOUSE]	47.83	2	9	9	44	1.02	0.99	0.86	0.93	0.90	1.14	1.11	1.10	1.05	0.97	0.97	0.93	0.87	32.71	4.34
H7BWY8	Pre-rRNA-processing protein TSR2 homolog OS=Mus musculus GN=Tsr2 PE=2 SV=1 - [H7BWY8_MOUSE]	46.15	3	6	6	64	1.35	0.88	0.69	0.97	0.76	1.18	0.87	1.10	0.83	1.08	0.83	1.10	0.88	21.37	4.26
A0A4W9	Neuronal growth regulator 1 OS=Mus musculus GN=Negr1 PE=2 SV=1 - [A0A4W9_MOUSE]	51.37	4	13	13	195	1.01	1.08	1.06	0.97	0.96	1.08	1.07	1.10	1.09	1.07	1.03	1.08	1.06	36.10	6.86
Q9QUN3	B-cell linker protein OS=Mus musculus GN=Blnk PE=1 SV=1 - [BLNK_MOUSE]	10.72	2	2	2	8	0.92	0.91	1.09	1.05	1.01	1.15	1.25	1.10	1.34	1.07	1.30	0.92	1.01	50.64	8.25
Q80WW9	DDRGK domain-containing protein 1 OS=Mus musculus GN=Ddrgk1 PE=1 SV=2 - [DDRGK_MOUSE]	5.40	1	1	1	2	1.15	1.11	0.97	0.91	0.79	1.34	1.16	1.10	0.96	1.28	1.11	1.45	1.27	35.96	5.35

B2RXC2	Inositol 1,4,5-trisphosphate 3-kinase B OS=Mus musculus GN=Itpkb PE=2 SV=1 - [B2RXC2_MOUSE]	3.08	1	1	2	5	1.28	1.06	0.83	0.97	0.76	1.10	0.86	1.10	0.86	1.13	0.88	1.16	0.91	102.61	8.00
F6W8I0	YjeF N-terminal domain-containing protein 3 OS=Mus musculus GN=Yjefn3 PE=2 SV=2 - [YJEN3_MOUSE]	3.59	1	1	1	1	0.97	1.03	1.05	1.02	1.05	1.09	1.12	1.10	1.13	1.04	1.07	1.18	1.21	27.00	7.40
Q03717	Potassium voltage-gated channel subfamily B member 1 OS=Mus musculus GN=Kcnb1 PE=1 SV=2 - [KCNB1_MOUSE]	13.54	1	6	6	13	0.99	1.29	1.30	0.97	1.05	1.07	1.07	1.10	1.14	0.93	1.00	0.93	0.95	95.53	8.16
Q8BTG7-2	Isoform 2 of Protein NDRG4 OS=Mus musculus GN=Ndrg4 - [NDRG4_MOUSE]	31.56	1	1	8	19	1.02	1.21	1.18	1.07	1.05	0.92	0.90	1.10	1.08	1.18	1.16	1.31	1.29	37.14	5.90
Q61686	Chromobox protein homolog 5 OS=Mus musculus GN=Cbx5 PE=1 SV=1 - [CBX5_MOUSE]	50.79	2	10	11	58	1.06	0.78	0.74	0.90	0.82	1.14	1.04	1.10	1.01	1.06	1.01	0.93	0.89	22.17	5.86
H3BLN5	Interleukin-33 (Fragment) OS=Mus musculus GN=Il33 PE=2 SV=1 - [H3BLN5_MOUSE]	6.58	2	1	1	1	1.08	1.12	1.04	0.84	0.78	1.13	1.04	1.10	1.02	1.09	1.01	1.29	1.20	25.63	9.07
Q6PGF2	MORN repeat-containing protein 4 OS=Mus musculus GN=Morn4 PE=2 SV=1 - [MORN4_MOUSE]	15.75	1	2	2	3	0.90	0.85	0.95	0.87	0.97	1.07	1.19	1.10	1.23	0.90	1.01	0.95	1.07	16.20	8.15
Q9DAR0	Uncharacterized protein C5orf49 homolog OS=Mus musculus PE=1 SV=1 - [CE049_MOUSE]	20.88	1	3	3	6	1.04	0.91	0.89	0.93	0.89	1.11	1.03	1.10	1.03	1.03	0.96	1.12	1.00	20.84	6.89
G3X922	MCG115602 OS=Mus musculus GN=Dnajc13 PE=4 SV=1 - [G3X922_MOUSE]	1.83	1	2	2	4	0.73	1.12	1.53	1.21	1.66	0.93	1.27	1.10	1.51	1.08	1.48	1.07	1.47	254.28	6.77
Q60932-2	Isoform Mt-VDAC1 of Voltage-dependent anion-selective channel protein 1 OS=Mus musculus GN=Vdac1 - [VDAC1_MOUSE]	80.21	3	18	19	454	1.03	1.01	0.98	1.15	1.09	1.14	1.08	1.10	1.05	1.14	1.09	1.12	1.10	30.74	8.54
Q99KH8	Serine/threonine-protein kinase 24 OS=Mus musculus GN=Stk24 PE=1 SV=1 - [STK24_MOUSE]	38.52	1	9	13	30	0.91	1.03	1.07	0.80	0.87	1.05	1.14	1.10	1.30	1.01	1.14	0.93	1.15	47.92	5.43
Q5QNQ6	Oxysterol-binding protein 2 OS=Mus musculus GN=Osbp2 PE=2 SV=1 - [OSBP2_MOUSE]	11.78	4	4	7	11	0.91	0.91	1.00	0.99	0.96	0.99	1.02	1.10	0.97	1.04	1.04	0.84	1.03	101.29	7.14

O35639	Annexin A3 OS=Mus musculus GN=Anxa3 PE=1 SV=4 - [ANXA3_MOUSE]	29.10	1	6	7	19	1.00	1.00	0.98	1.06	1.08	1.04	1.04	1.10	1.09	1.17	1.17	1.08	1.08	36.36	5.76
Q8R4I7	Neuropilin and tolloid-like protein 1 OS=Mus musculus GN=Neto1 PE=1 SV=2 - [NETO1_MOUSE]	5.82	1	2	2	3	1.06	0.91	0.86	0.86	0.81	1.11	1.04	1.10	1.04	1.01	0.96	0.99	0.93	60.20	6.99
Q8CE50	Sorting nexin-30 OS=Mus musculus GN=Snx30 PE=2 SV=1 - [SNX30_MOUSE]	11.21	1	5	5	8	1.05	1.17	1.14	1.05	1.02	0.95	0.91	1.10	1.03	0.99	0.97	1.15	1.08	49.49	5.35
E1U8D0	Protein SOGA1 OS=Mus musculus GN=Soga1 PE=1 SV=3 - [SOGA1_MOUSE]	3.46	2	2	4	12	0.92	1.14	1.25	0.94	1.04	1.15	1.24	1.10	1.28	1.28	1.28	1.23	1.34	159.09	6.46
B7ZNL3	Tpm1 protein OS=Mus musculus GN=Tpm1 PE=2 SV=1 - [B7ZNL3_MOUSE]	60.56	2	4	22	314	1.02	0.91	0.89	0.92	0.86	0.85	0.84	1.10	1.06	1.11	1.13	0.81	0.78	32.62	4.74
P50586-2	Isoform Short of Tubby protein OS=Mus musculus GN=Tub - [TUB_MOUSE]	11.58	2	2	2	4	0.91	0.88	0.97	1.03	1.13	0.96	1.05	1.10	1.21	0.86	0.95	1.00	1.02	49.53	9.41
Q8BH95	Enoyl-CoA hydratase, mitochondrial OS=Mus musculus GN=Echs1 PE=1 SV=1 - [ECHM_MOUSE]	39.66	2	10	10	34	1.01	0.85	0.83	1.01	1.03	1.13	1.17	1.10	1.12	1.08	1.09	1.08	1.09	31.45	8.48
Q99JP7	Gamma-glutamyltransferase 7 OS=Mus musculus GN=Ggt7 PE=1 SV=2 - [GGT7_MOUSE]	26.89	3	12	12	39	1.01	1.06	1.02	1.01	0.97	1.00	1.00	1.10	1.05	1.03	0.98	1.02	1.00	70.21	5.06
Q5UAK0-	Isoform 4 of Mesoderm induction early response protein 1 OS=Mus musculus GN=Mier1 - [MIER1_MOUSE]	5.49	5	2	2	4	1.29	1.04	0.81	0.95	0.74	1.12	0.87	1.10	0.85	0.93	0.76	1.02	0.88	57.79	4.46
Q9QYX7	Protein piccolo OS=Mus musculus GN=Pclo PE=1 SV=4 - [PCLO_MOUSE]	46.05	2	174	182	946	1.00	0.87	0.88	0.97	0.98	1.08	1.08	1.10	1.11	1.01	1.01	0.90	0.91	550.50	6.51
Q8BLD4	MCG16669, isoform CRA_f OS=Mus musculus GN=Tardbp PE=2 SV=1 - [Q8BLD4_MOUSE]	26.51	4	1	6	11	0.98	1.22	1.24	0.96	0.97	0.97	0.98	1.10	1.12	1.14	1.16	1.10	1.12	33.57	7.06
Q61315-4	Isoform 4 of Adenomatous polyposis coli protein OS=Mus musculus GN=Apc - [APC_MOUSE]	13.69	8	26	27	59	1.03	0.98	1.00	1.00	0.97	1.05	1.02	1.10	1.01	1.00	0.96	1.04	0.95	296.29	7.61
Q99N13-2	Isoform 2 of Histone deacetylase 9 OS=Mus musculus GN=Hdac9 - [HDAC9_MOUSE]	6.99	3	2	2	3	0.96	1.25	1.30	0.91	0.95	1.16	1.21	1.10	1.15	1.20	1.25	1.21	1.26	60.57	7.11
Q6IRU2	Tropomyosin alpha-4 chain OS=Mus musculus GN=Tpm4 PE=2 SV=3 - [TPM4_MOUSE]	55.65	1	14	17	96	1.04	0.81	0.79	0.90	0.92	1.14	1.10	1.10	1.06	1.02	0.99	0.93	0.92	28.45	4.68

Q01815-2	Isoform 2 of Voltage-dependent L-type calcium channel subunit alpha-1C OS=Mus musculus GN=Cacna1c - [CAC1C_MOUSE]	6.58	10	8	8	24	1.14	1.15	1.09	1.04	1.01	1.08	0.93	1.10	1.12	1.09	0.99	1.04	0.92	238.79	6.93
F7BX42	Neuronal pentraxin receptor OS=Mus musculus GN=Nptxr PE=2 SV=1 - [F7BX42_MOUSE]	40.57	4	14	14	52	1.14	1.06	0.90	0.96	0.80	1.05	0.89	1.10	0.85	1.04	0.86	1.13	0.92	52.28	6.13
Q924S8	Sprouty-related, EVH1 domain-containing protein 1 OS=Mus musculus GN=Spred1 PE=1 SV=1 - [SPRE1_MOUSE]	22.75	2	7	8	26	1.10	0.92	0.85	1.07	0.99	1.02	0.89	1.10	1.00	1.00	0.89	0.93	0.89	50.63	6.47
Q60805	Tyrosine-protein kinase Mer OS=Mus musculus GN=Mertk PE=1 SV=1 - [MERTK_MOUSE]	5.13	1	3	4	7	1.35	0.90	0.65	1.14	0.98	1.02	0.87	1.10	0.83	0.87	0.84	1.18	0.87	110.09	5.50
Q91X97	Neurocalcin-delta OS=Mus musculus GN=Ncald PE=1 SV=4 - [NCALD_MOUSE]	74.09	4	7	14	320	1.18	0.92	0.78	0.97	0.83	1.14	0.95	1.10	0.87	1.01	0.84	1.09	0.93	22.23	5.35
Q7TMI3-3	Isoform 3 of E3 ubiquitin-protein ligase UHRF2 OS=Mus musculus GN=Uhrf2 - [UHRF2_MOUSE]	6.02	3	1	1	2	0.89	1.04	1.16	1.04	1.17	1.03	1.15	1.10	1.23	1.07	1.20	1.24	1.40	24.12	7.12
Q8CHS8-2	Isoform 2 of Vacuolar protein sorting-associated protein 37A OS=Mus musculus GN=Vps37a - [VP37A_MOUSE]	8.96	3	2	2	3	0.88	1.14	1.29	0.77	0.87	1.02	1.14	1.11	1.25	1.05	1.19	0.95	1.08	38.43	5.64
Q8BFX1	E3 ubiquitin-protein ligase RNF187 OS=Mus musculus GN=Rnf187 PE=1 SV=2 - [RN187_MOUSE]	39.83	1	6	6	12	0.96	0.87	0.90	0.96	0.96	1.08	1.05	1.11	1.10	0.96	0.98	0.88	0.91	26.30	5.59
B2RTJ2	BTB/POZ domain-containing protein KCTD21 OS=Mus musculus GN=Kctd21 PE=2 SV=1 - [B2RTJ2_MOUSE]	6.54	2	1	1	4	0.96	1.00	1.04	0.94	0.98	0.92	0.96	1.11	1.15	1.06	1.11	1.04	1.08	29.64	6.79
Q3UHD9-	Isoform 2 of Arf-GAP with GTPase, ANK repeat and PH domain-containing protein 2 OS=Mus musculus GN=Agap2 - [AGAP2_MOUSE]	14.58	2	11	11	24	0.97	1.29	1.27	1.17	1.20	0.95	1.01	1.11	1.04	0.98	1.06	1.08	1.11	122.03	9.77
Q8K298	Actin-binding protein anillin OS=Mus musculus GN=Anln PE=1 SV=2 - [ANLN_MOUSE]	8.30	1	9	9	28	0.91	0.96	0.94	0.90	0.96	1.15	1.27	1.11	1.22	1.10	1.14	1.08	1.20	122.72	6.98

Q91VN6	Probable ATP-dependent RNA helicase DDX41 OS=Mus musculus GN=Ddx41 PE=1 SV=2 - [DDX41_MOUSE]	1.45	1	1	1	2	0.99	0.93	0.94	0.88	0.89	1.00	1.00	1.11	1.11	1.08	1.09	1.10	1.11	69.77	6.70
O35126	Atrophin-1 OS=Mus musculus GN=Atn1 PE=1 SV=1 - [ATN1_MOUSE]	5.53	2	3	3	9	0.99	0.89	0.93	0.89	1.00	1.12	1.16	1.11	1.18	1.02	0.97	0.98	1.03	123.65	8.95
Q8K274	Ketosamine-3-kinase OS=Mus musculus GN=Fn3krp PE=2 SV=2 - [KT3K_MOUSE]	18.77	1	5	5	14	1.04	1.09	1.01	1.10	1.04	1.12	1.06	1.11	1.10	1.19	1.19	1.11	1.10	34.45	7.87
A2AL79	Aspartyl/asparaginyl beta-hydroxylase OS=Mus musculus GN=Asph PE=2 SV=1 - [A2AL79_MOUSE]	14.21	8	1	2	7	0.83	0.76	0.91	0.89	1.07	1.13	1.35	1.11	1.32	1.14	1.37	0.88	1.06	20.71	4.39
O88983	Syntaxin-8 OS=Mus musculus GN=Stx8 PE=1 SV=1 - [STX8_MOUSE]	17.37	1	3	3	6	1.21	1.18	1.17	1.14	0.96	1.34	1.09	1.11	0.90	1.15	0.95	1.14	0.95	26.91	5.01
A9C437	Chloride channel protein 2 OS=Mus musculus GN=Clcn2 PE=2 SV=1 - [A9C437_MOUSE]	2.24	2	2	2	2	0.98	1.35	1.38	1.18	1.21	0.99	1.01	1.11	1.13	1.00	1.02	0.91	0.93	97.64	8.56
Q0KL01	UBX domain-containing protein 2B OS=Mus musculus GN=Ubxn2b PE=1 SV=2 - [UBX2B_MOUSE]	61.93	1	13	13	41	1.01	0.89	0.82	0.91	0.91	1.11	1.06	1.11	1.10	1.03	1.01	1.00	0.94	37.42	5.94
P55200-2	Isoform 2 of Histone-lysine N-methyltransferase 2A OS=Mus musculus GN=Kmt2a - [KMT2A_MOUSE]	0.45	2	1	1	2	1.21	1.15	0.95	1.22	1.01	1.37	1.13	1.11	0.91	1.38	1.14	1.39	1.15	429.10	9.16
A2A4A1	DnaJ homolog subfamily C member 24 OS=Mus musculus GN=Dnajc24 PE=2 SV=1 - [A2A4A1_MOUSE]	36.49	3	5	5	10	1.03	0.95	0.86	0.96	0.87	1.04	1.04	1.11	1.12	1.08	1.08	0.95	0.93	16.91	4.92
E9PUD6	Protein Zscan18 OS=Mus musculus GN=Zscan18 PE=4 SV=1 - [E9PUD6_MOUSE]	14.09	1	6	6	9	1.18	0.99	0.87	1.02	0.94	1.20	1.02	1.11	0.94	1.09	1.01	1.19	1.07	91.69	4.35
Q64012-2	Isoform 1 of RNA-binding protein Raly OS=Mus musculus GN=Raly - [RALY_MOUSE]	32.77	5	6	6	12	0.93	0.86	0.88	1.06	1.10	1.16	1.06	1.11	1.17	1.10	1.11	1.03	1.04	31.18	9.38
Q8BPB5	EGF-containing fibulin-like extracellular matrix protein 1 OS=Mus musculus GN=Efemp1 PE=2 SV=1 - [FBLN3_MOUSE]	6.49	1	1	1	6	1.01	1.42	1.40	1.23	1.22	1.26	1.24	1.11	1.09	1.26	1.25	1.22	1.22	54.92	5.14

P56873	Sjoegren syndrome/scleroderma autoantigen 1 homolog OS=Mus musculus GN=Sssca1 PE=2 SV=1 - [SSA27_MOUSE]	46.23	2	4	4	8	0.92	0.93	1.25	0.93	1.01	1.17	1.37	1.11	1.20	1.17	1.40	1.18	1.34	21.32	5.12
Q9DC04-1	Isoform 5 of Regulator of G-protein signaling 3 OS=Mus musculus GN=Rgs3 - [RGS3_MOUSE]	2.99	3	1	1	2	0.99	0.89	0.89	0.85	0.85	1.12	1.12	1.11	1.11	1.09	1.10	1.24	1.24	61.52	4.94
P58774-2	Isoform 2 of Tropomyosin beta chain OS=Mus musculus GN=Tpm2 - [TPM2_MOUSE]	58.80	6	3	21	219	1.06	0.87	0.82	0.92	0.82	0.91	0.90	1.11	1.04	1.14	1.08	0.90	0.85	32.94	4.67
P60824	Cold-inducible RNA-binding protein OS=Mus musculus GN=Cirbp PE=1 SV=1 - [CIRBP_MOUSE]	43.60	3	6	6	41	1.00	0.78	0.79	0.88	0.85	1.31	1.30	1.11	1.12	1.04	0.99	0.99	0.99	18.60	9.61
A2AWT5	Nucleolar transcription factor 1 OS=Mus musculus GN=Ubtf PE=4 SV=1 - [A2AWT5_MOUSE]	26.83	7	20	20	39	1.06	0.99	0.94	1.01	0.99	1.14	1.11	1.11	1.06	1.04	0.99	1.05	0.93	89.42	5.81
E9QPX3	NADH dehydrogenase [ubiquinone] iron-sulfur protein 4, mitochondrial OS=Mus musculus GN=Ndufs4 PE=4 SV=1 - [E9QPX3_MOUSE]	55.43	2	10	10	134	1.03	0.82	0.82	0.92	0.93	1.11	1.11	1.11	1.06	1.02	0.99	0.94	0.93	19.79	9.99
Q9CPR7	Suppressor of IKBKE 1 OS=Mus musculus GN=Sike1 PE=2 SV=1 - [SIKE1_MOUSE]	51.69	2	8	8	23	0.95	0.73	0.82	0.90	0.93	1.15	1.20	1.11	1.14	1.02	1.07	0.96	1.01	23.51	5.38
Q9ER00	Syntaxin-12 OS=Mus musculus GN=Stx12 PE=1 SV=1 - [STX12_MOUSE]	37.96	1	8	8	35	1.06	1.03	1.00	1.00	0.93	1.11	1.02	1.11	1.05	1.05	1.03	1.12	1.00	31.18	5.44
Q6KCD5-	Isoform 4 of Nipped-B-like protein OS=Mus musculus GN=Nipbl - [NIPBL_MOUSE]	6.48	3	1	1	4	0.95	0.96	1.01	0.86	0.91	1.07	1.13	1.11	1.17	0.92	0.98	0.90	0.95	32.72	5.77
Q80ZP8	Armet protein OS=Mus musculus GN=Manf PE=2 SV=1 - [Q80ZP8_MOUSE]	55.15	7	10	10	62	1.04	0.72	0.68	0.94	0.93	1.12	1.08	1.11	1.08	1.05	1.03	0.92	0.90	19.00	8.13
P70302	Stromal interaction molecule 1 OS=Mus musculus GN=Stim1 PE=1 SV=2 - [STIM1_MOUSE]	11.82	1	6	6	12	1.07	0.93	0.88	1.06	1.02	0.96	0.93	1.11	1.05	1.00	0.89	1.03	0.91	77.52	6.54

P24668	Cation-dependent mannose-6-phosphate receptor OS=Mus musculus GN=M6pr PE=1 SV=1 - [MPRD_MOUSE]	23.02	1	5	5	14	1.08	1.16	1.08	1.17	1.06	1.01	0.94	1.11	1.02	0.98	1.01	1.12	1.07	31.15	5.39
Q8C4G9	MCG21623, isoform CRA_b OS=Mus musculus GN=Gpr123 PE=2 SV=1 - [Q8C4G9_MOUSE]	6.06	1	2	2	3	1.03	1.10	1.06	1.01	0.98	1.13	1.08	1.11	1.07	1.09	1.05	1.15	1.12	63.46	7.43
Q80Y14	Glutaredoxin-related protein 5, mitochondrial OS=Mus musculus GN=Glrx5 PE=2 SV=2 - [GLRX5_MOUSE]	29.61	1	3	3	26	1.00	0.93	0.91	0.96	0.90	1.11	1.06	1.11	1.14	1.00	1.01	0.97	0.98	16.28	6.55
Q9QYE5	Protein jagged-2 OS=Mus musculus GN=Jag2 PE=2 SV=2 - [JAG2_MOUSE]	2.81	1	2	2	3	1.41	0.95	0.67	1.13	0.81	1.18	0.84	1.11	0.79	1.23	0.88	1.15	0.82	134.65	5.88
Q9CYN9	Renin receptor OS=Mus musculus GN=Atp6ap2 PE=2 SV=2 - [RENR_MOUSE]	13.43	1	3	3	6	0.98	1.43	1.29	1.28	1.40	1.04	1.10	1.11	1.13	1.06	1.16	1.24	1.27	39.07	5.54
O70176	Pituitary adenylate cyclase-activating polypeptide OS=Mus musculus GN=Adcyap1 PE=2 SV=1 - [PACA_MOUSE]	13.14	1	2	2	7	1.97	1.37	0.75	0.94	0.49	1.31	0.66	1.11	0.60	2.06	1.04	2.06	1.04	19.37	9.23
Q8C845	EF-hand domain-containing protein D2 OS=Mus musculus GN=Efhd2 PE=2 SV=1 - [Q8C845_MOUSE]	60.00	2	15	18	149	1.05	1.02	0.99	0.93	0.89	1.02	0.99	1.11	1.03	0.92	0.88	0.90	0.88	26.78	5.14
Q9WTR5	Cadherin-13 OS=Mus musculus GN=Cdh13 PE=1 SV=2 - [CAD13_MOUSE]	24.93	1	15	15	71	1.13	1.02	0.89	0.96	0.83	1.17	1.02	1.11	0.93	1.12	1.01	1.27	1.04	78.14	5.12
E9Q2N1	Bromodomain and WD repeat-containing protein 1 (Fragment) OS=Mus musculus GN=Brwd1 PE=2 SV=1 - [E9Q2N1_MOUSE]	2.31	4	3	3	5	1.07	0.95	0.87	0.88	0.83	1.01	0.94	1.11	1.06	1.01	0.95	1.03	0.96	294.50	8.28
Q3UDE2	Tubulin--tyrosine ligase-like protein 12 OS=Mus musculus GN=Ttll12 PE=1 SV=1 - [TTL12_MOUSE]	4.23	1	2	2	4	1.10	1.16	1.16	1.11	1.31	0.89	1.06	1.11	1.11	1.24	1.13	1.15	1.20	74.00	5.63
D3Z3F8	Spartin OS=Mus musculus GN=Spg20 PE=2 SV=1 - [D3Z3F8_MOUSE]	9.79	5	4	4	10	1.27	0.97	0.77	1.05	0.85	1.35	1.19	1.11	0.86	1.09	0.91	1.20	0.98	63.04	5.58

Q8BJM3	R3H and coiled-coil domain-containing protein 1-like OS=Mus musculus GN=R3hcc1l PE=2 SV=1 - [R3HCL_MOUSE]	5.81	2	3	3	4	0.98	1.15	1.11	0.92	0.97	1.04	1.20	1.11	1.21	1.18	1.25	0.92	0.94	84.43	4.87
Q8C5P7	Testis development-related protein OS=Mus musculus GN=Tdrp PE=2 SV=1 - [TDRP_MOUSE]	58.79	3	8	8	24	1.07	1.01	0.95	0.89	0.87	1.12	1.11	1.11	1.08	1.15	1.07	1.10	1.13	20.17	5.73
Q8BI72	CDKN2A-interacting protein OS=Mus musculus GN=Cdkn2aip PE=2 SV=1 - [CARF_MOUSE]	3.55	1	1	1	2	1.05	1.23	1.18	0.81	0.77	1.00	0.95	1.11	1.05	0.91	0.87	1.20	1.15	59.71	9.16
P57722-2	Isoform 2 of Poly(rC)-binding protein 3 OS=Mus musculus GN=Pcbp3 - [PCBP3_MOUSE]	34.86	5	4	9	35	1.01	1.11	0.99	0.96	1.00	1.02	1.11	1.11	1.14	0.88	1.10	1.10	1.14	39.14	7.52
Q99KR7	Peptidyl-prolyl cis-trans isomerase F, mitochondrial OS=Mus musculus GN=Ppif PE=1 SV=1 - [PPIF_MOUSE]	53.40	1	6	8	29	0.99	0.89	0.87	0.86	0.88	1.08	1.06	1.11	1.15	0.98	1.05	0.93	0.93	21.72	9.16
Q8CGA4	UPF0452 protein C7orf41 homolog OS=Mus musculus PE=2 SV=1 - [CG041_MOUSE]	19.08	1	2	2	8	1.03	1.11	1.22	0.92	1.00	0.97	0.92	1.11	1.07	1.00	1.04	1.13	1.12	14.83	4.17
Q9Z0P4	Paralemmin-1 OS=Mus musculus GN=Palm PE=1 SV=1 - [PALM_MOUSE]	71.80	1	5	28	304	0.96	1.09	1.02	0.87	0.95	1.12	1.16	1.11	1.18	1.02	1.08	0.95	1.03	41.59	4.84
Q9DBX2	Phosducin-like protein OS=Mus musculus GN=Pdcl PE=1 SV=1 - [PHLP_MOUSE]	16.61	2	3	3	3	1.02	1.04	1.04	0.91	0.88	1.14	1.14	1.11	1.09	1.07	1.05	1.13	1.11	34.38	4.87
Q9QZS3-[illegible]	Isoform 4 of Protein numb homolog OS=Mus musculus GN=Numb - [NUMB_MOUSE]	24.28	10	10	12	44	0.96	0.97	1.04	0.90	0.94	1.06	1.13	1.11	1.15	1.01	1.04	0.94	0.98	64.35	8.47
B2RVL6	Zinc finger CCHC domain-containing protein 24 OS=Mus musculus GN=Zcchc24 PE=2 SV=1 - [ZCH24_MOUSE]	11.20	1	2	2	3	1.03	0.98	0.95	0.99	0.97	1.01	0.98	1.11	1.08	1.12	1.09	1.04	1.01	26.94	8.70
Q80X80	C2 domain-containing protein 2-like OS=Mus musculus GN=C2cd2l PE=1 SV=3 - [C2C2L_MOUSE]	24.50	1	10	10	26	1.03	1.00	0.96	1.21	1.15	1.20	1.14	1.11	1.03	1.22	1.18	1.16	1.15	76.28	7.21
Q9JK53	Prolargin OS=Mus musculus GN=Prelp PE=2 SV=2 - [PRELP_MOUSE]	6.88	1	2	2	3	1.09	0.93	0.77	0.91	0.84	0.65	0.60	1.11	0.91	0.81	0.75	0.97	0.89	43.27	9.54

Q504N4	Deoxyguanosine kinase, isoform CRA_c OS=Mus musculus GN=Dguok PE=2 SV=1 - [Q504N4_MOUSE]	10.59	3	1	1	2	1.47	1.25	0.85	1.21	0.82	1.25	0.85	1.11	0.75	1.11	0.76	1.56	1.06	27.54	9.16
Q6PER3	Microtubule-associated protein RP/EB family member 3 OS=Mus musculus GN=Mapre3 PE=1 SV=1 - [MARE3_MOUSE]	37.72	2	1	10	54	0.97	0.87	0.87	1.02	1.08	1.01	1.09	1.11	1.20	0.97	1.01	0.96	1.08	31.95	5.54
Q6ZQ18-2	Isoform 2 of Protein EFR3 homolog B OS=Mus musculus GN=Efr3b - [EFR3B_MOUSE]	9.70	3	5	6	15	0.87	1.13	1.28	1.07	1.22	1.09	1.23	1.11	1.19	1.05	1.20	1.24	1.39	87.46	6.68
E9Q160	Protein Tro OS=Mus musculus GN=Tro PE=2 SV=1 - [E9Q160_MOUSE]	2.62	6	1	2	4	1.00	0.84	0.84	0.96	0.95	1.07	1.07	1.11	1.10	1.04	1.04	0.98	0.98	87.26	10.10
Q9JLR1	Protein transport protein Sec61 subunit alpha isoform 2 OS=Mus musculus GN=Sec61a2 PE=2 SV=3 - [S61A2_MOUSE]	5.67	2	1	2	2	1.31	2.66	2.03	1.44	1.10	0.69	0.53	1.11	0.85	1.04	0.80	0.66	0.51	52.21	8.06
E9QQ33	E3 ubiquitin-protein ligase SH3RF1 OS=Mus musculus GN=Sh3rf1 PE=4 SV=1 - [E9QQ33_MOUSE]	11.67	6	4	6	8	1.15	1.09	1.04	0.98	1.00	1.17	1.15	1.11	1.08	1.19	1.07	1.09	1.05	93.29	8.48
O35633-2	Isoform 2 of Vesicular inhibitory amino acid transporter OS=Mus musculus GN=Slc32a1 - [VIAAT_MOUSE]	23.03	2	6	7	114	1.13	1.07	1.02	1.00	0.89	1.12	0.97	1.11	0.97	1.15	1.04	1.13	0.98	56.81	6.90
Q9CPQ1	Cytochrome c oxidase subunit 6C OS=Mus musculus GN=Cox6c PE=1 SV=3 - [COX6C_MOUSE]	50.00	2	4	4	14	1.02	1.13	1.15	1.03	1.08	1.29	1.10	1.11	1.06	1.17	1.06	1.09	1.11	8.46	10.14
Q6NSW3-	Isoform 4 of A-kinase anchor protein SPHKAP OS=Mus musculus GN=Sphkap - [SPKAP_MOUSE]	4.67	5	6	6	10	1.04	1.06	1.04	1.02	0.97	1.06	0.96	1.11	1.06	1.07	0.98	1.13	1.12	173.24	5.08
Q9D0J8	Parathymosin OS=Mus musculus GN=Ptms PE=2 SV=3 - [PTMS_MOUSE]	23.76	1	3	3	186	0.99	0.80	0.81	0.92	0.94	1.14	1.14	1.11	1.12	1.00	1.02	0.92	0.95	11.42	4.22
E9QNR5	Thrombospondin type-1 domain-containing protein 7A OS=Mus musculus GN=Thsd7a PE=2 SV=1 - [E9QNR5_MOUSE]	10.64	6	13	13	24	1.02	1.02	1.02	1.07	1.04	1.07	1.09	1.11	1.09	1.08	1.09	1.14	1.21	183.33	7.20
Q8CBW3-	Isoform 5 of Abl interactor 1 OS=Mus musculus GN=Abi1 - [ABI1_MOUSE]	42.01	11	12	16	69	0.97	0.84	0.84	0.95	0.97	1.10	1.13	1.11	1.17	1.05	1.06	0.97	1.00	42.52	6.20

P70182-2	Isoform 2 of Phosphatidylinositol 4-phosphate 5-kinase type-1 alpha OS=Mus musculus GN=Pip5k1a - [PI51A_MOUSE]	11.08	7	2	3	18	1.02	1.09	1.07	0.98	0.96	1.12	1.37	1.11	1.09	1.05	1.03	1.01	1.00	38.00	8.47
Q69ZS7	HBS1-like protein OS=Mus musculus GN=Hbs1l PE=1 SV=2 - [HBS1L_MOUSE]	18.62	4	4	9	21	1.19	1.25	1.27	1.19	0.96	1.09	0.84	1.11	0.87	1.14	0.83	1.24	1.17	75.05	6.46
Q9R099	Transducin beta-like protein 2 OS=Mus musculus GN=Tbl2 PE=2 SV=2 - [TBL2_MOUSE]	14.71	3	4	4	8	1.05	1.15	1.12	1.04	1.05	1.01	0.93	1.11	0.90	1.12	0.93	1.15	0.96	49.55	9.04
Q9D842-2	Isoform 2 of Aprataxin and PNK-like factor OS=Mus musculus GN=Aplf - [APLF_MOUSE]	7.67	3	1	1	5	0.96	0.82	0.86	0.80	0.91	1.07	1.15	1.11	1.13	1.10	1.11	1.03	1.08	43.02	5.07
D3Z7P2	Transmembrane protein 109 (Fragment) OS=Mus musculus GN=Tmem109 PE=2 SV=1 - [D3Z7P2_MOUSE]	12.33	5	1	1	2	1.04	2.31	2.22	1.10	1.06	1.30	1.24	1.11	1.06	1.24	1.20	1.11	1.07	8.02	8.70
Q8QZT1	Acetyl-CoA acetyltransferase, mitochondrial OS=Mus musculus GN=Acat1 PE=1 SV=1 - [THIL_MOUSE]	38.92	1	13	13	107	0.96	0.97	1.00	0.99	1.05	1.09	1.14	1.11	1.17	1.08	1.16	1.10	1.17	44.79	8.51
Q9EQH4-	Isoform 2 of Transcription initiation factor TFIID subunit 8 OS=Mus musculus GN=Taf8 - [TAF8_MOUSE]	8.52	5	2	2	4	0.91	0.92	1.01	0.82	0.90	1.07	1.17	1.11	1.22	0.91	1.01	1.03	1.13	33.68	6.46
G5E8J9	SCY1-like protein 2 OS=Mus musculus GN=Scyl2 PE=2 SV=1 - [G5E8J9_MOUSE]	7.21	4	4	4	6	1.01	0.80	0.80	1.07	1.02	1.06	1.10	1.11	1.02	1.05	1.02	0.98	0.98	103.12	8.02
Q9WU01	KH domain-containing, RNA-binding, signal transduction-associated protein 2 OS=Mus musculus GN=Khdrbs2 PE=1 SV=1 - [KHDR2_MOUSE]	15.47	2	4	6	19	1.04	0.79	0.73	0.96	0.92	1.20	1.15	1.11	1.11	1.15	1.10	0.97	0.94	38.84	6.48
A2API8	A-kinase anchor protein 2 OS=Mus musculus GN=Akap2 PE=2 SV=1 - [A2API8_MOUSE]	33.77	6	1	23	103	0.94	1.03	1.09	0.93	0.99	1.03	1.08	1.11	1.18	1.06	1.13	1.02	1.08	101.54	5.20
Q9D735	Uncharacterized protein C19orf43 homolog OS=Mus musculus PE=2 SV=1 - [CS043_MOUSE]	23.12	1	3	3	3	1.10	0.94	0.72	0.88	0.92	0.90	1.16	1.11	1.37	0.95	1.10	0.76	0.75	18.37	9.67
Q9CQ69	Cytochrome b-c1 complex subunit 8 OS=Mus musculus GN=Uqcrq PE=1 SV=3 - [QCR8_MOUSE]	46.34	2	4	4	19	0.97	1.03	1.01	1.05	1.12	1.06	1.16	1.11	1.10	1.12	1.14	1.01	1.02	9.76	10.26

Q91Z50	Flap endonuclease 1 OS=Mus musculus GN=Fen1 PE=2 SV=1 - [Q91Z50_MOUSE]	11.32	2	3	3	4	1.01	0.94	1.05	0.88	0.88	1.31	1.29	1.11	1.31	1.12	1.15	1.11	1.13	42.60	8.34
E9PVS1	Inter-alpha-trypsin inhibitor heavy chain H3 OS=Mus musculus GN=Itih3 PE=2 SV=1 - [E9PVS1_MOUSE]	2.29	2	1	1	2	2.16	0.82	0.38	1.04	0.48	0.94	0.44	1.11	0.51	1.74	0.81	1.22	0.57	77.97	6.84
Q6PGJ3	L1 cell adhesion molecule OS=Mus musculus GN=L1cam PE=2 SV=1 - [Q6PGJ3_MOUSE]	36.06	7	34	34	229	1.07	0.90	0.91	0.98	0.92	1.15	1.07	1.11	1.04	1.12	1.04	1.05	1.01	140.83	5.96
Q91VM9	Inorganic pyrophosphatase 2, mitochondrial OS=Mus musculus GN=Ppa2 PE=2 SV=1 - [IPYR2_MOUSE]	60.91	5	16	16	65	0.87	0.88	1.01	0.87	0.99	0.99	1.20	1.11	1.26	1.00	1.10	0.93	1.11	38.09	6.98
Q8BJZ4	28S ribosomal protein S35, mitochondrial OS=Mus musculus GN=Mrps35 PE=2 SV=2 - [RT35_MOUSE]	20.94	1	4	4	9	0.76	0.77	0.80	0.98	1.17	1.00	1.06	1.11	1.22	1.01	1.16	1.08	1.17	35.95	8.59
Q9CYH6	Ribosome biogenesis regulatory protein homolog OS=Mus musculus GN=Rrs1 PE=2 SV=1 - [RRS1_MOUSE]	5.48	1	2	2	3	1.21	1.12	0.92	1.20	0.99	1.24	1.02	1.11	0.92	1.25	1.04	1.33	1.10	41.53	10.77
Q99P72	Reticulon-4 OS=Mus musculus GN=Rtn4 PE=1 SV=2 - [RTN4_MOUSE]	57.23	5	44	44	359	1.00	0.91	0.96	0.97	0.97	1.11	1.09	1.11	1.08	1.08	1.07	1.01	1.02	126.54	4.54
O54916	RalBP1-associated Eps domain-containing protein 1 OS=Mus musculus GN=Reps1 PE=1 SV=2 - [REPS1_MOUSE]	14.97	10	7	8	16	1.00	1.08	1.11	0.96	0.99	1.02	0.97	1.11	1.11	1.06	1.03	1.12	1.06	86.47	5.58
Q3UPL5	Uncharacterized protein C11orf96 homolog OS=Mus musculus GN=Ag2 PE=1 SV=2 - [CK096_MOUSE]	13.65	1	2	2	5	0.93	0.94	1.00	0.95	1.02	1.06	1.02	1.11	1.19	1.00	1.22	1.02	1.09	27.06	10.89
Q9DC33	High mobility group protein 20A OS=Mus musculus GN=Hmg20a PE=2 SV=1 - [HM20A_MOUSE]	19.94	3	5	5	15	1.11	1.13	0.99	1.03	0.91	0.99	0.88	1.11	0.93	1.02	0.99	1.09	0.98	39.89	6.40
Q9D168	Integrator complex subunit 12 OS=Mus musculus GN=Ints12 PE=1 SV=1 - [INT12_MOUSE]	19.74	1	6	6	11	1.00	0.78	0.76	0.92	0.91	1.11	1.10	1.11	1.09	0.99	0.98	0.97	1.02	48.54	9.69
Q9D1B9	39S ribosomal protein L28, mitochondrial OS=Mus musculus GN=Mrpl28 PE=2 SV=3 - [RM28_MOUSE]	6.23	1	2	2	3	0.92	1.45	1.56	1.16	1.25	1.12	1.20	1.11	1.20	0.99	1.07	0.85	0.92	30.15	9.29

P10711	Transcription elongation factor A protein 1 OS=Mus musculus GN=Tcea1 PE=1 SV=2 - [TCEA1_MOUSE]	56.81	6	16	16	54	1.02	0.91	0.90	0.95	0.90	1.13	1.10	1.11	1.12	1.06	1.02	1.00	0.96	33.86	8.38
Q9DCT8	Cysteine-rich protein 2 OS=Mus musculus GN=Crip2 PE=1 SV=1 - [CRIP2_MOUSE]	68.75	1	9	9	148	1.03	0.84	0.80	0.96	0.95	1.12	1.10	1.11	1.06	1.01	1.01	0.93	0.93	22.71	8.63
A2AWT3	Ataxin-7-like protein 3 OS=Mus musculus GN=Atxn7l3 PE=2 SV=1 - [AT7L3_MOUSE]	2.02	2	1	1	3	1.08	0.76	0.70	1.04	0.97	1.18	1.09	1.11	1.03	1.07	1.00	1.03	0.96	38.57	7.03
Q9CX86	Heterogeneous nuclear ribonucleoprotein A0 OS=Mus musculus GN=Hnrnpa0 PE=1 SV=1 - [ROA0_MOUSE]	36.72	1	6	8	30	0.83	0.77	0.92	0.86	1.03	1.01	1.27	1.11	1.33	0.93	1.17	0.94	1.20	30.51	9.31
Q8BUE5	Alpha-1D adrenergic receptor OS=Mus musculus GN=Adra1a PE=2 SV=1 - [Q8BUE5_MOUSE]	2.58	2	1	1	2	1.00	1.47	1.46	1.03	1.03	0.99	0.98	1.11	1.10	1.16	1.15	1.32	1.32	47.48	9.36
E9Q2D0	WAS/WASL-interacting protein family member 3 OS=Mus musculus GN=Wipf3 PE=4 SV=1 - [E9Q2D0_MOUSE]	58.39	2	2	18	103	1.07	0.91	0.80	0.92	0.80	1.16	1.09	1.11	1.02	1.03	0.96	1.01	0.95	49.23	10.18
Q8BIQ5	Cleavage stimulation factor subunit 2 OS=Mus musculus GN=Cstf2 PE=1 SV=2 - [CSTF2_MOUSE]	31.21	4	3	12	35	1.17	1.04	0.90	0.95	0.70	1.15	0.98	1.11	0.82	0.92	0.79	0.95	0.74	61.30	6.83
P28184	Metallothionein-3 OS=Mus musculus GN=Mt3 PE=1 SV=1 - [MT3_MOUSE]	41.18	1	3	3	19	1.14	0.71	0.78	0.86	0.85	0.93	0.91	1.11	0.88	0.93	0.86	0.70	0.80	7.00	7.47
D3Z719	Protein SMG9 (Fragment) OS=Mus musculus GN=Smg9 PE=2 SV=1 - [D3Z719_MOUSE]	12.38	3	2	2	5	0.96	0.70	0.73	0.88	0.91	1.19	1.23	1.11	1.15	1.01	1.06	0.95	0.96	22.54	8.10
P08228	Superoxide dismutase [Cu-Zn] OS=Mus musculus GN=Sod1 PE=1 SV=2 - [SODC_MOUSE]	59.09	1	11	11	404	1.06	0.95	0.90	0.94	0.87	1.15	1.07	1.11	1.06	1.08	1.03	1.03	0.99	15.93	6.51
Q8C419	Probable G-protein coupled receptor 158 OS=Mus musculus GN=Gpr158 PE=1 SV=2 - [GP158_MOUSE]	31.75	1	27	27	142	0.94	0.92	0.99	0.94	0.98	1.14	1.19	1.11	1.14	1.09	1.12	1.00	1.02	134.34	8.09
Q9Z0R4	Intersectin-1 OS=Mus musculus GN=Itsn1 PE=1 SV=2 - [ITSN1_MOUSE]	24.27	11	1	35	85	1.18	1.13	0.96	1.06	0.90	1.14	0.95	1.11	0.94	0.93	0.79	0.98	0.83	194.18	7.91

P42567	Epidermal growth factor receptor substrate 15 OS=Mus musculus GN=Eps15 PE=1 SV=1 - [EPS15_MOUSE]	33.00	7	18	18	39	1.02	0.97	0.98	0.99	1.03	1.04	1.09	1.11	1.16	1.04	1.09	1.06	1.02	98.41	4.60
P57774	Pro-neuropeptide Y OS=Mus musculus GN=Npy PE=2 SV=2 - [NPY_MOUSE]	29.90	1	2	2	2	1.27	0.96	0.75	0.90	0.71	1.24	0.97	1.11	0.88	0.98	0.78	1.02	0.80	10.87	7.12
P16627	Heat shock 70 kDa protein 1-like OS=Mus musculus GN=Hspa1l PE=2 SV=4 - [HS71L_MOUSE]	26.21	1	1	15	260	1.02	0.80	0.78	0.83	0.81	1.10	1.07	1.11	1.09	1.02	1.00	1.01	1.00	70.59	6.24
Q60864	Stress-induced-phosphoprotein 1 OS=Mus musculus GN=Stip1 PE=1 SV=1 - [STIP1_MOUSE]	70.17	1	45	45	566	1.09	0.81	0.76	0.95	0.88	1.13	1.04	1.11	1.03	1.04	0.98	0.95	0.91	62.54	6.80
Q5NCU4	SPARC OS=Mus musculus GN=Sparc PE=2 SV=1 - [Q5NCU4_MOUSE]	18.27	3	5	5	7	1.39	0.99	0.73	0.98	0.71	1.15	0.81	1.11	0.78	1.32	0.96	1.28	0.97	34.30	4.86
F6UK66	Coiled-coil domain-containing protein 50 (Fragment) OS=Mus musculus GN=Ccdc50 PE=4 SV=1 - [F6UK66_MOUSE]	32.42	4	5	5	22	1.05	1.00	1.02	0.92	0.87	1.21	1.15	1.11	1.06	1.07	1.02	0.97	1.01	29.78	6.67
O70139	cAMP-dependent protein kinase inhibitor gamma OS=Mus musculus GN=Pkig PE=2 SV=1 - [IPKG_MOUSE]	72.37	3	2	2	11	1.08	1.10	1.15	1.05	1.05	1.17	1.19	1.11	1.02	1.05	0.97	1.22	1.21	7.94	4.21
F6R8S6	Proline-rich AKT1 substrate 1 (Fragment) OS=Mus musculus GN=Akt1s1 PE=4 SV=1 - [F6R8S6_MOUSE]	33.47	4	1	4	29	0.98	0.85	0.86	0.86	0.88	1.26	1.27	1.11	1.13	0.91	0.93	1.01	1.03	26.45	4.58
Q923F1	Chloride channel, nucleotide-sensitive, 1A OS=Mus musculus GN=Clns1a PE=2 SV=1 - [Q923F1_MOUSE]	52.28	1	2	8	62	0.81	0.92	1.39	0.84	1.03	1.10	1.31	1.11	1.35	1.14	1.44	0.84	1.06	26.51	4.12
E9Q6Y8	Ubiquitin carboxyl-terminal hydrolase OS=Mus musculus GN=Usp31 PE=2 SV=1 - [E9Q6Y8_MOUSE]	10.49	1	9	9	16	1.03	1.02	1.06	1.06	0.95	1.07	1.02	1.11	1.00	1.18	1.10	1.16	1.09	146.23	9.17
A2AG50	MAP7 domain-containing protein 2 OS=Mus musculus GN=Map7d2 PE=1 SV=1 - [MA7D2_MOUSE]	43.92	6	26	31	92	1.06	0.76	0.74	0.87	0.86	1.14	1.09	1.11	1.02	1.03	0.94	0.92	0.86	86.00	8.81

O88746	Target of Myb protein 1 OS=Mus musculus GN=Tom1 PE=1 SV=1 - [TOM1_MOUSE]	47.15	2	8	14	34	0.90	1.12	1.10	1.04	1.02	1.13	1.19	1.11	1.19	1.05	1.06	1.04	1.08	54.29	4.94
Q8BHS8-2	Isoform 2 of Syntabulin OS=Mus musculus GN=Sybu - [SYBU_MOUSE]	4.97	6	2	3	12	1.25	1.05	0.92	0.99	0.85	1.13	0.91	1.11	0.93	1.14	1.04	1.19	0.99	72.85	6.38
P97794	ATP-sensitive inward rectifier potassium channel 8 OS=Mus musculus GN=Kcnj8 PE=2 SV=1 - [IRK8_MOUSE]	3.07	1	1	1	1	0.78	0.96	1.24	1.07	1.37	1.06	1.35	1.11	1.42	1.05	1.35	0.85	1.09	47.95	9.20
E9PVW4	V-set and immunoglobulin domain-containing protein 10 OS=Mus musculus GN=Vsig10 PE=2 SV=1 - [E9PVW4_MOUSE]	7.53	1	1	1	1	1.15	1.12	0.97	1.19	1.03	1.12	0.97	1.11	0.97	1.26	1.10	0.88	0.77	57.61	4.64
P54728	UV excision repair protein RAD23 homolog B OS=Mus musculus GN=Rad23b PE=1 SV=2 - [RD23B_MOUSE]	43.99	1	9	11	100	1.01	0.93	0.88	0.94	0.89	1.09	1.09	1.11	1.03	1.01	0.97	0.92	0.86	43.49	4.83
Q3TY65	Islet cell autoantigen 1-like protein OS=Mus musculus GN=Ica1l PE=2 SV=1 - [ICA1L_MOUSE]	3.25	1	1	1	2	0.81	1.13	1.39	1.02	1.26	1.02	1.25	1.11	1.37	1.37	1.69	1.12	1.38	48.13	4.91
D3Z1T2	MCG15614 OS=Mus musculus GN=2310009B15Rik PE=4 SV=1 - [D3Z1T2_MOUSE]	6.62	1	1	1	2	1.04	1.04	1.00	0.97	0.94	0.96	0.92	1.11	1.07	0.86	0.83	0.99	0.96	14.65	9.11
Q61527-3	Isoform JM-A CYT-2 of Receptor tyrosine-protein kinase erbB-4 OS=Mus musculus GN=Erbb4 - [ERBB4_MOUSE]	2.94	4	3	4	7	0.97	1.23	1.24	0.97	1.00	1.18	1.18	1.11	1.16	1.05	1.10	1.18	1.24	145.15	6.39
Q6DIB5	Multiple epidermal growth factor-like domains protein 10 OS=Mus musculus GN=Megf10 PE=1 SV=1 - [MEG10_MOUSE]	7.76	1	6	6	10	1.04	1.17	1.09	0.89	0.82	0.98	1.00	1.11	1.06	1.02	0.89	0.98	0.94	122.89	7.05
Q8K4P0	pre-mRNA 3' end processing protein WDR33 OS=Mus musculus GN=Wdr33 PE=2 SV=1 - [WDR33_MOUSE]	6.02	2	5	5	12	0.93	1.20	1.25	0.88	0.95	0.91	0.94	1.11	1.24	0.94	1.06	1.00	1.08	145.18	9.13
Q3TVI8	Pre-B-cell leukemia transcription factor-interacting protein 1 OS=Mus musculus GN=Pbxip1 PE=1 SV=2 - [PBIP1_MOUSE]	3.99	4	2	2	3	1.02	1.41	1.38	1.03	1.01	1.13	1.10	1.11	1.08	1.24	1.21	1.41	1.37	81.12	5.36

J3QMK2	MCG22959, isoform CRA_a OS=Mus musculus GN=Bbip1 PE=4 SV=1 - [J3QMK2_MOUSE]	41.79	1	2	2	4	1.05	0.95	0.90	1.00	0.95	1.14	1.08	1.11	1.05	1.07	1.02	1.14	1.09	7.59	8.76
Q9CR09	Ubiquitin-fold modifier-conjugating enzyme 1 OS=Mus musculus GN=Ufc1 PE=2 SV=1 - [UFC1_MOUSE]	22.75	2	4	4	9	0.78	0.90	1.15	1.07	1.49	1.02	1.10	1.11	1.31	1.13	1.32	0.93	1.23	19.47	7.40
P62334	26S protease regulatory subunit 10B OS=Mus musculus GN=Psmc6 PE=1 SV=1 - [PRS10_MOUSE]	53.73	1	15	16	48	0.99	1.15	1.15	1.06	1.03	1.04	1.01	1.11	1.09	1.09	1.08	1.09	1.07	44.15	7.49
P27546-2	Isoform 2 of Microtubule-associated protein 4 OS=Mus musculus GN=Map4 - [MAP4_MOUSE]	56.23	7	23	44	278	1.01	0.87	0.85	0.92	0.90	1.12	1.12	1.11	1.14	1.03	1.07	0.95	0.99	117.27	4.98
P63028	Translationally-controlled tumor protein OS=Mus musculus GN=Tpt1 PE=1 SV=1 - [TCTP_MOUSE]	47.09	2	5	5	109	0.99	0.72	0.77	0.87	0.85	1.14	1.14	1.11	1.11	0.97	0.94	0.92	0.88	19.45	4.86
Q8VI56-2	Isoform 2 of Low-density lipoprotein receptor-related protein 4 OS=Mus musculus GN=Lrp4 - [LRP4_MOUSE]	0.49	2	1	1	1	1.31	1.60	1.22	1.12	0.86	0.98	0.74	1.11	0.85	1.03	0.79	1.17	0.90	205.24	5.36
Q05915	GTP cyclohydrolase 1 OS=Mus musculus GN=Gch1 PE=2 SV=1 - [GCH1_MOUSE]	3.73	1	1	1	2	1.10	1.03	0.93	0.93	0.84	1.31	1.18	1.11	1.01	1.24	1.12	1.09	0.99	27.00	8.27
O54818	Tumor protein D53 OS=Mus musculus GN=Tpd52l1 PE=2 SV=1 - [TPD53_MOUSE]	29.41	1	4	4	19	1.10	0.81	0.68	0.93	0.85	1.04	1.01	1.11	1.02	1.02	0.95	1.00	0.91	22.50	6.21
Q8R035	Peptidyl-tRNA hydrolase ICT1, mitochondrial OS=Mus musculus GN=Ict1 PE=1 SV=1 - [ICT1_MOUSE]	26.21	4	5	5	8	0.95	0.76	0.83	0.79	0.83	1.08	1.15	1.11	1.20	1.07	1.22	1.07	1.06	23.46	10.18
Q9CRD0	OCIA domain-containing protein 1 OS=Mus musculus GN=Ociad1 PE=1 SV=1 - [OCAD1_MOUSE]	47.37	3	9	9	20	0.92	0.93	0.92	0.98	1.06	1.14	1.20	1.12	1.17	1.06	1.05	1.02	1.08	27.59	7.81
P62192	26S protease regulatory subunit 4 OS=Mus musculus GN=Psmc1 PE=1 SV=1 - [PRS4_MOUSE]	50.45	1	15	16	46	0.94	1.03	1.04	0.94	1.01	1.14	1.22	1.12	1.30	1.10	1.16	1.10	1.15	49.15	6.21
G3UYL7	Protein FAM13A OS=Mus musculus GN=Fam13a PE=2 SV=1 - [G3UYL7_MOUSE]	2.71	3	1	2	3	1.11	1.09	0.97	0.91	0.82	1.33	1.19	1.12	1.00	1.08	0.97	0.93	0.84	76.32	5.27

P54227	Stathmin OS=Mus musculus GN=Stmn1 PE=1 SV=2 - [STMN1_MOUSE]	71.14	3	12	15	316	0.98	0.85	0.87	0.89	0.91	1.11	1.13	1.12	1.07	1.03	0.98	0.91	0.96	17.26	5.97
B1ARW4	NADH dehydrogenase [ubiquinone] iron-sulfur protein 5 (Fragment) OS=Mus musculus GN=Ndufs5 PE=2 SV=1 - [B1ARW4_MOUSE]	45.05	2	4	4	72	0.96	0.91	0.92	0.94	0.93	1.08	1.09	1.12	1.16	1.01	1.03	0.98	0.99	10.90	8.94
O35526	Syntaxin-1A OS=Mus musculus GN=Stx1a PE=1 SV=3 - [STX1A_MOUSE]	63.89	2	19	20	529	1.00	0.95	0.97	0.98	0.99	1.13	1.13	1.12	1.09	1.04	1.02	0.94	0.98	33.03	5.24
P51880	Fatty acid-binding protein, brain OS=Mus musculus GN=Fabp7 PE=1 SV=2 - [FABP7_MOUSE]	40.15	2	4	4	21	1.13	0.81	0.77	0.91	0.80	1.14	1.09	1.12	1.02	1.06	0.96	1.14	1.02	14.88	5.63
Q6PDI6-3	Isoform 3 of Protein FAM63B OS=Mus musculus GN=Fam63b - [FA63B_MOUSE]	15.98	2	4	4	8	1.08	1.01	0.98	1.02	0.93	1.12	1.02	1.12	1.03	1.11	1.09	1.12	1.06	41.19	4.60
E9QML5	Zinc finger protein 638 OS=Mus musculus GN=Zfml PE=2 SV=1 - [E9QML5_MOUSE]	20.04	8	27	27	50	1.02	1.01	0.96	1.03	0.98	1.17	1.08	1.12	1.16	1.10	1.06	1.06	1.03	214.33	6.76
Q9CW79-	Isoform 2 of Golgin subfamily A member 1 OS=Mus musculus GN=Golga1 - [GOGA1_MOUSE]	12.01	2	6	6	12	0.92	1.08	1.18	0.97	0.99	1.16	1.09	1.12	1.19	1.07	1.08	1.08	1.13	84.25	5.50
G5E920	MCG114640 OS=Mus musculus GN=Gm6104 PE=4 SV=1 - [G5E920_MOUSE]	50.50	3	9	9	22	1.09	0.90	0.81	1.00	0.95	1.25	1.06	1.12	1.04	1.20	1.05	1.05	0.96	23.02	8.37
Q6PD26	GPI transamidase component PIG-S OS=Mus musculus GN=Pigs PE=1 SV=3 - [PIGS_MOUSE]	6.49	1	3	3	4	1.11	1.38	1.21	1.14	1.04	0.85	0.76	1.12	1.06	1.12	1.08	1.08	1.03	61.67	6.93
Q8K4G5-2	Isoform 2 of Actin-binding LIM protein 1 OS=Mus musculus GN=Ablim1 - [ABLM1_MOUSE]	58.35	16	32	33	166	1.04	0.93	0.89	0.92	0.89	1.12	1.06	1.12	1.06	1.00	0.95	0.95	0.89	78.91	8.25
Q920D3	Mediator of RNA polymerase II transcription subunit 28 OS=Mus musculus GN=Med28 PE=2 SV=2 - [MED28_MOUSE]	26.97	2	3	3	4	1.35	1.32	0.82	0.84	0.73	1.05	1.04	1.12	0.60	0.87	0.88	1.02	0.49	19.53	5.58
P62488	DNA-directed RNA polymerase II subunit RPB7 OS=Mus musculus GN=Polr2g PE=2 SV=1 - [RPB7_MOUSE]	8.72	1	1	1	2	1.08	1.23	1.14	1.15	1.07	1.23	1.13	1.12	1.03	1.16	1.07	1.04	0.96	19.28	5.54
E9Q179	G-rich sequence factor 1 OS=Mus musculus GN=Grsf1 PE=2 SV=1 - [E9Q179_MOUSE]	12.98	2	3	3	5	0.89	0.96	0.94	0.81	0.91	1.06	1.19	1.12	1.19	1.07	1.12	1.01	0.99	41.58	5.33

Q80W14-2	Isoform 2 of Pre-mRNA-processing factor 40 homolog B OS=Mus musculus GN=Prpf40b - [PR40B_MOUSE]	12.03	7	5	5	15	1.02	0.77	0.82	0.90	0.90	1.15	1.16	1.12	1.10	0.89	0.93	0.91	0.92	63.24	5.90
D3Z6Q9	Bridging integrator 2 OS=Mus musculus GN=Bin2 PE=2 SV=1 - [BIN2_MOUSE]	7.16	1	2	4	12	0.86	0.96	1.12	0.82	0.96	1.06	1.24	1.12	1.30	1.04	1.22	0.89	1.04	52.52	5.34
Q62415	Apoptosis-stimulating of p53 protein 1 OS=Mus musculus GN=Ppp1r13b PE=1 SV=2 - [ASPP1_MOUSE]	18.03	4	15	15	37	0.95	0.96	0.99	0.89	0.95	1.09	1.17	1.12	1.16	1.02	1.07	1.04	1.06	119.10	6.68
P63040	Complexin-1 OS=Mus musculus GN=Cplx1 PE=1 SV=1 - [CPLX1_MOUSE]	64.18	1	3	8	363	0.92	0.80	0.87	0.91	0.98	1.08	1.18	1.12	1.22	0.98	1.03	0.88	0.95	15.11	4.97
Q91X96	Guanine nucleotide exchange factor MSS4 OS=Mus musculus GN=Rabif PE=2 SV=1 - [MSS4_MOUSE]	22.76	1	3	3	7	1.09	0.95	0.95	0.95	0.82	1.17	1.02	1.12	1.06	1.11	1.01	1.13	0.95	13.91	5.52
Q91ZZ3	Beta-synuclein OS=Mus musculus GN=Sncb PE=1 SV=1 - [SYUB_MOUSE]	58.65	1	8	13	485	1.03	0.88	0.87	0.93	0.92	1.13	1.07	1.12	1.07	1.02	0.98	0.98	0.95	14.04	4.37
B1AXN9	Ribosomal protein S6 kinase alpha-3 OS=Mus musculus GN=Rps6ka3 PE=2 SV=1 - [B1AXN9_MOUSE]	2.67	2	2	2	2	1.15	1.54	1.34	1.17	1.01	1.00	0.87	1.12	0.97	1.14	0.99	1.35	1.18	80.62	7.15
B7ZCJ1	Rho GTPase-activating protein 21 OS=Mus musculus GN=Arhgap21 PE=4 SV=1 - [B7ZCJ1_MOUSE]	14.12	6	19	20	44	1.02	0.98	1.05	0.99	0.97	1.10	1.05	1.12	1.02	0.95	0.94	1.00	1.05	216.85	7.64
Q6NS69	APC membrane recruitment protein 3 OS=Mus musculus GN=Amer3 PE=2 SV=1 - [AMER3_MOUSE]	2.95	1	1	1	1	1.22	1.25	1.02	0.80	0.65	1.32	1.07	1.12	0.91	0.92	0.76	1.30	1.06	83.11	5.77
E9PV48	Protein I830012O16Rik OS=Mus musculus GN=I830012O16Rik PE=4 SV=1 - [E9PV48_MOUSE]	2.23	2	1	1	4	1.64	1.10	0.60	1.16	0.64	1.22	0.73	1.12	0.61	1.00	0.55	1.04	0.66	47.19	5.86
D3Z3A3	SPARC-related modular calcium-binding protein 1 OS=Mus musculus GN=Smoc1 PE=2 SV=1 - [D3Z3A3_MOUSE]	2.30	4	1	1	1	0.98	1.07	1.09	0.99	1.00	1.10	1.12	1.12	1.13	1.27	1.29	1.11	1.12	47.97	8.16
Q3UFQ8	Leucine-rich repeat-containing protein 16B OS=Mus musculus GN=Lrrc16b PE=2 SV=2 - [LR16B_MOUSE]	1.24	1	1	1	1	0.92	1.19	1.29	0.98	1.06	0.91	0.98	1.12	1.21	1.30	1.41	1.26	1.37	150.32	7.72

Q99L48	60S ribosomal export protein NMD3 OS=Mus musculus GN=Nmd3 PE=2 SV=1 - [NMD3_MOUSE]	3.38	1	1	1	4	0.84	0.93	1.10	0.93	1.11	1.04	1.23	1.12	1.32	0.83	0.99	0.84	1.00	57.57	7.17
G3X9D3	Podocalyxin-like 2 OS=Mus musculus GN=Podxl2 PE=4 SV=1 - [G3X9D3_MOUSE]	9.65	5	4	4	12	1.16	1.02	0.87	1.04	0.90	1.22	1.04	1.12	0.96	1.22	1.05	1.08	0.93	58.36	4.39
Q3U1V8	Mitogen-activated protein kinase kinase kinase 9 OS=Mus musculus GN=Map3k9 PE=2 SV=2 - [M3K9_MOUSE]	5.01	4	3	4	5	1.27	1.09	0.93	1.07	0.86	1.16	0.91	1.12	0.88	1.17	0.93	1.06	0.84	118.73	5.86
Q6GT24	Peroxiredoxin 6 OS=Mus musculus GN=Prdx6 PE=2 SV=1 - [Q6GT24_MOUSE]	74.11	4	17	17	227	0.95	0.89	0.93	0.93	0.96	1.10	1.11	1.12	1.16	1.03	1.10	1.01	1.03	24.81	6.37
P52760	Ribonuclease UK114 OS=Mus musculus GN=Hrsp12 PE=1 SV=3 - [UK114_MOUSE]	92.59	1	11	11	125	1.14	1.02	0.90	0.94	0.83	1.06	0.91	1.12	1.05	1.07	0.95	1.04	0.90	14.25	8.68
P06837	Neuromodulin OS=Mus musculus GN=Gap43 PE=1 SV=1 - [NEUM_MOUSE]	83.26	1	23	23	249	1.25	0.80	0.63	0.97	0.78	1.22	0.98	1.12	0.89	1.15	0.95	1.14	0.94	23.62	4.73
Q2PZL6	Protocadherin Fat 4 OS=Mus musculus GN=Fat4 PE=1 SV=2 - [FAT4_MOUSE]	0.52	1	1	2	3	0.96	0.97	1.01	0.88	0.91	1.14	1.18	1.12	1.16	1.24	1.30	1.24	1.29	539.98	4.91
Q9R1C7	Pre-mRNA-processing factor 40 homolog A OS=Mus musculus GN=Prpf40a PE=1 SV=1 - [PR40A_MOUSE]	20.88	3	16	16	42	1.10	0.93	0.80	0.95	0.89	1.14	1.00	1.12	0.99	1.02	0.92	1.00	0.93	108.41	7.69
Q8R1I1	Cytochrome b-c1 complex subunit 9 OS=Mus musculus GN=Uqcr10 PE=1 SV=1 - [QCR9_MOUSE]	26.56	1	1	1	8	1.12	0.87	0.78	1.18	1.05	1.18	1.03	1.12	0.99	1.08	0.98	1.22	1.09	7.44	9.19
G3UYB8	Proline-rich protein 3 (Fragment) OS=Mus musculus GN=Prr3 PE=2 SV=1 - [G3UYB8_MOUSE]	68.42	5	3	3	8	1.25	0.92	0.64	1.14	0.93	1.20	0.88	1.12	0.81	1.05	0.85	1.05	0.95	8.31	8.63
Q80W04	Transmembrane and coiled-coil domains protein 2 OS=Mus musculus GN=Tmcc2 PE=1 SV=1 - [TMCC2_MOUSE]	10.20	3	3	4	9	0.97	1.31	1.34	1.11	1.03	1.07	1.08	1.12	1.14	1.18	1.14	1.40	1.44	77.01	6.84
Q9QXN3-	Isoform 2 of Activating signal cointegrator 1 OS=Mus musculus GN=Trip4 - [TRIP4_MOUSE]	14.84	2	6	6	10	1.13	0.86	0.89	0.92	0.83	1.17	1.00	1.12	0.94	1.10	0.99	1.12	0.99	61.49	7.21

Q91YJ5	Translation initiation factor IF-2, mitochondrial OS=Mus musculus GN=Mtif2 PE=1 SV=2 - [IF2M_MOUSE]	2.34	1	1	1	3	0.89	1.12	1.17	1.25	1.06	0.85	1.04	1.12	1.03	0.80	0.90	0.70	0.79	81.24	7.24
Q9ESU6	Bromodomain-containing protein 4 OS=Mus musculus GN=Brd4 PE=1 SV=2 - [BRD4_MOUSE]	10.50	7	8	12	34	0.91	0.97	1.00	0.90	0.97	1.05	1.16	1.12	1.24	1.04	1.11	0.96	1.08	155.80	9.19
Q8BTV2-:	Isoform 2 of Cleavage and polyadenylation specificity factor subunit 7 OS=Mus musculus GN=Cpsf7 - [CPSF7_MOUSE]	19.26	3	6	6	12	0.96	0.99	1.09	0.99	1.04	0.98	0.98	1.12	1.13	1.02	1.06	0.93	1.00	51.03	7.78
Q62446	Peptidyl-prolyl cis-trans isomerase FKBP3 OS=Mus musculus GN=Fkbp3 PE=1 SV=2 - [FKBP3_MOUSE]	59.82	1	13	13	49	1.10	0.72	0.71	0.93	0.84	1.16	1.08	1.12	1.05	1.04	0.94	0.91	0.90	25.13	9.28
F6TY66	Claudin-10A (Fragment) OS=Mus musculus GN=Cldn10 PE=4 SV=1 - [F6TY66_MOUSE]	12.30	4	1	1	2	1.12	2.76	2.47	1.24	1.11	1.07	0.96	1.12	1.00	1.13	1.01	1.15	1.04	13.12	8.76
Q9JJN6	Beta-catenin-interacting protein 1 OS=Mus musculus GN=Ctnnbip1 PE=1 SV=1 - [CNBP1_MOUSE]	67.90	1	3	3	8	0.91	0.97	0.97	0.88	0.96	1.01	1.08	1.12	1.17	0.93	0.98	1.12	1.14	9.17	5.41
Q9JJG0-3	Isoform 3 of Transforming acidic coiled-coil-containing protein 2 OS=Mus musculus GN=Tacc2 - [TACC2_MOUSE]	25.98	7	14	17	31	1.09	0.85	0.78	0.96	0.88	1.17	1.05	1.12	1.07	1.05	1.02	0.96	0.87	103.21	4.93
G3UYV7	40S ribosomal protein S28 (Fragment) OS=Mus musculus GN=Rps28 PE=2 SV=1 - [G3UYV7_MOUSE]	57.14	3	3	3	33	0.96	0.93	1.00	0.87	0.86	1.10	1.17	1.12	1.18	0.98	1.03	0.90	0.96	6.34	9.96
Q6F3F9	G-protein coupled receptor 126 OS=Mus musculus GN=Gpr126 PE=1 SV=1 - [GP126_MOUSE]	1.12	1	1	1	1	0.91	0.71	0.78	0.90	0.99	1.20	1.31	1.12	1.23	1.14	1.26	1.07	1.18	129.64	6.68
Q80VW5-	Isoform 2 of Whirlin OS=Mus musculus GN=Dfnb31 - [WHRN_MOUSE]	1.54	1	1	1	1	0.78	1.14	1.46	0.81	1.03	0.98	1.25	1.12	1.43	0.91	1.17	0.95	1.23	96.95	8.70
Q5SUF2	Luc7-like protein 3 OS=Mus musculus GN=Luc7l3 PE=1 SV=1 - [LC7L3_MOUSE]	15.51	4	5	6	18	1.09	0.96	0.92	1.13	1.02	1.23	1.12	1.12	0.98	1.10	0.97	1.07	0.98	51.42	9.77
Q3U182	CREB-regulated transcription coactivator 2 OS=Mus musculus GN=Crtc2 PE=1 SV=2 - [CRTC2_MOUSE]	3.76	1	2	2	3	1.06	0.99	0.93	0.97	0.91	1.10	1.04	1.12	1.05	1.00	0.95	1.09	1.03	73.17	7.01

E9PYB0	Protein Ahnak2 (Fragment) OS=Mus musculus GN=Ahnak2 PE=2 SV=1 - [E9PYB0_MOUSE]	32.60	1	2	11	17	1.28	0.83	0.64	0.81	0.63	0.88	0.68	1.12	0.87	1.19	0.93	0.71	0.56	182.59	7.08
Q91WZ8-	Isoform 3 of Dysbindin OS=Mus musculus GN=Dtnbp1 - [DTBP1_MOUSE]	10.70	4	2	2	5	1.05	0.66	0.63	0.92	0.88	1.23	1.16	1.12	1.06	1.12	1.07	1.09	1.04	30.56	4.45
D3YWT1	MCG11326, isoform CRA_b OS=Mus musculus GN=Hnrnph3 PE=4 SV=1 - [D3YWT1_MOUSE]	26.89	2	5	6	14	1.13	0.95	0.82	1.13	0.96	1.15	0.95	1.12	0.87	1.11	0.96	1.14	1.02	35.16	6.87
Q9D6U8	Protein FAM162A OS=Mus musculus GN=Fam162a PE=2 SV=1 - [F162A_MOUSE]	16.77	1	3	3	13	1.11	1.02	0.93	1.16	1.08	1.21	1.09	1.12	1.06	1.25	1.04	1.16	1.18	17.71	9.88
Q6PHS9-5	Isoform 5 of Voltage-dependent calcium channel subunit alpha-2/delta-2 OS=Mus musculus GN=Cacna2d2 - [CA2D2_MOUSE]	12.78	4	1	13	27	0.82	1.16	1.41	0.98	1.19	1.17	1.43	1.12	1.36	1.05	1.29	0.73	0.90	129.88	5.60
Q9CYI4	Putative RNA-binding protein Luc7-like 1 OS=Mus musculus GN=Luc7l PE=2 SV=2 - [LUC7L_MOUSE]	18.33	7	5	7	16	1.20	1.15	0.85	1.12	1.09	1.08	0.97	1.12	1.07	1.22	1.05	1.12	1.02	43.91	9.88
Q9QYE6	Golgin subfamily A member 5 OS=Mus musculus GN=Golga5 PE=1 SV=2 - [GOGA5_MOUSE]	6.86	1	5	5	8	0.96	1.08	1.12	0.98	1.06	1.10	1.19	1.12	1.25	1.02	1.14	0.97	0.98	82.32	6.23
P70663	SPARC-like protein 1 OS=Mus musculus GN=Sparcl1 PE=1 SV=3 - [SPRL1_MOUSE]	49.08	1	21	21	83	0.97	0.87	0.89	0.90	0.93	1.11	1.12	1.12	1.14	0.98	1.03	0.90	0.96	72.24	4.60
P09055	Integrin beta-1 OS=Mus musculus GN=Itgb1 PE=1 SV=1 - [ITB1_MOUSE]	8.90	1	5	5	9	0.94	1.22	1.25	1.10	1.05	0.92	1.00	1.12	1.10	1.12	1.22	1.16	1.19	88.17	5.94
Q61466-2	Isoform 1 of SWI/SNF-related matrix-associated actin-dependent regulator of chromatin subfamily D member 1 OS=Mus musculus GN=Smarcd1 - [SMRD1_MOUSE]	9.34	4	2	3	8	1.03	1.16	1.18	0.99	0.81	1.09	1.05	1.12	0.91	1.14	0.91	1.31	1.33	58.02	9.17
Q64291	Keratin, type I cytoskeletal 12 OS=Mus musculus GN=Krt12 PE=1 SV=2 - [K1C12_MOUSE]	1.85	1	1	1	1	0.81	0.94	1.17	0.85	1.05	1.05	1.29	1.12	1.38	0.82	1.02	0.84	1.05	52.43	4.82
E9QK16	Protocadherin Fat 3 OS=Mus musculus GN=Fat3 PE=2 SV=1 - [E9QK16_MOUSE]	0.57	2	2	2	2	0.98	1.09	1.11	1.09	1.11	0.94	0.96	1.12	1.14	1.00	1.03	0.92	0.95	501.28	4.87

Q9CQ46	EF-hand calcium-binding domain-containing protein 2 OS=Mus musculus GN=Efcab2 PE=2 SV=1 - [EFCB2_MOUSE]	6.71	1	1	1	1	1.10	1.13	1.02	0.93	0.85	1.22	1.10	1.12	1.01	1.07	0.97	1.06	0.97	18.92	4.45
D3Z4B1	Intraflagellar transport protein 46 homolog (Fragment) OS=Mus musculus GN=Ift46 PE=2 SV=1 - [D3Z4B1_MOUSE]	9.73	2	1	1	1	0.91	0.63	0.69	0.78	0.86	1.17	1.28	1.12	1.23	0.98	1.08	0.78	0.86	12.81	4.04
Q9D113	DNL-type zinc finger protein OS=Mus musculus GN=Dnlz PE=2 SV=1 - [DNLZ_MOUSE]	12.43	1	2	2	5	1.06	1.13	0.98	0.94	0.88	1.04	1.04	1.12	1.14	0.93	0.84	0.91	0.85	19.39	9.48
O35857	Mitochondrial import inner membrane translocase subunit TIM44 OS=Mus musculus GN=Timm44 PE=2 SV=2 - [TIM44_MOUSE]	24.78	2	10	10	28	0.97	0.98	1.01	0.97	0.95	1.08	1.11	1.12	1.16	1.09	1.09	1.02	1.04	51.06	8.13
Q04886	Transcription factor SOX-8 OS=Mus musculus GN=Sox8 PE=2 SV=2 - [SOX8_MOUSE]	7.76	2	2	2	3	0.95	4.42	4.67	1.50	1.58	1.39	1.46	1.12	1.18	1.06	1.12	0.88	0.94	49.85	7.15
E9PWI6	GRIP and coiled-coil domain-containing protein 2 OS=Mus musculus GN=Gcc2 PE=2 SV=1 - [E9PWI6_MOUSE]	27.19	6	42	42	87	0.98	0.89	0.94	0.90	0.91	1.06	1.07	1.12	1.16	1.00	0.99	0.95	0.96	190.62	5.12
Q07079	Insulin-like growth factor-binding protein 5 OS=Mus musculus GN=Igfbp5 PE=1 SV=1 - [IBP5_MOUSE]	17.34	1	4	4	9	1.17	1.05	0.95	0.98	0.80	1.11	0.97	1.12	0.93	1.03	0.94	1.11	1.05	30.35	8.12
Q9QWV9	Cyclin-T1 OS=Mus musculus GN=Ccnt1 PE=1 SV=3 - [CCNT1_MOUSE]	2.21	1	1	1	1	1.02	0.64	0.63	1.00	0.98	1.08	1.06	1.12	1.10	1.06	1.05	0.88	0.87	80.55	8.68
P83741-4	Isoform 4 of Serine/threonine-protein kinase WNK1 OS=Mus musculus GN=Wnk1 - [WNK1_MOUSE]	10.90	13	16	20	43	1.03	0.87	0.88	0.97	0.96	1.16	1.21	1.12	1.18	1.16	1.16	1.12	1.15	225.27	6.51
Q501J7-2	Isoform 2 of Phosphatase and actin regulator 4 OS=Mus musculus GN=Phactr4 - [PHAR4_MOUSE]	13.04	3	5	7	20	1.15	1.08	0.93	0.98	0.83	1.23	1.04	1.12	0.98	1.01	0.96	0.95	1.00	73.36	6.38
O70230-3	Isoform 3 of Zinc finger protein 143 OS=Mus musculus GN=Znf143 - [ZN143_MOUSE]	8.69	3	2	2	3	0.72	0.60	0.83	0.63	0.88	1.19	1.64	1.12	1.56	0.98	1.37	0.85	1.18	65.99	6.32

P49312	Heterogeneous nuclear ribonucleoprotein A1 OS=Mus musculus GN=Hnrnpa1 PE=1 SV=2 - [ROA1_MOUSE]	57.19	4	13	18	299	0.96	0.85	0.90	0.87	0.91	1.07	1.11	1.12	1.20	0.98	1.03	0.92	1.00	34.18	9.23
J3QMA8	U11/U12 small nuclear ribonucleoprotein 48 kDa protein OS=Mus musculus GN=Snrnp48 PE=4 SV=1 - [J3QMA8_MOUSE]	3.65	3	1	1	2	1.35	3.88	2.87	1.24	0.92	1.16	0.85	1.12	0.83	1.04	0.77	1.14	0.85	32.07	7.55
Q3TKX1	ATPase, H+ transporting, lysosomal accessory protein OS=Mus musculus GN=Atp6ap1 PE=2 SV=1 - [Q3TKX1_MOUSE]	18.07	5	5	5	9	1.00	0.90	0.90	1.05	1.01	1.10	1.08	1.12	1.05	1.14	1.11	1.07	0.99	45.51	5.63
Q60865	Caprin-1 OS=Mus musculus GN=Caprin1 PE=1 SV=2 - [CAPR1_MOUSE]	21.07	4	11	11	76	1.02	0.76	0.73	0.95	0.90	1.17	1.09	1.12	1.08	1.06	1.01	0.95	0.90	78.12	5.25
Q8CHE4	PH domain leucine-rich repeat-containing protein phosphatase 1 OS=Mus musculus GN=Phlpp1 PE=2 SV=2 - [PHLP1_MOUSE]	6.11	1	4	4	6	0.97	1.04	1.10	0.90	0.95	1.10	1.16	1.12	1.09	1.14	1.10	0.99	1.03	182.25	6.19
Q8VIM9	Immunity-related GTPase family Q protein OS=Mus musculus GN=Irgq PE=2 SV=1 - [IRGQ_MOUSE]	20.93	1	10	10	36	0.93	1.13	1.23	1.05	1.04	1.06	1.13	1.12	1.14	1.06	1.18	1.09	1.15	59.29	4.83
Q6DIC0	Probable global transcription activator SNF2L2 OS=Mus musculus GN=Smarca2 PE=1 SV=1 - [SMCA2_MOUSE]	8.62	12	7	13	21	1.01	0.91	0.92	0.99	1.10	1.13	1.10	1.12	1.11	1.03	1.07	1.03	1.15	180.14	7.20
Q640M1	U3 small nucleolar RNA-associated protein 14 homolog A OS=Mus musculus GN=Utp14a PE=2 SV=1 - [UT14A_MOUSE]	1.56	1	1	1	2	1.08	1.13	1.05	0.99	0.92	1.07	0.99	1.12	1.03	1.06	0.98	1.06	0.98	87.21	9.20
D3YTQ3	Heterogeneous nuclear ribonucleoprotein D-like OS=Mus musculus GN=Hnrpdl PE=2 SV=1 - [D3YTQ3_MOUSE]	40.71	13	16	18	113	1.09	0.90	0.85	0.95	0.90	1.19	1.15	1.12	1.01	0.98	0.95	0.98	0.97	46.24	9.57
Q05A62-2	Isoform 2 of Dynein light chain 1, axonemal OS=Mus musculus GN=Dnal1 - [DNAL1_MOUSE]	40.91	6	5	5	8	0.89	0.66	0.74	0.85	0.97	1.08	1.20	1.12	1.27	0.95	1.09	0.95	1.09	19.87	5.49
Q68FE8	Zinc finger protein 280D OS=Mus musculus GN=Znf280d PE=2 SV=1 - [Z280D_MOUSE]	4.41	2	3	3	8	0.80	0.94	1.17	0.95	1.00	1.09	1.33	1.12	1.36	0.88	1.14	0.90	1.10	107.77	6.84

Q8K1N4-2	Isoform 2 of Spermatogenesis-associated serine-rich protein 2 OS=Mus musculus GN=Spats2 - [SPAS2_MOUSE]	11.08	2	3	3	7	0.91	1.09	1.05	0.83	0.87	1.08	1.14	1.12	1.23	1.04	1.05	0.89	0.96	39.47	6.95
H9KV15	Protein SON OS=Mus musculus GN=Son PE=2 SV=1 - [H9KV15_MOUSE]	12.33	9	17	17	36	1.01	0.97	0.93	0.98	1.00	1.09	1.14	1.12	1.17	1.10	1.12	1.12	1.08	253.88	5.40
E9Q5B2	Protein 0610011F06Rik OS=Mus musculus GN=0610011F06Rik PE=2 SV=1 - [E9Q5B2_MOUSE]	16.82	3	1	1	2	0.98	1.35	1.37	1.44	1.46	1.16	1.18	1.12	1.14	1.23	1.25	1.38	1.40	12.03	5.48
Q8BWP8-	Isoform 2 of N-acetyllactosaminide beta-1,3-N-acetylglucosaminyltransferase OS=Mus musculus GN=B3gnt1 - [B3GN1_MOUSE]	8.71	2	3	3	6	1.16	1.50	1.28	1.17	1.01	0.99	0.87	1.12	0.99	1.29	1.05	1.30	1.04	42.98	7.71
O08917	Flotillin-1 OS=Mus musculus GN=Flot1 PE=1 SV=1 - [FLOT1_MOUSE]	47.66	4	15	15	78	1.05	1.01	0.96	1.11	1.04	1.09	1.07	1.12	1.00	1.10	1.06	1.12	1.03	47.48	7.15
O35900	U6 snRNA-associated Sm-like protein LSm2 OS=Mus musculus GN=Lsm2 PE=2 SV=1 - [LSM2_MOUSE]	83.16	3	7	7	13	1.15	0.90	0.78	0.96	0.85	1.08	0.92	1.12	0.92	0.84	0.75	1.00	0.79	10.83	6.52
Q5HZI2	C2 calcium-dependent domain-containing protein 4C OS=Mus musculus GN=C2cd4cC2CD4 family PE=2 SV=1 - [C2C4C_MOUSE]	37.23	1	7	7	11	1.08	0.96	1.06	0.86	0.84	1.13	1.01	1.12	1.00	1.13	0.97	1.08	1.02	44.59	9.73
Q80VC9-2	Isoform 2 of Calmodulin-regulated spectrin-associated protein 3 OS=Mus musculus GN=Camsap3 - [CAMP3_MOUSE]	14.55	3	9	9	24	1.03	1.10	1.18	0.95	0.95	0.94	0.97	1.12	1.09	1.09	1.08	1.12	1.09	127.69	8.19
Q9D6W8	Uncharacterized protein C17orf59 homolog OS=Mus musculus PE=1 SV=1 - [CQ059_MOUSE]	25.56	1	4	4	8	0.97	1.05	1.15	0.90	0.99	1.03	1.13	1.12	1.17	1.15	1.24	1.17	1.27	37.99	5.34
Q3UHI0	Serine-rich coiled-coil domain-containing protein 2 OS=Mus musculus GN=Ccser2 PE=1 SV=1 - [CCSE2_MOUSE]	5.16	2	3	3	5	0.90	0.80	0.89	0.98	0.98	1.19	1.19	1.12	1.17	1.11	1.18	1.02	0.99	92.87	6.46
Q9Z0P4-2	Isoform 2 of Paralemmin-1 OS=Mus musculus GN=Palm - [PALM_MOUSE]	70.50	1	1	24	372	0.97	0.94	0.91	0.93	0.92	1.17	1.18	1.12	1.06	1.03	1.03	1.03	1.05	36.70	4.84

Q8CI32	BAG family molecular chaperone regulator 5 OS=Mus musculus GN=Bag5 PE=1 SV=1 - [BAG5_MOUSE]	23.94	2	7	7	15	0.98	0.89	0.91	1.05	1.08	1.08	1.03	1.12	1.11	1.13	1.18	1.17	1.14	50.91	6.05
Q5XJV6	Serine/threonine-protein kinase LMTK3 OS=Mus musculus GN=Lmtk3 PE=1 SV=1 - [LMTK3_MOUSE]	23.60	4	19	19	53	1.03	1.06	1.03	0.98	0.95	1.28	1.23	1.12	1.12	1.10	1.09	1.09	1.01	150.80	4.88
D3Z7N3	Cell growth-regulating nucleolar protein (Fragment) OS=Mus musculus GN=Lyar PE=2 SV=1 - [D3Z7N3_MOUSE]	10.53	6	2	2	9	1.02	1.00	0.97	1.03	1.05	1.04	0.99	1.12	1.18	1.26	1.20	1.12	1.08	21.60	8.69
Q9D6I9	Leucine rich adaptor protein 1 OS=Mus musculus GN=Lurap1 PE=2 SV=1 - [LURA1_MOUSE]	14.23	1	2	2	6	0.97	1.04	1.06	0.85	0.90	0.75	0.81	1.12	1.01	1.06	1.02	1.02	1.05	25.81	4.72
Q91XV3	Brain acid soluble protein 1 OS=Mus musculus GN=Basp1 PE=1 SV=3 - [BASP1_MOUSE]	91.15	1	25	25	1428	1.03	0.86	0.85	0.98	0.96	1.14	1.11	1.12	1.10	1.04	1.01	0.97	0.95	22.07	4.51
P43277	Histone H1.3 OS=Mus musculus GN=Hist1h1d PE=1 SV=2 - [H13_MOUSE]	43.44	3	2	16	81	1.04	0.67	0.65	0.97	0.93	1.13	1.08	1.12	1.08	1.10	1.06	0.99	0.95	22.09	11.03
Q9D9Z5	DET1- and DDB1-associated protein 1 OS=Mus musculus GN=Dda1 PE=2 SV=1 - [DDA1_MOUSE]	44.12	3	4	4	10	0.95	0.88	0.95	0.91	0.97	1.09	1.37	1.12	1.27	1.04	1.13	1.03	1.12	11.75	8.68
Q9ES97-2	Isoform 2 of Reticulon-3 OS=Mus musculus GN=Rtn3 - [RTN3_MOUSE]	50.79	5	37	37	153	0.97	0.94	0.98	0.95	0.97	1.14	1.16	1.12	1.16	1.02	1.05	0.97	1.00	101.86	4.93
D9HP81	Regulating synaptic membrane exocytosis 2 OS=Mus musculus GN=Rims2 PE=2 SV=1 - [D9HP81_MOUSE]	12.21	4	12	15	26	1.05	1.09	1.12	0.92	0.89	1.01	0.96	1.12	1.00	0.95	0.92	1.01	0.96	177.51	9.36
Q91Z38	Tetratricopeptide repeat protein 1 OS=Mus musculus GN=Ttc1 PE=2 SV=1 - [TTC1_MOUSE]	54.45	2	11	11	42	0.99	0.80	0.77	0.88	0.89	1.19	1.17	1.12	1.18	1.10	1.06	1.02	1.10	33.24	5.01
Q0KK56	Protein FAM184B OS=Mus musculus GN=Fam184b PE=2 SV=1 - [F184B_MOUSE]	2.34	2	1	2	3	0.89	1.20	1.35	0.92	1.03	1.06	1.19	1.12	1.27	0.99	1.12	0.79	0.90	107.53	5.78
Q9EST3-2	Isoform 2 of Eukaryotic translation initiation factor 4E transporter OS=Mus musculus GN=Eif4enif1 - [4ET_MOUSE]	7.72	5	5	5	12	1.11	1.00	0.84	0.95	0.89	1.15	1.00	1.12	1.06	1.06	1.03	1.05	0.96	105.33	7.72

D3Z641	Tetraspanin-5 OS=Mus musculus GN=Tspan5 PE=4 SV=1 - [D3Z641_MOUSE]	7.11	2	1	1	1	1.11	1.33	1.20	1.04	0.94	1.08	0.97	1.12	1.01	1.25	1.13	1.03	0.94	22.45	4.60
Q3TMX0	MCG4375, isoform CRA_b OS=Mus musculus GN=Sdcbp PE=2 SV=1 - [Q3TMX0_MOUSE]	16.78	5	2	2	7	1.21	1.53	1.26	1.29	0.95	1.10	0.91	1.12	0.90	1.27	1.08	1.27	1.12	32.23	7.15
Q69ZK6	Probable JmjC domain-containing histone demethylation protein 2C OS=Mus musculus GN=Jmjd1c PE=1 SV=3 - [JHD2C_MOUSE]	0.51	2	1	1	2	0.85	1.21	1.42	1.00	1.18	1.17	1.37	1.12	1.32	1.08	1.28	1.23	1.45	260.48	8.07
O55026-2	Isoform Short of Ectonucleoside triphosphate diphosphohydrolase 2 OS=Mus musculus GN=Entpd2 - [ENTP2_MOUSE]	31.82	2	3	3	6	1.01	1.25	1.10	1.10	0.96	0.99	0.96	1.12	0.88	1.12	1.09	1.18	0.92	13.85	6.52
Q9D6L8	Peptidyl-prolyl cis-trans isomerase-like 3 OS=Mus musculus GN=Ppil3 PE=2 SV=1 - [PPIL3_MOUSE]	8.07	1	1	1	4	1.27	0.85	0.67	0.94	0.74	1.39	1.09	1.12	0.88	1.08	0.86	1.19	0.94	18.12	6.79
O08663	Methionine aminopeptidase 2 OS=Mus musculus GN=Metap2 PE=1 SV=1 - [MAP2_MOUSE]	21.34	7	7	7	20	1.06	0.84	0.83	1.03	0.98	0.99	0.92	1.12	1.04	1.08	1.07	1.00	0.93	52.89	5.82
Q5SWP3	NAC-alpha domain-containing protein 1 OS=Mus musculus GN=Nacad PE=1 SV=1 - [NACAD_MOUSE]	19.28	2	18	18	42	0.98	0.85	0.88	0.90	0.92	1.15	1.16	1.12	1.12	1.02	1.03	0.88	0.92	156.67	4.56
Q8BKV0	Testican-3 OS=Mus musculus GN=Spock3 PE=2 SV=2 - [TICN3_MOUSE]	5.96	2	2	2	4	1.05	0.99	1.11	0.87	0.97	1.15	1.16	1.12	1.14	1.23	1.18	0.99	1.13	49.07	5.10
O08585	Clathrin light chain A OS=Mus musculus GN=Clta PE=1 SV=2 - [CLCA_MOUSE]	25.11	5	1	9	152	1.02	0.76	0.75	0.91	0.90	1.08	1.06	1.13	1.07	0.97	0.94	0.95	0.92	25.59	4.58
F7B0R9	Protein 2010300C02Rik (Fragment) OS=Mus musculus GN=2010300C02Rik PE=4 SV=1 - [F7B0R9_MOUSE]	68.56	1	1	31	158	0.96	0.84	0.88	1.07	1.11	1.14	1.19	1.13	1.17	1.03	1.08	1.05	1.10	69.48	9.39
P62317	Small nuclear ribonucleoprotein Sm D2 OS=Mus musculus GN=Snrpd2 PE=1 SV=1 - [SMD2_MOUSE]	40.68	2	5	5	22	0.96	0.89	0.98	0.88	0.96	1.12	1.06	1.13	1.18	0.98	1.09	0.98	0.99	13.52	9.91
P31786	Acyl-CoA-binding protein OS=Mus musculus GN=Dbi PE=1 SV=2 - [ACBP_MOUSE]	70.11	5	9	9	158	1.27	0.77	0.61	0.90	0.69	1.18	0.93	1.13	0.86	1.08	0.82	1.09	0.89	9.99	8.82

Q69ZL1	FYVE, RhoGEF and PH domain-containing protein 6 OS=Mus musculus GN=Fgd6 PE=1 SV=2 - [FGD6_MOUSE]	2.64	1	2	2	4	1.11	1.05	0.94	0.94	0.85	1.09	0.97	1.13	1.01	1.10	0.98	1.01	0.91	155.07	7.87
B2RQ57	Abelson tyrosine-protein kinase 2 OS=Mus musculus GN=Abl2 PE=2 SV=1 - [B2RQ57_MOUSE]	14.10	4	8	8	16	1.01	0.96	0.92	0.93	0.91	1.18	0.99	1.13	1.07	1.14	1.07	1.14	1.13	117.69	7.66
P17095	High mobility group protein HMG-I/HMG-Y OS=Mus musculus GN=Hmga1 PE=1 SV=4 - [HMGA1_MOUSE]	48.60	2	5	5	16	1.09	0.85	0.78	1.05	0.93	1.09	0.95	1.13	0.98	1.11	0.98	0.99	0.86	11.61	10.32
B2RUJ5	Amyloid beta A4 precursor protein-binding family A member 1 OS=Mus musculus GN=Apba1 PE=2 SV=2 - [APBA1_MOUSE]	27.55	2	12	13	79	0.96	1.03	1.16	0.98	1.00	1.03	1.09	1.13	1.14	0.99	1.00	1.00	1.07	92.85	4.88
P51125-3	Isoform 3 of Calpastatin OS=Mus musculus GN=Cast - [ICAL_MOUSE]	38.65	8	18	18	57	1.01	0.85	0.88	0.89	0.87	1.10	1.06	1.13	1.09	0.99	0.95	0.89	0.91	79.65	5.54
Q3UHX2	28 kDa heat- and acid-stable phosphoprotein OS=Mus musculus GN=Pdap1 PE=1 SV=1 - [HAP28_MOUSE]	42.54	1	9	9	88	0.97	0.91	0.94	0.90	0.94	1.16	1.17	1.13	1.12	1.05	1.02	0.86	0.91	20.59	7.39
Q9QYM9	Tomoregulin-2 OS=Mus musculus GN=Tmeff2 PE=2 SV=1 - [TEFF2_MOUSE]	4.55	1	2	2	4	1.02	0.84	0.80	1.07	1.01	1.16	1.13	1.13	1.11	1.12	1.10	1.05	1.00	41.39	5.15
Q8BTI8-3	Isoform 3 of Serine/arginine repetitive matrix protein 2 OS=Mus musculus GN=Srrm2 - [SRRM2_MOUSE]	21.17	3	37	37	144	1.04	1.05	0.98	1.04	0.98	1.10	1.05	1.13	1.09	1.11	1.09	1.09	1.02	284.99	12.03
E9PXC2	Upstream stimulatory factor 2 OS=Mus musculus GN=Usf2 PE=2 SV=1 - [E9PXC2_MOUSE]	7.44	5	1	1	10	0.87	1.02	1.15	0.86	0.99	1.15	1.19	1.13	1.23	1.11	1.22	1.13	1.24	24.00	5.86
Q9JK62	Potassium channel TASK2 OS=Mus musculus GN=Kcnk5 PE=2 SV=1 - [Q9JK62_MOUSE]	1.79	1	1	1	1	1.00	1.09	1.09	0.94	0.94	1.13	1.13	1.13	1.12	1.09	1.10	1.12	1.13	55.94	6.48
F7BTZ2	Protein PML (Fragment) OS=Mus musculus GN=Pml PE=4 SV=1 - [F7BTZ2_MOUSE]	5.54	6	3	3	6	0.83	1.04	1.25	0.89	1.07	1.13	1.33	1.13	1.22	1.07	1.25	1.02	1.23	64.57	5.07
Q3TRP8	Cartilage acidic protein 1 OS=Mus musculus GN=Cep68 PE=2 SV=1 - [Q3TRP8_MOUSE]	3.63	3	1	1	1	0.97	0.51	0.53	0.67	0.69	0.94	0.97	1.13	1.16	0.52	0.54	0.78	0.81	67.65	5.94

P26339	Chromogranin-A OS=Mus musculus GN=Chga PE=1 SV=1 - [CMGA_MOUSE]	31.53	1	10	10	47	1.15	0.94	0.78	0.98	0.91	1.29	1.10	1.13	0.93	1.12	1.06	0.97	0.93	51.76	4.72
Q9CQM5	Thioredoxin domain-containing protein 17 OS=Mus musculus GN=Txndc17 PE=1 SV=1 - [TXD17_MOUSE]	32.52	1	4	4	9	1.16	1.06	0.89	0.91	0.82	1.18	1.13	1.13	0.90	1.14	0.96	1.25	1.10	14.01	4.77
Q6ZWM4	N-alpha-acetyltransferase 38, NatC auxiliary subunit OS=Mus musculus GN=Naa38 PE=3 SV=3 - [NAA38_MOUSE]	78.13	1	5	5	27	1.02	0.88	0.90	0.95	0.90	1.10	1.12	1.13	1.15	1.07	1.01	1.09	1.03	10.40	4.48
Q8CBC4	Consortin OS=Mus musculus GN=Cnst PE=1 SV=1 - [CNST_MOUSE]	9.42	2	4	4	10	0.93	0.83	0.88	0.96	1.04	1.25	1.24	1.13	1.21	1.05	1.12	1.09	1.18	76.82	4.59
Q9QYG0	Protein NDRG2 OS=Mus musculus GN=Ndrg2 PE=1 SV=1 - [NDRG2_MOUSE]	69.00	1	2	15	126	0.90	0.96	1.02	1.20	1.26	1.29	1.57	1.13	1.30	1.10	1.18	1.09	1.13	40.76	5.40
P97813	Phospholipase D2 OS=Mus musculus GN=Pld2 PE=1 SV=2 - [PLD2_MOUSE]	4.07	2	1	1	1	1.38	1.24	0.89	0.97	0.70	0.89	0.64	1.13	0.81	0.85	0.62	0.81	0.59	106.10	7.39
A2ALU4	Protein Shroom2 OS=Mus musculus GN=Shroom2 PE=1 SV=1 - [SHRM2_MOUSE]	21.88	5	23	25	133	1.06	0.91	0.91	0.94	0.89	1.17	1.09	1.13	1.07	1.01	0.99	1.01	0.95	164.60	6.70
E9Q0W5	Protein FAM3C (Fragment) OS=Mus musculus GN=Fam3c PE=2 SV=1 - [E9Q0W5_MOUSE]	41.45	6	5	5	9	0.95	0.98	1.02	0.98	1.09	1.10	0.99	1.13	1.14	1.10	1.03	1.02	1.03	16.51	5.15
Q8BJ42	Disks large-associated protein 2 OS=Mus musculus GN=Dlgap2 PE=1 SV=2 - [DLGP2_MOUSE]	25.78	3	17	19	62	0.94	0.95	0.98	0.97	1.08	1.07	1.12	1.13	1.20	1.00	1.07	0.97	1.07	119.00	6.81
Q3UFS4	G patch domain-containing protein 11 OS=Mus musculus GN=Gpatch11 PE=2 SV=2 - [GPT11_MOUSE]	11.83	1	3	3	7	1.10	0.95	0.88	0.98	0.93	1.14	1.03	1.13	1.08	1.03	1.02	1.06	0.97	30.59	5.08
Q99L85	Elongator complex protein 5 OS=Mus musculus GN=Elp5 PE=2 SV=1 - [ELP5_MOUSE]	21.33	1	3	3	6	1.05	1.08	1.60	0.97	0.93	1.17	1.35	1.13	1.28	1.05	0.90	1.01	0.99	33.48	5.49
Q5NCR9	Nuclear speckle splicing regulatory protein 1 OS=Mus musculus GN=Nsrp1 PE=1 SV=1 - [NSRP1_MOUSE]	6.09	1	2	2	4	0.99	0.93	0.94	1.03	1.04	1.27	1.28	1.13	1.13	1.16	1.17	0.99	1.00	63.76	8.76

Q811L6	Microtubule-associated serine/threonine-protein kinase 4 OS=Mus musculus GN=Mast4 PE=1 SV=3 - [MAST4_MOUSE]	4.62	16	7	9	17	0.98	0.92	1.07	1.07	1.11	1.08	1.15	1.13	1.28	1.02	1.11	1.10	1.04	283.83	8.62
Q3UGC7	Eukaryotic translation initiation factor 3 subunit J-A OS=Mus musculus GN=Eif3j1 PE=2 SV=1 - [EI3JA_MOUSE]	52.87	2	15	15	80	1.00	0.94	0.97	0.94	0.93	1.11	1.16	1.13	1.12	1.07	1.06	0.97	1.01	29.33	4.81
Q8K1M6	Dynamin-1-like protein OS=Mus musculus GN=Dnm1l PE=1 SV=2 - [DNM1L_MOUSE]	53.23	3	2	32	91	0.89	1.19	1.35	1.25	1.31	1.00	1.02	1.13	1.23	0.98	1.10	1.17	1.36	82.61	7.05
Q9CQC9	GTP-binding protein SAR1b OS=Mus musculus GN=Sar1b PE=1 SV=1 - [SAR1B_MOUSE]	21.72	1	2	3	5	0.94	0.98	1.04	1.00	1.06	1.15	1.22	1.13	1.20	1.29	1.38	1.02	1.09	22.37	6.11
Q3TWW8	Protein Srsf6 OS=Mus musculus GN=Srsf6 PE=2 SV=1 - [Q3TWW8_MOUSE]	22.42	1	6	9	30	1.00	0.83	0.92	0.89	0.91	1.07	1.03	1.13	1.06	0.95	0.99	0.87	0.88	39.00	11.46
Q6IRU5-2	Isoform 2 of Clathrin light chain B OS=Mus musculus GN=Cltb - [CLCB_MOUSE]	40.76	3	1	14	114	1.04	0.80	0.77	0.93	0.90	1.22	1.16	1.13	1.08	1.04	1.00	0.84	0.81	23.16	4.68
Q61644	Protein kinase C and casein kinase substrate in neurons protein 1 OS=Mus musculus GN=Pacsin1 PE=1 SV=1 - [PACN1_MOUSE]	76.87	1	35	38	1045	1.00	0.87	0.88	0.90	0.89	1.13	1.13	1.13	1.12	1.02	1.01	0.95	0.96	50.54	5.24
Q99PV8-3	Isoform 3 of B-cell lymphoma/leukemia 11B OS=Mus musculus GN=Bcl11b - [BC11B_MOUSE]	2.17	3	1	1	1	1.04	0.95	0.91	0.93	0.89	1.33	1.28	1.13	1.09	1.38	1.33	1.51	1.46	74.13	7.69
Q3UIX4	Protein Srsf11 OS=Mus musculus GN=Srsf11 PE=2 SV=1 - [Q3UIX4_MOUSE]	11.34	8	4	4	13	1.08	1.05	1.07	0.95	0.97	1.19	1.14	1.13	1.06	1.02	1.02	1.17	1.26	53.07	10.58
O55042-2	Isoform 2 of Alpha-synuclein OS=Mus musculus GN=Snca - [SYUA_MOUSE]	76.03	2	11	15	767	1.04	0.80	0.80	0.93	0.91	1.15	1.11	1.13	1.10	1.08	1.03	1.00	0.99	12.34	9.20
P60840-2	Isoform 2 of Alpha-endosulfine OS=Mus musculus GN=Ensa - [ENSA_MOUSE]	74.36	3	9	10	134	1.03	0.97	0.93	0.94	0.92	1.12	1.12	1.13	1.11	1.04	1.03	0.96	0.98	12.91	8.27
P70174	Histamine H1 receptor OS=Mus musculus GN=Hrh1 PE=2 SV=2 - [HRH1_MOUSE]	4.71	1	2	2	3	1.09	1.42	1.30	1.07	0.98	1.10	1.00	1.13	1.03	1.18	1.08	1.21	1.11	55.65	9.32
E9Q9K8	Protein Akap6 OS=Mus musculus GN=Akap6 PE=2 SV=1 - [E9Q9K8_MOUSE]	0.74	1	1	1	2	0.92	0.73	0.79	0.84	0.91	1.09	1.18	1.13	1.22	0.94	1.02	0.66	0.72	254.17	5.06

Q7TMF3	NADH dehydrogenase [ubiquinone] 1 alpha subcomplex subunit 12 OS=Mus musculus GN=Ndufa12 PE=1 SV=2 - [NDUAC_MOUSE]	79.31	2	9	9	35	1.05	0.99	0.93	1.07	1.08	1.06	1.01	1.13	1.13	1.07	1.00	1.15	1.10	17.08	9.36
Q9CWE0	Mitochondrial fission regulator 1-like OS=Mus musculus GN=Mtfr1l PE=1 SV=1 - [MFR1L_MOUSE]	44.64	5	10	10	34	0.99	0.85	0.83	0.92	0.96	1.05	1.05	1.13	1.17	0.92	1.05	1.04	1.02	31.71	6.10
P17751	Triosephosphate isomerase OS=Mus musculus GN=Tpi1 PE=1 SV=4 - [TPIS_MOUSE]	68.56	2	23	23	763	0.95	0.79	0.84	0.89	0.93	1.08	1.15	1.13	1.19	0.98	1.05	0.96	1.01	32.17	5.74
G3X9S1	Protein FAM13C OS=Mus musculus GN=Fam13c PE=4 SV=1 - [G3X9S1_MOUSE]	22.74	4	9	10	15	1.06	1.00	0.87	0.95	0.88	1.10	1.06	1.13	1.09	1.03	1.04	1.09	1.03	57.87	6.73
Q6P1B9	Bin1 protein OS=Mus musculus GN=Bin1 PE=2 SV=1 - [Q6P1B9_MOUSE]	63.94	2	1	28	605	0.97	0.78	0.85	0.90	0.93	1.07	1.19	1.13	1.16	0.93	0.95	0.89	1.00	52.73	5.24
B9EKI3	TATA element modulatory factor OS=Mus musculus GN=Tmf1 PE=1 SV=2 - [TMF1_MOUSE]	20.16	2	19	19	34	1.01	1.02	0.96	0.90	0.94	1.05	1.07	1.13	1.15	1.00	1.01	0.92	0.93	121.73	4.87
Q9D385-2	Isoform 2 of ADP-ribosylation factor-like protein 2-binding protein OS=Mus musculus GN=Arl2bp - [AR2BP_MOUSE]	23.68	2	3	3	7	1.08	0.94	0.95	0.93	0.96	1.03	1.02	1.13	1.16	1.00	0.98	1.04	1.07	17.56	4.50
Q6PE13	Proline-rich transmembrane protein 3 OS=Mus musculus GN=Prrt3 PE=1 SV=1 - [PRRT3_MOUSE]	23.89	1	12	12	47	1.06	1.12	1.08	1.13	1.09	1.16	1.11	1.13	1.14	1.13	1.16	1.07	1.04	101.16	7.52
Q99JB8	Protein kinase C and casein kinase II substrate protein 3 OS=Mus musculus GN=Pacsin3 PE=1 SV=1 - [PACN3_MOUSE]	36.79	8	10	10	37	0.89	1.00	1.12	0.94	1.00	1.15	1.24	1.13	1.35	1.05	1.19	0.97	1.03	48.55	6.10
Q62356	Follistatin-related protein 1 OS=Mus musculus GN=Fstl1 PE=1 SV=2 - [FSTL1_MOUSE]	8.50	1	2	2	3	0.99	1.12	1.14	0.92	0.94	1.06	1.07	1.13	1.14	0.99	1.00	0.97	0.98	34.53	5.85
Q8BL97-3	Isoform 3 of Serine/arginine-rich splicing factor 7 OS=Mus musculus GN=Srsf7 - [SRSF7_MOUSE]	52.23	4	9	10	47	1.04	1.05	0.92	0.95	0.93	1.03	1.01	1.13	1.05	1.04	0.94	0.98	1.00	17.88	9.47

Q9D0W5	Peptidyl-prolyl cis-trans isomerase-like 1 OS=Mus musculus GN=Ppil1 PE=2 SV=1 - [PPIL1_MOUSE]	31.33	1	4	4	10	1.04	1.19	1.04	1.06	0.99	1.08	1.06	1.13	1.07	1.11	1.06	1.14	1.05	18.23	7.99
Q8BL66	Early endosome antigen 1 OS=Mus musculus GN=Eea1 PE=1 SV=2 - [EEA1_MOUSE]	59.39	1	80	80	349	1.05	0.84	0.81	0.93	0.89	1.17	1.09	1.13	1.06	1.03	0.98	0.96	0.92	160.82	5.77
D3Z7B8	Collagen alpha-1(XIII) chain OS=Mus musculus GN=Col13a1 PE=2 SV=1 - [D3Z7B8_MOUSE]	2.22	2	1	1	2	1.03	0.83	0.80	0.92	0.89	1.03	0.99	1.13	1.09	1.08	1.05	1.01	0.98	65.58	9.00
A2A7H6	F-box only protein 44 OS=Mus musculus GN=Fbxo44 PE=4 SV=1 - [A2A7H6_MOUSE]	6.70	1	1	1	1	0.90	0.81	0.91	0.96	1.07	1.13	1.26	1.13	1.26	1.17	1.31	1.04	1.16	25.68	7.80
F6V8Y6	Amyloid beta A4 precursor protein-binding family B member 2 (Fragment) OS=Mus musculus GN=Apbb2 PE=4 SV=1 - [F6V8Y6_MOUSE]	5.76	4	1	1	1	1.15	1.48	1.29	1.05	0.92	1.83	1.59	1.13	0.98	1.13	0.99	1.17	1.02	26.60	5.02
Q9JM13	Rab5 GDP/GTP exchange factor OS=Mus musculus GN=Rabgef1 PE=1 SV=1 - [RABX5_MOUSE]	17.72	3	8	8	20	0.92	0.79	0.89	0.94	1.03	1.08	1.20	1.13	1.21	1.06	1.15	0.99	1.06	56.83	6.79
Q8VDS4	Regulation of nuclear pre-mRNA domain-containing protein 1A OS=Mus musculus GN=Rprd1a PE=2 SV=1 - [RPR1A_MOUSE]	5.13	1	1	1	2	0.96	0.82	0.85	1.03	1.07	0.97	1.00	1.13	1.17	1.05	1.09	1.13	1.18	35.68	7.58
Q9Z0F7	Gamma-synuclein OS=Mus musculus GN=Sncg PE=1 SV=1 - [SYUG_MOUSE]	76.42	1	8	9	50	1.32	0.98	0.69	0.95	0.71	1.14	0.84	1.13	0.88	1.45	1.09	1.25	0.93	13.15	4.65
P61082	NEDD8-conjugating enzyme Ubc12 OS=Mus musculus GN=Ube2m PE=2 SV=1 - [UBC12_MOUSE]	40.44	5	7	7	18	0.94	1.09	1.12	0.99	1.07	1.13	1.19	1.13	1.24	1.11	1.12	1.10	1.14	20.89	7.69
F8WHU8	SRA stem-loop-interacting RNA-binding protein, mitochondrial (Fragment) OS=Mus musculus GN=Slirp PE=2 SV=1 - [F8WHU8_MOUSE]	61.76	4	4	4	11	0.96	0.85	0.90	0.91	1.01	1.13	1.24	1.13	1.33	1.09	1.30	0.92	1.12	11.44	9.41
Q02819	Nucleobindin-1 OS=Mus musculus GN=Nucb1 PE=1 SV=2 - [NUCB1_MOUSE]	66.88	4	31	31	169	1.04	0.96	0.94	0.93	0.89	1.12	1.10	1.13	1.08	1.05	1.02	0.99	0.95	53.38	5.07

Q3UN02	Lysocardiolipin acyltransferase 1 OS=Mus musculus GN=Lclat1 PE=2 SV=2 - [LCLT1_MOUSE]	5.05	1	2	2	3	0.93	1.26	1.59	1.27	1.36	0.88	0.94	1.13	1.43	0.87	0.93	1.05	1.12	44.37	8.53
J3QPS8	Eukaryotic translation initiation factor 5A-1 OS=Mus musculus GN=Eif5a PE=4 SV=1 - [J3QPS8_MOUSE]	88.16	1	6	6	157	1.05	1.07	1.00	0.85	0.84	1.04	1.05	1.13	1.04	1.05	1.00	1.00	0.97	8.43	4.41
Q62418-3	Isoform 3 of Drebrin-like protein OS=Mus musculus GN=Dbnl - [DBNL_MOUSE]	51.39	3	17	17	189	1.01	0.90	0.91	0.90	0.90	1.15	1.14	1.13	1.13	1.07	1.05	1.01	1.00	48.31	4.92
Q61475	Complement decay-accelerating factor, GPI-anchored OS=Mus musculus GN=Cd55 PE=2 SV=2 - [DAF1_MOUSE]	3.08	1	1	1	2	0.95	0.89	0.93	1.00	1.05	1.02	1.07	1.13	1.18	1.15	1.21	1.15	1.21	42.59	8.40
Q9Z0H8	CAP-Gly domain-containing linker protein 2 OS=Mus musculus GN=Clip2 PE=1 SV=2 - [CLIP2_MOUSE]	60.17	2	49	59	323	1.01	0.91	0.93	0.91	0.92	1.14	1.13	1.13	1.13	1.02	1.01	0.93	0.91	115.84	6.48
B7ZCR6	Protein Znf512b OS=Mus musculus GN=Znf512b PE=2 SV=1 - [B7ZCR6_MOUSE]	5.87	3	3	3	5	0.95	0.93	0.98	1.08	1.08	1.08	1.13	1.13	1.19	1.31	1.38	1.14	1.14	95.27	9.77
Q9WTX2	Interferon-inducible double stranded RNA-dependent protein kinase activator A OS=Mus musculus GN=Prkra PE=1 SV=1 - [PRKRA_MOUSE]	10.54	1	3	3	6	0.84	0.93	0.95	0.84	0.95	1.03	1.16	1.13	1.33	1.12	1.15	1.07	1.26	34.35	8.43
Q8CH25-2	Isoform 2 of SAFB-like transcription modulator OS=Mus musculus GN=Sltm - [SLTM_MOUSE]	18.56	2	17	17	43	1.01	0.89	0.91	0.96	1.00	1.10	1.09	1.13	1.17	0.99	1.00	0.96	0.95	115.11	8.05
Q61017	Guanine nucleotide-binding protein G(I)/G(S)/G(O) subunit gamma-T2 OS=Mus musculus GN=Gngt2 PE=2 SV=2 - [GBGT2_MOUSE]	10.14	1	1	1	2	1.22	0.87	0.72	1.04	0.85	1.08	0.88	1.13	0.93	1.14	0.93	1.11	0.91	7.80	5.52
Q9WU56-	Isoform 4 of tRNA pseudouridine synthase A, mitochondrial OS=Mus musculus GN=Pus1 - [TRUA_MOUSE]	3.75	5	1	1	2	1.10	2.34	2.12	0.99	0.90	1.14	1.03	1.13	1.02	0.93	0.85	1.07	0.97	38.90	8.13
Q8VC91	Ikbkg protein OS=Mus musculus GN=Ikbkg PE=2 SV=1 - [Q8VC91_MOUSE]	22.38	12	7	8	15	0.95	0.97	0.97	0.95	0.91	1.09	1.10	1.13	1.17	1.03	1.03	0.99	0.95	47.81	5.85

Q9QYC0-	Isoform 2 of Alpha-adducin OS=Mus musculus GN=Add1 - [ADDA_MOUSE]	54.75	6	1	25	222	1.20	0.93	0.78	0.92	0.76	1.09	0.91	1.13	0.94	1.06	0.88	1.15	0.96	69.84	6.51
Q8R326	Paraspeckle component 1 OS=Mus musculus GN=Pspc1 PE=1 SV=1 - [PSPC1_MOUSE]	46.27	3	17	17	52	0.97	0.94	0.95	0.91	0.93	1.04	1.08	1.13	1.20	0.89	0.96	0.91	0.96	58.72	6.67
Q6NZL0	Protein SOGA3 OS=Mus musculus GN=Soga3 PE=2 SV=2 - [SOGA3_MOUSE]	31.96	1	25	26	75	1.01	1.07	1.11	1.07	1.04	1.06	1.08	1.13	1.13	1.08	1.07	1.06	1.09	103.42	6.14
O88878	AN1-type zinc finger protein 5 OS=Mus musculus GN=Zfand5 PE=1 SV=1 - [ZFAN5_MOUSE]	24.41	3	2	3	7	1.01	1.23	1.27	0.88	0.89	1.32	1.30	1.13	1.12	1.11	1.15	1.21	1.24	23.04	8.51
Q9EQT6	Synaptotagmin-13 OS=Mus musculus GN=Syt13 PE=1 SV=1 - [SYT13_MOUSE]	3.52	1	1	1	2	1.01	1.10	1.08	1.21	1.19	1.32	1.30	1.13	1.11	1.29	1.27	1.03	1.02	46.84	7.53
Q9QYY0	GRB2-associated-binding protein 1 OS=Mus musculus GN=Gab1 PE=1 SV=2 - [GAB1_MOUSE]	21.15	1	11	11	25	0.97	0.98	1.03	0.91	0.90	1.10	1.12	1.13	1.12	1.08	1.07	1.08	1.14	76.76	5.67
Q3UFY8	Mitochondrial ribonuclease P protein 1 OS=Mus musculus GN=Trmt10c PE=2 SV=2 - [MRRP1_MOUSE]	7.73	1	2	2	3	1.08	0.93	0.85	0.91	0.84	1.00	0.92	1.13	1.04	0.93	0.86	1.09	1.00	48.36	9.38
Q9R0P9	Ubiquitin carboxyl-terminal hydrolase isozyme L1 OS=Mus musculus GN=Uchl1 PE=1 SV=1 - [UCHL1_MOUSE]	94.17	5	19	20	1749	0.96	0.75	0.78	0.88	0.91	1.10	1.13	1.13	1.17	1.04	1.08	0.98	1.02	24.82	5.24
E9Q0S6	Protein Tns1 OS=Mus musculus GN=Tns1 PE=2 SV=1 - [E9Q0S6_MOUSE]	9.59	1	10	11	24	0.94	1.00	0.98	0.90	1.00	1.03	1.11	1.13	1.20	1.03	1.11	0.98	1.15	201.26	7.75
Q9CU62	Structural maintenance of chromosomes protein 1A OS=Mus musculus GN=Smc1a PE=1 SV=4 - [SMC1A_MOUSE]	5.11	1	5	5	9	1.02	0.82	0.90	0.92	0.89	1.09	1.06	1.13	1.10	1.10	1.08	1.10	1.10	143.15	7.64
O35711-4	Isoform 4 of Liprin-beta-2 OS=Mus musculus GN=Ppfibp2 - [LIPB2_MOUSE]	5.20	4	2	2	3	1.09	1.23	1.13	0.96	0.88	1.31	1.19	1.13	1.03	1.50	1.38	1.62	1.49	101.08	6.15
P52503	NADH dehydrogenase [ubiquinone] iron-sulfur protein 6, mitochondrial OS=Mus musculus GN=Ndufs6 PE=1 SV=2 - [NDUS6_MOUSE]	64.66	2	8	8	399	1.01	0.91	0.97	0.93	0.96	1.12	1.13	1.13	1.13	1.05	1.05	0.97	1.00	13.01	8.65

P49586	Choline-phosphate cytidylyltransferase A OS=Mus musculus GN=Pcyt1a PE=1 SV=1 - [PCY1A_MOUSE]	11.17	2	2	3	5	0.97	1.29	1.33	0.97	1.00	1.03	1.06	1.13	1.17	0.94	0.97	0.95	0.98	41.64	7.03
F8VPR5	CREB-binding protein OS=Mus musculus GN=Crebbp PE=2 SV=1 - [F8VPR5_MOUSE]	4.01	2	4	6	11	0.98	1.14	1.18	0.95	0.87	1.20	1.05	1.13	1.09	1.19	1.09	1.19	1.19	265.40	8.50
Q03517	Secretogranin-2 OS=Mus musculus GN=Scg2 PE=1 SV=1 - [SCG2_MOUSE]	56.73	1	31	31	213	1.58	0.95	0.62	0.91	0.56	1.15	0.72	1.13	0.70	1.41	0.92	1.46	0.91	70.60	4.75
Q8VHL1	Histone-lysine N-methyltransferase SETD7 OS=Mus musculus GN=Setd7 PE=2 SV=2 - [SETD7_MOUSE]	57.92	1	14	14	191	0.93	0.87	0.97	0.86	0.91	1.13	1.25	1.13	1.20	0.99	1.10	0.91	1.00	40.48	4.65
Q9CYG7	Mitochondrial import receptor subunit TOM34 OS=Mus musculus GN=Tomm34 PE=2 SV=1 - [TOM34_MOUSE]	36.25	1	1	7	22	0.86	1.04	1.20	0.93	1.07	1.00	1.15	1.13	1.31	1.13	1.30	1.01	1.17	34.26	9.14
Q8K039	Uncharacterized protein KIAA1143 homolog OS=Mus musculus PE=1 SV=1 - [K1143_MOUSE]	33.55	1	4	4	9	1.06	0.87	0.81	0.88	1.01	1.03	1.02	1.13	0.94	1.04	0.97	0.83	1.11	17.47	8.48
E9PWR7	Ecotropic viral integration site 5 protein (Fragment) OS=Mus musculus GN=Evi5 PE=2 SV=1 - [E9PWR7_MOUSE]	2.42	3	1	1	2	1.10	1.08	0.98	1.14	1.04	0.93	0.84	1.13	1.03	1.23	1.12	1.22	1.11	76.58	7.20
Q791N7	DNA-directed RNA polymerase I subunit RPA12 OS=Mus musculus GN=Znrd1 PE=2 SV=1 - [RPA12_MOUSE]	17.89	1	1	1	2	0.91	1.08	1.18	0.80	0.88	0.99	1.09	1.13	1.24	0.96	1.06	0.88	0.96	13.64	5.27
Q8C4B4	Protein unc-119 homolog B OS=Mus musculus GN=Unc119b PE=2 SV=1 - [U119B_MOUSE]	7.97	1	2	2	2	1.08	1.18	1.08	1.12	1.04	1.13	1.04	1.13	1.04	1.13	1.04	1.22	1.13	28.29	5.72
A2BH40	AT-rich interactive domain-containing protein 1A OS=Mus musculus GN=Arid1a PE=1 SV=1 - [ARI1A_MOUSE]	6.66	5	8	8	18	1.02	0.88	0.96	0.99	0.99	1.10	1.09	1.13	1.08	0.96	0.97	1.03	1.10	241.94	6.68
B8JJI4	Vesicle transport protein SEC20 OS=Mus musculus GN=Bnip1 PE=2 SV=1 - [B8JJI4_MOUSE]	5.15	2	1	1	1	1.09	1.36	1.25	1.12	1.02	0.96	0.87	1.13	1.03	1.16	1.06	1.27	1.16	22.38	8.70
Q99PG2-2	Isoform 2 of Opioid growth factor receptor OS=Mus musculus GN=Ogfr - [OGFR_MOUSE]	32.83	2	9	9	17	1.00	1.00	1.08	0.97	0.99	1.13	1.14	1.13	1.15	1.21	1.18	1.11	1.13	66.70	4.84

Q9CU65	Zinc finger MYM-type protein 2 OS=Mus musculus GN=Zmym2 PE=2 SV=3 - [ZMYM2_MOUSE]	0.94	1	1	1	2	0.99	1.11	1.12	0.94	0.95	1.18	1.18	1.13	1.14	1.11	1.12	1.18	1.20	154.54	6.37
Q9D8Y8-	Isoform 3 of Inhibitor of growth protein 5 OS=Mus musculus GN=Ing5 - [ING5_MOUSE]	7.79	3	1	1	3	1.16	1.44	1.24	0.95	0.82	1.22	1.05	1.13	0.97	0.95	0.82	0.98	0.84	17.78	8.53
E9PUL5	Proline-rich transmembrane protein 2 OS=Mus musculus GN=Prrt2 PE=1 SV=1 - [PRRT2_MOUSE]	42.77	1	8	8	87	0.99	0.97	0.95	0.95	0.93	1.17	1.10	1.13	1.21	1.04	1.01	0.97	0.96	35.90	4.63
Q8BFW7	Lipoma-preferred partner homolog OS=Mus musculus GN=Lpp PE=1 SV=1 - [LPP_MOUSE]	46.82	5	15	15	64	1.03	0.90	0.85	0.96	0.93	1.09	1.10	1.13	1.09	1.12	1.13	1.04	1.01	65.85	7.37
Q8C2S5-3	Isoform 3 of F-box/LRR-repeat protein 5 OS=Mus musculus GN=Fbxl5 - [FBXL5_MOUSE]	1.77	4	1	1	3	0.96	1.23	1.19	0.98	0.98	0.95	0.98	1.13	1.02	0.95	1.01	1.20	1.22	70.88	5.34
P99027	60S acidic ribosomal protein P2 OS=Mus musculus GN=Rplp2 PE=1 SV=3 - [RLA2_MOUSE]	93.91	1	10	10	152	1.02	0.90	0.91	0.90	0.91	1.09	1.13	1.13	1.06	1.03	0.99	0.94	0.98	11.64	4.54
P63147	Ubiquitin-conjugating enzyme E2 B OS=Mus musculus GN=Ube2b PE=1 SV=1 - [UBE2B_MOUSE]	11.18	1	1	1	5	1.01	1.02	1.00	0.81	0.89	0.82	0.85	1.13	1.06	0.84	0.99	0.77	0.81	17.30	5.01
Q9CRC3	UPF0235 protein C15orf40 homolog OS=Mus musculus PE=2 SV=1 - [CO040_MOUSE]	30.16	1	3	3	7	1.18	0.81	0.67	0.96	0.82	1.19	1.01	1.13	0.96	1.22	1.01	1.09	0.90	13.18	9.16
Q8K0Z7	Translational activator of cytochrome c oxidase 1 OS=Mus musculus GN=Taco1 PE=2 SV=1 - [TACO1_MOUSE]	13.61	1	3	3	7	1.02	0.78	0.81	1.03	1.04	1.14	1.16	1.13	1.24	1.04	1.14	1.02	1.07	32.29	8.12
P34022	Ran-specific GTPase-activating protein OS=Mus musculus GN=Ranbp1 PE=1 SV=2 - [RANG_MOUSE]	64.04	2	8	8	97	1.05	0.81	0.83	0.97	0.90	1.17	1.13	1.13	1.13	1.04	1.02	1.02	0.97	23.58	5.22
P17141	Zinc finger protein 37 OS=Mus musculus GN=Zfp37 PE=2 SV=4 - [ZFP37_MOUSE]	10.94	152	4	5	10	0.97	1.07	1.05	1.02	1.02	1.18	1.16	1.13	1.02	1.18	1.18	1.14	0.99	67.18	8.82
Q8K135	Dyslexia-associated protein KIAA0319-like protein OS=Mus musculus GN=Kiaa0319l PE=1 SV=1 - [K319L_MOUSE]	3.34	2	3	3	4	0.98	1.44	1.68	1.12	1.14	1.02	1.08	1.13	1.00	1.11	1.05	1.00	0.98	115.24	6.16

D3Z7B7	Protein 4632428N05Rik OS=Mus musculus GN=4632428N05Rik PE=2 SV=1 - [D3Z7B7_MOUSE]	4.85	3	1	1	6	1.01	1.04	1.10	0.89	0.93	1.09	1.07	1.13	1.12	1.09	1.08	1.11	1.07	24.85	6.95
P32300	Delta-type opioid receptor OS=Mus musculus GN=Oprd1 PE=1 SV=1 - [OPRD_MOUSE]	4.30	1	1	1	1	1.11	1.48	1.32	0.89	0.80	1.08	0.96	1.13	1.01	1.10	0.99	1.06	0.95	40.53	9.22
Q9D6J3	Coiled-coil domain-containing protein 94 OS=Mus musculus GN=Ccdc94 PE=1 SV=1 - [CCD94_MOUSE]	20.06	1	4	5	12	1.15	0.81	0.89	0.93	0.77	1.17	1.23	1.13	1.00	1.11	0.97	1.11	0.97	35.97	6.07
Q9D1Q1	M-phase phosphoprotein 6 OS=Mus musculus GN=Mphosph6 PE=1 SV=1 - [MPH6_MOUSE]	7.45	1	1	1	10	1.13	1.41	1.22	0.96	0.93	1.13	1.02	1.13	1.08	1.06	1.02	0.94	0.96	19.07	5.30
P16254	Signal recognition particle 14 kDa protein OS=Mus musculus GN=Srp14 PE=1 SV=1 - [SRP14_MOUSE]	20.00	2	2	2	6	1.04	0.95	1.09	1.06	1.02	1.12	1.05	1.13	1.09	1.08	1.04	1.19	1.17	12.50	10.17
P84228	Histone H3.2 OS=Mus musculus GN=Hist1h3b PE=1 SV=2 - [H32_MOUSE]	59.56	2	2	9	61	1.21	1.31	1.08	0.93	0.77	1.07	0.92	1.13	0.83	1.32	0.82	1.10	0.91	15.38	11.27
Q9QYE3	B-cell lymphoma/leukemia 11A OS=Mus musculus GN=Bcl11a PE=1 SV=1 - [BC11A_MOUSE]	19.02	7	5	8	17	0.95	0.94	0.94	1.05	0.99	1.14	1.11	1.13	1.20	1.06	1.07	0.98	0.96	83.80	6.28
Q9WV02	RNA-binding motif protein, X chromosome OS=Mus musculus GN=Rbmx PE=1 SV=1 - [RBMX_MOUSE]	40.15	4	4	16	68	0.92	1.02	1.37	0.97	1.02	1.02	1.20	1.13	1.37	1.20	1.32	1.02	1.21	42.28	10.05
Q5SXC9	28S ribosomal protein S23, mitochondrial OS=Mus musculus GN=Mrps23 PE=2 SV=1 - [Q5SXC9_MOUSE]	11.00	4	1	1	1	0.73	1.09	1.49	1.13	1.55	1.05	1.43	1.13	1.55	1.10	1.52	0.91	1.24	11.66	10.05
Q8K3A0	Iron-sulfur cluster co-chaperone protein HscB, mitochondrial OS=Mus musculus GN=Hscb PE=2 SV=2 - [HSC20_MOUSE]	32.48	1	5	5	22	1.02	0.91	0.94	0.90	0.88	1.09	1.08	1.13	1.12	1.00	1.05	0.99	1.00	26.63	7.37
Q9ET80	Junctophilin-1 OS=Mus musculus GN=Jph1 PE=1 SV=1 - [JPH1_MOUSE]	8.94	1	4	4	8	0.93	1.11	1.14	1.03	1.10	1.29	1.37	1.13	1.19	1.04	1.05	0.98	1.02	71.86	9.22
Q8VDZ4	Palmitoyltransferase ZDHHC5 OS=Mus musculus GN=Zdhhc5 PE=1 SV=1 - [ZDHC5_MOUSE]	11.89	2	6	7	29	1.00	1.05	1.03	1.00	0.98	1.12	1.11	1.13	1.05	1.10	1.11	1.09	1.01	77.45	9.01

Q8VDI7	Ubiquitin-associated domain-containing protein 1 OS=Mus musculus GN=Ubac1 PE=2 SV=2 - [UBAC1_MOUSE]	16.38	3	6	6	16	0.97	0.95	1.07	0.94	1.00	1.11	1.14	1.14	1.26	1.05	1.10	1.06	1.10	45.50	4.97
Q8BHE3	Caytaxin OS=Mus musculus GN=Atcay PE=1 SV=1 - [ATCAY_MOUSE]	24.19	1	6	6	20	0.98	1.11	1.06	1.10	1.23	0.97	1.09	1.14	1.04	1.09	1.10	1.07	1.10	42.15	4.68
Q62407-2	Isoform 2 of Striated muscle-specific serine/threonine-protein kinase OS=Mus musculus GN=Speg - [SPEG_MOUSE]	8.59	5	5	5	10	1.17	1.37	1.11	0.98	0.86	1.31	1.11	1.14	1.03	1.22	0.97	1.12	0.96	93.63	9.55
H3BKH3	DNA-binding protein SATB2 OS=Mus musculus GN=Satb2 PE=2 SV=1 - [H3BKH3_MOUSE]	9.76	3	4	5	9	0.91	1.12	1.23	1.07	1.26	1.10	1.20	1.14	1.25	0.96	1.03	0.93	1.00	69.10	6.57
Q8R2Y8	Peptidyl-tRNA hydrolase 2, mitochondrial OS=Mus musculus GN=Ptrh2 PE=2 SV=1 - [PTH2_MOUSE]	24.86	1	3	3	6	1.19	0.85	0.67	1.03	1.02	1.25	1.17	1.14	1.04	1.22	1.04	1.04	1.07	19.51	7.42
P42703	Leukemia inhibitory factor receptor OS=Mus musculus GN=Lifr PE=1 SV=1 - [LIFR_MOUSE]	5.04	1	3	3	4	1.01	0.88	0.96	0.92	0.92	1.16	1.15	1.14	1.12	1.04	1.14	0.88	0.91	122.50	6.04
Q8CFV4	Neuritin OS=Mus musculus GN=Nrn1 PE=1 SV=1 - [NRN1_MOUSE]	13.38	2	2	2	14	0.94	0.94	1.17	0.99	1.19	1.04	1.19	1.14	1.10	1.11	1.07	0.99	1.18	15.34	6.99
P63276	40S ribosomal protein S17 OS=Mus musculus GN=Rps17 PE=1 SV=2 - [RS17_MOUSE]	55.56	1	6	6	18	1.02	0.84	0.79	1.05	0.95	1.08	0.98	1.14	0.94	1.06	1.07	1.02	1.03	15.51	9.85
Q03958	Prefoldin subunit 6 OS=Mus musculus GN=Pfdn6 PE=2 SV=1 - [PFD6_MOUSE]	51.18	1	1	9	58	1.05	0.53	0.51	0.85	0.80	1.20	1.13	1.14	1.07	1.07	1.02	0.83	0.79	14.45	8.88
Q3UGN9	Signal transducing adapter molecule 1 OS=Mus musculus GN=Stam PE=2 SV=1 - [Q3UGN9_MOUSE]	60.17	4	18	20	93	0.95	0.78	0.84	0.88	0.91	1.20	1.19	1.14	1.12	1.04	1.08	1.00	1.04	51.11	4.87
Q9D0I4	Syntaxin-17 OS=Mus musculus GN=Stx17 PE=1 SV=1 - [STX17_MOUSE]	23.26	2	1	4	6	1.12	1.14	1.02	0.97	0.87	1.27	1.13	1.14	1.01	1.25	1.12	1.15	1.03	33.20	6.74
Q78T54-2	Isoform 2 of Vacuolar ATPase assembly integral membrane protein VMA21 OS=Mus musculus GN=Vma21 - [VMA21_MOUSE]	14.46	2	1	1	2	1.25	1.41	1.13	1.13	0.90	0.88	0.70	1.14	0.91	0.98	0.79	1.28	1.03	9.33	9.99

E9Q355-3	Isoform 3 of Cation channel sperm-associated protein subunit gamma 1 OS=Mus musculus GN=Catsperg1 - [CTSG1_MOUSE]	3.88	3	1	1	2	1.13	0.87	0.76	1.36	1.20	1.10	0.96	1.14	1.00	1.15	1.02	0.97	0.86	91.66	7.58
Q9JK48	Endophilin-B1 OS=Mus musculus GN=Sh3glb1 PE=1 SV=1 - [SHLB1_MOUSE]	26.85	2	8	9	23	0.84	1.00	1.12	0.88	1.06	1.03	1.17	1.14	1.32	1.00	1.17	1.03	1.16	40.83	6.04
Q8BG75	Transmembrane protein 198 OS=Mus musculus GN=Tmem198 PE=2 SV=1 - [TM198_MOUSE]	3.61	1	2	2	3	0.84	1.04	1.23	1.02	1.20	1.03	1.21	1.14	1.34	1.07	1.27	0.98	1.16	39.73	9.92
Q6NVE9	Protein phosphatase PTC7 homolog OS=Mus musculus GN=Pptc7 PE=2 SV=1 - [PPTC7_MOUSE]	14.84	2	2	2	7	0.79	0.81	1.03	0.84	1.06	1.01	1.28	1.14	1.44	0.93	1.18	1.08	1.37	33.03	5.27
Q62523	Zyxin OS=Mus musculus GN=Zyx PE=1 SV=2 - [ZYX_MOUSE]	36.52	1	15	15	96	1.02	1.01	1.01	0.91	0.87	1.17	1.13	1.14	1.08	1.07	1.07	0.99	0.99	60.51	6.40
P48678	Prelamin-A/C OS=Mus musculus GN=Lmna PE=1 SV=2 - [LMNA_MOUSE]	61.95	4	40	42	525	0.98	0.98	1.02	0.89	0.93	1.07	1.09	1.14	1.17	0.98	1.01	0.92	0.94	74.19	6.98
O35621	Phosphomannomutase 1 OS=Mus musculus GN=Pmm1 PE=1 SV=1 - [PMM1_MOUSE]	33.59	3	7	8	19	0.91	0.89	0.84	0.93	0.99	1.10	1.18	1.14	1.21	0.99	1.13	1.10	1.14	29.76	5.47
Q8JZX4	Splicing factor 45 OS=Mus musculus GN=Rbm17 PE=1 SV=1 - [SPF45_MOUSE]	29.88	2	9	9	22	0.97	0.86	0.92	0.89	0.91	1.04	1.03	1.14	1.13	0.95	1.01	0.88	0.98	45.28	5.82
Q8BWJ4	Low-density lipoprotein receptor class A domain-containing protein 4 OS=Mus musculus GN=Ldlrad4 PE=2 SV=1 - [LRAD4_MOUSE]	2.94	1	1	1	1	1.20	1.49	1.24	0.96	0.80	1.06	0.88	1.14	0.95	1.12	0.93	1.11	0.93	33.84	5.45
E0CYG5	Transmembrane protein 191C OS=Mus musculus GN=Tmem191c PE=2 SV=1 - [E0CYG5_MOUSE]	18.33	3	1	1	2	1.00	1.11	1.10	0.94	0.93	1.18	1.17	1.14	1.13	1.02	1.02	1.12	1.12	6.24	4.72
Q80X41-5	Isoform 5 of Serine/threonine-protein kinase VRK1 OS=Mus musculus GN=Vrk1 - [VRK1_MOUSE]	2.90	3	1	1	2	0.94	0.99	1.06	0.86	0.92	1.05	1.12	1.14	1.21	1.04	1.11	1.05	1.13	47.20	9.11
Q8BH60-2	Isoform 2 of Golgi-associated PDZ and coiled-coil motif-containing protein OS=Mus musculus GN=Gopc - [GOPC_MOUSE]	11.43	3	3	3	37	0.86	1.05	1.25	0.95	1.06	0.96	0.99	1.14	1.34	0.99	1.10	1.10	1.09	49.76	6.35
Q8BXR9	Oxysterol-binding protein-related protein 6 OS=Mus musculus GN=Osbpl6 PE=2 SV=1 - [OSBL6_MOUSE]	7.51	3	6	6	15	0.99	0.99	1.10	1.11	1.09	1.04	1.07	1.14	1.05	1.02	1.02	1.02	1.01	108.85	7.27

P46414	Cyclin-dependent kinase inhibitor 1B OS=Mus musculus GN=Cdkn1b PE=1 SV=2 - [CDN1B_MOUSE]	49.75	1	7	7	55	0.97	1.00	1.02	0.91	0.93	1.16	1.14	1.14	1.17	1.05	1.11	0.97	1.04	22.18	7.02
P56380	Bis(5'-nucleosyl)-tetraphosphatase [asymmetrical] OS=Mus musculus GN=Nudt2 PE=1 SV=3 - [AP4A_MOUSE]	31.97	1	3	3	8	0.99	1.25	1.25	1.13	1.14	1.13	1.13	1.14	1.17	1.12	1.07	1.16	1.12	16.98	6.28
Q8BG31	Uncharacterized protein C8orf46 homolog OS=Mus musculus PE=2 SV=1 - [CH046_MOUSE]	5.80	1	1	1	2	0.96	0.67	0.69	0.94	0.97	1.13	1.17	1.14	1.18	1.05	1.09	0.88	0.92	22.48	9.95
Q3TMP1	General transcription factor IIIC, polypeptide 3 OS=Mus musculus GN=Gtf3c3 PE=2 SV=1 - [Q3TMP1_MOUSE]	0.91	1	1	1	2	1.28	0.92	0.71	0.94	0.74	1.21	0.94	1.14	0.88	1.16	0.90	0.77	0.60	100.56	5.15
P20029	78 kDa glucose-regulated protein OS=Mus musculus GN=Hspa5 PE=1 SV=3 - [GRP78_MOUSE]	59.54	1	38	41	613	1.02	0.88	0.85	0.89	0.87	1.10	1.09	1.14	1.12	1.06	1.05	0.97	0.96	72.38	5.16
P35979	60S ribosomal protein L12 OS=Mus musculus GN=Rpl12 PE=1 SV=2 - [RL12_MOUSE]	58.18	3	7	7	24	0.92	0.96	1.02	1.08	1.13	1.13	1.21	1.14	1.22	1.15	1.25	1.11	1.24	17.79	9.42
F8WHS3	Complement C1q tumor necrosis factor-related protein 5 (Fragment) OS=Mus musculus GN=C1qtnf5 PE=2 SV=1 - [F8WHS3_MOUSE]	6.73	2	1	1	2	1.34	0.76	0.57	0.77	0.58	1.05	0.78	1.14	0.85	1.28	0.96	1.73	1.30	23.19	7.40
E9QPE7	Myosin-11 OS=Mus musculus GN=Myh11 PE=4 SV=1 - [E9QPE7_MOUSE]	39.99	3	52	67	290	1.01	1.04	1.02	0.90	0.89	0.92	0.91	1.14	1.09	1.34	1.26	0.90	0.92	223.22	5.50
P0C7T6	Ataxin-1-like OS=Mus musculus GN=Atxn1l PE=1 SV=1 - [ATX1L_MOUSE]	1.89	1	1	1	2	1.00	0.98	0.98	0.99	1.00	1.18	1.18	1.14	1.13	0.99	1.00	0.91	0.91	73.33	6.49
Q99LC5	Electron transfer flavoprotein subunit alpha, mitochondrial OS=Mus musculus GN=Etfa PE=1 SV=2 - [ETFA_MOUSE]	41.14	1	10	10	24	0.98	0.85	0.83	1.00	0.98	1.18	1.15	1.14	1.09	1.17	1.14	1.11	1.14	34.99	8.38
Q3UHJ0	AP2-associated protein kinase 1 OS=Mus musculus GN=Aak1 PE=1 SV=2 - [AAK1_MOUSE]	62.98	16	38	39	257	1.01	0.92	0.94	0.96	0.94	1.12	1.11	1.14	1.14	1.04	1.07	1.01	1.03	103.28	6.70
B2RQG2	PHD finger protein 3 OS=Mus musculus GN=Phf3 PE=2 SV=1 - [B2RQG2_MOUSE]	6.96	1	10	10	17	0.99	1.09	1.11	0.99	0.98	1.16	1.11	1.14	1.15	1.04	1.02	1.02	1.05	225.40	6.70

Q9QZR0	E3 ubiquitin-protein ligase RNF25 OS=Mus musculus GN=Rnf25 PE=1 SV=2 - [RNF25_MOUSE]	23.90	5	7	7	20	0.98	0.98	1.02	0.89	0.89	1.08	1.09	1.14	1.13	0.92	1.01	1.07	1.05	51.19	6.29
Q8BLY3	Leucine-rich repeat and fibronectin type-III domain-containing protein 3 OS=Mus musculus GN=Lrfn3 PE=1 SV=1 - [LRFN3_MOUSE]	4.15	1	1	1	1	0.90	0.84	0.93	0.73	0.81	0.78	0.87	1.14	1.26	0.81	0.90	1.06	1.18	66.01	7.08
P31001	Desmin OS=Mus musculus GN=Des PE=1 SV=3 - [DESM_MOUSE]	38.17	1	12	16	74	0.99	1.01	1.14	0.95	1.00	1.28	1.33	1.14	1.20	1.08	1.15	1.01	1.05	53.47	5.27
Q5SUE3	Protein E130309D14Rik OS=Mus musculus GN=E130309D14Rik PE=4 SV=1 - [Q5SUE3_MOUSE]	4.01	1	1	1	1	0.93	1.02	1.10	0.97	1.04	1.12	1.20	1.14	1.22	1.06	1.14	1.24	1.33	33.35	7.62
Q8CFT0	RELT-like 2 OS=Mus musculus GN=Rell2 PE=2 SV=1 - [Q8CFT0_MOUSE]	33.67	8	5	5	41	1.03	1.00	1.01	0.91	0.99	1.26	1.12	1.14	1.14	1.13	1.10	0.92	0.86	31.44	6.46
P83917	Chromobox protein homolog 1 OS=Mus musculus GN=Cbx1 PE=1 SV=1 - [CBX1_MOUSE]	43.24	3	8	9	76	1.03	0.78	0.79	0.97	0.91	1.17	1.05	1.14	1.13	1.01	0.99	0.94	0.96	21.40	4.93
F7ATU7	Protein D630045J12Rik (Fragment) OS=Mus musculus GN=D630045J12Rik PE=4 SV=1 - [F7ATU7_MOUSE]	17.60	1	1	11	20	1.22	1.20	0.98	1.17	0.96	1.08	0.88	1.14	0.93	1.13	0.92	1.21	0.99	90.62	9.38
E9PXB9	Mitochondrial import inner membrane translocase subunit Tim22 OS=Mus musculus GN=Timm22 PE=2 SV=1 - [E9PXB9_MOUSE]	16.67	2	1	1	2	1.08	1.10	1.02	1.18	1.10	1.15	1.06	1.14	1.05	1.32	1.22	1.31	1.21	9.22	5.36
Q9CZ00-2	Isoform 2 of Dysbindin domain-containing protein 1 OS=Mus musculus GN=Dbndd1 - [DBND1_MOUSE]	37.17	8	3	3	10	1.18	0.80	0.82	0.86	0.72	1.23	1.03	1.14	1.04	1.02	0.94	1.06	0.94	12.46	4.91
Q6P9Q6	FK506-binding protein 15 OS=Mus musculus GN=Fkbp15 PE=1 SV=2 - [FKB15_MOUSE]	11.84	5	10	10	15	0.96	1.04	1.11	1.03	1.06	1.15	1.09	1.14	1.10	1.06	1.10	1.02	1.10	132.88	5.07
Q99K30	Epidermal growth factor receptor kinase substrate 8-like protein 2 OS=Mus musculus GN=Eps8l2 PE=1 SV=1 - [ES8L2_MOUSE]	14.40	1	8	8	22	0.85	0.84	0.95	0.87	1.03	1.00	1.19	1.14	1.39	0.77	0.92	0.71	0.88	82.18	7.18
Q9DBS5	Kinesin light chain 4 OS=Mus musculus GN=Klc4 PE=1 SV=1 - [KLC4_MOUSE]	18.74	3	4	9	18	1.10	1.03	0.93	0.98	1.01	1.09	0.98	1.14	1.03	1.17	1.06	1.13	1.03	68.57	6.09

P24472	Glutathione S-transferase A4 OS=Mus musculus GN=Gsta4 PE=1 SV=3 - [GSTA4_MOUSE]	24.77	1	5	5	14	1.12	0.94	0.98	1.06	1.00	1.08	0.88	1.14	0.97	1.14	0.93	1.10	0.99	25.55	7.39
Q91XD7	Cysteine-rich with EGF-like domain protein 1 OS=Mus musculus GN=Creld1 PE=2 SV=1 - [CREL1_MOUSE]	19.52	1	7	7	15	1.02	1.03	1.04	1.16	1.12	1.07	1.02	1.14	1.09	1.12	1.09	1.04	1.05	45.69	5.02
P17182	Alpha-enolase OS=Mus musculus GN=Eno1 PE=1 SV=3 - [ENOA_MOUSE]	77.42	5	23	32	1285	0.93	0.99	1.06	1.00	1.05	1.19	1.25	1.14	1.22	1.08	1.16	1.05	1.13	47.11	6.80
Q63912	Oligodendrocyte-myelin glycoprotein OS=Mus musculus GN=Omg PE=1 SV=1 - [OMGP_MOUSE]	27.50	2	13	13	40	0.98	0.94	0.95	0.94	0.90	1.07	1.04	1.14	1.05	1.02	0.98	1.10	1.05	49.25	8.41
A2A6K0	Troponin I, fast skeletal muscle (Fragment) OS=Mus musculus GN=Tnni2 PE=2 SV=1 - [A2A6K0_MOUSE]	22.96	3	3	3	10	0.96	1.48	1.41	0.85	0.84	18.05	20.56	1.14	1.05	1.05	1.03	1.15	1.07	15.73	8.57
Q9JIH2	Nuclear pore complex protein Nup50 OS=Mus musculus GN=Nup50 PE=1 SV=3 - [NUP50_MOUSE]	30.90	1	10	10	28	1.03	0.91	0.87	0.87	0.85	1.29	1.26	1.14	1.16	1.10	1.13	1.04	1.00	49.45	6.24
Q99K28	ADP-ribosylation factor GTPase-activating protein 2 OS=Mus musculus GN=Arfgap2 PE=1 SV=1 - [ARFG2_MOUSE]	22.50	2	12	12	25	1.00	1.03	1.05	1.01	1.03	1.15	1.13	1.14	1.16	1.08	1.10	1.07	1.00	56.56	8.18
Q497H0-2	Isoform 2 of AN1-type zinc finger protein 3 OS=Mus musculus GN=Zfand3 - [ZFAN3_MOUSE]	6.83	2	1	1	2	1.14	1.12	0.98	0.89	0.78	1.03	0.90	1.14	0.99	0.73	0.64	0.82	0.72	22.91	7.12
P08207	Protein S100-A10 OS=Mus musculus GN=S100a10 PE=2 SV=2 - [S10AA_MOUSE]	17.53	1	2	2	4	1.13	1.09	0.96	0.94	0.83	1.19	0.81	1.14	1.00	1.15	1.01	1.69	1.49	11.18	6.77
B9EJA2-2	Isoform 2 of Cortactin-binding protein 2 OS=Mus musculus GN=Cttnbp2 - [CTTB2_MOUSE]	55.87	8	35	36	105	0.97	0.87	0.90	0.87	0.93	1.10	1.16	1.14	1.18	1.02	1.04	0.95	1.02	67.51	8.22
Q8K2Q9	Shootin-1 OS=Mus musculus GN=Kiaa1598 PE=1 SV=1 - [SHOT1_MOUSE]	26.94	2	16	16	41	1.05	0.88	0.87	0.90	0.84	1.17	1.08	1.14	1.12	1.08	0.99	1.02	0.92	71.30	5.44
Q9CWW6	Peptidyl-prolyl cis-trans isomerase NIMA-interacting 4 OS=Mus musculus GN=Pin4 PE=2 SV=1 - [PIN4_MOUSE]	53.44	1	5	5	16	0.99	0.88	0.88	0.95	0.90	1.08	1.10	1.14	1.17	1.08	1.03	0.98	1.01	13.81	9.77
P62274	40S ribosomal protein S29 OS=Mus musculus GN=Rps29 PE=2 SV=2 - [RS29_MOUSE]	10.71	2	1	1	1	0.97	1.04	1.06	1.17	1.20	1.48	1.52	1.14	1.17	1.14	1.17	1.08	1.11	6.67	10.13

P51859	Hepatoma-derived growth factor OS=Mus musculus GN=Hdgf PE=1 SV=2 - [HDGF_MOUSE]	81.01	2	2	17	138	0.98	0.89	0.92	0.89	0.90	1.20	1.20	1.14	1.16	1.07	1.10	1.00	1.05	26.25	4.83
Q7TN29	Stromal membrane-associated protein 2 OS=Mus musculus GN=Smap2 PE=1 SV=1 - [SMAP2_MOUSE]	30.61	2	10	11	28	0.96	0.97	1.02	1.02	1.06	1.01	1.05	1.14	1.15	1.09	1.09	0.97	1.11	46.55	8.87
P04925	Major prion protein OS=Mus musculus GN=Prnp PE=1 SV=2 - [PRIO_MOUSE]	31.50	1	6	6	28	1.02	0.90	0.88	0.97	0.95	1.17	1.08	1.14	1.07	1.05	1.04	1.06	1.05	27.96	9.33
P03995-2	Isoform 2 of Glial fibrillary acidic protein OS=Mus musculus GN=Gfap - [GFAP_MOUSE]	56.54	2	20	22	101	1.39	1.06	0.74	1.04	0.75	1.09	0.76	1.14	0.83	1.54	1.13	1.11	0.77	49.33	5.50
P61961	Ubiquitin-fold modifier 1 OS=Mus musculus GN=Ufm1 PE=1 SV=1 - [UFM1_MOUSE]	58.82	4	4	4	12	1.05	1.14	0.93	0.87	0.92	1.07	1.10	1.14	1.17	0.83	1.14	0.73	0.72	9.11	9.31
Q9CYA0	Cysteine-rich with EGF-like domain protein 2 OS=Mus musculus GN=Creld2 PE=2 SV=1 - [CREL2_MOUSE]	18.57	1	6	6	9	1.09	0.83	0.76	1.03	0.94	1.12	1.03	1.14	1.02	1.09	0.99	1.02	0.98	38.19	4.58
O08795-2	Isoform 2 of Glucosidase 2 subunit beta OS=Mus musculus GN=Prkcsh - [GLU2B_MOUSE]	40.34	2	17	17	96	1.01	0.84	0.85	0.88	0.88	1.13	1.09	1.14	1.12	1.02	0.99	0.97	0.94	59.52	4.48
Q02614	SAP30-binding protein OS=Mus musculus GN=Sap30bp PE=2 SV=2 - [S30BP_MOUSE]	27.92	1	7	7	22	1.11	0.90	0.82	0.96	0.89	1.24	1.10	1.14	1.00	1.04	0.93	0.99	0.84	33.81	4.87
Q99MR6-	Isoform C of Serrate RNA effector molecule homolog OS=Mus musculus GN=Srrt - [SRRT_MOUSE]	20.14	4	15	15	43	0.92	0.93	1.01	0.93	0.97	1.01	1.13	1.14	1.23	0.98	1.12	1.04	1.14	99.38	5.92
A2A6M1	Vacuolar-sorting protein SNF8 OS=Mus musculus GN=Snf8 PE=2 SV=1 - [A2A6M1_MOUSE]	11.16	2	2	2	4	0.99	1.04	1.04	0.90	0.90	1.07	1.07	1.14	1.14	1.01	1.02	1.05	1.05	25.95	6.23
Q9CQ19	Myosin regulatory light polypeptide 9 OS=Mus musculus GN=Myl9 PE=1 SV=3 - [MYL9_MOUSE]	62.21	3	3	8	169	1.00	0.96	0.99	0.92	0.90	1.01	0.99	1.14	1.13	1.12	1.09	0.91	0.91	19.84	4.92
Q99MS7-	Isoform 4 of EH domain-binding protein 1-like protein 1 OS=Mus musculus GN=Ehbp1l1 - [EH1L1_MOUSE]	7.11	7	2	2	5	1.45	1.18	0.87	1.27	0.94	1.08	0.79	1.14	0.78	1.04	0.77	1.51	0.94	83.90	5.67

Q9CQT0	MCG22296, isoform CRA_b OS=Mus musculus GN=Thg1l PE=2 SV=1 - [Q9CQT0_MOUSE]	4.20	2	1	1	2	0.94	1.22	1.29	1.02	1.08	1.18	1.24	1.14	1.20	1.13	1.20	0.89	0.94	27.98	7.11
Q8R361	Rab11 family-interacting protein 5 OS=Mus musculus GN=Rab11fip5 PE=1 SV=2 - [RFIP5_MOUSE]	31.01	1	13	13	29	1.00	0.89	0.95	0.94	0.94	1.10	1.18	1.14	1.19	1.07	1.14	1.01	1.07	69.51	9.07
Q01705-4	Isoform 4 of Neurogenic locus notch homolog protein 1 OS=Mus musculus GN=Notch1 - [NOTC1_MOUSE]	2.11	4	3	4	7	0.86	1.28	1.33	0.98	1.15	1.09	1.27	1.14	1.29	1.30	1.53	1.28	1.34	269.00	5.07
Q05BH3	Dhx16 protein OS=Mus musculus GN=Dhx16 PE=2 SV=1 - [Q05BH3_MOUSE]	3.91	2	2	2	3	0.98	1.05	1.08	0.98	0.90	1.10	0.86	1.14	1.16	1.09	1.05	1.01	1.03	83.99	6.55
Q7TPB0	Lipid phosphate phosphatase-related protein type 3 OS=Mus musculus GN=Lppr3 PE=2 SV=1 - [LPPR3_MOUSE]	18.85	3	10	10	26	1.40	1.18	0.86	1.01	0.78	1.21	0.90	1.14	0.88	1.37	0.98	1.35	0.99	76.64	6.01
Q60872	Eukaryotic translation initiation factor 1A OS=Mus musculus GN=Eif1a PE=2 SV=3 - [IF1A_MOUSE]	14.58	8	1	2	4	1.24	1.14	0.92	1.15	0.93	0.83	0.67	1.14	0.92	1.19	0.96	1.14	0.92	16.49	5.24
P48771	Cytochrome c oxidase subunit 7A2, mitochondrial OS=Mus musculus GN=Cox7a2 PE=1 SV=2 - [CX7A2_MOUSE]	27.71	1	3	3	64	1.00	0.86	0.88	1.02	0.99	1.16	1.20	1.14	1.11	1.12	1.07	1.02	1.04	9.28	10.27
Q9JKF6	Poliovirus receptor-related protein 1 OS=Mus musculus GN=Pvrl1 PE=1 SV=3 - [PVRL1_MOUSE]	38.25	1	14	14	49	1.03	1.03	0.98	1.02	0.97	1.23	1.16	1.14	1.10	1.11	1.02	1.07	1.02	57.00	6.35
G5E884	Serine/threonine-protein kinase PAK 1 OS=Mus musculus GN=Pak1 PE=4 SV=1 - [G5E884_MOUSE]	65.81	2	15	24	201	0.93	0.83	0.85	0.88	0.94	1.06	1.10	1.14	1.18	0.92	1.00	0.90	0.94	60.57	5.86
Q9CPV2	Anaphase-promoting complex subunit 16 OS=Mus musculus GN=Anapc16 PE=2 SV=1 - [APC16_MOUSE]	17.27	1	2	2	3	1.13	0.81	0.72	0.94	0.83	1.18	1.04	1.14	1.01	1.00	0.89	0.97	0.86	11.66	4.97
P63089	Pleiotrophin OS=Mus musculus GN=Ptn PE=2 SV=1 - [PTN_MOUSE]	39.29	1	5	5	21	0.91	0.84	0.92	1.02	1.15	1.06	1.09	1.14	1.22	0.97	1.08	1.11	1.21	18.86	9.60

Q99LQ4	Coiled-coil domain-containing protein 23 OS=Mus musculus GN=Ccdc23 PE=2 SV=1 - [CCD23_MOUSE]	22.73	2	1	1	5	1.01	0.87	0.98	0.93	0.92	1.29	1.13	1.14	1.01	0.94	0.91	1.08	0.94	7.83	9.16
Q9CQR4	Acyl-coenzyme A thioesterase 13 OS=Mus musculus GN=Acot13 PE=1 SV=1 - [ACO13_MOUSE]	62.14	1	6	6	21	0.95	0.95	0.91	0.99	1.05	1.17	1.23	1.14	1.16	1.11	1.11	1.06	1.11	15.17	8.82
Q6NZN0-	Isoform 5 of RNA-binding protein 26 OS=Mus musculus GN=Rbm26 - [RBM26_MOUSE]	14.26	8	11	12	28	1.00	0.78	0.77	0.90	0.89	1.07	1.09	1.14	1.11	0.99	1.00	0.90	0.92	111.21	9.44
E9PXB7	E3 ubiquitin-protein ligase NEDD4-like OS=Mus musculus GN=Nedd4l PE=2 SV=1 - [E9PXB7_MOUSE]	17.52	5	10	12	23	0.98	1.11	1.20	0.96	0.96	1.03	1.13	1.14	1.19	1.02	1.09	1.02	0.96	112.15	5.87
Q9DCB1-	Isoform 3 of High mobility group nucleosome-binding domain-containing protein 3 OS=Mus musculus GN=Hmgn3 - [HMGN3_MOUSE]	33.68	3	2	2	11	0.92	0.81	0.66	0.92	0.93	1.18	1.15	1.14	1.07	1.08	1.07	0.96	0.98	10.24	9.95
Q9D0S9	Histidine triad nucleotide-binding protein 2, mitochondrial OS=Mus musculus GN=Hint2 PE=2 SV=1 - [HINT2_MOUSE]	42.33	1	4	4	17	1.03	1.03	0.95	1.08	0.98	1.12	1.00	1.14	1.05	1.02	0.97	0.97	0.95	17.31	9.82
P10854	Histone H2B type 1-M OS=Mus musculus GN=Hist1h2bm PE=1 SV=2 - [H2B1M_MOUSE]	57.94	5	5	9	121	1.08	0.97	0.91	1.09	1.07	1.19	1.14	1.14	1.07	1.18	1.10	1.21	1.14	13.93	10.32
Q60749	KH domain-containing, RNA-binding, signal transduction-associated protein 1 OS=Mus musculus GN=Khdrbs1 PE=1 SV=2 - [KHDR1_MOUSE]	13.77	1	3	5	17	1.09	0.81	0.78	0.84	0.87	1.08	1.08	1.14	1.10	0.98	0.96	0.83	0.91	48.34	8.72
E9PZF0	Nucleoside diphosphate kinase OS=Mus musculus GN=Gm20390 PE=2 SV=1 - [E9PZF0_MOUSE]	65.92	3	4	12	71	1.07	0.73	0.76	0.95	0.93	1.00	0.94	1.14	1.03	0.93	0.83	0.92	0.91	30.18	8.72
O35668	Huntingtin-associated protein 1 OS=Mus musculus GN=Hap1 PE=1 SV=1 - [HAP1_MOUSE]	19.27	5	11	11	28	2.68	1.22	0.40	0.99	0.35	1.28	0.43	1.14	0.39	2.44	0.85	2.41	0.87	70.07	4.74
Q99N92	39S ribosomal protein L27, mitochondrial OS=Mus musculus GN=Mrpl27 PE=2 SV=1 - [RM27_MOUSE]	12.84	1	2	2	2	1.04	0.97	0.93	1.00	0.96	0.99	0.94	1.14	1.09	1.03	0.98	0.96	0.92	15.93	10.17

A2A485	Protein Zmynd8 OS=Mus musculus GN=Zmynd8 PE=2 SV=1 - [A2A485_MOUSE]	3.02	10	3	3	5	0.97	0.88	1.04	0.94	1.01	1.04	1.02	1.14	1.05	0.98	1.01	0.97	1.01	120.76	6.65
Q8C7U1	NEDD4-binding protein 3 OS=Mus musculus GN=N4bp3 PE=2 SV=1 - [N4BP3_MOUSE]	2.79	3	1	2	2	0.97	1.15	1.18	0.89	0.91	1.23	1.26	1.14	1.17	1.07	1.10	0.80	0.82	60.01	8.16
O88856	Protein-tyrosine sulfotransferase 2 OS=Mus musculus GN=Tpst2 PE=1 SV=1 - [TPST2_MOUSE]	6.65	2	1	1	1	1.22	1.47	1.20	1.00	0.82	0.92	0.75	1.14	0.93	1.20	0.98	1.25	1.02	42.04	9.35
B2RRE7	OTU domain-containing protein 4 OS=Mus musculus GN=Otud4 PE=1 SV=1 - [OTUD4_MOUSE]	5.15	1	4	4	9	0.96	1.10	1.15	0.90	1.03	1.09	1.10	1.14	1.19	1.24	1.25	1.18	1.34	122.98	6.76
P55288	Cadherin-11 OS=Mus musculus GN=Cdh11 PE=1 SV=1 - [CAD11_MOUSE]	17.34	4	10	10	23	0.96	0.97	1.01	1.06	1.09	1.12	1.12	1.14	1.24	1.05	1.08	0.99	1.02	88.06	4.89
P49813	Tropomodulin-1 OS=Mus musculus GN=Tmod1 PE=2 SV=2 - [TMOD1_MOUSE]	49.86	1	16	16	55	0.92	0.91	0.99	0.94	1.04	1.20	1.25	1.14	1.19	1.04	1.16	1.01	1.11	40.44	5.10
Q9JHS3	Ragulator complex protein LAMTOR2 OS=Mus musculus GN=Lamtor2 PE=1 SV=1 - [LTOR2_MOUSE]	8.00	1	1	1	1	1.58	2.23	1.41	1.91	1.21	0.99	0.62	1.14	0.72	2.32	1.47	2.37	1.50	13.47	5.40
P62311	U6 snRNA-associated Sm-like protein LSm3 OS=Mus musculus GN=Lsm3 PE=3 SV=2 - [LSM3_MOUSE]	19.61	1	2	2	5	1.06	0.93	1.01	0.89	0.95	1.05	1.11	1.14	1.04	0.94	1.01	1.00	0.97	11.84	4.70
O70591	Prefoldin subunit 2 OS=Mus musculus GN=Pfdn2 PE=2 SV=2 - [PFD2_MOUSE]	50.65	2	7	7	114	1.06	0.87	0.83	0.94	0.88	1.17	1.11	1.14	1.08	1.09	1.03	1.04	0.97	16.52	6.58
E9PZ43	Microtubule-associated protein OS=Mus musculus GN=Map4 PE=2 SV=1 - [E9PZ43_MOUSE]	58.52	5	23	41	212	1.07	0.93	0.85	0.94	0.88	1.15	1.06	1.14	1.10	1.08	1.00	1.00	0.91	97.74	9.09
Q3SXD3	HD domain-containing protein 2 OS=Mus musculus GN=Hddc2 PE=2 SV=1 - [HDDC2_MOUSE]	34.17	4	4	5	14	1.09	1.19	0.92	0.99	0.93	1.29	1.15	1.14	1.05	1.31	1.14	1.21	1.06	22.74	5.02
Q9CYS6	Uncharacterized protein C2orf72 homolog OS=Mus musculus PE=2 SV=2 - [CB072_MOUSE]	4.20	2	1	1	2	1.07	0.93	0.87	1.03	0.97	1.16	1.08	1.14	1.07	1.07	1.00	1.01	0.95	30.30	6.05
A6H6A9	Rab GTPase-activating protein 1-like OS=Mus musculus GN=Rabgap1l PE=1 SV=1 - [RBG1L_MOUSE]	4.91	2	1	3	12	1.42	1.58	1.11	1.90	1.33	0.86	0.60	1.14	0.80	1.04	0.73	1.22	0.86	92.34	5.31

Q8BT51	Cytochrome c oxidase assembly factor 4 homolog, mitochondrial OS=Mus musculus GN=COA4 PE=3 SV=1 - [COA4_MOUSE]	17.24	1	2	2	14	1.04	1.17	0.99	0.97	0.87	1.14	0.96	1.14	1.08	1.08	0.97	0.90	0.88	10.13	6.90
O88951	Protein lin-7 homolog B OS=Mus musculus GN=Lin7b PE=1 SV=2 - [LIN7B_MOUSE]	30.92	1	2	7	48	0.96	0.98	1.05	0.99	1.09	1.06	1.15	1.14	1.26	1.11	1.18	0.98	1.08	22.90	8.68
D3YVV7	Protein Nova2 OS=Mus musculus GN=Nova2 PE=4 SV=2 - [D3YVV7_MOUSE]	23.98	2	6	8	17	0.90	0.90	1.05	0.96	1.08	1.17	1.27	1.14	1.10	0.96	0.98	0.99	1.11	49.04	8.16
Q9CQJ6	Density-regulated protein OS=Mus musculus GN=Denr PE=2 SV=1 - [DENR_MOUSE]	46.97	2	9	9	32	1.08	0.97	0.88	0.97	0.89	1.12	1.00	1.14	1.05	1.09	1.00	1.01	0.95	22.15	5.30
Q8BVU5	ADP-ribose pyrophosphatase, mitochondrial OS=Mus musculus GN=Nudt9 PE=2 SV=1 - [NUDT9_MOUSE]	21.43	4	7	7	19	0.91	0.95	1.00	0.94	1.04	1.10	1.22	1.14	1.19	0.97	1.04	1.01	1.13	38.58	6.76
Q3UTQ8	Cyclin-dependent kinase-like 5 OS=Mus musculus GN=Cdkl5 PE=2 SV=1 - [CDKL5_MOUSE]	9.81	3	7	8	14	0.96	0.91	0.94	1.04	1.12	1.14	1.17	1.14	1.20	1.12	1.16	1.00	1.07	105.42	9.58
F7D4S5	SLAIN motif-containing protein 1 (Fragment) OS=Mus musculus GN=Slain1 PE=2 SV=1 - [F7D4S5_MOUSE]	6.95	5	3	3	5	0.85	0.84	0.99	0.82	0.90	1.23	1.17	1.14	1.24	1.16	1.28	1.08	1.41	56.93	8.09
Q2KN98	Cytospin-A OS=Mus musculus GN=Specc1l PE=1 SV=1 - [CYTSA_MOUSE]	9.66	5	8	10	27	0.95	0.93	0.93	0.94	1.02	1.12	1.20	1.14	1.13	1.07	1.07	0.98	0.99	124.41	5.76
Q14CH0-2	Isoform 2 of Protein FAM171B OS=Mus musculus GN=Fam171b - [F171B_MOUSE]	21.44	2	13	13	28	1.08	0.98	0.89	1.04	0.92	1.15	1.04	1.14	1.00	1.07	0.98	1.02	0.92	91.48	8.37
O08919	Numb-like protein OS=Mus musculus GN=Numbl PE=1 SV=3 - [NUMBL_MOUSE]	26.82	1	10	12	43	0.99	0.90	0.91	0.95	0.91	1.11	1.13	1.14	1.17	1.04	1.04	1.08	1.08	64.09	8.82
Q8JZS0	Protein lin-7 homolog A OS=Mus musculus GN=Lin7a PE=1 SV=2 - [LIN7A_MOUSE]	58.37	3	5	12	72	0.86	0.81	0.99	0.82	0.98	1.04	1.17	1.14	1.28	0.89	1.01	0.91	0.99	25.98	8.72
Q9DCW4	Electron transfer flavoprotein subunit beta OS=Mus musculus GN=Etfb PE=1 SV=3 - [ETFB_MOUSE]	45.88	1	10	10	30	0.98	1.01	0.94	1.01	1.03	1.16	1.15	1.15	1.09	1.11	1.12	1.07	1.05	27.61	8.10
P70268	Serine/threonine-protein kinase N1 OS=Mus musculus GN=Pkn1 PE=1 SV=3 - [PKN1_MOUSE]	2.11	3	1	2	3	0.84	1.06	1.26	0.98	1.16	1.07	1.26	1.15	1.36	1.14	1.36	1.08	1.29	104.35	6.27

Q9ERB0	Synaptosomal-associated protein 29 OS=Mus musculus GN=Snap29 PE=1 SV=1 - [SNP29_MOUSE]	58.08	1	13	13	43	1.02	0.81	0.78	0.89	0.88	1.17	1.14	1.15	1.12	1.05	1.04	0.98	0.97	29.55	5.38
Q8BGK9	Uncharacterized protein C3orf18 homolog OS=Mus musculus PE=2 SV=1 - [CC018_MOUSE]	8.54	1	1	1	1	0.73	1.18	1.62	1.03	1.41	1.04	1.42	1.15	1.56	1.25	1.72	0.84	1.15	17.71	5.29
Q3V0A7	Protein 4930402H24Rik OS=Mus musculus GN=4930402H24Rik PE=2 SV=1 - [Q3V0A7_MOUSE]	0.76	2	1	1	1	0.94	0.88	0.93	0.97	1.03	1.09	1.15	1.15	1.21	1.11	1.18	1.02	1.09	117.45	6.47
G3X9V7	MCG19133, isoform CRA_b OS=Mus musculus GN=A830010M20Rik PE=4 SV=1 - [G3X9V7_MOUSE]	45.50	6	35	35	257	0.95	0.98	1.00	0.96	1.01	1.12	1.15	1.15	1.19	1.01	1.06	0.95	0.97	117.24	7.80
E9PYI7	Protein Lmo7 OS=Mus musculus GN=Lmo7 PE=2 SV=1 - [E9PYI7_MOUSE]	28.77	7	32	32	102	0.93	0.97	0.98	0.91	0.98	1.15	1.15	1.15	1.16	1.04	1.06	0.94	1.07	164.64	5.73
Q8R0L9-2	Isoform 2 of Transcriptional adapter 3 OS=Mus musculus GN=Tada3 - [TADA3_MOUSE]	19.13	3	6	6	11	0.95	0.98	0.85	0.90	0.90	1.11	1.03	1.15	1.08	1.09	1.08	0.99	0.92	46.59	6.57
F8VQ70	Protein Scaper OS=Mus musculus GN=Scaper PE=2 SV=1 - [F8VQ70_MOUSE]	1.29	1	1	1	2	0.93	0.91	0.98	1.07	1.15	1.11	1.18	1.15	1.23	1.00	1.08	1.10	1.18	157.68	7.31
Q9JKK1	Syntaxin-6 OS=Mus musculus GN=Stx6 PE=1 SV=1 - [STX6_MOUSE]	46.27	2	9	9	26	0.99	1.07	1.07	0.99	0.99	1.12	1.17	1.15	1.11	1.05	1.03	0.93	1.00	28.98	4.92
B8QI36	Liprin-alpha 4 OS=Mus musculus GN=Ppfia4 PE=2 SV=1 - [B8QI36_MOUSE]	12.47	1	7	11	22	1.05	1.01	1.01	1.06	0.97	1.06	1.01	1.15	1.05	1.21	1.08	1.16	1.13	133.63	6.27
Q921S7	39S ribosomal protein L37, mitochondrial OS=Mus musculus GN=Mrpl37 PE=2 SV=1 - [RM37_MOUSE]	3.55	1	1	1	2	0.91	0.86	0.94	0.96	1.06	1.01	1.10	1.15	1.25	0.96	1.06	1.08	1.19	48.31	8.84
Q8BJH1	Zinc finger C2HC domain-containing protein 1A OS=Mus musculus GN=Zc2hc1a PE=2 SV=1 - [ZC21A_MOUSE]	63.89	2	18	18	91	1.05	0.82	0.76	0.95	0.90	1.15	1.09	1.15	1.10	1.07	1.02	0.98	0.95	35.13	9.88
Q925B0	PRKC apoptosis WT1 regulator protein OS=Mus musculus GN=Pawr PE=1 SV=2 - [PAWR_MOUSE]	19.52	2	5	5	9	0.96	0.89	0.95	0.98	1.04	1.03	1.07	1.15	1.35	1.08	1.15	1.07	1.00	35.89	5.92
Q8BG26	RUN and SH3 domain-containing protein 1 OS=Mus musculus GN=Rusc1 PE=1 SV=2 - [RUSC1_MOUSE]	2.46	2	2	2	5	0.98	0.94	0.95	0.99	0.94	1.14	1.12	1.15	1.08	1.13	1.10	1.05	1.07	95.14	6.02

Q9ERT9	Protein phosphatase 1 regulatory subunit 1A OS=Mus musculus GN=Ppp1r1a PE=2 SV=1 - [PPR1A_MOUSE]	83.04	1	11	11	73	1.03	0.93	0.85	0.96	0.93	1.10	1.07	1.15	1.07	1.09	1.09	1.00	0.98	18.71	5.25
Q9JJU8	SH3 domain-binding glutamic acid-rich-like protein OS=Mus musculus GN=Sh3bgrl PE=3 SV=1 - [SH3L1_MOUSE]	93.86	1	12	12	84	1.05	0.88	0.86	0.91	0.85	1.15	1.10	1.15	1.08	1.07	1.06	1.05	1.03	12.80	4.92
Q8BXQ8	Protein FAM53C OS=Mus musculus GN=Fam53c PE=1 SV=1 - [FA53C_MOUSE]	4.33	1	1	1	1	1.26	0.70	0.56	1.01	0.81	1.13	0.89	1.15	0.91	1.18	0.94	1.08	0.86	43.23	7.72
Q810J8	Zinc finger FYVE domain-containing protein 1 OS=Mus musculus GN=Zfyve1 PE=2 SV=2 - [ZFYV1_MOUSE]	7.34	1	4	4	6	1.07	1.25	1.16	1.02	0.95	1.05	0.98	1.15	1.08	1.16	1.10	1.25	1.19	86.88	7.34
Q99P31	Hsp70-binding protein 1 OS=Mus musculus GN=Hspbp1 PE=2 SV=1 - [HPBP1_MOUSE]	23.25	1	4	4	12	0.82	1.11	1.25	1.19	1.20	0.98	1.12	1.15	1.27	1.14	1.29	1.00	1.18	39.14	5.36
Q99MP8-	Isoform 2 of BRCA1-associated protein OS=Mus musculus GN=Brap - [BRAP_MOUSE]	21.21	4	8	8	15	0.93	0.80	0.86	0.81	0.85	0.87	0.93	1.15	1.16	0.75	0.76	0.80	0.84	63.91	5.71
Q32M26	Uncharacterized protein C11orf87 homolog OS=Mus musculus PE=2 SV=1 - [CK087_MOUSE]	5.03	1	1	1	2	0.90	0.86	0.96	1.03	1.15	1.24	1.37	1.15	1.27	1.11	1.23	0.95	1.06	20.70	9.29
P62073	Mitochondrial import inner membrane translocase subunit Tim10 OS=Mus musculus GN=Timm10 PE=1 SV=1 - [TIM10_MOUSE]	53.33	1	5	5	218	1.05	0.93	0.88	0.97	0.91	1.11	1.07	1.15	1.09	1.08	1.02	1.05	0.99	10.33	6.29
Q9D6T0	Nitric oxide synthase-interacting protein OS=Mus musculus GN=Nosip PE=2 SV=1 - [NOSIP_MOUSE]	14.62	3	3	3	6	0.97	0.98	1.01	0.87	0.93	1.11	1.16	1.15	1.18	1.09	1.05	1.11	1.07	33.19	8.62
P47740	Fatty aldehyde dehydrogenase OS=Mus musculus GN=Aldh3a2 PE=2 SV=2 - [AL3A2_MOUSE]	2.48	3	1	1	3	1.21	1.64	1.35	1.35	1.11	0.93	0.76	1.15	0.94	0.86	0.71	1.04	1.06	53.94	8.35
E9Q2S0	Protein Pcdh7 OS=Mus musculus GN=Pcdh7 PE=2 SV=1 - [E9Q2S0_MOUSE]	27.57	5	23	23	63	0.97	0.99	1.02	0.97	1.02	1.09	1.11	1.15	1.13	1.01	1.04	1.03	1.05	136.69	5.17

Q9D820-2	Isoform 2 of Prolyl-tRNA synthetase associated domain-containing protein 1 OS=Mus musculus GN=Prorsd1 - [PRXD1_MOUSE]	30.37	2	3	3	4	0.98	1.18	1.45	0.90	0.90	1.20	1.47	1.15	1.15	1.08	0.99	1.20	1.25	15.05	7.18
Q9WVA2	Mitochondrial import inner membrane translocase subunit Tim8 A OS=Mus musculus GN=Timm8a1 PE=1 SV=1 - [TIM8A_MOUSE]	46.39	1	4	4	29	0.95	0.91	0.95	0.95	0.99	1.02	1.14	1.15	1.21	0.96	1.09	0.91	1.00	11.04	5.16
Q91W18-2	Isoform 2 of Tudor domain-containing protein 3 OS=Mus musculus GN=Tdrd3 - [TDRD3_MOUSE]	10.34	3	6	6	10	0.96	1.06	1.11	0.90	0.97	1.14	1.15	1.15	1.26	0.98	1.13	1.05	1.10	78.97	9.13
F8WHQ1	Tumor protein D52 OS=Mus musculus GN=Tpd52 PE=2 SV=1 - [F8WHQ1_MOUSE]	71.66	9	14	14	56	0.85	0.80	0.94	0.89	1.06	1.04	1.21	1.15	1.33	1.03	1.17	0.95	1.11	26.91	4.96
P58774	Tropomyosin beta chain OS=Mus musculus GN=Tpm2 PE=1 SV=1 - [TPM2_MOUSE]	52.46	6	3	21	190	1.16	1.13	1.14	0.91	0.74	8.61	8.65	1.15	0.86	1.37	1.03	1.10	0.89	32.82	4.70
B2KF73	Small EDRK-rich factor 1 OS=Mus musculus GN=Serf1 PE=4 SV=1 - [B2KF73_MOUSE]	16.67	3	1	1	1	0.99	0.90	0.92	0.81	0.82	1.10	1.11	1.15	1.16	1.04	1.05	0.86	0.87	5.66	10.08
Q69ZS8	Kazrin OS=Mus musculus GN=Kazn PE=1 SV=2 - [KAZRN_MOUSE]	28.11	3	3	14	65	1.15	1.13	1.01	0.97	0.84	1.16	1.05	1.15	1.00	1.01	0.93	1.12	1.02	86.66	6.90
Q9JJ43-2	Isoform 2 of RNA binding protein fox-1 homolog 1 OS=Mus musculus GN=Rbfox1 - [RFOX1_MOUSE]	21.54	4	1	7	19	0.91	1.04	1.14	0.92	1.01	1.08	1.19	1.15	1.26	0.96	1.06	1.07	1.17	40.47	7.33
O55022	Membrane-associated progesterone receptor component 1 OS=Mus musculus GN=Pgrmc1 PE=1 SV=4 - [PGRC1_MOUSE]	64.62	1	8	10	176	1.10	1.04	0.90	1.01	0.94	1.21	1.11	1.15	1.05	1.24	1.14	1.15	1.08	21.68	4.70
Q8BZJ8	Uncharacterized protein C8orf34 homolog OS=Mus musculus PE=2 SV=1 - [CH034_MOUSE]	7.63	7	3	3	6	1.06	0.87	0.81	0.97	0.88	1.16	1.08	1.15	1.04	1.12	1.02	1.13	1.04	51.05	4.97
P20357	Microtubule-associated protein 2 OS=Mus musculus GN=Map2 PE=1 SV=2 - [MTAP2_MOUSE]	80.42	8	132	155	3819	1.07	0.95	0.91	0.95	0.90	1.14	1.07	1.15	1.07	1.08	1.01	1.01	0.97	199.01	4.91

Q8BLI4	Dermatan-sulfate epimerase OS=Mus musculus GN=Dse PE=2 SV=1 - [DSE_MOUSE]	0.84	1	1	1	1	0.93	1.15	1.23	0.95	1.02	1.04	1.11	1.15	1.22	1.08	1.16	0.92	0.99	109.68	7.25
Q6A065	Centrosomal protein of 170 kDa OS=Mus musculus GN=Cep170 PE=1 SV=2 - [CE170_MOUSE]	36.46	4	45	47	139	1.08	0.94	0.91	0.96	0.91	1.13	1.04	1.15	1.05	1.05	0.99	0.98	0.94	174.94	7.17
Q8BRM2	RAB6-interacting golgin OS=Mus musculus GN=Gorab PE=1 SV=1 - [GORAB_MOUSE]	23.10	2	6	6	10	1.17	1.14	1.00	0.95	0.80	1.22	1.02	1.15	0.94	1.11	0.95	1.01	0.93	41.46	7.36
Q61164	Transcriptional repressor CTCF OS=Mus musculus GN=Ctcf PE=1 SV=2 - [CTCF_MOUSE]	4.35	1	2	2	4	1.01	0.71	0.70	1.15	1.14	1.20	1.19	1.15	0.99	0.96	0.95	0.95	0.95	83.69	6.90
Q68FM6	Protein phosphatase 1 regulatory subunit 29 OS=Mus musculus GN=Elfn2 PE=1 SV=1 - [PPR29_MOUSE]	22.24	1	12	14	45	1.06	0.98	0.95	0.99	0.91	1.12	1.04	1.15	1.02	1.12	0.99	1.08	1.00	89.97	7.52
F8VPL5	Mitogen-activated protein kinase kinase kinase kinase 4 OS=Mus musculus GN=Map4k4 PE=2 SV=1 - [F8VPL5_MOUSE]	4.70	3	1	5	8	1.12	1.09	0.97	1.06	0.95	0.96	0.85	1.15	1.02	1.02	0.91	1.07	0.96	140.72	7.47
D3YZ21	Protein D430041D05Rik OS=Mus musculus GN=D430041D05Rik PE=2 SV=1 - [D3YZ21_MOUSE]	14.78	3	1	13	45	1.05	0.76	0.73	0.98	0.94	1.05	0.99	1.15	1.09	1.07	1.02	1.02	0.98	153.28	9.39
Q6NZB0	DnaJ homolog subfamily C member 8 OS=Mus musculus GN=Dnajc8 PE=2 SV=2 - [DNJC8_MOUSE]	39.13	7	10	10	39	0.97	0.76	0.84	0.96	0.93	1.08	1.09	1.15	1.12	1.03	1.05	0.91	0.89	29.79	9.06
B1AUY8	N-alpha-acetyltransferase 10 OS=Mus musculus GN=Naa10 PE=2 SV=1 - [B1AUY8_MOUSE]	12.27	8	2	2	4	1.11	1.20	1.08	1.21	1.09	1.18	1.05	1.15	1.03	1.24	1.11	1.16	1.04	24.83	5.52
Q5SXY1	Cytospin-B OS=Mus musculus GN=Specc1 PE=1 SV=2 - [CYTSB_MOUSE]	34.49	3	27	29	120	0.98	0.92	0.92	0.91	0.92	1.12	1.14	1.15	1.15	1.04	1.03	1.03	1.04	118.03	6.64
Q99KX1	Myeloid leukemia factor 2 OS=Mus musculus GN=Mlf2 PE=1 SV=1 - [MLF2_MOUSE]	40.89	1	6	6	34	0.97	1.07	1.07	0.99	1.03	1.10	1.12	1.15	1.18	1.11	1.12	1.08	1.15	28.04	6.98
Q9D1X0	Nucleolar protein 3 OS=Mus musculus GN=Nol3 PE=1 SV=1 - [NOL3_MOUSE]	53.18	1	7	7	68	1.00	1.01	0.96	0.97	0.94	1.19	1.15	1.15	1.09	1.10	1.10	1.09	1.07	24.55	4.07

Q9DC29	ATP-binding cassette sub-family B member 6, mitochondrial OS=Mus musculus GN=Abcb6 PE=1 SV=1 - [ABCB6_MOUSE]	2.38	1	1	1	2	0.97	1.37	1.41	1.57	1.62	1.03	1.05	1.15	1.18	1.03	1.06	1.07	1.10	93.71	8.05
P70452	Syntaxin-4 OS=Mus musculus GN=Stx4 PE=1 SV=1 - [STX4_MOUSE]	25.84	2	6	6	16	1.08	1.15	1.10	1.00	0.94	1.08	1.05	1.15	1.09	1.16	1.15	1.08	1.10	34.14	6.14
Q9CQG9	Transmembrane protein 100 OS=Mus musculus GN=Tmem100 PE=1 SV=1 - [TM100_MOUSE]	6.72	1	1	1	1	1.08	0.88	0.81	0.88	0.81	1.25	1.15	1.15	1.06	1.07	0.99	1.05	0.97	14.49	9.50
Q9D4J1	EF-hand domain-containing protein D1 OS=Mus musculus GN=Efhd1 PE=2 SV=1 - [EFHD1_MOUSE]	37.92	1	4	7	24	1.26	1.07	0.87	1.02	0.79	1.08	0.82	1.15	0.92	0.93	0.80	0.96	0.87	26.98	6.02
Q3UHD1	Brain-specific angiogenesis inhibitor 1 OS=Mus musculus GN=Bai1 PE=1 SV=1 - [BAI1_MOUSE]	10.18	2	13	13	53	0.98	1.03	1.03	0.97	0.97	1.16	1.14	1.15	1.17	0.99	1.02	0.90	0.94	173.19	7.56
Q9CY16	28S ribosomal protein S28, mitochondrial OS=Mus musculus GN=Mrps28 PE=2 SV=1 - [RT28_MOUSE]	4.84	1	1	1	2	0.77	0.87	1.14	0.75	0.98	0.93	1.21	1.15	1.50	0.83	1.08	0.92	1.21	20.51	8.92
P47867-2	Isoform 2 of Secretogranin-3 OS=Mus musculus GN=Scg3 - [SCG3_MOUSE]	65.67	2	21	22	58	1.18	0.91	0.78	0.95	0.81	1.20	1.02	1.15	0.99	1.07	0.94	1.02	0.87	52.75	4.91
Q9Z0H3-2	Isoform B of SWI/SNF-related matrix-associated actin-dependent regulator of chromatin subfamily B member 1 OS=Mus musculus GN=Smarcb1 - [SNF5_MOUSE]	10.90	3	2	2	4	1.25	1.24	0.98	1.21	0.96	1.21	0.96	1.15	0.91	1.36	1.09	1.00	0.80	43.13	5.64
P61148	Fibroblast growth factor 1 OS=Mus musculus GN=Fgf1 PE=2 SV=1 - [FGF1_MOUSE]	36.13	5	5	5	10	1.07	0.80	0.80	0.92	0.84	1.24	1.08	1.15	1.10	1.12	1.08	1.00	0.99	17.41	7.02
Q3TGY1	Serine/Arginine-related protein 53 OS=Mus musculus GN=Rsrc1 PE=2 SV=1 - [Q3TGY1_MOUSE]	19.18	4	5	5	10	1.00	0.85	0.80	1.11	1.05	1.03	0.99	1.15	1.05	1.06	1.06	1.04	1.04	33.52	10.35
Q5EBJ4	Ermin OS=Mus musculus GN=Ermn PE=1 SV=1 - [ERMIN_MOUSE]	53.38	1	13	13	217	0.95	0.94	0.97	0.86	0.89	1.17	1.27	1.15	1.22	1.06	1.15	1.01	1.05	32.13	4.59
O35601-2	Isoform FYB-120 of FYN-binding protein OS=Mus musculus GN=Fyb - [FYB_MOUSE]	6.60	2	3	3	6	1.01	0.91	0.97	1.04	1.01	1.06	1.06	1.15	1.11	1.15	1.12	1.08	1.04	84.82	6.71

Q3UVU8	Disks large-associated protein 1 OS=Mus musculus GN=Dlgap1 PE=2 SV=1 - [Q3UVU8_MOUSE]	17.92	11	9	10	25	0.96	0.96	1.05	1.09	1.11	0.96	1.08	1.15	1.13	0.92	1.03	0.88	0.98	70.64	5.58
Q99JP4	Anaphase-promoting complex subunit CDC26 OS=Mus musculus GN=Cdc26 PE=2 SV=1 - [CDC26_MOUSE]	60.00	1	4	4	12	1.12	1.14	0.97	0.97	0.85	1.23	1.13	1.15	1.08	1.11	0.98	1.10	0.98	9.75	6.81
Q9DA69-	Isoform 3 of Intraflagellar transport protein 43 homolog OS=Mus musculus GN=Ift43 - [IFT43_MOUSE]	13.47	3	2	2	6	1.16	0.90	0.93	0.79	0.83	1.25	1.07	1.15	1.10	1.13	1.17	1.21	1.04	21.88	4.70
A2BI12	PC4 and SFRS1-interacting protein OS=Mus musculus GN=Psip1 PE=2 SV=1 - [A2BI12_MOUSE]	65.75	3	2	22	149	1.10	0.89	0.81	0.84	0.84	1.14	1.14	1.15	1.20	1.00	1.04	0.87	0.79	36.95	9.23
Q3TYE5	Limbic system-associated membrane protein OS=Mus musculus GN=Lsamp PE=2 SV=1 - [Q3TYE5_MOUSE]	44.38	3	15	15	401	1.06	0.94	0.89	1.01	0.94	1.18	1.10	1.15	1.08	1.13	1.07	1.11	1.05	37.29	6.68
Q62179	Semaphorin-4B OS=Mus musculus GN=Sema4b PE=1 SV=2 - [SEM4B_MOUSE]	1.58	1	1	1	2	1.17	1.29	1.10	1.21	1.03	1.26	1.07	1.15	0.98	1.25	1.07	1.19	1.02	91.33	8.15
Q3TI53-1	Isoform 1 of Schwannomin-interacting protein 1 OS=Mus musculus GN=Schip1 - [SCHI1_MOUSE]	10.96	5	3	3	5	1.06	1.04	0.89	0.97	0.94	1.37	1.06	1.15	0.95	1.23	1.03	1.45	1.11	49.95	5.15
F8VPK8	Protein Pcdh9 OS=Mus musculus GN=Pcdh9 PE=2 SV=1 - [F8VPK8_MOUSE]	23.12	1	18	18	50	0.99	1.09	1.13	1.02	1.05	1.12	1.12	1.15	1.17	1.04	1.05	0.99	0.99	136.06	5.50
Q9CQI4	Uncharacterized protein C11orf74 homolog OS=Mus musculus GN=Nwc PE=2 SV=1 - [CK074_MOUSE]	19.67	4	3	3	7	1.08	1.01	0.93	0.97	0.87	1.10	1.02	1.15	1.00	1.07	0.98	1.09	1.03	27.64	4.68
P63248	cAMP-dependent protein kinase inhibitor alpha OS=Mus musculus GN=Pkia PE=1 SV=2 - [IPKA_MOUSE]	71.05	1	5	5	35	1.05	0.94	1.07	0.94	0.85	1.09	1.02	1.15	1.06	1.02	0.94	1.04	0.96	7.96	4.54
Q8K2F0	Bromodomain-containing protein 3 OS=Mus musculus GN=Brd3 PE=1 SV=2 - [BRD3_MOUSE]	14.19	4	4	8	25	1.07	0.86	0.85	0.96	1.05	1.02	1.00	1.15	1.12	1.09	1.05	1.03	1.06	79.71	9.36
Q8BQP8	Rab11 family-interacting protein 4 OS=Mus musculus GN=Rab11fip4 PE=1 SV=1 - [RFIP4_MOUSE]	5.35	1	2	3	5	1.10	1.00	0.90	0.98	0.89	1.13	1.02	1.15	1.04	1.05	0.96	1.15	1.04	71.86	4.84

P43024	Cytochrome c oxidase subunit 6A1, mitochondrial OS=Mus musculus GN=Cox6a1 PE=1 SV=2 - [CX6A1_MOUSE]	61.26	2	4	4	102	1.06	0.97	0.86	1.11	1.03	1.17	1.11	1.15	1.09	1.15	1.07	1.10	1.03	12.34	9.98
Q8K3K8	Optineurin OS=Mus musculus GN=Optn PE=1 SV=1 - [OPTN_MOUSE]	23.63	1	12	12	26	1.09	0.96	0.77	0.91	0.82	1.14	1.01	1.15	0.98	1.08	1.05	1.05	0.95	66.98	5.26
Q7TSJ2	Microtubule-associated protein 6 OS=Mus musculus GN=Map6 PE=1 SV=2 - [MAP6_MOUSE]	87.20	4	91	91	926	1.03	0.88	0.87	0.92	0.91	1.15	1.11	1.15	1.11	1.05	1.03	1.00	0.99	96.39	9.50
D3Z3U7	Activated CDC42 kinase 1 OS=Mus musculus GN=Tnk2 PE=4 SV=1 - [D3Z3U7_MOUSE]	5.09	7	2	2	4	0.93	1.05	1.12	0.96	1.03	1.11	1.18	1.15	1.23	1.00	1.09	1.18	1.27	59.11	7.44
Q9D6S7	Ribosome-recycling factor, mitochondrial OS=Mus musculus GN=Mrrf PE=1 SV=1 - [RRFM_MOUSE]	41.98	1	7	7	45	1.22	0.91	0.76	1.04	0.87	1.18	0.97	1.15	0.98	1.14	0.94	1.15	0.98	29.03	9.85
P62075	Mitochondrial import inner membrane translocase subunit Tim13 OS=Mus musculus GN=Timm13 PE=1 SV=1 - [TIM13_MOUSE]	52.63	1	4	4	46	0.98	0.96	0.93	0.94	0.94	1.08	1.04	1.15	1.19	0.98	1.01	0.92	0.99	10.45	8.18
Q6P1D5	Seizure 6-like protein OS=Mus musculus GN=Sez6l PE=2 SV=1 - [SE6L1_MOUSE]	16.51	2	10	10	19	1.10	1.10	1.04	1.07	0.96	1.15	1.04	1.15	1.07	1.10	0.96	1.00	0.98	104.76	4.69
D3Z3Z3	Voltage-dependent L-type calcium channel subunit beta-3 OS=Mus musculus GN=Cacnb3 PE=2 SV=1 - [D3Z3Z3_MOUSE]	22.15	2	6	8	22	0.94	1.26	1.27	0.98	1.04	1.05	1.19	1.15	1.22	0.83	0.89	0.94	1.02	54.36	6.54
Q80VB6	Dclk1 protein OS=Mus musculus GN=Dclk1 PE=2 SV=1 - [Q80VB6_MOUSE]	49.31	2	1	16	50	0.98	0.89	0.90	1.02	1.04	1.05	1.06	1.15	1.17	1.12	1.14	0.95	0.97	40.36	9.67
A6H6A9-3	Isoform 3 of Rab GTPase-activating protein 1-like OS=Mus musculus GN=Rabgap1l - [RBG1L_MOUSE]	31.62	2	9	12	41	1.10	1.02	1.00	0.95	0.86	1.14	1.10	1.15	1.03	1.04	1.02	1.12	0.97	42.73	7.33
Q8CFQ9	Fusion, derived from t(1216) malignant liposarcoma (Human) OS=Mus musculus GN=Fus PE=2 SV=1 - [Q8CFQ9_MOUSE]	24.76	5	9	12	198	1.02	0.91	0.90	0.92	0.90	1.18	1.15	1.15	1.13	1.09	1.07	0.96	0.95	52.57	9.36

Q8BSZ2	AP-3 complex subunit sigma-2 OS=Mus musculus GN=Ap3s2 PE=1 SV=1 - [AP3S2_MOUSE]	15.03	1	3	3	4	1.01	1.00	0.98	1.17	1.16	1.01	1.05	1.15	1.13	1.06	1.06	0.91	1.06	22.00	5.22
Q00915	Retinol-binding protein 1 OS=Mus musculus GN=Rbp1 PE=2 SV=2 - [RET1_MOUSE]	60.74	1	7	7	39	1.09	0.87	0.77	0.95	0.83	1.11	0.99	1.15	1.02	1.14	0.98	1.18	1.09	15.84	5.25
Q8K354	Carbonyl reductase [NADPH] 3 OS=Mus musculus GN=Cbr3 PE=2 SV=1 - [CBR3_MOUSE]	43.32	1	7	9	29	0.89	1.12	1.24	1.21	1.29	1.13	1.22	1.15	1.24	1.18	1.31	1.34	1.52	30.93	6.57
H3BL68	PAB-dependent poly(A)-specific ribonuclease subunit 3 (Fragment) OS=Mus musculus GN=Pan3 PE=4 SV=1 - [H3BL68_MOUSE]	10.93	4	1	1	2	1.03	1.16	1.13	0.97	0.94	1.16	1.12	1.15	1.12	1.15	1.11	1.09	1.06	19.04	6.77
P15327	Bisphosphoglycerate mutase OS=Mus musculus GN=Bpgm PE=2 SV=2 - [PMGE_MOUSE]	15.44	4	3	3	8	0.98	1.47	1.53	0.96	1.03	1.13	1.12	1.15	1.17	1.18	1.22	1.16	1.17	29.96	7.06
P09405	Nucleolin OS=Mus musculus GN=Ncl PE=1 SV=2 - [NUCL_MOUSE]	44.55	1	38	38	347	1.03	0.82	0.80	0.96	0.92	1.18	1.14	1.15	1.12	1.07	1.02	0.96	0.94	76.68	4.75
Q6NS82-2	Isoform 2 of Protein FAM134A OS=Mus musculus GN=Fam134a - [F134A_MOUSE]	13.67	3	2	2	10	1.50	1.15	0.71	1.14	0.72	1.31	0.75	1.15	0.81	1.02	0.72	1.19	0.86	29.58	4.23
O35226	26S proteasome non-ATPase regulatory subunit 4 OS=Mus musculus GN=Psmd4 PE=1 SV=1 - [PSMD4_MOUSE]	30.59	2	1	10	36	0.96	0.52	0.54	0.72	0.75	1.15	1.19	1.15	1.20	1.28	1.34	1.20	1.25	40.68	4.79
F6X2H0	E3 ubiquitin-protein ligase TRIM41 (Fragment) OS=Mus musculus GN=Trim41 PE=4 SV=1 - [F6X2H0_MOUSE]	10.19	2	1	1	2	1.03	1.13	1.09	0.99	0.97	0.99	0.95	1.15	1.12	1.05	1.02	1.20	1.17	17.98	4.93
P27773	Protein disulfide-isomerase A3 OS=Mus musculus GN=Pdia3 PE=1 SV=2 - [PDIA3_MOUSE]	62.38	2	33	33	276	0.96	0.82	0.87	0.88	0.93	1.08	1.13	1.15	1.20	1.02	1.08	0.96	1.03	56.64	6.21
E9Q6R4	Protein Arid1b OS=Mus musculus GN=Arid1b PE=2 SV=1 - [E9Q6R4_MOUSE]	3.21	3	5	5	8	1.16	1.01	0.82	1.07	1.06	1.24	1.05	1.15	1.00	1.21	1.01	1.22	1.10	236.87	6.80
Q921C5	Protein bicaudal D homolog 2 OS=Mus musculus GN=Bicd2 PE=1 SV=1 - [BICD2_MOUSE]	13.66	4	8	8	14	0.94	0.96	1.21	0.82	0.97	1.13	1.30	1.16	1.36	1.04	1.14	1.01	1.02	93.33	5.44

Q6A0A2-2	Isoform 2 of La-related protein 4B OS=Mus musculus GN=Larp4b - [LAR4B_MOUSE]	9.44	3	5	5	11	1.01	1.12	1.07	0.94	0.92	1.07	1.04	1.16	1.16	1.05	1.03	1.04	1.05	72.49	6.23
Q9DCJ7	Aurora kinase A-interacting protein OS=Mus musculus GN=Aurkaip1 PE=2 SV=2 - [AKIP_MOUSE]	5.00	1	1	1	1	0.86	0.77	0.90	0.92	1.07	1.04	1.21	1.16	1.34	1.09	1.27	1.00	1.17	23.29	10.73
F6ZDS4	Nucleoprotein TPR OS=Mus musculus GN=Tpr PE=1 SV=1 - [TPR_MOUSE]	40.85	7	86	87	257	1.03	0.89	0.87	0.93	0.92	1.16	1.13	1.16	1.15	1.03	1.03	0.97	0.97	273.82	5.03
Q6P9K8	Caskin-1 OS=Mus musculus GN=Caskin1 PE=1 SV=2 - [CSKI1_MOUSE]	34.94	4	33	33	132	0.98	0.97	0.99	0.95	0.98	1.10	1.13	1.16	1.20	1.10	1.10	1.03	1.04	150.40	9.17
Q9DCB4	cAMP-regulated phosphoprotein 21 OS=Mus musculus GN=Arpp21 PE=1 SV=2 - [ARP21_MOUSE]	10.53	16	3	7	48	1.07	1.02	0.94	1.09	1.02	1.20	1.02	1.16	1.09	1.06	0.99	1.16	1.09	88.51	7.39
Q8BLH7	HIRA-interacting protein 3 OS=Mus musculus GN=Hirip3 PE=1 SV=1 - [HIRP3_MOUSE]	7.99	1	3	3	7	1.05	1.12	1.11	0.96	0.97	1.21	1.20	1.16	1.05	1.08	1.09	0.98	1.00	65.18	7.84
Q9EQM6	Microprocessor complex subunit DGCR8 OS=Mus musculus GN=Dgcr8 PE=2 SV=2 - [DGCR8_MOUSE]	2.59	1	1	1	2	0.87	0.86	0.99	0.94	1.08	0.99	1.13	1.16	1.33	1.11	1.28	0.91	1.05	86.27	6.06
J3QP80	MCG15559 OS=Mus musculus GN=Gm5814 PE=4 SV=1 - [J3QP80_MOUSE]	42.40	2	4	4	9	1.09	0.99	0.92	0.95	0.90	1.14	1.04	1.16	1.09	1.04	0.95	1.00	0.85	14.15	4.86
Q8BPU7	Engulfment and cell motility protein 1 OS=Mus musculus GN=Elmo1 PE=1 SV=2 - [ELMO1_MOUSE]	6.33	4	3	5	8	0.93	0.92	0.94	0.92	0.99	1.09	1.20	1.16	1.25	1.11	1.20	1.03	1.11	83.88	6.28
Q9D8B3	Charged multivesicular body protein 4b OS=Mus musculus GN=Chmp4b PE=2 SV=2 - [CHM4B_MOUSE]	54.02	1	13	13	104	1.04	0.83	0.81	0.93	0.92	1.18	1.12	1.16	1.11	1.08	1.06	0.98	0.91	24.92	4.82
F7BJK1	Protein Pcdh1 (Fragment) OS=Mus musculus GN=Pcdh1 PE=2 SV=1 - [F7BJK1_MOUSE]	43.83	1	24	30	209	1.03	1.08	1.04	0.97	0.96	1.14	1.10	1.16	1.12	1.14	1.11	1.15	1.09	116.45	5.08
Q8BKE9	Intraflagellar transport protein 74 homolog OS=Mus musculus GN=Ift74 PE=1 SV=2 - [IFT74_MOUSE]	19.67	2	9	9	22	1.03	0.79	0.84	0.94	0.87	1.16	1.05	1.16	1.06	1.01	0.92	0.98	0.93	69.26	6.00
Q8BL48-2	Isoform 2 of RING finger protein unkempt homolog OS=Mus musculus GN=Unk - [UNK_MOUSE]	11.92	2	6	8	19	1.02	1.17	1.16	0.97	0.87	0.98	0.91	1.16	1.08	1.05	1.07	0.97	0.97	86.76	6.92

P97450	ATP synthase-coupling factor 6, mitochondrial OS=Mus musculus GN=Atp5j PE=1 SV=1 - [ATP5J_MOUSE]	42.59	2	6	6	174	1.02	1.00	0.98	0.94	0.93	1.09	1.08	1.16	1.12	1.08	1.02	1.04	1.00	12.49	9.36
I6L974	TBC1 domain family member 17 OS=Mus musculus GN=Tbc1d17 PE=2 SV=1 - [I6L974_MOUSE]	2.01	2	1	1	1	1.13	3.56	3.15	1.16	1.02	1.00	0.88	1.16	1.02	1.22	1.08	1.42	1.26	62.06	5.91
E9Q942	Small integral membrane protein 13 OS=Mus musculus GN=Smim13 PE=3 SV=1 - [SIM13_MOUSE]	44.32	1	2	2	11	0.96	0.99	1.03	0.92	0.96	1.12	1.34	1.16	1.13	1.09	1.20	0.81	1.07	9.95	6.27
Q9DCT5	Stromal cell-derived factor 2 OS=Mus musculus GN=Sdf2 PE=2 SV=1 - [SDF2_MOUSE]	25.12	1	3	3	19	0.96	0.85	0.83	0.89	0.93	1.06	1.07	1.16	1.20	1.11	1.09	1.06	1.04	23.14	7.33
Q99PI5	Phosphatidate phosphatase LPIN2 OS=Mus musculus GN=Lpin2 PE=1 SV=2 - [LPIN2_MOUSE]	5.04	4	3	4	6	1.06	1.04	1.04	0.93	0.88	1.09	0.97	1.16	1.10	1.10	1.05	1.03	1.06	99.55	5.47
P56394	Cytochrome c oxidase copper chaperone OS=Mus musculus GN=Cox17 PE=2 SV=2 - [COX17_MOUSE]	20.63	1	1	1	10	1.05	0.49	0.45	0.97	0.84	1.25	1.07	1.16	1.05	1.05	0.90	0.87	0.79	6.78	7.69
A2AQ44	Formin-binding protein 1 OS=Mus musculus GN=Fnbp1 PE=2 SV=1 - [A2AQ44_MOUSE]	25.04	13	12	12	18	0.94	0.98	1.13	0.84	0.91	1.09	1.13	1.16	1.26	1.06	1.17	1.02	1.11	65.14	5.27
Q8CC27-4	Isoform 4 of Voltage-dependent L-type calcium channel subunit beta-2 OS=Mus musculus GN=Cacnb2 - [CACB2_MOUSE]	10.82	4	2	4	16	0.99	1.08	1.08	1.09	1.06	0.99	1.06	1.16	1.15	1.23	1.30	1.61	1.62	64.67	9.07
Q9Z2E1	Methyl-CpG-binding domain protein 2 OS=Mus musculus GN=Mbd2 PE=2 SV=2 - [MBD2_MOUSE]	16.91	2	6	7	10	1.08	0.83	0.91	0.93	0.89	1.15	1.12	1.16	1.04	1.16	1.00	0.86	0.91	43.47	10.04
Q9CR00	26S proteasome non-ATPase regulatory subunit 9 OS=Mus musculus GN=Psmd9 PE=1 SV=1 - [PSMD9_MOUSE]	45.95	1	10	10	68	1.09	1.05	0.94	0.95	0.90	1.11	1.07	1.16	1.05	1.08	1.03	1.06	1.00	24.70	6.43
Q9JMG1	Endothelial differentiation-related factor 1 OS=Mus musculus GN=Edf1 PE=1 SV=1 - [EDF1_MOUSE]	42.57	1	7	7	37	1.07	0.85	0.86	0.94	0.93	1.12	1.06	1.16	1.06	1.10	1.02	0.99	0.95	16.36	9.99

Q8BKX1-	Isoform 3 of Brain-specific angiogenesis inhibitor 1-associated protein 2 OS=Mus musculus GN=Baiap2 - [BAIP2_MOUSE]	63.55	4	2	26	134	1.31	0.99	0.80	1.03	0.81	1.33	1.03	1.16	1.22	1.01	1.14	1.05	0.98	56.83	8.97
E9PYL2	Protein Prr12 OS=Mus musculus GN=Prr12 PE=2 SV=1 - [E9PYL2_MOUSE]	3.98	1	4	4	9	0.95	0.95	0.99	0.97	1.02	1.26	1.35	1.16	1.25	1.11	1.12	0.96	1.00	211.75	7.65
P63166	Small ubiquitin-related modifier 1 OS=Mus musculus GN=Sumo1 PE=1 SV=1 - [SUMO1_MOUSE]	20.79	1	2	2	6	1.02	1.02	0.89	0.82	0.96	1.08	1.26	1.16	1.20	0.96	0.94	0.90	1.03	11.55	5.52
Q8R1K1	Ubiquitin-associated domain-containing protein 2 OS=Mus musculus GN=Ubac2 PE=2 SV=1 - [UBAC2_MOUSE]	4.64	1	1	1	1	1.36	0.91	0.67	1.48	1.09	0.98	0.72	1.16	0.85	0.83	0.61	0.93	0.69	39.03	9.44
Q3UCQ1	Forkhead box protein K2 OS=Mus musculus GN=Foxk2 PE=2 SV=3 - [FOXK2_MOUSE]	6.14	2	2	2	4	0.98	1.03	1.05	0.99	1.01	1.04	1.05	1.16	1.18	1.08	1.10	1.09	1.11	68.40	9.51
Q9CZH7	Matrix-remodeling-associated protein 7 OS=Mus musculus GN=Mxra7 PE=1 SV=2 - [MXRA7_MOUSE]	19.66	2	2	2	6	0.90	0.71	1.13	1.05	0.79	1.08	1.20	1.16	1.28	1.08	1.20	1.10	0.96	19.45	4.26
B1AU75	Nuclear autoantigenic sperm protein OS=Mus musculus GN=Nasp PE=4 SV=1 - [B1AU75_MOUSE]	19.40	9	11	12	16	0.95	0.98	1.15	0.89	1.05	1.09	1.08	1.16	1.22	1.03	1.10	0.91	0.93	83.96	4.37
F8VQ40	Laminin subunit alpha-1 OS=Mus musculus GN=Lama1 PE=4 SV=1 - [F8VQ40_MOUSE]	0.68	2	1	1	2	0.81	1.23	1.51	1.01	1.24	0.90	1.10	1.16	1.42	0.90	1.11	0.97	1.20	337.93	6.71
Q0VGU4	MCG18019 OS=Mus musculus GN=Vgf PE=2 SV=1 - [Q0VGU4_MOUSE]	68.40	1	33	33	184	1.17	1.03	0.88	0.99	0.81	1.20	1.01	1.16	1.01	1.19	1.02	1.14	1.00	68.19	4.70
Q8R0S4	Voltage-dependent L-type calcium channel subunit beta-4 OS=Mus musculus GN=Cacnb4 PE=1 SV=2 - [CACB4_MOUSE]	27.36	4	7	12	28	0.97	1.04	1.09	1.03	1.00	1.05	1.04	1.16	1.21	1.09	1.11	1.17	1.18	57.91	9.28
Q9Z0U1	Tight junction protein ZO-2 OS=Mus musculus GN=Tjp2 PE=1 SV=2 - [ZO2_MOUSE]	25.96	1	25	25	41	0.96	0.92	0.99	0.86	0.88	1.08	1.10	1.16	1.16	1.02	1.07	1.01	0.99	131.20	6.79

Q8C6B2-2	Isoform 2 of Rhotekin OS=Mus musculus GN=Rtkn - [RTKN_MOUSE]	7.44	4	2	3	5	0.90	0.99	1.10	1.07	1.20	0.90	1.00	1.16	1.29	1.05	1.17	1.12	1.25	61.52	6.00
O70400	PDZ and LIM domain protein 1 OS=Mus musculus GN=Pdlim1 PE=2 SV=4 - [PDLI1_MOUSE]	42.81	1	12	12	25	0.86	0.96	1.12	0.85	0.93	1.19	1.34	1.16	1.26	1.01	1.14	0.91	1.03	35.75	6.84
E9Q505	Amyloid beta A4 precursor protein-binding family A member 2 OS=Mus musculus GN=Apba2 PE=2 SV=1 - [E9Q505_MOUSE]	9.76	5	5	6	17	1.09	0.98	0.99	1.12	1.07	1.17	1.10	1.16	1.13	1.14	1.15	1.04	0.97	81.32	4.78
Q99KG3-2	Isoform 2 of RNA-binding protein 10 OS=Mus musculus GN=Rbm10 - [RBM10_MOUSE]	15.47	3	10	11	19	0.97	0.97	0.87	0.97	0.90	1.06	1.04	1.16	1.12	1.06	0.95	0.97	0.97	94.37	6.70
O35551	Rab GTPase-binding effector protein 1 OS=Mus musculus GN=Rabep1 PE=1 SV=2 - [RABE1_MOUSE]	52.78	8	47	48	182	0.98	0.84	0.89	0.88	0.89	1.12	1.15	1.16	1.16	0.99	1.03	0.92	0.96	99.46	5.01
D3YU00	StAR-related lipid transfer (START) domain containing 5, isoform CRA_a OS=Mus musculus GN=Stard5 PE=4 SV=1 - [D3YU00_MOUSE]	4.37	2	1	1	2	1.28	0.88	0.68	1.03	0.80	1.02	0.79	1.16	0.90	1.03	0.81	1.30	1.02	23.15	5.45
Q80YT7-2	Isoform 2 of Myomegalin OS=Mus musculus GN=Pde4dip - [MYOME_MOUSE]	5.19	5	4	4	6	0.89	1.09	1.22	0.90	1.01	1.14	1.27	1.16	1.29	1.27	1.42	1.14	1.29	126.54	5.34
Q99MQ5	Collagen alpha-1(XXV) chain OS=Mus musculus GN=Col25a1 PE=2 SV=2 - [COPA1_MOUSE]	7.81	2	2	2	5	0.84	1.00	1.02	0.98	1.02	1.25	0.97	1.16	1.26	1.19	1.10	1.18	1.29	65.34	8.51
Q99N69	Leupaxin OS=Mus musculus GN=Lpxn PE=1 SV=2 - [LPXN_MOUSE]	5.96	1	2	2	3	1.06	0.98	0.92	1.00	0.94	1.18	1.10	1.16	1.09	1.10	1.04	1.09	1.03	43.45	6.43
Q8R5L1	Complement component 1 Q subcomponent-binding protein, mitochondrial OS=Mus musculus GN=C1qbp PE=2 SV=1 - [Q8R5L1_MOUSE]	61.65	2	10	10	548	1.01	0.85	0.85	0.93	0.92	1.16	1.15	1.16	1.13	1.05	1.04	0.99	0.97	31.01	4.87
Q9Z315	U4/U6.U5 tri-snRNP-associated protein 1 OS=Mus musculus GN=Sart1 PE=2 SV=1 - [SNUT1_MOUSE]	45.04	1	26	26	68	1.09	1.06	0.89	1.04	0.95	1.17	1.10	1.16	1.06	1.12	1.04	1.09	0.98	90.83	5.82
B1AV75	Protein 2610301B20Rik OS=Mus musculus GN=2610301B20Rik PE=2 SV=1 - [B1AV75_MOUSE]	50.48	1	1	9	44	1.10	0.83	0.76	1.09	0.99	1.26	1.14	1.16	1.05	1.16	1.06	1.23	1.12	23.92	6.86

Q3TML0	Protein disulfide-isomerase A6 OS=Mus musculus GN=Pdia6 PE=2 SV=1 - [Q3TML0_MOUSE]	33.48	2	11	11	26	1.02	0.89	0.87	1.02	1.00	1.03	1.17	1.16	1.17	1.08	1.13	1.10	1.24	48.66	5.19
Q78WH7	Calcium/calmodulin-dependent protein kinase II inhibitor 2 OS=Mus musculus GN=Camk2n2 PE=2 SV=1 - [CK2N2_MOUSE]	54.43	1	2	4	25	1.06	0.91	0.83	0.95	0.89	1.13	1.04	1.16	1.06	1.02	0.94	1.05	0.95	8.62	5.48
Q6P9J5	KN motif and ankyrin repeat domain-containing protein 4 OS=Mus musculus GN=Kank4 PE=2 SV=1 - [KANK4_MOUSE]	11.02	3	6	6	11	0.93	0.79	0.85	0.95	0.98	1.05	1.09	1.16	1.21	1.05	1.15	1.03	1.13	110.34	4.88
Q9DBN4	Putative monooxygenase p33MONOX OS=Mus musculus GN=P33monox PE=1 SV=1 - [P33MX_MOUSE]	47.52	7	8	8	21	1.03	0.79	0.82	0.94	0.85	1.14	1.12	1.16	1.11	1.13	1.03	1.06	0.96	32.69	9.42
Q8CIP4	MAP/microtubule affinity-regulating kinase 4 OS=Mus musculus GN=Mark4 PE=1 SV=1 - [MARK4_MOUSE]	5.98	1	1	3	6	0.98	1.48	1.51	0.99	1.02	1.00	1.02	1.16	1.18	1.15	1.18	1.16	1.19	82.59	9.67
Q8C120	SH3 domain-containing RING finger protein 3 OS=Mus musculus GN=Sh3rf3 PE=2 SV=2 - [SH3R3_MOUSE]	2.73	3	1	3	3	0.95	0.62	0.65	1.00	1.05	1.14	1.19	1.16	1.22	0.97	1.02	0.96	1.01	93.07	8.53
E9QP85	Protein Dos OS=Mus musculus GN=Dos PE=4 SV=1 - [E9QP85_MOUSE]	29.23	4	15	15	33	1.17	1.04	0.95	1.01	0.83	1.14	0.98	1.16	1.04	1.15	1.02	1.13	1.03	74.09	6.33
Q8C525	Protein MB21D2 OS=Mus musculus GN=Mb21d2 PE=1 SV=1 - [M21D2_MOUSE]	3.74	2	1	1	2	0.89	1.04	1.17	1.11	1.25	0.76	0.85	1.16	1.30	1.05	1.18	1.06	1.19	48.45	7.68
P30275	Creatine kinase U-type, mitochondrial OS=Mus musculus GN=Ckmt1 PE=1 SV=1 - [KCRU_MOUSE]	60.29	5	18	20	217	1.01	0.98	0.98	1.03	1.03	1.15	1.12	1.16	1.15	1.08	1.08	1.11	1.10	46.97	8.16
A2ADB1	Msx2-interacting protein OS=Mus musculus GN=Spen PE=2 SV=1 - [A2ADB1_MOUSE]	1.49	5	4	4	7	1.05	1.26	1.20	0.94	0.90	0.94	0.86	1.16	1.11	0.92	0.77	1.05	1.01	395.74	8.56
J3QQ18	Ras GTPase-activating protein SynGAP OS=Mus musculus GN=Syngap1 PE=4 SV=1 - [J3QQ18_MOUSE]	36.01	4	1	35	159	0.98	1.12	1.13	1.02	1.02	1.16	1.19	1.16	1.18	1.02	0.99	1.01	1.06	144.78	9.01

Q3UDR8	Protein YIPF3 OS=Mus musculus GN=Yipf3 PE=1 SV=1 - [YIPF3_MOUSE]	6.92	1	1	1	2	1.07	1.04	0.97	1.01	0.94	1.05	0.98	1.16	1.08	1.17	1.10	1.15	1.08	37.97	5.94
D3Z2W0	TNFAIP3-interacting protein 1 OS=Mus musculus GN=Tnip1 PE=2 SV=1 - [D3Z2W0_MOUSE]	18.27	7	8	8	16	1.06	0.94	0.94	0.91	0.84	1.16	1.07	1.16	1.06	1.09	1.01	0.98	0.90	72.89	6.30
Q99LW6	YY1-associated factor 2 OS=Mus musculus GN=Yaf2 PE=1 SV=1 - [YAF2_MOUSE]	13.41	1	1	1	9	0.82	0.84	1.50	1.15	1.41	1.05	1.67	1.16	1.51	1.14	1.61	0.87	1.07	19.64	9.58
Q9D0L1	Zinc finger BED domain-containing protein 3 OS=Mus musculus GN=Zbed3 PE=1 SV=1 - [ZBED3_MOUSE]	4.39	1	1	1	3	1.11	1.00	1.04	0.88	0.66	1.24	0.87	1.16	1.13	1.10	0.98	1.22	0.88	25.59	6.54
Q6PCP5	Mitochondrial fission factor OS=Mus musculus GN=Mff PE=1 SV=1 - [MFF_MOUSE]	58.42	7	8	11	27	0.99	0.99	0.99	1.01	1.00	1.22	1.18	1.16	1.18	1.24	1.21	1.17	1.17	32.91	6.83
Q14BB9	MAP6 domain-containing protein 1 OS=Mus musculus GN=Map6d1 PE=1 SV=1 - [MA6D1_MOUSE]	59.69	1	6	6	11	0.81	0.95	1.29	0.92	1.13	0.97	1.19	1.16	1.54	1.00	1.47	0.96	1.24	20.42	9.88
F8WHT3	Protein PRRC2B OS=Mus musculus GN=Prrc2b PE=2 SV=1 - [F8WHT3_MOUSE]	24.98	4	13	35	93	1.02	1.08	1.15	1.00	1.03	1.21	1.13	1.16	1.16	1.18	1.13	1.13	1.07	242.97	8.47
Q8BP92	Reticulocalbin-2 OS=Mus musculus GN=Rcn2 PE=2 SV=1 - [RCN2_MOUSE]	68.44	2	17	17	337	1.13	0.91	0.80	0.96	0.86	1.16	1.03	1.16	1.02	1.10	0.98	1.01	0.92	37.25	4.42
Q9ESX4-2	Isoform 2 of Nucleolar protein of 40 kDa OS=Mus musculus GN=Zcchc17 - [NO40_MOUSE]	15.03	2	2	2	7	1.02	1.08	0.85	1.17	1.00	0.95	0.91	1.16	0.97	0.95	0.97	0.94	0.92	22.10	9.69
E9Q3M9	Protein 2010300C02Rik OS=Mus musculus GN=2010300C02Rik PE=2 SV=1 - [E9Q3M9_MOUSE]	60.68	2	17	47	252	1.09	1.11	1.02	1.05	0.92	1.23	1.10	1.16	1.07	1.20	1.09	1.28	1.15	125.83	7.37
Q505F5	Leucine-rich repeat-containing protein 47 OS=Mus musculus GN=Lrrc47 PE=1 SV=1 - [LRC47_MOUSE]	30.46	4	14	14	29	0.96	1.01	1.06	0.95	1.04	1.15	1.23	1.16	1.21	1.16	1.26	1.12	1.22	63.55	8.10
G3UZ60	Small ubiquitin-related modifier 3 OS=Mus musculus GN=Sumo3 PE=2 SV=1 - [G3UZ60_MOUSE]	28.00	7	1	2	19	1.11	0.74	0.66	0.94	0.85	1.23	1.11	1.16	1.05	1.02	0.93	1.00	0.90	8.60	9.20
B1ARW8	Uncharacterized protein C1orf122 homolog OS=Mus musculus PE=2 SV=1 - [CA122_MOUSE]	34.55	2	3	3	7	1.21	0.91	0.84	0.92	0.82	1.34	1.10	1.16	0.95	1.09	0.89	1.12	0.95	11.31	8.21

Q3UL36	Arginine and glutamate rich protein 1 OS=Mus musculus GN=Arglu1 PE=1 SV=2 - [ARGL1_MOUSE]	14.76	3	5	6	19	0.95	0.88	0.95	0.98	1.05	1.09	1.11	1.16	1.25	1.12	1.17	0.96	0.98	32.87	10.36
Q9D0R8	Protein LSM12 homolog OS=Mus musculus GN=Lsm12 PE=1 SV=1 - [LSM12_MOUSE]	26.15	1	3	3	9	0.85	0.70	0.82	0.79	0.93	1.01	1.18	1.16	1.25	0.92	1.05	0.98	1.13	21.69	7.74
Q8BWS5	G protein-regulated inducer of neurite outgrowth 3 OS=Mus musculus GN=Gprin3 PE=1 SV=1 - [GRIN3_MOUSE]	42.46	1	22	22	80	1.05	0.97	0.94	1.00	0.96	1.24	1.21	1.16	1.12	1.30	1.20	1.30	1.24	80.44	7.02
P29699	Alpha-2-HS-glycoprotein OS=Mus musculus GN=Ahsg PE=1 SV=1 - [FETUA_MOUSE]	7.25	1	2	2	2	0.96	0.78	0.81	0.58	0.60	1.45	1.50	1.16	1.20	1.14	1.18	0.57	0.60	37.30	6.51
J3QPM9	Guanine nucleotide-binding protein subunit gamma OS=Mus musculus GN=Gm3150 PE=3 SV=1 - [J3QPM9_MOUSE]	13.24	2	1	1	7	1.06	0.95	0.89	1.04	0.97	1.24	1.24	1.16	1.12	1.22	1.19	1.09	1.08	7.29	9.50
Q3V1V3	ESF1 homolog OS=Mus musculus GN=Esf1 PE=1 SV=1 - [ESF1_MOUSE]	3.91	1	3	3	6	1.05	0.93	0.85	1.03	0.87	1.22	1.00	1.16	1.11	1.03	0.98	1.21	1.06	97.99	5.05
P06728	Apolipoprotein A-IV OS=Mus musculus GN=Apoa4 PE=2 SV=3 - [APOA4_MOUSE]	17.97	1	7	7	13	1.01	1.28	1.25	0.92	0.93	1.19	1.19	1.16	1.23	2.81	2.92	1.39	1.42	45.00	5.47
Q9DCC5	Cbx3 protein OS=Mus musculus GN=Cbx3 PE=2 SV=1 - [Q9DCC5_MOUSE]	64.48	4	9	11	95	1.02	0.78	0.70	0.90	0.86	1.19	1.14	1.16	1.07	0.96	0.95	0.97	0.90	20.80	5.33
Q9DAF3	Protein DDI1 homolog 1 OS=Mus musculus GN=Ddi1 PE=1 SV=1 - [DDI1_MOUSE]	3.43	1	1	1	5	0.84	0.76	0.85	0.71	0.84	1.01	1.25	1.16	1.36	0.87	1.11	0.91	1.09	45.68	6.18
Q91VJ5	Polyglutamine-binding protein 1 OS=Mus musculus GN=Pqbp1 PE=2 SV=1 - [PQBP1_MOUSE]	40.30	3	8	8	41	1.08	1.15	0.97	0.95	0.90	1.18	1.02	1.16	1.09	1.12	0.98	1.04	0.94	30.58	6.23
Q8CJ19	Protein-methionine sulfoxide oxidase MICAL3 OS=Mus musculus GN=Mical3 PE=1 SV=2 - [MICA3_MOUSE]	24.84	7	37	38	112	1.01	1.04	1.04	1.02	1.04	1.15	1.17	1.16	1.17	1.10	1.09	1.07	1.07	223.58	5.47
Q91VW3	SH3 domain-binding glutamic acid-rich-like protein 3 OS=Mus musculus GN=Sh3bgrl3 PE=1 SV=1 - [SH3L3_MOUSE]	87.10	1	9	10	194	0.99	0.98	0.92	0.92	0.90	1.09	1.05	1.16	1.12	1.08	1.03	1.01	1.04	10.47	5.14

Q62347	Cyclic AMP-responsive element-binding protein 1 OS=Mus musculus GN=Creb1 PE=2 SV=1 - [Q62347_MOUSE]	20.56	22	4	4	19	1.04	0.97	0.93	0.98	0.94	1.26	1.21	1.16	1.13	1.07	0.98	1.00	0.94	30.94	6.37
P63271	Transcription elongation factor SPT4-A OS=Mus musculus GN=Supt4h1a PE=2 SV=1 - [SPT4A_MOUSE]	21.37	4	2	2	2	0.85	0.70	0.82	0.97	1.14	1.02	1.20	1.16	1.37	1.06	1.25	0.99	1.17	13.18	8.06
Q7TNF8-	Isoform 2 of Peripheral-type benzodiazepine receptor-associated protein 1 OS=Mus musculus GN=Bzrap1 - [RIMB1_MOUSE]	0.70	4	1	1	1	1.03	0.66	0.64	0.92	0.90	1.21	1.17	1.16	1.13	1.00	0.98	0.83	0.81	169.80	5.34
F8VQ95	Transforming acidic coiled-coil-containing protein 1 OS=Mus musculus GN=Tacc1 PE=2 SV=1 - [F8VQ95_MOUSE]	50.39	3	23	26	68	1.05	0.99	0.98	0.95	0.89	1.12	1.08	1.16	1.14	1.05	1.02	1.02	1.03	84.20	5.03
Q61792	LIM and SH3 domain protein 1 OS=Mus musculus GN=Lasp1 PE=1 SV=1 - [LASP1_MOUSE]	69.96	11	16	18	247	1.04	0.95	0.92	0.93	0.90	1.16	1.11	1.16	1.14	1.07	1.05	1.03	0.98	29.98	7.05
E0CXD4	Protein Pcdh1 OS=Mus musculus GN=Pcdh1 PE=2 SV=1 - [E0CXD4_MOUSE]	50.83	1	4	10	65	1.07	0.94	0.92	0.89	0.81	1.12	1.09	1.16	1.12	1.10	1.08	1.10	1.05	39.07	4.59
Q99LB4	Capping protein (Actin filament), gelsolin-like OS=Mus musculus GN=Capg PE=1 SV=1 - [Q99LB4_MOUSE]	4.01	2	1	1	2	1.01	1.06	1.04	1.15	1.14	1.49	1.47	1.16	1.14	1.21	1.19	1.13	1.11	38.74	6.95
O35691	Pinin OS=Mus musculus GN=Pnn PE=1 SV=4 - [PININ_MOUSE]	18.62	2	12	12	44	1.03	0.98	0.96	0.95	0.94	1.11	1.09	1.16	1.19	1.06	1.09	1.02	1.03	82.39	7.01
A2AGL3-	Isoform 2 of Ryanodine receptor 3 OS=Mus musculus GN=Ryr3 - [RYR3_MOUSE]	1.03	3	1	3	3	1.03	0.85	0.82	0.95	0.92	1.12	1.08	1.16	1.12	1.09	1.06	1.07	1.04	547.40	5.74
Q3URU2	Paternally-expressed gene 3 protein OS=Mus musculus GN=Peg3 PE=1 SV=1 - [PEG3_MOUSE]	4.33	2	5	5	11	1.17	1.05	0.99	1.09	0.95	1.19	0.98	1.16	1.16	1.18	1.13	1.10	1.08	178.82	5.45
A2ASS6	Titin OS=Mus musculus GN=Ttn PE=1 SV=1 - [TITIN_MOUSE]	0.14	7	1	5	29	1.35	1.12	0.83	0.73	0.54	9.36	6.91	1.16	0.86	1.28	0.95	1.20	0.89	3904.05	6.20
Q9EP71	Ankycorbin OS=Mus musculus GN=Rai14 PE=1 SV=1 - [RAI14_MOUSE]	16.45	1	13	14	25	0.98	0.90	0.86	0.89	0.89	1.13	1.13	1.16	1.16	1.05	1.08	0.96	0.93	108.79	6.27

Q9QXT0	Protein canopy homolog 2 OS=Mus musculus GN=Cnpy2 PE=2 SV=1 - [CNPY2_MOUSE]	51.65	1	8	8	80	1.08	1.05	1.03	0.94	0.89	1.15	1.08	1.16	1.10	1.13	1.08	1.03	1.05	20.75	5.07
Q8BQ46	Protein Taf15 OS=Mus musculus GN=Taf15 PE=2 SV=1 - [Q8BQ46_MOUSE]	21.90	2	7	10	47	1.14	0.86	0.75	0.96	0.89	1.22	1.12	1.16	1.02	1.01	0.95	0.96	0.96	58.57	8.40
Q71FD7	Filamin-binding LIM protein 1 OS=Mus musculus GN=Fblim1 PE=1 SV=2 - [FBLI1_MOUSE]	11.47	9	3	3	6	0.90	1.26	1.31	1.00	1.18	1.02	1.05	1.16	1.29	1.12	1.51	1.10	1.21	41.00	6.51
Q9DCH6	AN1-type zinc finger protein 6 OS=Mus musculus GN=Zfand6 PE=1 SV=1 - [ZFAN6_MOUSE]	9.87	1	1	2	5	1.05	1.36	1.20	0.94	0.86	1.29	1.22	1.16	1.17	1.05	1.18	0.99	0.94	23.99	6.51
A2AVJ7	Ribosome-binding protein 1 OS=Mus musculus GN=Rrbp1 PE=2 SV=1 - [A2AVJ7_MOUSE]	32.17	13	41	41	114	1.04	0.87	0.84	0.96	0.92	1.19	1.14	1.16	1.10	1.14	1.11	0.97	0.94	158.30	9.19
Q4V9W2	Protein SREK1IP1 OS=Mus musculus GN=Srek1ip1 PE=2 SV=1 - [SR1IP_MOUSE]	4.58	1	1	1	1	0.96	1.03	1.07	0.84	0.87	1.02	1.06	1.16	1.21	1.07	1.12	1.11	1.16	18.14	9.91
P60904	DnaJ homolog subfamily C member 5 OS=Mus musculus GN=Dnajc5 PE=1 SV=1 - [DNJC5_MOUSE]	58.08	3	6	6	55	1.19	0.98	0.83	1.03	0.92	1.24	1.05	1.16	0.99	1.11	0.93	1.12	1.03	22.09	5.07
Q5SNZ0-2	Isoform 2 of Girdin OS=Mus musculus GN=Ccdc88a - [GRDN_MOUSE]	14.04	4	21	21	37	0.97	1.00	1.11	0.97	1.04	1.15	1.17	1.16	1.20	1.00	1.05	0.98	1.05	212.37	5.99
Q62376	U1 small nuclear ribonucleoprotein 70 kDa OS=Mus musculus GN=Snrnp70 PE=1 SV=2 - [RU17_MOUSE]	16.74	2	6	6	27	1.11	0.92	0.85	1.05	0.96	1.19	1.04	1.16	1.02	1.09	1.03	0.94	0.88	51.96	9.94
Q8BVY0	Protein Rsl1d1 OS=Mus musculus GN=Rsl1d1 PE=2 SV=1 - [Q8BVY0_MOUSE]	3.98	1	1	1	3	1.11	1.11	1.00	1.10	0.99	1.10	0.99	1.16	1.05	1.23	1.11	1.09	0.99	50.39	9.98
Q8K070	Sterile alpha motif domain-containing protein 14 OS=Mus musculus GN=Samd14 PE=1 SV=1 - [SAM14_MOUSE]	26.62	2	7	7	16	1.31	0.87	0.83	0.91	0.77	1.22	0.95	1.16	0.93	1.27	1.04	1.25	1.03	45.07	9.47
Q8BQ47	Protein canopy homolog 4 OS=Mus musculus GN=Cnpy4 PE=1 SV=1 - [CNPY4_MOUSE]	32.24	2	6	6	24	1.06	0.92	0.87	0.95	0.86	1.13	1.04	1.16	1.03	1.08	0.91	0.97	0.90	28.08	4.77

Q04692	SWI/SNF-related matrix-associated actin-dependent regulator of chromatin subfamily A containing DEAD/H box 1 OS=Mus musculus GN=Smarcad1 PE=1 SV=2 - [SMRCD_MOUSE]	0.98	1	1	1	2	1.20	1.06	0.89	1.07	0.89	1.17	0.97	1.17	0.97	1.16	0.97	1.28	1.07	116.38	5.68
Q9D1H6	NADH dehydrogenase [ubiquinone] 1 alpha subcomplex assembly factor 4 OS=Mus musculus GN=Ndufaf4 PE=2 SV=1 - [NDUF4_MOUSE]	27.75	2	5	5	10	1.00	0.75	0.78	0.90	0.94	1.16	1.16	1.17	1.13	0.98	1.02	1.03	1.02	20.07	9.39
Q9CWZ3-	Isoform 2 of RNA-binding protein 8A OS=Mus musculus GN=Rbm8a - [RBM8A_MOUSE]	45.09	2	7	7	94	1.04	0.99	0.95	1.07	1.05	1.14	1.10	1.17	1.05	1.15	1.07	1.08	1.03	19.75	5.92
Q8CAF4-	Isoform 3 of NHS-like protein 1 OS=Mus musculus GN=Nhsl1 - [NHSL1_MOUSE]	6.06	3	5	5	11	0.98	1.07	1.13	0.91	0.97	1.11	1.14	1.17	1.19	1.20	1.16	0.97	1.05	169.35	8.07
Q8CFN5-	Isoform 3 of Myocyte-specific enhancer factor 2C OS=Mus musculus GN=Mef2c - [MEF2C_MOUSE]	12.90	6	3	4	8	0.89	1.14	1.22	0.94	1.01	1.22	1.35	1.17	1.32	1.09	1.17	0.81	0.97	47.93	7.84
F8WHG2	DNA-binding protein SATB1 OS=Mus musculus GN=Satb1 PE=2 SV=1 - [F8WHG2_MOUSE]	6.32	5	2	3	6	0.72	0.89	1.24	0.98	1.19	0.97	1.41	1.17	1.74	0.94	1.00	0.86	1.04	85.31	6.54
Q7TNE3	Sperm-associated antigen 7 OS=Mus musculus GN=Spag7 PE=1 SV=1 - [SPAG7_MOUSE]	25.99	2	6	6	14	1.04	0.99	0.97	0.95	0.93	1.24	1.12	1.17	1.06	1.20	1.07	1.00	0.89	25.91	7.43
P18760	Cofilin-1 OS=Mus musculus GN=Cfl1 PE=1 SV=3 - [COF1_MOUSE]	80.72	1	1	22	1007	1.00	0.78	0.78	0.89	0.90	1.15	1.14	1.17	1.16	1.01	1.00	1.01	1.03	18.55	8.09
Q4V9Z5-3	Isoform 3 of Seizure 6-like protein 2 OS=Mus musculus GN=Sez6l2 - [SE6L2_MOUSE]	21.32	2	1	13	47	0.91	1.14	1.25	0.98	1.07	1.17	1.28	1.17	1.28	1.08	1.19	0.92	1.01	93.31	4.84
P97452	Ribosome biogenesis protein BOP1 OS=Mus musculus GN=Bop1 PE=1 SV=1 - [BOP1_MOUSE]	3.83	1	2	2	4	1.07	1.07	1.00	1.03	0.97	0.92	0.86	1.17	1.09	1.02	0.95	1.07	1.01	82.49	6.28
P19973	Lymphocyte-specific protein 1 OS=Mus musculus GN=Lsp1 PE=1 SV=2 - [LSP1_MOUSE]	54.24	4	10	10	25	0.96	1.05	1.03	0.90	0.90	1.20	1.18	1.17	1.20	1.21	1.25	1.14	1.10	36.69	4.82

Q9D1L9	Ragulator complex protein LAMTOR5 OS=Mus musculus GN=Lamtor5 PE=2 SV=1 - [LTOR5_MOUSE]	81.32	2	4	4	21	1.15	0.95	0.82	1.03	0.90	1.17	1.06	1.17	1.10	1.11	1.01	1.07	0.93	9.64	4.87
Q8BGD9	Eukaryotic translation initiation factor 4B OS=Mus musculus GN=Eif4b PE=1 SV=1 - [IF4B_MOUSE]	46.64	1	29	29	229	1.05	0.91	0.91	0.97	0.91	1.20	1.12	1.17	1.11	1.09	1.03	1.01	0.98	68.80	5.67
Q3U319	E3 ubiquitin-protein ligase BRE1B OS=Mus musculus GN=Rnf40 PE=2 SV=2 - [BRE1B_MOUSE]	13.99	1	9	9	16	0.91	0.96	1.06	0.82	0.90	1.11	1.19	1.17	1.21	0.92	0.97	0.95	1.05	113.90	6.48
E9PWW9	Protein Rsf1 OS=Mus musculus GN=Rsf1 PE=2 SV=1 - [E9PWW9_MOUSE]	13.60	2	13	13	29	1.12	1.02	0.85	1.01	0.91	1.13	0.99	1.17	1.04	1.04	0.98	1.01	0.92	160.53	5.02
Q8BL06	Inactive ubiquitin carboxyl-terminal hydrolase 54 OS=Mus musculus GN=Usp54 PE=1 SV=2 - [UBP54_MOUSE]	1.13	1	1	2	3	1.02	1.32	1.29	1.14	1.12	1.19	1.17	1.17	1.14	1.15	1.13	1.20	1.19	176.55	7.62
Q9CZB6	Mediator of RNA polymerase II transcription subunit 7 OS=Mus musculus GN=Med7 PE=2 SV=2 - [MED7_MOUSE]	15.02	6	2	2	4	0.90	0.89	0.99	1.01	1.13	1.05	1.17	1.17	1.30	0.83	0.93	0.98	1.10	27.19	5.66
Q8BKX1-4	Isoform 4 of Brain-specific angiogenesis inhibitor 1-associated protein 2 OS=Mus musculus GN=Baiap2 - [BAIP2_MOUSE]	66.18	1	1	25	131	0.79	0.68	0.86	0.95	1.20	1.02	1.28	1.17	1.46	1.03	1.30	0.89	1.12	53.24	8.98
Q99LP6	GrpE protein homolog 1, mitochondrial OS=Mus musculus GN=Grpel1 PE=1 SV=1 - [GRPE1_MOUSE]	53.46	1	10	10	59	0.94	0.79	0.80	0.82	0.88	1.10	1.17	1.17	1.18	0.97	0.98	0.92	0.99	24.29	8.38
Q9JLB9	Poliovirus receptor-related protein 3 OS=Mus musculus GN=Pvrl3 PE=1 SV=1 - [PVRL3_MOUSE]	14.03	4	5	5	13	1.16	1.07	0.92	1.02	0.90	1.17	1.03	1.17	1.08	1.07	0.95	1.02	0.95	60.54	6.54
Q8BR92	Paralemmin-2 OS=Mus musculus GN=Palm2 PE=1 SV=1 - [PALM2_MOUSE]	60.37	2	12	19	90	0.99	0.88	0.87	0.94	0.95	1.17	1.13	1.17	1.14	1.09	1.10	0.96	0.98	42.07	5.15
Q7TN31	Angiogenic factor with G patch and FHA domains 1 OS=Mus musculus GN=Aggf1 PE=2 SV=1 - [AGGF1_MOUSE]	1.41	1	1	1	4	1.12	1.14	1.01	1.03	0.92	1.18	1.05	1.17	1.04	1.05	0.94	1.32	1.19	79.40	5.22

Q9Z1S8	GRB2-associated-binding protein 2 OS=Mus musculus GN=Gab2 PE=1 SV=2 - [GAB2_MOUSE]	9.32	2	4	4	8	0.99	0.89	0.91	0.90	0.88	1.14	1.16	1.17	1.16	1.07	1.12	1.20	1.09	73.16	8.31
Q8CH02	SURP and G-patch domain-containing protein 1 OS=Mus musculus GN=Sugp1 PE=1 SV=1 - [SUGP1_MOUSE]	36.86	1	18	18	45	1.00	0.92	0.93	0.96	0.94	1.24	1.21	1.17	1.13	1.08	1.02	0.99	0.92	72.60	7.64
Q8C9S4	Uncharacterized protein C10orf118 homolog OS=Mus musculus GN=Otg1 PE=2 SV=2 - [CJ118_MOUSE]	10.14	3	6	7	13	0.98	0.94	1.15	0.89	0.99	1.07	1.17	1.17	1.26	1.00	1.03	1.06	1.08	104.84	5.53
Q80YR5	Scaffold attachment factor B2 OS=Mus musculus GN=Safb2 PE=1 SV=2 - [SAFB2_MOUSE]	23.01	7	10	18	56	1.02	0.94	0.81	0.99	0.96	1.14	1.14	1.17	1.10	1.14	1.10	1.14	1.04	111.77	6.38
Q794H2	Nucleosome assembly protein 1-like 3 OS=Mus musculus GN=Nap1l3 PE=2 SV=1 - [NP1L3_MOUSE]	1.47	1	1	1	2	0.94	0.78	0.83	0.89	0.95	1.14	1.21	1.17	1.24	1.11	1.18	0.96	1.03	61.34	4.79
Q8WTY4	Anamorsin OS=Mus musculus GN=Ciapin1 PE=1 SV=1 - [CPIN1_MOUSE]	44.98	4	8	8	58	0.95	0.78	0.80	0.83	0.91	1.12	1.16	1.17	1.23	1.08	1.11	1.04	1.05	33.41	5.20
Q3UR78	Neuropeptide-like protein C4orf48 homolog OS=Mus musculus GN=Gm1673 PE=2 SV=2 - [CD048_MOUSE]	11.11	2	1	1	2	1.01	1.28	1.26	0.97	0.96	0.98	0.97	1.17	1.15	0.96	0.95	1.11	1.10	9.80	8.94
D3Z7G5	AN1-type zinc finger protein 1 OS=Mus musculus GN=Zfand1 PE=2 SV=1 - [D3Z7G5_MOUSE]	5.80	3	1	1	2	1.03	1.11	1.08	1.00	0.97	1.13	1.09	1.17	1.13	1.12	1.09	1.30	1.27	22.70	8.87
E9PUI5	Formin-binding protein 1-like OS=Mus musculus GN=Fnbp1l PE=2 SV=1 - [E9PUI5_MOUSE]	30.54	4	15	15	69	0.95	0.93	1.02	0.92	0.97	1.15	1.18	1.17	1.32	1.08	1.21	1.08	1.15	70.42	6.73
P23780	Beta-galactosidase OS=Mus musculus GN=Glb1 PE=2 SV=1 - [BGAL_MOUSE]	9.12	2	5	5	9	1.02	0.84	0.82	1.06	1.02	1.15	1.24	1.17	1.21	1.11	1.06	1.12	1.09	73.07	7.47
A2AEW8	GRIP1-associated protein 1 OS=Mus musculus GN=Gripap1 PE=2 SV=1 - [A2AEW8_MOUSE]	68.58	4	2	51	319	1.01	1.10	1.22	0.97	0.82	1.35	1.21	1.17	1.28	1.13	1.03	1.04	1.16	95.85	5.17
Q9CR61	NADH dehydrogenase [ubiquinone] 1 beta subcomplex subunit 7 OS=Mus musculus GN=Ndufb7 PE=1 SV=3 - [NDUB7_MOUSE]	59.12	1	7	7	39	0.93	0.96	0.98	0.95	1.14	1.11	1.31	1.17	1.20	1.09	1.19	0.97	1.07	16.32	8.18

A8Y5G5	Protein Zfyve9 OS=Mus musculus GN=Zfyve9 PE=2 SV=1 - [A8Y5G5_MOUSE]	9.19	2	7	7	23	0.88	1.20	1.20	0.95	1.00	1.12	1.28	1.17	1.27	1.01	1.10	1.01	1.17	146.24	5.01
F6T9H9	Myotubularin-related protein 1 (Fragment) OS=Mus musculus GN=Mtmr1 PE=4 SV=1 - [F6T9H9_MOUSE]	22.26	5	1	5	11	0.99	1.31	1.31	1.11	1.12	0.90	0.90	1.17	1.17	1.40	1.41	0.94	0.95	33.91	6.44
Q61645	Protein hairless OS=Mus musculus GN=Hr PE=2 SV=2 - [HAIR_MOUSE]	0.76	1	1	1	1	1.25	0.93	0.75	1.12	0.90	1.19	0.95	1.17	0.93	1.13	0.91	1.20	0.96	127.11	7.39
Q9Z2B9	Ribosomal protein S6 kinase alpha-4 OS=Mus musculus GN=Rps6ka4 PE=1 SV=2 - [KS6A4_MOUSE]	1.68	1	1	1	2	1.03	1.24	1.20	1.09	1.05	1.04	1.00	1.17	1.13	1.09	1.06	1.21	1.18	85.60	8.27
P63158	High mobility group protein B1 OS=Mus musculus GN=Hmgb1 PE=1 SV=2 - [HMGB1_MOUSE]	65.58	2	9	16	262	0.97	0.70	0.64	1.01	1.02	1.14	1.12	1.17	1.20	1.11	1.09	0.96	0.96	24.88	5.74
O89032-3	Isoform 3 of SH3 and PX domain-containing protein 2A OS=Mus musculus GN=Sh3pxd2a - [SPD2A_MOUSE]	10.45	3	7	7	9	1.14	1.00	0.83	1.01	0.89	1.10	1.00	1.17	1.01	1.08	0.98	1.09	0.93	118.96	8.18
A2AJH3	Glycylpeptide N-tetradecanoyltransferase 2 OS=Mus musculus GN=Nmt2 PE=2 SV=1 - [A2AJH3_MOUSE]	19.08	3	6	8	20	1.02	0.89	0.85	0.93	0.99	1.00	1.06	1.17	1.14	1.06	1.05	0.94	0.92	56.95	8.16
Q60605-2	Isoform Smooth muscle of Myosin light polypeptide 6 OS=Mus musculus GN=Myl6 - [MYL6_MOUSE]	76.16	1	1	10	375	1.02	0.96	0.96	0.96	0.97	1.11	1.11	1.17	1.13	1.13	1.13	1.08	1.04	16.95	4.55
Q6RUT7	Protein CCSMST1 OS=Mus musculus GN=Ccsmst1 PE=3 SV=1 - [CSMT1_MOUSE]	18.38	1	1	1	1	0.92	1.05	1.14	0.95	1.03	1.46	1.58	1.17	1.27	1.33	1.44	1.26	1.37	15.34	4.93
F6XQW1	Cohesin subunit SA-1 (Fragment) OS=Mus musculus GN=Stag1 PE=4 SV=1 - [F6XQW1_MOUSE]	2.88	5	1	2	3	0.83	1.31	1.58	0.92	1.12	1.13	1.36	1.17	1.41	0.91	1.11	0.83	1.00	99.93	5.26
Q8R2R3	Alpha- and gamma-adaptin-binding protein p34 OS=Mus musculus GN=Aagab PE=2 SV=2 - [AAGAB_MOUSE]	4.75	1	1	1	2	1.03	1.29	1.25	1.02	0.99	0.90	0.88	1.17	1.14	1.12	1.09	1.43	1.40	34.49	4.64
Q8BLQ9-	Isoform 3 of Cell adhesion molecule 2 OS=Mus musculus GN=Cadm2 - [CADM2_MOUSE]	57.22	5	15	16	106	0.94	0.94	0.99	0.98	1.08	1.09	1.20	1.17	1.18	1.01	1.05	0.99	1.02	43.49	5.33

D3YV27	Collagen alpha-2(IV) chain (Fragment) OS=Mus musculus GN=Col4a2 PE=2 SV=1 - [D3YV27_MOUSE]	4.05	2	1	1	4	0.92	0.95	1.03	0.89	0.97	1.06	1.15	1.17	1.27	1.12	1.23	0.94	1.02	29.08	6.57
Q7TNC4-	Isoform 2 of Putative RNA-binding protein Luc7-like 2 OS=Mus musculus GN=Luc7l2 - [LC7L2_MOUSE]	25.54	7	5	8	17	1.03	0.97	0.90	1.03	1.00	1.09	1.09	1.17	1.12	1.06	0.96	1.01	0.98	38.59	9.91
B2KFM4	Transcription factor SOX-5 OS=Mus musculus GN=Sox5 PE=2 SV=1 - [B2KFM4_MOUSE]	5.60	10	3	3	6	0.93	0.81	0.93	0.85	0.94	1.14	1.25	1.17	1.28	0.97	1.10	0.88	0.97	75.16	6.27
Q0VBD2	Protein MCM10 homolog OS=Mus musculus GN=Mcm10 PE=2 SV=1 - [MCM10_MOUSE]	2.37	1	1	1	1	1.43	1.28	0.89	1.52	1.06	1.27	0.88	1.17	0.82	1.13	0.79	1.92	1.34	98.34	8.85
E9Q9Q2	Protein R3hdm1 OS=Mus musculus GN=R3hdm1 PE=2 SV=1 - [E9Q9Q2_MOUSE]	5.55	1	2	4	9	1.17	1.22	1.04	0.96	0.82	1.11	0.95	1.17	1.00	1.05	0.90	1.07	0.92	124.31	8.70
O35618	Protein Mdm4 OS=Mus musculus GN=Mdm4 PE=1 SV=2 - [MDM4_MOUSE]	2.25	2	1	1	2	1.09	1.81	1.66	1.01	0.93	1.18	1.08	1.17	1.08	1.11	1.03	1.13	1.04	54.93	5.35
Q62189	U1 small nuclear ribonucleoprotein A OS=Mus musculus GN=Snrpa PE=2 SV=3 - [SNRPA_MOUSE]	23.34	4	4	5	23	1.03	1.10	1.15	0.96	1.00	1.16	1.16	1.17	1.17	1.11	1.13	1.18	1.14	31.81	9.80
Q91X51	Golgi reassembly-stacking protein 1 OS=Mus musculus GN=Gorasp1 PE=2 SV=3 - [GORS1_MOUSE]	15.70	1	4	4	7	1.10	1.28	1.11	1.02	0.93	1.25	1.18	1.17	1.04	1.05	0.91	1.19	1.03	46.85	4.65
D3Z6H8	S-adenosylmethionine decarboxylase proenzyme OS=Mus musculus GN=Amd2 PE=3 SV=1 - [D3Z6H8_MOUSE]	2.40	2	1	1	2	0.80	0.76	0.95	0.75	0.95	1.09	1.37	1.17	1.47	0.95	1.19	0.92	1.15	38.27	6.42
Q9D937	MCG127334 OS=Mus musculus GN=1810009A15Rik PE=2 SV=1 - [Q9D937_MOUSE]	22.76	1	2	2	3	1.16	0.90	0.78	1.19	1.03	1.22	1.05	1.17	1.01	1.33	1.15	1.21	1.05	14.09	11.47
Q3UJP5	Protein C8orf37 homolog OS=Mus musculus PE=1 SV=1 - [CH037_MOUSE]	54.07	1	1	9	42	1.03	0.90	0.87	0.89	0.87	0.95	0.92	1.17	1.13	0.95	0.93	1.28	1.24	23.83	6.86
D3YZP9	Coiled-coil domain-containing protein 6 OS=Mus musculus GN=Ccdc6 PE=3 SV=1 - [CCDC6_MOUSE]	38.59	3	21	21	135	1.04	0.94	0.92	0.90	0.87	1.14	1.10	1.17	1.13	1.08	1.05	1.03	1.00	52.91	7.34

Q9D4H2	GRIP and coiled-coil domain-containing protein 1 OS=Mus musculus GN=Gcc1 PE=1 SV=2 - [GCC1_MOUSE]	3.34	2	2	2	9	0.85	0.96	1.08	0.94	1.08	1.13	1.25	1.17	1.40	1.04	1.09	0.96	1.16	87.62	5.54
O35988	Syndecan-4 OS=Mus musculus GN=Sdc4 PE=1 SV=1 - [SDC4_MOUSE]	7.07	1	1	1	3	1.19	1.00	0.84	0.99	0.83	1.31	1.09	1.17	0.98	0.99	0.83	1.06	0.89	21.47	4.41
A2AH48	Retrotransposon gag domain-containing protein 1 OS=Mus musculus GN=Rgag1 PE=2 SV=1 - [A2AH48_MOUSE]	1.90	3	1	1	3	1.06	0.92	0.87	0.85	0.80	1.66	1.56	1.17	1.10	1.10	1.04	0.64	0.60	125.40	6.62
P10518	Delta-aminolevulinic acid dehydratase OS=Mus musculus GN=Alad PE=1 SV=1 - [HEM2_MOUSE]	65.76	1	13	13	133	1.07	1.06	1.03	0.96	0.90	1.11	1.00	1.17	1.05	1.02	0.91	1.01	0.96	36.00	6.79
E9PV44	ATPase inhibitor, mitochondrial OS=Mus musculus GN=Atpif1 PE=2 SV=1 - [E9PV44_MOUSE]	32.43	2	3	3	9	1.04	1.19	1.10	0.95	0.92	1.07	1.00	1.17	1.14	1.12	1.08	1.16	1.03	8.78	9.31
Q9R049-2	Isoform 2 of E3 ubiquitin-protein ligase AMFR OS=Mus musculus GN=Amfr - [AMFR_MOUSE]	4.38	2	2	2	5	0.97	1.24	1.18	0.96	0.96	1.19	1.02	1.17	1.32	1.23	1.23	1.07	0.92	72.71	6.46
E9Q1P8	Interferon regulatory factor 2-binding protein 2 OS=Mus musculus GN=Irf2bp2 PE=1 SV=1 - [I2BP2_MOUSE]	27.72	1	9	11	24	0.98	0.93	0.94	1.00	1.00	1.11	1.19	1.17	1.20	1.03	1.15	0.93	0.97	59.25	8.69
Q9JMG7	Hepatoma-derived growth factor-related protein 3 OS=Mus musculus GN=Hdgfrp3 PE=1 SV=2 - [HDGR3_MOUSE]	38.12	2	7	9	43	1.18	0.75	0.63	1.02	0.89	1.18	1.03	1.17	1.07	1.02	0.88	1.03	0.86	22.42	8.40
Q3TVA9	Coiled-coil domain-containing protein 136 OS=Mus musculus GN=Ccdc136 PE=2 SV=2 - [CC136_MOUSE]	19.89	7	16	17	72	0.93	0.95	1.01	0.98	1.06	1.18	1.27	1.17	1.25	1.03	1.10	1.00	1.03	131.85	4.84
B1AS06	Disks large-associated protein 3 OS=Mus musculus GN=Dlgap3 PE=2 SV=1 - [B1AS06_MOUSE]	25.88	2	15	15	46	0.97	1.00	1.00	1.03	1.08	1.10	1.07	1.17	1.19	0.93	1.02	1.05	1.08	104.61	8.81
P56812	Programmed cell death protein 5 OS=Mus musculus GN=Pdcd5 PE=1 SV=3 - [PDCD5_MOUSE]	73.81	2	10	10	35	1.06	0.85	0.83	0.92	0.87	1.17	1.09	1.17	1.12	1.08	1.08	1.01	1.00	14.27	5.68

Q9JIX8-4	Isoform 4 of Apoptotic chromatin condensation inducer in the nucleus OS=Mus musculus GN=Acin1 - [ACINU_MOUSE]	21.65	11	22	22	83	1.03	0.90	0.87	0.97	0.94	1.18	1.11	1.17	1.14	1.11	1.08	1.04	1.00	146.21	6.13
Q6XE40	MAGUK p55 subfamily member 3 OS=Mus musculus GN=Mpp3 PE=2 SV=1 - [Q6XE40_MOUSE]	34.02	3	14	14	29	0.90	0.94	1.04	0.98	1.10	1.07	1.19	1.17	1.27	1.04	1.19	0.97	1.06	66.45	6.47
D3YWP3	MCG1034428 OS=Mus musculus GN=Rpl23a-ps3 PE=3 SV=1 - [D3YWP3_MOUSE]	51.28	7	10	10	73	1.10	0.77	0.71	1.08	0.97	1.16	1.05	1.17	1.06	1.11	1.04	0.97	0.88	17.69	10.42
Q9ERG0-2	Isoform Alpha of LIM domain and actin-binding protein 1 OS=Mus musculus GN=Lima1 - [LIMA1_MOUSE]	20.57	2	10	10	15	0.99	0.93	0.93	0.95	0.99	1.21	1.16	1.17	1.18	0.96	1.02	0.96	0.97	65.98	5.91
P51954	Serine/threonine-protein kinase Nek1 OS=Mus musculus GN=Nek1 PE=1 SV=2 - [NEK1_MOUSE]	1.91	2	2	2	5	1.17	0.94	0.80	1.16	1.06	1.28	1.20	1.17	1.17	1.24	1.15	1.09	0.98	136.61	5.45
Q9DC28-2	Isoform 2 of Casein kinase I isoform delta OS=Mus musculus GN=Csnk1d - [KC1D_MOUSE]	14.18	4	2	3	6	0.85	1.32	1.54	1.02	1.19	1.13	1.31	1.17	1.37	0.98	1.15	0.91	1.07	46.79	9.64
Q9QYC1-	Isoform 2 of Pecanex-like protein 1 OS=Mus musculus GN=Pcnx - [PCX1_MOUSE]	0.44	2	1	1	4	1.08	1.31	1.15	1.15	1.05	1.05	0.97	1.17	1.06	0.92	0.86	1.03	0.96	248.01	7.20
E9PUD0	Zinc finger Ran-binding domain-containing protein 2 OS=Mus musculus GN=Zranb2 PE=2 SV=1 - [E9PUD0_MOUSE]	34.47	5	9	9	73	0.97	0.80	0.83	0.96	0.99	1.09	1.09	1.17	1.19	1.05	1.01	0.87	0.88	33.24	9.92
B2RWE3	CDGSH iron sulfur domain 3 OS=Mus musculus GN=Cisd3 PE=2 SV=1 - [B2RWE3_MOUSE]	10.45	2	1	1	1	0.70	0.54	0.77	0.97	1.39	1.21	1.72	1.17	1.67	1.10	1.58	0.78	1.11	14.98	9.95
Q5SUT0	RNA-binding protein EWS OS=Mus musculus GN=Ewsr1 PE=2 SV=1 - [Q5SUT0_MOUSE]	15.70	4	8	8	130	1.00	0.89	0.94	0.94	0.93	1.17	1.17	1.17	1.17	1.08	1.11	1.00	1.01	64.95	9.38
Q9DBR7	Protein phosphatase 1 regulatory subunit 12A OS=Mus musculus GN=Ppp1r12a PE=1 SV=2 - [MYPT1_MOUSE]	43.83	2	46	48	168	1.06	0.90	0.85	0.96	0.93	1.20	1.14	1.17	1.11	1.10	1.03	0.98	0.95	114.93	5.49

Q8BVL9	Janus kinase and microtubule-interacting protein 1 OS=Mus musculus GN=Jakmip1 PE=1 SV=2 - [JKIP1_MOUSE]	36.42	7	19	21	52	1.00	0.97	1.00	0.91	0.89	1.14	1.16	1.17	1.09	1.06	1.07	1.05	1.04	73.09	6.09
G3UWF6	MCG1027453 OS=Mus musculus GN=2010015L04Rik PE=4 SV=1 - [G3UWF6_MOUSE]	15.50	8	2	2	4	1.01	0.92	0.97	0.84	0.85	1.04	1.11	1.17	1.16	1.07	1.14	1.00	1.07	14.93	4.86
Q61768	Kinesin-1 heavy chain OS=Mus musculus GN=Kif5b PE=1 SV=3 - [KINH_MOUSE]	46.42	2	25	34	104	1.01	0.89	0.97	0.98	0.94	1.12	1.08	1.17	1.17	1.14	1.11	1.09	1.07	109.48	6.44
Q8K019-2	Isoform 2 of Bcl-2-associated transcription factor 1 OS=Mus musculus GN=Bclaf1 - [BCLF1_MOUSE]	29.23	4	24	25	87	1.02	0.90	0.82	0.94	0.92	1.13	1.10	1.17	1.14	1.09	1.03	0.92	0.93	105.76	9.99
Q8BZB3	Transmembrane protein C15orf27 homolog OS=Mus musculus PE=2 SV=3 - [CO027_MOUSE]	10.59	1	3	3	11	1.07	1.28	1.19	1.21	1.13	1.03	1.01	1.17	1.09	1.05	1.04	1.11	1.00	58.86	4.87
Q9CR02-2	Isoform 2 of Translation machinery-associated protein 16 OS=Mus musculus GN=Tma16 - [TMA16_MOUSE]	17.72	2	2	2	4	1.16	0.85	0.74	0.95	0.82	1.23	1.05	1.17	1.01	1.07	0.92	0.97	0.84	18.36	6.01
O89086	Putative RNA-binding protein 3 OS=Mus musculus GN=Rbm3 PE=1 SV=1 - [RBM3_MOUSE]	34.64	2	4	4	22	0.95	0.99	1.01	0.88	0.91	1.26	1.34	1.17	1.23	1.01	1.05	0.98	1.06	16.59	7.50
A2A9W7	ADP-ribosylation factor-binding protein GGA3 OS=Mus musculus GN=Gga3 PE=2 SV=1 - [A2A9W7_MOUSE]	14.22	3	7	7	17	0.92	0.98	1.09	1.08	1.15	1.05	1.10	1.17	1.18	1.11	1.13	1.13	1.18	70.15	6.14
P17156	Heat shock-related 70 kDa protein 2 OS=Mus musculus GN=Hspa2 PE=1 SV=2 - [HSP72_MOUSE]	51.66	1	13	30	377	1.01	0.86	0.93	0.97	1.00	1.20	1.24	1.18	1.18	1.15	1.18	1.14	1.12	69.60	5.67
Q9DBP5	UMP-CMP kinase OS=Mus musculus GN=Cmpk1 PE=1 SV=1 - [KCY_MOUSE]	62.24	3	10	11	65	0.97	0.76	0.78	0.89	0.89	1.17	1.15	1.18	1.19	1.04	1.06	1.03	1.00	22.15	5.83
Q6XPS7	L-threonine aldolase OS=Mus musculus GN=Tha1 PE=2 SV=1 - [Q6XPS7_MOUSE]	5.75	1	2	2	4	1.08	1.14	1.06	1.12	1.04	1.19	1.10	1.18	1.09	1.17	1.08	1.23	1.14	43.47	7.20
Q80TM6-	Isoform 2 of R3H domain-containing protein 2 OS=Mus musculus GN=R3hdm2 - [R3HD2_MOUSE]	15.93	11	10	12	33	0.89	1.04	1.10	0.99	1.09	1.11	1.23	1.18	1.26	1.03	1.14	0.98	1.09	108.73	8.69

Q99020	Heterogeneous nuclear ribonucleoprotein A/B OS=Mus musculus GN=Hnrnpab PE=1 SV=1 - [ROAA_MOUSE]	44.91	3	14	15	132	1.01	0.87	0.87	0.95	0.93	1.22	1.15	1.18	1.17	1.10	1.10	1.01	1.03	30.81	7.91
Q6WQJ1	Sn1-specific diacylglycerol lipase alpha OS=Mus musculus GN=Dagla PE=1 SV=2 - [DGLA_MOUSE]	16.19	1	10	10	20	0.98	1.22	1.15	1.08	1.13	0.94	1.02	1.18	0.97	0.98	0.97	0.93	0.96	115.30	6.42
Q9JKN6	RNA-binding protein Nova-1 OS=Mus musculus GN=Nova1 PE=1 SV=2 - [NOVA1_MOUSE]	7.50	1	1	3	6	0.73	0.89	1.22	0.99	1.35	1.00	1.36	1.18	1.60	0.97	1.32	1.18	1.62	51.72	8.72
P61315	Galactose-3-O-sulfotransferase 3 OS=Mus musculus GN=Gal3st3 PE=2 SV=1 - [G3ST3_MOUSE]	2.78	1	1	1	2	1.21	1.45	1.20	1.03	0.85	1.00	0.83	1.18	0.97	1.16	0.96	1.38	1.14	49.25	9.76
P63216	Guanine nucleotide-binding protein G(I)/G(S)/G(O) subunit gamma-3 OS=Mus musculus GN=Gng3 PE=1 SV=1 - [GBG3_MOUSE]	62.67	1	3	3	162	0.93	0.96	0.99	0.98	1.09	1.23	1.31	1.18	1.24	1.09	1.16	0.93	1.03	8.30	7.78
Q8K1I7	WAS/WASL-interacting protein family member 1 OS=Mus musculus GN=Wipf1 PE=1 SV=1 - [WIPF1_MOUSE]	14.81	2	4	4	28	1.01	1.02	0.96	0.96	0.92	1.12	1.06	1.18	1.28	1.22	1.17	1.17	1.11	50.05	11.41
D6RDT4	DNA polymerase epsilon subunit 3 OS=Mus musculus GN=Pole3 PE=2 SV=1 - [D6RDT4_MOUSE]	21.57	2	1	1	2	0.96	0.64	0.66	0.90	0.93	1.18	1.22	1.18	1.22	1.03	1.07	1.00	1.04	5.58	6.60
Q61120	SHC-transforming protein 3 OS=Mus musculus GN=Shc3 PE=1 SV=2 - [SHC3_MOUSE]	18.99	1	8	8	19	0.91	0.83	1.09	0.91	1.04	1.09	1.16	1.18	1.21	1.03	1.09	0.99	1.04	52.09	7.30
Q9DCJ9	N-acetylneuraminate lyase OS=Mus musculus GN=Npl PE=1 SV=1 - [NPL_MOUSE]	22.19	1	6	6	10	1.22	1.23	0.96	0.97	0.79	1.16	0.92	1.18	1.01	1.06	0.90	1.11	0.88	35.11	8.03
P54797	Transport and Golgi organization 2 homolog OS=Mus musculus GN=Tango2 PE=2 SV=1 - [TNG2_MOUSE]	9.42	4	2	2	4	1.02	1.01	0.99	1.16	1.14	1.08	1.05	1.18	1.15	1.16	1.14	1.07	1.06	30.93	5.10
A0JNS2	Igdcc4 protein OS=Mus musculus GN=Igdcc4 PE=2 SV=1 - [A0JNS2_MOUSE]	11.20	5	2	2	4	0.96	1.13	1.18	0.92	0.97	1.21	1.26	1.18	1.22	1.10	1.16	1.06	1.11	24.98	4.75

Q99LX5	Multiple myeloma tumor-associated protein 2 homolog OS=Mus musculus GN=Mmtag2 PE=2 SV=1 - [MMTA2_MOUSE]	12.69	1	3	3	6	1.05	1.00	0.95	1.01	0.96	1.20	1.14	1.18	1.20	1.23	1.24	1.03	0.99	29.28	9.86
Q8CCI1	Protein Gm17641 OS=Mus musculus GN=Gm17641 PE=2 SV=1 - [Q8CCI1_MOUSE]	5.85	1	1	1	1	1.20	1.41	1.18	1.02	0.85	1.25	1.04	1.18	0.98	1.14	0.95	1.07	0.89	19.63	12.00
Q501J6	Probable ATP-dependent RNA helicase DDX17 OS=Mus musculus GN=Ddx17 PE=2 SV=1 - [DDX17_MOUSE]	37.38	3	14	20	51	0.92	0.92	0.98	0.99	0.98	1.06	1.24	1.18	1.18	1.04	1.13	1.05	1.10	72.35	8.59
F7C957	PDZ and LIM domain protein 2 (Fragment) OS=Mus musculus GN=Pdlim2 PE=4 SV=1 - [F7C957_MOUSE]	7.28	2	1	1	2	1.02	1.13	1.10	0.97	0.95	1.02	1.00	1.18	1.15	1.11	1.09	1.22	1.20	16.71	8.60
Q5BL07-2	Isoform 2 of Peroxisome biogenesis factor 1 OS=Mus musculus GN=Pex1 - [PEX1_MOUSE]	1.13	2	1	1	1	0.76	2.24	2.92	1.11	1.45	1.06	1.37	1.18	1.53	0.92	1.20	0.89	1.16	136.66	6.21
Q8VHV1	Brain and acute leukemia cytoplasmic protein OS=Mus musculus GN=Baalc PE=2 SV=3 - [BAALC_MOUSE]	34.48	3	4	4	20	0.98	0.98	0.96	1.01	1.00	1.14	1.16	1.18	1.20	1.04	1.04	0.95	0.96	15.51	7.06
Q80TY4	Suppression of tumorigenicity 18 protein OS=Mus musculus GN=St18 PE=2 SV=2 - [ST18_MOUSE]	2.30	6	1	2	4	0.90	1.19	1.32	1.01	1.12	1.24	1.37	1.18	1.31	1.16	1.29	1.27	1.42	114.68	6.44
A6H644	MCG130490 OS=Mus musculus GN=Ppp1r12b PE=2 SV=1 - [A6H644_MOUSE]	33.97	3	26	28	69	1.02	0.94	0.98	0.99	0.95	1.23	1.19	1.18	1.12	1.07	1.02	1.03	1.00	110.91	5.74
Q7TSC1	Protein PRRC2A OS=Mus musculus GN=Prrc2a PE=1 SV=1 - [PRC2A_MOUSE]	28.82	2	40	41	161	1.07	0.99	0.97	1.01	0.95	1.21	1.10	1.18	1.13	1.15	1.06	1.13	1.07	229.06	9.39
O35387	HCLS1-associated protein X-1 OS=Mus musculus GN=Hax1 PE=1 SV=1 - [HAX1_MOUSE]	32.50	1	5	5	9	1.14	1.14	1.08	1.26	1.10	1.23	1.03	1.18	1.10	1.06	0.93	1.40	1.23	31.64	4.91
Q9WUC3	Lymphocyte antigen 6H OS=Mus musculus GN=Ly6h PE=2 SV=2 - [LY6H_MOUSE]	40.29	2	5	5	12	1.20	1.18	1.03	1.13	0.91	1.25	1.12	1.18	1.04	1.35	1.05	1.48	1.16	14.66	7.53
Q05DI7	Mtvr2 protein OS=Mus musculus GN=Fam89b PE=2 SV=1 - [Q05DI7_MOUSE]	18.12	3	2	2	6	1.09	1.04	0.95	1.02	0.94	1.24	1.13	1.18	1.06	1.27	1.06	1.28	1.07	14.92	10.20

B1AY10	Transcriptional repressor NF-X1 OS=Mus musculus GN=Nfx1 PE=2 SV=1 - [NFX1_MOUSE]	5.30	3	5	5	9	1.13	1.13	0.93	0.97	0.88	1.14	1.04	1.18	1.08	1.16	1.02	1.03	0.90	123.73	8.31
H3BL05	Zinc finger protein 428 (Fragment) OS=Mus musculus GN=Zfp428 PE=2 SV=1 - [H3BL05_MOUSE]	31.37	3	3	3	17	1.03	0.95	0.89	1.04	0.93	1.24	1.16	1.18	1.15	1.04	1.02	1.02	0.96	16.31	4.21
F8WIS9	Calcium/calmodulin-dependent protein kinase type II subunit alpha OS=Mus musculus GN=Camk2a PE=2 SV=1 - [F8WIS9_MOUSE]	53.58	4	1	21	207	1.40	1.28	0.91	1.30	0.93	0.84	0.60	1.18	0.84	0.87	0.62	1.17	0.84	55.31	7.44
Q8R4U7	Leucine zipper protein 1 OS=Mus musculus GN=Luzp1 PE=1 SV=2 - [LUZP1_MOUSE]	48.69	3	44	44	147	0.95	0.89	0.96	0.90	0.96	1.11	1.20	1.18	1.20	0.99	1.02	0.88	0.95	119.24	7.99
Q6NZP2	Vasculin-like protein 1 OS=Mus musculus GN=Gpbp1l1 PE=1 SV=1 - [GPBL1_MOUSE]	5.29	1	2	2	3	1.13	1.00	0.88	1.02	0.90	1.17	1.02	1.18	1.04	1.09	0.97	1.15	1.02	51.93	7.28
P35969	Vascular endothelial growth factor receptor 1 OS=Mus musculus GN=Flt1 PE=1 SV=1 - [VGFR1_MOUSE]	2.10	17	2	3	6	1.03	1.24	1.18	1.02	0.98	1.02	0.99	1.18	1.13	1.13	1.09	1.17	1.13	149.78	8.31
D3Z3G7	Protein yippee-like 3 OS=Mus musculus GN=Ypel3 PE=2 SV=1 - [D3Z3G7_MOUSE]	14.46	3	1	1	2	0.99	0.95	0.96	1.00	1.01	1.19	1.20	1.18	1.19	1.00	1.01	0.94	0.95	9.10	8.84
P99028	Cytochrome b-c1 complex subunit 6, mitochondrial OS=Mus musculus GN=Uqcrh PE=1 SV=2 - [QCR6_MOUSE]	74.16	2	8	8	247	1.07	0.95	0.87	0.95	0.88	1.18	1.08	1.18	1.08	1.06	1.02	1.03	0.95	10.43	4.87
P01806	Ig heavy chain V region 441 OS=Mus musculus PE=4 SV=1 - [HVM36_MOUSE]	13.79	5	1	1	1	1.23	3.72	3.00	1.34	1.08	1.53	1.24	1.18	0.95	1.11	0.90	0.99	0.81	12.90	8.27
Q810B9	SLIT and NTRK-like protein 3 OS=Mus musculus GN=Slitrk3 PE=2 SV=2 - [SLIK3_MOUSE]	1.33	1	1	1	1	1.15	0.96	0.84	0.94	0.82	1.19	1.03	1.18	1.02	0.98	0.85	0.81	0.71	109.34	7.47
Q8BHC9	Alpha-(1,3)-fucosyltransferase 11 OS=Mus musculus GN=Fut11 PE=2 SV=1 - [FUT11_MOUSE]	4.09	1	1	1	1	0.92	0.84	0.92	0.83	0.90	1.04	1.12	1.18	1.28	1.25	1.36	0.95	1.03	55.50	6.23
Q3UMT1	Protein phosphatase 1 regulatory subunit 12C OS=Mus musculus GN=Ppp1r12c PE=1 SV=1 - [PP12C_MOUSE]	41.43	2	24	24	88	1.07	1.01	0.94	0.98	0.91	1.14	1.06	1.18	1.06	1.11	1.01	1.06	1.00	84.63	6.00

P97461	40S ribosomal protein S5 OS=Mus musculus GN=Rps5 PE=2 SV=3 - [RS5_MOUSE]	21.08	3	4	4	9	1.07	0.91	0.85	1.12	1.01	1.18	1.06	1.18	1.17	1.20	1.13	1.20	1.02	22.88	9.72
D3YYW8	RNA-binding protein 42 OS=Mus musculus GN=Rbm42 PE=2 SV=1 - [D3YYW8_MOUSE]	4.68	3	1	1	1	0.85	0.98	1.14	0.89	1.05	1.16	1.35	1.18	1.38	1.42	1.67	1.57	1.85	47.18	9.50
D3YZC9	Splicing factor 1 OS=Mus musculus GN=Sf1 PE=2 SV=1 - [D3YZC9_MOUSE]	29.95	10	12	12	99	1.06	1.06	0.99	0.97	0.90	1.11	1.06	1.18	1.10	1.11	1.02	1.13	1.06	61.72	9.29
Q62219-2	Isoform 2 of Transforming growth factor beta-1-induced transcript 1 protein OS=Mus musculus GN=Tgfb1i1 - [TGFI1_MOUSE]	34.01	11	9	9	23	0.89	0.87	1.02	0.87	0.95	1.06	1.17	1.18	1.39	1.16	1.19	0.92	1.03	48.20	7.23
Q8VEB3	UNC119-binding protein C5orf30 homolog OS=Mus musculus GN=D1Ertd622e PE=2 SV=1 - [CE030_MOUSE]	10.14	1	2	2	3	0.86	0.96	1.11	0.90	1.04	1.10	1.27	1.18	1.36	1.01	1.17	0.96	1.11	23.12	9.45
Q9D7P6	Iron-sulfur cluster assembly enzyme ISCU, mitochondrial OS=Mus musculus GN=Iscu PE=1 SV=1 - [ISCU_MOUSE]	54.17	4	9	9	25	1.05	0.93	0.86	0.93	0.85	1.10	1.05	1.18	1.08	1.09	1.04	0.99	0.93	18.09	9.29
Q9EPU5	Tumor necrosis factor receptor superfamily member 21 OS=Mus musculus GN=Tnfrsf21 PE=1 SV=2 - [TNR21_MOUSE]	6.56	1	4	4	8	1.07	1.14	1.03	1.01	0.93	1.10	1.02	1.18	1.09	1.09	1.03	1.12	1.03	71.94	7.46
P19426-2	Isoform 2 of Negative elongation factor E OS=Mus musculus GN=Nelfe - [NELFE_MOUSE]	27.25	3	7	7	23	1.09	0.99	0.92	1.01	0.92	1.18	1.04	1.18	1.04	1.12	1.01	1.08	0.99	41.46	9.39
Q8C0L6-2	Isoform 2 of Peroxisomal N(1)-acetyl-spermine/spermidine oxidase OS=Mus musculus GN=Paox - [PAOX_MOUSE]	6.25	3	1	1	2	1.41	4.48	3.18	1.36	0.97	1.67	1.18	1.18	0.83	1.12	0.80	1.32	0.94	25.25	4.93
Q9DB20	ATP synthase subunit O, mitochondrial OS=Mus musculus GN=Atp5o PE=1 SV=1 - [ATPO_MOUSE]	54.93	3	12	12	81	0.98	0.94	0.96	0.97	0.98	1.20	1.20	1.18	1.20	1.13	1.16	1.10	1.16	23.35	9.99
Q9CYL5	Golgi-associated plant pathogenesis-related protein 1 OS=Mus musculus GN=Glipr2 PE=2 SV=3 - [GAPR1_MOUSE]	16.88	2	2	2	8	1.16	1.12	0.99	1.00	0.87	1.24	1.08	1.18	1.03	1.19	1.07	1.13	0.99	17.08	9.51
E9Q941	Protein Cdh18 OS=Mus musculus GN=Cdh18 PE=2 SV=1 - [E9Q941_MOUSE]	2.26	2	1	1	2	1.16	1.15	0.99	1.10	0.95	1.13	0.97	1.18	1.01	1.18	1.02	1.04	0.89	63.41	5.62

Q3U3E2	Protein FAM117B OS=Mus musculus GN=Fam117b PE=1 SV=1 - [F117B_MOUSE]	18.15	1	7	7	32	1.04	0.97	0.90	0.97	0.87	1.10	1.00	1.18	1.04	1.06	0.99	1.07	0.98	61.31	9.95
Q80XP8	Protein FAM76B OS=Mus musculus GN=Fam76b PE=2 SV=1 - [FA76B_MOUSE]	7.37	3	2	2	4	1.02	1.36	1.47	1.02	1.00	1.21	1.18	1.18	1.15	1.20	1.18	1.13	1.10	38.53	9.29
O08539	Myc box-dependent-interacting protein 1 OS=Mus musculus GN=Bin1 PE=1 SV=1 - [BIN1_MOUSE]	56.12	1	3	30	771	0.97	0.99	1.00	0.89	0.94	1.09	1.10	1.18	1.20	1.09	1.12	1.11	1.17	64.43	5.03
Q99KS2	Neugrin OS=Mus musculus GN=Ngrn PE=2 SV=3 - [NGRN_MOUSE]	4.44	1	1	1	2	1.07	1.06	0.99	0.99	0.93	1.19	1.11	1.18	1.10	1.15	1.08	1.26	1.19	32.43	8.90
I1E4X0	Disks large-associated protein 4 OS=Mus musculus GN=Dlgap4 PE=4 SV=1 - [I1E4X0_MOUSE]	40.57	6	2	11	39	1.12	0.80	0.72	1.01	0.90	1.14	1.01	1.18	1.05	1.01	0.90	1.03	0.92	26.89	4.88
Q6PD31-2	Isoform 2 of Trafficking kinesin-binding protein 1 OS=Mus musculus GN=Trak1 - [TRAK1_MOUSE]	4.43	2	2	2	2	0.75	0.75	1.00	0.83	1.09	0.86	1.13	1.18	1.56	0.86	1.14	0.96	1.28	69.57	5.19
F6VAL0	Mitochondrial fission factor (Fragment) OS=Mus musculus GN=Mff PE=4 SV=1 - [F6VAL0_MOUSE]	52.38	2	1	4	10	0.87	1.27	1.46	0.96	1.10	1.16	1.34	1.18	1.36	1.18	1.37	1.15	1.33	16.29	5.17
F8VPM7	ELKS/Rab6-interacting/CAST family member 1 OS=Mus musculus GN=Erc1 PE=2 SV=1 - [F8VPM7_MOUSE]	36.34	3	2	44	161	0.97	1.15	1.12	0.97	1.01	1.07	1.04	1.18	1.23	1.01	1.12	1.20	1.17	128.25	5.91
D3YX85	Arf-GAP with SH3 domain, ANK repeat and PH domain-containing protein 2 OS=Mus musculus GN=Asap2 PE=2 SV=2 - [D3YX85_MOUSE]	15.08	3	1	10	25	1.00	0.95	0.94	0.99	0.98	1.02	1.01	1.18	1.17	1.08	1.08	1.01	1.01	106.34	6.54
Q8R034	Anaphase-promoting complex subunit 13 OS=Mus musculus GN=Anapc13 PE=2 SV=1 - [APC13_MOUSE]	79.73	1	3	3	9	1.10	1.08	1.06	0.96	0.98	1.16	1.06	1.18	1.21	1.16	1.06	1.09	1.15	8.34	4.18
Q5RIM6	Nuclear receptor co-repressor 1, isoform CRA_a OS=Mus musculus GN=Ncor1 PE=4 SV=1 - [Q5RIM6_MOUSE]	6.85	15	10	12	22	1.00	0.97	0.99	0.95	0.96	1.10	1.05	1.18	1.10	1.15	1.06	1.12	1.07	270.62	6.93

F8VQJ3	Laminin subunit gamma-1 OS=Mus musculus GN=Lamc1 PE=2 SV=1 - [F8VQJ3_MOUSE]	10.52	4	14	14	23	0.96	1.05	1.05	0.90	0.96	1.05	1.08	1.18	1.22	1.15	1.10	1.05	0.98	177.07	5.19
F7DBB3	Protein Ahnak2 (Fragment) OS=Mus musculus GN=Ahnak2 PE=2 SV=1 - [F7DBB3_MOUSE]	19.73	1	2	11	18	1.02	0.79	0.78	0.89	0.87	1.17	1.14	1.18	1.15	1.17	1.15	1.01	0.99	166.39	5.78
B1AR74	Breast carcinoma-amplified sequence 3 homolog OS=Mus musculus GN=Bcas3 PE=4 SV=1 - [B1AR74_MOUSE]	13.26	3	1	10	31	1.21	0.88	0.72	1.03	0.85	1.25	1.02	1.18	0.97	1.12	0.93	1.15	0.95	102.71	7.49
Q6P8I4	PEST proteolytic signal-containing nuclear protein OS=Mus musculus GN=Pcnp PE=1 SV=1 - [PCNP_MOUSE]	56.74	4	7	7	36	1.05	0.98	0.91	0.93	0.90	1.13	1.08	1.18	1.12	1.11	1.05	1.07	1.02	18.95	7.49
Q80X50	Ubiquitin-associated protein 2-like OS=Mus musculus GN=Ubap2l PE=1 SV=1 - [UBP2L_MOUSE]	35.50	4	2	25	79	1.09	0.78	0.68	0.88	0.82	1.13	1.01	1.18	1.09	1.00	0.94	0.94	0.88	116.73	7.11
O54988-2	Isoform 2 of STE20-like serine/threonine-protein kinase OS=Mus musculus GN=Slk - [SLK_MOUSE]	39.02	5	28	30	80	1.00	0.93	0.97	0.87	0.93	1.15	1.15	1.18	1.28	1.11	1.14	1.06	1.09	137.63	5.08
Q9DC07	LIM zinc-binding domain-containing Nebulette OS=Mus musculus GN=Nebl PE=2 SV=1 - [LNEBL_MOUSE]	85.56	6	9	20	229	1.01	0.88	0.86	0.96	0.93	1.15	1.13	1.18	1.14	1.02	1.02	0.95	0.96	31.09	8.31
Q7TQF7	Amphiphysin OS=Mus musculus GN=Amph PE=1 SV=1 - [AMPH_MOUSE]	56.41	1	28	30	308	0.98	0.85	0.88	0.85	0.89	1.14	1.17	1.18	1.23	1.02	1.05	0.97	1.02	74.97	4.63
P29595	NEDD8 OS=Mus musculus GN=Nedd8 PE=1 SV=2 - [NEDD8_MOUSE]	53.09	1	5	5	84	1.02	0.99	0.98	0.93	0.93	1.11	1.12	1.18	1.13	1.08	1.12	1.08	1.04	8.97	7.25
D3Z5F7	Protein Gm20521 OS=Mus musculus GN=Gm20521 PE=2 SV=1 - [D3Z5F7_MOUSE]	33.33	11	8	8	17	1.00	0.92	0.94	0.90	0.97	1.15	1.26	1.18	1.34	1.00	1.15	1.12	1.22	37.22	8.40
Q5UE59	Kinesin light chain 1 OS=Mus musculus GN=Klc1 PE=2 SV=1 - [Q5UE59_MOUSE]	54.43	8	19	25	96	0.99	0.81	0.86	0.85	0.85	1.09	1.14	1.18	1.19	1.02	1.02	0.98	1.04	61.59	5.76
Q8VHR5	Transcriptional repressor p66-beta OS=Mus musculus GN=Gatad2b PE=1 SV=1 - [P66B_MOUSE]	36.20	2	13	13	50	1.02	0.99	0.96	0.97	0.94	1.14	1.19	1.18	1.16	1.12	1.11	1.16	1.09	65.37	9.70

Q6P6N5	Sprouty-related, EVH1 domain-containing protein 3 OS=Mus musculus GN=Spred3 PE=2 SV=1 - [SPRE3_MOUSE]	10.29	1	3	3	6	1.00	1.15	1.10	0.94	0.94	1.14	1.13	1.18	1.19	1.09	1.04	1.11	1.11	42.77	8.34
Q8BWY4	Metallo-beta-lactamase domain-containing protein 1 OS=Mus musculus GN=Mblac1 PE=2 SV=1 - [MBLC1_MOUSE]	4.23	1	1	1	2	1.15	0.69	0.60	0.91	0.79	1.19	1.03	1.18	1.03	1.08	0.94	0.89	0.78	26.91	4.88
P12961	Neuroendocrine protein 7B2 OS=Mus musculus GN=Scg5 PE=1 SV=1 - [7B2_MOUSE]	42.92	1	7	7	34	1.23	0.90	0.73	1.04	0.83	1.15	0.94	1.18	0.95	1.09	0.89	1.04	0.86	23.85	5.81
P60853	Leucine zipper putative tumor suppressor 1 OS=Mus musculus GN=Lzts1 PE=2 SV=3 - [LZTS1_MOUSE]	22.54	1	13	13	22	1.00	0.88	0.97	0.94	0.98	1.17	1.23	1.18	1.18	1.09	1.18	0.93	1.07	67.25	7.56
Q99NE5-7	Isoform 7 of Regulating synaptic membrane exocytosis protein 1 OS=Mus musculus GN=Rims1 - [RIMS1_MOUSE]	34.97	3	2	29	79	0.81	1.03	1.21	1.03	1.29	1.04	1.22	1.18	1.40	1.10	1.32	0.85	1.12	132.91	9.33
Q8CIN6	CUGBP Elav-like family member 3 OS=Mus musculus GN=Celf3 PE=2 SV=1 - [CELF3_MOUSE]	7.31	2	1	2	5	1.03	0.75	0.73	0.75	0.73	1.14	1.10	1.18	1.15	0.91	0.89	0.93	0.91	50.49	8.68
A2A5Y6	Microtubule-associated protein tau OS=Mus musculus GN=Mapt PE=2 SV=1 - [A2A5Y6_MOUSE]	61.01	5	12	45	748	1.01	0.91	0.92	0.86	0.86	1.14	1.13	1.18	1.16	1.00	0.96	0.87	0.89	78.04	6.51
Q8BYN3	Inositol-tetrakisphosphate 1-kinase OS=Mus musculus GN=Itpk1 PE=2 SV=1 - [ITPK1_MOUSE]	5.01	1	1	1	4	0.96	1.28	1.33	1.18	1.22	0.93	0.96	1.18	1.23	0.99	1.03	1.23	1.28	46.12	6.29
Q6A028	Switch-associated protein 70 OS=Mus musculus GN=Swap70 PE=1 SV=2 - [SWP70_MOUSE]	11.28	1	5	5	7	0.97	1.18	1.22	0.99	1.00	1.08	1.11	1.18	1.16	1.11	1.14	1.07	1.13	68.95	6.05
Q9D7M8	DNA-directed RNA polymerase II subunit RPB4 OS=Mus musculus GN=Polr2d PE=2 SV=2 - [RPB4_MOUSE]	11.27	1	1	1	2	0.77	0.67	0.87	0.91	1.18	1.23	1.59	1.18	1.53	1.03	1.34	0.90	1.17	16.30	4.79
E9Q6J5	Protein Bod1l OS=Mus musculus GN=Bod1l PE=2 SV=1 - [E9Q6J5_MOUSE]	9.40	1	15	17	33	1.04	0.99	0.98	0.97	0.89	1.21	1.13	1.18	1.15	1.11	1.07	1.09	1.08	327.25	5.33

E9Q9V3	Nuclear receptor corepressor 2 OS=Mus musculus GN=Ncor2 PE=2 SV=1 - [E9Q9V3_MOUSE]	7.29	8	11	13	26	1.07	0.89	0.91	1.03	0.96	1.18	1.07	1.18	1.10	1.14	1.03	1.07	1.02	253.70	7.56
Q91UZ8	MCG141285 OS=Mus musculus GN=Pcdhb11 PE=2 SV=1 - [Q91UZ8_MOUSE]	2.38	8	2	2	2	1.17	1.07	0.91	1.05	0.90	1.21	1.03	1.18	1.01	1.27	1.09	1.08	0.93	87.61	4.73
Q9DBG5	Perilipin-3 OS=Mus musculus GN=Plin3 PE=1 SV=1 - [PLIN3_MOUSE]	31.58	1	9	9	17	1.02	1.00	1.06	0.90	0.96	1.14	1.12	1.18	1.14	1.16	1.09	1.05	1.12	47.23	5.62
Q9CX60	Protein LBH OS=Mus musculus GN=Lbh PE=1 SV=1 - [LBH_MOUSE]	80.95	2	6	6	19	1.01	0.95	0.91	0.91	0.85	1.16	1.12	1.18	1.16	1.10	1.02	0.99	0.95	12.13	4.31
Q76LS9-2	Isoform 2 of Protein FAM63A OS=Mus musculus GN=Fam63a - [FA63A_MOUSE]	2.61	2	1	1	2	1.06	0.89	0.84	1.26	1.18	1.11	1.05	1.18	1.11	1.11	1.05	1.08	1.02	50.18	4.74
Q5SP85-2	Isoform 2 of Coiled-coil domain-containing protein 85A OS=Mus musculus GN=Ccdc85a - [CC85A_MOUSE]	29.35	4	9	10	31	0.98	0.94	0.92	0.91	0.93	1.15	1.19	1.18	1.19	1.06	1.00	0.98	1.02	50.39	8.00
Q8BFY6	Peflin OS=Mus musculus GN=Pef1 PE=2 SV=1 - [PEF1_MOUSE]	7.64	1	2	2	2	1.30	1.08	0.83	1.09	0.84	0.89	0.69	1.18	0.91	1.12	0.86	1.02	0.79	29.21	6.30
Q80YR4-2	Isoform 2 of Zinc finger protein 598 OS=Mus musculus GN=Znf598 - [ZN598_MOUSE]	7.95	3	5	5	10	0.89	0.95	1.09	0.90	0.98	1.19	1.34	1.18	1.30	1.06	1.17	1.02	1.13	96.28	8.29
Q8K2L8	Trafficking protein particle complex subunit 12 OS=Mus musculus GN=Trappc12 PE=1 SV=2 - [TPC12_MOUSE]	11.29	2	5	5	9	0.97	1.00	0.99	1.04	1.03	1.03	1.08	1.18	1.12	1.05	1.10	1.15	1.22	87.64	4.81
O08715-2	Isoform 1 of A-kinase anchor protein 1, mitochondrial OS=Mus musculus GN=Akap1 - [AKAP1_MOUSE]	24.45	6	7	7	10	0.98	0.84	0.79	0.94	0.96	1.16	1.16	1.18	1.10	1.16	1.11	1.13	1.08	57.64	4.94
Q9CRB2	H/ACA ribonucleoprotein complex subunit 2 OS=Mus musculus GN=Nhp2 PE=2 SV=1 - [NHP2_MOUSE]	30.07	1	3	3	8	1.01	0.95	0.88	1.01	1.00	1.18	1.05	1.19	1.17	1.19	1.19	1.03	1.01	17.24	8.41
Q99LA2	Protein Zfp207 OS=Mus musculus GN=Zfp207 PE=2 SV=1 - [Q99LA2_MOUSE]	17.24	6	7	7	30	1.02	0.96	0.96	0.98	0.94	1.19	1.15	1.19	1.17	1.11	1.09	1.05	0.95	49.76	9.10
A2AR78	TRAF family member-associated NF-kappa-B activator OS=Mus musculus GN=Tank PE=2 SV=1 - [A2AR78_MOUSE]	21.94	7	5	5	6	0.91	0.86	0.98	0.79	1.02	1.33	1.32	1.19	1.28	0.98	1.08	0.89	0.93	40.88	5.66

F6VCZ4	Uncharacterized protein OS=Mus musculus GN=Zfp422-rs1 PE=4 SV=2 - [F6VCZ4_MOUSE]	14.48	153	1	2	3	0.94	1.44	1.52	0.94	1.00	1.30	1.37	1.19	1.25	1.23	1.31	1.42	1.51	16.86	9.26
Q80YE4-4	Isoform 4 of Serine/threonine-protein kinase LMTK1 OS=Mus musculus GN=Aatk - [LMTK1_MOUSE]	19.29	6	12	12	23	1.03	1.06	1.03	1.07	1.04	1.20	1.15	1.19	1.15	1.12	1.08	1.01	0.95	127.46	4.49
B9EHL3	Ppp1r3f protein OS=Mus musculus GN=Ppp1r3f PE=2 SV=1 - [B9EHL3_MOUSE]	2.04	4	1	1	2	1.21	1.76	1.46	1.24	1.03	1.23	1.01	1.19	0.98	1.13	0.94	1.13	0.94	77.42	4.59
Q9EPJ9-2	Isoform 2 of ADP-ribosylation factor GTPase-activating protein 1 OS=Mus musculus GN=Arfgap1 - [ARFG1_MOUSE]	53.32	3	19	19	326	1.03	0.99	0.97	0.96	0.94	1.16	1.14	1.19	1.15	1.07	1.05	1.04	1.01	42.93	5.54
E9QN52	Leucine-rich repeat flightless-interacting protein 2 OS=Mus musculus GN=Lrrfip2 PE=2 SV=1 - [E9QN52_MOUSE]	39.52	3	12	15	60	1.04	0.96	0.94	0.88	0.89	1.07	1.10	1.19	1.21	1.01	0.96	1.01	0.90	47.10	5.68
O09174	Alpha-methylacyl-CoA racemase OS=Mus musculus GN=Amacr PE=1 SV=4 - [AMACR_MOUSE]	12.34	1	2	2	2	0.98	1.02	1.04	1.10	1.12	1.20	1.23	1.19	1.21	1.60	1.64	0.93	0.95	41.68	7.40
O08532-2	Isoform 2B of Voltage-dependent calcium channel subunit alpha-2/delta-1 OS=Mus musculus GN=Cacna2d1 - [CA2D1_MOUSE]	42.16	4	1	33	148	0.90	1.19	1.32	1.17	1.30	1.05	1.16	1.19	1.31	1.16	1.29	1.24	1.38	122.44	5.20
E9QKK1	Centromere-associated protein E OS=Mus musculus GN=Cenpe PE=3 SV=1 - [E9QKK1_MOUSE]	1.01	2	1	3	5	1.09	0.99	0.91	0.90	0.83	1.19	1.09	1.19	1.09	1.05	0.97	0.94	0.87	286.04	5.33
Q8K199	COX assembly mitochondrial protein 2 homolog OS=Mus musculus GN=Cmc2 PE=2 SV=1 - [COXM2_MOUSE]	53.16	5	3	3	10	0.91	1.04	1.11	0.91	1.00	1.08	1.14	1.19	1.30	0.92	0.97	0.90	0.96	9.41	8.24
A2ANP1	2310028H24Rik protein OS=Mus musculus GN=Fam219a PE=2 SV=1 - [A2ANP1_MOUSE]	53.57	1	1	4	35	0.87	0.99	1.24	0.93	1.03	1.14	1.20	1.19	1.25	1.10	1.27	1.01	1.21	18.64	4.70
Q922H9-2	Isoform 2 of Zinc finger protein 330 OS=Mus musculus GN=Znf330 - [ZN330_MOUSE]	11.90	2	3	3	15	1.12	1.10	0.88	1.01	0.91	1.13	1.00	1.19	1.05	1.11	0.97	1.01	0.90	34.87	6.44

Q9CQE5	Regulator of G-protein signaling 10 OS=Mus musculus GN=Rgs10 PE=2 SV=1 - [RGS10_MOUSE]	43.09	2	6	6	37	0.99	0.83	0.84	0.89	0.91	1.06	1.06	1.19	1.11	1.03	1.02	0.97	0.98	21.14	6.81
Q9JLQ0	CD2-associated protein OS=Mus musculus GN=Cd2ap PE=1 SV=3 - [CD2AP_MOUSE]	26.84	1	15	15	35	1.09	0.91	0.81	0.95	0.85	1.17	1.04	1.19	1.08	1.09	1.02	0.99	0.91	70.41	6.38
Q8R344	Coiled-coil domain-containing protein 12 OS=Mus musculus GN=Ccdc12 PE=1 SV=2 - [CCD12_MOUSE]	39.16	1	6	6	18	1.11	0.86	0.73	1.04	0.82	1.28	1.06	1.19	0.97	1.08	0.85	0.95	0.86	18.88	7.21
Q9DCB8	Iron-sulfur cluster assembly 2 homolog, mitochondrial OS=Mus musculus GN=Isca2 PE=2 SV=2 - [ISCA2_MOUSE]	37.66	1	5	5	12	1.05	0.85	0.82	0.91	0.89	1.24	1.17	1.19	1.12	1.06	1.04	1.00	0.94	16.71	5.60
Q923D5	WW domain-binding protein 11 OS=Mus musculus GN=Wbp11 PE=1 SV=2 - [WBP11_MOUSE]	25.27	1	17	17	35	1.10	0.97	0.92	0.99	0.97	1.17	1.12	1.19	1.05	1.07	1.04	1.03	0.96	69.83	8.40
Q9CQT5	Proteasome maturation protein OS=Mus musculus GN=Pomp PE=2 SV=1 - [POMP_MOUSE]	10.64	1	2	2	2	1.05	0.85	0.81	0.94	0.89	1.18	1.11	1.19	1.13	1.17	1.11	1.12	1.07	15.76	5.49
P16951-2	Isoform 2 of Cyclic AMP-dependent transcription factor ATF-2 OS=Mus musculus GN=Atf2 - [ATF2_MOUSE]	27.51	8	8	10	24	1.02	0.95	0.97	1.00	0.94	1.16	1.16	1.19	1.16	1.16	1.11	1.07	1.04	42.32	7.17
Q7TQD2	Tubulin polymerization-promoting protein OS=Mus musculus GN=Tppp PE=1 SV=1 - [TPPP_MOUSE]	67.43	1	17	17	558	1.02	0.93	0.90	0.96	0.93	1.15	1.12	1.19	1.15	1.09	1.07	1.04	1.02	23.56	9.42
Q99KW3-	Isoform 1 of TRIO and F-actin-binding protein OS=Mus musculus GN=Triobp - [TARA_MOUSE]	2.76	7	1	2	2	1.00	0.78	0.78	1.03	1.03	1.16	1.16	1.19	1.19	1.13	1.13	0.93	0.94	66.94	6.30
Q3ULL6	Protein Upf3b OS=Mus musculus GN=Upf3b PE=2 SV=1 - [Q3ULL6_MOUSE]	28.18	1	6	10	18	1.10	0.96	0.99	1.09	0.90	1.11	1.05	1.19	1.05	1.31	1.09	1.09	1.00	57.03	9.44
P56183-2	Isoform 2 of Ribosomal RNA processing protein 1 homolog A OS=Mus musculus GN=Rrp1 - [RRP1_MOUSE]	3.84	3	1	1	3	0.71	1.07	1.49	1.03	1.44	1.11	1.54	1.19	1.71	1.12	1.57	1.73	2.50	49.98	8.24
Q61286-2	Isoform ALF1A of Transcription factor 12 OS=Mus musculus GN=Tcf12 - [HTF4_MOUSE]	4.11	3	1	2	2	1.07	1.20	1.11	0.89	0.83	1.09	1.01	1.19	1.10	1.14	1.06	1.26	1.18	72.89	7.02

E9Q7Q3	Tropomyosin alpha-3 chain OS=Mus musculus GN=Tpm3 PE=2 SV=1 - [E9Q7Q3_MOUSE]	60.08	3	2	22	418	1.04	0.92	0.88	0.97	0.91	1.36	1.36	1.19	1.24	1.07	1.20	0.98	1.04	28.72	4.72
Q8BWZ3-	Isoform 2 of N-alpha-acetyltransferase 25, NatB auxiliary subunit OS=Mus musculus GN=Naa25 - [NAA25_MOUSE]	1.81	2	1	1	2	1.30	1.28	0.98	1.48	1.13	1.20	0.92	1.19	0.91	1.11	0.85	1.39	1.07	107.45	6.24
O08784	Treacle protein OS=Mus musculus GN=Tcof1 PE=1 SV=1 - [TCOF_MOUSE]	16.67	5	17	17	47	1.00	0.92	0.94	1.03	1.03	1.04	1.06	1.19	1.24	1.16	1.22	1.01	1.00	134.92	9.35
Q61324-2	Isoform 2 of Aryl hydrocarbon receptor nuclear translocator 2 OS=Mus musculus GN=Arnt2 - [ARNT2_MOUSE]	4.42	2	2	2	6	0.93	0.94	0.89	0.86	0.99	1.16	1.18	1.19	1.21	1.04	1.11	0.88	0.99	76.77	6.80
D3YXK2	Scaffold attachment factor B1 OS=Mus musculus GN=Safb PE=1 SV=2 - [SAFB1_MOUSE]	29.03	1	20	28	113	1.04	1.01	1.00	1.04	0.99	1.17	1.08	1.19	1.07	1.10	1.05	1.04	0.99	105.04	5.35
Q3TDN0	Protein dispatched homolog 1 OS=Mus musculus GN=Disp1 PE=1 SV=2 - [DISP1_MOUSE]	1.51	1	1	1	1	0.93	0.97	1.04	0.85	0.92	1.42	1.52	1.19	1.27	1.14	1.23	0.93	1.00	170.02	6.84
Q80YX1-	Isoform 2 of Tenascin OS=Mus musculus GN=Tnc - [TENA_MOUSE]	6.98	6	12	12	19	1.17	1.13	0.96	1.01	0.89	1.22	1.04	1.19	1.09	1.17	0.98	1.30	1.09	221.74	4.91
Q06890	Clusterin OS=Mus musculus GN=Clu PE=1 SV=1 - [CLUS_MOUSE]	27.90	6	10	10	58	1.00	0.92	0.86	0.95	0.93	1.15	1.16	1.19	1.17	1.09	1.08	1.03	1.03	51.62	5.67
Q8CCJ4	APC membrane recruitment protein 2 OS=Mus musculus GN=Amer2 PE=1 SV=2 - [AMER2_MOUSE]	34.52	1	6	14	47	0.98	1.12	1.24	0.90	0.92	1.13	1.26	1.19	1.14	1.14	1.15	1.06	1.04	69.89	6.64
Q8VI36-2	Isoform Alpha of Paxillin OS=Mus musculus GN=Pxn - [PAXI_MOUSE]	34.47	3	12	12	34	1.03	0.97	1.04	0.97	0.94	1.11	1.05	1.19	1.14	1.05	1.04	0.95	0.92	60.77	6.27
Q542V3	Serine/arginine-rich-splicing factor 4 OS=Mus musculus GN=Srsf4 PE=2 SV=1 - [Q542V3_MOUSE]	24.85	3	11	14	73	1.05	0.99	0.95	1.04	0.90	1.15	1.06	1.19	1.15	1.11	1.10	1.06	1.01	56.22	11.37
B2RSU6	Cingulin-like 1 OS=Mus musculus GN=Cgnl1 PE=2 SV=1 - [B2RSU6_MOUSE]	15.03	7	16	16	37	1.04	0.93	0.93	0.98	0.97	1.14	1.13	1.19	1.19	1.16	1.11	1.01	1.02	148.08	5.77
Q8BGD8	Cytochrome c oxidase assembly factor 6 homolog OS=Mus musculus GN=Coa6 PE=1 SV=1 - [COA6_MOUSE]	51.90	1	4	4	27	1.00	1.22	1.10	0.91	0.91	1.04	0.95	1.19	1.18	1.06	1.05	1.08	1.03	9.29	8.16

Q60987	Forkhead box protein G1 OS=Mus musculus GN=Foxg1 PE=2 SV=1 - [FOXG1_MOUSE]	5.41	1	1	1	1	0.87	0.94	1.07	0.95	1.09	1.09	1.25	1.19	1.36	1.14	1.30	1.01	1.16	51.59	8.87
Q8BUK6	Protein Hook homolog 3 OS=Mus musculus GN=Hook3 PE=1 SV=2 - [HOOK3_MOUSE]	41.78	2	24	24	93	0.99	0.90	0.90	0.90	0.86	1.17	1.15	1.19	1.20	1.09	1.10	1.04	1.00	83.17	5.19
P70349	Histidine triad nucleotide-binding protein 1 OS=Mus musculus GN=Hint1 PE=1 SV=3 - [HINT1_MOUSE]	77.78	2	7	7	102	0.98	0.82	0.85	0.92	0.95	1.16	1.15	1.19	1.18	1.12	1.16	1.05	1.06	13.77	6.87
Q8K285	FCH domain only protein 1 OS=Mus musculus GN=Fcho1 PE=1 SV=2 - [FCHO1_MOUSE]	1.60	1	1	1	2	1.06	1.14	1.07	0.97	0.91	1.22	1.15	1.19	1.12	1.18	1.11	1.26	1.19	95.06	7.11
Q60668-3	Isoform 3 of Heterogeneous nuclear ribonucleoprotein D0 OS=Mus musculus GN=Hnrnpd - [HNRPD_MOUSE]	38.89	11	11	13	121	1.03	0.77	0.75	0.92	0.90	1.23	1.24	1.19	1.17	1.14	1.15	1.04	1.05	32.73	8.16
J3QNW0	Cytosine-specific methyltransferase OS=Mus musculus GN=Dnmt1 PE=3 SV=1 - [J3QNW0_MOUSE]	0.67	3	1	1	2	1.00	0.84	0.83	0.93	0.93	1.09	1.08	1.19	1.18	0.96	0.96	1.04	1.04	169.85	7.69
Q8BR07-3	Isoform 3 of Protein bicaudal D homolog 1 OS=Mus musculus GN=Bicd1 - [BICD1_MOUSE]	5.21	6	3	3	7	0.93	1.24	1.37	1.02	1.04	1.06	0.86	1.19	1.19	1.11	0.94	1.34	1.05	94.77	5.63
A1BN54	Alpha actinin 1a OS=Mus musculus GN=Actn1 PE=2 SV=1 - [A1BN54_MOUSE]	65.05	1	2	54	316	1.00	1.14	1.16	1.05	1.04	1.06	1.07	1.19	1.27	0.96	1.00	1.11	1.16	102.66	5.48
Q922Y1	UBX domain-containing protein 1 OS=Mus musculus GN=Ubxn1 PE=1 SV=1 - [UBXN1_MOUSE]	47.14	1	9	9	37	0.93	0.99	0.95	0.88	0.92	1.14	1.24	1.19	1.29	1.11	1.13	1.04	1.10	33.55	5.26
O88207	Collagen alpha-1(V) chain OS=Mus musculus GN=Col5a1 PE=2 SV=2 - [CO5A1_MOUSE]	2.83	1	2	2	3	2.05	1.63	0.79	1.03	0.85	1.18	0.57	1.19	0.75	1.23	0.63	1.36	0.67	183.56	4.98
P58871	182 kDa tankyrase-1-binding protein OS=Mus musculus GN=Tnks1bp1 PE=1 SV=2 - [TB182_MOUSE]	55.64	1	64	64	279	1.01	1.03	1.02	0.96	0.95	1.17	1.18	1.19	1.20	1.10	1.08	0.99	0.98	181.71	4.88
Q9JLM8	Serine/threonine-protein kinase DCLK1 OS=Mus musculus GN=Dclk1 PE=1 SV=1 - [DCLK1_MOUSE]	41.27	1	3	25	119	1.14	1.24	1.34	1.06	1.04	0.95	0.97	1.19	1.11	1.06	1.10	1.07	0.99	84.10	8.87

Q6ZWZ7	60S ribosomal protein L17 OS=Mus musculus GN=Rpl17 PE=2 SV=1 - [Q6ZWZ7_MOUSE]	30.98	3	4	5	34	1.12	0.82	0.71	0.96	0.91	1.18	1.04	1.19	1.10	1.03	0.94	1.09	0.94	21.38	10.18
Q9QYK7	RING finger protein 11 OS=Mus musculus GN=Rnf11 PE=1 SV=1 - [RNF11_MOUSE]	13.64	1	2	2	8	1.05	1.10	1.05	0.97	0.90	1.11	1.02	1.19	1.11	1.08	1.03	1.18	1.11	17.45	4.78
Q8VHY0	Chondroitin sulfate proteoglycan 4 OS=Mus musculus GN=Cspg4 PE=1 SV=3 - [CSPG4_MOUSE]	8.98	3	13	13	21	0.96	1.09	1.17	1.00	1.02	1.03	1.01	1.19	1.16	1.08	1.15	1.10	1.11	252.15	5.44
Q05816	Fatty acid-binding protein, epidermal OS=Mus musculus GN=Fabp5 PE=1 SV=3 - [FABP5_MOUSE]	72.59	1	8	8	165	0.97	0.89	0.94	0.92	0.94	1.19	1.21	1.19	1.22	1.09	1.13	1.08	1.08	15.13	6.54
Q8R048	C-Myc-binding protein OS=Mus musculus GN=Mycbp PE=4 SV=1 - [Q8R048_MOUSE]	62.14	3	5	5	14	1.04	0.79	0.82	0.84	0.88	1.18	1.05	1.19	1.15	0.98	0.95	0.92	0.89	11.96	5.91
P70296	Phosphatidylethanolamine-binding protein 1 OS=Mus musculus GN=Pebp1 PE=1 SV=3 - [PEBP1_MOUSE]	75.94	6	10	10	563	0.97	0.87	0.93	0.88	0.90	1.12	1.14	1.19	1.19	1.08	1.12	1.04	1.10	20.82	5.40
Q9Z1M8	Protein Red OS=Mus musculus GN=Ik PE=2 SV=2 - [RED_MOUSE]	30.16	1	14	14	20	0.99	0.87	1.01	1.00	0.94	1.15	1.09	1.19	1.13	1.08	1.08	1.12	1.14	65.58	6.64
Q61733	28S ribosomal protein S31, mitochondrial OS=Mus musculus GN=Mrps31 PE=2 SV=1 - [RT31_MOUSE]	29.43	1	8	9	14	0.92	0.97	1.00	0.88	0.91	1.16	1.27	1.19	1.42	1.03	1.15	0.89	1.15	43.85	8.51
Q8BL65-4	Isoform 4 of Actin-binding LIM protein 2 OS=Mus musculus GN=Ablim2 - [ABLM2_MOUSE]	54.62	8	2	26	206	0.94	1.05	0.95	0.94	0.98	1.23	1.27	1.19	1.22	1.08	1.08	0.96	1.12	68.61	8.07
Q80Y56	Rabenosyn-5 OS=Mus musculus GN=Zfyve20 PE=2 SV=1 - [RBNS5_MOUSE]	10.73	2	6	6	13	1.08	0.95	0.90	0.98	0.91	1.21	1.14	1.19	1.10	1.18	1.11	1.18	1.10	88.44	5.30
E9Q8P8	Protein FAM131B OS=Mus musculus GN=Fam131b PE=2 SV=1 - [E9Q8P8_MOUSE]	39.17	3	14	14	75	0.95	1.01	1.04	0.98	1.03	1.18	1.21	1.19	1.22	1.06	1.10	1.00	1.05	38.93	4.48
Q8CHI8-5	Isoform 5 of E1A-binding protein p400 OS=Mus musculus GN=Ep400 - [EP400_MOUSE]	0.62	5	1	2	3	0.86	1.12	1.30	0.88	1.03	0.98	1.14	1.19	1.38	1.17	1.37	1.27	1.48	318.04	9.11

Q9D074	E3 ubiquitin-protein ligase MGRN1 OS=Mus musculus GN=Mgrn1 PE=1 SV=2 - [MGRN1_MOUSE]	1.50	5	1	1	2	1.04	1.24	1.19	1.03	0.99	1.22	1.17	1.19	1.14	1.03	1.00	0.97	0.94	58.44	4.88
Q8VEJ4	Notchless protein homolog 1 OS=Mus musculus GN=Nle1 PE=2 SV=4 - [NLE1_MOUSE]	11.34	1	3	3	6	1.07	0.94	0.88	0.94	0.88	1.07	1.02	1.19	1.10	0.96	0.90	0.92	0.85	53.10	7.17
P16045	Galectin-1 OS=Mus musculus GN=Lgals1 PE=1 SV=3 - [LEG1_MOUSE]	57.04	1	7	7	64	1.00	0.81	0.83	0.93	0.90	1.22	1.17	1.19	1.20	1.06	1.05	0.99	0.93	14.86	5.49
P70365-4	Isoform 4 of Nuclear receptor coactivator 1 OS=Mus musculus GN=Ncoa1 - [NCOA1_MOUSE]	7.07	4	7	7	12	0.90	0.89	1.01	0.93	1.03	1.23	1.21	1.19	1.19	1.11	1.23	1.03	1.08	144.67	6.24
Q9WV92-	Isoform 8 of Band 4.1-like protein 3 OS=Mus musculus GN=Epb41l3 - [E41L3_MOUSE]	48.29	1	1	41	288	1.17	1.34	1.15	1.08	0.92	1.09	0.93	1.19	1.02	1.13	0.97	0.94	0.81	116.71	5.22
Q62048	Astrocytic phosphoprotein PEA-15 OS=Mus musculus GN=Pea15 PE=1 SV=1 - [PEA15_MOUSE]	63.08	2	10	10	269	1.23	0.93	0.76	0.98	0.79	1.24	0.98	1.19	0.94	1.12	0.90	1.11	0.95	15.04	5.02
Q78TU8	Family with sequence similarity 107, member A OS=Mus musculus GN=Fam107a PE=2 SV=1 - [Q78TU8_MOUSE]	46.53	4	7	8	35	1.14	0.92	0.86	0.93	0.83	1.25	1.06	1.19	1.04	1.04	0.91	1.05	0.86	17.50	9.74
B7ZCI2	Nebulette OS=Mus musculus GN=Nebl PE=2 SV=1 - [B7ZCI2_MOUSE]	12.82	8	1	11	101	0.82	0.95	1.15	0.96	1.17	1.00	1.20	1.19	1.44	1.09	1.32	0.94	1.14	115.94	8.51
D3YY50	Copper homeostasis protein cutC homolog (Fragment) OS=Mus musculus GN=Cutc PE=2 SV=1 - [D3YY50_MOUSE]	4.72	3	1	1	2	0.99	1.06	1.07	1.05	1.07	1.15	1.16	1.19	1.20	1.12	1.13	1.13	1.15	27.14	6.68
Q9WU63	Heme-binding protein 2 OS=Mus musculus GN=Hebp2 PE=1 SV=1 - [HEBP2_MOUSE]	7.32	1	1	1	2	1.03	0.94	0.91	0.86	0.83	1.20	1.16	1.19	1.15	0.93	0.90	1.06	1.02	23.05	4.48
Q8CHY6	Transcriptional repressor p66 alpha OS=Mus musculus GN=Gatad2a PE=1 SV=2 - [P66A_MOUSE]	6.04	2	2	2	4	0.88	0.80	0.90	0.87	0.98	1.03	1.16	1.19	1.34	0.99	1.12	0.89	1.00	67.29	9.89
Q8R1H0	Homeodomain-only protein OS=Mus musculus GN=Hopx PE=1 SV=1 - [HOP_MOUSE]	49.32	1	4	4	9	0.98	1.20	1.20	1.00	1.02	1.20	1.19	1.19	1.05	1.11	1.19	1.13	1.10	8.28	4.88

E0CXA0	Hepatoma-derived growth factor (Fragment) OS=Mus musculus GN=Hdgf PE=2 SV=1 - [E0CXA0_MOUSE]	82.67	2	1	15	122	0.94	1.11	1.14	1.08	1.11	1.31	1.21	1.19	1.20	1.26	1.13	0.93	0.93	22.10	4.67
G3UZX2	Ubiquitin-conjugating enzyme E2 G2 (Fragment) OS=Mus musculus GN=Ube2g2 PE=2 SV=1 - [G3UZX2_MOUSE]	12.68	2	1	1	1	0.90	0.72	0.79	0.99	1.09	1.20	1.32	1.19	1.32	1.15	1.27	1.10	1.22	7.79	4.50
A2AA86	SUZ domain-containing protein 1 OS=Mus musculus GN=Szrd1 PE=2 SV=1 - [A2AA86_MOUSE]	66.67	4	7	7	23	1.02	0.86	0.85	0.97	0.92	1.17	1.10	1.19	1.20	1.13	1.06	0.98	1.02	14.62	11.46
Q8VE11	Myotubularin-related protein 6 OS=Mus musculus GN=Mtmr6 PE=1 SV=1 - [MTMR6_MOUSE]	2.27	1	1	1	2	1.06	1.18	1.12	1.02	0.97	1.01	0.96	1.19	1.12	1.09	1.03	1.05	0.99	70.89	7.83
A3KGF9	Uncharacterized protein C15orf52 homolog OS=Mus musculus PE=1 SV=1 - [CO052_MOUSE]	3.67	2	1	2	2	1.09	0.99	0.91	0.97	0.88	1.08	0.98	1.19	1.09	1.06	0.97	1.10	1.00	58.65	9.55
Q8CJ69	BMP-binding endothelial regulator protein OS=Mus musculus GN=Bmper PE=1 SV=1 - [BMPER_MOUSE]	1.90	1	1	1	2	0.90	0.82	0.91	1.05	1.16	0.97	1.07	1.19	1.32	1.09	1.21	0.97	1.08	76.10	7.80
Q61624	Zinc finger protein 148 OS=Mus musculus GN=Znf148 PE=1 SV=2 - [ZN148_MOUSE]	25.82	1	13	13	32	1.04	0.96	0.96	0.96	0.96	1.21	1.19	1.19	1.15	1.10	1.04	1.02	1.03	88.70	6.48
Q69ZZ6-2	Isoform 2 of Transmembrane and coiled-coil domains protein 1 OS=Mus musculus GN=Tmcc1 - [TMCC1_MOUSE]	15.19	6	5	6	14	1.08	0.97	0.88	1.16	1.06	1.12	1.11	1.19	1.10	1.15	1.06	1.06	1.11	52.89	7.06
B7ZMZ7	Dip2c protein OS=Mus musculus GN=Dip2c PE=2 SV=1 - [B7ZMZ7_MOUSE]	13.69	1	1	15	33	0.91	1.17	1.28	0.93	1.02	1.10	1.20	1.19	1.31	1.13	1.25	1.01	1.11	167.14	7.44
Q05DI3	Stathmin OS=Mus musculus GN=Stmn4 PE=2 SV=1 - [Q05DI3_MOUSE]	21.02	4	4	4	6	1.00	1.02	0.89	0.95	0.94	1.26	1.24	1.19	1.19	1.18	1.24	0.99	0.98	20.42	5.73
F8VQ93	PH-interacting protein OS=Mus musculus GN=Phip PE=4 SV=1 - [F8VQ93_MOUSE]	0.71	2	1	1	2	1.04	1.27	1.21	1.35	1.29	1.08	1.03	1.19	1.14	1.14	1.09	1.38	1.33	206.60	8.85

B2RUR8	OTU domain-containing protein 7B OS=Mus musculus GN=Otud7b PE=1 SV=1 - [OTU7B_MOUSE]	11.19	3	4	6	11	0.80	1.08	1.35	0.97	1.10	1.06	1.34	1.19	1.53	0.96	1.40	1.15	1.13	91.93	6.86
Q9R207	Nibrin OS=Mus musculus GN=Nbn PE=1 SV=1 - [NBN_MOUSE]	1.07	1	1	1	1	0.96	0.93	0.97	1.28	1.33	0.88	0.91	1.19	1.24	0.85	0.88	1.07	1.12	83.74	6.68
Q3TLH4	Protein PRRC2C OS=Mus musculus GN=Prrc2c PE=1 SV=3 - [PRC2C_MOUSE]	22.21	2	48	49	149	1.08	0.93	0.89	1.01	0.93	1.18	1.10	1.19	1.10	1.14	1.04	1.02	0.96	310.70	9.10
B1AVN9	Protein Phactr2 OS=Mus musculus GN=Phactr2 PE=2 SV=1 - [B1AVN9_MOUSE]	15.54	5	6	7	27	1.24	1.23	0.96	0.94	0.80	1.24	1.01	1.19	0.94	1.26	1.01	1.20	1.04	61.73	5.69
Q6P1H6-4	Isoform 4 of Ankyrin repeat and LEM domain-containing protein 2 OS=Mus musculus GN=Ankle2 - [ANKL2_MOUSE]	9.22	4	3	3	6	0.93	1.14	1.16	1.08	1.07	1.10	1.10	1.19	1.15	1.18	1.08	1.33	1.15	46.88	7.65
Q3V3R4	Integrin alpha-1 OS=Mus musculus GN=Itga1 PE=2 SV=2 - [ITA1_MOUSE]	1.78	1	2	2	3	1.14	1.13	0.99	1.24	1.09	1.25	1.08	1.19	1.04	1.23	1.08	1.21	1.06	130.73	6.20
P55194	SH3 domain-binding protein 1 OS=Mus musculus GN=Sh3bp1 PE=1 SV=2 - [3BP1_MOUSE]	7.99	4	3	3	5	0.79	0.76	1.03	0.89	1.21	0.90	1.00	1.19	1.62	1.01	1.37	0.88	1.20	65.24	5.68
Q99LU0	Charged multivesicular body protein 1b-1 OS=Mus musculus GN=Chmp1b1 PE=1 SV=1 - [CH1B1_MOUSE]	12.06	2	3	3	6	1.05	0.95	0.92	0.97	0.93	1.08	1.07	1.19	1.16	1.05	1.02	1.16	1.11	22.11	8.10
P61759	Prefoldin subunit 3 OS=Mus musculus GN=Vbp1 PE=2 SV=2 - [PFD3_MOUSE]	54.59	8	11	11	53	1.05	0.97	0.93	0.91	0.87	1.22	1.14	1.19	1.20	1.09	1.09	1.02	1.04	22.42	6.28
D3YYI8	Histone deacetylase OS=Mus musculus GN=Gm10093 PE=3 SV=1 - [D3YYI8_MOUSE]	14.52	2	1	4	11	1.12	0.92	0.82	0.99	0.88	1.36	1.20	1.19	1.06	1.33	1.19	1.10	0.98	55.01	5.43
P28738	Kinesin heavy chain isoform 5C OS=Mus musculus GN=Kif5c PE=1 SV=3 - [KIF5C_MOUSE]	45.40	3	29	40	109	0.99	0.90	0.88	0.99	0.99	1.14	1.21	1.19	1.25	1.07	1.14	0.94	1.00	109.21	6.19
P0CW02	Lymphocyte antigen 6C1 OS=Mus musculus GN=Ly6c1 PE=2 SV=1 - [LY6C1_MOUSE]	13.74	1	1	1	2	0.81	1.30	1.59	1.03	1.26	0.96	1.17	1.19	1.46	1.19	1.46	1.41	1.74	14.18	6.01
Q3UJB0	Protein Sf3b2 OS=Mus musculus GN=Sf3b2 PE=2 SV=1 - [Q3UJB0_MOUSE]	35.54	1	22	22	63	0.99	0.99	0.99	1.00	1.00	1.16	1.17	1.19	1.21	1.18	1.20	1.13	1.12	98.14	5.62

P16332	Methylmalonyl-CoA mutase, mitochondrial OS=Mus musculus GN=Mut PE=2 SV=2 - [MUTA_MOUSE]	21.39	1	12	12	26	0.95	1.06	1.05	1.22	1.29	1.07	1.00	1.20	1.04	1.24	1.26	1.28	1.30	82.79	6.89
A2AWS5	Histone deacetylase 5 OS=Mus musculus GN=Hdac5 PE=2 SV=1 - [A2AWS5_MOUSE]	6.89	7	5	5	8	1.02	1.13	1.01	0.98	0.89	1.21	1.07	1.20	1.06	1.17	0.97	1.31	1.06	111.39	5.72
Q9QYR6	Microtubule-associated protein 1A OS=Mus musculus GN=Map1a PE=1 SV=2 - [MAP1A_MOUSE]	58.29	3	132	133	1754	1.01	0.92	0.94	0.95	0.95	1.21	1.20	1.20	1.17	1.08	1.09	1.01	1.02	299.96	5.00
Q6P2L6-4	Isoform 4 of Histone-lysine N-methyltransferase NSD3 OS=Mus musculus GN=Whsc1l1 - [NSD3_MOUSE]	2.42	5	1	1	2	1.27	1.12	0.88	1.06	0.84	1.16	0.91	1.20	0.94	1.34	1.05	1.18	0.93	69.96	9.17
Q9D1Q4	Dolichol-phosphate mannosyltransferase subunit 3 OS=Mus musculus GN=Dpm3 PE=3 SV=1 - [DPM3_MOUSE]	10.87	1	1	1	1	0.80	1.43	1.77	0.89	1.10	1.02	1.25	1.20	1.48	1.43	1.78	0.59	0.73	10.13	7.08
Q80U72	Protein scribble homolog OS=Mus musculus GN=Scrib PE=1 SV=2 - [SCRIB_MOUSE]	4.53	7	5	5	8	1.11	1.28	1.15	1.03	0.91	1.12	1.01	1.20	1.03	1.19	1.06	1.10	0.99	173.95	5.12
Q9CR41	Huntingtin-interacting protein K OS=Mus musculus GN=Hypk PE=2 SV=2 - [HYPK_MOUSE]	24.81	2	2	2	17	1.08	0.84	0.74	0.91	0.85	1.14	1.08	1.20	1.10	1.12	1.04	0.95	0.92	14.67	4.93
Q8R1S4-2	Isoform 2 of Metastasis suppressor protein 1 OS=Mus musculus GN=Mtss1 - [MTSS1_MOUSE]	3.46	3	1	1	4	1.03	1.06	1.03	1.07	1.04	1.11	1.07	1.20	1.16	1.22	1.19	1.36	1.32	78.78	7.15
E0CY16	Cell adhesion molecule 1 OS=Mus musculus GN=Cadm1 PE=2 SV=1 - [E0CY16_MOUSE]	45.90	2	1	13	170	1.15	0.92	0.80	1.05	0.91	1.42	1.23	1.20	1.04	1.11	0.96	1.19	1.04	42.86	5.72
Q9QXD8	LIM domain-containing protein 1 OS=Mus musculus GN=Limd1 PE=1 SV=2 - [LIMD1_MOUSE]	15.87	1	6	6	10	0.99	0.88	0.82	0.87	0.94	1.16	1.19	1.20	1.29	1.15	1.12	0.96	1.03	71.38	6.32
Q8CDA1	Phosphatidylinositide phosphatase SAC2 OS=Mus musculus GN=Inpp5f PE=1 SV=1 - [SAC2_MOUSE]	7.77	4	8	8	11	1.08	1.21	1.14	1.07	1.02	1.15	1.08	1.20	1.07	1.24	1.16	1.24	1.20	127.53	7.11
Q91WV0	Protein Dr1 OS=Mus musculus GN=Dr1 PE=2 SV=1 - [NC2B_MOUSE]	15.91	1	3	3	6	1.00	1.04	1.03	0.93	0.92	1.02	1.01	1.20	1.18	1.04	1.03	1.06	1.06	19.42	4.75

Q9WV69-	Isoform 2 of Dematin OS=Mus musculus GN=Dmtn - [DEMA_MOUSE]	66.58	2	1	19	282	1.34	1.77	1.68	1.27	0.95	1.24	0.95	1.20	0.89	1.42	0.92	1.24	0.93	43.02	8.28
Q62419	Endophilin-A2 OS=Mus musculus GN=Sh3gl1 PE=1 SV=1 - [SH3G1_MOUSE]	65.49	1	17	24	113	0.96	0.92	0.99	0.89	0.97	1.18	1.23	1.20	1.26	1.03	1.11	0.98	1.00	41.49	5.72
Q8VEA4	Mitochondrial intermembrane space import and assembly protein 40 OS=Mus musculus GN=Chchd4 PE=1 SV=1 - [MIA40_MOUSE]	42.45	1	4	4	22	1.04	0.94	0.89	0.91	0.93	1.14	1.13	1.20	1.19	1.14	1.06	1.03	1.02	15.51	4.32
E9QLD2	Myelin transcription factor 1-like protein OS=Mus musculus GN=Myt1l PE=2 SV=1 - [E9QLD2_MOUSE]	12.15	11	11	12	24	1.01	0.93	0.85	0.98	0.94	1.23	1.19	1.20	1.16	1.08	1.07	1.05	0.97	132.68	4.93
P60761	Neurogranin OS=Mus musculus GN=Nrgn PE=1 SV=1 - [NEUG_MOUSE]	87.18	1	6	6	110	1.02	0.84	0.79	0.88	0.86	1.20	1.17	1.20	1.17	1.03	0.98	0.87	0.83	7.49	7.05
Q6PDM2	Serine/arginine-rich splicing factor 1 OS=Mus musculus GN=Srsf1 PE=1 SV=3 - [SRSF1_MOUSE]	53.23	6	16	16	138	1.01	0.98	0.95	0.95	0.93	1.16	1.14	1.20	1.18	1.07	1.04	1.01	0.99	27.73	10.36
Q80YN3	Breast carcinoma-amplified sequence 1 homolog OS=Mus musculus GN=Bcas1 PE=1 SV=3 - [BCAS1_MOUSE]	46.92	4	23	23	133	0.96	0.95	1.01	0.91	0.92	1.19	1.23	1.20	1.21	1.09	1.12	1.03	1.08	67.34	6.21
Q8C4Y1	Max protein OS=Mus musculus GN=Max PE=2 SV=1 - [Q8C4Y1_MOUSE]	33.11	3	4	4	10	1.01	1.08	0.99	0.90	0.92	1.12	1.05	1.20	1.10	1.17	1.04	1.06	1.03	17.16	6.55
Q5SQY2	Biorientation of chromosomes in cell division protein 1 OS=Mus musculus GN=Bod1 PE=2 SV=1 - [BOD1_MOUSE]	54.34	1	5	7	17	1.05	1.05	1.02	0.93	0.96	1.10	1.11	1.20	1.09	1.07	1.01	1.05	0.98	18.35	5.90
Q3TQ29	Pumilio homolog 2 OS=Mus musculus GN=Pum2 PE=2 SV=1 - [Q3TQ29_MOUSE]	4.08	10	3	4	8	1.03	1.22	1.19	1.07	1.02	1.18	1.15	1.20	1.17	1.12	1.12	1.14	1.03	105.57	6.86
Q9CR51	V-type proton ATPase subunit G 1 OS=Mus musculus GN=Atp6v1g1 PE=2 SV=3 - [VATG1_MOUSE]	53.39	1	4	4	33	1.13	1.07	0.98	0.90	0.80	1.20	1.04	1.20	1.06	1.06	0.96	1.08	0.93	13.72	7.97
Q61937	Nucleophosmin OS=Mus musculus GN=Npm1 PE=1 SV=1 - [NPM_MOUSE]	68.84	5	15	15	217	1.04	1.03	0.99	0.96	0.92	1.20	1.14	1.20	1.13	1.14	1.06	1.09	1.02	32.54	4.77

O70274	Protein tyrosine phosphatase type IVA 2 OS=Mus musculus GN=Ptp4a2 PE=1 SV=1 - [TP4A2_MOUSE]	17.37	2	3	3	4	1.18	1.55	1.14	1.21	0.92	0.99	0.72	1.20	0.91	1.08	0.95	1.11	0.91	19.11	8.37
E9PYN2	Zinc finger protein 346 OS=Mus musculus GN=Zfp346 PE=2 SV=1 - [E9PYN2_MOUSE]	10.31	9	2	2	4	1.05	0.97	0.92	1.05	1.00	1.17	1.11	1.20	1.14	1.21	1.16	1.26	1.20	29.00	8.44
Q9D4H4-2	Isoform 2 of Angiomotin-like protein 1 OS=Mus musculus GN=Amotl1 - [AMOL1_MOUSE]	4.42	2	2	4	12	1.15	0.85	0.73	0.94	0.83	1.19	1.00	1.20	1.02	1.21	1.03	1.08	0.93	98.36	7.37
E9QK34	Neuroligin-1 OS=Mus musculus GN=Nlgn1 PE=2 SV=1 - [E9QK34_MOUSE]	10.32	3	4	7	17	1.06	1.04	0.98	1.12	1.08	1.05	1.01	1.20	0.99	1.07	0.96	1.10	1.03	90.95	6.10
Q6PAM1	Alpha-taxilin OS=Mus musculus GN=Txlna PE=2 SV=1 - [TXLNA_MOUSE]	22.02	6	12	12	26	1.06	0.95	0.85	1.01	0.96	1.18	1.11	1.20	1.16	1.06	1.02	1.04	0.93	62.33	6.74
O70439	Syntaxin-7 OS=Mus musculus GN=Stx7 PE=1 SV=3 - [STX7_MOUSE]	52.87	2	8	8	25	1.07	1.20	1.09	1.00	0.94	1.15	1.09	1.20	1.17	1.19	1.13	1.15	1.06	29.80	5.78
O35449	Proline-rich transmembrane protein 1 OS=Mus musculus GN=Prrt1 PE=1 SV=1 - [PRRT1_MOUSE]	22.88	1	4	4	49	1.10	1.18	1.07	0.97	0.91	1.20	1.12	1.20	1.07	1.13	1.05	1.13	1.02	31.37	7.65
Q8CC35	Synaptopodin OS=Mus musculus GN=Synpo PE=1 SV=2 - [SYNPO_MOUSE]	49.30	4	35	35	400	0.96	0.93	0.96	0.93	0.97	1.15	1.19	1.20	1.24	1.04	1.08	0.97	1.01	99.49	9.42
Q1HKZ5	Mitogen-activated protein kinase kinase kinase 13 OS=Mus musculus GN=Map3k13 PE=2 SV=1 - [M3K13_MOUSE]	1.15	1	1	1	1	1.06	1.32	1.24	0.77	0.73	0.59	0.55	1.20	1.13	0.87	0.82	0.91	0.86	106.92	6.74
P47877	Insulin-like growth factor-binding protein 2 OS=Mus musculus GN=Igfbp2 PE=2 SV=2 - [IBP2_MOUSE]	8.20	2	2	2	6	1.06	1.04	0.97	0.98	0.87	1.19	1.01	1.20	1.07	1.26	1.11	1.06	1.00	32.83	7.64
Q3UDK1-	Isoform 2 of TRAF-type zinc finger domain-containing protein 1 OS=Mus musculus GN=Trafd1 - [TRAD1_MOUSE]	13.19	3	5	5	9	1.11	1.18	1.01	1.06	0.95	1.19	1.05	1.20	1.10	1.11	0.98	1.08	1.07	63.82	5.41
Q62093	Serine/arginine-rich splicing factor 2 OS=Mus musculus GN=Srsf2 PE=1 SV=4 - [SRSF2_MOUSE]	15.38	1	4	4	62	1.09	1.08	0.98	0.97	0.90	1.08	0.99	1.20	1.10	1.12	1.01	1.04	0.95	25.46	11.85

Q3THJ3	Probable RNA-binding protein EIF1AD OS=Mus musculus GN=Eif1ad PE=2 SV=2 - [EIF1A_MOUSE]	31.18	1	4	4	5	1.18	1.29	1.09	1.05	0.93	1.31	1.22	1.20	1.00	1.12	0.96	1.02	0.87	19.51	4.87
Q9D2P4	Ubiquitin-related modifier 1 homolog OS=Mus musculus GN=Urm1 PE=1 SV=1 - [URM1_MOUSE]	15.84	1	2	2	2	1.02	1.19	1.17	1.12	1.10	1.22	1.19	1.20	1.17	1.15	1.13	1.23	1.21	11.32	4.74
Q80XU3	Nuclear ubiquitous casein and cyclin-dependent kinase substrate 1 OS=Mus musculus GN=Nucks1 PE=1 SV=1 - [NUCKS_MOUSE]	46.15	1	7	7	115	1.12	1.04	0.90	1.07	0.90	1.24	1.05	1.20	1.08	1.14	1.03	1.07	0.97	26.30	5.14
Q91W59-2	Isoform 2 of RNA-binding motif, single-stranded-interacting protein 1 OS=Mus musculus GN=Rbms1 - [RBMS1_MOUSE]	10.81	11	3	3	6	0.95	0.78	0.81	0.93	0.98	1.24	1.28	1.20	1.27	1.00	1.06	0.81	0.86	39.98	8.12
Q5DTX6	Junctional protein associated with coronary artery disease OS=Mus musculus GN=Jcad PE=1 SV=2 - [JCAD_MOUSE]	24.92	1	22	22	60	0.92	1.01	1.00	0.95	1.04	1.18	1.28	1.20	1.27	1.12	1.17	1.09	1.14	144.71	6.52
D3YZR1	Protein TSSC4 (Fragment) OS=Mus musculus GN=Tssc4 PE=2 SV=1 - [D3YZR1_MOUSE]	43.32	5	6	6	9	1.07	0.98	1.04	0.93	0.84	1.12	1.11	1.20	1.09	1.14	1.00	1.16	1.10	23.29	4.64
P50518	V-type proton ATPase subunit E 1 OS=Mus musculus GN=Atp6v1e1 PE=1 SV=2 - [VATE1_MOUSE]	70.35	2	20	20	217	0.97	0.96	1.02	0.96	1.01	1.24	1.28	1.20	1.25	1.13	1.16	1.10	1.14	26.14	8.43
F7BIN0	Nucleolar protein 4 (Fragment) OS=Mus musculus GN=Nol4 PE=4 SV=1 - [F7BIN0_MOUSE]	14.32	6	2	4	5	1.06	1.31	1.23	1.15	1.08	1.47	1.38	1.20	1.12	1.13	1.06	1.00	0.94	45.98	5.50
Q8BZR6	TOM1-like protein 1 OS=Mus musculus GN=Tom1l1 PE=2 SV=1 - [Q8BZR6_MOUSE]	5.79	5	1	2	5	1.44	0.89	0.62	0.97	0.67	1.25	0.86	1.20	0.83	1.47	1.02	1.46	1.01	44.06	5.00
O35625-2	Isoform 2 of Axin-1 OS=Mus musculus GN=Axin1 - [AXIN1_MOUSE]	5.80	5	4	4	7	1.03	1.15	1.11	1.04	1.04	1.13	1.08	1.20	1.21	1.13	1.07	1.26	1.16	92.36	6.93
J3JS23	Zinc finger protein 62 (Fragment) OS=Mus musculus GN=Zfp62 PE=4 SV=1 - [J3JS23_MOUSE]	4.48	157	1	2	4	1.30	1.10	0.84	0.90	0.69	1.13	0.86	1.20	0.92	1.11	0.86	1.18	0.91	102.08	8.85

Q8R550	SH3 domain-containing kinase-binding protein 1 OS=Mus musculus GN=Sh3kbp1 PE=1 SV=1 - [SH3K1_MOUSE]	54.30	11	31	31	151	1.10	0.89	0.79	0.93	0.86	1.25	1.11	1.20	1.07	1.14	1.02	1.09	1.00	78.12	7.55
P47930-2	Isoform 2 of Fos-related antigen 2 OS=Mus musculus GN=Fosl2 - [FOSL2_MOUSE]	4.01	2	1	1	3	0.76	0.51	0.68	1.03	0.88	0.68	0.90	1.20	1.59	0.79	1.04	0.89	0.91	32.67	8.25
Q6PDY0	Coiled-coil domain-containing protein 85B OS=Mus musculus GN=Ccdc85b PE=1 SV=1 - [CC85B_MOUSE]	37.13	1	6	6	13	1.06	0.94	0.83	1.05	0.92	1.08	1.04	1.20	1.05	1.13	1.03	1.13	1.03	22.12	5.08
P49710	Hematopoietic lineage cell-specific protein OS=Mus musculus GN=Hcls1 PE=1 SV=2 - [HCLS1_MOUSE]	21.60	1	8	8	19	1.09	0.80	0.84	0.97	0.97	1.22	1.12	1.20	1.16	1.11	1.05	0.92	0.90	54.21	4.84
Q9CZD5	Translation initiation factor IF-3, mitochondrial OS=Mus musculus GN=Mtif3 PE=1 SV=1 - [IF3M_MOUSE]	15.22	3	4	4	8	0.97	0.97	0.97	0.79	0.80	1.18	1.18	1.20	1.25	1.11	1.15	0.96	0.96	31.72	9.42
Q8BIF0-2	Isoform 2 of CD99 antigen-like protein 2 OS=Mus musculus GN=Cd99l2 - [C99L2_MOUSE]	34.11	4	4	4	10	1.14	0.74	0.63	0.94	0.83	1.17	1.04	1.20	1.05	1.10	0.91	1.07	0.88	22.80	4.65
Q91YI1-2	Isoform 2 of Autophagy-related protein 13 OS=Mus musculus GN=Atg13 - [ATG13_MOUSE]	2.09	2	1	1	1	1.01	1.19	1.17	1.09	1.08	1.02	1.00	1.20	1.19	0.91	0.90	1.21	1.20	52.58	5.16
F2Z4B3	Cyclin-dependent kinase 2-associated protein 1 OS=Mus musculus GN=Cdk2ap1 PE=2 SV=1 - [F2Z4B3_MOUSE]	9.20	3	1	1	1	1.16	1.21	1.04	1.01	0.87	1.17	1.01	1.20	1.03	1.16	1.00	1.30	1.12	9.62	9.19
P70441	Na(+)/H(+) exchange regulatory cofactor NHE-RF1 OS=Mus musculus GN=Slc9a3r1 PE=1 SV=3 - [NHRF1_MOUSE]	64.79	2	22	22	319	1.00	0.77	0.77	0.94	0.92	1.22	1.19	1.20	1.18	1.01	1.00	0.90	0.89	38.58	5.90
B8JK56	Adenylate cyclase type 3 OS=Mus musculus GN=Adcy3 PE=3 SV=1 - [B8JK56_MOUSE]	2.62	2	2	2	3	1.10	1.40	1.27	1.08	0.98	1.01	0.91	1.20	1.09	1.20	1.09	1.17	1.06	128.91	6.29
Q9CR86	Calcium-regulated heat stable protein 1 OS=Mus musculus GN=Carhsp1 PE=1 SV=1 - [CHSP1_MOUSE]	54.73	2	5	5	15	0.93	0.74	0.90	0.83	0.90	1.18	1.51	1.20	1.36	1.13	1.19	1.11	1.25	16.05	8.21

Q9D0B5	Thiosulfate sulfurtransferase/rhodanese-like domain-containing protein 3 OS=Mus musculus GN=Tstd3 PE=2 SV=1 - [TSTD3_MOUSE]	35.67	1	5	5	13	0.98	0.77	0.85	0.79	0.86	1.10	1.19	1.20	1.22	1.04	1.04	0.87	0.91	17.26	8.13
P63037	DnaJ homolog subfamily A member 1 OS=Mus musculus GN=Dnaja1 PE=1 SV=1 - [DNJA1_MOUSE]	50.88	4	15	15	74	0.98	1.01	0.89	0.94	0.93	1.09	1.09	1.20	1.17	1.03	1.07	1.09	1.08	44.84	7.08
P61458	Pterin-4-alpha-carbinolamine dehydratase OS=Mus musculus GN=Pcbd1 PE=1 SV=2 - [PHS_MOUSE]	22.12	1	2	2	3	1.87	1.04	0.55	1.05	0.56	1.30	0.69	1.20	0.64	1.47	0.79	1.35	0.72	11.98	6.80
Q5SYH9	Neurofibromin (Fragment) OS=Mus musculus GN=Nf1 PE=2 SV=1 - [Q5SYH9_MOUSE]	6.78	1	1	4	6	0.98	1.09	1.11	0.88	0.89	0.84	0.85	1.20	1.22	0.94	0.96	1.24	1.26	92.15	8.15
A8Y5N8	Pleckstrin homology domain-containing family F member 2 (Fragment) OS=Mus musculus GN=Plekhf2 PE=2 SV=1 - [A8Y5N8_MOUSE]	10.66	2	1	1	2	0.99	0.82	0.83	1.07	1.08	1.12	1.13	1.20	1.21	1.00	1.01	1.12	1.13	22.09	9.45
P20917	Myelin-associated glycoprotein OS=Mus musculus GN=Mag PE=1 SV=2 - [MAG_MOUSE]	33.39	1	2	15	99	0.98	1.02	0.99	0.97	0.90	1.30	1.39	1.20	1.24	1.15	1.12	1.09	1.06	69.22	5.10
Q8R1Y2	Uncharacterized protein C16orf45 homolog OS=Mus musculus PE=2 SV=1 - [CP045_MOUSE]	29.06	3	7	7	12	1.19	0.82	0.70	1.06	0.88	1.32	1.12	1.20	0.98	1.30	1.09	1.16	0.98	23.45	5.63
Q3UP38	EF-hand calcium-binding domain-containing protein 4B OS=Mus musculus GN=Efcab4b PE=2 SV=1 - [EFC4B_MOUSE]	2.90	1	1	1	2	1.00	1.15	1.15	1.00	1.00	1.18	1.18	1.20	1.20	1.10	1.10	1.13	1.13	35.82	4.67
Q8VD63	Testis-specific Y-encoded-like protein 4 OS=Mus musculus GN=Tspyl4 PE=1 SV=1 - [TSYL4_MOUSE]	17.00	1	5	5	10	0.98	0.95	1.04	0.90	0.93	1.03	0.92	1.20	1.22	1.04	1.07	0.91	0.96	44.78	6.99
D3Z3A9	Reticulocalbin-3 (Fragment) OS=Mus musculus GN=Rcn3 PE=2 SV=1 - [D3Z3A9_MOUSE]	10.95	4	2	2	4	1.16	0.98	0.84	1.02	0.88	1.25	1.08	1.20	1.04	1.41	1.22	1.27	1.10	31.82	4.89
Q9CPW3	39S ribosomal protein L54, mitochondrial OS=Mus musculus GN=Mrpl54 PE=2 SV=1 - [RM54_MOUSE]	43.70	1	3	3	8	1.04	0.71	0.63	0.90	0.98	1.19	1.39	1.20	1.27	1.03	1.06	0.93	0.91	15.41	10.04

Q80WJ7	Protein LYRIC OS=Mus musculus GN=Mtdh PE=1 SV=1 - [LYRIC_MOUSE]	49.91	6	23	23	73	1.07	1.04	0.98	1.06	1.01	1.22	1.20	1.20	1.15	1.18	1.09	1.05	1.02	63.81	9.33
Q8BU04	Putative E3 ubiquitin-protein ligase UBR7 OS=Mus musculus GN=Ubr7 PE=2 SV=1 - [UBR7_MOUSE]	13.18	2	5	5	15	1.00	0.90	0.87	0.99	0.91	1.19	1.19	1.20	1.20	1.06	1.08	0.91	0.84	48.03	4.75
Q60605	Myosin light polypeptide 6 OS=Mus musculus GN=Myl6 PE=1 SV=3 - [MYL6_MOUSE]	76.16	1	1	10	291	1.05	1.02	0.99	0.96	0.89	1.12	1.07	1.20	1.19	1.16	1.16	1.05	0.98	16.92	4.65
Q8BHL8	Proteasome inhibitor PI31 subunit OS=Mus musculus GN=Psmf1 PE=1 SV=1 - [PSMF1_MOUSE]	20.66	1	4	4	9	0.92	1.02	1.07	0.95	1.11	0.86	1.15	1.20	1.34	1.03	1.08	0.88	1.12	29.65	5.25
Q3THE2	Myosin regulatory light chain 12B OS=Mus musculus GN=Myl12b PE=1 SV=2 - [ML12B_MOUSE]	62.21	3	1	8	344	1.07	1.24	1.13	1.00	0.91	1.12	1.06	1.20	1.15	1.21	1.12	1.22	1.12	19.77	4.84
Q9Z2D8	Methyl-CpG-binding domain protein 3 OS=Mus musculus GN=Mbd3 PE=1 SV=1 - [MBD3_MOUSE]	24.91	5	7	8	16	0.97	0.95	0.93	1.00	1.00	1.20	1.14	1.20	1.15	1.05	1.04	0.94	0.97	32.15	5.82
P62204	Calmodulin OS=Mus musculus GN=Calm1 PE=1 SV=2 - [CALM_MOUSE]	83.89	5	11	12	919	0.99	0.82	0.85	0.85	0.87	1.18	1.23	1.20	1.27	1.02	1.07	0.96	1.00	16.83	4.22
D3Z3Z8	DNA-directed RNA polymerase II subunit RPB9 (Fragment) OS=Mus musculus GN=Polr2i PE=2 SV=1 - [D3Z3Z8_MOUSE]	48.19	3	2	2	6	1.09	0.79	0.72	0.94	0.86	1.08	1.08	1.20	1.06	1.18	1.00	1.21	1.09	9.64	5.17
Q9D2P8	Myelin-associated oligodendrocyte basic protein OS=Mus musculus GN=Mobp PE=2 SV=1 - [MOBP_MOUSE]	17.06	1	3	3	4	0.81	0.86	0.91	0.92	1.13	1.36	1.67	1.20	1.48	1.28	1.58	0.99	1.29	19.19	11.14
Q0P678	Zinc finger CCCH domain-containing protein 18 OS=Mus musculus GN=Zc3h18 PE=1 SV=1 - [ZCH18_MOUSE]	16.14	6	10	10	35	1.02	1.19	1.09	0.97	0.93	1.23	1.10	1.20	1.11	1.10	1.13	1.06	0.97	105.63	7.80
M0QWP2	ER membrane protein complex subunit 8 (Fragment) OS=Mus musculus GN=Emc8 PE=4 SV=1 - [M0QWP2_MOUSE]	43.36	3	4	4	6	0.96	0.83	0.93	1.03	1.15	1.12	1.25	1.20	1.25	1.19	1.24	1.00	1.12	16.30	4.87

Q80Z38-3	Isoform 3 of SH3 and multiple ankyrin repeat domains protein 2 OS=Mus musculus GN=Shank2 - [SHAN2_MOUSE]	49.05	1	2	44	259	1.04	0.90	0.91	1.00	0.97	1.24	1.24	1.20	1.11	1.02	1.04	0.90	0.89	135.33	6.04
Q80U04-2	Isoform 2 of E3 ubiquitin-protein ligase Praja-2 OS=Mus musculus GN=Pja2 - [PJA2_MOUSE]	31.94	2	10	11	75	1.02	1.10	1.10	0.96	0.90	1.17	1.15	1.20	1.20	1.13	1.09	1.04	1.04	71.20	4.53
D3YWC9	MTSS1-like protein OS=Mus musculus GN=Mtss1l PE=2 SV=1 - [D3YWC9_MOUSE]	14.70	4	5	5	29	1.06	1.01	0.96	0.98	0.91	1.14	1.04	1.20	1.19	1.24	1.19	1.29	1.15	70.04	7.30
B2RW11	Ankyrin repeat domain 34A OS=Mus musculus GN=Ankrd34a PE=2 SV=1 - [B2RW11_MOUSE]	32.12	1	11	11	33	1.10	1.11	1.03	1.05	0.99	1.17	1.06	1.20	1.09	1.08	1.00	1.15	1.09	52.42	9.45
E9Q6B2	Coiled-coil domain-containing protein 85C OS=Mus musculus GN=Ccdc85c PE=2 SV=1 - [CC85C_MOUSE]	30.71	1	11	11	24	1.05	1.00	0.88	0.98	0.90	1.23	1.16	1.20	1.11	1.16	1.20	1.14	1.11	45.31	6.96
O08989	Ras-related protein M-Ras OS=Mus musculus GN=Mras PE=1 SV=1 - [RASM_MOUSE]	35.58	2	5	5	13	0.98	0.95	0.97	0.99	1.08	0.99	1.03	1.21	1.37	1.00	1.04	0.96	1.00	23.89	8.78
Q9Z2E2-5	Isoform 5 of Methyl-CpG-binding domain protein 1 OS=Mus musculus GN=Mbd1 - [MBD1_MOUSE]	4.04	5	2	2	3	1.00	1.05	1.05	1.02	1.02	1.25	1.24	1.21	1.20	1.13	1.13	1.20	1.20	62.04	8.59
Q5Y5T5	Probable palmitoyltransferase ZDHHC8 OS=Mus musculus GN=Zdhhc8 PE=1 SV=1 - [ZDHC8_MOUSE]	7.35	1	3	4	7	1.09	1.17	1.06	1.10	1.04	1.31	1.09	1.21	1.00	1.21	1.07	1.06	0.96	81.98	9.38
G3X9X5	Zinc finger CCCH type, antiviral 1, isoform CRA_b OS=Mus musculus GN=Zc3hav1 PE=4 SV=1 - [G3X9X5_MOUSE]	2.08	4	1	1	2	1.11	1.30	1.16	1.07	0.96	1.12	1.00	1.21	1.08	1.21	1.09	1.22	1.10	76.36	7.78
O89116	Vesicle transport through interaction with t-SNAREs homolog 1A OS=Mus musculus GN=Vti1a PE=1 SV=1 - [VTI1A_MOUSE]	17.51	1	2	2	15	0.91	1.06	1.09	0.96	1.08	0.99	1.08	1.21	1.19	1.05	1.06	0.97	0.99	24.97	6.40
O54824-2	Isoform 2 of Pro-interleukin-16 OS=Mus musculus GN=Il16 - [IL16_MOUSE]	2.56	2	1	1	2	0.99	0.80	0.81	0.98	0.98	1.17	1.17	1.21	1.21	1.20	1.21	1.01	1.02	66.43	6.29

E9QKG6	Ankyrin repeat domain-containing protein 17 OS=Mus musculus GN=Ankrd17 PE=2 SV=1 - [E9QKG6_MOUSE]	3.49	5	4	5	7	1.03	0.99	0.95	1.00	0.94	0.99	0.90	1.21	1.15	1.10	1.13	1.09	1.06	246.47	6.70
Q9D892	Inosine triphosphate pyrophosphatase OS=Mus musculus GN=Itpa PE=1 SV=2 - [ITPA_MOUSE]	47.98	1	6	6	17	1.04	0.94	0.87	1.07	0.97	1.18	1.14	1.21	1.13	1.18	1.13	1.13	1.11	21.88	5.87
P63213	Guanine nucleotide-binding protein G(I)/G(S)/G(O) subunit gamma-2 OS=Mus musculus GN=Gng2 PE=1 SV=2 - [GBG2_MOUSE]	54.93	1	4	5	95	1.01	0.80	0.84	0.98	1.00	1.30	1.31	1.21	1.17	1.19	1.18	1.08	1.05	7.85	7.99
P47879	Insulin-like growth factor-binding protein 4 OS=Mus musculus GN=Igfbp4 PE=2 SV=2 - [IBP4_MOUSE]	16.14	3	3	3	4	1.02	0.98	0.96	0.95	0.93	0.96	0.93	1.21	1.18	0.78	0.77	0.87	0.85	27.79	7.17
D3Z598	Latent-transforming growth factor beta-binding protein 4 OS=Mus musculus GN=Ltbp4 PE=2 SV=1 - [D3Z598_MOUSE]	8.99	7	8	8	16	0.97	1.16	1.23	0.95	0.98	0.90	1.08	1.21	1.29	1.26	1.36	1.14	1.19	166.66	5.11
Q9D289	Trafficking protein particle complex subunit 6B OS=Mus musculus GN=Trappc6b PE=2 SV=1 - [TPC6B_MOUSE]	8.23	1	1	1	2	1.18	1.26	1.06	1.03	0.87	1.09	0.92	1.21	1.02	1.38	1.17	1.24	1.05	17.92	8.68
Q9EQU5	Protein SET OS=Mus musculus GN=Set PE=1 SV=1 - [SET_MOUSE]	51.90	4	2	12	187	0.86	0.80	0.93	1.01	1.17	1.15	1.32	1.21	1.39	1.25	1.45	1.01	1.17	33.36	4.32
O88737	Protein bassoon OS=Mus musculus GN=Bsn PE=1 SV=4 - [BSN_MOUSE]	60.25	2	153	160	1487	1.00	0.97	0.97	0.96	0.95	1.18	1.17	1.21	1.20	1.08	1.08	1.01	1.00	418.59	7.71
Q9WV52	Pleckstrin-2 OS=Mus musculus GN=Plek2 PE=1 SV=1 - [PLEK2_MOUSE]	4.82	1	1	1	1	1.05	0.87	0.82	0.91	0.87	1.24	1.18	1.21	1.15	1.09	1.04	0.95	0.91	39.99	9.45
Q8K3I9	Glucocorticoid-induced transcript 1 protein OS=Mus musculus GN=Glcci1 PE=1 SV=1 - [GLCI1_MOUSE]	34.64	5	11	11	22	1.06	0.94	0.98	1.03	0.93	1.27	1.15	1.21	1.13	1.12	1.05	1.04	1.03	57.44	9.47
F8WI30	Sorting nexin-7 OS=Mus musculus GN=Snx7 PE=2 SV=1 - [F8WI30_MOUSE]	12.81	4	4	4	6	0.90	1.14	1.33	1.13	1.27	1.00	1.10	1.21	1.13	1.09	1.23	1.07	1.29	50.85	4.82
Q9JHS9	Spliceosome-associated protein CWC15 homolog OS=Mus musculus GN=Cwc15 PE=1 SV=1 - [CWC15_MOUSE]	24.45	1	6	6	12	1.06	0.85	0.77	0.99	0.92	1.21	1.25	1.21	1.22	1.03	0.99	0.96	1.06	26.61	5.71

D6RET2	Mesoderm induction early response protein 3 OS=Mus musculus GN=Mier3 PE=2 SV=1 - [D6RET2_MOUSE]	10.26	4	1	1	3	0.86	0.88	1.01	0.86	1.00	1.13	1.30	1.21	1.39	0.92	1.07	0.78	0.90	12.66	3.98
D3YTV4	Zinc finger protein with KRAB and SCAN domains 3 OS=Mus musculus GN=Zkscan3 PE=2 SV=1 - [D3YTV4_MOUSE]	12.06	4	2	2	3	1.08	1.46	1.35	0.95	0.89	1.26	1.17	1.21	1.12	1.17	1.09	1.21	1.13	28.83	4.63
Q02384	Son of sevenless homolog 2 OS=Mus musculus GN=Sos2 PE=1 SV=2 - [SOS2_MOUSE]	1.13	1	1	1	1	1.05	1.50	1.42	0.96	0.91	1.19	1.13	1.21	1.15	1.21	1.15	1.36	1.29	153.03	6.76
Q9CPW2	Adrenodoxin-like protein, mitochondrial OS=Mus musculus GN=Fdx1l PE=2 SV=1 - [ADXL_MOUSE]	47.13	2	7	7	28	1.08	0.94	0.87	0.95	0.89	1.19	0.99	1.21	1.03	1.00	0.87	1.00	0.90	18.75	6.04
P06797	Cathepsin L1 OS=Mus musculus GN=Ctsl1 PE=1 SV=2 - [CATL1_MOUSE]	41.02	1	7	7	40	1.05	0.94	0.85	0.88	0.87	1.09	1.06	1.21	1.16	1.01	1.02	0.95	0.95	37.52	6.83
E9Q7U0	Protein Kcnmb3 OS=Mus musculus GN=Kcnmb3 PE=4 SV=1 - [E9Q7U0_MOUSE]	12.55	1	1	1	1	1.27	0.57	0.45	0.96	0.76	1.42	1.11	1.21	0.95	1.40	1.10	1.07	0.85	26.47	7.91
Q7M750	Opalin OS=Mus musculus GN=Opalin PE=1 SV=1 - [OPALI_MOUSE]	32.17	1	3	3	23	0.99	1.12	1.20	0.94	0.99	1.14	1.18	1.21	1.27	1.25	1.28	1.14	1.21	15.82	4.88
O70166	Stathmin-3 OS=Mus musculus GN=Stmn3 PE=1 SV=1 - [STMN3_MOUSE]	39.44	1	7	7	18	1.03	0.88	0.90	0.99	0.99	1.28	1.29	1.21	1.23	1.13	1.13	1.16	1.17	20.94	7.49
Q6ZWZ6	40S ribosomal protein S12 OS=Mus musculus GN=Rps12 PE=2 SV=1 - [Q6ZWZ6_MOUSE]	46.21	2	6	6	42	0.95	0.72	0.75	0.84	0.90	1.17	1.26	1.21	1.26	0.99	1.08	1.00	1.05	14.51	7.21
Q80WT5-	Isoform 2 of Aftiphilin OS=Mus musculus GN=Aftph - [AFTIN_MOUSE]	29.87	5	18	18	54	1.03	0.88	0.84	0.91	0.90	1.14	1.12	1.21	1.13	1.11	1.10	1.02	1.00	98.43	4.51
Q60722	Transcription factor 4 OS=Mus musculus GN=Tcf4 PE=1 SV=1 - [ITF2_MOUSE]	6.72	5	2	3	7	0.92	1.09	1.25	0.97	1.00	1.00	1.08	1.21	1.23	0.99	1.07	1.03	1.07	71.58	7.17
Q8VHI6	Wiskott-Aldrich syndrome protein family member 3 OS=Mus musculus GN=Wasf3 PE=2 SV=1 - [WASF3_MOUSE]	30.94	2	9	10	45	1.01	0.93	0.90	0.93	0.96	1.17	1.15	1.21	1.23	1.15	1.10	1.08	1.14	55.17	6.37

Q9JHL1	Na(+)/H(+) exchange regulatory cofactor NHE-RF2 OS=Mus musculus GN=Slc9a3r2 PE=1 SV=2 - [NHRF2_MOUSE]	50.74	2	14	14	93	0.99	0.95	0.96	0.92	0.91	1.19	1.15	1.21	1.17	1.05	1.07	1.00	0.98	37.38	7.59
P97347	Repetin OS=Mus musculus GN=Rptn PE=1 SV=2 - [RPTN_MOUSE]	0.98	1	1	1	1	1.17	1.15	0.98	1.15	0.98	1.07	0.90	1.21	1.03	1.27	1.09	1.21	1.03	128.54	7.61
Q9ET26	RING finger protein 114 OS=Mus musculus GN=Rnf114 PE=2 SV=2 - [RN114_MOUSE]	22.27	1	3	3	8	1.16	0.98	0.88	0.92	0.83	1.27	1.12	1.21	1.02	1.10	1.05	1.31	1.18	25.73	7.03
Q9JIG7	Coiled-coil domain-containing protein 22 OS=Mus musculus GN=Ccdc22 PE=1 SV=1 - [CCD22_MOUSE]	7.34	1	4	4	7	0.97	1.27	0.80	0.98	1.04	0.96	1.03	1.21	1.21	1.39	1.13	1.22	1.01	70.80	6.01
Q91YE8-2	Isoform 2 of Synaptopodin-2 OS=Mus musculus GN=Synpo2 - [SYNP2_MOUSE]	8.72	4	4	4	7	0.91	0.80	1.01	0.98	1.08	1.57	1.80	1.21	1.47	1.37	1.58	1.07	1.27	80.86	9.32
Q5DQR4-	Isoform 4 of Syntaxin-binding protein 5-like OS=Mus musculus GN=Stxbp5l - [STB5L_MOUSE]	12.23	5	1	10	19	1.49	1.45	0.97	1.35	0.90	1.34	0.90	1.21	0.81	1.25	0.84	1.23	0.83	122.79	6.54
Q3U5F4	YrdC domain-containing protein, mitochondrial OS=Mus musculus GN=Yrdc PE=1 SV=1 - [YRDC_MOUSE]	5.36	1	1	1	2	1.08	3.27	3.01	0.99	0.92	1.20	1.10	1.21	1.11	1.12	1.03	1.15	1.06	29.44	6.07
Q9DCG9	tRNA methyltransferase 112 homolog OS=Mus musculus GN=Trmt112 PE=2 SV=1 - [TR112_MOUSE]	50.40	2	4	4	10	1.26	1.01	0.77	1.04	0.84	1.26	1.08	1.21	0.99	1.02	0.84	1.25	0.96	14.13	5.27
Q80UF4	Serologically defined colon cancer antigen 8 homolog OS=Mus musculus GN=Sdccag8 PE=1 SV=1 - [SDCG8_MOUSE]	1.39	1	1	1	2	1.08	1.06	0.98	1.00	0.93	1.06	0.98	1.21	1.12	1.14	1.06	1.25	1.16	82.93	6.52
P97868	E3 ubiquitin-protein ligase RBBP6 OS=Mus musculus GN=Rbbp6 PE=1 SV=5 - [RBBP6_MOUSE]	8.10	2	12	12	34	1.06	0.97	0.92	1.03	0.99	1.18	1.10	1.21	1.13	1.16	1.08	1.14	1.07	199.47	9.63
Q91VW5	Golgin subfamily A member 4 OS=Mus musculus GN=Golga4 PE=1 SV=2 - [GOGA4_MOUSE]	27.21	1	49	49	115	1.03	0.95	0.93	0.91	0.89	1.23	1.18	1.21	1.15	1.11	1.08	1.00	0.97	257.41	5.36
Q9CR80	Protein FAM32A OS=Mus musculus GN=Fam32a PE=2 SV=1 - [FA32A_MOUSE]	27.68	1	3	3	5	1.11	1.00	1.08	1.20	1.08	1.35	1.03	1.21	1.09	1.18	1.07	1.16	1.05	13.21	9.94

G5E8E1	Leucine rich repeat (In FLII) interacting protein 1, isoform CRA_e OS=Mus musculus GN=Lrrfip1 PE=4 SV=1 - [G5E8E1_MOUSE]	40.65	2	1	15	65	0.84	0.79	0.94	0.77	0.77	1.04	1.22	1.21	1.43	1.14	1.35	1.17	1.39	48.89	5.57
Q80WC7-	Isoform 2 of Arf-GAP domain and FG repeat-containing protein 2 OS=Mus musculus GN=Agfg2 - [AGFG2_MOUSE]	35.64	6	6	8	10	0.99	1.05	1.06	1.02	1.08	1.10	1.07	1.21	1.28	1.14	1.06	1.01	1.23	30.72	9.44
P28656	Nucleosome assembly protein 1-like 1 OS=Mus musculus GN=Nap1l1 PE=1 SV=2 - [NP1L1_MOUSE]	37.85	3	9	10	106	0.99	0.79	0.82	0.83	0.87	1.17	1.16	1.21	1.25	1.02	1.10	1.00	1.04	45.32	4.46
Q3UQN2-	Isoform 3 of FCH domain only protein 2 OS=Mus musculus GN=Fcho2 - [FCHO2_MOUSE]	3.38	2	1	1	2	0.99	1.05	1.06	0.99	1.00	1.13	1.13	1.21	1.22	1.14	1.16	1.11	1.13	46.94	6.34
D3YZY3	Transcription cofactor vestigial-like protein 4 (Fragment) OS=Mus musculus GN=Vgll4 PE=2 SV=1 - [D3YZY3_MOUSE]	8.47	3	1	1	2	0.86	1.34	1.56	0.89	1.04	1.35	1.57	1.21	1.41	1.16	1.36	1.08	1.26	20.40	8.94
Q6PD21	SH2 domain-containing adapter protein B OS=Mus musculus GN=Shb PE=1 SV=2 - [SHB_MOUSE]	6.16	3	2	2	4	1.02	0.99	0.97	0.95	0.93	1.06	1.04	1.21	1.19	0.96	0.94	1.18	1.16	54.67	8.78
Q9CQF7	Prefoldin 1 OS=Mus musculus GN=Pfdn1 PE=2 SV=1 - [Q9CQF7_MOUSE]	50.00	2	4	5	30	1.03	0.89	0.88	0.96	0.91	1.19	1.17	1.21	1.22	1.04	1.10	1.00	0.99	14.25	6.81
B1ASP0	Ribonucleoprotein PTB-binding 2 OS=Mus musculus GN=Raver2 PE=2 SV=1 - [B1ASP0_MOUSE]	10.02	2	1	1	1	0.79	1.16	1.46	0.96	1.21	0.95	1.19	1.21	1.53	0.87	1.10	1.13	1.43	49.62	7.37
Q3UTJ2-2	Isoform 2 of Sorbin and SH3 domain-containing protein 2 OS=Mus musculus GN=Sorbs2 - [SRBS2_MOUSE]	43.01	21	43	44	154	0.99	1.02	1.00	0.96	0.96	1.11	1.10	1.21	1.20	1.16	1.14	1.00	1.00	144.92	8.27
Q9DAY7	Mediator of RNA polymerase II transcription subunit 8 OS=Mus musculus GN=Med8 PE=2 SV=1 - [Q9DAY7_MOUSE]	8.12	2	1	1	2	0.81	1.48	1.83	0.76	0.94	0.70	0.86	1.21	1.50	0.56	0.69	0.66	0.81	25.94	6.90
Q9CWK3	CD2 antigen cytoplasmic tail-binding protein 2 OS=Mus musculus GN=Cd2bp2 PE=1 SV=1 - [CD2B2_MOUSE]	44.44	1	9	9	39	0.93	0.87	0.90	0.96	1.00	1.29	1.30	1.21	1.29	1.08	1.14	1.01	1.14	37.67	4.60

Q9WUK2	Eukaryotic translation initiation factor 4H OS=Mus musculus GN=Eif4h PE=1 SV=3 - [IF4H_MOUSE]	52.82	2	10	10	189	1.03	0.85	0.78	0.94	0.93	1.21	1.21	1.21	1.16	1.08	1.03	0.95	0.95	27.32	7.23
Q9WU12	SNRPN upstream reading frame protein OS=Mus musculus GN=Snurf PE=1 SV=1 - [SNURF_MOUSE]	32.39	1	2	2	3	1.24	1.88	1.52	0.94	0.76	1.19	0.95	1.21	0.98	1.18	0.95	1.10	0.89	8.40	10.58
Q6NZF1	Zinc finger CCCH domain-containing protein 11A OS=Mus musculus GN=Zc3h11a PE=1 SV=1 - [ZC11A_MOUSE]	19.07	1	12	12	24	1.03	0.87	0.90	0.95	0.93	1.20	1.16	1.21	1.14	1.10	1.01	1.06	1.00	86.44	8.13
A2AFI8	RalBP1-associated Eps domain-containing protein 2 OS=Mus musculus GN=Reps2 PE=2 SV=1 - [A2AFI8_MOUSE]	33.54	4	14	15	34	0.98	0.96	0.99	0.87	0.93	1.16	1.25	1.21	1.23	1.11	1.13	1.01	1.02	70.44	7.25
Q6A058	Armadillo repeat-containing X-linked protein 2 OS=Mus musculus GN=Armcx2 PE=2 SV=2 - [ARMX2_MOUSE]	6.38	1	3	3	6	0.98	1.22	1.18	0.98	0.95	1.12	1.08	1.21	1.18	1.17	1.13	1.15	1.18	80.99	9.33
D6RG94	Cadherin-8 OS=Mus musculus GN=Cdh8 PE=2 SV=1 - [D6RG94_MOUSE]	11.20	6	4	4	10	1.00	1.22	1.22	1.10	1.07	1.13	1.11	1.21	1.21	1.14	1.13	1.16	1.18	56.47	5.16
E9Q6Q2	Serine/threonine-protein kinase WNK2 OS=Mus musculus GN=Wnk2 PE=2 SV=1 - [E9Q6Q2_MOUSE]	19.74	18	24	26	71	1.02	0.92	0.94	1.00	0.96	1.25	1.20	1.22	1.22	1.22	1.18	1.16	1.15	210.99	5.78
Q9WTQ5	A-kinase anchor protein 12 OS=Mus musculus GN=Akap12 PE=1 SV=1 - [AKA12_MOUSE]	44.60	2	47	47	160	1.07	0.90	0.84	0.92	0.89	1.19	1.14	1.22	1.18	1.12	1.11	0.98	0.98	180.59	4.44
G5E850	Cytochrome b-5, isoform CRA_a OS=Mus musculus GN=Cyb5 PE=3 SV=1 - [G5E850_MOUSE]	56.12	3	4	4	31	0.95	1.07	1.10	1.04	0.99	1.25	1.23	1.22	1.24	1.13	1.13	1.08	1.09	11.14	5.30
E9QQ10	A-kinase anchor protein 9 OS=Mus musculus GN=Akap9 PE=2 SV=1 - [E9QQ10_MOUSE]	12.23	4	34	34	61	1.03	1.17	1.10	0.99	0.99	1.26	1.28	1.22	1.22	1.18	1.18	1.18	1.11	433.87	5.03
O08972	Dimethylarginine dimethylaminohydrolase 2, isoform CRA_a OS=Mus musculus GN=Ddah2 PE=2 SV=1 - [O08972_MOUSE]	32.38	3	5	6	19	1.05	1.02	0.96	1.00	0.94	1.29	1.17	1.22	1.16	1.36	1.41	1.46	1.34	25.35	6.06

Q9Z2U2-2	Isoform 2 of Zinc finger protein 292 OS=Mus musculus GN=Zfp292 - [ZN292_MOUSE]	1.26	2	1	1	1	1.23	1.01	0.82	1.32	1.07	1.48	1.20	1.22	0.98	1.17	0.95	1.47	1.19	300.26	7.56
Q61160	Protein FADD OS=Mus musculus GN=Fadd PE=1 SV=1 - [FADD_MOUSE]	4.39	1	1	1	2	0.74	0.91	1.22	0.78	1.05	1.00	1.34	1.22	1.63	0.99	1.33	1.01	1.36	22.95	6.04
Q63850	Nuclear pore glycoprotein p62 OS=Mus musculus GN=Nup62 PE=1 SV=2 - [NUP62_MOUSE]	24.71	1	10	10	53	1.02	0.91	0.92	0.90	0.88	1.17	1.12	1.22	1.19	1.05	1.04	0.97	0.95	53.22	5.31
Q9WTP2	Protein sprouty homolog 4 OS=Mus musculus GN=Spry4 PE=2 SV=1 - [SPY4_MOUSE]	9.67	1	2	2	4	1.08	1.22	1.13	1.01	0.93	1.11	1.02	1.22	1.12	1.12	1.04	1.04	0.97	32.50	7.88
B1AXI9	5-azacytidine induced gene 1 OS=Mus musculus GN=Azi1 PE=2 SV=1 - [B1AXI9_MOUSE]	3.59	3	3	3	5	1.04	1.12	1.04	1.02	0.98	1.14	1.05	1.22	1.13	1.08	1.00	0.96	0.92	120.16	8.65
Q6P6I8	Signal-regulatory protein alpha OS=Mus musculus GN=Sirpa PE=2 SV=1 - [Q6P6I8_MOUSE]	48.92	5	1	19	212	1.11	1.14	0.96	1.06	0.99	1.19	0.95	1.22	1.10	1.18	1.03	1.12	0.98	55.95	8.28
O88622-2	Isoform 2 of Poly(ADP-ribose) glycohydrolase OS=Mus musculus GN=Parg - [PARG_MOUSE]	4.13	5	3	3	5	0.96	0.75	0.77	0.97	1.02	1.10	1.16	1.22	1.16	1.13	1.13	1.14	1.07	103.71	6.80
Q9CQN7	39S ribosomal protein L41, mitochondrial OS=Mus musculus GN=Mrpl41 PE=2 SV=1 - [RM41_MOUSE]	51.85	2	6	6	14	1.02	0.77	0.76	0.91	0.90	1.35	1.30	1.22	1.18	1.12	1.10	1.08	1.06	15.25	9.82
Q6PAQ9	Transmembrane gamma-carboxyglutamic acid protein 3 OS=Mus musculus GN=Prrg3 PE=2 SV=2 - [TMG3_MOUSE]	6.06	1	1	1	2	1.00	1.05	1.05	1.01	1.01	1.16	1.16	1.22	1.22	1.05	1.06	0.91	0.92	25.86	6.30
Q9CZG3	COMM domain-containing protein 8 OS=Mus musculus GN=Commd8 PE=2 SV=1 - [COMD8_MOUSE]	14.21	1	2	2	3	1.28	1.16	0.91	1.21	0.73	1.28	1.07	1.22	1.24	1.16	0.99	1.45	1.14	20.84	5.59
E9Q2Y9	Desumoylating isopeptidase 1 OS=Mus musculus GN=Desi1 PE=2 SV=1 - [E9Q2Y9_MOUSE]	19.51	5	1	1	1	0.96	0.80	0.83	1.03	1.08	1.31	1.36	1.22	1.27	1.21	1.27	1.21	1.27	4.67	9.47
Q64487-1	Isoform C of Receptor-type tyrosine-protein phosphatase delta OS=Mus musculus GN=Ptprd - [PTPRD_MOUSE]	33.97	14	1	42	141	0.98	1.53	1.56	1.02	1.04	1.11	1.13	1.22	1.24	1.03	1.06	1.03	1.06	212.01	6.68

Q9WTJ4	Flt3-interacting zinc finger protein 1 OS=Mus musculus GN=Fiz1 PE=1 SV=1 - [FIZ1_MOUSE]	2.00	1	1	1	2	1.02	0.99	0.97	1.08	1.06	1.29	1.26	1.22	1.19	1.16	1.14	1.16	1.14	52.65	7.94
P15532	Nucleoside diphosphate kinase A OS=Mus musculus GN=Nme1 PE=1 SV=1 - [NDKA_MOUSE]	74.34	3	3	11	86	1.00	0.85	0.81	0.92	0.93	1.15	1.18	1.22	1.22	1.18	1.11	1.14	1.10	17.20	7.37
Q8BJ05-3	Isoform 3 of Zinc finger CCCH domain-containing protein 14 OS=Mus musculus GN=Zc3h14 - [ZC3HE_MOUSE]	31.95	3	1	13	30	1.03	0.42	0.44	1.03	1.00	1.43	1.39	1.22	1.18	1.04	1.01	0.95	0.92	67.80	8.06
B1AQX9	SRC kinase-signaling inhibitor 1 OS=Mus musculus GN=Srcin1 PE=2 SV=1 - [B1AQX9_MOUSE]	48.40	2	2	43	160	0.95	1.08	1.16	1.00	1.09	1.25	1.26	1.22	1.28	1.16	1.24	1.14	1.22	130.53	9.25
P14602-3	Isoform C of Heat shock protein beta-1 OS=Mus musculus GN=Hspb1 - [HSPB1_MOUSE]	38.29	4	4	4	5	1.20	1.07	0.86	1.13	0.93	1.09	0.91	1.22	0.98	1.24	1.01	1.12	0.97	19.37	6.95
D3YWN7	Ensconsin OS=Mus musculus GN=Map7 PE=4 SV=2 - [D3YWN7_MOUSE]	26.15	5	14	15	28	1.00	1.12	1.13	1.00	0.98	1.09	1.05	1.22	1.05	1.17	1.08	1.10	1.15	82.79	9.38
Q64704-3	Isoform 3C of Syntaxin-3 OS=Mus musculus GN=Stx3 - [STX3_MOUSE]	11.52	6	1	2	7	1.37	1.37	0.99	0.97	0.70	1.49	1.08	1.22	0.88	1.52	1.11	1.15	0.84	30.91	5.63
Q3UN16	Probable G-protein coupled receptor 162 OS=Mus musculus GN=Gpr162 PE=2 SV=2 - [GP162_MOUSE]	11.73	1	4	4	11	1.07	1.17	1.07	1.14	1.00	1.15	1.15	1.22	1.11	1.14	1.12	0.99	0.95	64.11	8.79
Q8C9B9	Death-inducer obliterator 1 OS=Mus musculus GN=Dido1 PE=1 SV=4 - [DIDO1_MOUSE]	6.43	4	8	9	14	1.05	0.98	0.93	1.02	1.04	1.18	1.10	1.22	1.21	1.22	1.16	1.16	1.09	247.02	7.91
Q80SY4	E3 ubiquitin-protein ligase MIB1 OS=Mus musculus GN=Mib1 PE=1 SV=1 - [MIB1_MOUSE]	0.99	1	1	1	2	1.03	1.03	1.00	1.10	1.07	1.24	1.20	1.22	1.18	1.13	1.10	1.07	1.05	110.02	6.92
Q8C180	Fibroblast growth factor receptor substrate 2 OS=Mus musculus GN=Frs2 PE=1 SV=3 - [FRS2_MOUSE]	12.99	1	4	4	8	1.12	0.96	0.81	1.01	0.89	1.28	1.16	1.22	1.12	1.22	1.11	1.06	0.93	56.76	6.16
Q8BMK4	Cytoskeleton-associated protein 4 OS=Mus musculus GN=Ckap4 PE=2 SV=2 - [CKAP4_MOUSE]	23.65	1	14	14	25	1.01	1.05	1.01	1.04	1.00	1.28	1.26	1.22	1.19	1.17	1.14	1.02	1.01	63.65	5.64

A2A6T1	Cerebellar degeneration related protein 2-like OS=Mus musculus GN=Cdr2l PE=2 SV=1 - [CDR2L_MOUSE]	13.98	1	5	5	19	1.00	0.97	0.97	0.85	0.85	1.25	1.14	1.22	1.22	0.90	0.96	1.07	0.96	53.18	5.76
E9QKB2	Intracellular hyaluronan-binding protein 4 OS=Mus musculus GN=Habp4 PE=2 SV=1 - [E9QKB2_MOUSE]	54.85	2	16	16	102	1.14	0.94	0.83	0.97	0.93	1.25	1.12	1.22	1.05	1.09	0.97	1.04	0.95	45.94	6.84
I6L9J1	Cadherin-22 OS=Mus musculus GN=Cdh22 PE=2 SV=1 - [I6L9J1_MOUSE]	2.45	3	1	1	2	0.96	1.05	1.09	0.85	0.88	1.14	1.18	1.22	1.27	0.98	1.03	0.87	0.91	44.67	4.77
A2AJW5	Uncharacterized protein OS=Mus musculus GN=Fam217b PE=4 SV=1 - [A2AJW5_MOUSE]	26.36	1	7	7	20	1.00	1.06	1.10	1.01	1.03	1.24	1.28	1.22	1.18	1.17	1.18	1.00	1.03	42.11	9.45
Q9D8S3	ADP-ribosylation factor GTPase-activating protein 3 OS=Mus musculus GN=Arfgap3 PE=2 SV=2 - [ARFG3_MOUSE]	14.72	1	6	6	9	1.16	1.10	1.00	1.05	0.92	1.14	0.98	1.22	1.10	1.19	1.03	1.10	1.06	57.42	8.47
Q9ESC8	AF4/FMR2 family member 4 OS=Mus musculus GN=Aff4 PE=1 SV=1 - [AFF4_MOUSE]	2.24	2	2	2	3	0.94	1.03	1.09	0.96	1.03	1.05	1.11	1.22	1.29	1.03	1.10	1.10	1.17	126.56	9.36
Q561M1	Acp1 protein OS=Mus musculus GN=Acp1 PE=2 SV=1 - [Q561M1_MOUSE]	49.37	2	1	4	39	0.98	0.92	1.02	0.96	0.95	1.00	1.06	1.22	1.24	1.07	1.12	1.02	1.09	17.91	6.54
P63038	60 kDa heat shock protein, mitochondrial OS=Mus musculus GN=Hspd1 PE=1 SV=1 - [CH60_MOUSE]	76.79	4	36	36	512	0.97	0.87	0.92	0.98	1.01	1.23	1.29	1.22	1.28	1.18	1.21	1.12	1.15	60.92	6.18
Q9JJX7	Tyrosyl-DNA phosphodiesterase 2 OS=Mus musculus GN=Tdp2 PE=1 SV=1 - [TYDP2_MOUSE]	3.78	1	1	1	3	0.89	1.05	0.98	0.91	1.02	1.54	1.72	1.22	1.37	0.83	0.94	1.32	1.25	41.01	5.50
P28798	Granulins OS=Mus musculus GN=Grn PE=1 SV=2 - [GRN_MOUSE]	16.98	4	7	7	15	0.95	0.85	1.03	0.99	1.05	1.13	1.22	1.22	1.24	1.08	1.05	1.06	1.12	63.41	6.80
Q69ZQ2	Pre-mRNA-splicing factor ISY1 homolog OS=Mus musculus GN=Isy1 PE=1 SV=2 - [ISY1_MOUSE]	25.61	1	6	6	10	0.95	0.86	0.91	1.03	1.05	1.07	1.07	1.22	1.30	1.05	1.04	1.05	1.10	32.97	5.17
P08551	Neurofilament light polypeptide OS=Mus musculus GN=Nefl PE=1 SV=5 - [NFL_MOUSE]	62.80	1	35	39	783	0.96	1.00	1.03	0.98	1.02	1.16	1.17	1.22	1.25	1.12	1.11	1.07	1.09	61.47	4.64
Q62011	Podoplanin OS=Mus musculus GN=Pdpn PE=1 SV=2 - [PDPN_MOUSE]	6.98	1	1	1	1	1.41	0.75	0.53	1.05	0.74	1.34	0.94	1.22	0.86	1.11	0.78	1.20	0.85	18.22	5.53

Q9CWU4	UPF0690 protein C1orf52 homolog OS=Mus musculus PE=1 SV=1 - [CA052_MOUSE]	52.22	3	7	7	38	1.05	0.76	0.74	0.94	0.94	1.18	1.18	1.22	1.22	1.08	1.10	0.88	0.92	20.09	5.11
Q8CCT4	Transcription elongation factor A protein-like 5 OS=Mus musculus GN=Tceal5 PE=1 SV=1 - [TCAL5_MOUSE]	59.50	3	8	15	171	0.83	0.62	0.78	0.89	1.11	1.07	1.47	1.22	1.57	1.09	1.47	0.77	1.02	22.02	6.20
Q9CZX8	40S ribosomal protein S19 OS=Mus musculus GN=Rps19 PE=1 SV=3 - [RS19_MOUSE]	49.66	5	10	10	31	0.98	0.89	0.91	0.90	0.94	1.17	1.13	1.22	1.25	1.07	1.14	1.02	1.03	16.08	10.40
Q9D8X2	Coiled-coil domain-containing protein 124 OS=Mus musculus GN=Ccdc124 PE=2 SV=1 - [CC124_MOUSE]	47.47	1	10	10	43	1.08	0.97	0.90	0.97	0.91	1.22	1.04	1.22	1.08	1.14	1.00	1.02	0.94	25.33	9.64
B1AWC9	Protein Pde4b OS=Mus musculus GN=Pde4b PE=2 SV=1 - [B1AWC9_MOUSE]	20.65	10	9	12	26	1.11	1.06	0.89	1.02	0.98	1.11	1.00	1.22	1.03	1.07	0.97	1.10	0.96	83.25	5.26
Q3V3V9-2	Isoform 2 of Leucine-rich repeat-containing protein 16C OS=Mus musculus GN=Rltpr - [LR16C_MOUSE]	31.57	2	16	16	91	1.08	1.20	1.08	1.01	0.93	1.15	1.05	1.22	1.10	1.11	1.03	1.06	0.99	79.71	6.77
B1ASC0	Brain-specific angiogenesis inhibitor 2 OS=Mus musculus GN=Bai2 PE=2 SV=2 - [B1ASC0_MOUSE]	5.91	7	5	6	14	1.12	1.34	1.14	1.11	1.01	1.10	1.00	1.22	1.12	1.12	0.99	1.20	1.07	160.46	7.12
Q9CQH7	Transcription factor BTF3 homolog 4 OS=Mus musculus GN=Btf3l4 PE=2 SV=1 - [BT3L4_MOUSE]	37.34	1	7	7	83	1.05	0.73	0.72	0.97	0.97	1.22	1.13	1.22	1.15	1.09	0.96	1.01	0.87	17.26	6.35
B1ATI9	Growth arrest-specific protein 7 OS=Mus musculus GN=Gas7 PE=4 SV=1 - [B1ATI9_MOUSE]	45.87	4	19	20	108	0.99	0.92	0.94	0.90	0.88	1.19	1.17	1.22	1.24	1.07	1.07	1.02	1.04	47.23	7.59
J3KMJ8	Axonemal dynein light chain domain-containing protein 1 (Fragment) OS=Mus musculus GN=Axdnd1 PE=4 SV=1 - [J3KMJ8_MOUSE]	0.80	2	1	1	1	1.16	0.86	0.74	1.25	1.08	1.20	1.03	1.22	1.05	0.95	0.82	1.13	0.98	102.24	6.13
Q3ZAQ4	Autophagy 9-like 1 protein OS=Mus musculus GN=Atg9a PE=2 SV=1 - [Q3ZAQ4_MOUSE]	4.65	2	2	2	8	1.13	1.48	1.31	1.34	1.18	1.12	0.99	1.22	1.08	1.10	0.97	1.13	1.00	94.36	6.64

Q9D1J3	SAP domain-containing ribonucleoprotein OS=Mus musculus GN=Sarnp PE=1 SV=3 - [SARNP_MOUSE]	25.24	1	4	4	22	1.04	0.94	0.87	0.98	0.90	1.23	1.19	1.22	1.19	1.04	0.99	1.04	1.01	23.52	6.65
Q91WG2	Rab GTPase-binding effector protein 2 OS=Mus musculus GN=Rabep2 PE=2 SV=3 - [RABE2_MOUSE]	35.56	5	16	16	39	0.96	0.92	0.97	0.91	0.94	1.18	1.19	1.22	1.25	1.06	1.12	1.02	1.06	62.09	4.89
Q5SRX1	TOM1-like protein 2 OS=Mus musculus GN=Tom1l2 PE=1 SV=1 - [TM1L2_MOUSE]	48.13	7	1	18	142	1.03	0.78	0.75	0.87	0.84	1.23	1.18	1.22	1.18	1.09	1.05	1.05	1.02	55.63	4.82
Q8VDY9	Caspase activity and apoptosis inhibitor 1 OS=Mus musculus GN=Caap1 PE=1 SV=2 - [CAAP1_MOUSE]	25.28	1	5	5	12	1.04	0.77	0.79	1.03	0.93	1.25	1.22	1.22	1.17	1.13	1.16	1.02	0.94	37.80	4.72
Q9JM96	Cdc42 effector protein 4 OS=Mus musculus GN=Cdc42ep4 PE=1 SV=1 - [BORG4_MOUSE]	52.44	3	12	12	60	1.07	0.96	0.90	0.98	0.92	1.27	1.18	1.22	1.15	1.20	1.14	1.04	0.95	37.85	5.36
Q80ZX8-2	Isoform 2 of Sperm-associated antigen 1 OS=Mus musculus GN=Spag1 - [SPAG1_MOUSE]	2.65	3	1	1	2	1.12	1.03	0.92	1.06	0.95	1.15	1.02	1.22	1.09	1.40	1.25	1.23	1.10	58.47	5.78
Q61074	Protein phosphatase 1G OS=Mus musculus GN=Ppm1g PE=2 SV=3 - [PPM1G_MOUSE]	33.58	1	13	13	37	0.99	0.92	0.96	0.97	0.91	1.32	1.32	1.22	1.16	1.07	1.09	1.04	1.07	58.69	4.39
Q9WTP7	GTP:AMP phosphotransferase AK3, mitochondrial OS=Mus musculus GN=Ak3 PE=1 SV=3 - [KAD3_MOUSE]	47.58	2	11	11	29	0.97	0.90	0.93	1.03	1.07	1.19	1.24	1.22	1.21	1.21	1.20	1.07	1.08	25.41	8.84
D3Z3M7	CAP-Gly domain-containing linker protein 1 OS=Mus musculus GN=Clip1 PE=2 SV=1 - [D3Z3M7_MOUSE]	59.24	3	4	78	345	1.26	0.71	0.66	1.04	0.86	1.22	1.02	1.22	0.94	1.14	0.90	1.14	0.93	147.91	5.31
Q3U898	Myeloma-overexpressed gene 2 protein homolog OS=Mus musculus GN=Myeov2 PE=2 SV=1 - [MYOV2_MOUSE]	35.09	2	1	1	15	1.07	0.94	0.79	0.87	0.83	1.45	1.26	1.22	1.16	1.22	0.98	1.17	1.15	6.19	3.83
Q3U1F9	Phosphoprotein associated with glycosphingolipid-enriched microdomains 1 OS=Mus musculus GN=Pag1 PE=1 SV=2 - [PHAG1_MOUSE]	39.86	1	10	10	29	1.01	1.06	1.20	0.95	0.94	1.19	1.19	1.22	1.22	1.18	1.12	1.05	1.08	46.52	4.81

E9QP59	Inner nuclear membrane protein Man1 OS=Mus musculus GN=Lemd3 PE=2 SV=1 - [E9QP59_MOUSE]	9.80	4	5	5	10	1.01	1.35	1.39	1.00	0.93	1.13	1.08	1.22	1.20	1.16	1.13	0.98	0.95	100.10	7.55
D3Z7E6	Platelet-activating factor acetylhydrolase IB subunit gamma OS=Mus musculus GN=Pafah1b3 PE=2 SV=1 - [D3Z7E6_MOUSE]	16.11	4	3	3	5	1.21	1.07	1.00	1.01	1.02	1.23	1.11	1.22	1.14	1.32	1.19	1.33	1.29	20.14	7.75
A2AHG0	Leucine zipper putative tumor suppressor 3 OS=Mus musculus GN=Lzts3 PE=2 SV=1 - [LZTS3_MOUSE]	29.86	3	14	15	49	1.00	1.05	1.06	0.94	0.98	1.07	1.09	1.22	1.21	1.04	1.08	1.04	1.07	74.94	6.99
P55821	Stathmin-2 OS=Mus musculus GN=Stmn2 PE=1 SV=1 - [STMN2_MOUSE]	21.79	1	3	6	103	0.94	0.87	0.97	0.96	1.00	1.26	1.23	1.22	1.27	1.26	1.25	1.19	1.28	20.81	8.32
Q80WT0	Junctophilin-4 OS=Mus musculus GN=Jph4 PE=2 SV=1 - [JPH4_MOUSE]	7.96	2	4	4	7	0.96	1.05	1.09	1.02	1.02	1.18	1.22	1.22	1.22	1.19	1.20	1.11	1.11	65.96	6.71
Q8JZM4	Delta and Notch-like epidermal growth factor-related receptor OS=Mus musculus GN=Dner PE=1 SV=1 - [DNER_MOUSE]	1.90	1	1	1	1	1.01	1.03	1.02	0.86	0.86	1.01	0.99	1.22	1.21	1.23	1.22	0.97	0.97	78.69	5.10
Q9R0P4	Small acidic protein OS=Mus musculus GN=Smap PE=1 SV=1 - [SMAP_MOUSE]	26.52	3	5	5	140	1.03	0.91	0.92	1.02	0.97	1.13	1.10	1.22	1.14	0.98	1.02	1.08	1.06	20.03	4.82
Q9CRB9	Coiled-coil-helix-coiled-coil-helix domain-containing protein 3, mitochondrial OS=Mus musculus GN=Chchd3 PE=1 SV=1 - [CHCH3_MOUSE]	40.09	3	2	12	132	1.00	0.84	0.85	0.98	0.97	1.24	1.27	1.22	1.24	1.20	1.17	1.08	1.12	26.32	8.37
Q9ER58	Testican-2 OS=Mus musculus GN=Spock2 PE=2 SV=1 - [TICN2_MOUSE]	40.43	2	14	14	46	1.11	0.85	0.78	1.00	0.91	1.22	1.08	1.22	1.12	1.11	1.04	1.03	0.98	46.83	4.87
O70146	Dual specificity testis-specific protein kinase 1 OS=Mus musculus GN=Tesk1 PE=2 SV=3 - [TESK1_MOUSE]	5.10	1	2	2	4	1.00	1.09	1.09	1.15	1.15	1.05	1.05	1.22	1.22	1.26	1.26	1.29	1.29	68.01	8.06
Q9CQR2	40S ribosomal protein S21 OS=Mus musculus GN=Rps21 PE=2 SV=1 - [RS21_MOUSE]	40.96	1	4	4	41	1.03	1.06	1.05	0.91	0.89	1.11	1.13	1.22	1.25	1.14	1.10	1.20	1.16	9.14	8.51
Q8C3W1	Uncharacterized protein C1orf198 homolog OS=Mus musculus PE=1 SV=1 - [CA198_MOUSE]	53.42	1	12	12	52	1.14	1.02	0.84	0.99	0.87	1.21	1.00	1.23	1.07	1.20	1.06	1.16	1.04	35.29	5.24

E9Q9A5	Bifunctional polynucleotide phosphatase/kinase OS=Mus musculus GN=Pnkp PE=2 SV=1 - [E9Q9A5_MOUSE]	2.26	4	1	1	2	0.94	1.61	1.70	1.17	1.24	0.99	1.04	1.23	1.29	0.99	1.05	1.12	1.19	53.28	7.39
Q9JL04	Formin-2 OS=Mus musculus GN=Fmn2 PE=1 SV=2 - [FMN2_MOUSE]	11.15	1	10	10	26	1.01	0.99	1.00	0.96	0.90	1.24	1.14	1.23	1.23	1.17	1.11	1.18	1.16	167.28	5.48
Q3UZ39-2	Isoform 2 of Leucine-rich repeat flightless-interacting protein 1 OS=Mus musculus GN=Lrrfip1 - [LRRF1_MOUSE]	38.22	3	9	23	85	1.02	0.90	0.86	0.95	0.97	1.18	1.15	1.23	1.16	1.04	1.05	1.00	0.94	71.26	6.04
D3Z5R4	WAS/WASL-interacting protein family member 3 OS=Mus musculus GN=Wipf3 PE=4 SV=1 - [D3Z5R4_MOUSE]	57.46	2	1	17	84	1.04	1.06	1.02	0.97	0.93	1.17	1.12	1.23	1.17	1.22	1.17	1.06	1.03	45.11	10.84
Q8R4R6	Nucleoporin NUP53 OS=Mus musculus GN=Nup35 PE=1 SV=2 - [NUP53_MOUSE]	23.69	2	5	5	11	0.94	0.87	0.92	0.96	0.99	1.09	1.19	1.23	1.21	0.95	1.05	1.10	1.08	34.76	9.25
Q0VF62	Bcas3 protein OS=Mus musculus GN=Bcas3 PE=2 SV=1 - [Q0VF62_MOUSE]	14.13	5	1	10	31	1.01	1.01	0.99	0.92	0.90	1.29	1.27	1.23	1.20	1.13	1.11	1.04	1.03	99.40	6.64
G3UW94	MCG14259, isoform CRA_b OS=Mus musculus GN=U2af1 PE=4 SV=1 - [G3UW94_MOUSE]	27.62	2	4	5	12	1.01	1.02	1.01	1.14	1.09	1.24	1.22	1.23	1.22	1.17	1.16	1.11	1.13	27.81	8.68
Q7TNS5	Protein B630019K06Rik OS=Mus musculus GN=B630019K06Rik PE=2 SV=1 - [Q7TNS5_MOUSE]	10.21	1	2	2	4	1.34	1.29	0.96	1.03	0.77	1.23	0.92	1.23	0.91	1.20	0.89	1.53	1.15	34.84	6.65
Q8CFE6	Sodium-coupled neutral amino acid transporter 2 OS=Mus musculus GN=Slc38a2 PE=1 SV=1 - [S38A2_MOUSE]	4.17	1	1	1	2	0.92	1.10	1.19	1.10	1.20	0.85	0.92	1.23	1.33	0.86	0.94	1.14	1.24	55.47	7.94
Q8BG30	Negative elongation factor A OS=Mus musculus GN=Nelfa PE=1 SV=1 - [NELFA_MOUSE]	3.96	1	1	1	4	1.15	1.23	1.07	1.11	0.97	1.30	1.13	1.23	1.07	1.24	1.08	1.09	0.95	57.55	9.11
Q3TIV5	Zinc finger CCCH domain-containing protein 15 OS=Mus musculus GN=Zc3h15 PE=1 SV=2 - [ZC3HF_MOUSE]	41.78	3	18	18	55	1.08	0.96	0.84	1.05	0.95	1.21	1.10	1.23	1.13	1.15	1.06	1.01	0.93	48.30	5.30

Q99PM3	Transcription initiation factor IIA subunit 1 OS=Mus musculus GN=Gtf2a1 PE=2 SV=2 - [TF2AA_MOUSE]	6.88	3	3	3	10	1.00	1.07	1.05	0.98	1.03	1.19	1.19	1.23	1.18	1.19	1.20	1.07	1.11	41.59	4.55
Q9R022	DnaJ homolog subfamily C member 12 OS=Mus musculus GN=Dnajc12 PE=2 SV=1 - [DJC12_MOUSE]	19.19	2	3	3	9	1.19	1.04	0.88	1.02	0.83	1.28	1.13	1.23	1.03	1.33	1.21	1.36	1.14	22.84	6.15
Q9D8L5	Coiled-coil domain-containing protein 91 OS=Mus musculus GN=Ccdc91 PE=2 SV=2 - [CCD91_MOUSE]	29.19	2	16	16	28	1.16	0.95	0.89	0.88	0.79	1.15	1.02	1.23	0.97	1.14	1.00	1.13	1.08	49.98	5.07
D3Z5I9	RNA-binding protein 33 OS=Mus musculus GN=Rbm33 PE=2 SV=1 - [D3Z5I9_MOUSE]	8.73	4	6	7	32	1.01	0.86	0.91	1.03	1.02	1.23	1.22	1.23	1.23	1.04	1.10	1.06	1.04	132.74	6.87
F6TWX0	Nuclear transcription factor Y subunit alpha (Fragment) OS=Mus musculus GN=Nfya PE=4 SV=1 - [F6TWX0_MOUSE]	9.13	6	1	1	2	1.11	1.12	1.01	1.41	1.27	1.50	1.34	1.23	1.10	1.44	1.29	1.24	1.12	22.74	9.63
Q921K9	B-cell CLL/lymphoma 7 protein family member B OS=Mus musculus GN=Bcl7b PE=1 SV=1 - [BCL7B_MOUSE]	41.09	4	4	6	13	0.85	0.82	0.96	0.92	1.15	0.99	1.36	1.23	1.44	1.11	1.32	0.92	1.16	22.22	4.75
G3X915	LysM and putative peptidoglycan-binding domain-containing protein 2 OS=Mus musculus GN=Lysmd2 PE=4 SV=1 - [G3X915_MOUSE]	33.49	3	5	6	14	1.08	1.07	1.01	0.96	0.86	1.28	1.14	1.23	1.10	1.07	0.97	1.07	0.98	23.58	5.52
Q62422	Osteoclast-stimulating factor 1 OS=Mus musculus GN=Ostf1 PE=1 SV=2 - [OSTF1_MOUSE]	29.77	1	4	4	11	0.97	0.69	0.76	0.72	0.80	1.07	1.09	1.23	1.22	1.16	1.16	0.84	0.96	23.77	5.68
A2A723	Mannosyl-oligosaccharide 1,2-alpha-mannosidase IB (Fragment) OS=Mus musculus GN=Man1a2 PE=2 SV=1 - [A2A723_MOUSE]	5.69	3	1	1	1	1.65	1.61	0.97	0.62	0.37	1.28	0.77	1.23	0.74	1.22	0.74	1.09	0.66	28.13	8.28
Q9JK92	Heat shock protein beta-8 OS=Mus musculus GN=Hspb8 PE=1 SV=1 - [HSPB8_MOUSE]	9.18	1	2	2	3	1.24	1.39	1.12	1.04	0.84	1.17	0.94	1.23	0.99	1.27	1.03	1.23	0.99	21.52	5.02

P97822	Acidic leucine-rich nuclear phosphoprotein 32 family member E OS=Mus musculus GN=Anp32e PE=1 SV=2 - [AN32E_MOUSE]	53.08	7	8	8	52	0.98	0.89	0.92	0.88	0.95	1.25	1.26	1.23	1.25	1.17	1.20	1.02	1.07	29.60	3.88
O88703	Potassium/sodium hyperpolarization-activated cyclic nucleotide-gated channel 2 OS=Mus musculus GN=Hcn2 PE=1 SV=1 - [HCN2_MOUSE]	9.39	3	4	5	31	1.10	1.04	0.98	0.99	0.88	1.11	0.92	1.23	0.98	1.03	0.98	1.00	0.86	94.66	8.73
Q8R1F0	Leydig cell tumor 10 kDa protein homolog OS=Mus musculus GN=D8Ertd738e PE=2 SV=1 - [L10K_MOUSE]	9.57	1	1	1	3	1.16	0.92	0.79	1.12	0.97	1.24	1.07	1.23	1.05	1.17	1.01	1.01	0.87	10.19	11.63
Q9DCI9	39S ribosomal protein L32, mitochondrial OS=Mus musculus GN=Mrpl32 PE=2 SV=1 - [RM32_MOUSE]	21.93	1	3	3	5	1.17	0.76	0.91	0.98	0.95	1.17	1.18	1.23	0.99	1.03	0.98	1.06	0.83	21.72	9.70
Q3U3C9-4	Isoform 4 of Genetic suppressor element 1 OS=Mus musculus GN=Gse1 - [GSE1_MOUSE]	3.74	4	2	3	4	1.15	1.20	1.05	1.10	0.96	1.06	0.92	1.23	1.07	0.82	0.71	1.06	0.93	132.22	7.52
O70481	E3 ubiquitin-protein ligase UBR1 OS=Mus musculus GN=Ubr1 PE=1 SV=2 - [UBR1_MOUSE]	1.76	1	2	2	3	1.15	1.29	1.12	1.16	1.01	1.21	1.04	1.23	1.06	1.30	1.13	1.20	1.04	200.11	6.05
P19246	Neurofilament heavy polypeptide OS=Mus musculus GN=Nefh PE=1 SV=3 - [NFH_MOUSE]	35.87	1	36	39	458	0.91	0.89	1.02	0.87	0.94	1.19	1.30	1.23	1.35	1.06	1.18	0.98	1.06	116.92	5.81
F6X4L9	2-methoxy-6-polyprenyl-1,4-benzoquinol methylase, mitochondrial (Fragment) OS=Mus musculus GN=Coq5 PE=4 SV=1 - [F6X4L9_MOUSE]	8.15	2	1	1	2	1.17	1.57	1.34	1.14	0.97	1.17	0.99	1.23	1.04	1.33	1.13	1.40	1.20	15.26	5.82
D6RIJ4	Nuclear autoantigen Sp-100 OS=Mus musculus GN=Sp100 PE=2 SV=1 - [D6RIJ4_MOUSE]	4.93	7	1	1	1	0.84	0.95	1.12	0.82	0.97	1.07	1.27	1.23	1.45	1.08	1.29	0.89	1.06	34.39	4.97
Q8BH80	Vesicle-associated membrane protein, associated protein B and C OS=Mus musculus GN=Vapb PE=2 SV=1 - [Q8BH80_MOUSE]	39.92	2	7	8	70	0.98	0.70	0.71	0.95	0.95	1.22	1.28	1.23	1.28	1.15	1.19	1.01	1.05	26.90	7.78

B1ATD5	MCG9626, isoform CRA_b OS=Mus musculus GN=Mtmr3 PE=4 SV=1 - [B1ATD5_MOUSE]	3.02	6	3	3	4	1.08	1.23	1.20	1.00	0.94	1.05	0.96	1.23	1.13	1.14	1.05	1.35	1.29	129.60	6.07
P24788	Cyclin-dependent kinase 11B OS=Mus musculus GN=Cdk11b PE=1 SV=2 - [CD11B_MOUSE]	6.63	3	5	5	9	1.04	0.84	0.87	1.00	0.98	1.22	1.15	1.23	1.22	1.11	1.07	1.00	0.99	91.46	5.39
Q80X50-2	Isoform 2 of Ubiquitin-associated protein 2-like OS=Mus musculus GN=Ubap2l - [UBP2L_MOUSE]	40.63	3	2	25	79	0.81	0.87	1.07	0.89	1.09	1.15	1.42	1.23	1.51	1.12	1.39	0.92	1.14	107.18	7.11
O09044	Synaptosomal-associated protein 23 OS=Mus musculus GN=Snap23 PE=1 SV=1 - [SNP23_MOUSE]	56.67	5	7	8	32	1.00	1.02	1.04	0.95	0.95	1.05	1.08	1.23	1.23	1.08	1.16	1.14	1.06	23.25	4.98
Q9CQF0	39S ribosomal protein L11, mitochondrial OS=Mus musculus GN=Mrpl11 PE=2 SV=1 - [RM11_MOUSE]	19.27	1	3	3	7	0.95	0.72	0.75	0.91	0.96	1.29	1.46	1.23	1.39	1.26	1.35	1.09	1.15	20.67	9.73
Q99N94	39S ribosomal protein L9, mitochondrial OS=Mus musculus GN=Mrpl9 PE=2 SV=2 - [RM09_MOUSE]	6.42	2	1	1	2	0.99	1.13	1.14	1.00	1.01	1.09	1.10	1.23	1.24	1.20	1.22	1.27	1.29	30.23	10.08
Q62170	P-selectin glycoprotein ligand 1 OS=Mus musculus GN=Selplg PE=1 SV=2 - [SELPL_MOUSE]	4.79	2	1	1	1	0.97	0.98	1.01	1.00	1.03	1.12	1.15	1.23	1.26	1.00	1.02	1.00	1.03	41.82	4.44
P47212	Galanin peptides OS=Mus musculus GN=Gal PE=2 SV=1 - [GALA_MOUSE]	17.74	1	1	1	3	1.09	0.96	0.88	0.66	0.61	1.05	0.96	1.23	1.13	1.29	1.19	1.16	1.07	13.46	6.54
Q8VD04	GRIP1-associated protein 1 OS=Mus musculus GN=Gripap1 PE=1 SV=1 - [GRAP1_MOUSE]	67.37	5	1	50	315	1.01	0.65	0.64	0.93	0.92	1.24	1.22	1.23	1.21	1.13	1.12	0.97	0.96	92.66	5.25
O35326	Serine/arginine-rich splicing factor 5 OS=Mus musculus GN=Srsf5 PE=1 SV=2 - [SRSF5_MOUSE]	29.37	5	7	9	35	1.07	0.83	0.80	0.98	0.95	1.19	1.11	1.23	1.10	1.09	0.96	0.99	0.93	30.87	11.56
Q3U0V1	Far upstream element-binding protein 2 OS=Mus musculus GN=Khsrp PE=1 SV=2 - [FUBP2_MOUSE]	47.19	1	28	30	313	0.99	0.95	0.95	0.94	0.95	1.21	1.23	1.23	1.25	1.10	1.11	1.05	1.08	76.73	7.33
E9Q3Q6	CD166 antigen OS=Mus musculus GN=Alcam PE=2 SV=1 - [E9Q3Q6_MOUSE]	38.42	5	15	15	60	1.13	0.94	0.88	1.08	0.91	1.27	1.11	1.23	1.02	1.24	1.06	1.26	1.03	63.63	7.27
E9Q983	Supervillin OS=Mus musculus GN=Svil PE=2 SV=1 - [E9Q983_MOUSE]	1.98	4	2	2	6	1.05	1.18	1.12	1.04	1.03	1.18	1.02	1.23	1.06	1.22	1.16	1.10	1.09	198.41	6.55

P63024	Vesicle-associated membrane protein 3 OS=Mus musculus GN=Vamp3 PE=1 SV=1 - [VAMP3_MOUSE]	48.54	1	2	6	52	1.13	1.15	1.01	0.94	0.85	1.23	1.01	1.23	1.07	1.13	1.18	1.19	1.15	11.47	8.50
Q61687	Transcriptional regulator ATRX OS=Mus musculus GN=Atrx PE=1 SV=3 - [ATRX_MOUSE]	9.33	8	16	16	43	0.96	0.98	0.99	1.02	1.06	1.28	1.29	1.23	1.28	1.10	1.17	1.03	1.09	278.41	6.68
Q9JJC6	RILP-like protein 1 OS=Mus musculus GN=Rilpl1 PE=1 SV=1 - [RIPL1_MOUSE]	59.85	1	17	17	70	1.04	1.09	1.06	0.93	0.95	1.16	1.16	1.23	1.26	1.13	1.10	0.94	1.06	47.29	5.16
Q9D2M8	Ubiquitin-conjugating enzyme E2 variant 2 OS=Mus musculus GN=Ube2v2 PE=2 SV=4 - [UB2V2_MOUSE]	51.03	8	2	6	80	1.08	1.20	1.12	1.05	0.93	1.17	1.13	1.23	1.11	1.09	0.98	1.11	1.00	16.36	8.09
Q3TUE1	Far upstream element-binding protein 1 OS=Mus musculus GN=Fubp1 PE=2 SV=1 - [Q3TUE1_MOUSE]	48.29	1	2	23	238	1.03	0.94	0.86	0.94	0.90	1.18	1.10	1.23	1.16	1.16	1.11	1.02	0.99	67.40	7.61
Q91VL8	Telomeric repeat-binding factor 2-interacting protein 1 OS=Mus musculus GN=Terf2ip PE=1 SV=1 - [TE2IP_MOUSE]	31.55	1	8	8	17	1.02	0.86	0.81	0.94	0.94	1.27	1.21	1.23	1.24	1.04	1.10	1.00	1.09	43.33	4.81
Q6NXJ0	Protein WWC2 OS=Mus musculus GN=Wwc2 PE=2 SV=1 - [WWC2_MOUSE]	2.11	1	2	2	2	1.10	1.28	1.15	1.02	0.92	1.25	1.12	1.23	1.11	1.22	1.10	1.19	1.08	132.54	5.71
O88602	Voltage-dependent calcium channel gamma-2 subunit OS=Mus musculus GN=Cacng2 PE=1 SV=1 - [CCG2_MOUSE]	14.55	1	2	4	10	1.00	1.62	1.68	1.00	1.00	1.08	1.18	1.23	1.23	0.95	0.95	1.04	1.08	35.87	8.98
Q9D0B0	Serine/arginine-rich splicing factor 9 OS=Mus musculus GN=Srsf9 PE=1 SV=1 - [SRSF9_MOUSE]	19.37	1	2	4	8	1.00	0.78	0.78	0.86	0.86	1.02	1.01	1.23	1.23	1.04	1.04	0.94	0.94	25.65	8.65
Q3UH68	LIM and calponin homology domains-containing protein 1 OS=Mus musculus GN=Limch1 PE=1 SV=2 - [LIMC1_MOUSE]	40.59	9	30	30	77	1.03	1.02	0.98	0.93	0.89	1.22	1.17	1.23	1.19	1.14	1.12	1.12	1.03	118.12	5.48
Q3U155	Coiled-coil domain-containing protein 174 OS=Mus musculus GN=Ccdc174 PE=2 SV=1 - [CC174_MOUSE]	8.35	1	3	3	4	0.97	0.92	1.16	1.01	1.12	1.08	1.35	1.23	1.19	1.17	1.24	1.08	1.16	53.91	6.21

Q9QUK9	MCG15083 OS=Mus musculus GN=Try5 PE=2 SV=1 - [Q9QUK9_MOUSE]	12.20	3	1	2	8	0.95	0.85	0.90	0.82	0.86	1.12	1.18	1.23	1.30	1.08	1.14	1.09	1.15	26.26	5.30
Q91X43	SH3 domain-containing protein 19 OS=Mus musculus GN=Sh3d19 PE=1 SV=2 - [SH319_MOUSE]	9.25	2	4	4	7	1.02	1.09	1.01	1.09	1.04	1.16	1.19	1.23	1.24	1.24	1.19	1.03	1.04	86.02	8.53
P43274	Histone H1.4 OS=Mus musculus GN=Hist1h1e PE=1 SV=2 - [H14_MOUSE]	39.73	3	3	13	80	1.09	0.86	0.77	0.97	1.00	1.10	0.96	1.23	1.07	1.15	1.07	1.03	0.95	21.96	11.11
P62892	60S ribosomal protein L39 OS=Mus musculus GN=Rpl39 PE=2 SV=2 - [RL39_MOUSE]	19.61	1	1	1	6	1.03	1.26	1.27	1.11	1.06	1.02	0.98	1.23	1.18	1.15	1.10	1.27	1.26	6.40	12.56
Q3TUF7-3	Isoform 3 of YEATS domain-containing protein 2 OS=Mus musculus GN=Yeats2 - [YETS2_MOUSE]	1.33	2	1	1	1	0.93	0.45	0.49	0.82	0.88	1.27	1.36	1.23	1.32	1.02	1.10	0.82	0.88	142.91	9.10
Q62481	Vacuolar protein sorting-associated protein 72 homolog OS=Mus musculus GN=Vps72 PE=2 SV=2 - [VPS72_MOUSE]	9.24	1	3	3	4	1.11	1.04	1.04	1.06	1.04	1.15	1.20	1.23	1.27	1.26	1.14	1.07	0.84	40.76	6.40
Q8BYK5-	Isoform 4 of Phosphatase and actin regulator 3 OS=Mus musculus GN=Phactr3 - [PHAR3_MOUSE]	16.22	5	8	8	18	1.10	1.08	1.04	1.09	0.97	1.25	1.14	1.23	1.12	1.18	1.08	1.14	1.09	58.58	9.14
Q68EF6	Brain-enriched guanylate kinase-associated protein OS=Mus musculus GN=Begain PE=1 SV=2 - [BEGIN_MOUSE]	60.00	2	28	28	93	0.98	0.94	0.99	0.98	0.96	1.12	1.18	1.23	1.28	1.08	1.11	1.02	1.09	65.27	5.87
B0V2H4	V-type proton ATPase subunit G 2 OS=Mus musculus GN=Atp6v1g2 PE=2 SV=1 - [B0V2H4_MOUSE]	81.72	3	10	10	295	0.97	1.05	1.09	0.89	0.94	1.18	1.22	1.23	1.29	1.14	1.20	1.18	1.23	10.88	10.24
Q91YQ3	Cold shock domain-containing protein C2 OS=Mus musculus GN=Csdc2 PE=2 SV=2 - [CSDC2_MOUSE]	12.99	2	2	2	4	0.82	0.99	1.20	1.03	1.04	1.27	1.27	1.23	1.43	1.02	1.05	1.07	1.29	16.84	7.55
A6H8H5	Potassium voltage-gated channel subfamily B member 2 OS=Mus musculus GN=Kcnb2 PE=2 SV=2 - [KCNB2_MOUSE]	9.70	1	6	6	12	1.10	1.04	1.05	1.02	0.92	1.17	1.03	1.23	1.13	1.12	1.03	0.94	0.92	102.27	6.10

Q8BHG2	UPF0587 protein C1orf123 homolog OS=Mus musculus PE=2 SV=1 - [CA123_MOUSE]	75.00	9	9	9	51	1.03	0.89	0.81	0.90	0.90	1.17	1.16	1.23	1.18	1.03	1.00	0.99	0.94	18.01	5.12
D3YYH0	PEX5-related protein OS=Mus musculus GN=Pex5l PE=2 SV=1 - [D3YYH0_MOUSE]	39.59	7	18	18	47	0.97	1.08	1.13	1.00	1.08	1.07	1.14	1.23	1.29	1.00	1.08	0.96	1.04	65.80	5.22
E9PV26	Protein BC068157 OS=Mus musculus GN=BC068157 PE=2 SV=1 - [E9PV26_MOUSE]	20.04	3	14	14	56	1.08	1.00	0.98	0.97	0.92	1.19	1.12	1.23	1.15	1.10	1.03	1.10	1.02	110.62	9.98
Q62384	Zinc finger protein ZPR1 OS=Mus musculus GN=Znf259 PE=1 SV=1 - [ZPR1_MOUSE]	38.34	3	13	13	25	1.01	0.98	0.95	0.84	0.87	1.11	1.13	1.23	1.30	1.09	1.11	1.01	1.04	50.68	4.78
F6YMV1	Angiomotin (Fragment) OS=Mus musculus GN=Amot PE=4 SV=1 - [F6YMV1_MOUSE]	24.85	5	10	12	32	1.11	0.97	0.89	1.01	0.88	1.30	1.13	1.24	1.13	1.21	1.09	1.10	0.99	75.68	7.94
P60041	Somatostatin OS=Mus musculus GN=Sst PE=2 SV=1 - [SMS_MOUSE]	37.93	1	2	2	7	1.18	1.26	1.07	1.22	0.95	1.33	1.06	1.24	1.04	1.25	1.05	1.20	1.17	12.74	5.52
Q3UMU9	Isoform 4 of Hepatoma-derived growth factor-related protein 2 OS=Mus musculus GN=Hdgfrp2 - [HDGR2_MOUSE]	17.72	4	9	11	27	1.15	1.18	0.96	1.00	0.91	1.06	0.94	1.24	1.07	1.13	1.04	1.05	1.02	68.49	9.00
Q8R4S0	Protein phosphatase 1 regulatory subunit 14C OS=Mus musculus GN=Ppp1r14c PE=1 SV=1 - [PP14C_MOUSE]	22.56	1	2	2	7	0.99	0.87	0.87	0.94	0.95	1.08	1.08	1.24	1.14	1.04	1.04	1.10	0.98	17.74	5.27
Q3UHD3	Microtubule-associated tumor suppressor candidate 2 homolog OS=Mus musculus GN=Mtus2 PE=2 SV=1 - [MTUS2_MOUSE]	16.33	7	17	17	36	0.97	0.92	1.01	0.88	0.95	1.16	1.26	1.24	1.25	1.09	1.19	1.00	1.07	147.27	8.24
Q3THK3	General transcription factor IIF subunit 1 OS=Mus musculus GN=Gtf2f1 PE=1 SV=2 - [T2FA_MOUSE]	24.41	1	10	10	20	1.09	1.04	1.01	1.09	1.02	1.19	1.03	1.24	1.13	1.30	1.15	1.15	1.07	57.21	7.01
Q8BN59	La-related protein 6 OS=Mus musculus GN=Larp6 PE=1 SV=1 - [LARP6_MOUSE]	5.89	1	2	2	3	1.12	1.23	1.09	1.10	0.98	1.07	0.95	1.24	1.10	1.05	0.94	1.91	1.71	54.84	7.90
E9Q9H2	DnaJ homolog subfamily C member 2 OS=Mus musculus GN=Dnajc2 PE=2 SV=1 - [E9Q9H2_MOUSE]	16.64	4	8	8	14	1.07	0.99	0.90	1.03	0.95	1.16	1.06	1.24	1.13	1.26	1.19	1.17	1.07	63.40	9.17

E9Q7G0	Protein Numa1 OS=Mus musculus GN=Numa1 PE=2 SV=1 - [E9Q7G0_MOUSE]	22.30	1	19	33	66	0.96	0.99	0.96	0.92	0.99	1.09	1.21	1.24	1.23	1.12	1.13	1.08	1.05	235.49	5.87
O88665	Bromodomain-containing protein 7 OS=Mus musculus GN=Brd7 PE=1 SV=1 - [BRD7_MOUSE]	7.22	1	3	3	8	1.05	1.19	1.13	0.96	0.99	1.09	1.09	1.24	1.08	1.15	1.08	1.21	1.13	73.95	6.38
Q8BK30	NADH dehydrogenase [ubiquinone] flavoprotein 3, mitochondrial OS=Mus musculus GN=Ndufv3 PE=2 SV=1 - [NDUV3_MOUSE]	46.15	1	2	4	73	0.61	0.70	1.13	0.82	1.33	1.05	1.70	1.24	2.06	1.07	1.78	0.76	1.14	11.81	9.35
F7DBT7	Regulatory factor X-associated protein OS=Mus musculus GN=Rfxap PE=2 SV=1 - [F7DBT7_MOUSE]	11.26	2	2	2	4	1.16	0.96	0.82	1.05	0.90	1.13	0.97	1.24	1.06	1.23	1.06	1.26	1.08	24.74	5.41
Q91VC9	Growth hormone-inducible transmembrane protein OS=Mus musculus GN=Ghitm PE=2 SV=1 - [GHITM_MOUSE]	3.18	1	1	1	2	1.11	1.43	1.28	1.02	0.92	1.09	0.97	1.24	1.11	1.30	1.17	1.45	1.31	37.25	9.80
Q99L02	PAXIP1-associated glutamate-rich protein 1 OS=Mus musculus GN=PAGR1 PE=1 SV=1 - [PAGR1_MOUSE]	20.55	2	4	4	13	0.95	1.09	1.02	0.92	0.98	1.09	1.03	1.24	1.25	1.04	0.98	1.01	0.95	27.71	4.56
P84104-2	Isoform Short of Serine/arginine-rich splicing factor 3 OS=Mus musculus GN=Srsf3 - [SRSF3_MOUSE]	45.16	3	5	6	100	1.05	0.85	0.92	1.00	0.98	1.11	1.10	1.24	1.13	1.11	1.04	1.05	1.03	14.19	10.08
P46467	Vacuolar protein sorting-associated protein 4B OS=Mus musculus GN=Vps4b PE=1 SV=2 - [VPS4B_MOUSE]	16.22	1	5	6	7	0.99	0.99	0.95	1.11	1.06	1.16	1.11	1.24	1.18	1.02	1.04	0.93	0.97	49.39	7.11
Q8K409	DNA polymerase beta OS=Mus musculus GN=Polb PE=2 SV=3 - [DPOLB_MOUSE]	3.88	1	1	1	3	0.98	1.91	1.94	0.88	0.89	1.25	1.27	1.24	1.25	1.10	1.12	1.08	1.10	38.26	8.85
Q9D7I5	Phospholysine phosphohistidine inorganic pyrophosphate phosphatase OS=Mus musculus GN=Lhpp PE=2 SV=1 - [LHPP_MOUSE]	15.56	2	2	2	10	0.93	1.03	1.02	0.97	1.04	1.21	1.29	1.24	1.34	1.30	1.39	1.12	1.22	29.13	5.10
P28667	MARCKS-related protein OS=Mus musculus GN=Marcksl1 PE=1 SV=2 - [MRP_MOUSE]	52.50	1	6	6	67	1.23	1.00	0.78	1.02	0.80	1.31	1.06	1.24	0.98	1.15	0.88	1.52	1.08	20.15	4.61

Q05AH6-2	Isoform 2 of Uncharacterized protein C11orf84 homolog OS=Mus musculus - [CK084_MOUSE]	12.70	2	2	2	3	0.99	1.16	1.16	1.08	1.08	1.09	1.09	1.24	1.24	1.14	1.15	1.10	1.11	34.32	4.96
Q3V0Q7	Transmembrane protease serine 12 OS=Mus musculus GN=Tmprss12 PE=2 SV=1 - [TMPSC_MOUSE]	5.65	1	1	1	1	0.78	0.88	1.13	0.66	0.85	1.33	1.69	1.24	1.58	1.01	1.30	0.64	0.83	36.89	6.67
D3YYE1	Acidic leucine-rich nuclear phosphoprotein 32 family member A (Fragment) OS=Mus musculus GN=Anp32a PE=2 SV=1 - [D3YYE1_MOUSE]	57.07	6	9	12	145	1.00	0.90	0.87	0.93	0.93	1.19	1.20	1.24	1.17	1.16	1.10	1.05	1.05	22.94	4.37
Q3ULB0	Protein Rbm6 OS=Mus musculus GN=Rbm6 PE=2 SV=1 - [Q3ULB0_MOUSE]	12.47	1	10	11	18	1.06	1.01	0.93	1.00	0.93	1.25	1.18	1.24	1.17	1.10	1.06	1.03	0.97	113.60	6.20
A2RTL5	Arginine/serine-rich coiled-coil protein 2 OS=Mus musculus GN=Rsrc2 PE=2 SV=1 - [RSRC2_MOUSE]	11.17	3	4	4	15	1.05	1.11	1.08	1.12	1.03	1.16	1.11	1.24	1.18	1.19	1.12	1.18	1.10	43.85	11.46
O70209	PDZ and LIM domain protein 3 OS=Mus musculus GN=Pdlim3 PE=1 SV=1 - [PDLI3_MOUSE]	29.43	1	7	7	10	0.96	1.01	0.93	0.95	1.05	1.21	1.24	1.24	1.16	1.18	1.14	1.04	1.08	34.28	7.91
Q8BH48	Ubiquitin-associated protein 1 OS=Mus musculus GN=Ubap1 PE=1 SV=1 - [UBAP1_MOUSE]	37.45	3	13	14	27	1.03	0.93	0.87	0.94	0.92	1.20	1.18	1.24	1.17	1.11	1.08	1.01	1.08	54.99	5.07
Q61048	WW domain-binding protein 4 OS=Mus musculus GN=Wbp4 PE=1 SV=4 - [WBP4_MOUSE]	14.63	1	4	4	7	1.07	0.98	0.91	0.97	0.94	1.15	1.04	1.24	1.15	1.14	1.02	0.91	0.85	42.11	7.36
O35852	Preprodynorphin (Fragment) OS=Mus musculus GN=Pdyn PE=2 SV=1 - [O35852_MOUSE]	26.21	2	5	5	8	1.33	0.95	0.88	0.95	0.78	1.16	0.87	1.24	0.82	1.53	1.08	1.62	1.31	27.99	5.63
Q9WV69	Dematin OS=Mus musculus GN=Dmtn PE=1 SV=1 - [DEMA_MOUSE]	67.16	2	3	21	323	1.06	0.97	0.88	0.98	0.96	1.28	1.21	1.24	1.23	1.13	1.11	1.02	0.98	45.44	8.41
Q3TLD5	Unconventional prefoldin RPB5 interactor OS=Mus musculus GN=Uri1 PE=1 SV=2 - [RMP_MOUSE]	28.25	2	9	9	27	1.02	1.00	1.05	0.95	0.99	1.27	1.31	1.24	1.23	1.15	1.11	1.07	1.09	59.05	5.07
Q67FY2-2	Isoform 2 of B-cell CLL/lymphoma 9-like protein OS=Mus musculus GN=Bcl9l - [BCL9L_MOUSE]	1.85	2	2	2	4	0.99	1.28	1.29	0.87	0.88	1.33	1.34	1.24	1.25	1.17	1.19	1.16	1.18	152.54	7.78

P08030	Adenine phosphoribosyltransferase OS=Mus musculus GN=Aprt PE=2 SV=2 - [APT_MOUSE]	5.56	1	1	1	1	1.00	1.58	1.57	1.06	1.05	1.15	1.14	1.24	1.23	0.88	0.88	0.81	0.81	19.71	6.79
Q8K2Q0	COMM domain-containing protein 9 OS=Mus musculus GN=Commd9 PE=2 SV=3 - [COMD9_MOUSE]	6.06	1	1	1	1	1.20	1.27	1.05	1.32	1.09	0.87	0.72	1.24	1.03	1.18	0.98	1.02	0.85	21.84	5.82
P50153	Guanine nucleotide-binding protein G(I)/G(S)/G(O) subunit gamma-4 OS=Mus musculus GN=Gng4 PE=2 SV=1 - [GBG4_MOUSE]	66.67	1	4	4	23	1.31	1.00	0.82	1.02	0.82	1.50	1.10	1.24	0.96	1.40	1.06	1.44	1.11	8.40	7.08
Q5SVR5	E3 ubiquitin-protein ligase RNF130 OS=Mus musculus GN=Rnf130 PE=2 SV=1 - [Q5SVR5_MOUSE]	11.46	2	3	3	6	1.18	0.98	1.06	0.98	0.86	1.00	0.88	1.24	1.04	1.17	0.98	1.15	0.94	42.40	9.19
Q9R226	KH domain-containing, RNA-binding, signal transduction-associated protein 3 OS=Mus musculus GN=Khdrbs3 PE=1 SV=1 - [KHDR3_MOUSE]	23.70	1	9	10	64	0.92	0.96	0.99	0.94	1.07	1.25	1.36	1.24	1.42	1.15	1.23	1.04	1.10	38.78	8.10
Q9Z0H7	B-cell lymphoma/leukemia 10 OS=Mus musculus GN=Bcl10 PE=1 SV=1 - [BCL10_MOUSE]	8.15	1	2	2	6	0.81	0.87	1.17	0.74	0.99	1.05	1.39	1.24	1.47	0.94	1.19	0.98	1.21	25.93	6.55
D3YXL3	Uncharacterized protein OS=Mus musculus GN=Gm5830 PE=4 SV=1 - [D3YXL3_MOUSE]	29.52	2	1	2	56	0.83	0.90	1.09	1.09	1.32	1.08	1.29	1.24	1.49	1.15	1.39	0.93	1.12	11.69	6.06
B1AXP6-	Isoform 2 of Mitochondrial import receptor subunit TOM5 homolog OS=Mus musculus GN=Tomm5 - [TOM5_MOUSE]	18.60	4	1	1	6	1.24	1.19	0.96	1.01	0.87	1.36	1.10	1.24	1.08	1.23	0.99	1.23	1.11	5.11	9.52
P12787	Cytochrome c oxidase subunit 5A, mitochondrial OS=Mus musculus GN=Cox5a PE=1 SV=2 - [COX5A_MOUSE]	63.01	1	11	12	262	0.92	0.77	0.82	0.85	0.91	1.22	1.33	1.24	1.34	1.11	1.21	1.06	1.13	16.09	6.54
Q3U1T3	Breast cancer metastasis-suppressor 1-like protein OS=Mus musculus GN=Brms1l PE=2 SV=1 - [BRM1L_MOUSE]	2.48	1	1	1	1	0.92	1.03	1.11	0.81	0.88	1.15	1.24	1.24	1.34	1.11	1.20	1.07	1.16	37.63	5.15

P97927	Laminin subunit alpha-4 OS=Mus musculus GN=Lama4 PE=1 SV=2 - [LAMA4_MOUSE]	7.21	1	8	9	14	0.98	1.17	1.14	1.01	0.95	1.21	1.17	1.24	1.26	1.16	1.12	1.17	1.10	201.69	6.21
Q3UX37	Protein Plekhg1 OS=Mus musculus GN=Plekhg1 PE=2 SV=1 - [Q3UX37_MOUSE]	1.01	2	1	1	2	1.14	1.24	1.09	1.22	1.07	1.25	1.09	1.24	1.09	1.09	0.96	1.09	0.96	155.78	6.40
P54254	Ataxin-1 OS=Mus musculus GN=Atxn1 PE=1 SV=2 - [ATX1_MOUSE]	4.42	2	2	2	8	0.98	1.15	1.11	0.97	0.84	1.09	1.14	1.24	1.27	1.18	1.21	1.08	0.95	83.74	8.16
Q8CCJ4-2	Isoform 2 of APC membrane recruitment protein 2 OS=Mus musculus GN=Amer2 - [AMER2_MOUSE]	24.54	1	1	9	28	0.62	1.44	2.34	0.99	1.62	0.98	1.58	1.24	2.01	0.93	1.52	0.92	1.49	57.32	8.44
D3Z511	Protein eva-1 homolog A (Fragment) OS=Mus musculus GN=Eva1a PE=2 SV=1 - [D3Z511_MOUSE]	16.13	2	2	2	6	1.12	1.24	1.08	1.03	0.98	1.25	1.11	1.24	1.11	1.13	1.01	1.05	0.99	17.64	6.55
Q8CI95	Oxysterol-binding protein-related protein 11 OS=Mus musculus GN=Osbpl11 PE=1 SV=2 - [OSB11_MOUSE]	2.13	2	1	1	2	1.01	0.97	0.96	0.95	0.94	1.21	1.18	1.24	1.22	1.23	1.22	1.17	1.16	83.58	7.01
Q922P9	Putative oxidoreductase GLYR1 OS=Mus musculus GN=Glyr1 PE=2 SV=1 - [GLYR1_MOUSE]	4.40	2	2	2	4	1.14	0.95	0.83	1.08	0.95	0.94	0.82	1.24	1.09	1.12	0.98	1.03	0.90	59.68	9.22
P46656	Adrenodoxin, mitochondrial OS=Mus musculus GN=Fdx1 PE=2 SV=1 - [ADX_MOUSE]	25.00	1	4	4	5	0.98	0.96	0.93	0.93	0.94	1.10	1.17	1.24	1.20	1.04	1.02	0.94	0.91	20.11	5.62
Q3UY34	Uncharacterized protein C12orf43 homolog OS=Mus musculus PE=2 SV=1 - [CL043_MOUSE]	43.75	2	9	9	24	1.07	1.16	1.08	1.04	1.01	1.17	1.11	1.24	1.16	1.11	1.09	0.95	0.92	27.61	8.70
Q9DBX1	Regulator of cell cycle RGCC OS=Mus musculus GN=Rgcc PE=1 SV=1 - [RGCC_MOUSE]	21.90	1	2	2	4	0.96	1.07	1.11	0.86	0.89	1.05	1.08	1.24	1.29	1.09	1.14	1.02	1.06	14.71	4.84
P63254	Cysteine-rich protein 1 OS=Mus musculus GN=Crip1 PE=2 SV=2 - [CRIP1_MOUSE]	12.99	1	2	2	13	0.97	0.93	0.82	0.89	0.85	1.15	1.07	1.24	1.23	1.13	1.12	0.98	0.99	8.54	8.57
Q91XF0	Pyridoxine-5'-phosphate oxidase OS=Mus musculus GN=Pnpo PE=1 SV=1 - [PNPO_MOUSE]	27.59	2	6	6	20	1.02	1.01	1.05	0.94	1.00	1.06	1.11	1.24	1.09	1.17	1.00	1.13	1.20	30.09	8.22
D3YUW7	Cingulin OS=Mus musculus GN=Cgn PE=2 SV=1 - [D3YUW7_MOUSE]	5.66	4	5	5	9	1.16	1.16	0.94	1.03	0.89	1.33	1.01	1.24	1.06	1.36	1.17	1.30	1.04	135.66	5.86

Q8CI08	SLAIN motif-containing protein 2 OS=Mus musculus GN=Slain2 PE=1 SV=2 - [SLAI2_MOUSE]	21.34	2	8	8	16	1.05	0.88	0.91	0.93	0.98	1.21	1.15	1.24	1.23	1.22	1.15	1.04	0.99	62.34	9.47
P43275	Histone H1.1 OS=Mus musculus GN=Hist1h1a PE=1 SV=2 - [H11_MOUSE]	19.72	1	3	6	11	0.93	1.04	1.12	1.06	1.06	1.19	1.23	1.24	1.27	1.20	1.30	1.06	1.13	21.77	10.93
Q70IV5-2	Isoform 2 of Synemin OS=Mus musculus GN=Synm - [SYNEM_MOUSE]	13.58	3	14	15	32	1.07	1.23	1.02	0.88	0.85	1.26	1.10	1.24	1.12	1.23	1.13	1.17	1.07	140.78	5.15
Q8CCN1	NACHT, LRR and PYD domains-containing protein 10 OS=Mus musculus GN=Nlrp10 PE=1 SV=1 - [NAL10_MOUSE]	2.53	1	1	1	2	0.87	1.28	1.47	1.05	1.21	1.16	1.32	1.24	1.42	1.32	1.51	0.94	1.08	76.32	6.60
G3X8V5	RIKEN cDNA 2610206B13, isoform CRA_b OS=Mus musculus GN=Rnf219 PE=4 SV=1 - [G3X8V5_MOUSE]	6.79	4	4	4	6	1.04	0.99	0.94	0.93	0.94	1.25	1.11	1.24	1.15	1.11	0.99	0.96	0.92	79.93	6.33
Q3TRR0	Microtubule-associated protein 9 OS=Mus musculus GN=Map9 PE=2 SV=2 - [MAP9_MOUSE]	30.50	2	15	15	59	1.05	0.86	0.78	1.00	0.96	1.16	1.14	1.24	1.06	1.13	1.02	0.87	0.91	73.47	7.62
Q9DBR0	A-kinase anchor protein 8 OS=Mus musculus GN=Akap8 PE=1 SV=1 - [AKAP8_MOUSE]	10.19	1	7	7	18	1.03	1.04	1.00	0.99	0.95	1.26	1.23	1.25	1.16	1.13	1.10	1.08	1.08	76.25	5.14
Q569Z6	Thyroid hormone receptor-associated protein 3 OS=Mus musculus GN=Thrap3 PE=1 SV=1 - [TR150_MOUSE]	22.92	4	19	20	70	0.99	0.78	0.81	0.97	0.96	1.17	1.19	1.25	1.22	1.07	1.07	1.01	1.00	108.11	10.17
E0CZ61	Protein Gm16039 (Fragment) OS=Mus musculus GN=Gm16039 PE=2 SV=1 - [E0CZ61_MOUSE]	22.12	5	1	1	6	1.00	1.09	1.08	1.01	1.02	1.11	1.12	1.25	1.21	1.24	1.13	1.01	1.00	10.95	4.59
E0CXY9	Arginine vasopressin-induced protein 1 (Fragment) OS=Mus musculus GN=Avpi1 PE=2 SV=1 - [E0CXY9_MOUSE]	11.39	2	1	1	4	1.36	1.85	1.36	0.85	0.63	1.15	0.84	1.25	0.92	1.04	0.77	1.10	0.81	8.94	9.96
Q8BRV5	Uncharacterized protein KIAA1671 OS=Mus musculus GN=Kiaa1671 PE=2 SV=1 - [K1671_MOUSE]	20.45	1	5	5	19	0.93	1.02	1.03	0.98	1.04	1.22	1.22	1.25	1.34	1.15	1.25	1.00	1.02	34.65	6.09

Q9CY97-2	Isoform 2 of RNA polymerase II subunit A C-terminal domain phosphatase SSU72 OS=Mus musculus GN=Ssu72 - [SSU72_MOUSE]	36.26	2	5	5	13	0.92	0.96	1.06	0.80	0.88	1.23	1.35	1.25	1.39	1.04	1.13	1.13	1.27	20.85	6.98
F8VQC7	Kinectin OS=Mus musculus GN=Ktn1 PE=2 SV=1 - [F8VQC7_MOUSE]	44.69	3	5	48	122	0.97	0.83	0.84	0.94	0.93	1.24	1.32	1.25	1.24	1.17	1.23	1.06	1.11	152.45	5.90
P51885	Lumican OS=Mus musculus GN=Lum PE=1 SV=2 - [LUM_MOUSE]	11.83	1	3	3	4	1.05	0.99	0.94	0.79	0.75	2.00	1.89	1.25	1.18	1.50	1.35	1.18	1.12	38.24	6.43
O54965-2	Isoform 2 of E3 ubiquitin-protein ligase RNF13 OS=Mus musculus GN=Rnf13 - [RNF13_MOUSE]	11.57	2	2	2	6	0.95	1.04	1.09	0.96	1.01	1.19	1.21	1.25	1.37	1.18	1.27	1.13	1.17	30.23	6.30
D3YZZ4	TSC22 domain family protein 4 OS=Mus musculus GN=Tsc22d4 PE=2 SV=1 - [D3YZZ4_MOUSE]	32.74	3	3	5	20	1.05	1.21	1.08	0.99	0.89	1.14	0.99	1.25	1.12	1.18	1.12	1.32	1.19	17.57	8.51
Q0P5V2	Sine oculis-binding protein homolog OS=Mus musculus GN=Sobp PE=2 SV=1 - [SOBP_MOUSE]	4.40	1	3	3	11	1.04	1.11	0.99	1.10	1.04	1.18	1.07	1.25	1.13	0.95	0.91	0.90	0.88	91.73	7.97
Q62084	Protein phosphatase 1 regulatory subunit 14B OS=Mus musculus GN=Ppp1r14b PE=1 SV=2 - [PP14B_MOUSE]	40.14	1	4	4	10	1.01	0.87	1.11	0.83	0.92	0.97	0.95	1.25	1.32	0.89	0.99	1.01	1.04	15.95	4.86
Q8R3P8	Protein Rnf113a1 OS=Mus musculus GN=Rnf113a1 PE=2 SV=1 - [Q8R3P8_MOUSE]	24.05	1	6	6	10	1.00	0.85	0.99	1.02	1.19	1.29	1.16	1.25	1.20	1.14	1.11	0.88	1.00	38.42	7.06
Q9QXV0	ProSAAS OS=Mus musculus GN=Pcsk1n PE=1 SV=2 - [PCSK1_MOUSE]	45.74	1	9	9	107	1.58	1.20	0.76	0.97	0.68	1.24	0.80	1.25	0.84	1.54	0.97	1.54	0.97	27.25	5.85
D3Z229	Histone-lysine N-methyltransferase NSD2 (Fragment) OS=Mus musculus GN=Whsc1 PE=2 SV=1 - [D3Z229_MOUSE]	20.00	6	3	3	4	1.07	0.77	0.78	1.02	0.95	1.32	1.23	1.25	1.18	1.19	1.11	1.01	0.90	24.73	8.63
Q80U49	Centrosomal protein of 170 kDa protein B OS=Mus musculus GN=Cep170b PE=1 SV=2 - [C170B_MOUSE]	41.61	2	47	49	141	0.98	1.02	1.04	0.95	0.97	1.21	1.23	1.25	1.25	1.14	1.13	1.05	1.09	170.72	6.87
Q8CHD8	Rab11 family-interacting protein 3 OS=Mus musculus GN=Rab11fip3 PE=1 SV=2 - [RFIP3_MOUSE]	5.64	3	3	4	6	1.02	1.04	1.08	0.90	0.88	1.28	1.24	1.25	1.24	1.07	1.11	1.10	1.13	118.46	4.39

Q8VE99	Coiled-coil domain-containing protein 115 OS=Mus musculus GN=Ccdc115 PE=2 SV=1 - [CC115_MOUSE]	9.44	1	1	1	5	1.05	0.91	0.87	1.32	1.26	1.11	1.06	1.25	1.19	1.22	1.16	1.06	1.01	19.73	8.81
Q8CI51	PDZ and LIM domain protein 5 OS=Mus musculus GN=Pdlim5 PE=1 SV=4 - [PDLI5_MOUSE]	35.53	3	11	15	54	1.00	1.03	1.03	0.96	0.97	1.12	1.19	1.25	1.22	1.15	1.15	1.06	1.11	63.26	8.25
P33174	Chromosome-associated kinesin KIF4 OS=Mus musculus GN=Kif4 PE=1 SV=3 - [KIF4_MOUSE]	1.46	1	1	2	2	1.09	0.91	0.83	0.91	0.83	1.17	1.07	1.25	1.14	1.25	1.15	1.14	1.05	139.43	6.68
Q9QYJ3	DnaJ homolog subfamily B member 1 OS=Mus musculus GN=Dnajb1 PE=2 SV=3 - [DNJB1_MOUSE]	20.59	1	6	7	18	0.93	0.82	0.88	0.94	0.99	1.25	1.26	1.25	1.23	1.16	1.08	1.00	1.04	38.14	8.63
Q9CQU1	Microfibrillar-associated protein 1 OS=Mus musculus GN=Mfap1 PE=1 SV=1 - [MFAP1_MOUSE]	36.45	1	12	12	33	1.03	0.89	0.90	0.96	0.98	1.16	1.12	1.25	1.25	1.10	1.08	1.06	1.09	51.92	4.98
E9Q7M2	Protein Tsc22d2 OS=Mus musculus GN=Tsc22d2 PE=2 SV=1 - [E9Q7M2_MOUSE]	8.19	1	4	6	20	0.97	0.80	0.89	0.98	0.95	1.21	1.18	1.25	1.20	1.12	1.16	1.10	1.06	78.15	4.89
Q91XC8	Death-associated protein 1 OS=Mus musculus GN=Dap PE=1 SV=3 - [DAP1_MOUSE]	24.51	1	1	1	9	0.89	0.73	0.81	0.96	1.07	1.18	1.32	1.25	1.40	1.19	1.34	1.08	1.21	11.15	9.36
P32648	VIP peptides OS=Mus musculus GN=Vip PE=1 SV=1 - [VIP_MOUSE]	27.06	1	3	3	16	1.01	1.01	0.95	0.84	0.81	1.10	1.10	1.25	1.22	0.99	1.05	0.82	0.91	19.04	6.60
Q8C5W3	Tubulin-specific chaperone cofactor E-like protein OS=Mus musculus GN=Tbcel PE=1 SV=1 - [TBCEL_MOUSE]	3.77	2	1	2	2	0.76	0.76	0.99	0.86	1.12	1.08	1.41	1.25	1.63	1.02	1.33	1.14	1.49	48.00	5.53
Q9D1F5	UPF0544 protein C5orf45 homolog OS=Mus musculus PE=2 SV=1 - [CE045_MOUSE]	2.69	1	1	1	2	1.07	1.02	0.96	0.94	0.88	1.19	1.10	1.25	1.16	1.10	1.03	1.15	1.08	36.87	7.99
Q9JHJ0	Tropomodulin-3 OS=Mus musculus GN=Tmod3 PE=1 SV=1 - [TMOD3_MOUSE]	28.41	1	7	8	18	1.09	0.86	0.85	0.87	0.82	1.11	1.17	1.25	1.27	1.15	1.17	0.99	0.98	39.48	5.14
Q8R1B5	Complexin-3 OS=Mus musculus GN=Cplx3 PE=1 SV=1 - [CPLX3_MOUSE]	61.39	2	8	8	33	0.99	0.86	0.82	0.96	0.98	1.23	1.27	1.25	1.26	1.07	1.11	0.86	0.97	17.57	4.89
E9Q1M6	Protein Maskbp3 OS=Mus musculus GN=Ankhd1 PE=4 SV=1 - [E9Q1M6_MOUSE]	3.82	5	5	6	9	0.99	1.14	1.15	0.95	0.93	1.24	1.19	1.25	1.17	1.23	1.25	1.34	1.34	263.24	5.92

Q6PFD9	Nuclear pore complex protein Nup98-Nup96 OS=Mus musculus GN=Nup98 PE=1 SV=2 - [NUP98_MOUSE]	9.86	1	12	12	29	0.96	0.86	0.95	0.92	0.98	1.07	1.16	1.25	1.24	1.05	1.08	0.96	1.06	197.12	6.18
Q99J83	Autophagy protein 5 OS=Mus musculus GN=Atg5 PE=1 SV=1 - [ATG5_MOUSE]	5.45	1	1	1	2	0.90	1.01	1.12	1.08	1.20	1.20	1.33	1.25	1.39	1.06	1.18	1.13	1.26	32.38	5.91
Q9CZR8	Elongation factor Ts, mitochondrial OS=Mus musculus GN=Tsfm PE=2 SV=1 - [EFTS_MOUSE]	29.32	3	7	7	16	1.06	1.02	1.00	0.95	1.02	1.10	1.22	1.25	1.23	1.21	1.22	1.21	1.15	35.31	7.06
Q99KJ8	Dynactin subunit 2 OS=Mus musculus GN=Dctn2 PE=1 SV=3 - [DCTN2_MOUSE]	65.17	1	23	25	219	0.96	0.84	0.89	0.85	0.88	1.27	1.30	1.25	1.32	1.13	1.18	1.01	1.07	44.09	5.26
Q9QYS9-	Isoform 4 of Protein quaking OS=Mus musculus GN=Qki - [QKI_MOUSE]	38.56	8	11	11	35	0.96	0.90	0.98	0.92	1.00	1.23	1.27	1.25	1.29	1.19	1.20	1.14	1.19	35.11	7.43
Q9CQN3	Mitochondrial import receptor subunit TOM6 homolog OS=Mus musculus GN=Tomm6 PE=3 SV=1 - [TOM6_MOUSE]	41.89	1	2	2	5	1.00	1.28	1.23	1.03	1.03	1.14	1.12	1.25	1.25	1.26	1.21	1.12	1.13	7.86	4.89
Q9WVH4	Forkhead box protein O3 OS=Mus musculus GN=Foxo3 PE=1 SV=1 - [FOXO3_MOUSE]	5.51	2	2	2	3	0.85	0.80	0.94	1.05	1.23	1.49	1.74	1.25	1.47	1.14	1.34	1.25	1.47	71.02	5.12
Q5SFM8	RNA-binding protein 27 OS=Mus musculus GN=Rbm27 PE=2 SV=3 - [RBM27_MOUSE]	6.04	3	4	5	8	1.01	0.83	0.85	1.01	1.00	1.18	1.25	1.25	1.33	1.13	1.17	1.14	1.17	118.48	9.19
Q8K3D3-	Isoform 2 of DNA repair protein SWI5 homolog OS=Mus musculus GN=Swi5 - [SWI5_MOUSE]	42.15	3	4	4	18	1.00	0.91	0.85	0.98	1.01	1.23	1.23	1.25	1.19	1.09	1.09	0.99	0.99	13.82	5.17
A3KGQ6	Actin-related protein 2/3 complex subunit 5-like protein OS=Mus musculus GN=Arpc5l PE=2 SV=1 - [A3KGQ6_MOUSE]	76.47	2	14	14	55	0.95	0.87	0.90	0.89	0.96	1.17	1.33	1.25	1.26	1.14	1.21	1.07	1.07	17.10	6.80
Q3UHF7	Transcription factor HIVEP2 OS=Mus musculus GN=Hivep2 PE=1 SV=1 - [ZEP2_MOUSE]	3.58	1	5	5	8	1.12	0.83	0.82	1.03	1.00	1.10	1.05	1.25	1.11	1.10	1.04	1.09	1.00	266.54	6.96
Q61206	Platelet-activating factor acetylhydrolase IB subunit beta OS=Mus musculus GN=Pafah1b2 PE=1 SV=2 - [PA1B2_MOUSE]	64.19	1	9	9	48	1.01	0.90	0.89	0.93	0.88	1.18	1.15	1.25	1.21	1.17	1.15	1.16	1.15	25.57	5.92

Q8CFC7-2	Isoform 2 of CLK4-associating serine/arginine rich protein OS=Mus musculus GN=Clasrp - [CLASR_MOUSE]	2.38	3	1	1	2	1.12	1.17	1.05	0.91	0.82	1.04	0.93	1.25	1.12	1.13	1.01	1.15	1.03	65.93	9.98
E9Q7P2	Protein Cacna1i OS=Mus musculus GN=Cacna1i PE=2 SV=1 - [E9Q7P2_MOUSE]	2.50	1	2	2	5	0.87	1.29	1.48	0.99	1.15	1.09	1.25	1.25	1.44	1.12	1.29	1.20	1.38	242.74	6.47
Q91YL2	E3 ubiquitin-protein ligase RNF126 OS=Mus musculus GN=Rnf126 PE=1 SV=1 - [RN126_MOUSE]	13.10	1	3	3	6	1.12	1.43	1.16	0.92	0.75	1.19	1.04	1.25	1.14	1.05	0.86	1.01	0.90	34.06	5.17
B1AZR7	Protein Pcdh11x OS=Mus musculus GN=Pcdh11x PE=2 SV=1 - [B1AZR7_MOUSE]	1.74	2	2	2	3	1.06	1.13	1.06	1.11	1.04	1.23	1.15	1.25	1.18	1.37	1.29	1.37	1.30	145.30	5.30
A2AVR1	Tetratricopeptide repeat protein 4 (Fragment) OS=Mus musculus GN=Ttc4 PE=2 SV=1 - [A2AVR1_MOUSE]	4.15	3	1	1	4	1.78	1.34	0.75	1.08	0.61	1.12	0.63	1.25	1.15	0.52	0.31	1.13	0.64	27.84	5.52
Q6ZWR6-	Isoform 2 of Nesprin-1 OS=Mus musculus GN=Syne1 - [SYNE1_MOUSE]	5.35	1	1	3	6	0.98	1.21	1.24	0.98	1.00	1.31	1.34	1.25	1.28	0.91	0.94	1.01	1.04	111.26	5.35
Q6R891	Neurabin-2 OS=Mus musculus GN=Ppp1r9b PE=1 SV=1 - [NEB2_MOUSE]	52.51	2	33	35	270	1.00	0.95	0.98	0.91	0.90	1.31	1.26	1.25	1.21	1.12	1.11	1.07	1.05	89.47	4.92
Q9Z2I2	Peptidyl-prolyl cis-trans isomerase FKBP1B OS=Mus musculus GN=Fkbp1b PE=1 SV=3 - [FKB1B_MOUSE]	28.70	1	2	2	18	0.96	0.89	0.92	0.96	0.95	1.35	1.38	1.25	1.39	1.13	1.18	0.95	1.02	11.79	8.47
Q99LJ0	CTTNBP2 N-terminal-like protein OS=Mus musculus GN=Cttnbp2nl PE=1 SV=1 - [CT2NL_MOUSE]	26.49	1	12	13	27	1.03	0.87	0.88	0.95	0.88	1.21	1.18	1.25	1.12	1.11	1.07	1.08	1.07	69.80	7.71
Q8BKR5	Protein phosphatase 1 regulatory subunit 37 OS=Mus musculus GN=Ppp1r37 PE=2 SV=1 - [PPR37_MOUSE]	6.04	1	2	3	5	1.55	1.27	0.82	1.27	0.82	1.18	0.76	1.25	0.81	1.07	0.69	1.94	1.25	77.53	5.06
Q8JZL3	Thiamine-triphosphatase OS=Mus musculus GN=Thtpa PE=1 SV=3 - [THTPA_MOUSE]	16.96	1	3	3	6	1.00	0.79	0.72	0.92	0.93	0.88	0.92	1.25	1.20	1.01	1.15	0.94	1.04	24.25	4.72
Q05BE0	Zc4h2 protein OS=Mus musculus GN=Zc4h2 PE=2 SV=1 - [Q05BE0_MOUSE]	11.73	3	3	3	6	0.85	1.00	1.22	1.04	0.88	1.36	1.45	1.25	1.48	1.22	1.22	1.10	1.24	23.05	7.18

F6ZIA4	Myelin basic protein (Fragment) OS=Mus musculus GN=Mbp PE=2 SV=1 - [F6ZIA4_MOUSE]	76.43	3	3	21	1034	1.16	0.59	0.53	0.81	0.85	1.32	1.13	1.25	1.08	0.82	0.66	1.12	0.93	17.10	10.87
Q6PIU9	Uncharacterized protein FLJ45252 homolog OS=Mus musculus PE=1 SV=2 - [YJ005_MOUSE]	22.03	1	5	5	12	1.07	1.17	1.06	0.97	0.89	1.26	1.11	1.25	1.14	1.11	1.08	1.08	1.05	37.68	5.07
Q91W45	Polyadenylate-binding protein-interacting protein 2B OS=Mus musculus GN=Paip2b PE=2 SV=2 - [PAI2B_MOUSE]	57.35	1	5	5	12	1.09	0.98	0.78	1.07	0.90	1.25	1.07	1.25	1.31	1.10	1.01	1.30	1.17	15.46	4.32
Q9CWZ7	Gamma-soluble NSF attachment protein OS=Mus musculus GN=Napg PE=1 SV=1 - [SNAG_MOUSE]	66.99	2	22	22	264	0.95	0.87	0.94	0.86	0.90	1.23	1.30	1.25	1.29	1.13	1.17	1.04	1.10	34.71	5.41
Q6PEV3	WAS/WASL-interacting protein family member 2 OS=Mus musculus GN=Wipf2 PE=2 SV=1 - [WIPF2_MOUSE]	49.77	2	14	14	87	1.02	0.94	0.88	0.95	0.91	1.20	1.16	1.25	1.19	1.11	1.05	1.01	0.99	46.27	10.99
F6XIX0	Synaptojanin-1 (Fragment) OS=Mus musculus GN=Synj1 PE=2 SV=1 - [F6XIX0_MOUSE]	93.20	1	1	4	14	1.28	1.56	1.22	1.03	0.81	1.37	1.07	1.25	0.98	1.33	1.04	10.01	7.84	10.79	8.19
Q8VDD8	WAS protein family homolog 1 OS=Mus musculus GN=Wash1 PE=2 SV=1 - [WASH1_MOUSE]	8.21	1	3	3	6	0.99	0.72	0.72	0.91	0.92	1.44	1.37	1.25	1.20	1.23	1.19	1.12	1.08	51.63	5.44
Q71FD5	E3 ubiquitin-protein ligase ZNRF2 OS=Mus musculus GN=Znrf2 PE=1 SV=1 - [ZNRF2_MOUSE]	35.71	7	6	6	14	1.16	0.99	0.76	0.98	0.87	1.34	1.05	1.25	1.04	1.20	0.92	1.14	0.95	23.69	6.76
Q80Y55	BSD domain-containing protein 1 OS=Mus musculus GN=Bsdc1 PE=2 SV=1 - [BSDC1_MOUSE]	38.41	1	11	11	29	1.09	1.07	0.96	1.04	0.89	1.26	1.12	1.25	1.15	1.17	1.09	1.14	1.08	46.92	4.40
Q8C739	Protein FAM110B OS=Mus musculus GN=Fam110b PE=2 SV=1 - [F110B_MOUSE]	19.40	1	5	5	11	1.11	1.03	0.89	0.92	0.91	1.16	1.11	1.26	1.17	1.15	1.10	1.15	1.06	40.34	9.23
Q9CQU5	ZW10 interactor OS=Mus musculus GN=Zwint PE=2 SV=1 - [ZWINT_MOUSE]	27.38	1	5	6	15	1.16	0.92	0.79	0.98	0.86	1.25	1.13	1.26	1.04	1.18	1.06	1.08	0.96	28.70	8.43
Q9CXP8	Guanine nucleotide-binding protein G(I)/G(S)/G(O) subunit gamma-10 OS=Mus musculus GN=Gng10 PE=3 SV=1 - [GBG10_MOUSE]	30.88	2	1	2	9	1.01	1.16	1.14	1.09	1.07	1.39	1.37	1.26	1.24	1.31	1.30	1.43	1.42	7.22	7.85

B1AXP3	MCG1697, isoform CRA_a OS=Mus musculus GN=Fam154a PE=4 SV=1 - [B1AXP3_MOUSE]	3.57	1	1	1	1	0.73	0.85	1.17	0.48	0.67	1.42	1.94	1.26	1.73	0.83	1.14	0.54	0.75	54.75	8.82
Q9CR27	WASH complex subunit CCDC53 OS=Mus musculus GN=Ccdc53 PE=2 SV=1 - [CCD53_MOUSE]	23.20	2	3	3	12	0.97	1.07	0.99	0.90	0.92	1.10	1.28	1.26	1.36	1.19	1.17	1.11	1.12	21.08	4.46
P56942	Pro-MCH OS=Mus musculus GN=Pmch PE=1 SV=2 - [MCH_MOUSE]	27.88	1	4	4	29	1.28	1.03	0.81	1.08	0.81	1.30	1.01	1.26	0.93	1.35	1.02	1.34	1.02	18.50	7.14
E2QRQ3	Double-stranded RNA-binding protein Staufen homolog 2 OS=Mus musculus GN=Stau2 PE=2 SV=1 - [E2QRQ3_MOUSE]	10.15	6	3	3	8	0.98	0.91	0.97	0.99	1.03	1.19	1.18	1.26	1.27	1.11	1.16	1.05	1.07	51.96	9.67
O88569	Heterogeneous nuclear ribonucleoproteins A2/B1 OS=Mus musculus GN=Hnrnpa2b1 PE=1 SV=2 - [ROA2_MOUSE]	66.01	3	24	29	753	1.01	0.92	0.91	0.91	0.89	1.19	1.15	1.26	1.22	1.12	1.09	1.10	1.08	37.38	8.95
Q8BR86-3	Isoform 3 of Kin of IRRE-like protein 3 OS=Mus musculus GN=Kirrel3 - [KIRR3_MOUSE]	1.47	5	1	1	1	1.24	1.11	0.89	0.83	0.67	0.99	0.79	1.26	1.01	1.08	0.87	1.09	0.88	67.05	7.53
M0QWR6	Mitochondrial import receptor subunit TOM40B OS=Mus musculus GN=Tomm40l PE=4 SV=1 - [M0QWR6_MOUSE]	32.65	5	1	1	2	1.23	0.63	0.51	1.53	1.24	1.43	1.15	1.26	1.01	1.47	1.20	1.47	1.19	5.37	7.36
Q6PDC0	RUN domain-containing protein 3B OS=Mus musculus GN=Rundc3b PE=2 SV=1 - [RUN3B_MOUSE]	13.48	2	4	4	7	1.33	1.39	1.00	1.39	1.00	1.16	0.86	1.26	1.00	1.18	0.98	1.25	1.04	45.11	5.17
O70200	Allograft inflammatory factor 1 OS=Mus musculus GN=Aif1 PE=1 SV=1 - [AIF1_MOUSE]	23.81	2	3	3	6	1.00	0.79	0.74	0.83	0.83	1.32	1.31	1.26	1.22	1.13	1.15	1.14	1.14	16.90	8.76
A2TJV2	Paralemmin-3 OS=Mus musculus GN=Palm3 PE=2 SV=1 - [PALM3_MOUSE]	18.80	1	10	10	29	1.14	1.09	1.01	0.96	0.84	1.24	1.09	1.26	1.12	1.04	0.97	1.12	1.00	78.74	4.50
P10107	Annexin A1 OS=Mus musculus GN=Anxa1 PE=1 SV=2 - [ANXA1_MOUSE]	8.38	1	2	2	3	1.10	1.18	1.07	1.12	1.02	1.24	1.13	1.26	1.14	1.20	1.09	1.13	1.03	38.71	7.37
Q91VM5	RNA binding motif protein, X-linked-like-1 OS=Mus musculus GN=Rbmxl1 PE=1 SV=1 - [RMXL1_MOUSE]	42.53	1	5	17	93	0.94	0.93	0.97	0.86	0.93	1.22	1.27	1.26	1.34	1.16	1.20	1.17	1.24	42.14	9.99

P62900	60S ribosomal protein L31 OS=Mus musculus GN=Rpl31 PE=2 SV=1 - [RL31_MOUSE]	32.80	1	4	4	22	1.05	1.06	0.97	1.05	0.99	1.06	1.03	1.26	1.10	1.03	1.03	1.10	1.02	14.45	10.54
Q62433	Protein NDRG1 OS=Mus musculus GN=Ndrg1 PE=1 SV=1 - [NDRG1_MOUSE]	34.01	6	9	9	69	1.03	1.02	1.05	1.06	1.01	1.22	1.17	1.26	1.23	1.22	1.21	1.23	1.19	42.98	6.10
Q810C0	SLIT and NTRK-like protein 2 OS=Mus musculus GN=Slitrk2 PE=1 SV=1 - [SLIK2_MOUSE]	4.02	1	3	3	5	1.04	1.06	0.87	1.01	0.96	1.13	0.93	1.26	1.14	1.19	1.00	1.11	0.78	95.38	7.65
Q80X59	Transmembrane and coiled-coil domain-containing protein 5B OS=Mus musculus GN=Tmco5b PE=2 SV=1 - [TMC5B_MOUSE]	3.26	1	1	1	1	1.15	0.81	0.70	0.95	0.82	1.29	1.11	1.26	1.09	1.16	1.01	1.12	0.98	36.04	6.32
P32020-2	Isoform SCP2 of Non-specific lipid-transfer protein OS=Mus musculus GN=Scp2 - [NLTP_MOUSE]	63.64	2	11	11	44	1.00	0.79	0.79	0.85	0.86	1.28	1.24	1.26	1.27	1.11	1.09	1.12	1.12	15.23	9.31
Q8BP27-2	Isoform 2 of Swi5-dependent recombination DNA repair protein 1 homolog OS=Mus musculus GN=Sfr1 - [SFR1_MOUSE]	36.30	3	7	7	22	0.99	0.97	1.11	0.85	0.96	1.20	1.25	1.26	1.32	1.09	1.14	0.92	1.05	33.51	5.14
P35459	Lymphocyte antigen 6D OS=Mus musculus GN=Ly6d PE=1 SV=1 - [LY6D_MOUSE]	11.02	1	1	1	2	0.87	1.17	1.35	0.88	1.01	1.33	1.53	1.26	1.45	1.18	1.36	1.08	1.24	13.39	7.50
E9Q1K7	Regulator of G-protein-signaling 12 OS=Mus musculus GN=Rgs12 PE=2 SV=1 - [E9Q1K7_MOUSE]	7.33	9	3	3	6	0.97	0.82	0.87	1.01	1.04	1.11	1.13	1.26	1.33	1.05	1.12	1.06	1.10	78.56	8.21
Q9DAI6	Protein FAM135B OS=Mus musculus GN=Fam135b PE=2 SV=3 - [F135B_MOUSE]	0.78	1	1	1	2	0.97	1.17	1.21	1.05	1.09	1.07	1.10	1.26	1.30	1.18	1.22	1.13	1.17	155.43	6.02
Q9WTX5	S-phase kinase-associated protein 1 OS=Mus musculus GN=Skp1 PE=1 SV=3 - [SKP1_MOUSE]	84.05	3	16	16	206	0.97	0.80	0.84	0.92	0.94	1.24	1.30	1.26	1.29	1.12	1.13	1.01	1.03	18.66	4.54
B1AXR5	Perilipin-2 (Fragment) OS=Mus musculus GN=Plin2 PE=4 SV=1 - [B1AXR5_MOUSE]	8.16	2	1	1	1	1.30	0.75	0.57	1.26	0.97	1.09	0.84	1.26	0.97	1.17	0.90	0.63	0.49	20.68	5.34
Q5SSZ5	Tensin-3 OS=Mus musculus GN=Tns3 PE=1 SV=1 - [TENS3_MOUSE]	4.93	2	3	4	8	1.05	1.00	0.97	1.23	1.11	1.24	1.19	1.26	1.22	1.19	1.16	1.25	1.16	155.49	6.65

F6XVP7	E3 ubiquitin-protein ligase RNF31 (Fragment) OS=Mus musculus GN=Rnf31 PE=4 SV=1 - [F6XVP7_MOUSE]	4.06	3	2	2	4	1.08	1.33	1.23	1.02	0.94	1.15	1.06	1.26	1.16	1.22	1.13	1.50	1.39	102.05	6.77
Q9Z1Y4	Thyroid receptor-interacting protein 6 OS=Mus musculus GN=Trip6 PE=1 SV=1 - [TRIP6_MOUSE]	8.33	1	2	2	4	0.83	1.01	1.21	0.92	1.10	1.13	1.34	1.26	1.51	1.30	1.56	1.14	1.36	50.90	7.31
A8Y5P4	MAP7 domain-containing protein 1 OS=Mus musculus GN=Map7d1 PE=2 SV=1 - [A8Y5P4_MOUSE]	24.32	5	17	18	45	1.03	0.86	0.86	0.97	0.96	1.21	1.15	1.26	1.16	1.12	1.08	1.01	1.00	90.04	10.04
Q9JI44	DNA methyltransferase 1-associated protein 1 OS=Mus musculus GN=Dmap1 PE=1 SV=1 - [DMAP1_MOUSE]	8.76	2	3	3	6	0.96	1.02	0.99	0.98	1.03	1.15	1.11	1.26	1.42	1.14	1.20	1.09	1.08	53.10	9.50
O08796	Eukaryotic elongation factor 2 kinase OS=Mus musculus GN=Eef2k PE=1 SV=1 - [EF2K_MOUSE]	2.35	1	1	1	1	0.89	0.95	1.07	0.99	1.11	1.11	1.24	1.26	1.41	0.95	1.07	0.85	0.96	81.69	5.38
A2AJ72	MCG130458 OS=Mus musculus GN=Fubp3 PE=4 SV=1 - [A2AJ72_MOUSE]	8.44	3	3	5	8	0.93	0.88	0.94	0.90	0.96	1.32	1.40	1.26	1.35	1.10	1.18	1.06	1.13	61.41	8.28
P59759	MKL/myocardin-like protein 2 OS=Mus musculus GN=Mkl2 PE=1 SV=1 - [MKL2_MOUSE]	33.52	12	22	27	65	1.08	0.89	0.87	1.04	0.99	1.34	1.27	1.26	1.14	1.19	1.08	1.11	1.07	117.47	6.16
Q6PFX2	BEN domain-containing protein 6 OS=Mus musculus GN=Bend6 PE=2 SV=1 - [BEND6_MOUSE]	12.81	3	3	3	9	0.93	0.87	1.00	0.81	0.94	1.25	1.47	1.26	1.49	1.15	1.32	0.99	1.15	31.25	8.92
Q8CB27	Ubiquitin thioesterase OTU1 OS=Mus musculus GN=Yod1 PE=1 SV=1 - [OTU1_MOUSE]	7.29	1	1	1	2	1.34	1.13	0.84	1.24	0.92	1.27	0.94	1.26	0.94	1.15	0.86	1.30	0.97	37.46	5.76
F8VQK5	SAM and SH3 domain-containing protein 1 OS=Mus musculus GN=Sash1 PE=2 SV=1 - [F8VQK5_MOUSE]	25.69	4	23	23	62	1.08	0.99	0.92	0.99	0.90	1.16	1.06	1.26	1.12	1.20	1.08	1.16	1.09	135.54	6.20
A2AQP0	Myosin-7B OS=Mus musculus GN=Myh7b PE=3 SV=1 - [MYH7B_MOUSE]	4.95	1	2	10	24	1.19	0.96	0.80	1.04	0.88	1.29	1.08	1.26	1.06	1.19	1.00	1.09	0.92	221.36	5.88

Q921P9	Transcription elongation factor A protein-like 1 OS=Mus musculus GN=Tceal1 PE=2 SV=1 - [TCAL1_MOUSE]	37.58	1	6	6	17	1.13	0.97	0.83	1.06	0.90	1.16	1.00	1.26	1.05	1.14	0.97	1.02	1.05	19.30	4.54
Q91WN1	DnaJ homolog subfamily C member 9 OS=Mus musculus GN=Dnajc9 PE=2 SV=2 - [DNJC9_MOUSE]	47.10	1	13	13	33	1.09	0.86	0.82	1.01	0.93	1.24	1.14	1.26	1.10	1.20	1.10	1.11	0.97	30.04	5.94
Q8R0A0	General transcription factor IIF subunit 2 OS=Mus musculus GN=Gtf2f2 PE=1 SV=1 - [T2FB_MOUSE]	49.80	1	10	10	32	1.00	0.95	0.94	0.94	0.97	1.23	1.26	1.26	1.24	1.16	1.12	0.98	1.12	28.36	9.22
A2A9P8	E3 ubiquitin-protein ligase MIB2 (Fragment) OS=Mus musculus GN=Mib2 PE=2 SV=1 - [A2A9P8_MOUSE]	4.80	3	1	1	1	1.36	1.64	1.20	1.22	0.90	0.76	0.56	1.26	0.93	1.32	0.97	1.82	1.34	41.14	8.63
Q9JIX0	Enhancer of yellow 2 transcription factor homolog OS=Mus musculus GN=Eny2 PE=2 SV=1 - [ENY2_MOUSE]	24.75	1	2	2	6	1.01	0.86	0.88	1.00	0.97	1.27	1.18	1.26	1.20	1.19	1.18	1.07	1.08	11.52	9.33
J3KMN0	Protein Kmt2d OS=Mus musculus GN=Kmt2d PE=4 SV=1 - [J3KMN0_MOUSE]	1.74	2	5	6	9	1.11	1.20	1.08	1.04	0.94	1.28	1.16	1.26	1.18	1.25	1.12	1.34	1.21	599.93	5.78
Q8BL65	Actin-binding LIM protein 2 OS=Mus musculus GN=Ablim2 PE=1 SV=1 - [ABLM2_MOUSE]	54.08	6	2	26	193	1.00	1.06	1.05	0.95	0.91	1.39	1.18	1.26	1.26	1.04	1.06	1.01	1.00	68.06	8.02
Q62425	NADH dehydrogenase [ubiquinone] 1 alpha subcomplex subunit 4 OS=Mus musculus GN=Ndufa4 PE=1 SV=2 - [NDUA4_MOUSE]	47.56	1	5	5	29	1.07	0.95	0.94	1.10	1.03	1.22	1.19	1.26	1.23	1.23	1.17	1.16	1.10	9.32	9.52
Q8R2U4	N-terminal Xaa-Pro-Lys N-methyltransferase 1 OS=Mus musculus GN=Ntmt1 PE=2 SV=3 - [NTM1A_MOUSE]	8.52	1	1	1	1	1.38	1.68	1.22	1.04	0.75	1.27	0.92	1.26	0.91	1.21	0.88	0.95	0.69	25.40	6.90
P26350	Prothymosin alpha OS=Mus musculus GN=Ptma PE=1 SV=2 - [PTMA_MOUSE]	23.42	2	5	5	90	0.96	0.76	0.80	0.99	0.97	1.30	1.23	1.26	1.25	1.11	1.14	1.06	1.03	12.25	3.79
O55176	E3 ubiquitin-protein ligase Praja-1 OS=Mus musculus GN=Pja1 PE=1 SV=3 - [PJA1_MOUSE]	26.82	6	8	9	31	1.10	1.11	0.97	1.05	0.94	1.34	1.18	1.26	1.13	1.10	1.03	1.09	0.94	63.87	4.97

Q9EQC8	Papillary renal cell carcinoma (Translocation-associated) OS=Mus musculus GN=Prcc PE=2 SV=1 - [Q9EQC8_MOUSE]	11.00	1	5	5	11	1.06	0.90	0.81	0.98	0.90	1.27	1.26	1.27	1.09	1.09	1.05	1.02	0.86	52.27	4.94
Q9CQ13	Coordinator of PRMT5 and differentiation stimulator OS=Mus musculus GN=Coprs PE=1 SV=1 - [COPRS_MOUSE]	24.86	1	3	3	5	1.14	1.83	1.52	1.01	0.89	1.11	0.96	1.27	1.17	1.22	1.03	1.20	1.02	18.65	4.08
A7YQ68	Prickle-like 2 (Drosophila) OS=Mus musculus GN=Prickle2 PE=2 SV=1 - [A7YQ68_MOUSE]	37.18	2	23	23	99	1.12	1.17	0.96	1.04	0.95	1.19	1.05	1.27	1.08	1.16	1.04	1.22	1.06	102.17	7.80
K3W4N2	Interleukin-18 OS=Mus musculus GN=Il18 PE=3 SV=1 - [K3W4N2_MOUSE]	16.15	2	2	2	3	0.92	0.77	0.84	0.79	0.86	0.97	1.05	1.27	1.37	0.97	1.06	0.99	1.07	22.14	4.91
D3Z5K6	Mitochondrial import inner membrane translocase subunit TIM14 OS=Mus musculus GN=Dnajc19 PE=2 SV=1 - [D3Z5K6_MOUSE]	29.09	5	2	3	6	1.04	0.60	0.58	0.92	0.88	1.25	1.19	1.27	1.21	1.07	1.02	0.91	0.87	12.18	10.10
Q3U5C7	Prickle-like protein 1 OS=Mus musculus GN=Prickle1 PE=1 SV=1 - [PRIC1_MOUSE]	7.09	1	5	5	7	1.00	1.19	1.12	1.15	1.11	1.25	1.20	1.27	1.29	1.04	1.11	0.87	1.02	94.07	6.28
P60879	Synaptosomal-associated protein 25 OS=Mus musculus GN=Snap25 PE=1 SV=1 - [SNP25_MOUSE]	77.67	1	5	23	1226	0.99	1.06	1.08	0.94	0.95	1.23	1.26	1.27	1.28	1.16	1.16	1.08	1.09	23.30	4.77
E0CYH0	MCG16685, isoform CRA_d OS=Mus musculus GN=Wtap PE=4 SV=1 - [E0CYH0_MOUSE]	20.71	5	6	6	11	0.93	0.88	0.97	0.85	0.91	1.32	1.42	1.27	1.36	1.18	1.32	0.98	1.07	44.18	5.25
Q99MU3-	Isoform 5 of Double-stranded RNA-specific adenosine deaminase OS=Mus musculus GN=Adar - [DSRAD_MOUSE]	4.95	5	4	4	5	0.98	1.01	1.03	0.98	0.98	1.22	1.27	1.27	1.25	1.14	1.12	1.11	1.09	102.51	8.78
E9Q8A6	Glucocorticoid receptor (Fragment) OS=Mus musculus GN=Nr3c1 PE=2 SV=1 - [E9Q8A6_MOUSE]	13.08	8	1	1	2	0.90	0.68	0.75	0.97	1.08	1.03	1.14	1.27	1.41	1.06	1.19	1.23	1.37	13.90	8.44
Q8VCF0	Mitochondrial antiviral-signaling protein OS=Mus musculus GN=Mavs PE=1 SV=1 - [MAVS_MOUSE]	5.17	1	2	2	7	1.04	1.04	0.99	1.00	1.01	1.25	1.19	1.27	1.22	1.28	1.23	1.11	1.09	53.37	6.37

Q78ZA7	Nucleosome assembly protein 1-like 4 OS=Mus musculus GN=Nap1l4 PE=1 SV=1 - [NP1L4_MOUSE]	56.00	1	13	14	105	1.03	0.94	0.94	0.88	0.92	1.21	1.16	1.27	1.22	1.14	1.13	1.01	1.03	42.65	4.67
Q91WE2	Protein FAM192A OS=Mus musculus GN=Fam192a PE=2 SV=1 - [F192A_MOUSE]	13.39	1	3	4	27	1.02	0.89	0.89	1.00	0.94	1.24	1.19	1.27	1.23	1.18	1.06	1.02	0.99	28.68	5.14
P46660	Alpha-internexin OS=Mus musculus GN=Ina PE=1 SV=2 - [AINX_MOUSE]	69.05	1	30	35	775	0.92	1.02	1.07	0.95	1.02	1.16	1.28	1.27	1.35	1.07	1.18	1.06	1.16	55.71	5.27
G5E8C8	Protein Zfp612 OS=Mus musculus GN=Zfp612 PE=4 SV=1 - [G5E8C8_MOUSE]	2.83	152	1	2	3	1.14	0.90	0.79	0.99	0.87	1.52	1.33	1.27	1.11	1.31	1.15	1.37	1.21	76.08	8.41
O08915	AH receptor-interacting protein OS=Mus musculus GN=Aip PE=1 SV=1 - [AIP_MOUSE]	33.64	2	8	8	13	0.92	0.86	0.93	0.87	0.95	1.20	1.31	1.27	1.37	1.07	1.17	1.01	1.06	37.58	6.40
P97855	Ras GTPase-activating protein-binding protein 1 OS=Mus musculus GN=G3bp1 PE=1 SV=1 - [G3BP1_MOUSE]	36.34	1	12	12	27	1.04	1.00	0.97	1.02	0.95	1.31	1.22	1.27	1.25	1.14	1.19	1.09	1.05	51.80	5.59
Q8BFR4	N-acetylglucosamine-6-sulfatase OS=Mus musculus GN=Gns PE=2 SV=1 - [GNS_MOUSE]	10.85	1	5	5	13	0.96	0.87	0.87	0.89	0.97	1.17	1.12	1.27	1.37	1.14	1.25	1.04	1.08	61.14	8.24
Q10470	Beta-1,4-mannosyl-glycoprotein 4-beta-N-acetylglucosaminyltransferase OS=Mus musculus GN=Mgat3 PE=1 SV=2 - [MGAT3_MOUSE]	2.79	2	1	1	2	1.17	0.96	0.83	0.91	0.78	1.11	0.94	1.27	1.08	0.90	0.78	1.01	0.87	61.96	8.15
P20152	Vimentin OS=Mus musculus GN=Vim PE=1 SV=3 - [VIME_MOUSE]	72.75	2	24	32	170	1.05	0.95	0.95	0.88	0.85	1.14	1.11	1.27	1.20	1.71	1.63	1.10	1.04	53.66	5.12
A2ABG4	Protein Tbc1d16 OS=Mus musculus GN=Tbc1d16 PE=2 SV=1 - [A2ABG4_MOUSE]	2.74	1	1	1	1	1.20	2.36	1.97	1.17	0.98	1.22	1.02	1.27	1.06	1.26	1.06	1.43	1.20	86.48	5.58
F7D1Q2	Protein Tmem44 (Fragment) OS=Mus musculus GN=Tmem44 PE=4 SV=1 - [F7D1Q2_MOUSE]	4.74	2	1	1	1	1.06	1.11	1.05	1.00	0.94	1.58	1.49	1.27	1.19	1.10	1.04	1.03	0.97	23.40	5.94
Q8R1N0	Zinc finger protein 830 OS=Mus musculus GN=Znf830 PE=1 SV=1 - [ZN830_MOUSE]	34.44	1	7	7	17	1.08	0.80	0.77	1.02	0.94	1.23	1.12	1.27	1.12	1.14	1.00	1.07	0.97	40.63	5.40
Q3TCX3-2	Isoform 2 of UPF0469 protein KIAA0907 OS=Mus musculus GN=Kiaa0907 - [K0907_MOUSE]	1.85	2	1	1	1	1.36	1.44	1.05	1.30	0.95	1.32	0.96	1.27	0.93	1.02	0.75	0.92	0.68	51.17	9.23

Q9D172	ES1 protein homolog, mitochondrial OS=Mus musculus GN=D10Jhu81e PE=1 SV=1 - [ES1_MOUSE]	52.26	1	10	10	59	0.85	0.79	0.94	0.92	1.06	1.18	1.43	1.27	1.52	1.17	1.39	1.11	1.32	28.07	8.78
Q5U4C3	Splicing factor, arginine/serine-rich 19 OS=Mus musculus GN=Scaf1 PE=1 SV=1 - [SFR19_MOUSE]	3.03	1	3	3	10	1.02	0.90	0.90	1.01	0.97	1.13	1.23	1.27	1.29	1.16	1.14	1.02	1.07	133.76	9.45
Q69ZC8	Uncharacterized protein KIAA1704 OS=Mus musculus GN=Kiaa1704 PE=1 SV=2 - [K1704_MOUSE]	28.61	1	6	6	17	1.08	0.87	0.76	0.97	0.91	1.23	1.12	1.27	1.19	1.10	1.03	1.10	1.01	38.93	5.41
Q9JJZ6	Krueppel-like factor 13 OS=Mus musculus GN=Klf13 PE=2 SV=2 - [KLF13_MOUSE]	5.88	1	1	1	2	1.01	0.93	0.92	0.94	0.93	1.12	1.10	1.27	1.25	1.09	1.07	1.06	1.04	31.12	9.64
Q2VPQ9	Chromatin modification-related protein MEAF6 OS=Mus musculus GN=Meaf6 PE=2 SV=1 - [EAF6_MOUSE]	16.75	3	2	2	4	1.06	1.21	1.15	1.09	1.03	1.18	1.13	1.27	1.20	1.39	1.26	1.28	1.22	21.64	9.32
Q9R087	Glypican-6 OS=Mus musculus GN=Gpc6 PE=1 SV=1 - [GPC6_MOUSE]	17.66	4	4	6	12	1.06	1.23	1.01	0.91	0.95	1.25	1.25	1.27	1.20	1.12	1.03	1.08	0.95	63.02	5.43
P35831	Tyrosine-protein phosphatase non-receptor type 12 OS=Mus musculus GN=Ptpn12 PE=1 SV=3 - [PTN12_MOUSE]	1.03	1	1	1	1	1.07	1.23	1.14	1.03	0.96	1.32	1.23	1.27	1.18	1.33	1.24	1.30	1.21	86.47	5.99
Q9R1L5	Microtubule-associated serine/threonine-protein kinase 1 OS=Mus musculus GN=Mast1 PE=1 SV=3 - [MAST1_MOUSE]	11.72	7	11	13	25	1.08	1.08	1.04	1.06	0.99	1.22	1.11	1.27	1.18	1.22	1.11	1.19	1.10	170.89	8.44
Q8CCP0-2	Isoform 2 of Nuclear export mediator factor Nemf OS=Mus musculus GN=Nemf - [NEMF_MOUSE]	3.14	4	3	3	3	1.77	1.54	0.96	1.31	1.08	1.71	1.02	1.27	1.04	1.16	0.91	1.00	0.82	101.97	6.18
Q8C8N3	Brorin OS=Mus musculus GN=Vwc2 PE=1 SV=1 - [VWC2_MOUSE]	9.26	1	2	2	5	1.06	1.13	0.91	1.05	0.89	1.13	1.11	1.27	1.07	1.01	0.84	0.91	0.79	35.36	5.38
Q9D7E4	UPF0449 protein C19orf25 homolog OS=Mus musculus PE=1 SV=1 - [CS025_MOUSE]	61.47	1	4	4	13	1.12	1.01	0.97	1.06	0.93	1.17	1.06	1.27	1.12	1.10	1.01	1.10	0.87	12.10	5.25
Q8BYI6-3	Isoform 3 of Lysophosphatidylcholine acyltransferase 2 OS=Mus musculus GN=Lpcat2 - [PCAT2_MOUSE]	3.39	3	1	1	1	0.83	0.86	1.02	0.87	1.05	1.19	1.41	1.27	1.52	1.08	1.30	0.90	1.08	25.47	10.11

P08553	Neurofilament medium polypeptide OS=Mus musculus GN=Nefm PE=1 SV=4 - [NFM_MOUSE]	71.46	2	56	61	1407	0.95	0.85	0.88	0.88	0.93	1.27	1.31	1.27	1.30	1.06	1.09	1.00	1.03	95.86	4.77
Q8BPI1-2	Isoform 2 of Protein kintoun OS=Mus musculus GN=Dnaaf2 - [KTU_MOUSE]	1.68	2	1	1	2	1.12	1.24	1.10	1.03	0.92	1.30	1.15	1.27	1.13	1.30	1.16	1.21	1.07	84.55	5.05
P09602	Non-histone chromosomal protein HMG-17 OS=Mus musculus GN=Hmgn2 PE=1 SV=2 - [HMGN2_MOUSE]	22.22	6	2	2	23	1.19	0.85	0.70	1.00	0.86	1.21	1.03	1.28	1.09	1.24	1.05	1.13	0.99	9.42	9.99
Q7TMK6	Protein Hook homolog 2 OS=Mus musculus GN=Hook2 PE=1 SV=3 - [HOOK2_MOUSE]	2.37	1	1	2	32	1.04	1.12	1.08	0.97	0.93	1.03	0.99	1.28	1.23	1.05	1.02	1.19	1.15	83.31	5.43
D9J2V6	Pre-B-cell leukemia transcription factor 1 OS=Mus musculus GN=Pbx1 PE=2 SV=1 - [D9J2V6_MOUSE]	4.13	3	1	1	1	0.86	1.03	1.21	1.16	1.35	1.69	1.97	1.28	1.48	1.16	1.35	1.30	1.52	37.03	6.60
Q5PR69-2	Isoform 2 of Uncharacterized protein KIAA1211 OS=Mus musculus GN=Kiaa1211 - [K1211_MOUSE]	30.65	5	23	23	72	1.32	1.01	0.80	1.07	0.87	1.31	1.02	1.28	1.00	1.36	1.05	1.20	0.97	131.87	5.35
Q9CZN4-3	Isoform 3 of Protein shisa-9 OS=Mus musculus GN=Shisa9 - [SHSA9_MOUSE]	9.46	4	3	3	6	1.08	1.12	1.06	1.04	0.95	1.25	1.11	1.28	1.18	1.09	1.02	1.11	0.98	46.68	8.62
F6Q8N5	Protein Upf3b (Fragment) OS=Mus musculus GN=Upf3b PE=4 SV=1 - [F6Q8N5_MOUSE]	22.69	1	1	5	7	1.07	0.65	0.61	0.95	0.89	1.14	1.06	1.28	1.19	1.05	0.99	0.97	0.91	32.41	9.74
B1AXE7	Zinc finger protein 185 (Fragment) OS=Mus musculus GN=Zfp185 PE=2 SV=1 - [B1AXE7_MOUSE]	15.38	3	1	1	2	1.09	0.98	0.90	1.31	1.21	1.19	1.09	1.28	1.17	1.30	1.20	1.00	0.92	5.82	7.43
Q64314	Hematopoietic progenitor cell antigen CD34 OS=Mus musculus GN=Cd34 PE=1 SV=1 - [CD34_MOUSE]	17.02	1	1	7	24	1.14	1.97	1.72	0.96	0.84	1.13	0.99	1.28	1.11	0.98	0.86	1.10	0.96	40.96	5.30
F6T4M4	Serine/arginine repetitive matrix protein 1 (Fragment) OS=Mus musculus GN=Srrm1 PE=2 SV=1 - [F6T4M4_MOUSE]	26.43	1	1	4	14	1.65	1.19	0.68	1.05	0.62	1.27	0.75	1.28	0.72	1.08	0.62	1.27	0.79	16.23	5.38

P46718	Programmed cell death protein 2 OS=Mus musculus GN=Pdcd2 PE=2 SV=2 - [PDCD2_MOUSE]	4.66	1	1	1	1	0.91	0.81	0.88	1.20	1.32	1.23	1.34	1.28	1.39	0.98	1.08	0.87	0.95	38.32	5.38
Q8R1U2	Cell growth regulator with EF hand domain protein 1 OS=Mus musculus GN=Cgref1 PE=2 SV=1 - [CGRE1_MOUSE]	16.37	2	3	3	5	1.09	1.13	0.96	0.97	0.89	1.11	1.18	1.28	1.08	1.17	1.08	1.18	1.08	30.83	4.31
Q9QZH3	Peptidyl-prolyl cis-trans isomerase E OS=Mus musculus GN=Ppie PE=2 SV=2 - [PPIE_MOUSE]	39.53	1	7	8	14	0.99	0.81	0.81	0.95	0.94	1.23	1.14	1.28	1.26	1.11	1.05	1.06	0.99	33.43	5.60
E0CY42	Chloride intracellular channel protein 6 (Fragment) OS=Mus musculus GN=Clic6 PE=2 SV=1 - [E0CY42_MOUSE]	24.00	2	1	1	3	1.15	0.64	0.55	1.11	0.97	0.99	0.85	1.28	1.10	1.56	1.36	1.18	1.03	11.29	5.14
F7CDW2	Serine-rich coiled-coil domain-containing protein 1 (Fragment) OS=Mus musculus GN=Ccser1 PE=4 SV=1 - [F7CDW2_MOUSE]	8.93	3	1	1	1	0.75	0.48	0.64	0.85	1.12	0.92	1.21	1.28	1.69	0.98	1.30	0.49	0.64	18.72	5.21
Q6ZQ03	Formin-binding protein 4 OS=Mus musculus GN=Fnbp4 PE=1 SV=2 - [FNBP4_MOUSE]	5.72	3	4	4	7	1.16	1.28	1.12	1.07	0.94	1.12	0.94	1.28	1.02	1.29	0.98	1.28	0.99	111.18	4.73
H7BX02	Probable E3 ubiquitin-protein ligase Roquin OS=Mus musculus GN=Rc3h1 PE=2 SV=1 - [H7BX02_MOUSE]	1.78	2	1	1	2	0.99	0.95	0.96	0.95	0.96	1.18	1.18	1.28	1.28	1.21	1.22	1.05	1.05	124.32	7.18
Q6PRU9	HemK methyltransferase family member 2, isoform CRA_a OS=Mus musculus GN=N6amt1 PE=2 SV=1 - [Q6PRU9_MOUSE]	12.32	4	1	1	2	0.98	1.21	1.23	0.97	0.99	1.13	1.14	1.28	1.30	1.20	1.22	1.39	1.42	14.68	5.21
Q80Y61	Brain-specific angiogenesis inhibitor 1-associated protein 2-like protein 2 OS=Mus musculus GN=Baiap2l2 PE=1 SV=1 - [BI2L2_MOUSE]	1.72	1	1	1	2	1.05	1.20	1.14	0.84	0.80	1.51	1.44	1.28	1.21	1.35	1.29	1.16	1.11	58.37	9.50
Q8BZ94-3	Isoform 3 of Zinc finger matrin-type protein 4 OS=Mus musculus GN=Zmat4 - [ZMAT4_MOUSE]	8.13	3	1	1	4	0.80	0.92	1.15	0.89	1.11	0.90	1.11	1.28	1.59	0.97	1.21	0.93	1.16	17.84	9.64

P10922	Histone H1.0 OS=Mus musculus GN=H1f0 PE=2 SV=4 - [H10_MOUSE]	31.44	1	8	8	57	1.03	0.93	0.94	0.98	0.97	1.07	1.04	1.28	1.21	1.16	1.10	1.10	1.08	20.85	10.90
Q62132-3	Isoform Gamma of Receptor-type tyrosine-protein phosphatase R OS=Mus musculus GN=Ptprr - [PTPRR_MOUSE]	2.18	3	1	1	1	0.97	1.43	1.47	1.25	1.29	0.98	1.01	1.28	1.32	1.32	1.36	1.13	1.17	46.56	7.85
Q5RL57	A kinase (PRKA) anchor protein 8-like OS=Mus musculus GN=Akap8l PE=2 SV=1 - [Q5RL57_MOUSE]	12.79	2	6	6	26	1.14	1.17	1.03	1.18	0.93	1.10	1.20	1.28	1.26	1.19	1.10	1.26	1.08	71.26	5.06
Q0VBM2	Protein FAM83B OS=Mus musculus GN=Fam83b PE=1 SV=1 - [FA83B_MOUSE]	1.28	1	1	1	4	0.70	0.87	1.24	1.04	1.48	0.97	1.37	1.28	1.82	1.16	1.65	0.94	1.34	114.61	9.11
Q7TSH4	Centriolar coiled-coil protein of 110 kDa OS=Mus musculus GN=Ccp110 PE=1 SV=1 - [CP110_MOUSE]	3.09	1	2	3	7	1.11	1.11	1.00	1.06	0.95	1.24	1.11	1.28	1.15	1.21	1.10	1.29	1.17	111.07	8.85
Q9D1H8	39S ribosomal protein L53, mitochondrial OS=Mus musculus GN=Mrpl53 PE=2 SV=1 - [RM53_MOUSE]	14.41	1	1	1	2	1.24	1.06	0.86	0.95	0.77	1.04	0.84	1.28	1.03	0.90	0.73	1.40	1.14	12.73	9.52
G3UX81	Protein Hmgxb4 OS=Mus musculus GN=Hmgxb4 PE=2 SV=1 - [G3UX81_MOUSE]	10.87	2	1	1	4	1.52	1.30	0.85	1.18	0.77	1.45	0.95	1.28	0.84	1.41	0.93	1.62	1.06	10.84	7.43
E9PZ00	Sulfated glycoprotein 1 OS=Mus musculus GN=Psap PE=2 SV=1 - [E9PZ00_MOUSE]	47.73	4	1	22	210	1.16	0.85	0.73	0.88	0.75	1.27	1.09	1.28	1.10	1.33	1.14	0.99	0.85	60.63	5.24
D3YZ20	DNA-directed RNA polymerase II subunit RPB11 OS=Mus musculus GN=Polr2j PE=2 SV=1 - [D3YZ20_MOUSE]	9.01	3	1	1	2	1.08	0.90	0.83	0.90	0.83	1.30	1.21	1.28	1.19	1.24	1.15	1.13	1.05	12.58	6.77
Q7TPM1	Protein PRRC2B OS=Mus musculus GN=Prrc2b PE=1 SV=1 - [PRC2B_MOUSE]	24.02	1	1	23	60	1.09	1.08	0.99	0.98	0.90	1.16	1.06	1.28	1.17	1.28	1.17	1.10	1.01	160.81	8.16
E9Q811	Serine/threonine-protein kinase STK11 OS=Mus musculus GN=Stk11 PE=2 SV=1 - [E9Q811_MOUSE]	9.68	4	2	2	4	0.92	0.97	1.06	1.24	1.35	0.84	0.91	1.28	1.39	0.98	1.06	0.96	1.05	34.61	5.44
E9Q0V6	NHS-like protein 2 OS=Mus musculus GN=Nhsl2 PE=2 SV=1 - [E9Q0V6_MOUSE]	16.00	2	11	11	18	1.07	0.98	0.94	1.04	1.03	1.40	1.42	1.28	1.36	1.40	1.38	1.42	1.36	132.27	8.48

Q9Z266	SNARE-associated protein Snapin OS=Mus musculus GN=Snapin PE=1 SV=1 - [SNAPN_MOUSE]	26.47	2	3	3	7	1.17	1.00	0.88	0.97	0.85	1.30	1.10	1.28	1.04	1.22	1.06	1.14	0.96	14.89	9.31
P58064	28S ribosomal protein S6, mitochondrial OS=Mus musculus GN=Mrps6 PE=2 SV=3 - [RT06_MOUSE]	25.60	1	3	3	4	0.98	0.81	0.88	0.94	0.96	1.22	1.23	1.28	1.30	1.29	1.32	1.16	1.23	14.30	9.50
D3Z6P2	Zinc finger protein 740 (Fragment) OS=Mus musculus GN=Zfp740 PE=2 SV=1 - [D3Z6P2_MOUSE]	11.21	4	1	1	2	0.96	0.95	0.98	1.03	1.07	1.11	1.14	1.28	1.33	1.09	1.13	1.46	1.51	12.10	9.50
Q99JX3	Golgi reassembly-stacking protein 2 OS=Mus musculus GN=Gorasp2 PE=1 SV=3 - [GORS2_MOUSE]	29.71	5	8	8	11	1.06	0.77	0.78	1.02	0.95	1.44	1.31	1.28	1.23	1.27	1.17	1.15	1.02	47.01	4.79
Q63918	Serum deprivation-response protein OS=Mus musculus GN=Sdpr PE=1 SV=3 - [SDPR_MOUSE]	30.62	1	11	11	29	1.09	0.93	0.87	0.90	0.90	1.24	1.12	1.28	1.28	1.09	1.07	1.04	1.00	46.74	5.21
Q99LT0	Protein dpy-30 homolog OS=Mus musculus GN=Dpy30 PE=1 SV=1 - [DPY30_MOUSE]	47.47	1	3	3	8	1.04	0.74	0.71	0.97	0.89	1.07	1.11	1.28	1.27	1.08	1.09	0.93	0.94	11.21	4.88
Q499E5	Storkhead-box protein 2 OS=Mus musculus GN=Stox2 PE=2 SV=2 - [STOX2_MOUSE]	12.74	3	9	9	15	1.18	1.12	0.93	1.17	0.91	1.40	1.05	1.28	1.07	1.26	1.08	1.23	1.06	102.69	8.46
E9PYX7	Afadin OS=Mus musculus GN=Mllt4 PE=2 SV=1 - [E9PYX7_MOUSE]	24.23	3	1	32	81	1.32	0.96	0.73	1.16	0.87	1.01	0.76	1.28	0.97	1.14	0.87	1.22	0.92	188.79	6.42
Q9CY50	Translocon-associated protein subunit alpha OS=Mus musculus GN=Ssr1 PE=1 SV=1 - [SSRA_MOUSE]	9.09	1	2	2	9	0.94	1.07	1.09	1.14	1.22	1.33	1.43	1.28	1.39	1.15	1.26	1.03	1.09	32.05	4.45
Q9QUN9	Dickkopf-related protein 3 OS=Mus musculus GN=Dkk3 PE=1 SV=1 - [DKK3_MOUSE]	35.82	1	9	9	40	0.97	0.99	1.05	1.00	1.04	1.22	1.25	1.28	1.30	1.01	1.10	0.88	0.89	38.36	4.54
Q6PGL7	WASH complex subunit FAM21 OS=Mus musculus GN=Fam21 PE=1 SV=1 - [FAM21_MOUSE]	46.70	2	38	38	129	1.00	0.87	0.86	0.90	0.87	1.28	1.28	1.28	1.27	1.15	1.15	1.03	1.04	145.22	4.77
Q5FW52-	Isoform 2 of Muscular LMNA-interacting protein OS=Mus musculus GN=Mlip - [MLIP_MOUSE]	51.10	2	7	7	35	0.85	0.74	0.93	0.93	1.09	1.20	1.39	1.28	1.43	1.09	1.27	0.89	1.02	24.99	6.81

E9QAF9	Protein TANC1 OS=Mus musculus GN=Tanc1 PE=2 SV=1 - [E9QAF9_MOUSE]	0.87	2	1	1	2	1.13	1.21	1.07	1.21	1.07	1.25	1.10	1.29	1.13	1.36	1.20	1.38	1.22	199.94	8.32
Q3UHY7	Coiled-coil domain containing 32 OS=Mus musculus GN=Ccdc32 PE=2 SV=1 - [Q3UHY7_MOUSE]	34.64	3	3	3	11	1.05	0.77	0.73	0.86	0.83	1.20	1.13	1.29	1.22	1.10	1.06	0.99	0.95	19.85	4.78
H3BJG4	Protein Trip11 (Fragment) OS=Mus musculus GN=Trip11 PE=2 SV=1 - [H3BJG4_MOUSE]	5.20	3	7	7	18	1.09	1.07	1.02	0.94	0.90	1.30	1.21	1.29	1.26	1.22	1.17	1.11	1.08	194.54	5.24
Q3UIA2-4	Isoform 4 of Rho GTPase-activating protein 17 OS=Mus musculus GN=Arhgap17 - [RHG17_MOUSE]	5.50	5	2	2	3	0.82	1.36	1.65	1.19	1.45	0.93	1.13	1.29	1.56	1.14	1.39	1.07	1.31	79.77	8.24
P29533	Vascular cell adhesion protein 1 OS=Mus musculus GN=Vcam1 PE=1 SV=1 - [VCAM1_MOUSE]	30.58	3	21	21	65	0.99	0.90	0.96	0.93	0.94	1.23	1.19	1.29	1.29	1.12	1.11	1.10	1.11	81.27	5.30
Q6PAL7	AT-hook DNA-binding motif-containing protein 1 OS=Mus musculus GN=Ahdc1 PE=1 SV=1 - [AHDC1_MOUSE]	2.45	1	2	2	4	1.00	1.19	1.19	1.03	1.02	1.33	1.32	1.29	1.28	1.19	1.19	1.38	1.38	167.98	8.97
Q6P8J7	Creatine kinase S-type, mitochondrial OS=Mus musculus GN=Ckmt2 PE=1 SV=1 - [KCRS_MOUSE]	10.98	1	1	3	11	1.15	0.81	0.70	0.86	0.75	1.50	1.30	1.29	1.12	1.24	1.08	1.03	0.90	47.44	8.40
Q91WK1	SPRY domain-containing protein 4 OS=Mus musculus GN=Spryd4 PE=2 SV=1 - [SPRY4_MOUSE]	11.59	1	1	1	1	1.06	0.84	0.79	1.13	1.07	1.48	1.39	1.29	1.21	0.81	0.76	0.81	0.77	23.26	9.45
Q68ED7	CREB-regulated transcription coactivator 1 OS=Mus musculus GN=Crtc1 PE=2 SV=1 - [CRTC1_MOUSE]	18.10	1	6	6	93	0.98	1.02	1.03	0.91	0.92	1.32	1.34	1.29	1.30	1.24	1.18	1.11	1.13	66.90	6.05
P43276	Histone H1.5 OS=Mus musculus GN=Hist1h1b PE=1 SV=2 - [H15_MOUSE]	30.04	1	5	9	30	1.04	0.88	0.83	1.07	1.04	1.22	1.23	1.29	1.26	1.03	1.08	0.99	0.93	22.56	10.92
Q8JZX9	Cdc42 effector protein 2 OS=Mus musculus GN=Cdc42ep2 PE=1 SV=1 - [BORG1_MOUSE]	48.60	1	5	5	15	1.13	1.18	0.99	1.03	0.94	1.21	1.22	1.29	1.17	1.25	1.19	1.19	1.15	22.98	5.54
Q69ZF8	E3 ubiquitin-protein ligase MSL2 OS=Mus musculus GN=Msl2 PE=2 SV=2 - [MSL2_MOUSE]	1.91	1	1	1	2	1.00	1.11	1.10	1.16	1.15	1.22	1.21	1.29	1.28	1.25	1.25	1.39	1.39	62.50	7.80

Q62511-2	Isoform 2 of E3 ubiquitin-protein ligase ZFP91 OS=Mus musculus GN=Zfp91 - [ZFP91_MOUSE]	4.18	3	1	1	2	1.25	1.08	0.86	1.26	1.01	1.29	1.03	1.29	1.02	1.13	0.90	1.18	0.94	51.51	6.58
Q924A2	Protein capicua homolog OS=Mus musculus GN=Cic PE=1 SV=2 - [CIC_MOUSE]	8.57	5	14	14	24	1.00	1.03	1.12	0.96	0.97	1.26	1.34	1.29	1.31	1.09	1.20	1.03	1.08	257.97	8.02
Q9D0R9	WD repeat-containing protein 89 OS=Mus musculus GN=Wdr89 PE=2 SV=1 - [WDR89_MOUSE]	8.03	1	1	1	3	0.87	1.06	1.21	0.95	0.85	1.17	1.40	1.29	1.47	1.25	1.51	0.87	0.98	42.44	5.66
Q9D8W7	OCIA domain-containing protein 2 OS=Mus musculus GN=Ociad2 PE=2 SV=1 - [OCAD2_MOUSE]	44.16	1	5	5	17	1.04	1.02	1.08	1.25	1.28	1.53	1.41	1.29	1.24	1.11	1.26	1.04	1.20	16.91	9.41
F6TKE8	Ly6/PLAUR domain-containing protein 1 (Fragment) OS=Mus musculus GN=Lypd1 PE=4 SV=1 - [F6TKE8_MOUSE]	14.63	4	1	1	9	1.53	1.37	0.92	1.11	0.77	1.38	0.93	1.29	0.85	1.51	0.95	1.76	1.17	9.07	4.88
D3YUP8	Inhibitor of growth protein 3 OS=Mus musculus GN=Ing3 PE=2 SV=1 - [D3YUP8_MOUSE]	7.80	3	2	2	4	0.95	0.99	1.03	0.90	0.94	1.24	1.29	1.29	1.35	1.06	1.12	1.07	1.12	45.53	5.63
Q99N91	39S ribosomal protein L34, mitochondrial OS=Mus musculus GN=Mrpl34 PE=2 SV=1 - [RM34_MOUSE]	25.00	1	2	2	4	0.94	1.09	1.15	1.05	1.12	1.24	1.31	1.29	1.37	1.22	1.30	1.15	1.23	10.52	11.81
D3Z069	Pleckstrin homology-like domain family B member 2 (Fragment) OS=Mus musculus GN=Phldb2 PE=2 SV=1 - [D3Z069_MOUSE]	0.88	3	1	1	2	2.55	3.39	1.33	0.75	0.29	0.89	0.35	1.29	0.51	1.03	0.47	0.93	0.36	127.10	6.27
Q61818-2	Isoform 2 of Retinoic acid-induced protein 1 OS=Mus musculus GN=Rai1 - [RAI1_MOUSE]	5.65	3	7	8	26	1.13	1.04	0.92	1.09	0.97	1.35	1.19	1.29	1.23	1.24	1.14	1.14	1.01	195.96	8.87
Q6IQX8	Protein Zfp219 OS=Mus musculus GN=Zfp219 PE=2 SV=1 - [Q6IQX8_MOUSE]	5.37	1	3	3	4	1.10	1.44	1.41	1.14	1.03	1.26	1.17	1.29	1.22	1.32	1.20	1.24	1.11	77.75	9.17
Q6P549	Phosphatidylinositol 3,4,5-trisphosphate 5-phosphatase 2 OS=Mus musculus GN=Inppl1 PE=1 SV=1 - [SHIP2_MOUSE]	1.43	1	1	1	2	1.17	1.32	1.12	1.01	0.87	1.39	1.18	1.29	1.10	1.13	0.96	1.22	1.04	138.89	6.54

F6ZGR6	Protein D430041D05Rik (Fragment) OS=Mus musculus GN=D430041D05Rik PE=4 SV=1 - [F6ZGR6_MOUSE]	12.16	2	1	13	46	1.28	1.04	0.81	1.18	0.92	1.32	1.03	1.29	1.00	1.09	0.85	1.13	0.88	181.02	9.38
Q9D358	Low molecular weight phosphotyrosine protein phosphatase OS=Mus musculus GN=Acp1 PE=1 SV=3 - [PPAC_MOUSE]	45.57	1	1	4	39	1.05	1.37	1.30	1.01	0.96	1.30	1.23	1.29	1.22	1.25	1.19	1.49	1.42	18.18	6.74
P97314	Cysteine and glycine-rich protein 2 OS=Mus musculus GN=Csrp2 PE=1 SV=3 - [CSRP2_MOUSE]	35.23	1	5	5	15	1.07	1.05	0.96	1.03	0.91	1.06	1.01	1.29	1.11	1.34	1.16	1.00	0.98	20.91	8.62
Q9D772	Protein FAM219A OS=Mus musculus GN=Fam219a PE=2 SV=2 - [F219A_MOUSE]	50.32	2	1	4	27	0.97	1.15	1.18	1.07	1.10	1.21	1.23	1.29	1.33	1.27	1.30	1.10	1.14	17.45	4.68
A6H6E2	Multimerin-2 OS=Mus musculus GN=Mmrn2 PE=2 SV=1 - [MMRN2_MOUSE]	6.79	1	4	4	7	0.98	1.17	1.06	0.94	0.96	0.88	0.68	1.29	0.93	1.01	0.81	0.93	0.75	105.14	5.57
Q9JJ43-5	Isoform 5 of RNA binding protein fox-1 homolog 1 OS=Mus musculus GN=Rbfox1 - [RFOX1_MOUSE]	20.91	3	1	7	23	0.90	0.96	1.12	0.86	0.97	1.25	1.38	1.29	1.50	1.12	1.31	1.11	1.28	40.26	5.99
Q8BU11	TOX high mobility group box family member 4 OS=Mus musculus GN=Tox4 PE=1 SV=3 - [TOX4_MOUSE]	12.44	8	6	6	16	1.08	0.89	0.83	1.04	0.91	1.38	1.30	1.29	1.15	1.18	1.08	1.03	0.91	65.92	5.01
D3YVF0	A-kinase anchor protein 5 OS=Mus musculus GN=Akap5 PE=2 SV=2 - [AKAP5_MOUSE]	71.68	2	34	34	320	1.02	1.00	1.01	1.01	1.00	1.27	1.23	1.29	1.23	1.27	1.23	1.21	1.21	79.35	4.75
Q8VCQ8	Caldesmon 1 OS=Mus musculus GN=Cald1 PE=2 SV=1 - [Q8VCQ8_MOUSE]	33.21	8	1	18	72	1.14	1.17	1.03	1.03	0.91	1.22	1.07	1.29	1.13	1.29	1.13	1.23	1.08	60.42	7.37
Q9R1C0	Transcription initiation factor TFIID subunit 7 OS=Mus musculus GN=Taf7 PE=1 SV=1 - [TAF7_MOUSE]	3.52	1	1	1	1	1.07	1.21	1.13	0.99	0.93	1.19	1.11	1.29	1.21	1.24	1.16	1.36	1.27	39.10	5.33
Q3UHB8	Coiled-coil domain-containing protein 177 OS=Mus musculus GN=Ccdc177 PE=1 SV=1 - [CC177_MOUSE]	22.95	1	12	12	42	1.02	1.08	1.09	1.04	1.01	1.23	1.15	1.29	1.26	1.19	1.13	1.15	1.08	79.81	10.80
G3X8Q1	Calcineurin binding protein 1, isoform CRA_a OS=Mus musculus GN=Cabin1 PE=4 SV=1 - [G3X8Q1_MOUSE]	1.74	1	2	2	6	0.99	1.47	1.48	1.05	1.05	1.26	1.26	1.29	1.30	1.17	1.18	1.10	1.10	243.02	6.01

Q8BHD8	Protein-L-isoaspartate O-methyltransferase domain-containing protein 2 OS=Mus musculus GN=Pcmtd2 PE=2 SV=1 - [PCMD2_MOUSE]	5.01	1	1	1	2	1.25	3.35	2.66	1.13	0.90	1.51	1.19	1.29	1.03	1.13	0.90	1.11	0.89	40.73	6.46
J3QPM4	Cytokine receptor-like factor 3 OS=Mus musculus GN=Crlf3 PE=4 SV=1 - [J3QPM4_MOUSE]	18.99	4	1	1	2	1.19	1.19	1.00	0.97	0.81	1.16	0.97	1.29	1.09	1.17	0.99	1.03	0.87	9.15	9.66
Q8R317	Ubiquilin-1 OS=Mus musculus GN=Ubqln1 PE=1 SV=1 - [UBQL1_MOUSE]	32.47	1	1	11	111	0.53	0.81	1.52	0.68	1.28	0.84	1.56	1.29	2.42	0.75	1.40	0.85	1.60	61.94	4.94
D3Z7J7	Deoxyhypusine hydroxylase (Fragment) OS=Mus musculus GN=Dohh PE=2 SV=1 - [D3Z7J7_MOUSE]	10.64	4	1	1	1	1.07	0.94	0.88	0.93	0.87	1.31	1.22	1.29	1.21	1.10	1.03	1.15	1.08	20.51	4.94
E9QMV2	Protein Gm6314 OS=Mus musculus GN=Abracl PE=4 SV=1 - [E9QMV2_MOUSE]	11.11	2	1	1	2	1.07	1.28	1.19	0.89	0.83	1.16	1.08	1.30	1.20	1.02	0.96	1.01	0.95	9.05	5.80
Q9WU84	Copper chaperone for superoxide dismutase OS=Mus musculus GN=Ccs PE=1 SV=1 - [CCS_MOUSE]	8.03	1	2	2	4	0.95	1.01	1.05	0.93	0.97	1.22	1.27	1.30	1.35	1.17	1.23	1.23	1.29	28.89	6.10
O08600	Endonuclease G, mitochondrial OS=Mus musculus GN=Endog PE=2 SV=1 - [NUCG_MOUSE]	9.18	1	2	2	4	0.93	1.12	1.20	1.16	1.24	1.30	1.38	1.30	1.38	1.39	1.49	1.36	1.46	32.17	9.54
P12025	Midkine OS=Mus musculus GN=Mdk PE=2 SV=2 - [MK_MOUSE]	7.14	1	1	1	2	1.21	1.15	0.95	1.02	0.84	1.55	1.28	1.30	1.07	1.47	1.22	1.62	1.34	15.42	9.70
A2AGL5	Apoptosis, caspase activation inhibitor, isoform CRA_c OS=Mus musculus GN=Aven PE=4 SV=1 - [A2AGL5_MOUSE]	36.41	2	5	5	9	1.03	0.89	0.89	0.94	0.95	1.14	1.16	1.30	1.16	1.17	1.03	0.97	0.94	24.06	4.59
Q8CB77	Transcription elongation factor B polypeptide 3 OS=Mus musculus GN=Tceb3 PE=1 SV=3 - [ELOA1_MOUSE]	4.79	1	4	4	11	1.06	0.99	0.92	0.94	0.92	1.30	1.29	1.30	1.25	1.17	1.11	1.19	1.13	87.11	9.61
P50571	Gamma-aminobutyric acid receptor subunit beta-1 OS=Mus musculus GN=Gabrb1 PE=1 SV=1 - [GBRB1_MOUSE]	11.60	2	1	4	9	1.49	1.69	1.13	1.41	0.95	1.14	0.76	1.30	0.87	1.12	0.75	1.45	0.97	54.07	8.78

O35730-3	Isoform 3 of E3 ubiquitin-protein ligase RING1 OS=Mus musculus GN=Ring1 - [RING1_MOUSE]	15.30	2	2	2	2	1.82	1.86	1.02	0.97	0.82	0.77	0.42	1.30	1.10	0.77	0.42	1.30	1.11	20.79	8.19
Q9JL19	Nuclear receptor coactivator 6 OS=Mus musculus GN=Ncoa6 PE=1 SV=1 - [NCOA6_MOUSE]	3.77	3	4	4	13	1.06	1.04	0.89	0.97	0.85	1.41	1.24	1.30	1.18	1.24	1.13	1.24	1.11	219.52	9.32
Q8K3J1	NADH dehydrogenase [ubiquinone] iron-sulfur protein 8, mitochondrial OS=Mus musculus GN=Ndufs8 PE=1 SV=1 - [NDUS8_MOUSE]	41.98	1	8	8	198	1.01	0.90	0.88	0.97	0.98	1.26	1.28	1.30	1.30	1.21	1.20	1.13	1.12	24.02	6.21
E0CY18	AN1-type zinc finger protein 2B (Fragment) OS=Mus musculus GN=Zfand2b PE=2 SV=1 - [E0CY18_MOUSE]	17.43	2	2	2	15	1.07	1.00	0.93	0.89	0.90	1.17	1.09	1.30	1.21	1.19	1.14	1.24	1.16	23.68	7.72
Q80Z37	E3 ubiquitin-protein ligase Topors OS=Mus musculus GN=Topors PE=1 SV=1 - [TOPRS_MOUSE]	1.45	1	1	1	2	0.96	1.22	1.27	1.06	1.11	1.10	1.14	1.30	1.35	1.17	1.22	1.19	1.25	117.01	9.52
Q5SUG2	Protein Cacna1g OS=Mus musculus GN=Cacna1g PE=2 SV=1 - [Q5SUG2_MOUSE]	0.97	11	1	1	2	0.89	1.01	1.13	1.02	1.15	1.06	1.18	1.30	1.46	1.10	1.24	0.86	0.97	241.29	7.23
Q8C624	Uncharacterized protein C16orf55 homolog OS=Mus musculus PE=2 SV=1 - [CP055_MOUSE]	13.64	1	1	1	1	1.04	1.02	0.98	0.92	0.88	1.13	1.08	1.30	1.25	1.13	1.09	0.95	0.92	15.03	9.28
Q8CI61	BAG family molecular chaperone regulator 4 OS=Mus musculus GN=Bag4 PE=1 SV=2 - [BAG4_MOUSE]	28.45	1	8	8	30	1.06	0.98	0.92	0.98	0.90	1.17	1.15	1.30	1.23	1.25	1.09	1.13	1.11	49.06	5.34
Q3UNH4	G protein-regulated inducer of neurite outgrowth 1 OS=Mus musculus GN=Gprin1 PE=1 SV=2 - [GRIN1_MOUSE]	66.74	1	62	62	341	1.08	0.94	0.89	0.94	0.88	1.32	1.24	1.30	1.20	1.19	1.11	1.11	1.05	95.44	7.93
Q9ERE8	Mesoderm development candidate 1 OS=Mus musculus GN=Mesdc1 PE=1 SV=1 - [MESD1_MOUSE]	3.04	1	1	1	1	1.23	1.32	1.07	0.95	0.77	1.54	1.24	1.30	1.05	0.64	0.52	0.98	0.80	37.76	8.15
B2RX88-2	Isoform 2 of Centrosome and spindle pole associated protein 1 OS=Mus musculus GN=Cspp1 - [CSPP1_MOUSE]	3.42	3	2	2	3	1.10	1.55	1.42	1.21	1.11	1.53	1.39	1.30	1.18	1.09	0.99	1.37	1.25	131.13	8.44

Q3THG9	Alanyl-tRNA editing protein Aarsd1 OS=Mus musculus GN=Aarsd1 PE=1 SV=2 - [AASD1_MOUSE]	9.71	3	3	3	9	1.00	1.15	1.04	1.07	1.08	1.40	1.44	1.30	1.34	1.28	1.32	1.38	1.30	44.94	6.42
Q2M3X8	Phosphatase and actin regulator 1 OS=Mus musculus GN=Phactr1 PE=1 SV=1 - [PHAR1_MOUSE]	40.00	5	13	16	84	1.06	1.07	1.05	1.01	1.00	1.27	1.30	1.30	1.31	1.22	1.20	1.33	1.30	66.24	6.96
E9Q7F2	E3 ubiquitin-protein ligase RNF169 OS=Mus musculus GN=Rnf169 PE=2 SV=1 - [RN169_MOUSE]	2.31	1	1	1	2	1.15	2.00	1.73	1.01	0.88	1.19	1.03	1.30	1.13	1.36	1.19	1.46	1.27	75.75	9.35
Q810B7	SLIT and NTRK-like protein 5 OS=Mus musculus GN=Slitrk5 PE=2 SV=1 - [SLIK5_MOUSE]	6.58	1	4	4	10	1.07	1.17	1.07	0.97	0.91	1.30	1.19	1.30	1.39	1.07	1.13	1.00	0.95	107.10	6.93
Q9JI10	Serine/threonine-protein kinase 3 OS=Mus musculus GN=Stk3 PE=1 SV=1 - [STK3_MOUSE]	7.65	4	1	3	4	0.99	0.96	0.96	0.86	0.86	0.99	0.99	1.30	1.31	1.09	1.10	1.11	1.11	56.82	4.98
Q6DVA0	LEM domain-containing protein 2 OS=Mus musculus GN=Lemd2 PE=1 SV=1 - [LEMD2_MOUSE]	3.33	1	1	1	2	1.02	1.40	1.37	0.76	0.74	1.21	1.18	1.30	1.28	1.20	1.18	1.21	1.19	57.47	9.01
P48725-3	Isoform 3 of Pericentrin OS=Mus musculus GN=Pcnt - [PCNT_MOUSE]	0.88	3	1	1	2	1.04	0.82	0.79	0.91	0.88	1.03	0.99	1.30	1.25	1.16	1.12	1.02	0.99	284.41	5.08
Q8C181	Muscleblind-like protein 2 OS=Mus musculus GN=Mbnl2 PE=2 SV=2 - [MBNL2_MOUSE]	19.57	4	1	5	8	0.85	0.77	0.91	0.96	1.12	1.26	1.47	1.30	1.52	1.05	1.24	0.94	1.10	40.13	9.00
P31230	Aminoacyl tRNA synthase complex-interacting multifunctional protein 1 OS=Mus musculus GN=Aimp1 PE=1 SV=2 - [AIMP1_MOUSE]	54.52	6	12	13	83	0.99	0.96	0.97	0.88	0.93	1.25	1.31	1.30	1.31	1.17	1.19	1.14	1.19	33.98	8.35
E9PX84	Disabled homolog 2 OS=Mus musculus GN=Dab2 PE=2 SV=1 - [E9PX84_MOUSE]	7.02	10	3	3	5	1.23	0.88	0.68	0.97	0.77	1.20	0.93	1.30	1.01	0.89	0.83	1.08	0.91	56.47	6.67
E9PV80	Nuclear receptor coactivator 2 OS=Mus musculus GN=Ncoa2 PE=4 SV=1 - [E9PV80_MOUSE]	0.72	2	1	1	2	1.10	1.30	1.18	1.10	1.00	1.41	1.28	1.30	1.19	1.54	1.41	1.23	1.13	151.26	6.62
E9PZM7	Protein Scaf11 OS=Mus musculus GN=Scaf11 PE=2 SV=1 - [E9PZM7_MOUSE]	5.70	1	6	7	26	1.13	1.00	0.88	1.05	0.95	1.21	1.04	1.30	1.09	1.16	1.00	1.08	1.02	162.00	6.77

A2A6A1	G patch domain-containing protein 8 OS=Mus musculus GN=Gpatch8 PE=2 SV=1 - [GPTC8_MOUSE]	4.25	1	4	4	8	1.08	1.24	1.15	1.04	0.97	1.27	1.04	1.31	1.13	1.09	1.07	1.14	1.05	164.89	7.64
D3YZ72	Complexin-1 (Fragment) OS=Mus musculus GN=Cplx1 PE=2 SV=1 - [D3YZ72_MOUSE]	48.05	1	1	4	37	1.13	1.10	1.29	1.22	0.89	1.36	1.26	1.31	1.25	1.40	1.17	1.51	1.14	8.84	5.06
O89084	cAMP-specific 3',5'-cyclic phosphodiesterase 4A OS=Mus musculus GN=Pde4a PE=2 SV=2 - [PDE4A_MOUSE]	5.92	3	2	4	11	1.11	0.92	0.83	0.86	0.77	1.19	1.07	1.31	1.17	1.10	0.99	1.01	0.91	93.50	5.26
Q9WUN8	Transcription factor LBX2 OS=Mus musculus GN=Lbx2 PE=2 SV=1 - [LBX2_MOUSE]	14.36	1	1	1	1	1.14	0.87	0.76	1.03	0.91	1.29	1.13	1.31	1.15	1.14	1.01	1.03	0.91	20.90	5.68
P61807	Stannin OS=Mus musculus GN=Snn PE=1 SV=1 - [SNN_MOUSE]	12.50	1	1	1	2	1.01	1.41	1.39	0.96	0.95	0.99	0.97	1.31	1.29	1.11	1.10	1.33	1.32	9.49	5.31
F7AIV2	SH2 domain-containing adapter protein F (Fragment) OS=Mus musculus GN=Shf PE=4 SV=1 - [F7AIV2_MOUSE]	45.88	8	7	7	14	1.01	0.82	0.74	1.00	0.93	1.22	1.15	1.31	1.25	1.10	1.04	1.09	0.98	37.74	5.05
Q45VK7	Cytoplasmic dynein 2 heavy chain 1 OS=Mus musculus GN=Dync2h1 PE=1 SV=1 - [DYHC2_MOUSE]	1.23	2	2	2	2	1.35	1.02	0.75	1.31	0.97	1.22	0.90	1.31	0.97	1.12	0.83	1.14	0.85	492.03	6.60
P20065-2	Isoform Short of Thymosin beta-4 OS=Mus musculus GN=Tmsb4x - [TYB4_MOUSE]	45.45	4	3	5	194	0.36	0.62	2.00	0.80	2.27	1.01	3.32	1.31	5.05	1.19	4.28	0.57	1.61	5.05	5.06
B9EKI5	Casitas B-lineage lymphoma b OS=Mus musculus GN=Cblb PE=1 SV=1 - [B9EKI5_MOUSE]	3.62	3	1	2	3	1.26	1.41	1.12	0.93	0.74	1.06	0.84	1.31	1.03	1.34	1.07	1.60	1.27	104.53	8.02
Q6IFZ6	Keratin, type II cytoskeletal 1b OS=Mus musculus GN=Krt77 PE=1 SV=1 - [K2C1B_MOUSE]	5.42	3	1	3	10	1.23	1.04	0.85	2.96	2.40	0.76	0.61	1.31	1.06	1.50	1.22	1.70	1.38	61.32	8.02
A2CI98	Dystrotelin OS=Mus musculus GN=Dytn PE=2 SV=1 - [DYTN_MOUSE]	2.76	1	1	1	7	1.09	0.66	0.62	0.96	0.90	1.19	1.07	1.31	1.12	1.07	0.99	0.94	0.86	74.01	9.19
P08226	Apolipoprotein E OS=Mus musculus GN=Apoe PE=1 SV=2 - [APOE_MOUSE]	50.80	4	18	18	74	1.08	1.11	0.99	1.02	0.92	1.47	1.28	1.31	1.20	1.43	1.17	1.31	1.23	35.84	5.68
Q5GH67	XK-related protein 4 OS=Mus musculus GN=Xkr4 PE=1 SV=1 - [XKR4_MOUSE]	5.41	1	2	2	2	0.76	1.11	1.46	1.06	1.40	0.99	1.30	1.31	1.72	1.29	1.70	1.15	1.52	71.46	8.03

Q8R5H6	Wiskott-Aldrich syndrome protein family member 1 OS=Mus musculus GN=Wasf1 PE=1 SV=2 - [WASF1_MOUSE]	33.81	1	14	16	66	0.93	0.87	1.01	0.87	0.94	1.25	1.29	1.31	1.35	1.12	1.18	1.13	1.10	61.47	6.37
P70335-2	Isoform 2 of Rho-associated protein kinase 1 OS=Mus musculus GN=Rock1 - [ROCK1_MOUSE]	6.50	2	3	8	25	1.06	1.10	0.94	0.97	0.90	1.01	0.98	1.31	1.24	1.03	0.96	1.09	0.98	157.94	5.86
Q8R149-2	Isoform 2 of BUD13 homolog OS=Mus musculus GN=Bud13 - [BUD13_MOUSE]	4.28	2	2	2	4	1.13	1.22	1.08	1.11	0.98	1.23	1.08	1.31	1.15	1.33	1.18	1.32	1.17	68.60	9.98
Q3TKY6	Peptidyl-prolyl cis-trans isomerase CWC27 homolog OS=Mus musculus GN=Cwc27 PE=1 SV=1 - [CWC27_MOUSE]	17.06	1	5	5	15	0.99	1.14	1.07	1.01	0.95	1.23	1.29	1.31	1.41	1.14	1.19	1.09	1.13	53.51	5.53
Q9R0Q7	Prostaglandin E synthase 3 OS=Mus musculus GN=Ptges3 PE=1 SV=1 - [TEBP_MOUSE]	75.00	2	10	10	197	1.03	0.80	0.76	0.90	0.86	1.28	1.20	1.31	1.27	1.12	1.09	1.12	1.06	18.71	4.55
Q80VY2-2	Isoform 2 of Protein FAM212B OS=Mus musculus GN=Fam212b - [F212B_MOUSE]	4.98	2	1	1	2	1.06	0.99	0.93	1.06	1.00	1.37	1.28	1.31	1.23	1.34	1.27	1.08	1.02	30.84	8.16
Q9CQA6	Coiled-coil-helix-coiled-coil-helix domain-containing protein 1 OS=Mus musculus GN=Chchd1 PE=2 SV=1 - [CHCH1_MOUSE]	39.83	1	3	3	7	0.93	0.59	0.69	0.83	0.89	1.28	1.39	1.31	1.37	1.16	1.22	1.06	1.11	13.60	10.15
Q9D2R6	Cytochrome c oxidase assembly protein 3 homolog, mitochondrial OS=Mus musculus GN=Coa3 PE=2 SV=1 - [COA3_MOUSE]	33.33	2	4	4	9	1.09	1.03	0.95	1.18	0.96	1.32	1.13	1.31	1.16	1.22	1.12	1.22	1.10	11.98	9.86
Q8K0X8	Fasciculation and elongation protein zeta-1 OS=Mus musculus GN=Fez1 PE=1 SV=2 - [FEZ1_MOUSE]	19.39	5	7	7	21	0.97	0.92	0.96	0.87	0.87	1.26	1.30	1.31	1.28	1.12	1.20	1.10	1.17	45.19	4.36
Q9JJ94	Sjoegren syndrome nuclear autoantigen 1 homolog OS=Mus musculus GN=Ssna1 PE=1 SV=1 - [SSNA1_MOUSE]	7.56	1	1	1	5	1.10	0.76	0.67	0.92	0.83	1.30	1.12	1.31	1.14	1.17	1.09	1.17	1.02	13.55	5.68
I3ITR1	MCG50313 OS=Mus musculus GN=AK157302 PE=4 SV=1 - [I3ITR1_MOUSE]	31.78	2	3	3	8	1.04	0.88	0.83	1.06	0.99	1.48	1.42	1.31	1.25	1.12	1.12	1.18	1.18	14.21	8.85
Q3UHU5-	Isoform 3 of Protein SOGA2 OS=Mus musculus GN=Soga2 - [SOGA2_MOUSE]	14.37	5	20	23	44	0.90	1.07	1.13	0.96	1.06	1.23	1.31	1.31	1.44	1.09	1.17	1.06	1.11	207.98	6.18

Q8K1J5	Protein SDE2 homolog OS=Mus musculus GN=Sde2 PE=1 SV=1 - [SDE2_MOUSE]	4.46	1	2	2	6	1.13	0.83	0.85	1.04	1.02	1.33	1.17	1.31	1.16	1.17	1.04	1.12	0.98	48.55	5.66
Q9ERR7	15 kDa selenoprotein OS=Mus musculus GN=Sep15 PE=1 SV=3 - [SEP15_MOUSE]	23.46	1	3	3	17	0.91	0.93	0.99	0.92	1.06	1.21	1.40	1.31	1.41	1.15	1.28	1.07	1.24	17.80	5.35
Q91VC7	Protein phosphatase 1 regulatory subunit 14A OS=Mus musculus GN=Ppp1r14a PE=2 SV=1 - [PP14A_MOUSE]	57.14	1	5	5	24	0.98	0.93	1.00	0.92	0.99	1.36	1.39	1.31	1.27	1.15	1.20	1.09	1.04	16.64	7.34
D3Z7C0	39S ribosomal protein L40, mitochondrial OS=Mus musculus GN=Mrpl40 PE=2 SV=1 - [D3Z7C0_MOUSE]	61.73	2	9	9	34	1.13	1.04	0.94	1.02	0.93	1.28	1.12	1.31	1.10	1.19	1.03	1.15	1.03	19.37	9.19
Q9D198	Pre-mRNA-splicing factor SYF2 OS=Mus musculus GN=Syf2 PE=2 SV=1 - [SYF2_MOUSE]	11.98	1	3	3	4	1.06	0.75	0.69	0.99	1.04	1.20	1.26	1.31	1.38	0.91	0.92	0.89	0.82	28.69	8.66
D3YX49	Protein Zfp384 OS=Mus musculus GN=Zfp384 PE=2 SV=1 - [D3YX49_MOUSE]	2.56	5	1	1	2	1.08	1.59	1.46	1.07	0.99	1.17	1.08	1.31	1.21	1.17	1.08	1.51	1.40	50.86	8.56
Q8K2D3	Enhancer of mRNA-decapping protein 3 OS=Mus musculus GN=Edc3 PE=2 SV=1 - [EDC3_MOUSE]	5.71	1	2	2	4	1.15	1.23	1.06	1.16	1.01	1.40	1.21	1.31	1.14	1.22	1.06	1.26	1.09	55.92	7.09
F6S0D5	Protein 0610010K14Rik (Fragment) OS=Mus musculus GN=0610010K14Rik PE=4 SV=1 - [F6S0D5_MOUSE]	20.00	10	2	2	4	1.05	0.89	0.85	0.90	0.85	1.18	1.12	1.31	1.25	1.16	1.10	1.17	1.12	8.53	10.07
Q9JMF3	Guanine nucleotide-binding protein G(I)/G(S)/G(O) subunit gamma-13 OS=Mus musculus GN=Gng13 PE=3 SV=1 - [GBG13_MOUSE]	47.76	1	4	4	19	1.18	0.99	0.87	1.14	0.97	1.51	1.23	1.31	1.13	1.22	1.03	1.25	1.01	7.97	5.45
Q8K2T8	RNA polymerase II-associated factor 1 homolog OS=Mus musculus GN=Paf1 PE=2 SV=1 - [PAF1_MOUSE]	22.24	1	9	9	22	1.03	0.87	0.75	0.88	0.86	1.43	1.30	1.31	1.28	1.28	1.27	1.09	1.15	60.48	4.65
E9Q7P9	Protein Cdhr2 OS=Mus musculus GN=Cdhr2 PE=2 SV=1 - [E9Q7P9_MOUSE]	0.69	1	1	1	5	0.98	1.14	1.17	1.03	1.03	1.23	1.22	1.31	1.37	1.21	1.26	1.34	1.33	142.55	4.44

P62315	Small nuclear ribonucleoprotein Sm D1 OS=Mus musculus GN=Snrpd1 PE=1 SV=1 - [SMD1_MOUSE]	16.81	1	1	1	2	1.06	1.21	1.16	1.22	1.18	1.12	1.04	1.32	1.27	1.27	1.20	1.41	1.36	13.27	11.56
Q8QZT2	Centriole, cilia and spindle-associated protein OS=Mus musculus GN=Ccsap PE=2 SV=1 - [CCSAP_MOUSE]	46.83	1	9	9	28	0.97	0.91	0.86	0.96	0.95	1.29	1.22	1.32	1.25	1.09	1.08	1.07	1.06	28.36	9.22
A6H8H2	DENN domain-containing protein 4C OS=Mus musculus GN=Dennd4c PE=1 SV=1 - [DEN4C_MOUSE]	0.94	2	1	1	2	1.23	1.21	0.98	1.16	0.94	1.24	1.00	1.32	1.07	0.89	0.73	1.09	0.89	211.32	6.40
P60879-2	Isoform 2 of Synaptosomal-associated protein 25 OS=Mus musculus GN=Snap25 - [SNP25_MOUSE]	72.33	1	3	20	1162	0.89	0.86	1.01	1.00	1.15	1.30	1.48	1.32	1.49	1.33	1.55	1.16	1.36	23.32	4.86
Q3TSG4	RNA demethylase ALKBH5 OS=Mus musculus GN=Alkbh5 PE=1 SV=2 - [ALKB5_MOUSE]	4.30	1	1	1	4	1.66	1.04	0.62	1.09	0.66	1.62	0.97	1.32	0.79	1.01	0.61	0.82	0.50	44.38	9.09
Q8CGB3-	Isoform 2 of Uveal autoantigen with coiled-coil domains and ankyrin repeats OS=Mus musculus GN=Uaca - [UACA_MOUSE]	2.96	3	3	3	4	1.06	0.92	0.83	0.98	0.96	1.11	1.00	1.32	1.19	1.22	1.17	1.12	1.06	116.45	7.43
Q7TNV0	Protein DEK OS=Mus musculus GN=Dek PE=1 SV=1 - [DEK_MOUSE]	21.05	3	8	8	17	1.07	0.91	0.89	1.15	1.04	1.24	1.16	1.32	1.13	1.30	1.14	1.16	1.10	43.13	6.86
Q9QXM1-	Isoform 3 of Junction-mediating and -regulatory protein OS=Mus musculus GN=Jmy - [JMY_MOUSE]	1.45	2	1	1	2	1.10	1.14	1.03	1.08	0.98	1.26	1.14	1.32	1.19	1.24	1.13	1.19	1.08	108.64	6.13
Q91W92	Cdc42 effector protein 1 OS=Mus musculus GN=Cdc42ep1 PE=1 SV=1 - [BORG5_MOUSE]	8.56	1	2	2	4	1.02	0.86	0.84	0.94	0.92	1.35	1.32	1.32	1.29	1.15	1.13	1.00	0.98	43.07	7.21
Q91WT4	DnaJ homolog subfamily C member 17 OS=Mus musculus GN=Dnajc17 PE=2 SV=2 - [DJC17_MOUSE]	3.63	1	1	1	2	1.08	1.14	1.06	0.85	0.79	1.07	0.99	1.32	1.22	1.00	0.93	1.14	1.06	34.44	8.57
Q8BFZ3	Beta-actin-like protein 2 OS=Mus musculus GN=Actbl2 PE=1 SV=1 - [ACTBL_MOUSE]	38.56	1	2	12	682	1.20	2.12	1.04	22.53	3.63	1.99	1.65	1.32	1.07	1.30	1.09	1.08	1.09	41.98	5.49
Q9JMC3	DnaJ homolog subfamily A member 4 OS=Mus musculus GN=Dnaja4 PE=2 SV=1 - [DNJA4_MOUSE]	23.68	2	7	8	31	1.04	1.09	1.07	1.02	1.02	1.29	1.26	1.32	1.30	1.17	1.14	1.09	1.14	44.87	7.58

Q61897	Keratin, type I cuticular Ha3-II OS=Mus musculus GN=Krt33b PE=2 SV=2 - [KT33B_MOUSE]	12.62	11	3	5	12	0.98	1.29	1.33	13.50	13.70	1.18	1.13	1.32	1.27	1.54	1.49	1.19	1.20	45.83	4.82
Q3US17	Zinc finger protein 48 OS=Mus musculus GN=Znf48 PE=2 SV=2 - [ZNF48_MOUSE]	5.75	1	2	2	3	1.08	1.27	1.17	1.15	1.06	1.32	1.21	1.32	1.21	1.49	1.38	1.35	1.25	64.56	9.41
F2Z3Y4	Nitrogen permease regulator 3-like protein OS=Mus musculus GN=Nprl3 PE=2 SV=1 - [F2Z3Y4_MOUSE]	23.38	3	1	1	1	0.96	1.66	1.73	0.89	0.92	0.83	0.86	1.32	1.37	1.31	1.37	1.15	1.20	8.56	9.98
Q9DBF7-3	Isoform 3 of Pre-mRNA-splicing factor CWC25 homolog OS=Mus musculus GN=Cwc25 - [CWC25_MOUSE]	3.08	2	1	1	2	0.90	1.08	1.19	0.98	1.09	1.08	1.19	1.32	1.46	1.10	1.22	0.97	1.08	37.66	10.21
Q8VC31	Coiled-coil domain-containing protein 9 OS=Mus musculus GN=Ccdc9 PE=2 SV=1 - [CCDC9_MOUSE]	14.73	5	7	8	22	1.05	1.06	0.98	1.08	1.01	1.24	1.19	1.32	1.21	1.27	1.14	1.14	1.07	61.41	5.00
Q6PHS9-4	Isoform 4 of Voltage-dependent calcium channel subunit alpha-2/delta-2 OS=Mus musculus GN=Cacna2d2 - [CA2D2_MOUSE]	12.64	4	1	13	28	1.30	1.56	1.20	1.05	0.80	1.32	1.01	1.32	1.01	1.41	1.08	1.34	1.03	129.57	5.60
P57784	U2 small nuclear ribonucleoprotein A' OS=Mus musculus GN=Snrpa1 PE=1 SV=2 - [RU2A_MOUSE]	17.25	2	4	4	6	0.96	0.94	0.94	0.99	1.03	1.29	1.31	1.32	1.29	1.23	1.26	1.13	1.12	28.34	8.62
Q76KF0-3	Isoform 2 of Semaphorin-6D OS=Mus musculus GN=Sema6d - [SEM6D_MOUSE]	1.70	7	1	1	1	1.13	1.08	0.95	1.04	0.92	1.00	0.88	1.32	1.16	1.04	0.92	1.06	0.94	111.70	8.66
Q3TFP0	Serine/arginine-rich-splicing factor 10 OS=Mus musculus GN=Srsf10 PE=2 SV=1 - [Q3TFP0_MOUSE]	33.52	4	3	5	37	0.99	1.09	1.07	0.94	0.94	1.21	1.16	1.32	1.31	1.14	1.12	1.14	1.17	22.12	10.33
A6X8Z5	Rho GTPase-activating protein 31 OS=Mus musculus GN=Arhgap31 PE=1 SV=1 - [RHG31_MOUSE]	1.96	4	1	2	3	1.14	1.71	1.50	1.08	0.95	1.05	0.92	1.32	1.16	1.34	1.18	1.19	1.04	155.18	5.77
Q6A044-2	Isoform 2 of Protein FAM189A1 OS=Mus musculus GN=Fam189a1 - [F1891_MOUSE]	10.07	2	1	1	1	1.27	0.41	0.32	0.78	0.62	1.33	1.04	1.32	1.03	0.89	0.70	0.84	0.66	14.73	6.25

Q9CWS0	N(G),N(G)-dimethylarginine dimethylaminohydrolase 1 OS=Mus musculus GN=Ddah1 PE=1 SV=3 - [DDAH1_MOUSE]	60.35	2	16	17	161	0.94	0.82	0.87	0.80	0.88	1.25	1.32	1.32	1.40	1.15	1.15	1.04	1.13	31.36	5.97
Q8BG81	Polymerase delta-interacting protein 3 OS=Mus musculus GN=Poldip3 PE=2 SV=1 - [PDIP3_MOUSE]	28.81	3	9	9	24	1.09	0.98	0.89	0.99	0.89	1.17	1.08	1.32	1.22	1.14	1.04	1.15	1.09	46.10	10.05
Q80U23-2	Isoform 2 of Syntaphilin OS=Mus musculus GN=Snph - [SNPH_MOUSE]	36.36	3	13	14	56	1.02	1.02	0.99	1.03	1.01	1.23	1.21	1.32	1.31	1.19	1.09	1.11	1.05	57.04	6.02
Q810V0	U3 small nucleolar ribonucleoprotein protein MPP10 OS=Mus musculus GN=Mphosph10 PE=1 SV=2 - [MPP10_MOUSE]	3.38	1	2	2	4	0.92	1.55	1.68	1.16	1.26	1.40	1.51	1.32	1.47	1.28	1.39	0.85	1.10	78.69	4.87
Q61103	Zinc finger protein ubi-d4 OS=Mus musculus GN=Dpf2 PE=1 SV=1 - [REQU_MOUSE]	23.53	3	6	6	14	1.07	1.26	1.15	1.15	1.04	1.11	1.19	1.32	1.24	1.28	1.28	1.19	1.16	44.20	6.47
Q3TPR7-2	Isoform 2 of Transmembrane protein 184C OS=Mus musculus GN=Tmem184c - [T184C_MOUSE]	1.93	1	1	1	2	1.13	1.27	1.11	1.08	0.95	1.05	0.92	1.32	1.16	1.27	1.12	1.11	0.98	71.07	5.08
Q3U7U3	F-box only protein 7 OS=Mus musculus GN=Fbxo7 PE=2 SV=2 - [FBX7_MOUSE]	7.46	5	2	2	3	1.79	1.35	0.75	1.00	0.56	1.42	0.79	1.33	0.74	1.22	0.69	1.90	1.06	57.61	5.83
Q9CSN1	SNW domain-containing protein 1 OS=Mus musculus GN=Snw1 PE=1 SV=3 - [SNW1_MOUSE]	37.69	1	14	14	62	1.06	0.88	0.86	0.99	0.95	1.33	1.20	1.33	1.21	1.18	1.08	1.08	1.00	61.44	9.48
Q8K0H5	Transcription initiation factor TFIID subunit 10 OS=Mus musculus GN=Taf10 PE=1 SV=1 - [TAF10_MOUSE]	11.47	1	1	1	5	1.00	1.03	1.07	1.06	1.01	1.38	1.29	1.33	1.19	1.22	1.24	1.18	1.14	21.83	6.55
D3Z6I8	Tropomyosin alpha-3 chain OS=Mus musculus GN=Tpm3 PE=2 SV=1 - [D3Z6I8_MOUSE]	54.66	3	2	22	416	1.11	0.99	0.81	0.97	0.89	1.22	1.09	1.33	1.11	1.16	1.03	1.06	0.96	28.71	4.79
Q8BGZ4-2	Isoform 2 of Cell division cycle protein 23 homolog OS=Mus musculus GN=Cdc23 - [CDC23_MOUSE]	3.55	3	1	1	1	1.04	0.65	0.63	0.86	0.83	1.38	1.32	1.33	1.28	1.24	1.20	1.04	1.00	55.77	7.75
F7D143	Uncharacterized protein KIAA0232 (Fragment) OS=Mus musculus GN=D5Ertd579e PE=2 SV=1 - [F7D143_MOUSE]	1.49	3	1	1	2	1.38	1.27	0.92	1.12	0.82	1.44	1.04	1.33	0.96	1.27	0.92	1.39	1.01	140.95	4.89

P21619	Lamin-B2 OS=Mus musculus GN=Lmnb2 PE=1 SV=2 - [LMNB2_MOUSE]	59.73	2	27	31	176	1.01	0.98	0.99	0.91	0.94	1.25	1.22	1.33	1.32	1.17	1.15	1.14	1.16	67.28	5.50
B1AZP2-2	Isoform 2 of Disks large-associated protein 4 OS=Mus musculus GN=Dlgap4 - [DLGP4_MOUSE]	31.14	10	13	23	77	0.99	1.05	0.93	1.08	1.02	1.26	1.22	1.33	1.22	1.13	1.08	1.12	1.06	107.49	7.30
Q3V1F8	Epoxide hydrolase 3 OS=Mus musculus GN=Ephx3 PE=2 SV=2 - [EPHX3_MOUSE]	1.91	2	1	1	1	0.91	1.16	1.28	0.86	0.95	1.34	1.48	1.33	1.46	1.20	1.33	0.80	0.89	41.83	7.75
D3YV00	Probable phospholipid-transporting ATPase IIB OS=Mus musculus GN=Atp9b PE=4 SV=1 - [D3YV00_MOUSE]	6.78	3	3	4	5	1.21	1.13	0.93	1.22	1.00	1.26	0.99	1.33	0.82	1.10	0.91	1.13	0.93	127.86	7.64
P05627	Transcription factor AP-1 OS=Mus musculus GN=Jun PE=1 SV=3 - [JUN_MOUSE]	6.59	1	1	2	4	1.29	1.23	0.95	1.08	0.84	1.32	1.02	1.33	1.03	1.49	1.16	1.34	1.04	35.92	8.75
D3YXZ3	Kinesin light chain 2 OS=Mus musculus GN=Klc2 PE=4 SV=1 - [D3YXZ3_MOUSE]	46.84	4	16	22	62	0.99	1.03	1.07	0.98	1.03	1.26	1.27	1.33	1.27	1.23	1.23	1.22	1.20	68.13	7.06
A3KGV1-	Isoform 7 of Outer dense fiber protein 2 OS=Mus musculus GN=Odf2 - [ODFP2_MOUSE]	4.10	9	2	2	4	0.94	1.08	1.14	0.99	1.05	1.10	1.16	1.33	1.40	1.19	1.26	1.08	1.15	70.72	6.67
Q8VEJ9	Vacuolar protein sorting-associated protein 4A OS=Mus musculus GN=Vps4a PE=1 SV=1 - [VPS4A_MOUSE]	10.53	1	3	4	7	1.05	0.98	0.94	1.03	0.98	1.32	1.19	1.33	1.33	1.19	1.19	1.24	1.09	48.88	7.80
P57776-3	Isoform 3 of Elongation factor 1-delta OS=Mus musculus GN=Eef1d - [EF1D_MOUSE]	42.58	10	2	23	122	0.96	1.12	1.21	0.96	1.00	1.26	1.24	1.33	1.28	1.24	1.35	1.27	1.32	72.89	6.43
Q9D0T1	NHP2-like protein 1 OS=Mus musculus GN=Nhp2l1 PE=2 SV=4 - [NH2L1_MOUSE]	27.34	2	3	3	6	1.00	1.00	1.04	1.03	1.02	1.35	1.30	1.33	1.36	1.24	1.30	1.24	1.29	14.16	8.46
Q9CQE9	FLYWCH family member 2 OS=Mus musculus GN=Flywch2 PE=2 SV=1 - [FWCH2_MOUSE]	22.30	3	2	2	4	1.22	1.28	1.04	0.94	0.77	1.56	1.27	1.33	1.08	1.23	1.00	1.25	1.03	14.45	8.13
Q9QXS6	Drebrin OS=Mus musculus GN=Dbn1 PE=1 SV=4 - [DREB_MOUSE]	50.57	4	29	29	357	1.01	0.92	0.92	0.96	0.95	1.30	1.31	1.33	1.33	1.19	1.18	1.12	1.11	77.24	4.49
Q8BVF2	Phosducin-like protein 3 OS=Mus musculus GN=Pdcl3 PE=1 SV=1 - [PDCL3_MOUSE]	4.17	1	1	1	5	1.07	0.83	0.80	0.93	0.87	1.21	1.17	1.33	1.16	1.21	1.16	1.15	1.08	27.56	4.72

P30681	High mobility group protein B2 OS=Mus musculus GN=Hmgb2 PE=1 SV=3 - [HMGB2_MOUSE]	28.57	1	5	6	69	0.98	0.84	0.87	1.13	1.15	1.30	1.30	1.33	1.31	1.26	1.31	1.08	1.12	24.15	7.31
E9PVZ8	Protein Golgb1 OS=Mus musculus GN=Golgb1 PE=2 SV=1 - [E9PVZ8_MOUSE]	26.07	4	66	67	144	1.04	0.95	0.90	1.01	0.98	1.28	1.23	1.33	1.25	1.23	1.18	1.09	1.05	369.93	5.05
A2AP88	Zinc finger CCCH domain-containing protein 6 OS=Mus musculus GN=Zc3h6 PE=4 SV=1 - [A2AP88_MOUSE]	3.91	2	3	3	5	1.19	1.12	0.94	1.09	0.95	1.38	1.16	1.33	1.20	1.26	1.17	1.36	1.15	131.18	6.99
Q922M7	Ashwin OS=Mus musculus PE=1 SV=1 - [ASHWN_MOUSE]	38.79	1	7	7	13	1.09	0.92	1.02	0.99	0.90	1.21	1.07	1.33	1.20	1.18	1.08	1.03	0.97	26.01	9.55
Q62241	U1 small nuclear ribonucleoprotein C OS=Mus musculus GN=Snrpc PE=2 SV=1 - [RU1C_MOUSE]	18.87	1	2	2	6	1.11	0.75	0.69	1.06	0.96	1.36	1.18	1.33	1.19	1.13	1.13	1.04	1.00	17.35	9.67
Q9CZA6-	Isoform 2 of Nuclear distribution protein nudE homolog 1 OS=Mus musculus GN=Nde1 - [NDE1_MOUSE]	47.63	6	8	13	30	1.01	1.01	0.88	0.92	0.89	1.28	1.26	1.33	1.32	1.19	1.16	1.21	1.17	35.87	5.19
Q64327	Male-enhanced antigen 1 OS=Mus musculus GN=Mea1 PE=2 SV=1 - [MEA1_MOUSE]	17.24	1	2	2	10	1.04	1.15	1.06	0.98	0.97	1.23	1.13	1.33	1.26	1.26	1.21	1.22	1.17	18.57	4.08
Q9WUR9	GTP:AMP phosphotransferase AK4, mitochondrial OS=Mus musculus GN=Ak4 PE=2 SV=1 - [KAD4_MOUSE]	30.49	3	4	4	10	1.01	1.31	1.21	1.19	1.20	1.35	1.35	1.33	1.26	1.46	1.53	1.27	1.26	25.05	7.53
Q8VD24	Zinc finger CCHC domain-containing protein 18 OS=Mus musculus GN=Zcchc18 PE=2 SV=1 - [ZCC18_MOUSE]	2.80	1	1	1	3	1.74	1.45	0.83	1.60	0.91	1.38	0.79	1.33	0.76	1.19	0.68	1.68	0.96	43.95	5.38
O70480	Vesicle-associated membrane protein 4 OS=Mus musculus GN=Vamp4 PE=1 SV=1 - [VAMP4_MOUSE]	35.46	2	4	4	32	1.10	1.25	1.13	1.11	0.89	1.17	1.09	1.33	1.20	1.20	1.09	1.15	1.02	16.34	7.36
Q8K4J6	MKL/myocardin-like protein 1 OS=Mus musculus GN=Mkl1 PE=1 SV=2 - [MKL1_MOUSE]	10.27	8	6	8	17	1.00	0.88	0.84	0.98	0.93	1.32	1.20	1.33	1.23	1.22	1.17	1.16	1.11	102.48	5.50

Q3ULJ3	MCG3697, isoform CRA_c OS=Mus musculus GN=Upf3a PE=2 SV=1 - [Q3ULJ3_MOUSE]	3.55	1	1	1	2	1.00	1.25	1.25	1.09	1.10	1.26	1.26	1.34	1.34	1.36	1.37	1.42	1.43	48.72	9.35
Q9R0Y5	Adenylate kinase isoenzyme 1 OS=Mus musculus GN=Ak1 PE=1 SV=1 - [KAD1_MOUSE]	78.87	2	17	17	259	0.95	0.91	0.98	0.88	0.93	1.25	1.33	1.34	1.39	1.15	1.21	1.14	1.19	21.53	5.81
Q925F2	Endothelial cell-selective adhesion molecule OS=Mus musculus GN=Esam PE=1 SV=1 - [ESAM_MOUSE]	6.09	1	1	1	7	0.84	0.98	1.14	0.93	1.12	1.18	1.38	1.34	1.37	1.08	1.31	0.90	1.04	41.78	9.26
Q8C5L3-3	Isoform 3 of CCR4-NOT transcription complex subunit 2 OS=Mus musculus GN=Cnot2 - [CNOT2_MOUSE]	3.30	5	1	1	1	0.61	0.93	1.52	0.95	1.55	1.12	1.82	1.34	2.18	1.34	2.20	0.99	1.62	50.19	7.93
E9PV16	Adenosylhomocysteinase OS=Mus musculus GN=Ahcyl2 PE=2 SV=1 - [E9PV16_MOUSE]	29.39	8	1	20	81	1.20	1.23	1.03	1.05	0.87	1.32	1.10	1.34	1.11	1.32	1.10	1.17	0.98	69.93	6.79
Q00420-3	Isoform 3 of GA-binding protein subunit beta-1 OS=Mus musculus GN=Gabpb1 - [GABP1_MOUSE]	2.09	2	1	1	1	0.93	1.24	1.32	1.02	1.09	1.18	1.26	1.34	1.43	1.19	1.28	1.00	1.07	41.23	4.84
Q9CW07	Protein phosphatase 1 regulatory subunit 3G OS=Mus musculus GN=Ppp1r3g PE=2 SV=2 - [PP13G_MOUSE]	4.03	1	1	1	2	0.86	1.02	1.18	1.04	1.21	1.33	1.54	1.34	1.55	1.13	1.32	1.03	1.20	37.80	5.00
Q9D4H9-	Isoform 3 of PHD finger protein 14 OS=Mus musculus GN=Phf14 - [PHF14_MOUSE]	5.13	4	3	3	3	1.06	1.02	0.97	1.04	0.98	1.33	1.25	1.34	1.19	1.30	1.23	1.22	1.16	98.71	5.34
G5E898	Periplakin OS=Mus musculus GN=Ppl PE=4 SV=1 - [G5E898_MOUSE]	0.80	2	1	2	3	0.96	1.14	1.18	0.93	0.97	1.18	1.21	1.34	1.38	1.29	1.34	1.19	1.23	203.66	5.48
Q9QX66-	Isoform 3 of Zinc finger protein neuro-d4 OS=Mus musculus GN=Dpf1 - [DPF1_MOUSE]	23.49	11	5	5	11	1.04	1.03	0.96	1.10	1.08	1.29	1.32	1.34	1.28	1.27	1.19	1.29	1.20	37.93	7.23
Q6P6J9	Thioredoxin domain-containing protein 15 OS=Mus musculus GN=Txndc15 PE=1 SV=1 - [TXD15_MOUSE]	10.76	1	2	2	4	1.02	1.05	1.03	1.03	1.01	1.07	1.04	1.34	1.30	1.27	1.24	1.07	1.05	38.12	4.77
H3BLE1	Metastasis-associated protein MTA3 (Fragment) OS=Mus musculus GN=Mta3 PE=2 SV=1 - [H3BLE1_MOUSE]	8.86	6	2	2	3	1.45	1.32	1.23	1.04	0.97	0.97	0.90	1.34	0.92	1.34	0.93	1.38	0.96	30.44	7.30

Q8CI70	Leucine-rich repeat-containing protein 20 OS=Mus musculus GN=Lrrc20 PE=2 SV=1 - [LRC20_MOUSE]	5.98	1	1	1	2	1.01	1.44	1.42	1.15	1.14	1.34	1.32	1.34	1.31	1.26	1.25	1.18	1.17	20.80	5.66
Q99LX0	Protein DJ-1 OS=Mus musculus GN=Park7 PE=1 SV=1 - [PARK7_MOUSE]	65.08	5	10	10	55	0.94	0.87	0.91	0.86	0.96	1.29	1.24	1.34	1.33	1.18	1.24	1.07	1.15	20.01	6.77
Q99N93	39S ribosomal protein L16, mitochondrial OS=Mus musculus GN=Mrpl16 PE=2 SV=1 - [RM16_MOUSE]	6.37	1	1	1	1	1.27	1.66	1.31	1.07	0.84	1.12	0.88	1.34	1.05	1.17	0.92	1.34	1.06	28.79	10.27
E0CZ90	Proteasome activator complex subunit 2 (Fragment) OS=Mus musculus GN=Psme2 PE=2 SV=1 - [E0CZ90_MOUSE]	13.94	3	2	2	2	1.74	1.16	0.66	1.76	1.01	1.12	0.64	1.34	0.76	1.33	0.77	1.57	0.90	18.67	4.88
Q62414	Neurogenic differentiation factor 2 OS=Mus musculus GN=Neurod2 PE=1 SV=3 - [NDF2_MOUSE]	3.39	1	1	1	2	1.01	1.75	1.73	1.03	1.02	1.36	1.34	1.34	1.32	1.11	1.10	1.22	1.21	41.47	6.76
D3Z6H3	Dynactin 6, isoform CRA_b OS=Mus musculus GN=Dctn6 PE=4 SV=1 - [D3Z6H3_MOUSE]	27.57	5	4	4	16	0.93	0.78	0.93	0.88	0.99	1.30	1.40	1.34	1.48	1.17	1.32	1.11	1.17	20.03	6.38
O88811	Signal transducing adapter molecule 2 OS=Mus musculus GN=Stam2 PE=1 SV=1 - [STAM2_MOUSE]	21.22	3	7	9	24	0.98	0.90	0.92	0.93	1.01	1.33	1.26	1.34	1.39	1.24	1.21	1.17	1.05	57.42	5.07
Q6P5H2	Nestin OS=Mus musculus GN=Nes PE=1 SV=1 - [NEST_MOUSE]	13.14	2	13	13	29	0.99	0.98	1.08	0.91	0.89	1.26	1.29	1.34	1.40	1.20	1.28	1.03	1.16	207.00	4.34
Q9JIK9	28S ribosomal protein S34, mitochondrial OS=Mus musculus GN=Mrps34 PE=2 SV=1 - [RT34_MOUSE]	36.70	1	6	6	15	1.01	1.07	1.14	1.02	1.02	1.31	1.09	1.34	1.16	1.25	1.22	1.16	1.12	25.81	10.43
Q9Z1P6	NADH dehydrogenase [ubiquinone] 1 alpha subcomplex subunit 7 OS=Mus musculus GN=Ndufa7 PE=1 SV=3 - [NDUA7_MOUSE]	60.18	1	8	8	50	1.00	0.82	0.82	0.92	0.92	1.32	1.30	1.34	1.30	1.16	1.18	1.15	1.18	12.57	10.17
E9PYF7	AP1AR OS=Mus musculus GN=Ap1ar PE=4 SV=1 - [E9PYF7_MOUSE]	11.41	1	2	2	4	0.81	1.04	1.37	0.78	0.83	1.20	1.48	1.34	1.23	0.98	1.21	0.84	1.23	33.76	4.87

Q8BU85	Methionine-R-sulfoxide reductase B3, mitochondrial OS=Mus musculus GN=Msrb3 PE=1 SV=2 - [MSRB3_MOUSE]	4.74	1	1	1	2	0.94	0.77	0.82	0.88	0.94	1.23	1.31	1.34	1.43	1.04	1.12	0.97	1.04	26.82	7.99
Q7TPS5-2	Isoform 2 of C2 domain-containing protein 5 OS=Mus musculus GN=C2cd5 - [C2CD5_MOUSE]	2.63	4	2	2	3	0.94	1.59	1.68	1.09	1.16	1.11	1.17	1.34	1.42	1.16	1.23	1.72	1.83	108.80	6.05
P04370	Myelin basic protein OS=Mus musculus GN=Mbp PE=1 SV=2 - [MBP_MOUSE]	60.40	3	2	18	975	1.03	1.23	1.19	1.27	1.23	1.43	1.37	1.34	1.29	1.54	1.50	1.22	1.18	27.15	9.58
Q8K124	Pleckstrin homology domain-containing family O member 2 OS=Mus musculus GN=Plekho2 PE=1 SV=1 - [PKHO2_MOUSE]	23.03	1	6	6	11	0.96	1.01	1.13	0.82	0.87	1.19	1.28	1.34	1.40	1.08	1.17	0.94	0.98	53.84	5.50
O08583	THO complex subunit 4 OS=Mus musculus GN=Alyref PE=1 SV=3 - [THOC4_MOUSE]	39.22	3	5	7	55	1.10	1.03	0.96	1.03	1.03	1.21	1.22	1.34	1.31	1.31	1.20	1.13	1.12	26.92	11.15
Q9QZ59-2	Isoform 2 of Doublesex- and mab-3-related transcription factor 1 OS=Mus musculus GN=Dmrt1 - [DMRT1_MOUSE]	15.57	3	1	1	2	1.04	1.03	0.99	0.60	0.58	1.36	1.30	1.34	1.29	1.15	1.11	1.61	1.56	21.88	8.90
Q8BHG9	CGG triplet repeat-binding protein 1 OS=Mus musculus GN=Cggbp1 PE=2 SV=1 - [CGBP1_MOUSE]	25.75	1	3	3	6	1.06	0.84	0.80	1.17	1.11	1.21	1.07	1.34	1.24	1.21	1.16	1.11	1.07	18.75	8.95
Q8VE88-2	Isoform 2 of Protein FAM114A2 OS=Mus musculus GN=Fam114a2 - [F1142_MOUSE]	25.31	2	7	7	10	0.94	0.78	0.85	0.97	0.99	1.32	1.40	1.34	1.36	1.28	1.36	1.09	1.09	53.34	4.89
Q8K3X4	Interferon regulatory factor 2-binding protein-like OS=Mus musculus GN=Irf2bpl PE=1 SV=1 - [I2BPL_MOUSE]	20.65	1	8	10	23	0.93	0.94	1.00	0.93	0.97	1.28	1.32	1.34	1.35	1.22	1.29	1.15	1.23	80.51	8.24
O09000	Nuclear receptor coactivator 3 OS=Mus musculus GN=Ncoa3 PE=1 SV=2 - [NCOA3_MOUSE]	1.00	3	1	1	1	0.97	0.94	0.97	1.07	1.10	1.08	1.11	1.34	1.38	1.26	1.30	0.94	0.97	151.48	7.77
Q9CQY2	RNMT-activating mini protein OS=Mus musculus GN=Fam103a1 PE=2 SV=1 - [RAM_MOUSE]	21.01	3	2	2	22	0.98	1.13	1.18	0.95	0.99	1.14	1.20	1.34	1.39	1.22	1.23	1.15	1.16	14.55	8.40

E9PV69	CDK2-associated and cullin domain-containing protein 1 OS=Mus musculus GN=Cacul1 PE=2 SV=1 - [E9PV69_MOUSE]	7.49	3	2	2	4	0.91	1.04	1.13	1.03	1.13	1.14	1.24	1.34	1.46	1.22	1.34	1.36	1.49	38.81	5.25
P28653	Biglycan OS=Mus musculus GN=Bgn PE=2 SV=1 - [PGS1_MOUSE]	12.47	1	3	3	7	1.00	1.22	1.22	1.09	1.04	1.40	1.30	1.34	1.34	1.62	1.55	1.32	1.18	41.61	7.27
Q3UD01	Putative ataxin-7-like protein 3B OS=Mus musculus GN=Atxn7l3b PE=3 SV=1 - [A7L3B_MOUSE]	20.62	1	1	1	2	0.93	0.95	1.02	0.79	0.85	0.87	0.93	1.34	1.44	0.81	0.87	0.92	0.98	10.72	4.32
E9Q784	Protein Zc3h13 OS=Mus musculus GN=Zc3h13 PE=2 SV=1 - [E9Q784_MOUSE]	4.80	1	6	6	14	1.03	1.13	0.95	0.97	0.93	1.31	1.12	1.34	1.15	1.27	1.15	1.25	1.17	203.64	9.38
Q8R1B0	SH3 and cysteine-rich domain-containing protein 2 OS=Mus musculus GN=Stac2 PE=2 SV=1 - [STAC2_MOUSE]	12.01	3	4	4	11	0.94	0.97	0.99	1.03	1.07	1.28	1.32	1.34	1.36	1.04	1.10	0.85	0.88	44.76	7.75
P59648	FXYD domain-containing ion transport regulator 7 OS=Mus musculus GN=Fxyd7 PE=1 SV=1 - [FXYD7_MOUSE]	20.00	1	1	1	1	1.12	0.94	0.83	0.91	0.81	1.15	1.02	1.35	1.20	1.14	1.02	1.19	1.06	8.48	7.84
F7AA26	Protein Gm20459 (Fragment) OS=Mus musculus GN=Gm20459 PE=4 SV=1 - [F7AA26_MOUSE]	33.51	6	1	29	133	1.15	1.13	0.98	0.96	0.83	1.23	1.07	1.35	1.17	1.23	1.07	1.22	1.06	126.01	5.27
O54724	Polymerase I and transcript release factor OS=Mus musculus GN=Ptrf PE=1 SV=1 - [PTRF_MOUSE]	28.32	1	8	8	83	0.95	0.91	0.97	0.86	0.91	1.17	1.23	1.35	1.55	1.07	1.18	0.88	0.97	43.93	5.52
Q9CQV1	Mitochondrial import inner membrane translocase subunit TIM16 OS=Mus musculus GN=Pam16 PE=2 SV=1 - [TIM16_MOUSE]	19.20	1	2	2	8	1.01	0.92	0.95	1.06	1.02	1.41	1.14	1.35	1.31	1.33	1.13	1.28	1.10	13.78	9.64
D3Z3N9	Neuronal tyrosine-phosphorylated phosphoinositide-3-kinase adapter 2 OS=Mus musculus GN=Nyap2 PE=2 SV=1 - [D3Z3N9_MOUSE]	22.73	4	1	11	44	1.05	1.02	0.97	0.96	0.91	1.29	1.22	1.35	1.27	1.10	1.04	1.08	1.03	73.84	8.65
Q7TPG7	Protein FAM19A2 OS=Mus musculus GN=Fam19a2 PE=2 SV=1 - [F19A2_MOUSE]	20.61	2	2	2	6	1.16	1.20	1.04	1.07	0.90	1.37	1.14	1.35	1.12	1.19	0.99	1.30	1.13	14.64	9.17
Q9JLB2	MAGUK p55 subfamily member 5 OS=Mus musculus GN=Mpp5 PE=1 SV=1 - [MPP5_MOUSE]	16.74	1	8	8	15	0.93	1.16	1.19	0.92	1.00	1.25	1.34	1.35	1.36	1.24	1.35	1.17	1.25	77.18	6.09

Q3UZV7-	Isoform 2 of UPF0577 protein KIAA1324-like homolog OS=Mus musculus - [K132L_MOUSE]	3.06	7	2	2	2	1.07	1.11	1.03	1.32	1.23	1.17	1.09	1.35	1.26	1.23	1.15	1.08	1.01	111.96	6.16
O35153	BET1-like protein OS=Mus musculus GN=Bet1l PE=3 SV=1 - [BET1L_MOUSE]	25.23	1	2	2	4	1.04	1.18	1.13	1.08	1.04	1.26	1.21	1.35	1.30	1.12	1.08	1.17	1.13	12.42	8.82
Q571K4	TGF-beta-activated kinase 1 and MAP3K7-binding protein 3 OS=Mus musculus GN=Tab3 PE=1 SV=2 - [TAB3_MOUSE]	11.17	1	5	5	11	0.92	0.93	1.01	0.89	1.01	1.29	1.35	1.35	1.49	1.23	1.30	1.12	1.21	78.98	8.50
O55074	A-kinase anchor protein 7 isoform alpha OS=Mus musculus GN=Akap7 PE=1 SV=4 - [AKA7A_MOUSE]	38.27	2	5	5	47	1.05	1.07	1.01	0.99	0.95	1.41	1.32	1.35	1.25	1.24	1.17	1.09	1.03	9.16	5.27
Q8BMD5-	Isoform 6 of Cytidine and dCMP deaminase domain-containing protein 1 OS=Mus musculus GN=Cdadc1 - [CDAC1_MOUSE]	3.66	6	1	1	1	1.30	0.93	0.72	1.21	0.93	1.31	1.00	1.35	1.04	1.27	0.98	0.93	0.71	43.35	7.05
Q9D067-4	Isoform 4 of Nuclear protein MDM1 OS=Mus musculus GN=Mdm1 - [MDM1_MOUSE]	3.92	7	2	2	5	1.13	0.96	0.80	1.04	0.90	1.10	0.98	1.36	1.12	1.41	1.18	1.14	0.95	74.51	9.33
H9H9S3	Zinc finger E-box-binding homeobox 2 OS=Mus musculus GN=Zeb2 PE=2 SV=1 - [H9H9S3_MOUSE]	9.31	2	8	9	19	1.08	1.14	1.10	0.93	0.87	1.37	1.20	1.36	1.27	1.18	1.11	1.14	1.08	136.40	6.38
Q2EMV9	Poly [ADP-ribose] polymerase 14 OS=Mus musculus GN=Parp14 PE=1 SV=3 - [PAR14_MOUSE]	0.83	2	1	2	3	1.08	0.92	0.85	1.17	1.08	0.94	0.87	1.36	1.26	1.31	1.22	1.57	1.46	203.67	6.79
Q9DB52	Protein FAM122A OS=Mus musculus GN=Fam122a PE=1 SV=1 - [F122A_MOUSE]	21.48	1	4	4	10	1.23	1.32	1.18	1.02	0.93	1.36	1.09	1.36	1.09	1.10	1.07	1.07	0.92	30.25	6.79
E9Q309	Protein Cep350 OS=Mus musculus GN=Cep350 PE=2 SV=1 - [E9Q309_MOUSE]	0.81	1	1	3	6	1.07	1.19	1.12	1.03	0.97	1.21	1.13	1.36	1.27	1.35	1.27	1.24	1.16	346.24	6.35
Q9D824-3	Isoform 3 of Pre-mRNA 3'-end-processing factor FIP1 OS=Mus musculus GN=Fip1l1 - [FIP1_MOUSE]	35.82	7	12	12	66	1.02	0.96	0.95	0.98	1.00	1.29	1.27	1.36	1.28	1.19	1.23	1.17	1.13	60.15	5.57
Q8VE65	Transcription initiation factor TFIID subunit 12 OS=Mus musculus GN=Taf12 PE=1 SV=1 - [TAF12_MOUSE]	11.80	2	2	2	4	0.96	0.82	0.86	0.86	0.90	1.22	1.27	1.36	1.41	1.19	1.24	1.05	1.10	17.86	7.99

E9Q5L7	PHD finger protein 10 OS=Mus musculus GN=Phf10 PE=2 SV=2 - [E9Q5L7_MOUSE]	4.27	4	1	1	1	1.20	1.14	0.95	1.10	0.92	1.23	1.02	1.36	1.13	1.19	0.99	1.19	1.00	26.88	5.94
Q9D0V7	Receptor-binding cancer antigen expressed on SiSo cells OS=Mus musculus GN=Ebag9 PE=1 SV=2 - [RCAS1_MOUSE]	16.90	1	2	2	4	1.10	1.27	0.98	1.32	1.16	1.59	1.32	1.36	1.23	1.59	1.46	1.47	1.27	24.30	6.29
Q8BJ05	Zinc finger CCCH domain-containing protein 14 OS=Mus musculus GN=Zc3h14 PE=1 SV=1 - [ZC3HE_MOUSE]	27.48	2	2	14	31	0.96	0.95	0.71	0.95	0.99	1.27	1.25	1.36	1.19	1.18	1.26	1.04	1.13	82.36	7.37
P23506	Protein-L-isoaspartate(D-aspartate) O-methyltransferase OS=Mus musculus GN=Pcmt1 PE=1 SV=3 - [PIMT_MOUSE]	63.44	7	13	13	69	0.95	0.80	0.87	0.97	1.05	1.39	1.42	1.36	1.39	1.25	1.29	1.13	1.22	24.62	7.65
O35085	Homeobox protein ARX OS=Mus musculus GN=Arx PE=2 SV=3 - [ARX_MOUSE]	2.66	1	1	1	2	1.10	1.64	1.49	1.20	1.09	1.22	1.11	1.36	1.23	1.29	1.18	1.47	1.34	58.45	5.24
Q9CXG3	Peptidyl-prolyl cis-trans isomerase-like 4 OS=Mus musculus GN=Ppil4 PE=2 SV=2 - [PPIL4_MOUSE]	11.79	2	4	4	8	1.05	1.09	0.96	0.97	0.90	1.20	1.10	1.36	1.20	1.08	1.03	1.07	0.96	57.19	6.11
P36993	Protein phosphatase 1B OS=Mus musculus GN=Ppm1b PE=2 SV=1 - [PPM1B_MOUSE]	30.26	5	2	10	32	0.88	0.88	1.01	0.92	1.05	1.04	1.18	1.36	1.55	1.09	1.25	0.96	1.10	42.77	5.19
Q9JIS8	Solute carrier family 12 member 4 OS=Mus musculus GN=Slc12a4 PE=1 SV=2 - [S12A4_MOUSE]	4.61	2	1	4	8	1.11	1.11	1.00	1.08	0.97	0.91	0.81	1.36	1.23	1.21	1.09	1.71	1.54	120.55	6.67
Q8BM39-	Isoform 2 of Pre-mRNA-splicing factor 18 OS=Mus musculus GN=Prpf18 - [PRP18_MOUSE]	6.86	2	1	1	1	0.97	1.13	1.16	0.92	0.95	1.22	1.25	1.36	1.40	1.20	1.24	1.25	1.30	20.66	4.98
G3X9I4	Aly/REF export factor 2 OS=Mus musculus GN=Alyref2 PE=4 SV=1 - [G3X9I4_MOUSE]	18.35	2	1	3	22	1.15	0.85	0.74	0.93	0.81	1.22	1.06	1.36	1.18	1.29	1.12	1.28	1.11	23.77	10.14
Q61245-2	Isoform Short of Collagen alpha-1(XI) chain OS=Mus musculus GN=Col11a1 - [COBA1_MOUSE]	0.52	2	1	1	2	1.11	1.13	1.02	1.08	0.98	2.05	1.84	1.36	1.23	1.53	1.38	1.22	1.10	171.30	5.66
Q60596	DNA repair protein XRCC1 OS=Mus musculus GN=Xrcc1 PE=1 SV=2 - [XRCC1_MOUSE]	5.07	1	3	3	4	0.96	0.88	0.83	0.83	0.88	1.33	1.20	1.36	1.23	1.15	1.05	1.15	1.04	68.93	6.33

Q99MR1	PERQ amino acid-rich with GYF domain-containing protein 1 OS=Mus musculus GN=Gigyf1 PE=1 SV=2 - [PERQ1_MOUSE]	11.40	1	11	11	23	1.04	1.04	0.95	1.00	1.00	1.30	1.14	1.36	1.18	1.24	1.10	1.17	1.04	116.17	5.39
O08599-2	Isoform 2 of Syntaxin-binding protein 1 OS=Mus musculus GN=Stxbp1 - [STXB1_MOUSE]	74.13	1	2	44	492	0.91	1.47	1.62	1.14	1.26	1.28	1.40	1.36	1.50	1.16	1.28	1.11	1.22	68.69	6.77
Q3UHX0	Nucleolar protein 8 OS=Mus musculus GN=Nol8 PE=1 SV=2 - [NOL8_MOUSE]	3.84	3	3	3	5	1.03	0.88	0.85	1.08	0.87	1.17	1.13	1.37	1.32	1.22	1.18	1.12	1.05	128.56	6.55
E9Q3G8	Protein Nup153 OS=Mus musculus GN=Nup153 PE=2 SV=1 - [E9Q3G8_MOUSE]	17.65	1	20	20	46	0.98	0.96	0.98	0.99	1.00	1.30	1.37	1.37	1.42	1.25	1.28	1.10	1.19	151.91	8.84
O35685	Nuclear migration protein nudC OS=Mus musculus GN=Nudc PE=1 SV=1 - [NUDC_MOUSE]	56.63	1	17	17	43	0.98	0.80	0.84	0.93	0.96	1.35	1.34	1.37	1.34	1.23	1.24	1.10	1.12	38.33	5.26
Q99JX1	Transcription initiation factor TFIID subunit 11 OS=Mus musculus GN=Taf11 PE=2 SV=1 - [TAF11_MOUSE]	12.80	1	1	1	2	1.14	1.09	0.96	0.63	0.55	1.37	1.20	1.37	1.07	1.25	0.99	1.08	0.85	23.32	5.24
Q8VD37-8	Isoform 7 of SH3-containing GRB2-like protein 3-interacting protein 1 OS=Mus musculus GN=Sgip1 - [SGIP1_MOUSE]	49.76	6	1	29	151	1.06	1.48	1.40	0.84	0.79	1.01	0.95	1.37	1.29	1.25	1.18	1.24	1.18	88.50	7.47
Q6NXI6-2	Isoform 2 of Regulation of nuclear pre-mRNA domain-containing protein 2 OS=Mus musculus GN=Rprd2 - [RPRD2_MOUSE]	6.04	2	5	5	8	1.05	0.97	0.91	0.98	0.96	1.27	1.15	1.37	1.15	1.38	1.24	1.36	1.24	151.31	6.93
Q80Y83-1	Isoform 11 of Dixin OS=Mus musculus GN=Dixdc1 - [DIXC1_MOUSE]	17.72	15	6	6	18	0.97	1.00	1.02	1.03	1.06	1.40	1.39	1.37	1.45	1.30	1.42	1.23	1.21	53.99	5.60
Q9CPT5	Nucleolar protein 16 OS=Mus musculus GN=Nop16 PE=2 SV=1 - [NOP16_MOUSE]	3.93	2	1	1	1	1.07	1.17	1.09	1.09	1.02	1.32	1.23	1.37	1.28	1.11	1.04	1.02	0.96	21.13	9.91
Q9JJR9	Nuclear receptor-interacting protein 3 OS=Mus musculus GN=Nrip3 PE=2 SV=1 - [NRIP3_MOUSE]	5.00	2	1	1	2	0.83	0.67	0.81	0.87	1.04	1.45	1.73	1.37	1.64	1.20	1.45	1.05	1.27	26.86	8.50
Q7TSF4	Leucine-rich repeat-containing protein FAM211A OS=Mus musculus GN=Fam211a PE=1 SV=1 - [F211A_MOUSE]	12.68	2	3	3	5	0.85	1.29	1.64	0.97	1.13	1.33	1.52	1.37	1.93	1.14	1.39	1.22	1.43	37.55	8.76

Q08890	Iduronate 2-sulfatase OS=Mus musculus GN=Ids PE=2 SV=3 - [IDS_MOUSE]	3.44	1	1	1	1	1.05	1.30	1.23	1.41	1.34	0.75	0.71	1.37	1.30	1.29	1.23	1.49	1.42	62.15	5.80
P17742	Peptidyl-prolyl cis-trans isomerase A OS=Mus musculus GN=Ppia PE=1 SV=2 - [PPIA_MOUSE]	80.49	3	16	17	687	0.94	0.79	0.91	0.85	0.94	1.27	1.34	1.37	1.39	1.17	1.21	1.10	1.23	17.96	7.90
Q60778	NF-kappa-B inhibitor beta OS=Mus musculus GN=Nfkbib PE=1 SV=2 - [IKBB_MOUSE]	4.46	1	1	1	1	0.90	1.05	1.16	1.01	1.12	1.39	1.54	1.37	1.52	1.26	1.40	1.20	1.34	37.94	4.74
Q6A068	Cell division cycle 5-like protein OS=Mus musculus GN=Cdc5l PE=1 SV=2 - [CDC5L_MOUSE]	31.05	1	15	15	40	0.92	0.90	0.99	1.03	1.05	1.32	1.43	1.37	1.44	1.18	1.29	1.07	1.15	92.13	8.02
Q4V9Z5	Seizure 6-like protein 2 OS=Mus musculus GN=Sez6l2 PE=2 SV=1 - [SE6L2_MOUSE]	20.11	1	1	13	47	1.09	1.28	1.17	1.09	1.00	1.12	1.03	1.37	1.26	1.28	1.18	1.27	1.17	97.44	4.87
Q505H4-3	Isoform 3 of von Willebrand factor C domain-containing protein 2-like OS=Mus musculus GN=Vwc2l - [VWC2L_MOUSE]	38.10	5	3	3	5	1.14	0.95	0.82	1.17	0.98	1.19	1.09	1.38	1.17	1.03	0.92	1.02	0.88	14.32	6.84
Q9DCD5	Tight junction-associated protein 1 OS=Mus musculus GN=Tjap1 PE=1 SV=1 - [TJAP1_MOUSE]	2.23	1	1	1	2	1.02	0.99	0.97	0.96	0.94	1.29	1.26	1.38	1.34	1.27	1.25	1.09	1.07	59.38	6.07
D3YUY0	Carbohydrate sulfotransferase 12 (Fragment) OS=Mus musculus GN=Chst12 PE=2 SV=1 - [D3YUY0_MOUSE]	10.11	2	1	1	1	2.12	1.72	0.81	1.65	0.78	2.14	1.01	1.38	0.65	1.35	0.64	1.22	0.58	20.56	6.27
D3Z7E1	RING finger and transmembrane domain-containing protein 2 (Fragment) OS=Mus musculus GN=Rnft2 PE=2 SV=1 - [D3Z7E1_MOUSE]	34.48	4	1	1	2	1.11	1.13	1.02	1.12	1.00	1.34	1.20	1.38	1.24	1.42	1.28	1.01	0.91	6.38	7.43
Q80TG9	Leucine-rich repeat and fibronectin type-III domain-containing protein 2 OS=Mus musculus GN=Lrfn2 PE=1 SV=2 - [LRFN2_MOUSE]	5.08	1	2	2	4	1.22	1.09	0.88	0.87	0.71	1.41	1.14	1.38	1.12	1.17	0.96	1.19	0.98	84.91	6.74
Q8R409	Protein HEXIM1 OS=Mus musculus GN=Hexim1 PE=1 SV=1 - [HEXI1_MOUSE]	30.34	1	7	7	15	0.99	1.03	1.04	1.06	1.02	1.31	1.29	1.38	1.41	1.30	1.33	1.23	1.17	40.22	5.38

P62915	Transcription initiation factor IIB OS=Mus musculus GN=Gtf2b PE=1 SV=1 - [TF2B_MOUSE]	12.97	1	3	3	5	0.99	0.92	0.93	1.21	1.08	1.13	1.00	1.38	1.40	1.26	1.24	1.25	1.15	34.80	8.35
Q8BHJ9	Pre-mRNA-splicing factor SLU7 OS=Mus musculus GN=Slu7 PE=1 SV=1 - [SLU7_MOUSE]	9.57	2	4	4	16	1.09	1.05	0.95	1.04	0.91	1.34	1.20	1.38	1.26	1.17	1.12	1.10	1.02	68.04	7.08
Q09XV5-	Isoform 2 of Chromodomain-helicase-DNA-binding protein 8 OS=Mus musculus GN=Chd8 - [CHD8_MOUSE]	2.26	3	1	1	2	0.95	1.41	1.48	1.00	1.05	1.21	1.26	1.38	1.44	1.26	1.32	1.39	1.46	80.90	7.12
P11087	Collagen alpha-1(I) chain OS=Mus musculus GN=Col1a1 PE=1 SV=4 - [CO1A1_MOUSE]	8.95	2	9	9	25	1.07	0.96	0.91	1.03	0.94	3.00	2.67	1.38	1.36	1.49	1.32	1.15	1.09	137.95	5.85
Q8K3I4	Rab effector MyRIP OS=Mus musculus GN=Myrip PE=1 SV=1 - [MYRIP_MOUSE]	5.96	1	3	3	6	1.14	0.96	0.84	1.16	1.01	1.40	1.22	1.39	1.23	1.27	1.11	1.15	1.01	94.86	6.07
Q91YN9	BAG family molecular chaperone regulator 2 OS=Mus musculus GN=Bag2 PE=1 SV=1 - [BAG2_MOUSE]	16.67	1	4	4	9	0.95	1.07	1.17	0.90	0.97	1.28	1.36	1.39	1.54	1.26	1.35	1.20	1.39	23.46	6.42
Q8BQ30	Phostensin OS=Mus musculus GN=Ppp1r18 PE=1 SV=1 - [PPR18_MOUSE]	8.42	2	3	3	9	1.09	0.82	0.85	0.91	0.88	1.35	1.25	1.39	1.38	1.38	1.34	1.13	1.04	65.59	5.39
E9PZD2	Protein Micall2 OS=Mus musculus GN=Micall2 PE=2 SV=1 - [E9PZD2_MOUSE]	1.62	2	1	1	1	0.93	1.05	1.12	0.95	1.02	1.35	1.44	1.39	1.48	1.42	1.53	1.56	1.67	98.73	9.76
Q8VEE1	LIM and cysteine-rich domains protein 1 OS=Mus musculus GN=Lmcd1 PE=1 SV=1 - [LMCD1_MOUSE]	30.68	1	9	9	23	0.99	0.83	0.93	0.94	0.92	1.40	1.30	1.39	1.38	1.21	1.24	1.27	1.15	40.97	7.93
P47880	Insulin-like growth factor-binding protein 6 OS=Mus musculus GN=Igfbp6 PE=2 SV=2 - [IBP6_MOUSE]	6.72	1	2	2	5	1.00	1.13	1.08	1.01	0.96	1.27	1.25	1.39	1.31	1.19	1.08	1.04	1.04	25.33	8.19
Q08091	Calponin-1 OS=Mus musculus GN=Cnn1 PE=2 SV=1 - [CNN1_MOUSE]	25.93	2	6	6	12	1.03	0.87	1.05	0.88	0.95	1.03	1.00	1.39	1.37	1.74	1.64	1.03	1.02	33.33	8.97
A2AWK6	Zinc finger protein ZIC 3 OS=Mus musculus GN=Zic3 PE=2 SV=1 - [A2AWK6_MOUSE]	5.69	6	1	1	2	0.99	0.87	0.88	1.18	1.19	1.22	1.22	1.39	1.40	1.18	1.19	1.11	1.12	27.83	8.98
E9Q722	Upstream stimulatory factor 1 OS=Mus musculus GN=Usf1 PE=2 SV=1 - [E9Q722_MOUSE]	13.18	2	3	3	4	1.00	0.86	0.89	0.97	0.98	1.40	1.40	1.39	1.44	1.41	1.42	1.23	1.23	32.00	5.50

Q3V113	Protein Pygo2 OS=Mus musculus GN=Pygo2 PE=2 SV=1 - [Q3V113_MOUSE]	10.87	2	2	2	4	0.96	1.00	1.03	1.01	1.05	1.30	1.34	1.39	1.44	1.08	1.13	1.14	1.19	37.51	6.52
Q9D1T5	Proline-rich protein 15 OS=Mus musculus GN=Prr15 PE=2 SV=1 - [PRR15_MOUSE]	37.70	1	2	2	3	1.09	1.12	1.02	1.08	0.99	1.23	1.12	1.39	1.27	1.18	1.08	1.07	0.98	13.65	8.13
B7ZCA7	Kazrin (Fragment) OS=Mus musculus GN=Kazn PE=2 SV=1 - [B7ZCA7_MOUSE]	42.15	1	1	8	21	0.86	1.12	1.30	0.95	1.11	1.21	1.40	1.39	1.61	1.40	1.64	1.22	1.42	29.61	8.54
Q922U2	Keratin, type II cytoskeletal 5 OS=Mus musculus GN=Krt5 PE=1 SV=1 - [K2C5_MOUSE]	7.59	7	1	5	10	1.16	1.39	1.20	2.22	1.92	0.96	0.83	1.39	1.20	1.23	1.07	1.88	1.63	61.73	7.75
G3UYW9	Protein Trak2 OS=Mus musculus GN=Trak2 PE=2 SV=1 - [G3UYW9_MOUSE]	7.59	2	4	4	6	0.93	0.76	0.94	0.79	0.90	1.17	1.32	1.39	1.42	1.03	1.11	0.88	1.04	75.65	4.93
Q62165	Dystroglycan OS=Mus musculus GN=Dag1 PE=1 SV=4 - [DAG1_MOUSE]	17.47	1	12	12	52	1.09	0.77	0.76	0.95	0.87	1.44	1.28	1.39	1.27	1.28	1.14	1.14	1.02	96.84	8.44
Q6NZA9	Transcription initiation factor TFIID subunit 9B OS=Mus musculus GN=Taf9b PE=1 SV=2 - [TAF9B_MOUSE]	3.21	3	1	1	2	1.06	0.89	0.85	0.61	0.58	0.98	0.92	1.40	1.32	0.84	0.79	0.75	0.71	27.16	9.58
Q7TPE5	Probable RNA polymerase II nuclear localization protein SLC7A6OS OS=Mus musculus GN=Slc7a6os PE=1 SV=1 - [S7A6O_MOUSE]	12.09	1	3	3	9	1.11	1.11	0.99	1.07	0.97	1.34	1.25	1.40	1.23	1.29	1.21	1.18	1.06	35.02	4.58
D3YYC2	Dr1-associated corepressor OS=Mus musculus GN=Drap1 PE=2 SV=1 - [D3YYC2_MOUSE]	20.37	5	2	2	6	1.21	1.21	1.00	1.07	0.88	1.55	1.16	1.40	1.23	1.31	1.18	1.22	1.09	17.58	5.44
F6XLQ6	FERM domain-containing protein 5 (Fragment) OS=Mus musculus GN=Frmd5 PE=4 SV=1 - [F6XLQ6_MOUSE]	15.53	9	1	1	1	1.46	1.10	0.75	1.76	1.20	1.45	0.99	1.40	0.96	1.46	1.01	1.81	1.24	22.70	7.27
Q6PIJ4	Nuclear factor related to kappa-B-binding protein OS=Mus musculus GN=Nfrkb PE=2 SV=1 - [NFRKB_MOUSE]	2.24	1	1	1	4	1.05	0.79	0.75	1.06	1.05	1.29	1.23	1.40	1.29	1.28	1.22	1.08	1.03	138.68	9.22
Q8BHT6-	Isoform 2 of Beta-1,3-glucosyltransferase OS=Mus musculus GN=B3galtl - [B3GLT_MOUSE]	19.42	2	1	1	1	0.80	1.38	1.72	0.97	1.21	1.28	1.59	1.40	1.74	1.19	1.48	1.10	1.37	11.87	6.25

Q8BH43	Wiskott-Aldrich syndrome protein family member 2 OS=Mus musculus GN=Wasf2 PE=1 SV=1 - [WASF2_MOUSE]	13.08	1	5	6	16	0.93	1.09	1.07	0.90	0.90	1.33	1.37	1.40	1.35	1.31	1.42	1.19	1.31	54.04	5.53
F8WIA3	Modulator of apoptosis 1 OS=Mus musculus GN=Moap1 PE=2 SV=1 - [F8WIA3_MOUSE]	28.00	4	1	1	4	1.15	1.16	1.01	1.00	0.87	1.49	1.28	1.40	1.21	1.15	1.00	1.36	1.18	7.89	4.09
E9QM14	E3 ubiquitin-protein ligase Hakai OS=Mus musculus GN=Cbll1 PE=2 SV=1 - [E9QM14_MOUSE]	12.02	5	3	3	9	1.02	1.33	1.33	0.96	0.99	1.28	1.26	1.40	1.38	1.21	1.21	1.41	1.32	46.40	8.62
G3UWD4	Periphilin 1, isoform CRA_b OS=Mus musculus GN=Pphln1 PE=4 SV=1 - [G3UWD4_MOUSE]	7.85	5	2	2	3	1.11	0.94	0.85	1.06	0.96	1.31	1.18	1.40	1.26	1.29	1.17	1.12	1.01	33.82	6.32
Q9QYP6	5-azacytidine-induced protein 2 OS=Mus musculus GN=Azi2 PE=1 SV=1 - [AZI2_MOUSE]	28.89	5	10	10	24	1.07	1.01	0.98	0.98	0.92	1.29	1.20	1.40	1.17	1.29	1.12	1.17	1.08	46.06	6.70
P38647	Stress-70 protein, mitochondrial OS=Mus musculus GN=Hspa9 PE=1 SV=3 - [GRP75_MOUSE]	56.11	1	36	36	295	0.93	0.88	0.96	0.90	0.99	1.36	1.46	1.40	1.49	1.25	1.35	1.10	1.18	73.42	6.07
P43300	Early growth response protein 3 OS=Mus musculus GN=Egr3 PE=2 SV=2 - [EGR3_MOUSE]	9.04	1	2	2	7	0.98	0.96	0.98	0.99	1.03	1.53	1.49	1.40	1.40	1.37	1.38	1.19	1.19	42.61	8.35
Q80X32	UPF0461 protein C5orf24 homolog OS=Mus musculus PE=1 SV=1 - [CE024_MOUSE]	7.45	1	1	1	2	1.10	0.61	0.55	1.19	1.08	1.43	1.30	1.40	1.28	1.24	1.14	1.17	1.07	20.13	9.92
Q8CEG5	Coiled-coil domain-containing protein 28B OS=Mus musculus GN=Ccdc28b PE=2 SV=3 - [CC28B_MOUSE]	21.50	4	3	3	6	0.82	0.83	1.10	0.89	0.90	1.29	1.60	1.41	1.70	1.01	1.23	0.99	1.21	22.02	5.36
Q3TC46	Protein PAT1 homolog 1 OS=Mus musculus GN=Patl1 PE=1 SV=2 - [PATL1_MOUSE]	1.82	1	1	1	1	1.25	1.20	0.96	1.14	0.91	1.37	1.10	1.41	1.12	1.28	1.03	1.11	0.89	86.72	6.37
Q5U5M8	Biogenesis of lysosome-related organelles complex 1 subunit 3 OS=Mus musculus GN=Bloc1s3 PE=1 SV=1 - [BL1S3_MOUSE]	9.23	1	1	1	2	1.21	1.14	0.94	1.05	0.87	1.39	1.14	1.41	1.16	1.44	1.19	1.44	1.19	20.39	4.92
Q8BTG7	Protein NDRG4 OS=Mus musculus GN=Ndrg4 PE=1 SV=1 - [NDRG4_MOUSE]	29.83	2	2	9	21	1.07	1.09	1.01	1.00	0.93	1.15	1.06	1.41	1.31	1.41	1.31	1.30	1.21	38.48	6.32

Q9D2H6-2	Isoform 2 of Transcription factor Sp2 OS=Mus musculus GN=Sp2 - [SP2_MOUSE]	2.48	4	1	1	2	1.14	1.07	0.93	1.25	1.09	1.50	1.31	1.41	1.23	1.48	1.30	1.44	1.27	64.09	9.92
F6YLP9	Pleckstrin homology domain-containing family A member 1 (Fragment) OS=Mus musculus GN=Plekha1 PE=4 SV=1 - [F6YLP9_MOUSE]	6.45	2	1	1	1	1.13	1.16	1.03	0.98	0.87	1.25	1.09	1.41	1.24	1.19	1.05	1.16	1.03	17.17	9.73
Q8BJ64	Choline dehydrogenase, mitochondrial OS=Mus musculus GN=Chdh PE=1 SV=1 - [CHDH_MOUSE]	5.54	1	2	2	6	0.54	0.77	1.41	0.86	1.59	0.95	2.02	1.41	2.60	1.25	2.45	0.68	1.41	66.37	8.51
A2A9T0-2	Isoform 2 of TANK-binding kinase 1-binding protein 1 OS=Mus musculus GN=Tbkbp1 - [TBKB1_MOUSE]	3.36	4	1	2	3	1.13	0.98	0.87	1.02	0.90	1.16	1.02	1.41	1.24	1.12	0.99	1.27	1.12	65.41	5.62
P10637-2	Isoform Tau-A of Microtubule-associated protein tau OS=Mus musculus GN=Mapt - [TAU_MOUSE]	79.77	4	1	34	695	1.13	1.34	1.24	0.95	0.89	1.46	1.38	1.41	1.03	1.32	1.14	1.33	1.42	44.87	8.27
B8JJC1	Tyrosyl-DNA phosphodiesterase 1 OS=Mus musculus GN=Tdp1 PE=4 SV=1 - [B8JJC1_MOUSE]	1.64	2	1	1	2	0.70	1.92	2.76	0.92	1.32	1.05	1.50	1.41	2.02	0.71	1.02	1.01	1.46	68.55	7.64
Q8BYZ1	ABI gene family member 3 OS=Mus musculus GN=Abi3 PE=2 SV=3 - [ABI3_MOUSE]	8.17	3	2	2	7	0.96	1.00	1.02	0.96	0.96	1.48	1.51	1.41	1.40	1.16	1.20	1.06	1.10	39.08	5.54
Q99JI1	Musculoskeletal embryonic nuclear protein 1 OS=Mus musculus GN=Mustn1 PE=2 SV=1 - [MSTN1_MOUSE]	18.29	1	1	1	2	1.07	1.00	0.93	1.03	0.96	1.41	1.31	1.41	1.31	1.24	1.15	1.12	1.05	8.94	9.64
E9PV59	Protein Prr24 OS=Mus musculus GN=Prr24 PE=4 SV=1 - [E9PV59_MOUSE]	23.08	1	1	1	2	1.03	1.06	1.03	1.01	0.98	1.33	1.28	1.41	1.36	1.38	1.34	1.17	1.14	13.04	11.22
E9QLH9	Tyrosine-protein kinase SgK223 OS=Mus musculus GN=D8Ertd82e PE=4 SV=1 - [E9QLH9_MOUSE]	4.15	2	4	4	8	1.14	1.27	1.08	1.12	1.02	1.30	1.14	1.42	1.24	1.25	1.09	1.17	1.05	147.85	7.36
B9EI21	Zinc finger matrin type 3 OS=Mus musculus GN=Zmat3 PE=2 SV=1 - [B9EI21_MOUSE]	10.00	2	2	2	5	1.05	0.89	0.90	1.12	0.96	1.41	1.13	1.42	1.11	1.33	1.09	1.01	0.96	32.03	9.11
D3Z4I3	RNA-binding protein 24 OS=Mus musculus GN=Rbm24 PE=3 SV=1 - [RBM24_MOUSE]	4.24	2	1	1	2	1.05	0.99	0.94	1.24	1.18	1.06	1.00	1.42	1.34	1.27	1.20	1.22	1.16	24.81	8.51

D3Z6F3	INO80 complex subunit C OS=Mus musculus GN=Ino80c PE=2 SV=1 - [D3Z6F3_MOUSE]	14.89	3	1	1	2	0.76	1.11	1.45	0.83	1.08	1.47	1.91	1.42	1.84	1.23	1.60	1.20	1.57	9.94	10.45
Q8CCF0-	Isoform 2 of U4/U6 small nuclear ribonucleoprotein Prp31 OS=Mus musculus GN=Prpf31 - [PRP31_MOUSE]	4.26	3	2	2	3	0.90	1.55	1.73	1.11	1.24	1.34	1.59	1.42	1.68	1.22	1.45	1.20	1.43	54.71	5.67
P29788	Vitronectin OS=Mus musculus GN=Vtn PE=1 SV=2 - [VTNC_MOUSE]	3.77	1	1	1	1	1.16	0.98	0.84	1.07	0.92	1.18	1.02	1.42	1.22	1.43	1.23	1.01	0.87	54.81	5.88
P81122	Insulin receptor substrate 2 OS=Mus musculus GN=Irs2 PE=1 SV=2 - [IRS2_MOUSE]	7.42	1	5	5	11	1.13	1.24	1.05	1.10	0.99	1.30	1.09	1.42	1.25	1.42	1.24	1.23	1.12	136.68	8.69
A2AEK3	Cleavage stimulation factor subunit 2 (Fragment) OS=Mus musculus GN=Cstf2 PE=2 SV=1 - [A2AEK3_MOUSE]	36.13	1	1	4	9	1.17	1.49	1.27	1.38	1.18	1.50	1.27	1.42	1.21	1.42	1.21	1.35	1.16	19.32	7.75
Q64152	Transcription factor BTF3 OS=Mus musculus GN=Btf3 PE=2 SV=3 - [BTF3_MOUSE]	48.04	2	6	6	31	0.98	0.80	0.88	0.93	0.91	1.35	1.29	1.42	1.26	1.27	1.11	1.04	0.99	22.02	9.52
Q7TQH0-	Isoform 2 of Ataxin-2-like protein OS=Mus musculus GN=Atxn2l - [ATX2L_MOUSE]	29.98	3	2	23	77	1.14	1.22	1.05	1.00	0.90	1.13	1.05	1.42	1.25	1.16	1.07	1.29	1.24	112.97	8.59
Q64318	Zinc finger E-box-binding homeobox 1 OS=Mus musculus GN=Zeb1 PE=1 SV=1 - [ZEB1_MOUSE]	4.03	4	2	3	6	1.27	1.38	1.53	1.05	1.11	1.53	1.38	1.42	1.16	1.29	1.14	1.24	1.08	122.39	4.88
A2AL12	Heterogeneous nuclear ribonucleoprotein A3 OS=Mus musculus GN=Hnrnpa3 PE=2 SV=1 - [A2AL12_MOUSE]	45.28	6	2	18	631	0.83	0.73	0.90	0.95	1.14	1.39	1.68	1.43	1.79	1.23	1.50	0.90	1.10	34.45	9.10
F6TRJ8	Coiled-coil domain-containing protein 85A (Fragment) OS=Mus musculus GN=Ccdc85a PE=4 SV=1 - [F6TRJ8_MOUSE]	17.36	1	1	2	9	1.04	1.00	0.96	0.97	0.93	1.30	1.24	1.43	1.36	1.17	1.12	1.10	1.06	15.96	6.27
F6V035	Protein Ccdc149 OS=Mus musculus GN=Ccdc149 PE=4 SV=1 - [F6V035_MOUSE]	16.41	1	6	7	15	1.06	1.04	1.03	0.97	0.91	1.48	1.44	1.43	1.37	1.13	1.13	1.10	1.03	58.83	5.57
P33435	Collagenase 3 OS=Mus musculus GN=Mmp13 PE=1 SV=1 - [MMP13_MOUSE]	3.18	1	1	1	1	1.23	1.10	0.89	1.07	0.87	1.51	1.22	1.43	1.16	0.89	0.72	0.78	0.63	54.15	5.34

Q6P5D4	Centrosomal protein of 135 kDa OS=Mus musculus GN=Cep135 PE=1 SV=1 - [CP135_MOUSE]	1.14	1	1	2	3	1.06	1.14	1.08	0.95	0.89	1.19	1.12	1.43	1.34	1.19	1.12	1.14	1.08	133.26	6.48
F8WIX8	Histone H2A OS=Mus musculus GN=Hist1h2al PE=2 SV=1 - [F8WIX8_MOUSE]	59.20	4	1	7	113	1.03	0.83	0.74	0.94	0.95	1.39	1.39	1.43	1.36	1.29	1.31	1.22	1.26	13.60	10.74
Q9CQ25	Mitotic-spindle organizing protein 2 OS=Mus musculus GN=Mzt2 PE=1 SV=1 - [MZT2_MOUSE]	22.01	1	2	2	4	1.02	1.00	0.98	1.04	1.03	1.37	1.34	1.43	1.40	1.34	1.32	1.12	1.10	16.51	10.18
Q6PGF5	BMS1 homolog, ribosome assembly protein (Yeast) OS=Mus musculus GN=Bms1 PE=2 SV=1 - [Q6PGF5_MOUSE]	1.32	1	1	1	5	1.02	1.20	1.18	1.14	1.13	1.21	1.18	1.43	1.41	1.41	1.39	1.14	1.13	145.32	6.27
D3YUN2	Suppressor of tumorigenicity 7 protein OS=Mus musculus GN=St7 PE=2 SV=1 - [D3YUN2_MOUSE]	6.56	11	1	1	2	2.13	2.96	1.38	1.48	0.69	1.05	0.49	1.44	0.67	1.49	0.70	0.96	0.45	57.41	7.56
Q8CDM3	X-linked retinitis pigmentosa GTPase regulator OS=Mus musculus GN=Rpgr PE=2 SV=1 - [Q8CDM3_MOUSE]	1.54	10	1	2	2	1.08	0.94	0.87	1.20	1.11	1.42	1.32	1.44	1.33	1.30	1.21	1.40	1.30	108.39	4.64
Q8K207	Uncharacterized protein C1orf21 homolog OS=Mus musculus PE=1 SV=1 - [CA021_MOUSE]	20.66	1	2	2	4	0.93	0.93	0.92	0.95	0.86	1.20	1.06	1.44	1.28	1.18	0.93	0.99	0.92	13.87	5.22
B2RUJ8	Rho GTPase activating protein 12 OS=Mus musculus GN=Arhgap12 PE=2 SV=1 - [B2RUJ8_MOUSE]	1.11	2	1	1	1	0.96	1.13	1.17	1.04	1.08	1.04	1.08	1.44	1.49	1.28	1.34	1.22	1.27	92.60	7.96
Q80XJ2	E3 ubiquitin-protein ligase RNF168 OS=Mus musculus GN=Rnf168 PE=2 SV=3 - [RN168_MOUSE]	3.89	3	2	2	10	1.00	0.98	0.90	1.08	1.07	1.28	1.22	1.44	1.41	1.26	1.18	1.09	1.07	64.74	5.59
Q9R0U9	Protein Tex21 OS=Mus musculus GN=Tex21 PE=2 SV=1 - [Q9R0U9_MOUSE]	1.91	1	1	1	1	2.11	1.24	0.58	1.34	0.63	1.16	0.55	1.44	0.68	1.11	0.52	2.87	1.36	65.37	7.72
Q7TPQ3-3	Isoform 3 of E3 ubiquitin-protein ligase SHPRH OS=Mus musculus GN=Shprh - [SHPRH_MOUSE]	1.16	4	1	1	1	1.17	1.16	0.99	1.35	1.15	1.45	1.23	1.44	1.22	1.13	0.97	0.72	0.61	117.98	7.75
Q8BR63	Protein FAM177A1 OS=Mus musculus GN=Fam177a1 PE=2 SV=1 - [F177A_MOUSE]	30.43	1	5	5	12	1.01	1.07	1.01	1.00	1.03	1.23	1.33	1.44	1.44	1.15	1.22	1.27	1.36	23.56	4.59

Q9DAW9	Calponin-3 OS=Mus musculus GN=Cnn3 PE=2 SV=1 - [CNN3_MOUSE]	39.39	3	9	9	30	0.92	0.95	0.95	1.02	1.04	1.45	1.43	1.44	1.52	1.34	1.33	1.18	1.21	36.41	5.72
Q9CPX8	Cytochrome b-c1 complex subunit 10 OS=Mus musculus GN=Uqcr11 PE=2 SV=1 - [QCR10_MOUSE]	21.43	1	1	1	3	1.18	0.76	0.64	1.20	1.01	1.48	1.25	1.44	1.21	1.31	1.27	1.18	1.02	6.53	9.82
Q9CTN8	Lipoma HMGIC fusion partner-like 3 protein OS=Mus musculus GN=Lhfpl3 PE=2 SV=2 - [LHPL3_MOUSE]	4.50	1	1	1	1	0.55	0.79	1.42	0.80	1.44	0.87	1.56	1.44	2.60	1.20	2.17	0.81	1.47	24.67	5.95
Q8CJF7	Protein ELYS OS=Mus musculus GN=Ahctf1 PE=1 SV=1 - [ELYS_MOUSE]	2.45	1	4	4	7	1.01	1.21	1.22	1.13	1.15	1.15	1.13	1.44	1.30	1.31	1.36	1.40	1.30	247.49	6.55
P42866-13	Isoform 13 of Mu-type opioid receptor OS=Mus musculus GN=Oprm1 - [OPRM_MOUSE]	2.21	1	1	1	1	0.81	1.03	1.27	1.03	1.27	1.37	1.68	1.44	1.78	1.12	1.39	1.20	1.48	50.68	9.07
P0C0S6	Histone H2A.Z OS=Mus musculus GN=H2afz PE=1 SV=2 - [H2AZ_MOUSE]	53.91	5	3	5	55	0.97	0.86	0.86	0.97	0.85	1.35	1.34	1.44	1.43	1.20	1.22	0.97	0.96	13.54	10.58
Q80TJ7	Histone lysine demethylase PHF8 OS=Mus musculus GN=Phf8 PE=1 SV=2 - [PHF8_MOUSE]	3.71	2	2	2	3	1.16	1.32	1.13	1.19	1.02	1.15	0.99	1.44	1.24	1.53	1.32	1.51	1.30	113.48	8.44
Q9ET54-3	Isoform 3 of Palladin OS=Mus musculus GN=Palld - [PALLD_MOUSE]	1.36	6	1	1	2	1.05	1.85	1.76	0.90	0.85	1.06	1.00	1.45	1.37	1.34	1.28	1.13	1.07	72.32	7.68
Q9D952	Envoplakin OS=Mus musculus GN=Evpl PE=2 SV=3 - [EVPL_MOUSE]	0.69	1	1	2	3	1.17	1.05	0.90	0.97	0.82	1.41	1.20	1.45	1.23	1.49	1.27	1.15	0.98	231.87	6.60
P29268	Connective tissue growth factor OS=Mus musculus GN=Ctgf PE=2 SV=3 - [CTGF_MOUSE]	9.20	2	3	3	7	0.97	1.11	1.20	0.98	1.00	1.13	1.21	1.45	1.62	1.15	1.28	1.08	1.23	37.80	7.81
Q9QZC2	Plexin-C1 OS=Mus musculus GN=Plxnc1 PE=1 SV=1 - [PLXC1_MOUSE]	1.97	1	2	2	3	1.08	1.73	1.59	1.20	1.10	1.17	1.07	1.45	1.33	1.53	1.41	1.51	1.39	176.36	7.75
P21126	Ubiquitin-like protein 4A OS=Mus musculus GN=Ubl4a PE=2 SV=1 - [UBL4A_MOUSE]	35.67	1	5	5	15	0.96	0.91	0.89	0.96	0.98	1.54	1.52	1.45	1.47	1.34	1.38	1.28	1.35	17.79	8.44
Q3U962	Collagen alpha-2(V) chain OS=Mus musculus GN=Col5a2 PE=1 SV=1 - [CO5A2_MOUSE]	1.67	1	1	1	2	1.02	1.34	1.31	1.09	1.06	1.44	1.40	1.45	1.42	1.40	1.38	1.51	1.48	144.93	6.70
Q5EG47	5'-AMP-activated protein kinase catalytic subunit alpha-1 OS=Mus musculus GN=Prkaa1 PE=1 SV=2 - [AAPK1_MOUSE]	7.69	1	1	3	4	1.26	1.22	0.97	1.10	0.87	1.61	1.27	1.45	1.15	1.38	1.09	1.01	0.80	63.89	8.12

Q3UQU0	Bromodomain-containing protein 9 OS=Mus musculus GN=Brd9 PE=2 SV=1 - [BRD9_MOUSE]	2.01	1	1	1	2	0.95	1.37	1.44	0.97	1.02	1.23	1.30	1.45	1.53	0.97	1.03	1.04	1.11	66.80	6.27
Q6ZWQ9	MCG5400 OS=Mus musculus GN=Myl12a PE=2 SV=1 - [Q6ZWQ9_MOUSE]	51.74	3	1	7	163	1.21	1.50	1.31	1.16	0.99	1.32	1.13	1.45	1.24	1.55	1.30	1.64	1.35	19.88	4.81
Q9EQU5-	Isoform 2 of Protein SET OS=Mus musculus GN=Set - [SET_MOUSE]	55.23	1	2	12	193	0.79	1.19	1.50	0.89	1.09	1.40	1.77	1.45	1.84	1.04	1.06	1.10	1.02	32.09	4.22
Q9R257	Heme-binding protein 1 OS=Mus musculus GN=Hebp1 PE=1 SV=2 - [HEBP1_MOUSE]	62.11	1	7	7	16	0.99	0.85	0.82	0.98	1.02	1.36	1.42	1.45	1.50	1.40	1.40	1.32	1.35	21.05	5.26
P48432	Transcription factor SOX-2 OS=Mus musculus GN=Sox2 PE=1 SV=2 - [SOX2_MOUSE]	6.27	2	1	1	4	1.16	1.27	1.09	1.27	1.10	1.27	1.09	1.45	1.25	1.32	1.14	1.30	1.13	34.43	9.82
P49919-2	Isoform KIP2b of Cyclin-dependent kinase inhibitor 1C OS=Mus musculus GN=Cdkn1c - [CDN1C_MOUSE]	5.07	2	1	1	2	1.04	1.15	1.10	1.03	0.99	1.39	1.33	1.46	1.39	1.33	1.28	1.26	1.21	35.88	4.26
Q8R0S1	Cyclic AMP-dependent transcription factor ATF-7 OS=Mus musculus GN=Atf7 PE=1 SV=1 - [ATF7_MOUSE]	7.99	2	1	3	6	1.18	1.49	1.26	1.10	0.94	1.46	1.24	1.46	1.23	1.43	1.21	1.55	1.32	44.58	8.35
Q61595-1	Isoform 14 of Kinectin OS=Mus musculus GN=Ktn1 - [KTN1_MOUSE]	44.75	11	2	45	110	1.22	1.17	1.13	1.27	1.14	1.82	1.57	1.46	1.20	1.45	1.47	1.42	1.17	141.00	6.27
E9Q8M1	Protein Zfp358 OS=Mus musculus GN=Zfp358 PE=4 SV=1 - [E9Q8M1_MOUSE]	1.75	1	1	1	3	0.76	1.10	1.45	1.03	0.95	1.28	1.51	1.46	1.01	1.41	1.87	1.31	1.74	59.75	7.01
P27661	Histone H2A.x OS=Mus musculus GN=H2afx PE=1 SV=2 - [H2AX_MOUSE]	60.14	1	2	8	88	1.21	1.09	0.84	1.23	1.14	1.26	1.28	1.46	1.82	1.16	0.95	1.30	1.36	15.13	10.74
O88685	26S protease regulatory subunit 6A OS=Mus musculus GN=Psmc3 PE=1 SV=2 - [PRS6A_MOUSE]	64.71	4	21	21	65	0.90	0.97	1.01	0.94	1.02	1.41	1.48	1.46	1.57	1.32	1.41	1.17	1.23	49.52	5.19
O54791	Transcription factor MafF OS=Mus musculus GN=Maff PE=2 SV=1 - [MAFF_MOUSE]	4.49	3	1	1	1	0.97	0.94	0.97	0.85	0.88	1.27	1.30	1.46	1.50	1.22	1.26	1.11	1.14	16.94	9.80
Q80UP8	Sodium-dependent phosphate transporter 2 OS=Mus musculus GN=Slc20a2 PE=1 SV=2 - [S20A2_MOUSE]	1.98	1	1	1	4	1.42	1.15	0.81	1.19	0.82	1.20	0.84	1.46	1.02	1.17	0.82	0.84	0.86	70.82	6.48

Q64314-2	Isoform Short of Hematopoietic progenitor cell antigen CD34 OS=Mus musculus GN=Cd34 - [CD34_MOUSE]	18.77	1	1	7	24	0.77	1.19	1.54	0.82	1.06	1.10	1.42	1.46	1.89	1.11	1.44	0.96	1.25	35.24	5.16
Q61189	Methylosome subunit pICln OS=Mus musculus GN=Clns1a PE=1 SV=1 - [ICLN_MOUSE]	53.39	1	1	7	42	1.09	0.84	0.76	1.06	0.97	1.31	1.19	1.46	1.33	1.26	1.15	1.04	0.95	26.00	4.12
Q8C1D8	Protein IWS1 homolog OS=Mus musculus GN=Iws1 PE=1 SV=1 - [IWS1_MOUSE]	9.79	2	5	6	11	1.11	0.85	0.72	1.05	0.93	1.41	1.26	1.47	1.29	1.35	1.21	1.21	1.08	85.20	4.67
Q5XJE5	RNA polymerase-associated protein LEO1 OS=Mus musculus GN=Leo1 PE=1 SV=2 - [LEO1_MOUSE]	8.55	2	5	5	7	1.24	0.97	0.78	1.03	0.87	1.35	1.08	1.47	1.18	1.34	1.08	1.26	1.02	75.55	4.46
Q8C1S0	Mediator of RNA polymerase II transcription subunit 19 OS=Mus musculus GN=Med19 PE=1 SV=1 - [MED19_MOUSE]	10.25	1	1	1	3	1.07	0.79	0.73	1.22	1.14	1.28	1.18	1.47	1.36	1.39	1.30	1.42	1.32	26.25	9.88
E9Q0F0	Protein Krt78 OS=Mus musculus GN=Krt78 PE=2 SV=1 - [E9Q0F0_MOUSE]	1.78	1	1	2	2	1.52	1.41	0.92	1.31	0.86	0.84	0.55	1.47	0.96	1.03	0.68	2.49	1.63	112.19	7.97
Q7TSH6	Protein Scaf4 OS=Mus musculus GN=Scaf4 PE=2 SV=1 - [Q7TSH6_MOUSE]	2.87	2	2	2	2	1.23	1.55	1.26	1.30	1.06	1.20	0.97	1.47	1.19	1.12	0.91	1.11	0.91	128.97	9.64
Q8BKZ9	Pyruvate dehydrogenase protein X component, mitochondrial OS=Mus musculus GN=Pdhx PE=2 SV=1 - [ODPX_MOUSE]	31.74	3	11	11	87	0.95	0.98	1.11	0.99	1.02	1.38	1.42	1.47	1.53	1.34	1.38	1.19	1.22	53.97	7.75
Q61702	Inter-alpha-trypsin inhibitor heavy chain H1 OS=Mus musculus GN=Itih1 PE=1 SV=2 - [ITIH1_MOUSE]	3.09	2	1	1	4	1.60	1.23	0.77	0.86	0.54	1.35	0.84	1.47	0.92	1.52	0.95	1.39	0.87	101.00	6.96
Q3USH1	Protein FAM196A OS=Mus musculus GN=Fam196a PE=2 SV=1 - [F196A_MOUSE]	8.77	2	3	3	5	1.00	0.88	0.88	0.94	0.91	1.49	1.25	1.47	1.44	1.43	1.37	1.52	1.42	46.19	7.14
A2A7S8	Uncharacterized protein KIAA1522 OS=Mus musculus GN=Kiaa1522 PE=1 SV=1 - [K1522_MOUSE]	17.47	4	9	9	15	1.08	1.35	1.26	1.05	0.92	1.48	1.25	1.47	1.28	1.37	1.22	1.09	1.08	104.78	9.69
F7BGR7	RNA-binding protein 4 OS=Mus musculus GN=Rbm4 PE=4 SV=1 - [F7BGR7_MOUSE]	25.37	9	1	11	19	1.01	0.95	0.93	1.52	1.51	1.67	1.64	1.47	1.45	1.49	1.47	1.22	1.21	52.76	7.30

Q5ND52	RNA methyltransferase-like protein 1 OS=Mus musculus GN=Rnmtl1 PE=2 SV=1 - [RMTL1_MOUSE]	2.63	1	1	1	1	1.04	1.32	1.26	0.97	0.93	1.14	1.08	1.48	1.41	1.31	1.26	1.18	1.13	46.77	9.39
Q7TPG8	Protein FAM19A1 OS=Mus musculus GN=Fam19a1 PE=2 SV=1 - [F19A1_MOUSE]	11.28	1	1	1	2	1.12	0.87	0.77	0.94	0.84	1.31	1.16	1.48	1.31	1.46	1.31	1.31	1.16	14.86	8.12
Q5SQM0	Echinoderm microtubule-associated protein-like 6 OS=Mus musculus GN=Eml6 PE=2 SV=1 - [EMAL6_MOUSE]	0.66	1	1	1	1	1.04	1.40	1.34	1.20	1.15	1.19	1.14	1.48	1.41	1.51	1.46	1.26	1.22	217.80	7.42
Q921N8	G protein pathway suppressor 2 OS=Mus musculus GN=Gps2 PE=2 SV=1 - [Q921N8_MOUSE]	3.36	1	1	1	2	1.00	1.29	1.29	0.96	0.97	1.09	1.09	1.48	1.48	1.19	1.19	1.32	1.33	36.71	9.60
D3YY34	Polyhomeotic-like protein 3 OS=Mus musculus GN=Phc3 PE=4 SV=1 - [D3YY34_MOUSE]	4.43	7	2	2	8	0.88	3.30	3.75	0.94	1.07	1.25	1.42	1.48	1.69	1.16	1.33	1.05	1.20	102.08	6.65
P97783	Protein AF1q OS=Mus musculus GN=Mllt11 PE=2 SV=1 - [AF1Q_MOUSE]	80.00	1	5	5	8	1.00	0.95	1.08	0.94	0.94	1.57	1.56	1.49	1.74	1.42	1.42	1.16	1.20	10.02	4.41
P70695	Fructose-1,6-bisphosphatase isozyme 2 OS=Mus musculus GN=Fbp2 PE=2 SV=2 - [F16P2_MOUSE]	2.95	1	1	1	1	1.58	1.22	0.77	1.44	0.91	1.23	0.77	1.49	0.94	0.96	0.61	1.06	0.67	36.92	6.16
O88990	Alpha-actinin-3 OS=Mus musculus GN=Actn3 PE=2 SV=1 - [ACTN3_MOUSE]	15.89	1	1	16	83	1.02	1.39	1.37	1.02	1.00	1.05	1.03	1.49	1.46	1.54	1.51	1.56	1.54	102.98	5.45
Q8C790-2	Isoform 2 of Protein FAM221A OS=Mus musculus GN=Fam221a - [F221A_MOUSE]	6.98	2	1	1	2	1.08	1.07	0.98	1.10	1.01	1.63	1.50	1.49	1.37	1.44	1.32	1.37	1.26	28.32	7.53
Q8BMK5-	Isoform 2 of Uncharacterized protein C12orf68 homolog OS=Mus musculus - [CL068_MOUSE]	19.08	3	2	2	4	1.16	1.37	1.18	0.93	0.80	1.20	1.03	1.49	1.28	1.04	0.90	1.19	1.03	16.20	4.27
Q9D173	Mitochondrial import receptor subunit TOM7 homolog OS=Mus musculus GN=Tomm7 PE=3 SV=1 - [TOM7_MOUSE]	25.45	1	1	1	2	1.23	0.98	0.80	0.92	0.75	1.53	1.24	1.49	1.21	1.14	0.93	1.47	1.20	6.17	10.17
Q8CES0	N-alpha-acetyltransferase 30 OS=Mus musculus GN=Naa30 PE=2 SV=2 - [NAA30_MOUSE]	15.11	3	3	3	7	1.24	1.14	0.92	1.12	0.99	1.49	1.20	1.49	1.27	1.34	1.16	1.26	1.01	39.41	5.64
Q9CY62	E3 ubiquitin-protein ligase RNF181 OS=Mus musculus GN=Rnf181 PE=2 SV=1 - [RN181_MOUSE]	32.73	5	4	4	11	1.19	0.94	0.84	1.11	0.99	1.41	1.10	1.49	1.09	1.25	1.08	1.09	0.89	19.09	6.04

P98063	Bone morphogenetic protein 1 OS=Mus musculus GN=Bmp1 PE=1 SV=2 - [BMP1_MOUSE]	1.61	1	1	1	2	1.52	1.22	0.80	1.33	0.87	1.49	0.98	1.50	0.98	1.17	0.78	0.98	0.65	111.60	6.95
Q9D0D5	General transcription factor IIE subunit 1 OS=Mus musculus GN=Gtf2e1 PE=2 SV=1 - [T2EA_MOUSE]	2.73	1	1	1	2	0.96	1.14	1.19	0.98	1.02	1.08	1.12	1.50	1.56	1.43	1.49	1.47	1.54	49.56	4.86
Q9CXL6	Doublecortin, isoform CRA_a OS=Mus musculus GN=Dcx PE=2 SV=1 - [Q9CXL6_MOUSE]	5.83	3	1	2	3	1.29	1.51	1.17	1.28	0.99	1.63	1.25	1.50	1.15	1.42	1.10	1.28	0.99	39.96	9.39
P28481-2	Isoform 3 of Collagen alpha-1(II) chain OS=Mus musculus GN=Col2a1 - [CO2A1_MOUSE]	2.37	7	2	2	3	1.50	0.97	0.65	1.21	0.81	3.99	2.65	1.50	1.00	1.54	1.03	1.28	0.86	131.78	8.25
J3QQ44	Endophilin-A3 OS=Mus musculus GN=Sh3gl3 PE=4 SV=1 - [J3QQ44_MOUSE]	45.19	3	1	10	28	1.02	1.27	1.24	1.22	1.19	1.47	1.43	1.50	1.46	1.60	1.57	1.41	1.38	35.08	5.03
Q64522	Histone H2A type 2-B OS=Mus musculus GN=Hist2h2ab PE=1 SV=3 - [H2A2B_MOUSE]	60.00	1	1	6	68	1.09	1.13	1.03	1.06	0.97	1.39	1.26	1.50	1.37	1.35	1.24	1.46	1.34	14.00	10.89
Q3U1D0-2	Isoform 2 of Protein Lines homolog OS=Mus musculus GN=Lins - [LINES_MOUSE]	3.61	3	1	1	1	0.81	0.97	1.19	1.15	1.42	1.27	1.56	1.50	1.85	1.42	1.76	1.25	1.54	55.42	6.48
Q7TN02	Mediator of RNA polymerase II transcription subunit 26 OS=Mus musculus GN=Med26 PE=2 SV=1 - [MED26_MOUSE]	8.50	1	2	2	4	1.22	0.95	0.78	1.04	0.83	1.33	1.08	1.50	1.23	0.85	0.70	0.95	0.78	64.64	9.19
Q69ZP3-2	Isoform 2 of Probable hydrolase PNKD OS=Mus musculus GN=Pnkd - [PNKD_MOUSE]	22.54	1	1	2	2	0.90	2.68	2.96	0.84	0.93	0.91	1.01	1.50	1.66	1.47	1.63	1.87	2.08	15.58	10.18
Q3USH5	Splicing factor, suppressor of white-apricot homolog OS=Mus musculus GN=Sfswap PE=1 SV=2 - [SFSWA_MOUSE]	8.36	1	4	4	12	0.91	1.06	1.06	1.01	1.03	1.20	1.29	1.50	1.39	1.03	1.12	1.16	1.13	104.13	8.05
Q9ERR1-2	Isoform 2 of Nuclear distribution protein nudE-like 1 OS=Mus musculus GN=Ndel1 - [NDEL1_MOUSE]	65.71	2	14	19	66	1.02	1.02	0.98	0.96	0.93	1.38	1.29	1.50	1.43	1.29	1.20	1.27	1.24	35.49	5.22
Q7TNS2	Mitochondrial inner membrane organizing system protein 1 OS=Mus musculus GN=Minos1 PE=2 SV=1 - [MOS1_MOUSE]	10.53	1	1	1	2	1.16	1.94	1.66	1.33	1.14	1.80	1.54	1.51	1.29	1.64	1.41	1.77	1.52	8.56	8.94

Q9CQ39	Mediator of RNA polymerase II transcription subunit 21 OS=Mus musculus GN=Med21 PE=1 SV=1 - [MED21_MOUSE]	22.22	1	2	2	5	1.20	1.22	0.90	1.04	1.00	1.24	1.09	1.51	1.25	1.30	1.09	1.33	1.01	15.58	4.53
Q6NV92	Protein Zfp941 OS=Mus musculus GN=Zfp941 PE=2 SV=1 - [Q6NV92_MOUSE]	2.38	2	1	1	3	1.17	1.06	0.91	0.82	0.70	1.32	1.12	1.51	1.29	1.31	1.12	0.97	0.83	75.90	9.32
A2AEW0	Transcription factor E3 OS=Mus musculus GN=Tfe3 PE=2 SV=1 - [A2AEW0_MOUSE]	13.59	19	7	7	10	1.07	0.96	1.00	0.98	0.93	1.22	1.12	1.51	1.40	1.13	1.24	1.17	1.06	57.70	6.37
L7N209	HBS1-like protein OS=Mus musculus GN=Hbs1l PE=4 SV=1 - [L7N209_MOUSE]	12.42	3	1	6	16	0.91	1.32	1.44	0.96	1.05	1.33	1.45	1.51	1.65	1.37	1.50	1.25	1.37	67.49	5.64
D6RET6	G patch domain-containing protein 1 OS=Mus musculus GN=Gpatch1 PE=2 SV=1 - [D6RET6_MOUSE]	1.70	2	1	1	2	1.14	1.26	1.11	0.99	0.87	1.38	1.20	1.51	1.32	1.49	1.31	1.11	0.98	97.83	6.80
Q9CW46	Ribonucleoprotein PTB-binding 1 OS=Mus musculus GN=Raver1 PE=1 SV=2 - [RAVR1_MOUSE]	4.41	1	2	2	11	1.02	1.05	1.08	0.94	0.97	1.33	1.37	1.52	1.35	1.36	1.41	1.50	1.47	79.33	8.72
O70279	Protein DGCR14 OS=Mus musculus GN=Dgcr14 PE=2 SV=2 - [DGC14_MOUSE]	11.90	2	3	3	7	1.13	1.18	1.15	1.14	1.01	1.46	1.51	1.52	1.37	1.26	1.12	1.14	1.01	52.57	6.70
E9PZQ0	Ryanodine receptor 1 OS=Mus musculus GN=Ryr1 PE=1 SV=1 - [RYR1_MOUSE]	0.48	2	1	2	2	1.23	1.19	0.97	0.95	0.77	1.46	1.18	1.52	1.23	1.29	1.05	1.27	1.03	564.68	5.27
A2A7T3	Arginine-glutamic acid dipeptide repeats protein OS=Mus musculus GN=Rere PE=2 SV=1 - [A2A7T3_MOUSE]	1.63	2	1	1	1	1.24	1.14	0.91	0.94	0.75	0.45	0.36	1.52	1.22	0.88	0.71	0.68	0.55	140.83	7.81
Q61207	Sulfated glycoprotein 1 OS=Mus musculus GN=Psap PE=1 SV=2 - [SAP_MOUSE]	47.76	3	1	22	209	1.19	1.05	0.88	1.03	0.87	1.18	0.99	1.52	1.28	1.33	1.12	1.43	1.20	61.38	5.19
Q9CY57-5	Isoform 5 of Chromatin target of PRMT1 protein OS=Mus musculus GN=Chtop - [CHTOP_MOUSE]	25.99	8	3	3	8	1.08	1.11	0.89	1.06	1.03	1.53	1.37	1.52	1.46	1.49	1.43	1.29	1.33	19.23	12.00
Q3ULB5	Serine/threonine-protein kinase PAK 6 OS=Mus musculus GN=Pak6 PE=2 SV=1 - [PAK6_MOUSE]	6.16	2	1	3	5	1.06	0.99	0.94	1.00	0.94	1.45	1.36	1.52	1.44	1.26	1.20	1.12	1.06	74.82	9.44

Q8K2V6	Importin-11 OS=Mus musculus GN=Ipo11 PE=1 SV=1 - [IPO11_MOUSE]	2.26	2	2	2	2	1.19	1.47	1.23	1.22	1.02	1.02	0.85	1.53	1.28	1.16	0.98	1.03	0.87	112.34	5.26
Q9ERQ3	Zinc finger protein 704 OS=Mus musculus GN=Znf704 PE=2 SV=1 - [ZN704_MOUSE]	17.14	1	5	5	9	1.35	1.14	0.80	1.06	0.81	1.36	1.07	1.53	1.10	1.22	0.85	1.33	0.99	61.11	7.24
Q61152	Tyrosine-protein phosphatase non-receptor type 18 OS=Mus musculus GN=Ptpn18 PE=1 SV=1 - [PTN18_MOUSE]	3.53	1	1	1	2	1.00	1.58	1.58	1.11	1.11	1.34	1.33	1.53	1.53	1.46	1.47	5.21	5.22	50.17	8.27
Q91X84-2	Isoform 2 of CREB-regulated transcription coactivator 3 OS=Mus musculus GN=Crtc3 - [CRTC3_MOUSE]	5.94	2	1	1	2	1.06	1.07	1.00	1.05	0.98	1.34	1.25	1.53	1.44	1.39	1.31	1.67	1.57	34.28	5.36
Q9JJ48	Zinc finger CCCH domain-containing protein 8 OS=Mus musculus GN=Zc3h8 PE=1 SV=2 - [ZC3H8_MOUSE]	5.25	1	1	1	1	0.98	1.20	1.22	0.94	0.96	1.21	1.22	1.54	1.56	1.07	1.09	1.31	1.34	34.89	9.01
Q924C6	Lysyl oxidase homolog 4 OS=Mus musculus GN=Loxl4 PE=2 SV=2 - [LOXL4_MOUSE]	2.38	2	1	1	3	1.56	4.87	3.13	1.77	1.14	1.85	1.18	1.54	0.98	1.39	0.89	1.32	0.85	84.72	7.99
O35738	Krueppel-like factor 12 OS=Mus musculus GN=Klf12 PE=2 SV=2 - [KLF12_MOUSE]	3.98	1	1	1	3	0.98	1.08	1.09	0.89	0.90	1.49	1.50	1.54	1.56	1.35	1.37	1.18	1.20	44.08	9.70
Q543V3	Insulin receptor substrate 1 OS=Mus musculus GN=Irs1 PE=2 SV=1 - [Q543V3_MOUSE]	3.74	2	2	2	3	0.91	1.14	1.25	1.16	1.27	1.98	2.16	1.54	1.68	1.21	1.33	0.97	1.07	130.50	8.78
M0QWS6	Protein FAM193A (Fragment) OS=Mus musculus GN=Fam193a PE=4 SV=1 - [M0QWS6_MOUSE]	10.45	3	1	1	15	1.27	1.36	1.07	1.07	0.92	1.30	1.11	1.54	1.11	1.44	1.11	1.33	1.01	15.33	9.57
Q99LI5	Zinc finger protein 281 OS=Mus musculus GN=Znf281 PE=1 SV=1 - [ZN281_MOUSE]	2.02	1	1	1	2	0.96	0.93	0.96	1.00	1.04	1.12	1.15	1.54	1.59	1.30	1.35	1.25	1.30	96.62	8.56
Q68FG3	Protein SPT2 homolog OS=Mus musculus GN=Spty2d1 PE=2 SV=1 - [SPT2_MOUSE]	4.25	1	2	2	3	1.04	1.27	1.22	1.15	1.11	1.31	1.25	1.54	1.47	1.42	1.36	1.33	1.28	74.77	9.76
O54786	DNA fragmentation factor subunit alpha OS=Mus musculus GN=Dffa PE=1 SV=2 - [DFFA_MOUSE]	24.77	1	1	6	18	0.88	1.45	1.65	0.87	0.99	1.28	1.45	1.54	1.75	1.14	1.30	1.04	1.19	36.55	4.91

E9PUY1	28S ribosomal protein S12, mitochondrial (Fragment) OS=Mus musculus GN=Mrps12 PE=2 SV=1 - [E9PUY1_MOUSE]	6.40	2	1	1	1	0.89	1.35	1.52	1.75	1.97	2.53	2.84	1.54	1.73	1.28	1.44	2.14	2.41	13.87	10.43
Q9CVI2-2	Isoform 2 of Protein FAM133B OS=Mus musculus GN=Fam133b - [F133B_MOUSE]	9.57	2	1	1	3	0.97	1.11	1.14	1.01	1.04	1.00	1.02	1.54	1.59	1.29	1.33	1.19	1.23	13.48	10.13
D6REH7	Protein 1110004E09Rik OS=Mus musculus GN=1110004E09Rik PE=2 SV=1 - [D6REH7_MOUSE]	17.93	3	1	1	1	1.25	0.59	0.47	0.99	0.79	1.71	1.36	1.55	1.23	0.73	0.58	1.04	0.83	16.11	5.77
E9Q3B5	Uncharacterized protein OS=Mus musculus GN=Gm10774 PE=3 SV=1 - [E9Q3B5_MOUSE]	29.85	2	2	2	3	0.89	0.72	0.80	0.78	0.87	1.71	1.90	1.55	1.73	1.15	1.29	1.14	1.28	7.64	8.48
Q3UXS0	Secretory carrier-associated membrane protein 3 OS=Mus musculus GN=Scamp3 PE=2 SV=1 - [Q3UXS0_MOUSE]	18.57	1	1	3	14	1.35	1.31	0.63	1.23	0.91	0.96	0.72	1.55	1.15	1.14	0.85	1.63	1.21	38.56	7.64
Q9EST5	Acidic leucine-rich nuclear phosphoprotein 32 family member B OS=Mus musculus GN=Anp32b PE=1 SV=1 - [AN32B_MOUSE]	18.38	3	2	5	30	1.15	1.01	1.11	1.19	1.03	1.61	1.39	1.56	1.34	1.26	1.09	1.80	1.57	31.06	4.01
Q9D6V8	Polyadenylate-binding protein-interacting protein 2 OS=Mus musculus GN=Paip2 PE=2 SV=1 - [PAIP2_MOUSE]	26.61	1	2	2	9	1.37	1.52	1.13	0.95	0.84	1.44	1.01	1.56	1.20	1.28	0.99	1.58	1.14	14.69	4.12
G3UW60	MCG14773 OS=Mus musculus GN=Susd5 PE=4 SV=1 - [G3UW60_MOUSE]	4.85	1	3	3	6	0.79	0.79	1.00	1.10	1.25	1.17	1.07	1.56	1.14	1.09	1.12	0.85	0.85	66.76	4.93
Q7M722	Taste receptor type 2 member 114 OS=Mus musculus GN=Tas2r114 PE=2 SV=1 - [TR114_MOUSE]	3.56	1	1	1	1	0.83	1.39	1.66	0.92	1.10	1.22	1.46	1.56	1.87	1.19	1.43	1.42	1.71	35.71	9.04
Q9DD24	WW domain-binding protein 5 OS=Mus musculus GN=Wbp5 PE=2 SV=1 - [WBP5_MOUSE]	8.65	1	1	1	7	1.19	1.33	1.04	1.16	0.97	1.56	1.21	1.56	1.28	1.40	1.15	1.30	1.11	12.66	4.84
O88492	Perilipin-4 OS=Mus musculus GN=Plin4 PE=1 SV=2 - [PLIN4_MOUSE]	6.27	2	2	2	3	1.05	0.91	0.86	0.96	0.91	1.82	1.73	1.56	1.48	1.43	1.37	1.38	1.32	139.33	8.59

Q99K90	TGF-beta-activated kinase 1 and MAP3K7-binding protein 2 OS=Mus musculus GN=Tab2 PE=1 SV=1 - [TAB2_MOUSE]	5.63	3	3	3	5	1.07	1.07	1.14	1.01	0.96	1.49	1.34	1.57	1.46	1.44	1.34	1.67	1.57	76.39	8.59
Q01149	Collagen alpha-2(I) chain OS=Mus musculus GN=Col1a2 PE=2 SV=2 - [CO1A2_MOUSE]	4.52	1	5	5	11	1.10	1.06	0.93	1.00	0.93	3.37	2.83	1.57	1.31	1.65	1.46	1.15	1.00	129.48	9.19
Q7M6Y6	Maestro heat-like repeat-containing protein family member 2B OS=Mus musculus GN=Mroh2b PE=1 SV=2 - [MRO2B_MOUSE]	0.76	1	1	1	1	0.70	1.03	1.48	0.89	1.27	1.61	2.30	1.57	2.25	1.12	1.61	0.80	1.15	180.36	6.61
E0CYD7	Putative E3 ubiquitin-protein ligase UNKL OS=Mus musculus GN=Unkl PE=2 SV=1 - [E0CYD7_MOUSE]	6.47	3	1	2	8	0.97	0.92	0.95	0.92	0.95	1.46	1.50	1.57	1.61	1.37	1.42	1.14	1.18	58.50	7.78
P35455	Vasopressin-neurophysin 2-copeptin OS=Mus musculus GN=Avp PE=2 SV=1 - [NEU2_MOUSE]	4.76	1	1	1	2	9.62	2.16	0.22	1.60	0.17	2.17	0.22	1.57	0.16	5.96	0.62	3.58	0.37	17.89	7.58
Q4VA61	Down syndrome cell adhesion molecule-like protein 1 homolog OS=Mus musculus GN=Dscaml1 PE=1 SV=2 - [DSCL1_MOUSE]	0.44	2	1	1	2	0.97	1.19	1.22	0.87	0.89	0.92	0.94	1.57	1.61	1.44	1.48	1.57	1.62	224.10	8.16
P15331-3	Isoform 5b of Peripherin OS=Mus musculus GN=Prph - [PERI_MOUSE]	8.46	5	1	4	13	1.22	1.22	1.00	1.37	1.12	1.45	1.18	1.57	1.28	1.38	1.13	1.35	1.11	52.65	5.45
Q8CJ40	Rootletin OS=Mus musculus GN=Crocc PE=1 SV=2 - [CROCC_MOUSE]	31.31	4	52	53	119	1.04	1.10	1.05	0.99	0.95	1.53	1.45	1.57	1.49	1.46	1.41	1.47	1.43	226.81	5.55
Q9QYR9	Acyl-coenzyme A thioesterase 2, mitochondrial OS=Mus musculus GN=Acot2 PE=1 SV=2 - [ACOT2_MOUSE]	16.11	1	2	5	12	1.26	1.34	1.06	1.17	0.92	0.97	0.77	1.58	1.25	1.23	0.97	1.52	1.21	49.63	7.36
E9PWX7	TBC1 domain family member 25 OS=Mus musculus GN=Tbc1d25 PE=2 SV=1 - [E9PWX7_MOUSE]	0.83	2	1	1	1	1.26	0.71	0.56	0.81	0.64	1.54	1.22	1.58	1.25	1.34	1.06	1.22	0.96	80.21	6.37
Q9D3P8	Plasminogen receptor (KT) OS=Mus musculus GN=Plgrkt PE=1 SV=1 - [PLRKT_MOUSE]	7.48	1	1	1	1	1.33	0.86	0.64	1.44	1.08	1.56	1.16	1.59	1.19	1.66	1.25	1.63	1.23	17.25	9.50
F6W8W7	Protein Taf4a (Fragment) OS=Mus musculus GN=Taf4a PE=4 SV=1 - [F6W8W7_MOUSE]	1.76	2	1	1	1	0.89	1.09	1.21	0.80	0.89	1.12	1.25	1.60	1.78	1.14	1.27	1.16	1.30	73.20	9.94

O08576-2	Isoform 2 of RUN domain-containing protein 3A OS=Mus musculus GN=Rundc3a - [RUN3A_MOUSE]	21.75	3	1	5	7	0.90	1.59	1.75	1.11	1.23	1.19	1.31	1.60	1.76	1.29	1.43	1.41	1.56	45.51	5.48
Q9CPV3	39S ribosomal protein L42, mitochondrial OS=Mus musculus GN=Mrpl42 PE=2 SV=1 - [RM42_MOUSE]	9.15	1	1	1	2	0.92	0.69	0.75	0.90	0.98	1.47	1.60	1.60	1.74	1.33	1.45	1.08	1.18	16.48	9.19
Q61495	Desmoglein-1-alpha OS=Mus musculus GN=Dsg1a PE=2 SV=2 - [DSG1A_MOUSE]	1.23	2	1	1	2	2.04	0.81	0.39	6.68	3.27	1.24	0.60	1.60	0.78	1.08	0.53	2.21	1.09	114.52	4.89
A2AR95-3	Isoform 3 of Low-density lipoprotein receptor class A domain-containing protein 3 OS=Mus musculus GN=Ldlrad3 - [LRAD3_MOUSE]	3.51	2	1	1	1	1.03	0.93	0.90	0.54	0.53	1.40	1.36	1.61	1.55	1.12	1.09	1.04	1.02	33.91	5.03
Q6PIX5	Inactive rhomboid protein 1 OS=Mus musculus GN=Rhbdf1 PE=1 SV=2 - [RHDF1_MOUSE]	4.09	5	2	2	4	1.02	1.39	1.36	1.09	1.06	1.30	1.26	1.61	1.57	1.35	1.32	1.30	1.27	97.23	8.59
P63078	Guanine nucleotide-binding protein G(I)/G(S)/G(O) subunit gamma-8 OS=Mus musculus GN=Gng8 PE=2 SV=1 - [GBG8_MOUSE]	22.86	1	1	1	2	1.49	1.30	0.87	1.01	0.68	1.43	0.95	1.61	1.08	1.65	1.11	3.05	2.05	7.84	7.14
Q9QZS0	Collagen alpha-3(IV) chain OS=Mus musculus GN=Col4a3 PE=1 SV=2 - [CO4A3_MOUSE]	0.66	1	1	1	1	0.91	1.13	1.24	0.97	1.07	1.43	1.56	1.61	1.77	1.93	2.12	1.18	1.30	161.62	9.48
Q5RL20	39S ribosomal protein L43, mitochondrial OS=Mus musculus GN=Mrpl43 PE=2 SV=1 - [Q5RL20_MOUSE]	6.92	2	1	1	2	1.03	1.21	1.17	1.22	1.18	1.57	1.51	1.62	1.56	1.57	1.53	1.37	1.33	17.87	10.58
Q9R0A0	Peroxisomal membrane protein PEX14 OS=Mus musculus GN=Pex14 PE=1 SV=1 - [PEX14_MOUSE]	17.55	2	5	5	21	1.06	1.22	1.26	1.08	1.11	1.68	1.58	1.62	1.70	1.63	1.62	1.48	1.41	41.18	5.11
H3BL28	Protein Ppp1r9a (Fragment) OS=Mus musculus GN=Ppp1r9a PE=2 SV=1 - [H3BL28_MOUSE]	48.46	8	1	40	216	1.23	0.88	0.71	1.02	0.83	1.21	0.98	1.62	1.31	1.29	1.05	1.19	0.97	116.41	4.96
Q61550	Double-strand-break repair protein rad21 homolog OS=Mus musculus GN=Rad21 PE=1 SV=3 - [RAD21_MOUSE]	2.05	1	1	1	3	1.12	1.13	1.01	1.33	1.19	1.50	1.34	1.62	1.45	1.70	1.52	1.24	1.11	72.04	4.64

D3Z607	Non-histone chromosomal protein HMG-14 OS=Mus musculus GN=Hmgn1 PE=2 SV=1 - [D3Z607_MOUSE]	47.67	2	3	3	100	1.07	1.13	0.90	0.91	0.81	1.25	0.81	1.63	1.13	1.51	0.95	1.65	1.09	9.02	9.31
A6H611	Mitochondrial intermediate peptidase OS=Mus musculus GN=Mipep PE=2 SV=1 - [MIPEP_MOUSE]	0.98	1	1	1	1	1.50	1.70	1.13	1.29	0.86	0.92	0.61	1.64	1.09	1.59	1.06	1.07	0.72	80.80	7.15
P02535-3	Isoform 3 of Keratin, type I cytoskeletal 10 OS=Mus musculus GN=Krt10 - [K1C10_MOUSE]	10.82	11	3	5	9	1.61	1.53	0.88	1.66	1.11	1.35	0.83	1.64	1.00	1.36	0.83	2.04	1.17	49.47	5.02
F8WGI9	Very low-density lipoprotein receptor OS=Mus musculus GN=Vldlr PE=2 SV=1 - [F8WGI9_MOUSE]	3.36	4	2	2	5	1.07	1.26	1.17	1.20	1.13	1.02	0.99	1.64	1.52	1.36	1.51	1.46	1.63	88.95	4.84
Q6DFY8	Protein FAM5B OS=Mus musculus GN=Fam5b PE=2 SV=1 - [FAM5B_MOUSE]	5.36	1	3	3	4	1.04	1.23	1.18	1.07	1.03	1.06	0.85	1.64	0.75	1.14	0.92	1.11	1.07	89.17	7.88
E9PZ30	Protein Ugt2b34 OS=Mus musculus GN=Ugt2b34 PE=2 SV=1 - [E9PZ30_MOUSE]	11.14	2	2	2	2	0.71	0.50	0.58	0.66	0.94	1.29	1.81	1.65	2.33	1.63	1.89	0.68	0.97	50.11	6.11
Q61033	Lamina-associated polypeptide 2, isoforms alpha/zeta OS=Mus musculus GN=Tmpo PE=1 SV=4 - [LAP2A_MOUSE]	21.50	2	2	11	35	1.00	1.22	1.22	1.09	1.09	1.15	1.15	1.65	1.65	1.26	1.27	1.21	1.22	75.12	8.05
Q5M6W3	Clathrin heavy chain linker domain-containing protein 1 OS=Mus musculus GN=Clhc1 PE=2 SV=1 - [CLHC1_MOUSE]	3.69	1	1	1	1	1.96	0.78	0.40	1.26	0.64	0.52	0.26	1.66	0.84	1.13	0.58	1.31	0.67	69.37	8.25
Q9WVF8	Tumor suppressor candidate 2 OS=Mus musculus GN=Tusc2 PE=1 SV=3 - [TUSC2_MOUSE]	50.00	1	4	4	9	1.04	1.01	0.93	0.96	0.89	1.55	1.47	1.66	1.85	1.50	1.32	1.33	1.44	12.13	9.69
Q9CY34	NEDD8-conjugating enzyme UBE2F OS=Mus musculus GN=Ube2f PE=1 SV=1 - [UB2FA_MOUSE]	11.35	2	2	2	3	1.18	0.99	0.84	1.01	0.86	1.36	1.15	1.67	1.41	1.47	1.25	1.44	1.23	21.10	6.54
Q6PDM1-	Isoform 4 of Male-specific lethal 1 homolog OS=Mus musculus GN=Msl1 - [MSL1_MOUSE]	7.56	10	2	3	5	1.49	0.99	0.66	1.08	0.68	0.91	0.72	1.68	1.38	1.54	1.27	1.14	0.94	49.18	8.94
Q8BLR5	PH and SEC7 domain-containing protein 4 OS=Mus musculus GN=Psd4 PE=2 SV=1 - [PSD4_MOUSE]	1.49	1	1	1	1	1.17	1.13	0.97	1.34	1.14	3.41	2.91	1.68	1.43	1.88	1.61	1.20	1.03	112.66	5.24

G5E8M8	Ligand-dependent nuclear receptor-interacting factor 1 OS=Mus musculus GN=Lrif1 PE=4 SV=1 - [G5E8M8_MOUSE]	12.24	3	2	2	3	0.95	0.78	0.82	0.98	1.03	1.20	1.26	1.68	1.76	1.51	1.59	1.05	1.10	27.03	9.39
A2AEE4	Proline/serine-rich coiled-coil protein 1 (Fragment) OS=Mus musculus GN=Psrc1 PE=2 SV=1 - [A2AEE4_MOUSE]	9.62	2	1	1	3	1.07	1.34	1.25	1.13	1.06	1.34	1.25	1.69	1.57	1.33	1.24	1.44	1.35	27.55	10.67
Q8BUM6	Protein FAM163B OS=Mus musculus GN=Fam163b PE=1 SV=1 - [F163B_MOUSE]	14.37	1	1	1	2	1.03	1.12	1.09	1.02	0.98	1.68	1.62	1.70	1.64	1.51	1.47	1.08	1.05	18.29	5.15
B1AYB7	Methyl-CpG-binding domain protein 5 OS=Mus musculus GN=Mbd5 PE=2 SV=1 - [B1AYB7_MOUSE]	4.21	4	1	1	1	1.34	1.53	1.13	0.94	0.70	1.30	0.96	1.73	1.29	0.63	0.47	1.04	0.77	90.01	9.54
Q8C5R2	Proline and serine-rich protein 2 OS=Mus musculus GN=Proser2 PE=1 SV=3 - [PRSR2_MOUSE]	3.40	1	1	1	2	1.09	1.10	1.00	0.97	0.89	1.46	1.33	1.75	1.59	1.46	1.34	1.08	0.98	50.39	6.30
G3UZ12	Protein Jumonji OS=Mus musculus GN=Jarid2 PE=2 SV=1 - [G3UZ12_MOUSE]	18.18	2	1	1	1	1.00	1.01	1.01	0.93	0.93	1.67	1.66	1.75	1.74	1.64	1.64	1.53	1.53	4.76	8.02
Q8BZR9	Uncharacterized protein C17orf85 homolog OS=Mus musculus PE=1 SV=1 - [CQ085_MOUSE]	5.20	3	2	2	5	1.11	1.31	1.18	1.34	1.21	2.14	1.92	1.75	1.57	1.54	1.39	1.24	1.12	70.00	5.80
M0QWU0	CXXC-type zinc finger protein 4 OS=Mus musculus GN=Cxxc4 PE=4 SV=1 - [M0QWU0_MOUSE]	16.12	1	2	2	3	0.99	1.05	1.07	1.04	1.05	1.29	1.30	1.75	1.77	1.63	1.65	1.46	1.48	36.42	9.07
Q8BGT0	Osteopetrosis-associated transmembrane protein 1 OS=Mus musculus GN=Ostm1 PE=1 SV=1 - [OSTM1_MOUSE]	4.44	1	1	1	2	0.95	1.11	1.18	1.16	1.22	1.70	1.79	1.76	1.85	1.75	1.85	1.95	2.06	37.98	6.39
P23949	Zinc finger protein 36, C3H1 type-like 2 OS=Mus musculus GN=Zfp36l2 PE=2 SV=1 - [TISD_MOUSE]	5.45	2	1	1	1	1.22	0.99	0.81	1.29	1.06	1.62	1.32	1.80	1.47	1.31	1.07	1.26	1.03	37.57	8.68
Q80T23-2	Isoform 2 of Synaptotagmin-like protein 5 OS=Mus musculus GN=Sytl5 - [SYTL5_MOUSE]	2.05	2	1	1	2	1.53	1.66	1.08	1.40	0.91	1.48	0.96	1.80	1.17	2.12	1.38	2.09	1.37	81.66	8.78

Q03719	Potassium voltage-gated channel subfamily D member 1 OS=Mus musculus GN=Kcnd1 PE=1 SV=1 - [KCND1_MOUSE]	3.53	1	1	1	1	2.77	15.50	5.59	3.17	1.14	3.48	1.25	1.80	0.65	2.37	0.86	2.16	0.78	71.65	8.21
Q8VE92	RNA-binding protein 4B OS=Mus musculus GN=Rbm4b PE=1 SV=1 - [RBM4B_MOUSE]	31.65	4	1	10	20	1.01	1.01	1.00	1.00	0.99	1.52	1.50	1.81	1.78	1.42	1.40	1.54	1.53	39.97	6.74
Q0PHV7	Dapper homolog 3 OS=Mus musculus GN=Dact3 PE=1 SV=1 - [DACT3_MOUSE]	4.75	1	2	2	4	1.05	1.09	1.04	0.94	0.89	1.13	1.07	1.81	1.72	1.42	1.35	1.65	1.57	63.25	10.42
Q5SRX1-	Isoform 2 of TOM1-like protein 2 OS=Mus musculus GN=Tom1l2 - [TM1L2_MOUSE]	46.82	6	1	18	142	0.86	0.82	0.95	1.05	1.22	1.15	1.33	1.81	2.11	1.53	1.78	1.25	1.46	53.35	4.87
Q99ML1	Bcl-2-binding component 3 OS=Mus musculus GN=Bbc3 PE=1 SV=1 - [BBC3_MOUSE]	4.15	1	1	1	3	1.12	1.22	1.09	0.97	0.87	1.64	1.46	1.82	1.62	1.82	1.63	1.66	1.49	20.74	7.42
E9Q3H6	Protein Coq10a OS=Mus musculus GN=Coq10a PE=2 SV=1 - [E9Q3H6_MOUSE]	3.86	1	1	1	1	1.55	1.88	1.21	2.13	1.37	2.52	1.61	1.83	1.17	2.21	1.42	1.41	0.91	28.91	9.82
Q9CR37	Pancreatic progenitor cell differentiation and proliferation factor OS=Mus musculus GN=Ppdpf PE=2 SV=1 - [PPDPF_MOUSE]	13.04	1	1	1	1	1.21	0.71	0.59	1.18	0.97	0.98	0.81	1.87	1.54	1.93	1.60	1.41	1.17	12.25	7.44
Q99M46	DNA-directed RNA polymerase II subunit RPB3 OS=Mus musculus GN=Polr2c PE=2 SV=1 - [Q99M46_MOUSE]	13.82	2	2	2	2	1.24	1.73	1.39	1.19	0.96	1.56	1.25	1.87	1.51	1.70	1.37	1.94	1.57	31.42	4.92
P97304	DNA-directed RNA polymerases I and III subunit RPAC2 OS=Mus musculus GN=Polr1d PE=2 SV=1 - [RPAC2_MOUSE]	21.80	1	2	2	3	1.02	0.86	0.84	0.97	0.95	1.76	1.70	1.88	1.83	1.52	1.49	1.49	1.46	15.06	6.90
D3Z723	Nuclear transcription factor Y subunit beta (Fragment) OS=Mus musculus GN=Nfyb PE=2 SV=1 - [D3Z723_MOUSE]	12.26	2	1	1	2	1.06	1.46	1.38	1.10	1.04	1.70	1.60	1.91	1.81	1.66	1.58	1.49	1.41	11.67	4.58
Q8BW74	Hepatic leukemia factor OS=Mus musculus GN=Hlf PE=2 SV=1 - [HLF_MOUSE]	5.76	1	1	1	2	0.65	0.90	1.37	0.71	1.09	1.62	2.48	1.92	2.94	1.60	2.46	1.24	1.91	33.13	8.32
Q60960	Importin subunit alpha-5 OS=Mus musculus GN=Kpna1 PE=1 SV=2 - [IMA5_MOUSE]	10.78	4	1	4	7	0.99	1.66	1.67	1.31	1.32	1.02	1.02	1.94	1.94	1.29	1.31	1.82	1.83	60.14	5.01

P07309	Transthyretin OS=Mus musculus GN=Ttr PE=1 SV=1 - [TTHY_MOUSE]	15.65	1	2	2	3	1.74	1.81	1.04	1.93	1.11	2.59	1.48	2.00	1.15	2.77	1.60	2.38	1.37	15.77	6.16
A2AJ76	Hemicentin-2 OS=Mus musculus GN=Hmcn2 PE=1 SV=1 - [HMCN2_MOUSE]	0.31	1	1	1	2	1.39	1.27	0.91	1.77	1.27	2.76	1.97	2.00	1.43	3.62	2.60	2.79	2.01	546.85	5.71
Q8K136	Sodium channel modifier 1 OS=Mus musculus GN=Scnm1 PE=1 SV=1 - [SCNM1_MOUSE]	11.79	2	2	2	2	1.83	1.03	0.56	4.08	2.23	1.45	0.79	2.03	1.10	1.38	0.76	2.11	1.16	25.81	8.66
Q8BP48	Methionine aminopeptidase 1 OS=Mus musculus GN=Metap1 PE=2 SV=1 - [MAP11_MOUSE]	3.37	1	1	1	1	1.00	1.02	1.02	1.13	1.13	1.35	1.34	2.04	2.04	1.93	1.93	1.84	1.84	43.19	7.17
E0CZF0	Uncharacterized protein OS=Mus musculus GN=Smdt1 PE=2 SV=1 - [E0CZF0_MOUSE]	29.58	2	1	1	3	1.45	1.16	0.80	1.38	0.95	1.44	0.99	2.04	1.40	1.64	1.13	1.66	1.14	8.14	4.61
Q9DBK0	Acyl-coenzyme A thioesterase 12 OS=Mus musculus GN=Acot12 PE=2 SV=1 - [ACO12_MOUSE]	1.80	1	1	1	1	3.10	30.26	9.73	4.28	1.38	4.54	1.45	2.06	0.66	1.88	0.61	1.38	0.44	61.72	7.18
Q8BHE4	Transmembrane protein 108 OS=Mus musculus GN=Tmem108 PE=2 SV=1 - [TM108_MOUSE]	2.79	1	1	1	2	1.88	1.61	0.86	1.19	0.63	2.27	1.20	2.08	1.11	1.54	0.82	1.33	0.71	59.61	8.44
D3YW07	Neuroglobin OS=Mus musculus GN=Ngb PE=2 SV=1 - [D3YW07_MOUSE]	20.65	3	1	1	2	2.24	1.48	0.66	1.56	0.70	1.13	0.50	2.12	0.94	2.06	0.92	1.87	0.84	10.38	6.76
E0CZ73	Occludin OS=Mus musculus GN=Ocln PE=2 SV=1 - [E0CZ73_MOUSE]	17.65	2	2	2	6	1.26	1.39	1.10	1.48	1.17	1.92	1.51	2.15	1.69	2.11	1.67	1.54	1.22	31.59	5.87
E9Q813	Ras-GEF domain-containing family member 1B OS=Mus musculus GN=Rasgef1b PE=2 SV=1 - [E9Q813_MOUSE]	7.42	3	1	1	1	1.26	1.80	1.43	1.43	1.14	1.74	1.37	2.15	1.70	1.98	1.57	1.83	1.46	50.26	8.06
D3Z4F2	Protein Zfp819 (Fragment) OS=Mus musculus GN=Zfp819 PE=2 SV=1 - [D3Z4F2_MOUSE]	12.12	2	1	1	1	0.97	0.98	1.00	1.01	1.04	1.74	1.78	2.16	2.21	1.77	1.82	1.60	1.65	11.49	5.48
A6PWD2-	Isoform 4 of Forkhead-associated domain-containing protein 1 OS=Mus musculus GN=Fhad1 - [FHAD1_MOUSE]	1.64	2	1	1	1	2.07	2.07	1.00	2.07	1.00	1.91	0.92	2.21	1.06	2.22	1.07	3.41	1.65	99.09	6.83

Q9CY58	Plasminogen activator inhibitor 1 RNA-binding protein OS=Mus musculus GN=Serbp1 PE=1 SV=2 - [PAIRB_MOUSE]	48.40	2	2	17	101	2.01	1.14	0.57	1.39	0.69	1.89	0.94	2.23	1.11	2.01	1.01	1.48	0.74	44.69	8.54
Q8VED5	Keratin, type II cytoskeletal 79 OS=Mus musculus GN=Krt79 PE=1 SV=2 - [K2C79_MOUSE]	4.90	3	1	3	4	1.75	1.20	0.68	5.33	3.05	1.41	0.80	2.27	1.29	1.77	1.01	1.43	0.82	57.52	7.69
A2AIV8	Caspase recruitment domain family, member 9 OS=Mus musculus GN=Card9 PE=2 SV=1 - [A2AIV8_MOUSE]	3.54	1	1	1	2	2.26	24.62	10.88	5.75	2.55	6.67	2.94	2.28	1.00	3.17	1.41	1.48	0.65	62.42	5.73
Q91XQ0-	Isoform 2 of Dynein heavy chain 8, axonemal OS=Mus musculus GN=Dnahc8 - [DYH8_MOUSE]	0.36	2	1	1	1	3.22	30.33	9.40	5.20	1.61	5.60	1.73	2.28	0.70	1.92	0.60	1.41	0.44	479.85	6.13
Q80YR6	DNA endonuclease RBBP8 OS=Mus musculus GN=Rbbp8 PE=1 SV=1 - [COM1_MOUSE]	0.78	1	1	1	1	1.70	0.73	0.43	5.82	3.42	1.20	0.70	2.32	1.36	1.27	0.75	2.13	1.25	100.77	6.68
F6VV25	Pleckstrin homology domain-containing family O member 1 (Fragment) OS=Mus musculus GN=Plekho1 PE=2 SV=1 - [F6VV25_MOUSE]	7.98	1	1	1	1	0.99	1.08	1.09	1.09	1.10	1.51	1.53	2.33	2.36	1.93	1.96	1.39	1.41	29.41	8.81
Q6NXL1	Protein Sec24d OS=Mus musculus GN=Sec24d PE=2 SV=1 - [Q6NXL1_MOUSE]	2.71	1	1	1	1	2.03	2.53	1.25	3.45	1.70	2.81	1.38	2.34	1.15	2.10	1.04	3.10	1.53	112.60	7.18
Q3TTY5	Keratin, type II cytoskeletal 2 epidermal OS=Mus musculus GN=Krt2 PE=1 SV=1 - [K22E_MOUSE]	13.15	2	3	5	8	1.91	1.55	0.81	3.32	1.74	1.53	0.91	2.66	1.44	1.76	0.92	2.21	1.27	70.88	8.06
Q8VGU8	Olfactory receptor 1353 OS=Mus musculus GN=Olfr1353 PE=2 SV=1 - [Q8VGU8_MOUSE]	3.24	1	1	1	2	2.14	19.20	8.93	3.41	1.59	3.63	1.69	2.73	1.27	2.28	1.06	1.41	0.66	34.98	8.37
Q80YF9	Rho GTPase-activating protein 33 OS=Mus musculus GN=Arhgap33 PE=1 SV=1 - [RHG33_MOUSE]	4.83	4	2	4	10	1.02	1.30	1.26	1.18	1.15	0.87	0.86	3.36	3.37	2.56	2.59	1.71	1.73	139.71	9.19
E9PUE6	Transmembrane protein 145 OS=Mus musculus GN=Tmem145 PE=2 SV=1 - [E9PUE6_MOUSE]	5.51	2	2	2	3	0.89	1.04	1.17	0.96	1.07	1.14	1.27	3.93	4.39	3.27	3.67	1.66	1.87	62.79	8.75

Q3UV17	Keratin, type II cytoskeletal 2 oral OS=Mus musculus GN=Krt76 PE=2 SV=1 - [K22O_MOUSE]	5.22	4	1	4	7	2.65	1.15	0.43	8.16	3.07	1.83	0.69	4.15	1.56	2.58	0.97	3.36	1.27	62.81	8.43
O70551	SRSF protein kinase 1 OS=Mus musculus GN=Srpk1 PE=1 SV=2 - [SRPK1_MOUSE]	4.78	2	1	3	4	0.75	1.14	1.51	1.08	1.43	0.89	1.18	8.48	11.20	6.30	8.36	1.93	2.56	73.04	6.19
E9PXF7	Protein Gm14446 OS=Mus musculus GN=Gm14446 PE=2 SV=1 - [E9PXF7_MOUSE]	4.04	2	1	2	5	1.07	0.75	0.70	2.07	1.94	3.63	3.39	10.23	9.56	8.60	8.08	3.88	3.64	54.44	8.16
