# Supplementary material for: Are you also what your mother eats? Distinct proteomic portrait as a result of maternal high-fat diet in the cerebral cortex of the adult mouse
Source: Int J Obes (Lond). 2015 Apr 21;39(8):1325–8. doi: 10.1038/ijo.2015.35 (PMC5399160; doi:10.1038/ijo.2015.35)
Supplement: Supplementary Table 2 [file ijo201535x4.pdf]

Accesion	Description	114/113	115/113	115/114	116/113	116/114	117/113	117/114	118/113	118/114	119/113	119/114	121/113	121/114
Q61897	Keratin, type I cuticular Ha3-II OS=Mus musculus GN=Krt33b PE=2 SV=2 - [KT33B_MOUSE]	-0.02	0.37	0.41	3.75	3.78	0.24	0.18	0.40	0.34	0.62	0.57	0.25	0.27
A2AIV8	Caspase recruitment domain family, member 9 OS=Mus musculus GN=Card9 PE=2 SV=1 - [A2AIV8_MOUSE]	1.17	4.62	3.44	2.52	1.35	2.74	1.55	1.19	0.01	1.66	0.49	0.56	-0.61
E9PYL4	H(+)/Cl(-) exchange transporter 7 OS=Mus musculus GN=Clcn7 PE=2 SV=1 - [E9PYL4_MOUSE]	-0.18	1.30	1.48	0.91	1.09	0.41	0.59	-0.05	0.13	0.37	0.56	0.99	1.18
E9PUY1	28S ribosomal protein S12, mitochondrial (Fragment) OS=Mus musculus GN=Mrps12 PE=2 SV=1 - [E9PUY1_MOUSE]	-0.17	0.43	0.60	0.81	0.98	1.34	1.50	0.63	0.79	0.36	0.53	1.09	1.27
Q7TNG8	Probable D-lactate dehydrogenase, mitochondrial OS=Mus musculus GN=Ldhd PE=1 SV=1 - [LDHD_MOUSE]	-0.11	0.82	0.93	0.69	0.80	0.22	0.33	0.14	0.24	0.57	0.68	0.13	0.24
Q640M6	Glycerophosphodiester phosphodiesterase domain-containing protein 5 OS=Mus musculus GN=Gdpd5 PE=2 SV=1 - [GDPD5_MOUSE]	-0.33	0.37	0.70	0.45	0.78	-0.87	-0.55	-1.17	-0.85	-0.17	0.16	0.06	0.39
P38585	Tubulin--tyrosine ligase OS=Mus musculus GN=Ttl PE=2 SV=2 - [TTL_MOUSE]	-0.11	0.40	0.51	0.59	0.70	0.22	0.32	-0.08	0.03	0.13	0.24	0.22	0.33
Q9DC29	ATP-binding cassette sub-family B member 6, mitochondrial OS=Mus musculus GN=Abcb6 PE=1 SV=1 - [ABCB6_MOUSE]	-0.04	0.45	0.49	0.65	0.69	0.04	0.07	0.20	0.24	0.04	0.08	0.09	0.14
Q91XQ0-2	Isoform 2 of Dynein heavy chain 8, axonemal OS=Mus musculus GN=Dnahc8 - [DYH8_MOUSE]	1.69	4.92	3.23	2.38	0.69	2.48	0.79	1.19	-0.51	0.94	-0.74	0.50	-1.19
Q8VGU8	Olfactory receptor 1353 OS=Mus musculus GN=Olfr1353 PE=2 SV=1 - [Q8VGU8_MOUSE]	1.10	4.26	3.16	1.77	0.67	1.86	0.75	1.45	0.34	1.19	0.09	0.50	-0.60
Q04886	Transcription factor SOX-8 OS=Mus musculus GN=Sox8 PE=2 SV=2 - [SOX8_MOUSE]	-0.08	2.14	2.22	0.58	0.66	0.48	0.55	0.16	0.24	0.09	0.17	-0.18	-0.10
A2ARS0	Ankyrin repeat domain-containing protein 63 OS=Mus musculus GN=Ankrd63 PE=4 SV=1 - [ANR63_MOUSE]	-0.02	0.39	0.47	0.53	0.66	0.07	0.06	-0.32	-0.32	0.45	0.47	0.72	0.76
O54828	Regulator of G-protein signaling 9 OS=Mus musculus GN=Rgs9 PE=1 SV=3 - [RGS9_MOUSE]	-0.12	0.42	0.57	0.39	0.63	0.00	0.26	-0.31	-0.33	0.34	0.66	0.77	0.83
D3YWY6	Mitochondrial pyruvate carrier 1 OS=Mus musculus GN=Mpc1 PE=2 SV=1 - [D3YWY6_MOUSE]	-0.09	0.39	0.47	0.52	0.61	0.16	0.24	-0.05	0.03	0.07	0.16	-0.15	-0.06
E9Q5B2	Protein 0610011F06Rik OS=Mus musculus GN=0610011F06Rik PE=2 SV=1 - [E9Q5B2_MOUSE]	-0.03	0.43	0.45	0.53	0.55	0.22	0.23	0.17	0.19	0.29	0.32	0.46	0.49
Q8C0K8	Protein Uvrag OS=Mus musculus GN=Uvrag PE=2 SV=1 - [Q8C0K8_MOUSE]	-0.29	0.85	1.13	0.26	0.55	0.10	0.38	0.00	0.28	-0.06	0.22	-0.01	0.28
Q3UIA2-4	Isoform 4 of Rho GTPase-activating protein 17 OS=Mus musculus GN=Arhgap17 - [RHG17_MOUSE]	-0.28	0.44	0.72	0.25	0.53	-0.10	0.17	0.36	0.64	0.19	0.48	0.10	0.39

Q91V61-2	Isoform 2 of Sideroflexin-3 OS=Mus musculus GN=Sfxn3 - [SFXN3_MOUSE]	-0.12	0.44	0.40	0.37	0.52	-0.75	-0.58	-0.75	-0.67	-0.38	-0.25	-0.39	-0.26
E9Q933	Transmembrane protein 11, mitochondrial OS=Mus musculus GN=Tmem11 PE=2 SV=1 - [E9Q933_MOUSE]	0.11	0.51	0.40	0.61	0.51	0.01	-0.11	0.01	-0.10	0.15	0.05	-0.06	-0.16
P28740-2	Isoform 2 of Kinesin-like protein KIF2A OS=Mus musculus GN=Kif2a - [KIF2A_MOUSE]	-0.19	0.46	0.65	0.30	0.50	0.22	0.41	-0.12	0.07	0.45	0.64	0.53	0.72
J3QPB5	Nucleoporin NDC1 OS=Mus musculus GN=Tmem48 PE=4 SV=1 - [J3QPB5_MOUSE]	-0.19	0.53	0.71	0.31	0.50	-0.30	-0.12	-0.09	0.09	-0.01	0.18	0.22	0.40
D3YYP5	Non-syndromic hearing impairment protein 5 homolog OS=Mus musculus GN=Dfna5 PE=2 SV=2 - [D3YYP5_MOUSE]	-0.19	0.47	0.66	0.30	0.49	0.03	0.21	0.11	0.30	0.02	0.21	0.44	0.63
Q9CYN9	Renin receptor OS=Mus musculus GN=Atp6ap2 PE=2 SV=2 - [RENR_MOUSE]	-0.03	0.52	0.37	0.35	0.48	0.06	0.13	0.15	0.17	0.08	0.22	0.31	0.35
Q9DBK0	Acyl-coenzyme A thioesterase 12 OS=Mus musculus GN=Acot12 PE=2 SV=1 - [ACO12_MOUSE]	1.63	4.92	3.28	2.10	0.46	2.18	0.54	1.04	-0.60	0.91	-0.72	0.46	-1.17
P58158	Galactosylgalactosylxylosylprotein 3-beta-glucuronosyltransferase 3 OS=Mus musculus GN=B3gat3 PE=2 SV=1 - [B3GA3_MOUSE]	0.02	0.43	0.41	0.47	0.45	-0.04	-0.06	0.02	-0.01	-0.19	-0.21	0.04	0.02
Q9Z2Y3-5	Isoform 5 of Homer protein homolog 1 OS=Mus musculus GN=Homer1 - [HOME1_MOUSE]	-0.04	0.54	0.58	0.40	0.44	0.27	0.31	0.12	0.15	-0.12	-0.07	0.23	0.27
Q60936	Chaperone activity of bc1 complex-like, mitochondrial OS=Mus musculus GN=Adck3 PE=2 SV=2 - [ADCK3_MOUSE]	0.18	0.61	0.44	0.59	0.41	0.04	-0.14	0.03	-0.15	-0.15	-0.32	0.25	0.07
A2A5R2	Brefeldin A-inhibited guanine nucleotide-exchange protein 2 OS=Mus musculus GN=Arfgef2 PE=1 SV=1 - [BIG2_MOUSE]	-0.06	0.42	0.50	0.37	0.41	-0.08	-0.02	0.14	0.19	0.19	0.28	0.32	0.36
P51906	Excitatory amino acid transporter 3 OS=Mus musculus GN=Slc1a1 PE=1 SV=2 - [EAA3_MOUSE]	0.07	0.51	0.44	0.48	0.41	0.08	0.01	-0.12	-0.19	-0.15	-0.21	0.20	0.13
Q60960	Importin subunit alpha-5 OS=Mus musculus GN=Kpna1 PE=1 SV=2 - [IMA5_MOUSE]	-0.01	0.73	0.74	0.39	0.40	0.03	0.03	0.95	0.96	0.37	0.38	0.86	0.87
Q8K1M6-3	Isoform 3 of Dynamin-1-like protein OS=Mus musculus GN=Dnm1l - [DNM1L_MOUSE]	-0.07	0.39	0.45	0.32	0.38	0.05	0.11	-0.07	-0.01	0.01	0.07	0.00	0.06
Q9D880	Mitochondrial import inner membrane translocase subunit TIM50 OS=Mus musculus GN=Timm50 PE=1 SV=1 - [TIM50_MOUSE]	-0.03	0.40	0.40	0.32	0.37	-0.20	-0.13	-0.15	-0.20	-0.03	0.05	0.08	0.06
F6Z458	Endoplasmic reticulum lectin 1 (Fragment) OS=Mus musculus GN=Erlec1 PE=2 SV=1 - [F6Z458_MOUSE]	-0.01	0.72	0.73	0.36	0.36	0.31	0.32	0.01	0.02	0.00	0.02	0.04	0.05
Q62132-3	Isoform Gamma of Receptor-type tyrosine-protein phosphatase R OS=Mus musculus GN=Ptprr - [PTPRR_MOUSE]	-0.05	0.51	0.55	0.32	0.36	-0.03	0.01	0.36	0.40	0.40	0.45	0.18	0.22

Q8R5A6	TBC1 domain family member 22A OS=Mus musculus GN=Tbc1d22a PE=2 SV=3 - [TB22A_MOUSE]	-0.08	0.62	0.70	0.29	0.36	0.11	0.17	0.06	0.13	0.15	0.23	0.33	0.40
F6QP10	Sn1-specific diacylglycerol lipase beta (Fragment) OS=Mus musculus GN=Daglb PE=4 SV=2 - [F6QP10_MOUSE]	-0.10	0.38	0.47	0.26	0.36	0.46	0.55	0.00	0.09	0.02	0.12	0.34	0.44
A3KG01	Eph receptor B2 OS=Mus musculus GN=Ephb2 PE=2 SV=2 - [A3KG01_MOUSE]	0.04	0.54	0.61	0.26	0.33	0.03	-0.03	0.10	0.06	-0.08	-0.12	-0.03	-0.07
Q3ZK22-3	Isoform 3 of Vezatin OS=Mus musculus GN=Vezt - [VEZA_MOUSE]	0.23	0.58	0.35	0.55	0.31	0.03	-0.21	-0.08	-0.32	0.17	-0.06	0.30	0.07
P61358	60S ribosomal protein L27 OS=Mus musculus GN=Rpl27 PE=2 SV=2 - [RL27_MOUSE]	-0.01	0.45	0.46	0.28	0.30	0.16	0.03	-0.04	-0.06	-0.06	-0.09	-0.04	-0.05
Q8BPB5	EGF-containing fibulin-like extracellular matrix protein 1 OS=Mus musculus GN=Efemp1 PE=2 SV=1 - [FBLN3_MOUSE]	0.01	0.50	0.49	0.30	0.29	0.33	0.32	0.15	0.13	0.33	0.33	0.29	0.28
Q6GQS1	Calcium-binding mitochondrial carrier protein SCaMC-3 OS=Mus musculus GN=Slc25a23 PE=2 SV=1 - [SCMC3_MOUSE]	0.22	0.61	0.39	0.44	0.29	-0.13	-0.38	-0.08	-0.32	-0.03	-0.24	0.06	0.01
A2AP32	NADH dehydrogenase [ubiquinone] 1 beta subcomplex subunit 6 OS=Mus musculus GN=Ndufb6 PE=2 SV=1 - [A2AP32_MOUSE]	-0.08	0.69	0.64	0.29	0.28	0.02	0.09	-0.02	0.09	0.05	0.07	0.06	0.04
Q8R1C0	Potassium voltage-gated channel subfamily C member 4 OS=Mus musculus GN=Kcnc4 PE=2 SV=1 - [KCNC4_MOUSE]	0.04	0.39	0.35	0.32	0.28	0.00	-0.05	0.13	0.08	0.24	0.19	0.02	-0.02
A9C437	Chloride channel protein 2 OS=Mus musculus GN=Clcn2 PE=2 SV=1 - [A9C437_MOUSE]	-0.03	0.44	0.47	0.24	0.27	-0.01	0.01	0.14	0.17	0.00	0.03	-0.14	-0.11
Q9JHS3	Ragulator complex protein LAMTOR2 OS=Mus musculus GN=Lamtor2 PE=1 SV=1 - [LTOR2_MOUSE]	0.66	1.16	0.49	0.94	0.27	-0.02	-0.68	0.19	-0.47	1.21	0.55	1.24	0.59
Q04859-3	Isoform 3 of Serine/threonine-protein kinase MAK OS=Mus musculus GN=Mak - [MAK_MOUSE]	-0.02	0.64	0.65	0.25	0.27	0.19	0.20	-0.27	-0.26	0.04	0.06	0.03	0.05
P28651	Carbonic anhydrase-related protein OS=Mus musculus GN=Ca8 PE=1 SV=5 - [CAH8_MOUSE]	0.00	1.30	1.29	0.27	0.27	0.44	0.43	-0.18	-0.19	0.10	0.10	0.14	0.14
Q8BM72	Heat shock 70 kDa protein 13 OS=Mus musculus GN=Hspa13 PE=2 SV=1 - [HSP13_MOUSE]	0.02	0.38	0.35	0.29	0.27	0.05	0.01	0.10	0.07	0.27	0.25	0.33	0.31
D3Z4I0	Protein Pigg OS=Mus musculus GN=Pigg PE=2 SV=1 - [D3Z4I0_MOUSE]	-0.01	0.63	0.64	0.26	0.27	-0.22	-0.22	-0.26	-0.26	0.28	0.29	0.22	0.23
Q8R1F1	Niban-like protein 1 OS=Mus musculus GN=Fam129b PE=1 SV=2 - [NIBL1_MOUSE]	0.01	0.41	0.41	0.27	0.26	-0.18	-0.19	-0.03	-0.04	-0.01	-0.02	0.29	0.28
Q8R3Q0	Store-operated calcium entry-associated regulatory factor OS=Mus musculus GN=Tmem66 PE=2 SV=2 - [SARAF_MOUSE]	0.04	0.78	0.74	0.30	0.26	-0.04	-0.09	-0.06	-0.10	0.18	0.14	-0.04	-0.08

Q9JKV5	Secretory carrier-associated membrane protein 4 OS=Mus musculus GN=Scamp4 PE=2 SV=1 - [SCAM4_MOUSE]	0.23	0.82	0.58	0.49	0.26	0.24	0.00	0.14	-0.10	0.15	-0.08	0.03	-0.20
Q8K1C0-4	Isoform 4 of Protein angel homolog 2 OS=Mus musculus GN=Angel2 - [ANGE2_MOUSE]	0.04	0.57	0.53	0.30	0.26	-0.13	-0.18	-0.01	-0.06	0.25	0.21	0.23	0.19
P27545	Ceramide synthase 1 OS=Mus musculus GN=Cers1 PE=1 SV=1 - [CERS1_MOUSE]	0.21	1.38	1.16	0.46	0.25	-0.24	-0.46	-0.68	-0.89	-0.02	-0.23	0.17	-0.03
Q3ZAQ4	Autophagy 9-like 1 protein OS=Mus musculus GN=Atg9a PE=2 SV=1 - [Q3ZAQ4_MOUSE]	0.18	0.57	0.39	0.42	0.24	0.17	-0.02	0.29	0.10	0.13	-0.04	0.18	0.00
A2AEK3	Cleavage stimulation factor subunit 2 (Fragment) OS=Mus musculus GN=Cstf2 PE=2 SV=1 - [A2AEK3_MOUSE]	0.23	0.57	0.34	0.46	0.24	0.58	0.35	0.51	0.27	0.50	0.28	0.43	0.21
Q91XB7	Protein YIF1A OS=Mus musculus GN=Yif1a PE=2 SV=1 - [YIF1A_MOUSE]	1.03	-0.34	-1.38	-0.27	-1.31	-0.17	-1.21	-0.39	-1.43	-0.50	-1.53	-0.49	-1.52
I7HPY0	SH3 domain-binding glutamic acid-rich-like protein 3 OS=Mus musculus GN=Sh3bgrl3 PE=4 SV=1 - [I7HPY0_MOUSE]	-0.18	-0.52	-0.34	-1.24	-1.06	-0.22	-0.04	-0.35	-0.18	-0.62	-0.43	-0.33	-0.15
O08677-2	Isoform LMW of Kininogen-1 OS=Mus musculus GN=Kng1 - [KNG1_MOUSE]	-0.17	-0.36	-0.33	-1.08	-0.92	0.02	0.19	-0.03	0.03	0.15	0.22	-0.64	-0.60
P01878	Ig alpha chain C region OS=Mus musculus PE=1 SV=1 - [IGHA_MOUSE]	0.27	-0.55	-1.07	-0.57	-0.84	-1.00	-1.47	-0.85	-1.22	-0.48	-0.97	-1.02	-1.38
F8WHS3	Complement C1q tumor necrosis factor-related protein 5 (Fragment) OS=Mus musculus GN=C1qtnf5 PE=2 SV=1 - [F8WHS3_MOUSE]	0.42	-0.40	-0.82	-0.38	-0.80	0.07	-0.35	0.19	-0.24	0.35	-0.06	0.79	0.38
Q6A044-2	Isoform 2 of Protein FAM189A1 OS=Mus musculus GN=Fam189a1 - [F1891_MOUSE]	0.35	-1.28	-1.63	-0.35	-0.70	0.42	0.06	0.40	0.05	-0.17	-0.51	-0.25	-0.59
E9PWX7	TBC1 domain family member 25 OS=Mus musculus GN=Tbc1d25 PE=2 SV=1 - [E9PWX7_MOUSE]	0.34	-0.49	-0.83	-0.30	-0.64	0.63	0.28	0.66	0.32	0.42	0.08	0.28	-0.05
Q64288	Olfactory marker protein OS=Mus musculus GN=Omp PE=1 SV=3 - [OMP_MOUSE]	-0.46	-1.58	-1.18	-1.00	-0.59	0.41	0.91	-1.02	-0.72	-0.51	-0.10	0.63	1.14
O70250	Phosphoglycerate mutase 2 OS=Mus musculus GN=Pgam2 PE=1 SV=3 - [PGAM2_MOUSE]	0.24	-0.37	-0.61	-0.31	-0.59	-0.96	-1.20	-1.09	-1.35	-0.87	-1.13	-1.14	-1.38
Q8BHA3	Probable D-tyrosyl-tRNA(Tyr) deacylase 2 OS=Mus musculus GN=Dtd2 PE=2 SV=1 - [DTD2_MOUSE]	0.18	-0.35	-0.62	-0.41	-0.57	-0.81	-1.14	-0.90	-1.23	-1.28	-1.39	-0.73	-0.83
P0C913	Overexpressed in colon carcinoma 1 protein homolog OS=Mus musculus PE=3 SV=1 - [OCC1_MOUSE]	-0.02	-0.55	-0.49	-0.28	-0.56	0.02	-0.26	-0.13	-0.12	-0.08	-0.15	-0.38	-0.36
O88653	Ragulator complex protein LAMTOR3 OS=Mus musculus GN=Lamtor3 PE=1 SV=1 - [LTOR3_MOUSE]	0.24	-0.90	-1.13	-0.30	-0.54	-0.64	-0.88	-0.71	-0.95	-1.00	-1.23	-0.94	-1.17
Q3TRP8	Cartilage acidic protein 1 OS=Mus musculus GN=Cep68 PE=2 SV=1 - [Q3TRP8_MOUSE]	-0.04	-0.97	-0.93	-0.58	-0.54	-0.08	-0.05	0.17	0.21	-0.94	-0.89	-0.36	-0.31

Q7TMK9	Heterogeneous nuclear ribonucleoprotein Q OS=Mus musculus GN=Syncrip PE=1 SV=2 - [HNRPQ_MOUSE]	-0.10	-0.65	-0.55	-0.62	-0.52	-0.27	-0.17	-0.12	-0.02	-0.79	-0.68	-0.51	-0.41
P00015	Cytochrome c, testis-specific OS=Mus musculus GN=Cyct PE=1 SV=3 - [CYC2_MOUSE]	-0.28	-1.03	-0.76	-0.79	-0.51	-0.83	-0.55	-0.67	-0.39	-0.96	-0.67	-0.93	-0.64
D3YWD1	Protein Col6a3 (Fragment) OS=Mus musculus GN=Col6a3 PE=4 SV=2 - [D3YWD1_MOUSE]	0.10	-0.48	-0.57	-0.40	-0.49	-0.01	-0.11	-0.49	-0.59	0.14	0.04	0.11	0.02
P01837	Ig kappa chain C region OS=Mus musculus PE=1 SV=1 - [IGKC_MOUSE]	0.25	-0.47	-0.73	-0.38	-0.47	-0.34	-0.65	-0.56	-0.62	0.33	0.24	-0.35	-0.46
Q8CIN6	CUGBP Elav-like family member 3 OS=Mus musculus GN=Celf3 PE=2 SV=1 - [CELF3_MOUSE]	0.04	-0.42	-0.46	-0.42	-0.46	0.19	0.14	0.24	0.20	-0.13	-0.17	-0.10	-0.14
Q99KW9	T-cell immunomodulatory protein OS=Mus musculus GN=Itfg1 PE=2 SV=2 - [TIP_MOUSE]	0.10	-0.53	-0.63	-0.36	-0.46	-0.06	-0.17	-0.88	-0.99	-0.05	-0.15	0.40	0.30
G3X8P9	Aldehyde oxidase OS=Mus musculus GN=Aox1 PE=4 SV=1 - [G3X8P9_MOUSE]	0.08	-1.14	-0.64	-0.36	-0.45	-0.33	0.17	-0.26	0.24	0.09	0.60	-0.62	-0.11
Q9Z204	Heterogeneous nuclear ribonucleoproteins C1/C2 OS=Mus musculus GN=Hnrnpc PE=1 SV=1 - [HNRPC_MOUSE]	-0.12	-0.82	-0.70	-0.56	-0.44	-0.30	-0.18	0.04	0.16	0.09	0.21	0.22	0.34
E9Q264	Protein Myh15 OS=Mus musculus GN=Myh15 PE=4 SV=1 - [E9Q264_MOUSE]	-0.03	-0.38	-0.35	-0.46	-0.43	-0.01	0.01	-0.33	-0.30	-0.07	-0.04	0.04	0.07
O35226	26S proteasome non-ATPase regulatory subunit 4 OS=Mus musculus GN=Psmd4 PE=1 SV=1 - [PSMD4_MOUSE]	-0.06	-0.93	-0.88	-0.48	-0.42	0.20	0.25	0.21	0.26	0.36	0.42	0.26	0.32
Q5RKZ7-3	Isoform 3 of Molybdenum cofactor biosynthesis protein 1 OS=Mus musculus GN=Mocs1 - [MOCS1_MOUSE]	0.08	-0.59	-0.67	-0.33	-0.41	-0.88	-0.96	-1.12	-1.20	-1.03	-1.11	-0.68	-0.76
Q8BMD2-3	Isoform 3 of Zinc finger protein DZIP1 OS=Mus musculus GN=Dzip1 - [DZIP1_MOUSE]	-0.46	-0.85	-0.39	-0.87	-0.40	-0.05	0.41	-0.12	0.33	0.03	0.49	-0.86	-0.39
Q9D4C5	ELL-associated factor 1 OS=Mus musculus GN=Eaf1 PE=1 SV=2 - [EAF1_MOUSE]	-0.02	-0.57	-0.56	-0.41	-0.40	0.17	0.18	0.12	0.13	0.01	0.03	0.24	0.26
P97352	Protein S100-A13 OS=Mus musculus GN=S100a13 PE=1 SV=1 - [S10AD_MOUSE]	0.13	-0.54	-0.54	-0.24	-0.39	0.01	-0.10	0.03	-0.19	-0.16	-0.21	-0.23	-0.27
H3BKC5	U6 snRNA-associated Sm-like protein LSm5 (Fragment) OS=Mus musculus GN=Lsm5 PE=2 SV=1 - [H3BKC5_MOUSE]	0.06	-0.66	-0.72	-0.31	-0.37	-0.31	-0.38	-0.35	-0.42	-0.47	-0.53	-0.46	-0.51
K4DI63	Cellular repressor of E1A-stimulated genes 1, isoform CRA_a OS=Mus musculus GN=Creg1 PE=4 SV=1 - [K4DI63_MOUSE]	-0.01	-0.46	-0.46	-0.35	-0.34	-0.86	-0.86	-0.55	-0.55	-0.79	-0.78	-0.75	-0.74
Q3UZP4	Small VCP/p97-interacting protein OS=Mus musculus GN=Svip PE=2 SV=1 - [SVIP_MOUSE]	0.02	-0.51	-0.54	-0.31	-0.33	0.16	0.12	0.07	0.04	-0.67	-0.69	-0.06	-0.08
Q03958	Prefoldin subunit 6 OS=Mus musculus GN=Pfdn6 PE=2 SV=1 - [PFD6_MOUSE]	0.08	-0.90	-0.98	-0.24	-0.32	0.26	0.18	0.18	0.10	0.10	0.03	-0.26	-0.34

Q62422	Osteoclast-stimulating factor 1 OS=Mus musculus GN=Ostf1 PE=1 SV=2 - [OSTF1_MOUSE]	-0.04	-0.53	-0.40	-0.46	-0.32	0.10	0.13	0.30	0.29	0.22	0.21	-0.26	-0.07
Q8K4S1	1-phosphatidylinositol 4,5-bisphosphate phosphodiesterase epsilon-1 OS=Mus musculus GN=Plce1 PE=1 SV=3 - [PLCE1_MOUSE]	-0.13	-0.57	-0.44	-0.43	-0.30	-0.21	-0.08	-0.01	0.11	-0.23	-0.09	-0.50	-0.37
F6TCF9	BAG family molecular chaperone regulator 1 OS=Mus musculus GN=Bag1 PE=2 SV=1 - [F6TCF9_MOUSE]	0.12	-0.52	-0.46	-0.27	-0.30	0.09	0.02	-0.02	-0.03	-0.18	-0.24	-0.26	-0.28
P20108	Thioredoxin-dependent peroxide reductase, mitochondrial OS=Mus musculus GN=Prdx3 PE=1 SV=1 - [PRDX3_MOUSE]	0.06	-0.37	-0.46	-0.25	-0.29	-1.14	-1.24	-1.22	-1.28	-1.22	-1.29	-1.10	-1.22
Q8CAY6	Acetyl-CoA acetyltransferase, cytosolic OS=Mus musculus GN=Acat2 PE=1 SV=2 - [THIC_MOUSE]	0.02	-0.47	-0.58	-0.29	-0.29	-0.51	-0.99	-0.85	-1.09	-0.76	-1.11	-0.77	-1.09
Q61112	45 kDa calcium-binding protein OS=Mus musculus GN=Sdf4 PE=2 SV=1 - [CAB45_MOUSE]	-0.01	-0.52	-0.46	-0.35	-0.29	0.24	0.28	0.09	0.17	-0.12	-0.07	-0.30	-0.30
Q8CGC4	Protein LSM14 homolog B OS=Mus musculus GN=Lsm14b PE=2 SV=3 - [LS14B_MOUSE]	-0.04	-0.39	-0.35	-0.33	-0.29	-0.29	-0.26	-0.05	-0.02	-0.55	-0.50	-0.60	-0.56
Q8C0L8	Conserved oligomeric Golgi complex subunit 5 OS=Mus musculus GN=Cog5 PE=2 SV=3 - [COG5_MOUSE]	-0.03	-0.39	-0.36	-0.32	-0.29	-0.83	-0.81	-0.62	-0.60	-0.84	-0.81	-0.58	-0.55
P15864	Histone H1.2 OS=Mus musculus GN=Hist1h1c PE=1 SV=2 - [H12_MOUSE]	-0.04	-0.97	-0.93	-0.32	-0.29	-0.36	-0.33	-0.32	-0.29	-0.17	-0.13	-0.36	-0.32
Q8R3C6-2	Isoform 2 of Probable RNA-binding protein 19 OS=Mus musculus GN=Rbm19 - [RBM19_MOUSE]	0.00	-0.48	-0.48	-0.28	-0.28	-0.07	-0.08	-0.17	-0.17	-0.07	-0.06	-0.22	-0.22
Q3V2N7	Protein AI413582 OS=Mus musculus GN=AI413582 PE=4 SV=1 - [Q3V2N7_MOUSE]	-0.05	-0.55	-0.51	-0.31	-0.27	-0.06	-0.02	-0.11	-0.07	-0.22	-0.17	-0.14	-0.09
O70200	Allograft inflammatory factor 1 OS=Mus musculus GN=Aif1 PE=1 SV=1 - [AIF1_MOUSE]	0.00	-0.35	-0.43	-0.26	-0.27	0.40	0.39	0.33	0.28	0.17	0.20	0.19	0.19
P45591	Cofilin-2 OS=Mus musculus GN=Cfl2 PE=1 SV=1 - [COF2_MOUSE]	-0.28	-0.57	-0.37	-0.53	-0.26	-0.13	0.15	-0.02	0.17	-0.41	-0.18	-0.47	-0.10
Q8BUR4	Dedicator of cytokinesis protein 1 OS=Mus musculus GN=Dock1 PE=1 SV=3 - [DOCK1_MOUSE]	-0.04	-0.62	-0.58	-0.30	-0.26	-1.09	-1.06	-0.68	-0.65	-0.65	-0.62	-0.98	-0.94
F6VJ09	Breast carcinoma-amplified sequence 3 homolog (Fragment) OS=Mus musculus GN=Bcas3 PE=4 SV=1 - [F6VJ09_MOUSE]	0.01	-0.34	-0.35	-0.25	-0.26	0.17	0.15	0.09	0.08	-0.12	-0.13	-0.25	-0.26
Q6T264	Mastermind-like protein 1 OS=Mus musculus GN=Maml1 PE=1 SV=2 - [MAML1_MOUSE]	2.23	2.59	0.36	-1.37	-3.60	-1.77	-4.01	-1.16	-3.39	-1.75	-3.98	-1.37	-3.59
P29974	cGMP-gated cation channel alpha-1 OS=Mus musculus GN=Cnga1 PE=2 SV=2 - [CNGA1_MOUSE]	2.51	3.36	0.85	-1.39	-3.90	-1.69	-4.20	-1.37	-3.89	-1.29	-3.79	-1.97	-4.47

O70472	Transmembrane protein 131 OS=Mus musculus GN=Tmem131 PE=1 SV=2 - [TM131_MOUSE]	2.49	2.16	-0.33	-1.62	-4.11	-1.66	-4.16	-1.17	-3.67	-0.96	-3.44	-0.19	-2.68
Q9DCD6	Gamma-aminobutyric acid receptor-associated protein OS=Mus musculus GN=Gabarap PE=1 SV=2 - [GBRAP_MOUSE]	-0.29	-1.30	-1.01	-0.53	-0.24	-1.65	-1.37	-1.83	-1.54	-1.86	-1.57	-1.91	-1.62
O55208	Factor in the germline alpha OS=Mus musculus GN=Figla PE=2 SV=1 - [FIGLA_MOUSE]	0.25	-0.22	-0.47	0.10	-0.15	-1.56	-1.82	-1.32	-1.57	-1.23	-1.47	-0.69	-0.94
P61963	DDB1- and CUL4-associated factor 7 OS=Mus musculus GN=Dcaf7 PE=2 SV=1 - [DCAF7_MOUSE]	0.05	-0.26	-0.32	-0.18	-0.22	-1.55	-1.56	-1.39	-1.45	-1.21	-1.26	-1.04	-1.09
Q3UGR5	Haloacid dehalogenase-like hydrolase domain-containing protein 2 OS=Mus musculus GN=Hdhd2 PE=1 SV=2 - [HDHD2_MOUSE]	0.22	-0.23	-0.57	-0.30	-0.51	-1.48	-1.70	-1.52	-1.86	-1.51	-1.72	-1.35	-1.55
P05202	Aspartate aminotransferase, mitochondrial OS=Mus musculus GN=Got2 PE=1 SV=1 - [AATM_MOUSE]	0.01	-0.26	-0.20	-0.27	-0.27	-1.47	-1.44	-1.42	-1.40	-1.42	-1.40	-1.23	-1.23
Q9DBB8	Trans-1,2-dihydrobenzene-1,2-diol dehydrogenase OS=Mus musculus GN=Dhdh PE=2 SV=1 - [DHDH_MOUSE]	0.31	-0.12	-0.41	-0.19	-0.47	-1.46	-1.70	-1.38	-1.63	-1.37	-1.53	-0.93	-1.17
Q99KJ6	Beta-glucuronidase OS=Mus musculus GN=Gusb PE=2 SV=1 - [Q99KJ6_MOUSE]	-0.02	0.09	0.10	-0.13	-0.11	-1.42	-1.41	-1.30	-1.29	-1.15	-1.13	-1.26	-1.24
Q11136	Xaa-Pro dipeptidase OS=Mus musculus GN=Pepd PE=2 SV=3 - [PEPD_MOUSE]	0.02	-0.18	-0.19	-0.32	-0.26	-1.41	-1.54	-1.50	-1.56	-1.49	-1.55	-1.58	-1.72
P61965	WD repeat-containing protein 5 OS=Mus musculus GN=Wdr5 PE=1 SV=1 - [WDR5_MOUSE]	-0.02	-0.63	-0.50	-0.11	-0.13	-1.40	-1.42	-1.44	-1.51	-1.34	-1.41	-1.15	-1.18
P63005	Platelet-activating factor acetylhydrolase IB subunit alpha OS=Mus musculus GN=Pafah1b1 PE=1 SV=2 - [LIS1_MOUSE]	-0.01	-0.38	-0.35	-0.24	-0.24	-1.38	-1.37	-1.27	-1.25	-1.23	-1.28	-1.15	-1.18
P05201	Aspartate aminotransferase, cytoplasmic OS=Mus musculus GN=Got1 PE=1 SV=3 - [AATC_MOUSE]	-0.01	-0.14	-0.17	-0.27	-0.26	-1.38	-1.37	-1.34	-1.36	-1.29	-1.32	-1.17	-1.20
E9Q1B3	Uncharacterized protein OS=Mus musculus GN=Gm7275 PE=4 SV=1 - [E9Q1B3_MOUSE]	0.09	1.07	0.97	-0.16	-0.25	-1.34	-1.44	-1.44	-1.53	-1.35	-1.44	-1.27	-1.36
P35505	Fumarylacetoacetase OS=Mus musculus GN=Fah PE=1 SV=2 - [FAAA_MOUSE]	0.18	-0.20	-0.34	-0.34	-0.45	-1.32	-1.55	-1.18	-1.34	-1.20	-1.32	-1.12	-1.32
Q8VDK1-2	Isoform 2 of Nitrilase homolog 1 OS=Mus musculus GN=Nit1 - [NIT1_MOUSE]	0.09	-0.19	-0.38	-0.19	-0.26	-1.31	-1.53	-1.31	-1.45	-1.25	-1.38	-1.21	-1.26
Q9CQ65	S-methyl-5'-thioadenosine phosphorylase OS=Mus musculus GN=Mtap PE=2 SV=1 - [MTAP_MOUSE]	0.23	0.00	-0.26	-0.19	-0.44	-1.29	-1.52	-1.33	-1.65	-1.22	-1.44	-1.11	-1.30
D3Z0K8	Thioredoxin reductase 2, mitochondrial OS=Mus musculus GN=Txnrd2 PE=2 SV=1 - [D3Z0K8_MOUSE]	-0.04	-0.27	-0.26	-0.31	-0.29	-1.28	-1.28	-1.29	-1.49	-1.49	-1.41	-1.21	-1.22

F6W7I2	Deoxyhypusine synthase (Fragment) OS=Mus musculus GN=Dhps PE=2 SV=1 - [F6W7I2_MOUSE]	0.05	0.24	0.18	-0.63	-0.68	-1.28	-1.34	-1.29	-1.35	-0.97	-1.03	-1.51	-1.56
Q8VEB4	Group XV phospholipase A2 OS=Mus musculus GN=Pla2g15 PE=1 SV=1 - [PAG15_MOUSE]	0.05	0.03	-0.08	-0.16	-0.30	-1.28	-1.27	-1.32	-1.47	-1.08	-1.17	-1.05	-1.07
O35459	Delta(3,5)-Delta(2,4)-dienoyl-CoA isomerase, mitochondrial OS=Mus musculus GN=Ech1 PE=2 SV=1 - [ECH1_MOUSE]	0.14	-0.08	-0.29	-0.32	-0.44	-1.27	-1.30	-0.83	-0.92	-0.57	-0.71	-0.52	-0.58
Q9CQT1	Methylthioribose-1-phosphate isomerase OS=Mus musculus GN=Mri1 PE=2 SV=1 - [MTNA_MOUSE]	-0.06	-0.14	-0.05	-0.46	-0.39	-1.25	-1.26	-1.18	-1.12	-1.31	-1.19	-1.30	-1.17
P23492	Purine nucleoside phosphorylase OS=Mus musculus GN=Pnp PE=1 SV=2 - [PNPH_MOUSE]	0.13	-0.07	-0.14	-0.20	-0.32	-1.24	-1.35	-1.23	-1.18	-1.19	-1.25	-0.88	-1.02
D3YXJ2	Protein Gm9920 OS=Mus musculus GN=Gm9920 PE=2 SV=1 - [D3YXJ2_MOUSE]	-0.15	-0.34	-0.19	-0.02	0.13	-1.24	-1.10	-0.68	-0.54	-1.41	-1.25	-1.15	-1.00
H3BLA9	Sentrin-specific protease 6 (Fragment) OS=Mus musculus GN=Senp6 PE=2 SV=1 - [H3BLA9_MOUSE]	0.23	0.07	-0.16	-0.15	-0.38	-1.24	-1.47	-1.25	-1.48	-0.97	-1.19	-0.81	-1.04
Q61532	Mitogen-activated protein kinase 6 OS=Mus musculus GN=Mapk6 PE=1 SV=3 - [MK06_MOUSE]	0.50	-0.31	-0.81	0.00	-0.50	-1.24	-1.75	-1.21	-1.72	-1.12	-1.62	-0.86	-1.36
Q8QZR5	Alanine aminotransferase 1 OS=Mus musculus GN=Gpt PE=2 SV=3 - [ALAT1_MOUSE]	0.12	-0.15	-0.21	-0.32	-0.42	-1.17	-1.33	-1.09	-1.12	-1.34	-1.24	-1.07	-1.21
Q8VCN5	Cystathionine gamma-lyase OS=Mus musculus GN=Cth PE=1 SV=1 - [CGL_MOUSE]	-0.14	0.06	0.19	-0.11	0.02	-1.15	-1.10	-1.06	-1.03	-0.95	-0.90	-0.86	-0.83
Q8R3P0	Aspartoacylase OS=Mus musculus GN=Aspa PE=1 SV=2 - [ACY2_MOUSE]	-0.45	-0.25	0.19	-0.34	0.10	-1.15	-0.69	-1.08	-0.57	-1.13	-0.69	-1.00	-0.61
Q571E4	N-acetylgalactosamine-6-sulfatase OS=Mus musculus GN=Galns PE=2 SV=2 - [GALNS_MOUSE]	-0.02	0.16	0.18	-0.49	-0.47	-1.14	-1.13	-1.15	-1.14	-1.26	-1.24	-1.08	-1.06
Q6NT99	Dual specificity protein phosphatase 23 OS=Mus musculus GN=Dusp23 PE=2 SV=1 - [DUS23_MOUSE]	0.15	0.07	-0.04	-0.08	-0.30	-1.14	-1.31	-1.14	-1.23	-1.11	-1.19	-0.56	-0.72
Q9JMH6-2	Isoform 2 of Thioredoxin reductase 1, cytoplasmic OS=Mus musculus GN=Txnrd1 - [TRXR1_MOUSE]	0.17	-0.07	-0.33	-0.11	-0.28	-1.14	-1.28	-0.97	-1.07	-0.99	-1.07	-0.81	-0.88
Q9CSP9-4	Isoform 4 of Tetratricopeptide repeat protein 14 OS=Mus musculus GN=Ttc14 - [TTC14_MOUSE]	-0.23	-0.08	0.15	-0.55	-0.32	-1.14	-0.91	-0.75	-0.52	-1.22	-0.99	-0.77	-0.54
P58058	NAD kinase OS=Mus musculus GN=Nadk PE=1 SV=2 - [NADK_MOUSE]	-0.21	0.25	0.45	-0.08	0.13	-1.13	-0.93	-1.11	-0.91	-1.19	-0.98	-1.03	-0.82
Q5M8N0	CB1 cannabinoid receptor-interacting protein 1 OS=Mus musculus GN=Cnrip1 PE=1 SV=1 - [CNRP1_MOUSE]	-0.07	-0.46	-0.33	-0.40	-0.23	-1.12	-1.04	-1.20	-1.09	-1.06	-1.00	-1.20	-1.07
Q9WV54	Acid ceramidase OS=Mus musculus GN=Asah1 PE=1 SV=1 - [ASAH1_MOUSE]	0.00	-0.19	-0.14	-0.18	-0.17	-1.11	-1.01	-0.90	-1.02	-0.92	-0.83	-0.81	-0.82

Q8CFD4	Sorting nexin-8 OS=Mus musculus GN=Snx8 PE=2 SV=1 - [SNX8_MOUSE]	0.04	-0.31	-0.35	-0.06	-0.10	-1.11	-1.15	-1.14	-1.19	-1.07	-1.10	-0.61	-0.64
P50428	Arylsulfatase A OS=Mus musculus GN=Arsa PE=2 SV=2 - [ARSA_MOUSE]	0.05	-0.34	-0.37	-0.18	-0.27	-1.11	-1.18	-1.02	-1.00	-1.04	-1.13	-0.78	-0.87
Q61233	Plastin-2 OS=Mus musculus GN=Lcp1 PE=1 SV=4 - [PLSL_MOUSE]	0.09	-0.04	-0.09	-0.05	-0.19	-1.10	-1.12	-1.07	-1.09	-1.01	-0.97	-1.01	-1.00
Q9DCF9-2	Isoform 2 of Translocon-associated protein subunit gamma OS=Mus musculus GN=Ssr3 - [SSRG_MOUSE]	-0.29	0.10	0.39	0.07	0.36	-1.10	-0.81	-0.96	-0.68	-0.82	-0.52	-0.72	-0.43
Q99KN2	Probable cytosolic iron-sulfur protein assembly protein CIAO1 OS=Mus musculus GN=Ciao1 PE=2 SV=1 - [CIAO1_MOUSE]	-0.09	-0.03	0.06	-0.20	-0.17	-1.10	-0.93	-1.09	-0.90	-1.05	-0.83	-0.93	-0.78
Q9CR39	WD repeat domain phosphoinositide-interacting protein 3 OS=Mus musculus GN=Wdr45b PE=2 SV=2 - [WIPI3_MOUSE]	0.17	-0.24	-0.39	-0.16	-0.35	-1.09	-1.36	-1.19	-1.39	-1.14	-1.36	-0.93	-1.13
Q8C7K6	Prenylcysteine oxidase-like OS=Mus musculus GN=Pcyox1l PE=2 SV=1 - [PCYXL_MOUSE]	0.12	0.08	-0.12	-0.20	-0.30	-1.09	-1.23	-1.21	-1.42	-0.82	-1.04	-0.78	-0.89
P40142	Transketolase OS=Mus musculus GN=Tkt PE=1 SV=1 - [TKT_MOUSE]	0.05	-0.32	-0.38	-0.27	-0.30	-1.09	-1.14	-1.00	-1.06	-1.00	-1.05	-0.90	-1.03
Q9Z2W0	Aspartyl aminopeptidase OS=Mus musculus GN=Dnpep PE=2 SV=2 - [DNPEP_MOUSE]	0.13	-0.27	-0.42	-0.21	-0.36	-1.09	-1.24	-1.06	-1.19	-1.01	-1.20	-0.91	-1.04
Q8BM88	Cathepsin O OS=Mus musculus GN=Ctso PE=2 SV=1 - [CATO_MOUSE]	0.02	0.44	0.41	-0.33	-0.36	-1.08	-1.12	-0.98	-1.01	-1.14	-1.17	-1.11	-1.13
Q9Z1Z2	Serine-threonine kinase receptor-associated protein OS=Mus musculus GN=Strap PE=1 SV=2 - [STRAP_MOUSE]	-0.04	-0.36	-0.21	-0.17	-0.12	-1.08	-0.99	-1.07	-0.98	-1.06	-1.06	-0.98	-0.98
Q3UWE6	MCG14935, isoform CRA_a OS=Mus musculus GN=Wdr20a PE=2 SV=1 - [Q3UWE6_MOUSE]	-0.02	0.01	-0.08	-0.17	-0.17	-1.08	-1.07	-1.06	-1.15	-1.14	-1.19	-0.93	-0.99
Q9CPU0	Lactoylglutathione lyase OS=Mus musculus GN=Glo1 PE=1 SV=3 - [LGUL_MOUSE]	-0.03	-0.50	-0.42	-0.23	-0.30	-1.08	-1.01	-1.02	-1.01	-1.00	-1.05	-0.98	-0.97
P51174	Long-chain specific acyl-CoA dehydrogenase, mitochondrial OS=Mus musculus GN=Acadl PE=2 SV=2 - [ACADL_MOUSE]	-0.03	-0.26	-0.16	-0.15	-0.12	-1.05	-1.00	-1.07	-0.96	-1.01	-1.02	-0.89	-0.86
Q924B0	Inositol (Myo)-1(Or 4)-monophosphatase 1 OS=Mus musculus GN=Impa1 PE=2 SV=1 - [Q924B0_MOUSE]	0.27	-0.18	-0.42	-0.01	-0.30	-1.04	-1.31	-0.94	-1.30	-0.82	-1.00	-0.65	-1.02
P63087-2	Isoform Gamma-2 of Serine/threonine-protein phosphatase PP1-gamma catalytic subunit OS=Mus musculus GN=Ppp1cc - [PP1G_MOUSE]	-0.05	-0.09	-0.03	-0.16	-0.06	-1.04	-0.83	-0.78	-0.67	-0.89	-0.71	-0.65	-0.55
P03921	NADH-ubiquinone oxidoreductase chain 5 OS=Mus musculus GN=Mtnd5 PE=3 SV=2 - [NU5M_MOUSE]	0.18	0.35	0.17	0.37	0.19	-1.04	-1.23	-0.80	-0.98	-0.43	-0.61	-0.15	-0.33
Q60571	Corticotropin-releasing factor-binding protein OS=Mus musculus GN=Crhbp PE=2 SV=1 - [CRHBP_MOUSE]	0.42	0.15	-0.28	-0.07	-0.49	-1.03	-1.46	-1.05	-1.47	-1.23	-1.65	-0.61	-1.03

Q8BWR2	PITH domain-containing protein 1 OS=Mus musculus GN=Pithd1 PE=2 SV=1 - [PITH1_MOUSE]	0.17	-0.21	-0.44	-0.23	-0.47	-1.03	-1.28	-0.80	-1.20	-0.87	-1.11	-0.57	-0.91
Q8JZR2	Adapter molecule crk OS=Mus musculus GN=Crk PE=2 SV=1 - [Q8JZR2_MOUSE]	-0.14	-0.29	-0.12	-0.39	-0.08	-0.99	-0.92	-0.86	-0.78	-0.99	-0.91	-0.85	-0.71
Q80X81	Acetyl-Coenzyme A acetyltransferase 3 OS=Mus musculus GN=Acat3 PE=2 SV=1 - [Q80X81_MOUSE]	0.25	0.03	-0.23	-0.30	-0.53	-0.98	-0.78	-0.85	-1.04	-1.04	-1.01	-0.83	-1.13
Q61474-2	Isoform 2 of RNA-binding protein Musashi homolog 1 OS=Mus musculus GN=Msi1 - [MSI1H_MOUSE]	0.22	-0.19	-0.41	-0.22	-0.44	-0.98	-1.21	-0.76	-0.98	-0.53	-0.75	-0.48	-0.70
Q3V1L4	Cytosolic purine 5'-nucleotidase OS=Mus musculus GN=Nt5c2 PE=1 SV=2 - [5NTC_MOUSE]	0.01	-0.19	-0.24	-0.23	-0.18	-0.98	-0.93	-1.00	-1.18	-0.97	-0.92	-0.78	-0.85
E0CX20	Protein BUD31 homolog OS=Mus musculus GN=Bud31 PE=4 SV=1 - [E0CX20_MOUSE]	-0.24	-0.64	-0.37	-0.26	0.03	-0.97	-0.74	-0.90	-0.64	-1.14	-0.82	-1.07	-0.74
Q8CJG1	Protein argonaute-1 OS=Mus musculus GN=Ago1 PE=1 SV=2 - [AGO1_MOUSE]	0.04	0.13	0.08	-0.14	-0.13	-0.96	-1.09	-0.73	-0.86	-0.86	-0.94	-0.41	-0.43
O08966	Solute carrier family 22 member 1 OS=Mus musculus GN=Slc22a1 PE=2 SV=2 - [S22A1_MOUSE]	0.36	0.07	-0.30	-0.45	-0.81	-0.96	-1.33	-0.67	-1.03	-0.91	-1.27	-0.56	-0.92
P62141	Serine/threonine-protein phosphatase PP1-beta catalytic subunit OS=Mus musculus GN=Ppp1cb PE=1 SV=3 - [PP1B_MOUSE]	-0.03	-0.14	-0.21	-0.18	-0.14	-0.96	-0.88	-0.81	-0.75	-0.84	-0.90	-0.77	-0.65
Q8CBC8	Branched-chain-amino-acid aminotransferase OS=Mus musculus GN=Bcat1 PE=2 SV=1 - [Q8CBC8_MOUSE]	0.04	-0.09	-0.19	-0.23	-0.25	-0.95	-0.97	-0.91	-0.93	-0.94	-0.87	-0.79	-0.89
Q3TTY0-3	Isoform 3 of Phospholipase B1, membrane-associated OS=Mus musculus GN=Plb1 - [PLB1_MOUSE]	0.51	-0.22	-0.73	-0.04	-0.55	-0.94	-1.46	-1.39	-1.91	-1.34	-1.85	-0.37	-0.88
D3YWZ5	Transporter (Fragment) OS=Mus musculus GN=Slc6a13 PE=2 SV=1 - [D3YWZ5_MOUSE]	0.08	0.07	-0.02	-0.02	-0.10	-0.94	-1.03	-0.42	-0.51	-0.28	-0.36	-0.56	-0.64
Q8BTY1	Kynurenine--oxoglutarate transaminase 1 OS=Mus musculus GN=Ccbl1 PE=2 SV=1 - [KAT1_MOUSE]	0.16	-0.23	-0.40	-0.35	-0.52	-0.94	-1.21	-0.94	-1.12	-0.84	-1.06	-0.70	-0.93
Q99LL5	Periodic tryptophan protein 1 homolog OS=Mus musculus GN=Pwp1 PE=1 SV=1 - [PWP1_MOUSE]	0.14	-0.06	-0.20	-0.13	-0.28	-0.93	-1.08	-0.78	-0.92	-1.19	-1.34	-0.91	-1.05
G5E8Q8	MCG115189 OS=Mus musculus GN=Gpr116 PE=4 SV=1 - [G5E8Q8_MOUSE]	-0.36	0.02	0.38	-0.22	0.14	-0.93	-0.58	-1.23	-0.87	-0.63	-0.27	-0.33	0.03
Q9QXP7	Complement C1q tumor necrosis factor-related protein 1 OS=Mus musculus GN=C1qtnf1 PE=2 SV=1 - [C1QT1_MOUSE]	0.32	0.22	-0.11	-0.12	-0.44	-0.92	-1.25	-1.01	-1.34	-1.01	-1.33	-0.94	-1.26
O89106	Bis(5'-adenosyl)-triphosphatase OS=Mus musculus GN=Fhit PE=2 SV=3 - [FHIT_MOUSE]	0.05	-0.11	-0.25	-0.36	-0.46	-0.92	-1.14	-1.16	-1.25	-1.40	-1.47	-1.11	-1.15

P07724	Serum albumin OS=Mus musculus GN=Alb PE=1 SV=3 - [ALBU_MOUSE]	-0.09	-0.31	-0.21	-0.79	-0.73	-0.92	-0.83	-0.69	-0.62	-0.59	-0.50	-0.94	-0.84
Q8CFI5	Probable proline--tRNA ligase, mitochondrial OS=Mus musculus GN=Pars2 PE=2 SV=2 - [SYPM_MOUSE]	0.09	-0.31	-0.28	-0.18	-0.30	-0.92	-0.97	-1.02	-1.01	-0.90	-0.97	-0.75	-0.80
P26043	Radixin OS=Mus musculus GN=Rdx PE=1 SV=3 - [RADI_MOUSE]	-0.29	-0.51	-0.12	-0.23	0.00	-0.92	-0.57	-0.87	-0.69	-1.04	-0.73	-0.91	-0.52
Q99KR3	Beta-lactamase-like protein 2 OS=Mus musculus GN=Lactb2 PE=1 SV=1 - [LACB2_MOUSE]	0.10	-0.05	-0.26	-0.21	-0.37	-0.92	-1.03	-0.82	-1.00	-0.78	-1.01	-0.46	-0.84
P01863	Ig gamma-2A chain C region, A allele OS=Mus musculus GN=Ighg PE=1 SV=1 - [GCAA_MOUSE]	0.23	0.16	-0.07	-0.19	-0.42	-0.92	-1.15	-0.60	-0.83	-0.10	-0.33	-0.14	-0.36
P47857	6-phosphofructokinase, muscle type OS=Mus musculus GN=Pfkm PE=1 SV=3 - [K6PF_MOUSE]	0.04	-0.14	-0.18	-0.08	-0.12	-0.91	-0.98	-0.98	-1.02	-0.92	-0.94	-0.83	-0.89
Q8BGF3	WD repeat-containing protein 92 OS=Mus musculus GN=Wdr92 PE=2 SV=1 - [WDR92_MOUSE]	-0.09	0.00	0.08	-0.35	-0.26	-0.91	-0.83	-0.68	-0.59	-0.78	-0.69	-0.51	-0.42
Q8VEB6	Zinc phosphodiesterase ELAC protein 1 OS=Mus musculus GN=Elac1 PE=2 SV=1 - [RNZ1_MOUSE]	0.19	0.06	-0.24	-0.11	-0.32	-0.91	-1.02	-1.00	-1.31	-0.85	-1.09	-0.63	-0.83
Q8CC86	Nicotinate phosphoribosyltransferase OS=Mus musculus GN=Naprt1 PE=2 SV=1 - [PNCB_MOUSE]	-0.19	-0.10	0.09	-0.50	-0.32	-0.91	-0.73	-0.89	-0.70	-0.65	-0.46	-0.89	-0.70
Q9D614	Syntaxin 17, isoform CRA_b OS=Mus musculus GN=Stx17 PE=2 SV=1 - [Q9D614_MOUSE]	-0.41	-0.65	-0.24	-1.17	-0.77	-0.91	-0.51	-0.82	-0.42	-0.86	-0.45	-1.53	-1.11
Q9D240	Pleckstrin homology domain-containing family J member 1 OS=Mus musculus GN=Plekhj1 PE=2 SV=1 - [PKHJ1_MOUSE]	0.09	-0.31	-0.41	-0.33	-0.42	-0.91	-1.01	-0.80	-0.90	-0.86	-0.95	-0.86	-0.94
Q9CWQ0	Diphthine synthase OS=Mus musculus GN=Dph5 PE=2 SV=2 - [DPH5_MOUSE]	0.38	0.16	-0.31	-0.09	-0.62	-0.91	-1.50	-0.86	-1.46	-0.80	-1.39	-0.58	-1.16
O08807	Peroxiredoxin-4 OS=Mus musculus GN=Prdx4 PE=1 SV=1 - [PRDX4_MOUSE]	0.08	-0.45	-0.48	-0.15	-0.22	-0.91	-0.99	-0.88	-0.88	-0.95	-0.94	-0.89	-0.88
Q8C605	6-phosphofructokinase OS=Mus musculus GN=Pfkp PE=2 SV=1 - [Q8C605_MOUSE]	0.01	-0.12	-0.10	-0.08	-0.09	-0.90	-0.91	-0.94	-0.93	-0.92	-0.93	-0.82	-0.84
Q8CCH2	NHL repeat-containing protein 3 OS=Mus musculus GN=Nhlrc3 PE=2 SV=1 - [NHLC3_MOUSE]	0.08	-0.09	-0.16	-0.19	-0.27	-0.90	-0.99	-0.70	-0.78	-0.16	-0.24	-0.33	-0.40
Q9CPY7-2	Isoform 2 of Cytosol aminopeptidase OS=Mus musculus GN=Lap3 - [AMPL_MOUSE]	0.15	-0.20	-0.28	-0.24	-0.35	-0.90	-0.98	-0.93	-0.97	-0.80	-0.89	-0.80	-0.89
Q9WUI0	Homeobox protein MIXL1 OS=Mus musculus GN=Mixl1 PE=1 SV=1 - [MIXL1_MOUSE]	0.81	1.25	0.43	-0.19	-1.00	-0.89	-1.72	-0.74	-1.56	-0.08	-0.89	-0.22	-1.03
Q64010	Adapter molecule crk OS=Mus musculus GN=Crk PE=1 SV=1 - [CRK_MOUSE]	-0.26	-0.32	-0.15	-0.23	0.03	-0.89	-0.78	-0.84	-0.59	-1.08	-0.84	-0.56	-0.38
Q3UR97	Protein Snx21 OS=Mus musculus GN=Snx21 PE=2 SV=1 - [Q3UR97_MOUSE]	0.00	-0.28	-0.28	-0.01	-0.02	-0.89	-0.89	-1.22	-1.22	-1.11	-1.11	-1.09	-1.09

P60487	Pyridoxal phosphate phosphatase OS=Mus musculus GN=Pdxp PE=1 SV=1 - [PLPP_MOUSE]	0.13	-0.03	-0.22	-0.13	-0.26	-0.89	-1.03	-0.80	-0.94	-0.75	-0.86	-0.66	-0.72
Q6P2B2	Casein kinase I isoform gamma-1 OS=Mus musculus GN=Csnk1g1 PE=2 SV=1 - [Q6P2B2_MOUSE]	-0.26	-0.24	0.01	-0.04	0.22	-0.88	-0.63	-0.62	-0.37	-0.52	-0.26	-0.51	-0.25
Q91VR7	Microtubule-associated proteins 1A/1B light chain 3A OS=Mus musculus GN=Map1lc3a PE=1 SV=1 - [MLP3A_MOUSE]	-0.20	-0.93	-0.82	-0.31	-0.10	-0.88	-0.67	-0.81	-0.60	-1.00	-0.77	-0.89	-0.62
Q9CX80	Cytoglobin OS=Mus musculus GN=Cygb PE=2 SV=1 - [CYGB_MOUSE]	0.28	-0.28	-0.33	-0.08	-0.45	-0.87	-1.29	-0.92	-1.21	-0.76	-1.02	-0.77	-0.99
P21956-2	Isoform 2 of Lactadherin OS=Mus musculus GN=Mfge8 - [MFGM_MOUSE]	0.21	-0.19	-0.40	0.09	-0.13	-0.87	-1.09	-0.81	-1.03	-0.82	-1.03	-0.64	-0.85
Q9JI46	Diphosphoinositol polyphosphate phosphohydrolase 1 OS=Mus musculus GN=Nudt3 PE=1 SV=1 - [NUDT3_MOUSE]	-0.22	-0.24	-0.11	-0.25	-0.06	-0.86	-0.74	-0.89	-0.65	-0.88	-0.71	-0.85	-0.72
Q8CDI6	Coiled-coil domain-containing protein 158 OS=Mus musculus GN=Ccdc158 PE=2 SV=1 - [CD158_MOUSE]	1.39	1.37	-0.03	-0.40	-1.79	-0.86	-2.26	-0.92	-2.32	-0.88	-2.27	-0.53	-1.92
P62137	Serine/threonine-protein phosphatase PP1-alpha catalytic subunit OS=Mus musculus GN=Ppp1ca PE=1 SV=1 - [PP1A_MOUSE]	0.04	0.13	0.10	-0.13	-0.03	-0.85	-0.71	-0.55	-0.53	-0.75	-0.58	-0.61	-0.44
Q8R0N6-2	Isoform 2 of Hydroxyacid-oxoacid transhydrogenase, mitochondrial OS=Mus musculus GN=Adhfe1 - [HOT_MOUSE]	0.02	-0.33	-0.25	-0.18	-0.27	-0.85	-0.87	-0.89	-0.93	-1.19	-1.20	-0.94	-0.94
Q9D0I6	WD repeat, SAM and U-box domain-containing protein 1 OS=Mus musculus GN=Wdsub1 PE=2 SV=1 - [WSDU1_MOUSE]	-0.14	-0.32	-0.18	-0.18	-0.04	-0.84	-0.71	-0.61	-0.47	-0.56	-0.41	-0.50	-0.35
Q8BTR5	Dual specificity phosphatase 28 OS=Mus musculus GN=Dusp28 PE=1 SV=1 - [DUS28_MOUSE]	-0.03	0.11	0.13	0.02	0.04	-0.84	-0.82	-0.89	-0.87	-0.91	-0.88	-0.95	-0.92
D3YZU6	Hydroxyacylglutathione hydrolase-like protein OS=Mus musculus GN=Haghl PE=4 SV=1 - [D3YZU6_MOUSE]	-0.03	-0.15	-0.16	-0.31	-0.22	-0.84	-1.04	-1.24	-1.29	-1.17	-1.25	-1.11	-0.93
Q9R111	Guanine deaminase OS=Mus musculus GN=Gda PE=1 SV=1 - [GUAD_MOUSE]	0.03	-0.04	-0.14	-0.18	-0.23	-0.84	-0.91	-0.88	-0.92	-0.89	-0.93	-0.68	-0.85
Q9CQM9	Glutaredoxin-3 OS=Mus musculus GN=Glrx3 PE=1 SV=1 - [GLRX3_MOUSE]	0.02	-0.38	-0.39	-0.20	-0.23	-0.84	-0.85	-0.71	-0.73	-0.82	-0.73	-0.73	-0.67
P42125	Enoyl-CoA delta isomerase 1, mitochondrial OS=Mus musculus GN=Eci1 PE=2 SV=2 - [ECI1_MOUSE]	0.16	-0.16	-0.42	-0.06	-0.25	-0.83	-1.21	-0.87	-1.19	-0.84	-1.08	-0.79	-1.15
Q91YA2	Serine/threonine-protein kinase H1 OS=Mus musculus GN=Pskh1 PE=2 SV=3 - [KPSH1_MOUSE]	0.00	-0.80	-0.80	-0.17	-0.17	-0.83	-0.84	-0.69	-0.70	-0.85	-0.85	-1.03	-1.02
Q8BHS3	Pre-mRNA-splicing factor RBM22 OS=Mus musculus GN=Rbm22 PE=2 SV=1 - [RBM22_MOUSE]	-0.23	-0.36	-0.31	-0.26	-0.02	-0.83	-0.69	-0.83	-0.69	-0.73	-0.51	-0.98	-0.77

Q3U6C5	Voltage-gated potassium channel subunit beta-1 OS=Mus musculus GN=Kcnab1 PE=2 SV=1 - [Q3U6C5_MOUSE]	0.03	-0.03	-0.07	-0.23	-0.26	-0.82	-0.78	-0.99	-0.94	-0.36	-0.31	-0.77	-0.72
Q9R1P3	Proteasome subunit beta type-2 OS=Mus musculus GN=Psmb2 PE=1 SV=1 - [PSB2_MOUSE]	-0.10	0.37	0.34	0.03	0.15	-0.82	-0.66	-1.01	-0.94	-0.82	-0.78	-0.51	-0.37
P60521	Gamma-aminobutyric acid receptor-associated protein-like 2 OS=Mus musculus GN=Gabarapl2 PE=1 SV=1 - [GBRL2_MOUSE]	-0.16	-0.43	-0.31	-0.35	-0.20	-0.81	-0.58	-0.65	-0.67	-0.79	-0.77	-0.71	-0.54
Q99K85	Phosphoserine aminotransferase OS=Mus musculus GN=Psat1 PE=1 SV=1 - [SERC_MOUSE]	-0.04	-0.29	-0.24	-0.09	-0.08	-0.80	-0.81	-0.87	-0.84	-0.76	-0.73	-0.71	-0.68
P24547	Inosine-5'-monophosphate dehydrogenase 2 OS=Mus musculus GN=Impdh2 PE=1 SV=2 - [IMDH2_MOUSE]	0.13	-0.01	-0.18	0.05	-0.10	-0.80	-0.97	-0.96	-1.07	-0.77	-0.93	-0.68	-0.89
Q9Z2Y8	Proline synthase co-transcribed bacterial homolog protein OS=Mus musculus GN=Prosc PE=1 SV=1 - [PROSC_MOUSE]	-0.15	-0.22	-0.07	-0.24	-0.09	-0.80	-0.67	-0.81	-0.58	-0.89	-0.75	-0.67	-0.57
Q6WVG3	BTB/POZ domain-containing protein KCTD12 OS=Mus musculus GN=Kctd12 PE=1 SV=1 - [KCD12_MOUSE]	0.12	-0.03	-0.03	-0.15	-0.25	-0.80	-0.85	-0.74	-0.98	-0.73	-0.79	-0.65	-0.71
Q32MT6	Prestin OS=Mus musculus GN=Slc26a5 PE=2 SV=1 - [Q32MT6_MOUSE]	0.10	-0.01	-0.11	0.40	0.30	-0.78	-0.89	-0.52	-0.63	0.12	0.01	-0.98	-1.08
Q61884	Meiosis-specific nuclear structural protein 1 OS=Mus musculus GN=Mns1 PE=1 SV=1 - [MNS1_MOUSE]	0.35	-0.36	-0.71	-0.07	-0.42	-0.78	-1.13	-0.42	-0.77	-0.44	-0.79	-0.46	-0.81
Q9ER35	Fructosamine-3-kinase OS=Mus musculus GN=Fn3k PE=2 SV=1 - [FN3K_MOUSE]	-0.03	0.11	0.07	-0.07	-0.09	-0.77	-0.53	-0.69	-0.61	-0.84	-0.64	-0.72	-0.57
Q9DBJ1	Phosphoglycerate mutase 1 OS=Mus musculus GN=Pgam1 PE=1 SV=3 - [PGAM1_MOUSE]	-0.11	-0.26	-0.15	-0.21	-0.09	-0.77	-0.67	-0.80	-0.70	-0.78	-0.67	-0.83	-0.70
P00405	Cytochrome c oxidase subunit 2 OS=Mus musculus GN=Mtco2 PE=1 SV=1 - [COX2_MOUSE]	-0.10	-0.17	0.23	0.16	0.29	-0.77	-0.54	-0.72	-0.57	-0.45	-0.35	-0.46	-0.33
Q9D0M5	Dynein light chain 2, cytoplasmic OS=Mus musculus GN=Dynll2 PE=1 SV=1 - [DYL2_MOUSE]	-0.11	-0.30	-0.23	-0.21	-0.12	-0.77	-0.64	-0.85	-0.58	-0.88	-0.74	-1.12	-0.70
Q8BWT1	3-ketoacyl-CoA thiolase, mitochondrial OS=Mus musculus GN=Acaa2 PE=1 SV=3 - [THIM_MOUSE]	0.15	-0.13	-0.27	-0.16	-0.28	-0.76	-1.06	-0.92	-0.99	-0.76	-0.96	-0.66	-0.80
G3UYJ3	Solute carrier family 25 member 51 (Fragment) OS=Mus musculus GN=Slc25a51 PE=2 SV=1 - [G3UYJ3_MOUSE]	-0.19	-0.06	0.13	0.02	0.20	-0.76	-0.58	-1.05	-0.87	-0.74	-0.55	-0.45	-0.26
H3BK32	Sentrin-specific protease 6 (Fragment) OS=Mus musculus GN=Senp6 PE=2 SV=1 - [H3BK32_MOUSE]	2.39	2.83	0.44	-0.39	-2.78	-0.76	-3.15	-0.67	-3.06	-0.82	-3.20	-0.56	-2.94

D3YTP0	Metalloreductase STEAP3 (Fragment) OS=Mus musculus GN=Steap3 PE=2 SV=1 - [D3YTP0_MOUSE]	-0.07	0.06	0.13	-0.18	-0.11	-0.75	-0.69	-0.57	-0.50	-0.65	-0.58	-0.69	-0.62
Q71RI9-2	Isoform 2 of Kynurenine--oxoglutarate transaminase 3 OS=Mus musculus GN=Ccbl2 - [KAT3_MOUSE]	-0.04	0.05	0.09	-0.14	-0.11	-0.75	-0.72	-0.63	-0.59	-0.65	-0.61	-0.51	-0.47
Q8BFQ4	WD repeat-containing protein 82 OS=Mus musculus GN=Wdr82 PE=1 SV=1 - [WDR82_MOUSE]	0.08	-0.07	-0.15	-0.09	-0.17	-0.75	-0.84	-0.38	-0.47	-0.46	-0.54	-1.01	-1.09
P50247	Adenosylhomocysteinase OS=Mus musculus GN=Ahcy PE=1 SV=3 - [SAHH_MOUSE]	-0.11	-0.22	0.02	-0.03	0.09	-0.74	-0.52	-0.85	-0.74	-0.71	-0.60	-0.74	-0.61
O88325	Alpha-N-acetylglucosaminidase OS=Mus musculus GN=Naglu PE=2 SV=1 - [O88325_MOUSE]	-0.10	-0.30	-0.20	-0.24	-0.14	-0.74	-0.65	-0.83	-0.74	-0.77	-0.67	-0.90	-0.80
A3KMP2-2	Isoform 2 of Tetratricopeptide repeat protein 38 OS=Mus musculus GN=Ttc38 - [TTC38_MOUSE]	-0.02	0.19	0.21	-0.30	-0.29	-0.74	-0.73	-0.87	-0.86	-0.69	-0.67	-0.49	-0.47
P03899	NADH-ubiquinone oxidoreductase chain 3 OS=Mus musculus GN=Mtnd3 PE=3 SV=3 - [NU3M_MOUSE]	0.15	0.46	0.31	0.57	0.42	-0.74	-0.89	-0.68	-0.83	-0.38	-0.53	0.15	0.01
Q8R1G2	Carboxymethylenebutenolidase homolog OS=Mus musculus GN=Cmbl PE=2 SV=1 - [CMBL_MOUSE]	0.35	-0.20	-0.56	-0.16	-0.47	-0.73	-1.07	-0.68	-0.97	-0.59	-0.89	-0.47	-0.82
Q9D8Z6	Autophagy-related protein 101 OS=Mus musculus GN=Atg101 PE=2 SV=1 - [ATGA1_MOUSE]	0.04	0.40	0.36	-0.14	-0.18	-0.73	-0.78	-0.63	-0.68	-0.51	-0.54	-0.65	-0.69
Q8CJG0	Protein argonaute-2 OS=Mus musculus GN=Ago2 PE=1 SV=3 - [AGO2_MOUSE]	-0.15	0.04	0.13	-0.08	0.10	-0.73	-0.63	-0.82	-0.68	-0.83	-0.53	-0.69	-0.44
Q9CR62	Mitochondrial 2-oxoglutarate/malate carrier protein OS=Mus musculus GN=Slc25a11 PE=1 SV=3 - [M2OM_MOUSE]	0.16	0.40	0.12	0.36	0.20	-0.73	-0.83	-0.73	-0.97	-0.37	-0.60	-0.15	-0.40
Q9R0D8	WD repeat-containing protein 54 OS=Mus musculus GN=Wdr54 PE=2 SV=1 - [WDR54_MOUSE]	0.13	-0.04	-0.07	-0.28	-0.36	-0.72	-0.87	-0.58	-0.80	-0.61	-0.75	-0.35	-0.48
Q8BRQ9	Sideroflexin-5 OS=Mus musculus GN=Sfxn5 PE=2 SV=1 - [Q8BRQ9_MOUSE]	0.01	0.32	0.23	0.31	0.33	-0.72	-0.41	-0.44	-0.53	-0.29	-0.34	-0.48	-0.40
D3Z1E2	Pleckstrin homology domain-containing family B member 1 (Fragment) OS=Mus musculus GN=Plekhb1 PE=2 SV=1 - [D3Z1E2_MOUSE]	0.06	0.27	0.20	-0.21	-0.29	-0.72	-0.78	-0.61	-0.77	-0.60	-0.72	-0.49	-0.57
Q9QWW1-2	Isoform 2 of Homer protein homolog 2 OS=Mus musculus GN=Homer2 - [HOME2_MOUSE]	0.17	-0.22	-0.36	-0.11	-0.25	-0.72	-0.91	-0.67	-0.80	-0.93	-1.18	-0.73	-0.92
Q8VHK5	Membrane protein MLC1 OS=Mus musculus GN=Mlc1 PE=2 SV=1 - [MLC1_MOUSE]	-0.12	0.16	0.27	-0.11	0.01	-0.72	-0.60	-0.55	-0.43	-0.39	-0.26	-0.47	-0.35
Q9CQQ7	ATP synthase subunit b, mitochondrial OS=Mus musculus GN=Atp5f1 PE=1 SV=1 - [AT5F1_MOUSE]	-0.05	0.05	0.10	0.18	0.27	-0.71	-0.67	-0.81	-0.72	-0.56	-0.48	-0.45	-0.45

O70435	Proteasome subunit alpha type-3 OS=Mus musculus GN=Psma3 PE=1 SV=3 - [PSA3_MOUSE]	-0.21	-0.12	0.02	0.00	0.18	-0.71	-0.41	-0.72	-0.45	-0.73	-0.57	-0.41	-0.27
Q4FJZ2	Importin subunit alpha OS=Mus musculus GN=Kpna6 PE=2 SV=1 - [Q4FJZ2_MOUSE]	0.01	-0.26	-0.26	-0.16	-0.27	-0.71	-0.61	-0.51	-0.52	-0.53	-0.56	-0.36	-0.34
Q5SWT3	Solute carrier family 25 member 35 OS=Mus musculus GN=Slc25a35 PE=2 SV=2 - [S2535_MOUSE]	-0.01	0.44	0.45	0.18	0.19	-0.71	-0.71	-0.74	-0.73	-0.49	-0.48	-0.16	-0.15
E0CY96	tRNA-splicing endonuclease subunit Sen15 OS=Mus musculus GN=Tsen15 PE=2 SV=1 - [E0CY96_MOUSE]	-0.28	-0.11	0.16	-0.34	-0.06	-0.70	-0.43	-0.66	-0.38	-0.46	-0.18	-0.60	-0.32
Q9DAR7	m7GpppX diphosphatase OS=Mus musculus GN=Dcps PE=1 SV=1 - [DCPS_MOUSE]	-0.17	-0.15	0.04	-0.31	0.09	-0.70	-0.51	-0.77	-0.54	-0.87	-0.51	-0.92	-0.68
Q9CZP5	Mitochondrial chaperone BCS1 OS=Mus musculus GN=Bcs1l PE=1 SV=1 - [BCS1_MOUSE]	-0.16	-0.03	0.23	0.15	0.32	-0.70	-0.54	-0.72	-0.56	-0.21	-0.05	-0.32	0.05
P11404	Fatty acid-binding protein, heart OS=Mus musculus GN=Fabp3 PE=1 SV=5 - [FABPH_MOUSE]	-0.06	-0.15	0.01	-0.18	-0.09	-0.70	-0.53	-0.57	-0.43	-0.81	-0.71	-0.71	-0.56
Q4VBE7	BTB/POZ domain-containing protein KCTD2 OS=Mus musculus GN=Kctd2 PE=2 SV=1 - [Q4VBE7_MOUSE]	0.20	0.38	0.18	0.18	-0.02	-0.70	-0.90	-0.43	-0.64	-0.68	-0.88	-0.58	-0.78
Q8VC57	BTB/POZ domain-containing protein KCTD5 OS=Mus musculus GN=Kctd5 PE=2 SV=1 - [KCTD5_MOUSE]	0.11	-0.04	-0.15	-0.08	-0.19	-0.69	-0.81	-0.59	-0.71	-0.54	-0.64	-0.33	-0.44
O55234	Proteasome subunit beta type-5 OS=Mus musculus GN=Psmb5 PE=1 SV=3 - [PSB5_MOUSE]	-0.18	0.07	0.27	0.07	0.24	-0.69	-0.46	-0.81	-0.55	-0.74	-0.52	-0.50	-0.30
P97470	Serine/threonine-protein phosphatase 4 catalytic subunit OS=Mus musculus GN=Ppp4c PE=1 SV=2 - [PP4C_MOUSE]	0.12	-0.01	-0.09	0.05	-0.11	-0.69	-0.93	-0.80	-0.86	-0.75	-0.86	-0.69	-0.78
Q8BG73	SH3 domain-binding glutamic acid-rich-like protein 2 OS=Mus musculus GN=Sh3bgrl2 PE=1 SV=1 - [SH3L2_MOUSE]	-0.17	-0.54	-0.27	-0.31	-0.07	-0.69	-0.49	-0.66	-0.50	-0.87	-0.66	-0.75	-0.54
Q9QUP5	Hyaluronan and proteoglycan link protein 1 OS=Mus musculus GN=Hapln1 PE=2 SV=1 - [HPLN1_MOUSE]	-0.10	-0.23	-0.11	-0.16	-0.04	-0.69	-0.52	-0.70	-0.59	-0.58	-0.45	-0.48	-0.45
P61971	Nuclear transport factor 2 OS=Mus musculus GN=Nutf2 PE=2 SV=1 - [NTF2_MOUSE]	-0.08	-0.12	-0.04	0.00	-0.03	-0.69	-0.52	-0.67	-0.58	-0.48	-0.43	-0.58	-0.47
O88968	Transcobalamin-2 OS=Mus musculus GN=Tcn2 PE=2 SV=1 - [TCO2_MOUSE]	0.00	-0.18	-0.18	-0.14	-0.14	-0.69	-0.69	-0.68	-0.68	-0.79	-0.79	-0.81	-0.81
Q8BFQ8	Parkinson disease 7 domain-containing protein 1 OS=Mus musculus GN=Pddc1 PE=1 SV=1 - [PDDC1_MOUSE]	0.16	-0.03	0.03	0.11	0.01	-0.68	-0.80	-0.77	-0.76	-0.68	-0.79	-0.65	-0.50
P09671	Superoxide dismutase [Mn], mitochondrial OS=Mus musculus GN=Sod2 PE=1 SV=3 - [SODM_MOUSE]	-0.01	-0.33	-0.31	-0.12	-0.10	-0.67	-0.63	-0.64	-0.65	-0.66	-0.64	-0.66	-0.68

Q60994	Adiponectin OS=Mus musculus GN=Adipoq PE=1 SV=2 - [ADIPO_MOUSE]	0.20	0.24	0.04	-0.05	-0.25	-0.67	-0.88	-0.74	-0.94	-0.45	-0.64	-0.53	-0.72
Q99PT1	Rho GDP-dissociation inhibitor 1 OS=Mus musculus GN=Arhgdia PE=1 SV=3 - [GDIR1_MOUSE]	0.08	-0.16	-0.24	0.01	-0.07	-0.67	-0.76	-0.71	-0.77	-0.74	-0.79	-0.71	-0.81
Q8QZY6	Tetraspanin-14 OS=Mus musculus GN=Tspan14 PE=1 SV=1 - [TSN14_MOUSE]	0.12	-0.12	-0.24	-0.11	-0.23	-0.66	-0.78	-0.32	-0.45	-0.31	-0.43	-0.86	-0.98
O88958	Glucosamine-6-phosphate isomerase 1 OS=Mus musculus GN=Gnpda1 PE=2 SV=3 - [GNPI1_MOUSE]	0.15	-0.32	-0.20	-0.19	-0.26	-0.65	-0.68	-0.90	-0.57	-0.75	-0.52	-0.85	-0.51
P11352	Glutathione peroxidase 1 OS=Mus musculus GN=Gpx1 PE=1 SV=2 - [GPX1_MOUSE]	-0.11	-0.19	-0.15	-0.09	0.06	-0.65	-0.54	-0.61	-0.55	-0.64	-0.51	-0.64	-0.39
Q8BGU2	Cerebellin-2 OS=Mus musculus GN=Cbln2 PE=1 SV=1 - [CBLN2_MOUSE]	0.08	-0.18	-0.26	-0.24	-0.26	-0.65	-0.74	-0.68	-0.77	-0.88	-0.96	-0.83	-0.80
P35700	Peroxiredoxin-1 OS=Mus musculus GN=Prdx1 PE=1 SV=1 - [PRDX1_MOUSE]	0.02	-0.26	-0.27	-0.14	-0.16	-0.64	-0.65	-0.53	-0.58	-0.65	-0.69	-0.68	-0.66
P56387	Dynein light chain Tctex-type 3 OS=Mus musculus GN=Dynlt3 PE=1 SV=1 - [DYLT3_MOUSE]	0.18	0.31	0.13	0.14	-0.05	-0.64	-0.83	-0.38	-0.57	-0.52	-0.70	-0.17	-0.35
P12382	6-phosphofructokinase, liver type OS=Mus musculus GN=Pfkl PE=1 SV=4 - [K6PL_MOUSE]	0.01	0.07	0.09	0.05	0.01	-0.64	-0.70	-0.56	-0.61	-0.63	-0.62	-0.41	-0.48
Q99JR1	Sideroflexin-1 OS=Mus musculus GN=Sfxn1 PE=1 SV=3 - [SFXN1_MOUSE]	0.05	0.57	0.51	0.23	0.13	-0.63	-0.66	-0.55	-0.49	-0.15	-0.25	-0.09	-0.10
Q9D2G2	Dihydrolipoyllysine-residue succinyltransferase component of 2-oxoglutarate dehydrogenase complex, mitochondrial OS=Mus musculus GN=Dlst PE=1 SV=1 - [ODO2_MOUSE]	0.01	-0.26	-0.23	-0.20	-0.22	-0.63	-0.63	-0.51	-0.53	-0.52	-0.54	-0.71	-0.69
D3YX28	Serine protease HTRA2, mitochondrial OS=Mus musculus GN=Htra2 PE=2 SV=1 - [D3YX28_MOUSE]	-0.07	-0.34	-0.22	-0.15	-0.16	-0.63	-0.57	-0.46	-0.43	-0.39	-0.38	-0.63	-0.54
P51910	Apolipoprotein D OS=Mus musculus GN=Apod PE=2 SV=1 - [APOD_MOUSE]	-0.15	-0.08	0.05	-0.12	0.05	-0.63	-0.55	-0.48	-0.52	-0.85	-0.66	-0.82	-0.61
Q61171	Peroxiredoxin-2 OS=Mus musculus GN=Prdx2 PE=1 SV=3 - [PRDX2_MOUSE]	0.06	-0.32	-0.37	-0.05	-0.11	-0.63	-0.66	-0.64	-0.68	-0.67	-0.68	-0.66	-0.74
Q9R1P1	Proteasome subunit beta type-3 OS=Mus musculus GN=Psmb3 PE=1 SV=1 - [PSB3_MOUSE]	-0.07	0.20	0.16	0.24	0.29	-0.62	-0.59	-0.66	-0.50	-0.48	-0.50	-0.45	-0.36
O88485	Cytoplasmic dynein 1 intermediate chain 1 OS=Mus musculus GN=Dync1i1 PE=1 SV=2 - [DC1I1_MOUSE]	-0.03	0.02	0.08	-0.13	-0.05	-0.62	-0.57	-0.64	-0.50	-0.62	-0.56	-0.67	-0.61
P17183	Gamma-enolase OS=Mus musculus GN=Eno2 PE=1 SV=2 - [ENOG_MOUSE]	0.01	-0.12	-0.14	-0.18	-0.18	-0.62	-0.65	-0.59	-0.58	-0.64	-0.64	-0.66	-0.69
Q9DD18	D-tyrosyl-tRNA(Tyr) deacylase 1 OS=Mus musculus GN=Dtd1 PE=1 SV=2 - [DTD1_MOUSE]	0.15	0.09	-0.06	-0.15	-0.19	-0.61	-0.73	-0.59	-0.64	-0.54	-0.71	-0.60	-0.66
P35290	Ras-related protein Rab-24 OS=Mus musculus GN=Rab24 PE=1 SV=2 - [RAB24_MOUSE]	-0.10	-0.23	0.00	0.01	0.00	-0.61	-0.54	-0.67	-0.69	-0.62	-0.50	-0.25	-0.25

Q9CQJ4	E3 ubiquitin-protein ligase RING2 OS=Mus musculus GN=Rnf2 PE=1 SV=1 - [RING2_MOUSE]	-0.10	-0.75	-0.66	-0.14	-0.04	-0.61	-0.52	-0.58	-0.48	-0.66	-0.56	-0.61	-0.51
Q9D404	3-oxoacyl-[acyl-carrier-protein] synthase, mitochondrial OS=Mus musculus GN=Oxsm PE=2 SV=1 - [OXSM_MOUSE]	0.00	-0.13	-0.15	-0.25	-0.23	-0.60	-0.67	-0.69	-0.69	-0.50	-0.65	-0.38	-0.13
P70677	Caspase-3 OS=Mus musculus GN=Casp3 PE=1 SV=1 - [CASP3_MOUSE]	0.27	-0.22	-0.46	-0.03	-0.24	-0.59	-0.93	-0.73	-0.99	-0.58	-0.77	-0.48	-0.64
O09131	Glutathione S-transferase omega-1 OS=Mus musculus GN=Gsto1 PE=2 SV=2 - [GSTO1_MOUSE]	-0.12	-0.35	-0.30	-0.18	-0.10	-0.59	-0.52	-0.72	-0.58	-0.64	-0.49	-0.59	-0.48
O08553	Dihydropyrimidinase-related protein 2 OS=Mus musculus GN=Dpysl2 PE=1 SV=2 - [DPYL2_MOUSE]	-0.12	-0.12	-0.03	-0.15	-0.03	-0.59	-0.45	-0.59	-0.44	-0.64	-0.48	-0.54	-0.41
Q91X72	Hemopexin OS=Mus musculus GN=Hpx PE=1 SV=2 - [HEMO_MOUSE]	-0.12	-0.25	-0.28	-0.58	-0.32	-0.59	-0.47	-1.09	-0.74	0.00	0.12	-0.53	-0.45
O09159	Lysosomal alpha-mannosidase OS=Mus musculus GN=Man2b1 PE=2 SV=4 - [MA2B1_MOUSE]	-0.09	-0.02	0.07	-0.31	-0.22	-0.58	-0.50	-0.83	-0.71	-0.72	-0.59	-0.62	-0.53
Q61599	Rho GDP-dissociation inhibitor 2 OS=Mus musculus GN=Arhgdib PE=1 SV=3 - [GDIR2_MOUSE]	-0.24	-0.44	-0.23	-0.05	0.18	-0.58	-0.45	-0.68	-0.51	-0.53	-0.38	-0.41	-0.21
Q99KC8	von Willebrand factor A domain-containing protein 5A OS=Mus musculus GN=Vwa5a PE=1 SV=2 - [VMA5A_MOUSE]	0.01	-0.13	-0.09	-0.23	-0.12	-0.58	-0.62	-0.54	-0.51	-0.51	-0.53	-0.51	-0.55
O88374	Branched-chain-amino-acid aminotransferase OS=Mus musculus GN=Bcat2 PE=2 SV=1 - [O88374_MOUSE]	-0.07	-0.32	0.10	0.35	0.01	-0.58	-0.88	-0.56	-0.50	-0.86	-0.79	-0.72	-0.28
P63300	Selenoprotein W OS=Mus musculus GN=Sepw1 PE=1 SV=3 - [SELW_MOUSE]	-0.01	-0.91	-0.91	-0.15	-0.14	-0.58	-0.58	-0.65	-0.65	-0.75	-0.74	-0.66	-0.65
P21460	Cystatin-C OS=Mus musculus GN=Cst3 PE=2 SV=2 - [CYTC_MOUSE]	-0.16	-0.24	-0.06	-0.24	-0.05	-0.58	-0.44	-0.55	-0.40	-0.51	-0.31	-0.57	-0.52
Q99PI8	Reticulon-4 receptor OS=Mus musculus GN=Rtn4r PE=2 SV=1 - [RTN4R_MOUSE]	-0.18	0.05	0.13	-0.08	0.10	-0.57	-0.43	-0.74	-0.63	-0.58	-0.48	-0.47	-0.31
P48962	ADP/ATP translocase 1 OS=Mus musculus GN=Slc25a4 PE=1 SV=4 - [ADT1_MOUSE]	0.01	0.17	0.22	0.42	0.39	-0.57	-0.59	-0.67	-0.66	-0.36	-0.35	-0.35	-0.24
Q8VEM8	Phosphate carrier protein, mitochondrial OS=Mus musculus GN=Slc25a3 PE=1 SV=1 - [MPCP_MOUSE]	-0.01	0.33	0.14	0.36	0.30	-0.57	-0.63	-0.66	-0.65	-0.36	-0.32	-0.19	-0.28
Q9Z2U0	Proteasome subunit alpha type-7 OS=Mus musculus GN=Psma7 PE=1 SV=1 - [PSA7_MOUSE]	-0.14	-0.10	0.09	-0.03	0.13	-0.57	-0.47	-0.54	-0.41	-0.62	-0.40	-0.51	-0.38
Q8BGN3	Ectonucleotide pyrophosphatase/phosphodiesterase family member 6 OS=Mus musculus GN=Enpp6 PE=2 SV=1 - [ENPP6_MOUSE]	-0.07	-0.09	0.11	-0.20	-0.06	-0.57	-0.45	-0.52	-0.40	-0.53	-0.42	-0.25	-0.13
Q9CQW2	ADP-ribosylation factor-like protein 8B OS=Mus musculus GN=Arl8b PE=2 SV=1 - [ARL8B_MOUSE]	0.18	-0.11	-0.30	0.07	-0.10	-0.56	-0.76	-0.54	-0.72	-0.48	-0.65	-0.41	-0.57

P42669	Transcriptional activator protein Pur-alpha OS=Mus musculus GN=Pura PE=1 SV=1 - [PURA_MOUSE]	0.06	-0.03	-0.16	-0.12	-0.17	-0.56	-0.65	-0.46	-0.54	-0.63	-0.65	-0.57	-0.61
F6QKK2	ADP-ribosylation factor-like protein 8A (Fragment) OS=Mus musculus GN=Arl8a PE=4 SV=1 - [F6QKK2_MOUSE]	0.20	-0.11	-0.31	0.17	-0.03	-0.56	-0.47	-0.75	-0.67	-0.64	-0.59	-0.53	-0.36
Q8K0S5	Reticulon-4 receptor-like 1 OS=Mus musculus GN=Rtn4rl1 PE=2 SV=1 - [R4RL1_MOUSE]	-0.07	-0.13	-0.06	-0.09	-0.03	-0.55	-0.50	-0.66	-0.60	-0.53	-0.46	-0.40	-0.34
P85094	Isochorismatase domain-containing protein 2A, mitochondrial OS=Mus musculus GN=Isoc2a PE=2 SV=1 - [ISC2A_MOUSE]	0.02	-0.04	-0.03	0.02	0.01	-0.55	-0.47	-0.72	-0.71	-0.43	-0.46	-0.26	-0.28
Q9D6M3	Mitochondrial glutamate carrier 1 OS=Mus musculus GN=Slc25a22 PE=1 SV=1 - [GHC1_MOUSE]	0.05	0.47	0.33	0.32	0.13	-0.55	-0.67	-0.56	-0.66	-0.18	-0.33	0.02	-0.06
Q99ME2	WD repeat-containing protein 6 OS=Mus musculus GN=Wdr6 PE=2 SV=1 - [WDR6_MOUSE]	0.18	0.31	0.07	0.03	-0.12	-0.55	-0.87	-0.47	-0.72	-0.28	-0.41	-0.05	-0.44
Q925I1	ATPase family AAA domain-containing protein 3 OS=Mus musculus GN=Atad3 PE=1 SV=1 - [ATAD3_MOUSE]	0.01	0.35	0.29	0.27	0.23	-0.55	-0.59	-0.50	-0.47	-0.40	-0.38	-0.18	-0.15
Q64378	Peptidyl-prolyl cis-trans isomerase FKBP5 OS=Mus musculus GN=Fkbp5 PE=1 SV=1 - [FKBP5_MOUSE]	-0.14	-0.37	-0.23	-0.46	-0.32	-0.54	-0.41	-0.63	-0.49	-0.66	-0.52	-0.61	-0.46
Q8C0M9	Isoaspartyl peptidase/L-asparaginase OS=Mus musculus GN=Asrgl1 PE=1 SV=1 - [ASGL1_MOUSE]	0.08	-0.31	-0.40	-0.16	-0.24	-0.54	-0.65	-0.49	-0.61	-0.53	-0.61	-0.39	-0.46
F7BX26	Serine/threonine-protein phosphatase (Fragment) OS=Mus musculus GN=Ppp5c PE=3 SV=1 - [F7BX26_MOUSE]	-0.02	-0.03	-0.03	-0.11	-0.10	-0.54	-0.50	-0.46	-0.47	-0.49	-0.47	-0.49	-0.47
O88967	ATP-dependent zinc metalloprotease YME1L1 OS=Mus musculus GN=Yme1l1 PE=2 SV=1 - [YMEL1_MOUSE]	0.14	0.22	0.07	0.37	0.23	-0.54	-0.69	-0.55	-0.69	-0.20	-0.34	-0.37	-0.51
P32211	Muscarinic acetylcholine receptor M4 OS=Mus musculus GN=Chrm4 PE=2 SV=1 - [ACM4_MOUSE]	0.06	-0.32	-0.38	-0.08	-0.14	-0.54	-0.60	-0.41	-0.47	-0.49	-0.54	-0.39	-0.45
Q8BM13	Noelin-2 OS=Mus musculus GN=Olfm2 PE=1 SV=2 - [NOE2_MOUSE]	-0.04	-0.19	-0.24	0.00	0.10	-0.53	-0.64	-0.55	-0.61	-0.70	-0.61	-0.58	-0.53
P51881	ADP/ATP translocase 2 OS=Mus musculus GN=Slc25a5 PE=1 SV=3 - [ADT2_MOUSE]	0.12	0.17	0.06	0.42	0.34	-0.53	-0.74	-0.68	-0.86	-0.35	-0.55	-0.26	-0.35
Q921I1	Serotransferrin OS=Mus musculus GN=Tf PE=1 SV=1 - [TRFE_MOUSE]	-0.11	-0.18	-0.07	-0.53	-0.41	-0.53	-0.44	-0.55	-0.45	0.02	0.13	-0.57	-0.39
Q91V09	WD repeat-containing protein 13 OS=Mus musculus GN=Wdr13 PE=1 SV=1 - [WDR13_MOUSE]	-0.02	0.13	0.06	-0.12	-0.13	-0.53	-0.60	-0.39	-0.45	-0.49	-0.48	-0.54	-0.52

P53395	Lipoamide acyltransferase component of branched-chain alpha-keto acid dehydrogenase complex, mitochondrial OS=Mus musculus GN=Dbt PE=2 SV=2 - [ODB2_MOUSE]	-0.04	-0.33	-0.44	-0.11	-0.12	-0.53	-0.51	-0.51	-0.41	-0.57	-0.46	-0.42	-0.38
P61329-2	Isoform 2 of Fibroblast growth factor 12 OS=Mus musculus GN=Fgf12 - [FGF12_MOUSE]	0.01	-0.38	-0.17	-0.19	-0.26	-0.53	-0.44	-0.54	-0.59	-0.52	-0.62	-0.50	-0.59
Q3TCR7	Dynamin-2 OS=Mus musculus GN=Dnm2 PE=2 SV=1 - [Q3TCR7_MOUSE]	0.04	0.32	0.28	0.25	0.31	-0.53	-0.58	-0.52	-0.46	-0.36	-0.29	-0.19	-0.12
Q8R086	Sulfite oxidase, mitochondrial OS=Mus musculus GN=Suox PE=1 SV=2 - [SUOX_MOUSE]	-0.01	-0.11	-0.10	0.19	0.20	-0.53	-0.53	-1.29	-1.29	-0.84	-0.83	-0.14	-0.13
P61982	14-3-3 protein gamma OS=Mus musculus GN=Ywhag PE=1 SV=2 - [1433G_MOUSE]	-0.07	-0.12	-0.05	-0.08	-0.02	-0.53	-0.42	-0.52	-0.37	-0.56	-0.43	-0.51	-0.42
Q64521	Glycerol-3-phosphate dehydrogenase, mitochondrial OS=Mus musculus GN=Gpd2 PE=1 SV=2 - [GPDM_MOUSE]	0.10	0.01	-0.10	0.03	-0.08	-0.52	-0.57	-0.57	-0.61	-0.54	-0.57	-0.51	-0.52
Q9DCC4	Pyrroline-5-carboxylate reductase 3 OS=Mus musculus GN=Pycrl PE=2 SV=2 - [P5CR3_MOUSE]	0.38	-0.11	-0.40	-0.04	-0.24	-0.52	-0.52	-0.35	-0.74	-0.38	-0.37	-0.39	-0.78
Q8C4U8	EGF-like repeat and discoidin I-like domain-containing protein 3 OS=Mus musculus GN=Edil3 PE=2 SV=1 - [Q8C4U8_MOUSE]	0.05	-0.03	0.00	-0.07	-0.10	-0.52	-0.46	-0.54	-0.45	-0.60	-0.65	-0.32	-0.34
P25785	Metalloproteinase inhibitor 2 OS=Mus musculus GN=Timp2 PE=1 SV=2 - [TIMP2_MOUSE]	0.03	-0.35	-0.03	-0.18	-0.37	-0.52	-0.59	-0.54	-0.57	-0.43	-0.27	-0.30	-0.41
Q00519	Xanthine dehydrogenase/oxidase OS=Mus musculus GN=Xdh PE=1 SV=5 - [XDH_MOUSE]	-0.11	-0.04	-0.18	-0.27	-0.15	-0.52	-0.87	-0.81	-0.65	-0.87	-0.62	-0.86	-0.74
Q9CXW3	Calcyclin-binding protein OS=Mus musculus GN=Cacybp PE=1 SV=1 - [CYBP_MOUSE]	0.29	-0.57	-0.72	-0.12	-0.36	-0.52	-0.78	-0.41	-0.74	-0.49	-0.78	-0.44	-0.67
P40237	CD82 antigen OS=Mus musculus GN=Cd82 PE=1 SV=1 - [CD82_MOUSE]	-0.03	-0.31	-0.30	0.06	0.03	-0.52	-0.41	-0.53	-0.52	-0.45	-0.37	-0.52	-0.40
Q924M7	Mannose-6-phosphate isomerase OS=Mus musculus GN=Mpi PE=2 SV=1 - [MPI_MOUSE]	0.15	-0.18	-0.33	-0.06	-0.17	-0.51	-0.72	-0.59	-0.66	-0.53	-0.66	-0.43	-0.60
O08749	Dihydrolipoyl dehydrogenase, mitochondrial OS=Mus musculus GN=Dld PE=1 SV=2 - [DLDH_MOUSE]	0.09	-0.15	-0.24	-0.29	-0.36	-0.51	-0.58	-0.54	-0.60	-0.56	-0.65	-0.29	-0.37
Q8BW96-2	Isoform 2 of Calcium/calmodulin-dependent protein kinase type 1D OS=Mus musculus GN=Camk1d - [KCC1D_MOUSE]	-0.04	-0.38	-0.35	-0.10	-0.15	-0.51	-0.57	-0.53	-0.53	-0.66	-0.68	-0.67	-0.74
P62881-2	Isoform 2 of Guanine nucleotide-binding protein subunit beta-5 OS=Mus musculus GN=Gnb5 - [GBB5_MOUSE]	-0.13	-0.17	-0.05	-0.06	0.02	-0.51	-0.44	-0.71	-0.57	-0.66	-0.52	-0.66	-0.50
Q3UPV6	Voltage-gated potassium channel subunit beta-2 OS=Mus musculus GN=Kcnab2 PE=2 SV=1 - [Q3UPV6_MOUSE]	-0.05	0.11	0.07	0.00	0.10	-0.51	-0.41	-0.54	-0.49	-0.54	-0.45	-0.49	-0.42

Q9D9V3-2	Isoform 2 of Ethylmalonyl-CoA decarboxylase OS=Mus musculus GN=Echdc1 - [ECHD1_MOUSE]	0.02	0.17	-0.21	0.00	-0.08	-0.51	-0.62	-0.64	-0.71	-0.55	-0.55	-1.00	-0.91
Q9DB50	AP-1 complex subunit sigma-2 OS=Mus musculus GN=Ap1s2 PE=2 SV=1 - [AP1S2_MOUSE]	-0.04	0.27	0.31	0.20	0.23	-0.50	-0.47	-0.73	-0.70	-0.43	-0.39	-0.47	-0.43
Q9D1X8	Tetraspanin 2, isoform CRA_b OS=Mus musculus GN=Tspan2 PE=2 SV=1 - [Q9D1X8_MOUSE]	0.19	0.58	0.19	0.11	-0.13	-0.50	-0.58	-0.51	-0.49	-0.30	-0.35	-0.42	-0.55
Q9CRB8	Mitochondrial fission process protein 1 OS=Mus musculus GN=Mtfp1 PE=1 SV=1 - [MTFP1_MOUSE]	-0.07	0.16	0.22	0.25	0.35	-0.50	-0.44	-0.60	-0.59	-0.41	-0.49	0.09	0.16
Q9D2F8	Protein 4930547C10Rik OS=Mus musculus GN=4930547C10Rik PE=2 SV=1 - [Q9D2F8_MOUSE]	0.75	1.06	0.31	-0.53	-1.28	-0.50	-1.25	-0.44	-1.19	-0.52	-1.27	-0.44	-1.19
Q8K215	LYR motif-containing protein 4 OS=Mus musculus GN=Lyrm4 PE=2 SV=1 - [LYRM4_MOUSE]	-0.02	-0.62	-0.60	-0.10	-0.08	-0.50	-0.49	-0.62	-0.61	-0.65	-0.63	-0.45	-0.43
Q80TB8	Synaptic vesicle membrane protein VAT-1 homolog-like OS=Mus musculus GN=Vat1l PE=2 SV=2 - [VAT1L_MOUSE]	0.43	0.17	-0.21	-0.01	-0.38	-0.50	-0.91	-0.77	-1.28	-0.30	-0.72	-0.13	-0.56
E9Q555	E3 ubiquitin-protein ligase RNF213 OS=Mus musculus GN=Rnf213 PE=2 SV=1 - [RN213_MOUSE]	0.10	0.07	-0.03	0.10	0.01	-0.49	-0.60	-0.51	-0.62	-0.20	-0.30	-0.51	-0.60
G3UZW8	Proteasome subunit beta type (Fragment) OS=Mus musculus GN=Psmb8 PE=2 SV=2 - [G3UZW8_MOUSE]	0.04	0.34	0.30	0.02	-0.02	-0.49	-0.54	-0.65	-0.69	-0.55	-0.59	-0.70	-0.73
Q9EPB4	Apoptosis-associated speck-like protein containing a CARD OS=Mus musculus GN=Pycard PE=1 SV=1 - [ASC_MOUSE]	0.04	-0.09	-0.12	-0.17	-0.15	-0.49	-0.62	-0.52	-0.46	-0.51	-0.64	-0.36	-0.41
Q9CRC9	Glucosamine-6-phosphate isomerase 2 OS=Mus musculus GN=Gnpda2 PE=2 SV=1 - [GNPI2_MOUSE]	-0.06	-0.07	0.12	-0.19	-0.12	-0.48	-0.55	-0.58	-0.59	-0.73	-0.53	-0.57	-0.20
Q9Z2L6	Multiple inositol polyphosphate phosphatase 1 OS=Mus musculus GN=Minpp1 PE=1 SV=3 - [MINP1_MOUSE]	-0.03	0.17	0.24	-0.07	0.02	-0.48	-0.46	-0.57	-0.47	-0.37	-0.42	-0.25	-0.24
Q9Z2X2	26S proteasome non-ATPase regulatory subunit 10 OS=Mus musculus GN=Psmd10 PE=1 SV=3 - [PSD10_MOUSE]	0.09	0.15	0.06	-0.02	-0.11	-0.48	-0.58	-0.36	-0.46	-0.39	-0.47	-0.11	-0.20
Q9JMA2	Queuine tRNA-ribosyltransferase OS=Mus musculus GN=Qtrt1 PE=1 SV=2 - [TGT_MOUSE]	0.11	0.67	0.56	0.09	-0.01	-0.48	-0.59	-0.64	-0.75	-0.36	-0.47	-0.14	-0.24
Q9JJA4	Ribosome biogenesis protein WDR12 OS=Mus musculus GN=Wdr12 PE=2 SV=1 - [WDR12_MOUSE]	-0.01	-0.33	-0.24	-0.01	-0.20	-0.48	-0.56	-0.43	-0.49	-0.60	-0.58	-0.27	-0.31
P56376	Acylphosphatase-1 OS=Mus musculus GN=Acyp1 PE=2 SV=2 - [ACYP1_MOUSE]	-0.04	-0.39	-0.42	-0.19	-0.07	-0.47	-0.41	-0.37	-0.37	-0.67	-0.43	-0.67	-0.43
Q9CXJ4	ATP-binding cassette sub-family B member 8, mitochondrial OS=Mus musculus GN=Abcb8 PE=2 SV=1 - [ABCB8_MOUSE]	0.04	0.37	0.36	0.22	0.22	-0.47	-0.48	-0.43	-0.48	-0.20	-0.14	-0.07	-0.07

Q922V4	Pleiotropic regulator 1 OS=Mus musculus GN=Plrg1 PE=2 SV=1 - [PLRG1_MOUSE]	0.15	-0.38	-0.35	-0.17	-0.38	-0.47	-0.79	-0.59	-0.63	-0.55	-0.82	-0.51	-0.75
Q64310	Surfeit locus protein 4 OS=Mus musculus GN=Surf4 PE=2 SV=1 - [SURF4_MOUSE]	0.02	0.39	0.36	0.05	0.03	-0.46	-0.50	-0.40	-0.43	-0.38	-0.40	-0.10	-0.12
Q9DB42	Zinc finger protein 593 OS=Mus musculus GN=Znf593 PE=2 SV=2 - [ZN593_MOUSE]	-0.01	-0.24	-0.23	-0.34	-0.33	-0.46	-0.46	-0.47	-0.47	-0.28	-0.27	-0.38	-0.37
P29758	Ornithine aminotransferase, mitochondrial OS=Mus musculus GN=Oat PE=1 SV=1 - [OAT_MOUSE]	0.14	-0.05	-0.12	0.08	-0.04	-0.46	-0.57	-0.37	-0.45	-0.35	-0.40	-0.38	-0.42
Q9D8B6	Protein FAM210B OS=Mus musculus GN=Fam210b PE=2 SV=3 - [F210B_MOUSE]	0.21	-0.31	-0.52	0.36	0.15	-0.45	-0.67	-0.59	-0.80	-0.25	-0.46	-0.22	-0.43
P22315	Ferrochelatase, mitochondrial OS=Mus musculus GN=Fech PE=1 SV=2 - [HEMH_MOUSE]	0.00	-0.20	-0.18	-0.14	-0.05	-0.44	-0.43	-0.53	-0.49	-0.48	-0.52	-0.58	-0.45
P26049	Gamma-aminobutyric acid receptor subunit alpha-3 OS=Mus musculus GN=Gabra3 PE=1 SV=1 - [GBRA3_MOUSE]	-0.01	-0.34	-0.33	0.18	0.19	-0.44	-0.44	-0.44	-0.43	-0.19	-0.17	-0.27	-0.26
Q8BGT1	Fibronectin leucine rich transmembrane protein 3 OS=Mus musculus GN=Flrt3 PE=2 SV=1 - [Q8BGT1_MOUSE]	0.13	-0.36	-0.43	0.01	-0.09	-0.44	-0.58	-0.67	-0.74	-0.71	-0.81	-0.56	-0.63
Q8BH59	Calcium-binding mitochondrial carrier protein Aralar1 OS=Mus musculus GN=Slc25a12 PE=1 SV=1 - [CMC1_MOUSE]	0.09	0.21	0.21	0.33	0.31	-0.44	-0.49	-0.47	-0.57	-0.29	-0.34	-0.07	-0.17
D3YVU9	3-hydroxymethyl-3-methylglutaryl-CoA lyase, cytoplasmic OS=Mus musculus GN=Hmgcll1 PE=2 SV=1 - [D3YVU9_MOUSE]	0.07	0.28	0.20	0.06	-0.01	-0.43	-0.51	-0.33	-0.41	-0.16	-0.23	-0.12	-0.19
O70370	Cathepsin S OS=Mus musculus GN=Ctss PE=2 SV=2 - [CATS_MOUSE]	0.08	-0.18	-0.20	-0.15	-0.20	-0.43	-0.57	-0.54	-0.89	-0.58	-0.69	-0.66	-0.66
Q8R033	LYR motif-containing protein 2 OS=Mus musculus GN=Lyrm2 PE=2 SV=1 - [LYRM2_MOUSE]	-0.01	0.36	0.36	-0.20	-0.19	-0.43	-0.43	-0.37	-0.37	-0.47	-0.46	-0.46	-0.45
Q810C1	SLIT and NTRK-like protein 1 OS=Mus musculus GN=Slitrk1 PE=2 SV=1 - [SLIK1_MOUSE]	-0.03	-0.46	-0.58	-0.13	-0.11	-0.42	-0.51	-0.41	-0.70	-0.42	-0.70	-0.42	-0.57
Q8BGN5	NIPA-like protein 3 OS=Mus musculus GN=Nipal3 PE=2 SV=1 - [NPAL3_MOUSE]	0.01	-0.06	-0.07	0.03	0.02	-0.42	-0.43	-0.41	-0.42	-0.29	-0.29	-0.27	-0.27
Q8VDP6	CDP-diacylglycerol--inositol 3-phosphatidyltransferase OS=Mus musculus GN=Cdipt PE=1 SV=1 - [CDIPT_MOUSE]	-0.03	0.05	0.08	0.25	0.28	-0.42	-0.40	-0.46	-0.43	-0.50	-0.47	0.04	0.08
E9PZ69	Transmembrane 9 superfamily member 2 OS=Mus musculus GN=Tm9sf2 PE=2 SV=1 - [E9PZ69_MOUSE]	0.12	0.10	0.21	0.24	0.30	-0.42	-0.67	-0.49	-0.58	-0.34	-0.11	-0.30	-0.31
Q9JHU9	Inositol-3-phosphate synthase 1 OS=Mus musculus GN=Isyna1 PE=2 SV=1 - [INO1_MOUSE]	0.29	0.02	-0.21	-0.17	-0.39	-0.42	-0.77	-0.34	-0.59	-0.30	-0.63	-0.10	-0.36
O88507	Ciliary neurotrophic factor receptor subunit alpha OS=Mus musculus GN=Cntfr PE=1 SV=2 - [CNTFR_MOUSE]	0.17	-0.02	-0.31	0.00	-0.25	-0.41	-0.47	-0.39	-0.50	-0.41	-0.70	-0.10	-0.27

Q99PH1	Leucine-rich repeat-containing protein 4 OS=Mus musculus GN=Lrrc4 PE=1 SV=2 - [LRRC4_MOUSE]	0.07	0.31	0.24	0.06	-0.01	-0.41	-0.49	-0.45	-0.52	-0.64	-0.71	-0.55	-0.62
P61922	4-aminobutyrate aminotransferase, mitochondrial OS=Mus musculus GN=Abat PE=1 SV=1 - [GABT_MOUSE]	-0.02	0.06	0.07	0.07	0.11	-0.41	-0.42	-0.53	-0.45	-0.25	-0.25	-0.22	-0.18
Q9CXT7-2	Isoform 2 of Transmembrane protein 192 OS=Mus musculus GN=Tmem192 - [TM192_MOUSE]	0.14	0.44	0.29	0.16	0.02	-0.40	-0.54	-0.31	-0.45	-0.25	-0.38	-0.13	-0.27
Q9JHW2	Omega-amidase NIT2 OS=Mus musculus GN=Nit2 PE=1 SV=1 - [NIT2_MOUSE]	-0.05	-0.20	-0.11	-0.05	0.02	-0.39	-0.42	-0.42	-0.48	-0.39	-0.36	-0.26	-0.19
P17563	Selenium-binding protein 1 OS=Mus musculus GN=Selenbp1 PE=1 SV=2 - [SBP1_MOUSE]	0.17	0.02	0.04	0.07	-0.10	-0.39	-0.50	-0.43	-0.50	-0.39	-0.57	-0.33	-0.49
Q9DC50	Peroxisomal carnitine O-octanoyltransferase OS=Mus musculus GN=Crot PE=1 SV=1 - [OCTC_MOUSE]	0.29	0.33	0.03	-0.51	-0.80	-0.39	-0.57	-0.46	-0.76	-0.36	-0.65	-0.68	-0.65
P83877	Thioredoxin-like protein 4A OS=Mus musculus GN=Txnl4a PE=2 SV=1 - [TXN4A_MOUSE]	0.19	-0.45	-0.68	-0.22	-0.41	-0.39	-0.71	-0.79	-1.15	-0.71	-0.98	-0.42	-0.56
A2ASZ8-4	Isoform 4 of Calcium-binding mitochondrial carrier protein SCaMC-2 OS=Mus musculus GN=Slc25a25 - [SCMC2_MOUSE]	0.13	0.17	0.05	0.21	0.17	-0.38	-0.59	-0.40	-0.42	-0.17	-0.46	-0.09	-0.32
Q8CC88	von Willebrand factor A domain-containing protein 8 OS=Mus musculus GN=Vwa8 PE=2 SV=2 - [VWA8_MOUSE]	0.05	0.30	0.26	0.20	0.12	-0.38	-0.47	-0.47	-0.63	-0.29	-0.26	-0.06	-0.28
Q3UYH7	Beta-adrenergic receptor kinase 2 OS=Mus musculus GN=Adrbk2 PE=2 SV=2 - [ARBK2_MOUSE]	0.14	-0.11	-0.32	0.13	-0.01	-0.38	-0.53	-0.48	-0.63	0.05	-0.09	-0.04	-0.18
P61021	Ras-related protein Rab-5B OS=Mus musculus GN=Rab5b PE=1 SV=1 - [RAB5B_MOUSE]	0.20	0.12	-0.16	0.33	0.08	-0.37	-0.53	-0.41	-0.52	-0.30	-0.62	-0.01	-0.23
Q9WVQ5	Methylthioribulose-1-phosphate dehydratase OS=Mus musculus GN=Apip PE=1 SV=1 - [MTNB_MOUSE]	0.15	-0.04	-0.43	-0.17	-0.19	-0.37	-0.44	-0.35	-0.52	-0.38	-0.59	-0.41	-0.77
Q791V5	Mitochondrial carrier homolog 2 OS=Mus musculus GN=Mtch2 PE=1 SV=1 - [MTCH2_MOUSE]	0.01	0.23	0.17	0.19	0.18	-0.37	-0.53	-0.35	-0.40	-0.25	-0.31	-0.01	-0.10
Q8BGA3	Leucine-rich repeat transmembrane neuronal protein 2 OS=Mus musculus GN=Lrrtm2 PE=2 SV=1 - [LRRT2_MOUSE]	0.08	-0.24	-0.36	0.06	0.14	-0.36	-0.46	-0.38	-0.47	-0.32	-0.41	-0.30	-0.23
O35129	Prohibitin-2 OS=Mus musculus GN=Phb2 PE=1 SV=1 - [PHB2_MOUSE]	0.08	0.26	0.20	0.23	0.17	-0.36	-0.40	-0.36	-0.51	-0.26	-0.33	-0.19	-0.23
Q99ML4	Protein FAM69B OS=Mus musculus GN=Fam69b PE=1 SV=1 - [FA69B_MOUSE]	0.10	0.40	0.30	0.17	0.06	-0.36	-0.47	-0.42	-0.52	-0.13	-0.23	0.03	-0.07
Q91YS8	Calcium/calmodulin-dependent protein kinase type 1 OS=Mus musculus GN=Camk1 PE=1 SV=1 - [KCC1A_MOUSE]	0.02	-0.46	-0.43	-0.16	-0.01	-0.36	-0.43	-0.39	-0.42	-0.45	-0.47	-0.55	-0.41

Q922P8	Transmembrane protein 132A OS=Mus musculus GN=Tmem132a PE=2 SV=2 - [T132A_MOUSE]	0.06	0.11	0.07	-0.02	-0.10	-0.35	-0.45	-0.42	-0.37	-0.37	-0.45	-0.52	-0.47
D3YWV5	Semaphorin-4A OS=Mus musculus GN=Sema4a PE=2 SV=1 - [D3YWV5_MOUSE]	0.13	-0.05	-0.26	0.13	0.02	-0.35	-0.65	-0.31	-0.45	-0.24	-0.27	-0.16	-0.30
P63328	Serine/threonine-protein phosphatase 2B catalytic subunit alpha isoform OS=Mus musculus GN=Ppp3ca PE=1 SV=1 - [PP2BA_MOUSE]	-0.05	-0.02	-0.09	-0.16	-0.10	-0.35	-0.43	-0.40	-0.52	-0.40	-0.50	-0.31	-0.31
O09164	Extracellular superoxide dismutase [Cu-Zn] OS=Mus musculus GN=Sod3 PE=1 SV=1 - [SODE_MOUSE]	0.03	-0.07	0.03	-0.14	-0.19	-0.35	-0.39	-0.43	-0.47	-0.27	-0.30	-0.02	-0.05
Q3UUQ7	GPI inositol-deacylase OS=Mus musculus GN=Pgap1 PE=1 SV=3 - [PGAP1_MOUSE]	0.38	0.27	-0.11	0.09	-0.29	-0.35	-0.73	-0.32	-0.71	-0.13	-0.51	0.22	-0.16
E9PXF7	Protein Gm14446 OS=Mus musculus GN=Gm14446 PE=2 SV=1 - [E9PXF7_MOUSE]	0.09	-0.42	-0.52	1.05	0.95	1.86	1.76	3.35	3.26	3.10	3.01	1.96	1.87
Q8BLR5	PH and SEC7 domain-containing protein 4 OS=Mus musculus GN=Psd4 PE=2 SV=1 - [PSD4_MOUSE]	0.22	0.17	-0.05	0.42	0.19	1.77	1.54	0.75	0.52	0.91	0.69	0.26	0.04
Q01149	Collagen alpha-2(I) chain OS=Mus musculus GN=Col1a2 PE=2 SV=2 - [CO1A2_MOUSE]	0.14	0.08	-0.10	0.00	-0.11	1.75	1.50	0.65	0.39	0.72	0.55	0.20	0.01
P11087	Collagen alpha-1(I) chain OS=Mus musculus GN=Col1a1 PE=1 SV=4 - [CO1A1_MOUSE]	0.10	-0.06	-0.13	0.04	-0.09	1.58	1.42	0.47	0.44	0.57	0.40	0.20	0.12
A2AJ76	Hemicentin-2 OS=Mus musculus GN=Hmcn2 PE=1 SV=1 - [HMCN2_MOUSE]	0.48	0.34	-0.14	0.83	0.35	1.46	0.98	1.00	0.52	1.86	1.38	1.48	1.01
Q8BZR9	Uncharacterized protein C17orf85 homolog OS=Mus musculus PE=1 SV=1 - [CQ085_MOUSE]	0.15	0.39	0.24	0.43	0.28	1.10	0.94	0.81	0.65	0.62	0.48	0.31	0.16
Q543V3	Insulin receptor substrate 1 OS=Mus musculus GN=Irs1 PE=2 SV=1 - [Q543V3_MOUSE]	-0.13	0.19	0.32	0.22	0.35	0.99	1.11	0.62	0.75	0.28	0.41	-0.04	0.09
E0CZ73	Occludin OS=Mus musculus GN=Ocln PE=2 SV=1 - [E0CZ73_MOUSE]	0.34	0.48	0.14	0.57	0.23	0.94	0.59	1.10	0.76	1.08	0.74	0.62	0.29
O88492	Perilipin-4 OS=Mus musculus GN=Plin4 PE=1 SV=2 - [PLIN4_MOUSE]	0.07	-0.14	-0.21	-0.06	-0.13	0.87	0.79	0.64	0.57	0.52	0.45	0.47	0.40
Q7TNS2	Mitochondrial inner membrane organizing system protein 1 OS=Mus musculus GN=Minos1 PE=2 SV=1 - [MOS1_MOUSE]	0.22	0.95	0.73	0.41	0.19	0.85	0.62	0.59	0.37	0.71	0.50	0.82	0.61
P97304	DNA-directed RNA polymerases I and III subunit RPAC2 OS=Mus musculus GN=Polr1d PE=2 SV=1 - [RPAC2_MOUSE]	0.03	-0.22	-0.25	-0.04	-0.07	0.81	0.77	0.91	0.87	0.61	0.57	0.58	0.54
E9Q813	Ras-GEF domain-containing family member 1B OS=Mus musculus GN=Rasgef1b PE=2 SV=1 - [E9Q813_MOUSE]	0.33	0.85	0.51	0.52	0.18	0.80	0.46	1.11	0.77	0.98	0.65	0.87	0.54

D3Z4F2	Protein Zfp819 (Fragment) OS=Mus musculus GN=Zfp819 PE=2 SV=1 - [D3Z4F2_MOUSE]	-0.04	-0.04	0.00	0.02	0.06	0.80	0.83	1.11	1.15	0.82	0.87	0.68	0.72
E9Q3B5	Uncharacterized protein OS=Mus musculus GN=Gm10774 PE=3 SV=1 - [E9Q3B5_MOUSE]	-0.16	-0.48	-0.31	-0.36	-0.20	0.77	0.93	0.63	0.79	0.20	0.37	0.19	0.36
D3Z723	Nuclear transcription factor Y subunit beta (Fragment) OS=Mus musculus GN=Nfyb PE=2 SV=1 - [D3Z723_MOUSE]	0.08	0.55	0.47	0.14	0.06	0.77	0.68	0.94	0.85	0.73	0.66	0.58	0.50
Q8BGT0	Osteopetrosis-associated transmembrane protein 1 OS=Mus musculus GN=Ostm1 PE=1 SV=1 - [OSTM1_MOUSE]	-0.08	0.16	0.23	0.21	0.29	0.77	0.84	0.81	0.89	0.80	0.89	0.96	1.04
D9J2V6	Pre-B-cell leukemia transcription factor 1 OS=Mus musculus GN=Pbx1 PE=2 SV=1 - [D9J2V6_MOUSE]	-0.22	0.05	0.27	0.21	0.43	0.76	0.98	0.35	0.57	0.21	0.44	0.38	0.60
Q8BUM6	Protein FAM163B OS=Mus musculus GN=Fam163b PE=1 SV=1 - [F163B_MOUSE]	0.05	0.17	0.12	0.02	-0.02	0.75	0.70	0.77	0.72	0.60	0.56	0.11	0.06
Q9R0A0	Peroxisomal membrane protein PEX14 OS=Mus musculus GN=Pex14 PE=1 SV=1 - [PEX14_MOUSE]	0.09	0.29	0.33	0.11	0.15	0.74	0.66	0.70	0.76	0.70	0.70	0.57	0.49
G3UZ12	Protein Jumonji OS=Mus musculus GN=Jarid2 PE=2 SV=1 - [G3UZ12_MOUSE]	0.00	0.01	0.01	-0.10	-0.11	0.74	0.73	0.81	0.80	0.72	0.72	0.61	0.61
F7BGR7	RNA-binding protein 4 OS=Mus musculus GN=Rbm4 PE=4 SV=1 - [F7BGR7_MOUSE]	0.02	-0.08	-0.10	0.61	0.59	0.74	0.71	0.56	0.54	0.57	0.56	0.29	0.27
Q99ML1	Bcl-2-binding component 3 OS=Mus musculus GN=Bbc3 PE=1 SV=1 - [BBC3_MOUSE]	0.16	0.29	0.13	-0.04	-0.20	0.71	0.55	0.86	0.70	0.86	0.71	0.73	0.58
Q8C790-2	Isoform 2 of Protein FAM221A OS=Mus musculus GN=Fam221a - [F221A_MOUSE]	0.12	0.10	-0.03	0.13	0.02	0.71	0.58	0.57	0.45	0.52	0.41	0.45	0.34
Q8BW74	Hepatic leukemia factor OS=Mus musculus GN=Hlf PE=2 SV=1 - [HLF_MOUSE]	-0.62	-0.16	0.46	-0.50	0.12	0.70	1.31	0.94	1.56	0.68	1.30	0.31	0.93
P23949	Zinc finger protein 36, C3H1 type-like 2 OS=Mus musculus GN=Zfp36l2 PE=2 SV=1 - [TISD_MOUSE]	0.29	-0.01	-0.30	0.37	0.08	0.70	0.40	0.84	0.55	0.39	0.10	0.33	0.04
Q7M6Y6	Maestro heat-like repeat-containing protein family member 2B OS=Mus musculus GN=Mroh2b PE=1 SV=2 - [MRO2B_MOUSE]	-0.52	0.04	0.56	-0.17	0.35	0.69	1.20	0.65	1.17	0.16	0.68	-0.32	0.21
Q9EST5	Acidic leucine-rich nuclear phosphoprotein 32 family member B OS=Mus musculus GN=Anp32b PE=1 SV=1 - [AN32B_MOUSE]	0.20	0.01	0.15	0.25	0.05	0.68	0.47	0.64	0.43	0.33	0.13	0.85	0.65
P97783	Protein AF1q OS=Mus musculus GN=Mllt11 PE=2 SV=1 - [AF1Q_MOUSE]	-0.01	-0.07	0.11	-0.09	-0.09	0.65	0.64	0.57	0.80	0.50	0.51	0.22	0.27
Q5RL20	39S ribosomal protein L43, mitochondrial OS=Mus musculus GN=Mrpl43 PE=2 SV=1 - [Q5RL20_MOUSE]	0.05	0.27	0.22	0.29	0.24	0.65	0.59	0.69	0.64	0.65	0.61	0.45	0.41
Q9WVF8	Tumor suppressor candidate 2 OS=Mus musculus GN=Tusc2 PE=1 SV=3 - [TUSC2_MOUSE]	0.05	0.01	-0.10	-0.06	-0.17	0.63	0.56	0.73	0.89	0.58	0.40	0.41	0.53

P21126	Ubiquitin-like protein 4A OS=Mus musculus GN=Ubl4a PE=2 SV=1 - [UBL4A_MOUSE]	-0.06	-0.14	-0.16	-0.06	-0.03	0.62	0.60	0.54	0.55	0.42	0.46	0.35	0.43
Q8CJ40	Rootletin OS=Mus musculus GN=Crocc PE=1 SV=2 - [CROCC_MOUSE]	0.05	0.14	0.06	-0.01	-0.08	0.62	0.54	0.65	0.58	0.54	0.49	0.55	0.52
Q9CY57-5	Isoform 5 of Chromatin target of PRMT1 protein OS=Mus musculus GN=Chtop - [CHTOP_MOUSE]	0.11	0.15	-0.17	0.08	0.05	0.62	0.46	0.61	0.54	0.58	0.51	0.37	0.41
P43300	Early growth response protein 3 OS=Mus musculus GN=Egr3 PE=2 SV=2 - [EGR3_MOUSE]	-0.03	-0.05	-0.03	-0.01	0.04	0.61	0.57	0.49	0.49	0.46	0.46	0.26	0.25
Q8VE92	RNA-binding protein 4B OS=Mus musculus GN=Rbm4b PE=1 SV=1 - [RBM4B_MOUSE]	0.01	0.01	0.00	0.00	-0.01	0.60	0.58	0.85	0.83	0.50	0.49	0.63	0.61
F6VV25	Pleckstrin homology domain-containing family O member 1 (Fragment) OS=Mus musculus GN=Plekho1 PE=2 SV=1 - [F6VV25_MOUSE]	-0.02	0.10	0.12	0.12	0.14	0.60	0.61	1.22	1.24	0.95	0.97	0.48	0.50
Q61550	Double-strand-break repair protein rad21 homolog OS=Mus musculus GN=Rad21 PE=1 SV=3 - [RAD21_MOUSE]	0.16	0.18	0.02	0.41	0.25	0.58	0.42	0.70	0.53	0.76	0.61	0.31	0.15
Q99K90	TGF-beta-activated kinase 1 and MAP3K7-binding protein 2 OS=Mus musculus GN=Tab2 PE=1 SV=1 - [TAB2_MOUSE]	0.10	0.10	0.19	0.02	-0.06	0.57	0.42	0.65	0.54	0.53	0.43	0.74	0.66
Q9WVH4	Forkhead box protein O3 OS=Mus musculus GN=Foxo3 PE=1 SV=1 - [FOXO3_MOUSE]	-0.23	-0.32	-0.09	0.07	0.30	0.57	0.80	0.32	0.55	0.18	0.42	0.32	0.56
O35738	Krueppel-like factor 12 OS=Mus musculus GN=Klf12 PE=2 SV=2 - [KLF12_MOUSE]	-0.03	0.11	0.13	-0.17	-0.15	0.57	0.59	0.62	0.64	0.43	0.46	0.24	0.27
F6V035	Protein Ccdc149 OS=Mus musculus GN=Ccdc149 PE=4 SV=1 - [F6V035_MOUSE]	0.09	0.06	0.04	-0.05	-0.14	0.57	0.52	0.51	0.45	0.17	0.18	0.14	0.04
Q8BYZ1	ABI gene family member 3 OS=Mus musculus GN=Abi3 PE=2 SV=3 - [ABI3_MOUSE]	-0.06	0.00	0.03	-0.07	-0.06	0.57	0.60	0.50	0.48	0.22	0.26	0.08	0.14
I3ITR1	MCG50313 OS=Mus musculus GN=AK157302 PE=4 SV=1 - [I3ITR1_MOUSE]	0.06	-0.18	-0.27	0.08	-0.01	0.56	0.51	0.39	0.33	0.17	0.16	0.24	0.24
Q9CPV3	39S ribosomal protein L42, mitochondrial OS=Mus musculus GN=Mrpl42 PE=2 SV=1 - [RM42_MOUSE]	-0.13	-0.54	-0.41	-0.15	-0.03	0.56	0.68	0.68	0.80	0.41	0.54	0.11	0.24
J3QQ44	Endophilin-A3 OS=Mus musculus GN=Sh3gl3 PE=4 SV=1 - [J3QQ44_MOUSE]	0.03	0.35	0.31	0.29	0.25	0.56	0.52	0.59	0.55	0.68	0.65	0.50	0.47
D3Z6F3	INO80 complex subunit C OS=Mus musculus GN=Ino80c PE=2 SV=1 - [D3Z6F3_MOUSE]	-0.39	0.15	0.53	-0.27	0.11	0.55	0.93	0.50	0.88	0.29	0.68	0.26	0.65
E0CYD7	Putative E3 ubiquitin-protein ligase UNKL OS=Mus musculus GN=Unkl PE=2 SV=1 - [E0CYD7_MOUSE]	-0.04	-0.12	-0.08	-0.11	-0.07	0.55	0.58	0.65	0.69	0.46	0.50	0.19	0.24
Q8C5R2	Proline and serine-rich protein 2 OS=Mus musculus GN=Proser2 PE=1 SV=3 - [PRSR2_MOUSE]	0.13	0.13	0.00	-0.04	-0.17	0.55	0.41	0.80	0.67	0.55	0.42	0.11	-0.02
O70279	Protein DGCR14 OS=Mus musculus GN=Dgcr14 PE=2 SV=2 - [DGC14_MOUSE]	0.17	0.24	0.21	0.18	0.02	0.55	0.60	0.60	0.45	0.33	0.16	0.19	0.01

Q9DAW9	Calponin-3 OS=Mus musculus GN=Cnn3 PE=2 SV=1 - [CNN3_MOUSE]	-0.12	-0.08	-0.08	0.02	0.05	0.54	0.52	0.53	0.60	0.42	0.41	0.23	0.28
Q9JJR9	Nuclear receptor-interacting protein 3 OS=Mus musculus GN=Nrip3 PE=2 SV=1 - [NRIP3_MOUSE]	-0.26	-0.57	-0.31	-0.21	0.05	0.54	0.79	0.45	0.71	0.27	0.53	0.08	0.34
Q3ULB5	Serine/threonine-protein kinase PAK 6 OS=Mus musculus GN=Pak6 PE=2 SV=1 - [PAK6_MOUSE]	0.08	-0.01	-0.09	0.00	-0.08	0.53	0.44	0.61	0.52	0.34	0.26	0.17	0.09
Q62165	Dystroglycan OS=Mus musculus GN=Dag1 PE=1 SV=4 - [DAG1_MOUSE]	0.12	-0.37	-0.39	-0.07	-0.20	0.52	0.36	0.48	0.34	0.36	0.19	0.19	0.03
Q3U962	Collagen alpha-2(V) chain OS=Mus musculus GN=Col5a2 PE=1 SV=1 - [CO5A2_MOUSE]	0.03	0.42	0.39	0.12	0.09	0.52	0.48	0.54	0.50	0.49	0.46	0.60	0.57
Q80X32	UPF0461 protein C5orf24 homolog OS=Mus musculus PE=1 SV=1 - [CE024_MOUSE]	0.13	-0.72	-0.86	0.25	0.11	0.52	0.37	0.49	0.35	0.32	0.18	0.23	0.10
Q9QZS0	Collagen alpha-3(IV) chain OS=Mus musculus GN=Col4a3 PE=1 SV=2 - [CO4A3_MOUSE]	-0.14	0.18	0.31	-0.04	0.10	0.52	0.64	0.69	0.82	0.95	1.09	0.24	0.38
Q8K2T8	RNA polymerase II-associated factor 1 homolog OS=Mus musculus GN=Paf1 PE=2 SV=1 - [PAF1_MOUSE]	0.05	-0.20	-0.41	-0.18	-0.22	0.51	0.38	0.39	0.36	0.36	0.34	0.12	0.20
P04370	Myelin basic protein OS=Mus musculus GN=Mbp PE=1 SV=2 - [MBP_MOUSE]	0.05	0.30	0.25	0.34	0.29	0.51	0.46	0.42	0.37	0.63	0.58	0.29	0.24
Q8CDM3	X-linked retinitis pigmentosa GTPase regulator OS=Mus musculus GN=Rpgr PE=2 SV=1 - [Q8CDM3_MOUSE]	0.11	-0.09	-0.20	0.26	0.16	0.51	0.40	0.52	0.41	0.38	0.28	0.49	0.38
B1AXP3	MCG1697, isoform CRA_a OS=Mus musculus GN=Fam154a PE=4 SV=1 - [B1AXP3_MOUSE]	-0.46	-0.24	0.23	-1.05	-0.59	0.50	0.96	0.33	0.79	-0.27	0.19	-0.88	-0.41
Q99JI1	Musculoskeletal embryonic nuclear protein 1 OS=Mus musculus GN=Mustn1 PE=2 SV=1 - [MSTN1_MOUSE]	0.10	0.00	-0.11	0.04	-0.06	0.50	0.39	0.50	0.39	0.31	0.21	0.16	0.06
O55074	A-kinase anchor protein 7 isoform alpha OS=Mus musculus GN=Akap7 PE=1 SV=4 - [AKA7A_MOUSE]	0.07	0.09	0.01	-0.01	-0.07	0.49	0.40	0.44	0.32	0.31	0.23	0.12	0.04
O88685	26S protease regulatory subunit 6A OS=Mus musculus GN=Psmc3 PE=1 SV=2 - [PRS6A_MOUSE]	-0.16	-0.04	0.02	-0.09	0.03	0.49	0.57	0.54	0.65	0.40	0.50	0.22	0.30
A2AR95-3	Isoform 3 of Low-density lipoprotein receptor class A domain-containing protein 3 OS=Mus musculus GN=Ldlrad3 - [LRAD3_MOUSE]	0.04	-0.11	-0.15	-0.88	-0.92	0.49	0.44	0.68	0.64	0.16	0.12	0.06	0.02
E9Q722	Upstream stimulatory factor 1 OS=Mus musculus GN=Usf1 PE=2 SV=1 - [E9Q722_MOUSE]	-0.01	-0.22	-0.17	-0.04	-0.04	0.49	0.49	0.48	0.53	0.49	0.50	0.29	0.30
Q80Y83-11	Isoform 11 of Dixin OS=Mus musculus GN=Dixdc1 - [DIXC1_MOUSE]	-0.04	0.00	0.03	0.05	0.09	0.49	0.48	0.45	0.53	0.37	0.50	0.30	0.27
Q3THG9	Alanyl-tRNA editing protein Aarsd1 OS=Mus musculus GN=Aarsd1 PE=1 SV=2 - [AASD1_MOUSE]	0.00	0.21	0.06	0.09	0.11	0.49	0.52	0.38	0.43	0.36	0.40	0.47	0.38

Q9EQU5-2	Isoform 2 of Protein SET OS=Mus musculus GN=Set - [SET_MOUSE]	-0.34	0.25	0.59	-0.16	0.12	0.48	0.82	0.54	0.88	0.06	0.08	0.14	0.03
Q810V0	U3 small nucleolar ribonucleoprotein protein MPP10 OS=Mus musculus GN=Mphosph10 PE=1 SV=2 - [MPP10_MOUSE]	-0.12	0.63	0.75	0.21	0.33	0.48	0.59	0.41	0.56	0.35	0.47	-0.23	0.14
Q8VEE1	LIM and cysteine-rich domains protein 1 OS=Mus musculus GN=Lmcd1 PE=1 SV=1 - [LMCD1_MOUSE]	-0.01	-0.28	-0.10	-0.09	-0.11	0.48	0.38	0.47	0.46	0.27	0.31	0.34	0.21
E9Q0V6	NHS-like protein 2 OS=Mus musculus GN=Nhsl2 PE=2 SV=1 - [E9Q0V6_MOUSE]	0.09	-0.03	-0.08	0.05	0.05	0.48	0.50	0.36	0.45	0.49	0.46	0.50	0.44
P28653	Biglycan OS=Mus musculus GN=Bgn PE=2 SV=1 - [PGS1_MOUSE]	0.00	0.29	0.29	0.13	0.06	0.48	0.38	0.43	0.42	0.70	0.64	0.40	0.24
Q60778	NF-kappa-B inhibitor beta OS=Mus musculus GN=Nfkbib PE=1 SV=2 - [IKBB_MOUSE]	-0.15	0.07	0.22	0.01	0.16	0.48	0.62	0.46	0.61	0.33	0.49	0.26	0.42
A2AL12	Heterogeneous nuclear ribonucleoprotein A3 OS=Mus musculus GN=Hnrnpa3 PE=2 SV=1 - [A2AL12_MOUSE]	-0.27	-0.45	-0.15	-0.08	0.19	0.48	0.75	0.51	0.84	0.29	0.59	-0.15	0.14
F8WIX8	Histone H2A OS=Mus musculus GN=Hist1h2al PE=2 SV=1 - [F8WIX8_MOUSE]	0.04	-0.27	-0.43	-0.08	-0.07	0.48	0.48	0.52	0.44	0.37	0.39	0.28	0.33
P23506	Protein-L-isoaspartate(D-aspartate) O-methyltransferase OS=Mus musculus GN=Pcmt1 PE=1 SV=3 - [PIMT_MOUSE]	-0.07	-0.33	-0.20	-0.05	0.06	0.48	0.50	0.44	0.47	0.32	0.36	0.18	0.29
P49919-2	Isoform KIP2b of Cyclin-dependent kinase inhibitor 1C OS=Mus musculus GN=Cdkn1c - [CDN1C_MOUSE]	0.06	0.20	0.14	0.04	-0.02	0.47	0.41	0.54	0.48	0.42	0.36	0.33	0.28
Q8BKZ9	Pyruvate dehydrogenase protein X component, mitochondrial OS=Mus musculus GN=Pdhx PE=2 SV=1 - [ODPX_MOUSE]	-0.08	-0.03	0.15	-0.01	0.03	0.46	0.51	0.56	0.62	0.43	0.47	0.25	0.28
Q9ERR1-2	Isoform 2 of Nuclear distribution protein nudE-like 1 OS=Mus musculus GN=Ndel1 - [NDEL1_MOUSE]	0.04	0.02	-0.03	-0.06	-0.10	0.46	0.37	0.59	0.52	0.37	0.27	0.35	0.31
Q8BGZ4-2	Isoform 2 of Cell division cycle protein 23 homolog OS=Mus musculus GN=Cdc23 - [CDC23_MOUSE]	0.05	-0.62	-0.67	-0.21	-0.26	0.46	0.40	0.41	0.35	0.31	0.26	0.05	0.00
Q9CQ25	Mitotic-spindle organizing protein 2 OS=Mus musculus GN=Mzt2 PE=1 SV=1 - [MZT2_MOUSE]	0.02	0.00	-0.03	0.06	0.04	0.46	0.43	0.52	0.49	0.43	0.40	0.17	0.14
P42866-13	Isoform 13 of Mu-type opioid receptor OS=Mus musculus GN=Oprm1 - [OPRM_MOUSE]	-0.31	0.04	0.35	0.04	0.34	0.45	0.75	0.53	0.83	0.16	0.47	0.26	0.57
P38647	Stress-70 protein, mitochondrial OS=Mus musculus GN=Hspa9 PE=1 SV=3 - [GRP75_MOUSE]	-0.10	-0.18	-0.06	-0.15	-0.01	0.45	0.55	0.49	0.57	0.32	0.43	0.14	0.24
Q9R257	Heme-binding protein 1 OS=Mus musculus GN=Hebp1 PE=1 SV=2 - [HEBP1_MOUSE]	-0.01	-0.24	-0.29	-0.03	0.02	0.45	0.51	0.54	0.58	0.49	0.49	0.40	0.43
Q62414	Neurogenic differentiation factor 2 OS=Mus musculus GN=Neurod2 PE=1 SV=3 - [NDF2_MOUSE]	0.01	0.81	0.79	0.05	0.03	0.44	0.43	0.42	0.40	0.15	0.14	0.29	0.28

Q05BE0	Zc4h2 protein OS=Mus musculus GN=Zc4h2 PE=2 SV=1 - [Q05BE0_MOUSE]	-0.23	0.00	0.28	0.06	-0.18	0.44	0.54	0.33	0.57	0.28	0.28	0.14	0.32
Q9QZ59-2	Isoform 2 of Doublesex- and mab-3-related transcription factor 1 OS=Mus musculus GN=Dmrt1 - [DMRT1_MOUSE]	0.05	0.04	-0.01	-0.73	-0.78	0.44	0.38	0.42	0.36	0.21	0.16	0.69	0.64
Q91VC7	Protein phosphatase 1 regulatory subunit 14A OS=Mus musculus GN=Ppp1r14a PE=2 SV=1 - [PP14A_MOUSE]	-0.02	-0.10	0.00	-0.12	-0.02	0.44	0.47	0.39	0.34	0.21	0.26	0.12	0.05
Q9WUR9	GTP:AMP phosphotransferase AK4, mitochondrial OS=Mus musculus GN=Ak4 PE=2 SV=1 - [KAD4_MOUSE]	0.02	0.39	0.27	0.25	0.27	0.44	0.43	0.41	0.33	0.54	0.61	0.35	0.34
Q91W92	Cdc42 effector protein 1 OS=Mus musculus GN=Cdc42ep1 PE=1 SV=1 - [BORG5_MOUSE]	0.03	-0.21	-0.24	-0.08	-0.11	0.44	0.40	0.40	0.36	0.20	0.17	-0.01	-0.03
Q9D0T1	NHP2-like protein 1 OS=Mus musculus GN=Nhp2l1 PE=2 SV=4 - [NH2L1_MOUSE]	0.00	0.00	0.06	0.04	0.03	0.44	0.38	0.41	0.44	0.31	0.38	0.31	0.37
O35685	Nuclear migration protein nudC OS=Mus musculus GN=Nudc PE=1 SV=1 - [NUDC_MOUSE]	-0.03	-0.31	-0.24	-0.11	-0.06	0.43	0.42	0.45	0.42	0.30	0.31	0.14	0.17
E9PZD2	Protein Micall2 OS=Mus musculus GN=Micall2 PE=2 SV=1 - [E9PZD2_MOUSE]	-0.10	0.07	0.16	-0.07	0.03	0.43	0.52	0.47	0.56	0.51	0.61	0.64	0.74
P0C0S6	Histone H2A.Z OS=Mus musculus GN=H2afz PE=1 SV=2 - [H2AZ_MOUSE]	-0.04	-0.22	-0.21	-0.05	-0.23	0.43	0.42	0.53	0.51	0.26	0.28	-0.05	-0.05
Q64152	Transcription factor BTF3 OS=Mus musculus GN=Btf3 PE=2 SV=3 - [BTF3_MOUSE]	-0.02	-0.32	-0.19	-0.10	-0.13	0.43	0.37	0.51	0.34	0.35	0.15	0.06	-0.02
Q8BP48	Methionine aminopeptidase 1 OS=Mus musculus GN=Metap1 PE=2 SV=1 - [MAP11_MOUSE]	0.00	0.03	0.03	0.17	0.18	0.43	0.42	1.03	1.03	0.95	0.95	0.88	0.88
Q9Z2I2	Peptidyl-prolyl cis-trans isomerase FKBP1B OS=Mus musculus GN=Fkbp1b PE=1 SV=3 - [FKB1B_MOUSE]	-0.06	-0.16	-0.11	-0.06	-0.08	0.43	0.47	0.33	0.48	0.17	0.23	-0.07	0.03
Q3V1F8	Epoxide hydrolase 3 OS=Mus musculus GN=Ephx3 PE=2 SV=2 - [EPHX3_MOUSE]	-0.14	0.21	0.35	-0.21	-0.07	0.43	0.56	0.41	0.55	0.27	0.41	-0.32	-0.17
Q8CI70	Leucine-rich repeat-containing protein 20 OS=Mus musculus GN=Lrrc20 PE=2 SV=1 - [LRC20_MOUSE]	0.02	0.53	0.51	0.21	0.19	0.42	0.40	0.42	0.39	0.34	0.32	0.24	0.22
Q8CCF0-2	Isoform 2 of U4/U6 small nuclear ribonucleoprotein Prp31 OS=Mus musculus GN=Prpf31 - [PRP31_MOUSE]	-0.16	0.63	0.79	0.15	0.31	0.42	0.67	0.50	0.75	0.29	0.54	0.26	0.52
Q61152	Tyrosine-protein phosphatase non-receptor type 18 OS=Mus musculus GN=Ptpn18 PE=1 SV=1 - [PTN18_MOUSE]	0.00	0.66	0.66	0.15	0.15	0.42	0.42	0.62	0.61	0.55	0.55	2.38	2.38
Q6PAL7	AT-hook DNA-binding motif-containing protein 1 OS=Mus musculus GN=Ahdc1 PE=1 SV=1 - [AHDC1_MOUSE]	0.00	0.25	0.25	0.04	0.04	0.42	0.41	0.36	0.36	0.25	0.25	0.47	0.47
Q9CY50	Translocon-associated protein subunit alpha OS=Mus musculus GN=Ssr1 PE=1 SV=1 - [SSRA_MOUSE]	-0.08	0.09	0.13	0.18	0.29	0.41	0.52	0.36	0.47	0.21	0.34	0.04	0.12

Q9CW07	Protein phosphatase 1 regulatory subunit 3G OS=Mus musculus GN=Ppp1r3g PE=2 SV=2 - [PP13G_MOUSE]	-0.22	0.02	0.24	0.05	0.27	0.41	0.63	0.42	0.63	0.18	0.40	0.05	0.27
Q9CW46	Ribonucleoprotein PTB-binding 1 OS=Mus musculus GN=Raver1 PE=1 SV=2 - [RAVR1_MOUSE]	0.03	0.07	0.11	-0.09	-0.05	0.41	0.46	0.60	0.44	0.44	0.50	0.58	0.56
L7N209	HBS1-like protein OS=Mus musculus GN=Hbs1l PE=4 SV=1 - [L7N209_MOUSE]	-0.13	0.40	0.53	-0.06	0.07	0.41	0.53	0.60	0.72	0.45	0.59	0.33	0.46
E9PV59	Protein Prr24 OS=Mus musculus GN=Prr24 PE=4 SV=1 - [E9PV59_MOUSE]	0.05	0.09	0.04	0.02	-0.03	0.41	0.36	0.50	0.45	0.47	0.43	0.23	0.19
Q7TSF4	Leucine-rich repeat-containing protein FAM211A OS=Mus musculus GN=Fam211a PE=1 SV=1 - [F211A_MOUSE]	-0.23	0.37	0.71	-0.04	0.18	0.41	0.61	0.45	0.95	0.19	0.47	0.28	0.51
P35459	Lymphocyte antigen 6D OS=Mus musculus GN=Ly6d PE=1 SV=1 - [LY6D_MOUSE]	-0.21	0.23	0.43	-0.19	0.02	0.41	0.61	0.33	0.53	0.23	0.44	0.10	0.32
Q3V0Q7	Transmembrane protease serine 12 OS=Mus musculus GN=Tmprss12 PE=2 SV=1 - [TMPSC_MOUSE]	-0.36	-0.18	0.17	-0.60	-0.24	0.41	0.76	0.31	0.66	0.02	0.38	-0.64	-0.27
Q8BH43	Wiskott-Aldrich syndrome protein family member 2 OS=Mus musculus GN=Wasf2 PE=1 SV=1 - [WASF2_MOUSE]	-0.11	0.12	0.09	-0.16	-0.16	0.41	0.46	0.49	0.43	0.39	0.50	0.25	0.39
Q67FY2-2	Isoform 2 of B-cell CLL/lymphoma 9-like protein OS=Mus musculus GN=Bcl9l - [BCL9L_MOUSE]	-0.02	0.35	0.37	-0.20	-0.18	0.41	0.42	0.31	0.33	0.23	0.25	0.22	0.24
Q8VE88-2	Isoform 2 of Protein FAM114A2 OS=Mus musculus GN=Fam114a2 - [F1142_MOUSE]	-0.09	-0.35	-0.23	-0.05	-0.01	0.41	0.48	0.42	0.45	0.36	0.44	0.13	0.13
E0CYH0	MCG16685, isoform CRA_d OS=Mus musculus GN=Wtap PE=4 SV=1 - [E0CYH0_MOUSE]	-0.10	-0.18	-0.05	-0.23	-0.14	0.40	0.50	0.34	0.45	0.23	0.40	-0.03	0.10
Q68ED7	CREB-regulated transcription coactivator 1 OS=Mus musculus GN=Crtc1 PE=2 SV=1 - [CRTC1_MOUSE]	-0.03	0.03	0.05	-0.13	-0.12	0.40	0.43	0.36	0.38	0.31	0.23	0.15	0.18
Q6A068	Cell division cycle 5-like protein OS=Mus musculus GN=Cdc5l PE=1 SV=2 - [CDC5L_MOUSE]	-0.11	-0.14	-0.02	0.05	0.07	0.40	0.52	0.46	0.53	0.24	0.37	0.10	0.20
A2AJ72	MCG130458 OS=Mus musculus GN=Fubp3 PE=4 SV=1 - [A2AJ72_MOUSE]	-0.10	-0.18	-0.08	-0.16	-0.06	0.40	0.49	0.34	0.43	0.14	0.24	0.08	0.18
Q9Z1P6	NADH dehydrogenase [ubiquinone] 1 alpha subcomplex subunit 7 OS=Mus musculus GN=Ndufa7 PE=1 SV=3 - [NDUA7_MOUSE]	0.00	-0.28	-0.29	-0.12	-0.11	0.40	0.38	0.42	0.38	0.22	0.24	0.20	0.24
Q6ZWR6-2	Isoform 2 of Nesprin-1 OS=Mus musculus GN=Syne1 - [SYNE1_MOUSE]	-0.04	0.28	0.31	-0.03	0.00	0.39	0.42	0.33	0.35	-0.13	-0.10	0.01	0.05
Q8R409	Protein HEXIM1 OS=Mus musculus GN=Hexim1 PE=1 SV=1 - [HEXI1_MOUSE]	-0.01	0.04	0.06	0.08	0.02	0.39	0.36	0.46	0.50	0.38	0.41	0.30	0.23
P60879-2	Isoform 2 of Synaptosomal-associated protein 25 OS=Mus musculus GN=Snap25 - [SNP25_MOUSE]	-0.16	-0.22	0.02	0.00	0.20	0.38	0.57	0.40	0.57	0.41	0.64	0.22	0.45

Q9QXS6	Drebrin OS=Mus musculus GN=Dbn1 PE=1 SV=4 - [DREB_MOUSE]	0.02	-0.13	-0.12	-0.06	-0.08	0.38	0.39	0.41	0.41	0.25	0.23	0.16	0.15
P30681	High mobility group protein B2 OS=Mus musculus GN=Hmgb2 PE=1 SV=3 - [HMGB2_MOUSE]	-0.03	-0.26	-0.20	0.17	0.20	0.38	0.38	0.41	0.40	0.34	0.39	0.11	0.17
Q3V113	Protein Pygo2 OS=Mus musculus GN=Pygo2 PE=2 SV=1 - [Q3V113_MOUSE]	-0.05	0.00	0.05	0.02	0.07	0.38	0.42	0.48	0.52	0.12	0.17	0.19	0.25
D3Z6H3	Dynactin 6, isoform CRA_b OS=Mus musculus GN=Dctn6 PE=4 SV=1 - [D3Z6H3_MOUSE]	-0.11	-0.37	-0.11	-0.19	-0.01	0.38	0.49	0.42	0.56	0.22	0.40	0.15	0.23
E9Q3G8	Protein Nup153 OS=Mus musculus GN=Nup153 PE=2 SV=1 - [E9Q3G8_MOUSE]	-0.02	-0.06	-0.03	-0.02	-0.01	0.38	0.46	0.45	0.50	0.32	0.36	0.13	0.25
O08600	Endonuclease G, mitochondrial OS=Mus musculus GN=Endog PE=2 SV=1 - [NUCG_MOUSE]	-0.10	0.16	0.26	0.22	0.31	0.37	0.46	0.37	0.47	0.47	0.57	0.44	0.54
Q8CB77	Transcription elongation factor B polypeptide 3 OS=Mus musculus GN=Tceb3 PE=1 SV=3 - [ELOA1_MOUSE]	0.08	-0.01	-0.12	-0.08	-0.11	0.37	0.37	0.38	0.32	0.23	0.15	0.25	0.18
Q8CEG5	Coiled-coil domain-containing protein 28B OS=Mus musculus GN=Ccdc28b PE=2 SV=3 - [CC28B_MOUSE]	-0.28	-0.28	0.13	-0.17	-0.16	0.37	0.68	0.49	0.77	0.01	0.30	-0.01	0.27
M0QWU0	CXXC-type zinc finger protein 4 OS=Mus musculus GN=Cxxc4 PE=4 SV=1 - [M0QWU0_MOUSE]	-0.02	0.08	0.09	0.06	0.08	0.37	0.38	0.81	0.83	0.70	0.72	0.55	0.57
E9PZ30	Protein Ugt2b34 OS=Mus musculus GN=Ugt2b34 PE=2 SV=1 - [E9PZ30_MOUSE]	-0.50	-0.99	-0.78	-0.59	-0.10	0.36	0.86	0.73	1.22	0.70	0.92	-0.55	-0.05
Q9QX66-3	Isoform 3 of Zinc finger protein neuro-d4 OS=Mus musculus GN=Dpf1 - [DPF1_MOUSE]	0.05	0.04	-0.06	0.13	0.11	0.36	0.40	0.42	0.36	0.35	0.26	0.37	0.27
Q571K4	TGF-beta-activated kinase 1 and MAP3K7-binding protein 3 OS=Mus musculus GN=Tab3 PE=1 SV=2 - [TAB3_MOUSE]	-0.13	-0.10	0.02	-0.16	0.01	0.36	0.43	0.44	0.58	0.30	0.38	0.16	0.27
P57784	U2 small nuclear ribonucleoprotein A' OS=Mus musculus GN=Snrpa1 PE=1 SV=2 - [RU2A_MOUSE]	-0.05	-0.08	-0.09	-0.01	0.05	0.36	0.39	0.40	0.37	0.29	0.33	0.18	0.17
Q8BHT6-2	Isoform 2 of Beta-1,3-glucosyltransferase OS=Mus musculus GN=B3galtl - [B3GLT_MOUSE]	-0.32	0.47	0.78	-0.04	0.27	0.36	0.67	0.49	0.80	0.25	0.57	0.13	0.45
Q91YN9	BAG family molecular chaperone regulator 2 OS=Mus musculus GN=Bag2 PE=1 SV=1 - [BAG2_MOUSE]	-0.08	0.10	0.22	-0.16	-0.05	0.36	0.44	0.47	0.62	0.33	0.43	0.26	0.47
Q6PGL7	WASH complex subunit FAM21 OS=Mus musculus GN=Fam21 PE=1 SV=1 - [FAM21_MOUSE]	0.01	-0.21	-0.22	-0.15	-0.19	0.36	0.36	0.36	0.34	0.21	0.20	0.04	0.06
Q8R1B0	SH3 and cysteine-rich domain-containing protein 2 OS=Mus musculus GN=Stac2 PE=2 SV=1 - [STAC2_MOUSE]	-0.08	-0.04	-0.02	0.04	0.10	0.36	0.40	0.43	0.44	0.06	0.14	-0.24	-0.19

Q9CQA6	Coiled-coil-helix-coiled-coil-helix domain-containing protein 1 OS=Mus musculus GN=Chchd1 PE=2 SV=1 - [CHCH1_MOUSE]	-0.10	-0.77	-0.54	-0.27	-0.17	0.35	0.48	0.39	0.45	0.21	0.29	0.09	0.15
O08599-2	Isoform 2 of Syntaxin-binding protein 1 OS=Mus musculus GN=Stxbp1 - [STXB1_MOUSE]	-0.14	0.56	0.69	0.20	0.33	0.35	0.49	0.45	0.58	0.21	0.35	0.15	0.29
Q8K3X4	Interferon regulatory factor 2-binding protein-like OS=Mus musculus GN=Irf2bpl PE=1 SV=1 - [I2BPL_MOUSE]	-0.11	-0.10	0.00	-0.10	-0.05	0.35	0.40	0.42	0.43	0.29	0.37	0.20	0.30
O54786	DNA fragmentation factor subunit alpha OS=Mus musculus GN=Dffa PE=1 SV=2 - [DFFA_MOUSE]	-0.19	0.54	0.72	-0.20	-0.01	0.35	0.53	0.62	0.81	0.19	0.38	0.06	0.25
Q2M3X8	Phosphatase and actin regulator 1 OS=Mus musculus GN=Phactr1 PE=1 SV=1 - [PHAR1_MOUSE]	0.08	0.10	0.07	0.02	0.00	0.35	0.38	0.38	0.39	0.28	0.26	0.41	0.38
P17742	Peptidyl-prolyl cis-trans isomerase A OS=Mus musculus GN=Ppia PE=1 SV=2 - [PPIA_MOUSE]	-0.08	-0.35	-0.14	-0.23	-0.09	0.35	0.42	0.46	0.47	0.22	0.28	0.14	0.30
O54791	Transcription factor MafF OS=Mus musculus GN=Maff PE=2 SV=1 - [MAFF_MOUSE]	-0.04	-0.08	-0.05	-0.23	-0.19	0.35	0.38	0.54	0.58	0.29	0.33	0.14	0.19
Q6GQT6	Sterol regulatory element-binding protein cleavage-activating protein OS=Mus musculus GN=Scap PE=1 SV=1 - [SCAP_MOUSE]	-0.22	0.49	0.70	0.04	0.26	-0.05	0.15	-0.58	-0.37	-0.84	-0.63	-0.76	-0.54
E9QP46	Nesprin-2 OS=Mus musculus GN=Syne2 PE=2 SV=1 - [E9QP46_MOUSE]	0.30	0.43	0.13	0.21	-0.09	-0.68	-0.98	-0.27	-0.57	-0.80	-1.10	-0.94	-1.23
Q9Z2Y3	Homer protein homolog 1 OS=Mus musculus GN=Homer1 PE=1 SV=2 - [HOME1_MOUSE]	-0.20	-0.17	0.00	-0.24	-0.04	-0.54	-0.39	-0.44	-0.23	-0.71	-0.53	-0.71	-0.50
Q8R4V2	Dual specificity protein phosphatase 15 OS=Mus musculus GN=Dusp15 PE=2 SV=3 - [DUS15_MOUSE]	-0.05	0.02	0.06	-0.17	-0.12	-0.38	-0.33	-0.52	-0.47	-0.70	-0.65	-0.58	-0.52
Q9CQI3	Glia maturation factor beta OS=Mus musculus GN=Gmfb PE=1 SV=3 - [GMFB_MOUSE]	-0.05	-0.31	-0.25	-0.28	-0.16	-0.50	-0.42	-0.41	-0.35	-0.65	-0.52	-0.53	-0.47
B1AU22	Fibroblast growth factor 13 (Fragment) OS=Mus musculus GN=Fgf13 PE=2 SV=1 - [B1AU22_MOUSE]	0.16	0.60	0.44	-0.10	-0.26	-0.43	-0.59	-0.27	-0.43	-0.65	-0.80	-0.83	-0.99
G5E8T9	Hydroxyacyl glutathione hydrolase OS=Mus musculus GN=Hagh PE=3 SV=1 - [G5E8T9_MOUSE]	-0.11	-0.30	-0.28	-0.28	-0.17	-0.62	-0.53	-0.39	-0.28	-0.63	-0.53	-0.56	-0.47
O09061	Proteasome subunit beta type-1 OS=Mus musculus GN=Psmb1 PE=1 SV=1 - [PSB1_MOUSE]	-0.27	0.23	0.47	-0.04	0.28	-0.56	-0.27	-0.60	-0.38	-0.62	-0.35	-0.63	-0.35
Q8K2P6	Rieske domain-containing protein OS=Mus musculus GN=Rfesd PE=1 SV=1 - [RFESD_MOUSE]	0.02	-0.34	-0.36	-0.20	-0.22	-0.33	-0.35	-0.21	-0.24	-0.61	-0.63	-0.50	-0.51
Q07113	Cation-independent mannose-6-phosphate receptor OS=Mus musculus GN=Igf2r PE=1 SV=1 - [MPRI_MOUSE]	-0.20	-0.67	-0.46	-0.30	-0.10	-0.57	-0.38	-0.50	-0.30	-0.61	-0.40	-0.67	-0.46

Q8BFS6	Calcineurin-like phosphoesterase domain-containing protein 1 OS=Mus musculus GN=Cpped1 PE=2 SV=1 - [CPPED_MOUSE]	0.00	-0.32	-0.31	-0.09	-0.08	-0.31	-0.32	-0.44	-0.49	-0.60	-0.64	-0.47	-0.47
Q9R1E6	Ectonucleotide pyrophosphatase/phosphodiesterase family member 2 OS=Mus musculus GN=Enpp2 PE=1 SV=3 - [ENPP2_MOUSE]	-0.07	-0.24	-0.17	0.11	0.18	-0.24	-0.18	-0.70	-0.63	-0.59	-0.52	-0.86	-0.78
Q9Z255	Ubiquitin-conjugating enzyme E2 A OS=Mus musculus GN=Ube2a PE=2 SV=1 - [UBE2A_MOUSE]	0.03	-0.12	-0.16	-0.26	-0.29	-0.36	-0.36	-0.41	-0.55	-0.58	-0.59	-0.56	-0.55
Q80UW8	DNA-directed RNA polymerases I, II, and III subunit RPABC1 OS=Mus musculus GN=Polr2e PE=2 SV=1 - [RPAB1_MOUSE]	-0.06	-0.31	-0.23	-0.20	-0.16	-0.42	-0.43	-0.27	-0.17	-0.57	-0.47	-0.56	-0.38
Q922Q1	MOSC domain-containing protein 2, mitochondrial OS=Mus musculus GN=Marc2 PE=1 SV=1 - [MOSC2_MOUSE]	-0.08	-0.51	-0.26	-0.28	-0.14	-0.40	-0.29	-0.28	-0.33	-0.57	-0.51	-0.56	-0.49
Q9QY93	dCTP pyrophosphatase 1 OS=Mus musculus GN=Dctpp1 PE=1 SV=1 - [DCTP1_MOUSE]	0.16	0.74	0.58	0.30	0.14	-0.13	-0.29	-0.23	-0.40	-0.55	-0.71	-0.45	-0.60
E9QA19	CCR4-NOT transcription complex subunit 4 OS=Mus musculus GN=Cnot4 PE=2 SV=1 - [E9QA19_MOUSE]	0.07	-0.17	-0.21	0.03	-0.05	-0.60	-0.60	-0.41	-0.33	-0.54	-0.43	-0.49	-0.43
Q8BR65	Sin3 histone deacetylase corepressor complex component SDS3 OS=Mus musculus GN=Suds3 PE=1 SV=1 - [SDS3_MOUSE]	-0.17	-0.38	-0.21	-0.23	-0.06	-0.46	-0.30	-0.69	-0.52	-0.53	-0.35	-0.50	-0.33
Q5DTY9	BTB/POZ domain-containing protein KCTD16 OS=Mus musculus GN=Kctd16 PE=1 SV=2 - [KCD16_MOUSE]	-0.08	-0.07	0.02	-0.01	0.05	-0.42	-0.38	-0.50	-0.43	-0.51	-0.44	-0.53	-0.41
E0CZ78	Serine/threonine-protein phosphatase OS=Mus musculus GN=Ppp3cb PE=2 SV=1 - [E0CZ78_MOUSE]	-0.08	0.10	0.20	-0.05	0.05	-0.46	-0.35	-0.48	-0.42	-0.50	-0.44	-0.40	-0.31
Q9CT10	Ran-binding protein 3 OS=Mus musculus GN=Ranbp3 PE=1 SV=2 - [RANB3_MOUSE]	0.01	-0.16	-0.19	-0.16	-0.17	-0.47	-0.37	-0.29	-0.32	-0.50	-0.53	-0.56	-0.50
Q9DC53	Copine-8 OS=Mus musculus GN=Cpne8 PE=2 SV=2 - [CPNE8_MOUSE]	0.09	-0.16	-0.08	0.30	0.10	-0.09	-0.13	-0.16	-0.25	-0.49	-0.53	-0.33	-0.37
Q58A65-2	Isoform 2 of C-Jun-amino-terminal kinase-interacting protein 4 OS=Mus musculus GN=Spag9 - [JIP4_MOUSE]	-0.06	-0.18	-0.10	-0.13	-0.05	-0.44	-0.37	-0.46	-0.35	-0.49	-0.40	-0.47	-0.40
Q69ZF7	Metal transporter CNNM4 OS=Mus musculus GN=Cnnm4 PE=1 SV=2 - [CNNM4_MOUSE]	-0.12	-0.16	-0.05	-0.48	-0.36	-0.26	-0.14	-0.85	-0.73	-0.48	-0.36	-0.47	-0.35
Q8VIJ6	Splicing factor, proline- and glutamine-rich OS=Mus musculus GN=Sfpq PE=1 SV=1 - [SFPQ_MOUSE]	-0.05	-0.23	-0.15	-0.15	-0.11	-0.34	-0.28	-0.25	-0.21	-0.48	-0.38	-0.45	-0.36
Q99MV7-3	Isoform 3 of RING finger protein 17 OS=Mus musculus GN=Rnf17 - [RNF17_MOUSE]	0.25	3.30	3.05	0.38	0.13	0.35	0.09	-0.22	-0.47	-0.48	-0.73	-1.05	-1.29

Q9CQE6	Histone chaperone ASF1A OS=Mus musculus GN=Asf1a PE=2 SV=1 - [ASF1A_MOUSE]	-0.08	-0.59	-0.51	-0.27	-0.18	-0.41	-0.33	-0.47	-0.39	-0.48	-0.39	-0.51	-0.42
Q93092	Transaldolase OS=Mus musculus GN=Taldo1 PE=1 SV=2 - [TALDO_MOUSE]	-0.08	-0.21	-0.01	-0.12	-0.02	-0.49	-0.35	-0.54	-0.45	-0.48	-0.37	-0.51	-0.38
Q80V26	Inositol monophosphatase 3 OS=Mus musculus GN=Impad1 PE=1 SV=1 - [IMPA3_MOUSE]	-0.06	-0.36	-0.31	-0.09	-0.03	-0.47	-0.42	-0.41	-0.35	-0.47	-0.42	-0.54	-0.48
Q64444	Carbonic anhydrase 4 OS=Mus musculus GN=Ca4 PE=1 SV=1 - [CAH4_MOUSE]	-0.03	-0.19	-0.11	-0.04	-0.03	-0.41	-0.34	-0.31	-0.29	-0.47	-0.43	-0.47	-0.37
Q8BX09-2	Isoform 2 of Retinoblastoma-binding protein 5 OS=Mus musculus GN=Rbbp5 - [RBBP5_MOUSE]	0.42	-0.17	-0.60	-0.12	-0.55	-0.49	-0.92	0.07	-0.36	-0.46	-0.88	-0.35	-0.77
Q9CQF4	Uncharacterized protein C6orf203 homolog OS=Mus musculus PE=1 SV=1 - [CF203_MOUSE]	0.04	-0.26	-0.38	-0.23	-0.31	-0.22	-0.36	-0.31	-0.34	-0.44	-0.63	-0.51	-0.61
P70362	Ubiquitin fusion degradation protein 1 homolog OS=Mus musculus GN=Ufd1l PE=1 SV=2 - [UFD1_MOUSE]	0.13	-0.40	-0.36	0.02	0.12	-0.28	-0.29	-0.35	-0.20	-0.43	-0.47	-0.47	-0.50
Q8R0F5	RNA-binding motif protein, X-linked 2 OS=Mus musculus GN=Rbmx2 PE=1 SV=1 - [RBMX2_MOUSE]	0.02	-0.23	-0.25	-0.31	-0.33	-0.12	-0.14	-0.13	-0.15	-0.43	-0.45	-0.42	-0.43
Q3UHN9	Bifunctional heparan sulfate N-deacetylase/N-sulfotransferase 1 OS=Mus musculus GN=Ndst1 PE=1 SV=2 - [NDST1_MOUSE]	-0.04	0.00	0.04	-0.62	-0.58	-0.06	-0.02	0.01	0.05	-0.43	-0.38	-0.44	-0.40
P47708	Rabphilin-3A OS=Mus musculus GN=Rph3a PE=1 SV=2 - [RP3A_MOUSE]	0.04	-0.06	-0.14	-0.05	-0.10	-0.36	-0.36	-0.29	-0.35	-0.42	-0.43	-0.50	-0.53
Q64487-5	Isoform E of Receptor-type tyrosine-protein phosphatase delta OS=Mus musculus GN=Ptprd - [PTPRD_MOUSE]	0.07	0.20	0.13	0.01	-0.17	-0.18	-0.27	-0.19	-0.26	-0.40	-0.43	-0.42	-0.47
Q921M3	Splicing factor 3B subunit 3 OS=Mus musculus GN=Sf3b3 PE=2 SV=1 - [SF3B3_MOUSE]	0.05	0.03	-0.01	-0.05	-0.11	-0.34	-0.40	-0.36	-0.38	-0.39	-0.48	-0.32	-0.37
Q3TJ22	Angio-associated migratory protein OS=Mus musculus GN=Aamp PE=2 SV=1 - [Q3TJ22_MOUSE]	-0.02	0.01	0.02	-0.11	-0.09	-0.36	-0.35	-0.38	-0.37	-0.38	-0.36	-0.34	-0.31
P01756	Ig heavy chain V region MOPC 104E OS=Mus musculus PE=1 SV=1 - [HVM12_MOUSE]	0.17	-0.50	-0.67	-0.02	-0.19	0.32	0.15	-0.63	-0.80	-0.35	-0.51	-0.99	-1.15
D3Z7R4	Synaptotagmin-1 (Fragment) OS=Mus musculus GN=Syt1 PE=2 SV=1 - [D3Z7R4_MOUSE]	0.09	-0.63	-0.75	0.04	-0.07	0.05	-0.11	-0.09	-0.27	-0.34	-0.46	-0.41	-0.52
Q99JW2	Aminoacylase-1 OS=Mus musculus GN=Acy1 PE=1 SV=1 - [ACY1_MOUSE]	0.02	0.04	-0.08	0.05	0.10	-0.57	-0.57	-0.53	-0.36	-0.33	-0.42	-0.40	-0.40
Q9CQ40	39S ribosomal protein L49, mitochondrial OS=Mus musculus GN=Mrpl49 PE=2 SV=1 - [RM49_MOUSE]	-0.03	-0.32	-0.16	-0.12	-0.05	-0.26	-0.24	-0.11	-0.17	-0.33	-0.35	-0.30	-0.31
Q91WK1	SPRY domain-containing protein 4 OS=Mus musculus GN=Spryd4 PE=2 SV=1 - [SPRY4_MOUSE]	0.08	-0.25	-0.34	0.18	0.09	0.56	0.47	0.36	0.28	-0.31	-0.39	-0.30	-0.38

P45878	Peptidyl-prolyl cis-trans isomerase FKBP2 OS=Mus musculus GN=Fkbp2 PE=1 SV=1 - [FKBP2_MOUSE]	0.02	-0.40	-0.48	-0.12	-0.18	-0.20	-0.23	-0.17	-0.18	-0.30	-0.40	-0.46	-0.47
Q99KZ6	Zinc finger protein 639 OS=Mus musculus GN=Znf639 PE=2 SV=1 - [ZN639_MOUSE]	0.06	-0.40	-0.46	-0.10	-0.16	0.11	0.05	-0.17	-0.23	-0.30	-0.35	-0.52	-0.58
Q61646	Haptoglobin OS=Mus musculus GN=Hp PE=1 SV=1 - [HPT_MOUSE]	0.79	0.85	0.06	0.44	-0.35	0.08	-0.72	-1.16	-1.95	4.23	3.45	3.08	2.30
O70551	SRSF protein kinase 1 OS=Mus musculus GN=Srpk1 PE=1 SV=2 - [SRPK1_MOUSE]	-0.41	0.19	0.59	0.11	0.52	-0.16	0.24	3.08	3.49	2.66	3.06	0.95	1.35
E9PUE6	Transmembrane protein 145 OS=Mus musculus GN=Tmem145 PE=2 SV=1 - [E9PUE6_MOUSE]	-0.16	0.06	0.22	-0.06	0.10	0.19	0.34	1.98	2.13	1.71	1.87	0.73	0.90
P06728	Apolipoprotein A-IV OS=Mus musculus GN=Apoa4 PE=2 SV=3 - [APOA4_MOUSE]	0.01	0.35	0.32	-0.13	-0.10	0.26	0.25	0.22	0.30	1.49	1.54	0.48	0.51
P07309	Transthyretin OS=Mus musculus GN=Ttr PE=1 SV=1 - [TTHY_MOUSE]	0.80	0.86	0.06	0.95	0.15	1.38	0.57	1.00	0.20	1.47	0.68	1.25	0.46
Q80YF9	Rho GTPase-activating protein 33 OS=Mus musculus GN=Arhgap33 PE=1 SV=1 - [RHG33_MOUSE]	0.02	0.38	0.33	0.24	0.20	-0.20	-0.22	1.75	1.75	1.36	1.37	0.77	0.79
Q80T23-2	Isoform 2 of Synaptotagmin-like protein 5 OS=Mus musculus GN=Sytl5 - [SYTL5_MOUSE]	0.62	0.73	0.11	0.48	-0.14	0.57	-0.06	0.85	0.22	1.08	0.47	1.07	0.45
P33622	Apolipoprotein C-III OS=Mus musculus GN=Apoc3 PE=2 SV=2 - [APOC3_MOUSE]	-0.57	0.01	0.58	-0.85	-0.28	-0.55	0.01	-0.11	0.45	0.92	1.49	0.30	0.87
Q99M46	DNA-directed RNA polymerase II subunit RPB3 OS=Mus musculus GN=Polr2c PE=2 SV=1 - [Q99M46_MOUSE]	0.31	0.79	0.48	0.25	-0.06	0.64	0.33	0.90	0.59	0.76	0.46	0.95	0.65
Q9D3P8	Plasminogen receptor (KT) OS=Mus musculus GN=Plgrkt PE=1 SV=1 - [PLRKT_MOUSE]	0.41	-0.22	-0.64	0.52	0.11	0.64	0.22	0.67	0.25	0.73	0.32	0.71	0.29
Q9R060	Cytosolic Fe-S cluster assembly factor NUBP1 OS=Mus musculus GN=Nubp1 PE=1 SV=1 - [NUBP1_MOUSE]	0.25	0.39	0.13	-0.85	-1.11	0.28	0.02	0.09	-0.17	0.67	0.42	0.80	0.55
Q9D0V7	Receptor-binding cancer antigen expressed on SiSo cells OS=Mus musculus GN=Ebag9 PE=1 SV=2 - [RCAS1_MOUSE]	0.14	0.35	-0.03	0.40	0.21	0.67	0.40	0.44	0.30	0.67	0.55	0.55	0.34
Q6ZWQ9	MCG5400 OS=Mus musculus GN=Myl12a PE=2 SV=1 - [Q6ZWQ9_MOUSE]	0.28	0.58	0.39	0.21	-0.02	0.40	0.17	0.54	0.31	0.63	0.38	0.71	0.44
O88990	Alpha-actinin-3 OS=Mus musculus GN=Actn3 PE=2 SV=1 - [ACTN3_MOUSE]	0.02	0.48	0.45	0.02	0.00	0.08	0.04	0.57	0.54	0.62	0.60	0.64	0.62
Q80TJ7	Histone lysine demethylase PHF8 OS=Mus musculus GN=Phf8 PE=1 SV=2 - [PHF8_MOUSE]	0.21	0.40	0.18	0.25	0.03	0.21	-0.02	0.53	0.31	0.61	0.40	0.60	0.38
Q9QZC2	Plexin-C1 OS=Mus musculus GN=Plxnc1 PE=1 SV=1 - [PLXC1_MOUSE]	0.12	0.79	0.67	0.26	0.14	0.22	0.10	0.53	0.41	0.61	0.49	0.59	0.48
Q5SRX1-2	Isoform 2 of TOM1-like protein 2 OS=Mus musculus GN=Tom1l2 - [TM1L2_MOUSE]	-0.22	-0.29	-0.07	0.07	0.29	0.20	0.41	0.86	1.08	0.61	0.84	0.32	0.54

Q5SQM0	Echinoderm microtubule-associated protein-like 6 OS=Mus musculus GN=Eml6 PE=2 SV=1 - [EMAL6_MOUSE]	0.06	0.48	0.42	0.26	0.20	0.25	0.19	0.56	0.50	0.60	0.54	0.34	0.28
Q6IFZ6	Keratin, type II cytoskeletal 1b OS=Mus musculus GN=Krt77 PE=1 SV=1 - [K2C1B_MOUSE]	0.30	0.06	-0.24	1.57	1.26	-0.40	-0.71	0.39	0.08	0.59	0.29	0.76	0.46
O35711-4	Isoform 4 of Liprin-beta-2 OS=Mus musculus GN=Ppfibp2 - [LIPB2_MOUSE]	0.13	0.30	0.17	-0.06	-0.19	0.39	0.25	0.18	0.05	0.59	0.46	0.70	0.57
P12660	Purkinje cell protein 2 OS=Mus musculus GN=Pcp2 PE=2 SV=2 - [PCP2_MOUSE]	0.30	-0.04	-0.35	0.34	0.03	0.33	0.03	-0.13	-0.43	0.59	0.29	0.69	0.40
Q3US17	Zinc finger protein 48 OS=Mus musculus GN=Znf48 PE=2 SV=2 - [ZNF48_MOUSE]	0.11	0.35	0.23	0.20	0.08	0.40	0.27	0.40	0.28	0.57	0.46	0.43	0.32
Q9D2H6-2	Isoform 2 of Transcription factor Sp2 OS=Mus musculus GN=Sp2 - [SP2_MOUSE]	0.19	0.09	-0.10	0.32	0.13	0.59	0.39	0.49	0.30	0.57	0.38	0.53	0.34
P62965	Cellular retinoic acid-binding protein 1 OS=Mus musculus GN=Crabp1 PE=1 SV=2 - [RABP1_MOUSE]	0.21	0.16	-0.19	-0.22	-0.46	0.09	-0.18	-0.31	-0.44	0.56	0.41	1.13	0.89
Q9CY34	NEDD8-conjugating enzyme UBE2F OS=Mus musculus GN=Ube2f PE=1 SV=1 - [UB2FA_MOUSE]	0.24	-0.01	-0.25	0.01	-0.22	0.44	0.20	0.74	0.49	0.56	0.32	0.53	0.29
P12025	Midkine OS=Mus musculus GN=Mdk PE=2 SV=2 - [MK_MOUSE]	0.27	0.20	-0.08	0.03	-0.25	0.64	0.35	0.37	0.10	0.56	0.29	0.70	0.43
Q69ZP3-2	Isoform 2 of Probable hydrolase PNKD OS=Mus musculus GN=Pnkd - [PNKD_MOUSE]	-0.15	1.42	1.57	-0.26	-0.11	-0.14	0.01	0.59	0.73	0.56	0.71	0.91	1.06
E9PVZ0	GTP-binding protein REM 2 OS=Mus musculus GN=Rem2 PE=2 SV=1 - [E9PVZ0_MOUSE]	-0.11	-0.36	-0.26	0.45	0.56	0.21	0.31	-0.23	-0.13	0.54	0.66	0.71	0.83
Q4VA61	Down syndrome cell adhesion molecule-like protein 1 homolog OS=Mus musculus GN=Dscaml1 PE=1 SV=2 - [DSCL1_MOUSE]	-0.04	0.25	0.28	-0.20	-0.16	-0.11	-0.08	0.65	0.68	0.53	0.57	0.65	0.69
Q60857	Sodium-dependent serotonin transporter OS=Mus musculus GN=Slc6a4 PE=1 SV=4 - [SC6A4_MOUSE]	0.16	0.44	0.27	0.19	0.02	0.31	0.14	-0.06	-0.23	0.51	0.35	0.51	0.35
Q3USH1	Protein FAM196A OS=Mus musculus GN=Fam196a PE=2 SV=1 - [F196A_MOUSE]	0.00	-0.18	-0.18	-0.09	-0.14	0.57	0.33	0.56	0.52	0.51	0.45	0.61	0.50
Q9D0D5	General transcription factor IIE subunit 1 OS=Mus musculus GN=Gtf2e1 PE=2 SV=1 - [T2EA_MOUSE]	-0.07	0.18	0.25	-0.03	0.03	0.11	0.17	0.58	0.64	0.51	0.58	0.56	0.62
Q3U1D0-2	Isoform 2 of Protein Lines homolog OS=Mus musculus GN=Lins - [LINES_MOUSE]	-0.31	-0.05	0.25	0.20	0.50	0.34	0.64	0.59	0.89	0.51	0.82	0.32	0.63
D3YYW8	RNA-binding protein 42 OS=Mus musculus GN=Rbm42 PE=2 SV=1 - [D3YYW8_MOUSE]	-0.23	-0.04	0.19	-0.16	0.07	0.21	0.44	0.24	0.46	0.51	0.74	0.65	0.88
Q68FG3	Protein SPT2 homolog OS=Mus musculus GN=Spty2d1 PE=2 SV=1 - [SPT2_MOUSE]	0.06	0.34	0.28	0.21	0.15	0.39	0.33	0.62	0.56	0.50	0.45	0.41	0.36
Q0PHV7	Dapper homolog 3 OS=Mus musculus GN=Dact3 PE=1 SV=1 - [DACT3_MOUSE]	0.07	0.13	0.06	-0.09	-0.16	0.17	0.10	0.85	0.78	0.50	0.43	0.72	0.65
E9Q8M1	Protein Zfp358 OS=Mus musculus GN=Zfp358 PE=4 SV=1 - [E9Q8M1_MOUSE]	-0.40	0.14	0.54	0.04	-0.07	0.36	0.60	0.54	0.01	0.50	0.91	0.39	0.80

Q8BTG7	Protein NDRG4 OS=Mus musculus GN=Ndrg4 PE=1 SV=1 - [NDRG4_MOUSE]	0.10	0.12	0.01	0.00	-0.10	0.20	0.08	0.49	0.39	0.49	0.39	0.38	0.28
B7ZCA7	Kazrin (Fragment) OS=Mus musculus GN=Kazn PE=2 SV=1 - [B7ZCA7_MOUSE]	-0.22	0.16	0.38	-0.07	0.15	0.27	0.48	0.48	0.69	0.49	0.71	0.28	0.51
Q8C1S0	Mediator of RNA polymerase II transcription subunit 19 OS=Mus musculus GN=Med19 PE=1 SV=1 - [MED19_MOUSE]	0.10	-0.34	-0.45	0.29	0.19	0.36	0.24	0.56	0.45	0.48	0.38	0.50	0.40
Q2VPQ9	Chromatin modification-related protein MEAF6 OS=Mus musculus GN=Meaf6 PE=2 SV=1 - [EAF6_MOUSE]	0.08	0.28	0.20	0.12	0.04	0.24	0.18	0.35	0.26	0.48	0.34	0.36	0.29
Q91X84-2	Isoform 2 of CREB-regulated transcription coactivator 3 OS=Mus musculus GN=Crtc3 - [CRTC3_MOUSE]	0.09	0.09	0.00	0.07	-0.02	0.42	0.32	0.62	0.52	0.47	0.38	0.74	0.65
Q6NXI6-2	Isoform 2 of Regulation of nuclear pre-mRNA domain-containing protein 2 OS=Mus musculus GN=Rprd2 - [RPRD2_MOUSE]	0.07	-0.05	-0.14	-0.03	-0.06	0.34	0.20	0.45	0.20	0.46	0.31	0.45	0.31
Q99PV8-3	Isoform 3 of B-cell lymphoma/leukemia 11B OS=Mus musculus GN=Bcl11b - [BC11B_MOUSE]	0.05	-0.07	-0.13	-0.11	-0.16	0.41	0.35	0.17	0.12	0.46	0.41	0.60	0.55
B1AZR7	Protein Pcdh11x OS=Mus musculus GN=Pcdh11x PE=2 SV=1 - [B1AZR7_MOUSE]	0.08	0.18	0.09	0.14	0.06	0.30	0.21	0.32	0.24	0.45	0.37	0.46	0.38
Q3ULJ3	MCG3697, isoform CRA_c OS=Mus musculus GN=Upf3a PE=2 SV=1 - [Q3ULJ3_MOUSE]	-0.01	0.32	0.32	0.13	0.13	0.34	0.34	0.42	0.42	0.45	0.45	0.51	0.52
O08972	Dimethylarginine dimethylaminohydrolase 2, isoform CRA_a OS=Mus musculus GN=Ddah2 PE=2 SV=1 - [O08972_MOUSE]	0.08	0.03	-0.06	0.00	-0.09	0.37	0.23	0.28	0.21	0.44	0.49	0.54	0.43
F8WGI9	Very low-density lipoprotein receptor OS=Mus musculus GN=Vldlr PE=2 SV=1 - [F8WGI9_MOUSE]	0.10	0.34	0.23	0.26	0.18	0.03	-0.02	0.72	0.61	0.44	0.60	0.55	0.70
Q64522	Histone H2A type 2-B OS=Mus musculus GN=Hist2h2ab PE=1 SV=3 - [H2A2B_MOUSE]	0.13	0.18	0.05	0.08	-0.05	0.47	0.34	0.59	0.45	0.44	0.31	0.55	0.43
Q6PIX5	Inactive rhomboid protein 1 OS=Mus musculus GN=Rhbdf1 PE=1 SV=2 - [RHDF1_MOUSE]	0.03	0.48	0.44	0.12	0.09	0.37	0.34	0.68	0.65	0.43	0.40	0.38	0.35
P35831	Tyrosine-protein phosphatase non-receptor type 12 OS=Mus musculus GN=Ptpn12 PE=1 SV=3 - [PTN12_MOUSE]	0.10	0.30	0.19	0.04	-0.06	0.40	0.29	0.35	0.24	0.42	0.31	0.38	0.28
A2AEE4	Proline/serine-rich coiled-coil protein 1 (Fragment) OS=Mus musculus GN=Psrc1 PE=2 SV=1 - [A2AEE4_MOUSE]	0.10	0.42	0.32	0.18	0.08	0.42	0.32	0.75	0.65	0.41	0.31	0.53	0.43
Q6RUT7	Protein CCSMST1 OS=Mus musculus GN=Ccsmst1 PE=3 SV=1 - [CSMT1_MOUSE]	-0.12	0.07	0.19	-0.07	0.04	0.55	0.66	0.23	0.34	0.41	0.53	0.33	0.45
E9PXB9	Mitochondrial import inner membrane translocase subunit Tim22 OS=Mus musculus GN=Timm22 PE=2 SV=1 - [E9PXB9_MOUSE]	0.11	0.14	0.03	0.24	0.13	0.20	0.08	0.19	0.08	0.40	0.29	0.39	0.28

Q9CXP8	Guanine nucleotide-binding protein G(I)/G(S)/G(O) subunit gamma-10 OS=Mus musculus GN=Gng10 PE=3 SV=1 - [GBG10_MOUSE]	0.01	0.21	0.19	0.12	0.10	0.48	0.46	0.33	0.31	0.39	0.38	0.52	0.51
Q2EMV9	Poly [ADP-ribose] polymerase 14 OS=Mus musculus GN=Parp14 PE=1 SV=3 - [PAR14_MOUSE]	0.10	-0.12	-0.23	0.22	0.12	-0.10	-0.21	0.44	0.33	0.39	0.29	0.65	0.55
Q8CJF7	Protein ELYS OS=Mus musculus GN=Ahctf1 PE=1 SV=1 - [ELYS_MOUSE]	0.01	0.28	0.29	0.18	0.20	0.20	0.18	0.53	0.38	0.39	0.45	0.48	0.38
Q01705-4	Isoform 4 of Neurogenic locus notch homolog protein 1 OS=Mus musculus GN=Notch1 - [NOTC1_MOUSE]	-0.21	0.36	0.41	-0.03	0.20	0.13	0.35	0.19	0.36	0.38	0.61	0.36	0.42
Q99LI5	Zinc finger protein 281 OS=Mus musculus GN=Znf281 PE=1 SV=1 - [ZN281_MOUSE]	-0.05	-0.10	-0.05	0.00	0.05	0.16	0.21	0.62	0.67	0.38	0.43	0.32	0.38
Q3UFQ8	Leucine-rich repeat-containing protein 16B OS=Mus musculus GN=Lrrc16b PE=2 SV=2 - [LR16B_MOUSE]	-0.12	0.26	0.37	-0.03	0.09	-0.13	-0.02	0.16	0.27	0.38	0.50	0.33	0.45
O08576-2	Isoform 2 of RUN domain-containing protein 3A OS=Mus musculus GN=Rundc3a - [RUN3A_MOUSE]	-0.14	0.67	0.81	0.15	0.29	0.26	0.39	0.68	0.82	0.37	0.51	0.50	0.64
Q08890	Iduronate 2-sulfatase OS=Mus musculus GN=Ids PE=2 SV=3 - [IDS_MOUSE]	0.07	0.38	0.30	0.49	0.42	-0.42	-0.50	0.46	0.38	0.37	0.29	0.58	0.50
B2RUJ8	Rho GTPase activating protein 12 OS=Mus musculus GN=Arhgap12 PE=2 SV=1 - [B2RUJ8_MOUSE]	-0.06	0.18	0.23	0.05	0.11	0.06	0.11	0.52	0.58	0.36	0.42	0.29	0.35
Q6IR34-3	Isoform 3 of G-protein-signaling modulator 1 OS=Mus musculus GN=Gpsm1 - [GPSM1_MOUSE]	0.06	-0.26	-0.36	-0.04	-0.15	0.36	0.19	0.08	0.01	0.36	0.35	0.58	0.34
G3UXB9	T-cell leukemia homeobox protein 1 OS=Mus musculus GN=Tlx1 PE=2 SV=1 - [G3UXB9_MOUSE]	-1.51	-0.36	1.15	-0.30	1.21	0.10	1.60	0.13	1.63	0.36	1.87	0.95	2.46
E1U8D0	Protein SOGA1 OS=Mus musculus GN=Soga1 PE=1 SV=3 - [SOGA1_MOUSE]	-0.11	0.20	0.33	-0.10	0.06	0.21	0.32	0.14	0.36	0.36	0.35	0.30	0.42
D3YVF0	A-kinase anchor protein 5 OS=Mus musculus GN=Akap5 PE=2 SV=2 - [AKAP5_MOUSE]	0.03	0.00	0.01	0.02	0.00	0.34	0.30	0.37	0.30	0.35	0.30	0.28	0.27
Q61327	Sodium-dependent dopamine transporter OS=Mus musculus GN=Slc6a3 PE=1 SV=2 - [SC6A3_MOUSE]	-0.19	-0.27	-0.08	0.45	0.64	0.00	0.19	-0.71	-0.53	0.34	0.53	0.92	1.11
Q61033	Lamina-associated polypeptide 2, isoforms alpha/zeta OS=Mus musculus GN=Tmpo PE=1 SV=4 - [LAP2A_MOUSE]	0.00	0.29	0.29	0.12	0.12	0.20	0.20	0.73	0.72	0.34	0.34	0.28	0.28
Q09XV5-2	Isoform 2 of Chromodomain-helicase-DNA-binding protein 8 OS=Mus musculus GN=Chd8 - [CHD8_MOUSE]	-0.07	0.50	0.56	0.00	0.06	0.27	0.33	0.47	0.53	0.34	0.40	0.48	0.54
Q8BHS6	Armadillo repeat-containing X-linked protein 3 OS=Mus musculus GN=Armcx3 PE=1 SV=1 - [ARMX3_MOUSE]	-0.02	0.19	0.21	0.15	0.17	0.27	0.29	0.00	0.02	0.34	0.36	0.44	0.46

O70146	Dual specificity testis-specific protein kinase 1 OS=Mus musculus GN=Tesk1 PE=2 SV=3 - [TESK1_MOUSE]	0.00	0.13	0.12	0.21	0.20	0.08	0.07	0.29	0.29	0.33	0.33	0.36	0.36
Q61016	Guanine nucleotide-binding protein G(I)/G(S)/G(O) subunit gamma-7 OS=Mus musculus GN=Gng7 PE=2 SV=2 - [GBG7_MOUSE]	-0.03	0.14	0.18	-0.09	-0.03	0.20	0.19	0.12	0.19	0.33	0.33	0.50	0.50
Q69ZF8	E3 ubiquitin-protein ligase MSL2 OS=Mus musculus GN=Msl2 PE=2 SV=2 - [MSL2_MOUSE]	0.01	0.15	0.14	0.21	0.20	0.29	0.28	0.37	0.35	0.32	0.32	0.47	0.47
Q8BXQ0	Ethanolamine kinase 1 OS=Mus musculus GN=Etnk1 PE=2 SV=1 - [Q8BXQ0_MOUSE]	-0.12	0.30	0.42	0.20	0.31	-0.05	0.06	-0.06	0.05	0.32	0.44	0.32	0.43
Q2PZL6	Protocadherin Fat 4 OS=Mus musculus GN=Fat4 PE=1 SV=2 - [FAT4_MOUSE]	-0.06	-0.04	0.01	-0.19	-0.13	0.19	0.24	0.16	0.21	0.32	0.37	0.30	0.36
P16332	Methylmalonyl-CoA mutase, mitochondrial OS=Mus musculus GN=Mut PE=2 SV=2 - [MUTA_MOUSE]	-0.07	0.08	0.07	0.29	0.37	0.10	0.00	0.26	0.05	0.31	0.33	0.36	0.38
P57776-3	Isoform 3 of Elongation factor 1-delta OS=Mus musculus GN=Eef1d - [EF1D_MOUSE]	-0.05	0.16	0.28	-0.06	0.00	0.34	0.31	0.41	0.36	0.31	0.43	0.35	0.40
F6VCZ4	Uncharacterized protein OS=Mus musculus GN=Zfp422-rs1 PE=4 SV=2 - [F6VCZ4_MOUSE]	-0.08	0.52	0.61	-0.08	0.00	0.38	0.46	0.25	0.32	0.30	0.39	0.51	0.60
E9Q1M6	Protein Maskbp3 OS=Mus musculus GN=Ankhd1 PE=4 SV=1 - [E9Q1M6_MOUSE]	-0.02	0.19	0.20	-0.07	-0.10	0.31	0.25	0.32	0.22	0.30	0.32	0.43	0.42
Q8CC27-4	Isoform 4 of Voltage-dependent L-type calcium channel subunit beta-2 OS=Mus musculus GN=Cacnb2 - [CACB2_MOUSE]	-0.01	0.11	0.12	0.13	0.08	-0.02	0.09	0.21	0.20	0.30	0.38	0.69	0.70
D3YXZ3	Kinesin light chain 2 OS=Mus musculus GN=Klc2 PE=4 SV=1 - [D3YXZ3_MOUSE]	-0.01	0.04	0.10	-0.02	0.05	0.34	0.35	0.41	0.35	0.29	0.30	0.29	0.27
E9PV69	CDK2-associated and cullin domain-containing protein 1 OS=Mus musculus GN=Cacul1 PE=2 SV=1 - [E9PV69_MOUSE]	-0.13	0.06	0.18	0.05	0.18	0.19	0.31	0.43	0.55	0.29	0.42	0.45	0.58
E9Q2Y9	Desumoylating isopeptidase 1 OS=Mus musculus GN=Desi1 PE=2 SV=1 - [E9Q2Y9_MOUSE]	-0.06	-0.33	-0.27	0.05	0.11	0.38	0.44	0.28	0.34	0.28	0.34	0.28	0.34
