# Supplementary material for: Are you also what your mother eats? Distinct proteomic portrait as a result of maternal high-fat diet in the cerebral cortex of the adult mouse
Source: Int J Obes (Lond). 2015 Apr 21;39(8):1325–8. doi: 10.1038/ijo.2015.35 (PMC5399160; doi:10.1038/ijo.2015.35)
Supplement: Supplementary Table 3 [file ijo201535x5.pdf]

Accesion	Description	114/113	115/113	115/114	116/113	116/114	117/113	117/114	118/113	118/114	119/113	119/114	121/113	121/114
Q5RL20	39S ribosomal protein L43, mitochondrial OS=Mus musculus GN=Mrpl43 PE=2 SV=1 - [Q5RL20_MOUSE]	0.05	0.27	0.22	0.29	0.24	0.65	0.59	0.69	0.64	0.65	0.61	0.45	0.41
Q3THG9	Alanyl-tRNA editing protein Aarsd1 OS=Mus musculus GN=Aarsd1 PE=1 SV=2 - [AASD1_MOUSE]	0.00	0.21	0.06	0.09	0.11	0.49	0.52	0.38	0.43	0.36	0.40	0.47	0.38
Q99ML1	Bcl-2-binding component 3 OS=Mus musculus GN=Bbc3 PE=1 SV=1 - [BBC3_MOUSE]	0.16	0.29	0.13	-0.04	-0.20	0.71	0.55	0.86	0.70	0.86	0.71	0.73	0.58
Q3U962	Collagen alpha-2(V) chain OS=Mus musculus GN=Col5a2 PE=1 SV=1 - [CO5A2_MOUSE]	0.03	0.42	0.39	0.12	0.09	0.52	0.48	0.54	0.50	0.49	0.46	0.60	0.57
M0QWU0	CXXC-type zinc finger protein 4 OS=Mus musculus GN=Cxxc4 PE=4 SV=1 - [M0QWU0_MOUSE]	-0.02	0.08	0.09	0.06	0.08	0.37	0.38	0.81	0.83	0.70	0.72	0.55	0.57
P97304	DNA-directed RNA polymerases I and III subunit RPAC2 OS=Mus musculus GN=Polr1d PE=2 SV=1 - [RPAC2_MOUSE]	0.03	-0.22	-0.25	-0.04	-0.07	0.81	0.77	0.91	0.87	0.61	0.57	0.58	0.54
O08600	Endonuclease G, mitochondrial OS=Mus musculus GN=Endog PE=2 SV=1 - [NUCG_MOUSE]	-0.10	0.16	0.26	0.22	0.31	0.37	0.46	0.37	0.47	0.47	0.57	0.44	0.54
J3QQ44	Endophilin-A3 OS=Mus musculus GN=Sh3gl3 PE=4 SV=1 - [J3QQ44_MOUSE]	0.03	0.35	0.31	0.29	0.25	0.56	0.52	0.59	0.55	0.68	0.65	0.50	0.47
Q9WUR9	GTP:AMP phosphotransferase AK4, mitochondrial OS=Mus musculus GN=Ak4 PE=2 SV=1 - [KAD4_MOUSE]	0.02	0.39	0.27	0.25	0.27	0.44	0.43	0.41	0.33	0.54	0.61	0.35	0.34
L7N209	HBS1-like protein OS=Mus musculus GN=Hbs1l PE=4 SV=1 - [L7N209_MOUSE]	-0.13	0.40	0.53	-0.06	0.07	0.41	0.53	0.60	0.72	0.45	0.59	0.33	0.46
Q9R257	Heme-binding protein 1 OS=Mus musculus GN=Hebp1 PE=1 SV=2 - [HEBP1_MOUSE]	-0.01	-0.24	-0.29	-0.03	0.02	0.45	0.51	0.54	0.58	0.49	0.49	0.40	0.43
A2AJ76	Hemicentin-2 OS=Mus musculus GN=Hmcn2 PE=1 SV=1 - [HMCN2_MOUSE]	0.48	0.34	-0.14	0.83	0.35	1.46	0.98	1.00	0.52	1.86	1.38	1.48	1.01
Q8BW74	Hepatic leukemia factor OS=Mus musculus GN=Hlf PE=2 SV=1 - [HLF_MOUSE]	-0.62	-0.16	0.46	-0.50	0.12	0.70	1.31	0.94	1.56	0.68	1.30	0.31	0.93
F8WIX8	Histone H2A OS=Mus musculus GN=Hist1h2al PE=2 SV=1 - [F8WIX8_MOUSE]	0.04	-0.27	-0.43	-0.08	-0.07	0.48	0.48	0.52	0.44	0.37	0.39	0.28	0.33

Q80Y83-11	Isoform 11 of Dixin OS=Mus musculus GN=Dixdc1 - [DIXC1_MOUSE]	-0.04	0.00	0.03	0.05	0.09	0.49	0.48	0.45	0.53	0.37	0.50	0.30	0.27
Q8C790-2	Isoform 2 of Protein FAM221A OS=Mus musculus GN=Fam221a - [F221A_MOUSE]	0.12	0.10	-0.03	0.13	0.02	0.71	0.58	0.57	0.45	0.52	0.41	0.45	0.34
Q9CY57-5	Isoform 5 of Chromatin target of PRMT1 protein OS=Mus musculus GN=Chtop - [CHTOP_MOUSE]	0.11	0.15	-0.17	0.08	0.05	0.62	0.46	0.61	0.54	0.58	0.51	0.37	0.41
P49919-2	Isoform KIP2b of Cyclin-dependent kinase inhibitor 1C OS=Mus musculus GN=Cdkn1c - [CDN1C_MOUSE]	0.06	0.20	0.14	0.04	-0.02	0.47	0.41	0.54	0.48	0.42	0.36	0.33	0.28
Q8BP48	Methionine aminopeptidase 1 OS=Mus musculus GN=Metap1 PE=2 SV=1 - [MAP11_MOUSE]	0.00	0.03	0.03	0.17	0.18	0.43	0.42	1.03	1.03	0.95	0.95	0.88	0.88
Q7TNS2	Mitochondrial inner membrane organizing system protein 1 OS=Mus musculus GN=Minos1 PE=2 SV=1 - [MOS1_MOUSE]	0.22	0.95	0.73	0.41	0.19	0.85	0.62	0.59	0.37	0.71	0.50	0.82	0.61
Q9D0T1	NHP2-like protein 1 OS=Mus musculus GN=Nhp2l1 PE=2 SV=4 - [NH2L1_MOUSE]	0.00	0.00	0.06	0.04	0.03	0.44	0.38	0.41	0.44	0.31	0.38	0.31	0.37
E9Q0V6	NHS-like protein 2 OS=Mus musculus GN=Nhsl2 PE=2 SV=1 - [E9Q0V6_MOUSE]	0.09	-0.03	-0.08	0.05	0.05	0.48	0.50	0.36	0.45	0.49	0.46	0.50	0.44
D3Z723	Nuclear transcription factor Y subunit beta (Fragment) OS=Mus musculus GN=Nfyb PE=2 SV=1 - [D3Z723_MOUSE]	0.08	0.55	0.47	0.14	0.06	0.77	0.68	0.94	0.85	0.73	0.66	0.58	0.50
E0CZ73	Occludin OS=Mus musculus GN=Ocln PE=2 SV=1 - [E0CZ73_MOUSE]	0.34	0.48	0.14	0.57	0.23	0.94	0.59	1.10	0.76	1.08	0.74	0.62	0.29
Q8BGT0	Osteopetrosis-associated transmembrane protein 1 OS=Mus musculus GN=Ostm1 PE=1 SV=1 - [OSTM1_MOUSE]	-0.08	0.16	0.23	0.21	0.29	0.77	0.84	0.81	0.89	0.80	0.89	0.96	1.04
O88492	Perilipin-4 OS=Mus musculus GN=Plin4 PE=1 SV=2 - [PLIN4_MOUSE]	0.07	-0.14	-0.21	-0.06	-0.13	0.87	0.79	0.64	0.57	0.52	0.45	0.47	0.40
Q9R0A0	Peroxisomal membrane protein PEX14 OS=Mus musculus GN=Pex14 PE=1 SV=1 - [PEX14_MOUSE]	0.09	0.29	0.33	0.11	0.15	0.74	0.66	0.70	0.76	0.70	0.70	0.57	0.49
F6VV25	Pleckstrin homology domain-containing family O member 1 (Fragment) OS=Mus musculus GN=Plekho1 PE=2 SV=1 - [F6VV25_MOUSE]	-0.02	0.10	0.12	0.12	0.14	0.60	0.61	1.22	1.24	0.95	0.97	0.48	0.50

E9PXF7	Protein Gm14446 OS=Mus musculus GN=Gm14446 PE=2 SV=1 - [E9PXF7_MOUSE]	0.09	-0.42	-0.52	1.05	0.95	1.86	1.76	3.35	3.26	3.10	3.01	1.96	1.87
G3UZ12	Protein Jumonji OS=Mus musculus GN=Jarid2 PE=2 SV=1 - [G3UZ12_MOUSE]	0.00	0.01	0.01	-0.10	-0.11	0.74	0.73	0.81	0.80	0.72	0.72	0.61	0.61
E9PZD2	Protein Micall2 OS=Mus musculus GN=Micall2 PE=2 SV=1 - [E9PZD2_MOUSE]	-0.10	0.07	0.16	-0.07	0.03	0.43	0.52	0.47	0.56	0.51	0.61	0.64	0.74
D3Z4F2	Protein Zfp819 (Fragment) OS=Mus musculus GN=Zfp819 PE=2 SV=1 - [D3Z4F2_MOUSE]	-0.04	-0.04	0.00	0.02	0.06	0.80	0.83	1.11	1.15	0.82	0.87	0.68	0.72
E9Q813	Ras-GEF domain-containing family member 1B OS=Mus musculus GN=Rasgef1b PE=2 SV=1 - [E9Q813_MOUSE]	0.33	0.85	0.51	0.52	0.18	0.80	0.46	1.11	0.77	0.98	0.65	0.87	0.54
Q9CW46	Ribonucleoprotein PTB-binding 1 OS=Mus musculus GN=Raver1 PE=1 SV=2 - [RAVR1_MOUSE]	0.03	0.07	0.11	-0.09	-0.05	0.41	0.46	0.60	0.44	0.44	0.50	0.58	0.56
F7BGR7	RNA-binding protein 4 OS=Mus musculus GN=Rbm4 PE=4 SV=1 - [F7BGR7_MOUSE]	0.02	-0.08	-0.10	0.61	0.59	0.74	0.71	0.56	0.54	0.57	0.56	0.29	0.27
Q8VE92	RNA-binding protein 4B OS=Mus musculus GN=Rbm4b PE=1 SV=1 - [RBM4B_MOUSE]	0.01	0.01	0.00	0.00	-0.01	0.60	0.58	0.85	0.83	0.50	0.49	0.63	0.61
Q8CJ40	Rootletin OS=Mus musculus GN=Crocc PE=1 SV=2 - [CROCC_MOUSE]	0.05	0.14	0.06	-0.01	-0.08	0.62	0.54	0.65	0.58	0.54	0.49	0.55	0.52
Q99K90	TGF-beta-activated kinase 1 and MAP3K7-binding protein 2 OS=Mus musculus GN=Tab2 PE=1 SV=1 - [TAB2_MOUSE]	0.10	0.10	0.19	0.02	-0.06	0.57	0.42	0.65	0.54	0.53	0.43	0.74	0.66
Q9WVF8	Tumor suppressor candidate 2 OS=Mus musculus GN=Tusc2 PE=1 SV=3 - [TUSC2_MOUSE]	0.05	0.01	-0.10	-0.06	-0.17	0.63	0.56	0.73	0.89	0.58	0.40	0.41	0.53
Q61152	Tyrosine-protein phosphatase non-receptor type 18 OS=Mus musculus GN=Ptpn18 PE=1 SV=1 - [PTN18_MOUSE]	0.00	0.66	0.66	0.15	0.15	0.42	0.42	0.62	0.61	0.55	0.55	2.38	2.38
P21126	Ubiquitin-like protein 4A OS=Mus musculus GN=Ubl4a PE=2 SV=1 - [UBL4A_MOUSE]	-0.06	-0.14	-0.16	-0.06	-0.03	0.62	0.60	0.54	0.55	0.42	0.46	0.35	0.43
E9Q722	Upstream stimulatory factor 1 OS=Mus musculus GN=Usf1 PE=2 SV=1 - [E9Q722_MOUSE]	-0.01	-0.22	-0.17	-0.04	-0.04	0.49	0.49	0.48	0.53	0.49	0.50	0.29	0.30
P61982	14-3-3 protein gamma OS=Mus musculus GN=Ywhag PE=1 SV=2 - [1433G_MOUSE]	-0.07	-0.12	-0.05	-0.08	-0.02	-0.53	-0.42	-0.52	-0.37	-0.56	-0.43	-0.51	-0.42

Q8BWT1	3-ketoacyl-CoA thiolase, mitochondrial OS=Mus musculus GN=Acaa2 PE=1 SV=3 - [THIM_MOUSE]	0.15	-0.13	-0.27	-0.16	-0.28	-0.76	-1.06	-0.92	-0.99	-0.76	-0.96	-0.66	-0.80
Q8C605	6-phosphofructokinase OS=Mus musculus GN=Pfkp PE=2 SV=1 - [Q8C605_MOUSE]	0.01	-0.12	-0.10	-0.08	-0.09	-0.90	-0.91	-0.94	-0.93	-0.92	-0.93	-0.82	-0.84
P12382	6-phosphofructokinase, liver type OS=Mus musculus GN=Pfkl PE=1 SV=4 - [K6PL_MOUSE]	0.01	0.07	0.09	0.05	0.01	-0.64	-0.70	-0.56	-0.61	-0.63	-0.62	-0.41	-0.48
P47857	6-phosphofructokinase, muscle type OS=Mus musculus GN=Pfkm PE=1 SV=3 - [K6PF_MOUSE]	0.04	-0.14	-0.18	-0.08	-0.12	-0.91	-0.98	-0.98	-1.02	-0.92	-0.94	-0.83	-0.89
Q80X81	Acetyl-Coenzyme A acetyltransferase 3 OS=Mus musculus GN=Acat3 PE=2 SV=1 - [Q80X81_MOUSE]	0.25	0.03	-0.23	-0.30	-0.53	-0.98	-0.78	-0.85	-1.04	-1.04	-1.01	-0.83	-1.13
Q9WV54	Acid ceramidase OS=Mus musculus GN=Asah1 PE=1 SV=1 - [ASAH1_MOUSE]	0.00	-0.19	-0.14	-0.18	-0.17	-1.11	-1.01	-0.90	-1.02	-0.92	-0.83	-0.81	-0.82
P56376	Acylphosphatase-1 OS=Mus musculus GN=Acyp1 PE=2 SV=2 - [ACYP1_MOUSE]	-0.04	-0.39	-0.42	-0.19	-0.07	-0.47	-0.41	-0.37	-0.37	-0.67	-0.43	-0.67	-0.43
Q64010	Adapter molecule crk OS=Mus musculus GN=Crk PE=1 SV=1 - [CRK_MOUSE]	-0.26	-0.32	-0.15	-0.23	0.03	-0.89	-0.78	-0.84	-0.59	-1.08	-0.84	-0.56	-0.38
Q8JZR2	Adapter molecule crk OS=Mus musculus GN=Crk PE=2 SV=1 - [Q8JZR2_MOUSE]	-0.14	-0.29	-0.12	-0.39	-0.08	-0.99	-0.92	-0.86	-0.78	-0.99	-0.91	-0.85	-0.71
P50247	Adenosylhomocysteinase OS=Mus musculus GN=Ahcy PE=1 SV=3 - [SAHH_MOUSE]	-0.11	-0.22	0.02	-0.03	0.09	-0.74	-0.52	-0.85	-0.74	-0.71	-0.60	-0.74	-0.61
Q60994	Adiponectin OS=Mus musculus GN=Adipoq PE=1 SV=2 - [ADIPO_MOUSE]	0.20	0.24	0.04	-0.05	-0.25	-0.67	-0.88	-0.74	-0.94	-0.45	-0.64	-0.53	-0.72
F6QKK2	ADP-ribosylation factor-like protein 8A (Fragment) OS=Mus musculus GN=Arl8a PE=4 SV=1 - [F6QKK2_MOUSE]	0.20	-0.11	-0.31	0.17	-0.03	-0.56	-0.47	-0.75	-0.67	-0.64	-0.59	-0.53	-0.36
Q9CQW2	ADP-ribosylation factor-like protein 8B OS=Mus musculus GN=Arl8b PE=2 SV=1 - [ARL8B_MOUSE]	0.18	-0.11	-0.30	0.07	-0.10	-0.56	-0.76	-0.54	-0.72	-0.48	-0.65	-0.41	-0.57
Q8QZR5	Alanine aminotransferase 1 OS=Mus musculus GN=Gpt PE=2 SV=3 - [ALAT1_MOUSE]	0.12	-0.15	-0.21	-0.32	-0.42	-1.17	-1.33	-1.09	-1.12	-1.34	-1.24	-1.07	-1.21
O88325	Alpha-N-acetylglucosaminidase OS=Mus musculus GN=Naglu PE=2 SV=1 - [O88325_MOUSE]	-0.10	-0.30	-0.20	-0.24	-0.14	-0.74	-0.65	-0.83	-0.74	-0.77	-0.67	-0.90	-0.80

Q9DB50	AP-1 complex subunit sigma-2 OS=Mus musculus GN=Ap1s2 PE=2 SV=1 - [AP1S2_MOUSE]	-0.04	0.27	0.31	0.20	0.23	-0.50	-0.47	-0.73	-0.70	-0.43	-0.39	-0.47	-0.43
P51910	Apolipoprotein D OS=Mus musculus GN=Apod PE=2 SV=1 - [APOD_MOUSE]	-0.15	-0.08	0.05	-0.12	0.05	-0.63	-0.55	-0.48	-0.52	-0.85	-0.66	-0.82	-0.61
Q9EPB4	Apoptosis-associated speck-like protein containing a CARD OS=Mus musculus GN=Pycard PE=1 SV=1 - [ASC_MOUSE]	0.04	-0.09	-0.12	-0.17	-0.15	-0.49	-0.62	-0.52	-0.46	-0.51	-0.64	-0.36	-0.41
P50428	Arylsulfatase A OS=Mus musculus GN=Arsa PE=2 SV=2 - [ARSA_MOUSE]	0.05	-0.34	-0.37	-0.18	-0.27	-1.11	-1.18	-1.02	-1.00	-1.04	-1.13	-0.78	-0.87
P05201	Aspartate aminotransferase, cytoplasmic OS=Mus musculus GN=Got1 PE=1 SV=3 - [AATC_MOUSE]	-0.01	-0.14	-0.17	-0.27	-0.26	-1.38	-1.37	-1.34	-1.36	-1.29	-1.32	-1.17	-1.20
P05202	Aspartate aminotransferase, mitochondrial OS=Mus musculus GN=Got2 PE=1 SV=1 - [AATM_MOUSE]	0.01	-0.26	-0.20	-0.27	-0.27	-1.47	-1.44	-1.42	-1.40	-1.42	-1.40	-1.23	-1.23
Q8R3P0	Aspartoacylase OS=Mus musculus GN=Aspa PE=1 SV=2 - [ACY2_MOUSE]	-0.45	-0.25	0.19	-0.34	0.10	-1.15	-0.69	-1.08	-0.57	-1.13	-0.69	-1.00	-0.61
Q9Z2W0	Aspartyl aminopeptidase OS=Mus musculus GN=Dnpep PE=2 SV=2 - [DNPEP_MOUSE]	0.13	-0.27	-0.42	-0.21	-0.36	-1.09	-1.24	-1.06	-1.19	-1.01	-1.20	-0.91	-1.04
Q9CQQ7	ATP synthase subunit b, mitochondrial OS=Mus musculus GN=Atp5f1 PE=1 SV=1 - [AT5F1_MOUSE]	-0.05	0.05	0.10	0.18	0.27	-0.71	-0.67	-0.81	-0.72	-0.56	-0.48	-0.45	-0.45
Q9D8Z6	Autophagy-related protein 101 OS=Mus musculus GN=Atg101 PE=2 SV=1 - [ATGA1_MOUSE]	0.04	0.40	0.36	-0.14	-0.18	-0.73	-0.78	-0.63	-0.68	-0.51	-0.54	-0.65	-0.69
Q99KJ6	Beta-glucuronidase OS=Mus musculus GN=Gusb PE=2 SV=1 - [Q99KJ6_MOUSE]	-0.02	0.09	0.10	-0.13	-0.11	-1.42	-1.41	-1.30	-1.29	-1.15	-1.13	-1.26	-1.24
Q99KR3	Beta-lactamase-like protein 2 OS=Mus musculus GN=Lactb2 PE=1 SV=1 - [LACB2_MOUSE]	0.10	-0.05	-0.26	-0.21	-0.37	-0.92	-1.03	-0.82	-1.00	-0.78	-1.01	-0.46	-0.84
O89106	Bis(5'-adenosyl)-triphosphatase OS=Mus musculus GN=Fhit PE=2 SV=3 - [FHIT_MOUSE]	0.05	-0.11	-0.25	-0.36	-0.46	-0.92	-1.14	-1.16	-1.25	-1.40	-1.47	-1.11	-1.15
Q8CBC8	Branched-chain-amino-acid aminotransferase OS=Mus musculus GN=Bcat1 PE=2 SV=1 - [Q8CBC8_MOUSE]	0.04	-0.09	-0.19	-0.23	-0.25	-0.95	-0.97	-0.91	-0.93	-0.94	-0.87	-0.79	-0.89

Q6WVG3	BTB/POZ domain-containing protein KCTD12 OS=Mus musculus GN=Kctd12 PE=1 SV=1 - [KCD12_MOUSE]	0.12	-0.03	-0.03	-0.15	-0.25	-0.80	-0.85	-0.74	-0.98	-0.73	-0.79	-0.65	-0.71
Q4VBE7	BTB/POZ domain-containing protein KCTD2 OS=Mus musculus GN=Kctd2 PE=2 SV=1 - [Q4VBE7_MOUSE]	0.20	0.38	0.18	0.18	-0.02	-0.70	-0.90	-0.43	-0.64	-0.68	-0.88	-0.58	-0.78
Q8VC57	BTB/POZ domain-containing protein KCTD5 OS=Mus musculus GN=Kctd5 PE=2 SV=1 - [KCTD5_MOUSE]	0.11	-0.04	-0.15	-0.08	-0.19	-0.69	-0.81	-0.59	-0.71	-0.54	-0.64	-0.33	-0.44
Q91YS8	Calcium/calmodulin-dependent protein kinase type 1 OS=Mus musculus GN=Camk1 PE=1 SV=1 - [KCC1A_MOUSE]	0.02	-0.46	-0.43	-0.16	-0.01	-0.36	-0.43	-0.39	-0.42	-0.45	-0.47	-0.55	-0.41
Q9CXW3	Calcyclin-binding protein OS=Mus musculus GN=Cacybp PE=1 SV=1 - [CYBP_MOUSE]	0.29	-0.57	-0.72	-0.12	-0.36	-0.52	-0.78	-0.41	-0.74	-0.49	-0.78	-0.44	-0.67
Q8R1G2	Carboxymethylenebutenolidase homolog OS=Mus musculus GN=Cmbl PE=2 SV=1 - [CMBL_MOUSE]	0.35	-0.20	-0.56	-0.16	-0.47	-0.73	-1.07	-0.68	-0.97	-0.59	-0.89	-0.47	-0.82
P70677	Caspase-3 OS=Mus musculus GN=Casp3 PE=1 SV=1 - [CASP3_MOUSE]	0.27	-0.22	-0.46	-0.03	-0.24	-0.59	-0.93	-0.73	-0.99	-0.58	-0.77	-0.48	-0.64
Q8BM88	Cathepsin O OS=Mus musculus GN=Ctso PE=2 SV=1 - [CATO_MOUSE]	0.02	0.44	0.41	-0.33	-0.36	-1.08	-1.12	-0.98	-1.01	-1.14	-1.17	-1.11	-1.13
O70370	Cathepsin S OS=Mus musculus GN=Ctss PE=2 SV=2 - [CATS_MOUSE]	0.08	-0.18	-0.20	-0.15	-0.20	-0.43	-0.57	-0.54	-0.89	-0.58	-0.69	-0.66	-0.66
Q5M8N0	CB1 cannabinoid receptor-interacting protein 1 OS=Mus musculus GN=Cnrip1 PE=1 SV=1 - [CNRP1_MOUSE]	-0.07	-0.46	-0.33	-0.40	-0.23	-1.12	-1.04	-1.20	-1.09	-1.06	-1.00	-1.20	-1.07
P40237	CD82 antigen OS=Mus musculus GN=Cd82 PE=1 SV=1 - [CD82_MOUSE]	-0.03	-0.31	-0.30	0.06	0.03	-0.52	-0.41	-0.53	-0.52	-0.45	-0.37	-0.52	-0.40
Q8BGU2	Cerebellin-2 OS=Mus musculus GN=Cbln2 PE=1 SV=1 - [CBLN2_MOUSE]	0.08	-0.18	-0.26	-0.24	-0.26	-0.65	-0.74	-0.68	-0.77	-0.88	-0.96	-0.83	-0.80
P29974	cGMP-gated cation channel alpha-1 OS=Mus musculus GN=Cnga1 PE=2 SV=2 - [CNGA1_MOUSE]	2.51	3.36	0.85	-1.39	-3.90	-1.69	-4.20	-1.37	-3.89	-1.29	-3.79	-1.97	-4.47
Q8CDI6	Coiled-coil domain-containing protein 158 OS=Mus musculus GN=Ccdc158 PE=2 SV=1 - [CD158_MOUSE]	1.39	1.37	-0.03	-0.40	-1.79	-0.86	-2.26	-0.92	-2.32	-0.88	-2.27	-0.53	-1.92

Q9QXP7	Complement C1q tumor necrosis factor-related protein 1 OS=Mus musculus GN=C1qtnf1 PE=2 SV=1 - [C1QT1_MOUSE]	0.32	0.22	-0.11	-0.12	-0.44	-0.92	-1.25	-1.01	-1.34	-1.01	-1.33	-0.94	-1.26
Q60571	Corticotropin-releasing factor-binding protein OS=Mus musculus GN=Crhbp PE=2 SV=1 - [CRHBP_MOUSE]	0.42	0.15	-0.28	-0.07	-0.49	-1.03	-1.46	-1.05	-1.47	-1.23	-1.65	-0.61	-1.03
Q8VCN5	Cystathionine gamma-lyase OS=Mus musculus GN=Cth PE=1 SV=1 - [CGL_MOUSE]	-0.14	0.06	0.19	-0.11	0.02	-1.15	-1.10	-1.06	-1.03	-0.95	-0.90	-0.86	-0.83
P00405	Cytochrome c oxidase subunit 2 OS=Mus musculus GN=Mtco2 PE=1 SV=1 - [COX2_MOUSE]	-0.10	-0.17	0.23	0.16	0.29	-0.77	-0.54	-0.72	-0.57	-0.45	-0.35	-0.46	-0.33
Q9CX80	Cytoglobin OS=Mus musculus GN=Cygb PE=2 SV=1 - [CYGB_MOUSE]	0.28	-0.28	-0.33	-0.08	-0.45	-0.87	-1.29	-0.92	-1.21	-0.76	-1.02	-0.77	-0.99
O88485	Cytoplasmic dynein 1 intermediate chain 1 OS=Mus musculus GN=Dync1i1 PE=1 SV=2 - [DC1I1_MOUSE]	-0.03	0.02	0.08	-0.13	-0.05	-0.62	-0.57	-0.64	-0.50	-0.62	-0.56	-0.67	-0.61
Q3V1L4	Cytosolic purine 5'-nucleotidase OS=Mus musculus GN=Nt5c2 PE=1 SV=2 - [5NTC_MOUSE]	0.01	-0.19	-0.24	-0.23	-0.18	-0.98	-0.93	-1.00	-1.18	-0.97	-0.92	-0.78	-0.85
P61963	DDB1- and CUL4-associated factor 7 OS=Mus musculus GN=Dcaf7 PE=2 SV=1 - [DCAF7_MOUSE]	0.05	-0.26	-0.32	-0.18	-0.22	-1.55	-1.56	-1.39	-1.45	-1.21	-1.26	-1.04	-1.09
O35459	Delta(3,5)-Delta(2,4)-dienoyl-CoA isomerase, mitochondrial OS=Mus musculus GN=Ech1 PE=2 SV=1 - [ECH1_MOUSE]	0.14	-0.08	-0.29	-0.32	-0.44	-1.27	-1.30	-0.83	-0.92	-0.57	-0.71	-0.52	-0.58
F6W7I2	Deoxyhypusine synthase (Fragment) OS=Mus musculus GN=Dhps PE=2 SV=1 - [F6W7I2_MOUSE]	0.05	0.24	0.18	-0.63	-0.68	-1.28	-1.34	-1.29	-1.35	-0.97	-1.03	-1.51	-1.56
Q9D2G2	Dihydrolipoyllysine-residue succinyltransferase component of 2-oxoglutarate dehydrogenase complex, mitochondrial OS=Mus musculus GN=Dlst PE=1 SV=1 - [ODO2_MOUSE]	0.01	-0.26	-0.23	-0.20	-0.22	-0.63	-0.63	-0.51	-0.53	-0.52	-0.54	-0.71	-0.69
O08553	Dihydropyrimidinase-related protein 2 OS=Mus musculus GN=Dpysl2 PE=1 SV=2 - [DPYL2_MOUSE]	-0.12	-0.12	-0.03	-0.15	-0.03	-0.59	-0.45	-0.59	-0.44	-0.64	-0.48	-0.54	-0.41

Q9JI46	Diphosphoinositol polyphosphate phosphohydrolase 1 OS=Mus musculus GN=Nudt3 PE=1 SV=1 - [NUDT3_MOUSE]	-0.22	-0.24	-0.11	-0.25	-0.06	-0.86	-0.74	-0.89	-0.65	-0.88	-0.71	-0.85	-0.72
Q9CWQ0	Diphthine synthase OS=Mus musculus GN=Dph5 PE=2 SV=2 - [DPH5_MOUSE]	0.38	0.16	-0.31	-0.09	-0.62	-0.91	-1.50	-0.86	-1.46	-0.80	-1.39	-0.58	-1.16
Q9DD18	D-tyrosyl-tRNA(Tyr) deacylase 1 OS=Mus musculus GN=Dtd1 PE=1 SV=2 - [DTD1_MOUSE]	0.15	0.09	-0.06	-0.15	-0.19	-0.61	-0.73	-0.59	-0.64	-0.54	-0.71	-0.60	-0.66
Q8BTR5	Dual specificity phosphatase 28 OS=Mus musculus GN=Dusp28 PE=1 SV=1 - [DUS28_MOUSE]	-0.03	0.11	0.13	0.02	0.04	-0.84	-0.82	-0.89	-0.87	-0.91	-0.88	-0.95	-0.92
Q6NT99	Dual specificity protein phosphatase 23 OS=Mus musculus GN=Dusp23 PE=2 SV=1 - [DUS23_MOUSE]	0.15	0.07	-0.04	-0.08	-0.30	-1.14	-1.31	-1.14	-1.23	-1.11	-1.19	-0.56	-0.72
Q9D0M5	Dynein light chain 2, cytoplasmic OS=Mus musculus GN=Dynll2 PE=1 SV=1 - [DYL2_MOUSE]	-0.11	-0.30	-0.23	-0.21	-0.12	-0.77	-0.64	-0.85	-0.58	-0.88	-0.74	-1.12	-0.70
Q9CQJ4	E3 ubiquitin-protein ligase RING2 OS=Mus musculus GN=Rnf2 PE=1 SV=1 - [RING2_MOUSE]	-0.10	-0.75	-0.66	-0.14	-0.04	-0.61	-0.52	-0.58	-0.48	-0.66	-0.56	-0.61	-0.51
Q8C4U8	EGF-like repeat and discoidin I-like domain-containing protein 3 OS=Mus musculus GN=Edil3 PE=2 SV=1 - [Q8C4U8_MOUSE]	0.05	-0.03	0.00	-0.07	-0.10	-0.52	-0.46	-0.54	-0.45	-0.60	-0.65	-0.32	-0.34
P42125	Enoyl-CoA delta isomerase 1, mitochondrial OS=Mus musculus GN=Eci1 PE=2 SV=2 - [ECI1_MOUSE]	0.16	-0.16	-0.42	-0.06	-0.25	-0.83	-1.21	-0.87	-1.19	-0.84	-1.08	-0.79	-1.15
O55208	Factor in the germline alpha OS=Mus musculus GN=Figla PE=2 SV=1 - [FIGLA_MOUSE]	0.25	-0.22	-0.47	0.10	-0.15	-1.56	-1.82	-1.32	-1.57	-1.23	-1.47	-0.69	-0.94
P11404	Fatty acid-binding protein, heart OS=Mus musculus GN=Fabp3 PE=1 SV=5 - [FABPH_MOUSE]	-0.06	-0.15	0.01	-0.18	-0.09	-0.70	-0.53	-0.57	-0.43	-0.81	-0.71	-0.71	-0.56
P22315	Ferrochelatase, mitochondrial OS=Mus musculus GN=Fech PE=1 SV=2 - [HEMH_MOUSE]	0.00	-0.20	-0.18	-0.14	-0.05	-0.44	-0.43	-0.53	-0.49	-0.48	-0.52	-0.58	-0.45
Q8BGT1	Fibronectin leucine rich transmembrane protein 3 OS=Mus musculus GN=Flrt3 PE=2 SV=1 - [Q8BGT1_MOUSE]	0.13	-0.36	-0.43	0.01	-0.09	-0.44	-0.58	-0.67	-0.74	-0.71	-0.81	-0.56	-0.63
Q9ER35	Fructosamine-3-kinase OS=Mus musculus GN=Fn3k PE=2 SV=1 - [FN3K_MOUSE]	-0.03	0.11	0.07	-0.07	-0.09	-0.77	-0.53	-0.69	-0.61	-0.84	-0.64	-0.72	-0.57

P35505	Fumarylacetoacetase OS=Mus musculus GN=Fah PE=1 SV=2 - [FAAA_MOUSE]	0.18	-0.20	-0.34	-0.34	-0.45	-1.32	-1.55	-1.18	-1.34	-1.20	-1.32	-1.12	-1.32
Q9DCD6	Gamma-aminobutyric acid receptor-associated protein OS=Mus musculus GN=Gabarap PE=1 SV=2 - [GBRAP_MOUSE]	-0.29	-1.30	-1.01	-0.53	-0.24	-1.65	-1.37	-1.83	-1.54	-1.86	-1.57	-1.91	-1.62
P60521	Gamma-aminobutyric acid receptor-associated protein-like 2 OS=Mus musculus GN=Gabarapl2 PE=1 SV=1 - [GBRL2_MOUSE]	-0.16	-0.43	-0.31	-0.35	-0.20	-0.81	-0.58	-0.65	-0.67	-0.79	-0.77	-0.71	-0.54
P17183	Gamma-enolase OS=Mus musculus GN=Eno2 PE=1 SV=2 - [ENOG_MOUSE]	0.01	-0.12	-0.14	-0.18	-0.18	-0.62	-0.65	-0.59	-0.58	-0.64	-0.64	-0.66	-0.69
O88958	Glucosamine-6-phosphate isomerase 1 OS=Mus musculus GN=Gnpda1 PE=2 SV=3 - [GNPI1_MOUSE]	0.15	-0.32	-0.20	-0.19	-0.26	-0.65	-0.68	-0.90	-0.57	-0.75	-0.52	-0.85	-0.51
Q9CQM9	Glutaredoxin-3 OS=Mus musculus GN=Glrx3 PE=1 SV=1 - [GLRX3_MOUSE]	0.02	-0.38	-0.39	-0.20	-0.23	-0.84	-0.85	-0.71	-0.73	-0.82	-0.73	-0.73	-0.67
P11352	Glutathione peroxidase 1 OS=Mus musculus GN=Gpx1 PE=1 SV=2 - [GPX1_MOUSE]	-0.11	-0.19	-0.15	-0.09	0.06	-0.65	-0.54	-0.61	-0.55	-0.64	-0.51	-0.64	-0.39
O09131	Glutathione S-transferase omega-1 OS=Mus musculus GN=Gsto1 PE=2 SV=2 - [GSTO1_MOUSE]	-0.12	-0.35	-0.30	-0.18	-0.10	-0.59	-0.52	-0.72	-0.58	-0.64	-0.49	-0.59	-0.48
Q64521	Glycerol-3-phosphate dehydrogenase, mitochondrial OS=Mus musculus GN=Gpd2 PE=1 SV=2 - [GPDM_MOUSE]	0.10	0.01	-0.10	0.03	-0.08	-0.52	-0.57	-0.57	-0.61	-0.54	-0.57	-0.51	-0.52
Q8VEB4	Group XV phospholipase A2 OS=Mus musculus GN=Pla2g15 PE=1 SV=1 - [PAG15_MOUSE]	0.05	0.03	-0.08	-0.16	-0.30	-1.28	-1.27	-1.32	-1.47	-1.08	-1.17	-1.05	-1.07
Q9R111	Guanine deaminase OS=Mus musculus GN=Gda PE=1 SV=1 - [GUAD_MOUSE]	0.03	-0.04	-0.14	-0.18	-0.23	-0.84	-0.91	-0.88	-0.92	-0.89	-0.93	-0.68	-0.85
Q3UGR5	Haloacid dehalogenase-like hydrolase domain-containing protein 2 OS=Mus musculus GN=Hdhd2 PE=1 SV=2 - [HDHD2_MOUSE]	0.22	-0.23	-0.57	-0.30	-0.51	-1.48	-1.70	-1.52	-1.86	-1.51	-1.72	-1.35	-1.55
Q9QUP5	Hyaluronan and proteoglycan link protein 1 OS=Mus musculus GN=Hapln1 PE=2 SV=1 - [HPLN1_MOUSE]	-0.10	-0.23	-0.11	-0.16	-0.04	-0.69	-0.52	-0.70	-0.59	-0.58	-0.45	-0.48	-0.45

D3YZU6	Hydroxyacylglutathione hydrolase-like protein OS=Mus musculus GN=Haghl PE=4 SV=1 - [D3YZU6_MOUSE]	-0.03	-0.15	-0.16	-0.31	-0.22	-0.84	-1.04	-1.24	-1.29	-1.17	-1.25	-1.11	-0.93
Q4FJZ2	Importin subunit alpha OS=Mus musculus GN=Kpna6 PE=2 SV=1 - [Q4FJZ2_MOUSE]	0.01	-0.26	-0.26	-0.16	-0.27	-0.71	-0.61	-0.51	-0.52	-0.53	-0.56	-0.36	-0.34
P24547	Inosine-5'-monophosphate dehydrogenase 2 OS=Mus musculus GN=Impdh2 PE=1 SV=2 - [IMDH2_MOUSE]	0.13	-0.01	-0.18	0.05	-0.10	-0.80	-0.97	-0.96	-1.07	-0.77	-0.93	-0.68	-0.89
Q924B0	Inositol (Myo)-1(Or 4)-monophosphatase 1 OS=Mus musculus GN=Impa1 PE=2 SV=1 - [Q924B0_MOUSE]	0.27	-0.18	-0.42	-0.01	-0.30	-1.04	-1.31	-0.94	-1.30	-0.82	-1.00	-0.65	-1.02
Q8C0M9	Isoaspartyl peptidase/L-asparaginase OS=Mus musculus GN=Asrgl1 PE=1 SV=1 - [ASGL1_MOUSE]	0.08	-0.31	-0.40	-0.16	-0.24	-0.54	-0.65	-0.49	-0.61	-0.53	-0.61	-0.39	-0.46
Q8BW96-2	Isoform 2 of Calcium/calmodulin-dependent protein kinase type 1D OS=Mus musculus GN=Camk1d - [KCC1D_MOUSE]	-0.04	-0.38	-0.35	-0.10	-0.15	-0.51	-0.57	-0.53	-0.53	-0.66	-0.68	-0.67	-0.74
Q9CPY7-2	Isoform 2 of Cytosol aminopeptidase OS=Mus musculus GN=Lap3 - [AMPL_MOUSE]	0.15	-0.20	-0.28	-0.24	-0.35	-0.90	-0.98	-0.93	-0.97	-0.80	-0.89	-0.80	-0.89
Q9D9V3-2	Isoform 2 of Ethylmalonyl-CoA decarboxylase OS=Mus musculus GN=Echdc1 - [ECHD1_MOUSE]	0.02	0.17	-0.21	0.00	-0.08	-0.51	-0.62	-0.64	-0.71	-0.55	-0.55	-1.00	-0.91
P61329-2	Isoform 2 of Fibroblast growth factor 12 OS=Mus musculus GN=Fgf12 - [FGF12_MOUSE]	0.01	-0.38	-0.17	-0.19	-0.26	-0.53	-0.44	-0.54	-0.59	-0.52	-0.62	-0.50	-0.59
P62881-2	Isoform 2 of Guanine nucleotide-binding protein subunit beta-5 OS=Mus musculus GN=Gnb5 - [GBB5_MOUSE]	-0.13	-0.17	-0.05	-0.06	0.02	-0.51	-0.44	-0.71	-0.57	-0.66	-0.52	-0.66	-0.50
Q9QWW1-2	Isoform 2 of Homer protein homolog 2 OS=Mus musculus GN=Homer2 - [HOME2_MOUSE]	0.17	-0.22	-0.36	-0.11	-0.25	-0.72	-0.91	-0.67	-0.80	-0.93	-1.18	-0.73	-0.92
Q8R0N6-2	Isoform 2 of Hydroxyacid-oxoacid transhydrogenase, mitochondrial OS=Mus musculus GN=Adhfe1 - [HOT_MOUSE]	0.02	-0.33	-0.25	-0.18	-0.27	-0.85	-0.87	-0.89	-0.93	-1.19	-1.20	-0.94	-0.94
Q71RI9-2	Isoform 2 of Kynurenine--oxoglutarate transaminase 3 OS=Mus musculus GN=Ccbl2 - [KAT3_MOUSE]	-0.04	0.05	0.09	-0.14	-0.11	-0.75	-0.72	-0.63	-0.59	-0.65	-0.61	-0.51	-0.47

P21956-2	Isoform 2 of Lactadherin OS=Mus musculus GN=Mfge8 - [MFGM_MOUSE]	0.21	-0.19	-0.40	0.09	-0.13	-0.87	-1.09	-0.81	-1.03	-0.82	-1.03	-0.64	-0.85
Q8VDK1-2	Isoform 2 of Nitrilase homolog 1 OS=Mus musculus GN=Nit1 - [NIT1_MOUSE]	0.09	-0.19	-0.38	-0.19	-0.26	-1.31	-1.53	-1.31	-1.45	-1.25	-1.38	-1.21	-1.26
Q61474-2	Isoform 2 of RNA-binding protein Musashi homolog 1 OS=Mus musculus GN=Msi1 - [MSI1H_MOUSE]	0.22	-0.19	-0.41	-0.22	-0.44	-0.98	-1.21	-0.76	-0.98	-0.53	-0.75	-0.48	-0.70
A3KMP2-2	Isoform 2 of Tetratricopeptide repeat protein 38 OS=Mus musculus GN=Ttc38 - [TTC38_MOUSE]	-0.02	0.19	0.21	-0.30	-0.29	-0.74	-0.73	-0.87	-0.86	-0.69	-0.67	-0.49	-0.47
Q9JMH6-2	Isoform 2 of Thioredoxin reductase 1, cytoplasmic OS=Mus musculus GN=Txnrd1 - [TRXR1_MOUSE]	0.17	-0.07	-0.33	-0.11	-0.28	-1.14	-1.28	-0.97	-1.07	-0.99	-1.07	-0.81	-0.88
Q9DCF9-2	Isoform 2 of Translocon-associated protein subunit gamma OS=Mus musculus GN=Ssr3 - [SSRG_MOUSE]	-0.29	0.10	0.39	0.07	0.36	-1.10	-0.81	-0.96	-0.68	-0.82	-0.52	-0.72	-0.43
Q3TTY0-3	Isoform 3 of Phospholipase B1, membrane-associated OS=Mus musculus GN=Plb1 - [PLB1_MOUSE]	0.51	-0.22	-0.73	-0.04	-0.55	-0.94	-1.46	-1.39	-1.91	-1.34	-1.85	-0.37	-0.88
Q9CSP9-4	Isoform 4 of Tetratricopeptide repeat protein 14 OS=Mus musculus GN=Ttc14 - [TTC14_MOUSE]	-0.23	-0.08	0.15	-0.55	-0.32	-1.14	-0.91	-0.75	-0.52	-1.22	-0.99	-0.77	-0.54
P63087-2	Isoform Gamma-2 of Serine/threonine-protein phosphatase PP1-gamma catalytic subunit OS=Mus musculus GN=Ppp1cc - [PP1G_MOUSE]	-0.05	-0.09	-0.03	-0.16	-0.06	-1.04	-0.83	-0.78	-0.67	-0.89	-0.71	-0.65	-0.55
Q8BTY1	Kynurenine--oxoglutarate transaminase 1 OS=Mus musculus GN=Ccbl1 PE=2 SV=1 - [KAT1_MOUSE]	0.16	-0.23	-0.40	-0.35	-0.52	-0.94	-1.21	-0.94	-1.12	-0.84	-1.06	-0.70	-0.93
Q9CPU0	Lactoylglutathione lyase OS=Mus musculus GN=Glo1 PE=1 SV=3 - [LGUL_MOUSE]	-0.03	-0.50	-0.42	-0.23	-0.30	-1.08	-1.01	-1.02	-1.01	-1.00	-1.05	-0.98	-0.97
Q99PH1	Leucine-rich repeat-containing protein 4 OS=Mus musculus GN=Lrrc4 PE=1 SV=2 - [LRRC4_MOUSE]	0.07	0.31	0.24	0.06	-0.01	-0.41	-0.49	-0.45	-0.52	-0.64	-0.71	-0.55	-0.62

P53395	Lipoamide acyltransferase component of branched-chain alpha-keto acid dehydrogenase complex, mitochondrial OS=Mus musculus GN=Dbt PE=2 SV=2 - [ODB2_MOUSE]	-0.04	-0.33	-0.44	-0.11	-0.12	-0.53	-0.51	-0.51	-0.41	-0.57	-0.46	-0.42	-0.38
P51174	Long-chain specific acyl-CoA dehydrogenase, mitochondrial OS=Mus musculus GN=Acadl PE=2 SV=2 - [ACADL_MOUSE]	-0.03	-0.26	-0.16	-0.15	-0.12	-1.05	-1.00	-1.07	-0.96	-1.01	-1.02	-0.89	-0.86
Q8R033	LYR motif-containing protein 2 OS=Mus musculus GN=Lyrm2 PE=2 SV=1 - [LYRM2_MOUSE]	-0.01	0.36	0.36	-0.20	-0.19	-0.43	-0.43	-0.37	-0.37	-0.47	-0.46	-0.46	-0.45
Q8K215	LYR motif-containing protein 4 OS=Mus musculus GN=Lyrm4 PE=2 SV=1 - [LYRM4_MOUSE]	-0.02	-0.62	-0.60	-0.10	-0.08	-0.50	-0.49	-0.62	-0.61	-0.65	-0.63	-0.45	-0.43
O09159	Lysosomal alpha-mannosidase OS=Mus musculus GN=Man2b1 PE=2 SV=4 - [MA2B1_MOUSE]	-0.09	-0.02	0.07	-0.31	-0.22	-0.58	-0.50	-0.83	-0.71	-0.72	-0.59	-0.62	-0.53
Q9DAR7	m7GpppX diphosphatase OS=Mus musculus GN=Dcps PE=1 SV=1 - [DCPS_MOUSE]	-0.17	-0.15	0.04	-0.31	0.09	-0.70	-0.51	-0.77	-0.54	-0.87	-0.51	-0.92	-0.68
Q924M7	Mannose-6-phosphate isomerase OS=Mus musculus GN=Mpi PE=2 SV=1 - [MPI_MOUSE]	0.15	-0.18	-0.33	-0.06	-0.17	-0.51	-0.72	-0.59	-0.66	-0.53	-0.66	-0.43	-0.60
Q6T264	Mastermind-like protein 1 OS=Mus musculus GN=Maml1 PE=1 SV=2 - [MAML1_MOUSE]	2.23	2.59	0.36	-1.37	-3.60	-1.77	-4.01	-1.16	-3.39	-1.75	-3.98	-1.37	-3.59
Q3UWE6	MCG14935, isoform CRA_a OS=Mus musculus GN=Wdr20a PE=2 SV=1 - [Q3UWE6_MOUSE]	-0.02	0.01	-0.08	-0.17	-0.17	-1.08	-1.07	-1.06	-1.15	-1.14	-1.19	-0.93	-0.99
Q61884	Meiosis-specific nuclear structural protein 1 OS=Mus musculus GN=Mns1 PE=1 SV=1 - [MNS1_MOUSE]	0.35	-0.36	-0.71	-0.07	-0.42	-0.78	-1.13	-0.42	-0.77	-0.44	-0.79	-0.46	-0.81
D3YTP0	Metalloreductase STEAP3 (Fragment) OS=Mus musculus GN=Steap3 PE=2 SV=1 - [D3YTP0_MOUSE]	-0.07	0.06	0.13	-0.18	-0.11	-0.75	-0.69	-0.57	-0.50	-0.65	-0.58	-0.69	-0.62
Q9CQT1	Methylthioribose-1-phosphate isomerase OS=Mus musculus GN=Mri1 PE=2 SV=1 - [MTNA_MOUSE]	-0.06	-0.14	-0.05	-0.46	-0.39	-1.25	-1.26	-1.18	-1.12	-1.31	-1.19	-1.30	-1.17
Q9WVQ5	Methylthioribulose-1-phosphate dehydratase OS=Mus musculus GN=Apip PE=1 SV=1 - [MTNB_MOUSE]	0.15	-0.04	-0.43	-0.17	-0.19	-0.37	-0.44	-0.35	-0.52	-0.38	-0.59	-0.41	-0.77

Q91VR7	Microtubule-associated proteins 1A/1B light chain 3A OS=Mus musculus GN=Map1lc3a PE=1 SV=1 - [MLP3A_MOUSE]	-0.20	-0.93	-0.82	-0.31	-0.10	-0.88	-0.67	-0.81	-0.60	-1.00	-0.77	-0.89	-0.62
Q61532	Mitogen-activated protein kinase 6 OS=Mus musculus GN=Mapk6 PE=1 SV=3 - [MK06_MOUSE]	0.50	-0.31	-0.81	0.00	-0.50	-1.24	-1.75	-1.21	-1.72	-1.12	-1.62	-0.86	-1.36
P32211	Muscarinic acetylcholine receptor M4 OS=Mus musculus GN=Chrm4 PE=2 SV=1 - [ACM4_MOUSE]	0.06	-0.32	-0.38	-0.08	-0.14	-0.54	-0.60	-0.41	-0.47	-0.49	-0.54	-0.39	-0.45
Q571E4	N-acetylgalactosamine-6-sulfatase OS=Mus musculus GN=Galns PE=2 SV=2 - [GALNS_MOUSE]	-0.02	0.16	0.18	-0.49	-0.47	-1.14	-1.13	-1.15	-1.14	-1.26	-1.24	-1.08	-1.06
P58058	NAD kinase OS=Mus musculus GN=Nadk PE=1 SV=2 - [NADK_MOUSE]	-0.21	0.25	0.45	-0.08	0.13	-1.13	-0.93	-1.11	-0.91	-1.19	-0.98	-1.03	-0.82
Q8CC86	Nicotinate phosphoribosyltransferase OS=Mus musculus GN=Naprt1 PE=2 SV=1 - [PNCB_MOUSE]	-0.19	-0.10	0.09	-0.50	-0.32	-0.91	-0.73	-0.89	-0.70	-0.65	-0.46	-0.89	-0.70
Q8BM13	Noelin-2 OS=Mus musculus GN=Olfm2 PE=1 SV=2 - [NOE2_MOUSE]	-0.04	-0.19	-0.24	0.00	0.10	-0.53	-0.64	-0.55	-0.61	-0.70	-0.61	-0.58	-0.53
P61971	Nuclear transport factor 2 OS=Mus musculus GN=Nutf2 PE=2 SV=1 - [NTF2_MOUSE]	-0.08	-0.12	-0.04	0.00	-0.03	-0.69	-0.52	-0.67	-0.58	-0.48	-0.43	-0.58	-0.47
P29758	Ornithine aminotransferase, mitochondrial OS=Mus musculus GN=Oat PE=1 SV=1 - [OAT_MOUSE]	0.14	-0.05	-0.12	0.08	-0.04	-0.46	-0.57	-0.37	-0.45	-0.35	-0.40	-0.38	-0.42
Q8BFQ8	Parkinson disease 7 domain-containing protein 1 OS=Mus musculus GN=Pddc1 PE=1 SV=1 - [PDDC1_MOUSE]	0.16	-0.03	0.03	0.11	0.01	-0.68	-0.80	-0.77	-0.76	-0.68	-0.79	-0.65	-0.50
Q64378	Peptidyl-prolyl cis-trans isomerase FKBP5 OS=Mus musculus GN=Fkbp5 PE=1 SV=1 - [FKBP5_MOUSE]	-0.14	-0.37	-0.23	-0.46	-0.32	-0.54	-0.41	-0.63	-0.49	-0.66	-0.52	-0.61	-0.46
Q99LL5	Periodic tryptophan protein 1 homolog OS=Mus musculus GN=Pwp1 PE=1 SV=1 - [PWP1_MOUSE]	0.14	-0.06	-0.20	-0.13	-0.28	-0.93	-1.08	-0.78	-0.92	-1.19	-1.34	-0.91	-1.05
P35700	Peroxiredoxin-1 OS=Mus musculus GN=Prdx1 PE=1 SV=1 - [PRDX1_MOUSE]	0.02	-0.26	-0.27	-0.14	-0.16	-0.64	-0.65	-0.53	-0.58	-0.65	-0.69	-0.68	-0.66
Q61171	Peroxiredoxin-2 OS=Mus musculus GN=Prdx2 PE=1 SV=3 - [PRDX2_MOUSE]	0.06	-0.32	-0.37	-0.05	-0.11	-0.63	-0.66	-0.64	-0.68	-0.67	-0.68	-0.66	-0.74
O08807	Peroxiredoxin-4 OS=Mus musculus GN=Prdx4 PE=1 SV=1 - [PRDX4_MOUSE]	0.08	-0.45	-0.48	-0.15	-0.22	-0.91	-0.99	-0.88	-0.88	-0.95	-0.94	-0.89	-0.88

Q9DC50	Peroxisomal carnitine O-octanoyltransferase OS=Mus musculus GN=Crot PE=1 SV=1 - [OCTC_MOUSE]	0.29	0.33	0.03	-0.51	-0.80	-0.39	-0.57	-0.46	-0.76	-0.36	-0.65	-0.68	-0.65
Q9DBJ1	Phosphoglycerate mutase 1 OS=Mus musculus GN=Pgam1 PE=1 SV=3 - [PGAM1_MOUSE]	-0.11	-0.26	-0.15	-0.21	-0.09	-0.77	-0.67	-0.80	-0.70	-0.78	-0.67	-0.83	-0.70
Q99K85	Phosphoserine aminotransferase OS=Mus musculus GN=Psat1 PE=1 SV=1 - [SERC_MOUSE]	-0.04	-0.29	-0.24	-0.09	-0.08	-0.80	-0.81	-0.87	-0.84	-0.76	-0.73	-0.71	-0.68
Q8BWR2	PITH domain-containing protein 1 OS=Mus musculus GN=Pithd1 PE=2 SV=1 - [PITH1_MOUSE]	0.17	-0.21	-0.44	-0.23	-0.47	-1.03	-1.28	-0.80	-1.20	-0.87	-1.11	-0.57	-0.91
Q61233	Plastin-2 OS=Mus musculus GN=Lcp1 PE=1 SV=4 - [PLSL_MOUSE]	0.09	-0.04	-0.09	-0.05	-0.19	-1.10	-1.12	-1.07	-1.09	-1.01	-0.97	-1.01	-1.00
P63005	Platelet-activating factor acetylhydrolase IB subunit alpha OS=Mus musculus GN=Pafah1b1 PE=1 SV=2 - [LIS1_MOUSE]	-0.01	-0.38	-0.35	-0.24	-0.24	-1.38	-1.37	-1.27	-1.25	-1.23	-1.28	-1.15	-1.18
D3Z1E2	Pleckstrin homology domain-containing family B member 1 (Fragment) OS=Mus musculus GN=Plekhb1 PE=2 SV=1 - [D3Z1E2_MOUSE]	0.06	0.27	0.20	-0.21	-0.29	-0.72	-0.78	-0.61	-0.77	-0.60	-0.72	-0.49	-0.57
Q9D240	Pleckstrin homology domain-containing family J member 1 OS=Mus musculus GN=Plekhj1 PE=2 SV=1 - [PKHJ1_MOUSE]	0.09	-0.31	-0.41	-0.33	-0.42	-0.91	-1.01	-0.80	-0.90	-0.86	-0.95	-0.86	-0.94
Q922V4	Pleiotropic regulator 1 OS=Mus musculus GN=Plrg1 PE=2 SV=1 - [PLRG1_MOUSE]	0.15	-0.38	-0.35	-0.17	-0.38	-0.47	-0.79	-0.59	-0.63	-0.55	-0.82	-0.51	-0.75
Q8BHS3	Pre-mRNA-splicing factor RBM22 OS=Mus musculus GN=Rbm22 PE=2 SV=1 - [RBM22_MOUSE]	-0.23	-0.36	-0.31	-0.26	-0.02	-0.83	-0.69	-0.83	-0.69	-0.73	-0.51	-0.98	-0.77
Q8C7K6	Prenylcysteine oxidase-like OS=Mus musculus GN=Pcyox1l PE=2 SV=1 - [PCYXL_MOUSE]	0.12	0.08	-0.12	-0.20	-0.30	-1.09	-1.23	-1.21	-1.42	-0.82	-1.04	-0.78	-0.89
Q99KN2	Probable cytosolic iron-sulfur protein assembly protein CIAO1 OS=Mus musculus GN=Ciao1 PE=2 SV=1 - [CIAO1_MOUSE]	-0.09	-0.03	0.06	-0.20	-0.17	-1.10	-0.93	-1.09	-0.90	-1.05	-0.83	-0.93	-0.78
Q8CFI5	Probable proline--tRNA ligase, mitochondrial OS=Mus musculus GN=Pars2 PE=2 SV=2 - [SYPM_MOUSE]	0.09	-0.31	-0.28	-0.18	-0.30	-0.92	-0.97	-1.02	-1.01	-0.90	-0.97	-0.75	-0.80

Q9Z2Y8	Proline synthase co-transcribed bacterial homolog protein OS=Mus musculus GN=Prosc PE=1 SV=1 - [PROSC_MOUSE]	-0.15	-0.22	-0.07	-0.24	-0.09	-0.80	-0.67	-0.81	-0.58	-0.89	-0.75	-0.67	-0.57
Q9Z2U0	Proteasome subunit alpha type-7 OS=Mus musculus GN=Psma7 PE=1 SV=1 - [PSA7_MOUSE]	-0.14	-0.10	0.09	-0.03	0.13	-0.57	-0.47	-0.54	-0.41	-0.62	-0.40	-0.51	-0.38
G3UZW8	Proteasome subunit beta type (Fragment) OS=Mus musculus GN=Psmb8 PE=2 SV=2 - [G3UZW8_MOUSE]	0.04	0.34	0.30	0.02	-0.02	-0.49	-0.54	-0.65	-0.69	-0.55	-0.59	-0.70	-0.73
Q9R1P3	Proteasome subunit beta type-2 OS=Mus musculus GN=Psmb2 PE=1 SV=1 - [PSB2_MOUSE]	-0.10	0.37	0.34	0.03	0.15	-0.82	-0.66	-1.01	-0.94	-0.82	-0.78	-0.51	-0.37
Q9R1P1	Proteasome subunit beta type-3 OS=Mus musculus GN=Psmb3 PE=1 SV=1 - [PSB3_MOUSE]	-0.07	0.20	0.16	0.24	0.29	-0.62	-0.59	-0.66	-0.50	-0.48	-0.50	-0.45	-0.36
Q9D2F8	Protein 4930547C10Rik OS=Mus musculus GN=4930547C10Rik PE=2 SV=1 - [Q9D2F8_MOUSE]	0.75	1.06	0.31	-0.53	-1.28	-0.50	-1.25	-0.44	-1.19	-0.52	-1.27	-0.44	-1.19
Q8CJG1	Protein argonaute-1 OS=Mus musculus GN=Ago1 PE=1 SV=2 - [AGO1_MOUSE]	0.04	0.13	0.08	-0.14	-0.13	-0.96	-1.09	-0.73	-0.86	-0.86	-0.94	-0.41	-0.43
Q8CJG0	Protein argonaute-2 OS=Mus musculus GN=Ago2 PE=1 SV=3 - [AGO2_MOUSE]	-0.15	0.04	0.13	-0.08	0.10	-0.73	-0.63	-0.82	-0.68	-0.83	-0.53	-0.69	-0.44
E0CX20	Protein BUD31 homolog OS=Mus musculus GN=Bud31 PE=4 SV=1 - [E0CX20_MOUSE]	-0.24	-0.64	-0.37	-0.26	0.03	-0.97	-0.74	-0.90	-0.64	-1.14	-0.82	-1.07	-0.74
D3YXJ2	Protein Gm9920 OS=Mus musculus GN=Gm9920 PE=2 SV=1 - [D3YXJ2_MOUSE]	-0.15	-0.34	-0.19	-0.02	0.13	-1.24	-1.10	-0.68	-0.54	-1.41	-1.25	-1.15	-1.00
Q3UR97	Protein Snx21 OS=Mus musculus GN=Snx21 PE=2 SV=1 - [Q3UR97_MOUSE]	0.00	-0.28	-0.28	-0.01	-0.02	-0.89	-0.89	-1.22	-1.22	-1.11	-1.11	-1.09	-1.09
P23492	Purine nucleoside phosphorylase OS=Mus musculus GN=Pnp PE=1 SV=2 - [PNPH_MOUSE]	0.13	-0.07	-0.14	-0.20	-0.32	-1.24	-1.35	-1.23	-1.18	-1.19	-1.25	-0.88	-1.02
P60487	Pyridoxal phosphate phosphatase OS=Mus musculus GN=Pdxp PE=1 SV=1 - [PLPP_MOUSE]	0.13	-0.03	-0.22	-0.13	-0.26	-0.89	-1.03	-0.80	-0.94	-0.75	-0.86	-0.66	-0.72
Q9DCC4	Pyrroline-5-carboxylate reductase 3 OS=Mus musculus GN=Pycrl PE=2 SV=2 - [P5CR3_MOUSE]	0.38	-0.11	-0.40	-0.04	-0.24	-0.52	-0.52	-0.35	-0.74	-0.38	-0.37	-0.39	-0.78
P26043	Radixin OS=Mus musculus GN=Rdx PE=1 SV=3 - [RADI_MOUSE]	-0.29	-0.51	-0.12	-0.23	0.00	-0.92	-0.57	-0.87	-0.69	-1.04	-0.73	-0.91	-0.52

Q99PI8	Reticulon-4 receptor OS=Mus musculus GN=Rtn4r PE=2 SV=1 - [RTN4R_MOUSE]	-0.18	0.05	0.13	-0.08	0.10	-0.57	-0.43	-0.74	-0.63	-0.58	-0.48	-0.47	-0.31
Q8K0S5	Reticulon-4 receptor-like 1 OS=Mus musculus GN=Rtn4rl1 PE=2 SV=1 - [R4RL1_MOUSE]	-0.07	-0.13	-0.06	-0.09	-0.03	-0.55	-0.50	-0.66	-0.60	-0.53	-0.46	-0.40	-0.34
Q99PT1	Rho GDP-dissociation inhibitor 1 OS=Mus musculus GN=Arhgdia PE=1 SV=3 - [GDIR1_MOUSE]	0.08	-0.16	-0.24	0.01	-0.07	-0.67	-0.76	-0.71	-0.77	-0.74	-0.79	-0.71	-0.81
P17563	Selenium-binding protein 1 OS=Mus musculus GN=Selenbp1 PE=1 SV=2 - [SBP1_MOUSE]	0.17	0.02	0.04	0.07	-0.10	-0.39	-0.50	-0.43	-0.50	-0.39	-0.57	-0.33	-0.49
P63300	Selenoprotein W OS=Mus musculus GN=Sepw1 PE=1 SV=3 - [SELW_MOUSE]	-0.01	-0.91	-0.91	-0.15	-0.14	-0.58	-0.58	-0.65	-0.65	-0.75	-0.74	-0.66	-0.65
H3BK32	Sentrin-specific protease 6 (Fragment) OS=Mus musculus GN=Senp6 PE=2 SV=1 - [H3BK32_MOUSE]	2.39	2.83	0.44	-0.39	-2.78	-0.76	-3.15	-0.67	-3.06	-0.82	-3.20	-0.56	-2.94
H3BLA9	Sentrin-specific protease 6 (Fragment) OS=Mus musculus GN=Senp6 PE=2 SV=1 - [H3BLA9_MOUSE]	0.23	0.07	-0.16	-0.15	-0.38	-1.24	-1.47	-1.25	-1.48	-0.97	-1.19	-0.81	-1.04
D3YX28	Serine protease HTRA2, mitochondrial OS=Mus musculus GN=Htra2 PE=2 SV=1 - [D3YX28_MOUSE]	-0.07	-0.34	-0.22	-0.15	-0.16	-0.63	-0.57	-0.46	-0.43	-0.39	-0.38	-0.63	-0.54
Q91YA2	Serine/threonine-protein kinase H1 OS=Mus musculus GN=Pskh1 PE=2 SV=3 - [KPSH1_MOUSE]	0.00	-0.80	-0.80	-0.17	-0.17	-0.83	-0.84	-0.69	-0.70	-0.85	-0.85	-1.03	-1.02
F7BX26	Serine/threonine-protein phosphatase (Fragment) OS=Mus musculus GN=Ppp5c PE=3 SV=1 - [F7BX26_MOUSE]	-0.02	-0.03	-0.03	-0.11	-0.10	-0.54	-0.50	-0.46	-0.47	-0.49	-0.47	-0.49	-0.47
P63328	Serine/threonine-protein phosphatase 2B catalytic subunit alpha isoform OS=Mus musculus GN=Ppp3ca PE=1 SV=1 - [PP2BA_MOUSE]	-0.05	-0.02	-0.09	-0.16	-0.10	-0.35	-0.43	-0.40	-0.52	-0.40	-0.50	-0.31	-0.31
P97470	Serine/threonine-protein phosphatase 4 catalytic subunit OS=Mus musculus GN=Ppp4c PE=1 SV=2 - [PP4C_MOUSE]	0.12	-0.01	-0.09	0.05	-0.11	-0.69	-0.93	-0.80	-0.86	-0.75	-0.86	-0.69	-0.78

P62137	Serine/threonine-protein phosphatase PP1-alpha catalytic subunit OS=Mus musculus GN=Ppp1ca PE=1 SV=1 - [PP1A_MOUSE]	0.04	0.13	0.10	-0.13	-0.03	-0.85	-0.71	-0.55	-0.53	-0.75	-0.58	-0.61	-0.44
P62141	Serine/threonine-protein phosphatase PP1-beta catalytic subunit OS=Mus musculus GN=Ppp1cb PE=1 SV=3 - [PP1B_MOUSE]	-0.03	-0.14	-0.21	-0.18	-0.14	-0.96	-0.88	-0.81	-0.75	-0.84	-0.90	-0.77	-0.65
Q9Z1Z2	Serine-threonine kinase receptor-associated protein OS=Mus musculus GN=Strap PE=1 SV=2 - [STRAP_MOUSE]	-0.04	-0.36	-0.21	-0.17	-0.12	-1.08	-0.99	-1.07	-0.98	-1.06	-1.06	-0.98	-0.98
P07724	Serum albumin OS=Mus musculus GN=Alb PE=1 SV=3 - [ALBU_MOUSE]	-0.09	-0.31	-0.21	-0.79	-0.73	-0.92	-0.83	-0.69	-0.62	-0.59	-0.50	-0.94	-0.84
Q8BG73	SH3 domain-binding glutamic acid-rich-like protein 2 OS=Mus musculus GN=Sh3bgrl2 PE=1 SV=1 - [SH3L2_MOUSE]	-0.17	-0.54	-0.27	-0.31	-0.07	-0.69	-0.49	-0.66	-0.50	-0.87	-0.66	-0.75	-0.54
Q810C1	SLIT and NTRK-like protein 1 OS=Mus musculus GN=Slitrk1 PE=2 SV=1 - [SLIK1_MOUSE]	-0.03	-0.46	-0.58	-0.13	-0.11	-0.42	-0.51	-0.41	-0.70	-0.42	-0.70	-0.42	-0.57
Q9CQ65	S-methyl-5'-thioadenosine phosphorylase OS=Mus musculus GN=Mtap PE=2 SV=1 - [MTAP_MOUSE]	0.23	0.00	-0.26	-0.19	-0.44	-1.29	-1.52	-1.33	-1.65	-1.22	-1.44	-1.11	-1.30
O08966	Solute carrier family 22 member 1 OS=Mus musculus GN=Slc22a1 PE=2 SV=2 - [S22A1_MOUSE]	0.36	0.07	-0.30	-0.45	-0.81	-0.96	-1.33	-0.67	-1.03	-0.91	-1.27	-0.56	-0.92
Q8CFD4	Sorting nexin-8 OS=Mus musculus GN=Snx8 PE=2 SV=1 - [SNX8_MOUSE]	0.04	-0.31	-0.35	-0.06	-0.10	-1.11	-1.15	-1.14	-1.19	-1.07	-1.10	-0.61	-0.64
P09671	Superoxide dismutase [Mn], mitochondrial OS=Mus musculus GN=Sod2 PE=1 SV=3 - [SODM_MOUSE]	-0.01	-0.33	-0.31	-0.12	-0.10	-0.67	-0.63	-0.64	-0.65	-0.66	-0.64	-0.66	-0.68
Q9D614	Syntaxin 17, isoform CRA_b OS=Mus musculus GN=Stx17 PE=2 SV=1 - [Q9D614_MOUSE]	-0.41	-0.65	-0.24	-1.17	-0.77	-0.91	-0.51	-0.82	-0.42	-0.86	-0.45	-1.53	-1.11
Q9D1X8	Tetraspanin 2, isoform CRA_b OS=Mus musculus GN=Tspan2 PE=2 SV=1 - [Q9D1X8_MOUSE]	0.19	0.58	0.19	0.11	-0.13	-0.50	-0.58	-0.51	-0.49	-0.30	-0.35	-0.42	-0.55
Q8QZY6	Tetraspanin-14 OS=Mus musculus GN=Tspan14 PE=1 SV=1 - [TSN14_MOUSE]	0.12	-0.12	-0.24	-0.11	-0.23	-0.66	-0.78	-0.32	-0.45	-0.31	-0.43	-0.86	-0.98

D3Z0K8	Thioredoxin reductase 2, mitochondrial OS=Mus musculus GN=Txnrd2 PE=2 SV=1 - [D3Z0K8_MOUSE]	-0.04	-0.27	-0.26	-0.31	-0.29	-1.28	-1.28	-1.29	-1.49	-1.49	-1.41	-1.21	-1.22
P83877	Thioredoxin-like protein 4A OS=Mus musculus GN=Txnl4a PE=2 SV=1 - [TXN4A_MOUSE]	0.19	-0.45	-0.68	-0.22	-0.41	-0.39	-0.71	-0.79	-1.15	-0.71	-0.98	-0.42	-0.56
Q9DBB8	Trans-1,2-dihydrobenzene-1,2-diol dehydrogenase OS=Mus musculus GN=Dhdh PE=2 SV=1 - [DHDH_MOUSE]	0.31	-0.12	-0.41	-0.19	-0.47	-1.46	-1.70	-1.38	-1.63	-1.37	-1.53	-0.93	-1.17
O88968	Transcobalamin-2 OS=Mus musculus GN=Tcn2 PE=2 SV=1 - [TCO2_MOUSE]	0.00	-0.18	-0.18	-0.14	-0.14	-0.69	-0.69	-0.68	-0.68	-0.79	-0.79	-0.81	-0.81
P42669	Transcriptional activator protein Pur-alpha OS=Mus musculus GN=Pura PE=1 SV=1 - [PURA_MOUSE]	0.06	-0.03	-0.16	-0.12	-0.17	-0.56	-0.65	-0.46	-0.54	-0.63	-0.65	-0.57	-0.61
P40142	Transketolase OS=Mus musculus GN=Tkt PE=1 SV=1 - [TKT_MOUSE]	0.05	-0.32	-0.38	-0.27	-0.30	-1.09	-1.14	-1.00	-1.06	-1.00	-1.05	-0.90	-1.03
Q922P8	Transmembrane protein 132A OS=Mus musculus GN=Tmem132a PE=2 SV=2 - [T132A_MOUSE]	0.06	0.11	0.07	-0.02	-0.10	-0.35	-0.45	-0.42	-0.37	-0.37	-0.45	-0.52	-0.47
E9Q1B3	Uncharacterized protein OS=Mus musculus GN=Gm7275 PE=4 SV=1 - [E9Q1B3_MOUSE]	0.09	1.07	0.97	-0.16	-0.25	-1.34	-1.44	-1.44	-1.53	-1.35	-1.44	-1.27	-1.36
Q3UPV6	Voltage-gated potassium channel subunit beta-2 OS=Mus musculus GN=Kcnab2 PE=2 SV=1 - [Q3UPV6_MOUSE]	-0.05	0.11	0.07	0.00	0.10	-0.51	-0.41	-0.54	-0.49	-0.54	-0.45	-0.49	-0.42
Q99KC8	von Willebrand factor A domain-containing protein 5A OS=Mus musculus GN=Vwa5a PE=1 SV=2 - [VMA5A_MOUSE]	0.01	-0.13	-0.09	-0.23	-0.12	-0.58	-0.62	-0.54	-0.51	-0.51	-0.53	-0.51	-0.55
Q9CR39	WD repeat domain phosphoinositide-interacting protein 3 OS=Mus musculus GN=Wdr45b PE=2 SV=2 - [WIPI3_MOUSE]	0.17	-0.24	-0.39	-0.16	-0.35	-1.09	-1.36	-1.19	-1.39	-1.14	-1.36	-0.93	-1.13
Q9D0I6	WD repeat, SAM and U-box domain-containing protein 1 OS=Mus musculus GN=Wdsub1 PE=2 SV=1 - [WSDU1_MOUSE]	-0.14	-0.32	-0.18	-0.18	-0.04	-0.84	-0.71	-0.61	-0.47	-0.56	-0.41	-0.50	-0.35
Q91V09	WD repeat-containing protein 13 OS=Mus musculus GN=Wdr13 PE=1 SV=1 - [WDR13_MOUSE]	-0.02	0.13	0.06	-0.12	-0.13	-0.53	-0.60	-0.39	-0.45	-0.49	-0.48	-0.54	-0.52

P61965	WD repeat-containing protein 5 OS=Mus musculus GN=Wdr5 PE=1 SV=1 - [WDR5_MOUSE]	-0.02	-0.63	-0.50	-0.11	-0.13	-1.40	-1.42	-1.44	-1.51	-1.34	-1.41	-1.15	-1.18
Q9R0D8	WD repeat-containing protein 54 OS=Mus musculus GN=Wdr54 PE=2 SV=1 - [WDR54_MOUSE]	0.13	-0.04	-0.07	-0.28	-0.36	-0.72	-0.87	-0.58	-0.80	-0.61	-0.75	-0.35	-0.48
Q8BFQ4	WD repeat-containing protein 82 OS=Mus musculus GN=Wdr82 PE=1 SV=1 - [WDR82_MOUSE]	0.08	-0.07	-0.15	-0.09	-0.17	-0.75	-0.84	-0.38	-0.47	-0.46	-0.54	-1.01	-1.09
Q8BGF3	WD repeat-containing protein 92 OS=Mus musculus GN=Wdr92 PE=2 SV=1 - [WDR92_MOUSE]	-0.09	0.00	0.08	-0.35	-0.26	-0.91	-0.83	-0.68	-0.59	-0.78	-0.69	-0.51	-0.42
Q11136	Xaa-Pro dipeptidase OS=Mus musculus GN=Pepd PE=2 SV=3 - [PEPD_MOUSE]	0.02	-0.18	-0.19	-0.32	-0.26	-1.41	-1.54	-1.50	-1.56	-1.49	-1.55	-1.58	-1.72
Q00519	Xanthine dehydrogenase/oxidase OS=Mus musculus GN=Xdh PE=1 SV=5 - [XDH_MOUSE]	-0.11	-0.04	-0.18	-0.27	-0.15	-0.52	-0.87	-0.81	-0.65	-0.87	-0.62	-0.86	-0.74
Q8VEB6	Zinc phosphodiesterase ELAC protein 1 OS=Mus musculus GN=Elac1 PE=2 SV=1 - [RNZ1_MOUSE]	0.19	0.06	-0.24	-0.11	-0.32	-0.91	-1.02	-1.00	-1.31	-0.85	-1.09	-0.63	-0.83
