# Supplementary figures and images for: Are you also what your mother eats? Distinct proteomic portrait as a result of maternal high-fat diet in the cerebral cortex of the adult mouse
Source: Int J Obes (Lond). 2015 Apr 21;39(8):1325–8. doi: 10.1038/ijo.2015.35 (PMC5399160; doi:10.1038/ijo.2015.35)

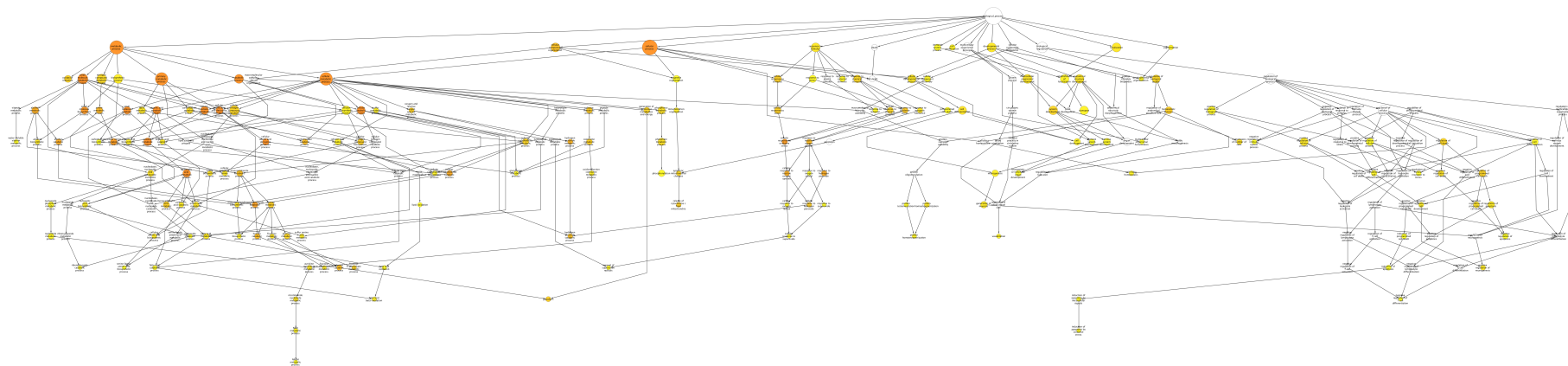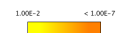

Supplement: Supplementary Figure 1 [file ijo201535x6.pdf]
