# Supplementary material for: Are you also what your mother eats? Distinct proteomic portrait as a result of maternal high-fat diet in the cerebral cortex of the adult mouse
Source: Int J Obes (Lond). 2015 Apr 21;39(8):1325–8. doi: 10.1038/ijo.2015.35 (PMC5399160; doi:10.1038/ijo.2015.35)
Supplement: Supplementary Figure and Table Legends [file ijo201535x7.doc]

**Supplementary Figure and Table Legends**

**Supplementary Figure 1.** Gene ontology (GO) terms related to biological processes that were significantly enriched (FDR corrected *p*-value <0.05, hypergeometric test) for the 251 proteins commonly up- or down-regulated in the HC and HH groups relative to the control (CC). Coloured nodes representing GO terms correspond to those that were significant according to the *p*-value color scale whereas white nodes were not significant. The size of the node reflects the number of proteins that mapped to the corresponding GO term. This figure is of high quality and can be enlarged in order to visualize the GO term on each node.

**Supplementary Table 1.** Total Proteome

**Supplementary Table 2**. Modulated Proteome

**Supplementary Table 3.** Commonly Modulated Proteins in the HC and HH groups

**Supplementary Methods 1**. High-pH Reverse Phase Peptide Fractionation

**Supplementary Methods 2**. LC-FT-Orbitrap MS Analysis
